# Beyond accuracy: a three-stage evaluation of large language models for postpartum health consultation and nursing decision support

**DOI:** 10.3389/fpubh.2026.1961647

**Published:** 2026-10-20

**Authors:** Xilin Zhao, Xinwen Zhang, Lei Liu

**Affiliations:** 1 Delivery Center, Xi’an People’s Hospital (Xi’an Fourth Hospital), Xi’an, Shaanxi, China; 2 Women’s Health Care Center, Xi’an People’s Hospital (Xi’an Fourth Hospital), Xi’an, Shaanxi, China

**Keywords:** digital health, health consultation, large language models, maternal health, nursing decision support, patient safety, postpartum care

## Abstract

**Background:**

Large language models may support postpartum health consultation and nursing care plan development, but their performance across different task types remains uncertain. This study compared the performance of ChatGPT 5.5 and DeepSeek V3 in these settings.

**Methods:**

A three-stage cross-sectional evaluation was conducted, comprising repeated querying of 30 guideline-based questions, consultations involving 120 postpartum women, and 120 de-identified retrospective clinical cases. Responses were evaluated by clinical experts using a blinded assessment process. In Stage 2, the postpartum women who submitted the questions also performed blinded evaluations from the user perspective. Paired-samples t tests or Wilcoxon signed-rank tests were used, as appropriate. Holm-Bonferroni correction was applied within outcome families defined by study stage and evaluator type, and intraclass correlation coefficients were calculated to assess agreement among clinical expert ratings.

**Results:**

In Stage 1, ChatGPT 5.5 received a higher correctness score than DeepSeek V3 (4.00 ± 0.00 versus 3.88 ± 0.33; Holm-adjusted *p* = 0.002). DeepSeek V3 received higher scores for completeness and clinical management appropriateness (both Holm-adjusted *p* < 0.001) and safety (Holm-adjusted *p* = 0.001), although its responses were more difficult to read. In Stage 2, ChatGPT 5.5 received higher patient ratings for satisfaction and usefulness and higher clinician ratings for correctness, completeness, helpfulness, and safety, whereas DeepSeek V3 received a higher clinician-rated readability score (all Holm-adjusted *p* < 0.001). In Stage 3, ChatGPT 5.5 received higher scores for clinical appropriateness, comprehensiveness, actionability, safety, and triage appropriateness (all Holm-adjusted *p* < 0.001), whereas DeepSeek V3 received a higher individualization score (Holm-adjusted *p* = 0.021).

**Conclusion:**

LLM performance varied depending on the task, evaluation metric, and use case. ChatGPT 5.5 received higher scores across most clinically relevant dimensions in real-world postpartum consultations and nursing care plan generation, whereas DeepSeek V3 received higher scores for completeness and safety in standardized questions, clinician-rated readability in real-world consultations, and individualization in care-plan generation. LLMs may support health education and preparation of initial nursing care plan drafts, but their outputs require clinical review.

## 1 Introduction

The postpartum period is a critical stage during which the maternal body gradually recovers from pregnancy and childbirth. It generally extends from delivery of the placenta to 6 weeks postpartum, or approximately 42 days (1, 2). This period involves multiple physiological and psychological changes, including uterine involution, lochia discharge, wound healing, initiation of lactation, pelvic floor recovery, and psychological adjustment. It is also a period in which postpartum complications and health problems are relatively common. Postpartum hemorrhage, puerperal infection, hypertensive disorders, venous thromboembolism, and postpartum mental health disorders may continue to threaten maternal safety and quality of life (1, 3, 4). Therefore, maternal health management during the postpartum period should address not only routine recovery but also the timely identification of abnormal symptoms and potential warning signs.

After hospital discharge, postpartum women must often manage their own recovery while caring for a newborn and adapting to changing family roles. Their health consultation needs are therefore highly immediate, individualized, and conversational. Many postpartum women repeatedly seek explanations and guidance regarding changes in lochia, perineal or abdominal incision care, breast engorgement and breastfeeding, urinary and bowel function, pain management, resumption of physical activity, psychological well-being, sexual activity, and contraception. However, routine outpatient services, postpartum visits, and nursing follow-up are constrained by limited time, workforce capacity, and accessibility, making it difficult to continuously address the diverse concerns that arise during recovery at home (1, 4). In addition, insufficient awareness of postpartum warning signs, such as severe bleeding, high fever, lower-extremity swelling or pain, chest tightness, shortness of breath, severe headache, visual disturbances, and thoughts of self-harm, may impair care-seeking decisions and increase the risk of delayed treatment (5). Therefore, accurate, comprehensible, and actionable health information is essential to support maternal self-care and timely access to professional assistance.

In recent years, large language models (LLMs) have shown potential for use in medical question answering, patient education, and clinical communication because of their natural language understanding and generation capabilities (6–8). Some models have demonstrated favorable clinical accuracy when responding to questions related to postpartum depression (9). However, the quality of LLM-generated responses may be influenced by the model version, language, prompt design, completeness of the input information, and repeated generation. These responses may also contain factual inaccuracies, omit important information, deviate from clinical guidelines, impose an excessive reading burden, or provide inappropriate triage recommendations (6, 8, 9). During the postpartum period, which involves both physiological recovery and the risk of acute complications, inaccurate or insufficient responses may cause women to underestimate warning signs, delay seeking medical care, or adopt inappropriate self-management measures. Therefore, before LLMs are used in postpartum health education or nursing follow-up, their performance should be systematically evaluated across multiple dimensions, including content quality, safety, comprehensibility, and clinical applicability.

### 1.1 Related work

Across medicine, LLMs have been investigated for medical question answering, patient education, and clinical communication. Reviews and benchmark studies indicate that these systems can generate clinically relevant information, but their performance varies across models and tasks, and concerns remain regarding factual accuracy, completeness, readability, and safety (6–8). These observations support evaluation frameworks that extend beyond factual correctness alone and include dimensions relevant to actual patient-facing use.

Within obstetrics and perinatal care, prior studies have examined distinct but relatively narrow use cases. Sezgin et al. (9) assessed the clinical accuracy of LLM responses to postpartum depression questions, while Khromchenko et al. (10) compared responses to commonly asked pregnancy questions. Yigit Yalçın et al. (11) used a multidimensional framework to evaluate responses to patient questions on gestational diabetes mellitus, and Friedman et al. (12) examined case-based decision-making in maternal-fetal medicine. These studies indicate that performance can vary with clinical topic, task structure, and evaluation framework, but they largely rely on predefined questions, condition-specific education, or simulated cases rather than combining patient-facing and clinician-facing tasks using authentic postpartum data. More recently, Tığlı et al. (13) evaluated ChatGPT, Gemini, and DeepSeek using multilingual real patient pregnancy questions and reported variation by model and language, while also highlighting concerns about reference reliability. Collectively, this literature supports broader evaluation across complementary real-world tasks.

### 1.2 Research gap and study questions

Important methodological gaps therefore remain. First, most studies have relied on predefined questions, patient-style questions, or hypothetical cases, which may not adequately reflect the spontaneous language and ongoing consultation needs of postpartum women during follow-up. Second, previous studies have generally focused on a single condition or task. Few have evaluated, within the same study framework, guideline-based knowledge, responses to authentic postpartum consultations, and nursing care-plan generation from real-world clinical data. Third, postpartum health consultation requires more than factual accuracy: responses must identify warning signs, provide actionable recommendations, remain understandable to users with varying levels of health literacy, and support appropriate escalation when needed. Evaluating standardized question answering alone may therefore be insufficient to determine the practical value of LLMs in postpartum care settings.

Against this background, we designed a three-stage cross-sectional model evaluation study to compare ChatGPT 5.5 and DeepSeek V3 in maternal health scenarios during the postpartum period. The evaluation was conducted at three levels: guideline-based knowledge, responses to real-world health consultations, and synthesis of clinical information. Particular attention was given to safety, individualization, actionability, and readability. Accordingly, this study addressed the following research questions:

RQ1: How do ChatGPT 5.5 and DeepSeek V3 differ in the quality, safety, and readability of responses to guideline-based postpartum clinical questions?
RQ2: How do the two models differ in clinician- and patient-rated performance when responding to real-world postpartum health consultation questions?
RQ3: How do the two models differ in their ability to generate clinically appropriate, individualized, comprehensive, actionable, and safe postpartum nursing care plans from real-world clinical case information?

Interrater reliability was additionally examined as a methodological quality check on the clinical expert evaluations.

## 2 Materials and methods

### 2.1 Study design

This three-stage cross-sectional model evaluation study was conducted at Xi’an People’s Hospital (Xi’an Fourth Hospital), Xi’an, Shaanxi Province, China. The real-world consultation questions used in Stage 2 and the retrospective case data used in Stage 3 were obtained from postpartum follow-up WeChat groups and the electronic medical record system between January and April 2026. All real-world questions and case data were screened, organized, and de-identified before model testing.

Model testing and response generation were completed between May 1 and June 30, 2026. Scoring and statistical analyses were conducted between June and July 2026. Stage 1 evaluated the quality of model responses to guideline-derived clinical questions and their readability in Chinese and English. Stage 2 assessed the quality of model responses to related questions raised by postpartum women concerning the same health topic. Stage 3 evaluated the models’ ability to generate postpartum maternal nursing care plans based on de-identified retrospective cases.

All original model outputs retained for analysis were preserved verbatim without deletion, correction, or manual editing. English translations in the Supplementary files were prepared using a web-based translation tool and manually corrected solely to facilitate international readership; they were not used for scoring or statistical analysis. Before evaluation, information identifying the source model was removed, and the responses were assigned randomized identification numbers.

The testing, scoring, and statistical analysis procedures for all three stages are presented in Figure 1.

**Figure 1:**
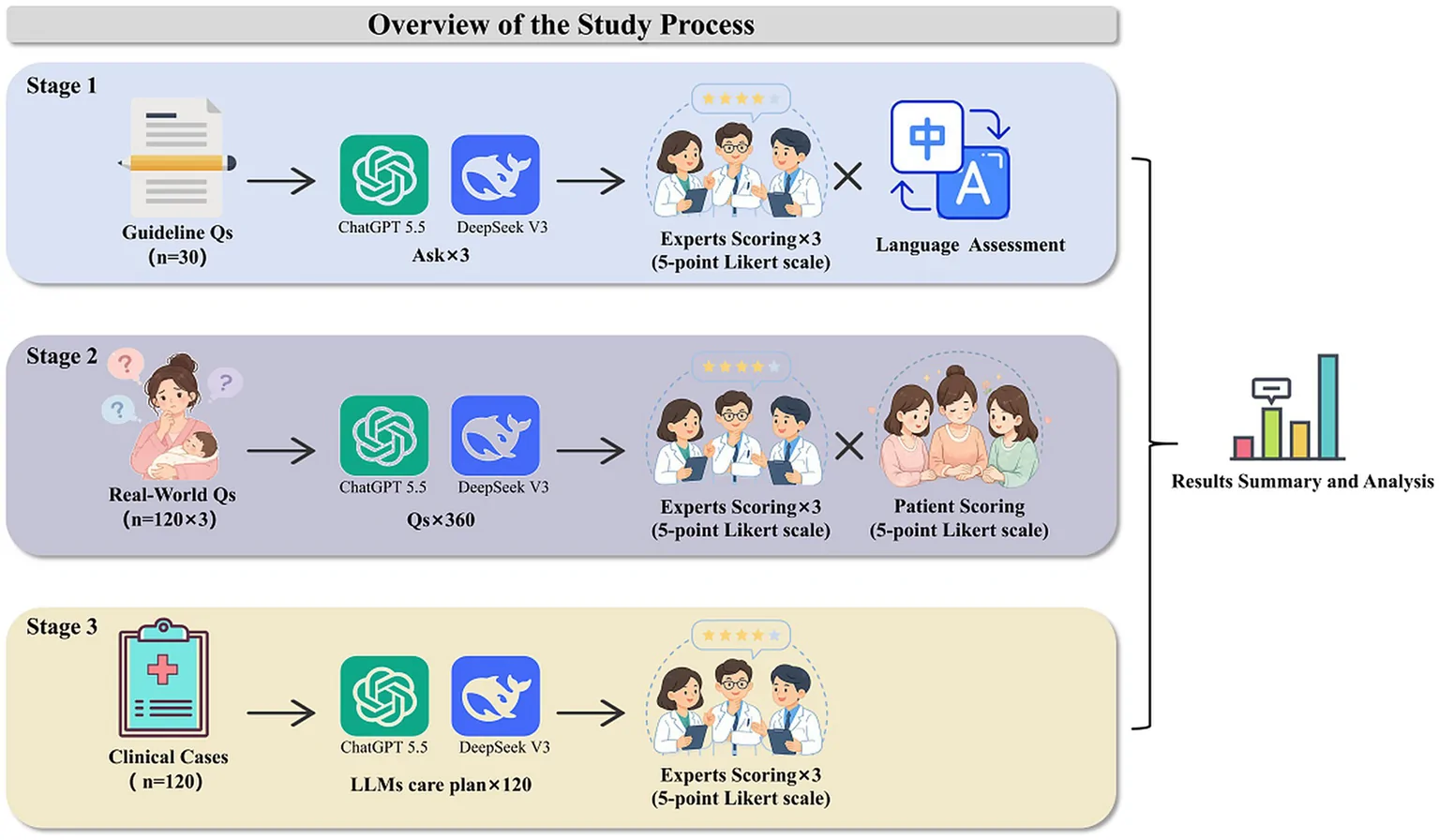
Overview of the three-stage study process. Stage 1 evaluated Chinese responses to repeated guideline-based prompts using expert ratings and assessed the readability of both Chinese and English responses. Stage 2 evaluated model responses to real postpartum consultation questions using both expert and patient ratings. Stage 3 evaluated postpartum maternal nursing care plans generated from de-identified standardized case summaries derived from real-world clinical records using expert ratings. Expert and patient ratings used a 5-point Likert scale, whereas automated readability measures were assessed separately.

### 2.2 Models and testing procedures

This study evaluated ChatGPT 5.5 and DeepSeek V3, both of which were publicly accessible through their official web interfaces during the study period. The model versions and reasoning modes displayed on each platform were used, and no application programming interfaces were employed. No additional background materials were provided to the models other than the predefined questions, prompts, or de-identified case information specified in the study protocol.

In Stage 1, standardized prompts developed in advance were used for both the Chinese- and English-language tests. Except for the language of presentation, the question content, task instructions, and output requirements were identical across the two language versions. Standardized Chinese-language prompts developed in advance were used separately in Stages 2 and 3. Each question or case was entered into a new, independent conversation to prevent any influence from previous contextual information. Standardized role instructions were intentionally included to reduce between-model variability in task interpretation and to simulate a structured clinical decision-support setting. The same task-specific prompt structure was applied to both models to ensure comparability. Accordingly, the present study evaluated model performance under standardized prompt-engineered conditions rather than completely unprompted or spontaneous consumer use.

In accordance with the study protocol, each question in Stage 1 was independently submitted three times. In Stages 2 and 3, only the first response generated by each model was recorded. The regenerate, continue-generation, and manual follow-up functions were not used. All original model outputs retained for analysis were preserved verbatim without deletion, correction, or manual editing. Detailed model access and testing settings are provided in Supplementary Table S5.

### 2.3 Stage 1: responses to clinical knowledge questions

Based on guidelines and standards issued by the World Health Organization, the National Institute for Health and Care Excellence, the American College of Obstetricians and Gynecologists, the Royal College of Obstetricians and Gynaecologists, the Faculty of Sexual and Reproductive Healthcare, and relevant Chinese authorities on maternal health management (1, 14–19), the research team developed 30 open-ended clinical knowledge questions. The questions covered postpartum visits, warning signs, lochia and bleeding, puerperal infection, wound care, breastfeeding, urinary and bowel function, pelvic floor recovery, thromboembolic risk, postpartum management of pregnancy complications, mental health, contraception, and health education.

The 30 questions were independently reviewed by two obstetricians and one midwifery nursing expert. The review focused on clinical relevance, consistency with guidelines, clarity of wording, and coverage of key topics. The research team revised the questions in accordance with expert feedback and finalized the question set.

All 30 questions were submitted to both models in Chinese and English. Clinical expert ratings were performed only for the Chinese-language responses, whereas the English-language responses were used exclusively for English readability assessment. A standardized prompt instructed each model to answer according to authoritative guidelines and established clinical principles. Each question was submitted three times in separate conversations, yielding a total of 360 responses. The complete Stage 1 questions, original model responses, English translations where applicable, expert ratings, and readability outcomes are provided in Supplementary Tables S6–S9.

### 2.4 Stage 2: responses to questions from postpartum women

A total of 120 postpartum women were included. Each participant asked three interrelated questions concerning one primary health topic, resulting in 360 original questions. Direct and indirect identifiers were removed. To the greatest extent possible, the researchers preserved the participants’ conversational wording, continuity of expression, and real-world context without altering the original meaning of the questions.

The inclusion criteria were as follows: (1) age ≥18 years; (2) within 42 days postpartum, receiving postpartum follow-up, and participating in a WeChat group managed by the clinical team; (3) able to clearly express maternal health concerns related to the postpartum period in Chinese; (4) able to ask three interrelated questions concerning the same primary health topic; and (5) provision of informed consent for the use of de-identified questions in this study.

The exclusion criteria were as follows: (1) questions exclusively concerning neonatal health without relevance to maternal health; (2) questions unrelated to maternal health during the postpartum period; (3) questions with unclear meaning, substantial information gaps, or an original intent that could not be reliably determined; (4) questions that completely duplicated previously included items; and (5) questions containing identifying information that could not be adequately removed.

The three questions submitted by each postpartum woman were entered separately into both models as independent prompts, and each question generated only one response. Thus, each model produced 360 single-question responses. The three questions from the same participant and their corresponding responses were then combined into one composite evaluation unit. This process yielded 120 composite response units per model and 240 units across both models. The participant-level composite unit was selected *a priori* because the three questions submitted by each woman represented related concerns within a single health topic and were therefore not statistically or clinically independent. Treating all 360 questions as independent observations could have resulted in pseudoreplication and underestimated within-participant correlation. The composite approach was intended to capture the overall usefulness of the model responses across a coherent consultation topic; it was not intended to imply that errors in individual responses were clinically equivalent or interchangeable.

For each composite unit, the clinical experts simultaneously reviewed the three related questions submitted by the same postpartum woman and the three corresponding responses generated by one model. The complete set was evaluated as a single unit representing one health topic, rather than scoring each response separately. In addition, each postpartum woman evaluated, from the user perspective, the responses generated by both models to her own questions. All ratings were based on the overall content of the original Chinese-language responses. The de-identified Stage 2 questions, original Chinese-language responses, English translations, and patient and clinical expert ratings are provided in Supplementary Tables S10 and S11.

### 2.5 Stage 3: generation of nursing care plans

A total of 120 postpartum cases with relatively complete clinical data were retrospectively selected from the hospital’s electronic medical record system. All case data were de-identified before being entered into the models. Information was organized as de-identified standardized case summaries derived from real-world clinical records using a consistent template that included maternal age, gravidity and parity, gestational age, mode of delivery, course of labor, intrapartum blood loss, condition of the perineal or abdominal incision, pregnancy-related complications, and major risk factors. Standardization referred only to the consistency of the input items and presentation format and did not alter the original clinical facts.

The inclusion criteria were as follows: (1) delivery at the study hospital between January and April 2026; (2) availability of the core information required to develop a postpartum nursing care plan, including maternal age, gravidity and parity, gestational age, mode of delivery, course of labor, intrapartum blood loss, condition of the perineal or abdominal incision, pregnancy-related complications, and major risk factors; and (3) clinical records that could be de-identified and structured using the standardized template.

The exclusion criteria were as follows: (1) substantial missingness in core clinical information; (2) evident inconsistencies in the medical records that could not be verified; (3) duplicate records from the same postpartum woman; and (4) records containing only neonatal information or insufficient maternal information to support nursing care plan development.

Each case was entered separately into both models, which were instructed to develop a postpartum maternal nursing care plan. The standardized Chinese prompt required the plan to include major nursing problems, nursing goals, specific nursing interventions, key observations, health education, circumstances requiring triage, referral, or prompt reporting to a physician, and follow-up recommendations. It also required the content to be safe, specific, and actionable and not to replace medical diagnosis or treatment. Each model generated one response for each case, yielding a total of 240 nursing care plans. The exact standardized Chinese prompt, de-identified case summaries, original Chinese-language nursing care plans, English translations, and expert ratings are provided in Supplementary Table S12.

### 2.6 Evaluation methods

Three clinical experts were selected on the basis of professional experience in obstetric clinical care and/or midwifery nursing, familiarity with postpartum management, and experience in maternal health education or clinical decision-making. Each expert had more than 15 years of clinical experience and held an associate senior professional title. Before formal scoring, the three evaluators participated in a calibration session using sample responses excluded from the final dataset. They reviewed the operational definitions of each rating dimension and discussed example responses representing different score levels. Formal evaluation began only after the scoring criteria had been reviewed and interpreted consistently.

Expert evaluation was conducted using a blinded approach. Before scoring, the researchers removed model names, platform information, and any content that might indicate the source model. Responses were then presented in a randomized order. All experts completed the formal evaluations independently, and no discussion was permitted during formal scoring.

In Stage 2, user-perspective evaluations were also conducted by the postpartum women. Each participant evaluated the responses generated by both models to her own three related questions. Model names and platform information were removed before evaluation, and the responses from the two models were presented in a randomized order.

All dimensions were rated using a 5-point Likert scale, with 1 indicating very poor performance or substantial failure to meet the criterion and 5 indicating excellent performance or complete fulfillment of the criterion.

The Stage 1 evaluation dimensions included correctness, completeness, guideline consistency, safety, and clinical management appropriateness. In Stage 2, the clinician-rated dimensions included correctness, completeness, readability, helpfulness, and safety, whereas the patient-rated dimensions included satisfaction, understandability, and usefulness. The Stage 3 evaluation dimensions included clinical appropriateness, individualization, comprehensiveness, actionability, safety, triage appropriateness, and structure and clarity.

For each expert-rated dimension, the median score assigned by the three clinical experts was used as the final expert score. In Stage 2, the user-perspective evaluation was completed by the postpartum woman who had submitted the corresponding questions, and her rating was used directly as the final user score for that composite evaluation unit. Operational definitions and scoring criteria for all evaluation dimensions are provided in Supplementary Table S1.

### 2.7 Readability assessment

In Stage 1, the Chinese-language responses were evaluated using a Chinese text readability analysis tool (20). The Chinese readability score, corresponding educational level, and recommended reading age were recorded. English-language responses were evaluated using the Flesch–Kincaid Grade Level (21) to estimate the level of English education required to understand the text.

In Stage 2, clinical experts and postpartum women separately evaluated the readability and understandability of the responses to reflect both professional judgment and the actual reading experience of the intended users. To avoid changes in vocabulary, syntax, and text structure caused by translation, the Chinese readability analysis in Stage 1 was based on Chinese responses generated directly from Chinese-language prompts. The English readability analysis was based on English responses generated directly from English-language prompts.

Chinese educational level and English reading-difficulty categories were treated as ordinal categorical variables. Chinese educational level was classified into two categories: junior high school and senior high school. Based on the Flesch–Kincaid Grade Level, English reading difficulty was classified into five categories: 7th grade: fairly easy to read; 8th & 9th grade: plain English; 10th to 12th grade: fairly difficult; college: difficult; and college graduate: very difficult. The specific classification thresholds are presented in Supplementary Table S2.

### 2.8 Statistical analysis

#### 2.8.1 Descriptive analysis of response quality and between-model comparisons

Statistical analyses were performed using IBM SPSS Statistics, version 27.0. Final scores for each response unit were summarized as means ± standard deviations, whereas categorical variables were presented as frequencies and percentages.

Because the responses generated by ChatGPT 5.5 and DeepSeek V3 were matched one-to-one for the same question, consultation unit, or clinical case, paired analyses were used for between-model comparisons.

In Stage 1, each individual Chinese-language response was treated as the unit of analysis. Every response was independently rated by three clinical experts, and the median of the three expert ratings was used as the final score for that response. Each of the 30 questions was independently submitted three times to each model, yielding 90 response-level final scores per model. Responses were paired according to the same question and repetition number, resulting in 90 matched response pairs. The Wilcoxon signed-rank test was used to compare the two models across all expert-rated dimensions.

The readability analyses of the Chinese- and English-language responses in Stage 1 also used each individual response as the unit of analysis. A total of 90 Chinese-language responses and 90 English-language responses from each model were included.

In Stage 2, the unit of analysis was the composite evaluation unit comprising the three related questions submitted by each postpartum woman and the corresponding responses. A total of 120 units were included for each model. In Stage 3, the unit of analysis was the nursing care plan generated for each retrospective case, with 120 care plans included for each model.

Between-model differences in each Likert-scale dimension were assessed using the Wilcoxon signed-rank test. For continuous readability measures, including the Chinese readability score, recommended reading age, and Flesch–Kincaid Grade Level, the normality of the paired differences was first assessed using the Shapiro–Wilk test. A paired-samples t test was used when the paired differences were normally distributed; otherwise, the Wilcoxon signed-rank test was applied. Chinese educational levels and English reading-difficulty categories were treated as ordinal categorical variables, summarized as frequencies and percentages, and compared between the two models using the Wilcoxon signed-rank test. Because multiple related outcome dimensions were compared between the two models, the Holm-Bonferroni procedure was applied within outcome families defined according to study stage and evaluator type to control the family-wise type I error rate. The five families were Stage 1 expert-rated clinical dimensions, Stage 1 readability outcomes, Stage 2 patient-rated dimensions, Stage 2 clinician-rated dimensions, and Stage 3 expert-rated nursing care-plan dimensions. Both unadjusted and Holm-adjusted *p* values were reported in Supplementary Table S3. All tests were two-sided, and statistical significance after multiplicity correction was determined using the Holm-adjusted *p* values, with *p* < 0.05 considered statistically significant.

#### 2.8.2 Interrater reliability analysis

To evaluate the consistency and reliability of the ratings, intraclass correlation coefficients (ICCs) were calculated. Interrater reliability was assessed separately by study stage and rating dimension. For each rating dimension, the ICC was calculated using the original scores assigned by the three clinical experts to the same response unit. Scores from different rating dimensions were not combined. In Stage 2, each composite evaluation unit was assessed from the patient perspective only by the postpartum woman who had submitted the corresponding questions. Therefore, ICCs were not calculated for patient ratings.

A two-way random-effects, absolute-agreement model was used to calculate the reliability of a single rater, ICC (2,1), and the reliability of the mean ratings of the three raters, ICC (2,3). ICC (2,1) reflects the reliability of ratings provided by an individual expert, whereas ICC (2,3) reflects the reliability of the average score assigned by the three experts as a rating panel. For comparisons between models, the median of the three expert ratings was still used as the final expert score.

For each study stage, the response units generated by ChatGPT 5.5 and DeepSeek V3 were pooled for the expert interrater reliability analysis. In Stage 1, a single Chinese-language response from one generation was treated as the basic unit. For each rating dimension, 180 responses were included, comprising 90 responses from each model, and ICCs were calculated separately for the five rating dimensions. In Stage 2, the composite evaluation unit consisted of the three related questions submitted by each postpartum woman and the corresponding model responses. For each clinician-rated dimension, 240 evaluation units were included, comprising 120 units from each model, and ICCs were calculated separately for the five clinician-rated dimensions. In Stage 3, a single nursing care plan was treated as the basic unit. For each rating dimension, 240 nursing care plans were included, comprising 120 plans from each model, and ICCs were calculated separately for the seven rating dimensions.

The ICC values were interpreted according to the recommendations of Koo and Li. An ICC below 0.50 indicated poor agreement or reliability, 0.50–0.75 indicated moderate agreement or reliability, 0.75–0.90 indicated good agreement or reliability, and an ICC above 0.90 indicated excellent agreement or reliability (22). Full stage- and dimension-specific ICC results are presented in Supplementary Table S4.

### 2.9 Ethical approval

This study was reviewed and approved by the Ethics Committee of Xi’an People’s Hospital (Xi’an Fourth Hospital) (approval No. KJLL-Z-K-2025148). All postpartum women included in Stage 2 provided written informed consent before question collection and agreed to the use of their de-identified health consultation questions for this study. Stage 3 used retrospective electronic medical record data, and the requirement for informed consent was waived with approval from the ethics committee.

All real-world questions and clinical case data were rigorously de-identified before being entered into the large language models. Names, national identification numbers, inpatient and outpatient record numbers, contact details, bed numbers, exact dates of clinical encounters, names of healthcare professionals, and any other potentially identifying information were removed. Only the minimum clinical information necessary to complete the research tasks was retained.

Model-generated responses were used exclusively for research evaluation and were not provided to postpartum women as formal medical or nursing advice or used to inform actual clinical decisions. In Stage 2, participants viewed the responses only during the study evaluation process after all model-identifying information had been removed. They were explicitly informed that the content could not replace professional medical advice.

## 3 Results

### 3.1 Characteristics of the postpartum consultation participants and retrospective cases

Stage 2 included 120 postpartum women, with a mean age of 30.64 ± 4.03 years and a mean postpartum interval of 17.23 ± 9.19 days. Of these participants, 63.3% had a college education or higher, 61.7% had undergone vaginal delivery, and 59.2% were primiparous. Thirty-six women (30.0%) had pregnancy- or postpartum-related conditions, mainly gestational diabetes mellitus, hypertensive disorders of pregnancy, anemia during pregnancy, and thyroid dysfunction.

Stage 3 included 120 retrospective cases. The mean maternal age was 32.48 ± 4.21 years, and the mean gestational age at delivery was 39.26 ± 1.44 weeks. Overall, 88.3% of the women had a college education or higher, and 74.2% were primiparous. Vaginal deliveries accounted for 75.0% of cases, including spontaneous vaginal delivery in 51.7% and forceps-assisted delivery in 23.3%, whereas cesarean delivery accounted for 25.0%. The mean neonatal birth weight was 3157.33 ± 441.93 g. A total of 105 cases (87.5%) had at least one pregnancy complication, delivery abnormality, or other perinatal risk factor. Further details are presented in Table 1.

**Table 1:** General characteristics of Stage 2 participants and Stage 3 retrospective cases.

Characteristic	Stage 2 (*n* = 120)	Stage 3 (*n* = 120)
Age, y	30.64 ± 4.03	32.48 ± 4.21
Associate degree or higher	76 (63.3)	106 (88.3)
Missing education data	—	3 (2.5)
Primiparous	71 (59.2)	89 (74.2)
Mode of delivery		
Vaginal delivery	74 (61.7)	90 (75.0)
Spontaneous vaginal delivery	—	62 (51.7)
Forceps-assisted delivery	—	28 (23.3)
Cesarean delivery	46 (38.3)	30 (25.0)
Postpartum day at consultation	17.23 ± 9.19	—
Gestational age at delivery, wk	—	39.26 ± 1.44
Preterm birth (<37 wk)	—	10 (8.3)
Pregnancy- or postpartum-related condition	36 (30.0)	—
Any maternal or perinatal risk factor	—	105 (87.5)
Female infant	—	64 (53.3)
Birth weight, g	—	3157.33 ± 441.93
Apgar score 1 min	—	9.64 ± 0.73
5 min	—	9.87 ± 0.37
10 min	—	9.93 ± 0.26

Data are mean ± SD or n (%). “—” indicates not applicable or not collected. Education data were missing for 3 Stage 3 cases; education percentages were calculated using the full Stage 3 sample (n = 120) as the denominator. Maternal or perinatal risk factors included pregnancy complications, premature rupture of membranes, fetal distress, abnormal delivery, and other documented risks. Apgar indicates Appearance, Pulse, Grimace, Activity, and Respiration; SD, standard deviation.

### 3.2 Overall performance of the two large language models on guideline-based clinical questions

Stage 1 included 30 guideline-based clinical questions, each of which was independently submitted to both models three times. Each model generated 90 Chinese-language responses for clinical expert evaluation and Chinese readability analysis, as well as 90 English-language responses for English readability analysis. Expert-rated comparisons were based on 90 paired Chinese-language responses.

ChatGPT 5.5 received a higher correctness score than DeepSeek V3, with mean scores of 4.00 ± 0.00 and 3.88 ± 0.33, respectively (Holm-adjusted *p* = 0.002). DeepSeek V3 received a higher completeness score than ChatGPT 5.5, with mean scores of 4.63 ± 0.61 and 3.99 ± 0.49, respectively (Holm-adjusted *p* < 0.001). The guideline consistency scores were 3.89 ± 0.44 for ChatGPT 5.5 and 3.88 ± 0.33 for DeepSeek V3, with no statistically significant difference between the models (Holm-adjusted *p* = 0.857).

DeepSeek V3 also received higher scores than ChatGPT 5.5 for safety, with mean scores of 4.48 ± 0.57 and 4.24 ± 0.43, respectively (Holm-adjusted *p* = 0.001), and for clinical management appropriateness, with mean scores of 4.40 ± 0.75 and 3.91 ± 0.55, respectively (Holm-adjusted *p* < 0.001). Comparisons of the two models across the five expert-rated dimensions are shown in Figure 2.

**Figure 2:**
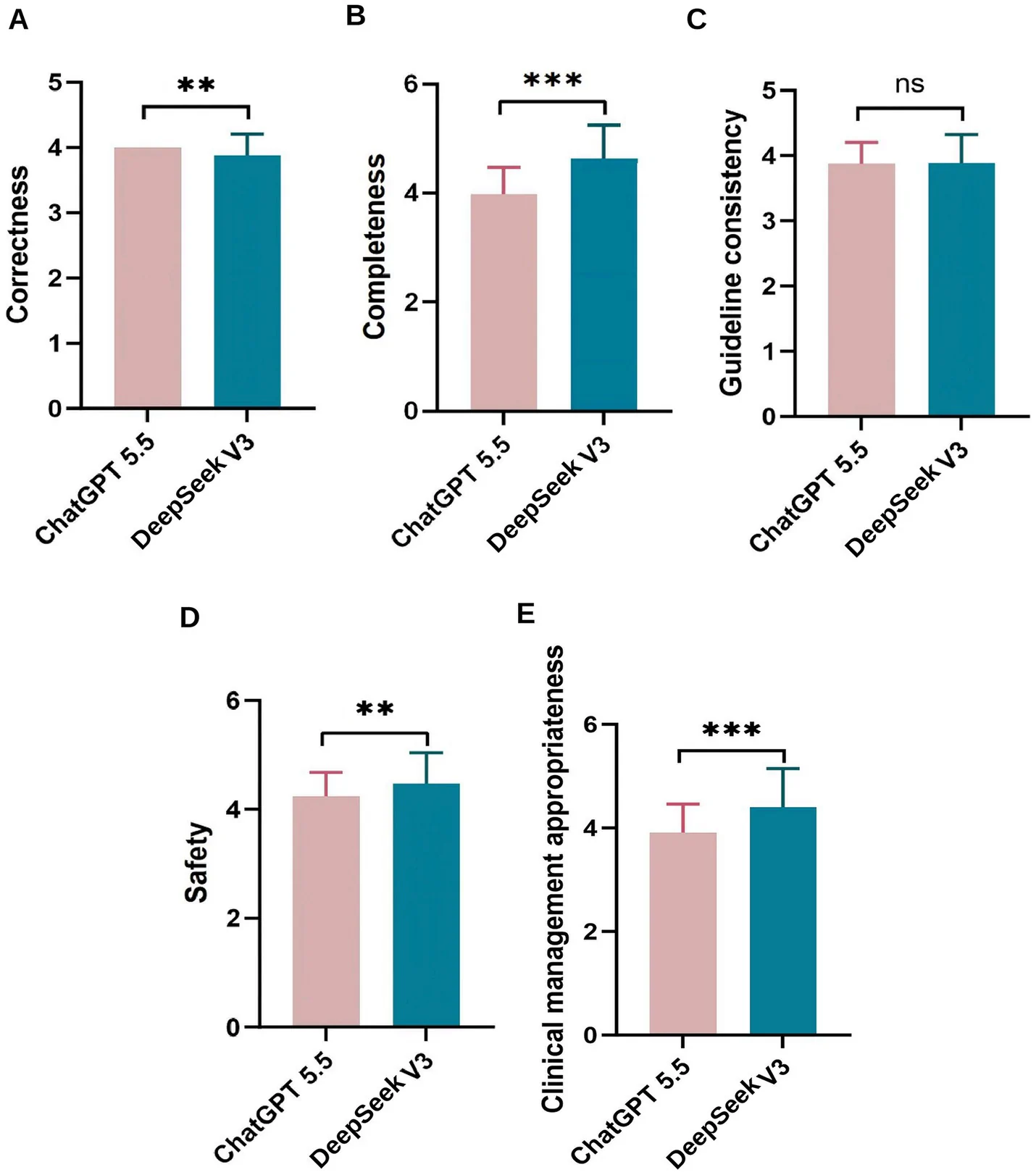
Expert-rated responses to guideline-based postpartum questions. **(A)** Correctness; **(B)** Completeness; **(C)** Guideline consistency; **(D)** Safety; and **(E)** Clinical management appropriateness. Values are presented as mean ± SD. Significance annotations are based on Holm-adjusted *p* values. **p < 0.01; ***p < 0.001; ns, not significant.

Readability-related measures showed that DeepSeek V3 had higher Chinese readability difficulty scores, recommended reading ages, and English grade levels than ChatGPT 5.5. The respective values were 14.94 ± 0.63 versus 13.68 ± 0.59 (Holm-adjusted *p* < 0.001), 14.97 ± 0.74 years versus 13.58 ± 0.76 years (Holm-adjusted *p* < 0.001), and 13.85 ± 3.32 versus 12.89 ± 2.29 (Holm-adjusted *p* = 0.016).

For the educational levels corresponding to the Chinese-language responses, 81 ChatGPT 5.5 responses (90.0%) were classified at the junior high school level and 9 (10.0%) at the senior high school level. In comparison, 21 DeepSeek V3 responses (23.3%) were classified at the junior high school level and 69 (76.7%) at the senior high school level. The distribution differed significantly between the two models (Holm-adjusted *p* < 0.001).

For English reading levels, the ChatGPT 5.5 responses were distributed as follows: 7th grade: fairly easy to read, 0 (0.0%); 8th & 9th grade: plain English, 3 (3.3%); 10th to 12th grade: fairly difficult, 29 (32.2%); college: difficult, 53 (58.9%); and college graduate: very difficult, 5 (5.6%). The corresponding distribution for DeepSeek V3 was 1 (1.1%), 5 (5.6%), 19 (21.1%), 43 (47.8%), and 22 (24.4%), respectively. The difference between the two models was statistically significant (Holm-adjusted *p* = 0.021). Differences in Chinese and English readability measures and the corresponding reading-level distributions are presented in Figure 3.

**Figure 3:**
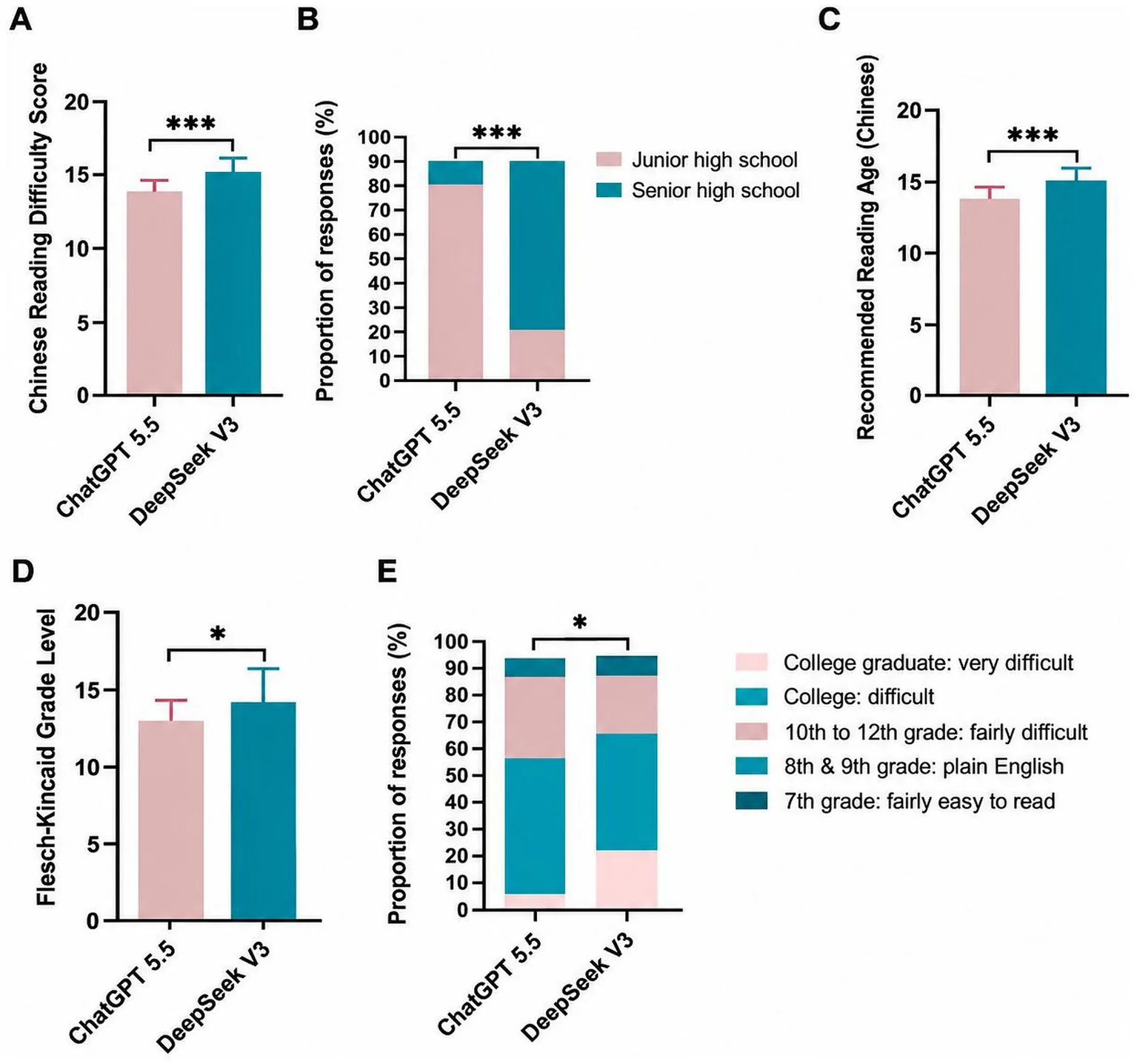
Readability analysis of Chinese and English responses generated by ChatGPT 5.5 and DeepSeek V3. **(A)** Chinese reading-difficulty score; **(B)** corresponding Chinese education level; **(C)** recommended Chinese reading age; **(D)** Flesch–Kincaid grade level; and **(E)** corresponding English reading-difficulty categories. Continuous variables are presented as mean ± SD and categorical variables are presented as proportions (%). Significance annotations are based on Holm-adjusted *p* values. *p < 0.05; ***p < 0.001.

In Stage 1, the single-rater reliability, ICC (2,1), ranged from 0.186 to 0.470, whereas the average-measures reliability for the three experts, ICC (2,3), ranged from 0.406 to 0.726. The ICC (2,3) values for completeness, guideline consistency, and clinical management appropriateness were 0.630, 0.591, and 0.726, respectively, indicating moderate reliability. The corresponding ICC (2,3) values for correctness and safety were 0.435 and 0.406, respectively, indicating poor reliability.

### 3.3 Overall performance of the two large language models on real-world consultation questions from postpartum women

In Stage 2, each model generated 120 composite evaluation units, yielding a total of 240 units. Responses were paired according to the questions submitted by the same postpartum woman, resulting in 120 matched consultation pairs.

Evaluations by postpartum women showed that ChatGPT 5.5 received higher scores than DeepSeek V3 for satisfaction, with mean scores of 4.30 ± 0.46 and 3.00 ± 0.00, respectively, and for usefulness, with mean scores of 4.22 ± 0.41 and 3.00 ± 0.00, respectively. Both differences remained statistically significant after multiplicity correction (both Holm-adjusted *p* < 0.001). The understandability scores were 4.03 ± 0.16 for ChatGPT 5.5 and 4.00 ± 0.00 for DeepSeek V3, with no statistically significant difference between the models (Holm-adjusted *p* = 0.083).

Clinician evaluations showed that ChatGPT 5.5 received higher scores than DeepSeek V3 for correctness, completeness, helpfulness, and safety. The respective mean scores were 4.75 ± 0.43 versus 3.35 ± 0.48, 4.68 ± 0.47 versus 3.00 ± 0.00, 4.71 ± 0.46 versus 3.00 ± 0.00, and 4.90 ± 0.30 versus 3.35 ± 0.48. All differences remained statistically significant after multiplicity correction (all Holm-adjusted *p* < 0.001). DeepSeek V3 received a higher clinician-rated readability score than ChatGPT 5.5, with mean scores of 4.60 ± 0.49 and 4.02 ± 0.13, respectively (Holm-adjusted *p* < 0.001). Patient- and clinician-rated performance across all dimensions is presented in Figure 4.

**Figure 4:**
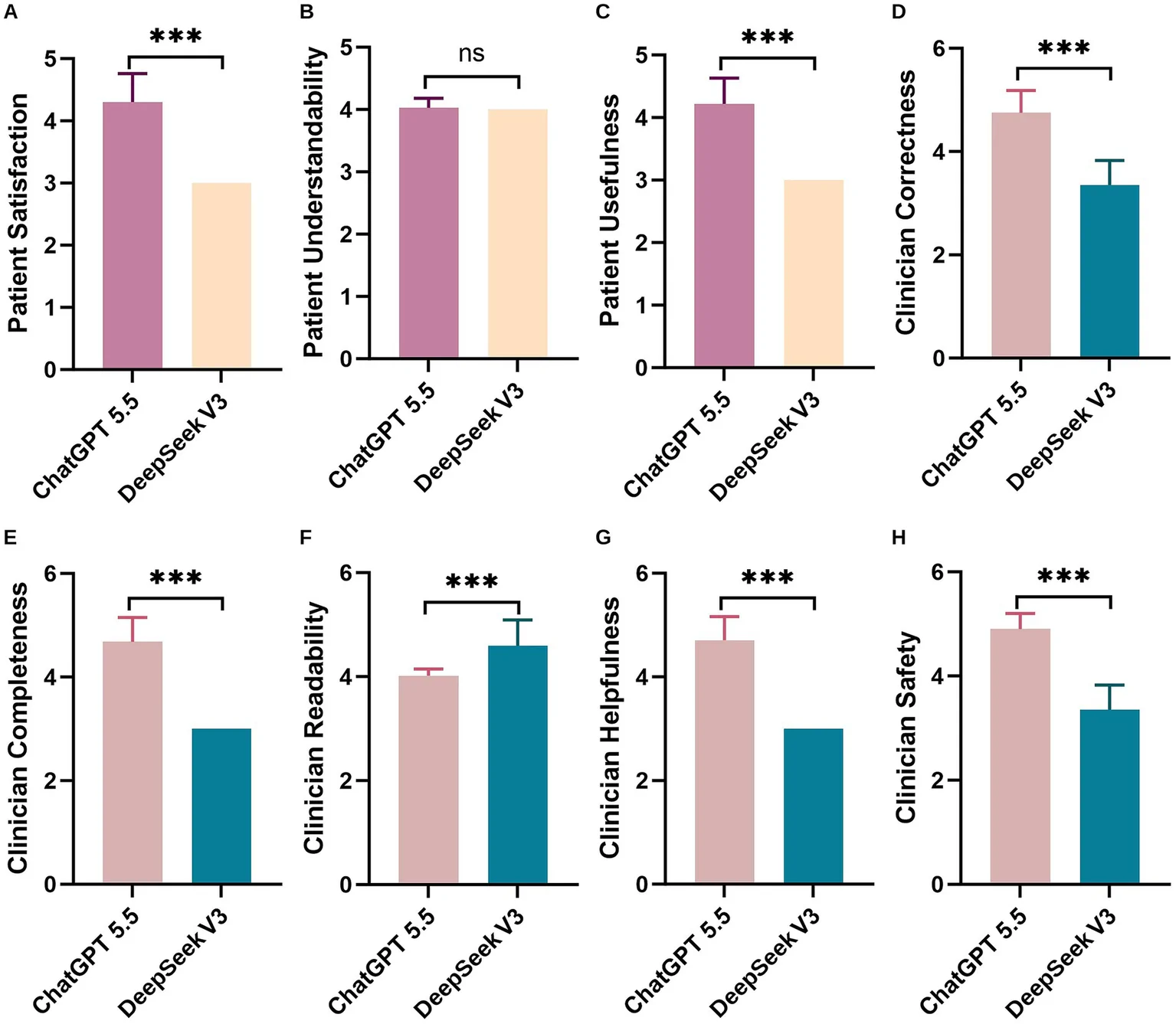
Patient and clinician ratings of postpartum consultation responses. **(A)** Patient satisfaction; **(B)** patient understandability; **(C)** patient usefulness; **(D)** clinician correctness; **(E)** clinician completeness; **(F)** clinician readability; **(G)** clinician helpfulness; and **(H)** clinician safety. Values are presented as mean ± SD. Significance annotations are based on Holm-adjusted *p* values. ***p < 0.001; ns, not significant.

In Stage 2, the ICC (2,1) values for correctness, completeness, readability, helpfulness, and safety were 0.741, 0.826, 0.339, 0.791, and 0.740, respectively. The corresponding ICC (2,3) values were 0.895, 0.934, 0.606, 0.919, and 0.895. The average-measures reliability of the three experts was excellent for completeness and helpfulness, good for correctness and safety, and moderate for readability.

### 3.4 Overall performance of the two large language models in nursing care plan generation

Stage 3 included 120 real-world clinical cases. Each model generated 120 nursing care plans, yielding a total of 240 plans. The plans were paired according to the same case, resulting in 120 matched pairs for comparison.

ChatGPT 5.5 received higher scores than DeepSeek V3 for clinical appropriateness, comprehensiveness, actionability, safety, and triage appropriateness. The respective mean scores were 4.66 ± 0.48 versus 3.61 ± 0.49, 4.66 ± 0.48 versus 3.39 ± 0.49, 4.16 ± 0.37 versus 3.65 ± 0.48, 5.00 ± 0.00 versus 3.61 ± 0.49, and 5.00 ± 0.00 versus 3.27 ± 0.44. All differences remained statistically significant after multiplicity correction (all Holm-adjusted *p* < 0.001).

DeepSeek V3 received a higher individualization score than ChatGPT 5.5, with mean scores of 4.24 ± 0.88 and 4.07 ± 0.80, respectively (Holm-adjusted *p* = 0.021). The structure and clarity scores were 3.68 ± 0.47 for ChatGPT 5.5 and 3.70 ± 0.46 for DeepSeek V3, with no statistically significant difference between the models (Holm-adjusted *p* = 0.806). Comparisons of the nursing care plans generated by the two models across all evaluation dimensions are presented in Figure 5.

**Figure 5:**
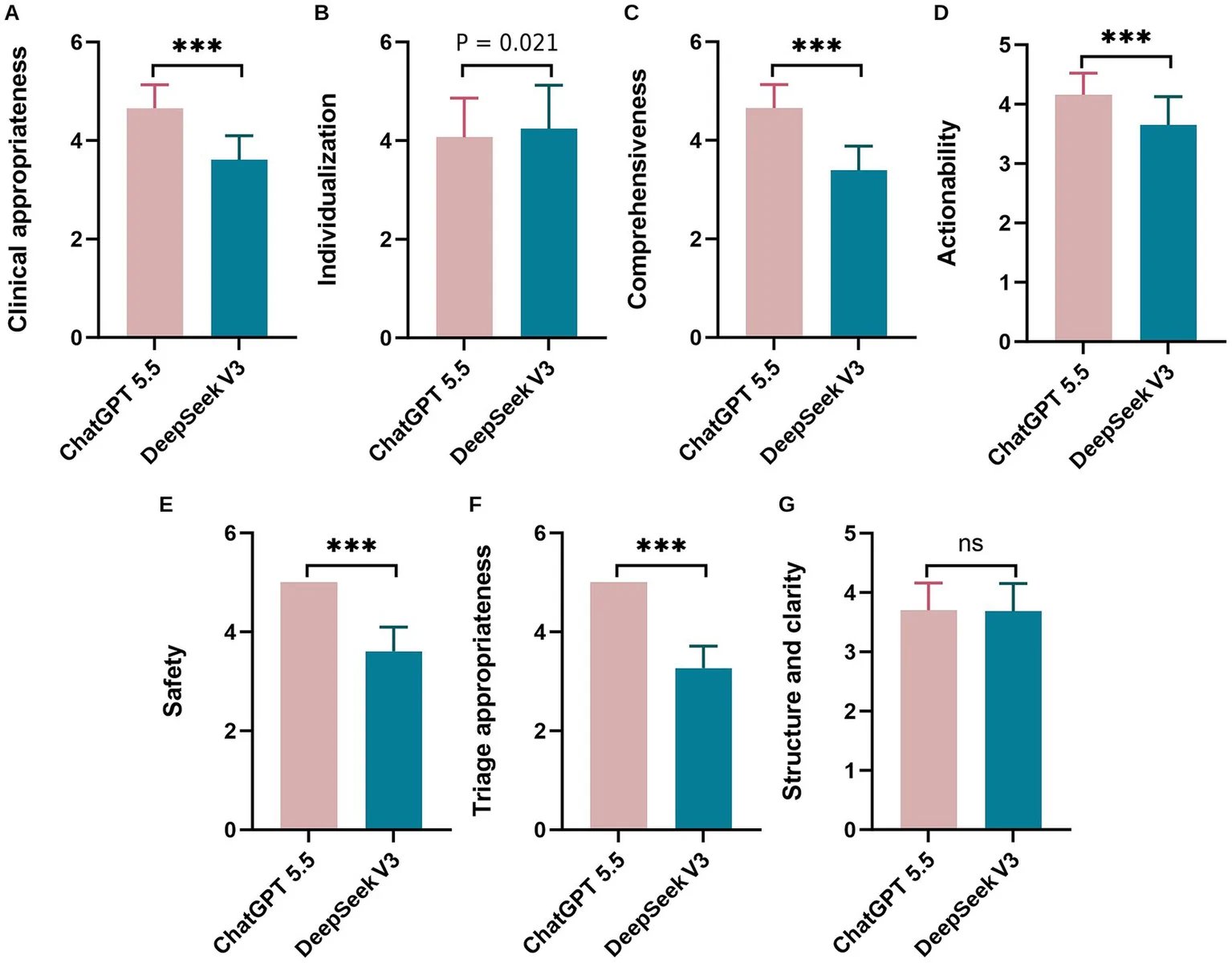
Expert ratings of model-generated postpartum nursing care plans. **(A)** Clinical appropriateness; **(B)** individualization; **(C)** comprehensiveness; **(D)** actionability; **(E)** safety; **(F)** triage appropriateness; and **(G)** structure and clarity. Values are presented as mean ± SD. Significance annotations are based on Holm-adjusted *p* values. For individualization, Holm-adjusted *p* = 0.021. ***p < 0.001; ns, not significant.

In Stage 3, the single-rater reliability, ICC (2,1), ranged from 0.446 to 0.749, whereas the average-measures reliability for the three experts, ICC (2,3), ranged from 0.708 to 0.899. The ICC (2,3) values for clinical appropriateness, individualization, comprehensiveness, safety, and triage appropriateness were 0.815, 0.875, 0.880, 0.841, and 0.899, respectively, indicating good reliability. The corresponding ICC (2,3) values for actionability and structure and clarity were 0.732 and 0.708, respectively, indicating moderate reliability.

## 4 Discussion

### 4.1 Main findings and study significance

This study compared the performance of ChatGPT 5.5 and DeepSeek V3 in postpartum maternal health scenarios using three types of tasks: guideline-based clinical questions, related real-world consultation questions raised by postpartum women concerning the same health topic, and nursing care plan generation based on real clinical cases. The main finding was that the relative strengths of the two models were clearly task dependent. DeepSeek V3 received higher scores for completeness, safety, and clinical management appropriateness when answering standardized questions in Stage 1. In contrast, ChatGPT 5.5 received higher scores for correctness, completeness, helpfulness, and safety in real-world postpartum consultations and for clinical appropriateness, comprehensiveness, actionability, safety, and triage appropriateness in case-based nursing care plan generation.

These findings suggest that predefined knowledge questions alone are insufficient to determine the clinical applicability of LLMs. Their ability to interpret real-world question phrasing, address multiple related concerns within the same health topic, and synthesize structured clinical case information also influences their practical utility in postpartum health education and nursing support. Previous studies have suggested that LLMs may support health education, information interpretation, and clinical documentation in obstetrics and gynecology. However, their clinical performance must be validated within specific tasks and care contexts (23, 24).

### 4.2 Standardized knowledge performance cannot substitute for evaluation in real-world clinical scenarios

In Stage 1, ChatGPT 5.5 received a higher correctness score, whereas DeepSeek V3 received higher scores for completeness, safety, and clinical management appropriateness. No statistically significant difference was observed in guideline consistency. These findings indicate that the quality of model responses to the same category of guideline-based questions is not determined by a single capability. Correctness reflects whether core medical facts are appropriately presented, whereas completeness reflects whether essential information is adequately covered. Safety and clinical management appropriateness are more directly related to the recognition of warning signs, recommendations for seeking medical care, and the avoidance of potential harm.

In an expert evaluation of responses to common obstetric questions, Peled et al. (25) found that ChatGPT performed well in terms of accuracy but remained limited in completeness and safety. Similarly, a comparative study by Lan et al. (26) based on real-world obstetric and gynecologic medical records showed limited agreement between consultation recommendations generated by ChatGPT-4 and those provided by physicians, with the overall quality of the model-generated recommendations being lower than that of physician-generated recommendations. Together with the findings of the present study, this evidence suggests that standardized knowledge testing may serve as one component of preliminary model assessment but cannot be used to infer that a model is ready to independently undertake clinical consultation or nursing decision-support tasks.

It should be noted that interrater reliability was relatively low for some dimensions in Stage 1, particularly correctness and safety. This does not necessarily indicate that the ratings were invalid. Rather, it suggests that the experts did not fully agree when distinguishing subtle differences among responses that all received relatively high scores. Because Stage 1 consisted mainly of standardized knowledge questions, overall model performance was high and ratings were concentrated toward the upper end of the scale. Under these conditions, experts may differ in distinguishing between good and better responses because of variations in clinical experience, sensitivity to potential risks, and interpretation of the rating criteria. The relatively low ICCs observed for some Stage 1 dimensions also indicate that the magnitude of between-model differences in these dimensions should be interpreted cautiously. Although use of the median rating from three independent experts reduces the influence of an individual evaluator, it does not eliminate uncertainty arising from disagreement among raters. Accordingly, Stage 1 findings for dimensions with poor interrater reliability should be regarded as exploratory rather than definitive evidence of model-level differences. By contrast, findings from the subsequent real-world tasks, for which average-measures interrater reliability was generally higher, may provide a more stable indication of the practical risks associated with using these models for clinical consultation and nursing support.

### 4.3 Readability should be evaluated using text-based measures, expert judgment, and user experience

In Stage 1, DeepSeek V3 had higher Chinese reading difficulty scores, recommended reading ages, and English grade levels than ChatGPT 5.5, indicating greater linguistic complexity. However, in the real-world consultations evaluated in Stage 2, clinicians assigned higher readability scores to DeepSeek V3, whereas postpartum women reported no statistically significant difference in understandability between the two models. This apparent discrepancy suggests that automated readability measures primarily capture the complexity of vocabulary, sentence length, and grammatical structure. They do not necessarily reflect users’ perceptions of information organization, logical presentation, or relevance to the question being asked.

Previous research has shown that medical information generated by LLMs for the general public may still require relatively advanced reading levels, highlighting the need to actively limit the complexity of terminology and sentence structures in patient education materials (27). In the context of postoperative gynecological consultation, AI chatbot responses may also exceed the health literacy level of the general patient population (28). Therefore, the quality of postpartum health education materials should not be assessed using a single readability formula alone. Patient understanding, clinician assessments of readability, and content correctness should be considered together.

### 4.4 Findings from real-world postpartum consultations highlight the clinical importance of content quality and safety

In the real-world postpartum consultation stage, ChatGPT 5.5 received higher patient ratings for satisfaction and usefulness and higher clinician ratings for correctness, completeness, helpfulness, and safety than DeepSeek V3. DeepSeek V3 received a higher score only for clinician-rated readability. These findings were not entirely consistent with the results of the standardized knowledge assessment in Stage 1, further suggesting that model performance in real-world contexts may be influenced by the conversational nature of the questions, the completeness of background information, and the complexity of multiple related concerns within the same health topic.

Ayers et al., in a study of questions posted on a public health consultation platform, found that responses generated by an artificial intelligence chatbot received high ratings for both quality and empathy (29). However, Beale et al., in a study of real postoperative gynecological questions, reported that although some chatbot responses approached physician responses in quality and accuracy, their reading level and clinical actionability still required careful assessment (28).

In the present study, the three related questions submitted by each postpartum woman on the same topic were combined into a single participant-level evaluation unit because the questions represented a coherent consultation topic and were not statistically independent. This approach reduced the risk of pseudoreplication and allowed overall usefulness across related concerns to be assessed. However, joint scoring may reduce the visibility of clinically important variation across individual questions; for example, a serious omission in one response could be diluted by adequate performance on the other two. In addition, because each question was entered in a separate conversation, the study did not simulate a genuine multi-turn dialogue. The Stage 2 findings therefore support evaluation of LLMs as assistive tools for drafting clinician responses, summarizing follow-up concerns, and preparing health education content, rather than as replacements for professional postpartum consultation.

### 4.5 Nursing care plan generation demonstrates potential for decision support but also underscores the need for human review

In the case-based nursing care plan stage, ChatGPT 5.5 received higher scores than DeepSeek V3 for clinical appropriateness, comprehensiveness, actionability, safety, and triage appropriateness, whereas DeepSeek V3 received a slightly higher score for individualization. Because the present study did not systematically code the types of errors, omissions, or reasoning pathways in each response, the specific mechanisms underlying these differences cannot be determined.

However, the central purpose of a postpartum nursing care plan extends beyond listing routine nursing interventions. It also requires the identification of risks that warrant timely escalation, including hemorrhage, infection, thromboembolism, hypertension-related symptoms, and psychological crises. Therefore, differences in safety and triage appropriateness have direct clinical relevance. Previous research has shown that LLMs can provide guideline-relevant information in clinical decision-support tasks based on structured cases, but may still generate recommendations that contain omissions, inaccuracies, or insufficient case specificity (30).

The findings of the present study suggest that model-generated nursing care plans may be useful for producing preliminary drafts, prompting key risk-assessment items, and supporting standardized clinical documentation. However, all such outputs should be reviewed and revised by obstetricians or midwives in conjunction with bedside assessment findings, changes in the patient’s condition, and institution-specific clinical workflows.

### 4.6 Interrater reliability findings support the use of multirater, multidimensional evaluation frameworks

In Stage 1, ICC (2, 3) values were relatively low for some dimensions. Interrater reliability was poor for correctness and safety and moderate for completeness, guideline consistency, and clinical management appropriateness. These differences may be related to the nature of the rating task. Responses to standardized guideline-based questions generally received high scores, making it difficult for evaluators to reach stable judgments regarding subtle differences in quality. By contrast, differences among responses to real-world consultations and case-based nursing care plan tasks were more pronounced and therefore easier for evaluators to distinguish. These findings underscore that reliability is dimension-specific and that model comparisons should be interpreted in conjunction with the uncertainty of human ratings rather than solely by statistical significance.

Previous research on LLMs in healthcare has emphasized the importance of clearly reporting model versions, input information, prompts, evaluator characteristics, and evaluation criteria, as well as assessing model performance separately across different tasks (24). In the present study, three experienced clinical experts completed calibration before independently rating de-identified responses under blinded conditions, and both single-rater and average-measures reliability were reported. This approach may help reduce bias arising from reliance on a single evaluator’s perspective.

### 4.7 Implications for clinical implementation, validation, and safety

The present findings should not be interpreted as evidence that either model is ready for autonomous clinical deployment. Before LLM-generated content is integrated into postpartum care workflows, prospective clinical validation, local workflow testing, continuous performance monitoring, and clearly defined human oversight mechanisms are required. Patient-facing outputs should clearly state their scope and prominently identify warning signs that warrant escalation, including severe bleeding, high fever, chest tightness or shortness of breath, lower-extremity swelling or pain, severe headache, visual disturbances, and thoughts of self-harm. For healthcare professional-facing applications, LLMs may assist with organizing follow-up concerns, drafting health education materials, constructing preliminary nursing care-plan frameworks, and supporting initial triage, but final content should be reviewed and confirmed by appropriately qualified professionals.

Clinical implementation should also incorporate explicit escalation rules, audit trails, model-version documentation, privacy protection, access control, and mechanisms for identifying and reporting unsafe outputs. When clinical case information is entered into a model, de-identification and data minimization should be consistently applied. Because model behavior can change after platform or model updates, validation should be treated as an ongoing process rather than a one-time assessment. These safeguards are particularly important given continuing concerns regarding privacy, allocation of responsibility, regulatory compliance, and the traceability of erroneous outputs (31–35).

### 4.8 Strengths and limitations

The strengths of this study include the evaluation of three distinct task types—knowledge assessment, real-world consultation, and clinical case synthesis—within a single research framework; the inclusion of real-world related questions submitted by 120 postpartum women concerning the same health topic and 120 real clinical cases; and the incorporation of both clinical expert and postpartum user perspectives. In addition, the study simultaneously assessed content quality, readability, and the interrater reliability of clinical expert evaluations.

This study also has several limitations. First, it was conducted at a single center, and the real-world questions and clinical case data were obtained primarily from one geographic region and one healthcare institution. Differences in patient populations, care processes, and patterns of language use may therefore limit the generalizability of the findings.

Second, only two model versions that were publicly accessible at the time of testing were compared. Model algorithms, internet connectivity, and platform default settings may change over time; therefore, the findings cannot be directly generalized to other model versions or deployment environments.

Third, in Stage 1, each individual response was treated as the unit of analysis, although the three repeated responses to the same question may have been correlated. The findings were not further validated using question-level aggregated analyses or statistical models capable of accounting for within-question correlation. In Stages 2 and 3, only the first generated response was recorded. In addition, standardized role-based prompts were intentionally used to reduce task-interpretation variability and improve between-model comparability, but this design may itself have influenced model performance. The study therefore reflects performance under structured prompting conditions rather than unrestricted consumer prompting. Different role instructions, prompt wording, levels of contextual information, or zero-context patient queries could produce different results. Future studies should systematically compare neutral prompts, role-based prompts, and alternative prompt configurations.

Fourth, although the clinical experts underwent calibration and evaluated the responses under blinded conditions, their ratings remained subjective, and some Stage 1 dimensions showed low interrater reliability. The use of the median rating from three experts reduces the influence of an individual evaluator but does not eliminate uncertainty caused by disagreement. In Stage 2, the three related responses were evaluated jointly as a composite unit; this approach may have obscured clinically important variation across individual questions, including the possibility that a serious omission in one response could be diluted by adequate performance on the other two. Individual question-level clinical ratings were not available. In addition, each composite unit was assessed from the patient perspective only by the postpartum woman who submitted the corresponding questions, so interrater reliability for patient ratings could not be assessed. Future studies should incorporate both question-level safety assessments and participant-level composite evaluations and examine whether maternal educational level, health literacy, or postpartum stage influences user ratings.

Fifth, this study evaluated the quality of model-generated responses and nursing care plans but did not assess the actual effects of model use on delays in seeking care, clinical workload, patient anxiety, health outcomes, or adverse events. Future multicenter prospective studies should evaluate different models within real-world follow-up workflows under preregistered prompting protocols and controlled model-version conditions, while incorporating clinical outcomes, implementation costs, and safety events into the evaluation framework.

## 5 Conclusion

In conclusion, both ChatGPT 5.5 and DeepSeek V3 demonstrated potential for application in postpartum maternal health settings, but LLM performance varied depending on the task, evaluation metric, and use case. ChatGPT 5.5 received higher scores across most key clinical dimensions in real-world postpartum consultations and case-based nursing care-plan generation, whereas DeepSeek V3 received higher scores for completeness and safety in some standardized responses, clinician-rated readability in real-world consultations, and individualization in care-plan generation. At present, LLMs are better suited for use as clinically reviewed tools for health education and nursing support than as independent sources of clinical guidance. Safe implementation depends on standardized and transparently reported prompting protocols, review by qualified professionals, protection of patient privacy, and ongoing validation in real-world settings.

## Data Availability

The de-identified model inputs, original model outputs, and derived ratings used in the analyses are provided in the supplementary files. The underlying source electronic medical records, original clinical records, and any direct or indirect identifiers are not publicly available. Additional de-identified data may be obtained from the corresponding author upon reasonable request, subject to approval by Xi’an People’s Hospital (Xi’an Fourth Hospital) and the applicable ethics and privacy requirements. Requests to access the datasets should be directed to Lei Liu, liulei2026@yeah.net.
