# Supplementary material for: Beyond accuracy: a three-stage evaluation of large language models for postpartum health consultation and nursing decision support
Source: Front Public Health. 2026 Oct 20;14:1961647. doi: 10.3389/fpubh.2026.1961647 (PMC13727638; doi:10.3389/fpubh.2026.1961647)
Supplement: Supplementary file 1 [file Table_1.docx]

**Supplementary Table S1. Operational definitions and scoring criteria for the three study stages**

All domains were rated on a 5-point Likert scale, with higher scores indicating better performance. Stage 1 clinical expert ratings were applied to Chinese responses only; automated readability outcomes were evaluated separately.

**Panel A. Stage 1: Clinical expert rating of Chinese responses to guideline-based clinical questions**

| **Domain** | **Operational definition** | **Scoring criteria (1–5)** |
| --- | --- | --- |
| **Correctness** | Extent to which the response is factually accurate, clinically sound, and consistent with accepted obstetric and postpartum-care principles. | **1 =** Predominantly incorrect, misleading, or clinically unsound. **2 =** Several important factual or clinical errors. **3 =** Generally correct but includes a clinically relevant error, ambiguity, or imprecision. **4 =** Accurate overall, with only minor imprecision unlikely to affect interpretation. **5 =** Fully accurate, clinically sound, and free of meaningful factual errors. |
| **Completeness** | Extent to which the response covers the core assessment points, common causes, management principles, precautions, red flags, and referral indications relevant to the question. | **1 =** Omits most essential content and does not address the main question. **2 =** Addresses only a small proportion of essential content; major omissions remain. **3 =** Covers the main issue but omits one or more important elements. **4 =** Covers nearly all essential elements, with only minor omissions. **5 =** Comprehensively covers all essential assessment, management, safety, and referral elements. |
| **Guideline consistency** | Extent to which the response accords with recommendations from the prespecified authoritative guidelines and relevant Chinese maternal-health standards. | **1 =** Substantially conflicts with authoritative guideline recommendations. **2 =** Multiple important deviations from guideline-based practice. **3 =** Broadly aligned but includes unclear, weakly supported, or partially inconsistent recommendations. **4 =** Consistent with guidelines, with only minor wording or emphasis differences. **5 =** Fully consistent with the relevant authoritative guideline recommendations. |
| **Safety** | Extent to which the response avoids harmful, misleading, or delay-inducing advice and appropriately highlights uncertainty, warning signs, and the need for professional evaluation. | **1 =** Advice is likely to cause harm or delay necessary care. **2 =** Important safety concerns or major omissions in red flags and escalation advice. **3 =** No overtly dangerous advice, but safeguards or escalation advice are incomplete. **4 =** Safe overall, with only minor omissions in precautionary or escalation language. **5 =** Clearly safe; appropriately emphasizes red flags and professional evaluation. |
| **Clinical management appropriateness** | Extent to which the assessment and management approach is logical, clinically appropriate, and able to distinguish normal postpartum recovery from abnormal findings. | **1 =** Management is inappropriate, illogical, or potentially harmful. **2 =** Major deficiencies in assessment, prioritization, or management. **3 =** Partially appropriate but lacks clear prioritization or normal–abnormal differentiation. **4 =** Appropriate and logical, with only minor gaps. **5 =** Clear, clinically appropriate, well prioritized, and provides suitable management and escalation principles. |

**Panel B. Stage 2: Patient rating of the composite evaluation unit**

| **Domain** | **Operational definition** | **Scoring criteria (1–5)** |
| --- | --- | --- |
| **Satisfaction** | The patient’s overall level of satisfaction with the model’s responses to her three related postpartum questions. | **1 =** Very dissatisfied; the response does not meet her needs. **2 =** Dissatisfied; major expectations or concerns are unmet. **3 =** Partly satisfied; the response only partly meets expectations. **4 =** Satisfied; meets most expectations with minor shortcomings. **5 =** Very satisfied; fully meets or exceeds expectations. |
| **Understandability** | Extent to which the patient can understand the wording, explanations, and recommended actions without professional assistance. | **1 =** Very difficult or impossible to understand. **2 =** Difficult to understand; substantial clarification is required. **3 =** Partly understandable, but some wording or concepts remain unclear. **4 =** Easy to understand, with only minor unclear wording. **5 =** Completely clear and easy to understand without additional explanation. |
| **Usefulness** | Extent to which the response provides information or advice that the patient considers relevant and practically helpful for self-care or deciding when to seek care. | **1 =** Not useful or potentially misleading. **2 =** Limited practical value; does not adequately support self-care or decision-making. **3 =** Moderately useful but lacks some relevant or actionable guidance. **4 =** Useful and practical, with only minor limitations. **5 =** Highly useful, directly relevant, actionable, and supportive of appropriate decisions. |

**Panel C. Stage 2: Clinical expert rating of the composite evaluation unit**

| **Domain** | **Operational definition** | **Scoring criteria (1–5)** |
| --- | --- | --- |
| **Correctness** | Extent to which the combined responses to the patient’s three related questions are factually accurate and clinically appropriate. | **1 =** Predominantly incorrect or misleading. **2 =** Several important clinical errors. **3 =** Generally correct but includes a clinically relevant error or ambiguity. **4 =** Accurate overall, with only minor imprecision. **5 =** Fully accurate and clinically sound. |
| **Completeness** | Extent to which the combined responses address all three related questions and include essential explanations, self-care guidance, warning signs, and follow-up advice. | **1 =** Fails to address most questions or essential content. **2 =** Addresses the questions only partially; major omissions remain. **3 =** Addresses the main concerns but omits one or more important elements. **4 =** Addresses nearly all concerns, with only minor omissions. **5 =** Fully addresses all three questions and all essential clinical and safety content. |
| **Readability** | Extent to which the response is clearly written, logically organized, appropriately concise, and suitable for a postpartum patient. | **1 =** Disorganized, overly technical, or extremely difficult to read. **2 =** Poorly organized or unnecessarily complex; substantial revision is needed. **3 =** Generally readable but includes notable complexity, repetition, or unclear wording. **4 =** Clear and well organized, with only minor readability problems. **5 =** Exceptionally clear, concise, logically structured, and patient appropriate. |
| **Helpfulness** | Extent to which the response directly addresses the patient’s concerns and provides relevant, practical, and supportive guidance. | **1 =** Does not address the patient’s needs or provides no meaningful help. **2 =** Limited help and lacks important practical guidance. **3 =** Partly helpful but insufficiently tailored or actionable. **4 =** Helpful and practical, with only minor limitations. **5 =** Highly helpful, directly responsive, practical, and supportive. |
| **Safety** | Extent to which the response avoids unsafe advice and appropriately identifies red flags, care escalation, and situations requiring professional assessment. | **1 =** Contains unsafe advice or is likely to delay necessary care. **2 =** Important safety problems or major omissions in red flags and escalation advice. **3 =** No overtly harmful advice, but safety-netting is incomplete. **4 =** Safe overall, with only minor omissions in precautions or escalation advice. **5 =** Fully safe, with clear red flags, appropriate escalation advice, and no harmful recommendations. |

**Panel D. Stage 3: Clinical expert rating of model-generated postpartum maternal nursing care plans**

| **Domain** | **Operational definition** | **Scoring criteria (1–5)** |
| --- | --- | --- |
| **Clinical appropriateness** | Extent to which the care plan is consistent with postpartum maternal-care principles, clinical routines, and the priorities indicated by the case. | **1 =** Clinically inappropriate or potentially harmful. **2 =** Major problems in care priorities or recommended management. **3 =** Partially appropriate but includes important weaknesses or questionable recommendations. **4 =** Clinically appropriate overall, with only minor deficiencies. **5 =** Fully appropriate, clinically sound, and well aligned with case priorities. |
| **Individualization** | Extent to which the plan incorporates case-specific information, including postpartum timing, mode of delivery, symptoms, feeding status, comorbidities, and risk factors. | **1 =** Generic plan with no meaningful use of case information. **2 =** Minimal case adaptation; most important individual factors are ignored. **3 =** Some case-specific content is included, but important factors are not integrated. **4 =** Well individualized, with only minor missed opportunities for tailoring. **5 =** Highly individualized and consistently integrates all clinically relevant case features. |
| **Comprehensiveness** | Extent to which the plan covers wound or incision care, lochia, breasts and breastfeeding, elimination, pain, activity, nutrition, mental health, follow-up, and risk monitoring. | **1 =** Most essential care domains are absent. **2 =** Several major care domains are missing. **3 =** Covers the main domains but omits one or more important areas. **4 =** Covers nearly all relevant domains, with only minor omissions. **5 =** Comprehensively covers all relevant maternal-care and follow-up domains. |
| **Actionability** | Extent to which the recommendations are specific, clear, feasible, and directly implementable rather than vague or generic. | **1 =** Recommendations are unusable, vague, or inappropriate. **2 =** Mostly vague; provides little concrete guidance. **3 =** Some actionable steps are provided, but important instructions remain nonspecific. **4 =** Mostly specific and feasible, with only minor ambiguity. **5 =** Consistently specific, clear, feasible, and readily implementable. |

**Panel D. Stage 3: Clinical expert rating of model-generated postpartum maternal nursing care plans (continued)**

| **Domain** | **Operational definition** | **Scoring criteria (1–5)** |
| --- | --- | --- |
| **Safety** | Extent to which the care plan avoids dangerous or erroneous recommendations and includes appropriate monitoring and safety-netting. | **1 =** Contains dangerous recommendations or serious safety failures. **2 =** Important safety concerns or critical monitoring omissions. **3 =** No overtly dangerous advice, but monitoring or precautions are incomplete. **4 =** Safe overall, with only minor omissions. **5 =** Fully safe, with appropriate monitoring, precautions, and no harmful recommendations. |
| **Triage appropriateness** | Extent to which the plan assigns an appropriate level of care—home observation, routine follow-up, prompt clinical review, or emergency care—based on case risks and warning signs. | **1 =** Clearly inappropriate triage that may cause serious delay or harm. **2 =** Major under-triage or over-triage. **3 =** Generally reasonable but includes incomplete or poorly prioritized escalation advice. **4 =** Appropriate triage overall, with only minor limitations. **5 =** Accurately distinguishes urgency and provides clear, case-appropriate escalation instructions. |
| **Structure and clarity** | Extent to which the care plan is logically organized and easy for patients, family members, or clinicians to follow and use. | **1 =** Disorganized and very difficult to follow. **2 =** Poor structure; key information is difficult to locate. **3 =** Acceptable organization but some sections are unclear, repetitive, or poorly ordered. **4 =** Clear and logically organized, with only minor presentation problems. **5 =** Exceptionally clear, concise, logically sequenced, and easy to use. |

Note: In Stage 1, each Chinese response was rated independently by three clinical experts. In Stage 2, the three responses generated for one participant’s three related questions were treated as one composite evaluation unit; the participant and the three clinical experts rated that unit independently. In Stage 3, each model-generated nursing care plan was rated independently by three clinical experts. For clinical expert-rated domains, the median of the three clinical expert scores was used as the final score for model comparison.
