# Supplementary material for: Beyond accuracy: a three-stage evaluation of large language models for postpartum health consultation and nursing decision support
Source: Front Public Health. 2026 Oct 20;14:1961647. doi: 10.3389/fpubh.2026.1961647 (PMC13727638; doi:10.3389/fpubh.2026.1961647)
Supplement: Supplementary file 2 [file Table_10.docx]

**Supplementary Table S10. Stage 2 ChatGPT 5.5 patient-derived questions, responses, English translations, and ratings**

Chinese original responses and their English translations are presented together. Ratings were assigned once to each composite evaluation unit based on the original Chinese responses; the English translations were not used for ratings.

| **Model name / 模型名称** | **Evaluator / 评分者** | **Language / 语言** | **Model testing period / 模型测试期** | **Notes / 备注** |
| --- | --- | --- | --- | --- |
| ChatGPT 5.5 | Corresponding postpartum participant; three blinded clinical experts / 对应产妇；3名盲法临床专家 | Chinese with English translation / 中文及英文翻译 | 1 May to 30 June 2026 / 2026年5月1日至6月30日 | Individual sex, age, and dates were removed to protect participant confidentiality. / 为保护参与者隐私，已删除个体性别、年龄和日期。 |

| **Scope / 范围** | 120 participants for this model; each participant contributed three related questions (Q1–Q3). / 本文件包含该模型对应的120名参与者，每名参与者提供3个相关问题（Q1–Q3）。 |
| --- | --- |
| Recording and translation rule / 记录与翻译规则 | Chinese original content and English translations are shown together. English translations were generated using a web-based translation tool and manually corrected; they were provided only for international readership and were not used for ratings. / 中文原文与英文翻译置于同一记录块中。英文翻译由网页翻译工具生成并经人工校正，仅供国际读者阅读，不用于评分。 |
| **Scoring rule / 评分规则** | Each participant’s three related questions and the three corresponding model responses constituted one composite evaluation unit. Scores were assigned once to the composite unit based on the original Chinese responses; Chinese and English were not scored separately. / 同一参与者的3个相关问题及模型对应的3个回答构成1个复合评价单元。评分基于中文原始回答，对该复合单元整体评分1次，不对中英文分别评分。 |
| **Participant ratings / 产妇评分** | The corresponding postpartum participant directly rated satisfaction, understandability, and usefulness. / 对应产妇本人直接评价满意度、可理解性和实用性。 |
| **Clinical expert ratings / 临床专家评分** | Three blinded clinical experts independently rated correctness, completeness, readability, helpfulness, and safety; the displayed score is the median of the three ratings. / 3名盲法临床专家独立评价正确性、完整性、可读性、帮助性和安全性；表中分数为3名专家评分的中位数。 |

**Patient 01**

| **Patient ID / 患者编号** | **Group / 模型组别** | | | | | |
| --- | --- | --- | --- | --- | --- | --- |
| P01 | ChatGPT 5.5 | | | | | |
| **General model response or patient education material (Chinese) / 模型总体回答或患者教育材料（中文）** | | | | | | |
| 顺产侧切后的轻中度疼痛多在产后数天逐渐减轻，1至2周内明显缓解，但个体差异较大。排尿时刺痛多与尿液刺激伤口有关，可以用流动清水由前向后轻轻冲洗，再用干净纸巾按压吸干，保持会阴清洁干燥。避免用力擦洗、坐浴过久或自行使用刺激性消毒液。若只是轻微红肿、无发热、疼痛逐渐减轻，可先观察并注意休息。若红肿加重、疼痛明显、伤口裂开、有脓性分泌物、恶露异味或体温达到38℃及以上，应尽快就医复查。以上建议仅适用于一般产褥期健康教育，不能替代线下面诊；若症状明显、持续加重或产妇感觉异常，应及时联系医生。 | | | | | | |
| **English translation of the Chinese general response / 中文总体回答英文翻译** | | | | | | |
| Mild to moderate pain after a vaginal birth with an episiotomy usually decreases over several days and often improves clearly within 1 to 2 weeks, although recovery varies. Stinging during urination is often caused by urine irritating the wound. You may gently rinse the area from front to back with running clean water, then pat it dry with clean tissue. Avoid rubbing, prolonged soaking, or using irritating disinfectants by yourself. If redness and swelling are mild, there is no fever, and pain is improving, you may observe carefully. Seek medical review if redness worsens, pain becomes stronger, the wound opens, pus appears, lochia smells foul, or your temperature reaches 38°C or higher. | | | | | | |
| **Item / 项目** | | **Q1 / 问题1** | | **Q2 / 问题2** | | **Q3 / 问题3** |
| **Patient question (Chinese original) / 患者原始问题（中文）** | | 我顺产侧切后伤口一直有点疼，这种疼痛一般多久会缓解？ | | 我现在上厕所时会阴伤口有刺痛感，可以用清水冲洗吗？ | | 如果侧切伤口有点红肿，但没有发烧，需要去医院看吗？ |
| **English translation of patient question / 患者问题英文翻译** | | How long does this kind of pain usually last after a vaginal birth with an episiotomy? | | When I urinate, my perineal wound stings. Can I rinse it with clean water? | | If the episiotomy wound is a little red and swollen but I do not have a fever, do I need to go to the hospital? |
| **Model response (Chinese) / 模型回答（中文）** | | 顺产侧切后的轻中度疼痛多在产后数天逐渐减轻，1至2周内明显缓解，但个体差异较大。排尿时刺痛多与尿液刺激伤口有关，可以用流动清水由前向后轻轻冲洗，再用干净纸巾按压吸干，保持会阴清洁干燥。 | | 避免用力擦洗、坐浴过久或自行使用刺激性消毒液。若只是轻微红肿、无发热、疼痛逐渐减轻，可先观察并注意休息。若红肿加重、疼痛明显、伤口裂开、有脓性分泌物、恶露异味或体温达到38℃及以上，应尽快就医复查。 | | 以上建议仅适用于一般产褥期健康教育，不能替代线下面诊；若症状明显、持续加重或产妇感觉异常，应及时联系医生。 |
| **English translation of model response / 模型回答英文翻译** | | Mild to moderate pain after a vaginal birth with an episiotomy usually decreases over several days and often improves clearly within 1 to 2 weeks, although recovery varies. Stinging during urination is often caused by urine irritating the wound. | | You may gently rinse the area from front to back with running clean water, then pat it dry with clean tissue. Avoid rubbing, prolonged soaking, or using irritating disinfectants by yourself. | | If redness and swelling are mild, there is no fever, and pain is improving, you may observe carefully. Seek medical review if redness worsens, pain becomes stronger, the wound opens, pus appears, lochia smells foul, or your temperature reaches 38°C or higher. |
| **Overall evaluation item / 整体评价项目** | | | **Score / 分数** | | **Comments / 备注** | |
| **Patient Satisfaction / 患者满意度** | | | 5 | |  | |
| **Patient Understandability / 患者可理解性** | | | 4 | |  | |
| **Patient Usefulness / 患者实用性** | | | 5 | |  | |
| **Clinician Correctness / 医生评分：正确性** | | | 5 | |  | |
| **Clinician Completeness / 医生评分：完整性** | | | 5 | |  | |
| **Clinician Readability / 医生评分：可读性** | | | 4 | |  | |
| **Clinician Helpfulness / 医生评分：帮助性** | | | 5 | |  | |
| **Clinician Safety / 医生评分：安全性** | | | 5 | |  | |

**Patient 02**

| **Patient ID / 患者编号** | **Group / 模型组别** | | | | | |
| --- | --- | --- | --- | --- | --- | --- |
| P02 | ChatGPT 5.5 | | | | | |
| **General model response or patient education material (Chinese) / 模型总体回答或患者教育材料（中文）** | | | | | | |
| 产后第5天恶露仍呈红色可以是正常恢复表现，尤其在活动、哺乳或休息不足后，量和颜色可能会有轻微波动。一般应逐渐由鲜红转为淡红、褐色，再变为黄色或白色，量也应慢慢减少。建议记录每天卫生巾更换次数、血块大小、气味和腹痛情况，避免过早劳累。若突然像月经量很多或更多，1小时浸透一片卫生巾，出现大血块、头晕心慌、下腹痛加重、发热或恶露臭味，不要在家等待，应尽快到医院排查晚期产后出血、胎盘胎膜残留或感染等情况。 | | | | | | |
| **English translation of the Chinese general response / 中文总体回答英文翻译** | | | | | | |
| On postpartum day 5, red lochia can still be part of normal recovery, especially if it becomes slightly heavier after activity, breastfeeding, or poor rest. In general, lochia should gradually change from bright red to pink or brown, then to yellowish or white, and the amount should slowly decrease. It is helpful to record how often pads are changed, the size of any clots, the odor, and abdominal pain. Avoid overexertion. If bleeding suddenly becomes very heavy, soaks a pad within an hour, contains large clots, or is accompanied by dizziness, palpitations, worsening lower abdominal pain, fever, or foul odor, do not wait at home. Go to the hospital to rule out delayed postpartum hemorrhage, retained tissue, or infection. | | | | | | |
| **Item / 项目** | | **Q1 / 问题1** | | **Q2 / 问题2** | | **Q3 / 问题3** |
| **Patient question (Chinese original) / 患者原始问题（中文）** | | 我产后第5天恶露还是红色的，这正常吗？ | | 恶露有时候多一点、有时候少一点，是不是说明子宫恢复不好？ | | 如果恶露突然变多，还带血块，应该怎么办？ |
| **English translation of patient question / 患者问题英文翻译** | | On postpartum day 5, my lochia is still red. Is this normal? | | Sometimes the lochia is heavier and sometimes it is lighter. Does that mean my uterus is not recovering well? | | What should I do if the lochia suddenly becomes heavier and contains blood clots? |
| **Model response (Chinese) / 模型回答（中文）** | | 产后第5天恶露仍呈红色可以是正常恢复表现，尤其在活动、哺乳或休息不足后，量和颜色可能会有轻微波动。 | | 一般应逐渐由鲜红转为淡红、褐色，再变为黄色或白色，量也应慢慢减少。建议记录每天卫生巾更换次数、血块大小、气味和腹痛情况，避免过早劳累。 | | 若突然像月经量很多或更多，1小时浸透一片卫生巾，出现大血块、头晕心慌、下腹痛加重、发热或恶露臭味，不要在家等待，应尽快到医院排查晚期产后出血、胎盘胎膜残留或感染等情况。 |
| **English translation of model response / 模型回答英文翻译** | | On postpartum day 5, red lochia can still be part of normal recovery, especially if it becomes slightly heavier after activity, breastfeeding, or poor rest. In general, lochia should gradually change from bright red to pink or brown, then to yellowish or white, and the amount should slowly decrease. | | It is helpful to record how often pads are changed, the size of any clots, the odor, and abdominal pain. Avoid overexertion. | | If bleeding suddenly becomes very heavy, soaks a pad within an hour, contains large clots, or is accompanied by dizziness, palpitations, worsening lower abdominal pain, fever, or foul odor, do not wait at home. Go to the hospital to rule out delayed postpartum hemorrhage, retained tissue, or infection. |
| **Overall evaluation item / 整体评价项目** | | | **Score / 分数** | | **Comments / 备注** | |
| **Patient Satisfaction / 患者满意度** | | | 4 | |  | |
| **Patient Understandability / 患者可理解性** | | | 4 | |  | |
| **Patient Usefulness / 患者实用性** | | | 4 | |  | |
| **Clinician Correctness / 医生评分：正确性** | | | 5 | |  | |
| **Clinician Completeness / 医生评分：完整性** | | | 4 | |  | |
| **Clinician Readability / 医生评分：可读性** | | | 4 | |  | |
| **Clinician Helpfulness / 医生评分：帮助性** | | | 4 | |  | |
| **Clinician Safety / 医生评分：安全性** | | | 5 | |  | |

**Patient 03**

| **Patient ID / 患者编号** | **Group / 模型组别** | | | | | |
| --- | --- | --- | --- | --- | --- | --- |
| P03 | ChatGPT 5.5 | | | | | |
| **General model response or patient education material (Chinese) / 模型总体回答或患者教育材料（中文）** | | | | | | |
| 剖宫产切口周围轻微发痒常见于伤口愈合和皮肤牵拉过程，但需要同时观察红、肿、热、痛和渗液变化。医生确认切口愈合良好后通常可以淋浴，洗澡时避免揉搓切口，不要长时间泡澡，洗后用干净毛巾轻轻按干，保持局部清洁干燥。少量清亮渗液且无明显疼痛时可先联系医生或门诊咨询，但如果渗液增多、变黄变绿、有异味、切口裂开、周围红肿扩大、疼痛加重或发热达到38℃及以上，应及时就医。不要自行挤压伤口或随意涂抹药物。以上建议仅适用于一般产褥期健康教育，不能替代线下面诊；若症状明显、持续加重或产妇感觉异常，应及时联系医生。 | | | | | | |
| **English translation of the Chinese general response / 中文总体回答英文翻译** | | | | | | |
| Mild itching around a cesarean incision is often related to wound healing and skin stretching, but you should also watch for increasing redness, swelling, warmth, pain, and drainage. After your clinician confirms that the incision is healing well, showering is usually allowed. During showering, avoid rubbing the incision and do not soak in a bath for a long time. Afterward, gently pat the area dry with a clean towel and keep it clean and dry. A small amount of clear drainage without obvious pain can be discussed with your clinician or postpartum clinic. Seek medical care promptly if drainage increases, becomes yellow or green, smells bad, the incision opens, redness spreads, pain worsens, or fever reaches 38°C or higher. Do not squeeze the wound or apply medicines without advice. | | | | | | |
| **Item / 项目** | | **Q1 / 问题1** | | **Q2 / 问题2** | | **Q3 / 问题3** |
| **Patient question (Chinese original) / 患者原始问题（中文）** | | 我剖宫产切口周围有点痒，是伤口在恢复还是感染了？ | | 剖宫产后多久可以洗澡？洗澡时切口需要怎么保护？ | | 如果切口有少量渗液，但是不太疼，需要马上就医吗？ |
| **English translation of patient question / 患者问题英文翻译** | | My cesarean incision feels a little itchy around it. Is it healing or infected? | | How soon can I take a shower after a cesarean birth, and how should I protect the incision while showering? | | If there is a small amount of discharge from the incision but it is not very painful, do I need medical care right away? |
| **Model response (Chinese) / 模型回答（中文）** | | 剖宫产切口周围轻微发痒常见于伤口愈合和皮肤牵拉过程，但需要同时观察红、肿、热、痛和渗液变化。医生确认切口愈合良好后通常可以淋浴，洗澡时避免揉搓切口，不要长时间泡澡，洗后用干净毛巾轻轻按干，保持局部清洁干燥。 | | 少量清亮渗液且无明显疼痛时可先联系医生或门诊咨询，但如果渗液增多、变黄变绿、有异味、切口裂开、周围红肿扩大、疼痛加重或发热达到38℃及以上，应及时就医。不要自行挤压伤口或随意涂抹药物。 | | 以上建议仅适用于一般产褥期健康教育，不能替代线下面诊；若症状明显、持续加重或产妇感觉异常，应及时联系医生。 |
| **English translation of model response / 模型回答英文翻译** | | Mild itching around a cesarean incision is often related to wound healing and skin stretching, but you should also watch for increasing redness, swelling, warmth, pain, and drainage. After your clinician confirms that the incision is healing well, showering is usually allowed. | | During showering, avoid rubbing the incision and do not soak in a bath for a long time. Afterward, gently pat the area dry with a clean towel and keep it clean and dry. A small amount of clear drainage without obvious pain can be discussed with your clinician or postpartum clinic. | | Seek medical care promptly if drainage increases, becomes yellow or green, smells bad, the incision opens, redness spreads, pain worsens, or fever reaches 38°C or higher. Do not squeeze the wound or apply medicines without advice. |
| **Overall evaluation item / 整体评价项目** | | | **Score / 分数** | | **Comments / 备注** | |
| **Patient Satisfaction / 患者满意度** | | | 5 | |  | |
| **Patient Understandability / 患者可理解性** | | | 4 | |  | |
| **Patient Usefulness / 患者实用性** | | | 4 | |  | |
| **Clinician Correctness / 医生评分：正确性** | | | 5 | |  | |
| **Clinician Completeness / 医生评分：完整性** | | | 5 | |  | |
| **Clinician Readability / 医生评分：可读性** | | | 4 | |  | |
| **Clinician Helpfulness / 医生评分：帮助性** | | | 5 | |  | |
| **Clinician Safety / 医生评分：安全性** | | | 5 | |  | |

**Patient 04**

| **Patient ID / 患者编号** | **Group / 模型组别** | | | | | |
| --- | --- | --- | --- | --- | --- | --- |
| P04 | ChatGPT 5.5 | | | | | |
| **General model response or patient education material (Chinese) / 模型总体回答或患者教育材料（中文）** | | | | | | |
| 产后一周乳房胀痛伴硬块，多见于乳汁淤积或乳腺管不通畅。处理重点是让乳汁顺利排出，而不是用力揉搓。喂奶前可以短时间温热敷，帮助乳汁流动，并让宝宝有效含接、增加患侧先吸或适当手挤奶。喂奶后若仍胀痛，可冷敷减轻肿胀。按摩应轻柔，从乳房外侧向乳头方向轻推，避免暴力按揉造成组织损伤。若出现乳房局部红、肿、热、痛，伴寒战、发热、明显乏力或症状24小时内不缓解，应考虑乳腺炎，及时就医评估是否需要药物治疗，同时通常仍可继续哺乳或排奶。 | | | | | | |
| **English translation of the Chinese general response / 中文总体回答英文翻译** | | | | | | |
| Breast engorgement with hard lumps about one week after birth is commonly related to milk stasis or blocked milk flow. The key is to help milk drain smoothly, not to massage forcefully. Before feeding, a brief warm compress may help milk flow, and effective latch should be encouraged. You may let the baby feed first on the affected side or express a small amount by hand if needed. After feeding, a cold compress can help reduce swelling and pain. Massage should be gentle, moving from the outer breast toward the nipple; forceful rubbing can injure breast tissue. If the breast becomes red, swollen, warm, and painful, or if chills, fever, marked fatigue, or no improvement within 24 hours occurs, mastitis should be considered. Seek medical evaluation, as medication may be needed, while breastfeeding or milk removal can usually continue. | | | | | | |
| **Item / 项目** | | **Q1 / 问题1** | | **Q2 / 问题2** | | **Q3 / 问题3** |
| **Patient question (Chinese original) / 患者原始问题（中文）** | | 我产后一周乳房很胀，还有硬块，是堵奶了吗？ | | 乳房胀痛时可以热敷还是冷敷？ | | 如果乳房红肿疼痛并且发烧，是不是乳腺炎？ |
| **English translation of patient question / 患者问题英文翻译** | | One week after birth, my breasts are very engorged and I can feel hard lumps. Is this a blocked duct? | | When my breasts are swollen and painful, should I use a warm compress or a cold compress? | | If the breast is red, swollen, painful, and I have a fever, could it be mastitis? |
| **Model response (Chinese) / 模型回答（中文）** | | 产后一周乳房胀痛伴硬块，多见于乳汁淤积或乳腺管不通畅。处理重点是让乳汁顺利排出，而不是用力揉搓。 | | 喂奶前可以短时间温热敷，帮助乳汁流动，并让宝宝有效含接、增加患侧先吸或适当手挤奶。喂奶后若仍胀痛，可冷敷减轻肿胀。 | | 按摩应轻柔，从乳房外侧向乳头方向轻推，避免暴力按揉造成组织损伤。若出现乳房局部红、肿、热、痛，伴寒战、发热、明显乏力或症状24小时内不缓解，应考虑乳腺炎，及时就医评估是否需要药物治疗，同时通常仍可继续哺乳或排奶。 |
| **English translation of model response / 模型回答英文翻译** | | Breast engorgement with hard lumps about one week after birth is commonly related to milk stasis or blocked milk flow. The key is to help milk drain smoothly, not to massage forcefully. Before feeding, a brief warm compress may help milk flow, and effective latch should be encouraged. | | You may let the baby feed first on the affected side or express a small amount by hand if needed. After feeding, a cold compress can help reduce swelling and pain. | | Massage should be gentle, moving from the outer breast toward the nipple; forceful rubbing can injure breast tissue. If the breast becomes red, swollen, warm, and painful, or if chills, fever, marked fatigue, or no improvement within 24 hours occurs, mastitis should be considered. Seek medical evaluation, as medication may be needed, while breastfeeding or milk removal can usually continue. |
| **Overall evaluation item / 整体评价项目** | | | **Score / 分数** | | **Comments / 备注** | |
| **Patient Satisfaction / 患者满意度** | | | 4 | |  | |
| **Patient Understandability / 患者可理解性** | | | 4 | |  | |
| **Patient Usefulness / 患者实用性** | | | 4 | |  | |
| **Clinician Correctness / 医生评分：正确性** | | | 4 | |  | |
| **Clinician Completeness / 医生评分：完整性** | | | 4 | |  | |
| **Clinician Readability / 医生评分：可读性** | | | 4 | |  | |
| **Clinician Helpfulness / 医生评分：帮助性** | | | 4 | |  | |
| **Clinician Safety / 医生评分：安全性** | | | 5 | |  | |

**Patient 05**

| **Patient ID / 患者编号** | **Group / 模型组别** | | | | | |
| --- | --- | --- | --- | --- | --- | --- |
| P05 | ChatGPT 5.5 | | | | | |
| **General model response or patient education material (Chinese) / 模型总体回答或患者教育材料（中文）** | | | | | | |
| 每次喂奶乳头明显疼痛，常与宝宝含接浅、姿势不合适或乳头反复摩擦有关。应让宝宝张大嘴，含住乳头和大部分乳晕，下巴贴近乳房，吸吮时不应只有乳头被拉扯。轻度破皮时一般可以继续母乳喂养，但要先纠正含接，喂后可挤少量乳汁涂在乳头并自然晾干，保持清洁。若需要药膏，应选择哺乳期可用产品，并按医护人员建议使用，喂奶前按说明处理。若疼痛越来越重、裂口出血明显、乳头有白斑或宝宝口腔疑似鹅口疮，应及时请助产士或医生评估。 | | | | | | |
| **English translation of the Chinese general response / 中文总体回答英文翻译** | | | | | | |
| Marked nipple pain at every feeding is often related to a shallow latch, poor positioning, or repeated friction. The baby should open the mouth wide, take the nipple and much of the areola into the mouth, and keep the chin close to the breast; feeding should not feel like the nipple alone is being pulled. With mild cracking, breastfeeding can usually continue, but latch should be corrected first. After feeding, you may express a little breast milk onto the nipple and let it air-dry, while keeping the area clean. If ointment is needed, use a product suitable for breastfeeding and follow medical advice and product instructions before feeding. Seek help from a midwife or doctor if pain worsens, bleeding cracks occur, white spots appear, or the baby may have oral thrush. | | | | | | |
| **Item / 项目** | | **Q1 / 问题1** | | **Q2 / 问题2** | | **Q3 / 问题3** |
| **Patient question (Chinese original) / 患者原始问题（中文）** | | 我每次喂奶乳头都很疼，是不是宝宝含接姿势不对？ | | 乳头有点破皮，还能继续母乳喂养吗？ | | 乳头皲裂可以自己涂药膏吗，会不会影响宝宝吃奶？ |
| **English translation of patient question / 患者问题英文翻译** | | My nipples hurt every time I breastfeed. Could this be due to the baby not latching properly? | | If my nipples are slightly cracked, can I continue breastfeeding? | | Can I apply ointment for nipple cracks by myself, and will it affect the baby during feeding? |
| **Model response (Chinese) / 模型回答（中文）** | | 每次喂奶乳头明显疼痛，常与宝宝含接浅、姿势不合适或乳头反复摩擦有关。应让宝宝张大嘴，含住乳头和大部分乳晕，下巴贴近乳房，吸吮时不应只有乳头被拉扯。 | | 轻度破皮时一般可以继续母乳喂养，但要先纠正含接，喂后可挤少量乳汁涂在乳头并自然晾干，保持清洁。 | | 若需要药膏，应选择哺乳期可用产品，并按医护人员建议使用，喂奶前按说明处理。若疼痛越来越重、裂口出血明显、乳头有白斑或宝宝口腔疑似鹅口疮，应及时请助产士或医生评估。 |
| **English translation of model response / 模型回答英文翻译** | | Marked nipple pain at every feeding is often related to a shallow latch, poor positioning, or repeated friction. The baby should open the mouth wide, take the nipple and much of the areola into the mouth, and keep the chin close to the breast; feeding should not feel like the nipple alone is being pulled. | | With mild cracking, breastfeeding can usually continue, but latch should be corrected first. After feeding, you may express a little breast milk onto the nipple and let it air-dry, while keeping the area clean. | | If ointment is needed, use a product suitable for breastfeeding and follow medical advice and product instructions before feeding. Seek help from a midwife or doctor if pain worsens, bleeding cracks occur, white spots appear, or the baby may have oral thrush. |
| **Overall evaluation item / 整体评价项目** | | | **Score / 分数** | | **Comments / 备注** | |
| **Patient Satisfaction / 患者满意度** | | | 4 | |  | |
| **Patient Understandability / 患者可理解性** | | | 4 | |  | |
| **Patient Usefulness / 患者实用性** | | | 4 | |  | |
| **Clinician Correctness / 医生评分：正确性** | | | 5 | |  | |
| **Clinician Completeness / 医生评分：完整性** | | | 4 | |  | |
| **Clinician Readability / 医生评分：可读性** | | | 4 | |  | |
| **Clinician Helpfulness / 医生评分：帮助性** | | | 4 | |  | |
| **Clinician Safety / 医生评分：安全性** | | | 5 | |  | |

**Patient 06**

| **Patient ID / 患者编号** | **Group / 模型组别** | | | | | |
| --- | --- | --- | --- | --- | --- | --- |
| P06 | ChatGPT 5.5 | | | | | |
| **General model response or patient education material (Chinese) / 模型总体回答或患者教育材料（中文）** | | | | | | |
| 产后第10天体温达到38℃，即使没有感冒症状，也建议重视并联系医生或到医院评估。产后发热可能与产褥感染、乳腺炎、泌尿系统感染、切口或会阴伤口感染、呼吸道感染等有关。请同时观察恶露气味和颜色、下腹痛、乳房红肿疼痛、尿痛尿频、切口渗液等情况。若发热伴恶露臭味、下腹痛加重、寒战、心跳明显加快或精神差，需要尽快就医，因为可能提示子宫内膜感染或其他感染。不要单纯依靠退烧药掩盖症状，也不要自行使用抗生素。以上建议仅适用于一般产褥期健康教育，不能替代线下面诊；若症状明显、持续加重或产妇感觉异常，应及时联系医生。 | | | | | | |
| **English translation of the Chinese general response / 中文总体回答英文翻译** | | | | | | |
| A temperature of 38°C on postpartum day 10 should be taken seriously even without cold symptoms. You should contact your clinician or go to the hospital for assessment. Postpartum fever may be related to puerperal infection, mastitis, urinary tract infection, cesarean or perineal wound infection, respiratory infection, or other causes. Observe the odor and color of lochia, lower abdominal pain, breast redness and pain, painful or frequent urination, and incision drainage. Fever together with foul-smelling lochia, worsening lower abdominal pain, chills, a very fast heartbeat, or feeling very unwell requires prompt medical care because it may suggest uterine infection or another infection. Do not rely only on fever-reducing medicine to hide symptoms, and do not use antibiotics by yourself. | | | | | | |
| **Item / 项目** | | **Q1 / 问题1** | | **Q2 / 问题2** | | **Q3 / 问题3** |
| **Patient question (Chinese original) / 患者原始问题（中文）** | | 我产后第10天突然发烧到38℃，但没有明显感冒症状，需要去医院吗？ | | 产后发热可能和哪些问题有关？ | | 如果发热同时恶露有味道，是不是产褥感染？ |
| **English translation of patient question / 患者问题英文翻译** | | On postpartum day 10, I suddenly developed a fever of 38℃ without obvious cold symptoms. Do I need to go to the hospital? | | What problems can postpartum fever be related to? | | If fever occurs together with foul-smelling lochia, could it be puerperal infection? |
| **Model response (Chinese) / 模型回答（中文）** | | 产后第10天体温达到38℃，即使没有感冒症状，也建议重视并联系医生或到医院评估。产后发热可能与产褥感染、乳腺炎、泌尿系统感染、切口或会阴伤口感染、呼吸道感染等有关。 | | 请同时观察恶露气味和颜色、下腹痛、乳房红肿疼痛、尿痛尿频、切口渗液等情况。若发热伴恶露臭味、下腹痛加重、寒战、心跳明显加快或精神差，需要尽快就医，因为可能提示子宫内膜感染或其他感染。不要单纯依靠退烧药掩盖症状，也不要自行使用抗生素。 | | 以上建议仅适用于一般产褥期健康教育，不能替代线下面诊；若症状明显、持续加重或产妇感觉异常，应及时联系医生。 |
| **English translation of model response / 模型回答英文翻译** | | A temperature of 38°C on postpartum day 10 should be taken seriously even without cold symptoms. You should contact your clinician or go to the hospital for assessment. | | Postpartum fever may be related to puerperal infection, mastitis, urinary tract infection, cesarean or perineal wound infection, respiratory infection, or other causes. Observe the odor and color of lochia, lower abdominal pain, breast redness and pain, painful or frequent urination, and incision drainage. | | Fever together with foul-smelling lochia, worsening lower abdominal pain, chills, a very fast heartbeat, or feeling very unwell requires prompt medical care because it may suggest uterine infection or another infection. Do not rely only on fever-reducing medicine to hide symptoms, and do not use antibiotics by yourself. |
| **Overall evaluation item / 整体评价项目** | | | **Score / 分数** | | **Comments / 备注** | |
| **Patient Satisfaction / 患者满意度** | | | 5 | |  | |
| **Patient Understandability / 患者可理解性** | | | 4 | |  | |
| **Patient Usefulness / 患者实用性** | | | 5 | |  | |
| **Clinician Correctness / 医生评分：正确性** | | | 5 | |  | |
| **Clinician Completeness / 医生评分：完整性** | | | 5 | |  | |
| **Clinician Readability / 医生评分：可读性** | | | 4 | |  | |
| **Clinician Helpfulness / 医生评分：帮助性** | | | 5 | |  | |
| **Clinician Safety / 医生评分：安全性** | | | 5 | |  | |

**Patient 07**

| **Patient ID / 患者编号** | **Group / 模型组别** | | | | | |
| --- | --- | --- | --- | --- | --- | --- |
| P07 | ChatGPT 5.5 | | | | | |
| **General model response or patient education material (Chinese) / 模型总体回答或患者教育材料（中文）** | | | | | | |
| 产后便秘和排便疼痛很常见，常与活动少、饮水不足、饮食纤维少、会阴疼痛或痔疮有关。建议增加温水摄入，多吃蔬菜、水果和全谷物，尽早适量下床活动，排便时不要久蹲和用力屏气。可以用温水清洁肛周，痔疮肿痛时在医生指导下选择外用药或软化大便药物，很多药物可在哺乳期安全使用，但不建议自行长期用泻药。短时间轻度用力一般不会让伤口裂开，但反复用力会加重疼痛和痔疮。若多日不排便、腹胀明显、便血多或疼痛剧烈，应就医处理。 | | | | | | |
| **English translation of the Chinese general response / 中文总体回答英文翻译** | | | | | | |
| Postpartum constipation and painful bowel movements are common and may be related to reduced activity, inadequate fluids, low fiber intake, perineal pain, or hemorrhoids. Drink more water, eat vegetables, fruit, and whole grains, and start gentle walking as tolerated. Avoid sitting on the toilet too long or holding your breath while straining. Clean the anal area with warm water. For swollen painful hemorrhoids, topical medicines or stool softeners may be used under medical guidance; many options are compatible with breastfeeding, but long-term self-use of laxatives is not recommended. Brief mild straining usually does not open a wound, but repeated straining can worsen pain and hemorrhoids. Seek medical care if there is no bowel movement for several days, marked bloating, significant rectal bleeding, or severe pain. | | | | | | |
| **Item / 项目** | | **Q1 / 问题1** | | **Q2 / 问题2** | | **Q3 / 问题3** |
| **Patient question (Chinese original) / 患者原始问题（中文）** | | 我产后好几天没大便，排便时很疼，应该怎么办？ | | 产后痔疮肿痛可以用药吗？会不会影响哺乳？ | | 便秘用力会不会影响会阴伤口或剖宫产切口恢复？ |
| **English translation of patient question / 患者问题英文翻译** | | I have not had a bowel movement for several days after birth, and it hurts when I try. What should I do? | | Can I use medicine for swollen painful hemorrhoids after birth? Will it affect breastfeeding? | | Will straining with constipation affect healing of the perineal wound or cesarean incision? |
| **Model response (Chinese) / 模型回答（中文）** | | 产后便秘和排便疼痛很常见，常与活动少、饮水不足、饮食纤维少、会阴疼痛或痔疮有关。建议增加温水摄入，多吃蔬菜、水果和全谷物，尽早适量下床活动，排便时不要久蹲和用力屏气。 | | 可以用温水清洁肛周，痔疮肿痛时在医生指导下选择外用药或软化大便药物，很多药物可在哺乳期安全使用，但不建议自行长期用泻药。 | | 短时间轻度用力一般不会让伤口裂开，但反复用力会加重疼痛和痔疮。若多日不排便、腹胀明显、便血多或疼痛剧烈，应就医处理。 |
| **English translation of model response / 模型回答英文翻译** | | Postpartum constipation and painful bowel movements are common and may be related to reduced activity, inadequate fluids, low fiber intake, perineal pain, or hemorrhoids. Drink more water, eat vegetables, fruit, and whole grains, and start gentle walking as tolerated. | | Avoid sitting on the toilet too long or holding your breath while straining. Clean the anal area with warm water. For swollen painful hemorrhoids, topical medicines or stool softeners may be used under medical guidance; many options are compatible with breastfeeding, but long-term self-use of laxatives is not recommended. | | Brief mild straining usually does not open a wound, but repeated straining can worsen pain and hemorrhoids. Seek medical care if there is no bowel movement for several days, marked bloating, significant rectal bleeding, or severe pain. |
| **Overall evaluation item / 整体评价项目** | | | **Score / 分数** | | **Comments / 备注** | |
| **Patient Satisfaction / 患者满意度** | | | 4 | |  | |
| **Patient Understandability / 患者可理解性** | | | 4 | |  | |
| **Patient Usefulness / 患者实用性** | | | 4 | |  | |
| **Clinician Correctness / 医生评分：正确性** | | | 5 | |  | |
| **Clinician Completeness / 医生评分：完整性** | | | 4 | |  | |
| **Clinician Readability / 医生评分：可读性** | | | 4 | |  | |
| **Clinician Helpfulness / 医生评分：帮助性** | | | 4 | |  | |
| **Clinician Safety / 医生评分：安全性** | | | 5 | |  | |

**Patient 08**

| **Patient ID / 患者编号** | **Group / 模型组别** | | | | | |
| --- | --- | --- | --- | --- | --- | --- |
| P08 | ChatGPT 5.5 | | | | | |
| **General model response or patient education material (Chinese) / 模型总体回答或患者教育材料（中文）** | | | | | | |
| 顺产后短时间小便刺痛可能与会阴伤口、尿道受压或轻微擦伤有关，但也要警惕泌尿系统感染和尿潴留。建议多饮水，排尿时可用温水轻轻冲洗会阴，排尿后保持局部干燥。若总感觉尿不干净、尿频尿急、尿痛加重、下腹胀痛或发热，应检查尿常规和残余尿量。产后偶尔漏尿常与盆底肌暂时松弛有关，不能简单等同于永久损伤，早期可在无明显疼痛和医生允许后做轻柔盆底肌训练。若漏尿持续、加重或影响生活，应在产后复查时做盆底评估并接受康复指导。 | | | | | | |
| **English translation of the Chinese general response / 中文总体回答英文翻译** | | | | | | |
| Mild stinging with urination soon after a vaginal birth may be caused by a perineal wound, urethral pressure, or minor irritation, but urinary tract infection and urinary retention should also be considered. Drink enough water, gently rinse the perineum with warm water during urination if needed, and keep the area dry afterward. If you always feel unable to empty the bladder, or have frequency, urgency, worsening pain, lower abdominal distention, or fever, urine testing and assessment of residual urine are needed. Occasional urine leakage after birth is often related to temporary pelvic floor weakness and does not necessarily mean permanent damage. Gentle pelvic floor exercises may begin when pain is controlled and your clinician allows. If leakage persists, worsens, or affects daily life, a pelvic floor assessment and rehabilitation guidance are recommended. | | | | | | |
| **Item / 项目** | | **Q1 / 问题1** | | **Q2 / 问题2** | | **Q3 / 问题3** |
| **Patient question (Chinese original) / 患者原始问题（中文）** | | 我顺产后小便时有点疼，这是正常的吗？ | | 产后总感觉尿不干净，需要检查吗？ | | 我产后偶尔漏尿，是不是盆底肌受损了？ |
| **English translation of patient question / 患者问题英文翻译** | | I have some pain when urinating after a vaginal birth. Is this normal? | | After birth, I always feel that I cannot empty my bladder completely. Do I need an examination? | | I occasionally leak urine after birth. Does this mean my pelvic floor muscles are damaged? |
| **Model response (Chinese) / 模型回答（中文）** | | 顺产后短时间小便刺痛可能与会阴伤口、尿道受压或轻微擦伤有关，但也要警惕泌尿系统感染和尿潴留。建议多饮水，排尿时可用温水轻轻冲洗会阴，排尿后保持局部干燥。 | | 若总感觉尿不干净、尿频尿急、尿痛加重、下腹胀痛或发热，应检查尿常规和残余尿量。 | | 产后偶尔漏尿常与盆底肌暂时松弛有关，不能简单等同于永久损伤，早期可在无明显疼痛和医生允许后做轻柔盆底肌训练。若漏尿持续、加重或影响生活，应在产后复查时做盆底评估并接受康复指导。 |
| **English translation of model response / 模型回答英文翻译** | | Mild stinging with urination soon after a vaginal birth may be caused by a perineal wound, urethral pressure, or minor irritation, but urinary tract infection and urinary retention should also be considered. Drink enough water, gently rinse the perineum with warm water during urination if needed, and keep the area dry afterward. | | If you always feel unable to empty the bladder, or have frequency, urgency, worsening pain, lower abdominal distention, or fever, urine testing and assessment of residual urine are needed. Occasional urine leakage after birth is often related to temporary pelvic floor weakness and does not necessarily mean permanent damage. | | Gentle pelvic floor exercises may begin when pain is controlled and your clinician allows. If leakage persists, worsens, or affects daily life, a pelvic floor assessment and rehabilitation guidance are recommended. |
| **Overall evaluation item / 整体评价项目** | | | **Score / 分数** | | **Comments / 备注** | |
| **Patient Satisfaction / 患者满意度** | | | 4 | |  | |
| **Patient Understandability / 患者可理解性** | | | 4 | |  | |
| **Patient Usefulness / 患者实用性** | | | 4 | |  | |
| **Clinician Correctness / 医生评分：正确性** | | | 4 | |  | |
| **Clinician Completeness / 医生评分：完整性** | | | 4 | |  | |
| **Clinician Readability / 医生评分：可读性** | | | 4 | |  | |
| **Clinician Helpfulness / 医生评分：帮助性** | | | 4 | |  | |
| **Clinician Safety / 医生评分：安全性** | | | 5 | |  | |

**Patient 09**

| **Patient ID / 患者编号** | **Group / 模型组别** | | | | | |
| --- | --- | --- | --- | --- | --- | --- |
| P09 | ChatGPT 5.5 | | | | | |
| **General model response or patient education material (Chinese) / 模型总体回答或患者教育材料（中文）** | | | | | | |
| 产后盆底肌训练通常可在身体状态允许、伤口疼痛不明显后从轻柔收缩开始，剖宫产或会阴伤口疼痛明显者应更谨慎。凯格尔运动的感觉应像主动收紧阴道和肛门、向上提起，保持正常呼吸，不要同时用力夹臀、收腹或憋气。可以先每次收缩3至5秒，再放松同样时间，逐渐增加次数，避免疲劳和疼痛。若训练时下腹痛、伤口牵拉或漏尿加重，应暂停并咨询。产后漏尿若超过数周仍无改善，或咳嗽、抱娃、运动时明显漏尿，建议在42天复查或盆底门诊进行评估，必要时接受规范康复。 | | | | | | |
| **English translation of the Chinese general response / 中文总体回答英文翻译** | | | | | | |
| Pelvic floor muscle exercises can usually start gently after birth when your general condition allows and wound pain is not significant. Women with cesarean or painful perineal wounds should be more cautious. A correct Kegel exercise should feel like tightening the vagina and anus and lifting upward, while breathing normally. You should not squeeze the buttocks, tighten the abdomen strongly, or hold your breath. Begin by contracting for 3 to 5 seconds and relaxing for the same length of time, then gradually increase repetitions without causing fatigue or pain. Stop and ask for advice if lower abdominal pain, wound pulling, or worsening leakage occurs. If urinary leakage does not improve after several weeks or is obvious with coughing, holding the baby, or exercise, pelvic floor assessment at the 42-day check-up or a rehabilitation clinic is recommended. | | | | | | |
| **Item / 项目** | | **Q1 / 问题1** | | **Q2 / 问题2** | | **Q3 / 问题3** |
| **Patient question (Chinese original) / 患者原始问题（中文）** | | 我产后什么时候可以开始做盆底肌训练？ | | 做凯格尔运动时应该怎么判断自己做对了？ | | 如果产后漏尿一直没有改善，需要去做盆底康复吗？ |
| **English translation of patient question / 患者问题英文翻译** | | When can I start pelvic floor muscle exercises after birth? | | How can I tell whether I am doing Kegel exercises correctly? | | If urinary leakage after birth does not improve, do I need pelvic floor rehabilitation? |
| **Model response (Chinese) / 模型回答（中文）** | | 产后盆底肌训练通常可在身体状态允许、伤口疼痛不明显后从轻柔收缩开始，剖宫产或会阴伤口疼痛明显者应更谨慎。凯格尔运动的感觉应像主动收紧阴道和肛门、向上提起，保持正常呼吸，不要同时用力夹臀、收腹或憋气。 | | 可以先每次收缩3至5秒，再放松同样时间，逐渐增加次数，避免疲劳和疼痛。 | | 若训练时下腹痛、伤口牵拉或漏尿加重，应暂停并咨询。产后漏尿若超过数周仍无改善，或咳嗽、抱娃、运动时明显漏尿，建议在42天复查或盆底门诊进行评估，必要时接受规范康复。 |
| **English translation of model response / 模型回答英文翻译** | | Pelvic floor muscle exercises can usually start gently after birth when your general condition allows and wound pain is not significant. Women with cesarean or painful perineal wounds should be more cautious. | | A correct Kegel exercise should feel like tightening the vagina and anus and lifting upward, while breathing normally. You should not squeeze the buttocks, tighten the abdomen strongly, or hold your breath. Begin by contracting for 3 to 5 seconds and relaxing for the same length of time, then gradually increase repetitions without causing fatigue or pain. | | Stop and ask for advice if lower abdominal pain, wound pulling, or worsening leakage occurs. If urinary leakage does not improve after several weeks or is obvious with coughing, holding the baby, or exercise, pelvic floor assessment at the 42-day check-up or a rehabilitation clinic is recommended. |
| **Overall evaluation item / 整体评价项目** | | | **Score / 分数** | | **Comments / 备注** | |
| **Patient Satisfaction / 患者满意度** | | | 4 | |  | |
| **Patient Understandability / 患者可理解性** | | | 4 | |  | |
| **Patient Usefulness / 患者实用性** | | | 4 | |  | |
| **Clinician Correctness / 医生评分：正确性** | | | 5 | |  | |
| **Clinician Completeness / 医生评分：完整性** | | | 5 | |  | |
| **Clinician Readability / 医生评分：可读性** | | | 4 | |  | |
| **Clinician Helpfulness / 医生评分：帮助性** | | | 5 | |  | |
| **Clinician Safety / 医生评分：安全性** | | | 5 | |  | |

**Patient 10**

| **Patient ID / 患者编号** | **Group / 模型组别** | | | | | |
| --- | --- | --- | --- | --- | --- | --- |
| P10 | ChatGPT 5.5 | | | | | |
| **General model response or patient education material (Chinese) / 模型总体回答或患者教育材料（中文）** | | | | | | |
| 顺产后若无明显出血、头晕、严重疼痛或医生限制，通常可以从短时间室内走动和轻柔散步开始，逐渐增加活动量。瑜伽、跑步和力量训练应等盆底、腹部和伤口恢复较好后再逐步恢复，产后复查后更稳妥。运动时以不疲劳、不疼痛、恶露不明显增多为原则。若活动后恶露短暂变红但很快减少，可先降低强度观察。若恶露明显增多、出现血块、下腹痛、头晕、心慌或切口疼痛，应停止运动并就医评估。产后恢复不要追求快速，应把休息、营养和盆底保护放在运动前面。 | | | | | | |
| **English translation of the Chinese general response / 中文总体回答英文翻译** | | | | | | |
| After an uncomplicated vaginal birth, if there is no heavy bleeding, dizziness, severe pain, or medical restriction, you may usually start with short indoor walking and gentle walks, then increase activity gradually. Yoga, running, and strength training should be resumed step by step after the pelvic floor, abdomen, and wounds have recovered better, and it is safer after the postpartum check-up. Exercise should not cause exhaustion, pain, or a clear increase in lochia. If lochia briefly becomes red after activity but soon decreases, reduce intensity and observe. If bleeding increases clearly, clots appear, lower abdominal pain, dizziness, palpitations, or incision pain occurs, stop exercising and seek medical assessment. Postpartum recovery should not be rushed; rest, nutrition, and pelvic floor protection come before intensive exercise. | | | | | | |
| **Item / 项目** | | **Q1 / 问题1** | | **Q2 / 问题2** | | **Q3 / 问题3** |
| **Patient question (Chinese original) / 患者原始问题（中文）** | | 我顺产后多久可以开始散步或做简单运动？ | | 产后多久可以恢复瑜伽、跑步或健身？ | | 如果运动后恶露变多，是不是说明运动太早了？ |
| **English translation of patient question / 患者问题英文翻译** | | How soon after a vaginal birth can I start walking or doing simple exercise? | | How soon after birth can I return to yoga, running, or fitness training? | | If lochia increases after exercise, does that mean I exercised too early? |
| **Model response (Chinese) / 模型回答（中文）** | | 顺产后若无明显出血、头晕、严重疼痛或医生限制，通常可以从短时间室内走动和轻柔散步开始，逐渐增加活动量。瑜伽、跑步和力量训练应等盆底、腹部和伤口恢复较好后再逐步恢复，产后复查后更稳妥。 | | 运动时以不疲劳、不疼痛、恶露不明显增多为原则。若活动后恶露短暂变红但很快减少，可先降低强度观察。 | | 若恶露明显增多、出现血块、下腹痛、头晕、心慌或切口疼痛，应停止运动并就医评估。产后恢复不要追求快速，应把休息、营养和盆底保护放在运动前面。 |
| **English translation of model response / 模型回答英文翻译** | | After an uncomplicated vaginal birth, if there is no heavy bleeding, dizziness, severe pain, or medical restriction, you may usually start with short indoor walking and gentle walks, then increase activity gradually. Yoga, running, and strength training should be resumed step by step after the pelvic floor, abdomen, and wounds have recovered better, and it is safer after the postpartum check-up. | | Exercise should not cause exhaustion, pain, or a clear increase in lochia. If lochia briefly becomes red after activity but soon decreases, reduce intensity and observe. | | If bleeding increases clearly, clots appear, lower abdominal pain, dizziness, palpitations, or incision pain occurs, stop exercising and seek medical assessment. Postpartum recovery should not be rushed; rest, nutrition, and pelvic floor protection come before intensive exercise. |
| **Overall evaluation item / 整体评价项目** | | | **Score / 分数** | | **Comments / 备注** | |
| **Patient Satisfaction / 患者满意度** | | | 4 | |  | |
| **Patient Understandability / 患者可理解性** | | | 4 | |  | |
| **Patient Usefulness / 患者实用性** | | | 4 | |  | |
| **Clinician Correctness / 医生评分：正确性** | | | 5 | |  | |
| **Clinician Completeness / 医生评分：完整性** | | | 4 | |  | |
| **Clinician Readability / 医生评分：可读性** | | | 4 | |  | |
| **Clinician Helpfulness / 医生评分：帮助性** | | | 4 | |  | |
| **Clinician Safety / 医生评分：安全性** | | | 4 | |  | |

**Patient 11**

| **Patient ID / 患者编号** | **Group / 模型组别** | | | | | |
| --- | --- | --- | --- | --- | --- | --- |
| P11 | ChatGPT 5.5 | | | | | |
| **General model response or patient education material (Chinese) / 模型总体回答或患者教育材料（中文）** | | | | | | |
| 喂奶时肚子一阵一阵疼，多数是哺乳刺激催产素释放，引起子宫收缩，属于常见的产后宫缩痛，尤其经产妇更明显。一般会随着产后天数增加逐渐减轻。可以热敷下腹、调整姿势、规律排尿，必要时在医生指导下使用哺乳期可用止痛药。需要警惕的是持续性下腹痛、压痛明显、恶露臭味、发热、寒战、出血增多或子宫压痛，这些可能提示感染或子宫复旧不良。判断子宫恢复不能只靠感觉，应结合恶露变化、腹痛情况和产后复查。若症状异常或恢复没有逐渐变好，应及时就医。 | | | | | | |
| **English translation of the Chinese general response / 中文总体回答英文翻译** | | | | | | |
| Abdominal pain in waves during breastfeeding is often caused by oxytocin release during nursing, which makes the uterus contract. This is a common postpartum afterpain and may be more obvious in women who have given birth before. It usually decreases over time. A warm compress on the lower abdomen, comfortable positioning, regular urination, and breastfeeding-compatible pain medicine under medical advice may help. Warning signs include persistent lower abdominal pain, marked tenderness, foul-smelling lochia, fever, chills, heavier bleeding, or uterine tenderness, which may suggest infection or poor uterine involution. Uterine recovery cannot be judged only by feeling; it should be considered together with lochia changes, pain, and postpartum examination. If symptoms are abnormal or not gradually improving, seek medical care. | | | | | | |
| **Item / 项目** | | **Q1 / 问题1** | | **Q2 / 问题2** | | **Q3 / 问题3** |
| **Patient question (Chinese original) / 患者原始问题（中文）** | | 我产后喂奶时肚子会一阵一阵疼，这是正常宫缩痛吗？ | | 产后下腹痛什么时候需要警惕感染或子宫恢复不好？ | | 怎么判断子宫有没有恢复好？ |
| **English translation of patient question / 患者问题英文翻译** | | When I breastfeed after birth, my abdomen hurts in waves. Is this normal afterpain? | | When should postpartum lower abdominal pain raise concern for infection or poor uterine recovery? | | How can I know whether my uterus is recovering well? |
| **Model response (Chinese) / 模型回答（中文）** | | 喂奶时肚子一阵一阵疼，多数是哺乳刺激催产素释放，引起子宫收缩，属于常见的产后宫缩痛，尤其经产妇更明显。一般会随着产后天数增加逐渐减轻。 | | 可以热敷下腹、调整姿势、规律排尿，必要时在医生指导下使用哺乳期可用止痛药。需要警惕的是持续性下腹痛、压痛明显、恶露臭味、发热、寒战、出血增多或子宫压痛，这些可能提示感染或子宫复旧不良。 | | 判断子宫恢复不能只靠感觉，应结合恶露变化、腹痛情况和产后复查。若症状异常或恢复没有逐渐变好，应及时就医。 |
| **English translation of model response / 模型回答英文翻译** | | Abdominal pain in waves during breastfeeding is often caused by oxytocin release during nursing, which makes the uterus contract. This is a common postpartum afterpain and may be more obvious in women who have given birth before. | | It usually decreases over time. A warm compress on the lower abdomen, comfortable positioning, regular urination, and breastfeeding-compatible pain medicine under medical advice may help. Warning signs include persistent lower abdominal pain, marked tenderness, foul-smelling lochia, fever, chills, heavier bleeding, or uterine tenderness, which may suggest infection or poor uterine involution. | | Uterine recovery cannot be judged only by feeling; it should be considered together with lochia changes, pain, and postpartum examination. If symptoms are abnormal or not gradually improving, seek medical care. |
| **Overall evaluation item / 整体评价项目** | | | **Score / 分数** | | **Comments / 备注** | |
| **Patient Satisfaction / 患者满意度** | | | 4 | |  | |
| **Patient Understandability / 患者可理解性** | | | 4 | |  | |
| **Patient Usefulness / 患者实用性** | | | 4 | |  | |
| **Clinician Correctness / 医生评分：正确性** | | | 5 | |  | |
| **Clinician Completeness / 医生评分：完整性** | | | 4 | |  | |
| **Clinician Readability / 医生评分：可读性** | | | 4 | |  | |
| **Clinician Helpfulness / 医生评分：帮助性** | | | 4 | |  | |
| **Clinician Safety / 医生评分：安全性** | | | 5 | |  | |

**Patient 12**

| **Patient ID / 患者编号** | **Group / 模型组别** | | | | | |
| --- | --- | --- | --- | --- | --- | --- |
| P12 | ChatGPT 5.5 | | | | | |
| **General model response or patient education material (Chinese) / 模型总体回答或患者教育材料（中文）** | | | | | | |
| 产后短时间想哭、敏感、担心自己做不好，可能是常见的产后情绪波动，常与激素变化、睡眠不足和照护压力有关，通常在家人支持和休息改善后逐渐缓解。产后抑郁则持续时间更长，表现为明显低落、兴趣下降、自责、失眠或嗜睡、食欲变化、难以照顾自己和宝宝等。若出现绝望感、不想活、自伤想法或担心会伤害宝宝，这是紧急信号，应立即告诉家人，不要独处，并尽快联系医生、心理科或急诊。情绪问题不是“矫情”，需要被认真对待，越早求助越容易恢复。 | | | | | | |
| **English translation of the Chinese general response / 中文总体回答英文翻译** | | | | | | |
| Crying easily, feeling sensitive, and worrying that you are not a good mother can be common postpartum emotional changes, often related to hormonal shifts, sleep deprivation, and caregiving stress. These feelings often improve with rest and family support. Postpartum depression usually lasts longer and may include persistent low mood, loss of interest, guilt, sleep or appetite changes, and difficulty caring for yourself or the baby. Feelings of hopelessness, not wanting to live, thoughts of self-harm, or fear that you may harm the baby are emergency warning signs. Tell a family member immediately, do not stay alone, and contact a doctor, mental health professional, or emergency service as soon as possible. Emotional problems are not weakness; they deserve serious attention, and early help supports recovery. | | | | | | |
| **Item / 项目** | | **Q1 / 问题1** | | **Q2 / 问题2** | | **Q3 / 问题3** |
| **Patient question (Chinese original) / 患者原始问题（中文）** | | 我产后总是想哭，觉得自己照顾不好孩子，这是正常的吗？ | | 产后情绪低落和产后抑郁有什么区别？ | | 如果我有时候觉得很绝望，甚至不想继续生活，应该怎么办？ |
| **English translation of patient question / 患者问题英文翻译** | | I often cry after birth and feel that I cannot take good care of my baby. Is this normal? | | What is the difference between postpartum blues and postpartum depression? | | What should I do if I sometimes feel very hopeless or even do not want to keep living? |
| **Model response (Chinese) / 模型回答（中文）** | | 产后短时间想哭、敏感、担心自己做不好，可能是常见的产后情绪波动，常与激素变化、睡眠不足和照护压力有关，通常在家人支持和休息改善后逐渐缓解。 | | 产后抑郁则持续时间更长，表现为明显低落、兴趣下降、自责、失眠或嗜睡、食欲变化、难以照顾自己和宝宝等。若出现绝望感、不想活、自伤想法或担心会伤害宝宝，这是紧急信号，应立即告诉家人，不要独处，并尽快联系医生、心理科或急诊。 | | 情绪问题不是“矫情”，需要被认真对待，越早求助越容易恢复。 |
| **English translation of model response / 模型回答英文翻译** | | Crying easily, feeling sensitive, and worrying that you are not a good mother can be common postpartum emotional changes, often related to hormonal shifts, sleep deprivation, and caregiving stress. These feelings often improve with rest and family support. | | Postpartum depression usually lasts longer and may include persistent low mood, loss of interest, guilt, sleep or appetite changes, and difficulty caring for yourself or the baby. Feelings of hopelessness, not wanting to live, thoughts of self-harm, or fear that you may harm the baby are emergency warning signs. | | Tell a family member immediately, do not stay alone, and contact a doctor, mental health professional, or emergency service as soon as possible. Emotional problems are not weakness; they deserve serious attention, and early help supports recovery. |
| **Overall evaluation item / 整体评价项目** | | | **Score / 分数** | | **Comments / 备注** | |
| **Patient Satisfaction / 患者满意度** | | | 4 | |  | |
| **Patient Understandability / 患者可理解性** | | | 5 | |  | |
| **Patient Usefulness / 患者实用性** | | | 4 | |  | |
| **Clinician Correctness / 医生评分：正确性** | | | 4 | |  | |
| **Clinician Completeness / 医生评分：完整性** | | | 4 | |  | |
| **Clinician Readability / 医生评分：可读性** | | | 5 | |  | |
| **Clinician Helpfulness / 医生评分：帮助性** | | | 4 | |  | |
| **Clinician Safety / 医生评分：安全性** | | | 5 | |  | |

**Patient 13**

| **Patient ID / 患者编号** | **Group / 模型组别** | | | | | |
| --- | --- | --- | --- | --- | --- | --- |
| P13 | ChatGPT 5.5 | | | | | |
| **General model response or patient education material (Chinese) / 模型总体回答或患者教育材料（中文）** | | | | | | |
| 产后睡眠差和持续紧张很常见，但如果明明很累仍长期睡不着、反复担心宝宝出事、无法放松或影响进食和照护，就需要关注产后焦虑。建议把夜间照护任务与家人分担，白天抓住宝宝睡觉时补眠，减少睡前刷手机和过度查信息，建立简单固定的睡前流程。可以把担心写下来，与家人和医护人员核对哪些需要处理，哪些属于过度担忧。焦虑会增加疲劳感，也可能影响泌乳反射和身体恢复。若焦虑持续超过两周、伴惊恐发作、明显失眠、情绪崩溃或自伤想法，应尽快寻求专业帮助。 | | | | | | |
| **English translation of the Chinese general response / 中文总体回答英文翻译** | | | | | | |
| Poor sleep and ongoing nervousness are common after birth, but if you are very tired yet cannot sleep for a long time, repeatedly fear that something will happen to the baby, cannot relax, or eating and caregiving are affected, postpartum anxiety should be considered. Share nighttime care with family members, nap when the baby sleeps during the day, reduce phone use and excessive online searching before bed, and create a simple bedtime routine. Write down worries and review them with family or health professionals to separate real problems from excessive fears. Anxiety can worsen fatigue and may affect milk let-down and physical recovery. If anxiety lasts more than two weeks, or is accompanied by panic attacks, severe insomnia, emotional breakdown, or self-harm thoughts, seek professional help promptly. | | | | | | |
| **Item / 项目** | | **Q1 / 问题1** | | **Q2 / 问题2** | | **Q3 / 问题3** |
| **Patient question (Chinese original) / 患者原始问题（中文）** | | 我产后睡眠很差，明明很累却睡不着，怎么办？ | | 我总担心宝宝出问题，整天很紧张，是产后焦虑吗？ | | 产后焦虑会不会影响身体恢复和母乳喂养？ |
| **English translation of patient question / 患者问题英文翻译** | | I sleep poorly after birth. I am very tired but still cannot fall asleep. What should I do? | | I am always worried that something will happen to my baby and feel nervous all day. Is this postpartum anxiety? | | Can postpartum anxiety affect physical recovery and breastfeeding? |
| **Model response (Chinese) / 模型回答（中文）** | | 产后睡眠差和持续紧张很常见，但如果明明很累仍长期睡不着、反复担心宝宝出事、无法放松或影响进食和照护，就需要关注产后焦虑。建议把夜间照护任务与家人分担，白天抓住宝宝睡觉时补眠，减少睡前刷手机和过度查信息，建立简单固定的睡前流程。 | | 可以把担心写下来，与家人和医护人员核对哪些需要处理，哪些属于过度担忧。 | | 焦虑会增加疲劳感，也可能影响泌乳反射和身体恢复。若焦虑持续超过两周、伴惊恐发作、明显失眠、情绪崩溃或自伤想法，应尽快寻求专业帮助。 |
| **English translation of model response / 模型回答英文翻译** | | Poor sleep and ongoing nervousness are common after birth, but if you are very tired yet cannot sleep for a long time, repeatedly fear that something will happen to the baby, cannot relax, or eating and caregiving are affected, postpartum anxiety should be considered. Share nighttime care with family members, nap when the baby sleeps during the day, reduce phone use and excessive online searching before bed, and create a simple bedtime routine. | | Write down worries and review them with family or health professionals to separate real problems from excessive fears. | | Anxiety can worsen fatigue and may affect milk let-down and physical recovery. If anxiety lasts more than two weeks, or is accompanied by panic attacks, severe insomnia, emotional breakdown, or self-harm thoughts, seek professional help promptly. |
| **Overall evaluation item / 整体评价项目** | | | **Score / 分数** | | **Comments / 备注** | |
| **Patient Satisfaction / 患者满意度** | | | 5 | |  | |
| **Patient Understandability / 患者可理解性** | | | 4 | |  | |
| **Patient Usefulness / 患者实用性** | | | 4 | |  | |
| **Clinician Correctness / 医生评分：正确性** | | | 5 | |  | |
| **Clinician Completeness / 医生评分：完整性** | | | 5 | |  | |
| **Clinician Readability / 医生评分：可读性** | | | 4 | |  | |
| **Clinician Helpfulness / 医生评分：帮助性** | | | 5 | |  | |
| **Clinician Safety / 医生评分：安全性** | | | 5 | |  | |

**Patient 14**

| **Patient ID / 患者编号** | **Group / 模型组别** | | | | | |
| --- | --- | --- | --- | --- | --- | --- |
| P14 | ChatGPT 5.5 | | | | | |
| **General model response or patient education material (Chinese) / 模型总体回答或患者教育材料（中文）** | | | | | | |
| 产后恢复性生活没有绝对统一时间，通常建议等恶露基本干净、伤口不痛、身体和心理都准备好后再考虑，42天复查后由医生确认恢复情况会更稳妥。若同房时疼痛，可能与会阴或剖宫产伤口、阴道干涩、盆底紧张、感染或心理紧张有关，不一定说明伤口没长好，但需要放慢节奏。哺乳期雌激素较低，阴道干涩较常见，可使用安全润滑剂并充分沟通。没有来月经也可能先排卵，因此仍需避孕。若同房后出血、疼痛明显、分泌物异常或下腹痛，应停止并就医。 | | | | | | |
| **English translation of the Chinese general response / 中文总体回答英文翻译** | | | | | | |
| There is no single fixed time for resuming sexual activity after birth. It is usually better to wait until lochia has mostly stopped, wounds are not painful, and you feel physically and emotionally ready. A postpartum check-up around 42 days can make this safer by confirming recovery. Pain during sex may be related to a perineal or cesarean wound, vaginal dryness, pelvic floor tension, infection, or anxiety; it does not always mean the wound has not healed, but the pace should be slowed. During breastfeeding, lower estrogen can make vaginal dryness common, so safe lubricant and good communication may help. Ovulation can occur before the first period, so contraception is still needed. Stop and seek care if bleeding, obvious pain, abnormal discharge, or lower abdominal pain occurs after sex. | | | | | | |
| **Item / 项目** | | **Q1 / 问题1** | | **Q2 / 问题2** | | **Q3 / 问题3** |
| **Patient question (Chinese original) / 患者原始问题（中文）** | | 产后多久可以恢复性生活？一定要等到42天复查后吗？ | | 如果恢复性生活时疼痛，是不是说明伤口没恢复好？ | | 哺乳期没有来月经，还需要避孕吗？ |
| **English translation of patient question / 患者问题英文翻译** | | How soon after birth can I resume sexual activity? Must I wait until the 42-day postpartum check-up? | | If sexual activity is painful after birth, does it mean the wound has not healed well? | | If I am breastfeeding and have not had my period, do I still need contraception? |
| **Model response (Chinese) / 模型回答（中文）** | | 产后恢复性生活没有绝对统一时间，通常建议等恶露基本干净、伤口不痛、身体和心理都准备好后再考虑，42天复查后由医生确认恢复情况会更稳妥。若同房时疼痛，可能与会阴或剖宫产伤口、阴道干涩、盆底紧张、感染或心理紧张有关，不一定说明伤口没长好，但需要放慢节奏。 | | 哺乳期雌激素较低，阴道干涩较常见，可使用安全润滑剂并充分沟通。 | | 没有来月经也可能先排卵，因此仍需避孕。若同房后出血、疼痛明显、分泌物异常或下腹痛，应停止并就医。 |
| **English translation of model response / 模型回答英文翻译** | | There is no single fixed time for resuming sexual activity after birth. It is usually better to wait until lochia has mostly stopped, wounds are not painful, and you feel physically and emotionally ready. | | A postpartum check-up around 42 days can make this safer by confirming recovery. Pain during sex may be related to a perineal or cesarean wound, vaginal dryness, pelvic floor tension, infection, or anxiety; it does not always mean the wound has not healed, but the pace should be slowed. During breastfeeding, lower estrogen can make vaginal dryness common, so safe lubricant and good communication may help. | | Ovulation can occur before the first period, so contraception is still needed. Stop and seek care if bleeding, obvious pain, abnormal discharge, or lower abdominal pain occurs after sex. |
| **Overall evaluation item / 整体评价项目** | | | **Score / 分数** | | **Comments / 备注** | |
| **Patient Satisfaction / 患者满意度** | | | 4 | |  | |
| **Patient Understandability / 患者可理解性** | | | 4 | |  | |
| **Patient Usefulness / 患者实用性** | | | 4 | |  | |
| **Clinician Correctness / 医生评分：正确性** | | | 5 | |  | |
| **Clinician Completeness / 医生评分：完整性** | | | 4 | |  | |
| **Clinician Readability / 医生评分：可读性** | | | 4 | |  | |
| **Clinician Helpfulness / 医生评分：帮助性** | | | 4 | |  | |
| **Clinician Safety / 医生评分：安全性** | | | 5 | |  | |

**Patient 15**

| **Patient ID / 患者编号** | **Group / 模型组别** | | | | | |
| --- | --- | --- | --- | --- | --- | --- |
| P15 | ChatGPT 5.5 | | | | | |
| **General model response or patient education material (Chinese) / 模型总体回答或患者教育材料（中文）** | | | | | | |
| 产后没有来月经并不代表不会怀孕，因为排卵可能早于第一次月经。哺乳期避孕应根据是否纯母乳、产后时间、是否有血栓风险、是否有高血压等情况选择。常用方法包括避孕套、单纯孕激素类避孕方法、宫内节育器等，含雌激素的复方避孕药一般不建议产后早期自行使用，尤其是血栓风险较高者。宫内节育器可在分娩后特定时间放置，也可在产后复查确认子宫恢复后放置，具体由医生判断。若有腹痛、发热、异常出血或疑似妊娠，应先检查再选择避孕方式。 | | | | | | |
| **English translation of the Chinese general response / 中文总体回答英文翻译** | | | | | | |
| Not having a period after birth does not mean you cannot become pregnant, because ovulation may occur before the first menstrual bleeding. Contraception during breastfeeding should be chosen according to whether breastfeeding is exclusive, how long it has been since birth, and whether there are risks such as thrombosis or high blood pressure. Common options include condoms, progestin-only methods, and intrauterine devices. Combined estrogen-containing pills are generally not recommended for self-use in the early postpartum period, especially when clot risk is higher. An intrauterine device may be placed at specific times after birth or after postpartum examination confirms uterine recovery; the exact timing should be decided by a clinician. If abdominal pain, fever, abnormal bleeding, or possible pregnancy occurs, you should be assessed before choosing a method. | | | | | | |
| **Item / 项目** | | **Q1 / 问题1** | | **Q2 / 问题2** | | **Q3 / 问题3** |
| **Patient question (Chinese original) / 患者原始问题（中文）** | | 产后还没来月经，会不会怀孕？ | | 哺乳期可以用哪些避孕方法？ | | 产后多久可以放置宫内节育器？ |
| **English translation of patient question / 患者问题英文翻译** | | If my period has not returned after birth, can I still become pregnant? | | What contraceptive methods can be used during breastfeeding? | | How soon after birth can an intrauterine device be placed? |
| **Model response (Chinese) / 模型回答（中文）** | | 产后没有来月经并不代表不会怀孕，因为排卵可能早于第一次月经。哺乳期避孕应根据是否纯母乳、产后时间、是否有血栓风险、是否有高血压等情况选择。 | | 常用方法包括避孕套、单纯孕激素类避孕方法、宫内节育器等，含雌激素的复方避孕药一般不建议产后早期自行使用，尤其是血栓风险较高者。 | | 宫内节育器可在分娩后特定时间放置，也可在产后复查确认子宫恢复后放置，具体由医生判断。若有腹痛、发热、异常出血或疑似妊娠，应先检查再选择避孕方式。 |
| **English translation of model response / 模型回答英文翻译** | | Not having a period after birth does not mean you cannot become pregnant, because ovulation may occur before the first menstrual bleeding. Contraception during breastfeeding should be chosen according to whether breastfeeding is exclusive, how long it has been since birth, and whether there are risks such as thrombosis or high blood pressure. | | Common options include condoms, progestin-only methods, and intrauterine devices. Combined estrogen-containing pills are generally not recommended for self-use in the early postpartum period, especially when clot risk is higher. | | An intrauterine device may be placed at specific times after birth or after postpartum examination confirms uterine recovery; the exact timing should be decided by a clinician. If abdominal pain, fever, abnormal bleeding, or possible pregnancy occurs, you should be assessed before choosing a method. |
| **Overall evaluation item / 整体评价项目** | | | **Score / 分数** | | **Comments / 备注** | |
| **Patient Satisfaction / 患者满意度** | | | 4 | |  | |
| **Patient Understandability / 患者可理解性** | | | 4 | |  | |
| **Patient Usefulness / 患者实用性** | | | 4 | |  | |
| **Clinician Correctness / 医生评分：正确性** | | | 5 | |  | |
| **Clinician Completeness / 医生评分：完整性** | | | 4 | |  | |
| **Clinician Readability / 医生评分：可读性** | | | 4 | |  | |
| **Clinician Helpfulness / 医生评分：帮助性** | | | 4 | |  | |
| **Clinician Safety / 医生评分：安全性** | | | 5 | |  | |

**Patient 16**

| **Patient ID / 患者编号** | **Group / 模型组别** | | | | | |
| --- | --- | --- | --- | --- | --- | --- |
| P16 | ChatGPT 5.5 | | | | | |
| **General model response or patient education material (Chinese) / 模型总体回答或患者教育材料（中文）** | | | | | | |
| 坐月子期间可以洗头、淋浴和保持个人清洁，所谓洗澡一定会落下“月子病”没有医学依据。关键是注意保暖、防滑、避免过度疲劳，洗后及时擦干头发和身体，不建议长时间泡澡或盆浴，尤其伤口未完全恢复时。产后可以使用空调，但温度不要过低，避免冷风直吹，房间要适当通风。饮食方面不应只吃油腻汤水，水果和蔬菜可以吃，有助于补充维生素、膳食纤维和预防便秘。若有腹泻、过敏或血糖问题，可根据个人情况调整种类和量。出现发热、伤口疼痛或异常出血时应就医。 | | | | | | |
| **English translation of the Chinese general response / 中文总体回答英文翻译** | | | | | | |
| During the postpartum confinement period, washing your hair, showering, and maintaining personal hygiene are allowed. The belief that bathing will inevitably cause a lasting postpartum illness has no medical basis. The key is to keep warm, avoid slipping, and avoid overexertion. Dry your hair and body promptly after washing. Prolonged soaking or tub baths are not recommended, especially before wounds have fully healed. Air conditioning can be used after birth, but the temperature should not be too low, direct cold air should be avoided, and the room should be ventilated appropriately. Diet should not rely only on oily soups. Fruit and vegetables are suitable and help provide vitamins, fiber, and constipation prevention. If diarrhea, allergy, or blood glucose problems occur, adjust choices and amounts individually. Seek care for fever, wound pain, or abnormal bleeding. | | | | | | |
| **Item / 项目** | | **Q1 / 问题1** | | **Q2 / 问题2** | | **Q3 / 问题3** |
| **Patient question (Chinese original) / 患者原始问题（中文）** | | 坐月子期间可以洗头洗澡吗？会不会落下月子病？ | | 产后可以吹空调吗？需要注意什么？ | | 坐月子期间可以吃水果和蔬菜吗？ |
| **English translation of patient question / 患者问题英文翻译** | | Can I wash my hair and take a shower during the postpartum confinement period? Will it cause lingering postpartum illness? | | Can I use air conditioning after birth? What should I pay attention to? | | Can I eat fruit and vegetables during the postpartum confinement period? |
| **Model response (Chinese) / 模型回答（中文）** | | 坐月子期间可以洗头、淋浴和保持个人清洁，所谓洗澡一定会落下“月子病”没有医学依据。关键是注意保暖、防滑、避免过度疲劳，洗后及时擦干头发和身体，不建议长时间泡澡或盆浴，尤其伤口未完全恢复时。 | | 产后可以使用空调，但温度不要过低，避免冷风直吹，房间要适当通风。饮食方面不应只吃油腻汤水，水果和蔬菜可以吃，有助于补充维生素、膳食纤维和预防便秘。 | | 若有腹泻、过敏或血糖问题，可根据个人情况调整种类和量。出现发热、伤口疼痛或异常出血时应就医。 |
| **English translation of model response / 模型回答英文翻译** | | During the postpartum confinement period, washing your hair, showering, and maintaining personal hygiene are allowed. The belief that bathing will inevitably cause a lasting postpartum illness has no medical basis. The key is to keep warm, avoid slipping, and avoid overexertion. | | Dry your hair and body promptly after washing. Prolonged soaking or tub baths are not recommended, especially before wounds have fully healed. Air conditioning can be used after birth, but the temperature should not be too low, direct cold air should be avoided, and the room should be ventilated appropriately. Diet should not rely only on oily soups. | | Fruit and vegetables are suitable and help provide vitamins, fiber, and constipation prevention. If diarrhea, allergy, or blood glucose problems occur, adjust choices and amounts individually. Seek care for fever, wound pain, or abnormal bleeding. |
| **Overall evaluation item / 整体评价项目** | | | **Score / 分数** | | **Comments / 备注** | |
| **Patient Satisfaction / 患者满意度** | | | 5 | |  | |
| **Patient Understandability / 患者可理解性** | | | 4 | |  | |
| **Patient Usefulness / 患者实用性** | | | 4 | |  | |
| **Clinician Correctness / 医生评分：正确性** | | | 4 | |  | |
| **Clinician Completeness / 医生评分：完整性** | | | 4 | |  | |
| **Clinician Readability / 医生评分：可读性** | | | 4 | |  | |
| **Clinician Helpfulness / 医生评分：帮助性** | | | 4 | |  | |
| **Clinician Safety / 医生评分：安全性** | | | 5 | |  | |

**Patient 17**

| **Patient ID / 患者编号** | **Group / 模型组别** | | | | | |
| --- | --- | --- | --- | --- | --- | --- |
| P17 | ChatGPT 5.5 | | | | | |
| **General model response or patient education material (Chinese) / 模型总体回答或患者教育材料（中文）** | | | | | | |
| 下奶并不依赖大量喝汤，真正重要的是尽早开奶、频繁有效吸吮、正确含接和母亲充分休息。汤水可以作为补液的一部分，但过多油腻浓汤可能增加胃肠负担和体重增长，并不一定增加奶量。哺乳期通常不需要严格忌口，应保证主食、优质蛋白、蔬菜水果和足量饮水。酒精、未煮熟食物、过量咖啡因以及产妇明确过敏或宝宝明显不适相关食物应避免或减少。产后贫血者应多吃瘦肉、动物肝脏、血制品、蛋类、豆类和绿叶菜，搭配维生素C丰富食物促进铁吸收，必要时按医嘱补铁并复查血常规。 | | | | | | |
| **English translation of the Chinese general response / 中文总体回答英文翻译** | | | | | | |
| Milk production does not depend on drinking large amounts of soup. What matters more is early breastfeeding, frequent and effective suckling, correct latch, and adequate maternal rest. Soup can be part of fluid intake, but too much oily concentrated soup may increase digestive burden and weight gain and does not necessarily increase milk supply. A breastfeeding diet usually does not require strict avoidance. Include staple foods, high-quality protein, vegetables, fruit, and enough fluids. Alcohol, undercooked foods, excessive caffeine, and foods that clearly trigger maternal allergy or obvious infant discomfort should be avoided or reduced. For postpartum anemia, eat lean meat, liver, blood products where culturally acceptable, eggs, beans, and leafy greens, and combine them with vitamin C-rich foods to improve iron absorption. Iron supplements and repeat blood tests should follow medical advice. | | | | | | |
| **Item / 项目** | | **Q1 / 问题1** | | **Q2 / 问题2** | | **Q3 / 问题3** |
| **Patient question (Chinese original) / 患者原始问题（中文）** | | 产后是不是必须大量喝汤才能下奶？ | | 哺乳期哪些食物需要避免？ | | 我产后贫血，饮食上应该注意什么？ |
| **English translation of patient question / 患者问题英文翻译** | | Do I have to drink a lot of soup after birth to produce breast milk? | | What foods should be avoided during breastfeeding? | | I have anemia after birth. What should I pay attention to in my diet? |
| **Model response (Chinese) / 模型回答（中文）** | | 下奶并不依赖大量喝汤，真正重要的是尽早开奶、频繁有效吸吮、正确含接和母亲充分休息。汤水可以作为补液的一部分，但过多油腻浓汤可能增加胃肠负担和体重增长，并不一定增加奶量。 | | 哺乳期通常不需要严格忌口，应保证主食、优质蛋白、蔬菜水果和足量饮水。 | | 酒精、未煮熟食物、过量咖啡因以及产妇明确过敏或宝宝明显不适相关食物应避免或减少。产后贫血者应多吃瘦肉、动物肝脏、血制品、蛋类、豆类和绿叶菜，搭配维生素C丰富食物促进铁吸收，必要时按医嘱补铁并复查血常规。 |
| **English translation of model response / 模型回答英文翻译** | | Milk production does not depend on drinking large amounts of soup. What matters more is early breastfeeding, frequent and effective suckling, correct latch, and adequate maternal rest. Soup can be part of fluid intake, but too much oily concentrated soup may increase digestive burden and weight gain and does not necessarily increase milk supply. | | A breastfeeding diet usually does not require strict avoidance. Include staple foods, high-quality protein, vegetables, fruit, and enough fluids. | | Alcohol, undercooked foods, excessive caffeine, and foods that clearly trigger maternal allergy or obvious infant discomfort should be avoided or reduced. For postpartum anemia, eat lean meat, liver, blood products where culturally acceptable, eggs, beans, and leafy greens, and combine them with vitamin C-rich foods to improve iron absorption. Iron supplements and repeat blood tests should follow medical advice. |
| **Overall evaluation item / 整体评价项目** | | | **Score / 分数** | | **Comments / 备注** | |
| **Patient Satisfaction / 患者满意度** | | | 4 | |  | |
| **Patient Understandability / 患者可理解性** | | | 4 | |  | |
| **Patient Usefulness / 患者实用性** | | | 5 | |  | |
| **Clinician Correctness / 医生评分：正确性** | | | 5 | |  | |
| **Clinician Completeness / 医生评分：完整性** | | | 5 | |  | |
| **Clinician Readability / 医生评分：可读性** | | | 4 | |  | |
| **Clinician Helpfulness / 医生评分：帮助性** | | | 5 | |  | |
| **Clinician Safety / 医生评分：安全性** | | | 5 | |  | |

**Patient 18**

| **Patient ID / 患者编号** | **Group / 模型组别** | | | | | |
| --- | --- | --- | --- | --- | --- | --- |
| P18 | ChatGPT 5.5 | | | | | |
| **General model response or patient education material (Chinese) / 模型总体回答或患者教育材料（中文）** | | | | | | |
| 哺乳期感冒、产后疼痛或感染用药时，不建议自行随意吃药，也不必一听到用药就停止哺乳。很多退热止痛药和部分抗生素在哺乳期可以使用，但需要根据药物种类、剂量、宝宝月龄、是否早产以及母亲病情判断。感冒时先保证休息、饮水和对症处理，避免含多种成分的复方感冒药自行混用。产后疼痛明显时可咨询医生选择相对安全的止痛药。若医生开抗生素，应主动说明正在哺乳，多数情况下可继续哺乳或排奶。用药期间注意观察宝宝嗜睡、腹泻、皮疹等异常，出现问题及时就医。 | | | | | | |
| **English translation of the Chinese general response / 中文总体回答英文翻译** | | | | | | |
| When treating a cold, postpartum pain, or infection while breastfeeding, you should not take medicines casually by yourself, but you also do not need to stop breastfeeding simply because medicine is needed. Many fever-reducing pain medicines and some antibiotics can be used during breastfeeding, but safety depends on the drug, dose, infant age, whether the baby was premature, and the mother's condition. For a cold, start with rest, fluids, and symptom relief, and avoid mixing multi-ingredient cold medicines on your own. For significant postpartum pain, ask a clinician to choose a relatively safe pain reliever. If antibiotics are prescribed, tell the doctor that you are breastfeeding; in many cases, breastfeeding or milk expression can continue. Watch the baby for unusual sleepiness, diarrhea, rash, or other symptoms and seek care if they occur. | | | | | | |
| **Item / 项目** | | **Q1 / 问题1** | | **Q2 / 问题2** | | **Q3 / 问题3** |
| **Patient question (Chinese original) / 患者原始问题（中文）** | | 哺乳期感冒了，可以自己吃感冒药吗？ | | 产后疼痛能不能吃止痛药？会不会影响母乳？ | | 如果需要吃抗生素，是不是必须停止哺乳？ |
| **English translation of patient question / 患者问题英文翻译** | | If I catch a cold while breastfeeding, can I take cold medicine by myself? | | Can I take pain medicine after birth, and will it affect breast milk? | | If I need antibiotics, must I stop breastfeeding? |
| **Model response (Chinese) / 模型回答（中文）** | | 哺乳期感冒、产后疼痛或感染用药时，不建议自行随意吃药，也不必一听到用药就停止哺乳。很多退热止痛药和部分抗生素在哺乳期可以使用，但需要根据药物种类、剂量、宝宝月龄、是否早产以及母亲病情判断。 | | 感冒时先保证休息、饮水和对症处理，避免含多种成分的复方感冒药自行混用。产后疼痛明显时可咨询医生选择相对安全的止痛药。 | | 若医生开抗生素，应主动说明正在哺乳，多数情况下可继续哺乳或排奶。用药期间注意观察宝宝嗜睡、腹泻、皮疹等异常，出现问题及时就医。 |
| **English translation of model response / 模型回答英文翻译** | | When treating a cold, postpartum pain, or infection while breastfeeding, you should not take medicines casually by yourself, but you also do not need to stop breastfeeding simply because medicine is needed. Many fever-reducing pain medicines and some antibiotics can be used during breastfeeding, but safety depends on the drug, dose, infant age, whether the baby was premature, and the mother's condition. | | For a cold, start with rest, fluids, and symptom relief, and avoid mixing multi-ingredient cold medicines on your own. For significant postpartum pain, ask a clinician to choose a relatively safe pain reliever. | | If antibiotics are prescribed, tell the doctor that you are breastfeeding; in many cases, breastfeeding or milk expression can continue. Watch the baby for unusual sleepiness, diarrhea, rash, or other symptoms and seek care if they occur. |
| **Overall evaluation item / 整体评价项目** | | | **Score / 分数** | | **Comments / 备注** | |
| **Patient Satisfaction / 患者满意度** | | | 4 | |  | |
| **Patient Understandability / 患者可理解性** | | | 4 | |  | |
| **Patient Usefulness / 患者实用性** | | | 4 | |  | |
| **Clinician Correctness / 医生评分：正确性** | | | 5 | |  | |
| **Clinician Completeness / 医生评分：完整性** | | | 5 | |  | |
| **Clinician Readability / 医生评分：可读性** | | | 4 | |  | |
| **Clinician Helpfulness / 医生评分：帮助性** | | | 5 | |  | |
| **Clinician Safety / 医生评分：安全性** | | | 5 | |  | |

**Patient 19**

| **Patient ID / 患者编号** | **Group / 模型组别** | | | | | |
| --- | --- | --- | --- | --- | --- | --- |
| P19 | ChatGPT 5.5 | | | | | |
| **General model response or patient education material (Chinese) / 模型总体回答或患者教育材料（中文）** | | | | | | |
| 孕期有高血压或子痫前期病史的产妇，即使产后血压暂时正常，也应继续监测，因为产后数天至数周仍可能出现血压升高。建议按医嘱在家记录血压，保持复诊，并注意头痛、眼花、胸闷、上腹痛、明显水肿、气短等症状。产后头痛和眼花可能与睡眠不足有关，也可能是高血压或子痫前期相关危险信号，不能只靠休息判断。若血压达到医生告知的警戒值，或出现持续头痛、视物模糊、胸痛、呼吸困难、抽搐、意识异常，应立即就医。降压药是否继续或调整，应由医生根据血压和哺乳情况决定。 | | | | | | |
| **English translation of the Chinese general response / 中文总体回答英文翻译** | | | | | | |
| Women who had high blood pressure or preeclampsia during pregnancy should continue monitoring after birth even if blood pressure is currently normal, because blood pressure can rise during the days or weeks after delivery. Record home blood pressure as advised, attend follow-up visits, and watch for headache, blurred vision, chest tightness, upper abdominal pain, marked swelling, or shortness of breath. Postpartum headache and visual symptoms may be related to sleep deprivation, but they can also be warning signs of hypertension or preeclampsia and should not be judged by rest alone. Seek urgent care if blood pressure reaches the warning level given by your clinician, or if persistent headache, blurred vision, chest pain, breathing difficulty, seizure, or confusion occurs. Continuing or adjusting blood pressure medicine should be decided by your doctor with breastfeeding considered. | | | | | | |
| **Item / 项目** | | **Q1 / 问题1** | | **Q2 / 问题2** | | **Q3 / 问题3** |
| **Patient question (Chinese original) / 患者原始问题（中文）** | | 我孕期有高血压，产后血压正常了还需要继续监测吗？ | | 产后头痛、眼花是不是和血压有关？ | | 如果产后血压又升高，需要马上去医院吗？ |
| **English translation of patient question / 患者问题英文翻译** | | I had high blood pressure during pregnancy. If my blood pressure is normal after birth, do I still need monitoring? | | Are postpartum headache and blurred vision related to blood pressure? | | If my blood pressure rises again after birth, do I need to go to the hospital immediately? |
| **Model response (Chinese) / 模型回答（中文）** | | 孕期有高血压或子痫前期病史的产妇，即使产后血压暂时正常，也应继续监测，因为产后数天至数周仍可能出现血压升高。建议按医嘱在家记录血压，保持复诊，并注意头痛、眼花、胸闷、上腹痛、明显水肿、气短等症状。 | | 产后头痛和眼花可能与睡眠不足有关，也可能是高血压或子痫前期相关危险信号，不能只靠休息判断。 | | 若血压达到医生告知的警戒值，或出现持续头痛、视物模糊、胸痛、呼吸困难、抽搐、意识异常，应立即就医。降压药是否继续或调整，应由医生根据血压和哺乳情况决定。 |
| **English translation of model response / 模型回答英文翻译** | | Women who had high blood pressure or preeclampsia during pregnancy should continue monitoring after birth even if blood pressure is currently normal, because blood pressure can rise during the days or weeks after delivery. Record home blood pressure as advised, attend follow-up visits, and watch for headache, blurred vision, chest tightness, upper abdominal pain, marked swelling, or shortness of breath. | | Postpartum headache and visual symptoms may be related to sleep deprivation, but they can also be warning signs of hypertension or preeclampsia and should not be judged by rest alone. | | Seek urgent care if blood pressure reaches the warning level given by your clinician, or if persistent headache, blurred vision, chest pain, breathing difficulty, seizure, or confusion occurs. Continuing or adjusting blood pressure medicine should be decided by your doctor with breastfeeding considered. |
| **Overall evaluation item / 整体评价项目** | | | **Score / 分数** | | **Comments / 备注** | |
| **Patient Satisfaction / 患者满意度** | | | 4 | |  | |
| **Patient Understandability / 患者可理解性** | | | 4 | |  | |
| **Patient Usefulness / 患者实用性** | | | 4 | |  | |
| **Clinician Correctness / 医生评分：正确性** | | | 5 | |  | |
| **Clinician Completeness / 医生评分：完整性** | | | 5 | |  | |
| **Clinician Readability / 医生评分：可读性** | | | 4 | |  | |
| **Clinician Helpfulness / 医生评分：帮助性** | | | 5 | |  | |
| **Clinician Safety / 医生评分：安全性** | | | 5 | |  | |

**Patient 20**

| **Patient ID / 患者编号** | **Group / 模型组别** | | | | | |
| --- | --- | --- | --- | --- | --- | --- |
| P20 | ChatGPT 5.5 | | | | | |
| **General model response or patient education material (Chinese) / 模型总体回答或患者教育材料（中文）** | | | | | | |
| 妊娠糖尿病产后仍需要随访血糖。即使产后血糖恢复正常，也不代表以后没有风险，因为妊娠糖尿病会增加今后发生2型糖尿病和再次妊娠糖尿病的可能。通常建议在产后6至12周进行糖代谢复查，之后定期筛查，具体时间按医生安排。日常应注意均衡饮食、控制精制糖和高油食物、逐步恢复运动、保持合适体重，并继续母乳喂养。若出现明显口渴、多尿、体重异常下降、伤口不易愈合或再次备孕，应提前检查血糖。家族史、肥胖和血糖异常者更要长期管理，而不是产后正常就完全不管。 | | | | | | |
| **English translation of the Chinese general response / 中文总体回答英文翻译** | | | | | | |
| Gestational diabetes requires postpartum glucose follow-up. Even if blood glucose becomes normal after birth, future risk is not gone, because gestational diabetes increases the chance of type 2 diabetes and diabetes in a future pregnancy. A glucose metabolism test is commonly recommended 6 to 12 weeks after birth, followed by regular screening according to your doctor's plan. Daily care should include a balanced diet, limiting refined sugar and high-fat foods, gradually returning to physical activity, maintaining a healthy weight, and continuing breastfeeding if possible. Check blood glucose earlier if marked thirst, frequent urination, unexplained weight loss, poor wound healing, or a plan for another pregnancy occurs. Women with family history, obesity, or abnormal glucose results need long-term management rather than ignoring the issue after one normal test. | | | | | | |
| **Item / 项目** | | **Q1 / 问题1** | | **Q2 / 问题2** | | **Q3 / 问题3** |
| **Patient question (Chinese original) / 患者原始问题（中文）** | | 我孕期有妊娠糖尿病，产后还需要测血糖吗？ | | 产后血糖正常了，是不是就不用管了？ | | 妊娠糖尿病会不会增加以后得糖尿病的风险？ |
| **English translation of patient question / 患者问题英文翻译** | | I had gestational diabetes during pregnancy. Do I still need to check my blood glucose after birth? | | If my postpartum blood glucose is normal, does that mean I no longer need to worry about it? | | Does gestational diabetes increase my risk of developing diabetes in the future? |
| **Model response (Chinese) / 模型回答（中文）** | | 妊娠糖尿病产后仍需要随访血糖。即使产后血糖恢复正常，也不代表以后没有风险，因为妊娠糖尿病会增加今后发生2型糖尿病和再次妊娠糖尿病的可能。 | | 通常建议在产后6至12周进行糖代谢复查，之后定期筛查，具体时间按医生安排。日常应注意均衡饮食、控制精制糖和高油食物、逐步恢复运动、保持合适体重，并继续母乳喂养。 | | 若出现明显口渴、多尿、体重异常下降、伤口不易愈合或再次备孕，应提前检查血糖。家族史、肥胖和血糖异常者更要长期管理，而不是产后正常就完全不管。 |
| **English translation of model response / 模型回答英文翻译** | | Gestational diabetes requires postpartum glucose follow-up. Even if blood glucose becomes normal after birth, future risk is not gone, because gestational diabetes increases the chance of type 2 diabetes and diabetes in a future pregnancy. | | A glucose metabolism test is commonly recommended 6 to 12 weeks after birth, followed by regular screening according to your doctor's plan. Daily care should include a balanced diet, limiting refined sugar and high-fat foods, gradually returning to physical activity, maintaining a healthy weight, and continuing breastfeeding if possible. | | Check blood glucose earlier if marked thirst, frequent urination, unexplained weight loss, poor wound healing, or a plan for another pregnancy occurs. Women with family history, obesity, or abnormal glucose results need long-term management rather than ignoring the issue after one normal test. |
| **Overall evaluation item / 整体评价项目** | | | **Score / 分数** | | **Comments / 备注** | |
| **Patient Satisfaction / 患者满意度** | | | 4 | |  | |
| **Patient Understandability / 患者可理解性** | | | 4 | |  | |
| **Patient Usefulness / 患者实用性** | | | 4 | |  | |
| **Clinician Correctness / 医生评分：正确性** | | | 4 | |  | |
| **Clinician Completeness / 医生评分：完整性** | | | 5 | |  | |
| **Clinician Readability / 医生评分：可读性** | | | 4 | |  | |
| **Clinician Helpfulness / 医生评分：帮助性** | | | 5 | |  | |
| **Clinician Safety / 医生评分：安全性** | | | 4 | |  | |

**Patient 21**

| **Patient ID / 患者编号** | **Group / 模型组别** | | | | | |
| --- | --- | --- | --- | --- | --- | --- |
| P21 | ChatGPT 5.5 | | | | | |
| **General model response or patient education material (Chinese) / 模型总体回答或患者教育材料（中文）** | | | | | | |
| 产后轻度双下肢水肿较常见，多与孕期体液潴留和活动减少有关，通常会逐渐减轻。但血栓风险在产褥期升高，不能忽视单侧腿部症状。若一侧小腿明显肿胀、疼痛、发热、发红，或两腿粗细差异明显，应尽快就医，不要自行按摩，以免延误或增加风险。产后胸闷、气短、胸痛、咳血、心跳很快或突然晕厥，可能与肺栓塞等严重问题有关，需要立即急诊处理。平时应尽早适量活动、补充水分，避免长期卧床。剖宫产、肥胖、高龄、血栓史等人群更应按医嘱预防。 | | | | | | |
| **English translation of the Chinese general response / 中文总体回答英文翻译** | | | | | | |
| Mild swelling in both legs after birth is common and is often related to fluid retention from pregnancy and reduced activity; it usually improves gradually. However, the risk of blood clots is higher during the postpartum period, so one-sided leg symptoms should not be ignored. If one calf becomes obviously swollen, painful, warm, red, or much larger than the other, seek medical care promptly and do not massage it yourself. Postpartum chest tightness, shortness of breath, chest pain, coughing blood, a very fast heartbeat, or sudden fainting may be related to pulmonary embolism or another serious condition and requires emergency care. In daily recovery, begin gentle activity early as tolerated, drink enough fluids, and avoid prolonged bed rest. Women with cesarean birth, obesity, older age, or a clot history should follow medical advice for prevention. | | | | | | |
| **Item / 项目** | | **Q1 / 问题1** | | **Q2 / 问题2** | | **Q3 / 问题3** |
| **Patient question (Chinese original) / 患者原始问题（中文）** | | 产后腿有点肿，是正常水肿还是血栓？ | | 如果一侧小腿疼痛、发热、肿胀，需要去医院吗？ | | 产后胸闷、气短会不会和肺栓塞有关？ |
| **English translation of patient question / 患者问题英文翻译** | | My legs are a little swollen after birth. Is it normal edema or a blood clot? | | If one calf is painful, warm, and swollen, do I need to go to the hospital? | | Could postpartum chest tightness and shortness of breath be related to pulmonary embolism? |
| **Model response (Chinese) / 模型回答（中文）** | | 产后轻度双下肢水肿较常见，多与孕期体液潴留和活动减少有关，通常会逐渐减轻。但血栓风险在产褥期升高，不能忽视单侧腿部症状。 | | 若一侧小腿明显肿胀、疼痛、发热、发红，或两腿粗细差异明显，应尽快就医，不要自行按摩，以免延误或增加风险。产后胸闷、气短、胸痛、咳血、心跳很快或突然晕厥，可能与肺栓塞等严重问题有关，需要立即急诊处理。 | | 平时应尽早适量活动、补充水分，避免长期卧床。剖宫产、肥胖、高龄、血栓史等人群更应按医嘱预防。 |
| **English translation of model response / 模型回答英文翻译** | | Mild swelling in both legs after birth is common and is often related to fluid retention from pregnancy and reduced activity; it usually improves gradually. However, the risk of blood clots is higher during the postpartum period, so one-sided leg symptoms should not be ignored. | | If one calf becomes obviously swollen, painful, warm, red, or much larger than the other, seek medical care promptly and do not massage it yourself. Postpartum chest tightness, shortness of breath, chest pain, coughing blood, a very fast heartbeat, or sudden fainting may be related to pulmonary embolism or another serious condition and requires emergency care. | | In daily recovery, begin gentle activity early as tolerated, drink enough fluids, and avoid prolonged bed rest. Women with cesarean birth, obesity, older age, or a clot history should follow medical advice for prevention. |
| **Overall evaluation item / 整体评价项目** | | | **Score / 分数** | | **Comments / 备注** | |
| **Patient Satisfaction / 患者满意度** | | | 4 | |  | |
| **Patient Understandability / 患者可理解性** | | | 4 | |  | |
| **Patient Usefulness / 患者实用性** | | | 4 | |  | |
| **Clinician Correctness / 医生评分：正确性** | | | 5 | |  | |
| **Clinician Completeness / 医生评分：完整性** | | | 4 | |  | |
| **Clinician Readability / 医生评分：可读性** | | | 4 | |  | |
| **Clinician Helpfulness / 医生评分：帮助性** | | | 4 | |  | |
| **Clinician Safety / 医生评分：安全性** | | | 5 | |  | |

**Patient 22**

| **Patient ID / 患者编号** | **Group / 模型组别** | | | | | |
| --- | --- | --- | --- | --- | --- | --- |
| P22 | ChatGPT 5.5 | | | | | |
| **General model response or patient education material (Chinese) / 模型总体回答或患者教育材料（中文）** | | | | | | |
| 剖宫产后并不是越卧床越好。若生命体征稳定、麻醉反应消退且医生允许，通常应尽早在帮助下翻身、坐起和下床短距离走动，这有助于肠道恢复、减少血栓风险和促进整体康复。刚开始活动要慢，先坐床边，再站立，避免头晕摔倒。抱孩子时可用枕头或腹带适当支撑腹部，避免突然用力、提重物和长时间弯腰。正常轻柔下床一般不会导致切口裂开，真正需要警惕的是切口红肿热痛、渗液、裂开、发热或腹痛加重。若活动后疼痛明显或出血增加，应暂停并请医护人员评估。 | | | | | | |
| **English translation of the Chinese general response / 中文总体回答英文翻译** | | | | | | |
| After a cesarean birth, staying in bed as much as possible is not the best approach. If vital signs are stable, anesthesia effects have worn off, and your clinician allows it, early turning, sitting, and short assisted walks are usually encouraged. This helps bowel recovery, lowers clot risk, and supports overall healing. Start slowly by sitting at the bedside, then standing, to avoid dizziness and falls. When holding the baby, use a pillow or abdominal support if helpful, and avoid sudden force, heavy lifting, and prolonged bending. Gentle normal movement usually does not open the incision. Warning signs include increasing redness, swelling, warmth, pain, drainage, wound separation, fever, or worsening abdominal pain. If activity causes obvious pain or heavier bleeding, stop and ask for medical assessment. | | | | | | |
| **Item / 项目** | | **Q1 / 问题1** | | **Q2 / 问题2** | | **Q3 / 问题3** |
| **Patient question (Chinese original) / 患者原始问题（中文）** | | 剖宫产后是不是要尽量卧床休息？ | | 剖宫产后多久可以下床走动和抱孩子？ | | 下床活动会不会导致切口裂开？ |
| **English translation of patient question / 患者问题英文翻译** | | After a cesarean birth, should I stay in bed as much as possible? | | How soon after a cesarean birth can I get out of bed and hold my baby? | | Will getting out of bed cause the incision to open? |
| **Model response (Chinese) / 模型回答（中文）** | | 剖宫产后并不是越卧床越好。若生命体征稳定、麻醉反应消退且医生允许，通常应尽早在帮助下翻身、坐起和下床短距离走动，这有助于肠道恢复、减少血栓风险和促进整体康复。 | | 刚开始活动要慢，先坐床边，再站立，避免头晕摔倒。抱孩子时可用枕头或腹带适当支撑腹部，避免突然用力、提重物和长时间弯腰。 | | 正常轻柔下床一般不会导致切口裂开，真正需要警惕的是切口红肿热痛、渗液、裂开、发热或腹痛加重。若活动后疼痛明显或出血增加，应暂停并请医护人员评估。 |
| **English translation of model response / 模型回答英文翻译** | | After a cesarean birth, staying in bed as much as possible is not the best approach. If vital signs are stable, anesthesia effects have worn off, and your clinician allows it, early turning, sitting, and short assisted walks are usually encouraged. This helps bowel recovery, lowers clot risk, and supports overall healing. | | Start slowly by sitting at the bedside, then standing, to avoid dizziness and falls. When holding the baby, use a pillow or abdominal support if helpful, and avoid sudden force, heavy lifting, and prolonged bending. | | Gentle normal movement usually does not open the incision. Warning signs include increasing redness, swelling, warmth, pain, drainage, wound separation, fever, or worsening abdominal pain. If activity causes obvious pain or heavier bleeding, stop and ask for medical assessment. |
| **Overall evaluation item / 整体评价项目** | | | **Score / 分数** | | **Comments / 备注** | |
| **Patient Satisfaction / 患者满意度** | | | 4 | |  | |
| **Patient Understandability / 患者可理解性** | | | 4 | |  | |
| **Patient Usefulness / 患者实用性** | | | 4 | |  | |
| **Clinician Correctness / 医生评分：正确性** | | | 5 | |  | |
| **Clinician Completeness / 医生评分：完整性** | | | 4 | |  | |
| **Clinician Readability / 医生评分：可读性** | | | 4 | |  | |
| **Clinician Helpfulness / 医生评分：帮助性** | | | 4 | |  | |
| **Clinician Safety / 医生评分：安全性** | | | 5 | |  | |

**Patient 23**

| **Patient ID / 患者编号** | **Group / 模型组别** | | | | | |
| --- | --- | --- | --- | --- | --- | --- |
| P23 | ChatGPT 5.5 | | | | | |
| **General model response or patient education material (Chinese) / 模型总体回答或患者教育材料（中文）** | | | | | | |
| 顺产撕裂伤口若出现异味、分泌物增多或疼痛加重，需要警惕感染，但也要结合恶露气味、局部红肿和体温判断。日常护理以清水温和冲洗、由前向后清洁、勤换卫生巾、保持干燥为主，不建议自行反复使用高浓度消毒液或阴道冲洗，以免刺激伤口和影响正常菌群。若分泌物少、无明显红肿和发热，可先加强清洁并尽快咨询产后门诊。若会阴伤口疼痛越来越明显，局部红肿热痛、流脓、裂开、恶露臭味或发热，应及时复查。疼痛突然加重或坐立困难，也应就医排除血肿。 | | | | | | |
| **English translation of the Chinese general response / 中文总体回答英文翻译** | | | | | | |
| If a vaginal tear wound has an unpleasant odor, increased discharge, or worsening pain, infection should be considered, but lochia odor, local redness and swelling, and body temperature should also be assessed. Daily care should focus on gentle rinsing with clean warm water, cleaning from front to back, changing pads often, and keeping the area dry. Repeated use of strong disinfectants or vaginal douching by yourself is not recommended because it can irritate the wound and disturb normal bacteria. If discharge is small and there is no obvious redness, swelling, or fever, improve hygiene and contact the postpartum clinic. Seek prompt review if pain becomes stronger, the area is red, swollen, warm, or painful, pus appears, the wound opens, lochia smells foul, or fever develops. Sudden severe pain or difficulty sitting should also be evaluated to rule out hematoma. | | | | | | |
| **Item / 项目** | | **Q1 / 问题1** | | **Q2 / 问题2** | | **Q3 / 问题3** |
| **Patient question (Chinese original) / 患者原始问题（中文）** | | 我顺产撕裂伤口有点异味，是不是感染了？ | | 会阴伤口如果有分泌物，需要用消毒液清洗吗？ | | 会阴伤口疼痛越来越明显，需要复查吗？ |
| **English translation of patient question / 患者问题英文翻译** | | My tear wound after vaginal birth has a slight odor. Is it infected? | | If there is discharge from the perineal wound, should I clean it with disinfectant? | | If the perineal wound pain is getting worse, do I need a follow-up check? |
| **Model response (Chinese) / 模型回答（中文）** | | 顺产撕裂伤口若出现异味、分泌物增多或疼痛加重，需要警惕感染，但也要结合恶露气味、局部红肿和体温判断。日常护理以清水温和冲洗、由前向后清洁、勤换卫生巾、保持干燥为主，不建议自行反复使用高浓度消毒液或阴道冲洗，以免刺激伤口和影响正常菌群。 | | 若分泌物少、无明显红肿和发热，可先加强清洁并尽快咨询产后门诊。 | | 若会阴伤口疼痛越来越明显，局部红肿热痛、流脓、裂开、恶露臭味或发热，应及时复查。疼痛突然加重或坐立困难，也应就医排除血肿。 |
| **English translation of model response / 模型回答英文翻译** | | If a vaginal tear wound has an unpleasant odor, increased discharge, or worsening pain, infection should be considered, but lochia odor, local redness and swelling, and body temperature should also be assessed. Daily care should focus on gentle rinsing with clean warm water, cleaning from front to back, changing pads often, and keeping the area dry. | | Repeated use of strong disinfectants or vaginal douching by yourself is not recommended because it can irritate the wound and disturb normal bacteria. If discharge is small and there is no obvious redness, swelling, or fever, improve hygiene and contact the postpartum clinic. | | Seek prompt review if pain becomes stronger, the area is red, swollen, warm, or painful, pus appears, the wound opens, lochia smells foul, or fever develops. Sudden severe pain or difficulty sitting should also be evaluated to rule out hematoma. |
| **Overall evaluation item / 整体评价项目** | | | **Score / 分数** | | **Comments / 备注** | |
| **Patient Satisfaction / 患者满意度** | | | 4 | |  | |
| **Patient Understandability / 患者可理解性** | | | 4 | |  | |
| **Patient Usefulness / 患者实用性** | | | 4 | |  | |
| **Clinician Correctness / 医生评分：正确性** | | | 5 | |  | |
| **Clinician Completeness / 医生评分：完整性** | | | 4 | |  | |
| **Clinician Readability / 医生评分：可读性** | | | 4 | |  | |
| **Clinician Helpfulness / 医生评分：帮助性** | | | 4 | |  | |
| **Clinician Safety / 医生评分：安全性** | | | 5 | |  | |

**Patient 24**

| **Patient ID / 患者编号** | **Group / 模型组别** | | | | | |
| --- | --- | --- | --- | --- | --- | --- |
| P24 | ChatGPT 5.5 | | | | | |
| **General model response or patient education material (Chinese) / 模型总体回答或患者教育材料（中文）** | | | | | | |
| 乳房有硬块但不发烧，多数是乳汁淤积或局部乳腺管不通。不要用力揉开，更不要让他人暴力按摩，以免造成乳腺组织损伤、皮下出血和炎症加重。可以继续哺乳，通常不需要暂停，关键是让宝宝有效含接和规律排奶。喂奶前短时间热敷，喂奶时变换姿势，让宝宝下巴朝向硬块方向，喂后仍胀可少量手挤或吸奶，避免过度排空刺激产奶过多。若出现发热、寒战、局部红肿热痛或硬块24至48小时不缓解，应就医判断是否乳腺炎。需要抗生素时，多数可选择哺乳期可用药物，不一定停止喂奶。 | | | | | | |
| **English translation of the Chinese general response / 中文总体回答英文翻译** | | | | | | |
| A hard breast lump without fever is often due to milk stasis or a blocked milk duct. Do not try to knead it forcefully, and avoid aggressive massage by others, as this can injure breast tissue, cause bruising, and worsen inflammation. Breastfeeding can usually continue and usually should not be stopped. The key is effective latch and regular milk removal. A brief warm compress before feeding may help. Changing feeding positions and placing the baby's chin toward the lump can improve drainage. If the breast remains uncomfortably full after feeding, express a small amount by hand or pump, but avoid excessive emptying that stimulates oversupply. If fever, chills, redness, warmth, pain, or no improvement within 24 to 48 hours occurs, seek medical care to assess for mastitis. If antibiotics are needed, many breastfeeding-compatible options are available, and stopping breastfeeding is not always necessary. | | | | | | |
| **Item / 项目** | | **Q1 / 问题1** | | **Q2 / 问题2** | | **Q3 / 问题3** |
| **Patient question (Chinese original) / 患者原始问题（中文）** | | 我乳房有硬块但不发烧，可以自己按摩揉开吗？ | | 堵奶时继续喂奶好，还是应该先暂停哺乳？ | | 乳腺炎需要吃抗生素吗？会不会影响宝宝吃奶？ |
| **English translation of patient question / 患者问题英文翻译** | | I have a breast lump but no fever. Can I massage it open by myself? | | When there is a blocked duct, is it better to continue breastfeeding or stop temporarily? | | Does mastitis require antibiotics, and will that affect the baby during feeding? |
| **Model response (Chinese) / 模型回答（中文）** | | 乳房有硬块但不发烧，多数是乳汁淤积或局部乳腺管不通。不要用力揉开，更不要让他人暴力按摩，以免造成乳腺组织损伤、皮下出血和炎症加重。 | | 可以继续哺乳，通常不需要暂停，关键是让宝宝有效含接和规律排奶。喂奶前短时间热敷，喂奶时变换姿势，让宝宝下巴朝向硬块方向，喂后仍胀可少量手挤或吸奶，避免过度排空刺激产奶过多。 | | 若出现发热、寒战、局部红肿热痛或硬块24至48小时不缓解，应就医判断是否乳腺炎。需要抗生素时，多数可选择哺乳期可用药物，不一定停止喂奶。 |
| **English translation of model response / 模型回答英文翻译** | | A hard breast lump without fever is often due to milk stasis or a blocked milk duct. Do not try to knead it forcefully, and avoid aggressive massage by others, as this can injure breast tissue, cause bruising, and worsen inflammation. Breastfeeding can usually continue and usually should not be stopped. | | The key is effective latch and regular milk removal. A brief warm compress before feeding may help. Changing feeding positions and placing the baby's chin toward the lump can improve drainage. | | If the breast remains uncomfortably full after feeding, express a small amount by hand or pump, but avoid excessive emptying that stimulates oversupply. If fever, chills, redness, warmth, pain, or no improvement within 24 to 48 hours occurs, seek medical care to assess for mastitis. If antibiotics are needed, many breastfeeding-compatible options are available, and stopping breastfeeding is not always necessary. |
| **Overall evaluation item / 整体评价项目** | | | **Score / 分数** | | **Comments / 备注** | |
| **Patient Satisfaction / 患者满意度** | | | 4 | |  | |
| **Patient Understandability / 患者可理解性** | | | 4 | |  | |
| **Patient Usefulness / 患者实用性** | | | 4 | |  | |
| **Clinician Correctness / 医生评分：正确性** | | | 4 | |  | |
| **Clinician Completeness / 医生评分：完整性** | | | 5 | |  | |
| **Clinician Readability / 医生评分：可读性** | | | 4 | |  | |
| **Clinician Helpfulness / 医生评分：帮助性** | | | 5 | |  | |
| **Clinician Safety / 医生评分：安全性** | | | 5 | |  | |

**Patient 25**

| **Patient ID / 患者编号** | **Group / 模型组别** | | | | | |
| --- | --- | --- | --- | --- | --- | --- |
| P25 | ChatGPT 5.5 | | | | | |
| **General model response or patient education material (Chinese) / 模型总体回答或患者教育材料（中文）** | | | | | | |
| 产后42天复查建议尽量按时进行，即使自我感觉恢复不错，也不能完全代替专业评估。复查通常包括血压、体重、恶露和月经情况、子宫恢复、会阴或剖宫产切口、盆底功能、乳房和哺乳、情绪状态、避孕和慢病随访等。对于妊娠期高血压、妊娠糖尿病、贫血、剖宫产或分娩并发症者，复查更重要。若出现大量出血、发热、恶露臭味、伤口红肿流脓、严重腹痛、胸闷气短、单侧腿痛肿、剧烈头痛眼花、自伤想法等情况，不能等到42天，应立即就医。产后护理是连续过程，不是一次检查就结束。 | | | | | | |
| **English translation of the Chinese general response / 中文总体回答英文翻译** | | | | | | |
| The 42-day postpartum check-up is strongly recommended even if you feel you are recovering well, because self-assessment cannot replace professional evaluation. The visit usually includes blood pressure, weight, lochia and menstruation, uterine recovery, perineal or cesarean wound healing, pelvic floor function, breast and breastfeeding issues, mood, contraception, and follow-up for chronic or pregnancy-related conditions. It is especially important after hypertensive disorders, gestational diabetes, anemia, cesarean birth, or delivery complications. Do not wait until 42 days if heavy bleeding, fever, foul-smelling lochia, wound redness or pus, severe abdominal pain, chest tightness, shortness of breath, one-sided leg pain and swelling, severe headache with visual symptoms, or thoughts of self-harm occur. Postpartum care is an ongoing process, not a single final visit. | | | | | | |
| **Item / 项目** | | **Q1 / 问题1** | | **Q2 / 问题2** | | **Q3 / 问题3** |
| **Patient question (Chinese original) / 患者原始问题（中文）** | | 产后42天复查一定要去吗？主要检查什么？ | | 如果我感觉恢复得还可以，可以不去产后复查吗？ | | 产后哪些情况不能等到42天复查，应该提前就医？ |
| **English translation of patient question / 患者问题英文翻译** | | Must I attend the 42-day postpartum check-up? What is mainly checked? | | If I feel that I have recovered fairly well, can I skip the postpartum check-up? | | What postpartum situations should not wait until the 42-day check-up and require earlier medical care? |
| **Model response (Chinese) / 模型回答（中文）** | | 产后42天复查建议尽量按时进行，即使自我感觉恢复不错，也不能完全代替专业评估。复查通常包括血压、体重、恶露和月经情况、子宫恢复、会阴或剖宫产切口、盆底功能、乳房和哺乳、情绪状态、避孕和慢病随访等。 | | 对于妊娠期高血压、妊娠糖尿病、贫血、剖宫产或分娩并发症者，复查更重要。 | | 若出现大量出血、发热、恶露臭味、伤口红肿流脓、严重腹痛、胸闷气短、单侧腿痛肿、剧烈头痛眼花、自伤想法等情况，不能等到42天，应立即就医。产后护理是连续过程，不是一次检查就结束。 |
| **English translation of model response / 模型回答英文翻译** | | The 42-day postpartum check-up is strongly recommended even if you feel you are recovering well, because self-assessment cannot replace professional evaluation. The visit usually includes blood pressure, weight, lochia and menstruation, uterine recovery, perineal or cesarean wound healing, pelvic floor function, breast and breastfeeding issues, mood, contraception, and follow-up for chronic or pregnancy-related conditions. | | It is especially important after hypertensive disorders, gestational diabetes, anemia, cesarean birth, or delivery complications. | | Do not wait until 42 days if heavy bleeding, fever, foul-smelling lochia, wound redness or pus, severe abdominal pain, chest tightness, shortness of breath, one-sided leg pain and swelling, severe headache with visual symptoms, or thoughts of self-harm occur. Postpartum care is an ongoing process, not a single final visit. |
| **Overall evaluation item / 整体评价项目** | | | **Score / 分数** | | **Comments / 备注** | |
| **Patient Satisfaction / 患者满意度** | | | 4 | |  | |
| **Patient Understandability / 患者可理解性** | | | 4 | |  | |
| **Patient Usefulness / 患者实用性** | | | 4 | |  | |
| **Clinician Correctness / 医生评分：正确性** | | | 5 | |  | |
| **Clinician Completeness / 医生评分：完整性** | | | 4 | |  | |
| **Clinician Readability / 医生评分：可读性** | | | 4 | |  | |
| **Clinician Helpfulness / 医生评分：帮助性** | | | 4 | |  | |
| **Clinician Safety / 医生评分：安全性** | | | 5 | |  | |

**Patient 26**

| **Patient ID / 患者编号** | **Group / 模型组别** | | | | | |
| --- | --- | --- | --- | --- | --- | --- |
| P26 | ChatGPT 5.5 | | | | | |
| **General model response or patient education material (Chinese) / 模型总体回答或患者教育材料（中文）** | | | | | | |
| 产后腰酸背痛不一定是“月子没坐好”，更常见原因包括孕期韧带松弛、腹肌和盆底力量下降、抱娃姿势不当、喂奶久坐、睡眠不足和剖宫产后活动代偿。喂奶时应让宝宝靠近身体，用枕头支撑手臂和腰背，不要低头弯腰去够宝宝。抱孩子时先靠近再抱起，避免扭腰，家务和重物应请家人分担。可以做温和拉伸和核心恢复训练，但不要急于大强度锻炼。若腰痛越来越重、放射到腿、伴麻木无力、发热、尿痛、外伤后疼痛或夜间痛明显，应及时就医排查骨盆、脊柱或泌尿系统问题。 | | | | | | |
| **English translation of the Chinese general response / 中文总体回答英文翻译** | | | | | | |
| Postpartum low back pain does not necessarily mean you did confinement incorrectly. More common causes include ligament relaxation from pregnancy, weakened abdominal and pelvic floor muscles, poor baby-holding posture, prolonged sitting during feeding, lack of sleep, and compensatory movements after cesarean birth. During feeding, bring the baby close to your body and support your arms and back with pillows instead of bending down toward the baby. When lifting the baby, move close first and avoid twisting the waist. Ask family members to share housework and heavy tasks. Gentle stretching and core recovery exercises may help, but intensive training should not be rushed. Seek medical care if pain worsens, radiates to the leg, is accompanied by numbness or weakness, fever, painful urination, pain after injury, or obvious night pain. Pelvic, spinal, or urinary problems may need evaluation. | | | | | | |
| **Item / 项目** | | **Q1 / 问题1** | | **Q2 / 问题2** | | **Q3 / 问题3** |
| **Patient question (Chinese original) / 患者原始问题（中文）** | | 我产后总觉得腰酸背痛，是不是坐月子没坐好？ | | 抱孩子和喂奶时怎样保护腰背？ | | 如果腰痛越来越重，什么时候需要就医？ |
| **English translation of patient question / 患者问题英文翻译** | | I often feel lower back pain after birth. Is it because I did not follow postpartum confinement properly? | | How can I protect my lower back when holding the baby and breastfeeding? | | If my back pain is getting worse, when should I seek medical care? |
| **Model response (Chinese) / 模型回答（中文）** | | 产后腰酸背痛不一定是“月子没坐好”，更常见原因包括孕期韧带松弛、腹肌和盆底力量下降、抱娃姿势不当、喂奶久坐、睡眠不足和剖宫产后活动代偿。喂奶时应让宝宝靠近身体，用枕头支撑手臂和腰背，不要低头弯腰去够宝宝。 | | 抱孩子时先靠近再抱起，避免扭腰，家务和重物应请家人分担。 | | 可以做温和拉伸和核心恢复训练，但不要急于大强度锻炼。若腰痛越来越重、放射到腿、伴麻木无力、发热、尿痛、外伤后疼痛或夜间痛明显，应及时就医排查骨盆、脊柱或泌尿系统问题。 |
| **English translation of model response / 模型回答英文翻译** | | Postpartum low back pain does not necessarily mean you did confinement incorrectly. More common causes include ligament relaxation from pregnancy, weakened abdominal and pelvic floor muscles, poor baby-holding posture, prolonged sitting during feeding, lack of sleep, and compensatory movements after cesarean birth. During feeding, bring the baby close to your body and support your arms and back with pillows instead of bending down toward the baby. | | When lifting the baby, move close first and avoid twisting the waist. Ask family members to share housework and heavy tasks. | | Gentle stretching and core recovery exercises may help, but intensive training should not be rushed. Seek medical care if pain worsens, radiates to the leg, is accompanied by numbness or weakness, fever, painful urination, pain after injury, or obvious night pain. Pelvic, spinal, or urinary problems may need evaluation. |
| **Overall evaluation item / 整体评价项目** | | | **Score / 分数** | | **Comments / 备注** | |
| **Patient Satisfaction / 患者满意度** | | | 5 | |  | |
| **Patient Understandability / 患者可理解性** | | | 4 | |  | |
| **Patient Usefulness / 患者实用性** | | | 4 | |  | |
| **Clinician Correctness / 医生评分：正确性** | | | 5 | |  | |
| **Clinician Completeness / 医生评分：完整性** | | | 4 | |  | |
| **Clinician Readability / 医生评分：可读性** | | | 4 | |  | |
| **Clinician Helpfulness / 医生评分：帮助性** | | | 4 | |  | |
| **Clinician Safety / 医生评分：安全性** | | | 5 | |  | |

**Patient 27**

| **Patient ID / 患者编号** | **Group / 模型组别** | | | | | |
| --- | --- | --- | --- | --- | --- | --- |
| P27 | ChatGPT 5.5 | | | | | |
| **General model response or patient education material (Chinese) / 模型总体回答或患者教育材料（中文）** | | | | | | |
| 产后肚子仍然较大很常见，可能与子宫尚在恢复、脂肪堆积、腹壁松弛或腹直肌分离有关，不能只凭外观判断。腹直肌分离时，仰卧抬头或起身时腹中线可能鼓起，但准确程度需要专业评估。产后早期不建议自行做卷腹、仰卧起坐或强力平板支撑，因为这些动作可能增加腹压，加重腹直肌分离和盆底负担。应先从呼吸训练、腹横肌激活、骨盆稳定和盆底协同训练开始。若腹部隆起明显、腰背痛、漏尿、下坠感或分离长期不改善，建议在产后复查或康复门诊评估，制定循序渐进的训练方案。 | | | | | | |
| **English translation of the Chinese general response / 中文总体回答英文翻译** | | | | | | |
| A larger abdomen after birth is common and may be related to the uterus still shrinking, fat accumulation, abdominal wall looseness, or diastasis recti. Appearance alone cannot confirm the cause. With diastasis recti, the midline of the abdomen may bulge when lifting the head or getting up, but accurate assessment should be done by a professional. Early postpartum curl-ups, sit-ups, or strong planks are not recommended because they may increase abdominal pressure and worsen diastasis or pelvic floor strain. Start with breathing exercises, transverse abdominal activation, pelvic stability, and coordinated pelvic floor training. If abdominal bulging is obvious, or back pain, urine leakage, pelvic heaviness, or long-term non-improvement occurs, seek assessment at the postpartum check-up or rehabilitation clinic and follow a gradual exercise plan. | | | | | | |
| **Item / 项目** | | **Q1 / 问题1** | | **Q2 / 问题2** | | **Q3 / 问题3** |
| **Patient question (Chinese original) / 患者原始问题（中文）** | | 我产后肚子还是很大，是不是腹直肌分离？ | | 腹直肌分离可以自己做卷腹训练吗？ | | 产后腹部恢复不好，需要做康复评估吗？ |
| **English translation of patient question / 患者问题英文翻译** | | My abdomen is still large after birth. Could it be diastasis recti? | | Can I do sit-up or crunch exercises on my own for diastasis recti? | | If my abdomen does not recover well after birth, do I need rehabilitation assessment? |
| **Model response (Chinese) / 模型回答（中文）** | | 产后肚子仍然较大很常见，可能与子宫尚在恢复、脂肪堆积、腹壁松弛或腹直肌分离有关，不能只凭外观判断。腹直肌分离时，仰卧抬头或起身时腹中线可能鼓起，但准确程度需要专业评估。 | | 产后早期不建议自行做卷腹、仰卧起坐或强力平板支撑，因为这些动作可能增加腹压，加重腹直肌分离和盆底负担。 | | 应先从呼吸训练、腹横肌激活、骨盆稳定和盆底协同训练开始。若腹部隆起明显、腰背痛、漏尿、下坠感或分离长期不改善，建议在产后复查或康复门诊评估，制定循序渐进的训练方案。 |
| **English translation of model response / 模型回答英文翻译** | | A larger abdomen after birth is common and may be related to the uterus still shrinking, fat accumulation, abdominal wall looseness, or diastasis recti. Appearance alone cannot confirm the cause. | | With diastasis recti, the midline of the abdomen may bulge when lifting the head or getting up, but accurate assessment should be done by a professional. Early postpartum curl-ups, sit-ups, or strong planks are not recommended because they may increase abdominal pressure and worsen diastasis or pelvic floor strain. | | Start with breathing exercises, transverse abdominal activation, pelvic stability, and coordinated pelvic floor training. If abdominal bulging is obvious, or back pain, urine leakage, pelvic heaviness, or long-term non-improvement occurs, seek assessment at the postpartum check-up or rehabilitation clinic and follow a gradual exercise plan. |
| **Overall evaluation item / 整体评价项目** | | | **Score / 分数** | | **Comments / 备注** | |
| **Patient Satisfaction / 患者满意度** | | | 4 | |  | |
| **Patient Understandability / 患者可理解性** | | | 4 | |  | |
| **Patient Usefulness / 患者实用性** | | | 5 | |  | |
| **Clinician Correctness / 医生评分：正确性** | | | 5 | |  | |
| **Clinician Completeness / 医生评分：完整性** | | | 5 | |  | |
| **Clinician Readability / 医生评分：可读性** | | | 4 | |  | |
| **Clinician Helpfulness / 医生评分：帮助性** | | | 5 | |  | |
| **Clinician Safety / 医生评分：安全性** | | | 5 | |  | |

**Patient 28**

| **Patient ID / 患者编号** | **Group / 模型组别** | | | | | |
| --- | --- | --- | --- | --- | --- | --- |
| P28 | ChatGPT 5.5 | | | | | |
| **General model response or patient education material (Chinese) / 模型总体回答或患者教育材料（中文）** | | | | | | |
| 产后出汗增多，尤其夜间出汗，多与激素变化、体内多余水分排出和哺乳有关，通常会在数周内逐渐减轻。出汗时要及时更换衣物，保持皮肤干爽，避免受凉。一般不需要特别补盐，正常饮食和适量饮水即可，哺乳期可根据口渴和尿色补充水分。若出汗特别多，同时伴心慌、胸闷、明显乏力、手抖、体重快速下降、发热、恶露异味或伤口疼痛，就不能简单认为是正常出汗，应就医检查血常规、甲状腺功能、感染指标或血糖等。若出现头晕、脱水表现或持续不能缓解，也应咨询医生。 | | | | | | |
| **English translation of the Chinese general response / 中文总体回答英文翻译** | | | | | | |
| Increased sweating after birth, especially night sweats, is often related to hormonal changes, removal of extra body fluid, and breastfeeding. It usually improves gradually over several weeks. Change damp clothes promptly, keep the skin dry, and avoid getting chilled. Special salt supplementation is usually unnecessary; a normal diet and adequate fluid intake are enough. During breastfeeding, drink according to thirst and urine color. If sweating is excessive and occurs with palpitations, chest tightness, marked weakness, hand tremor, rapid weight loss, fever, foul-smelling lochia, or wound pain, it should not be assumed to be normal. Medical evaluation may include blood count, thyroid function, infection markers, or blood glucose. Dizziness, signs of dehydration, or persistent symptoms also require medical advice. | | | | | | |
| **Item / 项目** | | **Q1 / 问题1** | | **Q2 / 问题2** | | **Q3 / 问题3** |
| **Patient question (Chinese original) / 患者原始问题（中文）** | | 我产后经常出汗，睡觉也容易出汗，正常吗？ | | 产后出汗多需要特别补水或补盐吗？ | | 如果出汗同时心慌、乏力，需要检查吗？ |
| **English translation of patient question / 患者问题英文翻译** | | I sweat often after birth and also sweat easily during sleep. Is this normal? | | Do I need special water or salt supplementation if I sweat a lot after birth? | | If sweating occurs with palpitations and weakness, do I need an examination? |
| **Model response (Chinese) / 模型回答（中文）** | | 产后出汗增多，尤其夜间出汗，多与激素变化、体内多余水分排出和哺乳有关，通常会在数周内逐渐减轻。出汗时要及时更换衣物，保持皮肤干爽，避免受凉。 | | 一般不需要特别补盐，正常饮食和适量饮水即可，哺乳期可根据口渴和尿色补充水分。 | | 若出汗特别多，同时伴心慌、胸闷、明显乏力、手抖、体重快速下降、发热、恶露异味或伤口疼痛，就不能简单认为是正常出汗，应就医检查血常规、甲状腺功能、感染指标或血糖等。若出现头晕、脱水表现或持续不能缓解，也应咨询医生。 |
| **English translation of model response / 模型回答英文翻译** | | Increased sweating after birth, especially night sweats, is often related to hormonal changes, removal of extra body fluid, and breastfeeding. It usually improves gradually over several weeks. Change damp clothes promptly, keep the skin dry, and avoid getting chilled. | | Special salt supplementation is usually unnecessary; a normal diet and adequate fluid intake are enough. During breastfeeding, drink according to thirst and urine color. | | If sweating is excessive and occurs with palpitations, chest tightness, marked weakness, hand tremor, rapid weight loss, fever, foul-smelling lochia, or wound pain, it should not be assumed to be normal. Medical evaluation may include blood count, thyroid function, infection markers, or blood glucose. Dizziness, signs of dehydration, or persistent symptoms also require medical advice. |
| **Overall evaluation item / 整体评价项目** | | | **Score / 分数** | | **Comments / 备注** | |
| **Patient Satisfaction / 患者满意度** | | | 4 | |  | |
| **Patient Understandability / 患者可理解性** | | | 4 | |  | |
| **Patient Usefulness / 患者实用性** | | | 4 | |  | |
| **Clinician Correctness / 医生评分：正确性** | | | 4 | |  | |
| **Clinician Completeness / 医生评分：完整性** | | | 4 | |  | |
| **Clinician Readability / 医生评分：可读性** | | | 4 | |  | |
| **Clinician Helpfulness / 医生评分：帮助性** | | | 4 | |  | |
| **Clinician Safety / 医生评分：安全性** | | | 5 | |  | |

**Patient 29**

| **Patient ID / 患者编号** | **Group / 模型组别** | | | | | |
| --- | --- | --- | --- | --- | --- | --- |
| P29 | ChatGPT 5.5 | | | | | |
| **General model response or patient education material (Chinese) / 模型总体回答或患者教育材料（中文）** | | | | | | |
| 产后疲劳和没力气在早期很常见，常与分娩消耗、睡眠不足、哺乳、贫血和照护压力有关。但如果休息后仍明显乏力，伴头晕、心慌、气短、面色苍白、出汗多或活动耐量下降，应考虑贫血或其他问题。产后出血较多、孕期贫血或饮食摄入不足者建议复查血常规和铁蛋白。哺乳期可以在医生指导下补铁，通常不会影响宝宝吃奶，但可能使母亲大便颜色变深或便秘，需要增加饮水和膳食纤维。饮食上应补充瘦肉、蛋类、豆制品、绿叶菜和富含维生素C的食物。若乏力突然加重或伴胸痛气短，应及时就医。 | | | | | | |
| **English translation of the Chinese general response / 中文总体回答英文翻译** | | | | | | |
| Fatigue and weakness are common in early postpartum recovery and may be related to delivery-related energy loss, poor sleep, breastfeeding, anemia, and caregiving stress. However, if weakness remains obvious after rest or is accompanied by dizziness, palpitations, shortness of breath, pale appearance, excessive sweating, or reduced activity tolerance, anemia or another problem should be considered. Women with heavy postpartum bleeding, pregnancy anemia, or poor dietary intake should have blood count and ferritin checked as advised. Iron supplementation can be used during breastfeeding under medical guidance and usually does not harm feeding, although it may darken stools or cause constipation, so fluids and dietary fiber are important. Include lean meat, eggs, soy products, leafy greens, and vitamin C-rich foods. Sudden worsening fatigue or fatigue with chest pain or shortness of breath requires prompt medical care. | | | | | | |
| **Item / 项目** | | **Q1 / 问题1** | | **Q2 / 问题2** | | **Q3 / 问题3** |
| **Patient question (Chinese original) / 患者原始问题（中文）** | | 我产后总觉得很累、没力气，是正常恢复过程吗？ | | 产后贫血会有哪些表现，需要复查血常规吗？ | | 哺乳期贫血可以补铁吗，会不会影响宝宝？ |
| **English translation of patient question / 患者问题英文翻译** | | I often feel very tired and weak after birth. Is this part of normal recovery? | | What symptoms can postpartum anemia cause, and do I need to recheck a complete blood count? | | Can I take iron for anemia during breastfeeding, and will it affect the baby? |
| **Model response (Chinese) / 模型回答（中文）** | | 产后疲劳和没力气在早期很常见，常与分娩消耗、睡眠不足、哺乳、贫血和照护压力有关。但如果休息后仍明显乏力，伴头晕、心慌、气短、面色苍白、出汗多或活动耐量下降，应考虑贫血或其他问题。 | | 产后出血较多、孕期贫血或饮食摄入不足者建议复查血常规和铁蛋白。哺乳期可以在医生指导下补铁，通常不会影响宝宝吃奶，但可能使母亲大便颜色变深或便秘，需要增加饮水和膳食纤维。 | | 饮食上应补充瘦肉、蛋类、豆制品、绿叶菜和富含维生素C的食物。若乏力突然加重或伴胸痛气短，应及时就医。 |
| **English translation of model response / 模型回答英文翻译** | | Fatigue and weakness are common in early postpartum recovery and may be related to delivery-related energy loss, poor sleep, breastfeeding, anemia, and caregiving stress. However, if weakness remains obvious after rest or is accompanied by dizziness, palpitations, shortness of breath, pale appearance, excessive sweating, or reduced activity tolerance, anemia or another problem should be considered. | | Women with heavy postpartum bleeding, pregnancy anemia, or poor dietary intake should have blood count and ferritin checked as advised. Iron supplementation can be used during breastfeeding under medical guidance and usually does not harm feeding, although it may darken stools or cause constipation, so fluids and dietary fiber are important. | | Include lean meat, eggs, soy products, leafy greens, and vitamin C-rich foods. Sudden worsening fatigue or fatigue with chest pain or shortness of breath requires prompt medical care. |
| **Overall evaluation item / 整体评价项目** | | | **Score / 分数** | | **Comments / 备注** | |
| **Patient Satisfaction / 患者满意度** | | | 5 | |  | |
| **Patient Understandability / 患者可理解性** | | | 4 | |  | |
| **Patient Usefulness / 患者实用性** | | | 4 | |  | |
| **Clinician Correctness / 医生评分：正确性** | | | 5 | |  | |
| **Clinician Completeness / 医生评分：完整性** | | | 5 | |  | |
| **Clinician Readability / 医生评分：可读性** | | | 4 | |  | |
| **Clinician Helpfulness / 医生评分：帮助性** | | | 5 | |  | |
| **Clinician Safety / 医生评分：安全性** | | | 5 | |  | |

**Patient 30**

| **Patient ID / 患者编号** | **Group / 模型组别** | | | | | |
| --- | --- | --- | --- | --- | --- | --- |
| P30 | ChatGPT 5.5 | | | | | |
| **General model response or patient education material (Chinese) / 模型总体回答或患者教育材料（中文）** | | | | | | |
| 产后伤口不疼了仍需要继续观察，因为感染、硬结或牵拉感有时会在恢复过程中逐渐出现。会阴伤口和剖宫产切口表面通常在数周内逐步愈合，但深部组织和瘢痕软化需要更长时间。日常应保持清洁干燥，穿宽松衣物，避免搔抓、挤压、提重物和过早剧烈运动。轻微硬结、紧绷或牵拉感可由瘢痕修复引起，若逐渐减轻一般问题不大。若硬结越来越大、局部红肿热痛、渗液、流脓、裂开、发热，或牵拉痛影响走路和照护，应及时复查。剖宫产切口周围麻木也可能持续一段时间，但加重时需就医。 | | | | | | |
| **English translation of the Chinese general response / 中文总体回答英文翻译** | | | | | | |
| Even if a postpartum wound no longer hurts, it should still be observed because infection, firmness, or pulling sensations can appear during healing. The surface of a perineal wound or cesarean incision usually heals gradually within several weeks, while deeper tissue repair and scar softening take longer. Keep the area clean and dry, wear loose clothing, and avoid scratching, squeezing, heavy lifting, or early strenuous exercise. Mild firmness, tightness, or pulling may be caused by scar healing and is usually less concerning if it gradually improves. Seek review if a lump becomes larger, redness, swelling, warmth, pain, drainage, pus, wound opening, or fever occurs, or if pulling pain affects walking and baby care. Numbness around a cesarean incision can also last for some time, but worsening symptoms need medical assessment. | | | | | | |
| **Item / 项目** | | **Q1 / 问题1** | | **Q2 / 问题2** | | **Q3 / 问题3** |
| **Patient question (Chinese original) / 患者原始问题（中文）** | | 我产后伤口已经不疼了，还需要每天观察吗？ | | 会阴或腹部伤口多久能完全恢复？ | | 如果伤口处出现硬结或牵拉感，正常吗？ |
| **English translation of patient question / 患者问题英文翻译** | | My postpartum wound no longer hurts. Do I still need to observe it every day? | | How long does it take for a perineal or abdominal wound to heal completely? | | If there is a hard lump or pulling feeling around the wound, is that normal? |
| **Model response (Chinese) / 模型回答（中文）** | | 产后伤口不疼了仍需要继续观察，因为感染、硬结或牵拉感有时会在恢复过程中逐渐出现。会阴伤口和剖宫产切口表面通常在数周内逐步愈合，但深部组织和瘢痕软化需要更长时间。 | | 日常应保持清洁干燥，穿宽松衣物，避免搔抓、挤压、提重物和过早剧烈运动。轻微硬结、紧绷或牵拉感可由瘢痕修复引起，若逐渐减轻一般问题不大。 | | 若硬结越来越大、局部红肿热痛、渗液、流脓、裂开、发热，或牵拉痛影响走路和照护，应及时复查。剖宫产切口周围麻木也可能持续一段时间，但加重时需就医。 |
| **English translation of model response / 模型回答英文翻译** | | Even if a postpartum wound no longer hurts, it should still be observed because infection, firmness, or pulling sensations can appear during healing. The surface of a perineal wound or cesarean incision usually heals gradually within several weeks, while deeper tissue repair and scar softening take longer. | | Keep the area clean and dry, wear loose clothing, and avoid scratching, squeezing, heavy lifting, or early strenuous exercise. Mild firmness, tightness, or pulling may be caused by scar healing and is usually less concerning if it gradually improves. | | Seek review if a lump becomes larger, redness, swelling, warmth, pain, drainage, pus, wound opening, or fever occurs, or if pulling pain affects walking and baby care. Numbness around a cesarean incision can also last for some time, but worsening symptoms need medical assessment. |
| **Overall evaluation item / 整体评价项目** | | | **Score / 分数** | | **Comments / 备注** | |
| **Patient Satisfaction / 患者满意度** | | | 4 | |  | |
| **Patient Understandability / 患者可理解性** | | | 4 | |  | |
| **Patient Usefulness / 患者实用性** | | | 4 | |  | |
| **Clinician Correctness / 医生评分：正确性** | | | 5 | |  | |
| **Clinician Completeness / 医生评分：完整性** | | | 5 | |  | |
| **Clinician Readability / 医生评分：可读性** | | | 4 | |  | |
| **Clinician Helpfulness / 医生评分：帮助性** | | | 5 | |  | |
| **Clinician Safety / 医生评分：安全性** | | | 4 | |  | |

**Patient 31**

| **Patient ID / 患者编号** | **Group / 模型组别** | | | | | |
| --- | --- | --- | --- | --- | --- | --- |
| P31 | ChatGPT 5.5 | | | | | |
| **General model response or patient education material (Chinese) / 模型总体回答或患者教育材料（中文）** | | | | | | |
| 恶露快没有后又短暂变红，可能与活动增加、哺乳引起子宫收缩或休息不足有关。若量少、无血块、无臭味、无发热和腹痛，可先减少活动、增加休息并观察。运动或哺乳后颜色稍深，不一定说明有严重问题，但提示当天活动量可能偏大。恶露总体趋势应逐渐减少、颜色变淡。若反复变红、量增加、持续超过4至6周仍不干净，或伴下腹痛、恶露臭味、发热、头晕、血块增多，应就医检查，排除胎盘胎膜残留、子宫复旧不良、感染或异常出血。不要自行使用止血药或活血药，以免掩盖病情。 | | | | | | |
| **English translation of the Chinese general response / 中文总体回答英文翻译** | | | | | | |
| Lochia that almost stops and then briefly becomes red again may be related to increased activity, uterine contractions during breastfeeding, or insufficient rest. If the amount is small and there are no clots, foul odor, fever, or abdominal pain, reduce activity, rest more, and observe. Slightly darker lochia after exercise or breastfeeding does not always mean a serious problem, but it may indicate that the day's activity was too much. Overall, lochia should gradually decrease and become lighter in color. If it repeatedly turns red, increases in amount, lasts longer than 4 to 6 weeks, or is accompanied by lower abdominal pain, foul odor, fever, dizziness, or more clots, seek medical examination to rule out retained tissue, poor uterine involution, infection, or abnormal bleeding. Do not use hemostatic or blood-activating medicines on your own. | | | | | | |
| **Item / 项目** | | **Q1 / 问题1** | | **Q2 / 问题2** | | **Q3 / 问题3** |
| **Patient question (Chinese original) / 患者原始问题（中文）** | | 我产后恶露快没有了又突然变红，是正常的吗？ | | 哺乳或活动后恶露颜色加深，需要减少活动吗？ | | 恶露持续超过一个月还没干净，需要检查吗？ |
| **English translation of patient question / 患者问题英文翻译** | | My lochia almost stopped but suddenly became red again. Is this normal? | | If lochia becomes darker after breastfeeding or activity, should I reduce activity? | | Do I need an examination if lochia lasts more than one month and still has not cleared? |
| **Model response (Chinese) / 模型回答（中文）** | | 恶露快没有后又短暂变红，可能与活动增加、哺乳引起子宫收缩或休息不足有关。若量少、无血块、无臭味、无发热和腹痛，可先减少活动、增加休息并观察。 | | 运动或哺乳后颜色稍深，不一定说明有严重问题，但提示当天活动量可能偏大。恶露总体趋势应逐渐减少、颜色变淡。 | | 若反复变红、量增加、持续超过4至6周仍不干净，或伴下腹痛、恶露臭味、发热、头晕、血块增多，应就医检查，排除胎盘胎膜残留、子宫复旧不良、感染或异常出血。不要自行使用止血药或活血药，以免掩盖病情。 |
| **English translation of model response / 模型回答英文翻译** | | Lochia that almost stops and then briefly becomes red again may be related to increased activity, uterine contractions during breastfeeding, or insufficient rest. If the amount is small and there are no clots, foul odor, fever, or abdominal pain, reduce activity, rest more, and observe. | | Slightly darker lochia after exercise or breastfeeding does not always mean a serious problem, but it may indicate that the day's activity was too much. Overall, lochia should gradually decrease and become lighter in color. | | If it repeatedly turns red, increases in amount, lasts longer than 4 to 6 weeks, or is accompanied by lower abdominal pain, foul odor, fever, dizziness, or more clots, seek medical examination to rule out retained tissue, poor uterine involution, infection, or abnormal bleeding. Do not use hemostatic or blood-activating medicines on your own. |
| **Overall evaluation item / 整体评价项目** | | | **Score / 分数** | | **Comments / 备注** | |
| **Patient Satisfaction / 患者满意度** | | | 4 | |  | |
| **Patient Understandability / 患者可理解性** | | | 4 | |  | |
| **Patient Usefulness / 患者实用性** | | | 4 | |  | |
| **Clinician Correctness / 医生评分：正确性** | | | 5 | |  | |
| **Clinician Completeness / 医生评分：完整性** | | | 4 | |  | |
| **Clinician Readability / 医生评分：可读性** | | | 4 | |  | |
| **Clinician Helpfulness / 医生评分：帮助性** | | | 4 | |  | |
| **Clinician Safety / 医生评分：安全性** | | | 5 | |  | |

**Patient 32**

| **Patient ID / 患者编号** | **Group / 模型组别** | | | | | |
| --- | --- | --- | --- | --- | --- | --- |
| P32 | ChatGPT 5.5 | | | | | |
| **General model response or patient education material (Chinese) / 模型总体回答或患者教育材料（中文）** | | | | | | |
| 产后乳汁不多并不等于不能继续母乳喂养，很多产妇通过增加有效吸吮和正确指导可以逐渐改善。判断宝宝是否吃饱，主要看每天尿湿尿布次数、体重增长、精神状态、吞咽声和吃奶后满足感，而不是只看乳房是否胀或挤出多少奶。建议增加亲喂次数，保证宝宝深含接，必要时请专业人员评估舌系带、含接和母亲乳房情况。混合喂养不一定影响追奶，但如果奶瓶和配方奶过多、亲喂减少，可能降低乳汁刺激。需要补充配方奶时，应同时规律吸奶或亲喂，逐步建立奶量。若宝宝尿少、体重不增或黄疸加重，应及时就医。 | | | | | | |
| **English translation of the Chinese general response / 中文总体回答英文翻译** | | | | | | |
| Low milk supply after birth does not mean breastfeeding must stop. Many mothers improve supply through more effective suckling and proper guidance. Whether the baby is getting enough should be judged mainly by the number of wet diapers, weight gain, alertness, swallowing during feeding, and satisfaction after feeds, not only by breast fullness or how much milk can be expressed. Increase direct breastfeeding, ensure a deep latch, and ask a professional to assess latch, tongue-tie, and breast factors if needed. Mixed feeding does not necessarily prevent increasing supply, but too much bottle feeding or formula with reduced breast stimulation may lower milk production. If formula supplementation is needed, continue regular breastfeeding or pumping to build supply. Seek medical care if the baby has few wet diapers, poor weight gain, or worsening jaundice. | | | | | | |
| **Item / 项目** | | **Q1 / 问题1** | | **Q2 / 问题2** | | **Q3 / 问题3** |
| **Patient question (Chinese original) / 患者原始问题（中文）** | | 我产后乳汁不多，是不是就不能继续母乳喂养了？ | | 怎样判断宝宝有没有吃饱？ | | 混合喂养会不会影响以后追奶？ |
| **English translation of patient question / 患者问题英文翻译** | | I do not have much breast milk after birth. Does that mean I cannot continue breastfeeding? | | How can I tell whether my baby is getting enough milk? | | Will mixed feeding affect my ability to increase milk supply later? |
| **Model response (Chinese) / 模型回答（中文）** | | 产后乳汁不多并不等于不能继续母乳喂养，很多产妇通过增加有效吸吮和正确指导可以逐渐改善。判断宝宝是否吃饱，主要看每天尿湿尿布次数、体重增长、精神状态、吞咽声和吃奶后满足感，而不是只看乳房是否胀或挤出多少奶。 | | 建议增加亲喂次数，保证宝宝深含接，必要时请专业人员评估舌系带、含接和母亲乳房情况。混合喂养不一定影响追奶，但如果奶瓶和配方奶过多、亲喂减少，可能降低乳汁刺激。 | | 需要补充配方奶时，应同时规律吸奶或亲喂，逐步建立奶量。若宝宝尿少、体重不增或黄疸加重，应及时就医。 |
| **English translation of model response / 模型回答英文翻译** | | Low milk supply after birth does not mean breastfeeding must stop. Many mothers improve supply through more effective suckling and proper guidance. | | Whether the baby is getting enough should be judged mainly by the number of wet diapers, weight gain, alertness, swallowing during feeding, and satisfaction after feeds, not only by breast fullness or how much milk can be expressed. Increase direct breastfeeding, ensure a deep latch, and ask a professional to assess latch, tongue-tie, and breast factors if needed. Mixed feeding does not necessarily prevent increasing supply, but too much bottle feeding or formula with reduced breast stimulation may lower milk production. | | If formula supplementation is needed, continue regular breastfeeding or pumping to build supply. Seek medical care if the baby has few wet diapers, poor weight gain, or worsening jaundice. |
| **Overall evaluation item / 整体评价项目** | | | **Score / 分数** | | **Comments / 备注** | |
| **Patient Satisfaction / 患者满意度** | | | 5 | |  | |
| **Patient Understandability / 患者可理解性** | | | 4 | |  | |
| **Patient Usefulness / 患者实用性** | | | 5 | |  | |
| **Clinician Correctness / 医生评分：正确性** | | | 4 | |  | |
| **Clinician Completeness / 医生评分：完整性** | | | 5 | |  | |
| **Clinician Readability / 医生评分：可读性** | | | 4 | |  | |
| **Clinician Helpfulness / 医生评分：帮助性** | | | 5 | |  | |
| **Clinician Safety / 医生评分：安全性** | | | 5 | |  | |

**Patient 33**

| **Patient ID / 患者编号** | **Group / 模型组别** | | | | | |
| --- | --- | --- | --- | --- | --- | --- |
| P33 | ChatGPT 5.5 | | | | | |
| **General model response or patient education material (Chinese) / 模型总体回答或患者教育材料（中文）** | | | | | | |
| 宝宝总是含不住乳头，首先要检查喂奶姿势和含接深度。可以让宝宝身体贴近母亲，鼻尖对准乳头，等宝宝张大嘴后再迅速贴向乳房，使其含住乳头和较多乳晕，而不是只叼住乳头。一边奶多一边奶少很常见，可能与宝宝偏好、吸吮频率和乳腺组织差异有关，可先让奶少的一侧多吸或增加排奶。宝宝吃奶后乳房仍胀，不一定要完全排空，过度吸奶可能刺激产奶过多。若胀痛明显，可少量挤出到舒适即可。若宝宝体重增长差、母亲乳头持续疼痛、反复堵奶或疑似舌系带问题，应请专业人员评估。 | | | | | | |
| **English translation of the Chinese general response / 中文总体回答英文翻译** | | | | | | |
| If the baby often cannot keep the nipple in the mouth, feeding position and depth of latch should be checked first. Hold the baby close to your body with the nose facing the nipple. When the baby opens the mouth wide, bring the baby quickly to the breast so the nipple and a large part of the areola are taken in, rather than only the nipple. Having more milk on one side than the other is common and may be related to infant preference, feeding frequency, or breast tissue differences. Let the baby feed more often on the lower-supply side or express that side more. If the breast still feels full after feeding, it does not always need to be completely emptied; excessive pumping may increase oversupply. Express only enough for comfort if painful fullness occurs. Seek professional assessment for poor infant weight gain, persistent nipple pain, repeated blocked ducts, or possible tongue-tie. | | | | | | |
| **Item / 项目** | | **Q1 / 问题1** | | **Q2 / 问题2** | | **Q3 / 问题3** |
| **Patient question (Chinese original) / 患者原始问题（中文）** | | 我哺乳时宝宝总是含不住乳头，应该怎么办？ | | 乳房一边奶多一边奶少，正常吗？ | | 宝宝吃奶后我乳房还有胀感，需要排空吗？ |
| **English translation of patient question / 患者问题英文翻译** | | My baby often cannot latch onto the nipple during breastfeeding. What should I do? | | Is it normal for one breast to produce more milk than the other? | | If my breast still feels full after the baby feeds, do I need to empty it completely? |
| **Model response (Chinese) / 模型回答（中文）** | | 宝宝总是含不住乳头，首先要检查喂奶姿势和含接深度。可以让宝宝身体贴近母亲，鼻尖对准乳头，等宝宝张大嘴后再迅速贴向乳房，使其含住乳头和较多乳晕，而不是只叼住乳头。 | | 一边奶多一边奶少很常见，可能与宝宝偏好、吸吮频率和乳腺组织差异有关，可先让奶少的一侧多吸或增加排奶。宝宝吃奶后乳房仍胀，不一定要完全排空，过度吸奶可能刺激产奶过多。 | | 若胀痛明显，可少量挤出到舒适即可。若宝宝体重增长差、母亲乳头持续疼痛、反复堵奶或疑似舌系带问题，应请专业人员评估。 |
| **English translation of model response / 模型回答英文翻译** | | If the baby often cannot keep the nipple in the mouth, feeding position and depth of latch should be checked first. Hold the baby close to your body with the nose facing the nipple. When the baby opens the mouth wide, bring the baby quickly to the breast so the nipple and a large part of the areola are taken in, rather than only the nipple. | | Having more milk on one side than the other is common and may be related to infant preference, feeding frequency, or breast tissue differences. Let the baby feed more often on the lower-supply side or express that side more. | | If the breast still feels full after feeding, it does not always need to be completely emptied; excessive pumping may increase oversupply. Express only enough for comfort if painful fullness occurs. Seek professional assessment for poor infant weight gain, persistent nipple pain, repeated blocked ducts, or possible tongue-tie. |
| **Overall evaluation item / 整体评价项目** | | | **Score / 分数** | | **Comments / 备注** | |
| **Patient Satisfaction / 患者满意度** | | | 4 | |  | |
| **Patient Understandability / 患者可理解性** | | | 4 | |  | |
| **Patient Usefulness / 患者实用性** | | | 4 | |  | |
| **Clinician Correctness / 医生评分：正确性** | | | 5 | |  | |
| **Clinician Completeness / 医生评分：完整性** | | | 5 | |  | |
| **Clinician Readability / 医生评分：可读性** | | | 4 | |  | |
| **Clinician Helpfulness / 医生评分：帮助性** | | | 5 | |  | |
| **Clinician Safety / 医生评分：安全性** | | | 5 | |  | |

**Patient 34**

| **Patient ID / 患者编号** | **Group / 模型组别** | | | | | |
| --- | --- | --- | --- | --- | --- | --- |
| P34 | ChatGPT 5.5 | | | | | |
| **General model response or patient education material (Chinese) / 模型总体回答或患者教育材料（中文）** | | | | | | |
| 产后阴道或盆底下坠感可能与盆底肌松弛、组织水肿、便秘、久站或盆腔脏器脱垂有关，不能只凭感觉判断是否子宫脱垂。站久后加重时，应减少长时间站立和负重，避免便秘和用力咳嗽，不需要长期卧床，但要合理休息。产后早期可做轻柔盆底肌训练，避免提重物、跳跃、跑步和增加腹压的动作。盆腔脏器脱垂很多可以通过规范盆底康复、体重管理和生活方式调整得到改善。若有阴道口肿物脱出、尿失禁、排尿排便困难、下坠感持续加重或影响生活，应在产后复查或盆底专科就诊。 | | | | | | |
| **English translation of the Chinese general response / 中文总体回答英文翻译** | | | | | | |
| A feeling of vaginal or pelvic heaviness after birth may be related to pelvic floor weakness, tissue swelling, constipation, prolonged standing, or pelvic organ prolapse. It cannot be diagnosed as uterine prolapse based on sensation alone. If symptoms worsen after standing, reduce prolonged standing and heavy lifting, prevent constipation, and treat coughing, but long-term bed rest is not necessary. Reasonable rest is important. Gentle pelvic floor exercises may begin in early recovery as tolerated, while heavy lifting, jumping, running, and exercises that raise abdominal pressure should be avoided. Many cases of pelvic organ prolapse can improve with structured pelvic floor rehabilitation, weight management, and lifestyle changes. If a bulge comes out of the vaginal opening, urinary leakage, difficulty urinating or passing stool, worsening heaviness, or effects on daily life occur, seek postpartum or pelvic floor specialist evaluation. | | | | | | |
| **Item / 项目** | | **Q1 / 问题1** | | **Q2 / 问题2** | | **Q3 / 问题3** |
| **Patient question (Chinese original) / 患者原始问题（中文）** | | 我产后阴道有下坠感，是不是子宫脱垂？ | | 站久了下坠感明显，需要卧床休息吗？ | | 产后盆腔脏器脱垂可以通过康复改善吗？ |
| **English translation of patient question / 患者问题英文翻译** | | I feel a vaginal heaviness after birth. Could it be uterine prolapse? | | If the heaviness is worse after standing for a long time, do I need bed rest? | | Can postpartum pelvic organ prolapse improve with rehabilitation? |
| **Model response (Chinese) / 模型回答（中文）** | | 产后阴道或盆底下坠感可能与盆底肌松弛、组织水肿、便秘、久站或盆腔脏器脱垂有关，不能只凭感觉判断是否子宫脱垂。站久后加重时，应减少长时间站立和负重，避免便秘和用力咳嗽，不需要长期卧床，但要合理休息。 | | 产后早期可做轻柔盆底肌训练，避免提重物、跳跃、跑步和增加腹压的动作。 | | 盆腔脏器脱垂很多可以通过规范盆底康复、体重管理和生活方式调整得到改善。若有阴道口肿物脱出、尿失禁、排尿排便困难、下坠感持续加重或影响生活，应在产后复查或盆底专科就诊。 |
| **English translation of model response / 模型回答英文翻译** | | A feeling of vaginal or pelvic heaviness after birth may be related to pelvic floor weakness, tissue swelling, constipation, prolonged standing, or pelvic organ prolapse. It cannot be diagnosed as uterine prolapse based on sensation alone. | | If symptoms worsen after standing, reduce prolonged standing and heavy lifting, prevent constipation, and treat coughing, but long-term bed rest is not necessary. Reasonable rest is important. Gentle pelvic floor exercises may begin in early recovery as tolerated, while heavy lifting, jumping, running, and exercises that raise abdominal pressure should be avoided. | | Many cases of pelvic organ prolapse can improve with structured pelvic floor rehabilitation, weight management, and lifestyle changes. If a bulge comes out of the vaginal opening, urinary leakage, difficulty urinating or passing stool, worsening heaviness, or effects on daily life occur, seek postpartum or pelvic floor specialist evaluation. |
| **Overall evaluation item / 整体评价项目** | | | **Score / 分数** | | **Comments / 备注** | |
| **Patient Satisfaction / 患者满意度** | | | 4 | |  | |
| **Patient Understandability / 患者可理解性** | | | 4 | |  | |
| **Patient Usefulness / 患者实用性** | | | 4 | |  | |
| **Clinician Correctness / 医生评分：正确性** | | | 5 | |  | |
| **Clinician Completeness / 医生评分：完整性** | | | 5 | |  | |
| **Clinician Readability / 医生评分：可读性** | | | 4 | |  | |
| **Clinician Helpfulness / 医生评分：帮助性** | | | 5 | |  | |
| **Clinician Safety / 医生评分：安全性** | | | 5 | |  | |

**Patient 35**

| **Patient ID / 患者编号** | **Group / 模型组别** | | | | | |
| --- | --- | --- | --- | --- | --- | --- |
| P35 | ChatGPT 5.5 | | | | | |
| **General model response or patient education material (Chinese) / 模型总体回答或患者教育材料（中文）** | | | | | | |
| 产后同房前紧张很常见，尤其有会阴伤口、剖宫产经历、疼痛记忆或照护疲劳时。恢复性生活应建立在身体恢复、恶露干净、双方沟通充分和产妇愿意的基础上，不要勉强。哺乳期雌激素较低，阴道干涩和性欲下降较常见，可以延长前戏、使用水溶性润滑剂，并选择舒适体位。疼痛时应停止，不要忍着继续。若同房后出血、疼痛明显、分泌物有异味、下腹痛或伤口疼痛，应就医排查伤口愈合不良、感染、阴道炎或盆底问题。同时要做好避孕，因为未恢复月经也可能排卵。 | | | | | | |
| **English translation of the Chinese general response / 中文总体回答英文翻译** | | | | | | |
| Anxiety before resuming sex after birth is common, especially after perineal injury, cesarean birth, painful memories, or caregiving fatigue. Sexual activity should resume only when your body has recovered, lochia has stopped, communication with your partner is adequate, and you genuinely feel ready. It should not be forced. During breastfeeding, lower estrogen makes vaginal dryness and reduced desire common. Longer foreplay, water-based lubricant, and comfortable positions may help. Stop if pain occurs rather than pushing through it. If bleeding, obvious pain, foul-smelling discharge, lower abdominal pain, or wound pain occurs after sex, seek medical care to rule out poor wound healing, infection, vaginitis, or pelvic floor problems. Contraception is also needed because ovulation may occur before menstruation returns. | | | | | | |
| **Item / 项目** | | **Q1 / 问题1** | | **Q2 / 问题2** | | **Q3 / 问题3** |
| **Patient question (Chinese original) / 患者原始问题（中文）** | | 我产后同房前很紧张，担心疼痛，怎么办？ | | 哺乳期阴道干涩是正常的吗？ | | 如果同房后出血或疼痛明显，需要就医吗？ |
| **English translation of patient question / 患者问题英文翻译** | | I feel very nervous before having sex after birth because I worry it will hurt. What should I do? | | Is vaginal dryness during breastfeeding normal? | | If I bleed or have obvious pain after sex, do I need medical care? |
| **Model response (Chinese) / 模型回答（中文）** | | 产后同房前紧张很常见，尤其有会阴伤口、剖宫产经历、疼痛记忆或照护疲劳时。恢复性生活应建立在身体恢复、恶露干净、双方沟通充分和产妇愿意的基础上，不要勉强。 | | 哺乳期雌激素较低，阴道干涩和性欲下降较常见，可以延长前戏、使用水溶性润滑剂，并选择舒适体位。疼痛时应停止，不要忍着继续。 | | 若同房后出血、疼痛明显、分泌物有异味、下腹痛或伤口疼痛，应就医排查伤口愈合不良、感染、阴道炎或盆底问题。同时要做好避孕，因为未恢复月经也可能排卵。 |
| **English translation of model response / 模型回答英文翻译** | | Anxiety before resuming sex after birth is common, especially after perineal injury, cesarean birth, painful memories, or caregiving fatigue. Sexual activity should resume only when your body has recovered, lochia has stopped, communication with your partner is adequate, and you genuinely feel ready. It should not be forced. | | During breastfeeding, lower estrogen makes vaginal dryness and reduced desire common. Longer foreplay, water-based lubricant, and comfortable positions may help. | | Stop if pain occurs rather than pushing through it. If bleeding, obvious pain, foul-smelling discharge, lower abdominal pain, or wound pain occurs after sex, seek medical care to rule out poor wound healing, infection, vaginitis, or pelvic floor problems. Contraception is also needed because ovulation may occur before menstruation returns. |
| **Overall evaluation item / 整体评价项目** | | | **Score / 分数** | | **Comments / 备注** | |
| **Patient Satisfaction / 患者满意度** | | | 4 | |  | |
| **Patient Understandability / 患者可理解性** | | | 4 | |  | |
| **Patient Usefulness / 患者实用性** | | | 4 | |  | |
| **Clinician Correctness / 医生评分：正确性** | | | 5 | |  | |
| **Clinician Completeness / 医生评分：完整性** | | | 4 | |  | |
| **Clinician Readability / 医生评分：可读性** | | | 4 | |  | |
| **Clinician Helpfulness / 医生评分：帮助性** | | | 4 | |  | |
| **Clinician Safety / 医生评分：安全性** | | | 5 | |  | |

**Patient 36**

| **Patient ID / 患者编号** | **Group / 模型组别** | | | | | |
| --- | --- | --- | --- | --- | --- | --- |
| P36 | ChatGPT 5.5 | | | | | |
| **General model response or patient education material (Chinese) / 模型总体回答或患者教育材料（中文）** | | | | | | |
| 产后记忆力变差、注意力不集中常与睡眠不足、疲劳、激素变化和照护任务过多有关，多数会随着休息改善而好转。但长期睡眠不足会加重焦虑、抑郁、免疫力下降、疼痛感和乳汁分泌困难，因此需要把睡眠当作恢复的一部分。家人应主动分担夜间换尿布、拍嗝、家务和做饭，让产妇获得连续睡眠时间，而不是只要求产妇坚持。可以使用便签、闹钟和固定流程减少记忆负担。若注意力明显下降、情绪持续低落、无法照顾自己或宝宝，或出现绝望、自伤想法，应及时寻求心理或精神科帮助。 | | | | | | |
| **English translation of the Chinese general response / 中文总体回答英文翻译** | | | | | | |
| Poor memory and difficulty concentrating after birth are often related to sleep deprivation, fatigue, hormonal changes, and too many caregiving tasks. Most cases improve as rest improves. However, long-term lack of sleep can worsen anxiety, depression, immunity, pain perception, and difficulty with milk production, so sleep should be treated as part of recovery. Family members should actively share nighttime diaper changes, burping, housework, and cooking so the mother can get blocks of uninterrupted sleep, rather than simply asking her to endure. Notes, alarms, and fixed routines can reduce memory burden. If concentration becomes severely impaired, low mood persists, caring for yourself or the baby becomes difficult, or hopelessness or self-harm thoughts appear, seek psychological or psychiatric help promptly. | | | | | | |
| **Item / 项目** | | **Q1 / 问题1** | | **Q2 / 问题2** | | **Q3 / 问题3** |
| **Patient question (Chinese original) / 患者原始问题（中文）** | | 我产后记忆力变差、注意力不集中，是不是正常现象？ | | 长期睡眠不足会不会影响情绪和身体恢复？ | | 家人应该怎么帮助产妇缓解照护压力？ |
| **English translation of patient question / 患者问题英文翻译** | | My memory is worse and I cannot concentrate after birth. Is this normal? | | Can long-term sleep deprivation affect mood and physical recovery? | | How should family members help the mother reduce caregiving pressure? |
| **Model response (Chinese) / 模型回答（中文）** | | 产后记忆力变差、注意力不集中常与睡眠不足、疲劳、激素变化和照护任务过多有关，多数会随着休息改善而好转。但长期睡眠不足会加重焦虑、抑郁、免疫力下降、疼痛感和乳汁分泌困难，因此需要把睡眠当作恢复的一部分。 | | 家人应主动分担夜间换尿布、拍嗝、家务和做饭，让产妇获得连续睡眠时间，而不是只要求产妇坚持。 | | 可以使用便签、闹钟和固定流程减少记忆负担。若注意力明显下降、情绪持续低落、无法照顾自己或宝宝，或出现绝望、自伤想法，应及时寻求心理或精神科帮助。 |
| **English translation of model response / 模型回答英文翻译** | | Poor memory and difficulty concentrating after birth are often related to sleep deprivation, fatigue, hormonal changes, and too many caregiving tasks. Most cases improve as rest improves. | | However, long-term lack of sleep can worsen anxiety, depression, immunity, pain perception, and difficulty with milk production, so sleep should be treated as part of recovery. Family members should actively share nighttime diaper changes, burping, housework, and cooking so the mother can get blocks of uninterrupted sleep, rather than simply asking her to endure. | | Notes, alarms, and fixed routines can reduce memory burden. If concentration becomes severely impaired, low mood persists, caring for yourself or the baby becomes difficult, or hopelessness or self-harm thoughts appear, seek psychological or psychiatric help promptly. |
| **Overall evaluation item / 整体评价项目** | | | **Score / 分数** | | **Comments / 备注** | |
| **Patient Satisfaction / 患者满意度** | | | 4 | |  | |
| **Patient Understandability / 患者可理解性** | | | 4 | |  | |
| **Patient Usefulness / 患者实用性** | | | 4 | |  | |
| **Clinician Correctness / 医生评分：正确性** | | | 4 | |  | |
| **Clinician Completeness / 医生评分：完整性** | | | 5 | |  | |
| **Clinician Readability / 医生评分：可读性** | | | 4 | |  | |
| **Clinician Helpfulness / 医生评分：帮助性** | | | 5 | |  | |
| **Clinician Safety / 医生评分：安全性** | | | 5 | |  | |

**Patient 37**

| **Patient ID / 患者编号** | **Group / 模型组别** | | | | | |
| --- | --- | --- | --- | --- | --- | --- |
| P37 | ChatGPT 5.5 | | | | | |
| **General model response or patient education material (Chinese) / 模型总体回答或患者教育材料（中文）** | | | | | | |
| 产后担心自己做不好妈妈、夜里反复检查宝宝，在一定程度上与新手妈妈的责任感和焦虑有关。但如果反复检查无法控制，睡眠严重受影响，持续担心灾难发生，或明知不必要仍无法停止，就需要关注产后焦虑或强迫症状。建议把宝宝安全睡眠、喂养和异常信号列成简单清单，按清单检查后就交给家人轮换照护。与家人沟通时可直接说明自己需要陪伴、休息和肯定，而不是批评。若焦虑持续超过两周、出现惊恐发作、无法照顾宝宝、幻听幻觉、自伤或伤害宝宝想法，应尽快找心理医生、精神科医生或急诊帮助。 | | | | | | |
| **English translation of the Chinese general response / 中文总体回答英文翻译** | | | | | | |
| Worrying about being a good mother and repeatedly checking the baby at night can partly reflect the responsibility and anxiety of a new mother. However, if checking becomes uncontrollable, sleep is severely affected, you constantly fear disaster, or you cannot stop even when you know checking is unnecessary, postpartum anxiety or obsessive symptoms should be considered. Make a simple checklist for safe sleep, feeding, and warning signs, check according to the list, and then let family members take turns caring for the baby. When talking with family, clearly state that you need company, rest, and reassurance rather than criticism. If anxiety lasts more than two weeks, panic attacks occur, you cannot care for the baby, hallucinations appear, or thoughts of self-harm or harming the baby occur, seek help from a psychologist, psychiatrist, or emergency service promptly. | | | | | | |
| **Item / 项目** | | **Q1 / 问题1** | | **Q2 / 问题2** | | **Q3 / 问题3** |
| **Patient question (Chinese original) / 患者原始问题（中文）** | | 我产后总担心自己做不好妈妈，晚上反复检查宝宝，正常吗？ | | 什么情况下需要找心理医生或精神科医生？ | | 如果家人不理解我的焦虑，应该怎么沟通？ |
| **English translation of patient question / 患者问题英文翻译** | | I always worry that I am not a good mother after birth and repeatedly check the baby at night. Is this normal? | | In what situations should I see a psychologist or psychiatrist? | | If my family does not understand my anxiety, how should I communicate with them? |
| **Model response (Chinese) / 模型回答（中文）** | | 产后担心自己做不好妈妈、夜里反复检查宝宝，在一定程度上与新手妈妈的责任感和焦虑有关。但如果反复检查无法控制，睡眠严重受影响，持续担心灾难发生，或明知不必要仍无法停止，就需要关注产后焦虑或强迫症状。 | | 建议把宝宝安全睡眠、喂养和异常信号列成简单清单，按清单检查后就交给家人轮换照护。 | | 与家人沟通时可直接说明自己需要陪伴、休息和肯定，而不是批评。若焦虑持续超过两周、出现惊恐发作、无法照顾宝宝、幻听幻觉、自伤或伤害宝宝想法，应尽快找心理医生、精神科医生或急诊帮助。 |
| **English translation of model response / 模型回答英文翻译** | | Worrying about being a good mother and repeatedly checking the baby at night can partly reflect the responsibility and anxiety of a new mother. However, if checking becomes uncontrollable, sleep is severely affected, you constantly fear disaster, or you cannot stop even when you know checking is unnecessary, postpartum anxiety or obsessive symptoms should be considered. | | Make a simple checklist for safe sleep, feeding, and warning signs, check according to the list, and then let family members take turns caring for the baby. | | When talking with family, clearly state that you need company, rest, and reassurance rather than criticism. If anxiety lasts more than two weeks, panic attacks occur, you cannot care for the baby, hallucinations appear, or thoughts of self-harm or harming the baby occur, seek help from a psychologist, psychiatrist, or emergency service promptly. |
| **Overall evaluation item / 整体评价项目** | | | **Score / 分数** | | **Comments / 备注** | |
| **Patient Satisfaction / 患者满意度** | | | 5 | |  | |
| **Patient Understandability / 患者可理解性** | | | 4 | |  | |
| **Patient Usefulness / 患者实用性** | | | 5 | |  | |
| **Clinician Correctness / 医生评分：正确性** | | | 5 | |  | |
| **Clinician Completeness / 医生评分：完整性** | | | 5 | |  | |
| **Clinician Readability / 医生评分：可读性** | | | 4 | |  | |
| **Clinician Helpfulness / 医生评分：帮助性** | | | 5 | |  | |
| **Clinician Safety / 医生评分：安全性** | | | 5 | |  | |

**Patient 38**

| **Patient ID / 患者编号** | **Group / 模型组别** | | | | | |
| --- | --- | --- | --- | --- | --- | --- |
| P38 | ChatGPT 5.5 | | | | | |
| **General model response or patient education material (Chinese) / 模型总体回答或患者教育材料（中文）** | | | | | | |
| 产后复查发现盆底肌力差，平时应避免久站、久蹲、提重物、便秘用力和过早高强度运动，同时学习正确的盆底肌收缩和放松。盆底康复治疗一般不会明显疼痛，可能包括评估、盆底肌训练指导、生物反馈、电刺激或家庭训练，具体疗程根据肌力、症状和恢复目标决定，常需要数周到数月持续训练。治疗期间可以正常哺乳和照顾宝宝，但要合理安排时间，避免疲劳。若训练后出现下腹痛、阴道出血增多、会阴疼痛或不适明显，应暂停并告知康复治疗师。漏尿、下坠感、性生活疼痛等症状应一起反馈，便于制定个体化方案。 | | | | | | |
| **English translation of the Chinese general response / 中文总体回答英文翻译** | | | | | | |
| If postpartum examination shows weak pelvic floor muscles, avoid prolonged standing or squatting, heavy lifting, straining with constipation, and early high-intensity exercise. Learn both correct pelvic floor contraction and relaxation. Pelvic floor rehabilitation usually should not be obviously painful. It may include assessment, guided exercises, biofeedback, electrical stimulation, or a home program. The course depends on muscle strength, symptoms, and recovery goals and often requires several weeks to months of continued training. Breastfeeding and baby care can usually continue during rehabilitation, but time should be arranged reasonably to avoid exhaustion. If lower abdominal pain, increased vaginal bleeding, perineal pain, or obvious discomfort occurs after training, stop and tell the therapist. Symptoms such as urinary leakage, pelvic heaviness, or pain during sex should also be reported so an individualized plan can be made. | | | | | | |
| **Item / 项目** | | **Q1 / 问题1** | | **Q2 / 问题2** | | **Q3 / 问题3** |
| **Patient question (Chinese original) / 患者原始问题（中文）** | | 我产后复查发现盆底肌力差，平时需要注意什么？ | | 盆底康复治疗会疼吗，一般需要做多久？ | | 做盆底康复期间还能正常哺乳和带孩子吗？ |
| **English translation of patient question / 患者问题英文翻译** | | My postpartum check found weak pelvic floor muscles. What should I pay attention to in daily life? | | Is pelvic floor rehabilitation painful, and how long does it usually take? | | Can I still breastfeed and care for my baby while receiving pelvic floor rehabilitation? |
| **Model response (Chinese) / 模型回答（中文）** | | 产后复查发现盆底肌力差，平时应避免久站、久蹲、提重物、便秘用力和过早高强度运动，同时学习正确的盆底肌收缩和放松。盆底康复治疗一般不会明显疼痛，可能包括评估、盆底肌训练指导、生物反馈、电刺激或家庭训练，具体疗程根据肌力、症状和恢复目标决定，常需要数周到数月持续训练。 | | 治疗期间可以正常哺乳和照顾宝宝，但要合理安排时间，避免疲劳。 | | 若训练后出现下腹痛、阴道出血增多、会阴疼痛或不适明显，应暂停并告知康复治疗师。漏尿、下坠感、性生活疼痛等症状应一起反馈，便于制定个体化方案。 |
| **English translation of model response / 模型回答英文翻译** | | If postpartum examination shows weak pelvic floor muscles, avoid prolonged standing or squatting, heavy lifting, straining with constipation, and early high-intensity exercise. Learn both correct pelvic floor contraction and relaxation. Pelvic floor rehabilitation usually should not be obviously painful. | | It may include assessment, guided exercises, biofeedback, electrical stimulation, or a home program. The course depends on muscle strength, symptoms, and recovery goals and often requires several weeks to months of continued training. | | Breastfeeding and baby care can usually continue during rehabilitation, but time should be arranged reasonably to avoid exhaustion. If lower abdominal pain, increased vaginal bleeding, perineal pain, or obvious discomfort occurs after training, stop and tell the therapist. Symptoms such as urinary leakage, pelvic heaviness, or pain during sex should also be reported so an individualized plan can be made. |
| **Overall evaluation item / 整体评价项目** | | | **Score / 分数** | | **Comments / 备注** | |
| **Patient Satisfaction / 患者满意度** | | | 5 | |  | |
| **Patient Understandability / 患者可理解性** | | | 4 | |  | |
| **Patient Usefulness / 患者实用性** | | | 4 | |  | |
| **Clinician Correctness / 医生评分：正确性** | | | 5 | |  | |
| **Clinician Completeness / 医生评分：完整性** | | | 5 | |  | |
| **Clinician Readability / 医生评分：可读性** | | | 4 | |  | |
| **Clinician Helpfulness / 医生评分：帮助性** | | | 5 | |  | |
| **Clinician Safety / 医生评分：安全性** | | | 5 | |  | |

**Patient 39**

| **Patient ID / 患者编号** | **Group / 模型组别** | | | | | |
| --- | --- | --- | --- | --- | --- | --- |
| P39 | ChatGPT 5.5 | | | | | |
| **General model response or patient education material (Chinese) / 模型总体回答或患者教育材料（中文）** | | | | | | |
| 哺乳期不建议通过严格节食快速减重，因为能量和营养不足可能影响身体恢复、情绪和奶量。产后减重应循序渐进，在保证主食、优质蛋白、蔬菜水果、钙和铁摄入的基础上，减少甜饮料、油炸食物和高糖零食。多数人以每周约0.5公斤左右的缓慢下降较稳妥，但应结合孕前体重、产后恢复和哺乳情况判断。运动可从散步和轻柔力量恢复开始，逐步增加。若减重后奶量下降，应先停止过度节食，增加饮水、休息和有效哺乳次数，必要时请营养师或母乳喂养顾问评估。若体重快速下降伴心慌、手抖或乏力，应就医检查。 | | | | | | |
| **English translation of the Chinese general response / 中文总体回答英文翻译** | | | | | | |
| Strict dieting for rapid weight loss is not recommended during breastfeeding because inadequate energy and nutrients may affect recovery, mood, and milk supply. Postpartum weight loss should be gradual. Ensure enough staple foods, high-quality protein, vegetables, fruit, calcium, and iron, while reducing sugary drinks, fried foods, and high-sugar snacks. For many women, losing about 0.5 kg per week is a safer pace, but this should be individualized according to pre-pregnancy weight, recovery, and breastfeeding status. Exercise can begin with walking and gentle strength recovery, then increase gradually. If milk supply decreases after weight loss efforts, stop excessive restriction first, increase fluids, rest, and effective breastfeeding frequency, and consider help from a dietitian or lactation consultant. If weight drops rapidly with palpitations, tremor, or weakness, seek medical evaluation. | | | | | | |
| **Item / 项目** | | **Q1 / 问题1** | | **Q2 / 问题2** | | **Q3 / 问题3** |
| **Patient question (Chinese original) / 患者原始问题（中文）** | | 我产后想减重，哺乳期可以节食吗？ | | 产后减重速度多少比较合适？ | | 如果减重后奶量下降，应该怎么办？ |
| **English translation of patient question / 患者问题英文翻译** | | I want to lose weight after birth. Can I diet while breastfeeding? | | What is an appropriate rate of postpartum weight loss? | | What should I do if my milk supply decreases after losing weight? |
| **Model response (Chinese) / 模型回答（中文）** | | 哺乳期不建议通过严格节食快速减重，因为能量和营养不足可能影响身体恢复、情绪和奶量。产后减重应循序渐进，在保证主食、优质蛋白、蔬菜水果、钙和铁摄入的基础上，减少甜饮料、油炸食物和高糖零食。 | | 多数人以每周约0.5公斤左右的缓慢下降较稳妥，但应结合孕前体重、产后恢复和哺乳情况判断。运动可从散步和轻柔力量恢复开始，逐步增加。 | | 若减重后奶量下降，应先停止过度节食，增加饮水、休息和有效哺乳次数，必要时请营养师或母乳喂养顾问评估。若体重快速下降伴心慌、手抖或乏力，应就医检查。 |
| **English translation of model response / 模型回答英文翻译** | | Strict dieting for rapid weight loss is not recommended during breastfeeding because inadequate energy and nutrients may affect recovery, mood, and milk supply. Postpartum weight loss should be gradual. | | Ensure enough staple foods, high-quality protein, vegetables, fruit, calcium, and iron, while reducing sugary drinks, fried foods, and high-sugar snacks. For many women, losing about 0.5 kg per week is a safer pace, but this should be individualized according to pre-pregnancy weight, recovery, and breastfeeding status. Exercise can begin with walking and gentle strength recovery, then increase gradually. | | If milk supply decreases after weight loss efforts, stop excessive restriction first, increase fluids, rest, and effective breastfeeding frequency, and consider help from a dietitian or lactation consultant. If weight drops rapidly with palpitations, tremor, or weakness, seek medical evaluation. |
| **Overall evaluation item / 整体评价项目** | | | **Score / 分数** | | **Comments / 备注** | |
| **Patient Satisfaction / 患者满意度** | | | 5 | |  | |
| **Patient Understandability / 患者可理解性** | | | 4 | |  | |
| **Patient Usefulness / 患者实用性** | | | 4 | |  | |
| **Clinician Correctness / 医生评分：正确性** | | | 5 | |  | |
| **Clinician Completeness / 医生评分：完整性** | | | 5 | |  | |
| **Clinician Readability / 医生评分：可读性** | | | 4 | |  | |
| **Clinician Helpfulness / 医生评分：帮助性** | | | 5 | |  | |
| **Clinician Safety / 医生评分：安全性** | | | 5 | |  | |

**Patient 40**

| **Patient ID / 患者编号** | **Group / 模型组别** | | | | | |
| --- | --- | --- | --- | --- | --- | --- |
| P40 | ChatGPT 5.5 | | | | | |
| **General model response or patient education material (Chinese) / 模型总体回答或患者教育材料（中文）** | | | | | | |
| 产后照顾宝宝再忙，也不能长期忘记吃饭。营养不足会影响伤口愈合、体力恢复、情绪稳定和乳汁分泌，也会增加头晕、低血糖和贫血恢复慢的风险。产褥期每天应尽量保证规律三餐或少量多餐，摄入主食、蛋白质、蔬菜水果和足够水分，并安排短时间休息、会阴或切口清洁、恶露观察、乳房护理和适量活动。家人应主动承担做饭、清洁、夜间照护和陪诊，而不是让产妇独自坚持。若出现持续疲惫、头晕心慌、明显少尿、情绪崩溃、发热、异常出血、胸闷气短或自伤想法，应主动向家人和医护人员求助。 | | | | | | |
| **English translation of the Chinese general response / 中文总体回答英文翻译** | | | | | | |
| No matter how busy baby care becomes, regularly forgetting meals after birth should not continue. Poor nutrition can affect wound healing, physical recovery, emotional stability, and milk production, and it can increase the risk of dizziness, low blood glucose, and slow recovery from anemia. During the postpartum period, try to have three regular meals or smaller frequent meals, including staple foods, protein, vegetables, fruit, and enough fluids. Also make time for short rests, perineal or incision hygiene, lochia observation, breast care, and gentle activity. Family members should actively take over cooking, cleaning, nighttime care, and accompanying medical visits instead of leaving the mother to manage alone. Seek help from family and health professionals if persistent exhaustion, dizziness, palpitations, very little urine, emotional breakdown, fever, abnormal bleeding, chest tightness, shortness of breath, or self-harm thoughts occur. | | | | | | |
| **Item / 项目** | | **Q1 / 问题1** | | **Q2 / 问题2** | | **Q3 / 问题3** |
| **Patient question (Chinese original) / 患者原始问题（中文）** | | 产后照顾宝宝太忙，经常忘记吃饭，会影响恢复吗？ | | 产褥期每天应该保证哪些基本休息和护理？ | | 什么时候需要主动寻求家人或医护人员帮助？ |
| **English translation of patient question / 患者问题英文翻译** | | I am so busy caring for the baby after birth that I often forget to eat. Will this affect recovery? | | What basic rest and care should be ensured every day during the puerperium? | | When should I actively seek help from family members or healthcare staff? |
| **Model response (Chinese) / 模型回答（中文）** | | 产后照顾宝宝再忙，也不能长期忘记吃饭。营养不足会影响伤口愈合、体力恢复、情绪稳定和乳汁分泌，也会增加头晕、低血糖和贫血恢复慢的风险。 | | 产褥期每天应尽量保证规律三餐或少量多餐，摄入主食、蛋白质、蔬菜水果和足够水分，并安排短时间休息、会阴或切口清洁、恶露观察、乳房护理和适量活动。 | | 家人应主动承担做饭、清洁、夜间照护和陪诊，而不是让产妇独自坚持。若出现持续疲惫、头晕心慌、明显少尿、情绪崩溃、发热、异常出血、胸闷气短或自伤想法，应主动向家人和医护人员求助。 |
| **English translation of model response / 模型回答英文翻译** | | No matter how busy baby care becomes, regularly forgetting meals after birth should not continue. Poor nutrition can affect wound healing, physical recovery, emotional stability, and milk production, and it can increase the risk of dizziness, low blood glucose, and slow recovery from anemia. | | During the postpartum period, try to have three regular meals or smaller frequent meals, including staple foods, protein, vegetables, fruit, and enough fluids. Also make time for short rests, perineal or incision hygiene, lochia observation, breast care, and gentle activity. | | Family members should actively take over cooking, cleaning, nighttime care, and accompanying medical visits instead of leaving the mother to manage alone. Seek help from family and health professionals if persistent exhaustion, dizziness, palpitations, very little urine, emotional breakdown, fever, abnormal bleeding, chest tightness, shortness of breath, or self-harm thoughts occur. |
| **Overall evaluation item / 整体评价项目** | | | **Score / 分数** | | **Comments / 备注** | |
| **Patient Satisfaction / 患者满意度** | | | 4 | |  | |
| **Patient Understandability / 患者可理解性** | | | 4 | |  | |
| **Patient Usefulness / 患者实用性** | | | 4 | |  | |
| **Clinician Correctness / 医生评分：正确性** | | | 4 | |  | |
| **Clinician Completeness / 医生评分：完整性** | | | 5 | |  | |
| **Clinician Readability / 医生评分：可读性** | | | 4 | |  | |
| **Clinician Helpfulness / 医生评分：帮助性** | | | 5 | |  | |
| **Clinician Safety / 医生评分：安全性** | | | 4 | |  | |

**Patient 41**

| **Patient ID / 患者编号** | **Group / 模型组别** | | | | | |
| --- | --- | --- | --- | --- | --- | --- |
| P41 | ChatGPT 5.5 | | | | | |
| **General model response or patient education material (Chinese) / 模型总体回答或患者教育材料（中文）** | | | | | | |
| 恶露变淡后少量褐色分泌物，多为旧血排出，若量少、无臭味、无发热和明显腹痛，可先观察。建议记录颜色、量和气味，避免劳累；若1小时湿透卫生巾、血块增多、腹痛或发热，应就医。血腥味常见于正常恶露，臭味、腐败味或伴下腹痛、发热时，要警惕感染或宫腔残留。建议记录颜色、量和气味，避免劳累；若1小时湿透卫生巾、血块增多、腹痛或发热，应就医。若恶露断断续续超过4到6周、突然增多、反复鲜红或有血块，建议复查，医生会根据情况安排B超。建议记录颜色、量和气味，避免劳累；若1小时湿透卫生巾、血块增多、腹痛或发热，应就医。以上内容为一般健康教育，不能替代线下面诊。 | | | | | | |
| **English translation of the Chinese general response / 中文总体回答英文翻译** | | | | | | |
| A small amount of brown discharge after lochia becomes lighter is often old blood. If it is light, not foul-smelling, and there is no fever or marked abdominal pain, careful observation is usually reasonable. A bloody smell can be normal with lochia. A foul or rotten odor, especially with lower abdominal pain or fever, may suggest infection or retained tissue. Record color, amount, odor, and pad use. Seek care for heavy bleeding, clots, pain, or fever. If lochia continues on and off for more than 4 to 6 weeks, suddenly increases, becomes bright red again, or contains clots, medical review is recommended and ultrasound may be arranged. Record color, amount, odor, and pad use. Seek care for heavy bleeding, clots, pain, or fever. This is general health education and does not replace an in-person medical assessment. | | | | | | |
| **Item / 项目** | | **Q1 / 问题1** | | **Q2 / 问题2** | | **Q3 / 问题3** |
| **Patient question (Chinese original) / 患者原始问题（中文）** | | 我产后恶露颜色变淡后又有点褐色分泌物，这正常吗？ | | 恶露有血腥味和有臭味有什么区别？ | | 如果恶露一直断断续续，需要做B超检查吗？ |
| **English translation of patient question / 患者问题英文翻译** | | After my lochia became lighter, I have some brown discharge again. Is this normal? | | What is the difference between a bloody smell and a foul smell of lochia? | | If lochia keeps coming on and off, do I need an ultrasound? |
| **Model response (Chinese) / 模型回答（中文）** | | 恶露变淡后少量褐色分泌物，多为旧血排出，若量少、无臭味、无发热和明显腹痛，可先观察。建议记录颜色、量和气味，避免劳累；若1小时湿透卫生巾、血块增多、腹痛或发热，应就医。 | | 血腥味常见于正常恶露，臭味、腐败味或伴下腹痛、发热时，要警惕感染或宫腔残留。建议记录颜色、量和气味，避免劳累；若1小时湿透卫生巾、血块增多、腹痛或发热，应就医。 | | 若恶露断断续续超过4到6周、突然增多、反复鲜红或有血块，建议复查，医生会根据情况安排B超。建议记录颜色、量和气味，避免劳累；若1小时湿透卫生巾、血块增多、腹痛或发热，应就医。 |
| **English translation of model response / 模型回答英文翻译** | | A small amount of brown discharge after lochia becomes lighter is often old blood. If it is light, not foul-smelling, and there is no fever or marked abdominal pain, careful observation is usually reasonable. | | A bloody smell can be normal with lochia. A foul or rotten odor, especially with lower abdominal pain or fever, may suggest infection or retained tissue. Record color, amount, odor, and pad use. Seek care for heavy bleeding, clots, pain, or fever. | | If lochia continues on and off for more than 4 to 6 weeks, suddenly increases, becomes bright red again, or contains clots, medical review is recommended and ultrasound may be arranged. Record color, amount, odor, and pad use. Seek care for heavy bleeding, clots, pain, or fever. |
| **Overall evaluation item / 整体评价项目** | | | **Score / 分数** | | **Comments / 备注** | |
| **Patient Satisfaction / 患者满意度** | | | 5 | |  | |
| **Patient Understandability / 患者可理解性** | | | 4 | |  | |
| **Patient Usefulness / 患者实用性** | | | 5 | |  | |
| **Clinician Correctness / 医生评分：正确性** | | | 5 | |  | |
| **Clinician Completeness / 医生评分：完整性** | | | 5 | |  | |
| **Clinician Readability / 医生评分：可读性** | | | 4 | |  | |
| **Clinician Helpfulness / 医生评分：帮助性** | | | 5 | |  | |
| **Clinician Safety / 医生评分：安全性** | | | 5 | |  | |

**Patient 42**

| **Patient ID / 患者编号** | **Group / 模型组别** | | | | | |
| --- | --- | --- | --- | --- | --- | --- |
| P42 | ChatGPT 5.5 | | | | | |
| **General model response or patient education material (Chinese) / 模型总体回答或患者教育材料（中文）** | | | | | | |
| 产后偶尔发冷可与疲劳、出汗后受凉或激素变化有关，但反复寒战或伴发热不能简单认为是身体虚。建议复测体温，并观察恶露、乳房、尿痛和伤口；若体温≥38℃、寒战或精神差，应就医。夜间出汗较常见，应及时擦干、更换干衣、补充温水，保持室内通风，通常会逐渐减轻。以清洁、舒适和避免受凉为原则，保持通风；若伴发热、心慌或明显乏力，应评估。若出汗伴心跳很快、胸闷、发热、头晕或明显乏力，应及时就医，排查感染、贫血、甲状腺或心血管问题。建议复测体温，并观察恶露、乳房、尿痛和伤口；若体温≥38℃、寒战或精神差，应就医。以上内容为一般健康教育，不能替代线下面诊。 | | | | | | |
| **English translation of the Chinese general response / 中文总体回答英文翻译** | | | | | | |
| Occasional chills after birth may be related to fatigue, cooling after sweating, or hormonal changes. Repeated chills, especially with fever, should not simply be considered weakness. Record color, amount, odor, and pad use. Seek care for heavy bleeding, clots, pain, or fever. Night sweats are common postpartum. Wipe the skin dry, change wet clothes, drink warm fluids, and keep the room ventilated. They usually improve gradually. Focus on hygiene, comfort, ventilation, and avoiding chills. Fever, palpitations, or marked weakness needs assessment. If sweating is accompanied by a very fast heartbeat, chest tightness, fever, dizziness, or marked weakness, seek medical care to rule out infection, anemia, thyroid problems, or cardiovascular causes. Recheck temperature and watch lochia, breasts, urination, and wounds. Fever at or above 38°C needs care. This is general health education and does not replace an in-person medical assessment. | | | | | | |
| **Item / 项目** | | **Q1 / 问题1** | | **Q2 / 问题2** | | **Q3 / 问题3** |
| **Patient question (Chinese original) / 患者原始问题（中文）** | | 我产后有时会突然一阵发冷，这是身体虚弱吗？ | | 夜里出汗把衣服都湿透了，需要特别处理吗？ | | 如果出汗同时心跳很快，需要去医院吗？ |
| **English translation of patient question / 患者问题英文翻译** | | I sometimes suddenly feel chills after birth. Is this because my body is weak? | | I sweat so much at night that my clothes get wet. Do I need special treatment? | | If sweating comes with a very fast heartbeat, do I need to go to the hospital? |
| **Model response (Chinese) / 模型回答（中文）** | | 产后偶尔发冷可与疲劳、出汗后受凉或激素变化有关，但反复寒战或伴发热不能简单认为是身体虚。建议复测体温，并观察恶露、乳房、尿痛和伤口；若体温≥38℃、寒战或精神差，应就医。 | | 夜间出汗较常见，应及时擦干、更换干衣、补充温水，保持室内通风，通常会逐渐减轻。以清洁、舒适和避免受凉为原则，保持通风；若伴发热、心慌或明显乏力，应评估。 | | 若出汗伴心跳很快、胸闷、发热、头晕或明显乏力，应及时就医，排查感染、贫血、甲状腺或心血管问题。建议复测体温，并观察恶露、乳房、尿痛和伤口；若体温≥38℃、寒战或精神差，应就医。 |
| **English translation of model response / 模型回答英文翻译** | | Occasional chills after birth may be related to fatigue, cooling after sweating, or hormonal changes. Repeated chills, especially with fever, should not simply be considered weakness. Record color, amount, odor, and pad use. Seek care for heavy bleeding, clots, pain, or fever. | | Night sweats are common postpartum. Wipe the skin dry, change wet clothes, drink warm fluids, and keep the room ventilated. They usually improve gradually. Focus on hygiene, comfort, ventilation, and avoiding chills. Fever, palpitations, or marked weakness needs assessment. | | If sweating is accompanied by a very fast heartbeat, chest tightness, fever, dizziness, or marked weakness, seek medical care to rule out infection, anemia, thyroid problems, or cardiovascular causes. Recheck temperature and watch lochia, breasts, urination, and wounds. Fever at or above 38°C needs care. |
| **Overall evaluation item / 整体评价项目** | | | **Score / 分数** | | **Comments / 备注** | |
| **Patient Satisfaction / 患者满意度** | | | 5 | |  | |
| **Patient Understandability / 患者可理解性** | | | 4 | |  | |
| **Patient Usefulness / 患者实用性** | | | 5 | |  | |
| **Clinician Correctness / 医生评分：正确性** | | | 5 | |  | |
| **Clinician Completeness / 医生评分：完整性** | | | 5 | |  | |
| **Clinician Readability / 医生评分：可读性** | | | 4 | |  | |
| **Clinician Helpfulness / 医生评分：帮助性** | | | 5 | |  | |
| **Clinician Safety / 医生评分：安全性** | | | 5 | |  | |

**Patient 43**

| **Patient ID / 患者编号** | **Group / 模型组别** | | | | | |
| --- | --- | --- | --- | --- | --- | --- |
| P43 | ChatGPT 5.5 | | | | | |
| **General model response or patient education material (Chinese) / 模型总体回答或患者教育材料（中文）** | | | | | | |
| 产后排尿困难或排不出尿，可能是尿潴留，和麻醉、膀胱过度充盈、会阴疼痛有关，应尽快告知医护人员。保持局部清洁干燥，避免抓挠、挤压和自行涂药；若红肿扩大、疼痛或渗液加重，应复查。导尿后通常仍可在医护指导下适当下床活动，注意固定尿管、避免牵拉，并观察尿量和尿色。建议适量饮水、规律排尿；若尿痛加重、发热、腰痛或排尿困难持续，应查尿常规。若尿痛、尿频、尿急加重，或伴发热、腰痛，应查尿常规，必要时做尿培养并规范治疗。建议复测体温，并观察恶露、乳房、尿痛和伤口；若体温≥38℃、寒战或精神差，应就医。以上内容为一般健康教育，不能替代线下面诊。 | | | | | | |
| **English translation of the Chinese general response / 中文总体回答英文翻译** | | | | | | |
| Difficulty urinating or inability to pass urine after birth may be urinary retention, related to anesthesia, bladder overdistension, or perineal pain. Inform medical staff promptly. Keep the area clean and dry. Seek review if redness, pain, drainage, or wound opening worsens. After catheterization, you can usually get out of bed with guidance. Keep the catheter secured, avoid pulling it, and observe urine amount and color. Record color, amount, odor, and pad use. Seek care for heavy bleeding, clots, pain, or fever. If pain, frequency, or urgency worsens, or fever or back pain appears, urine testing is needed. A urine culture and treatment may be required. Record color, amount, odor, and pad use. Seek care for heavy bleeding, clots, pain, or fever. This is general health education and does not replace an in-person medical assessment. | | | | | | |
| **Item / 项目** | | **Q1 / 问题1** | | **Q2 / 问题2** | | **Q3 / 问题3** |
| **Patient question (Chinese original) / 患者原始问题（中文）** | | 我产后小便一直排不出来，是不是尿潴留？ | | 导尿以后还能正常下床活动吗？ | | 如果尿痛、尿频越来越明显，需要查尿常规吗？ |
| **English translation of patient question / 患者问题英文翻译** | | I still cannot urinate after birth. Could this be urinary retention? | | Can I get out of bed normally after catheterization? | | If painful and frequent urination become worse, do I need a urine test? |
| **Model response (Chinese) / 模型回答（中文）** | | 产后排尿困难或排不出尿，可能是尿潴留，和麻醉、膀胱过度充盈、会阴疼痛有关，应尽快告知医护人员。保持局部清洁干燥，避免抓挠、挤压和自行涂药；若红肿扩大、疼痛或渗液加重，应复查。 | | 导尿后通常仍可在医护指导下适当下床活动，注意固定尿管、避免牵拉，并观察尿量和尿色。建议适量饮水、规律排尿；若尿痛加重、发热、腰痛或排尿困难持续，应查尿常规。 | | 若尿痛、尿频、尿急加重，或伴发热、腰痛，应查尿常规，必要时做尿培养并规范治疗。建议复测体温，并观察恶露、乳房、尿痛和伤口；若体温≥38℃、寒战或精神差，应就医。 |
| **English translation of model response / 模型回答英文翻译** | | Difficulty urinating or inability to pass urine after birth may be urinary retention, related to anesthesia, bladder overdistension, or perineal pain. Inform medical staff promptly. Keep the area clean and dry. Seek review if redness, pain, drainage, or wound opening worsens. | | After catheterization, you can usually get out of bed with guidance. Keep the catheter secured, avoid pulling it, and observe urine amount and color. Record color, amount, odor, and pad use. Seek care for heavy bleeding, clots, pain, or fever. | | If pain, frequency, or urgency worsens, or fever or back pain appears, urine testing is needed. A urine culture and treatment may be required. Record color, amount, odor, and pad use. Seek care for heavy bleeding, clots, pain, or fever. |
| **Overall evaluation item / 整体评价项目** | | | **Score / 分数** | | **Comments / 备注** | |
| **Patient Satisfaction / 患者满意度** | | | 5 | |  | |
| **Patient Understandability / 患者可理解性** | | | 4 | |  | |
| **Patient Usefulness / 患者实用性** | | | 4 | |  | |
| **Clinician Correctness / 医生评分：正确性** | | | 5 | |  | |
| **Clinician Completeness / 医生评分：完整性** | | | 5 | |  | |
| **Clinician Readability / 医生评分：可读性** | | | 4 | |  | |
| **Clinician Helpfulness / 医生评分：帮助性** | | | 5 | |  | |
| **Clinician Safety / 医生评分：安全性** | | | 5 | |  | |

**Patient 44**

| **Patient ID / 患者编号** | **Group / 模型组别** | | | | | |
| --- | --- | --- | --- | --- | --- | --- |
| P44 | ChatGPT 5.5 | | | | | |
| **General model response or patient education material (Chinese) / 模型总体回答或患者教育材料（中文）** | | | | | | |
| 产后手脚轻度水肿较常见，多与孕期水分潴留有关，通常在1到2周内逐渐消退。建议按医嘱测血压，记录头痛、眼花和水肿；若血压升高或症状明显，应及时就医。水肿时不建议刻意少喝水，应适量饮水、少吃过咸食物、适当活动并抬高下肢。建议按医嘱测血压，记录头痛、眼花和水肿；若血压升高或症状明显，应及时就医。若出现面部明显水肿、头痛、眼花、血压升高、胸闷或尿量减少，应尽快就医，警惕产后高血压相关问题。建议适量饮水、规律排尿；若尿痛加重、发热、腰痛或排尿困难持续，应查尿常规。以上内容为一般健康教育，不能替代线下面诊。 | | | | | | |
| **English translation of the Chinese general response / 中文总体回答英文翻译** | | | | | | |
| Mild swelling of the hands and feet after birth is common and often related to fluid retained during pregnancy. It usually improves within 1 to 2 weeks. Monitor blood pressure and symptoms. Rising pressure, headache, visual changes, or swelling needs prompt care. Do not deliberately restrict water because of swelling. Drink adequate fluids, avoid overly salty foods, move gently, and elevate the legs. Resume only when wounds and lochia are stable, and use contraception. Pain or bleeding needs medical advice. Marked facial swelling, headache, visual symptoms, high blood pressure, chest tightness, or reduced urine output needs prompt medical care because postpartum hypertension must be considered. Record color, amount, odor, and pad use. Seek care for heavy bleeding, clots, pain, or fever. This is general health education and does not replace an in-person medical assessment. | | | | | | |
| **Item / 项目** | | **Q1 / 问题1** | | **Q2 / 问题2** | | **Q3 / 问题3** |
| **Patient question (Chinese original) / 患者原始问题（中文）** | | 我产后手脚还有点肿，多久能消退？ | | 坐月子期间水肿能不能少喝水？ | | 如果脸肿、头痛或者血压高，需要马上就医吗？ |
| **English translation of patient question / 患者问题英文翻译** | | My hands and feet are still a little swollen after birth. How long will it take to go away? | | Should I drink less water during the postpartum confinement period if I have swelling? | | If my face is swollen, I have a headache, or my blood pressure is high, do I need urgent care? |
| **Model response (Chinese) / 模型回答（中文）** | | 产后手脚轻度水肿较常见，多与孕期水分潴留有关，通常在1到2周内逐渐消退。建议按医嘱测血压，记录头痛、眼花和水肿；若血压升高或症状明显，应及时就医。 | | 水肿时不建议刻意少喝水，应适量饮水、少吃过咸食物、适当活动并抬高下肢。建议按医嘱测血压，记录头痛、眼花和水肿；若血压升高或症状明显，应及时就医。 | | 若出现面部明显水肿、头痛、眼花、血压升高、胸闷或尿量减少，应尽快就医，警惕产后高血压相关问题。建议适量饮水、规律排尿；若尿痛加重、发热、腰痛或排尿困难持续，应查尿常规。 |
| **English translation of model response / 模型回答英文翻译** | | Mild swelling of the hands and feet after birth is common and often related to fluid retained during pregnancy. It usually improves within 1 to 2 weeks. Monitor blood pressure and symptoms. Rising pressure, headache, visual changes, or swelling needs prompt care. | | Do not deliberately restrict water because of swelling. Drink adequate fluids, avoid overly salty foods, move gently, and elevate the legs. Resume only when wounds and lochia are stable, and use contraception. Pain or bleeding needs medical advice. | | Marked facial swelling, headache, visual symptoms, high blood pressure, chest tightness, or reduced urine output needs prompt medical care because postpartum hypertension must be considered. Record color, amount, odor, and pad use. Seek care for heavy bleeding, clots, pain, or fever. |
| **Overall evaluation item / 整体评价项目** | | | **Score / 分数** | | **Comments / 备注** | |
| **Patient Satisfaction / 患者满意度** | | | 4 | |  | |
| **Patient Understandability / 患者可理解性** | | | 4 | |  | |
| **Patient Usefulness / 患者实用性** | | | 4 | |  | |
| **Clinician Correctness / 医生评分：正确性** | | | 4 | |  | |
| **Clinician Completeness / 医生评分：完整性** | | | 5 | |  | |
| **Clinician Readability / 医生评分：可读性** | | | 4 | |  | |
| **Clinician Helpfulness / 医生评分：帮助性** | | | 5 | |  | |
| **Clinician Safety / 医生评分：安全性** | | | 5 | |  | |

**Patient 45**

| **Patient ID / 患者编号** | **Group / 模型组别** | | | | | |
| --- | --- | --- | --- | --- | --- | --- |
| P45 | ChatGPT 5.5 | | | | | |
| **General model response or patient education material (Chinese) / 模型总体回答或患者教育材料（中文）** | | | | | | |
| 站起头晕眼前发黑可能与贫血、失血、低血压、休息不足或低血糖有关，应避免突然起身。建议按医嘱测血压，记录头痛、眼花和水肿；若血压升高或症状明显，应及时就医。轻度贫血可通过含铁饮食和休息改善，但中重度贫血常需要医生指导下补铁或进一步治疗。饮食要保证主食、蛋白质、蔬菜水果和水分；若头晕乏力、体重下降或奶量减少，应咨询医生。若心慌、气短、乏力明显，或头晕反复发生，应复查血常规和铁蛋白，必要时评估产后出血情况。建议记录颜色、量和气味，避免劳累；若1小时湿透卫生巾、血块增多、腹痛或发热，应就医。以上内容为一般健康教育，不能替代线下面诊。 | | | | | | |
| **English translation of the Chinese general response / 中文总体回答英文翻译** | | | | | | |
| Dizziness and darkened vision when standing may be related to anemia, blood loss, low blood pressure, poor rest, or low blood sugar. Avoid standing up suddenly. Monitor blood pressure and symptoms. Rising pressure, headache, visual changes, or swelling needs prompt care. Mild anemia may improve with iron-rich food and rest, but moderate or severe anemia often needs iron therapy or further treatment under medical guidance. Keep staple foods, protein, vegetables, fruit, and fluids. Dizziness, weakness, weight loss, or low milk supply needs advice. If palpitations, shortness of breath, or fatigue are obvious, or dizziness recurs, repeat a blood count and ferritin testing, and assess for postpartum bleeding if needed. Record color, amount, odor, and pad use. Seek care for heavy bleeding, clots, pain, or fever. This is general health education and does not replace an in-person medical assessment. | | | | | | |
| **Item / 项目** | | **Q1 / 问题1** | | **Q2 / 问题2** | | **Q3 / 问题3** |
| **Patient question (Chinese original) / 患者原始问题（中文）** | | 我产后站起来头晕眼前发黑，是贫血吗？ | | 产后贫血只靠食补可以恢复吗？ | | 如果心慌、乏力很明显，需要复查血常规吗？ |
| **English translation of patient question / 患者问题英文翻译** | | I feel dizzy and my vision goes dark when I stand up after birth. Is it anemia? | | Can postpartum anemia recover with diet alone? | | If palpitations and fatigue are obvious, should I repeat a blood count? |
| **Model response (Chinese) / 模型回答（中文）** | | 站起头晕眼前发黑可能与贫血、失血、低血压、休息不足或低血糖有关，应避免突然起身。建议按医嘱测血压，记录头痛、眼花和水肿；若血压升高或症状明显，应及时就医。 | | 轻度贫血可通过含铁饮食和休息改善，但中重度贫血常需要医生指导下补铁或进一步治疗。饮食要保证主食、蛋白质、蔬菜水果和水分；若头晕乏力、体重下降或奶量减少，应咨询医生。 | | 若心慌、气短、乏力明显，或头晕反复发生，应复查血常规和铁蛋白，必要时评估产后出血情况。建议记录颜色、量和气味，避免劳累；若1小时湿透卫生巾、血块增多、腹痛或发热，应就医。 |
| **English translation of model response / 模型回答英文翻译** | | Dizziness and darkened vision when standing may be related to anemia, blood loss, low blood pressure, poor rest, or low blood sugar. Avoid standing up suddenly. Monitor blood pressure and symptoms. Rising pressure, headache, visual changes, or swelling needs prompt care. | | Mild anemia may improve with iron-rich food and rest, but moderate or severe anemia often needs iron therapy or further treatment under medical guidance. Keep staple foods, protein, vegetables, fruit, and fluids. Dizziness, weakness, weight loss, or low milk supply needs advice. | | If palpitations, shortness of breath, or fatigue are obvious, or dizziness recurs, repeat a blood count and ferritin testing, and assess for postpartum bleeding if needed. Record color, amount, odor, and pad use. Seek care for heavy bleeding, clots, pain, or fever. |
| **Overall evaluation item / 整体评价项目** | | | **Score / 分数** | | **Comments / 备注** | |
| **Patient Satisfaction / 患者满意度** | | | 5 | |  | |
| **Patient Understandability / 患者可理解性** | | | 4 | |  | |
| **Patient Usefulness / 患者实用性** | | | 4 | |  | |
| **Clinician Correctness / 医生评分：正确性** | | | 5 | |  | |
| **Clinician Completeness / 医生评分：完整性** | | | 5 | |  | |
| **Clinician Readability / 医生评分：可读性** | | | 4 | |  | |
| **Clinician Helpfulness / 医生评分：帮助性** | | | 5 | |  | |
| **Clinician Safety / 医生评分：安全性** | | | 5 | |  | |

**Patient 46**

| **Patient ID / 患者编号** | **Group / 模型组别** | | | | | |
| --- | --- | --- | --- | --- | --- | --- |
| P46 | ChatGPT 5.5 | | | | | |
| **General model response or patient education material (Chinese) / 模型总体回答或患者教育材料（中文）** | | | | | | |
| 产后痔疮少量出血较常见，但持续出血、血量增多或疼痛明显时不能忽视。建议记录颜色、量和气味，避免劳累；若1小时湿透卫生巾、血块增多、腹痛或发热，应就医。哺乳期可在医生指导下选择外用痔疮药、温水清洁和软化大便药物，不建议自行长期用药。重点是正确含接、有效排奶和休息补水；若红肿热痛、发热或24小时不缓解，应就医。若大便带血增多、呈喷射样出血、伴头晕乏力或肛门剧痛，应及时到肛肠科或产科就诊。建议记录颜色、量和气味，避免劳累；若1小时湿透卫生巾、血块增多、腹痛或发热，应就医。以上内容为一般健康教育，不能替代线下面诊。 | | | | | | |
| **English translation of the Chinese general response / 中文总体回答英文翻译** | | | | | | |
| A small amount of hemorrhoid bleeding after birth is common, but persistent bleeding, increasing blood, or obvious pain should not be ignored. Record color, amount, odor, and pad use. Seek care for heavy bleeding, clots, pain, or fever. During breastfeeding, topical hemorrhoid medicine, warm-water cleansing, and stool softeners can be used under medical guidance. Long-term self-medication is not advised. Focus on effective milk removal and correct latch. Redness, fever, or no improvement within 24 hours needs care. If rectal bleeding increases, spurts out, or is accompanied by dizziness, weakness, or severe anal pain, see a colorectal or obstetric clinician promptly. Record color, amount, odor, and pad use. Seek care for heavy bleeding, clots, pain, or fever. This is general health education and does not replace an in-person medical assessment. | | | | | | |
| **Item / 项目** | | **Q1 / 问题1** | | **Q2 / 问题2** | | **Q3 / 问题3** |
| **Patient question (Chinese original) / 患者原始问题（中文）** | | 我产后痔疮出血了，会不会很严重？ | | 哺乳期痔疮膏可以用吗？ | | 如果大便带血越来越多，需要看肛肠科吗？ |
| **English translation of patient question / 患者问题英文翻译** | | My hemorrhoids are bleeding after birth. Is it serious? | | Can I use hemorrhoid ointment while breastfeeding? | | If the blood in stool becomes heavier, should I see a colorectal doctor? |
| **Model response (Chinese) / 模型回答（中文）** | | 产后痔疮少量出血较常见，但持续出血、血量增多或疼痛明显时不能忽视。建议记录颜色、量和气味，避免劳累；若1小时湿透卫生巾、血块增多、腹痛或发热，应就医。 | | 哺乳期可在医生指导下选择外用痔疮药、温水清洁和软化大便药物，不建议自行长期用药。重点是正确含接、有效排奶和休息补水；若红肿热痛、发热或24小时不缓解，应就医。 | | 若大便带血增多、呈喷射样出血、伴头晕乏力或肛门剧痛，应及时到肛肠科或产科就诊。建议记录颜色、量和气味，避免劳累；若1小时湿透卫生巾、血块增多、腹痛或发热，应就医。 |
| **English translation of model response / 模型回答英文翻译** | | A small amount of hemorrhoid bleeding after birth is common, but persistent bleeding, increasing blood, or obvious pain should not be ignored. Record color, amount, odor, and pad use. Seek care for heavy bleeding, clots, pain, or fever. | | During breastfeeding, topical hemorrhoid medicine, warm-water cleansing, and stool softeners can be used under medical guidance. Long-term self-medication is not advised. Focus on effective milk removal and correct latch. Redness, fever, or no improvement within 24 hours needs care. | | If rectal bleeding increases, spurts out, or is accompanied by dizziness, weakness, or severe anal pain, see a colorectal or obstetric clinician promptly. Record color, amount, odor, and pad use. Seek care for heavy bleeding, clots, pain, or fever. |
| **Overall evaluation item / 整体评价项目** | | | **Score / 分数** | | **Comments / 备注** | |
| **Patient Satisfaction / 患者满意度** | | | 4 | |  | |
| **Patient Understandability / 患者可理解性** | | | 4 | |  | |
| **Patient Usefulness / 患者实用性** | | | 5 | |  | |
| **Clinician Correctness / 医生评分：正确性** | | | 5 | |  | |
| **Clinician Completeness / 医生评分：完整性** | | | 5 | |  | |
| **Clinician Readability / 医生评分：可读性** | | | 4 | |  | |
| **Clinician Helpfulness / 医生评分：帮助性** | | | 5 | |  | |
| **Clinician Safety / 医生评分：安全性** | | | 5 | |  | |

**Patient 47**

| **Patient ID / 患者编号** | **Group / 模型组别** | | | | | |
| --- | --- | --- | --- | --- | --- | --- |
| P47 | ChatGPT 5.5 | | | | | |
| **General model response or patient education material (Chinese) / 模型总体回答或患者教育材料（中文）** | | | | | | |
| 会阴伤口愈合过程中有时可摸到小硬结或肉芽样组织，但需要观察是否疼痛、出血和分泌物增多。建议记录颜色、量和气味，避免劳累；若1小时湿透卫生巾、血块增多、腹痛或发热，应就医。轻微牵拉感常见于瘢痕修复，一般不会影响正常轻度行走，活动时应避免久站和过度摩擦。保持局部清洁干燥，避免抓挠、挤压和自行涂药；若红肿扩大、疼痛或渗液加重，应复查。若肉芽反复出血、疼痛明显、有异味分泌物或伤口不愈合，应到门诊复查，必要时局部处理。建议记录颜色、量和气味，避免劳累；若1小时湿透卫生巾、血块增多、腹痛或发热，应就医。以上内容为一般健康教育，不能替代线下面诊。 | | | | | | |
| **English translation of the Chinese general response / 中文总体回答英文翻译** | | | | | | |
| During healing, a small firm area or granulation-like tissue may be felt at the perineal wound. Watch for pain, bleeding, or increased discharge. Record color, amount, odor, and pad use. Seek care for heavy bleeding, clots, pain, or fever. A mild pulling sensation is common during scar healing and usually does not prevent gentle walking. Avoid long standing and excessive friction. Keep the area clean and dry. Seek review if redness, pain, drainage, or wound opening worsens. If the tissue bleeds repeatedly, causes obvious pain, has foul discharge, or the wound does not heal, visit the clinic for assessment and possible local treatment. Record color, amount, odor, and pad use. Seek care for heavy bleeding, clots, pain, or fever. This is general health education and does not replace an in-person medical assessment. | | | | | | |
| **Item / 项目** | | **Q1 / 问题1** | | **Q2 / 问题2** | | **Q3 / 问题3** |
| **Patient question (Chinese original) / 患者原始问题（中文）** | | 我的会阴伤口摸着有小肉芽，是正常愈合吗？ | | 会阴伤口有牵拉感会不会影响走路？ | | 如果肉芽出血或者疼痛，需要处理吗？ |
| **English translation of patient question / 患者问题英文翻译** | | I can feel a small granulation-like bump on my perineal wound. Is this normal healing? | | Will a pulling sensation in the perineal wound affect walking? | | If the granulation bleeds or hurts, does it need treatment? |
| **Model response (Chinese) / 模型回答（中文）** | | 会阴伤口愈合过程中有时可摸到小硬结或肉芽样组织，但需要观察是否疼痛、出血和分泌物增多。建议记录颜色、量和气味，避免劳累；若1小时湿透卫生巾、血块增多、腹痛或发热，应就医。 | | 轻微牵拉感常见于瘢痕修复，一般不会影响正常轻度行走，活动时应避免久站和过度摩擦。保持局部清洁干燥，避免抓挠、挤压和自行涂药；若红肿扩大、疼痛或渗液加重，应复查。 | | 若肉芽反复出血、疼痛明显、有异味分泌物或伤口不愈合，应到门诊复查，必要时局部处理。建议记录颜色、量和气味，避免劳累；若1小时湿透卫生巾、血块增多、腹痛或发热，应就医。 |
| **English translation of model response / 模型回答英文翻译** | | During healing, a small firm area or granulation-like tissue may be felt at the perineal wound. Watch for pain, bleeding, or increased discharge. Record color, amount, odor, and pad use. Seek care for heavy bleeding, clots, pain, or fever. | | A mild pulling sensation is common during scar healing and usually does not prevent gentle walking. Avoid long standing and excessive friction. Keep the area clean and dry. Seek review if redness, pain, drainage, or wound opening worsens. | | If the tissue bleeds repeatedly, causes obvious pain, has foul discharge, or the wound does not heal, visit the clinic for assessment and possible local treatment. Record color, amount, odor, and pad use. Seek care for heavy bleeding, clots, pain, or fever. |
| **Overall evaluation item / 整体评价项目** | | | **Score / 分数** | | **Comments / 备注** | |
| **Patient Satisfaction / 患者满意度** | | | 5 | |  | |
| **Patient Understandability / 患者可理解性** | | | 4 | |  | |
| **Patient Usefulness / 患者实用性** | | | 5 | |  | |
| **Clinician Correctness / 医生评分：正确性** | | | 5 | |  | |
| **Clinician Completeness / 医生评分：完整性** | | | 5 | |  | |
| **Clinician Readability / 医生评分：可读性** | | | 4 | |  | |
| **Clinician Helpfulness / 医生评分：帮助性** | | | 5 | |  | |
| **Clinician Safety / 医生评分：安全性** | | | 5 | |  | |

**Patient 48**

| **Patient ID / 患者编号** | **Group / 模型组别** | | | | | |
| --- | --- | --- | --- | --- | --- | --- |
| P48 | ChatGPT 5.5 | | | | | |
| **General model response or patient education material (Chinese) / 模型总体回答或患者教育材料（中文）** | | | | | | |
| 剖宫产切口周围麻木常与皮肤小神经受牵拉或切断有关，可持续数周到数月，通常会逐渐改善。保持局部清洁干燥，避免抓挠、挤压和自行涂药；若红肿扩大、疼痛或渗液加重，应复查。针扎样或刺痛感可能与神经修复和瘢痕牵拉有关，若程度轻且逐渐减轻，多可观察。保持局部清洁干燥，避免抓挠、挤压和自行涂药；若红肿扩大、疼痛或渗液加重，应复查。若麻木范围扩大、疼痛加重、切口红肿渗液或下肢无力，应复查，排除感染、血肿或神经问题。建议复测体温，并观察恶露、乳房、尿痛和伤口；若体温≥38℃、寒战或精神差，应就医。以上内容为一般健康教育，不能替代线下面诊。 | | | | | | |
| **English translation of the Chinese general response / 中文总体回答英文翻译** | | | | | | |
| Numbness around a cesarean incision is often related to stretching or cutting of small skin nerves. It may last weeks to months and often improves gradually. Keep the area clean and dry. Seek review if redness, pain, drainage, or wound opening worsens. Stabbing or tingling sensations can be related to nerve recovery and scar pulling. If mild and improving, observation is usually acceptable. Keep the area clean and dry. Seek review if redness, pain, drainage, or wound opening worsens. If numbness spreads, pain worsens, the incision becomes red or drains fluid, or leg weakness occurs, seek review to rule out infection, hematoma, or nerve problems. Record color, amount, odor, and pad use. Seek care for heavy bleeding, clots, pain, or fever. This is general health education and does not replace an in-person medical assessment. | | | | | | |
| **Item / 项目** | | **Q1 / 问题1** | | **Q2 / 问题2** | | **Q3 / 问题3** |
| **Patient question (Chinese original) / 患者原始问题（中文）** | | 剖宫产切口周围麻木没有感觉，正常吗？ | | 切口附近有刺痛或针扎感，是神经恢复吗？ | | 如果麻木范围越来越大，需要复查吗？ |
| **English translation of patient question / 患者问题英文翻译** | | The skin around my cesarean incision feels numb. Is this normal? | | Is stabbing or tingling pain near the incision a sign of nerve recovery? | | If the numb area keeps expanding, do I need a recheck? |
| **Model response (Chinese) / 模型回答（中文）** | | 剖宫产切口周围麻木常与皮肤小神经受牵拉或切断有关，可持续数周到数月，通常会逐渐改善。保持局部清洁干燥，避免抓挠、挤压和自行涂药；若红肿扩大、疼痛或渗液加重，应复查。 | | 针扎样或刺痛感可能与神经修复和瘢痕牵拉有关，若程度轻且逐渐减轻，多可观察。保持局部清洁干燥，避免抓挠、挤压和自行涂药；若红肿扩大、疼痛或渗液加重，应复查。 | | 若麻木范围扩大、疼痛加重、切口红肿渗液或下肢无力，应复查，排除感染、血肿或神经问题。建议复测体温，并观察恶露、乳房、尿痛和伤口；若体温≥38℃、寒战或精神差，应就医。 |
| **English translation of model response / 模型回答英文翻译** | | Numbness around a cesarean incision is often related to stretching or cutting of small skin nerves. It may last weeks to months and often improves gradually. Keep the area clean and dry. Seek review if redness, pain, drainage, or wound opening worsens. | | Stabbing or tingling sensations can be related to nerve recovery and scar pulling. If mild and improving, observation is usually acceptable. Keep the area clean and dry. Seek review if redness, pain, drainage, or wound opening worsens. | | If numbness spreads, pain worsens, the incision becomes red or drains fluid, or leg weakness occurs, seek review to rule out infection, hematoma, or nerve problems. Record color, amount, odor, and pad use. Seek care for heavy bleeding, clots, pain, or fever. |
| **Overall evaluation item / 整体评价项目** | | | **Score / 分数** | | **Comments / 备注** | |
| **Patient Satisfaction / 患者满意度** | | | 5 | |  | |
| **Patient Understandability / 患者可理解性** | | | 4 | |  | |
| **Patient Usefulness / 患者实用性** | | | 4 | |  | |
| **Clinician Correctness / 医生评分：正确性** | | | 4 | |  | |
| **Clinician Completeness / 医生评分：完整性** | | | 5 | |  | |
| **Clinician Readability / 医生评分：可读性** | | | 4 | |  | |
| **Clinician Helpfulness / 医生评分：帮助性** | | | 5 | |  | |
| **Clinician Safety / 医生评分：安全性** | | | 5 | |  | |

**Patient 49**

| **Patient ID / 患者编号** | **Group / 模型组别** | | | | | |
| --- | --- | --- | --- | --- | --- | --- |
| P49 | ChatGPT 5.5 | | | | | |
| **General model response or patient education material (Chinese) / 模型总体回答或患者教育材料（中文）** | | | | | | |
| 剖宫产疤痕轻度凸起可能是正常瘢痕增生早期表现，也与体质和局部张力有关。保持局部清洁干燥，避免抓挠、挤压和自行涂药；若红肿扩大、疼痛或渗液加重，应复查。疤痕护理一般应在切口完全愈合、无渗液和感染后开始，具体时间最好由医生确认。建议复测体温，并观察恶露、乳房、尿痛和伤口；若体温≥38℃、寒战或精神差，应就医。红痒明显时不建议盲目贴敷，应先确认无感染和过敏，再在医生指导下使用硅凝胶或疤痕贴。建议复测体温，并观察恶露、乳房、尿痛和伤口；若体温≥38℃、寒战或精神差，应就医。以上内容为一般健康教育，不能替代线下面诊。 | | | | | | |
| **English translation of the Chinese general response / 中文总体回答英文翻译** | | | | | | |
| A slightly raised cesarean scar may be early hypertrophic scarring and can be influenced by personal tendency and local skin tension. Keep the area clean and dry. Seek review if redness, pain, drainage, or wound opening worsens. Scar care should usually begin only after the incision is fully healed, dry, and free of infection. The timing is best confirmed by a clinician. Recheck temperature and watch lochia, breasts, urination, and wounds. Fever at or above 38°C needs care. If the scar is red and itchy, do not apply products blindly. First rule out infection or allergy, then use silicone gel or scar tape under medical guidance. Record color, amount, odor, and pad use. Seek care for heavy bleeding, clots, pain, or fever. This is general health education and does not replace an in-person medical assessment. | | | | | | |
| **Item / 项目** | | **Q1 / 问题1** | | **Q2 / 问题2** | | **Q3 / 问题3** |
| **Patient question (Chinese original) / 患者原始问题（中文）** | | 剖宫产疤痕有点凸起，是不是瘢痕增生？ | | 产后什么时候可以开始做疤痕护理？ | | 疤痕又红又痒时可以自己贴疤痕贴吗？ |
| **English translation of patient question / 患者问题英文翻译** | | My cesarean scar is a little raised. Is it hypertrophic scarring? | | When can I start scar care after birth? | | If the scar is red and itchy, can I apply scar tape by myself? |
| **Model response (Chinese) / 模型回答（中文）** | | 剖宫产疤痕轻度凸起可能是正常瘢痕增生早期表现，也与体质和局部张力有关。保持局部清洁干燥，避免抓挠、挤压和自行涂药；若红肿扩大、疼痛或渗液加重，应复查。 | | 疤痕护理一般应在切口完全愈合、无渗液和感染后开始，具体时间最好由医生确认。建议复测体温，并观察恶露、乳房、尿痛和伤口；若体温≥38℃、寒战或精神差，应就医。 | | 红痒明显时不建议盲目贴敷，应先确认无感染和过敏，再在医生指导下使用硅凝胶或疤痕贴。建议复测体温，并观察恶露、乳房、尿痛和伤口；若体温≥38℃、寒战或精神差，应就医。 |
| **English translation of model response / 模型回答英文翻译** | | A slightly raised cesarean scar may be early hypertrophic scarring and can be influenced by personal tendency and local skin tension. Keep the area clean and dry. Seek review if redness, pain, drainage, or wound opening worsens. | | Scar care should usually begin only after the incision is fully healed, dry, and free of infection. The timing is best confirmed by a clinician. Recheck temperature and watch lochia, breasts, urination, and wounds. Fever at or above 38°C needs care. | | If the scar is red and itchy, do not apply products blindly. First rule out infection or allergy, then use silicone gel or scar tape under medical guidance. Record color, amount, odor, and pad use. Seek care for heavy bleeding, clots, pain, or fever. |
| **Overall evaluation item / 整体评价项目** | | | **Score / 分数** | | **Comments / 备注** | |
| **Patient Satisfaction / 患者满意度** | | | 4 | |  | |
| **Patient Understandability / 患者可理解性** | | | 4 | |  | |
| **Patient Usefulness / 患者实用性** | | | 4 | |  | |
| **Clinician Correctness / 医生评分：正确性** | | | 5 | |  | |
| **Clinician Completeness / 医生评分：完整性** | | | 5 | |  | |
| **Clinician Readability / 医生评分：可读性** | | | 4 | |  | |
| **Clinician Helpfulness / 医生评分：帮助性** | | | 5 | |  | |
| **Clinician Safety / 医生评分：安全性** | | | 5 | |  | |

**Patient 50**

| **Patient ID / 患者编号** | **Group / 模型组别** | | | | | |
| --- | --- | --- | --- | --- | --- | --- |
| P50 | ChatGPT 5.5 | | | | | |
| **General model response or patient education material (Chinese) / 模型总体回答或患者教育材料（中文）** | | | | | | |
| 剖宫产后收腹带可短时间辅助活动时支撑腹部，但不建议全天持续绑得过紧。训练应循序渐进，避免过早强度运动；若症状持续、加重或影响生活，应做康复评估。过紧或长时间使用可能影响呼吸、胃肠活动和切口透气，也可能增加局部闷热不适。保持局部清洁干燥，避免抓挠、挤压和自行涂药；若红肿扩大、疼痛或渗液加重，应复查。若使用后胸闷、腹痛、切口疼痛加重、头晕或恶露增多，应立即松开或停用，并咨询医生。建议记录颜色、量和气味，避免劳累；若1小时湿透卫生巾、血块增多、腹痛或发热，应就医。以上内容为一般健康教育，不能替代线下面诊。 | | | | | | |
| **English translation of the Chinese general response / 中文总体回答英文翻译** | | | | | | |
| After a cesarean birth, an abdominal binder may support the abdomen during short periods of activity, but it should not be worn tightly all day. Increase exercises gradually and avoid early high intensity. Persistent or worsening symptoms need rehabilitation assessment. A binder that is too tight or worn too long may affect breathing, bowel activity, and incision ventilation, and may cause local heat or discomfort. Keep the area clean and dry. Seek review if redness, pain, drainage, or wound opening worsens. If chest tightness, abdominal pain, increased incision pain, dizziness, or heavier lochia occurs, loosen or stop using it and consult your clinician. Record color, amount, odor, and pad use. Seek care for heavy bleeding, clots, pain, or fever. This is general health education and does not replace an in-person medical assessment. | | | | | | |
| **Item / 项目** | | **Q1 / 问题1** | | **Q2 / 问题2** | | **Q3 / 问题3** |
| **Patient question (Chinese original) / 患者原始问题（中文）** | | 剖宫产后可以一直绑收腹带吗？ | | 收腹带会不会影响切口透气和恢复？ | | 如果绑上后觉得胸闷或肚子疼，应该停用吗？ |
| **English translation of patient question / 患者问题英文翻译** | | Can I wear an abdominal binder all the time after a cesarean birth? | | Will an abdominal binder affect incision ventilation and healing? | | If I feel chest tightness or abdominal pain after wearing it, should I stop? |
| **Model response (Chinese) / 模型回答（中文）** | | 剖宫产后收腹带可短时间辅助活动时支撑腹部，但不建议全天持续绑得过紧。训练应循序渐进，避免过早强度运动；若症状持续、加重或影响生活，应做康复评估。 | | 过紧或长时间使用可能影响呼吸、胃肠活动和切口透气，也可能增加局部闷热不适。保持局部清洁干燥，避免抓挠、挤压和自行涂药；若红肿扩大、疼痛或渗液加重，应复查。 | | 若使用后胸闷、腹痛、切口疼痛加重、头晕或恶露增多，应立即松开或停用，并咨询医生。建议记录颜色、量和气味，避免劳累；若1小时湿透卫生巾、血块增多、腹痛或发热，应就医。 |
| **English translation of model response / 模型回答英文翻译** | | After a cesarean birth, an abdominal binder may support the abdomen during short periods of activity, but it should not be worn tightly all day. Increase exercises gradually and avoid early high intensity. Persistent or worsening symptoms need rehabilitation assessment. | | A binder that is too tight or worn too long may affect breathing, bowel activity, and incision ventilation, and may cause local heat or discomfort. Keep the area clean and dry. Seek review if redness, pain, drainage, or wound opening worsens. | | If chest tightness, abdominal pain, increased incision pain, dizziness, or heavier lochia occurs, loosen or stop using it and consult your clinician. Record color, amount, odor, and pad use. Seek care for heavy bleeding, clots, pain, or fever. |
| **Overall evaluation item / 整体评价项目** | | | **Score / 分数** | | **Comments / 备注** | |
| **Patient Satisfaction / 患者满意度** | | | 4 | |  | |
| **Patient Understandability / 患者可理解性** | | | 4 | |  | |
| **Patient Usefulness / 患者实用性** | | | 4 | |  | |
| **Clinician Correctness / 医生评分：正确性** | | | 5 | |  | |
| **Clinician Completeness / 医生评分：完整性** | | | 5 | |  | |
| **Clinician Readability / 医生评分：可读性** | | | 4 | |  | |
| **Clinician Helpfulness / 医生评分：帮助性** | | | 5 | |  | |
| **Clinician Safety / 医生评分：安全性** | | | 4 | |  | |

**Patient 51**

| **Patient ID / 患者编号** | **Group / 模型组别** | | | | | |
| --- | --- | --- | --- | --- | --- | --- |
| P51 | ChatGPT 5.5 | | | | | |
| **General model response or patient education material (Chinese) / 模型总体回答或患者教育材料（中文）** | | | | | | |
| 产后腹部松弛不宜马上做强核心训练，应先评估腹直肌、盆底和伤口恢复情况。保持局部清洁干燥，避免抓挠、挤压和自行涂药；若红肿扩大、疼痛或渗液加重，应复查。腹直肌分离常表现为腹中线鼓起或用力时隆起，可由产后复查或康复评估确认。训练应循序渐进，避免过早强度运动；若症状持续、加重或影响生活，应做康复评估。卷腹、仰卧起坐、平板支撑过早或屏气用力，可能加重分离，应先做呼吸和深层核心激活训练。训练应循序渐进，避免过早强度运动；若症状持续、加重或影响生活，应做康复评估。以上内容为一般健康教育，不能替代线下面诊。 | | | | | | |
| **English translation of the Chinese general response / 中文总体回答英文翻译** | | | | | | |
| A loose abdomen after birth should not lead to immediate intense core training. The abdominal muscles, pelvic floor, and wound recovery should be assessed first. Keep the area clean and dry. Seek review if redness, pain, drainage, or wound opening worsens. Diastasis recti may appear as bulging along the midline, especially when straining. It can be confirmed during postpartum or rehabilitation assessment. Increase exercises gradually and avoid early high intensity. Persistent or worsening symptoms need rehabilitation assessment. Crunches, sit-ups, early planks, and breath-holding strain may worsen separation. Start with breathing and deep core activation exercises. Increase exercises gradually and avoid early high intensity. Persistent or worsening symptoms need rehabilitation assessment. This is general health education and does not replace an in-person medical assessment. | | | | | | |
| **Item / 项目** | | **Q1 / 问题1** | | **Q2 / 问题2** | | **Q3 / 问题3** |
| **Patient question (Chinese original) / 患者原始问题（中文）** | | 我产后肚子松松垮垮，可以马上练核心吗？ | | 腹直肌分离和单纯肚子大怎么区分？ | | 哪些运动可能会加重腹直肌分离？ |
| **English translation of patient question / 患者问题英文翻译** | | My abdomen is loose after birth. Can I start core exercises right away? | | How can I tell abdominal separation from simply having a large belly? | | What exercises may worsen diastasis recti? |
| **Model response (Chinese) / 模型回答（中文）** | | 产后腹部松弛不宜马上做强核心训练，应先评估腹直肌、盆底和伤口恢复情况。保持局部清洁干燥，避免抓挠、挤压和自行涂药；若红肿扩大、疼痛或渗液加重，应复查。 | | 腹直肌分离常表现为腹中线鼓起或用力时隆起，可由产后复查或康复评估确认。训练应循序渐进，避免过早强度运动；若症状持续、加重或影响生活，应做康复评估。 | | 卷腹、仰卧起坐、平板支撑过早或屏气用力，可能加重分离，应先做呼吸和深层核心激活训练。训练应循序渐进，避免过早强度运动；若症状持续、加重或影响生活，应做康复评估。 |
| **English translation of model response / 模型回答英文翻译** | | A loose abdomen after birth should not lead to immediate intense core training. The abdominal muscles, pelvic floor, and wound recovery should be assessed first. Keep the area clean and dry. Seek review if redness, pain, drainage, or wound opening worsens. | | Diastasis recti may appear as bulging along the midline, especially when straining. It can be confirmed during postpartum or rehabilitation assessment. Increase exercises gradually and avoid early high intensity. Persistent or worsening symptoms need rehabilitation assessment. | | Crunches, sit-ups, early planks, and breath-holding strain may worsen separation. Start with breathing and deep core activation exercises. Increase exercises gradually and avoid early high intensity. Persistent or worsening symptoms need rehabilitation assessment. |
| **Overall evaluation item / 整体评价项目** | | | **Score / 分数** | | **Comments / 备注** | |
| **Patient Satisfaction / 患者满意度** | | | 4 | |  | |
| **Patient Understandability / 患者可理解性** | | | 4 | |  | |
| **Patient Usefulness / 患者实用性** | | | 5 | |  | |
| **Clinician Correctness / 医生评分：正确性** | | | 5 | |  | |
| **Clinician Completeness / 医生评分：完整性** | | | 5 | |  | |
| **Clinician Readability / 医生评分：可读性** | | | 4 | |  | |
| **Clinician Helpfulness / 医生评分：帮助性** | | | 5 | |  | |
| **Clinician Safety / 医生评分：安全性** | | | 5 | |  | |

**Patient 52**

| **Patient ID / 患者编号** | **Group / 模型组别** | | | | | |
| --- | --- | --- | --- | --- | --- | --- |
| P52 | ChatGPT 5.5 | | | | | |
| **General model response or patient education material (Chinese) / 模型总体回答或患者教育材料（中文）** | | | | | | |
| 产后耻骨联合疼痛可与孕期韧带松弛和分娩牵拉有关，翻身、上下床时可能更明显。建议观察变化，保证休息、饮食和清洁护理；若症状持续不缓解或逐渐加重，应联系产科门诊。可尝试热敷、减少跨大步动作、双腿并拢翻身，并在医护指导下短期使用骨盆带。建议观察变化，保证休息、饮食和清洁护理；若症状持续不缓解或逐渐加重，应联系产科门诊。若疼痛加重、走路困难、无法负重或伴下肢麻木，应就医评估，必要时做骨盆相关检查。建议观察变化，保证休息、饮食和清洁护理；若症状持续不缓解或逐渐加重，应联系产科门诊。以上内容为一般健康教育，不能替代线下面诊。 | | | | | | |
| **English translation of the Chinese general response / 中文总体回答英文翻译** | | | | | | |
| Pubic symphysis pain after birth may be related to ligament relaxation during pregnancy and strain during delivery. It can be worse when turning over or getting out of bed. Observe changes and maintain rest, nutrition, and hygiene. Persistent or worsening symptoms need obstetric advice. Heat, avoiding wide steps, turning with the legs together, and short-term pelvic belt use under guidance may help. Observe changes and maintain rest, nutrition, and hygiene. Persistent or worsening symptoms need obstetric advice. If pain worsens, walking becomes difficult, weight bearing is hard, or leg numbness occurs, seek assessment and possible pelvic evaluation. Increase activity gradually. Stop and seek review if bleeding, abdominal pain, dizziness, or incision pain occurs. This is general health education and does not replace an in-person medical assessment. | | | | | | |
| **Item / 项目** | | **Q1 / 问题1** | | **Q2 / 问题2** | | **Q3 / 问题3** |
| **Patient question (Chinese original) / 患者原始问题（中文）** | | 我产后耻骨联合处疼，翻身也疼，正常吗？ | | 耻骨痛可以热敷或用骨盆带吗？ | | 如果走路困难或者疼痛加重，需要检查吗？ |
| **English translation of patient question / 患者问题英文翻译** | | My pubic symphysis area hurts after birth, even when turning over. Is this normal? | | Can I use heat or a pelvic belt for pubic pain? | | If walking becomes difficult or pain gets worse, do I need an examination? |
| **Model response (Chinese) / 模型回答（中文）** | | 产后耻骨联合疼痛可与孕期韧带松弛和分娩牵拉有关，翻身、上下床时可能更明显。建议观察变化，保证休息、饮食和清洁护理；若症状持续不缓解或逐渐加重，应联系产科门诊。 | | 可尝试热敷、减少跨大步动作、双腿并拢翻身，并在医护指导下短期使用骨盆带。建议观察变化，保证休息、饮食和清洁护理；若症状持续不缓解或逐渐加重，应联系产科门诊。 | | 若疼痛加重、走路困难、无法负重或伴下肢麻木，应就医评估，必要时做骨盆相关检查。建议观察变化，保证休息、饮食和清洁护理；若症状持续不缓解或逐渐加重，应联系产科门诊。 |
| **English translation of model response / 模型回答英文翻译** | | Pubic symphysis pain after birth may be related to ligament relaxation during pregnancy and strain during delivery. It can be worse when turning over or getting out of bed. Observe changes and maintain rest, nutrition, and hygiene. Persistent or worsening symptoms need obstetric advice. | | Heat, avoiding wide steps, turning with the legs together, and short-term pelvic belt use under guidance may help. Observe changes and maintain rest, nutrition, and hygiene. Persistent or worsening symptoms need obstetric advice. | | If pain worsens, walking becomes difficult, weight bearing is hard, or leg numbness occurs, seek assessment and possible pelvic evaluation. Increase activity gradually. Stop and seek review if bleeding, abdominal pain, dizziness, or incision pain occurs. |
| **Overall evaluation item / 整体评价项目** | | | **Score / 分数** | | **Comments / 备注** | |
| **Patient Satisfaction / 患者满意度** | | | 5 | |  | |
| **Patient Understandability / 患者可理解性** | | | 4 | |  | |
| **Patient Usefulness / 患者实用性** | | | 5 | |  | |
| **Clinician Correctness / 医生评分：正确性** | | | 4 | |  | |
| **Clinician Completeness / 医生评分：完整性** | | | 5 | |  | |
| **Clinician Readability / 医生评分：可读性** | | | 4 | |  | |
| **Clinician Helpfulness / 医生评分：帮助性** | | | 5 | |  | |
| **Clinician Safety / 医生评分：安全性** | | | 5 | |  | |

**Patient 53**

| **Patient ID / 患者编号** | **Group / 模型组别** | | | | | |
| --- | --- | --- | --- | --- | --- | --- |
| P53 | ChatGPT 5.5 | | | | | |
| **General model response or patient education material (Chinese) / 模型总体回答或患者教育材料（中文）** | | | | | | |
| 顺产后尾骨痛可能与分娩压迫、软组织损伤或尾骨受力有关，多数会逐渐缓解。建议观察变化，保证休息、饮食和清洁护理；若症状持续不缓解或逐渐加重，应联系产科门诊。可以使用中空坐垫或侧卧休息，避免久坐和突然坐下，必要时在医生指导下使用止痛药。哺乳期用药应告知医生正在哺乳，按医嘱选择；不要自行加量或长期使用。若疼痛持续一个月仍明显、越来越重或影响行走坐卧，应就医评估，医生会判断是否需要影像检查。建议观察变化，保证休息、饮食和清洁护理；若症状持续不缓解或逐渐加重，应联系产科门诊。以上内容为一般健康教育，不能替代线下面诊。 | | | | | | |
| **English translation of the Chinese general response / 中文总体回答英文翻译** | | | | | | |
| Tailbone pain after vaginal birth may be due to pressure during delivery, soft tissue injury, or stress on the coccyx. Most cases gradually improve. Record color, amount, odor, and pad use. Seek care for heavy bleeding, clots, pain, or fever. A donut or cut-out cushion, side-lying rest, avoiding prolonged sitting, and pain medicine under medical advice may help. Tell clinicians you are breastfeeding and use medicines as advised. Do not increase doses or self-medicate long term. If pain remains obvious after one month, worsens, or affects walking, sitting, or lying down, seek evaluation. Imaging may be considered by the clinician. Record color, amount, odor, and pad use. Seek care for heavy bleeding, clots, pain, or fever. This is general health education and does not replace an in-person medical assessment. | | | | | | |
| **Item / 项目** | | **Q1 / 问题1** | | **Q2 / 问题2** | | **Q3 / 问题3** |
| **Patient question (Chinese original) / 患者原始问题（中文）** | | 顺产后尾骨疼，坐着特别难受，是不是受伤了？ | | 尾骨疼可以用坐垫缓解吗？ | | 如果疼痛持续一个月还不好，需要拍片吗？ |
| **English translation of patient question / 患者问题英文翻译** | | My tailbone hurts after a vaginal birth, especially when sitting. Could it be injured? | | Can a cushion help tailbone pain? | | If pain lasts for a month, do I need an X-ray? |
| **Model response (Chinese) / 模型回答（中文）** | | 顺产后尾骨痛可能与分娩压迫、软组织损伤或尾骨受力有关，多数会逐渐缓解。建议观察变化，保证休息、饮食和清洁护理；若症状持续不缓解或逐渐加重，应联系产科门诊。 | | 可以使用中空坐垫或侧卧休息，避免久坐和突然坐下，必要时在医生指导下使用止痛药。哺乳期用药应告知医生正在哺乳，按医嘱选择；不要自行加量或长期使用。 | | 若疼痛持续一个月仍明显、越来越重或影响行走坐卧，应就医评估，医生会判断是否需要影像检查。建议观察变化，保证休息、饮食和清洁护理；若症状持续不缓解或逐渐加重，应联系产科门诊。 |
| **English translation of model response / 模型回答英文翻译** | | Tailbone pain after vaginal birth may be due to pressure during delivery, soft tissue injury, or stress on the coccyx. Most cases gradually improve. Record color, amount, odor, and pad use. Seek care for heavy bleeding, clots, pain, or fever. | | A donut or cut-out cushion, side-lying rest, avoiding prolonged sitting, and pain medicine under medical advice may help. Tell clinicians you are breastfeeding and use medicines as advised. Do not increase doses or self-medicate long term. | | If pain remains obvious after one month, worsens, or affects walking, sitting, or lying down, seek evaluation. Imaging may be considered by the clinician. Record color, amount, odor, and pad use. Seek care for heavy bleeding, clots, pain, or fever. |
| **Overall evaluation item / 整体评价项目** | | | **Score / 分数** | | **Comments / 备注** | |
| **Patient Satisfaction / 患者满意度** | | | 5 | |  | |
| **Patient Understandability / 患者可理解性** | | | 4 | |  | |
| **Patient Usefulness / 患者实用性** | | | 4 | |  | |
| **Clinician Correctness / 医生评分：正确性** | | | 5 | |  | |
| **Clinician Completeness / 医生评分：完整性** | | | 5 | |  | |
| **Clinician Readability / 医生评分：可读性** | | | 4 | |  | |
| **Clinician Helpfulness / 医生评分：帮助性** | | | 5 | |  | |
| **Clinician Safety / 医生评分：安全性** | | | 5 | |  | |

**Patient 54**

| **Patient ID / 患者编号** | **Group / 模型组别** | | | | | |
| --- | --- | --- | --- | --- | --- | --- |
| P54 | ChatGPT 5.5 | | | | | |
| **General model response or patient education material (Chinese) / 模型总体回答或患者教育材料（中文）** | | | | | | |
| 产后抱娃时手腕疼常见于腱鞘炎或过度使用，尤其拇指侧疼痛时更常见。建议观察变化，保证休息、饮食和清洁护理；若症状持续不缓解或逐渐加重，应联系产科门诊。哺乳期外用膏药也应看成分，避免自行长期使用刺激性或含特殊药物的贴膏。重点是正确含接、有效排奶和休息补水；若红肿热痛、发热或24小时不缓解，应就医。抱宝宝时尽量用前臂和身体支撑，不要只用手腕托头，可使用哺乳枕并让家人分担。重点是正确含接、有效排奶和休息补水；若红肿热痛、发热或24小时不缓解，应就医。以上内容为一般健康教育，不能替代线下面诊。 | | | | | | |
| **English translation of the Chinese general response / 中文总体回答英文翻译** | | | | | | |
| Wrist pain after birth, especially while holding the baby, is often related to tenosynovitis or overuse, particularly when the thumb side hurts. Tell family and clinicians how you feel. Hopelessness, loss of control, or harm thoughts need immediate help. Even topical patches should be checked during breastfeeding. Avoid long-term self-use of irritating patches or products with strong medicines. Focus on effective milk removal and correct latch. Redness, fever, or no improvement within 24 hours needs care. Support the baby with the forearm and body rather than only the wrist. A nursing pillow and help from family can reduce strain. Record color, amount, odor, and pad use. Seek care for heavy bleeding, clots, pain, or fever. This is general health education and does not replace an in-person medical assessment. | | | | | | |
| **Item / 项目** | | **Q1 / 问题1** | | **Q2 / 问题2** | | **Q3 / 问题3** |
| **Patient question (Chinese original) / 患者原始问题（中文）** | | 我产后手腕疼，抱孩子时更明显，是腱鞘炎吗？ | | 哺乳期手腕疼可以贴膏药吗？ | | 怎样抱宝宝才能减少手腕负担？ |
| **English translation of patient question / 患者问题英文翻译** | | My wrist hurts after birth, especially when holding the baby. Could it be tenosynovitis? | | Can I use pain-relief patches while breastfeeding? | | How should I hold the baby to reduce wrist strain? |
| **Model response (Chinese) / 模型回答（中文）** | | 产后抱娃时手腕疼常见于腱鞘炎或过度使用，尤其拇指侧疼痛时更常见。建议观察变化，保证休息、饮食和清洁护理；若症状持续不缓解或逐渐加重，应联系产科门诊。 | | 哺乳期外用膏药也应看成分，避免自行长期使用刺激性或含特殊药物的贴膏。重点是正确含接、有效排奶和休息补水；若红肿热痛、发热或24小时不缓解，应就医。 | | 抱宝宝时尽量用前臂和身体支撑，不要只用手腕托头，可使用哺乳枕并让家人分担。重点是正确含接、有效排奶和休息补水；若红肿热痛、发热或24小时不缓解，应就医。 |
| **English translation of model response / 模型回答英文翻译** | | Wrist pain after birth, especially while holding the baby, is often related to tenosynovitis or overuse, particularly when the thumb side hurts. Tell family and clinicians how you feel. Hopelessness, loss of control, or harm thoughts need immediate help. | | Even topical patches should be checked during breastfeeding. Avoid long-term self-use of irritating patches or products with strong medicines. Focus on effective milk removal and correct latch. Redness, fever, or no improvement within 24 hours needs care. | | Support the baby with the forearm and body rather than only the wrist. A nursing pillow and help from family can reduce strain. Record color, amount, odor, and pad use. Seek care for heavy bleeding, clots, pain, or fever. |
| **Overall evaluation item / 整体评价项目** | | | **Score / 分数** | | **Comments / 备注** | |
| **Patient Satisfaction / 患者满意度** | | | 4 | |  | |
| **Patient Understandability / 患者可理解性** | | | 4 | |  | |
| **Patient Usefulness / 患者实用性** | | | 4 | |  | |
| **Clinician Correctness / 医生评分：正确性** | | | 5 | |  | |
| **Clinician Completeness / 医生评分：完整性** | | | 5 | |  | |
| **Clinician Readability / 医生评分：可读性** | | | 4 | |  | |
| **Clinician Helpfulness / 医生评分：帮助性** | | | 5 | |  | |
| **Clinician Safety / 医生评分：安全性** | | | 5 | |  | |

**Patient 55**

| **Patient ID / 患者编号** | **Group / 模型组别** | | | | | |
| --- | --- | --- | --- | --- | --- | --- |
| P55 | ChatGPT 5.5 | | | | | |
| **General model response or patient education material (Chinese) / 模型总体回答或患者教育材料（中文）** | | | | | | |
| 乳房胀硬而宝宝吸不出时，可先短时间温敷、轻柔手挤少量乳汁，使乳晕变软后再让宝宝含接。重点是正确含接、有效排奶和休息补水；若红肿热痛、发热或24小时不缓解，应就医。吸奶器应以缓解胀痛和帮助排乳为目的，不宜长时间强吸，通常每次10到15分钟并观察乳头疼痛。重点是正确含接、有效排奶和休息补水；若红肿热痛、发热或24小时不缓解，应就医。若出现红斑、发热、寒战或症状24小时内不缓解，应及时就医，警惕乳腺炎或乳腺脓肿。建议复测体温，并观察恶露、乳房、尿痛和伤口；若体温≥38℃、寒战或精神差，应就医。以上内容为一般健康教育，不能替代线下面诊。 | | | | | | |
| **English translation of the Chinese general response / 中文总体回答英文翻译** | | | | | | |
| If the breast is very hard and the baby cannot latch or remove milk, use a brief warm compress and gently hand express a little milk to soften the areola first. Focus on effective milk removal and correct latch. Redness, fever, or no improvement within 24 hours needs care. A breast pump should be used to relieve engorgement and help drainage, not with prolonged strong suction. About 10 to 15 minutes each time is usually enough, while watching nipple pain. Focus on effective milk removal and correct latch. Redness, fever, or no improvement within 24 hours needs care. A red patch, fever, chills, or no improvement within 24 hours needs medical care because mastitis or breast abscess should be considered. Record color, amount, odor, and pad use. Seek care for heavy bleeding, clots, pain, or fever. This is general health education and does not replace an in-person medical assessment. | | | | | | |
| **Item / 项目** | | **Q1 / 问题1** | | **Q2 / 问题2** | | **Q3 / 问题3** |
| **Patient question (Chinese original) / 患者原始问题（中文）** | | 我乳房胀得很硬但宝宝吸不出来，怎么办？ | | 堵奶时用吸奶器吸多久比较合适？ | | 如果乳房有红斑并且发热，是不是要看医生？ |
| **English translation of patient question / 患者问题英文翻译** | | My breasts are very hard but the baby cannot suck the milk out. What should I do? | | How long should I use a breast pump when milk is blocked? | | If I have a red patch on the breast and fever, do I need to see a doctor? |
| **Model response (Chinese) / 模型回答（中文）** | | 乳房胀硬而宝宝吸不出时，可先短时间温敷、轻柔手挤少量乳汁，使乳晕变软后再让宝宝含接。重点是正确含接、有效排奶和休息补水；若红肿热痛、发热或24小时不缓解，应就医。 | | 吸奶器应以缓解胀痛和帮助排乳为目的，不宜长时间强吸，通常每次10到15分钟并观察乳头疼痛。重点是正确含接、有效排奶和休息补水；若红肿热痛、发热或24小时不缓解，应就医。 | | 若出现红斑、发热、寒战或症状24小时内不缓解，应及时就医，警惕乳腺炎或乳腺脓肿。建议复测体温，并观察恶露、乳房、尿痛和伤口；若体温≥38℃、寒战或精神差，应就医。 |
| **English translation of model response / 模型回答英文翻译** | | If the breast is very hard and the baby cannot latch or remove milk, use a brief warm compress and gently hand express a little milk to soften the areola first. Focus on effective milk removal and correct latch. Redness, fever, or no improvement within 24 hours needs care. | | A breast pump should be used to relieve engorgement and help drainage, not with prolonged strong suction. About 10 to 15 minutes each time is usually enough, while watching nipple pain. Focus on effective milk removal and correct latch. Redness, fever, or no improvement within 24 hours needs care. | | A red patch, fever, chills, or no improvement within 24 hours needs medical care because mastitis or breast abscess should be considered. Record color, amount, odor, and pad use. Seek care for heavy bleeding, clots, pain, or fever. |
| **Overall evaluation item / 整体评价项目** | | | **Score / 分数** | | **Comments / 备注** | |
| **Patient Satisfaction / 患者满意度** | | | 5 | |  | |
| **Patient Understandability / 患者可理解性** | | | 4 | |  | |
| **Patient Usefulness / 患者实用性** | | | 4 | |  | |
| **Clinician Correctness / 医生评分：正确性** | | | 5 | |  | |
| **Clinician Completeness / 医生评分：完整性** | | | 5 | |  | |
| **Clinician Readability / 医生评分：可读性** | | | 4 | |  | |
| **Clinician Helpfulness / 医生评分：帮助性** | | | 5 | |  | |
| **Clinician Safety / 医生评分：安全性** | | | 5 | |  | |

**Patient 56**

| **Patient ID / 患者编号** | **Group / 模型组别** | | | | | |
| --- | --- | --- | --- | --- | --- | --- |
| P56 | ChatGPT 5.5 | | | | | |
| **General model response or patient education material (Chinese) / 模型总体回答或患者教育材料（中文）** | | | | | | |
| 硬块反复出现常与含接不充分、排乳不均、压迫乳房或喂奶间隔过长有关，需要调整哺乳方式。重点是正确含接、有效排奶和休息补水；若红肿热痛、发热或24小时不缓解，应就医。深部硬块不建议用力按压或暴力揉搓，以免造成组织损伤和炎症加重。建议观察变化，保证休息、饮食和清洁护理；若症状持续不缓解或逐渐加重，应联系产科门诊。若硬块持续不消、变大、伴红肿发热，或同一部位反复出现，应到乳腺或母乳门诊评估，必要时做超声。建议复测体温，并观察恶露、乳房、尿痛和伤口；若体温≥38℃、寒战或精神差，应就医。以上内容为一般健康教育，不能替代线下面诊。 | | | | | | |
| **English translation of the Chinese general response / 中文总体回答英文翻译** | | | | | | |
| Repeated breast lumps are often related to poor latch, uneven milk removal, pressure on the breast, or long intervals between feeds. Feeding technique should be adjusted. Focus on effective milk removal and correct latch. Redness, fever, or no improvement within 24 hours needs care. Do not press deeply or massage forcefully, as this may injure breast tissue and worsen inflammation. Focus on effective milk removal and correct latch. Redness, fever, or no improvement within 24 hours needs care. If a lump persists, grows, comes with redness or fever, or recurs in the same area, visit a breast or lactation clinic. Ultrasound may be needed. Record color, amount, odor, and pad use. Seek care for heavy bleeding, clots, pain, or fever. This is general health education and does not replace an in-person medical assessment. | | | | | | |
| **Item / 项目** | | **Q1 / 问题1** | | **Q2 / 问题2** | | **Q3 / 问题3** |
| **Patient question (Chinese original) / 患者原始问题（中文）** | | 乳房硬块揉开后又反复出现，是不是喂奶方式不对？ | | 硬块位置很深，自己按压会不会损伤乳腺？ | | 如果硬块一直不消，需要做乳腺超声吗？ |
| **English translation of patient question / 患者问题英文翻译** | | A breast lump comes back after I massage it out. Is my feeding method wrong? | | The lump is deep. Could pressing it myself injure the breast? | | If the lump does not go away, do I need a breast ultrasound? |
| **Model response (Chinese) / 模型回答（中文）** | | 硬块反复出现常与含接不充分、排乳不均、压迫乳房或喂奶间隔过长有关，需要调整哺乳方式。重点是正确含接、有效排奶和休息补水；若红肿热痛、发热或24小时不缓解，应就医。 | | 深部硬块不建议用力按压或暴力揉搓，以免造成组织损伤和炎症加重。建议观察变化，保证休息、饮食和清洁护理；若症状持续不缓解或逐渐加重，应联系产科门诊。 | | 若硬块持续不消、变大、伴红肿发热，或同一部位反复出现，应到乳腺或母乳门诊评估，必要时做超声。建议复测体温，并观察恶露、乳房、尿痛和伤口；若体温≥38℃、寒战或精神差，应就医。 |
| **English translation of model response / 模型回答英文翻译** | | Repeated breast lumps are often related to poor latch, uneven milk removal, pressure on the breast, or long intervals between feeds. Feeding technique should be adjusted. Focus on effective milk removal and correct latch. Redness, fever, or no improvement within 24 hours needs care. | | Do not press deeply or massage forcefully, as this may injure breast tissue and worsen inflammation. Focus on effective milk removal and correct latch. Redness, fever, or no improvement within 24 hours needs care. | | If a lump persists, grows, comes with redness or fever, or recurs in the same area, visit a breast or lactation clinic. Ultrasound may be needed. Record color, amount, odor, and pad use. Seek care for heavy bleeding, clots, pain, or fever. |
| **Overall evaluation item / 整体评价项目** | | | **Score / 分数** | | **Comments / 备注** | |
| **Patient Satisfaction / 患者满意度** | | | 5 | |  | |
| **Patient Understandability / 患者可理解性** | | | 4 | |  | |
| **Patient Usefulness / 患者实用性** | | | 5 | |  | |
| **Clinician Correctness / 医生评分：正确性** | | | 4 | |  | |
| **Clinician Completeness / 医生评分：完整性** | | | 5 | |  | |
| **Clinician Readability / 医生评分：可读性** | | | 4 | |  | |
| **Clinician Helpfulness / 医生评分：帮助性** | | | 5 | |  | |
| **Clinician Safety / 医生评分：安全性** | | | 5 | |  | |

**Patient 57**

| **Patient ID / 患者编号** | **Group / 模型组别** | | | | | |
| --- | --- | --- | --- | --- | --- | --- |
| P57 | ChatGPT 5.5 | | | | | |
| **General model response or patient education material (Chinese) / 模型总体回答或患者教育材料（中文）** | | | | | | |
| 乳腺炎反复可能与排乳不充分、乳头损伤、过度挤压、疲劳或喂奶姿势有关。重点是正确含接、有效排奶和休息补水；若红肿热痛、发热或24小时不缓解，应就医。多数乳腺炎期间可继续哺乳或排奶，除非医生因特殊情况建议暂停某侧或调整方式。重点是正确含接、有效排奶和休息补水；若红肿热痛、发热或24小时不缓解，应就医。反复发作建议请母乳门诊评估含接、姿势和排乳节奏，并排查乳头皲裂和乳房局部病变。重点是正确含接、有效排奶和休息补水；若红肿热痛、发热或24小时不缓解，应就医。以上内容为一般健康教育，不能替代线下面诊。 | | | | | | |
| **English translation of the Chinese general response / 中文总体回答英文翻译** | | | | | | |
| Recurrent mastitis may be related to incomplete milk removal, nipple damage, forceful massage, fatigue, or poor feeding position. Focus on effective milk removal and correct latch. Redness, fever, or no improvement within 24 hours needs care. In most cases, breastfeeding or milk removal can continue during mastitis unless the clinician gives a specific reason to pause or adjust one side. Focus on effective milk removal and correct latch. Redness, fever, or no improvement within 24 hours needs care. With repeated episodes, a lactation clinic should assess latch, position, and feeding rhythm, and check for nipple cracks or local breast problems. Focus on effective milk removal and correct latch. Redness, fever, or no improvement within 24 hours needs care. This is general health education and does not replace an in-person medical assessment. | | | | | | |
| **Item / 项目** | | **Q1 / 问题1** | | **Q2 / 问题2** | | **Q3 / 问题3** |
| **Patient question (Chinese original) / 患者原始问题（中文）** | | 我乳腺炎刚好又复发，可能是什么原因？ | | 乳腺炎期间还能让宝宝吸患侧乳房吗？ | | 反复乳腺炎需要调整哺乳姿势吗？ |
| **English translation of patient question / 患者问题英文翻译** | | My mastitis improved but then came back. What might be the cause? | | Can the baby feed from the affected breast during mastitis? | | Does recurrent mastitis mean I should adjust breastfeeding positions? |
| **Model response (Chinese) / 模型回答（中文）** | | 乳腺炎反复可能与排乳不充分、乳头损伤、过度挤压、疲劳或喂奶姿势有关。重点是正确含接、有效排奶和休息补水；若红肿热痛、发热或24小时不缓解，应就医。 | | 多数乳腺炎期间可继续哺乳或排奶，除非医生因特殊情况建议暂停某侧或调整方式。重点是正确含接、有效排奶和休息补水；若红肿热痛、发热或24小时不缓解，应就医。 | | 反复发作建议请母乳门诊评估含接、姿势和排乳节奏，并排查乳头皲裂和乳房局部病变。重点是正确含接、有效排奶和休息补水；若红肿热痛、发热或24小时不缓解，应就医。 |
| **English translation of model response / 模型回答英文翻译** | | Recurrent mastitis may be related to incomplete milk removal, nipple damage, forceful massage, fatigue, or poor feeding position. Focus on effective milk removal and correct latch. Redness, fever, or no improvement within 24 hours needs care. | | In most cases, breastfeeding or milk removal can continue during mastitis unless the clinician gives a specific reason to pause or adjust one side. Focus on effective milk removal and correct latch. Redness, fever, or no improvement within 24 hours needs care. | | With repeated episodes, a lactation clinic should assess latch, position, and feeding rhythm, and check for nipple cracks or local breast problems. Focus on effective milk removal and correct latch. Redness, fever, or no improvement within 24 hours needs care. |
| **Overall evaluation item / 整体评价项目** | | | **Score / 分数** | | **Comments / 备注** | |
| **Patient Satisfaction / 患者满意度** | | | 4 | |  | |
| **Patient Understandability / 患者可理解性** | | | 4 | |  | |
| **Patient Usefulness / 患者实用性** | | | 5 | |  | |
| **Clinician Correctness / 医生评分：正确性** | | | 5 | |  | |
| **Clinician Completeness / 医生评分：完整性** | | | 5 | |  | |
| **Clinician Readability / 医生评分：可读性** | | | 4 | |  | |
| **Clinician Helpfulness / 医生评分：帮助性** | | | 5 | |  | |
| **Clinician Safety / 医生评分：安全性** | | | 5 | |  | |

**Patient 58**

| **Patient ID / 患者编号** | **Group / 模型组别** | | | | | |
| --- | --- | --- | --- | --- | --- | --- |
| P58 | ChatGPT 5.5 | | | | | |
| **General model response or patient education material (Chinese) / 模型总体回答或患者教育材料（中文）** | | | | | | |
| 奶量少不一定说明体质不适合母乳喂养，常与含接、喂奶次数、休息和母婴分离有关。重点是正确含接、有效排奶和休息补水；若红肿热痛、发热或24小时不缓解，应就医。追奶通常需要增加有效吸吮或排奶次数，可按医生或母乳顾问建议安排，一般不建议盲目频繁强吸。重点是正确含接、有效排奶和休息补水；若红肿热痛、发热或24小时不缓解，应就医。判断奶量要看宝宝体重增长、尿量、大便、吞咽声和精神状态，体重增长慢应请儿科和母乳门诊共同评估。重点是正确含接、有效排奶和休息补水；若红肿热痛、发热或24小时不缓解，应就医。以上内容为一般健康教育，不能替代线下面诊。 | | | | | | |
| **English translation of the Chinese general response / 中文总体回答英文翻译** | | | | | | |
| Low milk supply does not necessarily mean your body is unsuitable for breastfeeding. Latch, feeding frequency, rest, and separation from the baby often matter. Focus on effective milk removal and correct latch. Redness, fever, or no improvement within 24 hours needs care. Increasing effective breastfeeding or milk expression can help, but the plan should be guided by a clinician or lactation consultant. Avoid blindly pumping too often or too strongly. Focus on effective milk removal and correct latch. Redness, fever, or no improvement within 24 hours needs care. Milk adequacy should be judged by weight gain, wet diapers, stool pattern, swallowing, and alertness. Slow weight gain needs joint assessment by pediatrics and lactation services. Focus on effective milk removal and correct latch. Redness, fever, or no improvement within 24 hours needs care. This is general health education and does not replace an in-person medical assessment. | | | | | | |
| **Item / 项目** | | **Q1 / 问题1** | | **Q2 / 问题2** | | **Q3 / 问题3** |
| **Patient question (Chinese original) / 患者原始问题（中文）** | | 我奶量一直不够，是不是体质不适合母乳喂养？ | | 每天需要吸奶几次才有助于追奶？ | | 宝宝体重增长慢时应该怎么判断奶量够不够？ |
| **English translation of patient question / 患者问题英文翻译** | | My milk supply has always been low. Does that mean my body is not suitable for breastfeeding? | | How many times should I pump each day to help increase milk supply? | | If the baby gains weight slowly, how can I tell whether milk is enough? |
| **Model response (Chinese) / 模型回答（中文）** | | 奶量少不一定说明体质不适合母乳喂养，常与含接、喂奶次数、休息和母婴分离有关。重点是正确含接、有效排奶和休息补水；若红肿热痛、发热或24小时不缓解，应就医。 | | 追奶通常需要增加有效吸吮或排奶次数，可按医生或母乳顾问建议安排，一般不建议盲目频繁强吸。重点是正确含接、有效排奶和休息补水；若红肿热痛、发热或24小时不缓解，应就医。 | | 判断奶量要看宝宝体重增长、尿量、大便、吞咽声和精神状态，体重增长慢应请儿科和母乳门诊共同评估。重点是正确含接、有效排奶和休息补水；若红肿热痛、发热或24小时不缓解，应就医。 |
| **English translation of model response / 模型回答英文翻译** | | Low milk supply does not necessarily mean your body is unsuitable for breastfeeding. Latch, feeding frequency, rest, and separation from the baby often matter. Focus on effective milk removal and correct latch. Redness, fever, or no improvement within 24 hours needs care. | | Increasing effective breastfeeding or milk expression can help, but the plan should be guided by a clinician or lactation consultant. Avoid blindly pumping too often or too strongly. Focus on effective milk removal and correct latch. Redness, fever, or no improvement within 24 hours needs care. | | Milk adequacy should be judged by weight gain, wet diapers, stool pattern, swallowing, and alertness. Slow weight gain needs joint assessment by pediatrics and lactation services. Focus on effective milk removal and correct latch. Redness, fever, or no improvement within 24 hours needs care. |
| **Overall evaluation item / 整体评价项目** | | | **Score / 分数** | | **Comments / 备注** | |
| **Patient Satisfaction / 患者满意度** | | | 5 | |  | |
| **Patient Understandability / 患者可理解性** | | | 4 | |  | |
| **Patient Usefulness / 患者实用性** | | | 4 | |  | |
| **Clinician Correctness / 医生评分：正确性** | | | 5 | |  | |
| **Clinician Completeness / 医生评分：完整性** | | | 5 | |  | |
| **Clinician Readability / 医生评分：可读性** | | | 4 | |  | |
| **Clinician Helpfulness / 医生评分：帮助性** | | | 5 | |  | |
| **Clinician Safety / 医生评分：安全性** | | | 5 | |  | |

**Patient 59**

| **Patient ID / 患者编号** | **Group / 模型组别** | | | | | |
| --- | --- | --- | --- | --- | --- | --- |
| P59 | ChatGPT 5.5 | | | | | |
| **General model response or patient education material (Chinese) / 模型总体回答或患者教育材料（中文）** | | | | | | |
| 产后奶多漏奶较常见，尤其供需尚未稳定时，一般会随哺乳规律建立而改善。重点是正确含接、有效排奶和休息补水；若红肿热痛、发热或24小时不缓解，应就医。奶阵太急时可先手挤少量乳汁，再让宝宝含接，采取半躺位或让宝宝头部稍高，减少呛奶。重点是正确含接、有效排奶和休息补水；若红肿热痛、发热或24小时不缓解，应就医。乳房长期过度胀满容易增加堵奶风险，应按需哺乳，避免过度排空导致奶量进一步增多。重点是正确含接、有效排奶和休息补水；若红肿热痛、发热或24小时不缓解，应就医。以上内容为一般健康教育，不能替代线下面诊。 | | | | | | |
| **English translation of the Chinese general response / 中文总体回答英文翻译** | | | | | | |
| Oversupply and leaking milk are common, especially before supply and demand become stable. It often improves as feeding becomes regular. Focus on effective milk removal and correct latch. Redness, fever, or no improvement within 24 hours needs care. If let-down is too strong, express a little milk first, then latch the baby. A laid-back position or keeping the baby’s head slightly higher may reduce choking. Record color, amount, odor, and pad use. Seek care for heavy bleeding, clots, pain, or fever. Breasts that stay overly full may raise the risk of blocked ducts. Feed on demand, but avoid excessive emptying that may further increase supply. Focus on effective milk removal and correct latch. Redness, fever, or no improvement within 24 hours needs care. This is general health education and does not replace an in-person medical assessment. | | | | | | |
| **Item / 项目** | | **Q1 / 问题1** | | **Q2 / 问题2** | | **Q3 / 问题3** |
| **Patient question (Chinese original) / 患者原始问题（中文）** | | 我奶太多总是漏奶，正常吗？ | | 奶阵太急宝宝容易呛奶，应该怎么喂？ | | 乳房总是涨满会不会更容易堵奶？ |
| **English translation of patient question / 患者问题英文翻译** | | I have too much milk and keep leaking. Is this normal? | | My let-down is too strong and the baby often chokes. How should I feed? | | Will breasts that are always full be more likely to become blocked? |
| **Model response (Chinese) / 模型回答（中文）** | | 产后奶多漏奶较常见，尤其供需尚未稳定时，一般会随哺乳规律建立而改善。重点是正确含接、有效排奶和休息补水；若红肿热痛、发热或24小时不缓解，应就医。 | | 奶阵太急时可先手挤少量乳汁，再让宝宝含接，采取半躺位或让宝宝头部稍高，减少呛奶。重点是正确含接、有效排奶和休息补水；若红肿热痛、发热或24小时不缓解，应就医。 | | 乳房长期过度胀满容易增加堵奶风险，应按需哺乳，避免过度排空导致奶量进一步增多。重点是正确含接、有效排奶和休息补水；若红肿热痛、发热或24小时不缓解，应就医。 |
| **English translation of model response / 模型回答英文翻译** | | Oversupply and leaking milk are common, especially before supply and demand become stable. It often improves as feeding becomes regular. Focus on effective milk removal and correct latch. Redness, fever, or no improvement within 24 hours needs care. | | If let-down is too strong, express a little milk first, then latch the baby. A laid-back position or keeping the baby’s head slightly higher may reduce choking. Record color, amount, odor, and pad use. Seek care for heavy bleeding, clots, pain, or fever. | | Breasts that stay overly full may raise the risk of blocked ducts. Feed on demand, but avoid excessive emptying that may further increase supply. Focus on effective milk removal and correct latch. Redness, fever, or no improvement within 24 hours needs care. |
| **Overall evaluation item / 整体评价项目** | | | **Score / 分数** | | **Comments / 备注** | |
| **Patient Satisfaction / 患者满意度** | | | 4 | |  | |
| **Patient Understandability / 患者可理解性** | | | 4 | |  | |
| **Patient Usefulness / 患者实用性** | | | 4 | |  | |
| **Clinician Correctness / 医生评分：正确性** | | | 5 | |  | |
| **Clinician Completeness / 医生评分：完整性** | | | 5 | |  | |
| **Clinician Readability / 医生评分：可读性** | | | 4 | |  | |
| **Clinician Helpfulness / 医生评分：帮助性** | | | 5 | |  | |
| **Clinician Safety / 医生评分：安全性** | | | 5 | |  | |

**Patient 60**

| **Patient ID / 患者编号** | **Group / 模型组别** | | | | | |
| --- | --- | --- | --- | --- | --- | --- |
| P60 | ChatGPT 5.5 | | | | | |
| **General model response or patient education material (Chinese) / 模型总体回答或患者教育材料（中文）** | | | | | | |
| 乳头白色小点伴疼痛可能是乳头白泡、乳管口堵塞或摩擦损伤，需要结合含接和乳房情况判断。重点是正确含接、有效排奶和休息补水；若红肿热痛、发热或24小时不缓解，应就医。不建议自己挑破，以免感染和创面加重，可先热敷、调整含接并轻柔排乳。建议复测体温，并观察恶露、乳房、尿痛和伤口；若体温≥38℃、寒战或精神差，应就医。若疼痛影响喂奶、白点反复出现或伴乳房硬块，应到母乳门诊处理，必要时由专业人员进行局部治疗。重点是正确含接、有效排奶和休息补水；若红肿热痛、发热或24小时不缓解，应就医。以上内容为一般健康教育，不能替代线下面诊。 | | | | | | |
| **English translation of the Chinese general response / 中文总体回答英文翻译** | | | | | | |
| A painful white spot on the nipple may be a nipple bleb, blocked duct opening, or friction injury. Latch and breast condition should be assessed. Focus on effective milk removal and correct latch. Redness, fever, or no improvement within 24 hours needs care. Do not puncture it yourself because infection and wound worsening may occur. Try warm compresses, better latch, and gentle milk removal first. Recheck temperature and watch lochia, breasts, urination, and wounds. Fever at or above 38°C needs care. If pain affects feeding, the white spot recurs, or a breast lump is present, visit a lactation clinic. Local treatment by trained staff may be needed. Focus on effective milk removal and correct latch. Redness, fever, or no improvement within 24 hours needs care. This is general health education and does not replace an in-person medical assessment. | | | | | | |
| **Item / 项目** | | **Q1 / 问题1** | | **Q2 / 问题2** | | **Q3 / 问题3** |
| **Patient question (Chinese original) / 患者原始问题（中文）** | | 我的乳头上有白色小点，喂奶时很疼，是乳头白泡吗？ | | 乳头白泡可以自己挑破吗？ | | 如果乳头疼痛影响喂奶，需要去母乳门诊吗？ |
| **English translation of patient question / 患者问题英文翻译** | | There is a small white spot on my nipple and breastfeeding is painful. Could it be a nipple bleb? | | Can I puncture a nipple bleb by myself? | | If nipple pain affects feeding, should I go to a lactation clinic? |
| **Model response (Chinese) / 模型回答（中文）** | | 乳头白色小点伴疼痛可能是乳头白泡、乳管口堵塞或摩擦损伤，需要结合含接和乳房情况判断。重点是正确含接、有效排奶和休息补水；若红肿热痛、发热或24小时不缓解，应就医。 | | 不建议自己挑破，以免感染和创面加重，可先热敷、调整含接并轻柔排乳。建议复测体温，并观察恶露、乳房、尿痛和伤口；若体温≥38℃、寒战或精神差，应就医。 | | 若疼痛影响喂奶、白点反复出现或伴乳房硬块，应到母乳门诊处理，必要时由专业人员进行局部治疗。重点是正确含接、有效排奶和休息补水；若红肿热痛、发热或24小时不缓解，应就医。 |
| **English translation of model response / 模型回答英文翻译** | | A painful white spot on the nipple may be a nipple bleb, blocked duct opening, or friction injury. Latch and breast condition should be assessed. Focus on effective milk removal and correct latch. Redness, fever, or no improvement within 24 hours needs care. | | Do not puncture it yourself because infection and wound worsening may occur. Try warm compresses, better latch, and gentle milk removal first. Recheck temperature and watch lochia, breasts, urination, and wounds. Fever at or above 38°C needs care. | | If pain affects feeding, the white spot recurs, or a breast lump is present, visit a lactation clinic. Local treatment by trained staff may be needed. Focus on effective milk removal and correct latch. Redness, fever, or no improvement within 24 hours needs care. |
| **Overall evaluation item / 整体评价项目** | | | **Score / 分数** | | **Comments / 备注** | |
| **Patient Satisfaction / 患者满意度** | | | 4 | |  | |
| **Patient Understandability / 患者可理解性** | | | 4 | |  | |
| **Patient Usefulness / 患者实用性** | | | 4 | |  | |
| **Clinician Correctness / 医生评分：正确性** | | | 4 | |  | |
| **Clinician Completeness / 医生评分：完整性** | | | 5 | |  | |
| **Clinician Readability / 医生评分：可读性** | | | 4 | |  | |
| **Clinician Helpfulness / 医生评分：帮助性** | | | 5 | |  | |
| **Clinician Safety / 医生评分：安全性** | | | 4 | |  | |

**Patient 61**

| **Patient ID / 患者编号** | **Group / 模型组别** | | | | | |
| --- | --- | --- | --- | --- | --- | --- |
| P61 | ChatGPT 5.5 | | | | | |
| **General model response or patient education material (Chinese) / 模型总体回答或患者教育材料（中文）** | | | | | | |
| 哺乳时针扎样疼痛可见于奶阵、乳头损伤、乳管痉挛或感染，若持续或加重需评估。建议复测体温，并观察恶露、乳房、尿痛和伤口；若体温≥38℃、寒战或精神差，应就医。乳头和宝宝口腔都有白色附着物，可能提示念珠菌感染，应由医生或母乳门诊确认。建议复测体温，并观察恶露、乳房、尿痛和伤口；若体温≥38℃、寒战或精神差，应就医。疑似感染时多数情况下仍可继续哺乳，但母婴可能需要同时治疗，并注意乳头、奶具和手卫生。建议复测体温，并观察恶露、乳房、尿痛和伤口；若体温≥38℃、寒战或精神差，应就医。以上内容为一般健康教育，不能替代线下面诊。 | | | | | | |
| **English translation of the Chinese general response / 中文总体回答英文翻译** | | | | | | |
| Needle-like breast pain during feeding may be related to let-down, nipple injury, duct spasm, or infection. Persistent or worsening pain needs assessment. Recheck temperature and watch lochia, breasts, urination, and wounds. Fever at or above 38°C needs care. White patches on both the nipple and the baby’s mouth may suggest Candida infection and should be confirmed by a clinician or lactation clinic. Recheck temperature and watch lochia, breasts, urination, and wounds. Fever at or above 38°C needs care. In suspected infection, breastfeeding can often continue, but both mother and baby may need treatment. Nipple, pump, and hand hygiene are important. Recheck temperature and watch lochia, breasts, urination, and wounds. Fever at or above 38°C needs care. This is general health education and does not replace an in-person medical assessment. | | | | | | |
| **Item / 项目** | | **Q1 / 问题1** | | **Q2 / 问题2** | | **Q3 / 问题3** |
| **Patient question (Chinese original) / 患者原始问题（中文）** | | 我哺乳时乳房像针扎一样疼，是正常的吗？ | | 乳头和宝宝口腔都有白色东西，会不会是真菌感染？ | | 如果怀疑感染，还能继续母乳喂养吗？ |
| **English translation of patient question / 患者问题英文翻译** | | My breast feels like it is being pricked with needles during breastfeeding. Is this normal? | | Both my nipple and the baby’s mouth have white patches. Could it be a fungal infection? | | If infection is suspected, can I continue breastfeeding? |
| **Model response (Chinese) / 模型回答（中文）** | | 哺乳时针扎样疼痛可见于奶阵、乳头损伤、乳管痉挛或感染，若持续或加重需评估。建议复测体温，并观察恶露、乳房、尿痛和伤口；若体温≥38℃、寒战或精神差，应就医。 | | 乳头和宝宝口腔都有白色附着物，可能提示念珠菌感染，应由医生或母乳门诊确认。建议复测体温，并观察恶露、乳房、尿痛和伤口；若体温≥38℃、寒战或精神差，应就医。 | | 疑似感染时多数情况下仍可继续哺乳，但母婴可能需要同时治疗，并注意乳头、奶具和手卫生。建议复测体温，并观察恶露、乳房、尿痛和伤口；若体温≥38℃、寒战或精神差，应就医。 |
| **English translation of model response / 模型回答英文翻译** | | Needle-like breast pain during feeding may be related to let-down, nipple injury, duct spasm, or infection. Persistent or worsening pain needs assessment. Recheck temperature and watch lochia, breasts, urination, and wounds. Fever at or above 38°C needs care. | | White patches on both the nipple and the baby’s mouth may suggest Candida infection and should be confirmed by a clinician or lactation clinic. Recheck temperature and watch lochia, breasts, urination, and wounds. Fever at or above 38°C needs care. | | In suspected infection, breastfeeding can often continue, but both mother and baby may need treatment. Nipple, pump, and hand hygiene are important. Recheck temperature and watch lochia, breasts, urination, and wounds. Fever at or above 38°C needs care. |
| **Overall evaluation item / 整体评价项目** | | | **Score / 分数** | | **Comments / 备注** | |
| **Patient Satisfaction / 患者满意度** | | | 4 | |  | |
| **Patient Understandability / 患者可理解性** | | | 4 | |  | |
| **Patient Usefulness / 患者实用性** | | | 5 | |  | |
| **Clinician Correctness / 医生评分：正确性** | | | 5 | |  | |
| **Clinician Completeness / 医生评分：完整性** | | | 5 | |  | |
| **Clinician Readability / 医生评分：可读性** | | | 4 | |  | |
| **Clinician Helpfulness / 医生评分：帮助性** | | | 5 | |  | |
| **Clinician Safety / 医生评分：安全性** | | | 5 | |  | |

**Patient 62**

| **Patient ID / 患者编号** | **Group / 模型组别** | | | | | |
| --- | --- | --- | --- | --- | --- | --- |
| P62 | ChatGPT 5.5 | | | | | |
| **General model response or patient education material (Chinese) / 模型总体回答或患者教育材料（中文）** | | | | | | |
| 哺乳期月经恢复后奶量可能短暂波动，但通常不会长期影响母乳喂养。重点是正确含接、有效排奶和休息补水；若红肿热痛、发热或24小时不缓解，应就医。恶露多在产后逐渐变淡变少，月经常有较规律周期和经量变化；若不确定，应结合产后时间和症状判断。建议记录颜色、量和气味，避免劳累；若1小时湿透卫生巾、血块增多、腹痛或发热，应就医。若出血量很大、1小时湿透一片卫生巾、有大血块、头晕或腹痛明显，应及时就医。建议记录颜色、量和气味，避免劳累；若1小时湿透卫生巾、血块增多、腹痛或发热，应就医。以上内容为一般健康教育，不能替代线下面诊。 | | | | | | |
| **English translation of the Chinese general response / 中文总体回答英文翻译** | | | | | | |
| When menstruation returns during breastfeeding, milk supply may fluctuate briefly, but it usually does not cause a long-term problem. Focus on effective milk removal and correct latch. Redness, fever, or no improvement within 24 hours needs care. Lochia usually becomes lighter and less over time, while a period tends to have a more cyclical pattern. The timing after birth and symptoms should be considered. Record color, amount, odor, and pad use. Seek care for heavy bleeding, clots, pain, or fever. Very heavy bleeding, soaking one pad within an hour, large clots, dizziness, or significant abdominal pain needs prompt medical care. Record color, amount, odor, and pad use. Seek care for heavy bleeding, clots, pain, or fever. This is general health education and does not replace an in-person medical assessment. | | | | | | |
| **Item / 项目** | | **Q1 / 问题1** | | **Q2 / 问题2** | | **Q3 / 问题3** |
| **Patient question (Chinese original) / 患者原始问题（中文）** | | 哺乳期突然来月经，会影响奶量吗？ | | 月经恢复后恶露和月经怎么区分？ | | 如果月经量特别多，需要去医院吗？ |
| **English translation of patient question / 患者问题英文翻译** | | My period suddenly returned while I am breastfeeding. Will it affect milk supply? | | How can I tell the difference between lochia and a period after birth? | | If the menstrual flow is very heavy, do I need to go to the hospital? |
| **Model response (Chinese) / 模型回答（中文）** | | 哺乳期月经恢复后奶量可能短暂波动，但通常不会长期影响母乳喂养。重点是正确含接、有效排奶和休息补水；若红肿热痛、发热或24小时不缓解，应就医。 | | 恶露多在产后逐渐变淡变少，月经常有较规律周期和经量变化；若不确定，应结合产后时间和症状判断。建议记录颜色、量和气味，避免劳累；若1小时湿透卫生巾、血块增多、腹痛或发热，应就医。 | | 若出血量很大、1小时湿透一片卫生巾、有大血块、头晕或腹痛明显，应及时就医。建议记录颜色、量和气味，避免劳累；若1小时湿透卫生巾、血块增多、腹痛或发热，应就医。 |
| **English translation of model response / 模型回答英文翻译** | | When menstruation returns during breastfeeding, milk supply may fluctuate briefly, but it usually does not cause a long-term problem. Focus on effective milk removal and correct latch. Redness, fever, or no improvement within 24 hours needs care. | | Lochia usually becomes lighter and less over time, while a period tends to have a more cyclical pattern. The timing after birth and symptoms should be considered. Record color, amount, odor, and pad use. Seek care for heavy bleeding, clots, pain, or fever. | | Very heavy bleeding, soaking one pad within an hour, large clots, dizziness, or significant abdominal pain needs prompt medical care. Record color, amount, odor, and pad use. Seek care for heavy bleeding, clots, pain, or fever. |
| **Overall evaluation item / 整体评价项目** | | | **Score / 分数** | | **Comments / 备注** | |
| **Patient Satisfaction / 患者满意度** | | | 4 | |  | |
| **Patient Understandability / 患者可理解性** | | | 4 | |  | |
| **Patient Usefulness / 患者实用性** | | | 5 | |  | |
| **Clinician Correctness / 医生评分：正确性** | | | 5 | |  | |
| **Clinician Completeness / 医生评分：完整性** | | | 5 | |  | |
| **Clinician Readability / 医生评分：可读性** | | | 4 | |  | |
| **Clinician Helpfulness / 医生评分：帮助性** | | | 5 | |  | |
| **Clinician Safety / 医生评分：安全性** | | | 5 | |  | |

**Patient 63**

| **Patient ID / 患者编号** | **Group / 模型组别** | | | | | |
| --- | --- | --- | --- | --- | --- | --- |
| P63 | ChatGPT 5.5 | | | | | |
| **General model response or patient education material (Chinese) / 模型总体回答或患者教育材料（中文）** | | | | | | |
| 即使哺乳期没有来月经，也可能先排卵后再来月经，因此仍有怀孕可能。重点是正确含接、有效排奶和休息补水；若红肿热痛、发热或24小时不缓解，应就医。哺乳期避孕药不能随便吃，应选择适合哺乳期的方法，如避孕套、宫内节育器或医生建议的孕激素类方法。重点是正确含接、有效排奶和休息补水；若红肿热痛、发热或24小时不缓解，应就医。若已无保护同房，应尽快咨询医生，评估是否需要紧急避孕，并在适当时间进行妊娠检测。恢复前应确认恶露基本干净、伤口不疼，并做好避孕；若疼痛、出血或紧张明显，应咨询医生。以上内容为一般健康教育，不能替代线下面诊。 | | | | | | |
| **English translation of the Chinese general response / 中文总体回答英文翻译** | | | | | | |
| Even without menstruation, ovulation can occur before the first period returns during breastfeeding, so pregnancy is still possible. Focus on effective milk removal and correct latch. Redness, fever, or no improvement within 24 hours needs care. Do not choose contraceptive pills casually while breastfeeding. Suitable options may include condoms, an intrauterine device, or progestin-only methods recommended by a clinician. Focus on effective milk removal and correct latch. Redness, fever, or no improvement within 24 hours needs care. After unprotected sex, consult a clinician promptly to discuss emergency contraception if appropriate and take a pregnancy test at the right time. Resume only when wounds and lochia are stable, and use contraception. Pain or bleeding needs medical advice. This is general health education and does not replace an in-person medical assessment. | | | | | | |
| **Item / 项目** | | **Q1 / 问题1** | | **Q2 / 问题2** | | **Q3 / 问题3** |
| **Patient question (Chinese original) / 患者原始问题（中文）** | | 我还在哺乳，没有来月经也会排卵吗？ | | 哺乳期避孕药可以随便吃吗？ | | 如果已经同房但没有避孕，需要怎么处理？ |
| **English translation of patient question / 患者问题英文翻译** | | Can I ovulate while breastfeeding even if my period has not returned? | | Can I take any contraceptive pill while breastfeeding? | | If I already had sex without contraception, what should I do? |
| **Model response (Chinese) / 模型回答（中文）** | | 即使哺乳期没有来月经，也可能先排卵后再来月经，因此仍有怀孕可能。重点是正确含接、有效排奶和休息补水；若红肿热痛、发热或24小时不缓解，应就医。 | | 哺乳期避孕药不能随便吃，应选择适合哺乳期的方法，如避孕套、宫内节育器或医生建议的孕激素类方法。重点是正确含接、有效排奶和休息补水；若红肿热痛、发热或24小时不缓解，应就医。 | | 若已无保护同房，应尽快咨询医生，评估是否需要紧急避孕，并在适当时间进行妊娠检测。恢复前应确认恶露基本干净、伤口不疼，并做好避孕；若疼痛、出血或紧张明显，应咨询医生。 |
| **English translation of model response / 模型回答英文翻译** | | Even without menstruation, ovulation can occur before the first period returns during breastfeeding, so pregnancy is still possible. Focus on effective milk removal and correct latch. Redness, fever, or no improvement within 24 hours needs care. | | Do not choose contraceptive pills casually while breastfeeding. Suitable options may include condoms, an intrauterine device, or progestin-only methods recommended by a clinician. Focus on effective milk removal and correct latch. Redness, fever, or no improvement within 24 hours needs care. | | After unprotected sex, consult a clinician promptly to discuss emergency contraception if appropriate and take a pregnancy test at the right time. Resume only when wounds and lochia are stable, and use contraception. Pain or bleeding needs medical advice. |
| **Overall evaluation item / 整体评价项目** | | | **Score / 分数** | | **Comments / 备注** | |
| **Patient Satisfaction / 患者满意度** | | | 5 | |  | |
| **Patient Understandability / 患者可理解性** | | | 4 | |  | |
| **Patient Usefulness / 患者实用性** | | | 4 | |  | |
| **Clinician Correctness / 医生评分：正确性** | | | 5 | |  | |
| **Clinician Completeness / 医生评分：完整性** | | | 5 | |  | |
| **Clinician Readability / 医生评分：可读性** | | | 4 | |  | |
| **Clinician Helpfulness / 医生评分：帮助性** | | | 5 | |  | |
| **Clinician Safety / 医生评分：安全性** | | | 5 | |  | |

**Patient 64**

| **Patient ID / 患者编号** | **Group / 模型组别** | | | | | |
| --- | --- | --- | --- | --- | --- | --- |
| P64 | ChatGPT 5.5 | | | | | |
| **General model response or patient education material (Chinese) / 模型总体回答或患者教育材料（中文）** | | | | | | |
| 产后42天复查时能否做宫颈筛查，应根据恶露是否干净、宫颈恢复和既往筛查情况决定。建议记录颜色、量和气味，避免劳累；若1小时湿透卫生巾、血块增多、腹痛或发热，应就医。若有阴道炎症状，可能影响分泌物检查和宫颈细胞结果，医生通常会先评估和处理炎症。建议记录颜色、量和气味，避免劳累；若1小时湿透卫生巾、血块增多、腹痛或发热，应就医。白带异常伴瘙痒、异味、疼痛或出血时，不必等到42天，应提前就诊。建议记录颜色、量和气味，避免劳累；若1小时湿透卫生巾、血块增多、腹痛或发热，应就医。以上内容为一般健康教育，不能替代线下面诊。 | | | | | | |
| **English translation of the Chinese general response / 中文总体回答英文翻译** | | | | | | |
| Whether cervical screening can be done at the 42-day check-up depends on whether lochia has stopped, cervical recovery, and previous screening history. Record color, amount, odor, and pad use. Seek care for heavy bleeding, clots, pain, or fever. Vaginitis symptoms may affect discharge tests and cervical cytology. Clinicians often assess and treat inflammation first. Record color, amount, odor, and pad use. Seek care for heavy bleeding, clots, pain, or fever. Abnormal discharge with itching, odor, pain, or bleeding should be seen earlier rather than waiting until 42 days. Record color, amount, odor, and pad use. Seek care for heavy bleeding, clots, pain, or fever. This is general health education and does not replace an in-person medical assessment. | | | | | | |
| **Item / 项目** | | **Q1 / 问题1** | | **Q2 / 问题2** | | **Q3 / 问题3** |
| **Patient question (Chinese original) / 患者原始问题（中文）** | | 产后42天复查可以顺便做宫颈筛查吗？ | | 产后有阴道炎症状，会影响复查结果吗？ | | 如果白带异常伴有瘙痒，需要提前就诊吗？ |
| **English translation of patient question / 患者问题英文翻译** | | Can I have cervical screening during the 42-day postpartum check-up? | | Will symptoms of vaginitis affect the check-up results? | | If abnormal discharge is accompanied by itching, should I see a doctor earlier? |
| **Model response (Chinese) / 模型回答（中文）** | | 产后42天复查时能否做宫颈筛查，应根据恶露是否干净、宫颈恢复和既往筛查情况决定。建议记录颜色、量和气味，避免劳累；若1小时湿透卫生巾、血块增多、腹痛或发热，应就医。 | | 若有阴道炎症状，可能影响分泌物检查和宫颈细胞结果，医生通常会先评估和处理炎症。建议记录颜色、量和气味，避免劳累；若1小时湿透卫生巾、血块增多、腹痛或发热，应就医。 | | 白带异常伴瘙痒、异味、疼痛或出血时，不必等到42天，应提前就诊。建议记录颜色、量和气味，避免劳累；若1小时湿透卫生巾、血块增多、腹痛或发热，应就医。 |
| **English translation of model response / 模型回答英文翻译** | | Whether cervical screening can be done at the 42-day check-up depends on whether lochia has stopped, cervical recovery, and previous screening history. Record color, amount, odor, and pad use. Seek care for heavy bleeding, clots, pain, or fever. | | Vaginitis symptoms may affect discharge tests and cervical cytology. Clinicians often assess and treat inflammation first. Record color, amount, odor, and pad use. Seek care for heavy bleeding, clots, pain, or fever. | | Abnormal discharge with itching, odor, pain, or bleeding should be seen earlier rather than waiting until 42 days. Record color, amount, odor, and pad use. Seek care for heavy bleeding, clots, pain, or fever. |
| **Overall evaluation item / 整体评价项目** | | | **Score / 分数** | | **Comments / 备注** | |
| **Patient Satisfaction / 患者满意度** | | | 4 | |  | |
| **Patient Understandability / 患者可理解性** | | | 4 | |  | |
| **Patient Usefulness / 患者实用性** | | | 4 | |  | |
| **Clinician Correctness / 医生评分：正确性** | | | 4 | |  | |
| **Clinician Completeness / 医生评分：完整性** | | | 5 | |  | |
| **Clinician Readability / 医生评分：可读性** | | | 4 | |  | |
| **Clinician Helpfulness / 医生评分：帮助性** | | | 5 | |  | |
| **Clinician Safety / 医生评分：安全性** | | | 5 | |  | |

**Patient 65**

| **Patient ID / 患者编号** | **Group / 模型组别** | | | | | |
| --- | --- | --- | --- | --- | --- | --- |
| P65 | ChatGPT 5.5 | | | | | |
| **General model response or patient education material (Chinese) / 模型总体回答或患者教育材料（中文）** | | | | | | |
| 排便时阴道口压迫感可能与盆底肌松弛、阴道壁膨出或便秘用力有关，建议做盆底评估。建议增加饮水和膳食纤维，适量活动；若便血增多、腹胀明显或疼痛剧烈，应就医。盆底肌力差可能影响漏尿、下坠感和性生活舒适度，但多数可通过规范训练和康复改善。建议适量饮水、规律排尿；若尿痛加重、发热、腰痛或排尿困难持续，应查尿常规。轻度问题可在指导下居家训练，中重度或症状明显者建议到医院或康复门诊接受评估和治疗。训练应循序渐进，避免过早强度运动；若症状持续、加重或影响生活，应做康复评估。以上内容为一般健康教育，不能替代线下面诊。 | | | | | | |
| **English translation of the Chinese general response / 中文总体回答英文翻译** | | | | | | |
| Pressure at the vaginal opening during bowel movements may be related to pelvic floor weakness, vaginal wall bulging, or straining from constipation. Pelvic floor assessment is advised. Increase fluids, fiber, and gentle activity. More bleeding, marked bloating, or severe pain needs care. Weak pelvic floor muscles may affect leakage, heaviness, and comfort during sex, but many problems improve with proper training and rehabilitation. Increase exercises gradually and avoid early high intensity. Persistent or worsening symptoms need rehabilitation assessment. Mild cases may train at home with guidance. Moderate, severe, or symptomatic cases should be assessed and treated in a hospital or rehabilitation clinic. Increase exercises gradually and avoid early high intensity. Persistent or worsening symptoms need rehabilitation assessment. This is general health education and does not replace an in-person medical assessment. | | | | | | |
| **Item / 项目** | | **Q1 / 问题1** | | **Q2 / 问题2** | | **Q3 / 问题3** |
| **Patient question (Chinese original) / 患者原始问题（中文）** | | 我产后排便时阴道口有压迫感，是盆底问题吗？ | | 盆底肌力差会不会影响以后性生活？ | | 盆底康复一定要在医院做吗？ |
| **English translation of patient question / 患者问题英文翻译** | | I feel pressure at the vaginal opening when having a bowel movement. Is this a pelvic floor problem? | | Can weak pelvic floor muscles affect future sexual life? | | Does pelvic floor rehabilitation have to be done in the hospital? |
| **Model response (Chinese) / 模型回答（中文）** | | 排便时阴道口压迫感可能与盆底肌松弛、阴道壁膨出或便秘用力有关，建议做盆底评估。建议增加饮水和膳食纤维，适量活动；若便血增多、腹胀明显或疼痛剧烈，应就医。 | | 盆底肌力差可能影响漏尿、下坠感和性生活舒适度，但多数可通过规范训练和康复改善。建议适量饮水、规律排尿；若尿痛加重、发热、腰痛或排尿困难持续，应查尿常规。 | | 轻度问题可在指导下居家训练，中重度或症状明显者建议到医院或康复门诊接受评估和治疗。训练应循序渐进，避免过早强度运动；若症状持续、加重或影响生活，应做康复评估。 |
| **English translation of model response / 模型回答英文翻译** | | Pressure at the vaginal opening during bowel movements may be related to pelvic floor weakness, vaginal wall bulging, or straining from constipation. Pelvic floor assessment is advised. Increase fluids, fiber, and gentle activity. More bleeding, marked bloating, or severe pain needs care. | | Weak pelvic floor muscles may affect leakage, heaviness, and comfort during sex, but many problems improve with proper training and rehabilitation. Increase exercises gradually and avoid early high intensity. Persistent or worsening symptoms need rehabilitation assessment. | | Mild cases may train at home with guidance. Moderate, severe, or symptomatic cases should be assessed and treated in a hospital or rehabilitation clinic. Increase exercises gradually and avoid early high intensity. Persistent or worsening symptoms need rehabilitation assessment. |
| **Overall evaluation item / 整体评价项目** | | | **Score / 分数** | | **Comments / 备注** | |
| **Patient Satisfaction / 患者满意度** | | | 5 | |  | |
| **Patient Understandability / 患者可理解性** | | | 4 | |  | |
| **Patient Usefulness / 患者实用性** | | | 4 | |  | |
| **Clinician Correctness / 医生评分：正确性** | | | 5 | |  | |
| **Clinician Completeness / 医生评分：完整性** | | | 5 | |  | |
| **Clinician Readability / 医生评分：可读性** | | | 4 | |  | |
| **Clinician Helpfulness / 医生评分：帮助性** | | | 5 | |  | |
| **Clinician Safety / 医生评分：安全性** | | | 5 | |  | |

**Patient 66**

| **Patient ID / 患者编号** | **Group / 模型组别** | | | | | |
| --- | --- | --- | --- | --- | --- | --- |
| P66 | ChatGPT 5.5 | | | | | |
| **General model response or patient education material (Chinese) / 模型总体回答或患者教育材料（中文）** | | | | | | |
| 哺乳期雌激素水平较低，阴道干涩较常见，也可能与疲劳、紧张和伤口恢复有关。重点是正确含接、有效排奶和休息补水；若红肿热痛、发热或24小时不缓解，应就医。同房疼痛时可以使用水溶性润滑剂，并放慢节奏，不要勉强继续。恢复前应确认恶露基本干净、伤口不疼，并做好避孕；若疼痛、出血或紧张明显，应咨询医生。若疼痛持续、伴出血、异味分泌物、瘙痒或会阴疤痕疼痛，应检查阴道炎、伤口瘢痕、盆底紧张等问题。建议记录颜色、量和气味，避免劳累；若1小时湿透卫生巾、血块增多、腹痛或发热，应就医。以上内容为一般健康教育，不能替代线下面诊。 | | | | | | |
| **English translation of the Chinese general response / 中文总体回答英文翻译** | | | | | | |
| Lower estrogen during breastfeeding commonly causes vaginal dryness. Fatigue, anxiety, and wound recovery can also contribute. Focus on effective milk removal and correct latch. Redness, fever, or no improvement within 24 hours needs care. A water-based lubricant may be used for painful intercourse. Go slowly and do not force continuation if pain occurs. Resume only when wounds and lochia are stable, and use contraception. Pain or bleeding needs medical advice. Persistent pain, bleeding, foul discharge, itching, or perineal scar pain should be evaluated for vaginitis, scar problems, pelvic floor tension, and other causes. Record color, amount, odor, and pad use. Seek care for heavy bleeding, clots, pain, or fever. This is general health education and does not replace an in-person medical assessment. | | | | | | |
| **Item / 项目** | | **Q1 / 问题1** | | **Q2 / 问题2** | | **Q3 / 问题3** |
| **Patient question (Chinese original) / 患者原始问题（中文）** | | 哺乳期阴道干涩很明显，是激素变化造成的吗？ | | 同房疼痛可以使用润滑剂吗？ | | 如果疼痛一直持续，需要检查哪些问题？ |
| **English translation of patient question / 患者问题英文翻译** | | Is obvious vaginal dryness during breastfeeding caused by hormonal changes? | | Can I use lubricant if intercourse is painful? | | If pain continues, what problems need to be checked? |
| **Model response (Chinese) / 模型回答（中文）** | | 哺乳期雌激素水平较低，阴道干涩较常见，也可能与疲劳、紧张和伤口恢复有关。重点是正确含接、有效排奶和休息补水；若红肿热痛、发热或24小时不缓解，应就医。 | | 同房疼痛时可以使用水溶性润滑剂，并放慢节奏，不要勉强继续。恢复前应确认恶露基本干净、伤口不疼，并做好避孕；若疼痛、出血或紧张明显，应咨询医生。 | | 若疼痛持续、伴出血、异味分泌物、瘙痒或会阴疤痕疼痛，应检查阴道炎、伤口瘢痕、盆底紧张等问题。建议记录颜色、量和气味，避免劳累；若1小时湿透卫生巾、血块增多、腹痛或发热，应就医。 |
| **English translation of model response / 模型回答英文翻译** | | Lower estrogen during breastfeeding commonly causes vaginal dryness. Fatigue, anxiety, and wound recovery can also contribute. Focus on effective milk removal and correct latch. Redness, fever, or no improvement within 24 hours needs care. | | A water-based lubricant may be used for painful intercourse. Go slowly and do not force continuation if pain occurs. Resume only when wounds and lochia are stable, and use contraception. Pain or bleeding needs medical advice. | | Persistent pain, bleeding, foul discharge, itching, or perineal scar pain should be evaluated for vaginitis, scar problems, pelvic floor tension, and other causes. Record color, amount, odor, and pad use. Seek care for heavy bleeding, clots, pain, or fever. |
| **Overall evaluation item / 整体评价项目** | | | **Score / 分数** | | **Comments / 备注** | |
| **Patient Satisfaction / 患者满意度** | | | 4 | |  | |
| **Patient Understandability / 患者可理解性** | | | 4 | |  | |
| **Patient Usefulness / 患者实用性** | | | 5 | |  | |
| **Clinician Correctness / 医生评分：正确性** | | | 5 | |  | |
| **Clinician Completeness / 医生评分：完整性** | | | 5 | |  | |
| **Clinician Readability / 医生评分：可读性** | | | 4 | |  | |
| **Clinician Helpfulness / 医生评分：帮助性** | | | 5 | |  | |
| **Clinician Safety / 医生评分：安全性** | | | 5 | |  | |

**Patient 67**

| **Patient ID / 患者编号** | **Group / 模型组别** | | | | | |
| --- | --- | --- | --- | --- | --- | --- |
| P67 | ChatGPT 5.5 | | | | | |
| **General model response or patient education material (Chinese) / 模型总体回答或患者教育材料（中文）** | | | | | | |
| 咳嗽或打喷嚏漏尿常见于压力性尿失禁，规范凯格尔训练通常有帮助。建议适量饮水、规律排尿；若尿痛加重、发热、腰痛或排尿困难持续，应查尿常规。训练次数应循序渐进，可从每天数组、每组数次开始，重点是动作正确和不憋气。活动要逐步增加，以不疼痛、不疲劳、恶露不增多为准；异常出血或腹痛时应停止并复查。若超过3个月仍明显漏尿、影响生活或伴尿急尿痛，应到盆底门诊评估，是否需要尿动力检查由医生决定。建议适量饮水、规律排尿；若尿痛加重、发热、腰痛或排尿困难持续，应查尿常规。以上内容为一般健康教育，不能替代线下面诊。 | | | | | | |
| **English translation of the Chinese general response / 中文总体回答英文翻译** | | | | | | |
| Leaking with coughing or sneezing is often stress urinary incontinence. Proper Kegel exercises can usually help. Drink fluids and urinate regularly. Worsening pain, fever, back pain, or persistent difficulty needs urine testing. Increase exercises gradually, starting with several sets per day and a few contractions per set. Correct technique and normal breathing are more important than high numbers. Increase activity gradually. Stop and seek review if bleeding, abdominal pain, dizziness, or incision pain occurs. If leakage remains obvious after three months, affects life, or comes with urgency or pain, visit a pelvic floor clinic. A doctor will decide whether urodynamic testing is needed. Drink fluids and urinate regularly. Worsening pain, fever, back pain, or persistent difficulty needs urine testing. This is general health education and does not replace an in-person medical assessment. | | | | | | |
| **Item / 项目** | | **Q1 / 问题1** | | **Q2 / 问题2** | | **Q3 / 问题3** |
| **Patient question (Chinese original) / 患者原始问题（中文）** | | 我咳嗽或打喷嚏会漏尿，自己练凯格尔有用吗？ | | 凯格尔运动每天做多少次合适？ | | 漏尿超过三个月还没好，需要做尿动力检查吗？ |
| **English translation of patient question / 患者问题英文翻译** | | I leak urine when coughing or sneezing. Will Kegel exercises help? | | How many Kegel exercises should I do each day? | | If leakage lasts more than three months, do I need urodynamic testing? |
| **Model response (Chinese) / 模型回答（中文）** | | 咳嗽或打喷嚏漏尿常见于压力性尿失禁，规范凯格尔训练通常有帮助。建议适量饮水、规律排尿；若尿痛加重、发热、腰痛或排尿困难持续，应查尿常规。 | | 训练次数应循序渐进，可从每天数组、每组数次开始，重点是动作正确和不憋气。活动要逐步增加，以不疼痛、不疲劳、恶露不增多为准；异常出血或腹痛时应停止并复查。 | | 若超过3个月仍明显漏尿、影响生活或伴尿急尿痛，应到盆底门诊评估，是否需要尿动力检查由医生决定。建议适量饮水、规律排尿；若尿痛加重、发热、腰痛或排尿困难持续，应查尿常规。 |
| **English translation of model response / 模型回答英文翻译** | | Leaking with coughing or sneezing is often stress urinary incontinence. Proper Kegel exercises can usually help. Drink fluids and urinate regularly. Worsening pain, fever, back pain, or persistent difficulty needs urine testing. | | Increase exercises gradually, starting with several sets per day and a few contractions per set. Correct technique and normal breathing are more important than high numbers. Increase activity gradually. Stop and seek review if bleeding, abdominal pain, dizziness, or incision pain occurs. | | If leakage remains obvious after three months, affects life, or comes with urgency or pain, visit a pelvic floor clinic. A doctor will decide whether urodynamic testing is needed. Drink fluids and urinate regularly. Worsening pain, fever, back pain, or persistent difficulty needs urine testing. |
| **Overall evaluation item / 整体评价项目** | | | **Score / 分数** | | **Comments / 备注** | |
| **Patient Satisfaction / 患者满意度** | | | 4 | |  | |
| **Patient Understandability / 患者可理解性** | | | 4 | |  | |
| **Patient Usefulness / 患者实用性** | | | 5 | |  | |
| **Clinician Correctness / 医生评分：正确性** | | | 5 | |  | |
| **Clinician Completeness / 医生评分：完整性** | | | 5 | |  | |
| **Clinician Readability / 医生评分：可读性** | | | 4 | |  | |
| **Clinician Helpfulness / 医生评分：帮助性** | | | 5 | |  | |
| **Clinician Safety / 医生评分：安全性** | | | 5 | |  | |

**Patient 68**

| **Patient ID / 患者编号** | **Group / 模型组别** | | | | | |
| --- | --- | --- | --- | --- | --- | --- |
| P68 | ChatGPT 5.5 | | | | | |
| **General model response or patient education material (Chinese) / 模型总体回答或患者教育材料（中文）** | | | | | | |
| 阴道口有东西鼓出可能是阴道壁膨出或盆腔脏器脱垂，需要通过妇科检查确认。训练应循序渐进，避免过早强度运动；若症状持续、加重或影响生活，应做康复评估。轻度脱垂常可通过体重管理、避免便秘和规范盆底康复改善症状。建议增加饮水和膳食纤维，适量活动；若便血增多、腹胀明显或疼痛剧烈，应就医。若下坠明显、组织脱出影响行走排尿排便，或康复效果不佳，医生可能建议子宫托或进一步治疗。建议适量饮水、规律排尿；若尿痛加重、发热、腰痛或排尿困难持续，应查尿常规。以上内容为一般健康教育，不能替代线下面诊。 | | | | | | |
| **English translation of the Chinese general response / 中文总体回答英文翻译** | | | | | | |
| A bulge at the vaginal opening may be vaginal wall bulging or pelvic organ prolapse and should be confirmed by gynecologic examination. Increase exercises gradually and avoid early high intensity. Persistent or worsening symptoms need rehabilitation assessment. Mild prolapse symptoms may improve with weight management, avoiding constipation, and structured pelvic floor rehabilitation. Record color, amount, odor, and pad use. Seek care for heavy bleeding, clots, pain, or fever. If heaviness is obvious, tissue protrusion affects walking, urination, or bowel movements, or rehabilitation is insufficient, a pessary or further treatment may be advised. Drink fluids and urinate regularly. Worsening pain, fever, back pain, or persistent difficulty needs urine testing. This is general health education and does not replace an in-person medical assessment. | | | | | | |
| **Item / 项目** | | **Q1 / 问题1** | | **Q2 / 问题2** | | **Q3 / 问题3** |
| **Patient question (Chinese original) / 患者原始问题（中文）** | | 我感觉阴道口有东西鼓出来，是不是脱垂？ | | 轻度脱垂可以通过盆底康复恢复吗？ | | 什么情况下需要使用子宫托或进一步治疗？ |
| **English translation of patient question / 患者问题英文翻译** | | I feel something bulging at the vaginal opening. Could it be prolapse? | | Can mild prolapse improve with pelvic floor rehabilitation? | | When is a pessary or further treatment needed? |
| **Model response (Chinese) / 模型回答（中文）** | | 阴道口有东西鼓出可能是阴道壁膨出或盆腔脏器脱垂，需要通过妇科检查确认。训练应循序渐进，避免过早强度运动；若症状持续、加重或影响生活，应做康复评估。 | | 轻度脱垂常可通过体重管理、避免便秘和规范盆底康复改善症状。建议增加饮水和膳食纤维，适量活动；若便血增多、腹胀明显或疼痛剧烈，应就医。 | | 若下坠明显、组织脱出影响行走排尿排便，或康复效果不佳，医生可能建议子宫托或进一步治疗。建议适量饮水、规律排尿；若尿痛加重、发热、腰痛或排尿困难持续，应查尿常规。 |
| **English translation of model response / 模型回答英文翻译** | | A bulge at the vaginal opening may be vaginal wall bulging or pelvic organ prolapse and should be confirmed by gynecologic examination. Increase exercises gradually and avoid early high intensity. Persistent or worsening symptoms need rehabilitation assessment. | | Mild prolapse symptoms may improve with weight management, avoiding constipation, and structured pelvic floor rehabilitation. Record color, amount, odor, and pad use. Seek care for heavy bleeding, clots, pain, or fever. | | If heaviness is obvious, tissue protrusion affects walking, urination, or bowel movements, or rehabilitation is insufficient, a pessary or further treatment may be advised. Drink fluids and urinate regularly. Worsening pain, fever, back pain, or persistent difficulty needs urine testing. |
| **Overall evaluation item / 整体评价项目** | | | **Score / 分数** | | **Comments / 备注** | |
| **Patient Satisfaction / 患者满意度** | | | 5 | |  | |
| **Patient Understandability / 患者可理解性** | | | 4 | |  | |
| **Patient Usefulness / 患者实用性** | | | 4 | |  | |
| **Clinician Correctness / 医生评分：正确性** | | | 4 | |  | |
| **Clinician Completeness / 医生评分：完整性** | | | 5 | |  | |
| **Clinician Readability / 医生评分：可读性** | | | 4 | |  | |
| **Clinician Helpfulness / 医生评分：帮助性** | | | 5 | |  | |
| **Clinician Safety / 医生评分：安全性** | | | 5 | |  | |

**Patient 69**

| **Patient ID / 患者编号** | **Group / 模型组别** | | | | | |
| --- | --- | --- | --- | --- | --- | --- |
| P69 | ChatGPT 5.5 | | | | | |
| **General model response or patient education material (Chinese) / 模型总体回答或患者教育材料（中文）** | | | | | | |
| 侧切疤痕早期发硬较常见，随着瘢痕成熟，很多人会逐渐变软。保持局部清洁干燥，避免抓挠、挤压和自行涂药；若红肿扩大、疼痛或渗液加重，应复查。疤痕紧绷可能影响以后同房舒适度，可在伤口完全愈合后接受专业指导做疤痕护理。保持局部清洁干燥，避免抓挠、挤压和自行涂药；若红肿扩大、疼痛或渗液加重，应复查。若按压很痛、局部红肿、出血、分泌物或持续牵拉明显，应复查，排除感染、肉芽或瘢痕异常。建议记录颜色、量和气味，避免劳累；若1小时湿透卫生巾、血块增多、腹痛或发热，应就医。以上内容为一般健康教育，不能替代线下面诊。 | | | | | | |
| **English translation of the Chinese general response / 中文总体回答英文翻译** | | | | | | |
| A firm episiotomy scar is common early after healing. Many scars gradually soften as they mature. Keep the area clean and dry. Seek review if redness, pain, drainage, or wound opening worsens. A tight scar may affect comfort during intercourse later. After complete healing, scar care can be done with professional guidance. Keep the area clean and dry. Seek review if redness, pain, drainage, or wound opening worsens. Marked tenderness, redness, swelling, bleeding, discharge, or persistent pulling should be reviewed to rule out infection, granulation tissue, or abnormal scarring. Record color, amount, odor, and pad use. Seek care for heavy bleeding, clots, pain, or fever. This is general health education and does not replace an in-person medical assessment. | | | | | | |
| **Item / 项目** | | **Q1 / 问题1** | | **Q2 / 问题2** | | **Q3 / 问题3** |
| **Patient question (Chinese original) / 患者原始问题（中文）** | | 侧切疤痕摸着硬硬的，会慢慢变软吗？ | | 会阴疤痕紧绷会不会影响以后同房？ | | 疤痕按压很痛时需要复查吗？ |
| **English translation of patient question / 患者问题英文翻译** | | My episiotomy scar feels hard. Will it soften over time? | | Can a tight perineal scar affect intercourse later? | | If pressing the scar is very painful, do I need a recheck? |
| **Model response (Chinese) / 模型回答（中文）** | | 侧切疤痕早期发硬较常见，随着瘢痕成熟，很多人会逐渐变软。保持局部清洁干燥，避免抓挠、挤压和自行涂药；若红肿扩大、疼痛或渗液加重，应复查。 | | 疤痕紧绷可能影响以后同房舒适度，可在伤口完全愈合后接受专业指导做疤痕护理。保持局部清洁干燥，避免抓挠、挤压和自行涂药；若红肿扩大、疼痛或渗液加重，应复查。 | | 若按压很痛、局部红肿、出血、分泌物或持续牵拉明显，应复查，排除感染、肉芽或瘢痕异常。建议记录颜色、量和气味，避免劳累；若1小时湿透卫生巾、血块增多、腹痛或发热，应就医。 |
| **English translation of model response / 模型回答英文翻译** | | A firm episiotomy scar is common early after healing. Many scars gradually soften as they mature. Keep the area clean and dry. Seek review if redness, pain, drainage, or wound opening worsens. | | A tight scar may affect comfort during intercourse later. After complete healing, scar care can be done with professional guidance. Keep the area clean and dry. Seek review if redness, pain, drainage, or wound opening worsens. | | Marked tenderness, redness, swelling, bleeding, discharge, or persistent pulling should be reviewed to rule out infection, granulation tissue, or abnormal scarring. Record color, amount, odor, and pad use. Seek care for heavy bleeding, clots, pain, or fever. |
| **Overall evaluation item / 整体评价项目** | | | **Score / 分数** | | **Comments / 备注** | |
| **Patient Satisfaction / 患者满意度** | | | 4 | |  | |
| **Patient Understandability / 患者可理解性** | | | 4 | |  | |
| **Patient Usefulness / 患者实用性** | | | 4 | |  | |
| **Clinician Correctness / 医生评分：正确性** | | | 5 | |  | |
| **Clinician Completeness / 医生评分：完整性** | | | 5 | |  | |
| **Clinician Readability / 医生评分：可读性** | | | 4 | |  | |
| **Clinician Helpfulness / 医生评分：帮助性** | | | 5 | |  | |
| **Clinician Safety / 医生评分：安全性** | | | 5 | |  | |

**Patient 70**

| **Patient ID / 患者编号** | **Group / 模型组别** | | | | | |
| --- | --- | --- | --- | --- | --- | --- |
| P70 | ChatGPT 5.5 | | | | | |
| **General model response or patient education material (Chinese) / 模型总体回答或患者教育材料（中文）** | | | | | | |
| 剖宫产后咳嗽时切口疼多与腹压升高牵拉伤口有关，早期较常见。保持局部清洁干燥，避免抓挠、挤压和自行涂药；若红肿扩大、疼痛或渗液加重，应复查。咳嗽或起身时可用手或枕头轻托腹部，抱宝宝时让宝宝靠近身体，避免突然扭转和单手用力。保持局部清洁干燥，避免抓挠、挤压和自行涂药；若红肿扩大、疼痛或渗液加重，应复查。若突然撕裂样疼痛、切口裂开、渗血渗液、鼓包或发热，应及时就医。建议复测体温，并观察恶露、乳房、尿痛和伤口；若体温≥38℃、寒战或精神差，应就医。以上内容为一般健康教育，不能替代线下面诊。 | | | | | | |
| **English translation of the Chinese general response / 中文总体回答英文翻译** | | | | | | |
| Incision pain when coughing after a cesarean birth is often caused by increased abdominal pressure pulling the wound and is common early on. Keep the area clean and dry. Seek review if redness, pain, drainage, or wound opening worsens. Support the abdomen with your hand or a pillow when coughing or getting up. Hold the baby close to your body and avoid sudden twisting or one-handed strain. Keep the area clean and dry. Seek review if redness, pain, drainage, or wound opening worsens. Sudden tearing pain, wound opening, bleeding or drainage, a bulge, or fever requires prompt medical care. Record color, amount, odor, and pad use. Seek care for heavy bleeding, clots, pain, or fever. This is general health education and does not replace an in-person medical assessment. | | | | | | |
| **Item / 项目** | | **Q1 / 问题1** | | **Q2 / 问题2** | | **Q3 / 问题3** |
| **Patient question (Chinese original) / 患者原始问题（中文）** | | 剖宫产后咳嗽时切口疼，正常吗？ | | 抱宝宝时怎样避免牵拉腹部切口？ | | 如果切口突然有撕裂样疼痛，需要马上就医吗？ |
| **English translation of patient question / 患者问题英文翻译** | | My cesarean incision hurts when I cough. Is this normal? | | How can I avoid pulling the abdominal incision when holding the baby? | | If the incision suddenly has tearing pain, do I need urgent care? |
| **Model response (Chinese) / 模型回答（中文）** | | 剖宫产后咳嗽时切口疼多与腹压升高牵拉伤口有关，早期较常见。保持局部清洁干燥，避免抓挠、挤压和自行涂药；若红肿扩大、疼痛或渗液加重，应复查。 | | 咳嗽或起身时可用手或枕头轻托腹部，抱宝宝时让宝宝靠近身体，避免突然扭转和单手用力。保持局部清洁干燥，避免抓挠、挤压和自行涂药；若红肿扩大、疼痛或渗液加重，应复查。 | | 若突然撕裂样疼痛、切口裂开、渗血渗液、鼓包或发热，应及时就医。建议复测体温，并观察恶露、乳房、尿痛和伤口；若体温≥38℃、寒战或精神差，应就医。 |
| **English translation of model response / 模型回答英文翻译** | | Incision pain when coughing after a cesarean birth is often caused by increased abdominal pressure pulling the wound and is common early on. Keep the area clean and dry. Seek review if redness, pain, drainage, or wound opening worsens. | | Support the abdomen with your hand or a pillow when coughing or getting up. Hold the baby close to your body and avoid sudden twisting or one-handed strain. Keep the area clean and dry. Seek review if redness, pain, drainage, or wound opening worsens. | | Sudden tearing pain, wound opening, bleeding or drainage, a bulge, or fever requires prompt medical care. Record color, amount, odor, and pad use. Seek care for heavy bleeding, clots, pain, or fever. |
| **Overall evaluation item / 整体评价项目** | | | **Score / 分数** | | **Comments / 备注** | |
| **Patient Satisfaction / 患者满意度** | | | 4 | |  | |
| **Patient Understandability / 患者可理解性** | | | 4 | |  | |
| **Patient Usefulness / 患者实用性** | | | 4 | |  | |
| **Clinician Correctness / 医生评分：正确性** | | | 5 | |  | |
| **Clinician Completeness / 医生评分：完整性** | | | 4 | |  | |
| **Clinician Readability / 医生评分：可读性** | | | 4 | |  | |
| **Clinician Helpfulness / 医生评分：帮助性** | | | 4 | |  | |
| **Clinician Safety / 医生评分：安全性** | | | 4 | |  | |

**Patient 71**

| **Patient ID / 患者编号** | **Group / 模型组别** | | | | | |
| --- | --- | --- | --- | --- | --- | --- |
| P71 | ChatGPT 5.5 | | | | | |
| **General model response or patient education material (Chinese) / 模型总体回答或患者教育材料（中文）** | | | | | | |
| 产后反复低烧不能只当作感冒或劳累，应记录体温并观察恶露、乳房、尿痛和伤口情况。建议记录颜色、量和气味，避免劳累；若1小时湿透卫生巾、血块增多、腹痛或发热，应就医。乳房胀痛、堵奶或乳腺炎可引起发热，若伴红肿热痛更需要重视。建议复测体温，并观察恶露、乳房、尿痛和伤口；若体温≥38℃、寒战或精神差，应就医。体温达到38℃及以上，或低烧伴寒战、恶露臭味、下腹痛、乳房红肿、切口渗液，应尽快就医。建议记录颜色、量和气味，避免劳累；若1小时湿透卫生巾、血块增多、腹痛或发热，应就医。以上内容为一般健康教育，不能替代线下面诊。 | | | | | | |
| **English translation of the Chinese general response / 中文总体回答英文翻译** | | | | | | |
| Recurrent low-grade fever after birth should not be dismissed as fatigue or a cold. Record temperature and observe lochia, breasts, urinary symptoms, and wounds. Record color, amount, odor, and pad use. Seek care for heavy bleeding, clots, pain, or fever. Breast engorgement, blocked milk flow, or mastitis can cause fever, especially when redness, warmth, and pain are present. Record color, amount, odor, and pad use. Seek care for heavy bleeding, clots, pain, or fever. A temperature of 38°C or higher, or low fever with chills, foul lochia, lower abdominal pain, breast redness, or incision drainage, needs prompt medical care. Record color, amount, odor, and pad use. Seek care for heavy bleeding, clots, pain, or fever. This is general health education and does not replace an in-person medical assessment. | | | | | | |
| **Item / 项目** | | **Q1 / 问题1** | | **Q2 / 问题2** | | **Q3 / 问题3** |
| **Patient question (Chinese original) / 患者原始问题（中文）** | | 产后低烧反反复复，但没有感冒症状，怎么办？ | | 低烧和乳房胀痛有关吗？ | | 产后发热到多少度需要尽快去医院？ |
| **English translation of patient question / 患者问题英文翻译** | | I have recurrent low-grade fever after birth without cold symptoms. What should I do? | | Can low fever be related to breast engorgement? | | At what temperature should postpartum fever be seen urgently? |
| **Model response (Chinese) / 模型回答（中文）** | | 产后反复低烧不能只当作感冒或劳累，应记录体温并观察恶露、乳房、尿痛和伤口情况。建议记录颜色、量和气味，避免劳累；若1小时湿透卫生巾、血块增多、腹痛或发热，应就医。 | | 乳房胀痛、堵奶或乳腺炎可引起发热，若伴红肿热痛更需要重视。建议复测体温，并观察恶露、乳房、尿痛和伤口；若体温≥38℃、寒战或精神差，应就医。 | | 体温达到38℃及以上，或低烧伴寒战、恶露臭味、下腹痛、乳房红肿、切口渗液，应尽快就医。建议记录颜色、量和气味，避免劳累；若1小时湿透卫生巾、血块增多、腹痛或发热，应就医。 |
| **English translation of model response / 模型回答英文翻译** | | Recurrent low-grade fever after birth should not be dismissed as fatigue or a cold. Record temperature and observe lochia, breasts, urinary symptoms, and wounds. Record color, amount, odor, and pad use. Seek care for heavy bleeding, clots, pain, or fever. | | Breast engorgement, blocked milk flow, or mastitis can cause fever, especially when redness, warmth, and pain are present. Record color, amount, odor, and pad use. Seek care for heavy bleeding, clots, pain, or fever. | | A temperature of 38°C or higher, or low fever with chills, foul lochia, lower abdominal pain, breast redness, or incision drainage, needs prompt medical care. Record color, amount, odor, and pad use. Seek care for heavy bleeding, clots, pain, or fever. |
| **Overall evaluation item / 整体评价项目** | | | **Score / 分数** | | **Comments / 备注** | |
| **Patient Satisfaction / 患者满意度** | | | 4 | |  | |
| **Patient Understandability / 患者可理解性** | | | 4 | |  | |
| **Patient Usefulness / 患者实用性** | | | 4 | |  | |
| **Clinician Correctness / 医生评分：正确性** | | | 5 | |  | |
| **Clinician Completeness / 医生评分：完整性** | | | 5 | |  | |
| **Clinician Readability / 医生评分：可读性** | | | 4 | |  | |
| **Clinician Helpfulness / 医生评分：帮助性** | | | 5 | |  | |
| **Clinician Safety / 医生评分：安全性** | | | 5 | |  | |

**Patient 72**

| **Patient ID / 患者编号** | **Group / 模型组别** | | | | | |
| --- | --- | --- | --- | --- | --- | --- |
| P72 | ChatGPT 5.5 | | | | | |
| **General model response or patient education material (Chinese) / 模型总体回答或患者教育材料（中文）** | | | | | | |
| 下腹压痛伴恶露异味需警惕子宫内膜感染或宫腔残留，应尽快到产科复查。建议记录颜色、量和气味，避免劳累；若1小时湿透卫生巾、血块增多、腹痛或发热，应就医。子宫复旧不良可表现为恶露持续鲜红、量多、血块、下腹痛、子宫较大或压痛。建议记录颜色、量和气味，避免劳累；若1小时湿透卫生巾、血块增多、腹痛或发热，应就医。多数抗感染药物可选择哺乳期相对安全方案，不要自行停奶，应主动告诉医生正在哺乳。建议复测体温，并观察恶露、乳房、尿痛和伤口；若体温≥38℃、寒战或精神差，应就医。以上内容为一般健康教育，不能替代线下面诊。 | | | | | | |
| **English translation of the Chinese general response / 中文总体回答英文翻译** | | | | | | |
| Lower abdominal tenderness with foul-smelling lochia may suggest uterine infection or retained tissue and should be reviewed promptly by obstetrics. Record color, amount, odor, and pad use. Seek care for heavy bleeding, clots, pain, or fever. Poor uterine involution may cause persistent bright-red lochia, heavy bleeding, clots, lower abdominal pain, an enlarged uterus, or tenderness. Record color, amount, odor, and pad use. Seek care for heavy bleeding, clots, pain, or fever. Many anti-infection medicines can be selected to be compatible with breastfeeding. Do not stop breastfeeding by yourself, but tell the doctor you are breastfeeding. Recheck temperature and watch lochia, breasts, urination, and wounds. Fever at or above 38°C needs care. This is general health education and does not replace an in-person medical assessment. | | | | | | |
| **Item / 项目** | | **Q1 / 问题1** | | **Q2 / 问题2** | | **Q3 / 问题3** |
| **Patient question (Chinese original) / 患者原始问题（中文）** | | 我产后下腹按压疼，恶露也有点味道，是感染吗？ | | 子宫复旧不良会有哪些表现？ | | 如果医生说需要抗感染治疗，还能哺乳吗？ |
| **English translation of patient question / 患者问题英文翻译** | | My lower abdomen is tender after birth and the lochia has some odor. Is it infection? | | What are signs of poor uterine involution? | | If the doctor says I need anti-infection treatment, can I still breastfeed? |
| **Model response (Chinese) / 模型回答（中文）** | | 下腹压痛伴恶露异味需警惕子宫内膜感染或宫腔残留，应尽快到产科复查。建议记录颜色、量和气味，避免劳累；若1小时湿透卫生巾、血块增多、腹痛或发热，应就医。 | | 子宫复旧不良可表现为恶露持续鲜红、量多、血块、下腹痛、子宫较大或压痛。建议记录颜色、量和气味，避免劳累；若1小时湿透卫生巾、血块增多、腹痛或发热，应就医。 | | 多数抗感染药物可选择哺乳期相对安全方案，不要自行停奶，应主动告诉医生正在哺乳。建议复测体温，并观察恶露、乳房、尿痛和伤口；若体温≥38℃、寒战或精神差，应就医。 |
| **English translation of model response / 模型回答英文翻译** | | Lower abdominal tenderness with foul-smelling lochia may suggest uterine infection or retained tissue and should be reviewed promptly by obstetrics. Record color, amount, odor, and pad use. Seek care for heavy bleeding, clots, pain, or fever. | | Poor uterine involution may cause persistent bright-red lochia, heavy bleeding, clots, lower abdominal pain, an enlarged uterus, or tenderness. Record color, amount, odor, and pad use. Seek care for heavy bleeding, clots, pain, or fever. | | Many anti-infection medicines can be selected to be compatible with breastfeeding. Do not stop breastfeeding by yourself, but tell the doctor you are breastfeeding. Recheck temperature and watch lochia, breasts, urination, and wounds. Fever at or above 38°C needs care. |
| **Overall evaluation item / 整体评价项目** | | | **Score / 分数** | | **Comments / 备注** | |
| **Patient Satisfaction / 患者满意度** | | | 4 | |  | |
| **Patient Understandability / 患者可理解性** | | | 4 | |  | |
| **Patient Usefulness / 患者实用性** | | | 5 | |  | |
| **Clinician Correctness / 医生评分：正确性** | | | 4 | |  | |
| **Clinician Completeness / 医生评分：完整性** | | | 5 | |  | |
| **Clinician Readability / 医生评分：可读性** | | | 4 | |  | |
| **Clinician Helpfulness / 医生评分：帮助性** | | | 5 | |  | |
| **Clinician Safety / 医生评分：安全性** | | | 5 | |  | |

**Patient 73**

| **Patient ID / 患者编号** | **Group / 模型组别** | | | | | |
| --- | --- | --- | --- | --- | --- | --- |
| P73 | ChatGPT 5.5 | | | | | |
| **General model response or patient education material (Chinese) / 模型总体回答或患者教育材料（中文）** | | | | | | |
| 会阴伤口轻微裂开能否自行愈合，要看裂开深度、疼痛、感染和出血情况，建议复查。建议记录颜色、量和气味，避免劳累；若1小时湿透卫生巾、血块增多、腹痛或发热，应就医。伤口裂开后不建议自行坐浴或使用消毒液，先保持清洁干燥并听从医生处理意见。保持局部清洁干燥，避免抓挠、挤压和自行涂药；若红肿扩大、疼痛或渗液加重，应复查。若出血、流脓、异味、疼痛加重或裂口扩大，需及时就医，是否重新缝合由医生检查后决定。建议记录颜色、量和气味，避免劳累；若1小时湿透卫生巾、血块增多、腹痛或发热，应就医。以上内容为一般健康教育，不能替代线下面诊。 | | | | | | |
| **English translation of the Chinese general response / 中文总体回答英文翻译** | | | | | | |
| Whether a slightly opened perineal wound can heal by itself depends on depth, pain, infection, and bleeding. A recheck is recommended. Record color, amount, odor, and pad use. Seek care for heavy bleeding, clots, pain, or fever. After a wound opens, do not start sitz baths or disinfectants by yourself. Keep it clean and dry and follow medical advice. Keep the area clean and dry. Seek review if redness, pain, drainage, or wound opening worsens. Bleeding, pus, foul odor, worsening pain, or a larger opening requires medical care. A clinician will decide whether re-suturing is needed. Record color, amount, odor, and pad use. Seek care for heavy bleeding, clots, pain, or fever. This is general health education and does not replace an in-person medical assessment. | | | | | | |
| **Item / 项目** | | **Q1 / 问题1** | | **Q2 / 问题2** | | **Q3 / 问题3** |
| **Patient question (Chinese original) / 患者原始问题（中文）** | | 会阴伤口裂开一点点，会自己长好吗？ | | 伤口裂开后还能用坐浴吗？ | | 如果伤口出血或流脓，需要重新缝合吗？ |
| **English translation of patient question / 患者问题英文翻译** | | My perineal wound has opened a little. Will it heal by itself? | | Can I use a sitz bath after the wound opens? | | If the wound bleeds or drains pus, does it need to be stitched again? |
| **Model response (Chinese) / 模型回答（中文）** | | 会阴伤口轻微裂开能否自行愈合，要看裂开深度、疼痛、感染和出血情况，建议复查。建议记录颜色、量和气味，避免劳累；若1小时湿透卫生巾、血块增多、腹痛或发热，应就医。 | | 伤口裂开后不建议自行坐浴或使用消毒液，先保持清洁干燥并听从医生处理意见。保持局部清洁干燥，避免抓挠、挤压和自行涂药；若红肿扩大、疼痛或渗液加重，应复查。 | | 若出血、流脓、异味、疼痛加重或裂口扩大，需及时就医，是否重新缝合由医生检查后决定。建议记录颜色、量和气味，避免劳累；若1小时湿透卫生巾、血块增多、腹痛或发热，应就医。 |
| **English translation of model response / 模型回答英文翻译** | | Whether a slightly opened perineal wound can heal by itself depends on depth, pain, infection, and bleeding. A recheck is recommended. Record color, amount, odor, and pad use. Seek care for heavy bleeding, clots, pain, or fever. | | After a wound opens, do not start sitz baths or disinfectants by yourself. Keep it clean and dry and follow medical advice. Keep the area clean and dry. Seek review if redness, pain, drainage, or wound opening worsens. | | Bleeding, pus, foul odor, worsening pain, or a larger opening requires medical care. A clinician will decide whether re-suturing is needed. Record color, amount, odor, and pad use. Seek care for heavy bleeding, clots, pain, or fever. |
| **Overall evaluation item / 整体评价项目** | | | **Score / 分数** | | **Comments / 备注** | |
| **Patient Satisfaction / 患者满意度** | | | 4 | |  | |
| **Patient Understandability / 患者可理解性** | | | 4 | |  | |
| **Patient Usefulness / 患者实用性** | | | 4 | |  | |
| **Clinician Correctness / 医生评分：正确性** | | | 5 | |  | |
| **Clinician Completeness / 医生评分：完整性** | | | 5 | |  | |
| **Clinician Readability / 医生评分：可读性** | | | 4 | |  | |
| **Clinician Helpfulness / 医生评分：帮助性** | | | 5 | |  | |
| **Clinician Safety / 医生评分：安全性** | | | 5 | |  | |

**Patient 74**

| **Patient ID / 患者编号** | **Group / 模型组别** | | | | | |
| --- | --- | --- | --- | --- | --- | --- |
| P74 | ChatGPT 5.5 | | | | | |
| **General model response or patient education material (Chinese) / 模型总体回答或患者教育材料（中文）** | | | | | | |
| 切口边缘发红发热可能是感染、摩擦刺激或皮肤反应，需要结合疼痛、渗液和发热判断。建议复测体温，并观察恶露、乳房、尿痛和伤口；若体温≥38℃、寒战或精神差，应就医。不建议长期自行每天反复用碘伏消毒，以免刺激皮肤，切口护理应按医院指导执行。保持局部清洁干燥，避免抓挠、挤压和自行涂药；若红肿扩大、疼痛或渗液加重，应复查。若流黄色液体、脓液、异味、红肿扩大、疼痛加重或发热，应尽快复查。建议复测体温，并观察恶露、乳房、尿痛和伤口；若体温≥38℃、寒战或精神差，应就医。以上内容为一般健康教育，不能替代线下面诊。 | | | | | | |
| **English translation of the Chinese general response / 中文总体回答英文翻译** | | | | | | |
| Redness and warmth at the incision edge may be infection, friction irritation, or a skin reaction. Pain, drainage, and fever should be considered. Record color, amount, odor, and pad use. Seek care for heavy bleeding, clots, pain, or fever. Repeated daily iodine disinfection by yourself is not recommended because it may irritate the skin. Follow the wound-care instructions from the hospital. Recheck temperature and watch lochia, breasts, urination, and wounds. Fever at or above 38°C needs care. Yellow fluid, pus, odor, spreading redness, worsening pain, or fever requires prompt recheck. Record color, amount, odor, and pad use. Seek care for heavy bleeding, clots, pain, or fever. This is general health education and does not replace an in-person medical assessment. | | | | | | |
| **Item / 项目** | | **Q1 / 问题1** | | **Q2 / 问题2** | | **Q3 / 问题3** |
| **Patient question (Chinese original) / 患者原始问题（中文）** | | 剖宫产切口边缘发红发热，是不是感染？ | | 切口消毒可以每天用碘伏吗？ | | 如果切口流黄色液体，需要马上复查吗？ |
| **English translation of patient question / 患者问题英文翻译** | | The edge of my cesarean incision is red and warm. Is it infected? | | Can I disinfect the incision with iodine every day? | | If yellow fluid drains from the incision, do I need an immediate recheck? |
| **Model response (Chinese) / 模型回答（中文）** | | 切口边缘发红发热可能是感染、摩擦刺激或皮肤反应，需要结合疼痛、渗液和发热判断。建议复测体温，并观察恶露、乳房、尿痛和伤口；若体温≥38℃、寒战或精神差，应就医。 | | 不建议长期自行每天反复用碘伏消毒，以免刺激皮肤，切口护理应按医院指导执行。保持局部清洁干燥，避免抓挠、挤压和自行涂药；若红肿扩大、疼痛或渗液加重，应复查。 | | 若流黄色液体、脓液、异味、红肿扩大、疼痛加重或发热，应尽快复查。建议复测体温，并观察恶露、乳房、尿痛和伤口；若体温≥38℃、寒战或精神差，应就医。 |
| **English translation of model response / 模型回答英文翻译** | | Redness and warmth at the incision edge may be infection, friction irritation, or a skin reaction. Pain, drainage, and fever should be considered. Record color, amount, odor, and pad use. Seek care for heavy bleeding, clots, pain, or fever. | | Repeated daily iodine disinfection by yourself is not recommended because it may irritate the skin. Follow the wound-care instructions from the hospital. Recheck temperature and watch lochia, breasts, urination, and wounds. Fever at or above 38°C needs care. | | Yellow fluid, pus, odor, spreading redness, worsening pain, or fever requires prompt recheck. Record color, amount, odor, and pad use. Seek care for heavy bleeding, clots, pain, or fever. |
| **Overall evaluation item / 整体评价项目** | | | **Score / 分数** | | **Comments / 备注** | |
| **Patient Satisfaction / 患者满意度** | | | 4 | |  | |
| **Patient Understandability / 患者可理解性** | | | 4 | |  | |
| **Patient Usefulness / 患者实用性** | | | 4 | |  | |
| **Clinician Correctness / 医生评分：正确性** | | | 5 | |  | |
| **Clinician Completeness / 医生评分：完整性** | | | 4 | |  | |
| **Clinician Readability / 医生评分：可读性** | | | 4 | |  | |
| **Clinician Helpfulness / 医生评分：帮助性** | | | 4 | |  | |
| **Clinician Safety / 医生评分：安全性** | | | 5 | |  | |

**Patient 75**

| **Patient ID / 患者编号** | **Group / 模型组别** | | | | | |
| --- | --- | --- | --- | --- | --- | --- |
| P75 | ChatGPT 5.5 | | | | | |
| **General model response or patient education material (Chinese) / 模型总体回答或患者教育材料（中文）** | | | | | | |
| 切口下硬块可能是瘢痕、线结反应、血肿或积液，单靠触摸难以判断。保持局部清洁干燥，避免抓挠、挤压和自行涂药；若红肿扩大、疼痛或渗液加重，应复查。鼓包不疼也应观察大小、颜色、温度和是否随咳嗽突出，避免按压揉搓。保持局部清洁干燥，避免抓挠、挤压和自行涂药；若红肿扩大、疼痛或渗液加重，应复查。若硬块变大、发热、疼痛、发红、渗液或伴发热，应复查，医生可能安排超声排查血肿、积液或疝。建议复测体温，并观察恶露、乳房、尿痛和伤口；若体温≥38℃、寒战或精神差，应就医。以上内容为一般健康教育，不能替代线下面诊。 | | | | | | |
| **English translation of the Chinese general response / 中文总体回答英文翻译** | | | | | | |
| A hard lump under a cesarean incision may be scar tissue, suture reaction, hematoma, or fluid collection. Touch alone cannot confirm the cause. Keep the area clean and dry. Seek review if redness, pain, drainage, or wound opening worsens. Even if the bulge is not painful, observe its size, color, warmth, and whether it protrudes when coughing. Avoid pressing or rubbing it. Keep the area clean and dry. Seek review if redness, pain, drainage, or wound opening worsens. If it grows, becomes painful, red, warm, drains fluid, or fever occurs, seek review. Ultrasound may be used to check for hematoma, fluid, or hernia. Record color, amount, odor, and pad use. Seek care for heavy bleeding, clots, pain, or fever. This is general health education and does not replace an in-person medical assessment. | | | | | | |
| **Item / 项目** | | **Q1 / 问题1** | | **Q2 / 问题2** | | **Q3 / 问题3** |
| **Patient question (Chinese original) / 患者原始问题（中文）** | | 剖宫产切口下面有硬块，是血肿还是正常瘢痕？ | | 切口附近有鼓包，按着不太疼，需要观察吗？ | | 如果硬块变大或发热，需要做超声吗？ |
| **English translation of patient question / 患者问题英文翻译** | | There is a hard lump under my cesarean incision. Is it a hematoma or normal scar tissue? | | There is a bulge near the incision but it is not very painful. Can I observe it? | | If the lump gets bigger or I have fever, do I need an ultrasound? |
| **Model response (Chinese) / 模型回答（中文）** | | 切口下硬块可能是瘢痕、线结反应、血肿或积液，单靠触摸难以判断。保持局部清洁干燥，避免抓挠、挤压和自行涂药；若红肿扩大、疼痛或渗液加重，应复查。 | | 鼓包不疼也应观察大小、颜色、温度和是否随咳嗽突出，避免按压揉搓。保持局部清洁干燥，避免抓挠、挤压和自行涂药；若红肿扩大、疼痛或渗液加重，应复查。 | | 若硬块变大、发热、疼痛、发红、渗液或伴发热，应复查，医生可能安排超声排查血肿、积液或疝。建议复测体温，并观察恶露、乳房、尿痛和伤口；若体温≥38℃、寒战或精神差，应就医。 |
| **English translation of model response / 模型回答英文翻译** | | A hard lump under a cesarean incision may be scar tissue, suture reaction, hematoma, or fluid collection. Touch alone cannot confirm the cause. Keep the area clean and dry. Seek review if redness, pain, drainage, or wound opening worsens. | | Even if the bulge is not painful, observe its size, color, warmth, and whether it protrudes when coughing. Avoid pressing or rubbing it. Keep the area clean and dry. Seek review if redness, pain, drainage, or wound opening worsens. | | If it grows, becomes painful, red, warm, drains fluid, or fever occurs, seek review. Ultrasound may be used to check for hematoma, fluid, or hernia. Record color, amount, odor, and pad use. Seek care for heavy bleeding, clots, pain, or fever. |
| **Overall evaluation item / 整体评价项目** | | | **Score / 分数** | | **Comments / 备注** | |
| **Patient Satisfaction / 患者满意度** | | | 4 | |  | |
| **Patient Understandability / 患者可理解性** | | | 4 | |  | |
| **Patient Usefulness / 患者实用性** | | | 4 | |  | |
| **Clinician Correctness / 医生评分：正确性** | | | 5 | |  | |
| **Clinician Completeness / 医生评分：完整性** | | | 4 | |  | |
| **Clinician Readability / 医生评分：可读性** | | | 4 | |  | |
| **Clinician Helpfulness / 医生评分：帮助性** | | | 4 | |  | |
| **Clinician Safety / 医生评分：安全性** | | | 5 | |  | |

**Patient 76**

| **Patient ID / 患者编号** | **Group / 模型组别** | | | | | |
| --- | --- | --- | --- | --- | --- | --- |
| P76 | ChatGPT 5.5 | | | | | |
| **General model response or patient education material (Chinese) / 模型总体回答或患者教育材料（中文）** | | | | | | |
| 很多抗生素可在哺乳期使用，但具体要看药名、剂量和宝宝情况，应按医生处方服用。重点是正确含接、有效排奶和休息补水；若红肿热痛、发热或24小时不缓解，应就医。是否需要间隔哺乳取决于药物种类，多数常用药不需要固定暂停，不能自行延迟或停药。重点是正确含接、有效排奶和休息补水；若红肿热痛、发热或24小时不缓解，应就医。看病时应主动说明正在哺乳，尤其涉及抗生素、止痛药、镇静药、抗过敏药、激素和中成药。重点是正确含接、有效排奶和休息补水；若红肿热痛、发热或24小时不缓解，应就医。以上内容为一般健康教育，不能替代线下面诊。 | | | | | | |
| **English translation of the Chinese general response / 中文总体回答英文翻译** | | | | | | |
| Many antibiotics can be used while breastfeeding, but this depends on the medicine, dose, and the baby’s condition. Take them as prescribed. Focus on effective milk removal and correct latch. Redness, fever, or no improvement within 24 hours needs care. Whether feeding needs to be spaced depends on the drug. Many common medicines do not require routine interruption. Do not delay feeds or stop medicine by yourself. Focus on effective milk removal and correct latch. Redness, fever, or no improvement within 24 hours needs care. Always tell clinicians you are breastfeeding, especially before antibiotics, pain medicines, sedatives, allergy medicines, hormones, or herbal products are used. Focus on effective milk removal and correct latch. Redness, fever, or no improvement within 24 hours needs care. This is general health education and does not replace an in-person medical assessment. | | | | | | |
| **Item / 项目** | | **Q1 / 问题1** | | **Q2 / 问题2** | | **Q3 / 问题3** |
| **Patient question (Chinese original) / 患者原始问题（中文）** | | 产后医生给我开了抗生素，我还可以喂奶吗？ | | 吃药后需要间隔多久再哺乳？ | | 哪些药物需要提前告诉医生我正在哺乳？ |
| **English translation of patient question / 患者问题英文翻译** | | The doctor prescribed antibiotics after birth. Can I still breastfeed? | | How long should I wait after taking medicine before breastfeeding? | | What medicines should I tell the doctor about because I am breastfeeding? |
| **Model response (Chinese) / 模型回答（中文）** | | 很多抗生素可在哺乳期使用，但具体要看药名、剂量和宝宝情况，应按医生处方服用。重点是正确含接、有效排奶和休息补水；若红肿热痛、发热或24小时不缓解，应就医。 | | 是否需要间隔哺乳取决于药物种类，多数常用药不需要固定暂停，不能自行延迟或停药。重点是正确含接、有效排奶和休息补水；若红肿热痛、发热或24小时不缓解，应就医。 | | 看病时应主动说明正在哺乳，尤其涉及抗生素、止痛药、镇静药、抗过敏药、激素和中成药。重点是正确含接、有效排奶和休息补水；若红肿热痛、发热或24小时不缓解，应就医。 |
| **English translation of model response / 模型回答英文翻译** | | Many antibiotics can be used while breastfeeding, but this depends on the medicine, dose, and the baby’s condition. Take them as prescribed. Focus on effective milk removal and correct latch. Redness, fever, or no improvement within 24 hours needs care. | | Whether feeding needs to be spaced depends on the drug. Many common medicines do not require routine interruption. Do not delay feeds or stop medicine by yourself. Focus on effective milk removal and correct latch. Redness, fever, or no improvement within 24 hours needs care. | | Always tell clinicians you are breastfeeding, especially before antibiotics, pain medicines, sedatives, allergy medicines, hormones, or herbal products are used. Focus on effective milk removal and correct latch. Redness, fever, or no improvement within 24 hours needs care. |
| **Overall evaluation item / 整体评价项目** | | | **Score / 分数** | | **Comments / 备注** | |
| **Patient Satisfaction / 患者满意度** | | | 4 | |  | |
| **Patient Understandability / 患者可理解性** | | | 4 | |  | |
| **Patient Usefulness / 患者实用性** | | | 5 | |  | |
| **Clinician Correctness / 医生评分：正确性** | | | 4 | |  | |
| **Clinician Completeness / 医生评分：完整性** | | | 5 | |  | |
| **Clinician Readability / 医生评分：可读性** | | | 4 | |  | |
| **Clinician Helpfulness / 医生评分：帮助性** | | | 5 | |  | |
| **Clinician Safety / 医生评分：安全性** | | | 5 | |  | |

**Patient 77**

| **Patient ID / 患者编号** | **Group / 模型组别** | | | | | |
| --- | --- | --- | --- | --- | --- | --- |
| P77 | ChatGPT 5.5 | | | | | |
| **General model response or patient education material (Chinese) / 模型总体回答或患者教育材料（中文）** | | | | | | |
| 产后伤口疼影响休息时，可以在医生指导下使用哺乳期相对安全的止痛药，不必硬忍。重点是正确含接、有效排奶和休息补水；若红肿热痛、发热或24小时不缓解，应就医。部分药物会进入乳汁，但常用止痛药有可选择的安全方案，关键是按剂量和疗程使用。重点是正确含接、有效排奶和休息补水；若红肿热痛、发热或24小时不缓解，应就医。若止痛药仍不能缓解，或疼痛越来越重、伴红肿渗液、发热，应复查，排除感染、血肿或伤口裂开。建议复测体温，并观察恶露、乳房、尿痛和伤口；若体温≥38℃、寒战或精神差，应就医。以上内容为一般健康教育，不能替代线下面诊。 | | | | | | |
| **English translation of the Chinese general response / 中文总体回答英文翻译** | | | | | | |
| If postpartum wound pain affects sleep, breastfeeding-compatible pain medicine can be used under medical guidance. You do not need to endure severe pain. Focus on effective milk removal and correct latch. Redness, fever, or no improvement within 24 hours needs care. Some medicines enter breast milk, but safer options are available. Correct dose and duration are important. Focus on effective milk removal and correct latch. Redness, fever, or no improvement within 24 hours needs care. If pain medicine does not help, or pain worsens with redness, drainage, or fever, seek review to rule out infection, hematoma, or wound opening. Record color, amount, odor, and pad use. Seek care for heavy bleeding, clots, pain, or fever. This is general health education and does not replace an in-person medical assessment. | | | | | | |
| **Item / 项目** | | **Q1 / 问题1** | | **Q2 / 问题2** | | **Q3 / 问题3** |
| **Patient question (Chinese original) / 患者原始问题（中文）** | | 产后伤口疼影响睡觉，可以吃止痛药吗？ | | 哺乳期吃止痛药会不会进入乳汁？ | | 如果止痛药也缓解不了疼痛，需要复查吗？ |
| **English translation of patient question / 患者问题英文翻译** | | My wound pain affects sleep after birth. Can I take pain medicine? | | Will pain medicine taken during breastfeeding enter the milk? | | If pain medicine still does not relieve the pain, do I need a recheck? |
| **Model response (Chinese) / 模型回答（中文）** | | 产后伤口疼影响休息时，可以在医生指导下使用哺乳期相对安全的止痛药，不必硬忍。重点是正确含接、有效排奶和休息补水；若红肿热痛、发热或24小时不缓解，应就医。 | | 部分药物会进入乳汁，但常用止痛药有可选择的安全方案，关键是按剂量和疗程使用。重点是正确含接、有效排奶和休息补水；若红肿热痛、发热或24小时不缓解，应就医。 | | 若止痛药仍不能缓解，或疼痛越来越重、伴红肿渗液、发热，应复查，排除感染、血肿或伤口裂开。建议复测体温，并观察恶露、乳房、尿痛和伤口；若体温≥38℃、寒战或精神差，应就医。 |
| **English translation of model response / 模型回答英文翻译** | | If postpartum wound pain affects sleep, breastfeeding-compatible pain medicine can be used under medical guidance. You do not need to endure severe pain. Focus on effective milk removal and correct latch. Redness, fever, or no improvement within 24 hours needs care. | | Some medicines enter breast milk, but safer options are available. Correct dose and duration are important. Focus on effective milk removal and correct latch. Redness, fever, or no improvement within 24 hours needs care. | | If pain medicine does not help, or pain worsens with redness, drainage, or fever, seek review to rule out infection, hematoma, or wound opening. Record color, amount, odor, and pad use. Seek care for heavy bleeding, clots, pain, or fever. |
| **Overall evaluation item / 整体评价项目** | | | **Score / 分数** | | **Comments / 备注** | |
| **Patient Satisfaction / 患者满意度** | | | 4 | |  | |
| **Patient Understandability / 患者可理解性** | | | 4 | |  | |
| **Patient Usefulness / 患者实用性** | | | 5 | |  | |
| **Clinician Correctness / 医生评分：正确性** | | | 5 | |  | |
| **Clinician Completeness / 医生评分：完整性** | | | 5 | |  | |
| **Clinician Readability / 医生评分：可读性** | | | 4 | |  | |
| **Clinician Helpfulness / 医生评分：帮助性** | | | 5 | |  | |
| **Clinician Safety / 医生评分：安全性** | | | 5 | |  | |

**Patient 78**

| **Patient ID / 患者编号** | **Group / 模型组别** | | | | | |
| --- | --- | --- | --- | --- | --- | --- |
| P78 | ChatGPT 5.5 | | | | | |
| **General model response or patient education material (Chinese) / 模型总体回答或患者教育材料（中文）** | | | | | | |
| 哺乳期通常可以接种流感疫苗等多种非活疫苗，具体应根据疫苗种类和个人情况咨询接种医生。重点是正确含接、有效排奶和休息补水；若红肿热痛、发热或24小时不缓解，应就医。多数疫苗接种后不需要暂停哺乳，也不会影响母乳喂养。重点是正确含接、有效排奶和休息补水；若红肿热痛、发热或24小时不缓解，应就医。接种后低热可休息、补水并观察；若高热、过敏反应、胸闷气短或症状持续加重，应及时就医。建议复测体温，并观察恶露、乳房、尿痛和伤口；若体温≥38℃、寒战或精神差，应就医。以上内容为一般健康教育，不能替代线下面诊。 | | | | | | |
| **English translation of the Chinese general response / 中文总体回答英文翻译** | | | | | | |
| During breastfeeding, the influenza vaccine and many non-live vaccines can usually be given. The exact vaccine should be discussed with the vaccination clinician. Focus on effective milk removal and correct latch. Redness, fever, or no improvement within 24 hours needs care. Breastfeeding usually does not need to be paused after most vaccines, and milk feeding is not affected. Focus on effective milk removal and correct latch. Redness, fever, or no improvement within 24 hours needs care. For mild fever after vaccination, rest, drink fluids, and observe. High fever, allergic reaction, chest tightness, shortness of breath, or worsening symptoms needs medical care. Recheck temperature and watch lochia, breasts, urination, and wounds. Fever at or above 38°C needs care. This is general health education and does not replace an in-person medical assessment. | | | | | | |
| **Item / 项目** | | **Q1 / 问题1** | | **Q2 / 问题2** | | **Q3 / 问题3** |
| **Patient question (Chinese original) / 患者原始问题（中文）** | | 哺乳期可以接种流感疫苗或其他疫苗吗？ | | 接种疫苗后需要暂停哺乳吗？ | | 如果接种后发热，应该怎么处理？ |
| **English translation of patient question / 患者问题英文翻译** | | Can I receive the flu vaccine or other vaccines while breastfeeding? | | Do I need to stop breastfeeding after vaccination? | | If I have fever after vaccination, what should I do? |
| **Model response (Chinese) / 模型回答（中文）** | | 哺乳期通常可以接种流感疫苗等多种非活疫苗，具体应根据疫苗种类和个人情况咨询接种医生。重点是正确含接、有效排奶和休息补水；若红肿热痛、发热或24小时不缓解，应就医。 | | 多数疫苗接种后不需要暂停哺乳，也不会影响母乳喂养。重点是正确含接、有效排奶和休息补水；若红肿热痛、发热或24小时不缓解，应就医。 | | 接种后低热可休息、补水并观察；若高热、过敏反应、胸闷气短或症状持续加重，应及时就医。建议复测体温，并观察恶露、乳房、尿痛和伤口；若体温≥38℃、寒战或精神差，应就医。 |
| **English translation of model response / 模型回答英文翻译** | | During breastfeeding, the influenza vaccine and many non-live vaccines can usually be given. The exact vaccine should be discussed with the vaccination clinician. Focus on effective milk removal and correct latch. Redness, fever, or no improvement within 24 hours needs care. | | Breastfeeding usually does not need to be paused after most vaccines, and milk feeding is not affected. Focus on effective milk removal and correct latch. Redness, fever, or no improvement within 24 hours needs care. | | For mild fever after vaccination, rest, drink fluids, and observe. High fever, allergic reaction, chest tightness, shortness of breath, or worsening symptoms needs medical care. Recheck temperature and watch lochia, breasts, urination, and wounds. Fever at or above 38°C needs care. |
| **Overall evaluation item / 整体评价项目** | | | **Score / 分数** | | **Comments / 备注** | |
| **Patient Satisfaction / 患者满意度** | | | 5 | |  | |
| **Patient Understandability / 患者可理解性** | | | 4 | |  | |
| **Patient Usefulness / 患者实用性** | | | 4 | |  | |
| **Clinician Correctness / 医生评分：正确性** | | | 5 | |  | |
| **Clinician Completeness / 医生评分：完整性** | | | 5 | |  | |
| **Clinician Readability / 医生评分：可读性** | | | 4 | |  | |
| **Clinician Helpfulness / 医生评分：帮助性** | | | 5 | |  | |
| **Clinician Safety / 医生评分：安全性** | | | 5 | |  | |

**Patient 79**

| **Patient ID / 患者编号** | **Group / 模型组别** | | | | | |
| --- | --- | --- | --- | --- | --- | --- |
| P79 | ChatGPT 5.5 | | | | | |
| **General model response or patient education material (Chinese) / 模型总体回答或患者教育材料（中文）** | | | | | | |
| 产后怕热、心慌、手抖可能与甲状腺功能异常、贫血、焦虑或睡眠不足有关，需要检查。请告诉家人和医生，争取照护分担；若有绝望、失控感或伤害想法，应立即就医。怀疑产后甲状腺炎时，常需查甲状腺功能、甲状腺抗体，必要时由内分泌科进一步评估。建议观察变化，保证休息、饮食和清洁护理；若症状持续不缓解或逐渐加重，应联系产科门诊。甲状腺异常可能影响情绪、体力、心率和奶量，若症状明显，不要只当作产后劳累。重点是正确含接、有效排奶和休息补水；若红肿热痛、发热或24小时不缓解，应就医。以上内容为一般健康教育，不能替代线下面诊。 | | | | | | |
| **English translation of the Chinese general response / 中文总体回答英文翻译** | | | | | | |
| Feeling very hot, palpitations, and hand tremor after birth may be related to thyroid dysfunction, anemia, anxiety, or lack of sleep and should be checked. Tell family and clinicians how you feel. Hopelessness, loss of control, or harm thoughts need immediate help. Suspected postpartum thyroiditis often requires thyroid function tests and thyroid antibodies, with further endocrine evaluation if needed. Observe changes and maintain rest, nutrition, and hygiene. Persistent or worsening symptoms need obstetric advice. Thyroid problems can affect mood, energy, heart rate, and milk supply. Obvious symptoms should not be dismissed as ordinary postpartum fatigue. Focus on effective milk removal and correct latch. Redness, fever, or no improvement within 24 hours needs care. This is general health education and does not replace an in-person medical assessment. | | | | | | |
| **Item / 项目** | | **Q1 / 问题1** | | **Q2 / 问题2** | | **Q3 / 问题3** |
| **Patient question (Chinese original) / 患者原始问题（中文）** | | 我产后特别怕热、心慌、手抖，会不会是甲状腺问题？ | | 产后甲状腺炎需要做哪些检查？ | | 甲状腺异常会不会影响情绪和奶量？ |
| **English translation of patient question / 患者问题英文翻译** | | I feel very hot, have palpitations, and my hands shake after birth. Could it be a thyroid problem? | | What tests are needed for postpartum thyroiditis? | | Can thyroid problems affect mood and milk supply? |
| **Model response (Chinese) / 模型回答（中文）** | | 产后怕热、心慌、手抖可能与甲状腺功能异常、贫血、焦虑或睡眠不足有关，需要检查。请告诉家人和医生，争取照护分担；若有绝望、失控感或伤害想法，应立即就医。 | | 怀疑产后甲状腺炎时，常需查甲状腺功能、甲状腺抗体，必要时由内分泌科进一步评估。建议观察变化，保证休息、饮食和清洁护理；若症状持续不缓解或逐渐加重，应联系产科门诊。 | | 甲状腺异常可能影响情绪、体力、心率和奶量，若症状明显，不要只当作产后劳累。重点是正确含接、有效排奶和休息补水；若红肿热痛、发热或24小时不缓解，应就医。 |
| **English translation of model response / 模型回答英文翻译** | | Feeling very hot, palpitations, and hand tremor after birth may be related to thyroid dysfunction, anemia, anxiety, or lack of sleep and should be checked. Tell family and clinicians how you feel. Hopelessness, loss of control, or harm thoughts need immediate help. | | Suspected postpartum thyroiditis often requires thyroid function tests and thyroid antibodies, with further endocrine evaluation if needed. Observe changes and maintain rest, nutrition, and hygiene. Persistent or worsening symptoms need obstetric advice. | | Thyroid problems can affect mood, energy, heart rate, and milk supply. Obvious symptoms should not be dismissed as ordinary postpartum fatigue. Focus on effective milk removal and correct latch. Redness, fever, or no improvement within 24 hours needs care. |
| **Overall evaluation item / 整体评价项目** | | | **Score / 分数** | | **Comments / 备注** | |
| **Patient Satisfaction / 患者满意度** | | | 4 | |  | |
| **Patient Understandability / 患者可理解性** | | | 4 | |  | |
| **Patient Usefulness / 患者实用性** | | | 4 | |  | |
| **Clinician Correctness / 医生评分：正确性** | | | 5 | |  | |
| **Clinician Completeness / 医生评分：完整性** | | | 5 | |  | |
| **Clinician Readability / 医生评分：可读性** | | | 4 | |  | |
| **Clinician Helpfulness / 医生评分：帮助性** | | | 5 | |  | |
| **Clinician Safety / 医生评分：安全性** | | | 5 | |  | |

**Patient 80**

| **Patient ID / 患者编号** | **Group / 模型组别** | | | | | |
| --- | --- | --- | --- | --- | --- | --- |
| P80 | ChatGPT 5.5 | | | | | |
| **General model response or patient education material (Chinese) / 模型总体回答或患者教育材料（中文）** | | | | | | |
| 孕期高血压产后两周头痛需要重视，产后仍可能出现血压升高或子痫前期相关风险。建议按医嘱测血压，记录头痛、眼花和水肿；若血压升高或症状明显，应及时就医。产后血压至少应按医生要求持续监测到产后6周，血压不稳者需更长时间随访。建议按医嘱测血压，记录头痛、眼花和水肿；若血压升高或症状明显，应及时就医。若头痛明显、眼花、胸闷、上腹痛、气短、血压升高或抽搐，应立即急诊就医。建议按医嘱测血压，记录头痛、眼花和水肿；若血压升高或症状明显，应及时就医。以上内容为一般健康教育，不能替代线下面诊。 | | | | | | |
| **English translation of the Chinese general response / 中文总体回答英文翻译** | | | | | | |
| Headache two weeks after birth in someone with pregnancy hypertension should be taken seriously. High blood pressure and preeclampsia-related risks can still occur postpartum. Monitor blood pressure and symptoms. Rising pressure, headache, visual changes, or swelling needs prompt care. Postpartum blood pressure should be monitored at least as directed until 6 weeks after birth, and longer if it remains unstable. Record color, amount, odor, and pad use. Seek care for heavy bleeding, clots, pain, or fever. Severe headache, blurred vision, chest tightness, upper abdominal pain, shortness of breath, high blood pressure, or seizure requires emergency care. Record color, amount, odor, and pad use. Seek care for heavy bleeding, clots, pain, or fever. This is general health education and does not replace an in-person medical assessment. | | | | | | |
| **Item / 项目** | | **Q1 / 问题1** | | **Q2 / 问题2** | | **Q3 / 问题3** |
| **Patient question (Chinese original) / 患者原始问题（中文）** | | 我孕期血压高，产后两周又头痛，是不是危险信号？ | | 产后血压应该监测到什么时候？ | | 如果出现眼花、胸闷或上腹痛，需要急诊吗？ |
| **English translation of patient question / 患者问题英文翻译** | | I had high blood pressure during pregnancy and now have a headache two weeks after birth. Is this a danger sign? | | How long should postpartum blood pressure be monitored? | | If I have blurred vision, chest tightness, or upper abdominal pain, should I go to the emergency department? |
| **Model response (Chinese) / 模型回答（中文）** | | 孕期高血压产后两周头痛需要重视，产后仍可能出现血压升高或子痫前期相关风险。建议按医嘱测血压，记录头痛、眼花和水肿；若血压升高或症状明显，应及时就医。 | | 产后血压至少应按医生要求持续监测到产后6周，血压不稳者需更长时间随访。建议按医嘱测血压，记录头痛、眼花和水肿；若血压升高或症状明显，应及时就医。 | | 若头痛明显、眼花、胸闷、上腹痛、气短、血压升高或抽搐，应立即急诊就医。建议按医嘱测血压，记录头痛、眼花和水肿；若血压升高或症状明显，应及时就医。 |
| **English translation of model response / 模型回答英文翻译** | | Headache two weeks after birth in someone with pregnancy hypertension should be taken seriously. High blood pressure and preeclampsia-related risks can still occur postpartum. Monitor blood pressure and symptoms. Rising pressure, headache, visual changes, or swelling needs prompt care. | | Postpartum blood pressure should be monitored at least as directed until 6 weeks after birth, and longer if it remains unstable. Record color, amount, odor, and pad use. Seek care for heavy bleeding, clots, pain, or fever. | | Severe headache, blurred vision, chest tightness, upper abdominal pain, shortness of breath, high blood pressure, or seizure requires emergency care. Record color, amount, odor, and pad use. Seek care for heavy bleeding, clots, pain, or fever. |
| **Overall evaluation item / 整体评价项目** | | | **Score / 分数** | | **Comments / 备注** | |
| **Patient Satisfaction / 患者满意度** | | | 4 | |  | |
| **Patient Understandability / 患者可理解性** | | | 4 | |  | |
| **Patient Usefulness / 患者实用性** | | | 4 | |  | |
| **Clinician Correctness / 医生评分：正确性** | | | 4 | |  | |
| **Clinician Completeness / 医生评分：完整性** | | | 5 | |  | |
| **Clinician Readability / 医生评分：可读性** | | | 4 | |  | |
| **Clinician Helpfulness / 医生评分：帮助性** | | | 5 | |  | |
| **Clinician Safety / 医生评分：安全性** | | | 4 | |  | |

**Patient 81**

| **Patient ID / 患者编号** | **Group / 模型组别** | | | | | |
| --- | --- | --- | --- | --- | --- | --- |
| P81 | ChatGPT 5.5 | | | | | |
| **General model response or patient education material (Chinese) / 模型总体回答或患者教育材料（中文）** | | | | | | |
| 妊娠期糖尿病产后通常建议在6到12周复查75克糖耐量试验，具体按医生安排。建议适量饮水、规律排尿；若尿痛加重、发热、腰痛或排尿困难持续，应查尿常规。复查前是否空腹和哺乳安排应按检验要求执行，哺乳本身不是不复查的理由。重点是正确含接、有效排奶和休息补水；若红肿热痛、发热或24小时不缓解，应就医。若产后血糖仍偏高，应到内分泌科随访，并通过饮食、运动和体重管理降低今后糖尿病风险。建议适量饮水、规律排尿；若尿痛加重、发热、腰痛或排尿困难持续，应查尿常规。以上内容为一般健康教育，不能替代线下面诊。 | | | | | | |
| **English translation of the Chinese general response / 中文总体回答英文翻译** | | | | | | |
| After gestational diabetes, a 75-g oral glucose tolerance test is usually recommended at 6 to 12 weeks postpartum, according to the clinician’s plan. Postpartum blood glucose follow-up remains important, with balanced meals, gentle activity, and long-term monitoring. Fasting and breastfeeding arrangements before testing should follow laboratory instructions. Breastfeeding is not a reason to skip testing. Focus on effective milk removal and correct latch. Redness, fever, or no improvement within 24 hours needs care. If postpartum glucose remains high, endocrine follow-up is needed. Diet, exercise, and weight management help reduce future diabetes risk. Record color, amount, odor, and pad use. Seek care for heavy bleeding, clots, pain, or fever. This is general health education and does not replace an in-person medical assessment. | | | | | | |
| **Item / 项目** | | **Q1 / 问题1** | | **Q2 / 问题2** | | **Q3 / 问题3** |
| **Patient question (Chinese original) / 患者原始问题（中文）** | | 我孕期血糖高，产后多久做糖耐量复查？ | | 复查前还在哺乳，会影响血糖结果吗？ | | 如果产后血糖偏高，需要内分泌科随访吗？ |
| **English translation of patient question / 患者问题英文翻译** | | I had high blood sugar during pregnancy. When should I repeat an oral glucose tolerance test after birth? | | Will breastfeeding before the test affect the blood sugar result? | | If postpartum blood sugar is high, do I need endocrine follow-up? |
| **Model response (Chinese) / 模型回答（中文）** | | 妊娠期糖尿病产后通常建议在6到12周复查75克糖耐量试验，具体按医生安排。建议适量饮水、规律排尿；若尿痛加重、发热、腰痛或排尿困难持续，应查尿常规。 | | 复查前是否空腹和哺乳安排应按检验要求执行，哺乳本身不是不复查的理由。重点是正确含接、有效排奶和休息补水；若红肿热痛、发热或24小时不缓解，应就医。 | | 若产后血糖仍偏高，应到内分泌科随访，并通过饮食、运动和体重管理降低今后糖尿病风险。建议适量饮水、规律排尿；若尿痛加重、发热、腰痛或排尿困难持续，应查尿常规。 |
| **English translation of model response / 模型回答英文翻译** | | After gestational diabetes, a 75-g oral glucose tolerance test is usually recommended at 6 to 12 weeks postpartum, according to the clinician’s plan. Postpartum blood glucose follow-up remains important, with balanced meals, gentle activity, and long-term monitoring. | | Fasting and breastfeeding arrangements before testing should follow laboratory instructions. Breastfeeding is not a reason to skip testing. Focus on effective milk removal and correct latch. Redness, fever, or no improvement within 24 hours needs care. | | If postpartum glucose remains high, endocrine follow-up is needed. Diet, exercise, and weight management help reduce future diabetes risk. Record color, amount, odor, and pad use. Seek care for heavy bleeding, clots, pain, or fever. |
| **Overall evaluation item / 整体评价项目** | | | **Score / 分数** | | **Comments / 备注** | |
| **Patient Satisfaction / 患者满意度** | | | 5 | |  | |
| **Patient Understandability / 患者可理解性** | | | 4 | |  | |
| **Patient Usefulness / 患者实用性** | | | 4 | |  | |
| **Clinician Correctness / 医生评分：正确性** | | | 5 | |  | |
| **Clinician Completeness / 医生评分：完整性** | | | 5 | |  | |
| **Clinician Readability / 医生评分：可读性** | | | 4 | |  | |
| **Clinician Helpfulness / 医生评分：帮助性** | | | 5 | |  | |
| **Clinician Safety / 医生评分：安全性** | | | 5 | |  | |

**Patient 82**

| **Patient ID / 患者编号** | **Group / 模型组别** | | | | | |
| --- | --- | --- | --- | --- | --- | --- |
| P82 | ChatGPT 5.5 | | | | | |
| **General model response or patient education material (Chinese) / 模型总体回答或患者教育材料（中文）** | | | | | | |
| 哺乳期不建议极端减少主食，应保证能量、蛋白质、铁、钙和水分摄入。重点是正确含接、有效排奶和休息补水；若红肿热痛、发热或24小时不缓解，应就医。体重下降过快可能影响奶量、体力和情绪，也不利于伤口和盆底恢复。重点是正确含接、有效排奶和休息补水；若红肿热痛、发热或24小时不缓解，应就医。安全减重应以均衡饮食、规律哺乳、逐步增加运动为主，避免节食药、代餐极端方案和过早高强度训练。重点是正确含接、有效排奶和休息补水；若红肿热痛、发热或24小时不缓解，应就医。以上内容为一般健康教育，不能替代线下面诊。 | | | | | | |
| **English translation of the Chinese general response / 中文总体回答英文翻译** | | | | | | |
| During breastfeeding, extreme restriction of staple foods is not recommended. Energy, protein, iron, calcium, and fluids should be adequate. Focus on effective milk removal and correct latch. Redness, fever, or no improvement within 24 hours needs care. Losing weight too quickly may affect milk supply, energy, mood, wound healing, and pelvic floor recovery. Focus on effective milk removal and correct latch. Redness, fever, or no improvement within 24 hours needs care. Safe weight loss should focus on balanced meals, regular breastfeeding, and gradual exercise. Avoid diet pills, extreme meal replacements, and early high-intensity training. Focus on effective milk removal and correct latch. Redness, fever, or no improvement within 24 hours needs care. This is general health education and does not replace an in-person medical assessment. | | | | | | |
| **Item / 项目** | | **Q1 / 问题1** | | **Q2 / 问题2** | | **Q3 / 问题3** |
| **Patient question (Chinese original) / 患者原始问题（中文）** | | 哺乳期想减肥，可以少吃主食吗？ | | 产后体重下降太快会不会影响奶量？ | | 怎样减重既安全又不影响恢复？ |
| **English translation of patient question / 患者问题英文翻译** | | I want to lose weight while breastfeeding. Can I eat less staple food? | | Will losing weight too quickly affect milk supply? | | How can I lose weight safely without affecting recovery? |
| **Model response (Chinese) / 模型回答（中文）** | | 哺乳期不建议极端减少主食，应保证能量、蛋白质、铁、钙和水分摄入。重点是正确含接、有效排奶和休息补水；若红肿热痛、发热或24小时不缓解，应就医。 | | 体重下降过快可能影响奶量、体力和情绪，也不利于伤口和盆底恢复。重点是正确含接、有效排奶和休息补水；若红肿热痛、发热或24小时不缓解，应就医。 | | 安全减重应以均衡饮食、规律哺乳、逐步增加运动为主，避免节食药、代餐极端方案和过早高强度训练。重点是正确含接、有效排奶和休息补水；若红肿热痛、发热或24小时不缓解，应就医。 |
| **English translation of model response / 模型回答英文翻译** | | During breastfeeding, extreme restriction of staple foods is not recommended. Energy, protein, iron, calcium, and fluids should be adequate. Focus on effective milk removal and correct latch. Redness, fever, or no improvement within 24 hours needs care. | | Losing weight too quickly may affect milk supply, energy, mood, wound healing, and pelvic floor recovery. Focus on effective milk removal and correct latch. Redness, fever, or no improvement within 24 hours needs care. | | Safe weight loss should focus on balanced meals, regular breastfeeding, and gradual exercise. Avoid diet pills, extreme meal replacements, and early high-intensity training. Focus on effective milk removal and correct latch. Redness, fever, or no improvement within 24 hours needs care. |
| **Overall evaluation item / 整体评价项目** | | | **Score / 分数** | | **Comments / 备注** | |
| **Patient Satisfaction / 患者满意度** | | | 4 | |  | |
| **Patient Understandability / 患者可理解性** | | | 4 | |  | |
| **Patient Usefulness / 患者实用性** | | | 4 | |  | |
| **Clinician Correctness / 医生评分：正确性** | | | 5 | |  | |
| **Clinician Completeness / 医生评分：完整性** | | | 5 | |  | |
| **Clinician Readability / 医生评分：可读性** | | | 4 | |  | |
| **Clinician Helpfulness / 医生评分：帮助性** | | | 5 | |  | |
| **Clinician Safety / 医生评分：安全性** | | | 5 | |  | |

**Patient 83**

| **Patient ID / 患者编号** | **Group / 模型组别** | | | | | |
| --- | --- | --- | --- | --- | --- | --- |
| P83 | ChatGPT 5.5 | | | | | |
| **General model response or patient education material (Chinese) / 模型总体回答或患者教育材料（中文）** | | | | | | |
| 产后长期吃得太少可能影响体力、乳汁分泌和伤口恢复，应尽量少量多餐。重点是正确含接、有效排奶和休息补水；若红肿热痛、发热或24小时不缓解，应就医。素食产妇要注意蛋白质、铁、维生素B12、钙、维生素D和优质脂肪摄入。重点是正确含接、有效排奶和休息补水；若红肿热痛、发热或24小时不缓解，应就医。若经常头晕乏力、心慌或体重下降明显，应检查血常规、铁蛋白及相关营养指标。饮食要保证主食、蛋白质、蔬菜水果和水分；若头晕乏力、体重下降或奶量减少，应咨询医生。以上内容为一般健康教育，不能替代线下面诊。 | | | | | | |
| **English translation of the Chinese general response / 中文总体回答英文翻译** | | | | | | |
| Eating too little for a long time after birth may affect energy, milk production, and wound healing. Small frequent meals may help. Focus on effective milk removal and correct latch. Redness, fever, or no improvement within 24 hours needs care. A vegetarian breastfeeding mother should pay attention to protein, iron, vitamin B12, calcium, vitamin D, and healthy fats. Focus on effective milk removal and correct latch. Redness, fever, or no improvement within 24 hours needs care. Frequent dizziness, weakness, palpitations, or marked weight loss should prompt testing such as blood count, ferritin, and related nutrition markers. Keep staple foods, protein, vegetables, fruit, and fluids. Dizziness, weakness, weight loss, or low milk supply needs advice. This is general health education and does not replace an in-person medical assessment. | | | | | | |
| **Item / 项目** | | **Q1 / 问题1** | | **Q2 / 问题2** | | **Q3 / 问题3** |
| **Patient question (Chinese original) / 患者原始问题（中文）** | | 我产后胃口不好，吃得少会影响伤口恢复吗？ | | 素食产妇哺乳期需要特别补充什么？ | | 如果经常头晕乏力，需要查营养指标吗？ |
| **English translation of patient question / 患者问题英文翻译** | | My appetite is poor after birth. Will eating too little affect wound healing? | | What should a vegetarian mother pay special attention to while breastfeeding? | | If I often feel dizzy and weak, do I need nutrition tests? |
| **Model response (Chinese) / 模型回答（中文）** | | 产后长期吃得太少可能影响体力、乳汁分泌和伤口恢复，应尽量少量多餐。重点是正确含接、有效排奶和休息补水；若红肿热痛、发热或24小时不缓解，应就医。 | | 素食产妇要注意蛋白质、铁、维生素B12、钙、维生素D和优质脂肪摄入。重点是正确含接、有效排奶和休息补水；若红肿热痛、发热或24小时不缓解，应就医。 | | 若经常头晕乏力、心慌或体重下降明显，应检查血常规、铁蛋白及相关营养指标。饮食要保证主食、蛋白质、蔬菜水果和水分；若头晕乏力、体重下降或奶量减少，应咨询医生。 |
| **English translation of model response / 模型回答英文翻译** | | Eating too little for a long time after birth may affect energy, milk production, and wound healing. Small frequent meals may help. Focus on effective milk removal and correct latch. Redness, fever, or no improvement within 24 hours needs care. | | A vegetarian breastfeeding mother should pay attention to protein, iron, vitamin B12, calcium, vitamin D, and healthy fats. Focus on effective milk removal and correct latch. Redness, fever, or no improvement within 24 hours needs care. | | Frequent dizziness, weakness, palpitations, or marked weight loss should prompt testing such as blood count, ferritin, and related nutrition markers. Keep staple foods, protein, vegetables, fruit, and fluids. Dizziness, weakness, weight loss, or low milk supply needs advice. |
| **Overall evaluation item / 整体评价项目** | | | **Score / 分数** | | **Comments / 备注** | |
| **Patient Satisfaction / 患者满意度** | | | 4 | |  | |
| **Patient Understandability / 患者可理解性** | | | 4 | |  | |
| **Patient Usefulness / 患者实用性** | | | 4 | |  | |
| **Clinician Correctness / 医生评分：正确性** | | | 5 | |  | |
| **Clinician Completeness / 医生评分：完整性** | | | 4 | |  | |
| **Clinician Readability / 医生评分：可读性** | | | 4 | |  | |
| **Clinician Helpfulness / 医生评分：帮助性** | | | 4 | |  | |
| **Clinician Safety / 医生评分：安全性** | | | 5 | |  | |

**Patient 84**

| **Patient ID / 患者编号** | **Group / 模型组别** | | | | | |
| --- | --- | --- | --- | --- | --- | --- |
| P84 | ChatGPT 5.5 | | | | | |
| **General model response or patient education material (Chinese) / 模型总体回答或患者教育材料（中文）** | | | | | | |
| 哺乳期腰腿酸不一定都是缺钙，也可能与抱娃姿势、睡眠不足、肌肉劳损有关。重点是正确含接、有效排奶和休息补水；若红肿热痛、发热或24小时不缓解，应就医。哺乳期通常需要保证钙和维生素D摄入，可通过饮食、日照和医生建议补充。重点是正确含接、有效排奶和休息补水；若红肿热痛、发热或24小时不缓解，应就医。若腿抽筋频繁、疼痛明显、单侧肿胀或伴麻木，应就医评估电解质、维生素D和血栓风险。不要按摩肿痛肢体；若单侧腿肿痛、胸闷气短、胸痛或晕厥，应立即就医。以上内容为一般健康教育，不能替代线下面诊。 | | | | | | |
| **English translation of the Chinese general response / 中文总体回答英文翻译** | | | | | | |
| Back and leg soreness during breastfeeding is not always calcium deficiency. Holding posture, poor sleep, and muscle strain may also contribute. Focus on effective milk removal and correct latch. Redness, fever, or no improvement within 24 hours needs care. Calcium and vitamin D intake should usually be adequate during breastfeeding through diet, sunlight, and supplements if recommended. Focus on effective milk removal and correct latch. Redness, fever, or no improvement within 24 hours needs care. Frequent cramps, marked pain, one-sided swelling, or numbness should be evaluated for electrolytes, vitamin D status, and possible thrombosis. Monitor blood pressure and symptoms. Rising pressure, headache, visual changes, or swelling needs prompt care. This is general health education and does not replace an in-person medical assessment. | | | | | | |
| **Item / 项目** | | **Q1 / 问题1** | | **Q2 / 问题2** | | **Q3 / 问题3** |
| **Patient question (Chinese original) / 患者原始问题（中文）** | | 哺乳期腰腿酸，是不是缺钙？ | | 产后需要补钙和维生素D吗？ | | 如果腿抽筋频繁，需要去检查吗？ |
| **English translation of patient question / 患者问题英文翻译** | | I have back and leg soreness while breastfeeding. Is it calcium deficiency? | | Do I need calcium and vitamin D after birth? | | If leg cramps happen often, should I be checked? |
| **Model response (Chinese) / 模型回答（中文）** | | 哺乳期腰腿酸不一定都是缺钙，也可能与抱娃姿势、睡眠不足、肌肉劳损有关。重点是正确含接、有效排奶和休息补水；若红肿热痛、发热或24小时不缓解，应就医。 | | 哺乳期通常需要保证钙和维生素D摄入，可通过饮食、日照和医生建议补充。重点是正确含接、有效排奶和休息补水；若红肿热痛、发热或24小时不缓解，应就医。 | | 若腿抽筋频繁、疼痛明显、单侧肿胀或伴麻木，应就医评估电解质、维生素D和血栓风险。不要按摩肿痛肢体；若单侧腿肿痛、胸闷气短、胸痛或晕厥，应立即就医。 |
| **English translation of model response / 模型回答英文翻译** | | Back and leg soreness during breastfeeding is not always calcium deficiency. Holding posture, poor sleep, and muscle strain may also contribute. Focus on effective milk removal and correct latch. Redness, fever, or no improvement within 24 hours needs care. | | Calcium and vitamin D intake should usually be adequate during breastfeeding through diet, sunlight, and supplements if recommended. Focus on effective milk removal and correct latch. Redness, fever, or no improvement within 24 hours needs care. | | Frequent cramps, marked pain, one-sided swelling, or numbness should be evaluated for electrolytes, vitamin D status, and possible thrombosis. Monitor blood pressure and symptoms. Rising pressure, headache, visual changes, or swelling needs prompt care. |
| **Overall evaluation item / 整体评价项目** | | | **Score / 分数** | | **Comments / 备注** | |
| **Patient Satisfaction / 患者满意度** | | | 4 | |  | |
| **Patient Understandability / 患者可理解性** | | | 4 | |  | |
| **Patient Usefulness / 患者实用性** | | | 4 | |  | |
| **Clinician Correctness / 医生评分：正确性** | | | 4 | |  | |
| **Clinician Completeness / 医生评分：完整性** | | | 4 | |  | |
| **Clinician Readability / 医生评分：可读性** | | | 4 | |  | |
| **Clinician Helpfulness / 医生评分：帮助性** | | | 4 | |  | |
| **Clinician Safety / 医生评分：安全性** | | | 5 | |  | |

**Patient 85**

| **Patient ID / 患者编号** | **Group / 模型组别** | | | | | |
| --- | --- | --- | --- | --- | --- | --- |
| P85 | ChatGPT 5.5 | | | | | |
| **General model response or patient education material (Chinese) / 模型总体回答或患者教育材料（中文）** | | | | | | |
| 铁剂可能加重便秘，可增加水分、膳食纤维和活动量，必要时请医生调整铁剂种类或使用软便药。建议增加饮水和膳食纤维，适量活动；若便血增多、腹胀明显或疼痛剧烈，应就医。铁剂不建议和牛奶、钙片、茶或咖啡同服，以免影响吸收，可与维生素C丰富食物搭配。重点是正确含接、有效排奶和休息补水；若红肿热痛、发热或24小时不缓解，应就医。贫血长期不改善，应复查血常规、铁蛋白，评估持续出血、吸收不良、炎症或其他类型贫血。建议记录颜色、量和气味，避免劳累；若1小时湿透卫生巾、血块增多、腹痛或发热，应就医。以上内容为一般健康教育，不能替代线下面诊。 | | | | | | |
| **English translation of the Chinese general response / 中文总体回答英文翻译** | | | | | | |
| Iron can worsen constipation. Increase fluids, fiber, and activity; if needed, ask the clinician to change the iron form or add a stool softener. Increase fluids, fiber, and gentle activity. More bleeding, marked bloating, or severe pain needs care. Iron should not be taken with milk, calcium, tea, or coffee because absorption may be reduced. Vitamin C-rich foods may help absorption. Record color, amount, odor, and pad use. Seek care for heavy bleeding, clots, pain, or fever. Persistent anemia needs repeat blood count and ferritin testing, and evaluation for ongoing bleeding, poor absorption, inflammation, or other anemia types. Record color, amount, odor, and pad use. Seek care for heavy bleeding, clots, pain, or fever. This is general health education and does not replace an in-person medical assessment. | | | | | | |
| **Item / 项目** | | **Q1 / 问题1** | | **Q2 / 问题2** | | **Q3 / 问题3** |
| **Patient question (Chinese original) / 患者原始问题（中文）** | | 我补铁后便秘更严重，应该怎么办？ | | 铁剂可以和牛奶一起吃吗？ | | 如果贫血一直不改善，需要查什么原因？ |
| **English translation of patient question / 患者问题英文翻译** | | My constipation got worse after taking iron. What should I do? | | Can I take iron tablets with milk? | | If anemia does not improve, what causes should be checked? |
| **Model response (Chinese) / 模型回答（中文）** | | 铁剂可能加重便秘，可增加水分、膳食纤维和活动量，必要时请医生调整铁剂种类或使用软便药。建议增加饮水和膳食纤维，适量活动；若便血增多、腹胀明显或疼痛剧烈，应就医。 | | 铁剂不建议和牛奶、钙片、茶或咖啡同服，以免影响吸收，可与维生素C丰富食物搭配。重点是正确含接、有效排奶和休息补水；若红肿热痛、发热或24小时不缓解，应就医。 | | 贫血长期不改善，应复查血常规、铁蛋白，评估持续出血、吸收不良、炎症或其他类型贫血。建议记录颜色、量和气味，避免劳累；若1小时湿透卫生巾、血块增多、腹痛或发热，应就医。 |
| **English translation of model response / 模型回答英文翻译** | | Iron can worsen constipation. Increase fluids, fiber, and activity; if needed, ask the clinician to change the iron form or add a stool softener. Increase fluids, fiber, and gentle activity. More bleeding, marked bloating, or severe pain needs care. | | Iron should not be taken with milk, calcium, tea, or coffee because absorption may be reduced. Vitamin C-rich foods may help absorption. Record color, amount, odor, and pad use. Seek care for heavy bleeding, clots, pain, or fever. | | Persistent anemia needs repeat blood count and ferritin testing, and evaluation for ongoing bleeding, poor absorption, inflammation, or other anemia types. Record color, amount, odor, and pad use. Seek care for heavy bleeding, clots, pain, or fever. |
| **Overall evaluation item / 整体评价项目** | | | **Score / 分数** | | **Comments / 备注** | |
| **Patient Satisfaction / 患者满意度** | | | 5 | |  | |
| **Patient Understandability / 患者可理解性** | | | 4 | |  | |
| **Patient Usefulness / 患者实用性** | | | 4 | |  | |
| **Clinician Correctness / 医生评分：正确性** | | | 5 | |  | |
| **Clinician Completeness / 医生评分：完整性** | | | 5 | |  | |
| **Clinician Readability / 医生评分：可读性** | | | 4 | |  | |
| **Clinician Helpfulness / 医生评分：帮助性** | | | 5 | |  | |
| **Clinician Safety / 医生评分：安全性** | | | 5 | |  | |

**Patient 86**

| **Patient ID / 患者编号** | **Group / 模型组别** | | | | | |
| --- | --- | --- | --- | --- | --- | --- |
| P86 | ChatGPT 5.5 | | | | | |
| **General model response or patient education material (Chinese) / 模型总体回答或患者教育材料（中文）** | | | | | | |
| 长期睡眠不足会影响体力、情绪、免疫和乳汁分泌，也会降低照护安全性。重点是正确含接、有效排奶和休息补水；若红肿热痛、发热或24小时不缓解，应就医。白天补觉有帮助，建议家人分担夜间照护，产妇抓住宝宝睡眠间隙休息。请告诉家人和医生，争取照护分担；若有绝望、失控感或伤害想法，应立即就医。若连续多日难以入睡、心慌焦虑、情绪低落或影响照顾宝宝，应寻求产科、心理或精神专科帮助。请告诉家人和医生，争取照护分担；若有绝望、失控感或伤害想法，应立即就医。以上内容为一般健康教育，不能替代线下面诊。 | | | | | | |
| **English translation of the Chinese general response / 中文总体回答英文翻译** | | | | | | |
| Long-term sleep deprivation can affect energy, mood, immunity, milk production, and caregiving safety. Focus on effective milk removal and correct latch. Redness, fever, or no improvement within 24 hours needs care. Daytime naps are helpful. Family members should share nighttime care, and the mother should rest when the baby sleeps. Tell family and clinicians how you feel. Hopelessness, loss of control, or harm thoughts need immediate help. If insomnia lasts many days, or palpitations, anxiety, low mood, or impaired baby care occurs, seek help from obstetrics, psychology, or psychiatry. Record color, amount, odor, and pad use. Seek care for heavy bleeding, clots, pain, or fever. This is general health education and does not replace an in-person medical assessment. | | | | | | |
| **Item / 项目** | | **Q1 / 问题1** | | **Q2 / 问题2** | | **Q3 / 问题3** |
| **Patient question (Chinese original) / 患者原始问题（中文）** | | 宝宝夜里频繁醒，我长期睡不好，会影响恢复吗？ | | 白天补觉对产后恢复有帮助吗？ | | 如果失眠持续很多天，需要寻求医生帮助吗？ |
| **English translation of patient question / 患者问题英文翻译** | | My baby wakes often at night and I sleep poorly for a long time. Will this affect recovery? | | Does daytime napping help postpartum recovery? | | If insomnia lasts for many days, should I seek medical help? |
| **Model response (Chinese) / 模型回答（中文）** | | 长期睡眠不足会影响体力、情绪、免疫和乳汁分泌，也会降低照护安全性。重点是正确含接、有效排奶和休息补水；若红肿热痛、发热或24小时不缓解，应就医。 | | 白天补觉有帮助，建议家人分担夜间照护，产妇抓住宝宝睡眠间隙休息。请告诉家人和医生，争取照护分担；若有绝望、失控感或伤害想法，应立即就医。 | | 若连续多日难以入睡、心慌焦虑、情绪低落或影响照顾宝宝，应寻求产科、心理或精神专科帮助。请告诉家人和医生，争取照护分担；若有绝望、失控感或伤害想法，应立即就医。 |
| **English translation of model response / 模型回答英文翻译** | | Long-term sleep deprivation can affect energy, mood, immunity, milk production, and caregiving safety. Focus on effective milk removal and correct latch. Redness, fever, or no improvement within 24 hours needs care. | | Daytime naps are helpful. Family members should share nighttime care, and the mother should rest when the baby sleeps. Tell family and clinicians how you feel. Hopelessness, loss of control, or harm thoughts need immediate help. | | If insomnia lasts many days, or palpitations, anxiety, low mood, or impaired baby care occurs, seek help from obstetrics, psychology, or psychiatry. Record color, amount, odor, and pad use. Seek care for heavy bleeding, clots, pain, or fever. |
| **Overall evaluation item / 整体评价项目** | | | **Score / 分数** | | **Comments / 备注** | |
| **Patient Satisfaction / 患者满意度** | | | 4 | |  | |
| **Patient Understandability / 患者可理解性** | | | 4 | |  | |
| **Patient Usefulness / 患者实用性** | | | 4 | |  | |
| **Clinician Correctness / 医生评分：正确性** | | | 5 | |  | |
| **Clinician Completeness / 医生评分：完整性** | | | 5 | |  | |
| **Clinician Readability / 医生评分：可读性** | | | 4 | |  | |
| **Clinician Helpfulness / 医生评分：帮助性** | | | 5 | |  | |
| **Clinician Safety / 医生评分：安全性** | | | 5 | |  | |

**Patient 87**

| **Patient ID / 患者编号** | **Group / 模型组别** | | | | | |
| --- | --- | --- | --- | --- | --- | --- |
| P87 | ChatGPT 5.5 | | | | | |
| **General model response or patient education material (Chinese) / 模型总体回答或患者教育材料（中文）** | | | | | | |
| 产后情绪波动较常见，但不应被简单评价为太敏感，家人应给予理解和支持。请告诉家人和医生，争取照护分担；若有绝望、失控感或伤害想法，应立即就医。若情绪低落、焦虑、易怒持续超过两周，影响睡眠、进食和照护能力，应考虑专业评估。请告诉家人和医生，争取照护分担；若有绝望、失控感或伤害想法，应立即就医。经常哭、强烈内疚、失去兴趣、绝望或出现伤害自己想法时，应尽快就医或寻求心理危机帮助。请告诉家人和医生，争取照护分担；若有绝望、失控感或伤害想法，应立即就医。以上内容为一般健康教育，不能替代线下面诊。 | | | | | | |
| **English translation of the Chinese general response / 中文总体回答英文翻译** | | | | | | |
| Mood swings after birth are common, but they should not be dismissed as being too sensitive. Family understanding and support are important. Tell family and clinicians how you feel. Hopelessness, loss of control, or harm thoughts need immediate help. If low mood, anxiety, or irritability lasts more than two weeks and affects sleep, eating, or caregiving, professional assessment is needed. Tell family and clinicians how you feel. Hopelessness, loss of control, or harm thoughts need immediate help. Frequent crying, strong guilt, loss of interest, hopelessness, or thoughts of self-harm requires prompt medical or crisis support. Tell family and clinicians how you feel. Hopelessness, loss of control, or harm thoughts need immediate help. This is general health education and does not replace an in-person medical assessment. | | | | | | |
| **Item / 项目** | | **Q1 / 问题1** | | **Q2 / 问题2** | | **Q3 / 问题3** |
| **Patient question (Chinese original) / 患者原始问题（中文）** | | 我产后情绪忽高忽低，家人说我太敏感，正常吗？ | | 怎么判断是普通情绪波动还是需要干预？ | | 如果总是哭、内疚、没兴趣照顾自己，需要就医吗？ |
| **English translation of patient question / 患者问题英文翻译** | | My mood swings a lot after birth, and my family says I am too sensitive. Is this normal? | | How can I tell ordinary mood changes from a problem that needs intervention? | | If I often cry, feel guilty, and lose interest in caring for myself, should I see a doctor? |
| **Model response (Chinese) / 模型回答（中文）** | | 产后情绪波动较常见，但不应被简单评价为太敏感，家人应给予理解和支持。请告诉家人和医生，争取照护分担；若有绝望、失控感或伤害想法，应立即就医。 | | 若情绪低落、焦虑、易怒持续超过两周，影响睡眠、进食和照护能力，应考虑专业评估。请告诉家人和医生，争取照护分担；若有绝望、失控感或伤害想法，应立即就医。 | | 经常哭、强烈内疚、失去兴趣、绝望或出现伤害自己想法时，应尽快就医或寻求心理危机帮助。请告诉家人和医生，争取照护分担；若有绝望、失控感或伤害想法，应立即就医。 |
| **English translation of model response / 模型回答英文翻译** | | Mood swings after birth are common, but they should not be dismissed as being too sensitive. Family understanding and support are important. Tell family and clinicians how you feel. Hopelessness, loss of control, or harm thoughts need immediate help. | | If low mood, anxiety, or irritability lasts more than two weeks and affects sleep, eating, or caregiving, professional assessment is needed. Tell family and clinicians how you feel. Hopelessness, loss of control, or harm thoughts need immediate help. | | Frequent crying, strong guilt, loss of interest, hopelessness, or thoughts of self-harm requires prompt medical or crisis support. Tell family and clinicians how you feel. Hopelessness, loss of control, or harm thoughts need immediate help. |
| **Overall evaluation item / 整体评价项目** | | | **Score / 分数** | | **Comments / 备注** | |
| **Patient Satisfaction / 患者满意度** | | | 4 | |  | |
| **Patient Understandability / 患者可理解性** | | | 4 | |  | |
| **Patient Usefulness / 患者实用性** | | | 4 | |  | |
| **Clinician Correctness / 医生评分：正确性** | | | 5 | |  | |
| **Clinician Completeness / 医生评分：完整性** | | | 5 | |  | |
| **Clinician Readability / 医生评分：可读性** | | | 4 | |  | |
| **Clinician Helpfulness / 医生评分：帮助性** | | | 5 | |  | |
| **Clinician Safety / 医生评分：安全性** | | | 5 | |  | |

**Patient 88**

| **Patient ID / 患者编号** | **Group / 模型组别** | | | | | |
| --- | --- | --- | --- | --- | --- | --- |
| P88 | ChatGPT 5.5 | | | | | |
| **General model response or patient education material (Chinese) / 模型总体回答或患者教育材料（中文）** | | | | | | |
| 持续担心宝宝窒息、夜间不敢睡，可能属于产后焦虑表现，需要被认真对待。请告诉家人和医生，争取照护分担；若有绝望、失控感或伤害想法，应立即就医。反复检查短期会让人安心，但长期可能强化焦虑，应学习安全睡眠知识并逐步减少过度检查。请告诉家人和医生，争取照护分担；若有绝望、失控感或伤害想法，应立即就医。若焦虑影响吃饭、睡眠和照护，应向产科、儿保、心理科或精神科寻求帮助，并让家人分担夜间看护。请告诉家人和医生，争取照护分担；若有绝望、失控感或伤害想法，应立即就医。以上内容为一般健康教育，不能替代线下面诊。 | | | | | | |
| **English translation of the Chinese general response / 中文总体回答英文翻译** | | | | | | |
| Constant fear that the baby may suffocate and inability to sleep at night may be postpartum anxiety and should be taken seriously. Tell family and clinicians how you feel. Hopelessness, loss of control, or harm thoughts need immediate help. Repeated checking may reassure briefly but can reinforce anxiety over time. Learn safe-sleep guidance and gradually reduce excessive checking. Record color, amount, odor, and pad use. Seek care for heavy bleeding, clots, pain, or fever. If anxiety affects eating, sleep, or caregiving, seek help from obstetrics, child health, psychology, or psychiatry, and ask family to share nighttime care. Tell family and clinicians how you feel. Hopelessness, loss of control, or harm thoughts need immediate help. This is general health education and does not replace an in-person medical assessment. | | | | | | |
| **Item / 项目** | | **Q1 / 问题1** | | **Q2 / 问题2** | | **Q3 / 问题3** |
| **Patient question (Chinese original) / 患者原始问题（中文）** | | 我总担心宝宝窒息，晚上不敢睡，是产后焦虑吗？ | | 反复检查宝宝会不会越来越焦虑？ | | 这种焦虑影响吃饭睡觉时应该找谁帮助？ |
| **English translation of patient question / 患者问题英文翻译** | | I always worry that the baby may suffocate and I dare not sleep at night. Is this postpartum anxiety? | | Will repeatedly checking the baby make anxiety worse? | | If this anxiety affects eating and sleeping, who should I ask for help? |
| **Model response (Chinese) / 模型回答（中文）** | | 持续担心宝宝窒息、夜间不敢睡，可能属于产后焦虑表现，需要被认真对待。请告诉家人和医生，争取照护分担；若有绝望、失控感或伤害想法，应立即就医。 | | 反复检查短期会让人安心，但长期可能强化焦虑，应学习安全睡眠知识并逐步减少过度检查。请告诉家人和医生，争取照护分担；若有绝望、失控感或伤害想法，应立即就医。 | | 若焦虑影响吃饭、睡眠和照护，应向产科、儿保、心理科或精神科寻求帮助，并让家人分担夜间看护。请告诉家人和医生，争取照护分担；若有绝望、失控感或伤害想法，应立即就医。 |
| **English translation of model response / 模型回答英文翻译** | | Constant fear that the baby may suffocate and inability to sleep at night may be postpartum anxiety and should be taken seriously. Tell family and clinicians how you feel. Hopelessness, loss of control, or harm thoughts need immediate help. | | Repeated checking may reassure briefly but can reinforce anxiety over time. Learn safe-sleep guidance and gradually reduce excessive checking. Record color, amount, odor, and pad use. Seek care for heavy bleeding, clots, pain, or fever. | | If anxiety affects eating, sleep, or caregiving, seek help from obstetrics, child health, psychology, or psychiatry, and ask family to share nighttime care. Tell family and clinicians how you feel. Hopelessness, loss of control, or harm thoughts need immediate help. |
| **Overall evaluation item / 整体评价项目** | | | **Score / 分数** | | **Comments / 备注** | |
| **Patient Satisfaction / 患者满意度** | | | 4 | |  | |
| **Patient Understandability / 患者可理解性** | | | 4 | |  | |
| **Patient Usefulness / 患者实用性** | | | 4 | |  | |
| **Clinician Correctness / 医生评分：正确性** | | | 4 | |  | |
| **Clinician Completeness / 医生评分：完整性** | | | 5 | |  | |
| **Clinician Readability / 医生评分：可读性** | | | 4 | |  | |
| **Clinician Helpfulness / 医生评分：帮助性** | | | 5 | |  | |
| **Clinician Safety / 医生评分：安全性** | | | 5 | |  | |

**Patient 89**

| **Patient ID / 患者编号** | **Group / 模型组别** | | | | | |
| --- | --- | --- | --- | --- | --- | --- |
| P89 | ChatGPT 5.5 | | | | | |
| **General model response or patient education material (Chinese) / 模型总体回答或患者教育材料（中文）** | | | | | | |
| 出现让自己害怕的伤害宝宝念头时，应立即告诉可信任家人并联系医生，不要独自承担。请告诉家人和医生，争取照护分担；若有绝望、失控感或伤害想法，应立即就医。这种念头不等于你不是好妈妈，但提示你正承受很大压力，需要专业评估和保护性支持。建议观察变化，保证休息、饮食和清洁护理；若症状持续不缓解或逐渐加重，应联系产科门诊。若害怕自己失控、冲动强烈、听到异常声音或有伤害自己和宝宝风险，应马上就医或去急诊。请告诉家人和医生，争取照护分担；若有绝望、失控感或伤害想法，应立即就医。以上内容为一般健康教育，不能替代线下面诊。 | | | | | | |
| **English translation of the Chinese general response / 中文总体回答英文翻译** | | | | | | |
| If frightening thoughts of harming the baby appear, tell a trusted family member and contact a clinician immediately. Do not manage this alone. Record color, amount, odor, and pad use. Seek care for heavy bleeding, clots, pain, or fever. Such thoughts do not mean you are a bad mother, but they show that you are under serious stress and need professional assessment and protective support. Observe changes and maintain rest, nutrition, and hygiene. Persistent or worsening symptoms need obstetric advice. If you fear losing control, have strong impulses, hear unusual voices, or there is risk of harm to yourself or the baby, seek emergency care immediately. Tell family and clinicians how you feel. Hopelessness, loss of control, or harm thoughts need immediate help. This is general health education and does not replace an in-person medical assessment. | | | | | | |
| **Item / 项目** | | **Q1 / 问题1** | | **Q2 / 问题2** | | **Q3 / 问题3** |
| **Patient question (Chinese original) / 患者原始问题（中文）** | | 我有时脑子里冒出伤害宝宝的念头，自己也很害怕，怎么办？ | | 这种想法是不是说明我不是好妈妈？ | | 如果控制不住害怕和冲动，需要马上告诉医生吗？ |
| **English translation of patient question / 患者问题英文翻译** | | Sometimes thoughts of harming the baby come into my mind and I feel very scared. What should I do? | | Does this thought mean I am not a good mother? | | If I cannot control fear or impulses, should I tell the doctor immediately? |
| **Model response (Chinese) / 模型回答（中文）** | | 出现让自己害怕的伤害宝宝念头时，应立即告诉可信任家人并联系医生，不要独自承担。请告诉家人和医生，争取照护分担；若有绝望、失控感或伤害想法，应立即就医。 | | 这种念头不等于你不是好妈妈，但提示你正承受很大压力，需要专业评估和保护性支持。建议观察变化，保证休息、饮食和清洁护理；若症状持续不缓解或逐渐加重，应联系产科门诊。 | | 若害怕自己失控、冲动强烈、听到异常声音或有伤害自己和宝宝风险，应马上就医或去急诊。请告诉家人和医生，争取照护分担；若有绝望、失控感或伤害想法，应立即就医。 |
| **English translation of model response / 模型回答英文翻译** | | If frightening thoughts of harming the baby appear, tell a trusted family member and contact a clinician immediately. Do not manage this alone. Record color, amount, odor, and pad use. Seek care for heavy bleeding, clots, pain, or fever. | | Such thoughts do not mean you are a bad mother, but they show that you are under serious stress and need professional assessment and protective support. Observe changes and maintain rest, nutrition, and hygiene. Persistent or worsening symptoms need obstetric advice. | | If you fear losing control, have strong impulses, hear unusual voices, or there is risk of harm to yourself or the baby, seek emergency care immediately. Tell family and clinicians how you feel. Hopelessness, loss of control, or harm thoughts need immediate help. |
| **Overall evaluation item / 整体评价项目** | | | **Score / 分数** | | **Comments / 备注** | |
| **Patient Satisfaction / 患者满意度** | | | 5 | |  | |
| **Patient Understandability / 患者可理解性** | | | 4 | |  | |
| **Patient Usefulness / 患者实用性** | | | 4 | |  | |
| **Clinician Correctness / 医生评分：正确性** | | | 5 | |  | |
| **Clinician Completeness / 医生评分：完整性** | | | 5 | |  | |
| **Clinician Readability / 医生评分：可读性** | | | 4 | |  | |
| **Clinician Helpfulness / 医生评分：帮助性** | | | 5 | |  | |
| **Clinician Safety / 医生评分：安全性** | | | 5 | |  | |

**Patient 90**

| **Patient ID / 患者编号** | **Group / 模型组别** | | | | | |
| --- | --- | --- | --- | --- | --- | --- |
| P90 | ChatGPT 5.5 | | | | | |
| **General model response or patient education material (Chinese) / 模型总体回答或患者教育材料（中文）** | | | | | | |
| 产后因照护方式不同而烦躁很常见，应先区分原则性安全问题和生活习惯差异。建议观察变化，保证休息、饮食和清洁护理；若症状持续不缓解或逐渐加重，应联系产科门诊。可以把喂奶、拍嗝、换尿布、做饭、清洁和夜间照护分工写清楚，减少临时争执。重点是正确含接、有效排奶和休息补水；若红肿热痛、发热或24小时不缓解，应就医。若家庭矛盾影响休息、情绪或母婴安全，建议请护士、助产士、儿保或心理人员一起沟通指导。请告诉家人和医生，争取照护分担；若有绝望、失控感或伤害想法，应立即就医。以上内容为一般健康教育，不能替代线下面诊。 | | | | | | |
| **English translation of the Chinese general response / 中文总体回答英文翻译** | | | | | | |
| Irritability about different caregiving styles is common after birth. First separate true safety issues from differences in habits. Tell family and clinicians how you feel. Hopelessness, loss of control, or harm thoughts need immediate help. Write down task sharing for feeding support, burping, diaper changing, cooking, cleaning, and nighttime care to reduce conflict. Record color, amount, odor, and pad use. Seek care for heavy bleeding, clots, pain, or fever. If family conflict affects rest, mood, or maternal and baby safety, ask nurses, midwives, child health staff, or mental health professionals for guidance. Tell family and clinicians how you feel. Hopelessness, loss of control, or harm thoughts need immediate help. This is general health education and does not replace an in-person medical assessment. | | | | | | |
| **Item / 项目** | | **Q1 / 问题1** | | **Q2 / 问题2** | | **Q3 / 问题3** |
| **Patient question (Chinese original) / 患者原始问题（中文）** | | 家人照顾方式和我不一样，我很烦躁怎么办？ | | 产后可以怎样和家人分工照顾宝宝？ | | 如果家庭矛盾影响休息和情绪，需要请医护人员指导吗？ |
| **English translation of patient question / 患者问题英文翻译** | | My family cares for the baby differently from me, and I feel very irritable. What can I do? | | How can we divide baby-care tasks after birth? | | If family conflict affects rest and mood, should we ask medical staff for guidance? |
| **Model response (Chinese) / 模型回答（中文）** | | 产后因照护方式不同而烦躁很常见，应先区分原则性安全问题和生活习惯差异。建议观察变化，保证休息、饮食和清洁护理；若症状持续不缓解或逐渐加重，应联系产科门诊。 | | 可以把喂奶、拍嗝、换尿布、做饭、清洁和夜间照护分工写清楚，减少临时争执。重点是正确含接、有效排奶和休息补水；若红肿热痛、发热或24小时不缓解，应就医。 | | 若家庭矛盾影响休息、情绪或母婴安全，建议请护士、助产士、儿保或心理人员一起沟通指导。请告诉家人和医生，争取照护分担；若有绝望、失控感或伤害想法，应立即就医。 |
| **English translation of model response / 模型回答英文翻译** | | Irritability about different caregiving styles is common after birth. First separate true safety issues from differences in habits. Tell family and clinicians how you feel. Hopelessness, loss of control, or harm thoughts need immediate help. | | Write down task sharing for feeding support, burping, diaper changing, cooking, cleaning, and nighttime care to reduce conflict. Record color, amount, odor, and pad use. Seek care for heavy bleeding, clots, pain, or fever. | | If family conflict affects rest, mood, or maternal and baby safety, ask nurses, midwives, child health staff, or mental health professionals for guidance. Tell family and clinicians how you feel. Hopelessness, loss of control, or harm thoughts need immediate help. |
| **Overall evaluation item / 整体评价项目** | | | **Score / 分数** | | **Comments / 备注** | |
| **Patient Satisfaction / 患者满意度** | | | 4 | |  | |
| **Patient Understandability / 患者可理解性** | | | 4 | |  | |
| **Patient Usefulness / 患者实用性** | | | 4 | |  | |
| **Clinician Correctness / 医生评分：正确性** | | | 5 | |  | |
| **Clinician Completeness / 医生评分：完整性** | | | 5 | |  | |
| **Clinician Readability / 医生评分：可读性** | | | 4 | |  | |
| **Clinician Helpfulness / 医生评分：帮助性** | | | 5 | |  | |
| **Clinician Safety / 医生评分：安全性** | | | 4 | |  | |

**Patient 91**

| **Patient ID / 患者编号** | **Group / 模型组别** | | | | | |
| --- | --- | --- | --- | --- | --- | --- |
| P91 | ChatGPT 5.5 | | | | | |
| **General model response or patient education material (Chinese) / 模型总体回答或患者教育材料（中文）** | | | | | | |
| 返岗前2到4周可逐步调整作息、练习吸奶和让宝宝适应照护者，但不要突然改变太多。重点是正确含接、有效排奶和休息补水；若红肿热痛、发热或24小时不缓解，应就医。母乳保存要用清洁容器，标注日期，冷藏或冷冻，并按先存先用原则处理。建议提前调整作息和吸奶安排，母乳储存要清洁、标日期，并按先存先用处理。返岗后奶量下降可增加有效哺乳和规律吸奶，保证饮水、进食和休息，必要时咨询母乳门诊。重点是正确含接、有效排奶和休息补水；若红肿热痛、发热或24小时不缓解，应就医。以上内容为一般健康教育，不能替代线下面诊。 | | | | | | |
| **English translation of the Chinese general response / 中文总体回答英文翻译** | | | | | | |
| Two to four weeks before returning to work, gradually adjust routines, practice pumping, and help the baby adapt to caregivers. Avoid sudden major changes. Focus on effective milk removal and correct latch. Redness, fever, or no improvement within 24 hours needs care. Store milk in clean containers, label the date, refrigerate or freeze properly, and use the oldest milk first. Focus on effective milk removal and correct latch. Redness, fever, or no improvement within 24 hours needs care. If supply drops after returning to work, increase effective breastfeeding and regular pumping, maintain fluids, meals, and rest, and consult a lactation clinic if needed. Focus on effective milk removal and correct latch. Redness, fever, or no improvement within 24 hours needs care. This is general health education and does not replace an in-person medical assessment. | | | | | | |
| **Item / 项目** | | **Q1 / 问题1** | | **Q2 / 问题2** | | **Q3 / 问题3** |
| **Patient question (Chinese original) / 患者原始问题（中文）** | | 我准备产后返岗，什么时候开始调整作息比较好？ | | 上班后如何保存母乳比较安全？ | | 如果返岗后奶量下降，应该怎么调整？ |
| **English translation of patient question / 患者问题英文翻译** | | I am preparing to return to work after birth. When should I start adjusting my routine? | | How can I store breast milk safely after returning to work? | | If milk supply drops after returning to work, how should I adjust? |
| **Model response (Chinese) / 模型回答（中文）** | | 返岗前2到4周可逐步调整作息、练习吸奶和让宝宝适应照护者，但不要突然改变太多。重点是正确含接、有效排奶和休息补水；若红肿热痛、发热或24小时不缓解，应就医。 | | 母乳保存要用清洁容器，标注日期，冷藏或冷冻，并按先存先用原则处理。建议提前调整作息和吸奶安排，母乳储存要清洁、标日期，并按先存先用处理。 | | 返岗后奶量下降可增加有效哺乳和规律吸奶，保证饮水、进食和休息，必要时咨询母乳门诊。重点是正确含接、有效排奶和休息补水；若红肿热痛、发热或24小时不缓解，应就医。 |
| **English translation of model response / 模型回答英文翻译** | | Two to four weeks before returning to work, gradually adjust routines, practice pumping, and help the baby adapt to caregivers. Avoid sudden major changes. Focus on effective milk removal and correct latch. Redness, fever, or no improvement within 24 hours needs care. | | Store milk in clean containers, label the date, refrigerate or freeze properly, and use the oldest milk first. Focus on effective milk removal and correct latch. Redness, fever, or no improvement within 24 hours needs care. | | If supply drops after returning to work, increase effective breastfeeding and regular pumping, maintain fluids, meals, and rest, and consult a lactation clinic if needed. Focus on effective milk removal and correct latch. Redness, fever, or no improvement within 24 hours needs care. |
| **Overall evaluation item / 整体评价项目** | | | **Score / 分数** | | **Comments / 备注** | |
| **Patient Satisfaction / 患者满意度** | | | 5 | |  | |
| **Patient Understandability / 患者可理解性** | | | 4 | |  | |
| **Patient Usefulness / 患者实用性** | | | 5 | |  | |
| **Clinician Correctness / 医生评分：正确性** | | | 5 | |  | |
| **Clinician Completeness / 医生评分：完整性** | | | 5 | |  | |
| **Clinician Readability / 医生评分：可读性** | | | 4 | |  | |
| **Clinician Helpfulness / 医生评分：帮助性** | | | 5 | |  | |
| **Clinician Safety / 医生评分：安全性** | | | 5 | |  | |

**Patient 92**

| **Patient ID / 患者编号** | **Group / 模型组别** | | | | | |
| --- | --- | --- | --- | --- | --- | --- |
| P92 | ChatGPT 5.5 | | | | | |
| **General model response or patient education material (Chinese) / 模型总体回答或患者教育材料（中文）** | | | | | | |
| 产后大量掉发多与激素变化有关，常在产后数月出现，通常属于暂时现象。建议观察变化，保证休息、饮食和清洁护理；若症状持续不缓解或逐渐加重，应联系产科门诊。多数产后脱发会在6到12个月内逐渐改善，期间应避免过度拉扯、烫染和焦虑。请告诉家人和医生，争取照护分担；若有绝望、失控感或伤害想法，应立即就医。若掉发严重、伴乏力心慌、怕热怕冷、月经异常或头皮问题，应检查贫血、甲状腺和皮肤科情况。恢复前应确认恶露基本干净、伤口不疼，并做好避孕；若疼痛、出血或紧张明显，应咨询医生。以上内容为一般健康教育，不能替代线下面诊。 | | | | | | |
| **English translation of the Chinese general response / 中文总体回答英文翻译** | | | | | | |
| Significant hair shedding after birth is often related to hormonal changes and commonly appears several months postpartum. It is usually temporary. Focus on hygiene, comfort, ventilation, and avoiding chills. Fever, palpitations, or marked weakness needs assessment. Most postpartum hair loss improves gradually within 6 to 12 months. Avoid pulling, harsh chemical treatments, and excessive anxiety. Tell family and clinicians how you feel. Hopelessness, loss of control, or harm thoughts need immediate help. Severe hair loss with fatigue, palpitations, heat or cold intolerance, menstrual changes, or scalp problems should prompt checks for anemia, thyroid disease, and dermatologic causes. Keep staple foods, protein, vegetables, fruit, and fluids. Dizziness, weakness, weight loss, or low milk supply needs advice. This is general health education and does not replace an in-person medical assessment. | | | | | | |
| **Item / 项目** | | **Q1 / 问题1** | | **Q2 / 问题2** | | **Q3 / 问题3** |
| **Patient question (Chinese original) / 患者原始问题（中文）** | | 我产后开始大量掉头发，是正常的吗？ | | 产后脱发一般多久会好？ | | 如果掉发很严重，需要查贫血或甲状腺吗？ |
| **English translation of patient question / 患者问题英文翻译** | | I am losing a lot of hair after birth. Is this normal? | | How long does postpartum hair loss usually last? | | If hair loss is severe, should I check for anemia or thyroid problems? |
| **Model response (Chinese) / 模型回答（中文）** | | 产后大量掉发多与激素变化有关，常在产后数月出现，通常属于暂时现象。建议观察变化，保证休息、饮食和清洁护理；若症状持续不缓解或逐渐加重，应联系产科门诊。 | | 多数产后脱发会在6到12个月内逐渐改善，期间应避免过度拉扯、烫染和焦虑。请告诉家人和医生，争取照护分担；若有绝望、失控感或伤害想法，应立即就医。 | | 若掉发严重、伴乏力心慌、怕热怕冷、月经异常或头皮问题，应检查贫血、甲状腺和皮肤科情况。恢复前应确认恶露基本干净、伤口不疼，并做好避孕；若疼痛、出血或紧张明显，应咨询医生。 |
| **English translation of model response / 模型回答英文翻译** | | Significant hair shedding after birth is often related to hormonal changes and commonly appears several months postpartum. It is usually temporary. Focus on hygiene, comfort, ventilation, and avoiding chills. Fever, palpitations, or marked weakness needs assessment. | | Most postpartum hair loss improves gradually within 6 to 12 months. Avoid pulling, harsh chemical treatments, and excessive anxiety. Tell family and clinicians how you feel. Hopelessness, loss of control, or harm thoughts need immediate help. | | Severe hair loss with fatigue, palpitations, heat or cold intolerance, menstrual changes, or scalp problems should prompt checks for anemia, thyroid disease, and dermatologic causes. Keep staple foods, protein, vegetables, fruit, and fluids. Dizziness, weakness, weight loss, or low milk supply needs advice. |
| **Overall evaluation item / 整体评价项目** | | | **Score / 分数** | | **Comments / 备注** | |
| **Patient Satisfaction / 患者满意度** | | | 4 | |  | |
| **Patient Understandability / 患者可理解性** | | | 4 | |  | |
| **Patient Usefulness / 患者实用性** | | | 5 | |  | |
| **Clinician Correctness / 医生评分：正确性** | | | 4 | |  | |
| **Clinician Completeness / 医生评分：完整性** | | | 5 | |  | |
| **Clinician Readability / 医生评分：可读性** | | | 4 | |  | |
| **Clinician Helpfulness / 医生评分：帮助性** | | | 5 | |  | |
| **Clinician Safety / 医生评分：安全性** | | | 5 | |  | |

**Patient 93**

| **Patient ID / 患者编号** | **Group / 模型组别** | | | | | |
| --- | --- | --- | --- | --- | --- | --- |
| P93 | ChatGPT 5.5 | | | | | |
| **General model response or patient education material (Chinese) / 模型总体回答或患者教育材料（中文）** | | | | | | |
| 产后色斑可能随激素稳定逐渐变淡，但日晒会加重，应重视防晒。护理应选择温和产品，避免刺激和来源不明药膏；若红肿、渗液或症状明显，应专科评估。哺乳期可选择成分温和、正规品牌的护肤品和防晒产品，避免使用来源不明或强刺激产品。重点是正确含接、有效排奶和休息补水；若红肿热痛、发热或24小时不缓解，应就医。若皮肤瘙痒、起疹、红肿渗液或影响睡眠，应看皮肤科，说明正在哺乳。重点是正确含接、有效排奶和休息补水；若红肿热痛、发热或24小时不缓解，应就医。以上内容为一般健康教育，不能替代线下面诊。 | | | | | | |
| **English translation of the Chinese general response / 中文总体回答英文翻译** | | | | | | |
| Facial pigmentation after birth may fade as hormones stabilize, but sun exposure can worsen it. Sun protection is important. Use gentle products and avoid unknown ointments. Redness, oozing, severe itching, or systemic symptoms need specialist care. During breastfeeding, gentle skin-care and sunscreen products from reliable brands can be used. Avoid unknown or strongly irritating products. Focus on effective milk removal and correct latch. Redness, fever, or no improvement within 24 hours needs care. Itching, rash, redness, oozing, or sleep disturbance should be assessed by dermatology, and you should mention that you are breastfeeding. Record color, amount, odor, and pad use. Seek care for heavy bleeding, clots, pain, or fever. This is general health education and does not replace an in-person medical assessment. | | | | | | |
| **Item / 项目** | | **Q1 / 问题1** | | **Q2 / 问题2** | | **Q3 / 问题3** |
| **Patient question (Chinese original) / 患者原始问题（中文）** | | 我产后脸上色斑变明显，会自己淡吗？ | | 哺乳期可以使用护肤品或防晒吗？ | | 如果皮肤瘙痒起疹，需要看皮肤科吗？ |
| **English translation of patient question / 患者问题英文翻译** | | My facial pigmentation became more obvious after birth. Will it fade by itself? | | Can I use skin-care products or sunscreen while breastfeeding? | | If my skin itches and develops a rash, should I see a dermatologist? |
| **Model response (Chinese) / 模型回答（中文）** | | 产后色斑可能随激素稳定逐渐变淡，但日晒会加重，应重视防晒。护理应选择温和产品，避免刺激和来源不明药膏；若红肿、渗液或症状明显，应专科评估。 | | 哺乳期可选择成分温和、正规品牌的护肤品和防晒产品，避免使用来源不明或强刺激产品。重点是正确含接、有效排奶和休息补水；若红肿热痛、发热或24小时不缓解，应就医。 | | 若皮肤瘙痒、起疹、红肿渗液或影响睡眠，应看皮肤科，说明正在哺乳。重点是正确含接、有效排奶和休息补水；若红肿热痛、发热或24小时不缓解，应就医。 |
| **English translation of model response / 模型回答英文翻译** | | Facial pigmentation after birth may fade as hormones stabilize, but sun exposure can worsen it. Sun protection is important. Use gentle products and avoid unknown ointments. Redness, oozing, severe itching, or systemic symptoms need specialist care. | | During breastfeeding, gentle skin-care and sunscreen products from reliable brands can be used. Avoid unknown or strongly irritating products. Focus on effective milk removal and correct latch. Redness, fever, or no improvement within 24 hours needs care. | | Itching, rash, redness, oozing, or sleep disturbance should be assessed by dermatology, and you should mention that you are breastfeeding. Record color, amount, odor, and pad use. Seek care for heavy bleeding, clots, pain, or fever. |
| **Overall evaluation item / 整体评价项目** | | | **Score / 分数** | | **Comments / 备注** | |
| **Patient Satisfaction / 患者满意度** | | | 4 | |  | |
| **Patient Understandability / 患者可理解性** | | | 4 | |  | |
| **Patient Usefulness / 患者实用性** | | | 4 | |  | |
| **Clinician Correctness / 医生评分：正确性** | | | 5 | |  | |
| **Clinician Completeness / 医生评分：完整性** | | | 4 | |  | |
| **Clinician Readability / 医生评分：可读性** | | | 4 | |  | |
| **Clinician Helpfulness / 医生评分：帮助性** | | | 4 | |  | |
| **Clinician Safety / 医生评分：安全性** | | | 5 | |  | |

**Patient 94**

| **Patient ID / 患者编号** | **Group / 模型组别** | | | | | |
| --- | --- | --- | --- | --- | --- | --- |
| P94 | ChatGPT 5.5 | | | | | |
| **General model response or patient education material (Chinese) / 模型总体回答或患者教育材料（中文）** | | | | | | |
| 妊娠纹产后通常会从红紫色逐渐变浅，但很难完全消失。护理应选择温和产品，避免刺激和来源不明药膏；若红肿、渗液或症状明显，应专科评估。腹部皮肤松弛可轻柔护理，伤口未愈合或腹痛时不要用力按摩。保持局部清洁干燥，避免抓挠、挤压和自行涂药；若红肿扩大、疼痛或渗液加重，应复查。祛纹产品应避开乳头乳晕和伤口，选择成分明确、刺激性低的产品，不要使用含激素或来源不明产品。重点是正确含接、有效排奶和休息补水；若红肿热痛、发热或24小时不缓解，应就医。以上内容为一般健康教育，不能替代线下面诊。 | | | | | | |
| **English translation of the Chinese general response / 中文总体回答英文翻译** | | | | | | |
| Stretch marks often fade from red or purple to lighter colors after birth, but they rarely disappear completely. Record color, amount, odor, and pad use. Seek care for heavy bleeding, clots, pain, or fever. Loose abdominal skin can be cared for gently. Do not massage forcefully if wounds are unhealed or abdominal pain is present. Record color, amount, odor, and pad use. Seek care for heavy bleeding, clots, pain, or fever. Anti-stretch-mark products should be kept away from the nipple, areola, and wounds. Choose products with clear ingredients and low irritation, and avoid hormones or unknown products. Record color, amount, odor, and pad use. Seek care for heavy bleeding, clots, pain, or fever. This is general health education and does not replace an in-person medical assessment. | | | | | | |
| **Item / 项目** | | **Q1 / 问题1** | | **Q2 / 问题2** | | **Q3 / 问题3** |
| **Patient question (Chinese original) / 患者原始问题（中文）** | | 妊娠纹产后还能变淡吗？ | | 产后腹部皮肤松弛可以按摩吗？ | | 使用祛纹产品会不会影响哺乳？ |
| **English translation of patient question / 患者问题英文翻译** | | Can stretch marks become lighter after birth? | | Can I massage loose abdominal skin after birth? | | Will anti-stretch-mark products affect breastfeeding? |
| **Model response (Chinese) / 模型回答（中文）** | | 妊娠纹产后通常会从红紫色逐渐变浅，但很难完全消失。护理应选择温和产品，避免刺激和来源不明药膏；若红肿、渗液或症状明显，应专科评估。 | | 腹部皮肤松弛可轻柔护理，伤口未愈合或腹痛时不要用力按摩。保持局部清洁干燥，避免抓挠、挤压和自行涂药；若红肿扩大、疼痛或渗液加重，应复查。 | | 祛纹产品应避开乳头乳晕和伤口，选择成分明确、刺激性低的产品，不要使用含激素或来源不明产品。重点是正确含接、有效排奶和休息补水；若红肿热痛、发热或24小时不缓解，应就医。 |
| **English translation of model response / 模型回答英文翻译** | | Stretch marks often fade from red or purple to lighter colors after birth, but they rarely disappear completely. Record color, amount, odor, and pad use. Seek care for heavy bleeding, clots, pain, or fever. | | Loose abdominal skin can be cared for gently. Do not massage forcefully if wounds are unhealed or abdominal pain is present. Record color, amount, odor, and pad use. Seek care for heavy bleeding, clots, pain, or fever. | | Anti-stretch-mark products should be kept away from the nipple, areola, and wounds. Choose products with clear ingredients and low irritation, and avoid hormones or unknown products. Record color, amount, odor, and pad use. Seek care for heavy bleeding, clots, pain, or fever. |
| **Overall evaluation item / 整体评价项目** | | | **Score / 分数** | | **Comments / 备注** | |
| **Patient Satisfaction / 患者满意度** | | | 5 | |  | |
| **Patient Understandability / 患者可理解性** | | | 4 | |  | |
| **Patient Usefulness / 患者实用性** | | | 4 | |  | |
| **Clinician Correctness / 医生评分：正确性** | | | 5 | |  | |
| **Clinician Completeness / 医生评分：完整性** | | | 5 | |  | |
| **Clinician Readability / 医生评分：可读性** | | | 4 | |  | |
| **Clinician Helpfulness / 医生评分：帮助性** | | | 5 | |  | |
| **Clinician Safety / 医生评分：安全性** | | | 5 | |  | |

**Patient 95**

| **Patient ID / 患者编号** | **Group / 模型组别** | | | | | |
| --- | --- | --- | --- | --- | --- | --- |
| P95 | ChatGPT 5.5 | | | | | |
| **General model response or patient education material (Chinese) / 模型总体回答或患者教育材料（中文）** | | | | | | |
| 洗澡后怕冷不代表不能洗，可能与水温、室温、出汗和疲劳有关，应注意保暖和及时擦干。以清洁、舒适和避免受凉为原则，保持通风；若伴发热、心慌或明显乏力，应评估。产后一般建议淋浴，不建议盆浴或长时间泡澡，尤其恶露未净和伤口未愈合时。建议记录颜色、量和气味，避免劳累；若1小时湿透卫生巾、血块增多、腹痛或发热，应就医。剖宫产切口未完全愈合前，应避免揉搓和浸泡，洗后轻轻按干，若有渗液红肿应先咨询医生。保持局部清洁干燥，避免抓挠、挤压和自行涂药；若红肿扩大、疼痛或渗液加重，应复查。以上内容为一般健康教育，不能替代线下面诊。 | | | | | | |
| **English translation of the Chinese general response / 中文总体回答英文翻译** | | | | | | |
| Feeling cold after showering does not mean you cannot wash. It may relate to water temperature, room temperature, sweating, and fatigue. Keep warm and dry yourself promptly. Recheck temperature and watch lochia, breasts, urination, and wounds. Fever at or above 38°C needs care. A shower is generally preferred after birth. Tub bathing or long soaking is not recommended, especially before lochia stops and wounds heal. Record color, amount, odor, and pad use. Seek care for heavy bleeding, clots, pain, or fever. Before a cesarean incision is fully healed, avoid rubbing or soaking it. Pat it dry after showering, and consult a clinician first if there is drainage, redness, or swelling. Record color, amount, odor, and pad use. Seek care for heavy bleeding, clots, pain, or fever. This is general health education and does not replace an in-person medical assessment. | | | | | | |
| **Item / 项目** | | **Q1 / 问题1** | | **Q2 / 问题2** | | **Q3 / 问题3** |
| **Patient question (Chinese original) / 患者原始问题（中文）** | | 坐月子洗澡后觉得怕冷，是不是不能洗？ | | 产后洗澡应该选择淋浴还是盆浴？ | | 剖宫产切口没完全愈合前洗澡要注意什么？ |
| **English translation of patient question / 患者问题英文翻译** | | I felt cold after showering during the postpartum month. Does that mean I should not wash? | | Should I take a shower or a bath after birth? | | What should I pay attention to if the cesarean incision has not fully healed before showering? |
| **Model response (Chinese) / 模型回答（中文）** | | 洗澡后怕冷不代表不能洗，可能与水温、室温、出汗和疲劳有关，应注意保暖和及时擦干。以清洁、舒适和避免受凉为原则，保持通风；若伴发热、心慌或明显乏力，应评估。 | | 产后一般建议淋浴，不建议盆浴或长时间泡澡，尤其恶露未净和伤口未愈合时。建议记录颜色、量和气味，避免劳累；若1小时湿透卫生巾、血块增多、腹痛或发热，应就医。 | | 剖宫产切口未完全愈合前，应避免揉搓和浸泡，洗后轻轻按干，若有渗液红肿应先咨询医生。保持局部清洁干燥，避免抓挠、挤压和自行涂药；若红肿扩大、疼痛或渗液加重，应复查。 |
| **English translation of model response / 模型回答英文翻译** | | Feeling cold after showering does not mean you cannot wash. It may relate to water temperature, room temperature, sweating, and fatigue. Keep warm and dry yourself promptly. Recheck temperature and watch lochia, breasts, urination, and wounds. Fever at or above 38°C needs care. | | A shower is generally preferred after birth. Tub bathing or long soaking is not recommended, especially before lochia stops and wounds heal. Record color, amount, odor, and pad use. Seek care for heavy bleeding, clots, pain, or fever. | | Before a cesarean incision is fully healed, avoid rubbing or soaking it. Pat it dry after showering, and consult a clinician first if there is drainage, redness, or swelling. Record color, amount, odor, and pad use. Seek care for heavy bleeding, clots, pain, or fever. |
| **Overall evaluation item / 整体评价项目** | | | **Score / 分数** | | **Comments / 备注** | |
| **Patient Satisfaction / 患者满意度** | | | 4 | |  | |
| **Patient Understandability / 患者可理解性** | | | 4 | |  | |
| **Patient Usefulness / 患者实用性** | | | 4 | |  | |
| **Clinician Correctness / 医生评分：正确性** | | | 5 | |  | |
| **Clinician Completeness / 医生评分：完整性** | | | 5 | |  | |
| **Clinician Readability / 医生评分：可读性** | | | 4 | |  | |
| **Clinician Helpfulness / 医生评分：帮助性** | | | 5 | |  | |
| **Clinician Safety / 医生评分：安全性** | | | 5 | |  | |

**Patient 96**

| **Patient ID / 患者编号** | **Group / 模型组别** | | | | | |
| --- | --- | --- | --- | --- | --- | --- |
| P96 | ChatGPT 5.5 | | | | | |
| **General model response or patient education material (Chinese) / 模型总体回答或患者教育材料（中文）** | | | | | | |
| 产后可以使用空调，重点是避免直吹、温差过大和出汗后受凉。以清洁、舒适和避免受凉为原则，保持通风；若伴发热、心慌或明显乏力，应评估。室温一般保持舒适即可，常用范围约26℃左右，可根据体感和宝宝情况调整。请告诉家人和医生，争取照护分担；若有绝望、失控感或伤害想法，应立即就医。吹空调后关节酸痛不一定是受凉，也可能与疲劳和姿势有关；若疼痛明显、红肿发热或持续，应就医。建议复测体温，并观察恶露、乳房、尿痛和伤口；若体温≥38℃、寒战或精神差，应就医。以上内容为一般健康教育，不能替代线下面诊。 | | | | | | |
| **English translation of the Chinese general response / 中文总体回答英文翻译** | | | | | | |
| Air conditioning can be used after birth. Avoid direct airflow, large temperature differences, and getting chilled after sweating. Recheck temperature and watch lochia, breasts, urination, and wounds. Fever at or above 38°C needs care. The room should feel comfortable, often around 26°C, with adjustment based on the mother and baby. Recheck temperature and watch lochia, breasts, urination, and wounds. Fever at or above 38°C needs care. Joint aches after air conditioning are not necessarily from catching cold. Fatigue and posture may contribute. Obvious, swollen, hot, or persistent joint pain needs medical assessment. Focus on hygiene, comfort, ventilation, and avoiding chills. Fever, palpitations, or marked weakness needs assessment. This is general health education and does not replace an in-person medical assessment. | | | | | | |
| **Item / 项目** | | **Q1 / 问题1** | | **Q2 / 问题2** | | **Q3 / 问题3** |
| **Patient question (Chinese original) / 患者原始问题（中文）** | | 产后可以吹空调睡觉吗？ | | 空调温度多少比较合适？ | | 如果吹空调后关节酸痛，是受凉了吗？ |
| **English translation of patient question / 患者问题英文翻译** | | Can I sleep with air conditioning after birth? | | What air-conditioning temperature is suitable? | | If my joints ache after air conditioning, did I catch cold? |
| **Model response (Chinese) / 模型回答（中文）** | | 产后可以使用空调，重点是避免直吹、温差过大和出汗后受凉。以清洁、舒适和避免受凉为原则，保持通风；若伴发热、心慌或明显乏力，应评估。 | | 室温一般保持舒适即可，常用范围约26℃左右，可根据体感和宝宝情况调整。请告诉家人和医生，争取照护分担；若有绝望、失控感或伤害想法，应立即就医。 | | 吹空调后关节酸痛不一定是受凉，也可能与疲劳和姿势有关；若疼痛明显、红肿发热或持续，应就医。建议复测体温，并观察恶露、乳房、尿痛和伤口；若体温≥38℃、寒战或精神差，应就医。 |
| **English translation of model response / 模型回答英文翻译** | | Air conditioning can be used after birth. Avoid direct airflow, large temperature differences, and getting chilled after sweating. Recheck temperature and watch lochia, breasts, urination, and wounds. Fever at or above 38°C needs care. | | The room should feel comfortable, often around 26°C, with adjustment based on the mother and baby. Recheck temperature and watch lochia, breasts, urination, and wounds. Fever at or above 38°C needs care. | | Joint aches after air conditioning are not necessarily from catching cold. Fatigue and posture may contribute. Obvious, swollen, hot, or persistent joint pain needs medical assessment. Focus on hygiene, comfort, ventilation, and avoiding chills. Fever, palpitations, or marked weakness needs assessment. |
| **Overall evaluation item / 整体评价项目** | | | **Score / 分数** | | **Comments / 备注** | |
| **Patient Satisfaction / 患者满意度** | | | 4 | |  | |
| **Patient Understandability / 患者可理解性** | | | 4 | |  | |
| **Patient Usefulness / 患者实用性** | | | 4 | |  | |
| **Clinician Correctness / 医生评分：正确性** | | | 4 | |  | |
| **Clinician Completeness / 医生评分：完整性** | | | 4 | |  | |
| **Clinician Readability / 医生评分：可读性** | | | 4 | |  | |
| **Clinician Helpfulness / 医生评分：帮助性** | | | 4 | |  | |
| **Clinician Safety / 医生评分：安全性** | | | 5 | |  | |

**Patient 97**

| **Patient ID / 患者编号** | **Group / 模型组别** | | | | | |
| --- | --- | --- | --- | --- | --- | --- |
| P97 | ChatGPT 5.5 | | | | | |
| **General model response or patient education material (Chinese) / 模型总体回答或患者教育材料（中文）** | | | | | | |
| 坐月子不必完全不能吃水果，可选择新鲜、清洁、适量的水果，胃肠敏感者可吃常温水果。饮食要保证主食、蛋白质、蔬菜水果和水分；若头晕乏力、体重下降或奶量减少，应咨询医生。辛辣食物不必绝对禁止，但伤口疼痛、便秘、痔疮或胃肠不适时应少吃。保持局部清洁干燥，避免抓挠、挤压和自行涂药；若红肿扩大、疼痛或渗液加重，应复查。宝宝胀气不等于必须停止母乳，可先观察食物和症状关系，必要时咨询儿科或母乳门诊。请告诉家人和医生，争取照护分担；若有绝望、失控感或伤害想法，应立即就医。以上内容为一般健康教育，不能替代线下面诊。 | | | | | | |
| **English translation of the Chinese general response / 中文总体回答英文翻译** | | | | | | |
| Fruit does not need to be completely avoided during confinement. Choose fresh, clean, moderate portions; room-temperature fruit may suit sensitive stomachs. Recheck temperature and watch lochia, breasts, urination, and wounds. Fever at or above 38°C needs care. Spicy food is not absolutely forbidden, but it should be limited if wound pain, constipation, hemorrhoids, or stomach discomfort occurs. Keep the area clean and dry. Seek review if redness, pain, drainage, or wound opening worsens. Baby gas does not mean breastfeeding must stop. Observe whether a food is clearly related to symptoms and consult pediatrics or a lactation clinic if needed. Focus on effective milk removal and correct latch. Redness, fever, or no improvement within 24 hours needs care. This is general health education and does not replace an in-person medical assessment. | | | | | | |
| **Item / 项目** | | **Q1 / 问题1** | | **Q2 / 问题2** | | **Q3 / 问题3** |
| **Patient question (Chinese original) / 患者原始问题（中文）** | | 坐月子是不是不能吃凉的水果？ | | 产后饮食需要完全忌口辛辣吗？ | | 如果吃了某些食物后宝宝胀气，是不是要停止母乳？ |
| **English translation of patient question / 患者问题英文翻译** | | During confinement, must I avoid cold fruit? | | Do I need to completely avoid spicy food after birth? | | If the baby becomes gassy after I eat certain foods, should I stop breastfeeding? |
| **Model response (Chinese) / 模型回答（中文）** | | 坐月子不必完全不能吃水果，可选择新鲜、清洁、适量的水果，胃肠敏感者可吃常温水果。饮食要保证主食、蛋白质、蔬菜水果和水分；若头晕乏力、体重下降或奶量减少，应咨询医生。 | | 辛辣食物不必绝对禁止，但伤口疼痛、便秘、痔疮或胃肠不适时应少吃。保持局部清洁干燥，避免抓挠、挤压和自行涂药；若红肿扩大、疼痛或渗液加重，应复查。 | | 宝宝胀气不等于必须停止母乳，可先观察食物和症状关系，必要时咨询儿科或母乳门诊。请告诉家人和医生，争取照护分担；若有绝望、失控感或伤害想法，应立即就医。 |
| **English translation of model response / 模型回答英文翻译** | | Fruit does not need to be completely avoided during confinement. Choose fresh, clean, moderate portions; room-temperature fruit may suit sensitive stomachs. Recheck temperature and watch lochia, breasts, urination, and wounds. Fever at or above 38°C needs care. | | Spicy food is not absolutely forbidden, but it should be limited if wound pain, constipation, hemorrhoids, or stomach discomfort occurs. Keep the area clean and dry. Seek review if redness, pain, drainage, or wound opening worsens. | | Baby gas does not mean breastfeeding must stop. Observe whether a food is clearly related to symptoms and consult pediatrics or a lactation clinic if needed. Focus on effective milk removal and correct latch. Redness, fever, or no improvement within 24 hours needs care. |
| **Overall evaluation item / 整体评价项目** | | | **Score / 分数** | | **Comments / 备注** | |
| **Patient Satisfaction / 患者满意度** | | | 4 | |  | |
| **Patient Understandability / 患者可理解性** | | | 4 | |  | |
| **Patient Usefulness / 患者实用性** | | | 5 | |  | |
| **Clinician Correctness / 医生评分：正确性** | | | 5 | |  | |
| **Clinician Completeness / 医生评分：完整性** | | | 5 | |  | |
| **Clinician Readability / 医生评分：可读性** | | | 4 | |  | |
| **Clinician Helpfulness / 医生评分：帮助性** | | | 5 | |  | |
| **Clinician Safety / 医生评分：安全性** | | | 5 | |  | |

**Patient 98**

| **Patient ID / 患者编号** | **Group / 模型组别** | | | | | |
| --- | --- | --- | --- | --- | --- | --- |
| P98 | ChatGPT 5.5 | | | | | |
| **General model response or patient education material (Chinese) / 模型总体回答或患者教育材料（中文）** | | | | | | |
| 产后长途返程应根据分娩方式、出血、伤口、血压和体力决定，最好先咨询医生。建议记录颜色、量和气味，避免劳累；若1小时湿透卫生巾、血块增多、腹痛或发热，应就医。长时间久坐会增加血栓风险，旅途中应定时下车活动、补水并避免久坐不动。不要按摩肿痛肢体；若单侧腿肿痛、胸闷气短、胸痛或晕厥，应立即就医。若旅途中恶露明显增多、头晕心慌、胸闷气短、腿肿痛，应停止行程并尽快就医。建议记录颜色、量和气味，避免劳累；若1小时湿透卫生巾、血块增多、腹痛或发热，应就医。以上内容为一般健康教育，不能替代线下面诊。 | | | | | | |
| **English translation of the Chinese general response / 中文总体回答英文翻译** | | | | | | |
| Long-distance travel after birth should depend on delivery mode, bleeding, wounds, blood pressure, and physical strength. It is best to consult a clinician first. Record color, amount, odor, and pad use. Seek care for heavy bleeding, clots, pain, or fever. Prolonged sitting can increase thrombosis risk. Take breaks to walk, drink water, and avoid staying still for long periods. Resume only when wounds and lochia are stable, and use contraception. Pain or bleeding needs medical advice. If lochia increases clearly, dizziness, palpitations, chest tightness, shortness of breath, or leg swelling and pain occur during travel, stop and seek medical care promptly. Record color, amount, odor, and pad use. Seek care for heavy bleeding, clots, pain, or fever. This is general health education and does not replace an in-person medical assessment. | | | | | | |
| **Item / 项目** | | **Q1 / 问题1** | | **Q2 / 问题2** | | **Q3 / 问题3** |
| **Patient question (Chinese original) / 患者原始问题（中文）** | | 产后多久可以坐长途车回老家？ | | 长时间坐车会不会增加血栓风险？ | | 旅途中恶露增多或头晕应该怎么办？ |
| **English translation of patient question / 患者问题英文翻译** | | How soon after birth can I take a long-distance car ride home? | | Will sitting in a car for a long time increase the risk of thrombosis? | | What should I do if lochia increases or I feel dizzy during travel? |
| **Model response (Chinese) / 模型回答（中文）** | | 产后长途返程应根据分娩方式、出血、伤口、血压和体力决定，最好先咨询医生。建议记录颜色、量和气味，避免劳累；若1小时湿透卫生巾、血块增多、腹痛或发热，应就医。 | | 长时间久坐会增加血栓风险，旅途中应定时下车活动、补水并避免久坐不动。不要按摩肿痛肢体；若单侧腿肿痛、胸闷气短、胸痛或晕厥，应立即就医。 | | 若旅途中恶露明显增多、头晕心慌、胸闷气短、腿肿痛，应停止行程并尽快就医。建议记录颜色、量和气味，避免劳累；若1小时湿透卫生巾、血块增多、腹痛或发热，应就医。 |
| **English translation of model response / 模型回答英文翻译** | | Long-distance travel after birth should depend on delivery mode, bleeding, wounds, blood pressure, and physical strength. It is best to consult a clinician first. Record color, amount, odor, and pad use. Seek care for heavy bleeding, clots, pain, or fever. | | Prolonged sitting can increase thrombosis risk. Take breaks to walk, drink water, and avoid staying still for long periods. Resume only when wounds and lochia are stable, and use contraception. Pain or bleeding needs medical advice. | | If lochia increases clearly, dizziness, palpitations, chest tightness, shortness of breath, or leg swelling and pain occur during travel, stop and seek medical care promptly. Record color, amount, odor, and pad use. Seek care for heavy bleeding, clots, pain, or fever. |
| **Overall evaluation item / 整体评价项目** | | | **Score / 分数** | | **Comments / 备注** | |
| **Patient Satisfaction / 患者满意度** | | | 4 | |  | |
| **Patient Understandability / 患者可理解性** | | | 4 | |  | |
| **Patient Usefulness / 患者实用性** | | | 4 | |  | |
| **Clinician Correctness / 医生评分：正确性** | | | 5 | |  | |
| **Clinician Completeness / 医生评分：完整性** | | | 4 | |  | |
| **Clinician Readability / 医生评分：可读性** | | | 4 | |  | |
| **Clinician Helpfulness / 医生评分：帮助性** | | | 4 | |  | |
| **Clinician Safety / 医生评分：安全性** | | | 5 | |  | |

**Patient 99**

| **Patient ID / 患者编号** | **Group / 模型组别** | | | | | |
| --- | --- | --- | --- | --- | --- | --- |
| P99 | ChatGPT 5.5 | | | | | |
| **General model response or patient education material (Chinese) / 模型总体回答或患者教育材料（中文）** | | | | | | |
| 剖宫产后开车应等疼痛可控、反应灵活、能安全踩刹车并不受药物影响后再考虑。哺乳期用药应告知医生正在哺乳，按医嘱选择；不要自行加量或长期使用。急刹车可能牵拉腹部切口，早期建议避免独自长时间驾驶。保持局部清洁干燥，避免抓挠、挤压和自行涂药；若红肿扩大、疼痛或渗液加重，应复查。开车后轻微不适可休息观察；若腹痛明显、切口疼痛加重、恶露增多或头晕，应复查。建议记录颜色、量和气味，避免劳累；若1小时湿透卫生巾、血块增多、腹痛或发热，应就医。以上内容为一般健康教育，不能替代线下面诊。 | | | | | | |
| **English translation of the Chinese general response / 中文总体回答英文翻译** | | | | | | |
| After a cesarean birth, driving should wait until pain is controlled, reactions are quick, braking is safe, and medicines do not impair alertness. Tell clinicians you are breastfeeding and use medicines as advised. Do not increase doses or self-medicate long term. Sudden braking may pull the abdominal incision, so long solo driving should be avoided early on. Keep the area clean and dry. Seek review if redness, pain, drainage, or wound opening worsens. Mild discomfort after driving can be observed with rest. Marked abdominal pain, increased incision pain, heavier lochia, or dizziness should be checked. Record color, amount, odor, and pad use. Seek care for heavy bleeding, clots, pain, or fever. This is general health education and does not replace an in-person medical assessment. | | | | | | |
| **Item / 项目** | | **Q1 / 问题1** | | **Q2 / 问题2** | | **Q3 / 问题3** |
| **Patient question (Chinese original) / 患者原始问题（中文）** | | 剖宫产后多久可以自己开车？ | | 急刹车会不会牵拉切口？ | | 如果开车后腹部疼痛，需要休息还是复查？ |
| **English translation of patient question / 患者问题英文翻译** | | How soon can I drive by myself after a cesarean birth? | | Will sudden braking pull the incision? | | If I have abdominal pain after driving, should I rest or get checked? |
| **Model response (Chinese) / 模型回答（中文）** | | 剖宫产后开车应等疼痛可控、反应灵活、能安全踩刹车并不受药物影响后再考虑。哺乳期用药应告知医生正在哺乳，按医嘱选择；不要自行加量或长期使用。 | | 急刹车可能牵拉腹部切口，早期建议避免独自长时间驾驶。保持局部清洁干燥，避免抓挠、挤压和自行涂药；若红肿扩大、疼痛或渗液加重，应复查。 | | 开车后轻微不适可休息观察；若腹痛明显、切口疼痛加重、恶露增多或头晕，应复查。建议记录颜色、量和气味，避免劳累；若1小时湿透卫生巾、血块增多、腹痛或发热，应就医。 |
| **English translation of model response / 模型回答英文翻译** | | After a cesarean birth, driving should wait until pain is controlled, reactions are quick, braking is safe, and medicines do not impair alertness. Tell clinicians you are breastfeeding and use medicines as advised. Do not increase doses or self-medicate long term. | | Sudden braking may pull the abdominal incision, so long solo driving should be avoided early on. Keep the area clean and dry. Seek review if redness, pain, drainage, or wound opening worsens. | | Mild discomfort after driving can be observed with rest. Marked abdominal pain, increased incision pain, heavier lochia, or dizziness should be checked. Record color, amount, odor, and pad use. Seek care for heavy bleeding, clots, pain, or fever. |
| **Overall evaluation item / 整体评价项目** | | | **Score / 分数** | | **Comments / 备注** | |
| **Patient Satisfaction / 患者满意度** | | | 4 | |  | |
| **Patient Understandability / 患者可理解性** | | | 4 | |  | |
| **Patient Usefulness / 患者实用性** | | | 4 | |  | |
| **Clinician Correctness / 医生评分：正确性** | | | 5 | |  | |
| **Clinician Completeness / 医生评分：完整性** | | | 4 | |  | |
| **Clinician Readability / 医生评分：可读性** | | | 4 | |  | |
| **Clinician Helpfulness / 医生评分：帮助性** | | | 5 | |  | |
| **Clinician Safety / 医生评分：安全性** | | | 5 | |  | |

**Patient 100**

| **Patient ID / 患者编号** | **Group / 模型组别** | | | | | |
| --- | --- | --- | --- | --- | --- | --- |
| P100 | ChatGPT 5.5 | | | | | |
| **General model response or patient education material (Chinese) / 模型总体回答或患者教育材料（中文）** | | | | | | |
| 产后提重物和家务应逐步恢复，早期不建议搬重物、拖地用力或长时间弯腰。建议观察变化，保证休息、饮食和清洁护理；若症状持续不缓解或逐渐加重，应联系产科门诊。抱大宝可能增加腹压，对盆底、腹直肌和切口恢复有影响，应尽量坐着抱、缩短时间。保持局部清洁干燥，避免抓挠、挤压和自行涂药；若红肿扩大、疼痛或渗液加重，应复查。若提重物后下坠感、漏尿、腹痛、切口疼或恶露增多，应减少活动并做产后或盆底评估。建议记录颜色、量和气味，避免劳累；若1小时湿透卫生巾、血块增多、腹痛或发热，应就医。以上内容为一般健康教育，不能替代线下面诊。 | | | | | | |
| **English translation of the Chinese general response / 中文总体回答英文翻译** | | | | | | |
| Lifting heavy objects and housework should resume gradually after birth. Avoid heavy carrying, forceful mopping, and long bending early on. Plan routines and pumping in advance. Store milk cleanly, label dates, and use the oldest milk first. Holding an older child may increase abdominal pressure and affect pelvic floor, abdominal, and incision recovery. Sit while holding and keep it brief if possible. Keep the area clean and dry. Seek review if redness, pain, drainage, or wound opening worsens. If heaviness, leakage, abdominal pain, incision pain, or increased lochia appears after lifting, reduce activity and seek postpartum or pelvic floor assessment. Record color, amount, odor, and pad use. Seek care for heavy bleeding, clots, pain, or fever. This is general health education and does not replace an in-person medical assessment. | | | | | | |
| **Item / 项目** | | **Q1 / 问题1** | | **Q2 / 问题2** | | **Q3 / 问题3** |
| **Patient question (Chinese original) / 患者原始问题（中文）** | | 产后多久可以提重物或做家务？ | | 抱大宝会不会影响盆底和切口恢复？ | | 如果提重物后下坠感明显，需要减少活动吗？ |
| **English translation of patient question / 患者问题英文翻译** | | How soon after birth can I lift heavy objects or do housework? | | Will holding my older child affect pelvic floor or incision recovery? | | If heaviness becomes obvious after lifting, should I reduce activity? |
| **Model response (Chinese) / 模型回答（中文）** | | 产后提重物和家务应逐步恢复，早期不建议搬重物、拖地用力或长时间弯腰。建议观察变化，保证休息、饮食和清洁护理；若症状持续不缓解或逐渐加重，应联系产科门诊。 | | 抱大宝可能增加腹压，对盆底、腹直肌和切口恢复有影响，应尽量坐着抱、缩短时间。保持局部清洁干燥，避免抓挠、挤压和自行涂药；若红肿扩大、疼痛或渗液加重，应复查。 | | 若提重物后下坠感、漏尿、腹痛、切口疼或恶露增多，应减少活动并做产后或盆底评估。建议记录颜色、量和气味，避免劳累；若1小时湿透卫生巾、血块增多、腹痛或发热，应就医。 |
| **English translation of model response / 模型回答英文翻译** | | Lifting heavy objects and housework should resume gradually after birth. Avoid heavy carrying, forceful mopping, and long bending early on. Plan routines and pumping in advance. Store milk cleanly, label dates, and use the oldest milk first. | | Holding an older child may increase abdominal pressure and affect pelvic floor, abdominal, and incision recovery. Sit while holding and keep it brief if possible. Keep the area clean and dry. Seek review if redness, pain, drainage, or wound opening worsens. | | If heaviness, leakage, abdominal pain, incision pain, or increased lochia appears after lifting, reduce activity and seek postpartum or pelvic floor assessment. Record color, amount, odor, and pad use. Seek care for heavy bleeding, clots, pain, or fever. |
| **Overall evaluation item / 整体评价项目** | | | **Score / 分数** | | **Comments / 备注** | |
| **Patient Satisfaction / 患者满意度** | | | 4 | |  | |
| **Patient Understandability / 患者可理解性** | | | 4 | |  | |
| **Patient Usefulness / 患者实用性** | | | 4 | |  | |
| **Clinician Correctness / 医生评分：正确性** | | | 4 | |  | |
| **Clinician Completeness / 医生评分：完整性** | | | 5 | |  | |
| **Clinician Readability / 医生评分：可读性** | | | 4 | |  | |
| **Clinician Helpfulness / 医生评分：帮助性** | | | 5 | |  | |
| **Clinician Safety / 医生评分：安全性** | | | 4 | |  | |

**Patient 101**

| **Patient ID / 患者编号** | **Group / 模型组别** | | | | | |
| --- | --- | --- | --- | --- | --- | --- |
| P101 | ChatGPT 5.5 | | | | | |
| **General model response or patient education material (Chinese) / 模型总体回答或患者教育材料（中文）** | | | | | | |
| 剖宫产后再次怀孕的安全间隔应听从医生建议，通常不建议间隔过短。建议观察变化，保证休息、饮食和清洁护理；若症状持续不缓解或逐渐加重，应联系产科门诊。两次妊娠间隔太短可能增加子宫疤痕相关风险、早产、贫血和母体恢复不足。保持局部清洁干燥，避免抓挠、挤压和自行涂药；若红肿扩大、疼痛或渗液加重，应复查。计划再孕前建议做孕前咨询，结合手术记录、月经恢复和必要的超声评估子宫疤痕情况。保持局部清洁干燥，避免抓挠、挤压和自行涂药；若红肿扩大、疼痛或渗液加重，应复查。以上内容为一般健康教育，不能替代线下面诊。 | | | | | | |
| **English translation of the Chinese general response / 中文总体回答英文翻译** | | | | | | |
| The safe interval before another pregnancy after a cesarean birth should follow medical advice, and a very short interval is generally not recommended. Observe changes and maintain rest, nutrition, and hygiene. Persistent or worsening symptoms need obstetric advice. A short interpregnancy interval may increase risks related to the uterine scar, preterm birth, anemia, and incomplete maternal recovery. Keep the area clean and dry. Seek review if redness, pain, drainage, or wound opening worsens. Before planning another pregnancy, preconception counseling is advised, using the operative record, menstrual recovery, and ultrasound assessment of the uterine scar if needed. Keep the area clean and dry. Seek review if redness, pain, drainage, or wound opening worsens. This is general health education and does not replace an in-person medical assessment. | | | | | | |
| **Item / 项目** | | **Q1 / 问题1** | | **Q2 / 问题2** | | **Q3 / 问题3** |
| **Patient question (Chinese original) / 患者原始问题（中文）** | | 剖宫产后多久可以再次怀孕比较安全？ | | 两次怀孕间隔太短会有什么风险？ | | 计划再孕前需要检查子宫疤痕吗？ |
| **English translation of patient question / 患者问题英文翻译** | | How long after a cesarean birth is it safer to become pregnant again? | | What risks are associated with a very short interval between pregnancies? | | Before planning another pregnancy, do I need to check the uterine scar? |
| **Model response (Chinese) / 模型回答（中文）** | | 剖宫产后再次怀孕的安全间隔应听从医生建议，通常不建议间隔过短。建议观察变化，保证休息、饮食和清洁护理；若症状持续不缓解或逐渐加重，应联系产科门诊。 | | 两次妊娠间隔太短可能增加子宫疤痕相关风险、早产、贫血和母体恢复不足。保持局部清洁干燥，避免抓挠、挤压和自行涂药；若红肿扩大、疼痛或渗液加重，应复查。 | | 计划再孕前建议做孕前咨询，结合手术记录、月经恢复和必要的超声评估子宫疤痕情况。保持局部清洁干燥，避免抓挠、挤压和自行涂药；若红肿扩大、疼痛或渗液加重，应复查。 |
| **English translation of model response / 模型回答英文翻译** | | The safe interval before another pregnancy after a cesarean birth should follow medical advice, and a very short interval is generally not recommended. Observe changes and maintain rest, nutrition, and hygiene. Persistent or worsening symptoms need obstetric advice. | | A short interpregnancy interval may increase risks related to the uterine scar, preterm birth, anemia, and incomplete maternal recovery. Keep the area clean and dry. Seek review if redness, pain, drainage, or wound opening worsens. | | Before planning another pregnancy, preconception counseling is advised, using the operative record, menstrual recovery, and ultrasound assessment of the uterine scar if needed. Keep the area clean and dry. Seek review if redness, pain, drainage, or wound opening worsens. |
| **Overall evaluation item / 整体评价项目** | | | **Score / 分数** | | **Comments / 备注** | |
| **Patient Satisfaction / 患者满意度** | | | 4 | |  | |
| **Patient Understandability / 患者可理解性** | | | 4 | |  | |
| **Patient Usefulness / 患者实用性** | | | 5 | |  | |
| **Clinician Correctness / 医生评分：正确性** | | | 5 | |  | |
| **Clinician Completeness / 医生评分：完整性** | | | 5 | |  | |
| **Clinician Readability / 医生评分：可读性** | | | 4 | |  | |
| **Clinician Helpfulness / 医生评分：帮助性** | | | 5 | |  | |
| **Clinician Safety / 医生评分：安全性** | | | 5 | |  | |

**Patient 102**

| **Patient ID / 患者编号** | **Group / 模型组别** | | | | | |
| --- | --- | --- | --- | --- | --- | --- |
| P102 | ChatGPT 5.5 | | | | | |
| **General model response or patient education material (Chinese) / 模型总体回答或患者教育材料（中文）** | | | | | | |
| 第一胎剖宫产后下一胎是否能顺产，要看上次剖宫产原因、切口类型、孕期情况和医院条件。保持局部清洁干燥，避免抓挠、挤压和自行涂药；若红肿扩大、疼痛或渗液加重，应复查。疤痕厚度可作为参考，但不能单独决定分娩方式，还要评估胎儿大小、胎位和子宫破裂风险。保持局部清洁干燥，避免抓挠、挤压和自行涂药；若红肿扩大、疼痛或渗液加重，应复查。备孕或孕早期应带上次手术记录和出院资料给医生，有助于制定分娩计划。建议观察变化，保证休息、饮食和清洁护理；若症状持续不缓解或逐渐加重，应联系产科门诊。以上内容为一般健康教育，不能替代线下面诊。 | | | | | | |
| **English translation of the Chinese general response / 中文总体回答英文翻译** | | | | | | |
| Vaginal birth after a previous cesarean may be possible depending on the reason for the first cesarean, incision type, current pregnancy, and hospital resources. Keep the area clean and dry. Seek review if redness, pain, drainage, or wound opening worsens. Scar thickness is only one reference and cannot alone determine delivery mode. Fetal size, position, and uterine rupture risk also matter. Keep the area clean and dry. Seek review if redness, pain, drainage, or wound opening worsens. Bring the previous operative and discharge records before pregnancy or early in pregnancy so the clinician can plan care. Record color, amount, odor, and pad use. Seek care for heavy bleeding, clots, pain, or fever. This is general health education and does not replace an in-person medical assessment. | | | | | | |
| **Item / 项目** | | **Q1 / 问题1** | | **Q2 / 问题2** | | **Q3 / 问题3** |
| **Patient question (Chinese original) / 患者原始问题（中文）** | | 第一胎剖宫产，下一胎有没有机会顺产？ | | 剖宫产疤痕厚度会影响分娩方式吗？ | | 备孕前需要把上次手术记录带给医生看吗？ |
| **English translation of patient question / 患者问题英文翻译** | | After a first cesarean birth, is vaginal birth possible next time? | | Does cesarean scar thickness affect delivery mode? | | Before pregnancy, should I bring the previous surgical record to the doctor? |
| **Model response (Chinese) / 模型回答（中文）** | | 第一胎剖宫产后下一胎是否能顺产，要看上次剖宫产原因、切口类型、孕期情况和医院条件。保持局部清洁干燥，避免抓挠、挤压和自行涂药；若红肿扩大、疼痛或渗液加重，应复查。 | | 疤痕厚度可作为参考，但不能单独决定分娩方式，还要评估胎儿大小、胎位和子宫破裂风险。保持局部清洁干燥，避免抓挠、挤压和自行涂药；若红肿扩大、疼痛或渗液加重，应复查。 | | 备孕或孕早期应带上次手术记录和出院资料给医生，有助于制定分娩计划。建议观察变化，保证休息、饮食和清洁护理；若症状持续不缓解或逐渐加重，应联系产科门诊。 |
| **English translation of model response / 模型回答英文翻译** | | Vaginal birth after a previous cesarean may be possible depending on the reason for the first cesarean, incision type, current pregnancy, and hospital resources. Keep the area clean and dry. Seek review if redness, pain, drainage, or wound opening worsens. | | Scar thickness is only one reference and cannot alone determine delivery mode. Fetal size, position, and uterine rupture risk also matter. Keep the area clean and dry. Seek review if redness, pain, drainage, or wound opening worsens. | | Bring the previous operative and discharge records before pregnancy or early in pregnancy so the clinician can plan care. Record color, amount, odor, and pad use. Seek care for heavy bleeding, clots, pain, or fever. |
| **Overall evaluation item / 整体评价项目** | | | **Score / 分数** | | **Comments / 备注** | |
| **Patient Satisfaction / 患者满意度** | | | 5 | |  | |
| **Patient Understandability / 患者可理解性** | | | 4 | |  | |
| **Patient Usefulness / 患者实用性** | | | 5 | |  | |
| **Clinician Correctness / 医生评分：正确性** | | | 5 | |  | |
| **Clinician Completeness / 医生评分：完整性** | | | 5 | |  | |
| **Clinician Readability / 医生评分：可读性** | | | 4 | |  | |
| **Clinician Helpfulness / 医生评分：帮助性** | | | 5 | |  | |
| **Clinician Safety / 医生评分：安全性** | | | 5 | |  | |

**Patient 103**

| **Patient ID / 患者编号** | **Group / 模型组别** | | | | | |
| --- | --- | --- | --- | --- | --- | --- |
| P103 | ChatGPT 5.5 | | | | | |
| **General model response or patient education material (Chinese) / 模型总体回答或患者教育材料（中文）** | | | | | | |
| 双胎产后子宫更大、体力消耗更多，恢复可能比单胎慢一些，需要更充分休息。建议观察变化，保证休息、饮食和清洁护理；若症状持续不缓解或逐渐加重，应联系产科门诊。双胎产后贫血、腹直肌分离和盆底问题风险可能更高，建议按时复查并重视营养。训练应循序渐进，避免过早强度运动；若症状持续、加重或影响生活，应做康复评估。照顾两个宝宝应主动安排家人分班照护，保证产妇连续休息时间，必要时寻求社区和医护支持。请告诉家人和医生，争取照护分担；若有绝望、失控感或伤害想法，应立即就医。以上内容为一般健康教育，不能替代线下面诊。 | | | | | | |
| **English translation of the Chinese general response / 中文总体回答英文翻译** | | | | | | |
| After twin birth, the uterus is larger and physical demands are greater, so recovery may be slower than after singleton birth and requires more rest. Observe changes and maintain rest, nutrition, and hygiene. Persistent or worsening symptoms need obstetric advice. Risks of anemia, diastasis recti, and pelvic floor problems may be higher after twin birth. Timely review and nutrition are important. Increase exercises gradually and avoid early high intensity. Persistent or worsening symptoms need rehabilitation assessment. Care for two babies should be shared in shifts by family members so the mother has uninterrupted rest. Community and medical support may be needed. Record color, amount, odor, and pad use. Seek care for heavy bleeding, clots, pain, or fever. This is general health education and does not replace an in-person medical assessment. | | | | | | |
| **Item / 项目** | | **Q1 / 问题1** | | **Q2 / 问题2** | | **Q3 / 问题3** |
| **Patient question (Chinese original) / 患者原始问题（中文）** | | 双胎生产后恢复会不会比单胎更慢？ | | 双胎产后更容易贫血或盆底问题吗？ | | 照顾两个宝宝太累时怎样安排休息？ |
| **English translation of patient question / 患者问题英文翻译** | | Will recovery after twin birth be slower than after singleton birth? | | Are anemia or pelvic floor problems more common after twin birth? | | How should I arrange rest when caring for two babies is exhausting? |
| **Model response (Chinese) / 模型回答（中文）** | | 双胎产后子宫更大、体力消耗更多，恢复可能比单胎慢一些，需要更充分休息。建议观察变化，保证休息、饮食和清洁护理；若症状持续不缓解或逐渐加重，应联系产科门诊。 | | 双胎产后贫血、腹直肌分离和盆底问题风险可能更高，建议按时复查并重视营养。训练应循序渐进，避免过早强度运动；若症状持续、加重或影响生活，应做康复评估。 | | 照顾两个宝宝应主动安排家人分班照护，保证产妇连续休息时间，必要时寻求社区和医护支持。请告诉家人和医生，争取照护分担；若有绝望、失控感或伤害想法，应立即就医。 |
| **English translation of model response / 模型回答英文翻译** | | After twin birth, the uterus is larger and physical demands are greater, so recovery may be slower than after singleton birth and requires more rest. Observe changes and maintain rest, nutrition, and hygiene. Persistent or worsening symptoms need obstetric advice. | | Risks of anemia, diastasis recti, and pelvic floor problems may be higher after twin birth. Timely review and nutrition are important. Increase exercises gradually and avoid early high intensity. Persistent or worsening symptoms need rehabilitation assessment. | | Care for two babies should be shared in shifts by family members so the mother has uninterrupted rest. Community and medical support may be needed. Record color, amount, odor, and pad use. Seek care for heavy bleeding, clots, pain, or fever. |
| **Overall evaluation item / 整体评价项目** | | | **Score / 分数** | | **Comments / 备注** | |
| **Patient Satisfaction / 患者满意度** | | | 4 | |  | |
| **Patient Understandability / 患者可理解性** | | | 4 | |  | |
| **Patient Usefulness / 患者实用性** | | | 4 | |  | |
| **Clinician Correctness / 医生评分：正确性** | | | 5 | |  | |
| **Clinician Completeness / 医生评分：完整性** | | | 5 | |  | |
| **Clinician Readability / 医生评分：可读性** | | | 4 | |  | |
| **Clinician Helpfulness / 医生评分：帮助性** | | | 5 | |  | |
| **Clinician Safety / 医生评分：安全性** | | | 5 | |  | |

**Patient 104**

| **Patient ID / 患者编号** | **Group / 模型组别** | | | | | |
| --- | --- | --- | --- | --- | --- | --- |
| P104 | ChatGPT 5.5 | | | | | |
| **General model response or patient education material (Chinese) / 模型总体回答或患者教育材料（中文）** | | | | | | |
| 早产后担心宝宝很正常，但产妇自身伤口、恶露、血压、情绪和泌乳同样需要照护。建议记录颜色、量和气味，避免劳累；若1小时湿透卫生巾、血块增多、腹痛或发热，应就医。即使宝宝住院，产妇也应按时完成产后复查，出现发热、出血或情绪危机要提前就诊。建议记录颜色、量和气味，避免劳累；若1小时湿透卫生巾、血块增多、腹痛或发热，应就医。压力影响泌乳时，可联系产科、母乳门诊、新生儿科护士和心理支持人员，共同制定挤奶和休息计划。重点是正确含接、有效排奶和休息补水；若红肿热痛、发热或24小时不缓解，应就医。以上内容为一般健康教育，不能替代线下面诊。 | | | | | | |
| **English translation of the Chinese general response / 中文总体回答英文翻译** | | | | | | |
| Worrying about a preterm baby is natural, but the mother’s wound, lochia, blood pressure, mood, and lactation also need care. Record color, amount, odor, and pad use. Seek care for heavy bleeding, clots, pain, or fever. Even if the baby is hospitalized, the mother should attend postpartum check-ups. Fever, bleeding, or emotional crisis needs earlier care. Record color, amount, odor, and pad use. Seek care for heavy bleeding, clots, pain, or fever. If stress affects milk production, contact obstetrics, a lactation clinic, neonatal nurses, and mental health support to plan pumping and rest. Focus on effective milk removal and correct latch. Redness, fever, or no improvement within 24 hours needs care. This is general health education and does not replace an in-person medical assessment. | | | | | | |
| **Item / 项目** | | **Q1 / 问题1** | | **Q2 / 问题2** | | **Q3 / 问题3** |
| **Patient question (Chinese original) / 患者原始问题（中文）** | | 早产后我总担心宝宝，也忽略自己恢复，怎么办？ | | 宝宝住院期间我还需要做产后复查吗？ | | 压力太大影响泌乳时可以找谁帮助？ |
| **English translation of patient question / 患者问题英文翻译** | | After preterm birth, I worry about the baby and neglect my own recovery. What should I do? | | If the baby is hospitalized, do I still need postpartum check-ups? | | If stress affects milk production, who can I ask for help? |
| **Model response (Chinese) / 模型回答（中文）** | | 早产后担心宝宝很正常，但产妇自身伤口、恶露、血压、情绪和泌乳同样需要照护。建议记录颜色、量和气味，避免劳累；若1小时湿透卫生巾、血块增多、腹痛或发热，应就医。 | | 即使宝宝住院，产妇也应按时完成产后复查，出现发热、出血或情绪危机要提前就诊。建议记录颜色、量和气味，避免劳累；若1小时湿透卫生巾、血块增多、腹痛或发热，应就医。 | | 压力影响泌乳时，可联系产科、母乳门诊、新生儿科护士和心理支持人员，共同制定挤奶和休息计划。重点是正确含接、有效排奶和休息补水；若红肿热痛、发热或24小时不缓解，应就医。 |
| **English translation of model response / 模型回答英文翻译** | | Worrying about a preterm baby is natural, but the mother’s wound, lochia, blood pressure, mood, and lactation also need care. Record color, amount, odor, and pad use. Seek care for heavy bleeding, clots, pain, or fever. | | Even if the baby is hospitalized, the mother should attend postpartum check-ups. Fever, bleeding, or emotional crisis needs earlier care. Record color, amount, odor, and pad use. Seek care for heavy bleeding, clots, pain, or fever. | | If stress affects milk production, contact obstetrics, a lactation clinic, neonatal nurses, and mental health support to plan pumping and rest. Focus on effective milk removal and correct latch. Redness, fever, or no improvement within 24 hours needs care. |
| **Overall evaluation item / 整体评价项目** | | | **Score / 分数** | | **Comments / 备注** | |
| **Patient Satisfaction / 患者满意度** | | | 5 | |  | |
| **Patient Understandability / 患者可理解性** | | | 4 | |  | |
| **Patient Usefulness / 患者实用性** | | | 4 | |  | |
| **Clinician Correctness / 医生评分：正确性** | | | 4 | |  | |
| **Clinician Completeness / 医生评分：完整性** | | | 5 | |  | |
| **Clinician Readability / 医生评分：可读性** | | | 4 | |  | |
| **Clinician Helpfulness / 医生评分：帮助性** | | | 5 | |  | |
| **Clinician Safety / 医生评分：安全性** | | | 5 | |  | |

**Patient 105**

| **Patient ID / 患者编号** | **Group / 模型组别** | | | | | |
| --- | --- | --- | --- | --- | --- | --- |
| P105 | ChatGPT 5.5 | | | | | |
| **General model response or patient education material (Chinese) / 模型总体回答或患者教育材料（中文）** | | | | | | |
| 经历抢救或大出血后，应重视休息、补铁、营养、伤口和恶露观察，不宜过早劳累。建议记录颜色、量和气味，避免劳累；若1小时湿透卫生巾、血块增多、腹痛或发热，应就医。复查血常规时间应按出血量和贫血程度决定，常需在出院后或产后复查时再次评估。建议记录颜色、量和气味，避免劳累；若1小时湿透卫生巾、血块增多、腹痛或发热，应就医。反复害怕回想分娩、做噩梦、回避医院或持续紧张，可能需要心理支持，不是矫情。建议观察变化，保证休息、饮食和清洁护理；若症状持续不缓解或逐渐加重，应联系产科门诊。以上内容为一般健康教育，不能替代线下面诊。 | | | | | | |
| **English translation of the Chinese general response / 中文总体回答英文翻译** | | | | | | |
| After rescue treatment or major bleeding, rest, iron supplementation, nutrition, wound care, and lochia observation are important. Avoid early overexertion. Record color, amount, odor, and pad use. Seek care for heavy bleeding, clots, pain, or fever. The timing of repeat blood count depends on blood loss and anemia severity. It is often reassessed after discharge or at postpartum review. Record color, amount, odor, and pad use. Seek care for heavy bleeding, clots, pain, or fever. Repeated fear when recalling delivery, nightmares, avoidance of hospitals, or persistent tension may need psychological support and should not be dismissed. Observe changes and maintain rest, nutrition, and hygiene. Persistent or worsening symptoms need obstetric advice. This is general health education and does not replace an in-person medical assessment. | | | | | | |
| **Item / 项目** | | **Q1 / 问题1** | | **Q2 / 问题2** | | **Q3 / 问题3** |
| **Patient question (Chinese original) / 患者原始问题（中文）** | | 产后如果经历了抢救或大出血，身体恢复要注意什么？ | | 大出血后多久复查血常规比较合适？ | | 如果总是害怕回想分娩过程，需要心理支持吗？ |
| **English translation of patient question / 患者问题英文翻译** | | If I experienced rescue treatment or major bleeding after birth, what should I pay attention to during recovery? | | How soon should I repeat a blood count after major bleeding? | | If I keep feeling afraid when recalling the delivery, do I need psychological support? |
| **Model response (Chinese) / 模型回答（中文）** | | 经历抢救或大出血后，应重视休息、补铁、营养、伤口和恶露观察，不宜过早劳累。建议记录颜色、量和气味，避免劳累；若1小时湿透卫生巾、血块增多、腹痛或发热，应就医。 | | 复查血常规时间应按出血量和贫血程度决定，常需在出院后或产后复查时再次评估。建议记录颜色、量和气味，避免劳累；若1小时湿透卫生巾、血块增多、腹痛或发热，应就医。 | | 反复害怕回想分娩、做噩梦、回避医院或持续紧张，可能需要心理支持，不是矫情。建议观察变化，保证休息、饮食和清洁护理；若症状持续不缓解或逐渐加重，应联系产科门诊。 |
| **English translation of model response / 模型回答英文翻译** | | After rescue treatment or major bleeding, rest, iron supplementation, nutrition, wound care, and lochia observation are important. Avoid early overexertion. Record color, amount, odor, and pad use. Seek care for heavy bleeding, clots, pain, or fever. | | The timing of repeat blood count depends on blood loss and anemia severity. It is often reassessed after discharge or at postpartum review. Record color, amount, odor, and pad use. Seek care for heavy bleeding, clots, pain, or fever. | | Repeated fear when recalling delivery, nightmares, avoidance of hospitals, or persistent tension may need psychological support and should not be dismissed. Observe changes and maintain rest, nutrition, and hygiene. Persistent or worsening symptoms need obstetric advice. |
| **Overall evaluation item / 整体评价项目** | | | **Score / 分数** | | **Comments / 备注** | |
| **Patient Satisfaction / 患者满意度** | | | 4 | |  | |
| **Patient Understandability / 患者可理解性** | | | 4 | |  | |
| **Patient Usefulness / 患者实用性** | | | 4 | |  | |
| **Clinician Correctness / 医生评分：正确性** | | | 5 | |  | |
| **Clinician Completeness / 医生评分：完整性** | | | 5 | |  | |
| **Clinician Readability / 医生评分：可读性** | | | 4 | |  | |
| **Clinician Helpfulness / 医生评分：帮助性** | | | 5 | |  | |
| **Clinician Safety / 医生评分：安全性** | | | 5 | |  | |

**Patient 106**

| **Patient ID / 患者编号** | **Group / 模型组别** | | | | | |
| --- | --- | --- | --- | --- | --- | --- |
| P106 | ChatGPT 5.5 | | | | | |
| **General model response or patient education material (Chinese) / 模型总体回答或患者教育材料（中文）** | | | | | | |
| 孕期胆汁淤积产后通常需要按医生安排复查肝功能和胆汁酸，确认是否恢复。建议观察变化，保证休息、饮食和清洁护理；若症状持续不缓解或逐渐加重，应联系产科门诊。皮肤瘙痒产后可逐渐减轻，但若持续明显或加重，应复查，不要只等待。护理应选择温和产品，避免刺激和来源不明药膏；若红肿、渗液或症状明显，应专科评估。眼睛发黄、尿色很深、皮肤明显黄染、右上腹痛或乏力加重，应尽快就医。建议适量饮水、规律排尿；若尿痛加重、发热、腰痛或排尿困难持续，应查尿常规。以上内容为一般健康教育，不能替代线下面诊。 | | | | | | |
| **English translation of the Chinese general response / 中文总体回答英文翻译** | | | | | | |
| After intrahepatic cholestasis of pregnancy, liver function and bile acids should usually be rechecked as advised to confirm recovery. Observe changes and maintain rest, nutrition, and hygiene. Persistent or worsening symptoms need obstetric advice. Itching may gradually improve after birth, but persistent or worsening itching should be checked rather than simply observed. Record color, amount, odor, and pad use. Seek care for heavy bleeding, clots, pain, or fever. Yellow eyes, very dark urine, obvious jaundice, right upper abdominal pain, or worsening fatigue needs prompt medical care. Drink fluids and urinate regularly. Worsening pain, fever, back pain, or persistent difficulty needs urine testing. This is general health education and does not replace an in-person medical assessment. | | | | | | |
| **Item / 项目** | | **Q1 / 问题1** | | **Q2 / 问题2** | | **Q3 / 问题3** |
| **Patient question (Chinese original) / 患者原始问题（中文）** | | 我孕期有胆汁淤积，产后还需要复查肝功能吗？ | | 皮肤瘙痒产后没有完全消失正常吗？ | | 如果眼睛发黄或尿色很深，需要就医吗？ |
| **English translation of patient question / 患者问题英文翻译** | | I had cholestasis during pregnancy. Do I still need liver function tests after birth? | | Is it normal if skin itching has not completely disappeared after birth? | | If my eyes turn yellow or urine becomes very dark, should I see a doctor? |
| **Model response (Chinese) / 模型回答（中文）** | | 孕期胆汁淤积产后通常需要按医生安排复查肝功能和胆汁酸，确认是否恢复。建议观察变化，保证休息、饮食和清洁护理；若症状持续不缓解或逐渐加重，应联系产科门诊。 | | 皮肤瘙痒产后可逐渐减轻，但若持续明显或加重，应复查，不要只等待。护理应选择温和产品，避免刺激和来源不明药膏；若红肿、渗液或症状明显，应专科评估。 | | 眼睛发黄、尿色很深、皮肤明显黄染、右上腹痛或乏力加重，应尽快就医。建议适量饮水、规律排尿；若尿痛加重、发热、腰痛或排尿困难持续，应查尿常规。 |
| **English translation of model response / 模型回答英文翻译** | | After intrahepatic cholestasis of pregnancy, liver function and bile acids should usually be rechecked as advised to confirm recovery. Observe changes and maintain rest, nutrition, and hygiene. Persistent or worsening symptoms need obstetric advice. | | Itching may gradually improve after birth, but persistent or worsening itching should be checked rather than simply observed. Record color, amount, odor, and pad use. Seek care for heavy bleeding, clots, pain, or fever. | | Yellow eyes, very dark urine, obvious jaundice, right upper abdominal pain, or worsening fatigue needs prompt medical care. Drink fluids and urinate regularly. Worsening pain, fever, back pain, or persistent difficulty needs urine testing. |
| **Overall evaluation item / 整体评价项目** | | | **Score / 分数** | | **Comments / 备注** | |
| **Patient Satisfaction / 患者满意度** | | | 4 | |  | |
| **Patient Understandability / 患者可理解性** | | | 4 | |  | |
| **Patient Usefulness / 患者实用性** | | | 4 | |  | |
| **Clinician Correctness / 医生评分：正确性** | | | 5 | |  | |
| **Clinician Completeness / 医生评分：完整性** | | | 5 | |  | |
| **Clinician Readability / 医生评分：可读性** | | | 4 | |  | |
| **Clinician Helpfulness / 医生评分：帮助性** | | | 5 | |  | |
| **Clinician Safety / 医生评分：安全性** | | | 5 | |  | |

**Patient 107**

| **Patient ID / 患者编号** | **Group / 模型组别** | | | | | |
| --- | --- | --- | --- | --- | --- | --- |
| P107 | ChatGPT 5.5 | | | | | |
| **General model response or patient education material (Chinese) / 模型总体回答或患者教育材料（中文）** | | | | | | |
| 轻度肝功能异常不一定影响哺乳，但要明确原因并按医生要求复查。重点是正确含接、有效排奶和休息补水；若红肿热痛、发热或24小时不缓解，应就医。保肝药期间能否哺乳取决于具体药物，不要自行服用中草药或保健品。重点是正确含接、有效排奶和休息补水；若红肿热痛、发热或24小时不缓解，应就医。若出现黄疸、尿色加深、皮肤瘙痒明显、右上腹痛、恶心呕吐或乏力加重，应尽快复查肝功能。建议适量饮水、规律排尿；若尿痛加重、发热、腰痛或排尿困难持续，应查尿常规。以上内容为一般健康教育，不能替代线下面诊。 | | | | | | |
| **English translation of the Chinese general response / 中文总体回答英文翻译** | | | | | | |
| Mild liver function abnormality does not necessarily affect breastfeeding, but the cause should be identified and follow-up arranged. Focus on effective milk removal and correct latch. Redness, fever, or no improvement within 24 hours needs care. Whether breastfeeding can continue while taking liver medicines depends on the exact drug. Do not take herbal medicines or supplements by yourself. Focus on effective milk removal and correct latch. Redness, fever, or no improvement within 24 hours needs care. Jaundice, dark urine, marked itching, right upper abdominal pain, nausea, vomiting, or worsening fatigue means liver function should be rechecked soon. Drink fluids and urinate regularly. Worsening pain, fever, back pain, or persistent difficulty needs urine testing. This is general health education and does not replace an in-person medical assessment. | | | | | | |
| **Item / 项目** | | **Q1 / 问题1** | | **Q2 / 问题2** | | **Q3 / 问题3** |
| **Patient question (Chinese original) / 患者原始问题（中文）** | | 我产后肝功能有点异常，哺乳会受影响吗？ | | 吃保肝药期间还能喂奶吗？ | | 哪些症状说明需要尽快复查肝功能？ |
| **English translation of patient question / 患者问题英文翻译** | | My liver function is a little abnormal after birth. Will breastfeeding be affected? | | Can I breastfeed while taking liver-protective medicine? | | What symptoms mean I need to repeat liver function tests soon? |
| **Model response (Chinese) / 模型回答（中文）** | | 轻度肝功能异常不一定影响哺乳，但要明确原因并按医生要求复查。重点是正确含接、有效排奶和休息补水；若红肿热痛、发热或24小时不缓解，应就医。 | | 保肝药期间能否哺乳取决于具体药物，不要自行服用中草药或保健品。重点是正确含接、有效排奶和休息补水；若红肿热痛、发热或24小时不缓解，应就医。 | | 若出现黄疸、尿色加深、皮肤瘙痒明显、右上腹痛、恶心呕吐或乏力加重，应尽快复查肝功能。建议适量饮水、规律排尿；若尿痛加重、发热、腰痛或排尿困难持续，应查尿常规。 |
| **English translation of model response / 模型回答英文翻译** | | Mild liver function abnormality does not necessarily affect breastfeeding, but the cause should be identified and follow-up arranged. Focus on effective milk removal and correct latch. Redness, fever, or no improvement within 24 hours needs care. | | Whether breastfeeding can continue while taking liver medicines depends on the exact drug. Do not take herbal medicines or supplements by yourself. Focus on effective milk removal and correct latch. Redness, fever, or no improvement within 24 hours needs care. | | Jaundice, dark urine, marked itching, right upper abdominal pain, nausea, vomiting, or worsening fatigue means liver function should be rechecked soon. Drink fluids and urinate regularly. Worsening pain, fever, back pain, or persistent difficulty needs urine testing. |
| **Overall evaluation item / 整体评价项目** | | | **Score / 分数** | | **Comments / 备注** | |
| **Patient Satisfaction / 患者满意度** | | | 5 | |  | |
| **Patient Understandability / 患者可理解性** | | | 4 | |  | |
| **Patient Usefulness / 患者实用性** | | | 4 | |  | |
| **Clinician Correctness / 医生评分：正确性** | | | 5 | |  | |
| **Clinician Completeness / 医生评分：完整性** | | | 4 | |  | |
| **Clinician Readability / 医生评分：可读性** | | | 4 | |  | |
| **Clinician Helpfulness / 医生评分：帮助性** | | | 4 | |  | |
| **Clinician Safety / 医生评分：安全性** | | | 5 | |  | |

**Patient 108**

| **Patient ID / 患者编号** | **Group / 模型组别** | | | | | |
| --- | --- | --- | --- | --- | --- | --- |
| P108 | ChatGPT 5.5 | | | | | |
| **General model response or patient education material (Chinese) / 模型总体回答或患者教育材料（中文）** | | | | | | |
| 孕期甲减产后药量不能自己随意调整，应根据甲状腺功能和医生建议变化。哺乳期用药应告知医生正在哺乳，按医嘱选择；不要自行加量或长期使用。左甲状腺素通常可在哺乳期使用，按医嘱服用一般不会影响宝宝。重点是正确含接、有效排奶和休息补水；若红肿热痛、发热或24小时不缓解，应就医。产后常需在6周左右复查甲状腺功能，若心慌、怕冷、乏力或情绪异常，应提前咨询。请告诉家人和医生，争取照护分担；若有绝望、失控感或伤害想法，应立即就医。以上内容为一般健康教育，不能替代线下面诊。 | | | | | | |
| **English translation of the Chinese general response / 中文总体回答英文翻译** | | | | | | |
| The dose for hypothyroidism after birth should not be changed by yourself. It should be adjusted according to thyroid function and medical advice. Observe changes and maintain rest, nutrition, and hygiene. Persistent or worsening symptoms need obstetric advice. Levothyroxine is usually compatible with breastfeeding when taken as prescribed and generally does not harm the baby. Focus on effective milk removal and correct latch. Redness, fever, or no improvement within 24 hours needs care. Thyroid function is often rechecked around 6 weeks postpartum. Palpitations, cold intolerance, fatigue, or mood changes should prompt earlier consultation. Tell family and clinicians how you feel. Hopelessness, loss of control, or harm thoughts need immediate help. This is general health education and does not replace an in-person medical assessment. | | | | | | |
| **Item / 项目** | | **Q1 / 问题1** | | **Q2 / 问题2** | | **Q3 / 问题3** |
| **Patient question (Chinese original) / 患者原始问题（中文）** | | 我孕期有甲减，产后药量需要自己调整吗？ | | 哺乳期吃优甲乐会不会影响宝宝？ | | 产后多久复查甲状腺功能比较合适？ |
| **English translation of patient question / 患者问题英文翻译** | | I had hypothyroidism during pregnancy. Should I adjust my dose by myself after birth? | | Will levothyroxine affect the baby during breastfeeding? | | How soon after birth should I repeat thyroid function tests? |
| **Model response (Chinese) / 模型回答（中文）** | | 孕期甲减产后药量不能自己随意调整，应根据甲状腺功能和医生建议变化。哺乳期用药应告知医生正在哺乳，按医嘱选择；不要自行加量或长期使用。 | | 左甲状腺素通常可在哺乳期使用，按医嘱服用一般不会影响宝宝。重点是正确含接、有效排奶和休息补水；若红肿热痛、发热或24小时不缓解，应就医。 | | 产后常需在6周左右复查甲状腺功能，若心慌、怕冷、乏力或情绪异常，应提前咨询。请告诉家人和医生，争取照护分担；若有绝望、失控感或伤害想法，应立即就医。 |
| **English translation of model response / 模型回答英文翻译** | | The dose for hypothyroidism after birth should not be changed by yourself. It should be adjusted according to thyroid function and medical advice. Observe changes and maintain rest, nutrition, and hygiene. Persistent or worsening symptoms need obstetric advice. | | Levothyroxine is usually compatible with breastfeeding when taken as prescribed and generally does not harm the baby. Focus on effective milk removal and correct latch. Redness, fever, or no improvement within 24 hours needs care. | | Thyroid function is often rechecked around 6 weeks postpartum. Palpitations, cold intolerance, fatigue, or mood changes should prompt earlier consultation. Tell family and clinicians how you feel. Hopelessness, loss of control, or harm thoughts need immediate help. |
| **Overall evaluation item / 整体评价项目** | | | **Score / 分数** | | **Comments / 备注** | |
| **Patient Satisfaction / 患者满意度** | | | 4 | |  | |
| **Patient Understandability / 患者可理解性** | | | 5 | |  | |
| **Patient Usefulness / 患者实用性** | | | 4 | |  | |
| **Clinician Correctness / 医生评分：正确性** | | | 4 | |  | |
| **Clinician Completeness / 医生评分：完整性** | | | 4 | |  | |
| **Clinician Readability / 医生评分：可读性** | | | 5 | |  | |
| **Clinician Helpfulness / 医生评分：帮助性** | | | 5 | |  | |
| **Clinician Safety / 医生评分：安全性** | | | 5 | |  | |

**Patient 109**

| **Patient ID / 患者编号** | **Group / 模型组别** | | | | | |
| --- | --- | --- | --- | --- | --- | --- |
| P109 | ChatGPT 5.5 | | | | | |
| **General model response or patient education material (Chinese) / 模型总体回答或患者教育材料（中文）** | | | | | | |
| 产后牙龈出血可以看牙医，常与牙龈炎、清洁不足或激素变化有关。建议记录颜色、量和气味，避免劳累；若1小时湿透卫生巾、血块增多、腹痛或发热，应就医。哺乳期通常可以洗牙、补牙和必要的局部治疗，具体用药和麻醉由牙医评估。重点是正确含接、有效排奶和休息补水；若红肿热痛、发热或24小时不缓解，应就医。牙痛需要用药时一定告诉牙医正在哺乳，以便选择相对安全的止痛药、抗生素或麻醉药。重点是正确含接、有效排奶和休息补水；若红肿热痛、发热或24小时不缓解，应就医。以上内容为一般健康教育，不能替代线下面诊。 | | | | | | |
| **English translation of the Chinese general response / 中文总体回答英文翻译** | | | | | | |
| You can see a dentist for gum bleeding after birth. It may be related to gingivitis, insufficient cleaning, or hormonal changes. Record color, amount, odor, and pad use. Seek care for heavy bleeding, clots, pain, or fever. Dental cleaning, fillings, and necessary local treatment are usually possible during breastfeeding. Medicines and local anesthesia should be assessed by the dentist. Focus on effective milk removal and correct latch. Redness, fever, or no improvement within 24 hours needs care. If medicine is needed for toothache, tell the dentist you are breastfeeding so safer pain medicines, antibiotics, or anesthetics can be chosen. Focus on effective milk removal and correct latch. Redness, fever, or no improvement within 24 hours needs care. This is general health education and does not replace an in-person medical assessment. | | | | | | |
| **Item / 项目** | | **Q1 / 问题1** | | **Q2 / 问题2** | | **Q3 / 问题3** |
| **Patient question (Chinese original) / 患者原始问题（中文）** | | 产后牙龈出血可以看牙医吗？ | | 哺乳期能不能补牙或洗牙？ | | 如果牙痛需要用药，需要告诉牙医我在哺乳吗？ |
| **English translation of patient question / 患者问题英文翻译** | | Can I see a dentist for gum bleeding after birth? | | Can I have a filling or dental cleaning while breastfeeding? | | If I need medicine for toothache, should I tell the dentist I am breastfeeding? |
| **Model response (Chinese) / 模型回答（中文）** | | 产后牙龈出血可以看牙医，常与牙龈炎、清洁不足或激素变化有关。建议记录颜色、量和气味，避免劳累；若1小时湿透卫生巾、血块增多、腹痛或发热，应就医。 | | 哺乳期通常可以洗牙、补牙和必要的局部治疗，具体用药和麻醉由牙医评估。重点是正确含接、有效排奶和休息补水；若红肿热痛、发热或24小时不缓解，应就医。 | | 牙痛需要用药时一定告诉牙医正在哺乳，以便选择相对安全的止痛药、抗生素或麻醉药。重点是正确含接、有效排奶和休息补水；若红肿热痛、发热或24小时不缓解，应就医。 |
| **English translation of model response / 模型回答英文翻译** | | You can see a dentist for gum bleeding after birth. It may be related to gingivitis, insufficient cleaning, or hormonal changes. Record color, amount, odor, and pad use. Seek care for heavy bleeding, clots, pain, or fever. | | Dental cleaning, fillings, and necessary local treatment are usually possible during breastfeeding. Medicines and local anesthesia should be assessed by the dentist. Focus on effective milk removal and correct latch. Redness, fever, or no improvement within 24 hours needs care. | | If medicine is needed for toothache, tell the dentist you are breastfeeding so safer pain medicines, antibiotics, or anesthetics can be chosen. Focus on effective milk removal and correct latch. Redness, fever, or no improvement within 24 hours needs care. |
| **Overall evaluation item / 整体评价项目** | | | **Score / 分数** | | **Comments / 备注** | |
| **Patient Satisfaction / 患者满意度** | | | 4 | |  | |
| **Patient Understandability / 患者可理解性** | | | 4 | |  | |
| **Patient Usefulness / 患者实用性** | | | 4 | |  | |
| **Clinician Correctness / 医生评分：正确性** | | | 5 | |  | |
| **Clinician Completeness / 医生评分：完整性** | | | 5 | |  | |
| **Clinician Readability / 医生评分：可读性** | | | 4 | |  | |
| **Clinician Helpfulness / 医生评分：帮助性** | | | 5 | |  | |
| **Clinician Safety / 医生评分：安全性** | | | 5 | |  | |

**Patient 110**

| **Patient ID / 患者编号** | **Group / 模型组别** | | | | | |
| --- | --- | --- | --- | --- | --- | --- |
| P110 | ChatGPT 5.5 | | | | | |
| **General model response or patient education material (Chinese) / 模型总体回答或患者教育材料（中文）** | | | | | | |
| 产后长时间看手机会加重眼疲劳、头痛和睡眠不足，应定时休息并减少夜间强光刺激。建议按医嘱测血压，记录头痛、眼花和水肿；若血压升高或症状明显，应及时就医。月子里可以适量阅读和使用电脑，但要注意光线、距离和休息，不宜连续久看。以清洁、舒适和避免受凉为原则，保持通风；若伴发热、心慌或明显乏力，应评估。视物模糊伴头痛、眼花、胸闷或孕期高血压史时，应立即测血压并及时就医。建议按医嘱测血压，记录头痛、眼花和水肿；若血压升高或症状明显，应及时就医。以上内容为一般健康教育，不能替代线下面诊。 | | | | | | |
| **English translation of the Chinese general response / 中文总体回答英文翻译** | | | | | | |
| Long phone use after birth can worsen eye strain, headache, and poor sleep. Take regular breaks and reduce bright screen exposure at night. Record color, amount, odor, and pad use. Seek care for heavy bleeding, clots, pain, or fever. Moderate reading and computer use are acceptable during confinement, but pay attention to lighting, distance, and breaks. Avoid long continuous use. Focus on hygiene, comfort, ventilation, and avoiding chills. Fever, palpitations, or marked weakness needs assessment. Blurred vision with headache, visual spots, chest tightness, or a history of pregnancy hypertension should prompt blood pressure measurement and medical care. Record color, amount, odor, and pad use. Seek care for heavy bleeding, clots, pain, or fever. This is general health education and does not replace an in-person medical assessment. | | | | | | |
| **Item / 项目** | | **Q1 / 问题1** | | **Q2 / 问题2** | | **Q3 / 问题3** |
| **Patient question (Chinese original) / 患者原始问题（中文）** | | 产后看手机多了眼睛酸胀，会不会影响恢复？ | | 月子里可以正常阅读和使用电脑吗？ | | 如果视物模糊伴头痛，需要测血压吗？ |
| **English translation of patient question / 患者问题英文翻译** | | My eyes feel sore after looking at my phone a lot after birth. Will it affect recovery? | | Can I read and use a computer normally during confinement? | | If blurred vision comes with headache, should I measure blood pressure? |
| **Model response (Chinese) / 模型回答（中文）** | | 产后长时间看手机会加重眼疲劳、头痛和睡眠不足，应定时休息并减少夜间强光刺激。建议按医嘱测血压，记录头痛、眼花和水肿；若血压升高或症状明显，应及时就医。 | | 月子里可以适量阅读和使用电脑，但要注意光线、距离和休息，不宜连续久看。以清洁、舒适和避免受凉为原则，保持通风；若伴发热、心慌或明显乏力，应评估。 | | 视物模糊伴头痛、眼花、胸闷或孕期高血压史时，应立即测血压并及时就医。建议按医嘱测血压，记录头痛、眼花和水肿；若血压升高或症状明显，应及时就医。 |
| **English translation of model response / 模型回答英文翻译** | | Long phone use after birth can worsen eye strain, headache, and poor sleep. Take regular breaks and reduce bright screen exposure at night. Record color, amount, odor, and pad use. Seek care for heavy bleeding, clots, pain, or fever. | | Moderate reading and computer use are acceptable during confinement, but pay attention to lighting, distance, and breaks. Avoid long continuous use. Focus on hygiene, comfort, ventilation, and avoiding chills. Fever, palpitations, or marked weakness needs assessment. | | Blurred vision with headache, visual spots, chest tightness, or a history of pregnancy hypertension should prompt blood pressure measurement and medical care. Record color, amount, odor, and pad use. Seek care for heavy bleeding, clots, pain, or fever. |
| **Overall evaluation item / 整体评价项目** | | | **Score / 分数** | | **Comments / 备注** | |
| **Patient Satisfaction / 患者满意度** | | | 4 | |  | |
| **Patient Understandability / 患者可理解性** | | | 4 | |  | |
| **Patient Usefulness / 患者实用性** | | | 4 | |  | |
| **Clinician Correctness / 医生评分：正确性** | | | 5 | |  | |
| **Clinician Completeness / 医生评分：完整性** | | | 5 | |  | |
| **Clinician Readability / 医生评分：可读性** | | | 4 | |  | |
| **Clinician Helpfulness / 医生评分：帮助性** | | | 5 | |  | |
| **Clinician Safety / 医生评分：安全性** | | | 4 | |  | |

**Patient 111**

| **Patient ID / 患者编号** | **Group / 模型组别** | | | | | |
| --- | --- | --- | --- | --- | --- | --- |
| P111 | ChatGPT 5.5 | | | | | |
| **General model response or patient education material (Chinese) / 模型总体回答或患者教育材料（中文）** | | | | | | |
| 无痛分娩后头痛可能与疲劳、血压、贫血或麻醉相关问题有关，需要结合特点判断。建议按医嘱测血压，记录头痛、眼花和水肿；若血压升高或症状明显，应及时就医。躺下减轻、坐起加重的头痛，要警惕硬膜穿刺后头痛，应联系麻醉科或产科评估。建议按医嘱测血压，记录头痛、眼花和水肿；若血压升高或症状明显，应及时就医。头痛很重、呕吐、发热、颈强直、视物模糊、血压升高或肢体异常，应立即就医。建议复测体温，并观察恶露、乳房、尿痛和伤口；若体温≥38℃、寒战或精神差，应就医。以上内容为一般健康教育，不能替代线下面诊。 | | | | | | |
| **English translation of the Chinese general response / 中文总体回答英文翻译** | | | | | | |
| Headache after epidural analgesia may be related to fatigue, blood pressure, anemia, or anesthesia-related causes. The pattern matters. Increase fluids, fiber, and gentle activity. More bleeding, marked bloating, or severe pain needs care. A headache that improves when lying down and worsens when sitting may suggest post-dural puncture headache. Contact anesthesia or obstetrics for assessment. Monitor blood pressure and symptoms. Rising pressure, headache, visual changes, or swelling needs prompt care. Severe headache, vomiting, fever, neck stiffness, blurred vision, high blood pressure, or limb symptoms requires urgent care. Record color, amount, odor, and pad use. Seek care for heavy bleeding, clots, pain, or fever. This is general health education and does not replace an in-person medical assessment. | | | | | | |
| **Item / 项目** | | **Q1 / 问题1** | | **Q2 / 问题2** | | **Q3 / 问题3** |
| **Patient question (Chinese original) / 患者原始问题（中文）** | | 我打了无痛后产后头痛，是麻醉引起的吗？ | | 躺下好一些、坐起来头痛明显，需要注意什么？ | | 如果头痛很重或伴有呕吐，需要马上就医吗？ |
| **English translation of patient question / 患者问题英文翻译** | | I have a headache after epidural anesthesia. Could it be caused by anesthesia? | | It feels better when lying down but worse when sitting up. What should I pay attention to? | | If the headache is severe or comes with vomiting, do I need urgent care? |
| **Model response (Chinese) / 模型回答（中文）** | | 无痛分娩后头痛可能与疲劳、血压、贫血或麻醉相关问题有关，需要结合特点判断。建议按医嘱测血压，记录头痛、眼花和水肿；若血压升高或症状明显，应及时就医。 | | 躺下减轻、坐起加重的头痛，要警惕硬膜穿刺后头痛，应联系麻醉科或产科评估。建议按医嘱测血压，记录头痛、眼花和水肿；若血压升高或症状明显，应及时就医。 | | 头痛很重、呕吐、发热、颈强直、视物模糊、血压升高或肢体异常，应立即就医。建议复测体温，并观察恶露、乳房、尿痛和伤口；若体温≥38℃、寒战或精神差，应就医。 |
| **English translation of model response / 模型回答英文翻译** | | Headache after epidural analgesia may be related to fatigue, blood pressure, anemia, or anesthesia-related causes. The pattern matters. Increase fluids, fiber, and gentle activity. More bleeding, marked bloating, or severe pain needs care. | | A headache that improves when lying down and worsens when sitting may suggest post-dural puncture headache. Contact anesthesia or obstetrics for assessment. Monitor blood pressure and symptoms. Rising pressure, headache, visual changes, or swelling needs prompt care. | | Severe headache, vomiting, fever, neck stiffness, blurred vision, high blood pressure, or limb symptoms requires urgent care. Record color, amount, odor, and pad use. Seek care for heavy bleeding, clots, pain, or fever. |
| **Overall evaluation item / 整体评价项目** | | | **Score / 分数** | | **Comments / 备注** | |
| **Patient Satisfaction / 患者满意度** | | | 4 | |  | |
| **Patient Understandability / 患者可理解性** | | | 4 | |  | |
| **Patient Usefulness / 患者实用性** | | | 4 | |  | |
| **Clinician Correctness / 医生评分：正确性** | | | 5 | |  | |
| **Clinician Completeness / 医生评分：完整性** | | | 4 | |  | |
| **Clinician Readability / 医生评分：可读性** | | | 4 | |  | |
| **Clinician Helpfulness / 医生评分：帮助性** | | | 4 | |  | |
| **Clinician Safety / 医生评分：安全性** | | | 5 | |  | |

**Patient 112**

| **Patient ID / 患者编号** | **Group / 模型组别** | | | | | |
| --- | --- | --- | --- | --- | --- | --- |
| P112 | ChatGPT 5.5 | | | | | |
| **General model response or patient education material (Chinese) / 模型总体回答或患者教育材料（中文）** | | | | | | |
| 产后腰背痛多数与孕期负担、抱娃喂奶姿势和肌肉劳损有关，不一定是无痛后遗症。重点是正确含接、有效排奶和休息补水；若红肿热痛、发热或24小时不缓解，应就医。喂奶姿势不好会明显加重腰背痛，建议用枕头支撑、脚下垫高、避免低头弯腰。重点是正确含接、有效排奶和休息补水；若红肿热痛、发热或24小时不缓解，应就医。腰痛伴发热、下肢麻木无力、大小便异常或疼痛进行性加重，应及时检查。建议复测体温，并观察恶露、乳房、尿痛和伤口；若体温≥38℃、寒战或精神差，应就医。以上内容为一般健康教育，不能替代线下面诊。 | | | | | | |
| **English translation of the Chinese general response / 中文总体回答英文翻译** | | | | | | |
| Postpartum back pain is usually related to pregnancy load, holding and feeding posture, and muscle strain. It is not necessarily a long-term effect of epidural anesthesia. Increase exercises gradually and avoid early high intensity. Persistent or worsening symptoms need rehabilitation assessment. Poor breastfeeding posture can worsen back pain. Use pillows for support, raise the feet if needed, and avoid bending the neck and back for long periods. Focus on effective milk removal and correct latch. Redness, fever, or no improvement within 24 hours needs care. Back pain with fever, leg numbness or weakness, bowel or bladder changes, or progressive worsening should be examined promptly. Recheck temperature and watch lochia, breasts, urination, and wounds. Fever at or above 38°C needs care. This is general health education and does not replace an in-person medical assessment. | | | | | | |
| **Item / 项目** | | **Q1 / 问题1** | | **Q2 / 问题2** | | **Q3 / 问题3** |
| **Patient question (Chinese original) / 患者原始问题（中文）** | | 我产后腰背痛，是不是打无痛留下的后遗症？ | | 喂奶姿势不好会不会加重腰背痛？ | | 腰痛伴发热或下肢麻木时需要检查吗？ |
| **English translation of patient question / 患者问题英文翻译** | | Is postpartum back pain a long-term side effect of epidural anesthesia? | | Can poor breastfeeding posture worsen back pain? | | If back pain comes with fever or leg numbness, do I need an examination? |
| **Model response (Chinese) / 模型回答（中文）** | | 产后腰背痛多数与孕期负担、抱娃喂奶姿势和肌肉劳损有关，不一定是无痛后遗症。重点是正确含接、有效排奶和休息补水；若红肿热痛、发热或24小时不缓解，应就医。 | | 喂奶姿势不好会明显加重腰背痛，建议用枕头支撑、脚下垫高、避免低头弯腰。重点是正确含接、有效排奶和休息补水；若红肿热痛、发热或24小时不缓解，应就医。 | | 腰痛伴发热、下肢麻木无力、大小便异常或疼痛进行性加重，应及时检查。建议复测体温，并观察恶露、乳房、尿痛和伤口；若体温≥38℃、寒战或精神差，应就医。 |
| **English translation of model response / 模型回答英文翻译** | | Postpartum back pain is usually related to pregnancy load, holding and feeding posture, and muscle strain. It is not necessarily a long-term effect of epidural anesthesia. Increase exercises gradually and avoid early high intensity. Persistent or worsening symptoms need rehabilitation assessment. | | Poor breastfeeding posture can worsen back pain. Use pillows for support, raise the feet if needed, and avoid bending the neck and back for long periods. Focus on effective milk removal and correct latch. Redness, fever, or no improvement within 24 hours needs care. | | Back pain with fever, leg numbness or weakness, bowel or bladder changes, or progressive worsening should be examined promptly. Recheck temperature and watch lochia, breasts, urination, and wounds. Fever at or above 38°C needs care. |
| **Overall evaluation item / 整体评价项目** | | | **Score / 分数** | | **Comments / 备注** | |
| **Patient Satisfaction / 患者满意度** | | | 4 | |  | |
| **Patient Understandability / 患者可理解性** | | | 4 | |  | |
| **Patient Usefulness / 患者实用性** | | | 4 | |  | |
| **Clinician Correctness / 医生评分：正确性** | | | 4 | |  | |
| **Clinician Completeness / 医生评分：完整性** | | | 4 | |  | |
| **Clinician Readability / 医生评分：可读性** | | | 4 | |  | |
| **Clinician Helpfulness / 医生评分：帮助性** | | | 4 | |  | |
| **Clinician Safety / 医生评分：安全性** | | | 5 | |  | |

**Patient 113**

| **Patient ID / 患者编号** | **Group / 模型组别** | | | | | |
| --- | --- | --- | --- | --- | --- | --- |
| P113 | ChatGPT 5.5 | | | | | |
| **General model response or patient education material (Chinese) / 模型总体回答或患者教育材料（中文）** | | | | | | |
| 剖宫产麻醉后腿麻通常会随着麻醉药代谢逐渐恢复，具体时间因麻醉方式和个人情况不同。哺乳期用药应告知医生正在哺乳，按医嘱选择；不要自行加量或长期使用。第一次下床必须有人搀扶，先在床边坐稳，确认无头晕、腿软后再慢慢站立。建议观察变化，保证休息、饮食和清洁护理；若症状持续不缓解或逐渐加重，应联系产科门诊。若下肢无力、麻木持续不缓解、单侧明显异常或伴大小便异常，应立即告诉医生。建议适量饮水、规律排尿；若尿痛加重、发热、腰痛或排尿困难持续，应查尿常规。以上内容为一般健康教育，不能替代线下面诊。 | | | | | | |
| **English translation of the Chinese general response / 中文总体回答英文翻译** | | | | | | |
| Leg numbness after cesarean anesthesia usually improves as the anesthetic wears off, but timing varies by anesthesia type and individual condition. Observe changes and maintain rest, nutrition, and hygiene. Persistent or worsening symptoms need obstetric advice. The first time getting out of bed should be assisted. Sit at the bedside first, make sure there is no dizziness or leg weakness, then stand slowly. Observe changes and maintain rest, nutrition, and hygiene. Persistent or worsening symptoms need obstetric advice. Persistent leg weakness, numbness that does not improve, obvious one-sided symptoms, or bowel or bladder problems should be reported to a doctor immediately. Drink fluids and urinate regularly. Worsening pain, fever, back pain, or persistent difficulty needs urine testing. This is general health education and does not replace an in-person medical assessment. | | | | | | |
| **Item / 项目** | | **Q1 / 问题1** | | **Q2 / 问题2** | | **Q3 / 问题3** |
| **Patient question (Chinese original) / 患者原始问题（中文）** | | 剖宫产麻醉后腿麻多久能恢复？ | | 麻醉后下床第一次走路需要人扶吗？ | | 如果下肢一直无力或感觉异常，需要找医生吗？ |
| **English translation of patient question / 患者问题英文翻译** | | How long does leg numbness after cesarean anesthesia take to recover? | | Do I need someone to support me the first time I walk after anesthesia? | | If leg weakness or abnormal sensation persists, should I contact a doctor? |
| **Model response (Chinese) / 模型回答（中文）** | | 剖宫产麻醉后腿麻通常会随着麻醉药代谢逐渐恢复，具体时间因麻醉方式和个人情况不同。哺乳期用药应告知医生正在哺乳，按医嘱选择；不要自行加量或长期使用。 | | 第一次下床必须有人搀扶，先在床边坐稳，确认无头晕、腿软后再慢慢站立。建议观察变化，保证休息、饮食和清洁护理；若症状持续不缓解或逐渐加重，应联系产科门诊。 | | 若下肢无力、麻木持续不缓解、单侧明显异常或伴大小便异常，应立即告诉医生。建议适量饮水、规律排尿；若尿痛加重、发热、腰痛或排尿困难持续，应查尿常规。 |
| **English translation of model response / 模型回答英文翻译** | | Leg numbness after cesarean anesthesia usually improves as the anesthetic wears off, but timing varies by anesthesia type and individual condition. Observe changes and maintain rest, nutrition, and hygiene. Persistent or worsening symptoms need obstetric advice. | | The first time getting out of bed should be assisted. Sit at the bedside first, make sure there is no dizziness or leg weakness, then stand slowly. Observe changes and maintain rest, nutrition, and hygiene. Persistent or worsening symptoms need obstetric advice. | | Persistent leg weakness, numbness that does not improve, obvious one-sided symptoms, or bowel or bladder problems should be reported to a doctor immediately. Drink fluids and urinate regularly. Worsening pain, fever, back pain, or persistent difficulty needs urine testing. |
| **Overall evaluation item / 整体评价项目** | | | **Score / 分数** | | **Comments / 备注** | |
| **Patient Satisfaction / 患者满意度** | | | 4 | |  | |
| **Patient Understandability / 患者可理解性** | | | 4 | |  | |
| **Patient Usefulness / 患者实用性** | | | 4 | |  | |
| **Clinician Correctness / 医生评分：正确性** | | | 5 | |  | |
| **Clinician Completeness / 医生评分：完整性** | | | 5 | |  | |
| **Clinician Readability / 医生评分：可读性** | | | 4 | |  | |
| **Clinician Helpfulness / 医生评分：帮助性** | | | 5 | |  | |
| **Clinician Safety / 医生评分：安全性** | | | 5 | |  | |

**Patient 114**

| **Patient ID / 患者编号** | **Group / 模型组别** | | | | | |
| --- | --- | --- | --- | --- | --- | --- |
| P114 | ChatGPT 5.5 | | | | | |
| **General model response or patient education material (Chinese) / 模型总体回答或患者教育材料（中文）** | | | | | | |
| 产后腿部偶尔麻木可能与久坐、姿势压迫、腰椎问题或神经牵拉有关。建议观察变化，保证休息、饮食和清洁护理；若症状持续不缓解或逐渐加重，应联系产科门诊。坐久腿麻时应改变姿势、起身活动、避免盘腿久坐，并注意喂奶时腰背支撑。重点是正确含接、有效排奶和休息补水；若红肿热痛、发热或24小时不缓解，应就医。若一侧腿麻伴肿胀、疼痛、发热或皮肤颜色改变，不要按摩，应尽快就医排查血栓。建议复测体温，并观察恶露、乳房、尿痛和伤口；若体温≥38℃、寒战或精神差，应就医。以上内容为一般健康教育，不能替代线下面诊。 | | | | | | |
| **English translation of the Chinese general response / 中文总体回答英文翻译** | | | | | | |
| Occasional leg numbness after birth may be related to prolonged sitting, posture compression, lumbar problems, or nerve stretching. Observe changes and maintain rest, nutrition, and hygiene. Persistent or worsening symptoms need obstetric advice. If numbness occurs after sitting, change position, stand and move, avoid sitting cross-legged for long periods, and support the back during feeding. Resume only when wounds and lochia are stable, and use contraception. Pain or bleeding needs medical advice. One-sided numbness with swelling, pain, warmth, or skin color change should not be massaged. Seek medical care to rule out thrombosis. Monitor blood pressure and symptoms. Rising pressure, headache, visual changes, or swelling needs prompt care. This is general health education and does not replace an in-person medical assessment. | | | | | | |
| **Item / 项目** | | **Q1 / 问题1** | | **Q2 / 问题2** | | **Q3 / 问题3** |
| **Patient question (Chinese original) / 患者原始问题（中文）** | | 产后腿部偶尔麻木，是压迫神经了吗？ | | 坐久了腿麻应该怎么调整姿势？ | | 如果一条腿麻木伴肿胀疼痛，需要排查血栓吗？ |
| **English translation of patient question / 患者问题英文翻译** | | My legs are occasionally numb after birth. Could it be nerve compression? | | How should I adjust my posture if my legs become numb after sitting too long? | | If one leg is numb with swelling and pain, should thrombosis be ruled out? |
| **Model response (Chinese) / 模型回答（中文）** | | 产后腿部偶尔麻木可能与久坐、姿势压迫、腰椎问题或神经牵拉有关。建议观察变化，保证休息、饮食和清洁护理；若症状持续不缓解或逐渐加重，应联系产科门诊。 | | 坐久腿麻时应改变姿势、起身活动、避免盘腿久坐，并注意喂奶时腰背支撑。重点是正确含接、有效排奶和休息补水；若红肿热痛、发热或24小时不缓解，应就医。 | | 若一侧腿麻伴肿胀、疼痛、发热或皮肤颜色改变，不要按摩，应尽快就医排查血栓。建议复测体温，并观察恶露、乳房、尿痛和伤口；若体温≥38℃、寒战或精神差，应就医。 |
| **English translation of model response / 模型回答英文翻译** | | Occasional leg numbness after birth may be related to prolonged sitting, posture compression, lumbar problems, or nerve stretching. Observe changes and maintain rest, nutrition, and hygiene. Persistent or worsening symptoms need obstetric advice. | | If numbness occurs after sitting, change position, stand and move, avoid sitting cross-legged for long periods, and support the back during feeding. Resume only when wounds and lochia are stable, and use contraception. Pain or bleeding needs medical advice. | | One-sided numbness with swelling, pain, warmth, or skin color change should not be massaged. Seek medical care to rule out thrombosis. Monitor blood pressure and symptoms. Rising pressure, headache, visual changes, or swelling needs prompt care. |
| **Overall evaluation item / 整体评价项目** | | | **Score / 分数** | | **Comments / 备注** | |
| **Patient Satisfaction / 患者满意度** | | | 4 | |  | |
| **Patient Understandability / 患者可理解性** | | | 4 | |  | |
| **Patient Usefulness / 患者实用性** | | | 4 | |  | |
| **Clinician Correctness / 医生评分：正确性** | | | 5 | |  | |
| **Clinician Completeness / 医生评分：完整性** | | | 4 | |  | |
| **Clinician Readability / 医生评分：可读性** | | | 4 | |  | |
| **Clinician Helpfulness / 医生评分：帮助性** | | | 4 | |  | |
| **Clinician Safety / 医生评分：安全性** | | | 5 | |  | |

**Patient 115**

| **Patient ID / 患者编号** | **Group / 模型组别** | | | | | |
| --- | --- | --- | --- | --- | --- | --- |
| P115 | ChatGPT 5.5 | | | | | |
| **General model response or patient education material (Chinese) / 模型总体回答或患者教育材料（中文）** | | | | | | |
| 预防产后血栓应尽早适量活动、踝泵运动、补充水分，避免长时间卧床不动。不要按摩肿痛肢体；若单侧腿肿痛、胸闷气短、胸痛或晕厥，应立即就医。弹力袜对部分高风险产妇有帮助，但尺寸和压力等级应由医护人员指导。不要按摩肿痛肢体；若单侧腿肿痛、胸闷气短、胸痛或晕厥，应立即就医。小腿突然肿痛、单侧发热或压痛时不要按摩，以免血栓脱落，应立即就医。建议复测体温，并观察恶露、乳房、尿痛和伤口；若体温≥38℃、寒战或精神差，应就医。以上内容为一般健康教育，不能替代线下面诊。 | | | | | | |
| **English translation of the Chinese general response / 中文总体回答英文翻译** | | | | | | |
| To prevent postpartum thrombosis, begin gentle activity as early as allowed, do ankle pumps, drink enough fluids, and avoid prolonged bed rest. Increase activity gradually. Stop and seek review if bleeding, abdominal pain, dizziness, or incision pain occurs. Compression stockings may help some high-risk mothers, but size and pressure level should be guided by medical staff. Observe changes and maintain rest, nutrition, and hygiene. Persistent or worsening symptoms need obstetric advice. Sudden calf swelling, one-sided warmth, or tenderness should not be massaged because a clot could dislodge. Seek medical care immediately. Monitor blood pressure and symptoms. Rising pressure, headache, visual changes, or swelling needs prompt care. This is general health education and does not replace an in-person medical assessment. | | | | | | |
| **Item / 项目** | | **Q1 / 问题1** | | **Q2 / 问题2** | | **Q3 / 问题3** |
| **Patient question (Chinese original) / 患者原始问题（中文）** | | 我产后活动少，担心血栓，平时怎么预防？ | | 穿弹力袜对预防血栓有帮助吗？ | | 如果小腿突然肿痛，能不能先在家按摩？ |
| **English translation of patient question / 患者问题英文翻译** | | I am less active after birth and worry about thrombosis. How can I prevent it? | | Do compression stockings help prevent thrombosis? | | If my calf suddenly becomes swollen and painful, can I massage it at home first? |
| **Model response (Chinese) / 模型回答（中文）** | | 预防产后血栓应尽早适量活动、踝泵运动、补充水分，避免长时间卧床不动。不要按摩肿痛肢体；若单侧腿肿痛、胸闷气短、胸痛或晕厥，应立即就医。 | | 弹力袜对部分高风险产妇有帮助，但尺寸和压力等级应由医护人员指导。不要按摩肿痛肢体；若单侧腿肿痛、胸闷气短、胸痛或晕厥，应立即就医。 | | 小腿突然肿痛、单侧发热或压痛时不要按摩，以免血栓脱落，应立即就医。建议复测体温，并观察恶露、乳房、尿痛和伤口；若体温≥38℃、寒战或精神差，应就医。 |
| **English translation of model response / 模型回答英文翻译** | | To prevent postpartum thrombosis, begin gentle activity as early as allowed, do ankle pumps, drink enough fluids, and avoid prolonged bed rest. Increase activity gradually. Stop and seek review if bleeding, abdominal pain, dizziness, or incision pain occurs. | | Compression stockings may help some high-risk mothers, but size and pressure level should be guided by medical staff. Observe changes and maintain rest, nutrition, and hygiene. Persistent or worsening symptoms need obstetric advice. | | Sudden calf swelling, one-sided warmth, or tenderness should not be massaged because a clot could dislodge. Seek medical care immediately. Monitor blood pressure and symptoms. Rising pressure, headache, visual changes, or swelling needs prompt care. |
| **Overall evaluation item / 整体评价项目** | | | **Score / 分数** | | **Comments / 备注** | |
| **Patient Satisfaction / 患者满意度** | | | 4 | |  | |
| **Patient Understandability / 患者可理解性** | | | 4 | |  | |
| **Patient Usefulness / 患者实用性** | | | 4 | |  | |
| **Clinician Correctness / 医生评分：正确性** | | | 5 | |  | |
| **Clinician Completeness / 医生评分：完整性** | | | 4 | |  | |
| **Clinician Readability / 医生评分：可读性** | | | 4 | |  | |
| **Clinician Helpfulness / 医生评分：帮助性** | | | 4 | |  | |
| **Clinician Safety / 医生评分：安全性** | | | 5 | |  | |

**Patient 116**

| **Patient ID / 患者编号** | **Group / 模型组别** | | | | | |
| --- | --- | --- | --- | --- | --- | --- |
| P116 | ChatGPT 5.5 | | | | | |
| **General model response or patient education material (Chinese) / 模型总体回答或患者教育材料（中文）** | | | | | | |
| 产后突然胸痛、气短可能与肺栓塞、心脏问题、严重贫血、焦虑或感染有关，需高度重视。建议复测体温，并观察恶露、乳房、尿痛和伤口；若体温≥38℃、寒战或精神差，应就医。心慌可能由贫血、睡眠不足、甲状腺异常或心脏问题引起，需结合血压、脉搏和症状检查。建议按医嘱测血压，记录头痛、眼花和水肿；若血压升高或症状明显，应及时就医。胸闷气短伴出冷汗、胸痛、晕厥、咳血或一侧腿肿痛，应立即急诊。不要按摩肿痛肢体；若单侧腿肿痛、胸闷气短、胸痛或晕厥，应立即就医。以上内容为一般健康教育，不能替代线下面诊。 | | | | | | |
| **English translation of the Chinese general response / 中文总体回答英文翻译** | | | | | | |
| Sudden chest pain and shortness of breath after birth may be related to pulmonary embolism, heart disease, severe anemia, anxiety, or infection and should be taken seriously. Recheck temperature and watch lochia, breasts, urination, and wounds. Fever at or above 38°C needs care. Palpitations may result from anemia, poor sleep, thyroid problems, or heart conditions. Blood pressure, pulse, and symptoms should be assessed. Monitor blood pressure and symptoms. Rising pressure, headache, visual changes, or swelling needs prompt care. Chest tightness and shortness of breath with cold sweats, chest pain, fainting, coughing blood, or one-sided leg swelling and pain requires emergency care. Monitor blood pressure and symptoms. Rising pressure, headache, visual changes, or swelling needs prompt care. This is general health education and does not replace an in-person medical assessment. | | | | | | |
| **Item / 项目** | | **Q1 / 问题1** | | **Q2 / 问题2** | | **Q3 / 问题3** |
| **Patient question (Chinese original) / 患者原始问题（中文）** | | 产后突然胸口疼、气短，可能是什么原因？ | | 产后心慌是贫血还是心脏问题？ | | 胸闷气短伴出冷汗时是不是要急诊？ |
| **English translation of patient question / 患者问题英文翻译** | | What could sudden chest pain and shortness of breath after birth mean? | | Is postpartum palpitation caused by anemia or a heart problem? | | Does chest tightness and shortness of breath with cold sweats require emergency care? |
| **Model response (Chinese) / 模型回答（中文）** | | 产后突然胸痛、气短可能与肺栓塞、心脏问题、严重贫血、焦虑或感染有关，需高度重视。建议复测体温，并观察恶露、乳房、尿痛和伤口；若体温≥38℃、寒战或精神差，应就医。 | | 心慌可能由贫血、睡眠不足、甲状腺异常或心脏问题引起，需结合血压、脉搏和症状检查。建议按医嘱测血压，记录头痛、眼花和水肿；若血压升高或症状明显，应及时就医。 | | 胸闷气短伴出冷汗、胸痛、晕厥、咳血或一侧腿肿痛，应立即急诊。不要按摩肿痛肢体；若单侧腿肿痛、胸闷气短、胸痛或晕厥，应立即就医。 |
| **English translation of model response / 模型回答英文翻译** | | Sudden chest pain and shortness of breath after birth may be related to pulmonary embolism, heart disease, severe anemia, anxiety, or infection and should be taken seriously. Recheck temperature and watch lochia, breasts, urination, and wounds. Fever at or above 38°C needs care. | | Palpitations may result from anemia, poor sleep, thyroid problems, or heart conditions. Blood pressure, pulse, and symptoms should be assessed. Monitor blood pressure and symptoms. Rising pressure, headache, visual changes, or swelling needs prompt care. | | Chest tightness and shortness of breath with cold sweats, chest pain, fainting, coughing blood, or one-sided leg swelling and pain requires emergency care. Monitor blood pressure and symptoms. Rising pressure, headache, visual changes, or swelling needs prompt care. |
| **Overall evaluation item / 整体评价项目** | | | **Score / 分数** | | **Comments / 备注** | |
| **Patient Satisfaction / 患者满意度** | | | 4 | |  | |
| **Patient Understandability / 患者可理解性** | | | 5 | |  | |
| **Patient Usefulness / 患者实用性** | | | 4 | |  | |
| **Clinician Correctness / 医生评分：正确性** | | | 4 | |  | |
| **Clinician Completeness / 医生评分：完整性** | | | 4 | |  | |
| **Clinician Readability / 医生评分：可读性** | | | 4 | |  | |
| **Clinician Helpfulness / 医生评分：帮助性** | | | 4 | |  | |
| **Clinician Safety / 医生评分：安全性** | | | 5 | |  | |

**Patient 117**

| **Patient ID / 患者编号** | **Group / 模型组别** | | | | | |
| --- | --- | --- | --- | --- | --- | --- |
| P117 | ChatGPT 5.5 | | | | | |
| **General model response or patient education material (Chinese) / 模型总体回答或患者教育材料（中文）** | | | | | | |
| 糖尿病产妇切口愈合可能更慢，感染风险也较高，需要加强血糖和伤口管理。建议复测体温，并观察恶露、乳房、尿痛和伤口；若体温≥38℃、寒战或精神差，应就医。血糖控制不好会影响白细胞功能和组织修复，增加切口感染、裂开和渗液风险。建议复测体温，并观察恶露、乳房、尿痛和伤口；若体温≥38℃、寒战或精神差，应就医。切口有渗液、红肿、疼痛或发热时，应复查伤口并同时监测血糖，必要时调整降糖方案。建议复测体温，并观察恶露、乳房、尿痛和伤口；若体温≥38℃、寒战或精神差，应就医。以上内容为一般健康教育，不能替代线下面诊。 | | | | | | |
| **English translation of the Chinese general response / 中文总体回答英文翻译** | | | | | | |
| In mothers with diabetes, cesarean incision healing may be slower and infection risk may be higher, so blood glucose and wound care need attention. Recheck temperature and watch lochia, breasts, urination, and wounds. Fever at or above 38°C needs care. Poor glucose control can impair immune function and tissue repair, increasing the risk of infection, wound opening, and drainage. Recheck temperature and watch lochia, breasts, urination, and wounds. Fever at or above 38°C needs care. If the incision drains fluid, becomes red, painful, or fever occurs, the wound should be checked and blood glucose monitored. Diabetes treatment may need adjustment. Record color, amount, odor, and pad use. Seek care for heavy bleeding, clots, pain, or fever. This is general health education and does not replace an in-person medical assessment. | | | | | | |
| **Item / 项目** | | **Q1 / 问题1** | | **Q2 / 问题2** | | **Q3 / 问题3** |
| **Patient question (Chinese original) / 患者原始问题（中文）** | | 我有糖尿病，剖宫产切口恢复会不会更慢？ | | 血糖控制不好会影响伤口愈合吗？ | | 切口有渗液时需要同时复查血糖吗？ |
| **English translation of patient question / 患者问题英文翻译** | | I have diabetes. Will my cesarean incision heal more slowly? | | Will poor blood sugar control affect wound healing? | | If the incision drains fluid, should I also recheck blood sugar? |
| **Model response (Chinese) / 模型回答（中文）** | | 糖尿病产妇切口愈合可能更慢，感染风险也较高，需要加强血糖和伤口管理。建议复测体温，并观察恶露、乳房、尿痛和伤口；若体温≥38℃、寒战或精神差，应就医。 | | 血糖控制不好会影响白细胞功能和组织修复，增加切口感染、裂开和渗液风险。建议复测体温，并观察恶露、乳房、尿痛和伤口；若体温≥38℃、寒战或精神差，应就医。 | | 切口有渗液、红肿、疼痛或发热时，应复查伤口并同时监测血糖，必要时调整降糖方案。建议复测体温，并观察恶露、乳房、尿痛和伤口；若体温≥38℃、寒战或精神差，应就医。 |
| **English translation of model response / 模型回答英文翻译** | | In mothers with diabetes, cesarean incision healing may be slower and infection risk may be higher, so blood glucose and wound care need attention. Recheck temperature and watch lochia, breasts, urination, and wounds. Fever at or above 38°C needs care. | | Poor glucose control can impair immune function and tissue repair, increasing the risk of infection, wound opening, and drainage. Recheck temperature and watch lochia, breasts, urination, and wounds. Fever at or above 38°C needs care. | | If the incision drains fluid, becomes red, painful, or fever occurs, the wound should be checked and blood glucose monitored. Diabetes treatment may need adjustment. Record color, amount, odor, and pad use. Seek care for heavy bleeding, clots, pain, or fever. |
| **Overall evaluation item / 整体评价项目** | | | **Score / 分数** | | **Comments / 备注** | |
| **Patient Satisfaction / 患者满意度** | | | 5 | |  | |
| **Patient Understandability / 患者可理解性** | | | 4 | |  | |
| **Patient Usefulness / 患者实用性** | | | 4 | |  | |
| **Clinician Correctness / 医生评分：正确性** | | | 5 | |  | |
| **Clinician Completeness / 医生评分：完整性** | | | 4 | |  | |
| **Clinician Readability / 医生评分：可读性** | | | 4 | |  | |
| **Clinician Helpfulness / 医生评分：帮助性** | | | 5 | |  | |
| **Clinician Safety / 医生评分：安全性** | | | 5 | |  | |

**Patient 118**

| **Patient ID / 患者编号** | **Group / 模型组别** | | | | | |
| --- | --- | --- | --- | --- | --- | --- |
| P118 | ChatGPT 5.5 | | | | | |
| **General model response or patient education material (Chinese) / 模型总体回答或患者教育材料（中文）** | | | | | | |
| 产后尿频尿急加重可能是尿路感染，也可能与膀胱恢复有关，应查尿常规。建议复测体温，并观察恶露、乳房、尿痛和伤口；若体温≥38℃、寒战或精神差，应就医。哺乳期尿路感染可以选择相对安全的药物治疗，不建议自行买药或拖延。建议复测体温，并观察恶露、乳房、尿痛和伤口；若体温≥38℃、寒战或精神差，应就医。若伴腰痛、发热、寒战、恶心或尿血，应警惕肾盂肾炎，需尽快就医。建议复测体温，并观察恶露、乳房、尿痛和伤口；若体温≥38℃、寒战或精神差，应就医。以上内容为一般健康教育，不能替代线下面诊。 | | | | | | |
| **English translation of the Chinese general response / 中文总体回答英文翻译** | | | | | | |
| Worsening urinary frequency and urgency after birth may be urinary tract infection, though bladder recovery can also contribute. Urine testing is needed. Recheck temperature and watch lochia, breasts, urination, and wounds. Fever at or above 38°C needs care. Urinary tract infection can be treated during breastfeeding with medicines selected for safety. Do not buy medicine by yourself or delay care. Recheck temperature and watch lochia, breasts, urination, and wounds. Fever at or above 38°C needs care. Back pain, fever, chills, nausea, or blood in urine may suggest pyelonephritis and requires prompt medical care. Recheck temperature and watch lochia, breasts, urination, and wounds. Fever at or above 38°C needs care. This is general health education and does not replace an in-person medical assessment. | | | | | | |
| **Item / 项目** | | **Q1 / 问题1** | | **Q2 / 问题2** | | **Q3 / 问题3** |
| **Patient question (Chinese original) / 患者原始问题（中文）** | | 产后尿频尿急越来越明显，是尿路感染吗？ | | 哺乳期尿路感染可以吃药吗？ | | 如果有腰痛或发热，需要警惕肾盂肾炎吗？ |
| **English translation of patient question / 患者问题英文翻译** | | My urinary frequency and urgency are getting worse after birth. Is it a urinary tract infection? | | Can urinary tract infection be treated with medicine while breastfeeding? | | If I have back pain or fever, should I be concerned about pyelonephritis? |
| **Model response (Chinese) / 模型回答（中文）** | | 产后尿频尿急加重可能是尿路感染，也可能与膀胱恢复有关，应查尿常规。建议复测体温，并观察恶露、乳房、尿痛和伤口；若体温≥38℃、寒战或精神差，应就医。 | | 哺乳期尿路感染可以选择相对安全的药物治疗，不建议自行买药或拖延。建议复测体温，并观察恶露、乳房、尿痛和伤口；若体温≥38℃、寒战或精神差，应就医。 | | 若伴腰痛、发热、寒战、恶心或尿血，应警惕肾盂肾炎，需尽快就医。建议复测体温，并观察恶露、乳房、尿痛和伤口；若体温≥38℃、寒战或精神差，应就医。 |
| **English translation of model response / 模型回答英文翻译** | | Worsening urinary frequency and urgency after birth may be urinary tract infection, though bladder recovery can also contribute. Urine testing is needed. Recheck temperature and watch lochia, breasts, urination, and wounds. Fever at or above 38°C needs care. | | Urinary tract infection can be treated during breastfeeding with medicines selected for safety. Do not buy medicine by yourself or delay care. Recheck temperature and watch lochia, breasts, urination, and wounds. Fever at or above 38°C needs care. | | Back pain, fever, chills, nausea, or blood in urine may suggest pyelonephritis and requires prompt medical care. Recheck temperature and watch lochia, breasts, urination, and wounds. Fever at or above 38°C needs care. |
| **Overall evaluation item / 整体评价项目** | | | **Score / 分数** | | **Comments / 备注** | |
| **Patient Satisfaction / 患者满意度** | | | 4 | |  | |
| **Patient Understandability / 患者可理解性** | | | 4 | |  | |
| **Patient Usefulness / 患者实用性** | | | 4 | |  | |
| **Clinician Correctness / 医生评分：正确性** | | | 5 | |  | |
| **Clinician Completeness / 医生评分：完整性** | | | 4 | |  | |
| **Clinician Readability / 医生评分：可读性** | | | 4 | |  | |
| **Clinician Helpfulness / 医生评分：帮助性** | | | 4 | |  | |
| **Clinician Safety / 医生评分：安全性** | | | 5 | |  | |

**Patient 119**

| **Patient ID / 患者编号** | **Group / 模型组别** | | | | | |
| --- | --- | --- | --- | --- | --- | --- |
| P119 | ChatGPT 5.5 | | | | | |
| **General model response or patient education material (Chinese) / 模型总体回答或患者教育材料（中文）** | | | | | | |
| 产后腰两侧酸痛可能来自肌肉劳损、喂奶姿势，也可能与泌尿系统问题有关，要看尿液和发热情况。建议复测体温，并观察恶露、乳房、尿痛和伤口；若体温≥38℃、寒战或精神差，应就医。喝水太少会增加尿液浓缩、尿痛和尿路感染风险，哺乳期更要适量补水。建议复测体温，并观察恶露、乳房、尿痛和伤口；若体温≥38℃、寒战或精神差，应就医。尿色很深、尿血、尿痛、发热或腰痛明显时，应尽快查尿常规和肾功能。建议复测体温，并观察恶露、乳房、尿痛和伤口；若体温≥38℃、寒战或精神差，应就医。以上内容为一般健康教育，不能替代线下面诊。 | | | | | | |
| **English translation of the Chinese general response / 中文总体回答英文翻译** | | | | | | |
| Soreness on both sides of the lower back after birth may be muscle strain or feeding posture, but urinary tract problems are also possible depending on urine symptoms and fever. Recheck temperature and watch lochia, breasts, urination, and wounds. Fever at or above 38°C needs care. Too little water can concentrate urine and increase the risk of painful urination and urinary tract infection. Adequate fluids are especially important during breastfeeding. Recheck temperature and watch lochia, breasts, urination, and wounds. Fever at or above 38°C needs care. Very dark urine, blood in urine, painful urination, fever, or marked flank pain should be tested promptly with urine studies and kidney function if needed. Recheck temperature and watch lochia, breasts, urination, and wounds. Fever at or above 38°C needs care. This is general health education and does not replace an in-person medical assessment. | | | | | | |
| **Item / 项目** | | **Q1 / 问题1** | | **Q2 / 问题2** | | **Q3 / 问题3** |
| **Patient question (Chinese original) / 患者原始问题（中文）** | | 我产后腰两侧酸痛，和肾脏有关系吗？ | | 喝水少会不会导致尿路问题？ | | 如果尿色很深或尿血，需要马上检查吗？ |
| **English translation of patient question / 患者问题英文翻译** | | My lower back on both sides is sore after birth. Could it be related to the kidneys? | | Can drinking too little water cause urinary problems? | | If my urine is very dark or bloody, do I need immediate testing? |
| **Model response (Chinese) / 模型回答（中文）** | | 产后腰两侧酸痛可能来自肌肉劳损、喂奶姿势，也可能与泌尿系统问题有关，要看尿液和发热情况。建议复测体温，并观察恶露、乳房、尿痛和伤口；若体温≥38℃、寒战或精神差，应就医。 | | 喝水太少会增加尿液浓缩、尿痛和尿路感染风险，哺乳期更要适量补水。建议复测体温，并观察恶露、乳房、尿痛和伤口；若体温≥38℃、寒战或精神差，应就医。 | | 尿色很深、尿血、尿痛、发热或腰痛明显时，应尽快查尿常规和肾功能。建议复测体温，并观察恶露、乳房、尿痛和伤口；若体温≥38℃、寒战或精神差，应就医。 |
| **English translation of model response / 模型回答英文翻译** | | Soreness on both sides of the lower back after birth may be muscle strain or feeding posture, but urinary tract problems are also possible depending on urine symptoms and fever. Recheck temperature and watch lochia, breasts, urination, and wounds. Fever at or above 38°C needs care. | | Too little water can concentrate urine and increase the risk of painful urination and urinary tract infection. Adequate fluids are especially important during breastfeeding. Recheck temperature and watch lochia, breasts, urination, and wounds. Fever at or above 38°C needs care. | | Very dark urine, blood in urine, painful urination, fever, or marked flank pain should be tested promptly with urine studies and kidney function if needed. Recheck temperature and watch lochia, breasts, urination, and wounds. Fever at or above 38°C needs care. |
| **Overall evaluation item / 整体评价项目** | | | **Score / 分数** | | **Comments / 备注** | |
| **Patient Satisfaction / 患者满意度** | | | 4 | |  | |
| **Patient Understandability / 患者可理解性** | | | 4 | |  | |
| **Patient Usefulness / 患者实用性** | | | 4 | |  | |
| **Clinician Correctness / 医生评分：正确性** | | | 5 | |  | |
| **Clinician Completeness / 医生评分：完整性** | | | 4 | |  | |
| **Clinician Readability / 医生评分：可读性** | | | 4 | |  | |
| **Clinician Helpfulness / 医生评分：帮助性** | | | 4 | |  | |
| **Clinician Safety / 医生评分：安全性** | | | 5 | |  | |

**Patient 120**

| **Patient ID / 患者编号** | **Group / 模型组别** | | | | | |
| --- | --- | --- | --- | --- | --- | --- |
| P120 | ChatGPT 5.5 | | | | | |
| **General model response or patient education material (Chinese) / 模型总体回答或患者教育材料（中文）** | | | | | | |
| 产褥期必须立即就医的情况包括大量出血、1小时湿透卫生巾、大血块、发热、恶露臭味、剧烈腹痛、切口裂开或流脓。建议记录颜色、量和气味，避免劳累；若1小时湿透卫生巾、血块增多、腹痛或发热，应就医。严重头痛、眼花、血压升高、胸闷气短、胸痛、晕厥、一侧腿肿痛、明显抑郁或伤害自己和宝宝的想法，都是危险信号。建议记录颜色、量和气味，避免劳累；若1小时湿透卫生巾、血块增多、腹痛或发热，应就医。夜间突然不舒服且症状明显时，可以直接去急诊，不要等到第二天；同时让家人陪同并带上产检、分娩和用药资料。哺乳期用药应告知医生正在哺乳，按医嘱选择；不要自行加量或长期使用。以上内容为一般健康教育，不能替代线下面诊。 | | | | | | |
| **English translation of the Chinese general response / 中文总体回答英文翻译** | | | | | | |
| During the puerperium, seek immediate care for heavy bleeding, soaking one pad within an hour, large clots, fever, foul lochia, severe abdominal pain, wound opening, or pus. Record color, amount, odor, and pad use. Seek care for heavy bleeding, clots, pain, or fever. Severe headache, visual symptoms, high blood pressure, chest tightness, shortness of breath, chest pain, fainting, one-sided leg swelling and pain, severe depression, or thoughts of harming yourself or the baby are danger signs. If you suddenly feel clearly unwell at night, go directly to the emergency department rather than waiting until morning. Have a family member accompany you and bring prenatal, delivery, and medication records. This is general health education and does not replace an in-person medical assessment. | | | | | | |
| **Item / 项目** | | **Q1 / 问题1** | | **Q2 / 问题2** | | **Q3 / 问题3** |
| **Patient question (Chinese original) / 患者原始问题（中文）** | | 产褥期哪些症状属于必须马上去医院的情况？ | | 恶露、发热、头痛和胸闷这些症状怎么判断轻重？ | | 如果夜间突然不舒服，可以直接去急诊吗？ |
| **English translation of patient question / 患者问题英文翻译** | | What symptoms during the puerperium require going to the hospital immediately? | | How should I judge the severity of lochia, fever, headache, and chest tightness? | | If I suddenly feel unwell at night, can I go directly to the emergency department? |
| **Model response (Chinese) / 模型回答（中文）** | | 产褥期必须立即就医的情况包括大量出血、1小时湿透卫生巾、大血块、发热、恶露臭味、剧烈腹痛、切口裂开或流脓。建议记录颜色、量和气味，避免劳累；若1小时湿透卫生巾、血块增多、腹痛或发热，应就医。 | | 严重头痛、眼花、血压升高、胸闷气短、胸痛、晕厥、一侧腿肿痛、明显抑郁或伤害自己和宝宝的想法，都是危险信号。建议记录颜色、量和气味，避免劳累；若1小时湿透卫生巾、血块增多、腹痛或发热，应就医。 | | 夜间突然不舒服且症状明显时，可以直接去急诊，不要等到第二天；同时让家人陪同并带上产检、分娩和用药资料。哺乳期用药应告知医生正在哺乳，按医嘱选择；不要自行加量或长期使用。 |
| **English translation of model response / 模型回答英文翻译** | | During the puerperium, seek immediate care for heavy bleeding, soaking one pad within an hour, large clots, fever, foul lochia, severe abdominal pain, wound opening, or pus. Record color, amount, odor, and pad use. Seek care for heavy bleeding, clots, pain, or fever. | | Severe headache, visual symptoms, high blood pressure, chest tightness, shortness of breath, chest pain, fainting, one-sided leg swelling and pain, severe depression, or thoughts of harming yourself or the baby are danger signs. | | If you suddenly feel clearly unwell at night, go directly to the emergency department rather than waiting until morning. Have a family member accompany you and bring prenatal, delivery, and medication records. |
| **Overall evaluation item / 整体评价项目** | | | **Score / 分数** | | **Comments / 备注** | |
| **Patient Satisfaction / 患者满意度** | | | 5 | |  | |
| **Patient Understandability / 患者可理解性** | | | 4 | |  | |
| **Patient Usefulness / 患者实用性** | | | 4 | |  | |
| **Clinician Correctness / 医生评分：正确性** | | | 4 | |  | |
| **Clinician Completeness / 医生评分：完整性** | | | 5 | |  | |
| **Clinician Readability / 医生评分：可读性** | | | 4 | |  | |
| **Clinician Helpfulness / 医生评分：帮助性** | | | 5 | |  | |
| **Clinician Safety / 医生评分：安全性** | | | 4 | |  | |
