# Supplementary material for: Beyond accuracy: a three-stage evaluation of large language models for postpartum health consultation and nursing decision support
Source: Front Public Health. 2026 Oct 20;14:1961647. doi: 10.3389/fpubh.2026.1961647 (PMC13727638; doi:10.3389/fpubh.2026.1961647)
Supplement: Supplementary file 3 [file Table_11.docx]

**Supplementary Table S11. Stage 2 DeepSeek V3 patient-derived questions, responses, English translations, and ratings**

Chinese original responses and their English translations are presented together. Ratings were assigned once to each composite evaluation unit based on the original Chinese responses; the English translations were not used for ratings.

| **Model name / 模型名称** | **Evaluator / 评分者** | **Language / 语言** | **Model testing period / 模型测试期** | **Notes / 备注** |
| --- | --- | --- | --- | --- |
| DeepSeek V3 | Corresponding postpartum participant; three blinded clinical experts / 对应产妇；3名盲法临床专家 | Chinese with English translation / 中文及英文翻译 | 1 May to 30 June 2026 / 2026年5月1日至6月30日 | Individual sex, age, and dates were removed to protect participant confidentiality. / 为保护参与者隐私，已删除个体性别、年龄和日期。 |

| **Scope / 范围** | 120 participants for this model; each participant contributed three related questions (Q1–Q3). / 本文件包含该模型对应的120名参与者，每名参与者提供3个相关问题（Q1–Q3）。 |
| --- | --- |
| **Recording and translation rule / 记录与翻译规则** | Chinese original content and English translations are shown together. English translations were generated using a web-based translation tool and manually corrected; they were provided only for international readership and were not used for ratings. / 中文原文与英文翻译置于同一记录块中。英文翻译由网页翻译工具生成并经人工校正，仅供国际读者阅读，不用于评分。 |
| **Scoring rule / 评分规则** | Each participant’s three related questions and the three corresponding model responses constituted one composite evaluation unit. Scores were assigned once to the composite unit based on the original Chinese responses; Chinese and English were not scored separately. / 同一参与者的3个相关问题及模型对应的3个回答构成1个复合评价单元。评分基于中文原始回答，对该复合单元整体评分1次，不对中英文分别评分。 |
| **Participant ratings / 产妇评分** | The corresponding postpartum participant directly rated satisfaction, understandability, and usefulness. / 对应产妇本人直接评价满意度、可理解性和实用性。 |
| **Clinical expert ratings / 临床专家评分** | Three blinded clinical experts independently rated correctness, completeness, readability, helpfulness, and safety; the displayed score is the median of the three ratings. / 3名盲法临床专家独立评价正确性、完整性、可读性、帮助性和安全性；表中分数为3名专家评分的中位数。 |

**Patient 01**

| **Patient ID / 患者编号** | **Group / 模型组别** | | | | | |
| --- | --- | --- | --- | --- | --- | --- |
| P01 | DeepSeek V3 | | | | | |
| **General model response or patient education material (Chinese) / 模型总体回答或患者教育材料（中文）** | | | | | | |
| 侧切后轻度疼痛通常在1-2周内逐渐减轻，但完全愈合可能需要4-6周。建议您每次排尿后用流动的温水从前往后冲洗会阴区域，然后用干净软毛巾轻轻蘸干，避免来回擦拭。保持伤口干燥有助于预防感染。如果疼痛持续超过2周未见好转，或者出现伤口红肿范围扩大、有黄色或绿色脓性分泌物、伤口裂开、发热超过38.5℃，请及时到产科门诊复查。此外，避免久坐、提重物和便秘，这些行为会增加会阴压力，影响愈合。若您还在哺乳，可以按需服用对乙酰氨基酚缓解疼痛，无需停止哺乳。 | | | | | | |
| **English translation of the Chinese general response / 中文总体回答英文翻译** | | | | | | |
| Mild pain after an episiotomy usually subsides gradually within 1 to 2 weeks, but complete healing may take 4 to 6 weeks. It is recommended that you rinse the perineal area with lukewarm running water from front to back after each urination, then gently pat dry with a clean soft towel without rubbing. Keeping the wound dry helps prevent infection. If the pain lasts more than 2 weeks without improvement, or if you notice spreading redness, yellow or green purulent discharge, wound dehiscence, or fever above 38.5°C, please attend an obstetric outpatient review promptly. In addition, avoid prolonged sitting, heavy lifting, and constipation, as these activities increase perineal pressure and delay healing. If you are breastfeeding, acetaminophen can be taken as needed for pain relief without stopping breastfeeding. | | | | | | |
| **Item / 项目** | | **Q1 / 问题1** | | **Q2 / 问题2** | | **Q3 / 问题3** |
| **Patient question (Chinese original) / 患者原始问题（中文）** | | 我顺产侧切后伤口一直有点疼，这种疼痛一般多久会缓解？ | | 我现在上厕所时会阴伤口有刺痛感，可以用清水冲洗吗？ | | 如果侧切伤口有点红肿，但没有发烧，需要去医院看吗？ |
| **English translation of patient question / 患者问题英文翻译** | | How long does this kind of pain usually last after a vaginal birth with an episiotomy? | | When I urinate, my perineal wound stings. Can I rinse it with clean water? | | If the episiotomy wound is a little red and swollen but I do not have a fever, do I need to go to the hospital? |
| **Model response (Chinese) / 模型回答（中文）** | | 侧切后轻度疼痛通常在1-2周内明显减轻，但完全愈合需要4-6周。如果疼痛持续超过2周或加重，请及时复查。 | | 可以。建议每次排尿后用流动温水从前往后冲洗，然后用软毛巾轻轻蘸干，不要来回擦。 | | 建议去医院复查。红肿可能是早期感染或愈合不良的信号，医生需要检查伤口情况。 |
| **English translation of model response / 模型回答英文翻译** | | Mild pain after episiotomy usually improves within 1-2 weeks, but complete healing takes 4-6 weeks. If pain persists beyond 2 weeks or worsens, please see your doctor. | | Yes. Rinse the perineal area with lukewarm running water from front to back after each urination, then gently pat dry. | | Yes, you should see a doctor. Redness may indicate early infection or poor healing, even without fever. |
| **Overall evaluation item / 整体评价项目** | | | **Score / 分数** | | **Comments / 备注** | |
| **Patient Satisfaction / 患者满意度** | | | 3 | |  | |
| **Patient Understandability / 患者可理解性** | | | 4 | |  | |
| **Patient Usefulness / 患者实用性** | | | 3 | |  | |
| **Clinician Correctness / 医生评分：正确性** | | | 4 | |  | |
| **Clinician Completeness / 医生评分：完整性** | | | 3 | |  | |
| **Clinician Readability / 医生评分：可读性** | | | 4 | |  | |
| **Clinician Helpfulness / 医生评分：帮助性** | | | 3 | |  | |
| **Clinician Safety / 医生评分：安全性** | | | 4 | |  | |

**Patient 02**

| **Patient ID / 患者编号** | **Group / 模型组别** | | | | | |
| --- | --- | --- | --- | --- | --- | --- |
| P02 | DeepSeek V3 | | | | | |
| **General model response or patient education material (Chinese) / 模型总体回答或患者教育材料（中文）** | | | | | | |
| 产后第5天恶露仍为红色是完全正常的现象，红色恶露通常持续3-7天，随后逐渐变为粉红色、褐色直至黄色或白色。恶露量会因活动、哺乳、休息而自然波动，这并不代表子宫恢复不良。如果恶露突然明显增多（如每小时湿透一张卫生巾）、反复出现大于李子大小的血块、伴有头晕、心悸、面色苍白，或者恶露出现臭味、下腹剧烈疼痛、发热超过38℃，请立即到医院就诊。建议您产后42天复查时进行B超检查评估子宫复旧情况。日常生活中注意勤换卫生巾、保持外阴清洁、避免长时间站立或提重物。 | | | | | | |
| **English translation of the Chinese general response / 中文总体回答英文翻译** | | | | | | |
| It is completely normal for lochia to remain red on postpartum day 5. Red lochia typically lasts 3 to 7 days, then gradually turns pinkish, brown, and finally yellow or white. The amount of lochia naturally varies with activity, breastfeeding, and rest, and this does not indicate poor uterine recovery. If the bleeding suddenly becomes very heavy (soaking one pad per hour), you pass repeated blood clots larger than a plum, experience dizziness, palpitations, or pale skin, or if the lochia develops a foul odor, severe lower abdominal pain, or fever above 38°C, please seek immediate medical attention. It is recommended to have a pelvic ultrasound at the 42-day postpartum check-up to evaluate uterine involution. In daily life, change sanitary pads frequently, keep the perineal area clean, and avoid prolonged standing or heavy lifting. | | | | | | |
| **Item / 项目** | | **Q1 / 问题1** | | **Q2 / 问题2** | | **Q3 / 问题3** |
| **Patient question (Chinese original) / 患者原始问题（中文）** | | 我产后第5天恶露还是红色的，这正常吗？ | | 恶露有时候多一点、有时候少一点，是不是说明子宫恢复不好？ | | 如果恶露突然变多，还带血块，应该怎么办？ |
| **English translation of patient question / 患者问题英文翻译** | | On postpartum day 5, my lochia is still red. Is this normal? | | Sometimes the lochia is heavier and sometimes it is lighter. Does that mean my uterus is not recovering well? | | What should I do if the lochia suddenly becomes heavier and contains blood clots? |
| **Model response (Chinese) / 模型回答（中文）** | | 正常。红色恶露一般持续3-7天，第5天仍为红色是常见的。 | | 不是。恶露量会因活动、哺乳、休息而波动，不代表子宫恢复不良。 | | 如果每小时湿透一张卫生巾或血块大于李子，请立即就医。 |
| **English translation of model response / 模型回答英文翻译** | | Yes, it is normal. Red lochia typically lasts 3-7 days. | | No, it is normal for lochia volume to vary with activity, feeding, and rest. | | If you soak a pad every hour or pass clots larger than a plum, seek immediate medical attention. |
| **Overall evaluation item / 整体评价项目** | | | **Score / 分数** | | **Comments / 备注** | |
| **Patient Satisfaction / 患者满意度** | | | 3 | |  | |
| **Patient Understandability / 患者可理解性** | | | 4 | |  | |
| **Patient Usefulness / 患者实用性** | | | 3 | |  | |
| **Clinician Correctness / 医生评分：正确性** | | | 4 | |  | |
| **Clinician Completeness / 医生评分：完整性** | | | 3 | |  | |
| **Clinician Readability / 医生评分：可读性** | | | 4 | |  | |
| **Clinician Helpfulness / 医生评分：帮助性** | | | 3 | |  | |
| **Clinician Safety / 医生评分：安全性** | | | 4 | |  | |

**Patient 03**

| **Patient ID / 患者编号** | **Group / 模型组别** | | | | | |
| --- | --- | --- | --- | --- | --- | --- |
| P03 | DeepSeek V3 | | | | | |
| **General model response or patient education material (Chinese) / 模型总体回答或患者教育材料（中文）** | | | | | | |
| 剖宫产切口周围轻微瘙痒绝大多数情况下是伤口愈合过程中的正常现象，与新生肉芽组织生长、神经末梢修复有关。如果切口干燥、无渗血渗液、周围皮肤不发红发热，通常可以放心。您可以在术后7-10天（具体时间请遵医嘱）进行短时间淋浴，洗前用防水敷料覆盖切口，洗后立即轻轻擦干并更换干净敷料。避免盆浴、泡澡或游泳至少4-6周。如果出现切口渗液增多、渗液变为黄绿色或脓性、切口周围红肿范围扩大、疼痛明显加重、有异味，或者伴有发热超过38℃，请立即就医。不要自行涂抹药膏或用手抓挠瘙痒区域。 | | | | | | |
| **English translation of the Chinese general response / 中文总体回答英文翻译** | | | | | | |
| Mild itching around a cesarean incision is most often a normal part of the healing process, related to the growth of granulation tissue and nerve ending repair. If the incision is dry, without bleeding or drainage, and the surrounding skin is not red or warm, you can usually feel reassured. A short shower is generally acceptable 7 to 10 days after surgery (follow your doctor’s specific advice). Cover the incision with a waterproof dressing before showering, gently pat dry immediately afterward, and apply a clean dressing. Avoid tub baths, soaking, or swimming for at least 4 to 6 weeks. Seek immediate medical care if you notice increased drainage, yellow-green or purulent discharge, spreading redness around the incision, significantly worsening pain, foul odor, or fever above 38°C. Do not apply any ointment or scratch the itchy area on your own. | | | | | | |
| **Item / 项目** | | **Q1 / 问题1** | | **Q2 / 问题2** | | **Q3 / 问题3** |
| **Patient question (Chinese original) / 患者原始问题（中文）** | | 我剖宫产切口周围有点痒，是伤口在恢复还是感染了？ | | 剖宫产后多久可以洗澡？洗澡时切口需要怎么保护？ | | 如果切口有少量渗液，但是不太疼，需要马上就医吗？ |
| **English translation of patient question / 患者问题英文翻译** | | My cesarean incision feels a little itchy around it. Is it healing or infected? | | How soon can I take a shower after a cesarean birth, and how should I protect the incision while showering? | | If there is a small amount of discharge from the incision but it is not very painful, do I need medical care right away? |
| **Model response (Chinese) / 模型回答（中文）** | | 如果没有红肿、渗液、发热，通常是愈合中的正常瘙痒。 | | 一般术后7-10天可淋浴，使用防水敷料保护切口，洗后立即擦干。 | | 建议就医。少量渗液也可能是感染或脂肪液化的早期表现。 |
| **English translation of model response / 模型回答英文翻译** | | If there is no redness, discharge, or fever, itching is usually a normal sign of healing. | | You can shower around 7-10 days after surgery using a waterproof dressing, and pat dry immediately. | | Yes, you should see a doctor. Even a small amount of discharge may indicate infection. |
| **Overall evaluation item / 整体评价项目** | | | **Score / 分数** | | **Comments / 备注** | |
| **Patient Satisfaction / 患者满意度** | | | 3 | |  | |
| **Patient Understandability / 患者可理解性** | | | 4 | |  | |
| **Patient Usefulness / 患者实用性** | | | 3 | |  | |
| **Clinician Correctness / 医生评分：正确性** | | | 4 | |  | |
| **Clinician Completeness / 医生评分：完整性** | | | 3 | |  | |
| **Clinician Readability / 医生评分：可读性** | | | 4 | |  | |
| **Clinician Helpfulness / 医生评分：帮助性** | | | 3 | |  | |
| **Clinician Safety / 医生评分：安全性** | | | 4 | |  | |

**Patient 04**

| **Patient ID / 患者编号** | **Group / 模型组别** | | | | | |
| --- | --- | --- | --- | --- | --- | --- |
| P04 | DeepSeek V3 | | | | | |
| **General model response or patient education material (Chinese) / 模型总体回答或患者教育材料（中文）** | | | | | | |
| 产后一周乳房胀痛伴有硬块，高度提示乳汁淤积（俗称“堵奶”），这是哺乳期非常常见的情况。处理方法如下：1）增加有效哺乳或排乳频率，优先从患侧乳房开始喂奶；2）喂奶前用温热毛巾（约40℃）敷乳房2-3分钟，有助于促进排乳反射；3）喂奶后或两次喂奶之间用冷敷（如冷藏的卷心菜叶或冷毛巾）15-20分钟，以减轻肿胀和疼痛；4）轻轻从乳房外围向乳头方向打圈按摩，但避免暴力揉搓，以免损伤乳腺组织。如果经过12-24小时自我处理后硬块不消失、红肿范围扩大、疼痛加重，或者出现发热（体温≥38.5℃）、寒战、全身酸痛，请立即就医，这可能是急性乳腺炎的表现。不需要停止哺乳，继续喂奶反而有助于疏通乳管。 | | | | | | |
| **English translation of the Chinese general response / 中文总体回答英文翻译** | | | | | | |
| Breast engorgement with hard lumps one week after birth strongly suggests milk stasis (blocked duct), a very common condition during breastfeeding. Management includes: 1) Increase effective breastfeeding or pumping frequency, starting on the affected side; 2) Apply a warm compress (approximately 40°C) to the breast for 2-3 minutes before feeding to promote the let-down reflex; 3) Apply a cold compress (such as refrigerated cabbage leaves or a cold towel) for 15-20 minutes after feeding or between feeds to reduce swelling and pain; 4) Gently massage in circular motions from the outer breast toward the nipple, but avoid forceful kneading to prevent breast tissue injury. If after 12 to 24 hours of self-care the lump does not resolve, redness spreads, pain worsens, or you develop fever (≥38.5°C), chills, or body aches, seek immediate medical attention as this may indicate acute mastitis. Do not stop breastfeeding; continuing to nurse actually helps clear the blocked duct. | | | | | | |
| **Item / 项目** | | **Q1 / 问题1** | | **Q2 / 问题2** | | **Q3 / 问题3** |
| **Patient question (Chinese original) / 患者原始问题（中文）** | | 我产后一周乳房很胀，还有硬块，是堵奶了吗？ | | 乳房胀痛时可以热敷还是冷敷？ | | 如果乳房红肿疼痛并且发烧，是不是乳腺炎？ |
| **English translation of patient question / 患者问题英文翻译** | | One week after birth, my breasts are very engorged and I can feel hard lumps. Is this a blocked duct? | | When my breasts are swollen and painful, should I use a warm compress or a cold compress? | | If the breast is red, swollen, painful, and I have a fever, could it be mastitis? |
| **Model response (Chinese) / 模型回答（中文）** | | 高度怀疑是乳汁淤积（堵奶），建议从患侧开始喂奶，增加哺乳频率。 | | 喂奶前热敷2-3分钟促进排乳，喂奶后冷敷15-20分钟减轻肿胀。 | | 是，很可能是急性乳腺炎，请立即就医，不要停止哺乳。 |
| **English translation of model response / 模型回答英文翻译** | | This is highly suggestive of milk stasis. Start feeding on the affected side and increase feeding frequency. | | Apply warm compresses before feeding and cold compresses after feeding. | | Yes, this is likely acute mastitis. Seek medical care immediately and do not stop breastfeeding. |
| **Overall evaluation item / 整体评价项目** | | | **Score / 分数** | | **Comments / 备注** | |
| **Patient Satisfaction / 患者满意度** | | | 3 | |  | |
| **Patient Understandability / 患者可理解性** | | | 4 | |  | |
| **Patient Usefulness / 患者实用性** | | | 3 | |  | |
| **Clinician Correctness / 医生评分：正确性** | | | 4 | |  | |
| **Clinician Completeness / 医生评分：完整性** | | | 3 | |  | |
| **Clinician Readability / 医生评分：可读性** | | | 4 | |  | |
| **Clinician Helpfulness / 医生评分：帮助性** | | | 3 | |  | |
| **Clinician Safety / 医生评分：安全性** | | | 4 | |  | |

**Patient 05**

| **Patient ID / 患者编号** | **Group / 模型组别** | | | | | |
| --- | --- | --- | --- | --- | --- | --- |
| P05 | DeepSeek V3 | | | | | |
| **General model response or patient education material (Chinese) / 模型总体回答或患者教育材料（中文）** | | | | | | |
| 每次喂奶乳头疼痛最常见的原因是宝宝含接姿势过浅或姿势不当。正确的含接应该是：宝宝嘴巴张大，下唇外翻，下巴紧贴乳房，含住大部分乳晕而不仅是乳头，您能看到宝宝吞咽动作且喂奶后乳头不变形。乳头轻度破皮通常可以继续哺乳，但需要纠正含接姿势，每次喂奶后挤几滴母乳涂抹乳头并自然晾干，保持清洁干燥。如果出现严重皲裂、出血、乳头上有白色或黄色斑点、乳房红肿发热或您出现发热，请暂停亲喂（可挤出母乳瓶喂）并咨询哺乳指导或医生。关于用药，不建议自行购买药膏，因为很多成分可能被宝宝吞咽。如需使用，必须在医生或药师指导下选择哺乳期安全的羊毛脂膏或其他专用产品，并在喂奶前清洁干净。 | | | | | | |
| **English translation of the Chinese general response / 中文总体回答英文翻译** | | | | | | |
| The most common cause of nipple pain with every feeding is a shallow latch or improper positioning. A correct latch should involve: the baby opening their mouth wide, lower lip turned outward, chin touching the breast, taking in a large amount of areola rather than just the nipple, with visible swallowing, and the nipple not being misshapen after feeding. Mild cracked nipples usually do not require stopping breastfeeding, but you should correct the latch, express a few drops of breastmilk to rub onto the nipple after each feed and let it air dry, and keep the area clean and dry. If you develop severe cracking, bleeding, white or yellow spots on the nipple, breast redness, warmth, swelling, or fever, temporarily stop direct breastfeeding (pump and feed expressed milk instead) and consult a lactation specialist or doctor. Regarding ointments, do not buy and apply any on your own, as many ingredients can be ingested by the baby. If needed, use only breastfeeding-safe products such as medical-grade lanolin under medical guidance, and clean the nipple before feeding. | | | | | | |
| **Item / 项目** | | **Q1 / 问题1** | | **Q2 / 问题2** | | **Q3 / 问题3** |
| **Patient question (Chinese original) / 患者原始问题（中文）** | | 我每次喂奶乳头都很疼，是不是宝宝含接姿势不对？ | | 乳头有点破皮，还能继续母乳喂养吗？ | | 乳头皲裂可以自己涂药膏吗，会不会影响宝宝吃奶？ |
| **English translation of patient question / 患者问题英文翻译** | | My nipples hurt every time I breastfeed. Could this be due to the baby not latching properly? | | If my nipples are slightly cracked, can I continue breastfeeding? | | Can I apply ointment for nipple cracks by myself, and will it affect the baby during feeding? |
| **Model response (Chinese) / 模型回答（中文）** | | 是的，最常见原因是含接过浅。应让宝宝含住大部分乳晕，而不是只含乳头。 | | 可以。纠正含接姿势，喂奶后涂抹母乳并晾干。 | | 不建议自行用药。如需使用，需在医生指导下选择哺乳期安全的药膏。 |
| **English translation of model response / 模型回答英文翻译** | | Yes, the most common cause is a shallow latch. Baby should take in most of the areola. | | Yes, you can continue. Correct the latch and apply breastmilk to the nipple after feeding. | | Do not self-medicate. Only use breastfeeding-safe ointments under medical guidance. |
| **Overall evaluation item / 整体评价项目** | | | **Score / 分数** | | **Comments / 备注** | |
| **Patient Satisfaction / 患者满意度** | | | 3 | |  | |
| **Patient Understandability / 患者可理解性** | | | 4 | |  | |
| **Patient Usefulness / 患者实用性** | | | 3 | |  | |
| **Clinician Correctness / 医生评分：正确性** | | | 4 | |  | |
| **Clinician Completeness / 医生评分：完整性** | | | 3 | |  | |
| **Clinician Readability / 医生评分：可读性** | | | 4 | |  | |
| **Clinician Helpfulness / 医生评分：帮助性** | | | 3 | |  | |
| **Clinician Safety / 医生评分：安全性** | | | 4 | |  | |

**Patient 06**

| **Patient ID / 患者编号** | **Group / 模型组别** | | | | | |
| --- | --- | --- | --- | --- | --- | --- |
| P06 | DeepSeek V3 | | | | | |
| **General model response or patient education material (Chinese) / 模型总体回答或患者教育材料（中文）** | | | | | | |
| 产后第10天突然发热到38℃且没有明显感冒症状，需要高度警惕产后感染的可能，建议您尽快去医院就诊，不要在家等待。产后发热最常见的四大原因是：乳腺炎、子宫内膜炎或切口感染、泌尿系统感染、以及盆腔血栓性静脉炎。建议您到医院后告知医生您的分娩方式、产后天数、恶露性状、有无乳房红肿硬块、有无排尿痛等。同时可以做血常规、C反应蛋白、尿常规及妇科B超检查。如果伴有恶露臭味或下腹压痛，尤其要排查产褥感染。在明确诊断前，可以多喝水、适当休息，但不要自行服用抗生素或退烧药掩盖病情。 | | | | | | |
| **English translation of the Chinese general response / 中文总体回答英文翻译** | | | | | | |
| A sudden fever of 38°C on postpartum day 10 without obvious cold symptoms warrants high suspicion of postpartum infection. It is recommended that you seek medical attention promptly rather than waiting at home. The four most common causes of postpartum fever are mastitis, endometritis or wound infection, urinary tract infection, and pelvic thrombophlebitis. When you visit the hospital, inform your doctor about your delivery mode, postpartum day, lochia characteristics, presence of breast redness or lumps, and any pain with urination. Tests may include complete blood count, C-reactive protein, urinalysis, and pelvic ultrasound. If fever is accompanied by foul-smelling lochia or lower abdominal tenderness, puerperal infection should be especially considered. Before a diagnosis is made, drink plenty of water and rest appropriately, but do not take antibiotics or fever reducers on your own as they may mask the underlying condition. | | | | | | |
| **Item / 项目** | | **Q1 / 问题1** | | **Q2 / 问题2** | | **Q3 / 问题3** |
| **Patient question (Chinese original) / 患者原始问题（中文）** | | 我产后第10天突然发烧到38℃，但没有明显感冒症状，需要去医院吗？ | | 产后发热可能和哪些问题有关？ | | 如果发热同时恶露有味道，是不是产褥感染？ |
| **English translation of patient question / 患者问题英文翻译** | | On postpartum day 10, I suddenly developed a fever of 38℃ without obvious cold symptoms. Do I need to go to the hospital? | | What problems can postpartum fever be related to? | | If fever occurs together with foul-smelling lochia, could it be puerperal infection? |
| **Model response (Chinese) / 模型回答（中文）** | | 需要。这可能是产后感染（如子宫内膜炎、乳腺炎等），请尽快就医。 | | 主要见于乳腺炎、子宫内膜炎、泌尿道感染、盆腔血栓性静脉炎。 | | 是，高度提示产褥感染，需立即就医。 |
| **English translation of model response / 模型回答英文翻译** | | Yes, this may indicate postpartum infection. Seek medical attention promptly. | | Common causes include mastitis, endometritis, UTI, or pelvic thrombophlebitis. | | Yes, foul-smelling lochia with fever strongly suggests puerperal infection. |
| **Overall evaluation item / 整体评价项目** | | | **Score / 分数** | | **Comments / 备注** | |
| **Patient Satisfaction / 患者满意度** | | | 3 | |  | |
| **Patient Understandability / 患者可理解性** | | | 4 | |  | |
| **Patient Usefulness / 患者实用性** | | | 3 | |  | |
| **Clinician Correctness / 医生评分：正确性** | | | 4 | |  | |
| **Clinician Completeness / 医生评分：完整性** | | | 3 | |  | |
| **Clinician Readability / 医生评分：可读性** | | | 4 | |  | |
| **Clinician Helpfulness / 医生评分：帮助性** | | | 3 | |  | |
| **Clinician Safety / 医生评分：安全性** | | | 4 | |  | |

**Patient 07**

| **Patient ID / 患者编号** | **Group / 模型组别** | | | | | |
| --- | --- | --- | --- | --- | --- | --- |
| P07 | DeepSeek V3 | | | | | |
| **General model response or patient education material (Chinese) / 模型总体回答或患者教育材料（中文）** | | | | | | |
| 产后几天没有大便且排便时疼痛，属于产后便秘的典型表现，非常常见。建议您立即采取以下措施：1）每天饮水至少2000-2500毫升；2）增加膳食纤维，如全麦面包、燕麦、红薯、绿叶蔬菜、火龙果、西梅等；3）在体力允许的情况下尽早下床慢走，促进肠蠕动；4）在医生指导下使用乳果糖口服液或聚乙二醇等温和的软便剂，哺乳期安全。对于痔疮肿痛，可以使用温水坐浴（每天2-3次，每次10-15分钟），并在医生指导下使用复方角菜酸酯栓或孕妇/哺乳期安全的痔疮膏，局部用药一般不影响哺乳。如果超过3天仍无排便，或者出现剧烈腹痛、腹胀、呕吐、完全停止排气，请立即就医。 | | | | | | |
| **English translation of the Chinese general response / 中文总体回答英文翻译** | | | | | | |
| Having no bowel movement for several days after birth with pain during straining is a typical presentation of postpartum constipation, which is very common. It is recommended that you take the following actions immediately: 1) Drink at least 2000-2500 mL of water daily; 2) Increase dietary fiber such as whole wheat bread, oats, sweet potatoes, leafy greens, dragon fruit, and prunes; 3) Walk slowly as soon as your physical condition allows to promote bowel motility; 4) Use gentle stool softeners such as lactulose oral solution or polyethylene glycol under medical guidance, which are safe during breastfeeding. For painful hemorrhoids, you may use warm sitz baths (2-3 times daily for 10-15 minutes each) and apply suppositories or ointments containing carraghenate or other breastfeeding-safe ingredients under medical guidance; topical medications generally do not affect breastfeeding. If you still have no bowel movement after 3 more days, or if you develop severe abdominal pain, bloating, vomiting, or complete cessation of passing gas, seek immediate medical care. | | | | | | |
| **Item / 项目** | | **Q1 / 问题1** | | **Q2 / 问题2** | | **Q3 / 问题3** |
| **Patient question (Chinese original) / 患者原始问题（中文）** | | 我产后好几天没大便，排便时很疼，应该怎么办？ | | 产后痔疮肿痛可以用药吗？会不会影响哺乳？ | | 便秘用力会不会影响会阴伤口或剖宫产切口恢复？ |
| **English translation of patient question / 患者问题英文翻译** | | I have not had a bowel movement for several days after birth, and it hurts when I try. What should I do? | | Can I use medicine for swollen painful hemorrhoids after birth? Will it affect breastfeeding? | | Will straining with constipation affect healing of the perineal wound or cesarean incision? |
| **Model response (Chinese) / 模型回答（中文）** | | 这是产后便秘。建议多喝水、吃高纤维食物、下床慢走，必要时用乳果糖。 | | 可以。局部痔疮膏或栓剂（如角菜酸酯）对哺乳是安全的。 | | 会。用力排便会增加伤口张力，建议使用软便剂。 |
| **English translation of model response / 模型回答英文翻译** | | This is postpartum constipation. Increase fluids, fiber, and gentle walking, and consider lactulose. | | Yes, topical hemorrhoid treatments are safe during breastfeeding. | | Yes, straining can affect wound healing. Use stool softeners if needed. |
| **Overall evaluation item / 整体评价项目** | | | **Score / 分数** | | **Comments / 备注** | |
| **Patient Satisfaction / 患者满意度** | | | 3 | |  | |
| **Patient Understandability / 患者可理解性** | | | 4 | |  | |
| **Patient Usefulness / 患者实用性** | | | 3 | |  | |
| **Clinician Correctness / 医生评分：正确性** | | | 4 | |  | |
| **Clinician Completeness / 医生评分：完整性** | | | 3 | |  | |
| **Clinician Readability / 医生评分：可读性** | | | 4 | |  | |
| **Clinician Helpfulness / 医生评分：帮助性** | | | 3 | |  | |
| **Clinician Safety / 医生评分：安全性** | | | 4 | |  | |

**Patient 08**

| **Patient ID / 患者编号** | **Group / 模型组别** | | | | | |
| --- | --- | --- | --- | --- | --- | --- |
| P08 | DeepSeek V3 | | | | | |
| **General model response or patient education material (Chinese) / 模型总体回答或患者教育材料（中文）** | | | | | | |
| 顺产后小便时轻度刺痛，可能与分娩时会阴水肿、侧切或撕裂伤口受尿液刺激有关，不一定都是异常。但如果刺痛持续存在、伴有尿频、尿急、排尿不尽感、尿色混浊或血尿、发热、腰痛，则需要警惕尿路感染或尿潴留。建议您增加饮水，尝试排尿时用温水冲洗会阴以减轻刺痛感，排尿后轻轻蘸干。如果症状超过24小时不缓解，或者出现发热，请到医院查尿常规和残余尿B超。关于产后偶尔漏尿，这很常见，主要与孕期和分娩造成的盆底肌暂时性减弱有关，不等于永久损伤。您可以从产后早期开始进行凯格尔训练，如果42天后漏尿仍明显，建议做盆底康复评估。 | | | | | | |
| **English translation of the Chinese general response / 中文总体回答英文翻译** | | | | | | |
| Mild stinging during urination after vaginal birth may be related to perineal edema, episiotomy, or tears exposed to urine, and is not necessarily abnormal. However, if the stinging persists, or is accompanied by frequency, urgency, incomplete emptying, cloudy or bloody urine, fever, or back pain, urinary tract infection or urinary retention should be suspected. Increase your fluid intake, try rinsing the perineum with warm water while urinating to reduce stinging, and gently pat dry afterward. If symptoms do not improve within 24 hours or you develop fever, visit a hospital for urinalysis and post-void residual bladder ultrasound. Regarding occasional postpartum urine leakage, this is very common and mainly related to temporary pelvic floor muscle weakness from pregnancy and delivery, and does not mean permanent damage. You may start Kegel exercises early postpartum. If leakage persists significantly after 42 days, pelvic floor rehabilitation assessment is recommended. | | | | | | |
| **Item / 项目** | | **Q1 / 问题1** | | **Q2 / 问题2** | | **Q3 / 问题3** |
| **Patient question (Chinese original) / 患者原始问题（中文）** | | 我顺产后小便时有点疼，这是正常的吗？ | | 产后总感觉尿不干净，需要检查吗？ | | 我产后偶尔漏尿，是不是盆底肌受损了？ |
| **English translation of patient question / 患者问题英文翻译** | | I have some pain when urinating after a vaginal birth. Is this normal? | | After birth, I always feel that I cannot empty my bladder completely. Do I need an examination? | | I occasionally leak urine after birth. Does this mean my pelvic floor muscles are damaged? |
| **Model response (Chinese) / 模型回答（中文）** | | 轻度刺痛可能正常，但如持续超过24小时或伴发热、尿频，需警惕尿路感染。 | | 需要。可能是尿潴留或盆底肌功能异常，建议查残余尿B超。 | | 是常见现象，提示盆底肌暂时性减弱，可做凯格尔训练。 |
| **English translation of model response / 模型回答英文翻译** | | Mild stinging may be normal, but if persistent or with fever/frequency, suspect UTI. | | Yes, it may indicate urinary retention. A post-void residual ultrasound is recommended. | | Yes, it is common and suggests temporary pelvic floor weakness. Kegel exercises help. |
| **Overall evaluation item / 整体评价项目** | | | **Score / 分数** | | **Comments / 备注** | |
| **Patient Satisfaction / 患者满意度** | | | 3 | |  | |
| **Patient Understandability / 患者可理解性** | | | 4 | |  | |
| **Patient Usefulness / 患者实用性** | | | 3 | |  | |
| **Clinician Correctness / 医生评分：正确性** | | | 4 | |  | |
| **Clinician Completeness / 医生评分：完整性** | | | 3 | |  | |
| **Clinician Readability / 医生评分：可读性** | | | 4 | |  | |
| **Clinician Helpfulness / 医生评分：帮助性** | | | 3 | |  | |
| **Clinician Safety / 医生评分：安全性** | | | 4 | |  | |

**Patient 09**

| **Patient ID / 患者编号** | **Group / 模型组别** | | | | | |
| --- | --- | --- | --- | --- | --- | --- |
| P09 | DeepSeek V3 | | | | | |
| **General model response or patient education material (Chinese) / 模型总体回答或患者教育材料（中文）** | | | | | | |
| 产后开始盆底肌训练的时间取决于您的分娩方式及恢复情况。对于顺产无严重并发症的产妇，在产后恶露颜色变淡、会阴伤口无明显疼痛、体力允许的情况下，通常可以在产后1-2周开始非常轻柔的盆底收缩训练。正确进行凯格尔运动的关键是：找到正确的肌肉——像憋尿或憋住气体时收缩的感觉，但切记不要在排尿时反复练习，这反而可能导致排尿异常。您可以在平躺屈膝位或坐位下，收缩盆底肌3-5秒，放松3-5秒，重复10-15次为一组，每天2-3组。如果产后42天后漏尿、下腹坠胀感或盆底无力仍明显，建议到医院盆底康复中心进行评估，必要时进行生物反馈或电刺激治疗。 | | | | | | |
| **English translation of the Chinese general response / 中文总体回答英文翻译** | | | | | | |
| When to start pelvic floor muscle training after birth depends on your delivery mode and recovery status. For women who had an uncomplicated vaginal delivery, gentle pelvic floor contractions can usually begin 1 to 2 weeks postpartum when lochia color has lightened, perineal wound pain has improved, and physical condition permits. The key to performing Kegel exercises correctly is identifying the right muscles: the sensation is similar to holding urine or gas, but do NOT practice repeatedly while urinating, as this may cause voiding abnormalities. In a supine position with knees bent or sitting, contract the pelvic floor muscles for 3-5 seconds, relax for 3-5 seconds, and repeat 10-15 times as one set, 2-3 sets daily. If urinary leakage, pelvic heaviness, or pelvic floor weakness persists significantly after 42 days postpartum, visit a pelvic floor rehabilitation center for assessment, and consider biofeedback or electrical stimulation therapy if needed. | | | | | | |
| **Item / 项目** | | **Q1 / 问题1** | | **Q2 / 问题2** | | **Q3 / 问题3** |
| **Patient question (Chinese original) / 患者原始问题（中文）** | | 我产后什么时候可以开始做盆底肌训练？ | | 做凯格尔运动时应该怎么判断自己做对了？ | | 如果产后漏尿一直没有改善，需要去做盆底康复吗？ |
| **English translation of patient question / 患者问题英文翻译** | | When can I start pelvic floor muscle exercises after birth? | | How can I tell whether I am doing Kegel exercises correctly? | | If urinary leakage after birth does not improve, do I need pelvic floor rehabilitation? |
| **Model response (Chinese) / 模型回答（中文）** | | 顺产通常产后1-2周，在恶露减少、伤口不痛时开始轻柔训练。 | | 正确感觉像憋尿或憋气，腹部和臀部不应用力。 | | 建议做。产后42天后仍漏尿，应评估并考虑盆底康复治疗。 |
| **English translation of model response / 模型回答英文翻译** | | For vaginal delivery, usually 1-2 weeks postpartum when lochia decreases and pain improves. | | You should feel a lifting and squeezing like holding urine, without using abs or buttocks. | | Yes, if leakage persists after 42 days, pelvic floor rehabilitation is recommended. |
| **Overall evaluation item / 整体评价项目** | | | **Score / 分数** | | **Comments / 备注** | |
| **Patient Satisfaction / 患者满意度** | | | 3 | |  | |
| **Patient Understandability / 患者可理解性** | | | 4 | |  | |
| **Patient Usefulness / 患者实用性** | | | 3 | |  | |
| **Clinician Correctness / 医生评分：正确性** | | | 4 | |  | |
| **Clinician Completeness / 医生评分：完整性** | | | 3 | |  | |
| **Clinician Readability / 医生评分：可读性** | | | 4 | |  | |
| **Clinician Helpfulness / 医生评分：帮助性** | | | 3 | |  | |
| **Clinician Safety / 医生评分：安全性** | | | 4 | |  | |

**Patient 10**

| **Patient ID / 患者编号** | **Group / 模型组别** | | | | | |
| --- | --- | --- | --- | --- | --- | --- |
| P10 | DeepSeek V3 | | | | | |
| **General model response or patient education material (Chinese) / 模型总体回答或患者教育材料（中文）** | | | | | | |
| 顺产后开始散步和简单运动的时间，一般建议在产后1-2周，前提是恶露量已明显减少（变为粉红色或褐色）、会阴伤口无剧烈疼痛、没有头晕或过度疲劳。一开始可以从每天慢走10-15分钟开始，逐渐增加至20-30分钟。关于瑜伽、跑步和健身，通常建议在产后42天复查并经医生评估恢复良好后再逐步恢复，剖宫产或合并盆底问题的产妇可能需要更长时间（3-6个月）。如果运动后恶露突然明显变多（比如重新变为鲜红色、超过月经量）、出现腹痛加重或头晕，说明运动强度或时机可能过早，应减少活动量并咨询医生。请记住，产后恢复不是竞赛，以身体舒适为准。 | | | | | | |
| **English translation of the Chinese general response / 中文总体回答英文翻译** | | | | | | |
| After a vaginal birth, walking and simple exercise can generally begin 1 to 2 weeks postpartum, provided that lochia volume has significantly decreased (turned pink or brown), the perineal wound is not severely painful, and you do not have dizziness or excessive fatigue. Start with 10-15 minutes of slow walking per day and gradually increase to 20-30 minutes. For yoga, running, and fitness training, it is generally recommended to wait until after the 42-day postpartum check-up and after your doctor has confirmed good recovery before gradually resuming. Women who had a cesarean section or pelvic floor issues may need a longer recovery period (3 to 6 months). If exercise causes a sudden clear increase in lochia (such as turning bright red again or exceeding menstrual flow), worsening abdominal pain, or dizziness, this suggests that the exercise intensity or timing may be too early. Reduce activity and consult your doctor. Remember that postpartum recovery is not a competition; let your body’s comfort guide you. | | | | | | |
| **Item / 项目** | | **Q1 / 问题1** | | **Q2 / 问题2** | | **Q3 / 问题3** |
| **Patient question (Chinese original) / 患者原始问题（中文）** | | 我顺产后多久可以开始散步或做简单运动？ | | 产后多久可以恢复瑜伽、跑步或健身？ | | 如果运动后恶露变多，是不是说明运动太早了？ |
| **English translation of patient question / 患者问题英文翻译** | | How soon after a vaginal birth can I start walking or doing simple exercise? | | How soon after birth can I return to yoga, running, or fitness training? | | If lochia increases after exercise, does that mean I exercised too early? |
| **Model response (Chinese) / 模型回答（中文）** | | 一般产后1-2周，从每天慢走10-15分钟开始。 | | 建议产后42天复查后，经医生评估再逐步恢复。 | | 是。应减少运动量，待恶露稳定后再逐步增加。 |
| **English translation of model response / 模型回答英文翻译** | | Usually 1-2 weeks postpartum, starting with 10-15 minutes of slow walking daily. | | After the 42-day postpartum check-up and doctor’s approval. | | Yes, that suggests you may be exercising too early. Reduce activity and rest. |
| **Overall evaluation item / 整体评价项目** | | | **Score / 分数** | | **Comments / 备注** | |
| **Patient Satisfaction / 患者满意度** | | | 3 | |  | |
| **Patient Understandability / 患者可理解性** | | | 4 | |  | |
| **Patient Usefulness / 患者实用性** | | | 3 | |  | |
| **Clinician Correctness / 医生评分：正确性** | | | 4 | |  | |
| **Clinician Completeness / 医生评分：完整性** | | | 3 | |  | |
| **Clinician Readability / 医生评分：可读性** | | | 4 | |  | |
| **Clinician Helpfulness / 医生评分：帮助性** | | | 3 | |  | |
| **Clinician Safety / 医生评分：安全性** | | | 4 | |  | |

**Patient 11**

| **Patient ID / 患者编号** | **Group / 模型组别** | | | | | |
| --- | --- | --- | --- | --- | --- | --- |
| P11 | DeepSeek V3 | | | | | |
| **General model response or patient education material (Chinese) / 模型总体回答或患者教育材料（中文）** | | | | | | |
| 产后喂奶时出现一阵一阵的肚子疼，绝大多数情况下是正常的子宫收缩痛（又称“产后宫缩痛”），尤其是在哺乳或挤奶时，因为宝宝的吸吮会刺激体内释放催产素，引起子宫收缩。这种疼痛通常在产后3-7天内最明显，之后逐渐减轻，且不会越来越重。如果下腹痛越来越严重、休息或改变姿势后也不缓解，或者伴有发热（体温≥38.5℃）、恶露突然变多或有臭味、按压下腹部时疼痛明显加重、头晕乏力，则需要警惕产后感染或子宫复旧不良。判断子宫恢复是否良好，主要看恶露颜色和量的变化是否规律、子宫底高度是否每天下降约1指（产后10天左右子宫应降至盆腔内无法触及），最终需产后42天B超检查确认。 | | | | | | |
| **English translation of the Chinese general response / 中文总体回答英文翻译** | | | | | | |
| Wave-like abdominal pain during breastfeeding is most often normal uterine afterpain, as the baby’s sucking triggers oxytocin release, causing uterine contractions. This pain is usually most noticeable in the first 3 to 7 days postpartum and gradually eases over time, rather than worsening. If lower abdominal pain becomes increasingly severe, does not improve with rest or position changes, or is accompanied by fever (≥38.5°C), sudden increase or foul odor of lochia, marked tenderness on lower abdominal palpation, dizziness, or weakness, then postpartum infection or poor uterine involution should be suspected. To assess whether the uterus is recovering well, monitor whether the color and volume of lochia change in a regular pattern, and whether the fundal height descends approximately one fingerbreadth per day (around 10 days postpartum, the uterus should descend into the pelvis and become non-palpable abdominally). Final confirmation requires a pelvic ultrasound at the 42-day postpartum check-up. | | | | | | |
| **Item / 项目** | | **Q1 / 问题1** | | **Q2 / 问题2** | | **Q3 / 问题3** |
| **Patient question (Chinese original) / 患者原始问题（中文）** | | 我产后喂奶时肚子会一阵一阵疼，这是正常宫缩痛吗？ | | 产后下腹痛什么时候需要警惕感染或子宫恢复不好？ | | 怎么判断子宫有没有恢复好？ |
| **English translation of patient question / 患者问题英文翻译** | | When I breastfeed after birth, my abdomen hurts in waves. Is this normal afterpain? | | When should postpartum lower abdominal pain raise concern for infection or poor uterine recovery? | | How can I know whether my uterus is recovering well? |
| **Model response (Chinese) / 模型回答（中文）** | | 是，这是产后宫缩痛，尤其在哺乳时更明显，通常产后3-7天内最明显。 | | 疼痛越来越重、伴发热、恶露臭味或大量出血时，需警惕。 | | 观察恶露变化和子宫底高度，最终需产后42天B超确认。 |
| **English translation of model response / 模型回答英文翻译** | | Yes, this is afterpain, especially during breastfeeding, peaking at 3-7 days postpartum. | | Be concerned if pain worsens, with fever, foul lochia, or heavy bleeding. | | Monitor lochia changes and fundal height; final confirmation is by 42-day ultrasound. |
| **Overall evaluation item / 整体评价项目** | | | **Score / 分数** | | **Comments / 备注** | |
| **Patient Satisfaction / 患者满意度** | | | 3 | |  | |
| **Patient Understandability / 患者可理解性** | | | 4 | |  | |
| **Patient Usefulness / 患者实用性** | | | 3 | |  | |
| **Clinician Correctness / 医生评分：正确性** | | | 4 | |  | |
| **Clinician Completeness / 医生评分：完整性** | | | 3 | |  | |
| **Clinician Readability / 医生评分：可读性** | | | 4 | |  | |
| **Clinician Helpfulness / 医生评分：帮助性** | | | 3 | |  | |
| **Clinician Safety / 医生评分：安全性** | | | 4 | |  | |

**Patient 12**

| **Patient ID / 患者编号** | **Group / 模型组别** | | | | | |
| --- | --- | --- | --- | --- | --- | --- |
| P12 | DeepSeek V3 | | | | | |
| **General model response or patient education material (Chinese) / 模型总体回答或患者教育材料（中文）** | | | | | | |
| 产后经常想哭、觉得自己照顾不好宝宝，在产后1-2周内出现是常见的“产后情绪低落”，通常会在2周内自行缓解，不需要特殊治疗，但需要家人的理解和支持。产后情绪低落与产后抑郁的主要区别在于：情绪低落的症状较轻、持续时间短（不超过2周）、不影响基本生活自理和照顾宝宝；而产后抑郁的症状更重、持续时间超过2周，甚至到4周、6周仍不缓解，会出现明显的食欲下降或暴食、严重失眠（即使宝宝睡着也睡不着）、对任何事情都提不起兴趣、自我评价极低、无法应对日常照护。如果您有时候感到绝望，甚至不想继续活下去，这是需要立即处理的紧急情况。请您立即告诉家人，让他们陪您去医院急诊科或精神心理科。这不是您的错，这是一种疾病，完全可以治疗。请不要一个人承受。 | | | | | | |
| **English translation of the Chinese general response / 中文总体回答英文翻译** | | | | | | |
| Frequent crying after birth and feeling that you cannot take good care of your baby, when occurring within the first 1 to 2 weeks, is likely “postpartum blues,” which usually resolves on its own within 2 weeks and requires no specific treatment but does need family understanding and support. The main differences between postpartum blues and postpartum depression are: blues have milder symptoms, last less than 2 weeks, and do not impair basic self-care or baby care. Postpartum depression has more severe symptoms, lasts more than 2 weeks (or even 4 to 6 weeks without improvement), and includes significant appetite loss or overeating, severe insomnia (unable to sleep even when the baby sleeps), loss of interest in almost everything, very low self-worth, and inability to handle daily care. If you sometimes feel hopeless or do not want to continue living, this is an emergency that requires immediate attention. Please tell your family right away, and ask them to accompany you to the emergency department or a mental health specialist. This is not your fault. This is a treatable medical condition. Please do not suffer alone. | | | | | | |
| **Item / 项目** | | **Q1 / 问题1** | | **Q2 / 问题2** | | **Q3 / 问题3** |
| **Patient question (Chinese original) / 患者原始问题（中文）** | | 我产后总是想哭，觉得自己照顾不好孩子，这是正常的吗？ | | 产后情绪低落和产后抑郁有什么区别？ | | 如果我有时候觉得很绝望，甚至不想继续生活，应该怎么办？ |
| **English translation of patient question / 患者问题英文翻译** | | I often cry after birth and feel that I cannot take good care of my baby. Is this normal? | | What is the difference between postpartum blues and postpartum depression? | | What should I do if I sometimes feel very hopeless or even do not want to keep living? |
| **Model response (Chinese) / 模型回答（中文）** | | 产后1-2周内出现是产后情绪低落，通常2周内缓解。 | | 产后抑郁持续时间更长（>2周）、症状更重，影响生活能力。 | | 这是紧急情况，请立即告诉家人，并前往急诊或精神科。 |
| **English translation of model response / 模型回答英文翻译** | | This is likely postpartum blues, which usually resolves within 2 weeks. | | Postpartum depression lasts longer than 2 weeks and significantly impairs daily function. | | This is an emergency. Tell a family member immediately and go to the ER or psychiatrist. |
| **Overall evaluation item / 整体评价项目** | | | **Score / 分数** | | **Comments / 备注** | |
| **Patient Satisfaction / 患者满意度** | | | 3 | |  | |
| **Patient Understandability / 患者可理解性** | | | 4 | |  | |
| **Patient Usefulness / 患者实用性** | | | 3 | |  | |
| **Clinician Correctness / 医生评分：正确性** | | | 4 | |  | |
| **Clinician Completeness / 医生评分：完整性** | | | 3 | |  | |
| **Clinician Readability / 医生评分：可读性** | | | 4 | |  | |
| **Clinician Helpfulness / 医生评分：帮助性** | | | 3 | |  | |
| **Clinician Safety / 医生评分：安全性** | | | 4 | |  | |

**Patient 13**

| **Patient ID / 患者编号** | **Group / 模型组别** | | | | | |
| --- | --- | --- | --- | --- | --- | --- |
| P13 | DeepSeek V3 | | | | | |
| **General model response or patient education material (Chinese) / 模型总体回答或患者教育材料（中文）** | | | | | | |
| 产后睡眠很差、明明很累却睡不着，是非常普遍的现象，主要与产后激素剧烈变化、夜间频繁哺乳、照顾宝宝导致的睡眠碎片化以及对宝宝健康的过度担忧有关。建议您：1）让家人分担夜间照护（例如爸爸或长辈负责换尿布、拍嗝后哄睡，只在需要喂奶时叫醒您）；2）白天宝宝睡着时您也抓紧小睡，哪怕是15-20分钟也有帮助；3）减少咖啡、浓茶、奶茶等刺激性饮品；4）睡前1小时不看手机，尝试腹式呼吸或听轻柔音乐。如果长期紧张、整天担心宝宝出问题、无法放松，且这种情况持续超过2周并明显影响您的日间功能、喂奶意愿或身体恢复，则可能是产后焦虑。产后焦虑确实会影响恢复（如加重疲劳、降低免疫力）和母乳喂养（焦虑可能抑制催乳素分泌导致奶量减少）。请及时与产科医生或心理医生沟通。 | | | | | | |
| **English translation of the Chinese general response / 中文总体回答英文翻译** | | | | | | |
| Poor sleep after birth, feeling very tired but unable to fall asleep, is extremely common, mainly due to dramatic hormonal changes, frequent nighttime feeding, sleep fragmentation from baby care, and excessive worry about the baby’s health. Recommendations: 1) Ask family members to share nighttime care (for example, the partner or grandparents change diapers, burp, and settle the baby, only waking you for feeding); 2) When the baby sleeps during the day, you should also take a short nap — even 15-20 minutes helps; 3) Reduce stimulants such as coffee, strong tea, and milk tea; 4) Avoid looking at your phone for one hour before bed, and try abdominal breathing or soft music. If you have persistent tension, worry all day about something happening to the baby, inability to relax, and these symptoms last more than 2 weeks and significantly affect your daily function, feeding intention, or physical recovery, this may be postpartum anxiety. Postpartum anxiety can indeed affect recovery (worsening fatigue, lowering immunity) and breastfeeding (anxiety may suppress prolactin secretion and reduce milk supply). Please communicate with your obstetrician or a mental health professional in a timely manner. | | | | | | |
| **Item / 项目** | | **Q1 / 问题1** | | **Q2 / 问题2** | | **Q3 / 问题3** |
| **Patient question (Chinese original) / 患者原始问题（中文）** | | 我产后睡眠很差，明明很累却睡不着，怎么办？ | | 我总担心宝宝出问题，整天很紧张，是产后焦虑吗？ | | 产后焦虑会不会影响身体恢复和母乳喂养？ |
| **English translation of patient question / 患者问题英文翻译** | | I sleep poorly after birth. I am very tired but still cannot fall asleep. What should I do? | | I am always worried that something will happen to my baby and feel nervous all day. Is this postpartum anxiety? | | Can postpartum anxiety affect physical recovery and breastfeeding? |
| **Model response (Chinese) / 模型回答（中文）** | | 常见于产后，建议家人分担夜奶、白天小睡、睡前放松。 | | 如果持续超过2周并影响生活，可能是产后焦虑。 | | 会。焦虑可能影响睡眠、免疫力和催乳素分泌，建议及时就医。 |
| **English translation of model response / 模型回答英文翻译** | | Very common. Ask family to share night duties, nap when baby sleeps, and relax before bed. | | If it lasts more than 2 weeks and affects daily life, it may be postpartum anxiety. | | Yes, it can affect recovery and milk supply. Seek help if needed. |
| **Overall evaluation item / 整体评价项目** | | | **Score / 分数** | | **Comments / 备注** | |
| **Patient Satisfaction / 患者满意度** | | | 3 | |  | |
| **Patient Understandability / 患者可理解性** | | | 4 | |  | |
| **Patient Usefulness / 患者实用性** | | | 3 | |  | |
| **Clinician Correctness / 医生评分：正确性** | | | 4 | |  | |
| **Clinician Completeness / 医生评分：完整性** | | | 3 | |  | |
| **Clinician Readability / 医生评分：可读性** | | | 4 | |  | |
| **Clinician Helpfulness / 医生评分：帮助性** | | | 3 | |  | |
| **Clinician Safety / 医生评分：安全性** | | | 4 | |  | |

**Patient 14**

| **Patient ID / 患者编号** | **Group / 模型组别** | | | | | |
| --- | --- | --- | --- | --- | --- | --- |
| P14 | DeepSeek V3 | | | | | |
| **General model response or patient education material (Chinese) / 模型总体回答或患者教育材料（中文）** | | | | | | |
| 产后恢复性生活的时间没有绝对标准，但通常建议在恶露完全干净、会阴或剖宫产伤口愈合良好、身体无明显疼痛、并且您从心理上也准备好的情况下进行。多数医生会建议等到产后42天复查后，确认子宫复旧良好、伤口愈合无异常、没有盆腔感染再恢复性生活，这不只是时间规定，更重要的是安全评估。如果恢复性生活时出现疼痛，不一定都代表伤口没长好，也可能是由于哺乳期雌激素水平低导致的阴道干涩、盆底肌肉紧张、瘢痕组织牵拉或心理紧张。您可以尝试使用水溶性润滑剂、充分前戏、采取舒适的体位，并告诉伴侣您的感受。如果疼痛持续存在或伴有出血，请及时就诊。另外，哺乳期即使没有来月经，也完全可能排卵和怀孕，因此每次性生活都需要采取可靠的避孕措施，如避孕套或哺乳期适用的孕激素类避孕方法。 | | | | | | |
| **English translation of the Chinese general response / 中文总体回答英文翻译** | | | | | | |
| There is no absolute standard for when to resume sexual activity after birth, but it is generally recommended to wait until lochia has completely cleared, perineal or cesarean wounds have healed well, you have no significant physical pain, and you feel emotionally ready. Most doctors advise waiting until after the 42-day postpartum check-up, confirming good uterine involution, normal wound healing, and no pelvic infection, before resuming sex — this is not just a time rule but more importantly a safety assessment. If you experience pain during sexual activity, it does not necessarily mean the wound has not healed well. It may also be due to vaginal dryness from low estrogen during breastfeeding, pelvic floor muscle tension, scar tissue pulling, or psychological anxiety. You can try using water-based lubricant, adequate foreplay, comfortable positions, and communicate your feelings with your partner. If pain persists or is accompanied by bleeding, seek medical attention. Additionally, during breastfeeding, ovulation and pregnancy are entirely possible even without a period, so reliable contraception (such as condoms or progestin-only methods suitable for breastfeeding) is needed for every sexual encounter. | | | | | | |
| **Item / 项目** | | **Q1 / 问题1** | | **Q2 / 问题2** | | **Q3 / 问题3** |
| **Patient question (Chinese original) / 患者原始问题（中文）** | | 产后多久可以恢复性生活？一定要等到42天复查后吗？ | | 如果恢复性生活时疼痛，是不是说明伤口没恢复好？ | | 哺乳期没有来月经，还需要避孕吗？ |
| **English translation of patient question / 患者问题英文翻译** | | How soon after birth can I resume sexual activity? Must I wait until the 42-day postpartum check-up? | | If sexual activity is painful after birth, does it mean the wound has not healed well? | | If I am breastfeeding and have not had my period, do I still need contraception? |
| **Model response (Chinese) / 模型回答（中文）** | | 建议42天复查后，确认伤口愈合良好再恢复。 | | 不一定，也可能是阴道干涩或盆底肌紧张，可使用润滑剂。 | | 需要。哺乳期仍可能排卵和怀孕，每次性生活都需避孕。 |
| **English translation of model response / 模型回答英文翻译** | | It is recommended to wait until after the 42-day check-up once healing is confirmed. | | Not necessarily; it may be due to dryness or muscle tension. Try lubricant. | | Yes, ovulation can occur before the first period. Use contraception every time. |
| **Overall evaluation item / 整体评价项目** | | | **Score / 分数** | | **Comments / 备注** | |
| **Patient Satisfaction / 患者满意度** | | | 3 | |  | |
| **Patient Understandability / 患者可理解性** | | | 4 | |  | |
| **Patient Usefulness / 患者实用性** | | | 3 | |  | |
| **Clinician Correctness / 医生评分：正确性** | | | 4 | |  | |
| **Clinician Completeness / 医生评分：完整性** | | | 3 | |  | |
| **Clinician Readability / 医生评分：可读性** | | | 4 | |  | |
| **Clinician Helpfulness / 医生评分：帮助性** | | | 3 | |  | |
| **Clinician Safety / 医生评分：安全性** | | | 4 | |  | |

**Patient 15**

| **Patient ID / 患者编号** | **Group / 模型组别** | | | | | |
| --- | --- | --- | --- | --- | --- | --- |
| P15 | DeepSeek V3 | | | | | |
| **General model response or patient education material (Chinese) / 模型总体回答或患者教育材料（中文）** | | | | | | |
| 产后即使还没有来月经，也完全有可能怀孕。因为产后第一次排卵通常发生在第一次月经之前，您无法提前知道哪天排卵。哺乳期闭经避孕法仅在严格符合以下三个条件时才有一定的避孕效果：产后6个月内、完全纯母乳喂养（白天晚上都喂、不加配方奶和辅食）、并且没有恢复月经。只要有一个条件不满足，就必须采取其他避孕措施。哺乳期可以安全使用的避孕方法包括：1）避孕套（最简单、无激素影响）；2）单纯孕激素避孕药（需医生开具，不影响乳汁）；3）孕激素宫内节育器（如曼月乐）；4）皮下埋植剂。关于宫内节育器放置时间：顺产通常在产后4-6周（恶露干净、子宫恢复正常后）放置；剖宫产建议产后6个月或更晚，具体需经医生评估。产后42天复查时就可以咨询医生并预约放置。 | | | | | | |
| **English translation of the Chinese general response / 中文总体回答英文翻译** | | | | | | |
| You can absolutely become pregnant after birth even if your period has not yet returned, because the first ovulation usually occurs before the first menstrual period, and you cannot predict which day ovulation will happen. The lactational amenorrhea method is effective only when all three of the following strict criteria are met: within 6 months postpartum, fully exclusive breastfeeding (day and night, no formula or solids), and no return of menstruation. If any one condition is not met, other contraceptive measures must be used. Contraceptive methods safe for breastfeeding include: 1) Condoms (simplest, no hormonal effects); 2) Progestin-only pills (prescribed by a doctor, do not affect milk); 3) Progestin intrauterine devices (such as Mirena); 4) Subdermal implants. Regarding timing of IUD placement: for vaginal delivery, it is usually placed at 4 to 6 weeks postpartum (after lochia has cleared and the uterus has normalized); for cesarean section, placement is often recommended after 6 months or later, depending on medical evaluation. You can consult your doctor about this at the 42-day postpartum check-up and schedule placement. | | | | | | |
| **Item / 项目** | | **Q1 / 问题1** | | **Q2 / 问题2** | | **Q3 / 问题3** |
| **Patient question (Chinese original) / 患者原始问题（中文）** | | 产后还没来月经，会不会怀孕？ | | 哺乳期可以用哪些避孕方法？ | | 产后多久可以放置宫内节育器？ |
| **English translation of patient question / 患者问题英文翻译** | | If my period has not returned after birth, can I still become pregnant? | | What contraceptive methods can be used during breastfeeding? | | How soon after birth can an intrauterine device be placed? |
| **Model response (Chinese) / 模型回答（中文）** | | 会。产后首次排卵在第一次月经之前，必须避孕。 | | 安全方法包括避孕套、单纯孕激素避孕药、宫内节育器等。 | | 顺产通常产后4-6周，剖宫产建议产后6个月或更晚。 |
| **English translation of model response / 模型回答英文翻译** | | Yes, ovulation occurs before the first period. Contraception is necessary. | | Safe options include condoms, progestin-only pills, IUDs. | | For vaginal delivery, usually 4-6 weeks; for C-section, after 6 months. |
| **Overall evaluation item / 整体评价项目** | | | **Score / 分数** | | **Comments / 备注** | |
| **Patient Satisfaction / 患者满意度** | | | 3 | |  | |
| **Patient Understandability / 患者可理解性** | | | 4 | |  | |
| **Patient Usefulness / 患者实用性** | | | 3 | |  | |
| **Clinician Correctness / 医生评分：正确性** | | | 4 | |  | |
| **Clinician Completeness / 医生评分：完整性** | | | 3 | |  | |
| **Clinician Readability / 医生评分：可读性** | | | 4 | |  | |
| **Clinician Helpfulness / 医生评分：帮助性** | | | 3 | |  | |
| **Clinician Safety / 医生评分：安全性** | | | 4 | |  | |

**Patient 16**

| **Patient ID / 患者编号** | **Group / 模型组别** | | | | | |
| --- | --- | --- | --- | --- | --- | --- |
| P16 | DeepSeek V3 | | | | | |
| **General model response or patient education material (Chinese) / 模型总体回答或患者教育材料（中文）** | | | | | | |
| 坐月子期间完全可以洗头、洗澡，这是现代产后护理的基本常识。过去“月子不能洗头洗澡”的说法源于旧时居住条件差、没有热水和暖气、容易受凉感染，现在完全不适用。洗头和淋浴不仅不会导致“月子病”，反而能保持皮肤清洁、减少感染风险、促进血液循环、改善情绪。注意事项：1）水温适宜（38-40℃），时间不宜过长（5-10分钟）；2）洗完后立即用干毛巾擦干身体，用电吹风热风吹干头发；3）避免受凉，浴室温度保持温暖，洗后穿好衣服；4）剖宫产产妇在切口完全干燥、无渗液后可淋浴（通常术后7-10天），期间可用防水敷料保护切口，不要盆浴。关于空调：夏季完全可以开空调，室温保持在26-28℃，风口不要直吹产妇和婴儿，每天开窗通风1-2次。关于水果蔬菜：完全可以吃，推荐吃常温或稍微加热的水果（如苹果、香蕉、橙子、火龙果），以及各类蔬菜，它们提供维生素、膳食纤维，有助于预防便秘和促进恢复。 | | | | | | |
| **English translation of the Chinese general response / 中文总体回答英文翻译** | | | | | | |
| You can absolutely wash your hair and take a shower during the postpartum confinement period. This is basic knowledge of modern postpartum care. The old saying that “you cannot wash hair or bathe during confinement” originated from poor living conditions in the past, without hot water or heating, making people prone to getting chilled or infected. It is completely outdated. Washing hair and showering do not cause “confinement illnesses”; on the contrary, they keep skin clean, reduce infection risk, promote blood circulation, and improve mood. Precautions: 1) Use warm water (38-40°C) and keep the shower brief (5-10 minutes); 2) Dry your body immediately with a clean towel and blow-dry hair with warm air; 3) Avoid getting chilled — keep the bathroom warm and put on clothes after showering; 4) For cesarean births, once the incision is completely dry and without drainage (usually 7 to 10 days post-surgery), you may shower, covering the incision with a waterproof dressing; do not take tub baths. Regarding air conditioning: it is perfectly fine to use air conditioning in summer, keeping the room at 26-28°C, with the air vent not blowing directly on the mother and baby, and open windows for ventilation 1-2 times per day. Regarding fruits and vegetables: you can definitely eat them. Choose fruits at room temperature or slightly warmed (such as apples, bananas, oranges, dragon fruit) and various vegetables. They provide vitamins and dietary fiber, helping prevent constipation and promote recovery. | | | | | | |
| **Item / 项目** | | **Q1 / 问题1** | | **Q2 / 问题2** | | **Q3 / 问题3** |
| **Patient question (Chinese original) / 患者原始问题（中文）** | | 坐月子期间可以洗头洗澡吗？会不会落下月子病？ | | 产后可以吹空调吗？需要注意什么？ | | 坐月子期间可以吃水果和蔬菜吗？ |
| **English translation of patient question / 患者问题英文翻译** | | Can I wash my hair and take a shower during the postpartum confinement period? Will it cause lingering postpartum illness? | | Can I use air conditioning after birth? What should I pay attention to? | | Can I eat fruit and vegetables during the postpartum confinement period? |
| **Model response (Chinese) / 模型回答（中文）** | | 可以。现代条件下洗头洗澡不会导致“月子病”，反而有助于清洁和恢复。 | | 可以。室温保持26-28℃，风口不直吹，定期通风。 | | 可以。推荐常温或加热后的水果蔬菜，有助于预防便秘。 |
| **English translation of model response / 模型回答英文翻译** | | Yes. In modern settings, washing hair and showering do not cause “confinement illness”. | | Yes, keep room 26-28°C, avoid direct air flow, and ventilate regularly. | | Yes. Room-temperature or lightly warmed fruits and vegetables help prevent constipation. |
| **Overall evaluation item / 整体评价项目** | | | **Score / 分数** | | **Comments / 备注** | |
| **Patient Satisfaction / 患者满意度** | | | 3 | |  | |
| **Patient Understandability / 患者可理解性** | | | 4 | |  | |
| **Patient Usefulness / 患者实用性** | | | 3 | |  | |
| **Clinician Correctness / 医生评分：正确性** | | | 4 | |  | |
| **Clinician Completeness / 医生评分：完整性** | | | 3 | |  | |
| **Clinician Readability / 医生评分：可读性** | | | 4 | |  | |
| **Clinician Helpfulness / 医生评分：帮助性** | | | 3 | |  | |
| **Clinician Safety / 医生评分：安全性** | | | 4 | |  | |

**Patient 17**

| **Patient ID / 患者编号** | **Group / 模型组别** | | | | | |
| --- | --- | --- | --- | --- | --- | --- |
| P17 | DeepSeek V3 | | | | | |
| **General model response or patient education material (Chinese) / 模型总体回答或患者教育材料（中文）** | | | | | | |
| 产后下奶并不需要大量喝油腻的汤水，这是一个非常普遍的误区。真正促进乳汁分泌的关键是：频繁有效的吸吮（每天8-12次或更多）、充足的水分摄入（每天2000-3000毫升，包括白开水、清淡汤水、牛奶、豆浆等）、均衡的营养（优质蛋白、维生素、矿物质）以及充分休息和放松的心情。建议您每天保证肉、蛋、豆制品、绿叶蔬菜、主食和水果的摄入。哺乳期需要避免的食物包括：1）酒精（任何含酒精的饮品都会进入乳汁，影响宝宝神经系统发育）；2）生食或未煮熟的海鲜、肉类（预防寄生虫或细菌感染）；3）过量的咖啡因（每天不超过200-300mg，约1-2小杯咖啡）；4）您自己观察到吃了之后宝宝出现过敏或不适的食物（如某些海鲜、牛奶、鸡蛋等，因人而异）。关于产后贫血：如果您被诊断为贫血，饮食上应增加富含铁的食物，如瘦红肉、动物肝脏（每周1-2次，每次50-100g）、动物血制品、黑木耳、菠菜、豆类、红枣等，同时搭配富含维生素C的食物（如橙子、猕猴桃、青椒）促进铁吸收。请按照医嘱服用补铁药物，不要自行停药，因为饮食补铁往往不足以纠正中重度贫血。 | | | | | | |
| **English translation of the Chinese general response / 中文总体回答英文翻译** | | | | | | |
| Postpartum milk production does not require drinking large amounts of greasy soup. This is a very common misconception. The real keys to promoting milk supply are: frequent effective sucking (8-12 or more times per day), adequate fluid intake (2000-3000 mL per day, including water, light soups, milk, soy milk, etc.), balanced nutrition (high-quality protein, vitamins, minerals), and adequate rest and a relaxed mood. It is recommended that you consume meat, eggs, soy products, leafy greens, staple foods, and fruits daily. Foods to avoid during breastfeeding include: 1) Alcohol (any alcoholic beverage enters breastmilk and affects the baby’s nervous system development); 2) Raw or undercooked seafood and meat (to prevent parasitic or bacterial infection); 3) Excessive caffeine (no more than 200-300 mg per day, about 1-2 small cups of coffee); 4) Foods that you personally observe cause allergy or discomfort in your baby after you eat them (such as certain seafood, milk, eggs — varies by individual). Regarding postpartum anemia: If you have been diagnosed with anemia, increase iron-rich foods in your diet, such as lean red meat, animal liver (1-2 times per week, 50-100g each time), animal blood products, black fungus, spinach, legumes, red dates, etc. Also pair these with vitamin C-rich foods (oranges, kiwis, bell peppers) to enhance iron absorption. Take prescribed iron supplements as directed and do not stop on your own, as dietary iron alone is often insufficient to correct moderate to severe anemia. | | | | | | |
| **Item / 项目** | | **Q1 / 问题1** | | **Q2 / 问题2** | | **Q3 / 问题3** |
| **Patient question (Chinese original) / 患者原始问题（中文）** | | 产后是不是必须大量喝汤才能下奶？ | | 哺乳期哪些食物需要避免？ | | 我产后贫血，饮食上应该注意什么？ |
| **English translation of patient question / 患者问题英文翻译** | | Do I have to drink a lot of soup after birth to produce breast milk? | | What foods should be avoided during breastfeeding? | | I have anemia after birth. What should I pay attention to in my diet? |
| **Model response (Chinese) / 模型回答（中文）** | | 不是。关键是频繁有效吸吮、充足水分、均衡营养和休息。 | | 酒精、生食、过量咖啡因、引起宝宝过敏的食物。 | | 多吃红肉、动物肝脏、黑木耳、菠菜，并搭配维生素C促进铁吸收。 |
| **English translation of model response / 模型回答英文翻译** | | No. The key is frequent effective sucking, adequate fluids, balanced nutrition, and rest. | | Avoid alcohol, raw foods, excess caffeine, and allergens for the baby. | | Eat red meat, liver, black fungus, spinach with vitamin C-rich foods to enhance iron absorption. |
| **Overall evaluation item / 整体评价项目** | | | **Score / 分数** | | **Comments / 备注** | |
| **Patient Satisfaction / 患者满意度** | | | 3 | |  | |
| **Patient Understandability / 患者可理解性** | | | 4 | |  | |
| **Patient Usefulness / 患者实用性** | | | 3 | |  | |
| **Clinician Correctness / 医生评分：正确性** | | | 4 | |  | |
| **Clinician Completeness / 医生评分：完整性** | | | 3 | |  | |
| **Clinician Readability / 医生评分：可读性** | | | 4 | |  | |
| **Clinician Helpfulness / 医生评分：帮助性** | | | 3 | |  | |
| **Clinician Safety / 医生评分：安全性** | | | 4 | |  | |

**Patient 18**

| **Patient ID / 患者编号** | **Group / 模型组别** | | | | | |
| --- | --- | --- | --- | --- | --- | --- |
| P18 | DeepSeek V3 | | | | | |
| **General model response or patient education material (Chinese) / 模型总体回答或患者教育材料（中文）** | | | | | | |
| 哺乳期感冒了，请不要自己随便买感冒药吃。很多复方感冒药中含有伪麻黄碱、氯苯那敏、可待因等成分，这些成分可能通过乳汁影响宝宝（如引起嗜睡、烦躁、喂养困难、呼吸抑制等），或者减少您的乳汁分泌。正确做法是：1）如果症状轻微（流鼻涕、轻度咳嗽、无发热），可以先多喝温水、多休息、使用生理盐水喷鼻或雾化吸入，通常可以自愈；2）如果需要用药，请务必咨询医生或药师，告知您正在哺乳，他们会选择哺乳期安全的药物（如对乙酰氨基酚退热止痛、氯雷他定抗过敏等）；3）服药前阅读说明书中的“哺乳期用药”部分。关于产后疼痛能否吃止痛药：可以，但也要选择安全的药物。哺乳期首选的止痛药是对乙酰氨基酚（扑热息痛）和布洛芬，这两种药物进入乳汁的量极少，被认为对母乳喂养的婴儿安全，不需要停止哺乳。如果需要使用抗生素，绝大部分抗生素（如青霉素类、头孢菌素类、大环内酯类）在哺乳期是安全的，不需要停止哺乳。只有在使用极少数特殊抗生素时（如四环素类长期使用、氯霉素等），医生才会建议暂停哺乳。请千万不要因为需要用药而擅自停止哺乳，突然停止哺乳可能导致乳房胀痛、堵奶甚至乳腺炎。 | | | | | | |
| **English translation of the Chinese general response / 中文总体回答英文翻译** | | | | | | |
| If you catch a cold while breastfeeding, do not buy and take cold medicines on your own. Many over-the-counter combination cold products contain pseudoephedrine, chlorpheniramine, codeine, or other ingredients that can pass into breastmilk and affect the baby (causing drowsiness, irritability, feeding difficulties, respiratory depression, etc.) or reduce your milk supply. The correct approach: 1) If symptoms are mild (runny nose, mild cough, no fever), drink warm water, rest more, use saline nasal spray or nebulized saline — this often resolves on its own; 2) If medication is needed, always consult a doctor or pharmacist and inform them that you are breastfeeding. They can select breastfeeding-safe options (such as acetaminophen for fever/pain, loratadine for allergies); 3) Before taking any medication, read the “breastfeeding” section of the label. Regarding pain medication after birth: yes, you can take pain relievers, but you must choose safe ones. The first-line pain relievers during breastfeeding are acetaminophen (paracetamol) and ibuprofen. Both enter breastmilk in very small amounts and are considered safe for nursing infants. You do not need to stop breastfeeding. If you need antibiotics, the vast majority (such as penicillins, cephalosporins, macrolides) are safe during breastfeeding and do not require stopping breastfeeding. Only in rare cases with specific antibiotics (such as long-term tetracyclines, chloramphenicol) might a doctor advise temporarily stopping breastfeeding. Please do not stop breastfeeding on your own just because you need medication — abruptly stopping can cause breast engorgement, blocked ducts, or even mastitis. | | | | | | |
| **Item / 项目** | | **Q1 / 问题1** | | **Q2 / 问题2** | | **Q3 / 问题3** |
| **Patient question (Chinese original) / 患者原始问题（中文）** | | 哺乳期感冒了，可以自己吃感冒药吗？ | | 产后疼痛能不能吃止痛药？会不会影响母乳？ | | 如果需要吃抗生素，是不是必须停止哺乳？ |
| **English translation of patient question / 患者问题英文翻译** | | If I catch a cold while breastfeeding, can I take cold medicine by myself? | | Can I take pain medicine after birth, and will it affect breast milk? | | If I need antibiotics, must I stop breastfeeding? |
| **Model response (Chinese) / 模型回答（中文）** | | 不建议。很多复方感冒药可能影响乳汁和宝宝，应咨询医生。 | | 可以。对乙酰氨基酚和布洛芬对哺乳是安全的。 | | 不是。绝大多数抗生素（如青霉素类、头孢类）在哺乳期是安全的。 |
| **English translation of model response / 模型回答英文翻译** | | No. Many cold medicines can affect milk or baby. Consult a doctor first. | | Yes. Acetaminophen and ibuprofen are safe during breastfeeding. | | No, most antibiotics (penicillins, cephalosporins) are safe during breastfeeding. |
| **Overall evaluation item / 整体评价项目** | | | **Score / 分数** | | **Comments / 备注** | |
| **Patient Satisfaction / 患者满意度** | | | 3 | |  | |
| **Patient Understandability / 患者可理解性** | | | 4 | |  | |
| **Patient Usefulness / 患者实用性** | | | 3 | |  | |
| **Clinician Correctness / 医生评分：正确性** | | | 4 | |  | |
| **Clinician Completeness / 医生评分：完整性** | | | 3 | |  | |
| **Clinician Readability / 医生评分：可读性** | | | 4 | |  | |
| **Clinician Helpfulness / 医生评分：帮助性** | | | 3 | |  | |
| **Clinician Safety / 医生评分：安全性** | | | 4 | |  | |

**Patient 19**

| **Patient ID / 患者编号** | **Group / 模型组别** | | | | | |
| --- | --- | --- | --- | --- | --- | --- |
| P19 | DeepSeek V3 | | | | | |
| **General model response or patient education material (Chinese) / 模型总体回答或患者教育材料（中文）** | | | | | | |
| 您孕期有高血压，即使产后血压恢复正常，仍然需要继续监测。因为一部分妊娠期高血压或子痫前期患者的血压会在产后几天到几周内再次升高，甚至出现产后子痫或心脑血管并发症。建议您产后每天早晚各测量一次血压，并记录下来，至少持续监测到产后6周。产后头痛、眼花、视力模糊、胸闷、上腹部疼痛（尤其在右上腹或心口位置）、恶心呕吐、双下肢明显水肿，这些都是可能与血压升高有关的重要警示症状，出现任何一个都请立即测量血压。如果收缩压≥140 mmHg和/或舒张压≥90 mmHg，尤其是在伴有上述症状时，请尽快就医。如果收缩压≥160 mmHg或舒张压≥110 mmHg，请立即去急诊，不要等待。请不要自行停用或调整降压药物，所有用药调整必须在医生指导下进行。产后复查时医生会根据您的血压情况决定是否继续用药或停药。 | | | | | | |
| **English translation of the Chinese general response / 中文总体回答英文翻译** | | | | | | |
| You had high blood pressure during pregnancy. Even if your blood pressure becomes normal after delivery, you still need to continue monitoring it. This is because a proportion of patients with gestational hypertension or preeclampsia will experience a rise in blood pressure again in the days to weeks after birth, and may develop postpartum eclampsia or cardiovascular complications. It is recommended that you measure your blood pressure twice daily (morning and evening) and record the values, continuing for at least 6 weeks postpartum. Postpartum headache, blurred vision, visual changes, chest tightness, upper abdominal pain (especially in the right upper quadrant or epigastric area), nausea/vomiting, and marked leg swelling are all important warning signs that may be related to elevated blood pressure. If any of these occur, measure your blood pressure immediately. If your systolic blood pressure is ≥140 mmHg and/or diastolic ≥90 mmHg, especially with the above symptoms, seek medical attention promptly. If your systolic BP is ≥160 mmHg or diastolic ≥110 mmHg, go to the emergency department immediately without waiting. Do not stop or adjust your blood pressure medications on your own; all medication changes must be under a doctor’s guidance. At your postpartum check-up, the doctor will decide whether to continue or discontinue medication based on your blood pressure status. | | | | | | |
| **Item / 项目** | | **Q1 / 问题1** | | **Q2 / 问题2** | | **Q3 / 问题3** |
| **Patient question (Chinese original) / 患者原始问题（中文）** | | 我孕期有高血压，产后血压正常了还需要继续监测吗？ | | 产后头痛、眼花是不是和血压有关？ | | 如果产后血压又升高，需要马上去医院吗？ |
| **English translation of patient question / 患者问题英文翻译** | | I had high blood pressure during pregnancy. If my blood pressure is normal after birth, do I still need monitoring? | | Are postpartum headache and blurred vision related to blood pressure? | | If my blood pressure rises again after birth, do I need to go to the hospital immediately? |
| **Model response (Chinese) / 模型回答（中文）** | | 需要。产后血压可能再次升高，建议每天监测到产后6周。 | | 是。这些可能是高血压的警示症状，请立即测量血压。 | | 如果收缩压≥160或舒张压≥110，请立即去急诊。 |
| **English translation of model response / 模型回答英文翻译** | | Yes, blood pressure can rise again. Monitor daily for at least 6 weeks. | | Yes, these can be warning signs of high blood pressure. Check BP immediately. | | If systolic ≥160 or diastolic ≥110, go to the emergency room immediately. |
| **Overall evaluation item / 整体评价项目** | | | **Score / 分数** | | **Comments / 备注** | |
| **Patient Satisfaction / 患者满意度** | | | 3 | |  | |
| **Patient Understandability / 患者可理解性** | | | 4 | |  | |
| **Patient Usefulness / 患者实用性** | | | 3 | |  | |
| **Clinician Correctness / 医生评分：正确性** | | | 4 | |  | |
| **Clinician Completeness / 医生评分：完整性** | | | 3 | |  | |
| **Clinician Readability / 医生评分：可读性** | | | 4 | |  | |
| **Clinician Helpfulness / 医生评分：帮助性** | | | 3 | |  | |
| **Clinician Safety / 医生评分：安全性** | | | 4 | |  | |

**Patient 20**

| **Patient ID / 患者编号** | **Group / 模型组别** | | | | | |
| --- | --- | --- | --- | --- | --- | --- |
| P20 | DeepSeek V3 | | | | | |
| **General model response or patient education material (Chinese) / 模型总体回答或患者教育材料（中文）** | | | | | | |
| 您孕期有妊娠期糖尿病，产后仍然需要定期监测血糖。因为部分妊娠期糖尿病患者产后会发展为持续性的血糖异常（糖尿病前期或2型糖尿病）。通常建议在产后6-12周进行口服葡萄糖耐量试验（OGTT）评估糖代谢状态，而不是仅测空腹血糖或随意指尖血糖。即使产后血糖完全正常，您以后发生2型糖尿病的风险也比没有妊娠期糖尿病的女性高出数倍，因此不能“完全不用管”。建议您保持健康的生活方式：均衡饮食（控制精制碳水化合物和添加糖的摄入）、规律运动（如每周150分钟中等强度有氧运动）、控制体重在健康范围（BMI＜24），并且每年至少复查一次空腹血糖或糖化血红蛋白。妊娠期糖尿病确实会显著增加未来患2型糖尿病的风险，但通过长期的饮食、运动和体重管理，可以有效降低这一风险。如果产后OGTT提示糖尿病前期或糖尿病，请及时到内分泌科就诊。 | | | | | | |
| **English translation of the Chinese general response / 中文总体回答英文翻译** | | | | | | |
| You had gestational diabetes during pregnancy. After delivery, you still need to monitor your blood glucose regularly. This is because some women with gestational diabetes will develop persistent glucose abnormalities (prediabetes or type 2 diabetes) postpartum. It is generally recommended to undergo an oral glucose tolerance test at 6 to 12 weeks postpartum to assess your metabolic status, rather than just checking fasting glucose or random fingerstick tests. Even if your postpartum glucose is completely normal, your future risk of type 2 diabetes is several times higher than that of women without gestational diabetes, so you cannot “completely ignore it.” You are advised to maintain a healthy lifestyle: a balanced diet (limit refined carbohydrates and added sugars), regular physical activity (such as 150 minutes of moderate-intensity aerobic exercise per week), maintaining a healthy weight (BMI <24), and having at least an annual check of fasting glucose or hemoglobin A1c. Gestational diabetes does significantly increase your future risk of developing type 2 diabetes, but long-term lifestyle management (diet, exercise, weight control) can effectively reduce this risk. If your postpartum OGTT indicates prediabetes or diabetes, please see an endocrinologist promptly. | | | | | | |
| **Item / 项目** | | **Q1 / 问题1** | | **Q2 / 问题2** | | **Q3 / 问题3** |
| **Patient question (Chinese original) / 患者原始问题（中文）** | | 我孕期有妊娠糖尿病，产后还需要测血糖吗？ | | 产后血糖正常了，是不是就不用管了？ | | 妊娠糖尿病会不会增加以后得糖尿病的风险？ |
| **English translation of patient question / 患者问题英文翻译** | | I had gestational diabetes during pregnancy. Do I still need to check my blood glucose after birth? | | If my postpartum blood glucose is normal, does that mean I no longer need to worry about it? | | Does gestational diabetes increase my risk of developing diabetes in the future? |
| **Model response (Chinese) / 模型回答（中文）** | | 需要。建议产后6-12周做口服葡萄糖耐量试验。 | | 不是。未来糖尿病风险仍高，需每年复查血糖。 | | 会。但通过饮食、运动和体重管理可有效降低风险。 |
| **English translation of model response / 模型回答英文翻译** | | Yes, you need an oral glucose tolerance test at 6-12 weeks postpartum. | | No. Your future diabetes risk is higher. Annual glucose checks are recommended. | | Yes, but you can reduce risk with diet, exercise, and weight management. |
| **Overall evaluation item / 整体评价项目** | | | **Score / 分数** | | **Comments / 备注** | |
| **Patient Satisfaction / 患者满意度** | | | 3 | |  | |
| **Patient Understandability / 患者可理解性** | | | 4 | |  | |
| **Patient Usefulness / 患者实用性** | | | 3 | |  | |
| **Clinician Correctness / 医生评分：正确性** | | | 4 | |  | |
| **Clinician Completeness / 医生评分：完整性** | | | 3 | |  | |
| **Clinician Readability / 医生评分：可读性** | | | 4 | |  | |
| **Clinician Helpfulness / 医生评分：帮助性** | | | 3 | |  | |
| **Clinician Safety / 医生评分：安全性** | | | 4 | |  | |

**Patient 21**

| **Patient ID / 患者编号** | **Group / 模型组别** | | | | | |
| --- | --- | --- | --- | --- | --- | --- |
| P21 | DeepSeek V3 | | | | | |
| **General model response or patient education material (Chinese) / 模型总体回答或患者教育材料（中文）** | | | | | | |
| 产后腿有点肿，需要区分正常水肿和血栓。正常水肿通常是双侧对称的，按压后有凹陷，早晨较轻、下午或久站后加重，不伴有剧烈疼痛、皮温升高或皮肤颜色改变，且会随休息抬高下肢而逐渐减轻。如果是一侧小腿（或大腿）出现肿胀、疼痛、皮温升高、皮肤发红或发紫、按压时有明显压痛点，要高度警惕深静脉血栓，需要立即去医院做下肢血管超声检查。如果同时出现胸痛、胸闷、气短、呼吸困难、咯血或晕厥，这可能是血栓脱落引起肺栓塞的表现，属于危急情况，请立即拨打急救电话或去急诊。产后由于血液处于高凝状态，血栓风险比非孕期显著增加，尤其是剖宫产、长时间卧床、肥胖、高龄或有血栓病史的产妇。请千万不要按摩、热敷或活动那条疼痛肿胀的腿，以免血栓脱落。 | | | | | | |
| **English translation of the Chinese general response / 中文总体回答英文翻译** | | | | | | |
| Having slightly swollen legs after birth requires distinguishing between normal edema and a blood clot. Normal edema is usually bilateral and symmetric, with pitting on pressure, worse in the afternoon or after standing, better in the morning or with leg elevation, and not accompanied by severe pain, warmth, or skin color changes. If one calf (or thigh) becomes swollen, painful, warm to the touch, red or purple, and has point tenderness on pressure, deep vein thrombosis should be highly suspected. You should go to the hospital immediately for a lower extremity vascular ultrasound. If you also experience chest pain, chest tightness, shortness of breath, difficulty breathing, coughing up blood, or fainting, this may indicate pulmonary embolism from a dislodged clot — this is an emergency. Call emergency services or go to the emergency department immediately. Postpartum, due to a hypercoagulable state, the risk of blood clots is significantly higher than in non-pregnant women, especially after cesarean section, prolonged bed rest, obesity, advanced maternal age, or a history of thrombosis. Do NOT massage, apply heat, or exercise the painful, swollen leg, as this may dislodge the clot. | | | | | | |
| **Item / 项目** | | **Q1 / 问题1** | | **Q2 / 问题2** | | **Q3 / 问题3** |
| **Patient question (Chinese original) / 患者原始问题（中文）** | | 产后腿有点肿，是正常水肿还是血栓？ | | 如果一侧小腿疼痛、发热、肿胀，需要去医院吗？ | | 产后胸闷、气短会不会和肺栓塞有关？ |
| **English translation of patient question / 患者问题英文翻译** | | My legs are a little swollen after birth. Is it normal edema or a blood clot? | | If one calf is painful, warm, and swollen, do I need to go to the hospital? | | Could postpartum chest tightness and shortness of breath be related to pulmonary embolism? |
| **Model response (Chinese) / 模型回答（中文）** | | 双侧对称、晨轻暮重多为正常水肿；一侧肿痛发热需警惕血栓。 | | 需要立即去医院做下肢血管超声，排查深静脉血栓。 | | 会。这是危急情况，请立即拨打急救电话。 |
| **English translation of model response / 模型回答英文翻译** | | Bilateral and morning-light edema is normal. Unilateral swelling with pain/warmth may be DVT. | | Yes, go immediately for a lower extremity ultrasound to rule out DVT. | | Yes, this could be pulmonary embolism. Call emergency services immediately. |
| **Overall evaluation item / 整体评价项目** | | | **Score / 分数** | | **Comments / 备注** | |
| **Patient Satisfaction / 患者满意度** | | | 3 | |  | |
| **Patient Understandability / 患者可理解性** | | | 4 | |  | |
| **Patient Usefulness / 患者实用性** | | | 3 | |  | |
| **Clinician Correctness / 医生评分：正确性** | | | 4 | |  | |
| **Clinician Completeness / 医生评分：完整性** | | | 3 | |  | |
| **Clinician Readability / 医生评分：可读性** | | | 4 | |  | |
| **Clinician Helpfulness / 医生评分：帮助性** | | | 3 | |  | |
| **Clinician Safety / 医生评分：安全性** | | | 4 | |  | |

**Patient 22**

| **Patient ID / 患者编号** | **Group / 模型组别** | | | | | |
| --- | --- | --- | --- | --- | --- | --- |
| P22 | DeepSeek V3 | | | | | |
| **General model response or patient education material (Chinese) / 模型总体回答或患者教育材料（中文）** | | | | | | |
| 剖宫产后不建议长期卧床休息。恰恰相反，在身体条件允许的情况下，早期下床活动对您的恢复非常有益。一般建议术后24-48小时内，在护士或家人搀扶下慢慢下床站立或缓慢行走几步。早期下床的好处包括：促进肠道蠕动恢复、预防便秘和肠粘连、预防下肢静脉血栓、改善肺部通气、减少肺部感染风险、以及帮助子宫恢复。关于抱孩子，通常术后2-4周内尽量避免频繁或长时间抱孩子，尤其要避免抱起较重的宝宝或提举超过5公斤的物品。需要抱宝宝时，可以坐在有靠背的椅子上，用枕头或哺乳枕垫在腹部刀口前方，让宝宝的身体靠在枕头上而不是直接压在您的腹部。正常下床活动和轻度走动一般不会导致切口裂开，因为腹部切口有多层缝合（包括腹直肌前鞘），在术后7-10天皮肤愈合后，日常活动产生的张力远远低于切口能承受的强度。只有在剧烈咳嗽、用力排便、猛然提重物或剧烈运动时才可能发生切口裂开。 | | | | | | |
| **English translation of the Chinese general response / 中文总体回答英文翻译** | | | | | | |
| Long-term bed rest is not recommended after a cesarean birth. On the contrary, early ambulation, as tolerated, is very beneficial for your recovery. It is generally recommended to get out of bed with the help of a nurse or family member and slowly stand or take a few steps within 24 to 48 hours after surgery. The benefits of early ambulation include: promoting bowel motility, preventing constipation and intestinal adhesions, preventing lower extremity deep vein thrombosis, improving lung ventilation, reducing pulmonary infection risk, and helping uterine involution. Regarding holding the baby: within the first 2 to 4 weeks after surgery, try to avoid frequent or prolonged holding of the baby, especially lifting a heavier baby or any object heavier than 5 kg. When you need to hold the baby, sit in a chair with back support, place a pillow or nursing pillow in front of the incision, and let the baby rest against the pillow rather than directly on your abdomen. Getting out of bed and gentle walking generally will not cause the incision to open, because the abdominal incision is closed in multiple layers (including the rectus sheath). After skin healing at 7 to 10 days, the tension from daily activities is far below what the incision can withstand. Incisional dehiscence usually occurs only with severe coughing, straining during bowel movements, sudden heavy lifting, or vigorous exercise. | | | | | | |
| **Item / 项目** | | **Q1 / 问题1** | | **Q2 / 问题2** | | **Q3 / 问题3** |
| **Patient question (Chinese original) / 患者原始问题（中文）** | | 剖宫产后是不是要尽量卧床休息？ | | 剖宫产后多久可以下床走动和抱孩子？ | | 下床活动会不会导致切口裂开？ |
| **English translation of patient question / 患者问题英文翻译** | | After a cesarean birth, should I stay in bed as much as possible? | | How soon after a cesarean birth can I get out of bed and hold my baby? | | Will getting out of bed cause the incision to open? |
| **Model response (Chinese) / 模型回答（中文）** | | 不是。早期下床活动有助于恢复，预防血栓和便秘。 | | 术后24-48小时内可下床；抱孩子建议2-4周后，避免提重物。 | | 正常走动不会。剧烈咳嗽、用力排便、提重物才可能裂开。 |
| **English translation of model response / 模型回答英文翻译** | | No. Early ambulation helps recovery and prevents clots and constipation. | | You can walk in 24-48 hours; hold baby after 2-4 weeks, avoiding heavy lifting. | | No, normal walking won’t. Severe coughing, straining, or heavy lifting might. |
| **Overall evaluation item / 整体评价项目** | | | **Score / 分数** | | **Comments / 备注** | |
| **Patient Satisfaction / 患者满意度** | | | 3 | |  | |
| **Patient Understandability / 患者可理解性** | | | 4 | |  | |
| **Patient Usefulness / 患者实用性** | | | 3 | |  | |
| **Clinician Correctness / 医生评分：正确性** | | | 4 | |  | |
| **Clinician Completeness / 医生评分：完整性** | | | 3 | |  | |
| **Clinician Readability / 医生评分：可读性** | | | 4 | |  | |
| **Clinician Helpfulness / 医生评分：帮助性** | | | 3 | |  | |
| **Clinician Safety / 医生评分：安全性** | | | 4 | |  | |

**Patient 23**

| **Patient ID / 患者编号** | **Group / 模型组别** | | | | | |
| --- | --- | --- | --- | --- | --- | --- |
| P23 | DeepSeek V3 | | | | | |
| **General model response or patient education material (Chinese) / 模型总体回答或患者教育材料（中文）** | | | | | | |
| 会阴伤口有轻微异味，不一定是感染，也可能是正常的伤口渗出液混合汗液或分泌物引起的味道。但如果异味明显加重、变成腥臭味或腐臭味，同时伴有黄色或黄绿色的脓性分泌物、伤口周围红肿范围扩大、疼痛越来越重、用手触摸伤口局部温度升高、伤口裂开，或者您出现发热（≥38℃），那么高度提示伤口感染。关于会阴伤口分泌物的清洗，通常用流动的温水从前向后冲洗即可，每天1-2次，便后也要冲洗。不需要常规使用消毒液（如碘伏、高锰酸钾等）反复清洗，因为过度使用消毒液可能破坏正常菌群、延迟伤口愈合、甚至引起刺激或过敏。只有在医生明确诊断为感染并建议使用特定消毒剂时，才按医嘱使用。如果会阴伤口疼痛越来越明显，尤其是在产后3-5天后疼痛不减反增，而不是逐渐减轻，这是一个非常值得警惕的信号，强烈建议您尽快到医院产科或妇科门诊复查，让医生做妇科检查评估伤口情况。 | | | | | | |
| **English translation of the Chinese general response / 中文总体回答英文翻译** | | | | | | |
| A mild odor from the perineal wound does not necessarily mean infection; it may be a normal smell from wound exudate mixed with sweat or vaginal discharge. However, if the odor becomes distinctly foul or putrid, and is accompanied by yellow or green purulent discharge, spreading redness around the wound, worsening pain, local warmth on palpation, wound dehiscence, or fever (≥38°C), then wound infection is highly suspected. Regarding cleaning of perineal discharge: generally, rinsing with lukewarm running water from front to back, once or twice daily and after each bowel movement, is sufficient. There is no need to routinely use disinfectants (such as povidone-iodine or potassium permanganate) repeatedly, as overuse of disinfectants may disrupt normal flora, delay wound healing, or even cause irritation or allergy. Only use disinfectants if a doctor has clearly diagnosed an infection and specifically recommended them. If perineal wound pain becomes increasingly worse — especially if it does not improve but worsens after the first 3 to 5 days — this is a very concerning sign. It is strongly recommended that you return to the obstetric or gynecology outpatient clinic as soon as possible for a physical examination to assess the wound. | | | | | | |
| **Item / 项目** | | **Q1 / 问题1** | | **Q2 / 问题2** | | **Q3 / 问题3** |
| **Patient question (Chinese original) / 患者原始问题（中文）** | | 我顺产撕裂伤口有点异味，是不是感染了？ | | 会阴伤口如果有分泌物，需要用消毒液清洗吗？ | | 会阴伤口疼痛越来越明显，需要复查吗？ |
| **English translation of patient question / 患者问题英文翻译** | | My tear wound after vaginal birth has a slight odor. Is it infected? | | If there is discharge from the perineal wound, should I clean it with disinfectant? | | If the perineal wound pain is getting worse, do I need a follow-up check? |
| **Model response (Chinese) / 模型回答（中文）** | | 不一定。但如果异味加重、伴脓性分泌物或红肿疼痛，可能是感染。 | | 不需要。用温水冲洗即可，过度消毒反而影响愈合。 | | 需要。这是感染或愈合不良的信号，请尽快复查。 |
| **English translation of model response / 模型回答英文翻译** | | Not necessarily. But if odor worsens with pus, redness, or pain, suspect infection. | | No, just rinse with warm water. Overuse of disinfectants delays healing. | | Yes, worsening pain suggests infection or poor healing. See a doctor. |
| **Overall evaluation item / 整体评价项目** | | | **Score / 分数** | | **Comments / 备注** | |
| **Patient Satisfaction / 患者满意度** | | | 3 | |  | |
| **Patient Understandability / 患者可理解性** | | | 4 | |  | |
| **Patient Usefulness / 患者实用性** | | | 3 | |  | |
| **Clinician Correctness / 医生评分：正确性** | | | 4 | |  | |
| **Clinician Completeness / 医生评分：完整性** | | | 3 | |  | |
| **Clinician Readability / 医生评分：可读性** | | | 4 | |  | |
| **Clinician Helpfulness / 医生评分：帮助性** | | | 3 | |  | |
| **Clinician Safety / 医生评分：安全性** | | | 4 | |  | |

**Patient 24**

| **Patient ID / 患者编号** | **Group / 模型组别** | | | | | |
| --- | --- | --- | --- | --- | --- | --- |
| P24 | DeepSeek V3 | | | | | |
| **General model response or patient education material (Chinese) / 模型总体回答或患者教育材料（中文）** | | | | | | |
| 乳房有硬块但不发烧，首先高度怀疑是乳汁淤积（堵奶），这种情况绝大多数不需要停止哺乳。相反，继续哺乳或挤奶是最好的疏通方法。您可以从硬块处向乳头方向非常轻柔地打圈按摩，然后优先从患侧乳房开始喂奶，让宝宝下巴对准硬块的方向吸吮。喂奶前温敷2-3分钟、喂奶后冷敷15分钟可以帮助缓解。但请一定不要用力揉搓或暴力按压硬块，这会导致乳腺组织水肿和炎症加重，甚至诱发乳腺炎。关于堵奶时能否继续喂奶：完全可以，而且强烈建议继续喂奶，因为宝宝的吸吮是最高效的排乳方式。突然暂停哺乳反而会让乳汁更淤积，增加乳腺炎风险。只有在医生明确诊断为化脓性乳腺炎、需要切开引流或使用对哺乳有影响的特殊药物时，才可能需要暂停亲喂（但仍可挤出弃去）。关于乳腺炎是否需要抗生素：如果是轻度乳腺炎，有时仅靠增加排乳和休息可自愈；但如果出现发热、红肿范围扩大、疼痛剧烈、或者24-48小时无改善，通常需要使用抗生素。哺乳期可以安全使用的抗生素很多（如头孢类、青霉素类、克林霉素等），绝大多数不影响继续哺乳。请让医生开具哺乳期安全的药物，切勿自行使用喹诺酮类或四环素类。 | | | | | | |
| **English translation of the Chinese general response / 中文总体回答英文翻译** | | | | | | |
| A breast lump without fever is most likely milk stasis (blocked duct). In this situation, stopping breastfeeding is almost never needed. On the contrary, continuing to breastfeed or pump is the best way to clear the duct. You can gently massage in circles from the lump toward the nipple, then start feeding on the affected side, with the baby’s chin pointing toward the lump. Warm compresses for 2-3 minutes before feeding and cold compresses for 15 minutes after feeding can help. However, do NOT forcefully knead or violently press the lump, as this can cause breast tissue edema and inflammation, and even trigger mastitis. Regarding whether to continue breastfeeding with a blocked duct: yes, absolutely, and it is strongly recommended. The baby’s sucking is the most effective way to drain milk. Suddenly stopping breastfeeding will worsen milk stasis and increase the risk of mastitis. Only if a doctor diagnoses suppurative mastitis requiring incision and drainage, or prescribes specific medications that are not breastfeeding-safe, might you need to temporarily stop direct feeding (but still pump and discard). Regarding whether mastitis requires antibiotics: For mild mastitis, sometimes increased milk removal and rest can resolve it. However, if you develop fever, spreading redness, severe pain, or no improvement in 24-48 hours, antibiotics are usually necessary. Many antibiotics are safe during breastfeeding (such as cephalosporins, penicillins, clindamycin), and most do not require stopping breastfeeding. Have your doctor prescribe breastfeeding-safe medications, and do not use fluoroquinolones or tetracyclines on your own. | | | | | | |
| **Item / 项目** | | **Q1 / 问题1** | | **Q2 / 问题2** | | **Q3 / 问题3** |
| **Patient question (Chinese original) / 患者原始问题（中文）** | | 我乳房有硬块但不发烧，可以自己按摩揉开吗？ | | 堵奶时继续喂奶好，还是应该先暂停哺乳？ | | 乳腺炎需要吃抗生素吗？会不会影响宝宝吃奶？ |
| **English translation of patient question / 患者问题英文翻译** | | I have a breast lump but no fever. Can I massage it open by myself? | | When there is a blocked duct, is it better to continue breastfeeding or stop temporarily? | | Does mastitis require antibiotics, and will that affect the baby during feeding? |
| **Model response (Chinese) / 模型回答（中文）** | | 可以轻柔按摩从硬块向乳头方向，但不要暴力揉搓。 | | 继续喂奶更好。宝宝吸吮是最有效的疏通方式。 | | 需要时使用哺乳期安全的抗生素（如头孢类），不需要停止哺乳。 |
| **English translation of model response / 模型回答英文翻译** | | Yes, gentle massage from lump toward nipple, but avoid forceful kneading. | | Continue breastfeeding. Baby’s sucking is the most effective way to clear the duct. | | Yes, but use breastfeeding-safe antibiotics (e.g., cephalosporins). No need to stop nursing. |
| **Overall evaluation item / 整体评价项目** | | | **Score / 分数** | | **Comments / 备注** | |
| **Patient Satisfaction / 患者满意度** | | | 3 | |  | |
| **Patient Understandability / 患者可理解性** | | | 4 | |  | |
| **Patient Usefulness / 患者实用性** | | | 3 | |  | |
| **Clinician Correctness / 医生评分：正确性** | | | 4 | |  | |
| **Clinician Completeness / 医生评分：完整性** | | | 3 | |  | |
| **Clinician Readability / 医生评分：可读性** | | | 4 | |  | |
| **Clinician Helpfulness / 医生评分：帮助性** | | | 3 | |  | |
| **Clinician Safety / 医生评分：安全性** | | | 4 | |  | |

**Patient 25**

| **Patient ID / 患者编号** | **Group / 模型组别** | | | | | |
| --- | --- | --- | --- | --- | --- | --- |
| P25 | DeepSeek V3 | | | | | |
| **General model response or patient education material (Chinese) / 模型总体回答或患者教育材料（中文）** | | | | | | |
| 产后42天复查是产褥期非常重要的一次全面健康评估，强烈建议您按时去，即使您自我感觉恢复得还不错。因为很多产后恢复问题（如子宫复旧不良、轻度盆底功能障碍、血压或血糖异常、贫血、情绪问题等）在早期可能没有明显症状，但如果不及时发现和处理，可能会留下长期影响。42天复查的主要内容通常包括：1）问诊：恶露情况、伤口愈合、排便排尿、睡眠情绪、哺乳问题等；2）体格检查：子宫复旧、会阴或剖宫产伤口、乳房、血压、体重；3）辅助检查：血常规（排除贫血）、尿常规、妇科B超（评估子宫大小、有无残留物）；4）盆底功能初步评估；5）避孕咨询和指导。不能等到42天复查、需要提前就医的情况包括：大量出血（超过月经量或每小时湿透一张卫生巾）、发热≥38℃、严重腹痛、伤口红肿流脓或裂开、胸闷气短或呼吸困难、单侧小腿肿胀疼痛、以及任何有自伤或伤害宝宝的想法。出现以上任何一种情况，请立即就医。 | | | | | | |
| **English translation of the Chinese general response / 中文总体回答英文翻译** | | | | | | |
| The 42-day postpartum check-up is a very important comprehensive health assessment during the puerperium. It is strongly recommended that you attend it as scheduled, even if you feel you have recovered quite well. This is because many postpartum issues (such as poor uterine involution, mild pelvic floor dysfunction, blood pressure or glucose abnormalities, anemia, mood problems) may not have obvious symptoms early on, but if not detected and managed in time, they can have long-term consequences. The main components of the 42-day check-up typically include: 1) Interview: lochia status, wound healing, bowel and bladder function, sleep and mood, breastfeeding issues; 2) Physical examination: uterine involution, perineal or cesarean wound, breasts, blood pressure, weight; 3) Laboratory and imaging: complete blood count, urinalysis, pelvic ultrasound (to assess uterine size and retained products); 4) Preliminary pelvic floor function assessment; 5) Contraception counseling and guidance. Situations that should NOT wait until the 42-day check-up but require earlier medical care include: heavy bleeding (more than a menstrual period or soaking one pad per hour), fever ≥38°C, severe abdominal pain, wound redness with pus drainage or dehiscence, chest tightness, shortness of breath, or difficulty breathing, unilateral calf swelling and pain, and any thoughts of harming yourself or the baby. If any of these occur, seek immediate medical care. | | | | | | |
| **Item / 项目** | | **Q1 / 问题1** | | **Q2 / 问题2** | | **Q3 / 问题3** |
| **Patient question (Chinese original) / 患者原始问题（中文）** | | 产后42天复查一定要去吗？主要检查什么？ | | 如果我感觉恢复得还可以，可以不去产后复查吗？ | | 产后哪些情况不能等到42天复查，应该提前就医？ |
| **English translation of patient question / 患者问题英文翻译** | | Must I attend the 42-day postpartum check-up? What is mainly checked? | | If I feel that I have recovered fairly well, can I skip the postpartum check-up? | | What postpartum situations should not wait until the 42-day check-up and require earlier medical care? |
| **Model response (Chinese) / 模型回答（中文）** | | 一定要去。主要检查子宫复旧、伤口、盆底功能、血尿常规等。 | | 不可以。很多问题（如盆底功能障碍）早期无症状。 | | 大量出血、发热≥38℃、严重腹痛、伤口流脓、胸闷、单腿肿痛等。 |
| **English translation of model response / 模型回答英文翻译** | | Yes, it is essential. It checks uterine involution, wounds, pelvic floor, blood/urine tests. | | No, many issues (like pelvic floor dysfunction) have no early symptoms. | | Heavy bleeding, fever ≥38°C, severe pain, wound pus, chest tightness, unilateral leg swelling. |
| **Overall evaluation item / 整体评价项目** | | | **Score / 分数** | | **Comments / 备注** | |
| **Patient Satisfaction / 患者满意度** | | | 3 | |  | |
| **Patient Understandability / 患者可理解性** | | | 4 | |  | |
| **Patient Usefulness / 患者实用性** | | | 3 | |  | |
| **Clinician Correctness / 医生评分：正确性** | | | 4 | |  | |
| **Clinician Completeness / 医生评分：完整性** | | | 3 | |  | |
| **Clinician Readability / 医生评分：可读性** | | | 4 | |  | |
| **Clinician Helpfulness / 医生评分：帮助性** | | | 3 | |  | |
| **Clinician Safety / 医生评分：安全性** | | | 4 | |  | |

**Patient 26**

| **Patient ID / 患者编号** | **Group / 模型组别** | | | | | |
| --- | --- | --- | --- | --- | --- | --- |
| P26 | DeepSeek V3 | | | | | |
| **General model response or patient education material (Chinese) / 模型总体回答或患者教育材料（中文）** | | | | | | |
| 产后总觉得腰酸背痛，绝大多数情况下并不是因为“月子没坐好”，而是与以下因素有关：孕期体内分泌松弛素导致骨盆和脊柱韧带松弛；产后腹部核心肌群力量减弱，腰椎代偿性负荷增加；长时间弯腰喂奶、换尿布、抱孩子；睡眠不足导致肌肉疲劳恢复慢。因此请不要为此自责或焦虑。抱孩子和喂奶时保护腰背的方法：1）喂奶时使用哺乳枕或普通枕头垫高宝宝，让宝宝的高度刚好到达乳房位置，您不需要弯腰或耸肩，同时用靠枕支撑您的腰背部；2）抱宝宝时尽量让宝宝贴近您的身体，核心收紧，不要向一侧倾斜或挺着肚子；3）避免长时间保持同一个姿势，每20-30分钟更换姿势或站起来走动一下；4）从地面抱起宝宝时，先屈膝下蹲、保持腰背挺直，而不是直接弯腰。如果腰痛越来越重，出现以下情况时需要就医：疼痛向下放射到臀部或大腿后方、腿部麻木或无力、伴有发热或排尿异常（尿失禁或尿潴留）、或者休息和改变姿势后完全不缓解。这些情况可能提示椎间盘问题、骶髂关节紊乱或少见但严重的脊柱感染。 | | | | | | |
| **English translation of the Chinese general response / 中文总体回答英文翻译** | | | | | | |
| Persistent lower back pain after birth is most often NOT due to “not doing confinement properly,” but rather related to the following factors: pregnancy-induced relaxin secretion causing laxity of pelvic and spinal ligaments; weakened abdominal core muscles postpartum, leading to compensatory increased load on the lumbar spine; prolonged bending while feeding, diaper changing, or holding the baby; and sleep deprivation causing delayed muscle fatigue recovery. So please do not blame or feel anxious about this. Ways to protect your back when holding the baby and breastfeeding: 1) When breastfeeding, use a nursing pillow or regular pillow to raise the baby so that the baby reaches your breast without you needing to bend or shrug, and use a back pillow to support your lumbar spine; 2) When holding the baby, bring the baby close to your body, engage your core, and avoid leaning to one side or arching your belly; 3) Avoid staying in the same position for too long; change positions or stand up and walk every 20-30 minutes; 4) When picking up the baby from the floor, first squat with knees bent, keeping your back straight, rather than bending directly at the waist. Seek medical care if your back pain becomes increasingly severe and is accompanied by: pain radiating to the buttock or back of the thigh, leg numbness or weakness, fever, or urinary symptoms (incontinence or retention), or if it does not improve at all with rest and position changes. These may indicate a disc problem, sacroiliac joint dysfunction, or a rare but serious spinal infection. | | | | | | |
| **Item / 项目** | | **Q1 / 问题1** | | **Q2 / 问题2** | | **Q3 / 问题3** |
| **Patient question (Chinese original) / 患者原始问题（中文）** | | 我产后总觉得腰酸背痛，是不是坐月子没坐好？ | | 抱孩子和喂奶时怎样保护腰背？ | | 如果腰痛越来越重，什么时候需要就医？ |
| **English translation of patient question / 患者问题英文翻译** | | I often feel lower back pain after birth. Is it because I did not follow postpartum confinement properly? | | How can I protect my lower back when holding the baby and breastfeeding? | | If my back pain is getting worse, when should I seek medical care? |
| **Model response (Chinese) / 模型回答（中文）** | | 不是。主要与韧带松弛、核心肌力减弱、姿势不当有关。 | | 使用哺乳枕、腰靠，屈膝下蹲，不弯腰。 | | 疼痛放射到腿部、伴麻木、无力或发热时，需就医。 |
| **English translation of model response / 模型回答英文翻译** | | No. It’s mainly due to ligament laxity, weak core muscles, and poor posture. | | Use a nursing pillow, back support, squat to pick up baby, and avoid bending. | | Seek care if pain radiates to leg, with numbness, weakness, or fever. |
| **Overall evaluation item / 整体评价项目** | | | **Score / 分数** | | **Comments / 备注** | |
| **Patient Satisfaction / 患者满意度** | | | 3 | |  | |
| **Patient Understandability / 患者可理解性** | | | 4 | |  | |
| **Patient Usefulness / 患者实用性** | | | 3 | |  | |
| **Clinician Correctness / 医生评分：正确性** | | | 4 | |  | |
| **Clinician Completeness / 医生评分：完整性** | | | 3 | |  | |
| **Clinician Readability / 医生评分：可读性** | | | 4 | |  | |
| **Clinician Helpfulness / 医生评分：帮助性** | | | 3 | |  | |
| **Clinician Safety / 医生评分：安全性** | | | 4 | |  | |

**Patient 27**

| **Patient ID / 患者编号** | **Group / 模型组别** | | | | | |
| --- | --- | --- | --- | --- | --- | --- |
| P27 | DeepSeek V3 | | | | | |
| **General model response or patient education material (Chinese) / 模型总体回答或患者教育材料（中文）** | | | | | | |
| 产后肚子仍然很大，可能的原因包括：子宫尚未完全恢复至孕前大小（产后6周左右基本恢复）、腹部脂肪堆积、以及腹直肌分离（Diastasis Recti Abdominis, DRA）。腹直肌分离是指孕期增大的子宫把腹壁两条纵向的腹直肌从中间的白线拉开，导致腹部松弛、突出，甚至在用力时出现中线隆起。您可以在家自测：仰卧屈膝，将手指横放在肚脐上下方，轻轻抬起头和肩膀，感受腹直肌中间的缝隙宽度。如果超过2指宽（约2厘米），且伴有腹部无力或腰背痛，建议产后42天复查时请医生或盆底康复治疗师评估。关于腹直肌分离能否自己做卷腹训练：绝对不可以。卷腹、仰卧起坐、平板支撑等传统腹肌训练会显著增加腹内压，反而加重腹直肌分离，甚至导致盆底功能障碍。产后腹部恢复应先从深层核心训练开始：腹式呼吸（吸气时腹部向外扩张，呼气时收缩腹部并感受腹直肌向中线靠拢）、盆底肌收缩、以及四点跪位的“鸟狗式”等低负荷训练。如果产后3-6个月腹部仍恢复不良，建议到医院进行专业的腹直肌康复评估和治疗（如手法复位、神经肌肉电刺激等）。 | | | | | | |
| **English translation of the Chinese general response / 中文总体回答英文翻译** | | | | | | |
| A still-large abdomen after birth may be due to: the uterus not yet fully returning to pre-pregnancy size (it usually recovers by around 6 weeks postpartum), abdominal fat accumulation, and diastasis recti abdominis (DRA). DRA refers to the separation of the two longitudinal rectus abdominis muscles along the midline linea alba due to the expanding uterus during pregnancy, resulting in a loose, protruding abdomen and even a midline ridge when tensing the muscles. You can perform a simple self-test at home: lie on your back with knees bent, place your fingers horizontally above and below the belly button, gently lift your head and shoulders, and feel the width of the gap between the two rectus muscles. If the gap is more than 2 finger-widths (approximately 2 cm), especially with abdominal weakness or back pain, ask your doctor or pelvic floor rehabilitation therapist to evaluate it at the 42-day postpartum check-up. Regarding whether you can do crunches or sit-ups for DRA: absolutely not. Crunches, sit-ups, planks, and other traditional abdominal exercises significantly increase intra-abdominal pressure, which can worsen DRA and even cause pelvic floor dysfunction. Postpartum abdominal recovery should start with deep core training: diaphragmatic breathing (abdomen expands on inhalation, contracts on exhalation, with a sensation of the rectus muscles moving toward the midline), pelvic floor contractions, and low-load exercises such as the quadruped “bird-dog” position. If the abdomen has not recovered well by 3 to 6 months postpartum, seek professional DRA rehabilitation assessment and treatment (such as manual reduction, neuromuscular electrical stimulation) at a hospital. | | | | | | |
| **Item / 项目** | | **Q1 / 问题1** | | **Q2 / 问题2** | | **Q3 / 问题3** |
| **Patient question (Chinese original) / 患者原始问题（中文）** | | 我产后肚子还是很大，是不是腹直肌分离？ | | 腹直肌分离可以自己做卷腹训练吗？ | | 产后腹部恢复不好，需要做康复评估吗？ |
| **English translation of patient question / 患者问题英文翻译** | | My abdomen is still large after birth. Could it be diastasis recti? | | Can I do sit-up or crunch exercises on my own for diastasis recti? | | If my abdomen does not recover well after birth, do I need rehabilitation assessment? |
| **Model response (Chinese) / 模型回答（中文）** | | 可能。建议42天复查时评估，不要自行做卷腹。 | | 不可以。卷腹会加重分离，应从腹式呼吸、盆底训练开始。 | | 建议做。可进行手法复位、电刺激等专业康复。 |
| **English translation of model response / 模型回答英文翻译** | | Possibly. Get assessed at 42-day check-up and avoid sit-ups. | | No, sit-ups worsen it. Start with diaphragmatic breathing and pelvic floor exercises. | | Yes, consider professional rehabilitation like manual therapy or electrical stimulation. |
| **Overall evaluation item / 整体评价项目** | | | **Score / 分数** | | **Comments / 备注** | |
| **Patient Satisfaction / 患者满意度** | | | 3 | |  | |
| **Patient Understandability / 患者可理解性** | | | 4 | |  | |
| **Patient Usefulness / 患者实用性** | | | 3 | |  | |
| **Clinician Correctness / 医生评分：正确性** | | | 4 | |  | |
| **Clinician Completeness / 医生评分：完整性** | | | 3 | |  | |
| **Clinician Readability / 医生评分：可读性** | | | 4 | |  | |
| **Clinician Helpfulness / 医生评分：帮助性** | | | 3 | |  | |
| **Clinician Safety / 医生评分：安全性** | | | 4 | |  | |

**Patient 28**

| **Patient ID / 患者编号** | **Group / 模型组别** | | | | | |
| --- | --- | --- | --- | --- | --- | --- |
| P28 | DeepSeek V3 | | | | | |
| **General model response or patient education material (Chinese) / 模型总体回答或患者教育材料（中文）** | | | | | | |
| 产后经常出汗，甚至睡觉时也出汗，在产后第一周内非常常见，这被称为“产后褥汗”，是正常的生理现象。原因是孕期您体内潴留了大量水分（血容量增加约40-50%），产后通过尿液和汗液的形式将这些多余水分排出体外，通常会持续1-2周，然后逐渐减轻。所以请您不要担心，这不代表身体虚弱或“出虚汗”。产后出汗多需要适当补充水分，建议每天饮水2000-2500毫升，可以喝温开水、清淡汤水或哺乳期适用的营养饮品。关于补盐：除非您在炎热环境中大量出汗，否则一般不需要额外补充盐分，正常饮食中的盐就足够了。过度补盐反而可能增加肾脏负担或引起水肿。如果出汗同时伴有明显心慌（心率明显增快）、极度乏力、体重不明原因显著下降、或者发热（体温≥38℃），则需要到医院检查，排除甲状腺功能亢进、感染或其它代谢性疾病。如果出汗持续超过4周仍无改善，也可以就诊内分泌科进一步评估。 | | | | | | |
| **English translation of the Chinese general response / 中文总体回答英文翻译** | | | | | | |
| Frequent sweating after birth, even during sleep, is very common in the first week postpartum. This is called postpartum diuresis and sweating, and it is a normal physiological phenomenon. The reason is that during pregnancy, your body retained a large amount of fluid (blood volume increased by approximately 40-50%). After birth, this excess fluid is eliminated through urine and sweat, typically lasting 1 to 2 weeks before gradually improving. Please do not worry about this; it does not indicate weakness or “pathological sweating.” With increased postpartum sweating, you need to adequately replenish fluids. It is recommended to drink 2000-2500 mL of water daily, including warm water, light soups, or nutritional drinks suitable for breastfeeding. Regarding salt supplementation: unless you are sweating profusely in a hot environment, additional salt is generally not needed. The salt in a normal diet is sufficient. Excessive salt intake may increase kidney burden or cause edema. If sweating is accompanied by significant palpitations (rapid heart rate), extreme weakness, unexplained significant weight loss, or fever (≥38°C), then you should see a doctor to rule out hyperthyroidism, infection, or other metabolic disorders. If sweating persists for more than 4 weeks without improvement, you may also visit an endocrinologist for further evaluation. | | | | | | |
| **Item / 项目** | | **Q1 / 问题1** | | **Q2 / 问题2** | | **Q3 / 问题3** |
| **Patient question (Chinese original) / 患者原始问题（中文）** | | 我产后经常出汗，睡觉也容易出汗，正常吗？ | | 产后出汗多需要特别补水或补盐吗？ | | 如果出汗同时心慌、乏力，需要检查吗？ |
| **English translation of patient question / 患者问题英文翻译** | | I sweat often after birth and also sweat easily during sleep. Is this normal? | | Do I need special water or salt supplementation if I sweat a lot after birth? | | If sweating occurs with palpitations and weakness, do I need an examination? |
| **Model response (Chinese) / 模型回答（中文）** | | 正常，这是产后褥汗，排出孕期多余水分，一般持续1-2周。 | | 需要补水（每天2000-2500ml），但不需要额外补盐。 | | 需要。排除甲亢、感染或代谢问题。 |
| **English translation of model response / 模型回答英文翻译** | | Normal. This is postpartum sweating to eliminate excess fluid, lasting 1-2 weeks. | | Hydrate well (2000-2500ml/day), but no extra salt needed. | | Yes, to rule out hyperthyroidism, infection, or metabolic disorders. |
| **Overall evaluation item / 整体评价项目** | | | **Score / 分数** | | **Comments / 备注** | |
| **Patient Satisfaction / 患者满意度** | | | 3 | |  | |
| **Patient Understandability / 患者可理解性** | | | 4 | |  | |
| **Patient Usefulness / 患者实用性** | | | 3 | |  | |
| **Clinician Correctness / 医生评分：正确性** | | | 4 | |  | |
| **Clinician Completeness / 医生评分：完整性** | | | 3 | |  | |
| **Clinician Readability / 医生评分：可读性** | | | 4 | |  | |
| **Clinician Helpfulness / 医生评分：帮助性** | | | 3 | |  | |
| **Clinician Safety / 医生评分：安全性** | | | 4 | |  | |

**Patient 29**

| **Patient ID / 患者编号** | **Group / 模型组别** | | | | | |
| --- | --- | --- | --- | --- | --- | --- |
| P29 | DeepSeek V3 | | | | | |
| **General model response or patient education material (Chinese) / 模型总体回答或患者教育材料（中文）** | | | | | | |
| 产后总觉得很累、没力气，在产后初期是常见的，因为分娩消耗巨大体力、产后睡眠被频繁打断、以及激素剧烈变化都会导致疲劳。但如果疲劳感非常突出，伴有头晕、站起来时眼前发黑、心慌、面色苍白、活动后气喘、注意力不集中、指甲变脆或口角开裂，则需要警惕产后贫血。产后贫血最常见的表现就是持续性的、无法通过休息改善的乏力，以及上述缺氧相关症状。建议您在产后42天复查时务必检查血常规，必要时提前去医院查。关于产后贫血是否需要复查血常规：是的，产后贫血患者应在医生指导下复查血常规，评估贫血纠正情况。通常口服补铁2-4周后复查，如果血红蛋白上升不理想，可能需要调整剂量或寻找其他原因。关于哺乳期贫血能否补铁：完全可以，而且非常必要。常用的口服补铁剂（如硫酸亚铁、富马酸亚铁、葡萄糖酸亚铁、多糖铁复合物等）在哺乳期是安全的，铁剂进入乳汁的量极少，不会对宝宝造成不良影响。为了增加铁的吸收率，建议与维生素C（如橙汁、猕猴桃）同服，避免与茶、咖啡、牛奶、钙片同服（会抑制吸收）。服用铁剂后大便变黑是正常现象，请不用担心。 | | | | | | |
| **English translation of the Chinese general response / 中文总体回答英文翻译** | | | | | | |
| Feeling very tired and weak after birth is common in the early postpartum period, because childbirth consumes enormous energy, sleep is frequently interrupted, and hormone levels change dramatically. However, if the fatigue is extreme and accompanied by dizziness, black spots before the eyes on standing, palpitations, pale skin, shortness of breath with activity, difficulty concentrating, brittle nails, or cracked mouth corners, then postpartum anemia should be suspected. The most common presentation of postpartum anemia is persistent fatigue that does not improve with rest, along with the above symptoms of oxygen deprivation. It is recommended that you have a complete blood count at the 42-day postpartum check-up, or earlier if needed. Regarding whether postpartum anemia requires repeat blood count: yes. Patients with postpartum anemia should follow up with a blood count under medical guidance to assess improvement. Typically, it is repeated 2 to 4 weeks after starting oral iron therapy. If hemoglobin does not rise as expected, the dose may need adjustment or other causes should be investigated. Regarding whether iron can be taken for anemia during breastfeeding: absolutely yes, and it is very necessary. Common oral iron supplements (ferrous sulfate, ferrous fumarate, ferrous gluconate, iron polysaccharide complex, etc.) are safe during breastfeeding. Iron enters breastmilk in very small amounts and does not harm the baby. To increase iron absorption, take it with vitamin C (such as orange juice or kiwi) and avoid taking it with tea, coffee, milk, or calcium supplements (which inhibit absorption). Blackening of stools is a normal effect of iron supplements and is not a concern. | | | | | | |
| **Item / 项目** | | **Q1 / 问题1** | | **Q2 / 问题2** | | **Q3 / 问题3** |
| **Patient question (Chinese original) / 患者原始问题（中文）** | | 我产后总觉得很累、没力气，是正常恢复过程吗？ | | 产后贫血会有哪些表现，需要复查血常规吗？ | | 哺乳期贫血可以补铁吗，会不会影响宝宝？ |
| **English translation of patient question / 患者问题英文翻译** | | I often feel very tired and weak after birth. Is this part of normal recovery? | | What symptoms can postpartum anemia cause, and do I need to recheck a complete blood count? | | Can I take iron for anemia during breastfeeding, and will it affect the baby? |
| **Model response (Chinese) / 模型回答（中文）** | | 如果持续不缓解，伴头晕、心慌、面色苍白，需警惕产后贫血。 | | 乏力、头晕、气喘、注意力不集中等，需要复查血常规。 | | 可以。常用口服铁剂对哺乳是安全的，不影响宝宝。 |
| **English translation of model response / 模型回答英文翻译** | | If persistent with dizziness, palpitations, or pallor, suspect postpartum anemia. | | Fatigue, dizziness, shortness of breath, poor concentration. Yes, need CBC. | | Yes. Oral iron supplements are safe during breastfeeding. |
| **Overall evaluation item / 整体评价项目** | | | **Score / 分数** | | **Comments / 备注** | |
| **Patient Satisfaction / 患者满意度** | | | 3 | |  | |
| **Patient Understandability / 患者可理解性** | | | 4 | |  | |
| **Patient Usefulness / 患者实用性** | | | 3 | |  | |
| **Clinician Correctness / 医生评分：正确性** | | | 4 | |  | |
| **Clinician Completeness / 医生评分：完整性** | | | 3 | |  | |
| **Clinician Readability / 医生评分：可读性** | | | 4 | |  | |
| **Clinician Helpfulness / 医生评分：帮助性** | | | 3 | |  | |
| **Clinician Safety / 医生评分：安全性** | | | 4 | |  | |

**Patient 30**

| **Patient ID / 患者编号** | **Group / 模型组别** | | | | | |
| --- | --- | --- | --- | --- | --- | --- |
| P30 | DeepSeek V3 | | | | | |
| **General model response or patient education material (Chinese) / 模型总体回答或患者教育材料（中文）** | | | | | | |
| 即使产后伤口已经不疼了，仍然建议您每天观察伤口情况。因为疼痛消失只是伤口愈合的一个方面，不代表没有潜在问题。观察内容包括：伤口颜色（是否比周围皮肤更红）、有无渗液或渗血（渗液是什么颜色、量多少、有无气味）、伤口边缘是否对齐闭合、有无异常隆起或凹陷、以及有无硬结。会阴伤口（侧切或撕裂伤）通常在产后2-4周基本愈合，但深部组织的完全修复可能需要6-8周甚至更长。剖宫产腹部切口的皮肤表面愈合较快（通常7-10天），但皮下脂肪、筋膜和肌肉的完全修复需要3-6个月。因此，术后数月内切口局部有轻微硬结、牵拉感、偶尔针刺样感觉，在大多数情况下是正常的瘢痕修复过程，尤其是在天气变化、活动后或姿势改变时更明显。但如果硬结逐渐增大、出现红肿热痛、渗液增多或有异味，则可能是瘢痕增生异常或感染，需要就医。另外，如果瘢痕明显隆起超过皮肤表面、呈红色、伴有明显瘙痒或疼痛，可能是瘢痕疙瘩或增生性瘢痕，可以咨询皮肤科或整形外科进一步处理。 | | | | | | |
| **English translation of the Chinese general response / 中文总体回答英文翻译** | | | | | | |
| Even if your postpartum wound no longer hurts, you are still advised to observe it daily. The disappearance of pain is only one aspect of wound healing and does not mean there are no underlying issues. Observation should include: wound color (whether it is redder than surrounding skin), presence of drainage or bleeding (color, amount, odor), whether the wound edges are well approximated, whether there is abnormal bulging or depression, and whether there are hard nodules. Perineal wounds (episiotomy or tears) usually heal substantially within 2 to 4 weeks postpartum, but complete deep tissue repair may take 6 to 8 weeks or longer. The skin surface of a cesarean abdominal incision heals relatively quickly (usually 7 to 10 days), but complete repair of subcutaneous fat, fascia, and muscle takes 3 to 6 months. Therefore, mild hardness, pulling sensation, and occasional needle-like sensations around the incision for several months postpartum are mostly normal scar healing processes, especially noticeable with weather changes, activity, or position changes. However, if the hard nodule gradually enlarges, becomes red, warm, painful, or has increased or foul-smelling drainage, this may indicate abnormal scar proliferation or infection requiring medical attention. Additionally, if the scar is significantly raised above the skin surface, red, and accompanied by marked itching or pain, it may be a keloid or hypertrophic scar; you may consult a dermatologist or plastic surgeon for further management. | | | | | | |
| **Item / 项目** | | **Q1 / 问题1** | | **Q2 / 问题2** | | **Q3 / 问题3** |
| **Patient question (Chinese original) / 患者原始问题（中文）** | | 我产后伤口已经不疼了，还需要每天观察吗？ | | 会阴或腹部伤口多久能完全恢复？ | | 如果伤口处出现硬结或牵拉感，正常吗？ |
| **English translation of patient question / 患者问题英文翻译** | | My postpartum wound no longer hurts. Do I still need to observe it every day? | | How long does it take for a perineal or abdominal wound to heal completely? | | If there is a hard lump or pulling feeling around the wound, is that normal? |
| **Model response (Chinese) / 模型回答（中文）** | | 需要。观察颜色、渗液、硬结，早期发现异常。 | | 会阴2-4周，剖宫产皮肤7-10天，深层组织需3-6个月。 | | 多数正常，是瘢痕修复过程。但硬结增大、红肿热痛需就医。 |
| **English translation of model response / 模型回答英文翻译** | | Yes. Check color, discharge, lumps daily to catch problems early. | | Perineal: 2-4 weeks; C-section skin: 7-10 days; deep layers: 3-6 months. | | Usually normal (scar healing). But see a doctor if it enlarges or becomes red/warm/painful. |
| **Overall evaluation item / 整体评价项目** | | | **Score / 分数** | | **Comments / 备注** | |
| **Patient Satisfaction / 患者满意度** | | | 3 | |  | |
| **Patient Understandability / 患者可理解性** | | | 4 | |  | |
| **Patient Usefulness / 患者实用性** | | | 3 | |  | |
| **Clinician Correctness / 医生评分：正确性** | | | 4 | |  | |
| **Clinician Completeness / 医生评分：完整性** | | | 3 | |  | |
| **Clinician Readability / 医生评分：可读性** | | | 4 | |  | |
| **Clinician Helpfulness / 医生评分：帮助性** | | | 3 | |  | |
| **Clinician Safety / 医生评分：安全性** | | | 4 | |  | |

**Patient 31**

| **Patient ID / 患者编号** | **Group / 模型组别** | | | | | |
| --- | --- | --- | --- | --- | --- | --- |
| P31 | DeepSeek V3 | | | | | |
| **General model response or patient education material (Chinese) / 模型总体回答或患者教育材料（中文）** | | | | | | |
| 产后恶露快干净了（变成黄色或白色）又突然变红，不一定都是异常的。常见原因包括：哺乳后因催产素释放导致子宫收缩、排出少量宫腔内残留的陈旧积血；或者您活动量突然增加（如久站、提重物、长时间抱孩子）导致子宫创面少量渗血。如果只是短暂变红，量少，没有腹痛、发热、臭味，并且很快又转为正常颜色，一般可以暂时观察，适当减少活动量。但如果颜色反复变成鲜红色、出血量超过月经量、需要每小时更换卫生巾、伴有大血块（大于拇指）、持续超过1周不转为正常、或者伴有下腹痛、发热、恶露臭味，则需要到医院做妇科B超检查，排除宫腔残留物（如胎盘、胎膜残留）、子宫复旧不良或产褥感染。另外，恶露持续超过4-6周还不干净，无论量多少，都建议在产后42天复查时做B超评估。长期不干净的恶露可能提示子宫内膜炎、宫腔残留或剖宫产切口愈合不良（切口憩室）等问题。 | | | | | | |
| **English translation of the Chinese general response / 中文总体回答英文翻译** | | | | | | |
| Lochia that has almost cleared (turned yellow or white) and then suddenly becomes red again is not always abnormal. Common causes include: after breastfeeding, oxytocin release causes uterine contractions, expelling a small amount of old retained blood; or a sudden increase in your activity level (prolonged standing, heavy lifting, long periods of holding the baby) causes a small amount of oozing from the uterine wound. If the return to red is brief, the amount is small, without abdominal pain, fever, foul odor, and it quickly returns to normal color again, you may simply observe and reduce activity appropriately. However, if the color repeatedly turns bright red, the bleeding is heavier than a menstrual period, requires changing a pad every hour, includes large clots (larger than a thumb), persists for more than 1 week without returning to normal, or is accompanied by lower abdominal pain, fever, or foul odor, then you need a pelvic ultrasound to rule out retained products of conception, poor uterine involution, or puerperal infection. Additionally, if lochia persists beyond 4 to 6 weeks without clearing, regardless of the amount, it is recommended to have a pelvic ultrasound at the 42-day postpartum check-up. Persistently prolonged lochia may suggest endometritis, retained products, or poor cesarean scar healing (such as an isthmocele). | | | | | | |
| **Item / 项目** | | **Q1 / 问题1** | | **Q2 / 问题2** | | **Q3 / 问题3** |
| **Patient question (Chinese original) / 患者原始问题（中文）** | | 我产后恶露快没有了又突然变红，是正常的吗？ | | 哺乳或活动后恶露颜色加深，需要减少活动吗？ | | 恶露持续超过一个月还没干净，需要检查吗？ |
| **English translation of patient question / 患者问题英文翻译** | | My lochia almost stopped but suddenly became red again. Is this normal? | | If lochia becomes darker after breastfeeding or activity, should I reduce activity? | | Do I need an examination if lochia lasts more than one month and still has not cleared? |
| **Model response (Chinese) / 模型回答（中文）** | | 如果量少、无腹痛发热，可能正常；但反复变红或量多需B超。 | | 可以适当减少活动，如持续增多则需就医。 | | 需要。做B超排除宫腔残留或子宫复旧不良。 |
| **English translation of model response / 模型回答英文翻译** | | If small amount, no pain/fever, maybe normal. Recurrence or heavy bleeding needs ultrasound. | | You can reduce activity temporarily. If bleeding increases, seek care. | | Yes, ultrasound to rule out retained products or poor involution. |
| **Overall evaluation item / 整体评价项目** | | | **Score / 分数** | | **Comments / 备注** | |
| **Patient Satisfaction / 患者满意度** | | | 3 | |  | |
| **Patient Understandability / 患者可理解性** | | | 4 | |  | |
| **Patient Usefulness / 患者实用性** | | | 3 | |  | |
| **Clinician Correctness / 医生评分：正确性** | | | 4 | |  | |
| **Clinician Completeness / 医生评分：完整性** | | | 3 | |  | |
| **Clinician Readability / 医生评分：可读性** | | | 4 | |  | |
| **Clinician Helpfulness / 医生评分：帮助性** | | | 3 | |  | |
| **Clinician Safety / 医生评分：安全性** | | | 4 | |  | |

**Patient 32**

| **Patient ID / 患者编号** | **Group / 模型组别** | | | | | |
| --- | --- | --- | --- | --- | --- | --- |
| P32 | DeepSeek V3 | | | | | |
| **General model response or patient education material (Chinese) / 模型总体回答或患者教育材料（中文）** | | | | | | |
| 产后乳汁不多，绝不代表您不能继续母乳喂养。产后早期（尤其是前3-5天）初乳量本身就很少，但完全能满足新生儿的营养需求，因为初乳浓缩了高密度的免疫物质和营养。乳汁量会在产后3-5天左右显著增加，这个过程称为“奶阵启动”或“下奶”。判断宝宝有没有吃饱的标准不是看您感觉奶多不多，而是观察宝宝的客观表现：1）每天有6片以上沉甸甸的湿尿布（不是只有一点点湿）；2）每天有2-5次黄色糊状或稀糊状大便；3）体重增长符合生长曲线（出生后前3天生理性体重下降不超过7-10%，之后每周增长约150-200克）；4）喂奶时能看到宝宝有明显的吞咽动作（深而慢的吸吮，伴随“咕咚”声）。关于混合喂养是否会影响追奶：混合喂养本身不一定影响追奶，但关键在于不要因为添加了配方奶就减少了吸吮频率。追奶的核心是“排得多、产得多”——只要您保证每天8-12次以上的有效排乳（亲喂或使用医用级吸奶器），即使补充少量配方奶，也可以逐渐追奶成功。建议您咨询哺乳指导或产科护士，帮助您评估哺乳姿势和含接，制定个体化的追奶计划。 | | | | | | |
| **English translation of the Chinese general response / 中文总体回答英文翻译** | | | | | | |
| Having a low milk supply after birth absolutely does not mean you cannot continue breastfeeding. In the early postpartum period (especially the first 3-5 days), colostrum volume is naturally small, but it fully meets the newborn’s nutritional needs because colostrum is concentrated with high-density immune factors and nutrients. Milk volume will increase significantly around day 3 to 5 postpartum, a process called lactogenesis II or “milk coming in.” The criteria for judging whether the baby is getting enough milk are not based on how much milk you feel you have, but on the baby’s objective signs: 1) At least 6 heavy wet diapers per day (not just slightly damp); 2) 2-5 yellow pasty or loose pasty stools per day; 3) Weight gain follows the growth curve (physiological weight loss in the first 3 days no more than 7-10%, then gaining approximately 150-200 grams per week); 4) During feeding, you can clearly see the baby swallowing (deep, slow sucking with audible gulps). Regarding whether mixed feeding affects the ability to increase milk supply later: mixed feeding does not necessarily prevent you from increasing milk supply, but the key is not to reduce sucking frequency just because you have added formula. The core principle of increasing milk supply is “more removal, more production.” As long as you ensure 8-12 or more effective milk removals per day (via direct breastfeeding or a hospital-grade pump), even with a small amount of formula supplementation, you can gradually increase your milk supply. It is recommended that you consult a lactation specialist or obstetric nurse to evaluate your feeding position and latch, and to develop an individualized plan to increase milk supply. | | | | | | |
| **Item / 项目** | | **Q1 / 问题1** | | **Q2 / 问题2** | | **Q3 / 问题3** |
| **Patient question (Chinese original) / 患者原始问题（中文）** | | 我产后乳汁不多，是不是就不能继续母乳喂养了？ | | 怎样判断宝宝有没有吃饱？ | | 混合喂养会不会影响以后追奶？ |
| **English translation of patient question / 患者问题英文翻译** | | I do not have much breast milk after birth. Does that mean I cannot continue breastfeeding? | | How can I tell whether my baby is getting enough milk? | | Will mixed feeding affect my ability to increase milk supply later? |
| **Model response (Chinese) / 模型回答（中文）** | | 不是。产后早期初乳量少是正常的，应继续频繁吸吮。 | | 看尿量（每天6片以上湿尿布）、大便、体重增长和吞咽动作。 | | 不会，关键是不减少吸吮频率，保证每天8-12次以上。 |
| **English translation of model response / 模型回答英文翻译** | | No, early low volume is normal. Keep feeding frequently. | | Check urine output (6+ wet diapers/day), stools, weight gain, and swallowing. | | No, as long as you maintain 8-12+ feeding/pumping sessions per day. |
| **Overall evaluation item / 整体评价项目** | | | **Score / 分数** | | **Comments / 备注** | |
| **Patient Satisfaction / 患者满意度** | | | 3 | |  | |
| **Patient Understandability / 患者可理解性** | | | 4 | |  | |
| **Patient Usefulness / 患者实用性** | | | 3 | |  | |
| **Clinician Correctness / 医生评分：正确性** | | | 4 | |  | |
| **Clinician Completeness / 医生评分：完整性** | | | 3 | |  | |
| **Clinician Readability / 医生评分：可读性** | | | 4 | |  | |
| **Clinician Helpfulness / 医生评分：帮助性** | | | 3 | |  | |
| **Clinician Safety / 医生评分：安全性** | | | 4 | |  | |

**Patient 33**

| **Patient ID / 患者编号** | **Group / 模型组别** | | | | | |
| --- | --- | --- | --- | --- | --- | --- |
| P33 | DeepSeek V3 | | | | | |
| **General model response or patient education material (Chinese) / 模型总体回答或患者教育材料（中文）** | | | | | | |
| 宝宝哺乳时总是含不住乳头，这是初产妇非常常见的问题，绝大多数与哺乳姿势不当或宝宝含接技巧不成熟有关。正确的解决方案是调整姿势，而不是硬塞或放弃母乳喂养。标准含接的步骤：1）将宝宝抱到乳房高度，宝宝的身体应该贴近您的身体，肚子对肚子，宝宝的鼻子对着您的乳头；2）用乳头轻轻触碰宝宝的上唇或鼻子，刺激宝宝张大嘴巴；3）当宝宝嘴张到最大时（像打哈欠一样），迅速将宝宝的头靠近乳房，让宝宝的下巴先接触乳晕下方，然后含住大部分乳晕（不仅是乳头），上方的乳晕比下方露出更多；4）含接正确时，您能看到宝宝下颌运动、耳前肌肉活动、能听到吞咽声，而且喂奶后乳头不扁、不疼。乳房一边奶多一边奶少是非常常见的现象，绝大多数情况下是正常的，不需要特殊处理。您可以每次从奶少的一侧开始喂奶，以刺激该侧泌乳，逐渐缩小差距。关于宝宝吃奶后乳房还有胀感是否需要排空：一般情况下，不建议每次哺乳后都完全排空乳房，因为过度排空会刺激产生更多的乳汁，反而容易导致奶量过多、反复堵奶。只需挤到乳房感觉不胀、舒适为止即可。如果您有过反复堵奶或乳腺炎史，则需要更谨慎。 | | | | | | |
| **English translation of the Chinese general response / 中文总体回答英文翻译** | | | | | | |
| If the baby often cannot latch onto the nipple during breastfeeding, this is a very common problem for first-time mothers, most often related to improper positioning or immature latching skills. The correct solution is to adjust the position, not to force it or give up breastfeeding. Steps for a correct latch: 1) Bring the baby to breast height. The baby’s body should be close to yours, belly to belly, with the baby’s nose opposite your nipple; 2) Gently touch the baby’s upper lip or nose with your nipple to stimulate the baby to open wide; 3) When the baby’s mouth is wide open (like a yawn), quickly bring the baby’s head to the breast, so that the baby’s chin touches the lower areola first, then the baby takes in most of the areola (not just the nipple), with more areola visible above than below; 4) When the latch is correct, you will see jaw movement, preauricular muscle activity, hear swallowing, and the nipple will not be flattened or painful after feeding. One breast producing more milk than the other is very common and is usually normal, requiring no special treatment. You can start each feed on the lower-supply side to stimulate that breast and gradually reduce the difference. Regarding whether you need to empty the breast completely if it still feels full after feeding: generally, complete emptying after every feed is not recommended, as over-emptying can stimulate even more milk production, leading to oversupply and recurrent blocked ducts. Instead, express only until the breast feels comfortable and not engorged. If you have a history of recurrent blocked ducts or mastitis, you need to be more cautious. | | | | | | |
| **Item / 项目** | | **Q1 / 问题1** | | **Q2 / 问题2** | | **Q3 / 问题3** |
| **Patient question (Chinese original) / 患者原始问题（中文）** | | 我哺乳时宝宝总是含不住乳头，应该怎么办？ | | 乳房一边奶多一边奶少，正常吗？ | | 宝宝吃奶后我乳房还有胀感，需要排空吗？ |
| **English translation of patient question / 患者问题英文翻译** | | My baby often cannot latch onto the nipple during breastfeeding. What should I do? | | Is it normal for one breast to produce more milk than the other? | | If my breast still feels full after the baby feeds, do I need to empty it completely? |
| **Model response (Chinese) / 模型回答（中文）** | | 调整姿势，让宝宝张大嘴，含住大部分乳晕而不是仅乳头。 | | 正常。每次从奶少的一侧开始喂。 | | 排到不胀、舒适即可，过度排空会导致奶量过多。 |
| **English translation of model response / 模型回答英文翻译** | | Adjust position. Baby should take most of the areola, not just the nipple. | | Normal. Start each feed on the lower-supply side. | | Express until comfortable, not fully empty, to avoid oversupply. |
| **Overall evaluation item / 整体评价项目** | | | **Score / 分数** | | **Comments / 备注** | |
| **Patient Satisfaction / 患者满意度** | | | 3 | |  | |
| **Patient Understandability / 患者可理解性** | | | 4 | |  | |
| **Patient Usefulness / 患者实用性** | | | 3 | |  | |
| **Clinician Correctness / 医生评分：正确性** | | | 4 | |  | |
| **Clinician Completeness / 医生评分：完整性** | | | 3 | |  | |
| **Clinician Readability / 医生评分：可读性** | | | 4 | |  | |
| **Clinician Helpfulness / 医生评分：帮助性** | | | 3 | |  | |
| **Clinician Safety / 医生评分：安全性** | | | 4 | |  | |

**Patient 34**

| **Patient ID / 患者编号** | **Group / 模型组别** | | | | | |
| --- | --- | --- | --- | --- | --- | --- |
| P34 | DeepSeek V3 | | | | | |
| **General model response or patient education material (Chinese) / 模型总体回答或患者教育材料（中文）** | | | | | | |
| 产后阴道有下坠感（感觉有东西要掉出来），不一定就是子宫脱垂。轻度盆腔器官脱垂可能完全没有下坠感，而有明显下坠感的人也可能只是盆底肌松弛或阴道壁膨出，而非真正的子宫脱垂。请您先不要过度紧张。站久了下坠感明显加重，这恰恰提示盆底支撑结构力量不足。应对措施不是长期卧床休息（长期卧床反而会加重肌肉萎缩），而是：1）避免久站、久蹲、提重物（超过5公斤）、慢性咳嗽和便秘，这些都会增加腹内压；2）尝试凯格尔运动加强盆底肌；3）产后42天复查时主动要求医生做盆腔器官脱垂定量评估。关于是否需要卧床休息：对于轻中度盆腔器官脱垂，不推荐长期卧床，适当的活动和正确的盆底训练更有益。只有在极重度脱垂或脱垂术后早期，医生才会建议卧床。关于盆腔脏器脱垂能否通过康复改善：绝大多数轻中度脱垂可以通过盆底康复治疗（生物反馈、电刺激、盆底肌训练）显著改善症状，甚至使脱垂程度减轻。重度脱垂或康复效果不佳者，可考虑手术治疗（如盆底重建术）。请不要因为害怕脱垂而不敢活动、不敢锻炼，科学的康复才是正确的选择。 | | | | | | |
| **English translation of the Chinese general response / 中文总体回答英文翻译** | | | | | | |
| A sensation of vaginal heaviness after birth (feeling like something is falling out) does not necessarily mean uterine prolapse. Mild pelvic organ prolapse may have no sensation of heaviness at all, while someone with significant heaviness may only have pelvic floor muscle weakness or vaginal wall bulging, not true uterine prolapse. Please do not be overly anxious. The fact that heaviness worsens significantly after prolonged standing actually indicates insufficient strength of the pelvic floor support structures. The solution is not prolonged bed rest (which worsens muscle atrophy), but rather: 1) Avoid prolonged standing, prolonged squatting, heavy lifting (over 5 kg), chronic coughing, and constipation — all of which increase intra-abdominal pressure; 2) Perform Kegel exercises to strengthen the pelvic floor muscles; 3) At the 42-day postpartum check-up, proactively ask your doctor to perform a quantitative assessment of pelvic organ prolapse. Regarding whether bed rest is needed: For mild to moderate pelvic organ prolapse, prolonged bed rest is not recommended. Moderate activity and correct pelvic floor training are more beneficial. Only with very severe prolapse or early after prolapse surgery would a doctor recommend bed rest. Regarding whether pelvic organ prolapse can improve with rehabilitation: The vast majority of mild to moderate prolapse can achieve significant symptom improvement and even a reduction in prolapse stage with pelvic floor rehabilitation (biofeedback, electrical stimulation, pelvic floor muscle training). Severe prolapse or those with poor response to rehabilitation may consider surgical treatment (such as pelvic floor reconstruction). Please do not avoid activity or exercise for fear of prolapse; scientific rehabilitation is the correct approach. | | | | | | |
| **Item / 项目** | | **Q1 / 问题1** | | **Q2 / 问题2** | | **Q3 / 问题3** |
| **Patient question (Chinese original) / 患者原始问题（中文）** | | 我产后阴道有下坠感，是不是子宫脱垂？ | | 站久了下坠感明显，需要卧床休息吗？ | | 产后盆腔脏器脱垂可以通过康复改善吗？ |
| **English translation of patient question / 患者问题英文翻译** | | I feel a vaginal heaviness after birth. Could it be uterine prolapse? | | If the heaviness is worse after standing for a long time, do I need bed rest? | | Can postpartum pelvic organ prolapse improve with rehabilitation? |
| **Model response (Chinese) / 模型回答（中文）** | | 不一定，也可能是盆底肌松弛或阴道壁膨出，需医生评估。 | | 不需要长期卧床。应做凯格尔运动，避免久站久蹲。 | | 绝大多数轻中度脱垂可通过盆底康复显著改善。 |
| **English translation of model response / 模型回答英文翻译** | | Not necessarily; could be pelvic floor weakness or vaginal wall bulging. Get assessed. | | No prolonged bed rest. Do Kegel exercises and avoid prolonged standing/squatting. | | Yes, most mild to moderate prolapse improves with pelvic floor rehabilitation. |
| **Overall evaluation item / 整体评价项目** | | | **Score / 分数** | | **Comments / 备注** | |
| **Patient Satisfaction / 患者满意度** | | | 3 | |  | |
| **Patient Understandability / 患者可理解性** | | | 4 | |  | |
| **Patient Usefulness / 患者实用性** | | | 3 | |  | |
| **Clinician Correctness / 医生评分：正确性** | | | 4 | |  | |
| **Clinician Completeness / 医生评分：完整性** | | | 3 | |  | |
| **Clinician Readability / 医生评分：可读性** | | | 4 | |  | |
| **Clinician Helpfulness / 医生评分：帮助性** | | | 3 | |  | |
| **Clinician Safety / 医生评分：安全性** | | | 4 | |  | |

**Patient 35**

| **Patient ID / 患者编号** | **Group / 模型组别** | | | | | |
| --- | --- | --- | --- | --- | --- | --- |
| P35 | DeepSeek V3 | | | | | |
| **General model response or patient education material (Chinese) / 模型总体回答或患者教育材料（中文）** | | | | | | |
| 产后同房前感到紧张、担心疼痛，这是非常普遍且完全正常的心理反应，尤其是在初产妇或经历过分娩创伤、会阴侧切或撕裂的女性中。请您和伴侣充分沟通，告诉他您的担忧，好的伴侣会理解并配合您。建议采取以下措施：1）选择身体状态好、情绪放松的时间，不要疲劳或匆忙时尝试；2）确保恶露已经完全干净、伤口愈合良好（通常产后42天复查后）；3）充分前戏，至少15-20分钟，让身体自然产生润滑；4）使用水溶性润滑剂，哺乳期因雌激素水平低，阴道干涩非常常见，润滑剂可以显著减轻不适和疼痛；5）采取女士可控的体位，如女上位或侧卧位，这样您可以控制进入的深度和速度；6）从非插入式亲密接触开始，逐步过渡。关于哺乳期阴道干涩：这是完全正常的生理现象，因为哺乳期高水平的催乳素抑制了雌激素分泌，导致阴道黏膜变薄、弹性下降、分泌物减少。这不是“病”，也不需要特殊治疗，使用润滑剂即可。如果同房后出现明显疼痛、鲜红色出血、异常分泌物（黄色、绿色、有臭味）或持续数小时的不适，请及时就医，排除伤口裂开、感染或其他问题。 | | | | | | |
| **English translation of the Chinese general response / 中文总体回答英文翻译** | | | | | | |
| Feeling nervous and worried about pain before resuming sexual activity after birth is very common and completely normal, especially in first-time mothers or those who experienced birth trauma, episiotomy, or lacerations. Communicate openly with your partner, tell them about your concerns, and a good partner will understand and cooperate with you. Recommended measures: 1) Choose a time when you are physically well and emotionally relaxed — do not attempt when tired or in a hurry; 2) Ensure lochia has completely cleared and the wound has healed well (usually after the 42-day postpartum check-up); 3) Have adequate foreplay, at least 15-20 minutes, to allow natural lubrication; 4) Use a water-based lubricant. Due to low estrogen levels during breastfeeding, vaginal dryness is very common, and lubricant can significantly reduce discomfort and pain; 5) Adopt positions that you can control, such as woman-on-top or side-lying, so you can control the depth and speed of penetration; 6) Start with non-penetrative intimate contact and gradually transition. Regarding vaginal dryness during breastfeeding: this is completely normal physiology. High prolactin levels during breastfeeding suppress estrogen secretion, leading to thinner vaginal mucosa, decreased elasticity, and reduced secretions. This is not a “disease” and requires no specific treatment other than lubricant. If you experience significant pain, bright red bleeding, abnormal discharge (yellow, green, foul-smelling), or persistent discomfort for hours after sex, seek medical attention to rule out wound dehiscence, infection, or other issues. | | | | | | |
| **Item / 项目** | | **Q1 / 问题1** | | **Q2 / 问题2** | | **Q3 / 问题3** |
| **Patient question (Chinese original) / 患者原始问题（中文）** | | 我产后同房前很紧张，担心疼痛，怎么办？ | | 哺乳期阴道干涩是正常的吗？ | | 如果同房后出血或疼痛明显，需要就医吗？ |
| **English translation of patient question / 患者问题英文翻译** | | I feel very nervous before having sex after birth because I worry it will hurt. What should I do? | | Is vaginal dryness during breastfeeding normal? | | If I bleed or have obvious pain after sex, do I need medical care? |
| **Model response (Chinese) / 模型回答（中文）** | | 正常。建议充分前戏、使用润滑剂、采取可控体位。 | | 正常，因哺乳期雌激素水平低，使用水溶性润滑剂即可。 | | 需要。排除伤口裂开、感染或其他问题。 |
| **English translation of model response / 模型回答英文翻译** | | Normal. Use foreplay, lubricant, and woman-controlled positions. | | Yes, due to low estrogen. Water-based lubricant helps. | | Yes, to rule out wound dehiscence, infection, or other issues. |
| **Overall evaluation item / 整体评价项目** | | | **Score / 分数** | | **Comments / 备注** | |
| **Patient Satisfaction / 患者满意度** | | | 3 | |  | |
| **Patient Understandability / 患者可理解性** | | | 4 | |  | |
| **Patient Usefulness / 患者实用性** | | | 3 | |  | |
| **Clinician Correctness / 医生评分：正确性** | | | 4 | |  | |
| **Clinician Completeness / 医生评分：完整性** | | | 3 | |  | |
| **Clinician Readability / 医生评分：可读性** | | | 4 | |  | |
| **Clinician Helpfulness / 医生评分：帮助性** | | | 3 | |  | |
| **Clinician Safety / 医生评分：安全性** | | | 4 | |  | |

**Patient 36**

| **Patient ID / 患者编号** | **Group / 模型组别** | | | | | |
| --- | --- | --- | --- | --- | --- | --- |
| P36 | DeepSeek V3 | | | | | |
| **General model response or patient education material (Chinese) / 模型总体回答或患者教育材料（中文）** | | | | | | |
| 产后记忆力变差、注意力不集中，是绝大多数产妇都会经历的“妈妈脑”或“产后脑雾”现象，是完全正常的。主要原因包括：睡眠剥夺（照顾新生儿导致连续睡眠很少超过3-4小时）、激素剧烈波动（尤其是雌激素和催产素的变化）、以及身体和心理压力。这并不是您的“脑子变笨了”，也不是永久性的，通常在产后几个月到一年内随着睡眠改善而逐渐恢复。长期睡眠不足确实会显著影响情绪（更容易烦躁、焦虑、抑郁）、免疫系统（更容易感冒或感染）、以及身体恢复（伤口愈合减慢、血压波动）。因此，保证休息是恢复的核心。家人帮助产妇缓解照护压力的具体方法：1）夜间分担：爸爸或长辈负责换尿布、拍嗝、哄睡，只在需要喂奶时才叫醒妈妈，让妈妈能连续睡3-4小时；2）白天接手：家人每天至少接手1-2小时，让妈妈可以安心洗澡、吃饭、小睡或出门透透气；3）家务分担：不要期望妈妈同时照顾宝宝和做全部家务，家人主动承担做饭、打扫、洗衣等；4）情绪支持：不评价、不指责、多倾听，主动询问“你需要什么帮助”而不是“你怎么还做不好”。 | | | | | | |
| **English translation of the Chinese general response / 中文总体回答英文翻译** | | | | | | |
| Poor memory and reduced concentration after birth — often called “mommy brain” or postpartum brain fog — is experienced by the vast majority of new mothers and is completely normal. The main causes include: sleep deprivation (caring for a newborn results in very few consecutive sleep periods longer than 3-4 hours), dramatic hormonal fluctuations (especially changes in estrogen and oxytocin), and physical and psychological stress. This does not mean your brain is “deteriorating,” nor is it permanent. It typically gradually improves over the first several months to a year as sleep improves. Long-term sleep deprivation does significantly affect mood (increased irritability, anxiety, depression), the immune system (increased susceptibility to colds or infections), and physical recovery (slower wound healing, blood pressure fluctuations). Therefore, ensuring rest is central to recovery. Specific ways family members can help reduce the mother’s caregiving pressure: 1) Nighttime sharing: the partner or grandparents change diapers, burp, and settle the baby, waking the mother only for feeding, so she can get 3-4 hours of consecutive sleep; 2) Daytime relief: family members take over for at least 1-2 hours each day, allowing the mother to shower, eat, nap, or go outside without the baby; 3) Household sharing: do not expect the mother to care for the baby and do all housework; family members take initiative to cook, clean, do laundry, etc.; 4) Emotional support: avoid criticism or blame, listen more, and proactively ask “What help do you need?” rather than “Why aren’t you doing better?” | | | | | | |
| **Item / 项目** | | **Q1 / 问题1** | | **Q2 / 问题2** | | **Q3 / 问题3** |
| **Patient question (Chinese original) / 患者原始问题（中文）** | | 我产后记忆力变差、注意力不集中，是不是正常现象？ | | 长期睡眠不足会不会影响情绪和身体恢复？ | | 家人应该怎么帮助产妇缓解照护压力？ |
| **English translation of patient question / 患者问题英文翻译** | | My memory is worse and I cannot concentrate after birth. Is this normal? | | Can long-term sleep deprivation affect mood and physical recovery? | | How should family members help the mother reduce caregiving pressure? |
| **Model response (Chinese) / 模型回答（中文）** | | 正常，称为“妈妈脑”，与睡眠不足、激素变化有关。 | | 会。影响情绪、免疫力和恢复，应争取家人分担夜奶。 | | 分担夜奶、白天接手、做家务、情绪支持。 |
| **English translation of model response / 模型回答英文翻译** | | Normal, called “mommy brain,” due to sleep deprivation and hormonal changes. | | Yes, it affects mood, immunity, and recovery. Ask family to help at night. | | Share night feeds, take over baby care during the day, help with housework, and provide emotional support. |
| **Overall evaluation item / 整体评价项目** | | | **Score / 分数** | | **Comments / 备注** | |
| **Patient Satisfaction / 患者满意度** | | | 3 | |  | |
| **Patient Understandability / 患者可理解性** | | | 4 | |  | |
| **Patient Usefulness / 患者实用性** | | | 3 | |  | |
| **Clinician Correctness / 医生评分：正确性** | | | 4 | |  | |
| **Clinician Completeness / 医生评分：完整性** | | | 3 | |  | |
| **Clinician Readability / 医生评分：可读性** | | | 4 | |  | |
| **Clinician Helpfulness / 医生评分：帮助性** | | | 3 | |  | |
| **Clinician Safety / 医生评分：安全性** | | | 4 | |  | |

**Patient 37**

| **Patient ID / 患者编号** | **Group / 模型组别** | | | | | |
| --- | --- | --- | --- | --- | --- | --- |
| P37 | DeepSeek V3 | | | | | |
| **General model response or patient education material (Chinese) / 模型总体回答或患者教育材料（中文）** | | | | | | |
| 产后总担心自己做不好妈妈、晚上反复检查宝宝，这在产后早期尤其是在新生儿期，很多妈妈都会有类似行为，可以看作是过度警觉的母性本能。但如果这种担心和反复检查已经严重影响到您的睡眠（例如明明宝宝睡得很好，您却每小时起来检查一次）、导致您无法放松、整天处于惊恐状态、或者反复出现伤害自己或宝宝的想法，那就超出了正常范围，需要专业帮助。需要找心理医生或精神科医生的情况包括：1）连续超过2周几乎每天心情低落、焦虑或易怒；2）对以前喜欢的事情完全失去兴趣；3）睡眠问题（即使宝宝睡了也睡不着，或者总做噩梦）；4）反复出现死亡或自杀的想法，或者反复出现想要伤害宝宝的想法（哪怕只是瞬间念头）；5）出现幻听或妄想（比如听到声音命令您做某件事）。如果家人不理解您的焦虑，建议您：1）选择家人情绪平稳的时候，用“我陈述句”表达，例如“妈妈/老公，我知道你关心我，但我最近总是控制不住地担心，睡不好，我真的很痛苦，我需要去看医生”；2）邀请家人陪同您一起就诊，让医生向他们解释这是产后焦虑或产后抑郁，不是“矫情”或“想太多”；3）给家人看权威的产后心理卫生科普材料。请记住，寻求帮助是勇敢的表现。 | | | | | | |
| **English translation of the Chinese general response / 中文总体回答英文翻译** | | | | | | |
| Worrying about not being a good mother and repeatedly checking the baby at night — many mothers have similar behaviors in the early postpartum period, especially during the newborn stage. This can be seen as a hypervigilant maternal instinct. However, if this worry and repeated checking severely affect your sleep (for example, checking the baby every hour even when the baby is sleeping well), prevent you from relaxing, keep you in a constant state of panic, or involve recurrent thoughts of harming yourself or the baby, then it is beyond the normal range and requires professional help. Situations in which you should see a psychologist or psychiatrist include: 1) Persistent low mood, anxiety, or irritability nearly every day for more than 2 weeks; 2) Complete loss of interest in previously enjoyed activities; 3) Sleep problems (unable to sleep even when the baby sleeps, or recurrent nightmares); 4) Recurrent thoughts of death or suicide, or recurrent thoughts of wanting to hurt the baby (even fleeting); 5) Hallucinations or delusions (such as hearing voices commanding you to do something). If your family does not understand your anxiety, suggestions: 1) Choose a calm time and use “I statements,” such as “Mom/honey, I know you care about me, but I can’t stop worrying and can’t sleep well. I am really suffering. I need to see a doctor”; 2) Invite a family member to accompany you to the appointment so the doctor can explain that this is postpartum anxiety or depression, not “being oversensitive” or “overthinking”; 3) Share authoritative postpartum mental health educational materials with your family. Remember, seeking help is a sign of courage. | | | | | | |
| **Item / 项目** | | **Q1 / 问题1** | | **Q2 / 问题2** | | **Q3 / 问题3** |
| **Patient question (Chinese original) / 患者原始问题（中文）** | | 我产后总担心自己做不好妈妈，晚上反复检查宝宝，正常吗？ | | 什么情况下需要找心理医生或精神科医生？ | | 如果家人不理解我的焦虑，应该怎么沟通？ |
| **English translation of patient question / 患者问题英文翻译** | | I always worry that I am not a good mother after birth and repeatedly check the baby at night. Is this normal? | | In what situations should I see a psychologist or psychiatrist? | | If my family does not understand my anxiety, how should I communicate with them? |
| **Model response (Chinese) / 模型回答（中文）** | | 轻度是正常的母性警觉。如严重影响睡眠或生活，需警惕产后焦虑。 | | 情绪持续低落>2周、完全无兴趣、失眠、自杀或伤婴念头。 | | 用“我陈述句”表达感受，请医生向家人解释，给家人看科普材料。 |
| **English translation of model response / 模型回答英文翻译** | | Mild checking is normal. If severe, suspect postpartum anxiety. | | Persistent low mood >2 weeks, loss of interest, insomnia, suicidal or harm-to-baby thoughts. | | Use “I” statements, have doctor explain to family, and share educational materials. |
| **Overall evaluation item / 整体评价项目** | | | **Score / 分数** | | **Comments / 备注** | |
| **Patient Satisfaction / 患者满意度** | | | 3 | |  | |
| **Patient Understandability / 患者可理解性** | | | 4 | |  | |
| **Patient Usefulness / 患者实用性** | | | 3 | |  | |
| **Clinician Correctness / 医生评分：正确性** | | | 4 | |  | |
| **Clinician Completeness / 医生评分：完整性** | | | 3 | |  | |
| **Clinician Readability / 医生评分：可读性** | | | 4 | |  | |
| **Clinician Helpfulness / 医生评分：帮助性** | | | 3 | |  | |
| **Clinician Safety / 医生评分：安全性** | | | 4 | |  | |

**Patient 38**

| **Patient ID / 患者编号** | **Group / 模型组别** | | | | | |
| --- | --- | --- | --- | --- | --- | --- |
| P38 | DeepSeek V3 | | | | | |
| **General model response or patient education material (Chinese) / 模型总体回答或患者教育材料（中文）** | | | | | | |
| 产后复查发现盆底肌力差，您不是一个人，这是非常普遍的现象。盆底肌力差不等于一定会发生脱垂或尿失禁，但确实是需要重视的信号。日常生活中需要注意：1）避免所有会明显增加腹内压的行为：提重物（超过5公斤）、久蹲、用力排便、慢性咳嗽、频繁憋尿；2）治疗和预防便秘，保持大便柔软通畅（多吃膳食纤维、多喝水、必要时使用乳果糖等软便剂）；3）坚持正确的盆底肌训练（凯格尔运动），每天做3-5组，每组10-15次收缩，每次收缩保持3-5秒，放松3-5秒；4）产后42天复查时主动要求做盆底功能评估。关于盆底康复治疗是否疼痛：规范的盆底康复（生物反馈、电刺激）通常不会有明显疼痛，只会有轻微的肌肉收缩感或电流刺激感（治疗师会从低能量开始）。如果感到疼痛，可以随时告诉治疗师降低强度。疗程因人而异，一般建议每周2-3次，持续8-12周为一个疗程，之后根据复查结果决定是否需要继续。关于做盆底康复期间能否正常哺乳和带孩子：完全可以。盆底康复治疗通常每次30分钟左右，治疗时您可以带上宝宝（很多医院有母婴室或允许家属陪同），治疗结束后即可正常哺乳和照顾宝宝，不受任何影响。 | | | | | | |
| **English translation of the Chinese general response / 中文总体回答英文翻译** | | | | | | |
| If your postpartum check-up found weak pelvic floor muscles, you are not alone — this is very common. Weak pelvic floor muscles do not necessarily mean you will develop prolapse or urinary incontinence, but it is a sign that deserves attention. Daily precautions include: 1) Avoid all behaviors that significantly increase intra-abdominal pressure: heavy lifting (over 5 kg), prolonged squatting, straining to defecate, chronic coughing, frequently holding urine; 2) Treat and prevent constipation, keeping stools soft and easy to pass (eat more dietary fiber, drink more water, use stool softeners like lactulose if needed); 3) Perform correct pelvic floor muscle training (Kegel exercises) daily: 3-5 sets per day, 10-15 contractions per set, each contraction holding for 3-5 seconds, relaxing for 3-5 seconds; 4) At the 42-day postpartum check-up, proactively ask for a pelvic floor function assessment. Regarding whether pelvic floor rehabilitation is painful: Standard rehabilitation (biofeedback, electrical stimulation) is usually not painful, only a mild sensation of muscle contraction or electrical stimulation (the therapist will start at a low intensity). If you feel pain, you can tell the therapist to reduce the intensity at any time. The treatment course varies per individual, but generally 2-3 sessions per week for 8 to 12 weeks is one course, after which follow-up assessment determines whether to continue. Regarding whether you can breastfeed and care for your baby normally during pelvic floor rehabilitation: absolutely yes. Each rehabilitation session usually lasts about 30 minutes. You can bring your baby (many hospitals have mother-baby rooms or allow family accompaniment), and after the session you can immediately resume breastfeeding and baby care without any restriction. | | | | | | |
| **Item / 项目** | | **Q1 / 问题1** | | **Q2 / 问题2** | | **Q3 / 问题3** |
| **Patient question (Chinese original) / 患者原始问题（中文）** | | 我产后复查发现盆底肌力差，平时需要注意什么？ | | 盆底康复治疗会疼吗，一般需要做多久？ | | 做盆底康复期间还能正常哺乳和带孩子吗？ |
| **English translation of patient question / 患者问题英文翻译** | | My postpartum check found weak pelvic floor muscles. What should I pay attention to in daily life? | | Is pelvic floor rehabilitation painful, and how long does it usually take? | | Can I still breastfeed and care for my baby while receiving pelvic floor rehabilitation? |
| **Model response (Chinese) / 模型回答（中文）** | | 避免提重物、久蹲、便秘，坚持凯格尔训练。 | | 不疼，通常每周2-3次，持续8-12周。 | | 可以。治疗不影响哺乳和照顾宝宝。 |
| **English translation of model response / 模型回答英文翻译** | | Avoid heavy lifting, squatting, constipation, and do Kegel exercises. | | Not painful, typically 2-3 sessions/week for 8-12 weeks. | | Yes, it does not interfere with breastfeeding or baby care. |
| **Overall evaluation item / 整体评价项目** | | | **Score / 分数** | | **Comments / 备注** | |
| **Patient Satisfaction / 患者满意度** | | | 3 | |  | |
| **Patient Understandability / 患者可理解性** | | | 4 | |  | |
| **Patient Usefulness / 患者实用性** | | | 3 | |  | |
| **Clinician Correctness / 医生评分：正确性** | | | 3 | |  | |
| **Clinician Completeness / 医生评分：完整性** | | | 3 | |  | |
| **Clinician Readability / 医生评分：可读性** | | | 5 | |  | |
| **Clinician Helpfulness / 医生评分：帮助性** | | | 3 | |  | |
| **Clinician Safety / 医生评分：安全性** | | | 3 | |  | |

**Patient 39**

| **Patient ID / 患者编号** | **Group / 模型组别** | | | | | |
| --- | --- | --- | --- | --- | --- | --- |
| P39 | DeepSeek V3 | | | | | |
| **General model response or patient education material (Chinese) / 模型总体回答或患者教育材料（中文）** | | | | | | |
| 产后想减重是非常正常的愿望，但哺乳期绝对不要严格节食。因为哺乳期每天通过乳汁消耗约400-500千卡热量，这本身已经是一种“天然的减重优势”。如果您再过度限制能量摄入（比如每天低于1500-1800千卡），会导致：营养不足影响身体恢复、乳汁质量下降、疲劳加重、情绪变差，甚至可能因为身体应激而导致奶量减少。产后健康的减重速度是每周减0.5-1公斤，不要追求快速减重。方法包括：均衡饮食（控制精制碳水和添加糖、增加优质蛋白和蔬菜）、规律中等强度运动（如快走、产后瑜伽、游泳，需在伤口愈合和盆底评估后进行）、保证充足睡眠（睡眠不足会升高皮质醇，促进腹部脂肪堆积）。如果减重后奶量确实下降，请采取以下措施：1）放慢减重速度，增加能量摄入200-300千卡/天；2）增加饮水量（每天2500-3000毫升）；3）增加有效哺乳或挤奶频率（每天至少8-10次）；4）可以在加餐时吃一些传统认为帮助下奶的食物（如燕麦、黑芝麻、木瓜鲫鱼汤等，但不要依赖油腻汤水）。绝大多数情况下，只要不是过度减重，奶量可以恢复。如果持续下降，请咨询哺乳指导或营养科医生。 | | | | | | |
| **English translation of the Chinese general response / 中文总体回答英文翻译** | | | | | | |
| Wanting to lose weight after birth is completely normal, but during breastfeeding, you must absolutely not follow a strict diet. Breastfeeding itself burns approximately 400-500 calories per day through milk production, which is already a “natural weight loss advantage.” If you severely restrict energy intake (for example, below 1500-1800 calories per day), you risk: nutritional deficiencies impairing physical recovery, reduced milk quality, worsening fatigue, worsened mood, and even decreased milk supply due to physical stress. A healthy postpartum weight loss rate is 0.5 to 1 kg per week — do not aim for rapid weight loss. Approaches include: a balanced diet (control refined carbs and added sugars, increase high-quality protein and vegetables), regular moderate-intensity exercise (such as brisk walking, postpartum yoga, swimming — but only after wound healing and pelvic floor assessment), and adequate sleep (sleep deprivation raises cortisol and promotes abdominal fat accumulation). If your milk supply does drop with weight loss, take these steps: 1) Slow your weight loss rate and increase energy intake by 200-300 calories/day; 2) Increase fluid intake (2500-3000 mL/day); 3) Increase effective breastfeeding or pumping frequency (at least 8-10 times/day); 4) Add snacks that are traditionally believed to support milk supply (such as oats, black sesame, papaya with crucian carp soup — but do not rely on greasy soups). In most cases, as long as weight loss is not excessive, milk supply can recover. If it continues to drop, consult a lactation specialist or nutritionist. | | | | | | |
| **Item / 项目** | | **Q1 / 问题1** | | **Q2 / 问题2** | | **Q3 / 问题3** |
| **Patient question (Chinese original) / 患者原始问题（中文）** | | 我产后想减重，哺乳期可以节食吗？ | | 产后减重速度多少比较合适？ | | 如果减重后奶量下降，应该怎么办？ |
| **English translation of patient question / 患者问题英文翻译** | | I want to lose weight after birth. Can I diet while breastfeeding? | | What is an appropriate rate of postpartum weight loss? | | What should I do if my milk supply decreases after losing weight? |
| **Model response (Chinese) / 模型回答（中文）** | | 不可以。哺乳期每天消耗400-500千卡，过度节食影响奶量和恢复。 | | 每周0.5-1公斤，通过均衡饮食和运动。 | | 增加能量摄入200-300千卡/天，多喝水，增加哺乳频率。 |
| **English translation of model response / 模型回答英文翻译** | | No. Breastfeeding burns 400-500 kcal/day. Extreme dieting harms milk supply and recovery. | | 0.5-1 kg per week through balanced diet and exercise. | | Increase calories by 200-300/day, hydrate more, and feed more often. |
| **Overall evaluation item / 整体评价项目** | | | **Score / 分数** | | **Comments / 备注** | |
| **Patient Satisfaction / 患者满意度** | | | 3 | |  | |
| **Patient Understandability / 患者可理解性** | | | 4 | |  | |
| **Patient Usefulness / 患者实用性** | | | 3 | |  | |
| **Clinician Correctness / 医生评分：正确性** | | | 4 | |  | |
| **Clinician Completeness / 医生评分：完整性** | | | 3 | |  | |
| **Clinician Readability / 医生评分：可读性** | | | 4 | |  | |
| **Clinician Helpfulness / 医生评分：帮助性** | | | 3 | |  | |
| **Clinician Safety / 医生评分：安全性** | | | 4 | |  | |

**Patient 40**

| **Patient ID / 患者编号** | **Group / 模型组别** | | | | | |
| --- | --- | --- | --- | --- | --- | --- |
| P40 | DeepSeek V3 | | | | | |
| **General model response or patient education material (Chinese) / 模型总体回答或患者教育材料（中文）** | | | | | | |
| 产后照顾宝宝太忙、经常忘记吃饭，这会严重影响您的身体恢复、情绪状态和乳汁分泌，请您务必重视。产后身体需要额外的能量和营养素来修复组织、制造血液、产生乳汁、维持免疫力。经常漏餐会导致：低血糖（头晕、心慌、出冷汗）、体力恢复延迟、免疫力下降（更容易感染）、情绪不稳定（易怒、焦虑）、乳汁分泌减少。产褥期每天应该保证以下基本内容：1）三餐定时（可以设置手机闹钟提醒），实在忙不过来时准备健康加餐（酸奶、坚果、全麦饼干、水果）；2）每天饮水至少2000毫升，放一个水杯在身边随时喝；3）会阴或切口护理：每天清洗、更换干净内裤和卫生巾，观察伤口有无红肿渗液；4）短时休息：抓住宝宝睡觉的碎片时间，躺下闭眼休息15-20分钟，不一定要睡着；5）适度活动：每天下床缓慢走动、做温和的伸展。什么时候需要主动寻求帮助：当您出现以下任何情况时，请不要犹豫，立即向家人、朋友或医护人员求助：1）发热≥38.5℃；2）产后出血突然增多（超过月经量）；3）难以忍受的疼痛（伤口、乳房、腹痛）；4）情绪崩溃、想哭停不下来、觉得活不下去或有伤害自己/宝宝的想法；5）连续超过24小时无法入睡；6）无法照顾自己最基本的需求（吃饭、喝水、上厕所）。请记住，您不是超人，求助是负责任的表现。 | | | | | | |
| **English translation of the Chinese general response / 中文总体回答英文翻译** | | | | | | |
| If you are so busy caring for the baby after birth that you often forget to eat, this will seriously affect your physical recovery, emotional state, and milk production. Please take this seriously. After birth, your body needs extra energy and nutrients to repair tissues, produce blood, generate milk, and maintain immunity. Frequent missed meals can cause: low blood sugar (dizziness, palpitations, cold sweats), delayed physical recovery, decreased immunity (increased susceptibility to infection), emotional instability (irritability, anxiety), and decreased milk production. During the puerperium, you should ensure the following basics daily: 1) Three regular meals (set phone alarms if needed), and keep healthy snacks (yogurt, nuts, whole-grain crackers, fruit) on hand if you cannot prepare a full meal; 2) Drink at least 2000 mL of water daily — keep a water bottle nearby to sip throughout the day; 3) Perineal or incision care: clean daily, change into clean underwear and sanitary pads, and observe for redness, swelling, or drainage; 4) Short rest periods: use the fragmented times when the baby sleeps, lie down and rest with eyes closed for 15-20 minutes — you do not have to fall asleep; 5) Moderate activity: slow walking and gentle stretching daily. When to actively seek help: Do not hesitate to ask family, friends, or medical staff for help if you experience any of the following: 1) Fever ≥38.5°C; 2) Sudden increase in postpartum bleeding (more than a menstrual period); 3) Unbearable pain (wound, breast, abdomen); 4) Emotional breakdown, uncontrollable crying, feeling that life is not worth living, or thoughts of harming yourself or the baby; 5) Inability to sleep for more than 24 consecutive hours; 6) Inability to meet your most basic needs (eating, drinking, using the toilet). Remember, you are not a superhero. Asking for help is a sign of responsibility. | | | | | | |
| **Item / 项目** | | **Q1 / 问题1** | | **Q2 / 问题2** | | **Q3 / 问题3** |
| **Patient question (Chinese original) / 患者原始问题（中文）** | | 产后照顾宝宝太忙，经常忘记吃饭，会影响恢复吗？ | | 产褥期每天应该保证哪些基本休息和护理？ | | 什么时候需要主动寻求家人或医护人员帮助？ |
| **English translation of patient question / 患者问题英文翻译** | | I am so busy caring for the baby after birth that I often forget to eat. Will this affect recovery? | | What basic rest and care should be ensured every day during the puerperium? | | When should I actively seek help from family members or healthcare staff? |
| **Model response (Chinese) / 模型回答（中文）** | | 会。会导致低血糖、免疫力下降、奶量减少，请定时吃饭。 | | 三餐定时、补水、伤口护理、短时休息、温和活动。 | | 发热≥38.5℃、大出血、剧烈疼痛、情绪崩溃、无法入睡、无法自理。 |
| **English translation of model response / 模型回答英文翻译** | | Yes, it can cause low blood sugar, reduced immunity, and low milk supply. Set meal reminders. | | Regular meals, hydration, wound care, short rests, gentle activity. | | Fever ≥38.5°C, heavy bleeding, severe pain, emotional breakdown, no sleep for 24h, unable to self-care. |
| **Overall evaluation item / 整体评价项目** | | | **Score / 分数** | | **Comments / 备注** | |
| **Patient Satisfaction / 患者满意度** | | | 3 | |  | |
| **Patient Understandability / 患者可理解性** | | | 4 | |  | |
| **Patient Usefulness / 患者实用性** | | | 3 | |  | |
| **Clinician Correctness / 医生评分：正确性** | | | 4 | |  | |
| **Clinician Completeness / 医生评分：完整性** | | | 3 | |  | |
| **Clinician Readability / 医生评分：可读性** | | | 4 | |  | |
| **Clinician Helpfulness / 医生评分：帮助性** | | | 3 | |  | |
| **Clinician Safety / 医生评分：安全性** | | | 4 | |  | |

**Patient 41**

| **Patient ID / 患者编号** | **Group / 模型组别** | | | | | |
| --- | --- | --- | --- | --- | --- | --- |
| P41 | DeepSeek V3 | | | | | |
| **General model response or patient education material (Chinese) / 模型总体回答或患者教育材料（中文）** | | | | | | |
| 产后恶露颜色从红色变为淡粉色、褐色再到白色，是正常的演变过程。如果在颜色变淡后又出现少量褐色或咖啡色分泌物，通常是宫腔内残留的陈旧积血被逐渐排出，只要量少、无臭味、无腹痛、不伴发热，一般不需要特殊处理。血腥味是恶露的正常气味，但如果出现明显的腥臭味、腐臭味，同时伴有下腹痛、发热、分泌物增多或颜色变为黄绿色，则提示产褥感染可能，请及时就医。如果恶露断断续续超过4周仍不干净，建议在产后42天复查时做妇科B超，排除宫腔残留物、子宫复旧不良或剖宫产切口憩室等问题。 | | | | | | |
| **English translation of the Chinese general response / 中文总体回答英文翻译** | | | | | | |
| Postpartum lochia normally changes from red to pink, brown, and then white. If a small amount of brown or coffee-colored discharge appears after the color has lightened, it is usually old blood retained in the uterus being gradually expelled. As long as the amount is small, there is no foul odor, no abdominal pain, and no fever, no special treatment is needed. A bloody odor is normal for lochia, but if a distinct foul or putrid odor develops, accompanied by lower abdominal pain, fever, increased discharge, or yellow-green color, it suggests possible puerperal infection and requires prompt medical attention. If lochia persists intermittently for more than 4 weeks without clearing, it is recommended to have a pelvic ultrasound at the 42-day postpartum check-up to rule out retained products of conception, poor uterine involution, or cesarean scar defect (isthmocele). | | | | | | |
| **Item / 项目** | | **Q1 / 问题1** | | **Q2 / 问题2** | | **Q3 / 问题3** |
| **Patient question (Chinese original) / 患者原始问题（中文）** | | 我产后恶露颜色变淡后又有点褐色分泌物，这正常吗？ | | 恶露有血腥味和有臭味有什么区别？ | | 如果恶露一直断断续续，需要做B超检查吗？ |
| **English translation of patient question / 患者问题英文翻译** | | After my lochia became lighter in color, I started having some brown discharge again after birth. Is this normal? | | What is the difference between a bloody smell and a foul odor in lochia? | | If my lochia continues on and off, do I need a B-ultrasound examination? |
| **Model response (Chinese) / 模型回答（中文）** | | 正常。褐色分泌物多为陈旧积血，一般无需处理。 | | 血腥味正常，臭味提示感染，需就医。 | | 建议做B超，排除宫腔残留或子宫复旧不良。 |
| **English translation of model response / 模型回答英文翻译** | | Normal. Brown discharge is usually old blood, generally no need for treatment. | | Bloody odor is normal; foul odor suggests infection and requires medical attention. | | Ultrasound is recommended to rule out retained products or poor involution. |
| **Overall evaluation item / 整体评价项目** | | | **Score / 分数** | | **Comments / 备注** | |
| **Patient Satisfaction / 患者满意度** | | | 3 | |  | |
| **Patient Understandability / 患者可理解性** | | | 4 | |  | |
| **Patient Usefulness / 患者实用性** | | | 3 | |  | |
| **Clinician Correctness / 医生评分：正确性** | | | 4 | |  | |
| **Clinician Completeness / 医生评分：完整性** | | | 3 | |  | |
| **Clinician Readability / 医生评分：可读性** | | | 4 | |  | |
| **Clinician Helpfulness / 医生评分：帮助性** | | | 3 | |  | |
| **Clinician Safety / 医生评分：安全性** | | | 4 | |  | |

**Patient 42**

| **Patient ID / 患者编号** | **Group / 模型组别** | | | | | |
| --- | --- | --- | --- | --- | --- | --- |
| P42 | DeepSeek V3 | | | | | |
| **General model response or patient education material (Chinese) / 模型总体回答或患者教育材料（中文）** | | | | | | |
| 产后突然一阵发冷，同时没有发热，多数情况下是因为产后激素波动、疲劳、夜间睡眠不足或环境温度变化引起的血管舒缩反应，并非“身体虚弱”。一般注意保暖、加盖薄被、喝些温水即可缓解。夜里出汗把衣服湿透是产后“褥汗”的典型表现，是身体排出孕期潴留水分的正常过程，通常持续1-2周。建议您勤换衣物、床单，适当补充水分，不需要额外补盐。但如果出汗的同时伴有明显心慌、心跳过快（静息心率超过100次/分）、极度乏力、体重明显下降或出现发热，则需到医院检查甲状腺功能、血常规等，排除产后甲状腺炎、感染或贫血。 | | | | | | |
| **English translation of the Chinese general response / 中文总体回答英文翻译** | | | | | | |
| Sudden chills without fever after birth are usually due to postpartum hormonal fluctuations, fatigue, sleep deprivation, or environmental temperature changes causing vasomotor responses, not “weakness.” Keeping warm, covering with a light blanket, and drinking warm water usually help. Night sweats that soak through clothes are a typical manifestation of postpartum sweating, a normal process of eliminating excess fluid retained during pregnancy, typically lasting 1-2 weeks. Change clothes and sheets frequently and replenish fluids; no extra salt is needed. However, if sweating is accompanied by significant palpitations, tachycardia (resting heart rate >100/min), extreme weakness, unexplained weight loss, or fever, you should see a doctor to check thyroid function and blood count, ruling out postpartum thyroiditis, infection, or anemia. | | | | | | |
| **Item / 项目** | | **Q1 / 问题1** | | **Q2 / 问题2** | | **Q3 / 问题3** |
| **Patient question (Chinese original) / 患者原始问题（中文）** | | 我产后有时会突然一阵发冷，这是身体虚弱吗？ | | 夜里出汗把衣服都湿透了，需要特别处理吗？ | | 如果出汗同时心跳很快，需要去医院吗？ |
| **English translation of patient question / 患者问题英文翻译** | | After birth, I sometimes suddenly feel a chill. Is this due to physical weakness? | | At night I sweat so much that my clothes are soaked. Do I need special treatment? | | If I sweat and my heartbeat is very fast at the same time, do I need to go to the hospital? |
| **Model response (Chinese) / 模型回答（中文）** | | 常见于激素波动或疲劳，注意保暖休息。 | | 正常，及时更换衣物、补充水分即可。 | | 需要，排查甲状腺功能异常或感染。 |
| **English translation of model response / 模型回答英文翻译** | | Common due to hormone changes or fatigue; keep warm and rest. | | Normal; change clothes and rehydrate. | | Yes, to rule out thyroid dysfunction or infection. |
| **Overall evaluation item / 整体评价项目** | | | **Score / 分数** | | **Comments / 备注** | |
| **Patient Satisfaction / 患者满意度** | | | 3 | |  | |
| **Patient Understandability / 患者可理解性** | | | 4 | |  | |
| **Patient Usefulness / 患者实用性** | | | 3 | |  | |
| **Clinician Correctness / 医生评分：正确性** | | | 3 | |  | |
| **Clinician Completeness / 医生评分：完整性** | | | 3 | |  | |
| **Clinician Readability / 医生评分：可读性** | | | 5 | |  | |
| **Clinician Helpfulness / 医生评分：帮助性** | | | 3 | |  | |
| **Clinician Safety / 医生评分：安全性** | | | 3 | |  | |

**Patient 43**

| **Patient ID / 患者编号** | **Group / 模型组别** | | | | | |
| --- | --- | --- | --- | --- | --- | --- |
| P43 | DeepSeek V3 | | | | | |
| **General model response or patient education material (Chinese) / 模型总体回答或患者教育材料（中文）** | | | | | | |
| 产后小便排不出来，医学上称为产后尿潴留。顺产后4-6小时或剖宫产拔除尿管后6小时内如果不能自行排尿，或排尿后仍有明显尿不净感、膀胱区胀痛，就需要告诉医护人员。可以尝试听流水声、温水冲洗会阴、热敷下腹部等方法帮助排尿。如果上述方法无效，可能需要导尿。导尿后一般不影响下床活动，但要避免剧烈运动和长时间站立。如果排尿时疼痛、尿频、尿急越来越明显，或者出现发热、腰痛，应尽早查尿常规，排除尿路感染。产后尿潴留如果得不到及时处理，会增加泌尿系感染和长期排尿功能障碍的风险。 | | | | | | |
| **English translation of the Chinese general response / 中文总体回答英文翻译** | | | | | | |
| Inability to urinate after birth is called postpartum urinary retention. If you cannot void within 4-6 hours after a vaginal delivery or within 6 hours after catheter removal following a cesarean section, or if you have persistent sensation of incomplete emptying and suprapubic distension, inform medical staff. Try running water, warm perineal rinsing, or lower abdominal warmth to help void. If these fail, catheterization may be needed. After catheter removal, ambulation is generally fine, but avoid vigorous activity and prolonged standing. If painful urination, frequency, urgency, or fever and back pain develop, get a urinalysis early to rule out urinary tract infection. Untreated postpartum urinary retention increases the risk of UTI and long-term voiding dysfunction. | | | | | | |
| **Item / 项目** | | **Q1 / 问题1** | | **Q2 / 问题2** | | **Q3 / 问题3** |
| **Patient question (Chinese original) / 患者原始问题（中文）** | | 我产后小便一直排不出来，是不是尿潴留？ | | 导尿以后还能正常下床活动吗？ | | 如果尿痛、尿频越来越明显，需要查尿常规吗？ |
| **English translation of patient question / 患者问题英文翻译** | | After birth, I have been unable to urinate. Is this urinary retention? | | Can I still get out of bed and move around normally after catheterization? | | If painful urination and frequent urination become more obvious, do I need a urine routine test? |
| **Model response (Chinese) / 模型回答（中文）** | | 可能，产后6小时仍不能排尿应告知医护。 | | 可以，但避免剧烈活动。 | | 需要，排除尿路感染。 |
| **English translation of model response / 模型回答英文翻译** | | Possible; if unable to void for 6 hours postpartum, inform medical staff. | | Yes, but avoid vigorous activity. | | Yes, to rule out UTI. |
| **Overall evaluation item / 整体评价项目** | | | **Score / 分数** | | **Comments / 备注** | |
| **Patient Satisfaction / 患者满意度** | | | 3 | |  | |
| **Patient Understandability / 患者可理解性** | | | 4 | |  | |
| **Patient Usefulness / 患者实用性** | | | 3 | |  | |
| **Clinician Correctness / 医生评分：正确性** | | | 3 | |  | |
| **Clinician Completeness / 医生评分：完整性** | | | 3 | |  | |
| **Clinician Readability / 医生评分：可读性** | | | 5 | |  | |
| **Clinician Helpfulness / 医生评分：帮助性** | | | 3 | |  | |
| **Clinician Safety / 医生评分：安全性** | | | 3 | |  | |

**Patient 44**

| **Patient ID / 患者编号** | **Group / 模型组别** | | | | | |
| --- | --- | --- | --- | --- | --- | --- |
| P44 | DeepSeek V3 | | | | | |
| **General model response or patient education material (Chinese) / 模型总体回答或患者教育材料（中文）** | | | | | | |
| 产后手脚轻度水肿，在产后1-2周内大多会自行消退，这是孕期体内多余水分逐渐排出的正常现象。坐月子期间不应刻意少喝水，相反，每天保证2000-2500毫升的饮水量有助于肾脏排出多余水分，防止便秘和尿路感染。但如果您发现脚肿的同时伴有面部浮肿、持续头痛（尤其是后枕部）、视力模糊、恶心或血压升高（收缩压≥140 mmHg），请立即就医，这可能是产后子痫前期的表现。如果水肿不对称，只有一条腿肿痛、皮温升高，也需急诊排除下肢深静脉血栓。 | | | | | | |
| **English translation of the Chinese general response / 中文总体回答英文翻译** | | | | | | |
| Mild hand and foot edema after birth usually resolves within 1-2 weeks as the body eliminates excess fluid retained during pregnancy. Do not restrict water intake during confinement; on the contrary, drinking 2000-2500 mL daily helps kidneys excrete extra fluid and prevents constipation and UTIs. However, if swelling is accompanied by facial edema, persistent headache (especially occipital), blurred vision, nausea, or elevated blood pressure (systolic ≥140 mmHg), seek immediate medical attention as this may indicate postpartum preeclampsia. If edema is asymmetric (one leg swollen, painful, warm), also go to the emergency department to rule out deep vein thrombosis. | | | | | | |
| **Item / 项目** | | **Q1 / 问题1** | | **Q2 / 问题2** | | **Q3 / 问题3** |
| **Patient question (Chinese original) / 患者原始问题（中文）** | | 我产后手脚还有点肿，多久能消退？ | | 坐月子期间水肿能不能少喝水？ | | 如果脸肿、头痛或者血压高，需要马上就医吗？ |
| **English translation of patient question / 患者问题英文翻译** | | My hands and feet are still a little swollen after birth. How long will it take to go away? | | During the confinement period, can I drink less water because of swelling? | | If my face is swollen, I have a headache, or my blood pressure is high, do I need immediate medical care? |
| **Model response (Chinese) / 模型回答（中文）** | | 通常1-2周消退。 | | 不能，正常饮水有助代谢。 | | 需要，警惕子痫前期。 |
| **English translation of model response / 模型回答英文翻译** | | Usually resolves within 1-2 weeks. | | No, adequate hydration helps metabolism. | | Yes, suspect preeclampsia. |
| **Overall evaluation item / 整体评价项目** | | | **Score / 分数** | | **Comments / 备注** | |
| **Patient Satisfaction / 患者满意度** | | | 3 | |  | |
| **Patient Understandability / 患者可理解性** | | | 4 | |  | |
| **Patient Usefulness / 患者实用性** | | | 3 | |  | |
| **Clinician Correctness / 医生评分：正确性** | | | 3 | |  | |
| **Clinician Completeness / 医生评分：完整性** | | | 3 | |  | |
| **Clinician Readability / 医生评分：可读性** | | | 5 | |  | |
| **Clinician Helpfulness / 医生评分：帮助性** | | | 3 | |  | |
| **Clinician Safety / 医生评分：安全性** | | | 3 | |  | |

**Patient 45**

| **Patient ID / 患者编号** | **Group / 模型组别** | | | | | |
| --- | --- | --- | --- | --- | --- | --- |
| P45 | DeepSeek V3 | | | | | |
| **General model response or patient education material (Chinese) / 模型总体回答或患者教育材料（中文）** | | | | | | |
| 产后站起来头晕、眼前发黑，常见于体位性低血压或贫血。建议您起床或站立时动作放慢，先在床边坐2-3分钟，再慢慢站起。如果是轻度贫血（血红蛋白90-110 g/L），可以通过饮食补充红肉、动物肝脏、黑木耳、菠菜等富含铁的食物，同时搭配橙子、猕猴桃等维生素C丰富的食物促进铁吸收。但如果贫血较重（血红蛋白低于90 g/L）或食补2-4周后头晕乏力仍不改善，需要口服铁剂治疗。如果心慌、乏力非常明显，甚至活动后气喘、面色苍白，请及时复查血常规，评估贫血程度并查找原因。 | | | | | | |
| **English translation of the Chinese general response / 中文总体回答英文翻译** | | | | | | |
| Dizziness and black spots upon standing after birth are often due to orthostatic hypotension or anemia. Rise slowly: sit at the bedside for 2-3 minutes before standing. For mild anemia (Hb 90-110 g/L), dietary iron from red meat, liver, black fungus, spinach, combined with vitamin C-rich foods like oranges and kiwis, can help. For moderate to severe anemia (Hb <90 g/L) or persistent symptoms after 2-4 weeks of dietary adjustment, oral iron supplements are needed. If palpitations, fatigue, shortness of breath on exertion, or pallor are pronounced, have a repeat CBC promptly to assess anemia and identify the cause. | | | | | | |
| **Item / 项目** | | **Q1 / 问题1** | | **Q2 / 问题2** | | **Q3 / 问题3** |
| **Patient question (Chinese original) / 患者原始问题（中文）** | | 我产后站起来头晕眼前发黑，是贫血吗？ | | 产后贫血只靠食补可以恢复吗？ | | 如果心慌、乏力很明显，需要复查血常规吗？ |
| **English translation of patient question / 患者问题英文翻译** | | After birth, I feel dizzy and my vision goes dark when I stand up. Is this anemia? | | Can postpartum anemia recover through diet alone? | | If palpitations and fatigue are very obvious, do I need to repeat a complete blood count? |
| **Model response (Chinese) / 模型回答（中文）** | | 可能是贫血或体位性低血压，缓慢起身。 | | 轻度可以，中重度需补铁剂。 | | 需要。 |
| **English translation of model response / 模型回答英文翻译** | | Possible anemia or orthostatic hypotension; rise slowly. | | Mild anemia may recover with diet; moderate/severe needs iron supplements. | | Yes. |
| **Overall evaluation item / 整体评价项目** | | | **Score / 分数** | | **Comments / 备注** | |
| **Patient Satisfaction / 患者满意度** | | | 3 | |  | |
| **Patient Understandability / 患者可理解性** | | | 4 | |  | |
| **Patient Usefulness / 患者实用性** | | | 3 | |  | |
| **Clinician Correctness / 医生评分：正确性** | | | 3 | |  | |
| **Clinician Completeness / 医生评分：完整性** | | | 3 | |  | |
| **Clinician Readability / 医生评分：可读性** | | | 5 | |  | |
| **Clinician Helpfulness / 医生评分：帮助性** | | | 3 | |  | |
| **Clinician Safety / 医生评分：安全性** | | | 3 | |  | |

**Patient 46**

| **Patient ID / 患者编号** | **Group / 模型组别** | | | | | |
| --- | --- | --- | --- | --- | --- | --- |
| P46 | DeepSeek V3 | | | | | |
| **General model response or patient education material (Chinese) / 模型总体回答或患者教育材料（中文）** | | | | | | |
| 产后痔疮出血很常见，尤其是在分娩时用力过度或产后便秘的情况下。只要出血量不大（纸巾擦拭有少许鲜血或排便后滴几滴），一般不会造成严重后果。建议您保持大便柔软通畅：多喝水、吃高纤维食物（火龙果、西梅、燕麦、绿叶菜），必要时在医生指导下使用乳果糖。同时每天温水坐浴2-3次，每次10-15分钟。哺乳期可以使用角菜酸酯乳膏或栓剂，局部用药几乎不进入乳汁，对宝宝安全。如果大便带血越来越多，血色鲜红呈喷射状，或伴有头晕、乏力、面色苍白，应尽快到肛肠科就诊，排除痔疮破裂大出血或合并其他肠道问题。 | | | | | | |
| **English translation of the Chinese general response / 中文总体回答英文翻译** | | | | | | |
| Postpartum hemorrhoid bleeding is very common, especially after straining during delivery or due to constipation. As long as bleeding is minimal (a few drops of bright red blood on toilet paper or after defecation), it rarely causes serious consequences. Keep stools soft and smooth: drink plenty of water, eat high-fiber foods (dragon fruit, prunes, oats, leafy greens), and use lactulose if needed under medical guidance. Also use warm sitz baths 2-3 times daily for 10-15 minutes. Topical hemorrhoid creams or suppositories (e.g., titanium dioxide) are safe during breastfeeding as systemic absorption is minimal. If bleeding increases (squirting bright red blood) or is accompanied by dizziness, fatigue, or pallor, see a proctologist promptly to rule out severe bleeding or other intestinal issues. | | | | | | |
| **Item / 项目** | | **Q1 / 问题1** | | **Q2 / 问题2** | | **Q3 / 问题3** |
| **Patient question (Chinese original) / 患者原始问题（中文）** | | 我产后痔疮出血了，会不会很严重？ | | 哺乳期痔疮膏可以用吗？ | | 如果大便带血越来越多，需要看肛肠科吗？ |
| **English translation of patient question / 患者问题英文翻译** | | My hemorrhoids are bleeding after birth. Could this be serious? | | Can I use hemorrhoid ointment while breastfeeding? | | If the amount of blood in my stool keeps increasing, do I need to see a colorectal specialist? |
| **Model response (Chinese) / 模型回答（中文）** | | 常见，保持大便通畅、温水坐浴。 | | 可用角菜酸酯等哺乳期安全药膏。 | | 需要。 |
| **English translation of model response / 模型回答英文翻译** | | Common; keep stools soft and sitz bath. | | Yes, use breastfeeding-safe ointments (e.g., titanium dioxide). | | Yes. |
| **Overall evaluation item / 整体评价项目** | | | **Score / 分数** | | **Comments / 备注** | |
| **Patient Satisfaction / 患者满意度** | | | 3 | |  | |
| **Patient Understandability / 患者可理解性** | | | 4 | |  | |
| **Patient Usefulness / 患者实用性** | | | 3 | |  | |
| **Clinician Correctness / 医生评分：正确性** | | | 3 | |  | |
| **Clinician Completeness / 医生评分：完整性** | | | 3 | |  | |
| **Clinician Readability / 医生评分：可读性** | | | 5 | |  | |
| **Clinician Helpfulness / 医生评分：帮助性** | | | 3 | |  | |
| **Clinician Safety / 医生评分：安全性** | | | 3 | |  | |

**Patient 47**

| **Patient ID / 患者编号** | **Group / 模型组别** | | | | | |
| --- | --- | --- | --- | --- | --- | --- |
| P47 | DeepSeek V3 | | | | | |
| **General model response or patient education material (Chinese) / 模型总体回答或患者教育材料（中文）** | | | | | | |
| 会阴伤口上长出小肉芽，通常是过度增生的肉芽组织，属于伤口愈合过程中的一种常见现象。只要肉芽不出血、不疼痛、不影响走路或同房，一般不需要处理，多数会在数月内自行萎缩或变平。如果肉芽引起擦拭时出血、按压疼痛或明显影响生活，可以在产后复查时请医生用硝酸银烧灼或门诊切除，操作简单。伤口的牵拉感会随着疤痕软化而逐渐减轻，一般不会影响走路。但如果牵拉感伴有剧烈疼痛、伤口红肿或分泌物增多，则需就医排查感染或疤痕内异症。 | | | | | | |
| **English translation of the Chinese general response / 中文总体回答英文翻译** | | | | | | |
| Small granulation tissue on a perineal wound is a common phenomenon during healing. As long as it does not bleed, hurt, or interfere with walking or intercourse, it usually requires no treatment and often shrinks or flattens within months. If it bleeds when wiped, is painful, or bothers daily life, a doctor can treat it with silver nitrate or remove it in the clinic. A pulling sensation typically improves as the scar softens and rarely affects walking. However, if pulling is accompanied by severe pain, redness, or increased discharge, seek medical attention to rule out infection or scar endometriosis. | | | | | | |
| **Item / 项目** | | **Q1 / 问题1** | | **Q2 / 问题2** | | **Q3 / 问题3** |
| **Patient question (Chinese original) / 患者原始问题（中文）** | | 我的会阴伤口摸着有小肉芽，是正常愈合吗？ | | 会阴伤口有牵拉感会不会影响走路？ | | 如果肉芽出血或者疼痛，需要处理吗？ |
| **English translation of patient question / 患者问题英文翻译** | | I can feel a small granulation-like growth on my perineal wound. Is this normal healing? | | Will a pulling sensation in the perineal wound affect walking? | | If the granulation tissue bleeds or is painful, does it need treatment? |
| **Model response (Chinese) / 模型回答（中文）** | | 常见，无不适可不处理。 | | 一般不影响，会随疤痕软化减轻。 | | 需要，可门诊去除。 |
| **English translation of model response / 模型回答英文翻译** | | Common; no treatment needed if asymptomatic. | | Usually not; improves as scar softens. | | Yes, can be removed in clinic. |
| **Overall evaluation item / 整体评价项目** | | | **Score / 分数** | | **Comments / 备注** | |
| **Patient Satisfaction / 患者满意度** | | | 3 | |  | |
| **Patient Understandability / 患者可理解性** | | | 4 | |  | |
| **Patient Usefulness / 患者实用性** | | | 3 | |  | |
| **Clinician Correctness / 医生评分：正确性** | | | 3 | |  | |
| **Clinician Completeness / 医生评分：完整性** | | | 3 | |  | |
| **Clinician Readability / 医生评分：可读性** | | | 5 | |  | |
| **Clinician Helpfulness / 医生评分：帮助性** | | | 3 | |  | |
| **Clinician Safety / 医生评分：安全性** | | | 3 | |  | |

**Patient 48**

| **Patient ID / 患者编号** | **Group / 模型组别** | | | | | |
| --- | --- | --- | --- | --- | --- | --- |
| P48 | DeepSeek V3 | | | | | |
| **General model response or patient education material (Chinese) / 模型总体回答或患者教育材料（中文）** | | | | | | |
| 剖宫产切口周围麻木是正常现象，因为手术切断了皮肤表面的小神经末梢。这种麻木感通常在术后6个月内逐渐恢复，部分人可能需要1年以上。切口附近偶尔出现刺痛、针扎感或过电感，恰恰是神经再生的表现，属于好现象，不需要担心。但如果麻木的范围越来越大，或者出现下肢无力、走路不稳、大小便异常，则需尽快复查，排除脊髓或神经根受压的可能。日常生活中注意保持切口清洁干燥，避免摩擦和搔抓，随着时间推移，感觉会慢慢恢复。 | | | | | | |
| **English translation of the Chinese general response / 中文总体回答英文翻译** | | | | | | |
| Numbness around a cesarean incision is normal due to severance of small cutaneous nerve endings. This numbness usually recovers within 6 months, though some may take over a year. Occasional stabbing, pins-and-needles, or electric sensations near the incision indicate nerve regeneration and are a good sign. However, if the numb area expands progressively, or you develop leg weakness, unsteady gait, or bladder/bowel dysfunction, seek prompt evaluation to rule out spinal or nerve root compression. Keep the incision clean and dry, avoid rubbing or scratching, and sensation will gradually return over time. | | | | | | |
| **Item / 项目** | | **Q1 / 问题1** | | **Q2 / 问题2** | | **Q3 / 问题3** |
| **Patient question (Chinese original) / 患者原始问题（中文）** | | 剖宫产切口周围麻木没有感觉，正常吗？ | | 切口附近有刺痛或针扎感，是神经恢复吗？ | | 如果麻木范围越来越大，需要复查吗？ |
| **English translation of patient question / 患者问题英文翻译** | | It feels numb around my cesarean incision with reduced sensation. Is this normal? | | Is a stinging or needle-like sensation near the incision a sign that the nerves are recovering? | | If the numb area becomes larger and larger, do I need a follow-up examination? |
| **Model response (Chinese) / 模型回答（中文）** | | 正常，因皮神经切断，多数半年内恢复。 | | 是，正常现象。 | | 需要，排除神经压迫。 |
| **English translation of model response / 模型回答英文翻译** | | Normal due to severed nerves; usually recovers within 6 months. | | Yes, normal sign of nerve regeneration. | | Yes, to rule out nerve compression. |
| **Overall evaluation item / 整体评价项目** | | | **Score / 分数** | | **Comments / 备注** | |
| **Patient Satisfaction / 患者满意度** | | | 3 | |  | |
| **Patient Understandability / 患者可理解性** | | | 4 | |  | |
| **Patient Usefulness / 患者实用性** | | | 3 | |  | |
| **Clinician Correctness / 医生评分：正确性** | | | 3 | |  | |
| **Clinician Completeness / 医生评分：完整性** | | | 3 | |  | |
| **Clinician Readability / 医生评分：可读性** | | | 5 | |  | |
| **Clinician Helpfulness / 医生评分：帮助性** | | | 3 | |  | |
| **Clinician Safety / 医生评分：安全性** | | | 3 | |  | |

**Patient 49**

| **Patient ID / 患者编号** | **Group / 模型组别** | | | | | |
| --- | --- | --- | --- | --- | --- | --- |
| P49 | DeepSeek V3 | | | | | |
| **General model response or patient education material (Chinese) / 模型总体回答或患者教育材料（中文）** | | | | | | |
| 剖宫产疤痕凸起，如果是红色、质地硬、高出皮肤表面且伴有瘙痒，可能是增生性瘢痕或瘢痕疙瘩，与个人体质有关。疤痕护理最好从拆线后2周开始，使用硅胶贴或硅酮凝胶，每天贴敷12小时以上，坚持3-6个月可有效抑制增生、软化疤痕。疤痕又红又痒时，可以继续使用硅胶贴，同时避免抓挠，也可以外涂硅酮凝胶。如果瘙痒严重影响睡眠或疤痕持续增大，可咨询皮肤科医生考虑局部注射激素或激光治疗。在切口完全愈合后，适当按摩也有助于改善疤痕外观。 | | | | | | |
| **English translation of the Chinese general response / 中文总体回答英文翻译** | | | | | | |
| A raised cesarean scar that is red, hard, itchy, and above the skin surface may be hypertrophic scar or keloid, related to individual predisposition. Scar care is best started 2 weeks after suture removal using silicone sheets or gel, applied for >12 hours daily for 3-6 months to effectively inhibit proliferation and soften the scar. When the scar is red and itchy, continue using silicone products and avoid scratching; silicone gel also helps. If itching severely affects sleep or the scar continues to enlarge, consult a dermatologist about intralesional steroid injections or laser therapy. Once the incision is fully healed, gentle massage can also improve appearance. | | | | | | |
| **Item / 项目** | | **Q1 / 问题1** | | **Q2 / 问题2** | | **Q3 / 问题3** |
| **Patient question (Chinese original) / 患者原始问题（中文）** | | 剖宫产疤痕有点凸起，是不是瘢痕增生？ | | 产后什么时候可以开始做疤痕护理？ | | 疤痕又红又痒时可以自己贴疤痕贴吗？ |
| **English translation of patient question / 患者问题英文翻译** | | My cesarean scar is a little raised. Is this scar hyperplasia? | | When can I start scar care after birth? | | If the scar is red and itchy, can I use scar patches by myself? |
| **Model response (Chinese) / 模型回答（中文）** | | 可能是，与个人体质有关。 | | 拆线后2周可开始用硅胶贴或疤痕膏。 | | 可以，避免抓挠。 |
| **English translation of model response / 模型回答英文翻译** | | Possible hypertrophic scar; related to individual predisposition. | | Silicone sheets/gel can be started 2 weeks after suture removal. | | Yes, but avoid scratching. |
| **Overall evaluation item / 整体评价项目** | | | **Score / 分数** | | **Comments / 备注** | |
| **Patient Satisfaction / 患者满意度** | | | 3 | |  | |
| **Patient Understandability / 患者可理解性** | | | 4 | |  | |
| **Patient Usefulness / 患者实用性** | | | 3 | |  | |
| **Clinician Correctness / 医生评分：正确性** | | | 3 | |  | |
| **Clinician Completeness / 医生评分：完整性** | | | 3 | |  | |
| **Clinician Readability / 医生评分：可读性** | | | 5 | |  | |
| **Clinician Helpfulness / 医生评分：帮助性** | | | 3 | |  | |
| **Clinician Safety / 医生评分：安全性** | | | 3 | |  | |

**Patient 50**

| **Patient ID / 患者编号** | **Group / 模型组别** | | | | | |
| --- | --- | --- | --- | --- | --- | --- |
| P50 | DeepSeek V3 | | | | | |
| **General model response or patient education material (Chinese) / 模型总体回答或患者教育材料（中文）** | | | | | | |
| 剖宫产后不建议全天绑收腹带。长时间绑腹带会影响局部血液循环、不利于切口透气，还可能因腹内压增高导致盆腔器官脱垂或加重腹直肌分离。正确的方法是：每天间断使用2-6小时，比如下床活动或长时间站立时绑，睡觉和卧床休息时取下。收腹带不宜过紧，以能伸进一个手指为宜。如果绑上后感到胸闷、气短、恶心或切口疼痛加剧，说明过紧或时间过长，应立即松开并停用。想要恢复腹部形态，更有效的方法是产后42天后进行核心肌群训练和盆底康复。 | | | | | | |
| **English translation of the Chinese general response / 中文总体回答英文翻译** | | | | | | |
| Do not wear an abdominal binder all day after a cesarean section. Prolonged use impairs blood circulation, reduces incision aeration, and may increase intra-abdominal pressure, worsening pelvic organ prolapse or diastasis recti. Use it intermittently for 2-6 hours daily, e.g., when walking or standing; remove it when lying down or sleeping. It should not be too tight (one finger should fit under it). If you experience chest tightness, shortness of breath, nausea, or increased incision pain, loosen or remove it immediately. For abdominal shape recovery, core muscle training and pelvic floor rehabilitation after 42 days are more effective. | | | | | | |
| **Item / 项目** | | **Q1 / 问题1** | | **Q2 / 问题2** | | **Q3 / 问题3** |
| **Patient question (Chinese original) / 患者原始问题（中文）** | | 剖宫产后可以一直绑收腹带吗？ | | 收腹带会不会影响切口透气和恢复？ | | 如果绑上后觉得胸闷或肚子疼，应该停用吗？ |
| **English translation of patient question / 患者问题英文翻译** | | Can I keep wearing an abdominal binder all the time after a cesarean birth? | | Will an abdominal binder affect incision ventilation and healing? | | If I feel chest tightness or abdominal pain after wearing it, should I stop using it? |
| **Model response (Chinese) / 模型回答（中文）** | | 不建议全天绑，每天间断使用2-6小时。 | | 过紧或长期使用会影响。 | | 立即停用。 |
| **English translation of model response / 模型回答英文翻译** | | Not 24/7; use intermittently 2-6 hours/day. | | Too tight or prolonged use impairs ventilation and healing. | | Stop immediately. |
| **Overall evaluation item / 整体评价项目** | | | **Score / 分数** | | **Comments / 备注** | |
| **Patient Satisfaction / 患者满意度** | | | 3 | |  | |
| **Patient Understandability / 患者可理解性** | | | 4 | |  | |
| **Patient Usefulness / 患者实用性** | | | 3 | |  | |
| **Clinician Correctness / 医生评分：正确性** | | | 3 | |  | |
| **Clinician Completeness / 医生评分：完整性** | | | 3 | |  | |
| **Clinician Readability / 医生评分：可读性** | | | 5 | |  | |
| **Clinician Helpfulness / 医生评分：帮助性** | | | 3 | |  | |
| **Clinician Safety / 医生评分：安全性** | | | 3 | |  | |

**Patient 51**

| **Patient ID / 患者编号** | **Group / 模型组别** | | | | | |
| --- | --- | --- | --- | --- | --- | --- |
| P51 | DeepSeek V3 | | | | | |
| **General model response or patient education material (Chinese) / 模型总体回答或患者教育材料（中文）** | | | | | | |
| 产后肚子松松垮垮，不要急着练核心。首先应自测或请医生评估是否存在腹直肌分离。自测方法：仰卧屈膝，头肩轻轻抬起，用手指在肚脐上下方探查腹直肌中间的缝隙。如果缝隙超过2指宽（约2厘米），即为腹直肌分离。在分离未闭合前，绝对不要做卷腹、仰卧起坐、平板支撑等传统腹肌训练，这些动作会加剧分离。正确做法是：先进行腹式呼吸、盆底肌收缩和低负荷核心训练（如四点跪位的鸟狗式）。如果产后3-6个月腹部仍未改善，应到康复科接受专业治疗。 | | | | | | |
| **English translation of the Chinese general response / 中文总体回答英文翻译** | | | | | | |
| If your abdomen is still loose after birth, do not rush into core exercises. First, self-test or have a professional assess for diastasis recti. Lie on your back with knees bent, gently lift your head and shoulders, and feel the gap between the rectus muscles above and below the belly button. If the gap exceeds 2 finger-widths (about 2 cm), it is diastasis recti. Before the gap closes, avoid crunches, sit-ups, planks — these worsen separation. Instead, perform diaphragmatic breathing, pelvic floor contractions, and low-load exercises like quadruped bird-dog. If no improvement by 3-6 months, seek professional rehabilitation. | | | | | | |
| **Item / 项目** | | **Q1 / 问题1** | | **Q2 / 问题2** | | **Q3 / 问题3** |
| **Patient question (Chinese original) / 患者原始问题（中文）** | | 我产后肚子松松垮垮，可以马上练核心吗？ | | 腹直肌分离和单纯肚子大怎么区分？ | | 哪些运动可能会加重腹直肌分离？ |
| **English translation of patient question / 患者问题英文翻译** | | My abdomen feels loose and flabby after birth. Can I start core exercises right away? | | How can I distinguish diastasis recti from simply having a larger abdomen? | | Which exercises may worsen diastasis recti? |
| **Model response (Chinese) / 模型回答（中文）** | | 不可以，先评估腹直肌分离。 | | 分离>2指需康复，单纯肚子大多为脂肪。 | | 卷腹、仰卧起坐等。 |
| **English translation of model response / 模型回答英文翻译** | | No, assess diastasis recti first. | | Separation >2 fingers needs rehab; simple big belly is usually fat. | | Crunches, sit-ups, etc. |
| **Overall evaluation item / 整体评价项目** | | | **Score / 分数** | | **Comments / 备注** | |
| **Patient Satisfaction / 患者满意度** | | | 3 | |  | |
| **Patient Understandability / 患者可理解性** | | | 4 | |  | |
| **Patient Usefulness / 患者实用性** | | | 3 | |  | |
| **Clinician Correctness / 医生评分：正确性** | | | 3 | |  | |
| **Clinician Completeness / 医生评分：完整性** | | | 3 | |  | |
| **Clinician Readability / 医生评分：可读性** | | | 5 | |  | |
| **Clinician Helpfulness / 医生评分：帮助性** | | | 3 | |  | |
| **Clinician Safety / 医生评分：安全性** | | | 3 | |  | |

**Patient 52**

| **Patient ID / 患者编号** | **Group / 模型组别** | | | | | |
| --- | --- | --- | --- | --- | --- | --- |
| P52 | DeepSeek V3 | | | | | |
| **General model response or patient education material (Chinese) / 模型总体回答或患者教育材料（中文）** | | | | | | |
| 产后耻骨联合处疼痛，翻身、走路或上下楼梯时加重，常见于耻骨联合分离或韧带损伤，与孕期松弛素分泌有关。多数人在产后2-4周内自行缓解。您可以热敷疼痛区域，每次15-20分钟，每天2-3次；也可使用骨盆带提供外支撑，但不要绑得过紧，以免影响活动。注意避免双腿大幅度分开的动作（如深蹲、跨坐），侧卧时在两膝之间夹一个枕头。如果疼痛导致走路困难、无法翻身，或伴有发烧、腿麻、大小便异常，请及时到骨科或康复科检查，必要时拍X线或MRI。 | | | | | | |
| **English translation of the Chinese general response / 中文总体回答英文翻译** | | | | | | |
| Pain over the pubic symphysis that worsens with turning, walking, or climbing stairs is often due to pubic symphysis diastasis or ligament injury related to relaxin. Most cases resolve within 2-4 weeks. Apply warm compresses for 15-20 minutes 2-3 times daily. A pelvic belt can provide external support but do not overtighten. Avoid wide leg movements (deep squatting, straddling). Place a pillow between your knees when side-lying. If pain prevents walking or turning, or is accompanied by fever, leg numbness, or bladder/bowel changes, see an orthopedist or rehabilitation specialist for evaluation. | | | | | | |
| **Item / 项目** | | **Q1 / 问题1** | | **Q2 / 问题2** | | **Q3 / 问题3** |
| **Patient question (Chinese original) / 患者原始问题（中文）** | | 我产后耻骨联合处疼，翻身也疼，正常吗？ | | 耻骨痛可以热敷或用骨盆带吗？ | | 如果走路困难或者疼痛加重，需要检查吗？ |
| **English translation of patient question / 患者问题英文翻译** | | I have pain around the pubic symphysis after birth, and turning over also hurts. Is this normal? | | Can I use warm compresses or a pelvic belt for pubic pain? | | If walking becomes difficult or the pain worsens, do I need an examination? |
| **Model response (Chinese) / 模型回答（中文）** | | 常见，与松弛素有关，多休息。 | | 可以，但骨盆带不宜过紧。 | | 需要，骨科或康复科评估。 |
| **English translation of model response / 模型回答英文翻译** | | Common due to relaxin; rest more. | | Yes, but pelvic belt not too tight. | | Yes, orthopedics/rehab assessment. |
| **Overall evaluation item / 整体评价项目** | | | **Score / 分数** | | **Comments / 备注** | |
| **Patient Satisfaction / 患者满意度** | | | 3 | |  | |
| **Patient Understandability / 患者可理解性** | | | 4 | |  | |
| **Patient Usefulness / 患者实用性** | | | 3 | |  | |
| **Clinician Correctness / 医生评分：正确性** | | | 3 | |  | |
| **Clinician Completeness / 医生评分：完整性** | | | 3 | |  | |
| **Clinician Readability / 医生评分：可读性** | | | 5 | |  | |
| **Clinician Helpfulness / 医生评分：帮助性** | | | 3 | |  | |
| **Clinician Safety / 医生评分：安全性** | | | 3 | |  | |

**Patient 53**

| **Patient ID / 患者编号** | **Group / 模型组别** | | | | | |
| --- | --- | --- | --- | --- | --- | --- |
| P53 | DeepSeek V3 | | | | | |
| **General model response or patient education material (Chinese) / 模型总体回答或患者教育材料（中文）** | | | | | | |
| 顺产后尾骨疼，坐位时尤其明显，多半是分娩时胎头压迫尾骨所致，少数情况下可能发生尾骨骨折或脱位。多数产妇在产后4-6周内疼痛会自然缓解。您可以购买中空坐垫（中间镂空，避免尾骨直接受压）来减轻不适。坐下时身体稍前倾，避免仰坐。如果疼痛持续超过一个月仍不减轻，或坐立不安、无法正常坐姿，建议拍尾骨侧位X线片排除骨折。即便有轻微骨折，通常也是保守治疗（坐垫、止痛药、避免久坐），极少需要手术。同时注意保持大便通畅，避免排便时用力加重疼痛。 | | | | | | |
| **English translation of the Chinese general response / 中文总体回答英文翻译** | | | | | | |
| Coccyx pain after vaginal delivery, especially when sitting, is often due to pressure from the baby’s head. Rarely, it may be a coccyx fracture or dislocation. Most women recover naturally within 4-6 weeks. Use a donut-shaped cushion (hollow in the middle) to relieve direct pressure. Lean slightly forward when sitting. If pain persists more than one month or severely affects sitting, a lateral X-ray of the coccyx can rule out fracture. Even minor fractures are managed conservatively with cushions, pain relievers, and avoiding prolonged sitting. Keep stools soft to avoid straining. | | | | | | |
| **Item / 项目** | | **Q1 / 问题1** | | **Q2 / 问题2** | | **Q3 / 问题3** |
| **Patient question (Chinese original) / 患者原始问题（中文）** | | 顺产后尾骨疼，坐着特别难受，是不是受伤了？ | | 尾骨疼可以用坐垫缓解吗？ | | 如果疼痛持续一个月还不好，需要拍片吗？ |
| **English translation of patient question / 患者问题英文翻译** | | After a vaginal birth, my tailbone hurts and sitting is very uncomfortable. Could it be injured? | | Can I use a cushion to relieve tailbone pain? | | If the pain has not improved after one month, do I need an X-ray? |
| **Model response (Chinese) / 模型回答（中文）** | | 可能分娩压迫所致，多数自愈。 | | 可用中空坐垫。 | | 需要，排除骨折。 |
| **English translation of model response / 模型回答英文翻译** | | Likely from delivery pressure; usually self-resolves. | | Use a hollow seat cushion. | | Yes, to rule out fracture. |
| **Overall evaluation item / 整体评价项目** | | | **Score / 分数** | | **Comments / 备注** | |
| **Patient Satisfaction / 患者满意度** | | | 3 | |  | |
| **Patient Understandability / 患者可理解性** | | | 4 | |  | |
| **Patient Usefulness / 患者实用性** | | | 3 | |  | |
| **Clinician Correctness / 医生评分：正确性** | | | 3 | |  | |
| **Clinician Completeness / 医生评分：完整性** | | | 3 | |  | |
| **Clinician Readability / 医生评分：可读性** | | | 5 | |  | |
| **Clinician Helpfulness / 医生评分：帮助性** | | | 3 | |  | |
| **Clinician Safety / 医生评分：安全性** | | | 3 | |  | |

**Patient 54**

| **Patient ID / 患者编号** | **Group / 模型组别** | | | | | |
| --- | --- | --- | --- | --- | --- | --- |
| P54 | DeepSeek V3 | | | | | |
| **General model response or patient education material (Chinese) / 模型总体回答或患者教育材料（中文）** | | | | | | |
| 产后手腕疼，抱孩子时加重，大拇指活动受限，很可能是“妈妈手”（狭窄性腱鞘炎）。这是因为激素变化和频繁抱娃姿势不当导致肌腱和腱鞘水肿、摩擦。治疗首先应减少诱发动作：抱宝宝时五指并拢、用手臂和躯干力量，避免只用拇指和手腕。可以佩戴护腕或拇指固定夹板，每天外涂双氯芬酸凝胶（哺乳期可用，注意洗手）。如果疼痛严重，可在医生指导下局部注射激素。同时，调整抱姿：让宝宝靠近身体，用前臂托住头颈，另一只手托臀部，分散力量。若保守治疗2-4周无效，需到骨科或康复科进一步处理。 | | | | | | |
| **English translation of the Chinese general response / 中文总体回答英文翻译** | | | | | | |
| Postpartum wrist pain worse with holding the baby and limited thumb movement is often de Quervain’s tenosynovitis (“mother’s thumb”). It results from hormonal changes and improper positioning causing tendon sheath inflammation. First, reduce aggravating movements: hold the baby with fingers together and use arm/trunk strength, avoiding thumb-only lifts. Wear a wrist/thumb splint. Topical diclofenac gel is safe during breastfeeding (wash hands after use). For severe pain, local steroid injection may be considered. Adjust holding technique: bring baby close, support head/neck with forearm and buttocks with other hand. If no improvement in 2-4 weeks, see an orthopedist. | | | | | | |
| **Item / 项目** | | **Q1 / 问题1** | | **Q2 / 问题2** | | **Q3 / 问题3** |
| **Patient question (Chinese original) / 患者原始问题（中文）** | | 我产后手腕疼，抱孩子时更明显，是腱鞘炎吗？ | | 哺乳期手腕疼可以贴膏药吗？ | | 怎样抱宝宝才能减少手腕负担？ |
| **English translation of patient question / 患者问题英文翻译** | | My wrist hurts after birth and it is more obvious when I hold the baby. Is this tenosynovitis? | | Can I apply medicated patches for wrist pain while breastfeeding? | | How should I hold the baby to reduce the burden on my wrists? |
| **Model response (Chinese) / 模型回答（中文）** | | 很可能是“妈妈手”，减少抱娃、戴护腕。 | | 可外用，避免含麝香成分。 | | 用手臂和躯干力量，不用手腕。 |
| **English translation of model response / 模型回答英文翻译** | | Likely de Quervain’s tenosynovitis; reduce lifting and wear splint. | | Yes, but avoid musk-containing patches. | | Use arm and trunk strength, avoid wrist strain. |
| **Overall evaluation item / 整体评价项目** | | | **Score / 分数** | | **Comments / 备注** | |
| **Patient Satisfaction / 患者满意度** | | | 3 | |  | |
| **Patient Understandability / 患者可理解性** | | | 4 | |  | |
| **Patient Usefulness / 患者实用性** | | | 3 | |  | |
| **Clinician Correctness / 医生评分：正确性** | | | 3 | |  | |
| **Clinician Completeness / 医生评分：完整性** | | | 3 | |  | |
| **Clinician Readability / 医生评分：可读性** | | | 5 | |  | |
| **Clinician Helpfulness / 医生评分：帮助性** | | | 3 | |  | |
| **Clinician Safety / 医生评分：安全性** | | | 3 | |  | |

**Patient 55**

| **Patient ID / 患者编号** | **Group / 模型组别** | | | | | |
| --- | --- | --- | --- | --- | --- | --- |
| P55 | DeepSeek V3 | | | | | |
| **General model response or patient education material (Chinese) / 模型总体回答或患者教育材料（中文）** | | | | | | |
| 乳房胀得很硬、宝宝吸不出来，这是典型的生理性乳胀。处理方法：喂奶前用温毛巾敷乳房2-3分钟，然后用手从乳房外围向乳头方向轻轻打圈按摩，同时用拇指和食指在乳晕周围做“反向按压软化”，使乳晕变软便于宝宝含接。如果宝宝仍然吸不出，可以使用吸奶器，每次不超过15-20分钟，避免损伤乳腺。喂奶后冷敷15分钟减轻水肿。注意不要暴力揉搓，否则会加重炎症。如果乳房出现红斑、发热（体温≥38.5℃）、全身酸痛，可能已发展为急性乳腺炎，请及时就医，不要中断哺乳。 | | | | | | |
| **English translation of the Chinese general response / 中文总体回答英文翻译** | | | | | | |
| Hard, engorged breast with poor latch is classic physiological engorgement. Before feeding, apply warm compresses for 2-3 minutes, then gently massage from outer breast toward the nipple. Perform “reverse pressure softening” on the areola to make it softer and easier for the baby to latch. If the baby still cannot suck, use a pump for 15-20 minutes per session. After feeding, apply cold compresses for 15 minutes to reduce edema. Do not knead forcefully — it can worsen inflammation. If you develop a red patch on the breast, fever (≥38.5°C), or body aches, this may be acute mastitis; seek medical care without stopping breastfeeding. | | | | | | |
| **Item / 项目** | | **Q1 / 问题1** | | **Q2 / 问题2** | | **Q3 / 问题3** |
| **Patient question (Chinese original) / 患者原始问题（中文）** | | 我乳房胀得很硬但宝宝吸不出来，怎么办？ | | 堵奶时用吸奶器吸多久比较合适？ | | 如果乳房有红斑并且发热，是不是要看医生？ |
| **English translation of patient question / 患者问题英文翻译** | | My breasts are very hard and swollen, but the baby cannot suck the milk out. What should I do? | | When I have a blocked duct, how long is it appropriate to use a breast pump? | | If my breast has red patches and I have a fever, should I see a doctor? |
| **Model response (Chinese) / 模型回答（中文）** | | 反向按压软化乳晕后再喂。 | | 每次不超过15-20分钟。 | | 是，可能乳腺炎。 |
| **English translation of model response / 模型回答英文翻译** | | Reverse pressure softening then feed. | | No more than 15-20 minutes per session. | | Yes, possible mastitis. |
| **Overall evaluation item / 整体评价项目** | | | **Score / 分数** | | **Comments / 备注** | |
| **Patient Satisfaction / 患者满意度** | | | 3 | |  | |
| **Patient Understandability / 患者可理解性** | | | 4 | |  | |
| **Patient Usefulness / 患者实用性** | | | 3 | |  | |
| **Clinician Correctness / 医生评分：正确性** | | | 3 | |  | |
| **Clinician Completeness / 医生评分：完整性** | | | 3 | |  | |
| **Clinician Readability / 医生评分：可读性** | | | 5 | |  | |
| **Clinician Helpfulness / 医生评分：帮助性** | | | 3 | |  | |
| **Clinician Safety / 医生评分：安全性** | | | 3 | |  | |

**Patient 56**

| **Patient ID / 患者编号** | **Group / 模型组别** | | | | | |
| --- | --- | --- | --- | --- | --- | --- |
| P56 | DeepSeek V3 | | | | | |
| **General model response or patient education material (Chinese) / 模型总体回答或患者教育材料（中文）** | | | | | | |
| 乳房硬块揉开后又反复出现，通常是引流不畅所致，常见于喂奶姿势固定、宝宝含接不良或奶水过多的产妇。建议您每次喂奶时变换不同体位（如摇篮式、足球式、侧卧式），让宝宝的下巴对准硬块方向。不要用力按压深部硬块，以免损伤乳腺组织，可以用轻柔按摩和冷热交替敷来辅助。如果硬块持续超过1周不消，或者同一位置反复出现肿块，应做乳腺超声检查，排除囊肿、脓肿或纤维腺瘤。同时注意检查有无乳头白泡，如有可用温水湿敷后让宝宝吸开，不要自行挑破。 | | | | | | |
| **English translation of the Chinese general response / 中文总体回答英文翻译** | | | | | | |
| Recurrence of a breast lump after it has resolved usually indicates poor drainage, common with fixed feeding positions, poor latch, or oversupply. Change breastfeeding positions (cradle, football, side-lying) so the baby’s chin points toward the lump. Do not press deep lumps hard — this can injure breast tissue. Gentle massage and alternating warm/cold compresses may help. If a lump persists more than 1 week or repeatedly appears in the same spot, get a breast ultrasound to rule out cyst, abscess, or fibroadenoma. Also check for white blebs on the nipple; if present, apply warm compresses and have the baby suck — do not puncture yourself. | | | | | | |
| **Item / 项目** | | **Q1 / 问题1** | | **Q2 / 问题2** | | **Q3 / 问题3** |
| **Patient question (Chinese original) / 患者原始问题（中文）** | | 乳房硬块揉开后又反复出现，是不是喂奶方式不对？ | | 硬块位置很深，自己按压会不会损伤乳腺？ | | 如果硬块一直不消，需要做乳腺超声吗？ |
| **English translation of patient question / 患者问题英文翻译** | | A breast lump comes back repeatedly after being relieved. Is my breastfeeding method wrong? | | The lump is very deep. Could pressing it by myself damage the breast tissue? | | If the lump does not go away, do I need a breast ultrasound? |
| **Model response (Chinese) / 模型回答（中文）** | | 常见于引流不畅，调整喂奶姿势和频率。 | | 不要用力按压，以免损伤。 | | 需要，超过1周不消应做超声。 |
| **English translation of model response / 模型回答英文翻译** | | Often due to poor drainage; adjust position and frequency. | | Do not press hard to avoid gland damage. | | Yes, if persists >1 week. |
| **Overall evaluation item / 整体评价项目** | | | **Score / 分数** | | **Comments / 备注** | |
| **Patient Satisfaction / 患者满意度** | | | 3 | |  | |
| **Patient Understandability / 患者可理解性** | | | 4 | |  | |
| **Patient Usefulness / 患者实用性** | | | 3 | |  | |
| **Clinician Correctness / 医生评分：正确性** | | | 3 | |  | |
| **Clinician Completeness / 医生评分：完整性** | | | 3 | |  | |
| **Clinician Readability / 医生评分：可读性** | | | 5 | |  | |
| **Clinician Helpfulness / 医生评分：帮助性** | | | 3 | |  | |
| **Clinician Safety / 医生评分：安全性** | | | 3 | |  | |

**Patient 57**

| **Patient ID / 患者编号** | **Group / 模型组别** | | | | | |
| --- | --- | --- | --- | --- | --- | --- |
| P57 | DeepSeek V3 | | | | | |
| **General model response or patient education material (Chinese) / 模型总体回答或患者教育材料（中文）** | | | | | | |
| 急性乳腺炎刚好又复发，常见原因包括：乳汁未完全排空、乳头有微小破损导致细菌反复感染、宝宝口腔细菌、或者抗生素疗程不足。乳腺炎期间，可以继续让宝宝吸吮患侧乳房，这有助于排空乳汁、缓解炎症；如果疼痛剧烈，可先喂健侧，待奶阵出现后再换到患侧。反复发作的乳腺炎需要重新评估哺乳姿势和含接技巧，建议咨询母乳喂养门诊或专科护士，必要时做乳汁细菌培养和药敏试验，选择敏感抗生素并完成足疗程治疗。同时注意休息、多喝水，不要自行提前停药。 | | | | | | |
| **English translation of the Chinese general response / 中文总体回答英文翻译** | | | | | | |
| Recurrent mastitis after recent resolution is often due to incomplete milk removal, cracked nipples allowing bacterial entry, the baby’s oral bacteria, or insufficient antibiotic course. During mastitis, continue nursing on the affected side to empty the breast and reduce inflammation. If too painful, start on the healthy side and switch once the let-down occurs. For recurrent mastitis, re-evaluate feeding position and latch, consult a lactation clinic, and consider breastmilk culture and sensitivity to guide antibiotic selection. Complete the full course. Also rest, hydrate, and do not stop antibiotics early on your own. | | | | | | |
| **Item / 项目** | | **Q1 / 问题1** | | **Q2 / 问题2** | | **Q3 / 问题3** |
| **Patient question (Chinese original) / 患者原始问题（中文）** | | 我乳腺炎刚好又复发，可能是什么原因？ | | 乳腺炎期间还能让宝宝吸患侧乳房吗？ | | 反复乳腺炎需要调整哺乳姿势吗？ |
| **English translation of patient question / 患者问题英文翻译** | | My mastitis just improved but then recurred. What could be the reason? | | During mastitis, can I still let the baby suck on the affected breast? | | For recurrent mastitis, do I need to adjust my breastfeeding position? |
| **Model response (Chinese) / 模型回答（中文）** | | 乳汁未排空、乳头损伤或细菌耐药。 | | 可以，疼痛剧烈时可先喂健侧。 | | 需要，并咨询母乳门诊。 |
| **English translation of model response / 模型回答英文翻译** | | Incomplete emptying, nipple damage, or bacterial resistance. | | Yes; if too painful, start from healthy side. | | Yes, and consult lactation clinic. |
| **Overall evaluation item / 整体评价项目** | | | **Score / 分数** | | **Comments / 备注** | |
| **Patient Satisfaction / 患者满意度** | | | 3 | |  | |
| **Patient Understandability / 患者可理解性** | | | 4 | |  | |
| **Patient Usefulness / 患者实用性** | | | 3 | |  | |
| **Clinician Correctness / 医生评分：正确性** | | | 3 | |  | |
| **Clinician Completeness / 医生评分：完整性** | | | 3 | |  | |
| **Clinician Readability / 医生评分：可读性** | | | 5 | |  | |
| **Clinician Helpfulness / 医生评分：帮助性** | | | 3 | |  | |
| **Clinician Safety / 医生评分：安全性** | | | 3 | |  | |

**Patient 58**

| **Patient ID / 患者编号** | **Group / 模型组别** | | | | | |
| --- | --- | --- | --- | --- | --- | --- |
| P58 | DeepSeek V3 | | | | | |
| **General model response or patient education material (Chinese) / 模型总体回答或患者教育材料（中文）** | | | | | | |
| 产后奶量不够，绝大多数女性都可以通过科学方法追奶成功，极少有人真正“体质不适合”。追奶的核心是“多排多产”：每天亲喂或吸奶至少8-12次，夜间也要保证2-3次，因为夜间催乳素分泌最旺盛。判断宝宝吃饱的客观指标是：每天6片以上沉甸甸的湿尿布、2-5次黄色糊状大便、每周体重增长150-200克。混合喂养本身不会影响追奶，但添加配方奶时一定要先让宝宝吸吮乳房每侧至少10-15分钟，再补喂配方奶。不要因为加了奶粉就减少吸吮次数。如果体重增长慢，请在医生或哺乳指导帮助下评估含接和吞咽效率。 | | | | | | |
| **English translation of the Chinese general response / 中文总体回答英文翻译** | | | | | | |
| The vast majority of women can successfully increase milk supply, and very few are truly “not suited” for breastfeeding. The principle is “more removal, more production”: nurse or pump at least 8-12 times daily, including 2-3 times at night when prolactin peaks. Objective signs of adequate intake: ≥6 heavy wet diapers per day, 2-5 yellow seedy stools, and weight gain of 150-200 g per week. Mixed feeding does not preclude increasing supply, but always allow the baby to suck at each breast for at least 10-15 minutes before offering formula. Do not reduce suckling frequency because of formula. If weight gain is slow, have latch and swallowing efficiency assessed. | | | | | | |
| **Item / 项目** | | **Q1 / 问题1** | | **Q2 / 问题2** | | **Q3 / 问题3** |
| **Patient question (Chinese original) / 患者原始问题（中文）** | | 我奶量一直不够，是不是体质不适合母乳喂养？ | | 每天需要吸奶几次才有助于追奶？ | | 宝宝体重增长慢时应该怎么判断奶量够不够？ |
| **English translation of patient question / 患者问题英文翻译** | | My milk supply has always been insufficient. Does this mean my body is not suitable for breastfeeding? | | How many times a day should I pump to help increase milk supply? | | If the baby gains weight slowly, how should I judge whether the milk supply is enough? |
| **Model response (Chinese) / 模型回答（中文）** | | 不是，可通过频繁吸吮增加。 | | 每天8-12次。 | | 看尿量、便次，必要时补充配方奶。 |
| **English translation of model response / 模型回答英文翻译** | | No, you can increase supply with frequent nursing. | | 8-12 times per day. | | Check urine output and stool frequency; supplement if needed. |
| **Overall evaluation item / 整体评价项目** | | | **Score / 分数** | | **Comments / 备注** | |
| **Patient Satisfaction / 患者满意度** | | | 3 | |  | |
| **Patient Understandability / 患者可理解性** | | | 4 | |  | |
| **Patient Usefulness / 患者实用性** | | | 3 | |  | |
| **Clinician Correctness / 医生评分：正确性** | | | 3 | |  | |
| **Clinician Completeness / 医生评分：完整性** | | | 3 | |  | |
| **Clinician Readability / 医生评分：可读性** | | | 5 | |  | |
| **Clinician Helpfulness / 医生评分：帮助性** | | | 3 | |  | |
| **Clinician Safety / 医生评分：安全性** | | | 3 | |  | |

**Patient 59**

| **Patient ID / 患者编号** | **Group / 模型组别** | | | | | |
| --- | --- | --- | --- | --- | --- | --- |
| P59 | DeepSeek V3 | | | | | |
| **General model response or patient education material (Chinese) / 模型总体回答或患者教育材料（中文）** | | | | | | |
| 奶量太多总是漏奶，属于高泌乳素分泌的正常现象，尤其常见于产后早期。您可以使用防溢乳垫，并勤更换。如果奶阵太急导致宝宝呛奶，可以采取半卧位或后躺式喂奶，利用重力减缓流速；也可以在喂奶前用手挤出少量前奶，直到奶流变慢再让宝宝含接。乳房经常涨满确实会增加堵奶和乳腺炎风险，因此不需要过度排空。感觉涨奶时，可以少量挤出到舒服为止，不要完全排空，否则会刺激更多产奶。随着宝宝长大，供需平衡会自然形成，漏奶和胀满会逐渐改善。 | | | | | | |
| **English translation of the Chinese general response / 中文总体回答英文翻译** | | | | | | |
| Oversupply with leaking milk is a normal phenomenon of high prolactin, common early postpartum. Use nursing pads and change them frequently. If a fast let-down makes the baby choke, feed in a semi-reclining or laid-back position to slow flow with gravity, or express a small amount of foremilk before latching. Frequent engorgement increases the risk of blocked ducts and mastitis, so do not over-empty. When feeling full, express just enough to feel comfortable — complete emptying stimulates even more production. As the baby grows, supply will naturally regulate, and leaking/engorgement will improve. | | | | | | |
| **Item / 项目** | | **Q1 / 问题1** | | **Q2 / 问题2** | | **Q3 / 问题3** |
| **Patient question (Chinese original) / 患者原始问题（中文）** | | 我奶太多总是漏奶，正常吗？ | | 奶阵太急宝宝容易呛奶，应该怎么喂？ | | 乳房总是涨满会不会更容易堵奶？ |
| **English translation of patient question / 患者问题英文翻译** | | I have too much milk and it keeps leaking. Is this normal? | | My let-down is too fast and the baby easily chokes. How should I feed the baby? | | Will breasts that are always overfull be more likely to develop blocked ducts? |
| **Model response (Chinese) / 模型回答（中文）** | | 正常，使用防溢乳垫。 | | 半卧位喂奶，或先挤出少量再喂。 | | 会，按需排空即可。 |
| **English translation of model response / 模型回答英文翻译** | | Normal, use nursing pads. | | Feed in semi-reclining position or express a little first. | | Yes, empty on demand. |
| **Overall evaluation item / 整体评价项目** | | | **Score / 分数** | | **Comments / 备注** | |
| **Patient Satisfaction / 患者满意度** | | | 3 | |  | |
| **Patient Understandability / 患者可理解性** | | | 4 | |  | |
| **Patient Usefulness / 患者实用性** | | | 3 | |  | |
| **Clinician Correctness / 医生评分：正确性** | | | 3 | |  | |
| **Clinician Completeness / 医生评分：完整性** | | | 3 | |  | |
| **Clinician Readability / 医生评分：可读性** | | | 5 | |  | |
| **Clinician Helpfulness / 医生评分：帮助性** | | | 3 | |  | |
| **Clinician Safety / 医生评分：安全性** | | | 3 | |  | |

**Patient 60**

| **Patient ID / 患者编号** | **Group / 模型组别** | | | | | |
| --- | --- | --- | --- | --- | --- | --- |
| P60 | DeepSeek V3 | | | | | |
| **General model response or patient education material (Chinese) / 模型总体回答或患者教育材料（中文）** | | | | | | |
| 乳头上的白色小点，喂奶时剧痛，这是乳头白泡，通常是一根乳孔被乳汁沉积或薄层上皮堵塞所致。处理方法是：喂奶前用温水湿敷白泡3-5分钟，然后用干净的纱布轻轻摩擦，再让宝宝用力吸吮，多数可自行破开。不要自己用针挑破，以免感染和形成疤痕。如果白泡持续不破，疼痛影响喂奶，可以请母乳门诊医生在无菌条件下用针挑开。同时注意纠正含接姿势，避免再次发生。白泡打开后，可能会流出少量浓稠乳汁，之后疼痛会迅速缓解。保持乳头清洁干燥，哺乳后涂抹少量母乳即可。 | | | | | | |
| **English translation of the Chinese general response / 中文总体回答英文翻译** | | | | | | |
| A white dot on the nipple that causes severe pain during feeding is a milk bleb — a blocked nipple pore due to dried milk or a thin layer of epithelium. Before feeding, apply warm compresses for 3-5 minutes, then gently rub with a clean gauze and have the baby suck vigorously; most will open spontaneously. Do not puncture yourself with a needle to avoid infection and scarring. If the bleb persists and pain interferes with feeding, a lactation consultant or doctor can open it under sterile conditions. Also correct latch to prevent recurrence. Once opened, thick milk may flow out, and pain quickly resolves. Keep the nipple clean and apply a drop of breastmilk after feeding. | | | | | | |
| **Item / 项目** | | **Q1 / 问题1** | | **Q2 / 问题2** | | **Q3 / 问题3** |
| **Patient question (Chinese original) / 患者原始问题（中文）** | | 我的乳头上有白色小点，喂奶时很疼，是乳头白泡吗？ | | 乳头白泡可以自己挑破吗？ | | 如果乳头疼痛影响喂奶，需要去母乳门诊吗？ |
| **English translation of patient question / 患者问题英文翻译** | | There is a small white spot on my nipple and it hurts when I breastfeed. Is this a milk bleb? | | Can I puncture a milk bleb by myself? | | If nipple pain affects breastfeeding, do I need to visit a lactation clinic? |
| **Model response (Chinese) / 模型回答（中文）** | | 是，温水湿敷后让宝宝吸吮。 | | 不要自己挑，以免感染。 | | 需要。 |
| **English translation of model response / 模型回答英文翻译** | | Yes, apply warm compress then have baby suck. | | Do not poke yourself to avoid infection. | | Yes. |
| **Overall evaluation item / 整体评价项目** | | | **Score / 分数** | | **Comments / 备注** | |
| **Patient Satisfaction / 患者满意度** | | | 3 | |  | |
| **Patient Understandability / 患者可理解性** | | | 4 | |  | |
| **Patient Usefulness / 患者实用性** | | | 3 | |  | |
| **Clinician Correctness / 医生评分：正确性** | | | 3 | |  | |
| **Clinician Completeness / 医生评分：完整性** | | | 3 | |  | |
| **Clinician Readability / 医生评分：可读性** | | | 5 | |  | |
| **Clinician Helpfulness / 医生评分：帮助性** | | | 3 | |  | |
| **Clinician Safety / 医生评分：安全性** | | | 3 | |  | |

**Patient 61**

| **Patient ID / 患者编号** | **Group / 模型组别** | | | | | |
| --- | --- | --- | --- | --- | --- | --- |
| P61 | DeepSeek V3 | | | | | |
| **General model response or patient education material (Chinese) / 模型总体回答或患者教育材料（中文）** | | | | | | |
| 哺乳时乳房针扎样疼痛，不伴有红肿热痛，可能是乳头血管痉挛（雷诺现象），多因寒冷或含接不当引起。如果乳头和宝宝口腔内都有白色凝乳状物，且乳头疼痛呈灼烧感，很可能是念珠菌感染（鹅口疮）。怀疑感染时，不需要停止哺乳，但需要母亲和宝宝同时治疗：母亲在乳头上涂抹制霉菌素或氟康唑（遵医嘱），宝宝口腔用制霉菌素混悬液涂擦。同时注意勤换内衣、煮沸消毒奶瓶和吸奶器配件。如果疼痛持续且抗真菌治疗无效，应进一步排查其他原因。继续哺乳对宝宝有益，不会加重感染。 | | | | | | |
| **English translation of the Chinese general response / 中文总体回答英文翻译** | | | | | | |
| Sharp, stabbing breast pain during breastfeeding without redness or warmth may be nipple vasospasm (Raynaud’s phenomenon), often triggered by cold or poor latch. If both the nipple and baby’s mouth have white patches and pain is burning, suspect Candida infection (thrush). Do not stop breastfeeding; treat both mother and baby simultaneously. Mother applies nystatin or fluconazole (as prescribed) to the nipples; baby uses nystatin oral suspension. Change bras frequently and boil pump parts. If pain persists despite antifungal therapy, investigate other causes. Continuing breastfeeding is beneficial and does not worsen infection. | | | | | | |
| **Item / 项目** | | **Q1 / 问题1** | | **Q2 / 问题2** | | **Q3 / 问题3** |
| **Patient question (Chinese original) / 患者原始问题（中文）** | | 我哺乳时乳房像针扎一样疼，是正常的吗？ | | 乳头和宝宝口腔都有白色东西，会不会是真菌感染？ | | 如果怀疑感染，还能继续母乳喂养吗？ |
| **English translation of patient question / 患者问题英文翻译** | | When I breastfeed, my breast has a needle-like pain. Is this normal? | | There are white patches on both my nipple and the baby's mouth. Could this be a fungal infection? | | If infection is suspected, can I continue breastfeeding? |
| **Model response (Chinese) / 模型回答（中文）** | | 可能为血管痉挛或念珠菌感染，需评估。 | | 很可能是鹅口疮，需同时治疗。 | | 可以，但需遵医嘱用抗真菌药。 |
| **English translation of model response / 模型回答英文翻译** | | Possible vasospasm or Candida infection; need evaluation. | | Likely thrush; treat both mother and baby. | | Yes, but use antifungal medication as prescribed. |
| **Overall evaluation item / 整体评价项目** | | | **Score / 分数** | | **Comments / 备注** | |
| **Patient Satisfaction / 患者满意度** | | | 3 | |  | |
| **Patient Understandability / 患者可理解性** | | | 4 | |  | |
| **Patient Usefulness / 患者实用性** | | | 3 | |  | |
| **Clinician Correctness / 医生评分：正确性** | | | 3 | |  | |
| **Clinician Completeness / 医生评分：完整性** | | | 3 | |  | |
| **Clinician Readability / 医生评分：可读性** | | | 5 | |  | |
| **Clinician Helpfulness / 医生评分：帮助性** | | | 3 | |  | |
| **Clinician Safety / 医生评分：安全性** | | | 3 | |  | |

**Patient 62**

| **Patient ID / 患者编号** | **Group / 模型组别** | | | | | |
| --- | --- | --- | --- | --- | --- | --- |
| P62 | DeepSeek V3 | | | | | |
| **General model response or patient education material (Chinese) / 模型总体回答或患者教育材料（中文）** | | | | | | |
| 哺乳期突然来月经，可能会暂时轻微减少奶量，因为月经前雌激素和孕激素升高会抑制催乳素分泌，但月经结束后奶量通常自行恢复。此时不必焦虑，增加亲喂或吸奶频率即可。月经与恶露的区别：月经有周期性（每个月一次），色鲜红或暗红，持续3-7天，无臭味；恶露则产后初为红色，逐渐变淡，4-6周内干净，无周期性。如果月经量特别大（每小时湿透一片卫生巾，或有大血块）、持续时间超过7天、伴有剧烈腹痛，需到医院检查，排除子宫复旧不良、宫腔残留或其他妇科疾病。 | | | | | | |
| **English translation of the Chinese general response / 中文总体回答英文翻译** | | | | | | |
| A sudden return of menstruation during breastfeeding may temporarily slightly reduce milk supply because premenstrual estrogen and progesterone suppress prolactin. Supply usually recovers after menstruation ends. Do not worry; increase nursing or pumping frequency. Distinguishing menstruation from lochia: menstruation is cyclic (every month), bright or dark red, lasts 3-7 days, no odor. Lochia is red initially, gradually lightens, clears within 4-6 weeks, not cyclic. If bleeding is extremely heavy (soaking a pad per hour or large clots), lasts >7 days, or is accompanied by severe abdominal pain, see a doctor to rule out poor involution, retained products, or other conditions. | | | | | | |
| **Item / 项目** | | **Q1 / 问题1** | | **Q2 / 问题2** | | **Q3 / 问题3** |
| **Patient question (Chinese original) / 患者原始问题（中文）** | | 哺乳期突然来月经，会影响奶量吗？ | | 月经恢复后恶露和月经怎么区分？ | | 如果月经量特别多，需要去医院吗？ |
| **English translation of patient question / 患者问题英文翻译** | | If my period suddenly returns during breastfeeding, will it affect my milk supply? | | After menstruation returns, how can I distinguish lochia from a menstrual period? | | If the menstrual flow is very heavy, do I need to go to the hospital? |
| **Model response (Chinese) / 模型回答（中文）** | | 可能暂时轻微减少，多吸吮可恢复。 | | 月经有周期、色红、无臭味；异常恶露有异味或伴发热。 | | 每小时湿透一片卫生巾需就医。 |
| **English translation of model response / 模型回答英文翻译** | | May temporarily slightly reduce supply; frequent nursing helps. | | Menstruation: cyclic, red, no odor; abnormal lochia has foul smell or fever. | | If soaking a pad per hour, seek care. |
| **Overall evaluation item / 整体评价项目** | | | **Score / 分数** | | **Comments / 备注** | |
| **Patient Satisfaction / 患者满意度** | | | 3 | |  | |
| **Patient Understandability / 患者可理解性** | | | 4 | |  | |
| **Patient Usefulness / 患者实用性** | | | 3 | |  | |
| **Clinician Correctness / 医生评分：正确性** | | | 4 | |  | |
| **Clinician Completeness / 医生评分：完整性** | | | 3 | |  | |
| **Clinician Readability / 医生评分：可读性** | | | 4 | |  | |
| **Clinician Helpfulness / 医生评分：帮助性** | | | 3 | |  | |
| **Clinician Safety / 医生评分：安全性** | | | 4 | |  | |

**Patient 63**

| **Patient ID / 患者编号** | **Group / 模型组别** | | | | | |
| --- | --- | --- | --- | --- | --- | --- |
| P63 | DeepSeek V3 | | | | | |
| **General model response or patient education material (Chinese) / 模型总体回答或患者教育材料（中文）** | | | | | | |
| 即使产后没有来月经，也可能已经恢复排卵。第一次排卵通常出现在第一次月经之前，因此哺乳期同房不避孕就有怀孕的可能。哺乳期不能随便吃复方口服避孕药（含雌激素），因为雌激素会抑制乳汁分泌，甚至导致奶量明显下降。哺乳期安全的避孕方法包括：避孕套、单纯孕激素避孕药（需医生处方）、孕激素宫内节育器（顺产后4-6周、剖宫产后6个月放置）、皮下埋植剂。如果已经同房且没有避孕，可在72小时内放置含铜宫内节育器作为紧急避孕，或口服左炔诺孕酮（不影响哺乳）。 | | | | | | |
| **English translation of the Chinese general response / 中文总体回答英文翻译** | | | | | | |
| Ovulation can occur before the first menstrual period postpartum, so you can become pregnant even without a period. Do not randomly take combined oral contraceptives (containing estrogen) during breastfeeding, as estrogen suppresses milk production. Breastfeeding-safe contraceptive methods include condoms, progestin-only pills (prescription), progestin IUD (placed 4-6 weeks after vaginal delivery or 6 months after C-section), and implants. If you have had unprotected intercourse, a copper IUD can be inserted within 72 hours as emergency contraception, or you can take levonorgestrel (does not affect breastfeeding). | | | | | | |
| **Item / 项目** | | **Q1 / 问题1** | | **Q2 / 问题2** | | **Q3 / 问题3** |
| **Patient question (Chinese original) / 患者原始问题（中文）** | | 我还在哺乳，没有来月经也会排卵吗？ | | 哺乳期避孕药可以随便吃吗？ | | 如果已经同房但没有避孕，需要怎么处理？ |
| **English translation of patient question / 患者问题英文翻译** | | I am still breastfeeding and have not had my period. Can I still ovulate? | | Can I take contraceptive pills freely during breastfeeding? | | If I have already had sex without contraception, what should I do? |
| **Model response (Chinese) / 模型回答（中文）** | | 会，需避孕。 | | 不能，含雌激素的会抑制泌乳。 | | 72小时内可放置宫内节育器或口服紧急避孕药（左炔诺孕酮）。 |
| **English translation of model response / 模型回答英文翻译** | | Yes, ovulation can occur; need contraception. | | No, estrogen-containing pills reduce milk supply. | | IUD or emergency contraceptive (levonorgestrel) within 72 hours. |
| **Overall evaluation item / 整体评价项目** | | | **Score / 分数** | | **Comments / 备注** | |
| **Patient Satisfaction / 患者满意度** | | | 3 | |  | |
| **Patient Understandability / 患者可理解性** | | | 4 | |  | |
| **Patient Usefulness / 患者实用性** | | | 3 | |  | |
| **Clinician Correctness / 医生评分：正确性** | | | 3 | |  | |
| **Clinician Completeness / 医生评分：完整性** | | | 3 | |  | |
| **Clinician Readability / 医生评分：可读性** | | | 5 | |  | |
| **Clinician Helpfulness / 医生评分：帮助性** | | | 3 | |  | |
| **Clinician Safety / 医生评分：安全性** | | | 3 | |  | |

**Patient 64**

| **Patient ID / 患者编号** | **Group / 模型组别** | | | | | |
| --- | --- | --- | --- | --- | --- | --- |
| P64 | DeepSeek V3 | | | | | |
| **General model response or patient education material (Chinese) / 模型总体回答或患者教育材料（中文）** | | | | | | |
| 产后42天复查时，可以做宫颈癌筛查（TCT和HPV），但建议等到恶露完全干净、没有明显出血时再进行，以免血液干扰结果。如果产后有阴道炎症状（白带增多、颜色黄绿、有异味、外阴瘙痒），最好先治疗后再做筛查，否则炎症细胞可能影响判读。白带异常伴有瘙痒，不应等到42天，应提前到妇科就诊，查白带常规明确感染类型（细菌、滴虫或念珠菌），及时用药。如果正在使用阴道栓剂，需停药3天后再做宫颈筛查。哺乳期治疗阴道炎安全有效，不影响宝宝。 | | | | | | |
| **English translation of the Chinese general response / 中文总体回答英文翻译** | | | | | | |
| At the 42-day postpartum check-up, cervical cancer screening (TCT and HPV) can be done, but it is best to wait until lochia has completely cleared to avoid blood interfering with results. If you have symptoms of vaginitis (increased discharge, yellow-green, foul odor, itching), treat the infection first before screening, as inflammatory cells may affect interpretation. Abnormal discharge with itching should not wait until 42 days; see a gynecologist earlier for wet mount and appropriate treatment. If using vaginal suppositories, stop for 3 days before screening. Vaginitis treatment during breastfeeding is safe and effective. | | | | | | |
| **Item / 项目** | | **Q1 / 问题1** | | **Q2 / 问题2** | | **Q3 / 问题3** |
| **Patient question (Chinese original) / 患者原始问题（中文）** | | 产后42天复查可以顺便做宫颈筛查吗？ | | 产后有阴道炎症状，会影响复查结果吗？ | | 如果白带异常伴有瘙痒，需要提前就诊吗？ |
| **English translation of patient question / 患者问题英文翻译** | | Can I have cervical screening during the 42-day postpartum check-up? | | If I have symptoms of vaginitis after birth, will it affect the follow-up results? | | If abnormal discharge is accompanied by itching, do I need to seek care earlier? |
| **Model response (Chinese) / 模型回答（中文）** | | 可以，若无明显出血。 | | 不影响取样，但最好先治疗。 | | 需要。 |
| **English translation of model response / 模型回答英文翻译** | | Yes, if no active bleeding. | | Does not affect sampling, but better to treat first. | | Yes. |
| **Overall evaluation item / 整体评价项目** | | | **Score / 分数** | | **Comments / 备注** | |
| **Patient Satisfaction / 患者满意度** | | | 3 | |  | |
| **Patient Understandability / 患者可理解性** | | | 4 | |  | |
| **Patient Usefulness / 患者实用性** | | | 3 | |  | |
| **Clinician Correctness / 医生评分：正确性** | | | 3 | |  | |
| **Clinician Completeness / 医生评分：完整性** | | | 3 | |  | |
| **Clinician Readability / 医生评分：可读性** | | | 5 | |  | |
| **Clinician Helpfulness / 医生评分：帮助性** | | | 3 | |  | |
| **Clinician Safety / 医生评分：安全性** | | | 3 | |  | |

**Patient 65**

| **Patient ID / 患者编号** | **Group / 模型组别** | | | | | |
| --- | --- | --- | --- | --- | --- | --- |
| P65 | DeepSeek V3 | | | | | |
| **General model response or patient education material (Chinese) / 模型总体回答或患者教育材料（中文）** | | | | | | |
| 排便时阴道口有压迫感，甚至感觉有东西要掉出来，这通常是盆底支持结构减弱的表现，不一定就是子宫脱垂，也可能是阴道前壁或后壁膨出。建议您产后42天复查时主动要求做盆底功能评估（POP-Q评分）。盆底肌力差确实可能影响性生活质量，表现为同房时阴道松弛、感觉减退或疼痛，但通过盆底康复（凯格尔训练、生物反馈、电刺激）可以有效改善。盆底康复不一定必须在医院做：轻度患者可在指导下居家使用阴道哑铃或手机App辅助训练；中重度或康复效果不佳者，建议在医院进行规范化治疗。关键是坚持，至少3个月。 | | | | | | |
| **English translation of the Chinese general response / 中文总体回答英文翻译** | | | | | | |
| A sensation of pressure or bulging at the vaginal opening during defecation usually indicates weakened pelvic floor support, not necessarily uterine prolapse — it could be anterior or posterior vaginal wall prolapse. At the 42-day check-up, ask for a pelvic floor assessment (POP-Q score). Weak pelvic floor muscles may affect sexual satisfaction (looseness, reduced sensation, or pain), but pelvic floor rehabilitation (Kegel exercises, biofeedback, electrical stimulation) can effectively improve it. Rehabilitation does not necessarily need to be in a hospital: mild cases can use vaginal cones or apps at home with guidance; moderate to severe or non-responsive cases benefit from supervised hospital treatment. Consistency for at least 3 months is key. | | | | | | |
| **Item / 项目** | | **Q1 / 问题1** | | **Q2 / 问题2** | | **Q3 / 问题3** |
| **Patient question (Chinese original) / 患者原始问题（中文）** | | 我产后排便时阴道口有压迫感，是盆底问题吗？ | | 盆底肌力差会不会影响以后性生活？ | | 盆底康复一定要在医院做吗？ |
| **English translation of patient question / 患者问题英文翻译** | | When I have a bowel movement after birth, I feel pressure at the vaginal opening. Is this a pelvic floor problem? | | Can weak pelvic floor muscles affect future sexual life? | | Does pelvic floor rehabilitation have to be done in the hospital? |
| **Model response (Chinese) / 模型回答（中文）** | | 提示盆底支持减弱，建议评估。 | | 可能影响性满意度，康复可改善。 | | 不一定，轻症可居家训练，严重需医院。 |
| **English translation of model response / 模型回答英文翻译** | | Suggests weakened pelvic support; get assessed. | | May affect sexual satisfaction; rehab helps. | | Not necessarily; mild cases can do home exercises, severe need hospital. |
| **Overall evaluation item / 整体评价项目** | | | **Score / 分数** | | **Comments / 备注** | |
| **Patient Satisfaction / 患者满意度** | | | 3 | |  | |
| **Patient Understandability / 患者可理解性** | | | 4 | |  | |
| **Patient Usefulness / 患者实用性** | | | 3 | |  | |
| **Clinician Correctness / 医生评分：正确性** | | | 3 | |  | |
| **Clinician Completeness / 医生评分：完整性** | | | 3 | |  | |
| **Clinician Readability / 医生评分：可读性** | | | 5 | |  | |
| **Clinician Helpfulness / 医生评分：帮助性** | | | 3 | |  | |
| **Clinician Safety / 医生评分：安全性** | | | 3 | |  | |

**Patient 66**

| **Patient ID / 患者编号** | **Group / 模型组别** | | | | | |
| --- | --- | --- | --- | --- | --- | --- |
| P66 | DeepSeek V3 | | | | | |
| **General model response or patient education material (Chinese) / 模型总体回答或患者教育材料（中文）** | | | | | | |
| 哺乳期阴道干涩是非常常见的生理现象，原因是高水平的催乳素抑制了雌激素分泌，导致阴道黏膜变薄、分泌物减少。这并不意味着“病”，也不需要激素治疗。同房疼痛时，使用水溶性润滑剂是最简单有效的办法，对宝宝完全安全。充分前戏（15-20分钟）也可以帮助身体自然润滑。如果疼痛一直持续，即使使用润滑剂也无法缓解，需要检查：盆底肌是否过度紧张（高张力性盆底功能障碍）、有无阴道炎或疤痕挛缩。必要时可进行盆底物理治疗或阴道扩张训练。请不要因为疼痛而回避同房，积极处理才能改善。 | | | | | | |
| **English translation of the Chinese general response / 中文总体回答英文翻译** | | | | | | |
| Vaginal dryness during breastfeeding is a very common physiological phenomenon caused by high prolactin suppressing estrogen, leading to thinner vaginal mucosa and reduced secretions. This is not a disease and does not need hormone therapy. For painful intercourse, water-based lubricants are the simplest and most effective solution, completely safe for the baby. Adequate foreplay (15-20 minutes) also helps natural lubrication. If pain persists despite lubricant, evaluate for overactive pelvic floor (high-tone dysfunction), vaginitis, or scar contracture. Pelvic floor physical therapy or vaginal dilators may help. Do not avoid sex because of pain; proactive management improves the condition. | | | | | | |
| **Item / 项目** | | **Q1 / 问题1** | | **Q2 / 问题2** | | **Q3 / 问题3** |
| **Patient question (Chinese original) / 患者原始问题（中文）** | | 哺乳期阴道干涩很明显，是激素变化造成的吗？ | | 同房疼痛可以使用润滑剂吗？ | | 如果疼痛一直持续，需要检查哪些问题？ |
| **English translation of patient question / 患者问题英文翻译** | | I have obvious vaginal dryness while breastfeeding. Is this caused by hormonal changes? | | Can I use lubricant for pain during intercourse? | | If the pain continues, what problems need to be checked? |
| **Model response (Chinese) / 模型回答（中文）** | | 是，低雌激素所致，正常。 | | 可以，水溶性润滑剂安全有效。 | | 检查盆底、阴道炎、激素水平。 |
| **English translation of model response / 模型回答英文翻译** | | Yes, due to low estrogen, normal. | | Yes, water-based lubricant is safe and effective. | | Check pelvic floor, vaginitis, hormone levels. |
| **Overall evaluation item / 整体评价项目** | | | **Score / 分数** | | **Comments / 备注** | |
| **Patient Satisfaction / 患者满意度** | | | 3 | |  | |
| **Patient Understandability / 患者可理解性** | | | 4 | |  | |
| **Patient Usefulness / 患者实用性** | | | 3 | |  | |
| **Clinician Correctness / 医生评分：正确性** | | | 3 | |  | |
| **Clinician Completeness / 医生评分：完整性** | | | 3 | |  | |
| **Clinician Readability / 医生评分：可读性** | | | 5 | |  | |
| **Clinician Helpfulness / 医生评分：帮助性** | | | 3 | |  | |
| **Clinician Safety / 医生评分：安全性** | | | 3 | |  | |

**Patient 67**

| **Patient ID / 患者编号** | **Group / 模型组别** | | | | | |
| --- | --- | --- | --- | --- | --- | --- |
| P67 | DeepSeek V3 | | | | | |
| **General model response or patient education material (Chinese) / 模型总体回答或患者教育材料（中文）** | | | | | | |
| 咳嗽、打喷嚏时漏尿，是典型的压力性尿失禁，自己练习凯格尔运动非常有用，但前提是方法正确。正确做法：找到盆底肌（像憋尿或憋气的感觉），收缩时腹部、臀部、大腿不应用力。每天做3组，每组8-12次，每次收缩保持3-5秒，放松3-5秒。坚持8-12周通常可见改善。如果漏尿超过3个月仍无好转，或漏尿量增多、影响日常生活，建议到盆底康复中心做尿动力检查，评估尿道闭合压和膀胱功能，再制定个体化治疗方案。同时避免提重物、久蹲、慢性咳嗽等增加腹压的行为。 | | | | | | |
| **English translation of the Chinese general response / 中文总体回答英文翻译** | | | | | | |
| Leaking urine with coughing or sneezing is typical stress urinary incontinence. Kegel exercises are very helpful if done correctly. Locate the pelvic floor muscles (like holding urine or gas). Contract without using abs, buttocks, or thighs. Do 3 sets daily, 8-12 contractions per set, each hold 3-5 seconds, relax 3-5 seconds. Improvement is usually seen after 8-12 weeks. If leakage persists more than 3 months or worsens, a urodynamic study may be needed to assess urethral closure pressure and bladder function, guiding individualized treatment. Also avoid heavy lifting, prolonged squatting, and chronic coughing. | | | | | | |
| **Item / 项目** | | **Q1 / 问题1** | | **Q2 / 问题2** | | **Q3 / 问题3** |
| **Patient question (Chinese original) / 患者原始问题（中文）** | | 我咳嗽或打喷嚏会漏尿，自己练凯格尔有用吗？ | | 凯格尔运动每天做多少次合适？ | | 漏尿超过三个月还没好，需要做尿动力检查吗？ |
| **English translation of patient question / 患者问题英文翻译** | | I leak urine when I cough or sneeze. Is practicing Kegel exercises by myself useful? | | How many times a day is appropriate for Kegel exercises? | | If urine leakage has not improved after more than three months, do I need urodynamic testing? |
| **Model response (Chinese) / 模型回答（中文）** | | 有用，需正确练习。 | | 每天3组，每组8-12次，每次收缩3-5秒。 | | 建议做。 |
| **English translation of model response / 模型回答英文翻译** | | Yes, if done correctly. | | 3 sets of 8-12 contractions per day, each hold 3-5 seconds. | | Yes. |
| **Overall evaluation item / 整体评价项目** | | | **Score / 分数** | | **Comments / 备注** | |
| **Patient Satisfaction / 患者满意度** | | | 3 | |  | |
| **Patient Understandability / 患者可理解性** | | | 4 | |  | |
| **Patient Usefulness / 患者实用性** | | | 3 | |  | |
| **Clinician Correctness / 医生评分：正确性** | | | 3 | |  | |
| **Clinician Completeness / 医生评分：完整性** | | | 3 | |  | |
| **Clinician Readability / 医生评分：可读性** | | | 5 | |  | |
| **Clinician Helpfulness / 医生评分：帮助性** | | | 3 | |  | |
| **Clinician Safety / 医生评分：安全性** | | | 3 | |  | |

**Patient 68**

| **Patient ID / 患者编号** | **Group / 模型组别** | | | | | |
| --- | --- | --- | --- | --- | --- | --- |
| P68 | DeepSeek V3 | | | | | |
| **General model response or patient education material (Chinese) / 模型总体回答或患者教育材料（中文）** | | | | | | |
| 感觉阴道口有东西鼓出来，很可能是盆腔器官脱垂（POP），最常见的是膀胱膨出、子宫脱垂或直肠膨出。轻度脱垂（脱垂最远处在处女膜缘内）通常没有明显症状，可以通过盆底康复训练改善。您需要做的是：产后42天复查时请医生做POP-Q评分。对于轻中度脱垂，坚持凯格尔运动、避免增加腹压的行为（提重物、便秘、慢性咳嗽），多数可以稳定或好转。如果脱垂到达或超过处女膜缘，或引起排尿排便困难、同房障碍，医生可能建议使用子宫托。子宫托是一种硅胶支撑环，可自行取戴，需要定期随访。极少数重度脱垂需手术治疗。 | | | | | | |
| **English translation of the Chinese general response / 中文总体回答英文翻译** | | | | | | |
| A bulging sensation at the vaginal opening is likely pelvic organ prolapse (POP), most commonly cystocele, uterine prolapse, or rectocele. Mild prolapse (the lowest point within the hymen) often has no symptoms and can improve with pelvic floor rehabilitation. At the 42-day check-up, have a POP-Q score done. For mild to moderate prolapse, adherence to Kegel exercises and avoiding increased intra-abdominal pressure (heavy lifting, constipation, chronic cough) usually stabilizes or improves it. If prolapse reaches or protrudes beyond the hymen, or causes voiding/defecation difficulty or pain with intercourse, a pessary (silicone support device) may be recommended, requiring regular follow-up. Severe cases may need surgery. | | | | | | |
| **Item / 项目** | | **Q1 / 问题1** | | **Q2 / 问题2** | | **Q3 / 问题3** |
| **Patient question (Chinese original) / 患者原始问题（中文）** | | 我感觉阴道口有东西鼓出来，是不是脱垂？ | | 轻度脱垂可以通过盆底康复恢复吗？ | | 什么情况下需要使用子宫托或进一步治疗？ |
| **English translation of patient question / 患者问题英文翻译** | | I feel something bulging out at the vaginal opening. Is this prolapse? | | Can mild prolapse improve through pelvic floor rehabilitation? | | In what situations is a pessary or further treatment needed? |
| **Model response (Chinese) / 模型回答（中文）** | | 可能，需妇科检查。 | | 可以改善。 | | 症状明显或脱垂超过处女膜缘。 |
| **English translation of model response / 模型回答英文翻译** | | Possible; need gynecologic exam. | | Yes, can improve. | | If symptoms significant or prolapse beyond hymen. |
| **Overall evaluation item / 整体评价项目** | | | **Score / 分数** | | **Comments / 备注** | |
| **Patient Satisfaction / 患者满意度** | | | 3 | |  | |
| **Patient Understandability / 患者可理解性** | | | 4 | |  | |
| **Patient Usefulness / 患者实用性** | | | 3 | |  | |
| **Clinician Correctness / 医生评分：正确性** | | | 3 | |  | |
| **Clinician Completeness / 医生评分：完整性** | | | 3 | |  | |
| **Clinician Readability / 医生评分：可读性** | | | 5 | |  | |
| **Clinician Helpfulness / 医生评分：帮助性** | | | 3 | |  | |
| **Clinician Safety / 医生评分：安全性** | | | 3 | |  | |

**Patient 69**

| **Patient ID / 患者编号** | **Group / 模型组别** | | | | | |
| --- | --- | --- | --- | --- | --- | --- |
| P69 | DeepSeek V3 | | | | | |
| **General model response or patient education material (Chinese) / 模型总体回答或患者教育材料（中文）** | | | | | | |
| 侧切疤痕摸起来硬硬的，这是因为愈合过程中胶原蛋白无序沉积形成的疤痕组织。通常在产后3-6个月内会逐渐软化、颜色变浅。疤痕紧绷感可能会影响同房时的舒适度，特别是疤痕位于阴道后壁或侧壁时。可以通过疤痕按摩来改善：洗净双手，用润滑剂（如维生素E油、水溶性润滑剂）涂在疤痕上，由轻到重按压、打圈、横向拉伸，每天5-10分钟。如果按摩后疼痛加重或疤痕处有硬结、按压剧痛，需要复查，排除疤痕内异症（异位的子宫内膜在疤痕内生长，周期性疼痛）或神经瘤。这些情况可在门诊手术切除。 | | | | | | |
| **English translation of the Chinese general response / 中文总体回答英文翻译** | | | | | | |
| A hard episiotomy scar is due to disorganized collagen deposition during healing. It usually softens and lightens in color within 3-6 months. Scar tightness may affect comfort during intercourse, especially if the scar is on the posterior or lateral vaginal wall. Scar massage can help: wash hands, apply lubricant (vitamin E oil or water-based lubricant), then press, circle, and horizontally stretch the scar with gradually increasing pressure for 5-10 minutes daily. If massage increases pain, or if a tender nodule develops, see a doctor to rule out scar endometriosis (ectopic endometrial tissue causing cyclic pain) or neuroma. These can be excised in a clinic procedure. | | | | | | |
| **Item / 项目** | | **Q1 / 问题1** | | **Q2 / 问题2** | | **Q3 / 问题3** |
| **Patient question (Chinese original) / 患者原始问题（中文）** | | 侧切疤痕摸着硬硬的，会慢慢变软吗？ | | 会阴疤痕紧绷会不会影响以后同房？ | | 疤痕按压很痛时需要复查吗？ |
| **English translation of patient question / 患者问题英文翻译** | | The episiotomy scar feels hard to the touch. Will it gradually become softer? | | Will tightness of the perineal scar affect future intercourse? | | If pressing the scar is very painful, do I need a follow-up examination? |
| **Model response (Chinese) / 模型回答（中文）** | | 会，通常6个月内改善。 | | 可能，可用按摩和扩张器缓解。 | | 需要，排除疤痕内异症或神经瘤。 |
| **English translation of model response / 模型回答英文翻译** | | Yes, usually within 6 months. | | Possible; massage and dilators help. | | Yes, to rule out endometriosis or neuroma. |
| **Overall evaluation item / 整体评价项目** | | | **Score / 分数** | | **Comments / 备注** | |
| **Patient Satisfaction / 患者满意度** | | | 3 | |  | |
| **Patient Understandability / 患者可理解性** | | | 4 | |  | |
| **Patient Usefulness / 患者实用性** | | | 3 | |  | |
| **Clinician Correctness / 医生评分：正确性** | | | 3 | |  | |
| **Clinician Completeness / 医生评分：完整性** | | | 3 | |  | |
| **Clinician Readability / 医生评分：可读性** | | | 5 | |  | |
| **Clinician Helpfulness / 医生评分：帮助性** | | | 3 | |  | |
| **Clinician Safety / 医生评分：安全性** | | | 3 | |  | |

**Patient 70**

| **Patient ID / 患者编号** | **Group / 模型组别** | | | | | |
| --- | --- | --- | --- | --- | --- | --- |
| P70 | DeepSeek V3 | | | | | |
| **General model response or patient education material (Chinese) / 模型总体回答或患者教育材料（中文）** | | | | | | |
| 剖宫产后咳嗽时切口疼痛是正常的，因为咳嗽会突然增加腹内压，牵拉到尚未完全愈合的腹壁各层组织。您可以采取“夹板固定法”：咳嗽前用手或小枕头轻轻压住切口区域，然后再咳。抱宝宝时，避免直接用腹部力量：先坐稳，让宝宝靠近胸口，用前臂和躯干托住，必要时使用哺乳枕或婴儿背带分散压力。如果切口突然出现撕裂样剧烈疼痛，尤其是伴有局部隆起、渗血或渗液增多，可能是切口裂开或血肿，请立即就医。正常情况下，随着伤口愈合，咳嗽痛会在术后2-4周明显减轻。 | | | | | | |
| **English translation of the Chinese general response / 中文总体回答英文翻译** | | | | | | |
| Pain with coughing after a cesarean section is normal because coughing suddenly increases intra-abdominal pressure, straining the healing abdominal layers. Use “splinting”: press a hand or small pillow firmly over the incision before coughing. When holding the baby, avoid abdominal strain: sit firmly, bring the baby close to your chest, support with forearms and torso, and use a nursing pillow or carrier to distribute weight. If you experience sudden tearing pain, especially with a bulging lump or increased bleeding/drainage, this could indicate wound dehiscence or hematoma — seek immediate medical care. Normally, cough-related pain improves significantly within 2-4 weeks. | | | | | | |
| **Item / 项目** | | **Q1 / 问题1** | | **Q2 / 问题2** | | **Q3 / 问题3** |
| **Patient question (Chinese original) / 患者原始问题（中文）** | | 剖宫产后咳嗽时切口疼，正常吗？ | | 抱宝宝时怎样避免牵拉腹部切口？ | | 如果切口突然有撕裂样疼痛，需要马上就医吗？ |
| **English translation of patient question / 患者问题英文翻译** | | After a cesarean birth, my incision hurts when I cough. Is this normal? | | How can I avoid pulling on the abdominal incision when holding the baby? | | If the incision suddenly has tearing-like pain, do I need immediate medical care? |
| **Model response (Chinese) / 模型回答（中文）** | | 正常，用手轻压切口再咳。 | | 用腿部力量，腹部不用力。 | | 需要。 |
| **English translation of model response / 模型回答英文翻译** | | Normal; splint incision with hand when coughing. | | Use leg strength, avoid abdominal strain. | | Yes. |
| **Overall evaluation item / 整体评价项目** | | | **Score / 分数** | | **Comments / 备注** | |
| **Patient Satisfaction / 患者满意度** | | | 3 | |  | |
| **Patient Understandability / 患者可理解性** | | | 4 | |  | |
| **Patient Usefulness / 患者实用性** | | | 3 | |  | |
| **Clinician Correctness / 医生评分：正确性** | | | 3 | |  | |
| **Clinician Completeness / 医生评分：完整性** | | | 3 | |  | |
| **Clinician Readability / 医生评分：可读性** | | | 5 | |  | |
| **Clinician Helpfulness / 医生评分：帮助性** | | | 3 | |  | |
| **Clinician Safety / 医生评分：安全性** | | | 3 | |  | |

**Patient 71**

| **Patient ID / 患者编号** | **Group / 模型组别** | | | | | |
| --- | --- | --- | --- | --- | --- | --- |
| P71 | DeepSeek V3 | | | | | |
| **General model response or patient education material (Chinese) / 模型总体回答或患者教育材料（中文）** | | | | | | |
| 产后反复低烧（37.5-38℃），没有感冒症状，需要警惕产后感染，常见部位包括子宫内膜炎、乳腺炎、泌尿道感染或切口感染。低烧可能与乳房胀痛有关，特别是如果伴有乳房红肿、硬块，提示可能是亚临床乳腺炎。建议您每天监测体温4次，记录最高值。如果体温超过38.5℃，或低烧持续超过3天，或伴有恶露臭味、下腹压痛、尿痛、切口红肿，请尽快到医院就诊，查血常规、C反应蛋白、尿常规和盆腔B超。不要自行服用退烧药掩盖病情。明确诊断后，医生会开具哺乳期安全的抗生素。 | | | | | | |
| **English translation of the Chinese general response / 中文总体回答英文翻译** | | | | | | |
| Recurrent low-grade fever (37.5-38°C) without cold symptoms after birth warrants suspicion of postpartum infection, including endometritis, mastitis, UTI, or wound infection. Low fever may relate to breast engorgement, especially if accompanied by breast redness or lumps (subclinical mastitis). Monitor temperature 4 times daily and record the peak. If temperature exceeds 38.5°C, low fever persists >3 days, or you develop foul lochia, lower abdominal tenderness, dysuria, or incisional redness, see a doctor promptly for CBC, CRP, urinalysis, and pelvic ultrasound. Do not take fever reducers to mask the illness. Breastfeeding-safe antibiotics can be prescribed once diagnosed. | | | | | | |
| **Item / 项目** | | **Q1 / 问题1** | | **Q2 / 问题2** | | **Q3 / 问题3** |
| **Patient question (Chinese original) / 患者原始问题（中文）** | | 产后低烧反反复复，但没有感冒症状，怎么办？ | | 低烧和乳房胀痛有关吗？ | | 产后发热到多少度需要尽快去医院？ |
| **English translation of patient question / 患者问题英文翻译** | | I have repeated low-grade fever after birth but no cold symptoms. What should I do? | | Can low-grade fever be related to breast engorgement? | | How high does a postpartum fever need to be before I should go to the hospital promptly? |
| **Model response (Chinese) / 模型回答（中文）** | | 查血常规、C反应蛋白及盆腔超声。 | | 可能，需排除乳腺炎。 | | ≥38.5℃或持续超过24小时。 |
| **English translation of model response / 模型回答英文翻译** | | Get CBC, CRP, and pelvic ultrasound. | | Possible; rule out mastitis. | | ≥38.5°C or lasting >24 hours. |
| **Overall evaluation item / 整体评价项目** | | | **Score / 分数** | | **Comments / 备注** | |
| **Patient Satisfaction / 患者满意度** | | | 3 | |  | |
| **Patient Understandability / 患者可理解性** | | | 4 | |  | |
| **Patient Usefulness / 患者实用性** | | | 3 | |  | |
| **Clinician Correctness / 医生评分：正确性** | | | 3 | |  | |
| **Clinician Completeness / 医生评分：完整性** | | | 3 | |  | |
| **Clinician Readability / 医生评分：可读性** | | | 5 | |  | |
| **Clinician Helpfulness / 医生评分：帮助性** | | | 3 | |  | |
| **Clinician Safety / 医生评分：安全性** | | | 3 | |  | |

**Patient 72**

| **Patient ID / 患者编号** | **Group / 模型组别** | | | | | |
| --- | --- | --- | --- | --- | --- | --- |
| P72 | DeepSeek V3 | | | | | |
| **General model response or patient education material (Chinese) / 模型总体回答或患者教育材料（中文）** | | | | | | |
| 产后下腹按压痛，恶露有臭味，是产褥感染的典型表现，最常见的是子宫内膜炎。除了压痛和异味，您可能还会有发热、恶露量增多、颜色发暗或脓性。子宫复旧不良的主要表现是恶露持续时间长（超过6周不干净）、子宫底下降缓慢或按压时子宫较大、下腹坠胀感。如果您被诊断为产褥感染，医生会使用抗生素治疗。绝大多数抗生素（如头孢类、青霉素类、克林霉素）在哺乳期是安全的，不需要停止哺乳。请告知医生您正在哺乳，医生会选择不影响宝宝的药物。治疗期间继续喂奶，反而有助于排空子宫和乳汁，促进恢复。 | | | | | | |
| **English translation of the Chinese general response / 中文总体回答英文翻译** | | | | | | |
| Lower abdominal tenderness with foul-smelling lochia is typical of puerperal infection, most commonly endometritis. Other signs may include fever, increased lochia, dark or purulent discharge. Poor uterine involution presents as prolonged lochia (>6 weeks), slow descent of the fundus, and pelvic heaviness. If diagnosed with puerperal infection, antibiotics will be prescribed. Most antibiotics (cephalosporins, penicillins, clindamycin) are safe during breastfeeding and do not require stopping. Tell your doctor you are breastfeeding so they can choose baby-safe medications. Continuing to nurse or pump helps empty the uterus and breasts, aiding recovery. | | | | | | |
| **Item / 项目** | | **Q1 / 问题1** | | **Q2 / 问题2** | | **Q3 / 问题3** |
| **Patient question (Chinese original) / 患者原始问题（中文）** | | 我产后下腹按压疼，恶露也有点味道，是感染吗？ | | 子宫复旧不良会有哪些表现？ | | 如果医生说需要抗感染治疗，还能哺乳吗？ |
| **English translation of patient question / 患者问题英文翻译** | | My lower abdomen is tender when pressed after birth, and my lochia has some odor. Is this an infection? | | What are the signs of poor uterine involution? | | If the doctor says I need anti-infection treatment, can I still breastfeed? |
| **Model response (Chinese) / 模型回答（中文）** | | 高度怀疑产褥感染，需抗生素治疗。 | | 恶露不净、子宫大、下腹坠。 | | 大多数抗生素安全，告知医生继续哺乳。 |
| **English translation of model response / 模型回答英文翻译** | | Highly suspect puerperal infection; need antibiotics. | | Prolonged lochia, enlarged uterus, pelvic heaviness. | | Most antibiotics are safe; inform doctor you are breastfeeding. |
| **Overall evaluation item / 整体评价项目** | | | **Score / 分数** | | **Comments / 备注** | |
| **Patient Satisfaction / 患者满意度** | | | 3 | |  | |
| **Patient Understandability / 患者可理解性** | | | 4 | |  | |
| **Patient Usefulness / 患者实用性** | | | 3 | |  | |
| **Clinician Correctness / 医生评分：正确性** | | | 3 | |  | |
| **Clinician Completeness / 医生评分：完整性** | | | 3 | |  | |
| **Clinician Readability / 医生评分：可读性** | | | 5 | |  | |
| **Clinician Helpfulness / 医生评分：帮助性** | | | 3 | |  | |
| **Clinician Safety / 医生评分：安全性** | | | 3 | |  | |

**Patient 73**

| **Patient ID / 患者编号** | **Group / 模型组别** | | | | | |
| --- | --- | --- | --- | --- | --- | --- |
| P73 | DeepSeek V3 | | | | | |
| **General model response or patient education material (Chinese) / 模型总体回答或患者教育材料（中文）** | | | | | | |
| 会阴伤口裂开一点点（小于1厘米），如果裂口表浅、没有活动性出血、没有脓性分泌物，且周围皮肤不红不肿，一般可以自己长好。您需要保持局部清洁干燥：每次便后温水冲洗，轻轻蘸干，可以继续温水坐浴每天2次。避免久坐、下蹲、提重物和便秘。如果裂口较深、出血明显、流脓或裂口边缘发黑，则需到医院处理，可能需要清创后重新缝合。注意观察有没有发热、疼痛加重。如果裂口愈合不良超过2周，也请复查。绝大多数轻度裂开通过保守治疗可愈合，无需过度担心。 | | | | | | |
| **English translation of the Chinese general response / 中文总体回答英文翻译** | | | | | | |
| If a perineal wound is gaping slightly (<1 cm) with a superficial opening, no active bleeding, no pus, and no surrounding redness/swelling, it usually heals on its own. Keep the area clean and dry: rinse with warm water after each bowel movement, pat dry gently, and continue warm sitz baths twice daily. Avoid prolonged sitting, squatting, heavy lifting, and constipation. If the gap is deep, bleeding significantly, purulent, or the edges are black, seek medical attention — possible debridement and resuturing. Monitor for fever or worsening pain. If poor healing lasts >2 weeks, have it rechecked. Most minor dehiscence heals with conservative care. | | | | | | |
| **Item / 项目** | | **Q1 / 问题1** | | **Q2 / 问题2** | | **Q3 / 问题3** |
| **Patient question (Chinese original) / 患者原始问题（中文）** | | 会阴伤口裂开一点点，会自己长好吗？ | | 伤口裂开后还能用坐浴吗？ | | 如果伤口出血或流脓，需要重新缝合吗？ |
| **English translation of patient question / 患者问题英文翻译** | | My perineal wound has opened slightly. Can it heal on its own? | | Can I still use a sitz bath after the wound has opened? | | If the wound bleeds or drains pus, does it need to be sutured again? |
| **Model response (Chinese) / 模型回答（中文）** | | 小于1厘米、无感染可自愈。 | | 可以，温水坐浴每日2次。 | | 需要清创缝合。 |
| **English translation of model response / 模型回答英文翻译** | | If gap <1cm without infection, may heal on its own. | | Yes, sitz bath twice daily. | | Yes, debridement and resuturing. |
| **Overall evaluation item / 整体评价项目** | | | **Score / 分数** | | **Comments / 备注** | |
| **Patient Satisfaction / 患者满意度** | | | 3 | |  | |
| **Patient Understandability / 患者可理解性** | | | 4 | |  | |
| **Patient Usefulness / 患者实用性** | | | 3 | |  | |
| **Clinician Correctness / 医生评分：正确性** | | | 3 | |  | |
| **Clinician Completeness / 医生评分：完整性** | | | 3 | |  | |
| **Clinician Readability / 医生评分：可读性** | | | 5 | |  | |
| **Clinician Helpfulness / 医生评分：帮助性** | | | 3 | |  | |
| **Clinician Safety / 医生评分：安全性** | | | 3 | |  | |

**Patient 74**

| **Patient ID / 患者编号** | **Group / 模型组别** | | | | | |
| --- | --- | --- | --- | --- | --- | --- |
| P74 | DeepSeek V3 | | | | | |
| **General model response or patient education material (Chinese) / 模型总体回答或患者教育材料（中文）** | | | | | | |
| 剖宫产切口边缘发红、发热，是局部感染的早期表现。如果红肿范围小（局限在针眼周围）、没有渗液，可以每天用碘伏消毒1次，保持干燥。如果红肿范围扩大、触碰疼痛加剧，或出现黄色渗液、发热，说明感染加重，需马上到医院处理，医生可能会切开引流并口服或静脉使用抗生素。切勿自行挤压、热敷或涂抹药膏。切口消毒可以使用碘伏，但不要过度擦拭，每天一次即可，之后用无菌纱布覆盖。注意观察切口有无硬结、波动感（脓液形成）。早期处理效果好，绝大多数不会留下严重后遗症。 | | | | | | |
| **English translation of the Chinese general response / 中文总体回答英文翻译** | | | | | | |
| Redness and warmth around a cesarean incision edge suggest early local infection. If the redness is limited to small areas around suture sites with no drainage, clean with povidone-iodine once daily and keep dry. If redness spreads, pain increases, or yellow drainage or fever develops, the infection is worsening — seek medical care promptly. The doctor may need to incise and drain pus and prescribe antibiotics. Do not squeeze, apply heat, or use ointments on your own. Povidone-iodine can be used once daily, then cover with sterile gauze. Watch for fluctuance (pus collection). Early treatment is highly effective and rarely leaves serious sequelae. | | | | | | |
| **Item / 项目** | | **Q1 / 问题1** | | **Q2 / 问题2** | | **Q3 / 问题3** |
| **Patient question (Chinese original) / 患者原始问题（中文）** | | 剖宫产切口边缘发红发热，是不是感染？ | | 切口消毒可以每天用碘伏吗？ | | 如果切口流黄色液体，需要马上复查吗？ |
| **English translation of patient question / 患者问题英文翻译** | | The edge of my cesarean incision is red and warm. Is this an infection? | | Can I disinfect the incision with povidone-iodine every day? | | If yellow fluid comes out of the incision, do I need an immediate follow-up visit? |
| **Model response (Chinese) / 模型回答（中文）** | | 提示感染，需就医。 | | 可以，但避免过度潮湿。 | | 需要，可能脓肿。 |
| **English translation of model response / 模型回答英文翻译** | | Suggests infection; seek evaluation. | | Yes, but avoid excessive moisture. | | Yes, possible abscess. |
| **Overall evaluation item / 整体评价项目** | | | **Score / 分数** | | **Comments / 备注** | |
| **Patient Satisfaction / 患者满意度** | | | 3 | |  | |
| **Patient Understandability / 患者可理解性** | | | 4 | |  | |
| **Patient Usefulness / 患者实用性** | | | 3 | |  | |
| **Clinician Correctness / 医生评分：正确性** | | | 3 | |  | |
| **Clinician Completeness / 医生评分：完整性** | | | 3 | |  | |
| **Clinician Readability / 医生评分：可读性** | | | 5 | |  | |
| **Clinician Helpfulness / 医生评分：帮助性** | | | 3 | |  | |
| **Clinician Safety / 医生评分：安全性** | | | 3 | |  | |

**Patient 75**

| **Patient ID / 患者编号** | **Group / 模型组别** | | | | | |
| --- | --- | --- | --- | --- | --- | --- |
| P75 | DeepSeek V3 | | | | | |
| **General model response or patient education material (Chinese) / 模型总体回答或患者教育材料（中文）** | | | | | | |
| 剖宫产切口下方摸到硬块，可能是正常瘢痕增生、皮下血肿、血清肿或腹壁子宫内膜异位症。如果硬块不痛、不红、不发热，且随着时间推移逐渐变小或质地变软，多半是正常瘢痕或吸收中的血清肿，可以观察。如果硬块突然增大、出现红肿热痛、按压剧痛，或伴有发热，需要做超声检查明确性质。如果是血肿或脓肿，可能需要穿刺引流。如果是腹壁子宫内膜异位症（表现为与月经周期相关的疼痛性硬块），则需手术切除。建议您在产后42天复查时让医生触诊，必要时预约超声。 | | | | | | |
| **English translation of the Chinese general response / 中文总体回答英文翻译** | | | | | | |
| A hard lump under a cesarean incision may be normal scar proliferation, subcutaneous hematoma, seroma, or abdominal wall endometriosis. If the lump is painless, not red or warm, and gradually shrinks or softens, it is likely a normal scar or resolving seroma; observation is fine. If it suddenly enlarges, becomes red, warm, painful, or you develop fever, an ultrasound is needed. Hematoma or abscess may require drainage. Abdominal wall endometriosis (a painful lump that cycles with menstruation) requires surgical excision. Have the doctor palpate it at your 42-day check-up and arrange an ultrasound if needed. | | | | | | |
| **Item / 项目** | | **Q1 / 问题1** | | **Q2 / 问题2** | | **Q3 / 问题3** |
| **Patient question (Chinese original) / 患者原始问题（中文）** | | 剖宫产切口下面有硬块，是血肿还是正常瘢痕？ | | 切口附近有鼓包，按着不太疼，需要观察吗？ | | 如果硬块变大或发热，需要做超声吗？ |
| **English translation of patient question / 患者问题英文翻译** | | There is a hard lump under my cesarean incision. Is it a hematoma or a normal scar? | | There is a bulge near the incision that is not very painful when pressed. Can I observe it? | | If the lump becomes larger or I develop fever, do I need an ultrasound? |
| **Model response (Chinese) / 模型回答（中文）** | | 需超声鉴别。 | | 可观察，若增大或疼痛则就诊。 | | 需要。 |
| **English translation of model response / 模型回答英文翻译** | | Need ultrasound to differentiate. | | Observe; if enlarges or painful, see doctor. | | Yes. |
| **Overall evaluation item / 整体评价项目** | | | **Score / 分数** | | **Comments / 备注** | |
| **Patient Satisfaction / 患者满意度** | | | 3 | |  | |
| **Patient Understandability / 患者可理解性** | | | 4 | |  | |
| **Patient Usefulness / 患者实用性** | | | 3 | |  | |
| **Clinician Correctness / 医生评分：正确性** | | | 3 | |  | |
| **Clinician Completeness / 医生评分：完整性** | | | 3 | |  | |
| **Clinician Readability / 医生评分：可读性** | | | 5 | |  | |
| **Clinician Helpfulness / 医生评分：帮助性** | | | 3 | |  | |
| **Clinician Safety / 医生评分：安全性** | | | 3 | |  | |

**Patient 76**

| **Patient ID / 患者编号** | **Group / 模型组别** | | | | | |
| --- | --- | --- | --- | --- | --- | --- |
| P76 | DeepSeek V3 | | | | | |
| **General model response or patient education material (Chinese) / 模型总体回答或患者教育材料（中文）** | | | | | | |
| 产后医生开的抗生素，大多数在哺乳期是安全的，不需要停止哺乳。常见的哺乳期安全抗生素包括青霉素类、头孢菌素类、大环内酯类（阿奇霉素、克拉霉素）、克林霉素等。您需要告知医生您正在哺乳，医生会避开有潜在风险的药物（如四环素类长期使用、氯霉素、喹诺酮类）。服药后，可以尽量在喂奶后马上服药，或者在下次喂奶前间隔2-4小时，以降低乳汁中的药物浓度。不要因为担心而拒绝用药，延误感染治疗对您和宝宝都有害。如果医生特别嘱咐暂停哺乳，届时再按医嘱执行。 | | | | | | |
| **English translation of the Chinese general response / 中文总体回答英文翻译** | | | | | | |
| Most antibiotics prescribed postpartum are safe during breastfeeding and do not require stopping. Commonly safe ones include penicillins, cephalosporins, macrolides (azithromycin, clarithromycin), and clindamycin. Inform your doctor that you are breastfeeding so they avoid potentially risky drugs (long-term tetracyclines, chloramphenicol, fluoroquinolones). To further reduce milk drug levels, take the medication immediately after feeding or leave a 2-4 hour gap before the next feed. Do not refuse antibiotics out of fear — delaying treatment harms both you and your baby. Only stop breastfeeding if specifically instructed by your doctor. | | | | | | |
| **Item / 项目** | | **Q1 / 问题1** | | **Q2 / 问题2** | | **Q3 / 问题3** |
| **Patient question (Chinese original) / 患者原始问题（中文）** | | 产后医生给我开了抗生素，我还可以喂奶吗？ | | 吃药后需要间隔多久再哺乳？ | | 哪些药物需要提前告诉医生我正在哺乳？ |
| **English translation of patient question / 患者问题英文翻译** | | The doctor prescribed antibiotics after birth. Can I still breastfeed? | | How long should I wait after taking medicine before breastfeeding? | | Which medicines require me to tell the doctor in advance that I am breastfeeding? |
| **Model response (Chinese) / 模型回答（中文）** | | 大多数可以，如青霉素、头孢类。 | | 可服药前喂奶，或间隔2-4小时。 | | 所有药物，就诊时告知。 |
| **English translation of model response / 模型回答英文翻译** | | Most (e.g., penicillins, cephalosporins) are safe. | | Feed before dose or separate by 2-4 hours. | | All medications; always inform doctor. |
| **Overall evaluation item / 整体评价项目** | | | **Score / 分数** | | **Comments / 备注** | |
| **Patient Satisfaction / 患者满意度** | | | 3 | |  | |
| **Patient Understandability / 患者可理解性** | | | 4 | |  | |
| **Patient Usefulness / 患者实用性** | | | 3 | |  | |
| **Clinician Correctness / 医生评分：正确性** | | | 3 | |  | |
| **Clinician Completeness / 医生评分：完整性** | | | 3 | |  | |
| **Clinician Readability / 医生评分：可读性** | | | 5 | |  | |
| **Clinician Helpfulness / 医生评分：帮助性** | | | 3 | |  | |
| **Clinician Safety / 医生评分：安全性** | | | 3 | |  | |

**Patient 77**

| **Patient ID / 患者编号** | **Group / 模型组别** | | | | | |
| --- | --- | --- | --- | --- | --- | --- |
| P77 | DeepSeek V3 | | | | | |
| **General model response or patient education material (Chinese) / 模型总体回答或患者教育材料（中文）** | | | | | | |
| 产后伤口疼痛影响睡觉，可以安全使用止痛药。哺乳期首选的止痛药是对乙酰氨基酚（扑热息痛）和布洛芬。这两种药物进入乳汁的量极少，被认为是哺乳期最安全的止痛药。常规剂量下，不需要暂停哺乳。服药后约1-2小时血药浓度达到高峰，您可以选择在喂奶后立即服药，或者选择在喂奶前服药，使下次喂奶时浓度已下降。如果服用常规剂量止痛药后疼痛仍无法缓解，或疼痛越来越重，请尽快复查，排除伤口感染、血肿或神经损伤。不要随意使用含可待因或阿司匹林的止痛药。 | | | | | | |
| **English translation of the Chinese general response / 中文总体回答英文翻译** | | | | | | |
| If postpartum wound pain interferes with sleep, you can safely use pain relievers. First-line choices during breastfeeding are acetaminophen (paracetamol) and ibuprofen. Both enter breastmilk in very small amounts and are considered the safest options. At standard doses, no need to stop breastfeeding. The peak drug level occurs about 1-2 hours after taking it; you can take it immediately after feeding or before feeding so that levels drop by the next feed. If pain persists or worsens despite medication, have the wound rechecked to rule out infection, hematoma, or nerve injury. Do not use painkillers containing codeine or aspirin. | | | | | | |
| **Item / 项目** | | **Q1 / 问题1** | | **Q2 / 问题2** | | **Q3 / 问题3** |
| **Patient question (Chinese original) / 患者原始问题（中文）** | | 产后伤口疼影响睡觉，可以吃止痛药吗？ | | 哺乳期吃止痛药会不会进入乳汁？ | | 如果止痛药也缓解不了疼痛，需要复查吗？ |
| **English translation of patient question / 患者问题英文翻译** | | Postpartum wound pain affects my sleep. Can I take painkillers? | | Will painkillers taken during breastfeeding enter the breast milk? | | If painkillers do not relieve the pain, do I need a follow-up examination? |
| **Model response (Chinese) / 模型回答（中文）** | | 可以，对乙酰氨基酚或布洛芬安全。 | | 少量进入，通常不影响宝宝。 | | 需要。 |
| **English translation of model response / 模型回答英文翻译** | | Yes, acetaminophen or ibuprofen is safe. | | Minimal transfer; rarely affects baby. | | Yes. |
| **Overall evaluation item / 整体评价项目** | | | **Score / 分数** | | **Comments / 备注** | |
| **Patient Satisfaction / 患者满意度** | | | 3 | |  | |
| **Patient Understandability / 患者可理解性** | | | 4 | |  | |
| **Patient Usefulness / 患者实用性** | | | 3 | |  | |
| **Clinician Correctness / 医生评分：正确性** | | | 3 | |  | |
| **Clinician Completeness / 医生评分：完整性** | | | 3 | |  | |
| **Clinician Readability / 医生评分：可读性** | | | 5 | |  | |
| **Clinician Helpfulness / 医生评分：帮助性** | | | 3 | |  | |
| **Clinician Safety / 医生评分：安全性** | | | 3 | |  | |

**Patient 78**

| **Patient ID / 患者编号** | **Group / 模型组别** | | | | | |
| --- | --- | --- | --- | --- | --- | --- |
| P78 | DeepSeek V3 | | | | | |
| **General model response or patient education material (Chinese) / 模型总体回答或患者教育材料（中文）** | | | | | | |
| 哺乳期可以接种流感疫苗、新冠疫苗、百白破疫苗等灭活疫苗，这些疫苗不会通过乳汁影响宝宝，反而可以通过母乳传递抗体，为宝宝提供被动免疫。接种后不需要暂停哺乳。减毒活疫苗（如麻腮风疫苗、水痘疫苗、鼻喷流感疫苗）在哺乳期理论上也是安全的，但建议咨询医生。如果接种后出现发热，可以服用对乙酰氨基酚退热，继续正常哺乳。接种疫苗是预防传染病的有效手段，哺乳期妈妈接种疫苗不仅保护自己，也保护无法接种某些疫苗的婴儿。请放心接种。 | | | | | | |
| **English translation of the Chinese general response / 中文总体回答英文翻译** | | | | | | |
| Breastfeeding mothers can receive inactivated vaccines such as flu, COVID-19, and Tdap. These vaccines do not pass into milk to harm the baby; instead, maternal antibodies are transferred via breastmilk, providing passive immunity. No need to stop breastfeeding after vaccination. Live attenuated vaccines (MMR, varicella, nasal flu spray) are also considered safe during lactation, but consult your doctor. If you develop fever after vaccination, take acetaminophen and continue nursing. Vaccination protects both the mother and the baby, who may be too young for certain vaccines. Feel confident to get vaccinated. | | | | | | |
| **Item / 项目** | | **Q1 / 问题1** | | **Q2 / 问题2** | | **Q3 / 问题3** |
| **Patient question (Chinese original) / 患者原始问题（中文）** | | 哺乳期可以接种流感疫苗或其他疫苗吗？ | | 接种疫苗后需要暂停哺乳吗？ | | 如果接种后发热，应该怎么处理？ |
| **English translation of patient question / 患者问题英文翻译** | | Can I receive the flu vaccine or other vaccines while breastfeeding? | | Do I need to stop breastfeeding after vaccination? | | If I develop fever after vaccination, what should I do? |
| **Model response (Chinese) / 模型回答（中文）** | | 可以，灭活疫苗安全。 | | 不需要。 | | 可用对乙酰氨基酚，继续哺乳。 |
| **English translation of model response / 模型回答英文翻译** | | Yes, inactivated vaccines are safe. | | No. | | Take acetaminophen and continue breastfeeding. |
| **Overall evaluation item / 整体评价项目** | | | **Score / 分数** | | **Comments / 备注** | |
| **Patient Satisfaction / 患者满意度** | | | 3 | |  | |
| **Patient Understandability / 患者可理解性** | | | 4 | |  | |
| **Patient Usefulness / 患者实用性** | | | 3 | |  | |
| **Clinician Correctness / 医生评分：正确性** | | | 3 | |  | |
| **Clinician Completeness / 医生评分：完整性** | | | 3 | |  | |
| **Clinician Readability / 医生评分：可读性** | | | 5 | |  | |
| **Clinician Helpfulness / 医生评分：帮助性** | | | 3 | |  | |
| **Clinician Safety / 医生评分：安全性** | | | 3 | |  | |

**Patient 79**

| **Patient ID / 患者编号** | **Group / 模型组别** | | | | | |
| --- | --- | --- | --- | --- | --- | --- |
| P79 | DeepSeek V3 | | | | | |
| **General model response or patient education material (Chinese) / 模型总体回答或患者教育材料（中文）** | | | | | | |
| 产后特别怕热、心慌、手抖、体重下降，同时可能伴有烦躁、失眠、多汗，要警惕产后甲状腺炎（通常先出现甲亢期，后可转为甲减）。请到医院抽血检查甲状腺功能（TSH、FT3、FT4）和抗甲状腺过氧化物酶抗体、促甲状腺素受体抗体。甲状腺功能异常会影响情绪（焦虑或抑郁）、影响奶量（甲亢可能使奶量减少，甲减也会影响泌乳）。治疗方案需根据甲功结果决定：甲亢期通常不需要抗甲状腺药物（多数自限性），甲减期可能需要补充左甲状腺素。哺乳期使用抗甲亢药物（丙硫氧嘧啶或甲巯咪唑）和左甲状腺素都是安全的。 | | | | | | |
| **English translation of the Chinese general response / 中文总体回答英文翻译** | | | | | | |
| Postpartum heat intolerance, palpitations, hand tremors, weight loss, along with irritability and insomnia, should raise suspicion for postpartum thyroiditis (often presents with a transient thyrotoxic phase followed by hypothyroidism). Check thyroid function (TSH, FT3, FT4), anti-TPO, and TSH receptor antibodies. Thyroid dysfunction affects mood (anxiety or depression) and milk supply (both hyper- and hypothyroidism can reduce milk). Treatment depends on results: the thyrotoxic phase is usually self-limiting and requires no antithyroid drugs; hypothyroidism may need levothyroxine. Antithyroid drugs (propylthiouracil, methimazole) and levothyroxine are safe during breastfeeding. | | | | | | |
| **Item / 项目** | | **Q1 / 问题1** | | **Q2 / 问题2** | | **Q3 / 问题3** |
| **Patient question (Chinese original) / 患者原始问题（中文）** | | 我产后特别怕热、心慌、手抖，会不会是甲状腺问题？ | | 产后甲状腺炎需要做哪些检查？ | | 甲状腺异常会不会影响情绪和奶量？ |
| **English translation of patient question / 患者问题英文翻译** | | After birth, I am especially heat-intolerant, have palpitations, and my hands tremble. Could this be a thyroid problem? | | What tests are needed for postpartum thyroiditis? | | Can thyroid abnormalities affect mood and milk supply? |
| **Model response (Chinese) / 模型回答（中文）** | | 可能，查甲状腺功能。 | | TSH、FT3、FT4、抗甲状腺抗体。 | | 会，需内分泌科管理。 |
| **English translation of model response / 模型回答英文翻译** | | Possible; check thyroid function. | | TSH, FT3, FT4, anti-thyroid antibodies. | | Yes; endocrinology management needed. |
| **Overall evaluation item / 整体评价项目** | | | **Score / 分数** | | **Comments / 备注** | |
| **Patient Satisfaction / 患者满意度** | | | 3 | |  | |
| **Patient Understandability / 患者可理解性** | | | 4 | |  | |
| **Patient Usefulness / 患者实用性** | | | 3 | |  | |
| **Clinician Correctness / 医生评分：正确性** | | | 3 | |  | |
| **Clinician Completeness / 医生评分：完整性** | | | 3 | |  | |
| **Clinician Readability / 医生评分：可读性** | | | 5 | |  | |
| **Clinician Helpfulness / 医生评分：帮助性** | | | 3 | |  | |
| **Clinician Safety / 医生评分：安全性** | | | 3 | |  | |

**Patient 80**

| **Patient ID / 患者编号** | **Group / 模型组别** | | | | | |
| --- | --- | --- | --- | --- | --- | --- |
| P80 | DeepSeek V3 | | | | | |
| **General model response or patient education material (Chinese) / 模型总体回答或患者教育材料（中文）** | | | | | | |
| 您孕前或孕期有高血压，产后两周又出现头痛，这是需要警惕的危险信号。产后血压仍可能在数周内波动，头痛、眼花、胸闷、上腹痛可能是产后子痫前期的表现。请立即测量血压：如果收缩压≥140 mmHg或舒张压≥90 mmHg，尽快就医；如果收缩压≥160 mmHg或舒张压≥110 mmHg，立即去急诊。产后血压应至少监测到6周，每天早晚各测一次并记录。即使血压正常，如果出现上述症状也要测血压，因为有些患者在血压升高之前就有症状。不要自行停用降压药，产后复查时医生会根据情况调整。 | | | | | | |
| **English translation of the Chinese general response / 中文总体回答英文翻译** | | | | | | |
| If you had hypertension before or during pregnancy and develop headache two weeks postpartum, this is a warning sign. Postpartum blood pressure can fluctuate for weeks. Headache, blurred vision, chest pain, or epigastric pain may indicate postpartum preeclampsia. Measure BP immediately: if systolic ≥140 or diastolic ≥90, seek medical care; if systolic ≥160 or diastolic ≥110, go to the emergency department. Monitor BP at least until 6 weeks postpartum, twice daily. Even if BP is normal, symptoms can precede elevation. Do not stop antihypertensive medications on your own; your doctor will adjust at postpartum visits. | | | | | | |
| **Item / 项目** | | **Q1 / 问题1** | | **Q2 / 问题2** | | **Q3 / 问题3** |
| **Patient question (Chinese original) / 患者原始问题（中文）** | | 我孕期血压高，产后两周又头痛，是不是危险信号？ | | 产后血压应该监测到什么时候？ | | 如果出现眼花、胸闷或上腹痛，需要急诊吗？ |
| **English translation of patient question / 患者问题英文翻译** | | I had high blood pressure during pregnancy and now have a headache two weeks after birth. Is this a warning sign? | | How long should postpartum blood pressure be monitored? | | If I have blurred vision, chest tightness, or upper abdominal pain, do I need emergency care? |
| **Model response (Chinese) / 模型回答（中文）** | | 是，立即测血压。 | | 至少监测到产后6周。 | | 需要，警惕子痫。 |
| **English translation of model response / 模型回答英文翻译** | | Yes, check BP immediately. | | Monitor at least 6 weeks postpartum. | | Yes, suspect eclampsia. |
| **Overall evaluation item / 整体评价项目** | | | **Score / 分数** | | **Comments / 备注** | |
| **Patient Satisfaction / 患者满意度** | | | 3 | |  | |
| **Patient Understandability / 患者可理解性** | | | 4 | |  | |
| **Patient Usefulness / 患者实用性** | | | 3 | |  | |
| **Clinician Correctness / 医生评分：正确性** | | | 3 | |  | |
| **Clinician Completeness / 医生评分：完整性** | | | 3 | |  | |
| **Clinician Readability / 医生评分：可读性** | | | 5 | |  | |
| **Clinician Helpfulness / 医生评分：帮助性** | | | 3 | |  | |
| **Clinician Safety / 医生评分：安全性** | | | 3 | |  | |

**Patient 81**

| **Patient ID / 患者编号** | **Group / 模型组别** | | | | | |
| --- | --- | --- | --- | --- | --- | --- |
| P81 | DeepSeek V3 | | | | | |
| **General model response or patient education material (Chinese) / 模型总体回答或患者教育材料（中文）** | | | | | | |
| 妊娠期糖尿病患者产后应做糖耐量复查，时间在产后4-12周。建议进行75g口服葡萄糖耐量试验，而不是只测空腹血糖。哺乳不会影响糖耐量结果，您可以正常哺乳。如果产后血糖正常，不代表“完全不用管”：您未来发生2型糖尿病的风险比没有妊娠期糖尿病的女性高7-10倍。因此需要每年复查空腹血糖或糖化血红蛋白，并保持健康生活方式（控制碳水摄入、每周150分钟运动、维持健康体重）。如果产后血糖偏高（空腹≥5.6 mmol/L或糖耐量2小时≥7.8 mmol/L），应到内分泌科进一步评估和管理。 | | | | | | |
| **English translation of the Chinese general response / 中文总体回答英文翻译** | | | | | | |
| Women with gestational diabetes should have a postpartum glucose tolerance test at 4-12 weeks. A 75g oral glucose tolerance test is recommended, not just fasting glucose. Breastfeeding does not affect the results. Normal postpartum glucose does not mean you can “ignore it” — your future risk of type 2 diabetes is 7-10 times higher than women without GDM. Therefore, check fasting glucose or HbA1c annually and maintain a healthy lifestyle (controlled carbs, 150 min/week exercise, healthy weight). If postpartum glucose is elevated (fasting ≥5.6 mmol/L or 2h OGTT ≥7.8 mmol/L), see an endocrinologist. | | | | | | |
| **Item / 项目** | | **Q1 / 问题1** | | **Q2 / 问题2** | | **Q3 / 问题3** |
| **Patient question (Chinese original) / 患者原始问题（中文）** | | 我孕期血糖高，产后多久做糖耐量复查？ | | 复查前还在哺乳，会影响血糖结果吗？ | | 如果产后血糖偏高，需要内分泌科随访吗？ |
| **English translation of patient question / 患者问题英文翻译** | | I had high blood sugar during pregnancy. How long after birth should I have an oral glucose tolerance test? | | If I am still breastfeeding before the test, will it affect the blood glucose results? | | If my postpartum blood glucose is high, do I need follow-up with an endocrinologist? |
| **Model response (Chinese) / 模型回答（中文）** | | 产后4-12周。 | | 不影响。 | | 需要。 |
| **English translation of model response / 模型回答英文翻译** | | 4-12 weeks postpartum. | | No. | | Yes. |
| **Overall evaluation item / 整体评价项目** | | | **Score / 分数** | | **Comments / 备注** | |
| **Patient Satisfaction / 患者满意度** | | | 3 | |  | |
| **Patient Understandability / 患者可理解性** | | | 4 | |  | |
| **Patient Usefulness / 患者实用性** | | | 3 | |  | |
| **Clinician Correctness / 医生评分：正确性** | | | 3 | |  | |
| **Clinician Completeness / 医生评分：完整性** | | | 3 | |  | |
| **Clinician Readability / 医生评分：可读性** | | | 4 | |  | |
| **Clinician Helpfulness / 医生评分：帮助性** | | | 3 | |  | |
| **Clinician Safety / 医生评分：安全性** | | | 3 | |  | |

**Patient 82**

| **Patient ID / 患者编号** | **Group / 模型组别** | | | | | |
| --- | --- | --- | --- | --- | --- | --- |
| P82 | DeepSeek V3 | | | | | |
| **General model response or patient education material (Chinese) / 模型总体回答或患者教育材料（中文）** | | | | | | |
| 哺乳期想减肥，不要少吃主食。碳水化合物是乳汁中乳糖的主要来源，也是维持精力和情绪的基础。过度限制碳水会导致疲劳、奶量下降、情绪低落。产后健康减重速度为每周0.5-1公斤，过快减重（每周超过1.5公斤）会显著影响奶量。安全减重方法：均衡饮食（每餐有主食、蛋白质、蔬菜），每天摄入1800-2000千卡；每周进行150分钟中等强度运动（快走、产后瑜伽、游泳）；保证每天饮水2500毫升以上。如果减重后奶量下降，请增加能量摄入200-300千卡/天，增加哺乳或吸奶频率，奶量通常可恢复。 | | | | | | |
| **English translation of the Chinese general response / 中文总体回答英文翻译** | | | | | | |
| Do not drastically cut carbs when trying to lose weight while breastfeeding. Carbohydrates provide lactose for milk and sustain energy and mood. Severely limiting carbs causes fatigue, reduced milk supply, and low mood. Healthy weight loss rate is 0.5-1 kg/week. Losing >1.5 kg/week significantly affects milk supply. Safe approach: balanced meals (carbs, protein, vegetables) totaling 1800-2000 kcal/day; 150 minutes/week of moderate exercise (brisk walking, postpartum yoga, swimming); drink >2500 mL water daily. If milk supply drops, increase calorie intake by 200-300 kcal/day and nurse/pump more frequently — supply usually recovers. | | | | | | |
| **Item / 项目** | | **Q1 / 问题1** | | **Q2 / 问题2** | | **Q3 / 问题3** |
| **Patient question (Chinese original) / 患者原始问题（中文）** | | 哺乳期想减肥，可以少吃主食吗？ | | 产后体重下降太快会不会影响奶量？ | | 怎样减重既安全又不影响恢复？ |
| **English translation of patient question / 患者问题英文翻译** | | I want to lose weight while breastfeeding. Can I eat fewer staple foods? | | Will losing weight too quickly after birth affect my milk supply? | | How can I lose weight safely without affecting recovery? |
| **Model response (Chinese) / 模型回答（中文）** | | 不可以，会影响奶量和精力。 | | 会，每周减0.5-1kg为宜。 | | 均衡饮食+适度运动+充足饮水。 |
| **English translation of model response / 模型回答英文翻译** | | No, it affects milk supply and energy. | | Yes; safe rate 0.5-1 kg/week. | | Balanced diet + moderate exercise + adequate hydration. |
| **Overall evaluation item / 整体评价项目** | | | **Score / 分数** | | **Comments / 备注** | |
| **Patient Satisfaction / 患者满意度** | | | 3 | |  | |
| **Patient Understandability / 患者可理解性** | | | 4 | |  | |
| **Patient Usefulness / 患者实用性** | | | 3 | |  | |
| **Clinician Correctness / 医生评分：正确性** | | | 3 | |  | |
| **Clinician Completeness / 医生评分：完整性** | | | 3 | |  | |
| **Clinician Readability / 医生评分：可读性** | | | 5 | |  | |
| **Clinician Helpfulness / 医生评分：帮助性** | | | 3 | |  | |
| **Clinician Safety / 医生评分：安全性** | | | 3 | |  | |

**Patient 83**

| **Patient ID / 患者编号** | **Group / 模型组别** | | | | | |
| --- | --- | --- | --- | --- | --- | --- |
| P83 | DeepSeek V3 | | | | | |
| **General model response or patient education material (Chinese) / 模型总体回答或患者教育材料（中文）** | | | | | | |
| 产后胃口不好、吃得少，会影响伤口愈合、体力恢复和乳汁分泌。建议少食多餐，每天5-6顿，每顿量少但营养密集。素食产妇哺乳期尤其需要补充：维生素B12（强化谷物、营养酵母或补充剂）、铁（黑木耳、红枣、菠菜，配合维生素C）、钙（豆腐、芝麻酱、深绿色蔬菜）、锌（坚果、豆类）、优质蛋白（豆制品、藜麦、鹰嘴豆）。如果经常头晕、乏力、面色苍白、指甲脆裂，应查血常规、铁蛋白和维生素B12水平，明确有无贫血或缺乏症。根据结果进行针对性补充，不要盲目补。 | | | | | | |
| **English translation of the Chinese general response / 中文总体回答英文翻译** | | | | | | |
| Poor appetite and low food intake postpartum impair wound healing, energy, and milk production. Eat small frequent meals (5-6 per day) with nutrient-dense choices. Vegetarian mothers need special attention: vitamin B12 (fortified cereals, nutritional yeast, or supplement), iron (black fungus, red dates, spinach with vitamin C), calcium (tofu, tahini, dark leafy greens), zinc (nuts, legumes), and high-quality protein (soy products, quinoa, chickpeas). If dizziness, fatigue, pallor, or brittle nails occur, check CBC, ferritin, and vitamin B12 levels to identify deficiencies and supplement accordingly. | | | | | | |
| **Item / 项目** | | **Q1 / 问题1** | | **Q2 / 问题2** | | **Q3 / 问题3** |
| **Patient question (Chinese original) / 患者原始问题（中文）** | | 我产后胃口不好，吃得少会影响伤口恢复吗？ | | 素食产妇哺乳期需要特别补充什么？ | | 如果经常头晕乏力，需要查营养指标吗？ |
| **English translation of patient question / 患者问题英文翻译** | | My appetite is poor after birth and I eat very little. Will this affect wound healing? | | What special supplements do vegetarian mothers need during breastfeeding? | | If I often feel dizzy and weak, do I need to check nutritional indicators? |
| **Model response (Chinese) / 模型回答（中文）** | | 会，少食多餐。 | | 维生素B12、铁、钙、锌、优质蛋白。 | | 需要，查血常规、铁蛋白、维生素B12。 |
| **English translation of model response / 模型回答英文翻译** | | Yes; eat small frequent meals. | | Vitamin B12, iron, calcium, zinc, high-quality protein. | | Yes, check CBC, ferritin, vitamin B12. |
| **Overall evaluation item / 整体评价项目** | | | **Score / 分数** | | **Comments / 备注** | |
| **Patient Satisfaction / 患者满意度** | | | 3 | |  | |
| **Patient Understandability / 患者可理解性** | | | 4 | |  | |
| **Patient Usefulness / 患者实用性** | | | 3 | |  | |
| **Clinician Correctness / 医生评分：正确性** | | | 3 | |  | |
| **Clinician Completeness / 医生评分：完整性** | | | 3 | |  | |
| **Clinician Readability / 医生评分：可读性** | | | 5 | |  | |
| **Clinician Helpfulness / 医生评分：帮助性** | | | 3 | |  | |
| **Clinician Safety / 医生评分：安全性** | | | 3 | |  | |

**Patient 84**

| **Patient ID / 患者编号** | **Group / 模型组别** | | | | | |
| --- | --- | --- | --- | --- | --- | --- |
| P84 | DeepSeek V3 | | | | | |
| **General model response or patient education material (Chinese) / 模型总体回答或患者教育材料（中文）** | | | | | | |
| 哺乳期腰腿酸，不一定是缺钙，更多是因为产后腰背肌劳损、喂奶姿势不良、核心肌力减弱。但哺乳期确实需要增加钙和维生素D的摄入，因为乳汁会消耗母体钙。推荐每日钙摄入1000-1200毫克（相当于500毫升牛奶+100克豆腐+一把芝麻），维生素D 600-800 IU。如果腿抽筋频繁（每周超过3次），尤其是夜间抽筋，应查血钙、镁、磷和维生素D水平。注意：补钙最好从食物中获取，钙片应在医生指导下服用，过量补钙可能引起便秘或肾结石。同时注意保暖、拉伸小腿肌肉。 | | | | | | |
| **English translation of the Chinese general response / 中文总体回答英文翻译** | | | | | | |
| Back and leg aches during breastfeeding are not necessarily due to calcium deficiency; they are often from muscle strain, poor feeding posture, and weak core strength. However, calcium and vitamin D needs are increased because breastmilk uses maternal stores. Recommended daily intake: calcium 1000-1200 mg (e.g., 500 ml milk + 100 g tofu + a spoon of sesame seeds), vitamin D 600-800 IU. If leg cramps are frequent (>3 times/week), especially at night, check serum calcium, magnesium, phosphorus, and vitamin D. Obtain calcium from food when possible; supplements should be taken under medical guidance to avoid constipation or kidney stones. Also keep warm and stretch calf muscles. | | | | | | |
| **Item / 项目** | | **Q1 / 问题1** | | **Q2 / 问题2** | | **Q3 / 问题3** |
| **Patient question (Chinese original) / 患者原始问题（中文）** | | 哺乳期腰腿酸，是不是缺钙？ | | 产后需要补钙和维生素D吗？ | | 如果腿抽筋频繁，需要去检查吗？ |
| **English translation of patient question / 患者问题英文翻译** | | I have soreness in my lower back and legs while breastfeeding. Is this due to calcium deficiency? | | Do I need calcium and vitamin D supplements after birth? | | If leg cramps occur frequently, do I need an examination? |
| **Model response (Chinese) / 模型回答（中文）** | | 不一定是，多为劳损或姿势不良。 | | 需要，每日钙1000-1200mg，维生素D 600IU。 | | 需要，查血钙、镁、磷、维生素D。 |
| **English translation of model response / 模型回答英文翻译** | | Not necessarily; often due to strain or poor posture. | | Yes, calcium 1000-1200mg/day, vitamin D 600 IU/day. | | Yes, check calcium, magnesium, phosphorus, vitamin D. |
| **Overall evaluation item / 整体评价项目** | | | **Score / 分数** | | **Comments / 备注** | |
| **Patient Satisfaction / 患者满意度** | | | 3 | |  | |
| **Patient Understandability / 患者可理解性** | | | 4 | |  | |
| **Patient Usefulness / 患者实用性** | | | 3 | |  | |
| **Clinician Correctness / 医生评分：正确性** | | | 3 | |  | |
| **Clinician Completeness / 医生评分：完整性** | | | 3 | |  | |
| **Clinician Readability / 医生评分：可读性** | | | 5 | |  | |
| **Clinician Helpfulness / 医生评分：帮助性** | | | 3 | |  | |
| **Clinician Safety / 医生评分：安全性** | | | 3 | |  | |

**Patient 85**

| **Patient ID / 患者编号** | **Group / 模型组别** | | | | | |
| --- | --- | --- | --- | --- | --- | --- |
| P85 | DeepSeek V3 | | | | | |
| **General model response or patient education material (Chinese) / 模型总体回答或患者教育材料（中文）** | | | | | | |
| 补铁后便秘更严重是常见副作用。您可以：1）增加膳食纤维，如燕麦、红薯、火龙果、绿叶菜；2）每天饮水2500毫升以上；3）在医生指导下换用对胃肠道刺激较小的铁剂，如蛋白琥珀酸铁、多糖铁复合物或液体铁剂。铁剂不应与牛奶、咖啡、茶、钙片同服，这些会抑制铁吸收，间隔至少2小时。如果贫血一直不改善（口服铁剂4-8周后血红蛋白不升或上升<10g/L），需要查找原因：有无慢性失血（消化道出血、月经过多）、吸收不良（胃切除、炎症性肠病）、或地中海贫血。请到血液科进一步检查。 | | | | | | |
| **English translation of the Chinese general response / 中文总体回答英文翻译** | | | | | | |
| Iron-induced constipation is a common side effect. You can: 1) increase dietary fiber (oats, sweet potatoes, dragon fruit, leafy greens); 2) drink >2500 mL water daily; 3) under medical guidance, switch to a better-tolerated iron preparation (iron protein succinylate, polysaccharide iron complex, or liquid iron). Do not take iron with milk, coffee, tea, or calcium supplements — separate by at least 2 hours. If anemia does not improve (Hb rises <10 g/L after 4-8 weeks of oral iron), investigate causes: chronic blood loss (GI bleeding, menorrhagia), malabsorption, or thalassemia. See a hematologist. | | | | | | |
| **Item / 项目** | | **Q1 / 问题1** | | **Q2 / 问题2** | | **Q3 / 问题3** |
| **Patient question (Chinese original) / 患者原始问题（中文）** | | 我补铁后便秘更严重，应该怎么办？ | | 铁剂可以和牛奶一起吃吗？ | | 如果贫血一直不改善，需要查什么原因？ |
| **English translation of patient question / 患者问题英文翻译** | | My constipation became worse after taking iron. What should I do? | | Can iron supplements be taken with milk? | | If anemia does not improve, what causes need to be checked? |
| **Model response (Chinese) / 模型回答（中文）** | | 多吃纤维、多喝水，或改用液体铁剂。 | | 不可以，间隔2小时。 | | 查慢性失血、吸收不良、地中海贫血等。 |
| **English translation of model response / 模型回答英文翻译** | | Increase fiber/water or switch to liquid iron. | | No, separate by 2 hours. | | Check for chronic blood loss, malabsorption, thalassemia. |
| **Overall evaluation item / 整体评价项目** | | | **Score / 分数** | | **Comments / 备注** | |
| **Patient Satisfaction / 患者满意度** | | | 3 | |  | |
| **Patient Understandability / 患者可理解性** | | | 4 | |  | |
| **Patient Usefulness / 患者实用性** | | | 3 | |  | |
| **Clinician Correctness / 医生评分：正确性** | | | 3 | |  | |
| **Clinician Completeness / 医生评分：完整性** | | | 3 | |  | |
| **Clinician Readability / 医生评分：可读性** | | | 5 | |  | |
| **Clinician Helpfulness / 医生评分：帮助性** | | | 3 | |  | |
| **Clinician Safety / 医生评分：安全性** | | | 3 | |  | |

**Patient 86**

| **Patient ID / 患者编号** | **Group / 模型组别** | | | | | |
| --- | --- | --- | --- | --- | --- | --- |
| P86 | DeepSeek V3 | | | | | |
| **General model response or patient education material (Chinese) / 模型总体回答或患者教育材料（中文）** | | | | | | |
| 宝宝夜里频繁醒，您长期睡不好，会严重影响产后恢复：伤口愈合减慢、免疫力下降、情绪不稳定、甚至增加产后抑郁风险。白天补觉非常有益，每次20-30分钟的小睡就能显著改善疲劳感和认知功能。您可以利用宝宝白天小睡的时间同步休息，不要做家务。如果失眠持续超过2周，表现为入睡困难、早醒、睡眠浅、多梦，即使宝宝睡了您也睡不着，或者白天极度困倦，应寻求医生帮助。心理科医生可以评估是否存在产后焦虑或抑郁，并指导行为治疗或短期使用哺乳期安全的助眠药物。 | | | | | | |
| **English translation of the Chinese general response / 中文总体回答英文翻译** | | | | | | |
| Frequent night wakings and chronic sleep deprivation severely impair postpartum recovery: delayed wound healing, reduced immunity, mood instability, and increased risk of postpartum depression. Daytime napping is very beneficial — even 20-30 minutes can significantly improve fatigue and cognitive function. Nap when the baby naps; do not do housework instead. If insomnia lasts >2 weeks (difficulty falling asleep, early waking, light sleep, nightmares, or being unable to sleep even when the baby sleeps), seek help. A psychologist can assess for postpartum anxiety/depression and recommend behavioral therapy or short-term use of breastfeeding-safe sleep aids. | | | | | | |
| **Item / 项目** | | **Q1 / 问题1** | | **Q2 / 问题2** | | **Q3 / 问题3** |
| **Patient question (Chinese original) / 患者原始问题（中文）** | | 宝宝夜里频繁醒，我长期睡不好，会影响恢复吗？ | | 白天补觉对产后恢复有帮助吗？ | | 如果失眠持续很多天，需要寻求医生帮助吗？ |
| **English translation of patient question / 患者问题英文翻译** | | The baby wakes frequently at night and I have poor sleep for a long time. Will this affect recovery? | | Is daytime napping helpful for postpartum recovery? | | If insomnia continues for many days, do I need to seek help from a doctor? |
| **Model response (Chinese) / 模型回答（中文）** | | 会，影响伤口愈合、情绪、免疫。 | | 有帮助，每次20-30分钟。 | | 需要，超过2周应咨询。 |
| **English translation of model response / 模型回答英文翻译** | | Yes, impairs wound healing, mood, immunity. | | Yes, 20-30 minute naps help. | | Yes, if >2 weeks, consult doctor. |
| **Overall evaluation item / 整体评价项目** | | | **Score / 分数** | | **Comments / 备注** | |
| **Patient Satisfaction / 患者满意度** | | | 3 | |  | |
| **Patient Understandability / 患者可理解性** | | | 4 | |  | |
| **Patient Usefulness / 患者实用性** | | | 3 | |  | |
| **Clinician Correctness / 医生评分：正确性** | | | 3 | |  | |
| **Clinician Completeness / 医生评分：完整性** | | | 3 | |  | |
| **Clinician Readability / 医生评分：可读性** | | | 5 | |  | |
| **Clinician Helpfulness / 医生评分：帮助性** | | | 3 | |  | |
| **Clinician Safety / 医生评分：安全性** | | | 3 | |  | |

**Patient 87**

| **Patient ID / 患者编号** | **Group / 模型组别** | | | | | |
| --- | --- | --- | --- | --- | --- | --- |
| P87 | DeepSeek V3 | | | | | |
| **General model response or patient education material (Chinese) / 模型总体回答或患者教育材料（中文）** | | | | | | |
| 产后情绪忽高忽低，被家人说“太敏感”，这种现象在产后1-2周内很常见，称为“产后情绪低落”，通常2周内自行缓解。但如果情绪波动超过2周，或者症状加重，出现持续悲伤、内疚、对什么都提不起兴趣、无法照顾自己或宝宝，则可能是产后抑郁。判断是否需要干预的标准：症状是否影响日常生活？是否超过2周？是否有自杀或伤婴念头？如果总是哭泣、内疚、不想打扮、不想吃饭，请尽快到心理科或精神科就诊。产后抑郁不是性格缺陷，而是一种可以治愈的疾病，药物治疗和心理治疗均有效且哺乳期安全。 | | | | | | |
| **English translation of the Chinese general response / 中文总体回答英文翻译** | | | | | | |
| Mood swings within the first 1-2 weeks postpartum are common and called “postpartum blues,” usually resolving within 2 weeks. However, if mood swings persist beyond 2 weeks or worsen with persistent sadness, guilt, loss of interest, inability to care for yourself or baby, this may be postpartum depression. Criterion: Do symptoms affect daily life? Last >2 weeks? Include suicidal or harm-to-baby thoughts? If you are crying frequently, feel guilty, neglect self-care, please see a psychiatrist or psychologist. Postpartum depression is not a character flaw but a treatable illness. Both medication and psychotherapy are effective and safe during breastfeeding. | | | | | | |
| **Item / 项目** | | **Q1 / 问题1** | | **Q2 / 问题2** | | **Q3 / 问题3** |
| **Patient question (Chinese original) / 患者原始问题（中文）** | | 我产后情绪忽高忽低，家人说我太敏感，正常吗？ | | 怎么判断是普通情绪波动还是需要干预？ | | 如果总是哭、内疚、没兴趣照顾自己，需要就医吗？ |
| **English translation of patient question / 患者问题英文翻译** | | My mood goes up and down after birth, and my family says I am too sensitive. Is this normal? | | How can I tell whether it is ordinary mood fluctuation or something that needs intervention? | | If I keep crying, feel guilty, and have no interest in caring for myself, do I need medical care? |
| **Model response (Chinese) / 模型回答（中文）** | | 常见，但超过2周需警惕。 | | 症状重、持续>2周、影响生活需干预。 | | 需要。 |
| **English translation of model response / 模型回答英文翻译** | | Common, but watch if >2 weeks. | | Severe symptoms, >2 weeks, affecting daily life. | | Yes. |
| **Overall evaluation item / 整体评价项目** | | | **Score / 分数** | | **Comments / 备注** | |
| **Patient Satisfaction / 患者满意度** | | | 3 | |  | |
| **Patient Understandability / 患者可理解性** | | | 4 | |  | |
| **Patient Usefulness / 患者实用性** | | | 3 | |  | |
| **Clinician Correctness / 医生评分：正确性** | | | 3 | |  | |
| **Clinician Completeness / 医生评分：完整性** | | | 3 | |  | |
| **Clinician Readability / 医生评分：可读性** | | | 5 | |  | |
| **Clinician Helpfulness / 医生评分：帮助性** | | | 3 | |  | |
| **Clinician Safety / 医生评分：安全性** | | | 3 | |  | |

**Patient 88**

| **Patient ID / 患者编号** | **Group / 模型组别** | | | | | |
| --- | --- | --- | --- | --- | --- | --- |
| P88 | DeepSeek V3 | | | | | |
| **General model response or patient education material (Chinese) / 模型总体回答或患者教育材料（中文）** | | | | | | |
| 总担心宝宝窒息，晚上反复检查，甚至不敢睡，这是产后焦虑的典型表现。适度的警觉是母性本能，但过度焦虑会严重影响您的生活和健康。反复检查会形成恶性循环：检查次数越多，焦虑反而越重。您可以尝试设定“检查次数上限”，比如每晚只检查2次，然后逐渐减少。同时学习婴儿猝死综合征的预防知识：让宝宝仰卧、使用硬床垫、避免床上软物品和过暖。如果焦虑已经影响吃饭、睡眠、日常照顾，或您感觉无法控制，请向心理医生或精神科医生求助。认知行为治疗对产后焦虑非常有效。 | | | | | | |
| **English translation of the Chinese general response / 中文总体回答英文翻译** | | | | | | |
| Persistent worry about your baby suffocating, repeatedly checking at night, even being afraid to sleep — these are typical signs of postpartum anxiety. Moderate alertness is maternal instinct, but excessive anxiety severely affects your health and daily life. Repeated checking creates a vicious cycle: more checking, more anxiety. Try setting a limit on how many times you check per night, e.g., twice, then gradually reduce. Learn SIDS prevention: baby on back, firm mattress, no loose bedding or overheating. If anxiety interferes with eating, sleeping, or caring for the baby, or you feel out of control, seek help from a psychologist or psychiatrist. Cognitive-behavioral therapy is very effective. | | | | | | |
| **Item / 项目** | | **Q1 / 问题1** | | **Q2 / 问题2** | | **Q3 / 问题3** |
| **Patient question (Chinese original) / 患者原始问题（中文）** | | 我总担心宝宝窒息，晚上不敢睡，是产后焦虑吗？ | | 反复检查宝宝会不会越来越焦虑？ | | 这种焦虑影响吃饭睡觉时应该找谁帮助？ |
| **English translation of patient question / 患者问题英文翻译** | | I always worry that the baby will suffocate and I dare not sleep at night. Is this postpartum anxiety? | | Will repeatedly checking the baby make me more anxious? | | When this anxiety affects eating and sleeping, who should I ask for help? |
| **Model response (Chinese) / 模型回答（中文）** | | 是，如严重影响生活需干预。 | | 会，尝试设定检查次数并逐渐减少。 | | 心理医生或精神科。 |
| **English translation of model response / 模型回答英文翻译** | | Yes, if severe, need intervention. | | Yes, try setting limits and reducing gradually. | | Psychologist or psychiatrist. |
| **Overall evaluation item / 整体评价项目** | | | **Score / 分数** | | **Comments / 备注** | |
| **Patient Satisfaction / 患者满意度** | | | 3 | |  | |
| **Patient Understandability / 患者可理解性** | | | 4 | |  | |
| **Patient Usefulness / 患者实用性** | | | 3 | |  | |
| **Clinician Correctness / 医生评分：正确性** | | | 3 | |  | |
| **Clinician Completeness / 医生评分：完整性** | | | 3 | |  | |
| **Clinician Readability / 医生评分：可读性** | | | 5 | |  | |
| **Clinician Helpfulness / 医生评分：帮助性** | | | 3 | |  | |
| **Clinician Safety / 医生评分：安全性** | | | 3 | |  | |

**Patient 89**

| **Patient ID / 患者编号** | **Group / 模型组别** | | | | | |
| --- | --- | --- | --- | --- | --- | --- |
| P89 | DeepSeek V3 | | | | | |
| **General model response or patient education material (Chinese) / 模型总体回答或患者教育材料（中文）** | | | | | | |
| 如果您有时脑子里冒出伤害宝宝的念头，即使只是瞬间，也让您自己非常害怕，请不要自责。这种“侵入性思维”是产后精神疾病（如产后抑郁伴焦虑或产后强迫）的症状，绝不是您“不是好妈妈”的证据。很多产妇会出现类似的可怕想法，但只有极少数人会付诸行动。您需要马上告诉您的家人和医生，尤其是精神科医生。这是可以治疗的。请不要独自承受，也不要因为羞耻而隐瞒。如果控制不住害怕和冲动，或者觉得可能真的会伤害宝宝，请立即去急诊或拨打心理危机干预热线。早期治疗可以完全恢复。 | | | | | | |
| **English translation of the Chinese general response / 中文总体回答英文翻译** | | | | | | |
| If you sometimes have intrusive thoughts of harming your baby — even fleeting — and they terrify you, do not blame yourself. These thoughts are symptoms of a postpartum psychiatric condition (such as depression with anxiety or postpartum OCD) and do not mean you are a “bad mother.” Many women experience such thoughts, but very few act on them. Tell your family and doctor immediately, especially a psychiatrist. This is treatable. Do not suffer alone or hide out of shame. If you fear losing control or truly believe you might harm your baby, go to the emergency department or call a crisis hotline now. Early treatment leads to full recovery. | | | | | | |
| **Item / 项目** | | **Q1 / 问题1** | | **Q2 / 问题2** | | **Q3 / 问题3** |
| **Patient question (Chinese original) / 患者原始问题（中文）** | | 我有时脑子里冒出伤害宝宝的念头，自己也很害怕，怎么办？ | | 这种想法是不是说明我不是好妈妈？ | | 如果控制不住害怕和冲动，需要马上告诉医生吗？ |
| **English translation of patient question / 患者问题英文翻译** | | Sometimes thoughts of harming the baby suddenly come into my mind, and I am very scared. What should I do? | | Do these thoughts mean I am not a good mother? | | If I cannot control my fear and impulses, do I need to tell a doctor immediately? |
| **Model response (Chinese) / 模型回答（中文）** | | 立即寻求精神科帮助，这是疾病症状。 | | 不是，这是产后精神疾病表现。 | | 需要，立即去急诊。 |
| **English translation of model response / 模型回答英文翻译** | | Seek immediate psychiatric help; it's a symptom. | | No, it's a manifestation of postpartum psychiatric illness. | | Yes, go to ER immediately. |
| **Overall evaluation item / 整体评价项目** | | | **Score / 分数** | | **Comments / 备注** | |
| **Patient Satisfaction / 患者满意度** | | | 3 | |  | |
| **Patient Understandability / 患者可理解性** | | | 4 | |  | |
| **Patient Usefulness / 患者实用性** | | | 3 | |  | |
| **Clinician Correctness / 医生评分：正确性** | | | 3 | |  | |
| **Clinician Completeness / 医生评分：完整性** | | | 3 | |  | |
| **Clinician Readability / 医生评分：可读性** | | | 5 | |  | |
| **Clinician Helpfulness / 医生评分：帮助性** | | | 3 | |  | |
| **Clinician Safety / 医生评分：安全性** | | | 3 | |  | |

**Patient 90**

| **Patient ID / 患者编号** | **Group / 模型组别** | | | | | |
| --- | --- | --- | --- | --- | --- | --- |
| P90 | DeepSeek V3 | | | | | |
| **General model response or patient education material (Chinese) / 模型总体回答或患者教育材料（中文）** | | | | | | |
| 家人照顾孩子的方式和您不一样，让您很烦躁，这是产后很容易出现的矛盾。建议您主动和家人（尤其是伴侣和长辈）进行一次平静的沟通，明确分工。例如：夜班由伴侣负责换尿布、拍嗝、哄睡，只在喂奶时叫醒您；白天老人帮忙做家务、做饭，您负责喂奶和宝宝洗澡。列出一个简单的时间表和任务清单，避免“谁有空谁做”的模糊分工。如果家庭矛盾已经严重影响您的休息和情绪，甚至让您产生逃避或伤害念头，请主动请产科护士、社区医生或心理医生介入指导。家庭支持是产后恢复的基石，不要一个人扛着。 | | | | | | |
| **English translation of the Chinese general response / 中文总体回答英文翻译** | | | | | | |
| Feeling irritated when family members care for the baby differently is common postpartum. Have a calm conversation with your partner and elders to clearly divide tasks. For example, partner handles night-time diaper changes, burping, and settling, waking you only for feeding; grandparents help with housework and cooking during the day. Create a simple schedule and task list to avoid vague “whoever is free” arrangements. If family conflicts severely affect your rest and mood, or cause thoughts of escape or harm, proactively ask an obstetric nurse, community doctor, or psychologist to mediate. Family support is the cornerstone of postpartum recovery; do not bear it alone. | | | | | | |
| **Item / 项目** | | **Q1 / 问题1** | | **Q2 / 问题2** | | **Q3 / 问题3** |
| **Patient question (Chinese original) / 患者原始问题（中文）** | | 家人照顾方式和我不一样，我很烦躁怎么办？ | | 产后可以怎样和家人分工照顾宝宝？ | | 如果家庭矛盾影响休息和情绪，需要请医护人员指导吗？ |
| **English translation of patient question / 患者问题英文翻译** | | My family cares for the baby differently from me, and I feel very irritable. What should I do? | | How can I divide baby care tasks with my family after birth? | | If family conflicts affect my rest and mood, should I ask healthcare staff for guidance? |
| **Model response (Chinese) / 模型回答（中文）** | | 沟通并分工，制定清晰计划。 | | 比如夜班、白天喂养、家务分开。 | | 需要。 |
| **English translation of model response / 模型回答英文翻译** | | Communicate and divide roles; make a clear plan. | | E.g., night shifts, daytime feeds, household tasks. | | Yes. |
| **Overall evaluation item / 整体评价项目** | | | **Score / 分数** | | **Comments / 备注** | |
| **Patient Satisfaction / 患者满意度** | | | 3 | |  | |
| **Patient Understandability / 患者可理解性** | | | 4 | |  | |
| **Patient Usefulness / 患者实用性** | | | 3 | |  | |
| **Clinician Correctness / 医生评分：正确性** | | | 3 | |  | |
| **Clinician Completeness / 医生评分：完整性** | | | 3 | |  | |
| **Clinician Readability / 医生评分：可读性** | | | 5 | |  | |
| **Clinician Helpfulness / 医生评分：帮助性** | | | 3 | |  | |
| **Clinician Safety / 医生评分：安全性** | | | 3 | |  | |

**Patient 91**

| **Patient ID / 患者编号** | **Group / 模型组别** | | | | | |
| --- | --- | --- | --- | --- | --- | --- |
| P91 | DeepSeek V3 | | | | | |
| **General model response or patient education material (Chinese) / 模型总体回答或患者教育材料（中文）** | | | | | | |
| 准备产后返岗，建议提前2-3周开始调整作息，逐步适应上班节奏。您可以根据通勤时间提前起床，并调整宝宝的喂养和睡眠时间表。上班后保存母乳：使用清洁的吸奶器，每3-4小时泵奶一次，将乳汁储存在专用储奶袋或瓶内，标注日期和时间，立即放入冰包（保持4℃以下）。到家后放入冰箱冷藏（可保存3-5天）或冷冻（可保存3-6个月）。如果返岗后奶量下降，不要慌张：增加夜间和周末的亲喂频率，利用夜间催乳素分泌高峰追奶；确保上班期间足够饮水和摄入能量；使用吸奶器时尝试放松和看宝宝照片。通常1-2周后会恢复。 | | | | | | |
| **English translation of the Chinese general response / 中文总体回答英文翻译** | | | | | | |
| Preparing to return to work, start adjusting your sleep/wake schedule 2-3 weeks in advance. Wake up earlier according to commute time and gradually adjust baby’s feeding/sleeping routine. At work, use a clean pump every 3-4 hours, store milk in sterile bags/bottles with date and time, and place immediately in a cooler bag (<4°C). Refrigerate (3-5 days) or freeze (3-6 months) at home. If milk supply drops, do not panic: increase nighttime and weekend direct feeding, take advantage of nighttime prolactin peaks, stay hydrated and well-fed at work, and try relaxing or looking at baby’s photos while pumping. Supply usually recovers within 1-2 weeks. | | | | | | |
| **Item / 项目** | | **Q1 / 问题1** | | **Q2 / 问题2** | | **Q3 / 问题3** |
| **Patient question (Chinese original) / 患者原始问题（中文）** | | 我准备产后返岗，什么时候开始调整作息比较好？ | | 上班后如何保存母乳比较安全？ | | 如果返岗后奶量下降，应该怎么调整？ |
| **English translation of patient question / 患者问题英文翻译** | | I am preparing to return to work after birth. When should I start adjusting my routine? | | How can I store breast milk safely after returning to work? | | If my milk supply decreases after returning to work, how should I adjust? |
| **Model response (Chinese) / 模型回答（中文）** | | 返岗前2周逐步调整。 | | 每3-4小时泵奶，用冰包储存。 | | 增加夜间或周末亲喂频率。 |
| **English translation of model response / 模型回答英文翻译** | | Start adjusting 2 weeks before returning to work. | | Pump every 3-4 hours, store in cooler with ice packs. | | Increase direct breastfeeding at night and weekends. |
| **Overall evaluation item / 整体评价项目** | | | **Score / 分数** | | **Comments / 备注** | |
| **Patient Satisfaction / 患者满意度** | | | 3 | |  | |
| **Patient Understandability / 患者可理解性** | | | 4 | |  | |
| **Patient Usefulness / 患者实用性** | | | 3 | |  | |
| **Clinician Correctness / 医生评分：正确性** | | | 3 | |  | |
| **Clinician Completeness / 医生评分：完整性** | | | 3 | |  | |
| **Clinician Readability / 医生评分：可读性** | | | 5 | |  | |
| **Clinician Helpfulness / 医生评分：帮助性** | | | 3 | |  | |
| **Clinician Safety / 医生评分：安全性** | | | 3 | |  | |

**Patient 92**

| **Patient ID / 患者编号** | **Group / 模型组别** | | | | | |
| --- | --- | --- | --- | --- | --- | --- |
| P92 | DeepSeek V3 | | | | | |
| **General model response or patient education material (Chinese) / 模型总体回答或患者教育材料（中文）** | | | | | | |
| 产后大量掉头发，医学上称为“产后休止期脱发”，通常在产后3-6个月出现，是正常的生理现象。因为孕期高水平的雌激素使头发生长期延长、脱发减少；产后雌激素骤降，大量毛囊进入休止期，同步脱落。这种脱发不会导致秃发，一般持续3-6个月，最长不超过1年，之后头发会自行再生。您不需要特殊治疗，但可以注意营养均衡（富含蛋白质、铁、锌、维生素B族），避免过度烫染和紧张焦虑。如果脱发非常严重（成片脱发、发际线明显后移）或超过1年未恢复，应查血常规、铁蛋白、甲状腺功能，排除贫血、缺锌或甲减。 | | | | | | |
| **English translation of the Chinese general response / 中文总体回答英文翻译** | | | | | | |
| Postpartum hair shedding (telogen effluvium) typically occurs at 3-6 months and is a normal physiological phenomenon. High estrogen during pregnancy prolonged the growth phase; after delivery, estrogen drops sharply, and many follicles enter the resting phase and shed simultaneously. This does not cause baldness, lasts 3-6 months (up to 1 year), and regrowth occurs spontaneously. No specific treatment is needed, but maintain good nutrition (protein, iron, zinc, B vitamins) and avoid excessive styling or stress. If shedding is very severe (patchy baldness, receding hairline) or lasts >1 year, check CBC, ferritin, and thyroid function to rule out anemia, zinc deficiency, or hypothyroidism. | | | | | | |
| **Item / 项目** | | **Q1 / 问题1** | | **Q2 / 问题2** | | **Q3 / 问题3** |
| **Patient question (Chinese original) / 患者原始问题（中文）** | | 我产后开始大量掉头发，是正常的吗？ | | 产后脱发一般多久会好？ | | 如果掉发很严重，需要查贫血或甲状腺吗？ |
| **English translation of patient question / 患者问题英文翻译** | | I have started losing a lot of hair after birth. Is this normal? | | How long does postpartum hair loss usually last? | | If hair loss is severe, do I need to check for anemia or thyroid problems? |
| **Model response (Chinese) / 模型回答（中文）** | | 正常，产后休止期脱发，6-12个月恢复。 | | 多数1年内自行恢复。 | | 需要。 |
| **English translation of model response / 模型回答英文翻译** | | Normal telogen effluvium; recovers in 6-12 months. | | Most recover within 1 year. | | Yes. |
| **Overall evaluation item / 整体评价项目** | | | **Score / 分数** | | **Comments / 备注** | |
| **Patient Satisfaction / 患者满意度** | | | 3 | |  | |
| **Patient Understandability / 患者可理解性** | | | 4 | |  | |
| **Patient Usefulness / 患者实用性** | | | 3 | |  | |
| **Clinician Correctness / 医生评分：正确性** | | | 3 | |  | |
| **Clinician Completeness / 医生评分：完整性** | | | 3 | |  | |
| **Clinician Readability / 医生评分：可读性** | | | 5 | |  | |
| **Clinician Helpfulness / 医生评分：帮助性** | | | 3 | |  | |
| **Clinician Safety / 医生评分：安全性** | | | 3 | |  | |

**Patient 93**

| **Patient ID / 患者编号** | **Group / 模型组别** | | | | | |
| --- | --- | --- | --- | --- | --- | --- |
| P93 | DeepSeek V3 | | | | | |
| **General model response or patient education material (Chinese) / 模型总体回答或患者教育材料（中文）** | | | | | | |
| 产后脸上的色斑（黄褐斑）会因为激素水平下降而逐渐变淡，但完全消退可能需要数月到一年。避免日晒是最重要的，因为紫外线会刺激黑色素细胞，使色斑加重甚至持续存在。哺乳期可以使用安全的护肤品和防晒霜：选择物理防晒剂（氧化锌、二氧化钛）或成分简单的化学防晒霜（如阿伏苯宗、奥克立林），它们不会进入乳汁。如果皮肤出现红色瘙痒性皮疹，可能是产后湿疹或妊娠多形疹，应看皮肤科，不要自行使用强效激素药膏。日常使用温和保湿霜和维生素C类抗氧化产品有助于淡化色斑。 | | | | | | |
| **English translation of the Chinese general response / 中文总体回答英文翻译** | | | | | | |
| Postpartum facial melasma gradually fades as hormone levels drop, but complete resolution may take months to a year. Sun protection is crucial because UV light stimulates melanocytes and can worsen or prolong melasma. Safe skincare and sunscreen during breastfeeding include physical sunscreens (zinc oxide, titanium dioxide) or simple chemical ones (avobenzone, octocrylene); they do not enter milk. If you develop red, itchy rashes, see a dermatologist (possible postpartum eczema or polymorphic eruption of pregnancy) — do not use potent steroids on your own. Gentle moisturizers and vitamin C products may help lighten spots. | | | | | | |
| **Item / 项目** | | **Q1 / 问题1** | | **Q2 / 问题2** | | **Q3 / 问题3** |
| **Patient question (Chinese original) / 患者原始问题（中文）** | | 我产后脸上色斑变明显，会自己淡吗？ | | 哺乳期可以使用护肤品或防晒吗？ | | 如果皮肤瘙痒起疹，需要看皮肤科吗？ |
| **English translation of patient question / 患者问题英文翻译** | | The dark patches on my face became more obvious after birth. Will they fade on their own? | | Can I use skincare products or sunscreen while breastfeeding? | | If I have itchy skin or a rash, do I need to see a dermatologist? |
| **Model response (Chinese) / 模型回答（中文）** | | 会变淡，但需防晒。 | | 可以，用温和产品及物理防晒。 | | 需要。 |
| **English translation of model response / 模型回答英文翻译** | | Fades over time but needs sun protection. | | Yes, use gentle products and physical sunscreen. | | Yes. |
| **Overall evaluation item / 整体评价项目** | | | **Score / 分数** | | **Comments / 备注** | |
| **Patient Satisfaction / 患者满意度** | | | 3 | |  | |
| **Patient Understandability / 患者可理解性** | | | 4 | |  | |
| **Patient Usefulness / 患者实用性** | | | 3 | |  | |
| **Clinician Correctness / 医生评分：正确性** | | | 3 | |  | |
| **Clinician Completeness / 医生评分：完整性** | | | 3 | |  | |
| **Clinician Readability / 医生评分：可读性** | | | 5 | |  | |
| **Clinician Helpfulness / 医生评分：帮助性** | | | 3 | |  | |
| **Clinician Safety / 医生评分：安全性** | | | 3 | |  | |

**Patient 94**

| **Patient ID / 患者编号** | **Group / 模型组别** | | | | | |
| --- | --- | --- | --- | --- | --- | --- |
| P94 | DeepSeek V3 | | | | | |
| **General model response or patient education material (Chinese) / 模型总体回答或患者教育材料（中文）** | | | | | | |
| 妊娠纹在产后会从红色或紫红色逐渐变为银白色、白色，但不会完全消失。变淡的过程需要6-12个月甚至更久。产后腹部皮肤松弛可以通过按摩改善局部循环，但按摩并不能去除妊娠纹。真正有效的方法包括：坚持使用含有积雪草苷、透明质酸、维A酯的护肤品（维A酸类在哺乳期禁用），以及医学美容治疗（点阵激光、微针、射频等），但需等断奶后再考虑。市面上正规的祛纹产品一般不影响哺乳，因为它们外用于皮肤，吸收极少。请选择正规品牌，避免三无产品。同时保持皮肤湿润，适度锻炼核心肌群，改善整体体态。 | | | | | | |
| **English translation of the Chinese general response / 中文总体回答英文翻译** | | | | | | |
| Stretch marks will change from red/purple to silver-white over time, but they never completely disappear. Lightening takes 6-12 months or longer. Postpartum loose abdominal skin can be massaged to improve circulation, but massage does not remove stretch marks. More effective options include creams with Centella asiatica, hyaluronic acid, or vitamin A esters (retinoids are contraindicated during breastfeeding), and cosmetic procedures like fractional laser, microneedling, or radiofrequency — but wait until weaning. Reputable stretch mark products are generally safe during breastfeeding due to minimal absorption. Choose legitimate brands, keep skin hydrated, and exercise core muscles to improve overall appearance. | | | | | | |
| **Item / 项目** | | **Q1 / 问题1** | | **Q2 / 问题2** | | **Q3 / 问题3** |
| **Patient question (Chinese original) / 患者原始问题（中文）** | | 妊娠纹产后还能变淡吗？ | | 产后腹部皮肤松弛可以按摩吗？ | | 使用祛纹产品会不会影响哺乳？ |
| **English translation of patient question / 患者问题英文翻译** | | Can stretch marks become lighter after birth? | | Can I massage loose abdominal skin after birth? | | Will using stretch-mark products affect breastfeeding? |
| **Model response (Chinese) / 模型回答（中文）** | | 会从红色变为银白色，不能完全消失。 | | 可按摩改善循环，但不能去除。 | | 正规产品不影响。 |
| **English translation of model response / 模型回答英文翻译** | | Change from red to silver-white; never disappear completely. | | Massage improves circulation but does not remove. | | Standard products are safe. |
| **Overall evaluation item / 整体评价项目** | | | **Score / 分数** | | **Comments / 备注** | |
| **Patient Satisfaction / 患者满意度** | | | 3 | |  | |
| **Patient Understandability / 患者可理解性** | | | 4 | |  | |
| **Patient Usefulness / 患者实用性** | | | 3 | |  | |
| **Clinician Correctness / 医生评分：正确性** | | | 3 | |  | |
| **Clinician Completeness / 医生评分：完整性** | | | 3 | |  | |
| **Clinician Readability / 医生评分：可读性** | | | 5 | |  | |
| **Clinician Helpfulness / 医生评分：帮助性** | | | 3 | |  | |
| **Clinician Safety / 医生评分：安全性** | | | 3 | |  | |

**Patient 95**

| **Patient ID / 患者编号** | **Group / 模型组别** | | | | | |
| --- | --- | --- | --- | --- | --- | --- |
| P95 | DeepSeek V3 | | | | | |
| **General model response or patient education material (Chinese) / 模型总体回答或患者教育材料（中文）** | | | | | | |
| 坐月子洗澡后觉得怕冷，不是因为“不能洗”，而是因为产后体温调节中枢功能尚未完全恢复，加上浴室温度变化、洗浴后水分蒸发带走热量所致。您完全可以洗澡，但要采取正确方式：选择淋浴（绝对禁止盆浴，直到恶露干净），水温38-40℃，时间不超过10分钟。洗完后立即用干毛巾擦干全身，穿上保暖衣物，用电吹风热风彻底吹干头发。剖宫产切口未完全愈合前，可以用防水敷料贴住切口，淋浴后轻轻擦干周围皮肤，取下敷料，保持切口干燥。如果怕冷明显，可以浴室开浴霸，洗后喝一杯温姜茶。保持温暖，不会落下“月子病”。 | | | | | | |
| **English translation of the Chinese general response / 中文总体回答英文翻译** | | | | | | |
| Feeling cold after a shower during confinement is not because you “shouldn’t bathe,” but because the postpartum thermoregulatory system is still recovering, combined with temperature changes and evaporative cooling. You absolutely can shower, but do it correctly: choose a shower (no tub baths until lochia is gone), water at 38-40°C, for no more than 10 minutes. Immediately dry your body with a towel, put on warm clothes, and blow-dry your hair completely. For cesarean incisions not yet fully healed, cover with a waterproof dressing, shower, gently dry around it, remove the dressing, and keep the incision dry. If you feel very cold, use a bathroom heater and drink a cup of warm ginger tea afterward. Staying warm will not cause “confinement illness.” | | | | | | |
| **Item / 项目** | | **Q1 / 问题1** | | **Q2 / 问题2** | | **Q3 / 问题3** |
| **Patient question (Chinese original) / 患者原始问题（中文）** | | 坐月子洗澡后觉得怕冷，是不是不能洗？ | | 产后洗澡应该选择淋浴还是盆浴？ | | 剖宫产切口没完全愈合前洗澡要注意什么？ |
| **English translation of patient question / 患者问题英文翻译** | | I feel cold after showering during the confinement period. Does this mean I should not shower? | | After birth, should I choose a shower or a tub bath? | | What should I pay attention to when showering before a cesarean incision has fully healed? |
| **Model response (Chinese) / 模型回答（中文）** | | 不是，是体温调节未恢复，注意保暖即可。 | | 淋浴，恶露干净前禁止盆浴。 | | 贴防水敷料，速洗擦干。 |
| **English translation of model response / 模型回答英文翻译** | | No, due to poor thermoregulation; keep warm. | | Shower only; no tub baths until lochia stops. | | Cover with waterproof dressing, quick shower, pat dry. |
| **Overall evaluation item / 整体评价项目** | | | **Score / 分数** | | **Comments / 备注** | |
| **Patient Satisfaction / 患者满意度** | | | 3 | |  | |
| **Patient Understandability / 患者可理解性** | | | 4 | |  | |
| **Patient Usefulness / 患者实用性** | | | 3 | |  | |
| **Clinician Correctness / 医生评分：正确性** | | | 3 | |  | |
| **Clinician Completeness / 医生评分：完整性** | | | 3 | |  | |
| **Clinician Readability / 医生评分：可读性** | | | 5 | |  | |
| **Clinician Helpfulness / 医生评分：帮助性** | | | 3 | |  | |
| **Clinician Safety / 医生评分：安全性** | | | 3 | |  | |

**Patient 96**

| **Patient ID / 患者编号** | **Group / 模型组别** | | | | | |
| --- | --- | --- | --- | --- | --- | --- |
| P96 | DeepSeek V3 | | | | | |
| **General model response or patient education material (Chinese) / 模型总体回答或患者教育材料（中文）** | | | | | | |
| 产后可以吹空调睡觉，尤其是在夏季，否则过热会影响休息和情绪。关键是不要让空调风口直接对着您和宝宝。建议将空调温度设定在24-26℃，使用睡眠模式或加装挡风板。每天开窗通风1-2次，每次15-30分钟。如果吹空调后感觉关节酸痛，这并不说明“受凉生病了”，更可能是因为产后韧带松弛、疲劳姿势或长时间抱孩子导致的肌肉劳损。关节酸痛可以通过热敷、温和拉伸和改善姿势来缓解。空调本身不会导致关节炎。请放心使用空调，保持舒适室温对母婴都好。 | | | | | | |
| **English translation of the Chinese general response / 中文总体回答英文翻译** | | | | | | |
| Using air conditioning while sleeping postpartum is fine, especially in summer; overheating disrupts rest and mood. The key is not to blow air directly at you or the baby. Set the AC to 24-26°C, use sleep mode or a baffle. Open windows for ventilation 1-2 times daily for 15-30 minutes. If you feel joint aches after AC use, it does not mean you “got cold” — it is more likely due to postpartum ligament laxity, fatigue, or muscle strain from holding the baby. Joint aches can be relieved with warm compresses, gentle stretching, and improved posture. AC does not cause arthritis. Use it to maintain comfortable room temperature for both mother and baby. | | | | | | |
| **Item / 项目** | | **Q1 / 问题1** | | **Q2 / 问题2** | | **Q3 / 问题3** |
| **Patient question (Chinese original) / 患者原始问题（中文）** | | 产后可以吹空调睡觉吗？ | | 空调温度多少比较合适？ | | 如果吹空调后关节酸痛，是受凉了吗？ |
| **English translation of patient question / 患者问题英文翻译** | | Can I sleep with the air conditioner on after birth? | | What air-conditioning temperature is appropriate? | | If my joints ache after using air conditioning, does it mean I caught a chill? |
| **Model response (Chinese) / 模型回答（中文）** | | 可以，风口不直吹。 | | 24-26℃。 | | 多为疲劳或姿势问题，非“受凉”。 |
| **English translation of model response / 模型回答英文翻译** | | Yes, but avoid direct airflow. | | 24-26°C. | | Usually due to fatigue or posture, not "cold exposure". |
| **Overall evaluation item / 整体评价项目** | | | **Score / 分数** | | **Comments / 备注** | |
| **Patient Satisfaction / 患者满意度** | | | 3 | |  | |
| **Patient Understandability / 患者可理解性** | | | 4 | |  | |
| **Patient Usefulness / 患者实用性** | | | 3 | |  | |
| **Clinician Correctness / 医生评分：正确性** | | | 3 | |  | |
| **Clinician Completeness / 医生评分：完整性** | | | 3 | |  | |
| **Clinician Readability / 医生评分：可读性** | | | 5 | |  | |
| **Clinician Helpfulness / 医生评分：帮助性** | | | 3 | |  | |
| **Clinician Safety / 医生评分：安全性** | | | 3 | |  | |

**Patient 97**

| **Patient ID / 患者编号** | **Group / 模型组别** | | | | | |
| --- | --- | --- | --- | --- | --- | --- |
| P97 | DeepSeek V3 | | | | | |
| **General model response or patient education material (Chinese) / 模型总体回答或患者教育材料（中文）** | | | | | | |
| 坐月子可以吃常温的水果，不需要加热到烫嘴，但避免吃从冰箱里刚拿出来的冰镇水果，以免刺激胃肠道。产后饮食不需要完全忌口辛辣，少量吃辣椒、大蒜等通常不会影响伤口愈合或乳汁质量，但如果您发现吃了某种食物后宝宝出现烦躁、胀气、皮疹或腹泻，可以暂停该食物2周再试，观察是否相关。常见的易过敏食物包括牛奶、鸡蛋、坚果、海鲜等。如果宝宝没有不适，您完全可以正常饮食。关键是均衡营养，而不是盲目忌口。摄入充足的蔬菜水果有利于预防产后便秘。 | | | | | | |
| **English translation of the Chinese general response / 中文总体回答英文翻译** | | | | | | |
| Fruits at room temperature are fine during confinement; no need to heat them until hot, but avoid fruits straight from the refrigerator to prevent gastrointestinal discomfort. You do not need to completely avoid spicy foods postpartum. Small amounts of chili, garlic, etc., usually do not affect wound healing or milk quality. However, if you notice that after eating a certain food, your baby becomes fussy, gassy, develops a rash or diarrhea, avoid that food for 2 weeks then try again to see if it is related. Common allergens include milk, eggs, nuts, and seafood. If the baby is fine, eat a normal diet. Balanced nutrition, not unnecessary avoidance, is key. Adequate fruits and vegetables help prevent postpartum constipation. | | | | | | |
| **Item / 项目** | | **Q1 / 问题1** | | **Q2 / 问题2** | | **Q3 / 问题3** |
| **Patient question (Chinese original) / 患者原始问题（中文）** | | 坐月子是不是不能吃凉的水果？ | | 产后饮食需要完全忌口辛辣吗？ | | 如果吃了某些食物后宝宝胀气，是不是要停止母乳？ |
| **English translation of patient question / 患者问题英文翻译** | | Is it true that I cannot eat cold fruit during the confinement period? | | Do I need to completely avoid spicy foods after birth? | | If the baby has gas after I eat certain foods, do I need to stop breastfeeding? |
| **Model response (Chinese) / 模型回答（中文）** | | 可以吃常温水果，避免冰镇。 | | 不必完全忌口，少量观察宝宝反应。 | | 暂停该食物2周再试。 |
| **English translation of model response / 模型回答英文翻译** | | Fruits at room temperature are fine; avoid refrigerated. | | No need to completely avoid; try small amounts and observe baby. | | Stop that food for 2 weeks then rechallenge. |
| **Overall evaluation item / 整体评价项目** | | | **Score / 分数** | | **Comments / 备注** | |
| **Patient Satisfaction / 患者满意度** | | | 3 | |  | |
| **Patient Understandability / 患者可理解性** | | | 4 | |  | |
| **Patient Usefulness / 患者实用性** | | | 3 | |  | |
| **Clinician Correctness / 医生评分：正确性** | | | 3 | |  | |
| **Clinician Completeness / 医生评分：完整性** | | | 3 | |  | |
| **Clinician Readability / 医生评分：可读性** | | | 5 | |  | |
| **Clinician Helpfulness / 医生评分：帮助性** | | | 3 | |  | |
| **Clinician Safety / 医生评分：安全性** | | | 3 | |  | |

**Patient 98**

| **Patient ID / 患者编号** | **Group / 模型组别** | | | | | |
| --- | --- | --- | --- | --- | --- | --- |
| P98 | DeepSeek V3 | | | | | |
| **General model response or patient education material (Chinese) / 模型总体回答或患者教育材料（中文）** | | | | | | |
| 产后6周以上一般可以坐长途车，但要根据您的身体恢复情况。长时间坐车（超过4小时）会增加下肢深静脉血栓的风险，因为产后血液仍处于高凝状态。建议：途中每隔1-2小时停车，下车走动5-10分钟，或做踝泵运动（勾脚尖、绷脚尖）。穿医用弹力袜（白天穿）也有效。多喝水，不憋尿。如果在旅途中恶露突然增多（变为鲜红色、超过月经量）、出现头晕、胸闷、呼吸困难或单侧小腿肿痛，请立即停车休息，如症状不缓解或加重，应就近去医院。如果出发前您已经存在发热、大量出血或剧烈疼痛，请暂缓出行。 | | | | | | |
| **English translation of the Chinese general response / 中文总体回答英文翻译** | | | | | | |
| Long car rides are generally safe after 6 weeks postpartum, depending on your recovery. Prolonged sitting (>4 hours) increases the risk of deep vein thrombosis due to persistent hypercoagulability. Recommendations: stop every 1-2 hours to walk for 5-10 minutes, or do ankle pumps (point and flex feet). Wear medical compression stockings (during the day). Hydrate well. If during the trip lochia suddenly increases (bright red, heavier than a period), or you develop dizziness, chest tightness, shortness of breath, or unilateral calf swelling/pain, stop and rest; if symptoms persist or worsen, seek medical care nearby. If you already have fever, heavy bleeding, or severe pain before departure, postpone travel. | | | | | | |
| **Item / 项目** | | **Q1 / 问题1** | | **Q2 / 问题2** | | **Q3 / 问题3** |
| **Patient question (Chinese original) / 患者原始问题（中文）** | | 产后多久可以坐长途车回老家？ | | 长时间坐车会不会增加血栓风险？ | | 旅途中恶露增多或头晕应该怎么办？ |
| **English translation of patient question / 患者问题英文翻译** | | How long after birth can I take a long-distance car ride back to my hometown? | | Will sitting in a car for a long time increase the risk of thrombosis? | | What should I do if my lochia increases or I feel dizzy during the trip? |
| **Model response (Chinese) / 模型回答（中文）** | | 产后6周以上，途中每小时起身活动。 | | 会，穿弹力袜、多喝水。 | | 停车休息，必要时就医。 |
| **English translation of model response / 模型回答英文翻译** | | After 6 weeks; get up and move every hour. | | Yes; wear compression stockings and hydrate. | | Stop and rest; seek care if severe. |
| **Overall evaluation item / 整体评价项目** | | | **Score / 分数** | | **Comments / 备注** | |
| **Patient Satisfaction / 患者满意度** | | | 3 | |  | |
| **Patient Understandability / 患者可理解性** | | | 4 | |  | |
| **Patient Usefulness / 患者实用性** | | | 3 | |  | |
| **Clinician Correctness / 医生评分：正确性** | | | 3 | |  | |
| **Clinician Completeness / 医生评分：完整性** | | | 3 | |  | |
| **Clinician Readability / 医生评分：可读性** | | | 5 | |  | |
| **Clinician Helpfulness / 医生评分：帮助性** | | | 3 | |  | |
| **Clinician Safety / 医生评分：安全性** | | | 3 | |  | |

**Patient 99**

| **Patient ID / 患者编号** | **Group / 模型组别** | | | | | |
| --- | --- | --- | --- | --- | --- | --- |
| P99 | DeepSeek V3 | | | | | |
| **General model response or patient education material (Chinese) / 模型总体回答或患者教育材料（中文）** | | | | | | |
| 剖宫产后开车，一般建议至少等到术后6-8周，并且要经过医生确认切口愈合良好、腹部肌力基本恢复。因为急刹车或突然变向时需要腹部肌肉和核心力量，如果力量不足，不仅会牵拉切口引起疼痛，还可能影响反应时间，增加事故风险。在开车前，您可以尝试在停车状态下模拟急刹车动作：快速用右脚踩死刹车，感受是否有切口疼痛或无力感。如果开车后出现腹部疼痛，应停车休息；如果疼痛持续或加剧，需到医院复查。最初几次开车尽量短途，并有人陪同。完全恢复通常需要3个月以上。 | | | | | | |
| **English translation of the Chinese general response / 中文总体回答英文翻译** | | | | | | |
| Driving after a cesarean section is generally recommended after 6-8 weeks, following confirmation of good wound healing and adequate abdominal strength. Emergency braking or sudden turns require abdominal muscle power; if insufficient, you may experience pain, delayed reaction, and increased accident risk. Before driving, simulate emergency braking while parked: quickly depress the brake pedal with your right foot and check for incisional pain or weakness. If you experience abdominal pain after driving, stop and rest; if pain persists or worsens, see a doctor. Start with short trips accompanied by someone. Full recovery may take >3 months. | | | | | | |
| **Item / 项目** | | **Q1 / 问题1** | | **Q2 / 问题2** | | **Q3 / 问题3** |
| **Patient question (Chinese original) / 患者原始问题（中文）** | | 剖宫产后多久可以自己开车？ | | 急刹车会不会牵拉切口？ | | 如果开车后腹部疼痛，需要休息还是复查？ |
| **English translation of patient question / 患者问题英文翻译** | | How long after a cesarean birth can I drive by myself? | | Could sudden braking pull on the incision? | | If I have abdominal pain after driving, should I rest or have a follow-up examination? |
| **Model response (Chinese) / 模型回答（中文）** | | 至少6-8周，确保紧急刹车时无痛。 | | 会，需确认腹部力量足够。 | | 先休息，持续则复查。 |
| **English translation of model response / 模型回答英文翻译** | | At least 6-8 weeks, ensure no pain during emergency braking. | | Yes, ensure abdominal strength adequate. | | Rest first; if persists, follow up. |
| **Overall evaluation item / 整体评价项目** | | | **Score / 分数** | | **Comments / 备注** | |
| **Patient Satisfaction / 患者满意度** | | | 3 | |  | |
| **Patient Understandability / 患者可理解性** | | | 4 | |  | |
| **Patient Usefulness / 患者实用性** | | | 3 | |  | |
| **Clinician Correctness / 医生评分：正确性** | | | 3 | |  | |
| **Clinician Completeness / 医生评分：完整性** | | | 3 | |  | |
| **Clinician Readability / 医生评分：可读性** | | | 5 | |  | |
| **Clinician Helpfulness / 医生评分：帮助性** | | | 3 | |  | |
| **Clinician Safety / 医生评分：安全性** | | | 3 | |  | |

**Patient 100**

| **Patient ID / 患者编号** | **Group / 模型组别** | | | | | |
| --- | --- | --- | --- | --- | --- | --- |
| P100 | DeepSeek V3 | | | | | |
| **General model response or patient education material (Chinese) / 模型总体回答或患者教育材料（中文）** | | | | | | |
| 产后提重物和做重家务，顺产建议6周内不提超过5公斤的物品，剖宫产建议8周内不提。抱大宝（体重通常已经超过5-10公斤）会显著增加盆底压力和切口张力，可能诱发或加重盆腔器官脱垂、尿失禁，以及腹直肌分离。您需要调整抱姿：尽量坐着抱，让大宝坐在您腿上，您用双臂环抱，腹部不承力；或者使用婴儿背带/腰凳将重量分散到肩背。如果提重物后感觉到会阴部或下腹下坠感明显，说明活动量过大，应立即停止，卧床休息，并做凯格尔训练。若下坠感持续不缓解或出现漏尿加重，请做盆底康复评估。 | | | | | | |
| **English translation of the Chinese general response / 中文总体回答英文翻译** | | | | | | |
| For heavy lifting and strenuous housework, avoid lifting >5 kg for 6 weeks after vaginal delivery and 8 weeks after cesarean section. Lifting an older child (usually >5-10 kg) significantly increases pelvic floor pressure and wound tension, potentially causing or worsening prolapse, incontinence, and diastasis recti. Modify your technique: lift while sitting, with the child on your lap and your arms around them, avoiding abdominal strain; or use a baby carrier to distribute weight to shoulders/back. If you feel marked pelvic heaviness after lifting, stop immediately, rest lying down, and do Kegel exercises. If heaviness persists or leakage worsens, seek pelvic floor assessment. | | | | | | |
| **Item / 项目** | | **Q1 / 问题1** | | **Q2 / 问题2** | | **Q3 / 问题3** |
| **Patient question (Chinese original) / 患者原始问题（中文）** | | 产后多久可以提重物或做家务？ | | 抱大宝会不会影响盆底和切口恢复？ | | 如果提重物后下坠感明显，需要减少活动吗？ |
| **English translation of patient question / 患者问题英文翻译** | | How long after birth can I lift heavy objects or do housework? | | Will carrying my older child affect pelvic floor and incision recovery? | | If I have an obvious downward pressure sensation after lifting heavy objects, should I reduce activity? |
| **Model response (Chinese) / 模型回答（中文）** | | 6周内不提>5kg，8周后渐增。 | | 会加重损伤，建议坐着抱或使用工具。 | | 需要，并做盆底评估。 |
| **English translation of model response / 模型回答英文翻译** | | Avoid lifting >5kg within 6 weeks; gradually after 8 weeks. | | Yes; sit while holding or use aids. | | Yes, reduce activity and get pelvic floor assessment. |
| **Overall evaluation item / 整体评价项目** | | | **Score / 分数** | | **Comments / 备注** | |
| **Patient Satisfaction / 患者满意度** | | | 3 | |  | |
| **Patient Understandability / 患者可理解性** | | | 4 | |  | |
| **Patient Usefulness / 患者实用性** | | | 3 | |  | |
| **Clinician Correctness / 医生评分：正确性** | | | 3 | |  | |
| **Clinician Completeness / 医生评分：完整性** | | | 3 | |  | |
| **Clinician Readability / 医生评分：可读性** | | | 5 | |  | |
| **Clinician Helpfulness / 医生评分：帮助性** | | | 3 | |  | |
| **Clinician Safety / 医生评分：安全性** | | | 3 | |  | |

**Patient 101**

| **Patient ID / 患者编号** | **Group / 模型组别** | | | | | |
| --- | --- | --- | --- | --- | --- | --- |
| P101 | DeepSeek V3 | | | | | |
| **General model response or patient education material (Chinese) / 模型总体回答或患者教育材料（中文）** | | | | | | |
| 剖宫产后再次怀孕，安全间隔建议为18-24个月。间隔过短（少于12个月）会增加子宫破裂、前置胎盘、胎盘植入和早产的风险。这是因为子宫疤痕需要充分愈合和重塑，时间不足会导致疤痕组织薄弱。在计划再孕前，建议您做一次阴道超声评估子宫下段疤痕的连续性（测量疤痕厚度和有无缺损，即切口憩室）。如果疤痕愈合不良（如憩室较大、残留肌层薄），医生可能会建议更长的间隔或孕前手术修复。即使间隔足够，下次妊娠也应在产科高危门诊管理，分娩方式需个体化评估。 | | | | | | |
| **English translation of the Chinese general response / 中文总体回答英文翻译** | | | | | | |
| The recommended interpregnancy interval after a cesarean section is 18-24 months. Short intervals (<12 months) increase risks of uterine rupture, placenta previa, placenta accreta, and preterm birth. Uterine scars need adequate time to heal and remodel. Before planning another pregnancy, have a transvaginal ultrasound to assess scar continuity (measure thickness and presence of a defect, i.e., isthmocele). If the scar is poorly healed (large defect, thin residual myometrium), a longer interval or preoperative scar revision may be advised. Even with an appropriate interval, subsequent pregnancies should be managed in a high-risk obstetric clinic. | | | | | | |
| **Item / 项目** | | **Q1 / 问题1** | | **Q2 / 问题2** | | **Q3 / 问题3** |
| **Patient question (Chinese original) / 患者原始问题（中文）** | | 剖宫产后多久可以再次怀孕比较安全？ | | 两次怀孕间隔太短会有什么风险？ | | 计划再孕前需要检查子宫疤痕吗？ |
| **English translation of patient question / 患者问题英文翻译** | | How long after a cesarean birth is it safer to become pregnant again? | | What are the risks if the interval between pregnancies is too short? | | Before planning another pregnancy, do I need to check the uterine scar? |
| **Model response (Chinese) / 模型回答（中文）** | | 建议间隔18-24个月。 | | 子宫破裂、前置胎盘风险增加。 | | 需要，做阴道超声评估疤痕连续性。 |
| **English translation of model response / 模型回答英文翻译** | | Recommend 18-24 months. | | Increased risk of uterine rupture and placenta previa. | | Yes, transvaginal ultrasound to assess scar integrity. |
| **Overall evaluation item / 整体评价项目** | | | **Score / 分数** | | **Comments / 备注** | |
| **Patient Satisfaction / 患者满意度** | | | 3 | |  | |
| **Patient Understandability / 患者可理解性** | | | 4 | |  | |
| **Patient Usefulness / 患者实用性** | | | 3 | |  | |
| **Clinician Correctness / 医生评分：正确性** | | | 3 | |  | |
| **Clinician Completeness / 医生评分：完整性** | | | 3 | |  | |
| **Clinician Readability / 医生评分：可读性** | | | 5 | |  | |
| **Clinician Helpfulness / 医生评分：帮助性** | | | 3 | |  | |
| **Clinician Safety / 医生评分：安全性** | | | 3 | |  | |

**Patient 102**

| **Patient ID / 患者编号** | **Group / 模型组别** | | | | | |
| --- | --- | --- | --- | --- | --- | --- |
| P102 | DeepSeek V3 | | | | | |
| **General model response or patient education material (Chinese) / 模型总体回答或患者教育材料（中文）** | | | | | | |
| 第一胎剖宫产，第二胎有机会顺产（VBAC），但需要满足条件：上次剖宫产为子宫下段横切口、无子宫破裂史、本次妊娠无剖宫产指征（如前置胎盘、巨大儿）、医院具备紧急剖宫产条件。疤痕厚度不是决定VBAC成功与否的唯一指标，但过薄的疤痕（<2.0-2.5mm）会降低成功率并增加风险。备孕时请带上上一次的手术记录（包括手术指征、切口类型、缝合方式、有无术后感染）给产科医生看。医生会综合评估，并在孕晚期再次评估，和您共同决定分娩方式。VBAC成功率约60-80%，子宫破裂风险约0.5-1%。 | | | | | | |
| **English translation of the Chinese general response / 中文总体回答英文翻译** | | | | | | |
| Vaginal birth after cesarean (VBAC) is possible but requires certain criteria: previous low-transverse uterine incision, no prior uterine rupture, no current contraindications (e.g., placenta previa, macrosomia), and a hospital capable of emergency C-section. Scar thickness is not the only predictor but very thin scars (<2.0-2.5 mm) lower success and increase risk. When planning pregnancy, bring your previous operative report (indication, incision type, suture technique, any infection) to your obstetrician. They will make an overall assessment and decide together with you near term. VBAC success rate is about 60-80%, and uterine rupture risk about 0.5-1%. | | | | | | |
| **Item / 项目** | | **Q1 / 问题1** | | **Q2 / 问题2** | | **Q3 / 问题3** |
| **Patient question (Chinese original) / 患者原始问题（中文）** | | 第一胎剖宫产，下一胎有没有机会顺产？ | | 剖宫产疤痕厚度会影响分娩方式吗？ | | 备孕前需要把上次手术记录带给医生看吗？ |
| **English translation of patient question / 患者问题英文翻译** | | After a first cesarean birth, is there a chance of vaginal birth in the next pregnancy? | | Will the thickness of the cesarean scar affect the delivery method? | | Before trying to conceive, do I need to bring the previous surgical record to the doctor? |
| **Model response (Chinese) / 模型回答（中文）** | | 有机会（VBAC），需符合条件。 | | 是参考指标之一。 | | 需要。 |
| **English translation of model response / 模型回答英文翻译** | | Possible (VBAC) if criteria met. | | It is one factor. | | Yes. |
| **Overall evaluation item / 整体评价项目** | | | **Score / 分数** | | **Comments / 备注** | |
| **Patient Satisfaction / 患者满意度** | | | 3 | |  | |
| **Patient Understandability / 患者可理解性** | | | 4 | |  | |
| **Patient Usefulness / 患者实用性** | | | 3 | |  | |
| **Clinician Correctness / 医生评分：正确性** | | | 3 | |  | |
| **Clinician Completeness / 医生评分：完整性** | | | 3 | |  | |
| **Clinician Readability / 医生评分：可读性** | | | 4 | |  | |
| **Clinician Helpfulness / 医生评分：帮助性** | | | 3 | |  | |
| **Clinician Safety / 医生评分：安全性** | | | 3 | |  | |

**Patient 103**

| **Patient ID / 患者编号** | **Group / 模型组别** | | | | | |
| --- | --- | --- | --- | --- | --- | --- |
| P103 | DeepSeek V3 | | | | | |
| **General model response or patient education material (Chinese) / 模型总体回答或患者教育材料（中文）** | | | | | | |
| 双胎产后恢复通常比单胎慢，因为子宫过度扩张、孕期体重增加更多、产后出血风险更高。双胎产妇更容易发生产后贫血（血容量增加更大导致血液稀释）和盆底功能障碍（双胎妊娠对盆底组织的压力更大）。产后42天复查时一定要查血常规和盆底评估。照顾两个宝宝非常劳累，您需要主动安排休息：家人实行“轮班制”，例如妈妈负责夜间喂奶，伴侣负责换尿布和安抚，白天家中老人或月嫂负责一个宝宝，您只负责另一个。每天保证有连续3-4小时的睡眠。如果觉得无法应对，不要犹豫，请用月嫂或申请社区产后护理支持。 | | | | | | |
| **English translation of the Chinese general response / 中文总体回答英文翻译** | | | | | | |
| Recovery after twins is usually slower than after a singleton due to overdistended uterus, greater weight gain, and higher risk of postpartum hemorrhage. Twin mothers are more prone to anemia (greater plasma volume expansion) and pelvic floor dysfunction (increased pressure). At the 42-day check-up, definitely get a CBC and pelvic floor assessment. Caring for two babies is exhausting; schedule rest with family using a shift system. For example, mother handles night feeds, partner handles diapers and soothing, grandparents or a nanny care for one baby during the day, leaving you with only the other. Aim for 3-4 hours of uninterrupted sleep daily. If overwhelmed, hire a postpartum doula or seek community support. | | | | | | |
| **Item / 项目** | | **Q1 / 问题1** | | **Q2 / 问题2** | | **Q3 / 问题3** |
| **Patient question (Chinese original) / 患者原始问题（中文）** | | 双胎生产后恢复会不会比单胎更慢？ | | 双胎产后更容易贫血或盆底问题吗？ | | 照顾两个宝宝太累时怎样安排休息？ |
| **English translation of patient question / 患者问题英文翻译** | | Will recovery after twin delivery be slower than after a singleton delivery? | | Are anemia or pelvic floor problems more likely after twin delivery? | | When caring for two babies is too tiring, how should I arrange rest? |
| **Model response (Chinese) / 模型回答（中文）** | | 通常更慢，因子宫过度扩张。 | | 是。 | | 家人轮换值班，保证休息。 |
| **English translation of model response / 模型回答英文翻译** | | Usually slower due to overdistended uterus. | | Yes. | | Rotate shifts with family to ensure rest. |
| **Overall evaluation item / 整体评价项目** | | | **Score / 分数** | | **Comments / 备注** | |
| **Patient Satisfaction / 患者满意度** | | | 3 | |  | |
| **Patient Understandability / 患者可理解性** | | | 4 | |  | |
| **Patient Usefulness / 患者实用性** | | | 3 | |  | |
| **Clinician Correctness / 医生评分：正确性** | | | 3 | |  | |
| **Clinician Completeness / 医生评分：完整性** | | | 3 | |  | |
| **Clinician Readability / 医生评分：可读性** | | | 5 | |  | |
| **Clinician Helpfulness / 医生评分：帮助性** | | | 3 | |  | |
| **Clinician Safety / 医生评分：安全性** | | | 3 | |  | |

**Patient 104**

| **Patient ID / 患者编号** | **Group / 模型组别** | | | | | |
| --- | --- | --- | --- | --- | --- | --- |
| P104 | DeepSeek V3 | | | | | |
| **General model response or patient education material (Chinese) / 模型总体回答或患者教育材料（中文）** | | | | | | |
| 早产后您总担心宝宝，忽略自己恢复，这是可以理解的，但请记住：只有您自己恢复好了，才能更好地照顾宝宝。宝宝在新生儿重症监护室期间，您仍然需要按时做产后复查（42天），尤其是如果早产伴随胎盘残留、感染或高血压等情况。压力大会抑制催乳素分泌，导致奶量不足。您可以向新生儿科的护士或哺乳指导求助，学习用手挤奶或吸奶器追奶，维持泌乳。同时，您也需要心理支持：早产妈妈的焦虑和愧疚感非常常见，可以和同病房的妈妈交流，或寻求心理医生帮助。宝宝最终会回家，您需要健康的身体和心态。 | | | | | | |
| **English translation of the Chinese general response / 中文总体回答英文翻译** | | | | | | |
| Worrying about your preterm baby while neglecting your own recovery is understandable, but remember: you need to recover well to care for your baby. While your baby is in the NICU, still attend your 42-day postpartum check-up, especially if preterm birth was complicated by retained placenta, infection, or hypertension. Stress reduces prolactin and milk supply. Ask NICU nurses or lactation consultants to teach hand expression or pumping to maintain lactation. You also need psychological support: anxiety and guilt are common among preterm mothers. Talk to other NICU mothers or seek a psychologist. Your baby will come home eventually; you need a healthy body and mind. | | | | | | |
| **Item / 项目** | | **Q1 / 问题1** | | **Q2 / 问题2** | | **Q3 / 问题3** |
| **Patient question (Chinese original) / 患者原始问题（中文）** | | 早产后我总担心宝宝，也忽略自己恢复，怎么办？ | | 宝宝住院期间我还需要做产后复查吗？ | | 压力太大影响泌乳时可以找谁帮助？ |
| **English translation of patient question / 患者问题英文翻译** | | After preterm birth, I always worry about the baby and neglect my own recovery. What should I do? | | While the baby is hospitalized, do I still need a postpartum check-up? | | If too much stress affects milk production, who can I ask for help? |
| **Model response (Chinese) / 模型回答（中文）** | | 先关注自身恢复，才能更好照顾宝宝。 | | 需要，产后42天复查不能省。 | | 心理支持或母乳喂养指导。 |
| **English translation of model response / 模型回答英文翻译** | | Focus on your own recovery first. | | Yes, 6-week checkup is necessary. | | Seek psychological support or lactation guidance. |
| **Overall evaluation item / 整体评价项目** | | | **Score / 分数** | | **Comments / 备注** | |
| **Patient Satisfaction / 患者满意度** | | | 3 | |  | |
| **Patient Understandability / 患者可理解性** | | | 4 | |  | |
| **Patient Usefulness / 患者实用性** | | | 3 | |  | |
| **Clinician Correctness / 医生评分：正确性** | | | 3 | |  | |
| **Clinician Completeness / 医生评分：完整性** | | | 3 | |  | |
| **Clinician Readability / 医生评分：可读性** | | | 5 | |  | |
| **Clinician Helpfulness / 医生评分：帮助性** | | | 3 | |  | |
| **Clinician Safety / 医生评分：安全性** | | | 3 | |  | |

**Patient 105**

| **Patient ID / 患者编号** | **Group / 模型组别** | | | | | |
| --- | --- | --- | --- | --- | --- | --- |
| P105 | DeepSeek V3 | | | | | |
| **General model response or patient education material (Chinese) / 模型总体回答或患者教育材料（中文）** | | | | | | |
| 如果产后经历了抢救或大出血（产后出血量≥1000毫升），恢复期要特别注意：加强营养（高蛋白、高铁、高维生素），口服铁剂至少3个月，避免劳累和再次怀孕至少1年。大出血后2周和6周应复查血常规，评估贫血改善情况。如果总是害怕回想分娩过程，做噩梦、回避与分娩相关的事情、心跳加速、出汗，可能是创伤后应激障碍，需要心理干预。请不要默默承受，告诉您的产科医生或心理医生。认知行为治疗和眼动脱敏再加工治疗对PTSD有效。同时，家人要多陪伴、多倾听，不要催促“快点忘记”。 | | | | | | |
| **English translation of the Chinese general response / 中文总体回答英文翻译** | | | | | | |
| If you experienced a massive hemorrhage (≥1000 ml) or resuscitation, focus on recovery: high-protein, high-iron, high-vitamin diet, oral iron for at least 3 months, avoid heavy work and pregnancy for at least 1 year. Repeat CBC at 2 and 6 weeks postpartum to assess anemia improvement. If you have persistent fear of recalling the delivery, nightmares, avoidance of delivery-related cues, palpitations, or sweating, this may be post-traumatic stress disorder (PTSD) and requires psychological intervention. Do not suffer in silence; tell your obstetrician or psychologist. Cognitive-behavioral therapy and EMDR are effective. Family members should accompany and listen, not pressure you to “forget it quickly.” | | | | | | |
| **Item / 项目** | | **Q1 / 问题1** | | **Q2 / 问题2** | | **Q3 / 问题3** |
| **Patient question (Chinese original) / 患者原始问题（中文）** | | 产后如果经历了抢救或大出血，身体恢复要注意什么？ | | 大出血后多久复查血常规比较合适？ | | 如果总是害怕回想分娩过程，需要心理支持吗？ |
| **English translation of patient question / 患者问题英文翻译** | | If I experienced emergency treatment or massive bleeding after birth, what should I pay attention to during recovery? | | How long after massive bleeding is it appropriate to repeat a complete blood count? | | If I am always afraid when recalling the birth process, do I need psychological support? |
| **Model response (Chinese) / 模型回答（中文）** | | 加强营养、补铁，避免劳累。 | | 2周后复查。 | | 需要，可能为创伤后应激障碍。 |
| **English translation of model response / 模型回答英文翻译** | | Strengthen nutrition, iron supplementation, avoid exertion. | | Repeat CBC after 2 weeks. | | Yes, possible PTSD; psychological intervention needed. |
| **Overall evaluation item / 整体评价项目** | | | **Score / 分数** | | **Comments / 备注** | |
| **Patient Satisfaction / 患者满意度** | | | 3 | |  | |
| **Patient Understandability / 患者可理解性** | | | 4 | |  | |
| **Patient Usefulness / 患者实用性** | | | 3 | |  | |
| **Clinician Correctness / 医生评分：正确性** | | | 3 | |  | |
| **Clinician Completeness / 医生评分：完整性** | | | 3 | |  | |
| **Clinician Readability / 医生评分：可读性** | | | 5 | |  | |
| **Clinician Helpfulness / 医生评分：帮助性** | | | 3 | |  | |
| **Clinician Safety / 医生评分：安全性** | | | 3 | |  | |

**Patient 106**

| **Patient ID / 患者编号** | **Group / 模型组别** | | | | | |
| --- | --- | --- | --- | --- | --- | --- |
| P106 | DeepSeek V3 | | | | | |
| **General model response or patient education material (Chinese) / 模型总体回答或患者教育材料（中文）** | | | | | | |
| 您孕期有胆汁淤积，产后应复查肝功能（ALT、AST、GGT）和总胆汁酸，一般在产后4-6周进行。大多数患者产后肝功能和胆汁酸会自行恢复正常，皮肤瘙痒也会随之消失。如果产后皮肤瘙痒持续不消失，或反复发作，需要排除其他肝病（如病毒性肝炎、原发性胆汁性胆管炎）或皮肤病。如果出现眼睛发黄、尿色加深（浓茶色）、大便颜色变浅、右上腹持续疼痛，请立即就医，查肝功能、胆红素和腹部超声。产后胆汁淤积不会传染给宝宝，也不会影响哺乳，但需要监测母亲肝脏情况。 | | | | | | |
| **English translation of the Chinese general response / 中文总体回答英文翻译** | | | | | | |
| You had intrahepatic cholestasis of pregnancy. Postpartum, check liver function (ALT, AST, GGT) and total bile acids at 4-6 weeks. Most patients normalize spontaneously, and pruritus resolves. If itching persists or recurs, rule out other liver diseases (viral hepatitis, primary biliary cholangitis) or dermatologic conditions. If you develop yellow eyes, dark urine (tea-colored), pale stools, or persistent right upper quadrant pain, seek immediate medical care for liver function, bilirubin, and abdominal ultrasound. Postpartum cholestasis does not affect the baby or breastfeeding, but maternal liver health needs monitoring. | | | | | | |
| **Item / 项目** | | **Q1 / 问题1** | | **Q2 / 问题2** | | **Q3 / 问题3** |
| **Patient question (Chinese original) / 患者原始问题（中文）** | | 我孕期有胆汁淤积，产后还需要复查肝功能吗？ | | 皮肤瘙痒产后没有完全消失正常吗？ | | 如果眼睛发黄或尿色很深，需要就医吗？ |
| **English translation of patient question / 患者问题英文翻译** | | I had cholestasis during pregnancy. Do I still need to recheck liver function after birth? | | Is it normal if skin itching has not completely disappeared after birth? | | If my eyes turn yellow or my urine is very dark, do I need medical care? |
| **Model response (Chinese) / 模型回答（中文）** | | 需要，复查肝功能和胆汁酸。 | | 不正常，需排除其他肝病或皮肤病。 | | 需要，立即就医。 |
| **English translation of model response / 模型回答英文翻译** | | Yes, repeat LFTs and bile acids. | | No; rule out other liver or skin diseases. | | Yes, immediately. |
| **Overall evaluation item / 整体评价项目** | | | **Score / 分数** | | **Comments / 备注** | |
| **Patient Satisfaction / 患者满意度** | | | 3 | |  | |
| **Patient Understandability / 患者可理解性** | | | 4 | |  | |
| **Patient Usefulness / 患者实用性** | | | 3 | |  | |
| **Clinician Correctness / 医生评分：正确性** | | | 3 | |  | |
| **Clinician Completeness / 医生评分：完整性** | | | 3 | |  | |
| **Clinician Readability / 医生评分：可读性** | | | 5 | |  | |
| **Clinician Helpfulness / 医生评分：帮助性** | | | 3 | |  | |
| **Clinician Safety / 医生评分：安全性** | | | 3 | |  | |

**Patient 107**

| **Patient ID / 患者编号** | **Group / 模型组别** | | | | | |
| --- | --- | --- | --- | --- | --- | --- |
| P107 | DeepSeek V3 | | | | | |
| **General model response or patient education material (Chinese) / 模型总体回答或患者教育材料（中文）** | | | | | | |
| 产后肝功能轻度异常，一般不影响哺乳，但需要明确病因。常见原因包括：妊娠期肝内胆汁淤积恢复期、HELLP综合征后、药物性肝损伤、或慢性乙型肝炎等。大多数保肝药（如谷胱甘肽、水飞蓟素、多烯磷脂酰胆碱）在哺乳期是安全的，但某些药物（如甘草酸制剂大剂量使用）需谨慎。请告知医生您正在哺乳。如果出现以下症状，需要尽快复查肝功能：恶心呕吐加重、明显乏力、皮肤巩膜黄染加深、尿色加深、右上腹疼痛加剧。如果肝功能持续异常超过6个月，应到肝病科进一步检查。 | | | | | | |
| **English translation of the Chinese general response / 中文总体回答英文翻译** | | | | | | |
| Mildly elevated postpartum liver enzymes generally do not require stopping breastfeeding, but the cause should be identified. Common causes: recovering from intrahepatic cholestasis, post-HELLP syndrome, drug-induced injury, or chronic hepatitis B. Most hepatoprotective drugs (glutathione, silymarin, polyenylphosphatidylcholine) are safe during breastfeeding, but some (e.g., high-dose glycyrrhizin) need caution. Tell your doctor you are breastfeeding. If you experience worsening nausea/vomiting, marked fatigue, deepening jaundice, dark urine, or worsening right upper quadrant pain, repeat LFTs promptly. If LFTs remain abnormal >6 months, see a hepatologist. | | | | | | |
| **Item / 项目** | | **Q1 / 问题1** | | **Q2 / 问题2** | | **Q3 / 问题3** |
| **Patient question (Chinese original) / 患者原始问题（中文）** | | 我产后肝功能有点异常，哺乳会受影响吗？ | | 吃保肝药期间还能喂奶吗？ | | 哪些症状说明需要尽快复查肝功能？ |
| **English translation of patient question / 患者问题英文翻译** | | My liver function is a little abnormal after birth. Will breastfeeding be affected? | | Can I breastfeed while taking liver-protective medication? | | Which symptoms mean I need to recheck liver function as soon as possible? |
| **Model response (Chinese) / 模型回答（中文）** | | 轻中度异常一般可哺乳，但需查明原因。 | | 部分药慎用，需咨询医生。 | | 乏力、恶心、黄疸加重、尿黄。 |
| **English translation of model response / 模型回答英文翻译** | | Mild to moderate elevation usually allows breastfeeding, but find cause. | | Some need caution; consult doctor. | | Worsening fatigue, nausea, jaundice, dark urine. |
| **Overall evaluation item / 整体评价项目** | | | **Score / 分数** | | **Comments / 备注** | |
| **Patient Satisfaction / 患者满意度** | | | 3 | |  | |
| **Patient Understandability / 患者可理解性** | | | 4 | |  | |
| **Patient Usefulness / 患者实用性** | | | 3 | |  | |
| **Clinician Correctness / 医生评分：正确性** | | | 3 | |  | |
| **Clinician Completeness / 医生评分：完整性** | | | 3 | |  | |
| **Clinician Readability / 医生评分：可读性** | | | 5 | |  | |
| **Clinician Helpfulness / 医生评分：帮助性** | | | 3 | |  | |
| **Clinician Safety / 医生评分：安全性** | | | 3 | |  | |

**Patient 108**

| **Patient ID / 患者编号** | **Group / 模型组别** | | | | | |
| --- | --- | --- | --- | --- | --- | --- |
| P108 | DeepSeek V3 | | | | | |
| **General model response or patient education material (Chinese) / 模型总体回答或患者教育材料（中文）** | | | | | | |
| 孕期有甲减，产后药量绝不能自己调整。因为产后甲状腺激素需求会发生剧烈变化，通常在分娩后1-2周内需要减少左甲状腺素剂量（很多患者可降至孕前剂量），产后6周左右复查后再精细调整。哺乳期吃左甲状腺素（优甲乐）对宝宝绝对安全，因为该药进入乳汁量极微，不会影响宝宝甲状腺功能，也不会抑制宝宝自身激素分泌。建议产后6-8周复查甲状腺功能（TSH、FT4），之后每4-6周复查一次直到稳定，然后每3个月复查一次至产后1年。如果出现心慌、怕热、体重下降或怕冷、乏力、便秘，随时复查。 | | | | | | |
| **English translation of the Chinese general response / 中文总体回答英文翻译** | | | | | | |
| If you had hypothyroidism during pregnancy, do not adjust your postpartum levothyroxine dose on your own. Thyroid hormone requirements change dramatically; usually the dose needs to be reduced within 1-2 weeks postpartum (often to pre-pregnancy dose), then fine-tuned after a check at around 6 weeks. Levothyroxine is absolutely safe during breastfeeding, as very little transfers to milk and does not affect the baby’s thyroid function. Check thyroid function (TSH, FT4) at 6-8 weeks postpartum, then every 4-6 weeks until stable, then every 3 months up to 1 year. If you develop palpitations, heat intolerance, weight loss, or conversely cold intolerance, fatigue, constipation, check earlier. | | | | | | |
| **Item / 项目** | | **Q1 / 问题1** | | **Q2 / 问题2** | | **Q3 / 问题3** |
| **Patient question (Chinese original) / 患者原始问题（中文）** | | 我孕期有甲减，产后药量需要自己调整吗？ | | 哺乳期吃优甲乐会不会影响宝宝？ | | 产后多久复查甲状腺功能比较合适？ |
| **English translation of patient question / 患者问题英文翻译** | | I had hypothyroidism during pregnancy. Should I adjust the medication dose by myself after birth? | | Will taking Euthyrox while breastfeeding affect the baby? | | How long after birth is it appropriate to recheck thyroid function? |
| **Model response (Chinese) / 模型回答（中文）** | | 不可以，需医生调整。 | | 不影响，安全。 | | 产后6-8周。 |
| **English translation of model response / 模型回答英文翻译** | | No, doctor adjusts dose. | | Safe, does not affect baby. | | 6-8 weeks postpartum. |
| **Overall evaluation item / 整体评价项目** | | | **Score / 分数** | | **Comments / 备注** | |
| **Patient Satisfaction / 患者满意度** | | | 3 | |  | |
| **Patient Understandability / 患者可理解性** | | | 4 | |  | |
| **Patient Usefulness / 患者实用性** | | | 3 | |  | |
| **Clinician Correctness / 医生评分：正确性** | | | 3 | |  | |
| **Clinician Completeness / 医生评分：完整性** | | | 3 | |  | |
| **Clinician Readability / 医生评分：可读性** | | | 4 | |  | |
| **Clinician Helpfulness / 医生评分：帮助性** | | | 3 | |  | |
| **Clinician Safety / 医生评分：安全性** | | | 3 | |  | |

**Patient 109**

| **Patient ID / 患者编号** | **Group / 模型组别** | | | | | |
| --- | --- | --- | --- | --- | --- | --- |
| P109 | DeepSeek V3 | | | | | |
| **General model response or patient education material (Chinese) / 模型总体回答或患者教育材料（中文）** | | | | | | |
| 产后牙龈出血可以看牙医，但要告知医生您最近分娩以及是否在哺乳。牙龈出血常见原因是妊娠期牙龈炎（激素水平变化导致）或产后没有恢复好的牙周问题。哺乳期可以安全地补牙、洗牙、拔牙，但需要避免X线检查（如必须拍片，可用铅衣保护，且不影响哺乳）。如果需要用药（如抗生素、止痛药），务必告诉牙医您在哺乳，他们会选择安全的药物（如阿莫西林、头孢类、对乙酰氨基酚、布洛芬）。避免使用含可待因的止痛药。局部麻醉（利多卡因、阿替卡因）也是安全的。不要因哺乳而延误治疗口腔感染。 | | | | | | |
| **English translation of the Chinese general response / 中文总体回答英文翻译** | | | | | | |
| Postpartum gum bleeding can be evaluated by a dentist; inform them of your recent delivery and that you are breastfeeding. Common causes are pregnancy gingivitis or unresolved periodontal issues. Breastfeeding mothers can safely have fillings, cleanings, and extractions, but avoid X-rays if possible (if necessary, use a lead apron; it does not affect milk). If medication is needed (antibiotics, pain relievers), tell the dentist you are breastfeeding so they can choose safe options (amoxicillin, cephalosporins, acetaminophen, ibuprofen). Avoid codeine-containing painkillers. Local anesthetics (lidocaine, articaine) are safe. Do not delay dental treatment for infection due to breastfeeding concerns. | | | | | | |
| **Item / 项目** | | **Q1 / 问题1** | | **Q2 / 问题2** | | **Q3 / 问题3** |
| **Patient question (Chinese original) / 患者原始问题（中文）** | | 产后牙龈出血可以看牙医吗？ | | 哺乳期能不能补牙或洗牙？ | | 如果牙痛需要用药，需要告诉牙医我在哺乳吗？ |
| **English translation of patient question / 患者问题英文翻译** | | Can I see a dentist for bleeding gums after birth? | | Can I have a filling or dental cleaning while breastfeeding? | | If I need medication for toothache, should I tell the dentist that I am breastfeeding? |
| **Model response (Chinese) / 模型回答（中文）** | | 可以，告知分娩史。 | | 可以（非X线）。 | | 需要。 |
| **English translation of model response / 模型回答英文翻译** | | Yes, inform about recent delivery. | | Yes (without X-ray). | | Yes. |
| **Overall evaluation item / 整体评价项目** | | | **Score / 分数** | | **Comments / 备注** | |
| **Patient Satisfaction / 患者满意度** | | | 3 | |  | |
| **Patient Understandability / 患者可理解性** | | | 4 | |  | |
| **Patient Usefulness / 患者实用性** | | | 3 | |  | |
| **Clinician Correctness / 医生评分：正确性** | | | 3 | |  | |
| **Clinician Completeness / 医生评分：完整性** | | | 3 | |  | |
| **Clinician Readability / 医生评分：可读性** | | | 4 | |  | |
| **Clinician Helpfulness / 医生评分：帮助性** | | | 3 | |  | |
| **Clinician Safety / 医生评分：安全性** | | | 3 | |  | |

**Patient 110**

| **Patient ID / 患者编号** | **Group / 模型组别** | | | | | |
| --- | --- | --- | --- | --- | --- | --- |
| P110 | DeepSeek V3 | | | | | |
| **General model response or patient education material (Chinese) / 模型总体回答或患者教育材料（中文）** | | | | | | |
| 产后看手机多了眼睛酸胀，是因为长时间近距离用眼导致视疲劳，加上产后睡眠不足、泪液分泌减少，并不会对产后恢复造成长期影响。建议您遵循“20-20-20”原则：每看手机20分钟，抬头看20英尺（6米）外的远处至少20秒。月子里可以正常阅读和使用电脑，但同样需要定时休息。如果出现视物模糊伴头痛，尤其是前额或后枕部跳痛，同时有血压升高、恶心，需立即测量血压，排除产后子痫前期或高血压脑病。单纯的视疲劳不会引起头痛和血压升高，如果这些症状同时出现，请及时就医。 | | | | | | |
| **English translation of the Chinese general response / 中文总体回答英文翻译** | | | | | | |
| Eye soreness after prolonged smartphone use is due to digital eye strain and insufficient sleep, not long-term harm. Follow the 20-20-20 rule: every 20 minutes, look 20 feet away for 20 seconds. Reading and computer use are allowed during confinement, but take regular breaks. If blurred vision occurs with headache (especially throbbing frontal or occipital), along with elevated blood pressure or nausea, measure BP immediately to rule out postpartum preeclampsia or hypertensive encephalopathy. Isolated eye strain does not cause headache and high BP; if these occur together, seek medical attention. | | | | | | |
| **Item / 项目** | | **Q1 / 问题1** | | **Q2 / 问题2** | | **Q3 / 问题3** |
| **Patient question (Chinese original) / 患者原始问题（中文）** | | 产后看手机多了眼睛酸胀，会不会影响恢复？ | | 月子里可以正常阅读和使用电脑吗？ | | 如果视物模糊伴头痛，需要测血压吗？ |
| **English translation of patient question / 患者问题英文翻译** | | My eyes feel sore and swollen from using my phone too much after birth. Will this affect recovery? | | Can I read and use a computer normally during the confinement period? | | If blurred vision is accompanied by headache, do I need to measure my blood pressure? |
| **Model response (Chinese) / 模型回答（中文）** | | 多为视疲劳，适当休息。 | | 可以，每30分钟休息5分钟。 | | 需要，排除子痫前期。 |
| **English translation of model response / 模型回答英文翻译** | | Usually eye strain; rest. | | Yes, take 5-min break every 30 minutes. | | Yes, to rule out preeclampsia. |
| **Overall evaluation item / 整体评价项目** | | | **Score / 分数** | | **Comments / 备注** | |
| **Patient Satisfaction / 患者满意度** | | | 3 | |  | |
| **Patient Understandability / 患者可理解性** | | | 4 | |  | |
| **Patient Usefulness / 患者实用性** | | | 3 | |  | |
| **Clinician Correctness / 医生评分：正确性** | | | 3 | |  | |
| **Clinician Completeness / 医生评分：完整性** | | | 3 | |  | |
| **Clinician Readability / 医生评分：可读性** | | | 5 | |  | |
| **Clinician Helpfulness / 医生评分：帮助性** | | | 3 | |  | |
| **Clinician Safety / 医生评分：安全性** | | | 3 | |  | |

**Patient 111**

| **Patient ID / 患者编号** | **Group / 模型组别** | | | | | |
| --- | --- | --- | --- | --- | --- | --- |
| P111 | DeepSeek V3 | | | | | |
| **General model response or patient education material (Chinese) / 模型总体回答或患者教育材料（中文）** | | | | | | |
| 打了无痛（硬膜外麻醉）后出现产后头痛，且躺下好转、坐起或站立时明显加重，这是典型的硬脊膜穿刺后头痛（PDPH），发生率约1-2%。原因是麻醉针意外刺破硬脊膜，导致脑脊液漏出、颅内压降低。大多数轻中度头痛在保守治疗下5-7天内自愈。您可以平卧休息、多喝水（每天3升以上）、饮用含咖啡因的饮料（如咖啡、茶，每天1-2杯），咖啡因可收缩脑血管缓解疼痛。如果头痛剧烈、无法坐起、伴有呕吐、视力模糊或复视，请立即就医，麻醉科医生可能会行“血补片”治疗（抽取您自身血液注入硬膜外腔封堵漏口），效果立竿见影。 | | | | | | |
| **English translation of the Chinese general response / 中文总体回答英文翻译** | | | | | | |
| Post-dural puncture headache (PDPH) after epidural anesthesia presents as a headache that is better lying down and worse sitting/standing. It occurs when the needle accidentally punctures the dura, causing cerebrospinal fluid leak and low intracranial pressure. Most mild to moderate cases resolve within 5-7 days with conservative treatment. Lie flat, hydrate (>3 L/day), and consume caffeinated beverages (1-2 cups coffee/tea) which constrict cerebral vessels. If headache is severe, prevents sitting up, or is accompanied by vomiting, blurred or double vision, seek immediate medical care. An anesthesiologist may perform an epidural blood patch (injecting your own blood to seal the leak), which provides immediate relief. | | | | | | |
| **Item / 项目** | | **Q1 / 问题1** | | **Q2 / 问题2** | | **Q3 / 问题3** |
| **Patient question (Chinese original) / 患者原始问题（中文）** | | 我打了无痛后产后头痛，是麻醉引起的吗？ | | 躺下好一些、坐起来头痛明显，需要注意什么？ | | 如果头痛很重或伴有呕吐，需要马上就医吗？ |
| **English translation of patient question / 患者问题英文翻译** | | I have a headache after birth after receiving labor analgesia. Could it be caused by anesthesia? | | The headache is better when I lie down and worse when I sit up. What should I pay attention to? | | If the headache is severe or accompanied by vomiting, do I need immediate medical care? |
| **Model response (Chinese) / 模型回答（中文）** | | 可能为硬脊膜穿刺后头痛。 | | 多饮水、咖啡因，平卧休息。 | | 需要，可能需血补丁治疗。 |
| **English translation of model response / 模型回答英文翻译** | | Possible post-dural puncture headache. | | Hydrate, caffeine, lie flat. | | Yes, possible blood patch. |
| **Overall evaluation item / 整体评价项目** | | | **Score / 分数** | | **Comments / 备注** | |
| **Patient Satisfaction / 患者满意度** | | | 3 | |  | |
| **Patient Understandability / 患者可理解性** | | | 4 | |  | |
| **Patient Usefulness / 患者实用性** | | | 3 | |  | |
| **Clinician Correctness / 医生评分：正确性** | | | 3 | |  | |
| **Clinician Completeness / 医生评分：完整性** | | | 3 | |  | |
| **Clinician Readability / 医生评分：可读性** | | | 5 | |  | |
| **Clinician Helpfulness / 医生评分：帮助性** | | | 3 | |  | |
| **Clinician Safety / 医生评分：安全性** | | | 3 | |  | |

**Patient 112**

| **Patient ID / 患者编号** | **Group / 模型组别** | | | | | |
| --- | --- | --- | --- | --- | --- | --- |
| P112 | DeepSeek V3 | | | | | |
| **General model response or patient education material (Chinese) / 模型总体回答或患者教育材料（中文）** | | | | | | |
| 产后腰背痛，绝大多数与打无痛无关。孕期松弛素导致韧带松弛，产后核心肌群薄弱，加上长时间弯腰喂奶、换尿布、抱孩子，才是主要原因。喂奶姿势至关重要：坐在有靠背的椅子上，腰后垫枕，使用哺乳枕将宝宝垫到乳房高度，避免您弯腰；也可以侧躺喂奶。如果腰痛向下放射到臀部、大腿后方，或伴有腿麻、足下垂、大小便异常，需排除腰椎间盘突出或骶髂关节紊乱。腰痛伴发热，要考虑肾盂肾炎或脊柱感染。这些情况需要骨科或康复科就诊。单纯的劳损性腰痛，通过姿势纠正和核心训练可以改善。 | | | | | | |
| **English translation of the Chinese general response / 中文总体回答英文翻译** | | | | | | |
| Postpartum back pain is almost never related to epidural anesthesia. The main causes are relaxin-induced ligament laxity, weak core muscles, and prolonged bending while feeding, changing diapers, or holding the baby. Feeding posture is key: sit in a supportive chair with a back pillow, use a nursing pillow to bring the baby to breast height, or side-lying feed. If pain radiates to the buttock or back of thigh, or is accompanied by leg numbness, foot drop, or bladder/bowel changes, rule out disc herniation or sacroiliac joint dysfunction. Back pain with fever suggests pyelonephritis or spinal infection. These require orthopaedic evaluation. Simple mechanical back pain improves with posture correction and core exercises. | | | | | | |
| **Item / 项目** | | **Q1 / 问题1** | | **Q2 / 问题2** | | **Q3 / 问题3** |
| **Patient question (Chinese original) / 患者原始问题（中文）** | | 我产后腰背痛，是不是打无痛留下的后遗症？ | | 喂奶姿势不好会不会加重腰背痛？ | | 腰痛伴发热或下肢麻木时需要检查吗？ |
| **English translation of patient question / 患者问题英文翻译** | | I have low back pain after birth. Is it a long-term effect of labor analgesia? | | Can a poor breastfeeding posture worsen low back pain? | | If low back pain is accompanied by fever or numbness in the lower limbs, do I need an examination? |
| **Model response (Chinese) / 模型回答（中文）** | | 不是，与韧带松弛、劳损有关。 | | 会，用哺乳枕支撑。 | | 需要，排除感染或神经问题。 |
| **English translation of model response / 模型回答英文翻译** | | No, due to ligament laxity and strain. | | Yes, use nursing pillow. | | Yes, to rule out infection or nerve issue. |
| **Overall evaluation item / 整体评价项目** | | | **Score / 分数** | | **Comments / 备注** | |
| **Patient Satisfaction / 患者满意度** | | | 3 | |  | |
| **Patient Understandability / 患者可理解性** | | | 4 | |  | |
| **Patient Usefulness / 患者实用性** | | | 3 | |  | |
| **Clinician Correctness / 医生评分：正确性** | | | 3 | |  | |
| **Clinician Completeness / 医生评分：完整性** | | | 3 | |  | |
| **Clinician Readability / 医生评分：可读性** | | | 5 | |  | |
| **Clinician Helpfulness / 医生评分：帮助性** | | | 3 | |  | |
| **Clinician Safety / 医生评分：安全性** | | | 3 | |  | |

**Patient 113**

| **Patient ID / 患者编号** | **Group / 模型组别** | | | | | |
| --- | --- | --- | --- | --- | --- | --- |
| P113 | DeepSeek V3 | | | | | |
| **General model response or patient education material (Chinese) / 模型总体回答或患者教育材料（中文）** | | | | | | |
| 剖宫产麻醉后腿麻，一般是因为麻醉药物尚未完全代谢，或硬膜外置管压迫神经。通常术后几小时到1-2天内会完全恢复。第一次下床前，先确认双腿有足够力量：在床上活动脚踝、勾脚尖、抬腿，感觉力量恢复后再尝试坐起。第一次下床必须有人搀扶，因为麻药可能影响平衡和血压，容易跌倒。如果术后3天仍存在下肢无力、感觉异常（如针扎感、烧灼感、对温度不敏感），或麻木范围扩大、伴有大小便失禁，需立即告知医生，排除硬膜外血肿或神经损伤（罕见）。绝大多数产妇恢复良好。 | | | | | | |
| **English translation of the Chinese general response / 中文总体回答英文翻译** | | | | | | |
| Leg numbness after cesarean anesthesia usually resolves within hours to 1-2 days as the drugs wear off or the epidural catheter is removed. Before first ambulation, check leg strength: wiggle ankles, point/flex toes, lift legs. Get up with assistance — anesthesia can affect balance and blood pressure, increasing fall risk. If leg weakness or sensory changes (pins, needles, burning, temperature insensitivity) persist beyond 3 days, or numbness expands and is accompanied by incontinence, immediately notify your doctor to rule out epidural hematoma or nerve injury (rare). The vast majority recover well. | | | | | | |
| **Item / 项目** | | **Q1 / 问题1** | | **Q2 / 问题2** | | **Q3 / 问题3** |
| **Patient question (Chinese original) / 患者原始问题（中文）** | | 剖宫产麻醉后腿麻多久能恢复？ | | 麻醉后下床第一次走路需要人扶吗？ | | 如果下肢一直无力或感觉异常，需要找医生吗？ |
| **English translation of patient question / 患者问题英文翻译** | | How long does leg numbness after cesarean anesthesia usually take to recover? | | When I get out of bed for the first time after anesthesia, do I need someone to support me? | | If my lower limbs remain weak or feel abnormal, do I need to contact a doctor? |
| **Model response (Chinese) / 模型回答（中文）** | | 几小时至1-2天。 | | 需要，防跌倒。 | | 需要。 |
| **English translation of model response / 模型回答英文翻译** | | Hours to 1-2 days. | | Yes, to prevent falls. | | Yes. |
| **Overall evaluation item / 整体评价项目** | | | **Score / 分数** | | **Comments / 备注** | |
| **Patient Satisfaction / 患者满意度** | | | 3 | |  | |
| **Patient Understandability / 患者可理解性** | | | 4 | |  | |
| **Patient Usefulness / 患者实用性** | | | 3 | |  | |
| **Clinician Correctness / 医生评分：正确性** | | | 3 | |  | |
| **Clinician Completeness / 医生评分：完整性** | | | 3 | |  | |
| **Clinician Readability / 医生评分：可读性** | | | 4 | |  | |
| **Clinician Helpfulness / 医生评分：帮助性** | | | 3 | |  | |
| **Clinician Safety / 医生评分：安全性** | | | 3 | |  | |

**Patient 114**

| **Patient ID / 患者编号** | **Group / 模型组别** | | | | | |
| --- | --- | --- | --- | --- | --- | --- |
| P114 | DeepSeek V3 | | | | | |
| **General model response or patient education material (Chinese) / 模型总体回答或患者教育材料（中文）** | | | | | | |
| 产后腿部偶尔麻木，多是因为长时间保持同一个姿势压迫神经所致，比如久坐、久卧、盘腿喂奶。活动或改变姿势后麻木感通常会很快消失。建议您不要久坐超过1小时，每40分钟左右起来走动一下；坐着时双脚平放，不要跷二郎腿；喂奶时经常换边，使用支撑枕。如果麻木出现在单条腿，同时伴有肿胀、疼痛、皮温升高、皮肤颜色变红或发紫，这是深静脉血栓的危险信号，请立即就医，不要按摩。此外，如果麻木持续存在、范围扩大，或伴有肌肉萎缩，需看神经科排查腰椎或外周神经问题。 | | | | | | |
| **English translation of the Chinese general response / 中文总体回答英文翻译** | | | | | | |
| Occasional postpartum leg numbness is usually positional nerve compression from prolonged sitting, lying, or cross-legged feeding. Numbness resolves quickly after moving. Do not sit for >1 hour without walking; keep feet flat on the floor, avoid crossing legs; change feeding positions often. If numbness occurs in one leg with swelling, pain, warmth, or redness, this suggests deep vein thrombosis — seek immediate care, do not massage. If numbness is persistent, expanding, or accompanied by muscle wasting, see a neurologist to rule out lumbar or peripheral nerve issues. | | | | | | |
| **Item / 项目** | | **Q1 / 问题1** | | **Q2 / 问题2** | | **Q3 / 问题3** |
| **Patient question (Chinese original) / 患者原始问题（中文）** | | 产后腿部偶尔麻木，是压迫神经了吗？ | | 坐久了腿麻应该怎么调整姿势？ | | 如果一条腿麻木伴肿胀疼痛，需要排查血栓吗？ |
| **English translation of patient question / 患者问题英文翻译** | | I occasionally have leg numbness after birth. Is this due to nerve compression? | | If my legs become numb after sitting for a long time, how should I adjust my posture? | | If one leg is numb with swelling and pain, do I need to rule out thrombosis? |
| **Model response (Chinese) / 模型回答（中文）** | | 多为姿势压迫，活动后缓解。 | | 改变姿势、走动。 | | 需要，紧急排查深静脉血栓。 |
| **English translation of model response / 模型回答英文翻译** | | Often positional; relieved by movement. | | Change position and walk. | | Yes, urgent rule out DVT. |
| **Overall evaluation item / 整体评价项目** | | | **Score / 分数** | | **Comments / 备注** | |
| **Patient Satisfaction / 患者满意度** | | | 3 | |  | |
| **Patient Understandability / 患者可理解性** | | | 4 | |  | |
| **Patient Usefulness / 患者实用性** | | | 3 | |  | |
| **Clinician Correctness / 医生评分：正确性** | | | 3 | |  | |
| **Clinician Completeness / 医生评分：完整性** | | | 3 | |  | |
| **Clinician Readability / 医生评分：可读性** | | | 5 | |  | |
| **Clinician Helpfulness / 医生评分：帮助性** | | | 3 | |  | |
| **Clinician Safety / 医生评分：安全性** | | | 3 | |  | |

**Patient 115**

| **Patient ID / 患者编号** | **Group / 模型组别** | | | | | |
| --- | --- | --- | --- | --- | --- | --- |
| P115 | DeepSeek V3 | | | | | |
| **General model response or patient education material (Chinese) / 模型总体回答或患者教育材料（中文）** | | | | | | |
| 产后活动少确实会增加血栓风险。预防措施包括：尽早下床活动（术后6小时内开始踝泵运动，术后24小时内下床走动）；多喝水（每天2000-2500毫升）；穿医用弹力袜（白天穿，晚上脱），尤其适合剖宫产、肥胖或有血栓史的产妇。如果小腿突然肿痛、发热、按压痛，绝对不要在家按摩、热敷或拉伸，因为可能使血栓脱落造成肺栓塞。请立即去医院做下肢血管超声。同时，注意观察有无胸痛、呼吸困难、咯血、晕厥，出现这些症状要直接去急诊。产后6周内都是血栓高风险期，不可大意。 | | | | | | |
| **English translation of the Chinese general response / 中文总体回答英文翻译** | | | | | | |
| Reduced activity postpartum increases thrombosis risk. Preventive measures: early ambulation (start ankle pumps within 6 hours, walk within 24 hours); hydrate (2000-2500 mL/day); wear medical compression stockings (daytime), especially after C-section, for obesity, or with history of thrombosis. If a calf suddenly becomes swollen, painful, warm, or tender, do not massage, apply heat, or stretch at home — this could dislodge a clot causing pulmonary embolism. Go immediately for a lower extremity ultrasound. Also watch for chest pain, dyspnea, hemoptysis, or fainting; if any develop, go to the ER. The risk remains high for 6 weeks postpartum. | | | | | | |
| **Item / 项目** | | **Q1 / 问题1** | | **Q2 / 问题2** | | **Q3 / 问题3** |
| **Patient question (Chinese original) / 患者原始问题（中文）** | | 我产后活动少，担心血栓，平时怎么预防？ | | 穿弹力袜对预防血栓有帮助吗？ | | 如果小腿突然肿痛，能不能先在家按摩？ |
| **English translation of patient question / 患者问题英文翻译** | | I move very little after birth and worry about blood clots. How can I prevent them in daily life? | | Are compression stockings helpful for preventing thrombosis? | | If my calf suddenly becomes swollen and painful, can I massage it at home first? |
| **Model response (Chinese) / 模型回答（中文）** | | 尽早下床、踝泵运动、多喝水。 | | 有帮助，高风险人群白天穿。 | | 不能，立即就医，禁止按摩。 |
| **English translation of model response / 模型回答英文翻译** | | Early ambulation, ankle pumps, adequate hydration. | | Yes, wear during daytime if high risk. | | No, go to hospital immediately; do not massage. |
| **Overall evaluation item / 整体评价项目** | | | **Score / 分数** | | **Comments / 备注** | |
| **Patient Satisfaction / 患者满意度** | | | 3 | |  | |
| **Patient Understandability / 患者可理解性** | | | 4 | |  | |
| **Patient Usefulness / 患者实用性** | | | 3 | |  | |
| **Clinician Correctness / 医生评分：正确性** | | | 3 | |  | |
| **Clinician Completeness / 医生评分：完整性** | | | 3 | |  | |
| **Clinician Readability / 医生评分：可读性** | | | 5 | |  | |
| **Clinician Helpfulness / 医生评分：帮助性** | | | 3 | |  | |
| **Clinician Safety / 医生评分：安全性** | | | 3 | |  | |

**Patient 116**

| **Patient ID / 患者编号** | **Group / 模型组别** | | | | | |
| --- | --- | --- | --- | --- | --- | --- |
| P116 | DeepSeek V3 | | | | | |
| **General model response or patient education material (Chinese) / 模型总体回答或患者教育材料（中文）** | | | | | | |
| 产后突然胸痛、气短，是非常危险的信号，必须立即急诊。最需要排除的急症包括：肺栓塞（血栓脱落至肺动脉）、围产期心肌病（心力衰竭）、以及气胸、急性心肌梗死等。产后心慌也很常见，但需要区分是良性心动过速（如贫血、焦虑、发热）还是心脏本身问题。如果心慌伴有晕厥感、黑矇、胸痛、呼吸困难，或静息心率持续超过120次/分，请勿在家观察。胸闷气短伴出冷汗、嘴唇发紫、血压下降，要警惕心源性休克或肺栓塞。告诉医生您最近的产次、分娩方式、有无血栓风险因素，他们会立即做心电图、心脏超声和D-二聚体检查。 | | | | | | |
| **English translation of the Chinese general response / 中文总体回答英文翻译** | | | | | | |
| Sudden chest pain and shortness of breath postpartum are red-flag symptoms requiring immediate emergency evaluation. Life-threatening conditions to rule out include pulmonary embolism, peripartum cardiomyopathy, pneumothorax, and acute myocardial infarction. Palpitations are common but need differentiation: benign causes (anemia, anxiety, fever) vs. cardiac pathology. If palpitations are accompanied by near-syncope, blackout, chest pain, dyspnea, or resting heart rate >120/min, do not observe at home. Chest tightness with cold sweat, cyanosis, or hypotension suggests cardiogenic shock or PE. Tell the doctor your delivery date, mode, and thrombosis risk factors; they will quickly perform ECG, echocardiogram, and D-dimer. | | | | | | |
| **Item / 项目** | | **Q1 / 问题1** | | **Q2 / 问题2** | | **Q3 / 问题3** |
| **Patient question (Chinese original) / 患者原始问题（中文）** | | 产后突然胸口疼、气短，可能是什么原因？ | | 产后心慌是贫血还是心脏问题？ | | 胸闷气短伴出冷汗时是不是要急诊？ |
| **English translation of patient question / 患者问题英文翻译** | | After birth, I suddenly have chest pain and shortness of breath. What could be the cause? | | Are postpartum palpitations due to anemia or a heart problem? | | If chest tightness and shortness of breath occur with cold sweats, do I need emergency care? |
| **Model response (Chinese) / 模型回答（中文）** | | 需警惕肺栓塞、心肌病或贫血。 | | 可能两者都有，需检查。 | | 是。 |
| **English translation of model response / 模型回答英文翻译** | | Suspect PE, cardiomyopathy, or anemia. | | Could be either; need evaluation. | | Yes. |
| **Overall evaluation item / 整体评价项目** | | | **Score / 分数** | | **Comments / 备注** | |
| **Patient Satisfaction / 患者满意度** | | | 3 | |  | |
| **Patient Understandability / 患者可理解性** | | | 4 | |  | |
| **Patient Usefulness / 患者实用性** | | | 3 | |  | |
| **Clinician Correctness / 医生评分：正确性** | | | 3 | |  | |
| **Clinician Completeness / 医生评分：完整性** | | | 3 | |  | |
| **Clinician Readability / 医生评分：可读性** | | | 5 | |  | |
| **Clinician Helpfulness / 医生评分：帮助性** | | | 3 | |  | |
| **Clinician Safety / 医生评分：安全性** | | | 3 | |  | |

**Patient 117**

| **Patient ID / 患者编号** | **Group / 模型组别** | | | | | |
| --- | --- | --- | --- | --- | --- | --- |
| P117 | DeepSeek V3 | | | | | |
| **General model response or patient education material (Chinese) / 模型总体回答或患者教育材料（中文）** | | | | | | |
| 有糖尿病的产妇，剖宫产切口愈合确实比血糖正常的产妇慢，而且感染风险更高。控制好血糖是促进愈合的关键。术后应监测血糖，空腹血糖控制在4.4-7.0 mmol/L，餐后2小时血糖<10.0 mmol/L。如果切口有渗液（清亮、淡黄色或脓性），除了处理伤口本身，一定要同时复查血糖和糖化血红蛋白，了解近2-3个月的血糖控制情况。如果血糖控制差，即使使用抗生素和局部换药，切口也很难愈合。可能需要内分泌科会诊调整降糖方案。哺乳期可以使用胰岛素和大多数口服降糖药（二甲双胍、格列本脲等），无需停止哺乳。 | | | | | | |
| **English translation of the Chinese general response / 中文总体回答英文翻译** | | | | | | |
| Mothers with diabetes have slower cesarean incision healing and higher infection risk. Good glycemic control is key. Monitor blood glucose postpartum; target fasting 4.4-7.0 mmol/L and 2h postprandial <10.0 mmol/L. If the incision has drainage (clear, yellow, or purulent), besides wound care, check blood glucose and HbA1c to assess control over the prior 2-3 months. Poor control impairs healing even with antibiotics. An endocrinology consult may be needed to adjust therapy. Insulin and most oral hypoglycemics (metformin, glyburide) are safe during breastfeeding; no need to stop. | | | | | | |
| **Item / 项目** | | **Q1 / 问题1** | | **Q2 / 问题2** | | **Q3 / 问题3** |
| **Patient question (Chinese original) / 患者原始问题（中文）** | | 我有糖尿病，剖宫产切口恢复会不会更慢？ | | 血糖控制不好会影响伤口愈合吗？ | | 切口有渗液时需要同时复查血糖吗？ |
| **English translation of patient question / 患者问题英文翻译** | | I have diabetes. Will my cesarean incision heal more slowly? | | Will poor blood glucose control affect wound healing? | | If there is discharge from the incision, do I also need to recheck blood glucose? |
| **Model response (Chinese) / 模型回答（中文）** | | 会，需加强血糖控制。 | | 会，增加感染和愈合不良风险。 | | 需要。 |
| **English translation of model response / 模型回答英文翻译** | | Yes, tighter glucose control needed. | | Yes, increases infection and poor healing risk. | | Yes. |
| **Overall evaluation item / 整体评价项目** | | | **Score / 分数** | | **Comments / 备注** | |
| **Patient Satisfaction / 患者满意度** | | | 3 | |  | |
| **Patient Understandability / 患者可理解性** | | | 4 | |  | |
| **Patient Usefulness / 患者实用性** | | | 3 | |  | |
| **Clinician Correctness / 医生评分：正确性** | | | 3 | |  | |
| **Clinician Completeness / 医生评分：完整性** | | | 3 | |  | |
| **Clinician Readability / 医生评分：可读性** | | | 5 | |  | |
| **Clinician Helpfulness / 医生评分：帮助性** | | | 3 | |  | |
| **Clinician Safety / 医生评分：安全性** | | | 3 | |  | |

**Patient 118**

| **Patient ID / 患者编号** | **Group / 模型组别** | | | | | |
| --- | --- | --- | --- | --- | --- | --- |
| P118 | DeepSeek V3 | | | | | |
| **General model response or patient education material (Chinese) / 模型总体回答或患者教育材料（中文）** | | | | | | |
| 产后尿频、尿急越来越明显，最常见的原因是尿路感染。因为分娩可能导致膀胱排空不全、导尿操作或会阴水肿，细菌容易滋生。请查尿常规，如果提示白细胞或亚硝酸盐阳性，需要抗生素治疗。哺乳期可以安全使用抗生素，如头孢菌素类、呋喃妥因、阿莫西林等。如果出现腰痛（肋脊角叩痛）、发热（≥38.5℃）、寒战，说明感染可能已上行至肾脏，即急性肾盂肾炎，需要立即就医，通常需要住院静脉抗生素治疗。在治疗期间，多喝水、勤排尿，不要憋尿。一般抗生素治疗3-5天症状会明显改善。 | | | | | | |
| **English translation of the Chinese general response / 中文总体回答英文翻译** | | | | | | |
| Worsening urinary frequency and urgency postpartum most often indicate a urinary tract infection. Incomplete bladder emptying, catheterization, or perineal edema can promote bacterial growth. Get a urinalysis. If white blood cells or nitrites are positive, antibiotics are needed. Safe breastfeeding options include cephalosporins, nitrofurantoin, or amoxicillin. If you develop flank pain (costovertebral angle tenderness), fever ≥38.5°C, or chills, the infection may have ascended to the kidneys — acute pyelonephritis — requiring immediate care, often with IV antibiotics. Hydrate well and void frequently. Symptoms typically improve within 3-5 days of appropriate antibiotics. | | | | | | |
| **Item / 项目** | | **Q1 / 问题1** | | **Q2 / 问题2** | | **Q3 / 问题3** |
| **Patient question (Chinese original) / 患者原始问题（中文）** | | 产后尿频尿急越来越明显，是尿路感染吗？ | | 哺乳期尿路感染可以吃药吗？ | | 如果有腰痛或发热，需要警惕肾盂肾炎吗？ |
| **English translation of patient question / 患者问题英文翻译** | | My urinary frequency and urgency are becoming more obvious after birth. Is this a urinary tract infection? | | Can urinary tract infection be treated with medication while breastfeeding? | | If I have low back pain or fever, do I need to be alert for pyelonephritis? |
| **Model response (Chinese) / 模型回答（中文）** | | 可能，查尿常规。 | | 可以，用头孢类或呋喃妥因等安全药物。 | | 需要，立即就医。 |
| **English translation of model response / 模型回答英文翻译** | | Possible; get urinalysis. | | Yes, use safe antibiotics like cephalosporins or nitrofurantoin. | | Yes, seek immediate care. |
| **Overall evaluation item / 整体评价项目** | | | **Score / 分数** | | **Comments / 备注** | |
| **Patient Satisfaction / 患者满意度** | | | 3 | |  | |
| **Patient Understandability / 患者可理解性** | | | 4 | |  | |
| **Patient Usefulness / 患者实用性** | | | 3 | |  | |
| **Clinician Correctness / 医生评分：正确性** | | | 3 | |  | |
| **Clinician Completeness / 医生评分：完整性** | | | 3 | |  | |
| **Clinician Readability / 医生评分：可读性** | | | 5 | |  | |
| **Clinician Helpfulness / 医生评分：帮助性** | | | 3 | |  | |
| **Clinician Safety / 医生评分：安全性** | | | 3 | |  | |

**Patient 119**

| **Patient ID / 患者编号** | **Group / 模型组别** | | | | | |
| --- | --- | --- | --- | --- | --- | --- |
| P119 | DeepSeek V3 | | | | | |
| **General model response or patient education material (Chinese) / 模型总体回答或患者教育材料（中文）** | | | | | | |
| 产后腰两侧酸痛，可能与肾脏有关，尤其是伴有发热、寒战、尿频尿急或排尿痛时，要怀疑肾盂肾炎。如果酸痛不伴发热，也可能是腰部肌肉劳损或腰椎问题。饮水过少会增加尿路感染和尿路结石的风险，因此产后每天应饮水2000-2500毫升。如果出现尿色很深（呈浓茶色、酱油色）或肉眼血尿，必须马上检查：尿常规、肾功能、泌尿系超声，排除肾小球肾炎、肾结石或肾静脉血栓。产后肾静脉血栓虽然罕见，但高危因素（剖宫产、子痫前期、肾病综合征）存在时可能发生，表现为突发腰痛、血尿、双下肢不对称水肿。 | | | | | | |
| **English translation of the Chinese general response / 中文总体回答英文翻译** | | | | | | |
| Postpartum flank pain may be renal in origin, especially with fever, chills, urinary frequency, or dysuria — suspect pyelonephritis. Without fever, it could be muscular strain. Low water intake increases UTI and stone risk; drink 2000-2500 mL daily. If urine is very dark (tea-colored, cola-colored) or you see gross hematuria, get immediate urinalysis, renal function, and urinary tract ultrasound to rule out glomerulonephritis, stones, or renal vein thrombosis. Postpartum renal vein thrombosis is rare but possible with risk factors (C-section, preeclampsia, nephrotic syndrome), presenting as sudden flank pain, hematuria, and asymmetric leg swelling. | | | | | | |
| **Item / 项目** | | **Q1 / 问题1** | | **Q2 / 问题2** | | **Q3 / 问题3** |
| **Patient question (Chinese original) / 患者原始问题（中文）** | | 我产后腰两侧酸痛，和肾脏有关系吗？ | | 喝水少会不会导致尿路问题？ | | 如果尿色很深或尿血，需要马上检查吗？ |
| **English translation of patient question / 患者问题英文翻译** | | I have soreness on both sides of my lower back after birth. Could it be related to the kidneys? | | Can drinking too little water cause urinary tract problems? | | If my urine is very dark or there is blood in the urine, do I need an immediate examination? |
| **Model response (Chinese) / 模型回答（中文）** | | 可能，如肾盂肾炎或结石。 | | 会。 | | 需要。 |
| **English translation of model response / 模型回答英文翻译** | | Possible, e.g., pyelonephritis or stone. | | Yes. | | Yes. |
| **Overall evaluation item / 整体评价项目** | | | **Score / 分数** | | **Comments / 备注** | |
| **Patient Satisfaction / 患者满意度** | | | 3 | |  | |
| **Patient Understandability / 患者可理解性** | | | 4 | |  | |
| **Patient Usefulness / 患者实用性** | | | 3 | |  | |
| **Clinician Correctness / 医生评分：正确性** | | | 3 | |  | |
| **Clinician Completeness / 医生评分：完整性** | | | 3 | |  | |
| **Clinician Readability / 医生评分：可读性** | | | 4 | |  | |
| **Clinician Helpfulness / 医生评分：帮助性** | | | 3 | |  | |
| **Clinician Safety / 医生评分：安全性** | | | 3 | |  | |

**Patient 120**

| **Patient ID / 患者编号** | **Group / 模型组别** | | | | | |
| --- | --- | --- | --- | --- | --- | --- |
| P120 | DeepSeek V3 | | | | | |
| **General model response or patient education material (Chinese) / 模型总体回答或患者教育材料（中文）** | | | | | | |
| 产褥期必须马上去医院的紧急情况包括：1）大出血：每小时湿透一张卫生巾，或排出大于李子大小的血块；2）剧烈头痛、视力模糊、眼前闪光、上腹痛、恶心——警惕子痫前期；3）胸痛、呼吸困难、咯血、单侧腿肿痛——警惕肺栓塞或深静脉血栓；4）癫痫发作或意识丧失；5）体温≥39℃且伴随寒战；6）自伤或伤害宝宝的念头。如何判断症状轻重：恶露臭味+发热+下腹痛→产褥感染，应尽快就医；头痛+血压升高→子痫前期；胸闷+气短+血氧下降→心肺急症。任何在夜间出现的上述症状，都不要等到天亮，直接去急诊。产后42天内，您的身体仍在恢复，警惕性要高。 | | | | | | |
| **English translation of the Chinese general response / 中文总体回答英文翻译** | | | | | | |
| Postpartum emergencies requiring immediate medical attention include: 1) Massive bleeding: soaking a pad per hour or passing clots larger than a plum; 2) Severe headache, blurred vision, flashes, epigastric pain, nausea — suspect preeclampsia; 3) Chest pain, dyspnea, hemoptysis, unilateral leg swelling/pain — suspect PE or DVT; 4) Seizure or loss of consciousness; 5) Fever ≥39°C with chills; 6) Thoughts of harming yourself or the baby. How to assess severity: foul lochia + fever + lower abdominal pain → puerperal infection; headache + high BP → preeclampsia; chest tightness + dyspnea → cardiopulmonary emergency. If any of these occur at night, go directly to the ER — do not wait until morning. You are still recovering in the first 42 days; stay vigilant. | | | | | | |
| **Item / 项目** | | **Q1 / 问题1** | | **Q2 / 问题2** | | **Q3 / 问题3** |
| **Patient question (Chinese original) / 患者原始问题（中文）** | | 产褥期哪些症状属于必须马上去医院的情况？ | | 恶露、发热、头痛和胸闷这些症状怎么判断轻重？ | | 如果夜间突然不舒服，可以直接去急诊吗？ |
| **English translation of patient question / 患者问题英文翻译** | | Which symptoms during the puerperium require immediate hospital care? | | How should I judge the severity of symptoms such as lochia, fever, headache, and chest tightness? | | If I suddenly feel unwell at night, can I go directly to the emergency department? |
| **Model response (Chinese) / 模型回答（中文）** | | 大出血（1小时湿透1片卫生巾）、剧烈头痛、视力改变、胸痛、呼吸困难、癫痫、单侧腿肿痛。 | | 恶露臭味+发热提示感染；头痛+血压高提示子痫前期；胸闷+气短提示心肺问题。 | | 可以，不要等到天亮。 |
| **English translation of model response / 模型回答英文翻译** | | Massive bleeding (soaking pad/hour), severe headache, vision changes, chest pain, dyspnea, seizure, unilateral leg swelling/pain. | | Foul lochia + fever suggests infection; headache + high BP suggests preeclampsia; chest tightness + dyspnea suggests cardiopulmonary issue. | | Yes, go to ER immediately; do not wait until morning. |
| **Overall evaluation item / 整体评价项目** | | | **Score / 分数** | | **Comments / 备注** | |
| **Patient Satisfaction / 患者满意度** | | | 3 | |  | |
| **Patient Understandability / 患者可理解性** | | | 4 | |  | |
| **Patient Usefulness / 患者实用性** | | | 3 | |  | |
| **Clinician Correctness / 医生评分：正确性** | | | 4 | |  | |
| **Clinician Completeness / 医生评分：完整性** | | | 3 | |  | |
| **Clinician Readability / 医生评分：可读性** | | | 4 | |  | |
| **Clinician Helpfulness / 医生评分：帮助性** | | | 3 | |  | |
| **Clinician Safety / 医生评分：安全性** | | | 4 | |  | |
