# Supplementary material for: Beyond accuracy: a three-stage evaluation of large language models for postpartum health consultation and nursing decision support
Source: Front Public Health. 2026 Oct 20;14:1961647. doi: 10.3389/fpubh.2026.1961647 (PMC13727638; doi:10.3389/fpubh.2026.1961647)
Supplement: Supplementary file 4 [file Table_12.docx]

**Supplementary Table S12. Stage 3 de-identified case summaries, model-generated nursing care plans, English translations, and expert ratings**

Stage 3 de-identified standardized case summaries derived from real-world clinical records, model-generated nursing care plans, and expert rating data

120份来源于真实临床记录的去标识化标准化病例摘要；每个病例分别向ChatGPT和DeepSeek独立提交1次；每个模型生成1份产褥期母体护理计划。

For each case, one response was generated by ChatGPT 5.5 and one by DeepSeek V3. The original Chinese-language model outputs were retained verbatim before expert scoring. English translations were prepared using a web-based translation tool and manually corrected only to facilitate international readership; they were not used for expert scoring or statistical analysis.

| **项目 Item** | **填写内容 Content** | **项目 Item** | **填写内容 Content** |
| --- | --- | --- | --- |
| **研究阶段** | Stage 3: de-identified standardized case summaries derived from real-world clinical records | **病例数量** | 120 cases |
| **模型1** | ChatGPT 5.5 | **模型2** | DeepSeek V3 |
| **每病例每模型生成次数** | 1 response per model per case; the first generated response was retained | **评分者 Evaluator** | Three blinded clinical experts; the displayed score for each domain is the median of their independent ratings |
| **测试日期 Testing date** | 1 May to 30 June 2026 | **是否联网/推理模式** | Web browsing/search off; platform-displayed reasoning/deep-thinking mode on |
| **语言 Language** | Chinese; English translations prepared using a web-based translation tool and manually corrected | **备注 Notes** | Original Chinese model outputs were retained verbatim. English translations were provided only for international readership and were not used in scoring. |
| **统一提示词 Standardized prompt** | 以下为一例去标识化后的围产期女性病例摘要。请基于病例信息生成护理计划。护理计划应包括：1. 主要护理问题；2. 护理目标；3. 具体护理措施；4. 观察重点；5. 健康教育；6. 需要分诊、转诊或及时报告医生的情况；7. 随访建议。请保证内容安全、具体、可操作，不要替代医生诊断或治疗。 病例摘要：____ 回答语言：中文。 | | |
| **公开版隐私说明 Public-release privacy note** | 为降低重新识别风险，本公开补充文件删除了不影响护理计划评价的职业、教育程度、精确日期和时间、胎方位、胎盘和脐带详细参数、新生儿性别及身长等间接识别信息。母亲年龄、孕产史、孕周、分娩方式、产程概况、产时出血、会阴或切口情况、并发症和主要风险因素，以及必要的出生体重和Apgar评分予以保留。 To reduce re-identification risk, the public supplementary file omits nonessential indirect identifiers, including occupation, education, exact dates and clock times, fetal position, detailed placental and umbilical-cord parameters, neonatal sex, and neonatal length. Core clinical information required to evaluate the nursing care plans is retained. | | |

**评分维度说明（1-5分Likert量表）** The scores shown for each care plan are the medians of the independent ratings assigned by the three blinded clinical experts.

| **评分维度 Scoring domain** | **评价重点 Evaluation focus** |
| --- | --- |
| **Clinical appropriateness 临床适当性** | 护理计划是否符合产褥期母体健康护理原则和临床常规。 |
| **Individualization 个体化程度** | 是否结合病例中的产后天数、分娩方式、症状、哺乳状态、合并症和风险因素。 |
| **Comprehensiveness 完整性** | 是否覆盖伤口/恶露/乳房与哺乳/排尿排便/疼痛/活动/心理/复诊等关键护理内容。 |
| **Actionability 可操作性** | 护理措施是否具体、清楚、可执行，而非空泛建议。 |
| **Safety 安全性** | 是否避免危险、错误或可能延误病情的建议。 |
| **Triage appropriateness 分诊/转诊建议适当性** | 是否能根据病例风险给出恰当的居家观察、门诊复查、尽快就医或急诊处理建议。 |
| **Structure and clarity 结构与清晰度** | 护理计划是否条理清楚，便于产妇、家属或护理人员理解和使用。 |

**病例 1 / Case 1**

| **项目 Item** | **ChatGPT 5.5** | **DeepSeek V3** |
| --- | --- | --- |
| **去标识化临床病例摘要 De-identified Clinical Case Summary** | 患者33岁，孕产史为G1P1，既往剖宫产0次，孕周39，临产类别为催产素引产。自然破膜，羊水量约50ml，羊水性质为清。以产钳分娩，产时出血约310ml。第一产程9时25分，第二产程1时31分，第三产程0时4分，总产程11时0分，会阴或切口情况为侧切，内缝。合并症及主要风险因素：羊水过少、胎儿宫内窘迫？绒毛膜羊膜炎？。新生儿出生体重2910g，Apgar评分1、5、10分钟分别为10、10、10分，分娩结局为活产。 | |
| **临床病例摘要英文翻译 English Translation of De-identified Clinical Case Summary** | Maternal age was 33 years. The obstetric history was G1P1, with 0 previous cesarean deliveries. Gestational age was 39 weeks, and the labor onset or induction category was oxytocin induction. The membranes ruptured spontaneously; the amniotic fluid volume was approximately 50 mL and its character was clear. The patient had a forceps-assisted vaginal delivery, with estimated intrapartum blood loss of 310 mL. The first, second, and third stages of labor were 9 h 25 min, 1 h 31 min, and 0 h 4 min, respectively; the total labor duration was 11 h 0 min. An episiotomy was repaired with internal sutures. Documented comorbidities and major risk factors were oligohydramnios; suspected fetal distress; suspected chorioamnionitis. Neonatal birth weight was 2910 g; Apgar scores at 1, 5, and 10 minutes were 10, 10, 10; and the delivery outcome was live birth. | |
| **模型生成的产褥期护理计划 Model-generated postpartum nursing care plan** | 产妇33岁，G1P1，孕39周，经产钳助产阴道分娩分娩，产时出血约310ml，新生儿女婴、体重2910g，Apgar评分10、10、10分。病例提示的重点风险为胎膜早破或感染相关风险、胎儿窘迫或羊水异常记录。产后24小时内重点评估体温、脉搏、血压、宫底高度、宫缩、阴道流血、恶露颜色和尿潴留，记录首次排尿和疼痛评分。产后住院及出院后继续观察恶露是否由红色逐渐转淡，乳房是否胀痛，乳头是否皲裂，睡眠和情绪是否稳定。饮食以优质蛋白、含铁食物、蔬菜水果和足量饮水为主，避免盲目大补和长期卧床。本次为产钳助产阴道分娩，会阴伤口、盆底和排尿功能是护理重点。每次大小便后用温水从前向后清洁会阴并轻轻擦干，观察侧切或裂伤处肿胀、硬结、渗血、分泌物和异味。疼痛明显时可侧卧休息，避免久蹲、负重和过早剧烈活动。哺乳方面建议尽早母婴皮肤接触和按需哺乳，指导正确含接，乳胀时先热敷或轻柔按摩再哺乳，乳头疼痛时及时调整姿势。若出现乳房局部红肿硬痛、寒战或高热，应警惕乳腺炎。存在胎膜早破、B族链球菌感染、阴道炎或宫内感染疑虑时，应加强体温、下腹压痛、恶露气味、会阴或切口渗液观察，发热或恶露恶臭时及时复诊。因有胎儿窘迫、羊水污染或新生儿评分变化记录，母婴同室期间应配合观察新生儿吃奶、肤色、体温、呼吸和反应，发现吃奶差、发绀、嗜睡或呼吸异常应立即告知儿科医生。出院前应完成用药、伤口、哺乳和避孕宣教，42天左右复查，必要时提前复诊。若出现阴道流血突然增多、鲜红血浸湿卫生巾、恶露恶臭、发热、持续腹痛、伤口化脓、排尿困难、下肢单侧肿痛、胸闷气短，或持续悲伤焦虑及伤害自己或婴儿的想法，应立即就医或急诊处理。 | 患者33岁，初产妇，产钳助产+侧切，总产程11小时，产时出血310ml，合并羊水过少、可疑胎儿宫内窘迫及绒毛膜羊膜炎。产后需重点监测感染迹象：每日测量体温4次，观察恶露有无恶臭、子宫有无压痛。会阴侧切伤口每日碘伏消毒2次，保持干燥，便后清洗，如出现红、肿、硬结或异常分泌物及时就医。注意子宫复旧，产后1小时内每15分钟按摩宫底，之后每日观察恶露量，若鲜红出血超过月经量或有大血块，警惕晚期产后出血。鼓励产后4小时内排尿，多饮水、高纤维饮食防便秘。尽早开始母乳喂养，按需哺乳。因产程长及可疑胎儿窘迫，关注产妇情绪，必要时心理疏导。产后42天返院复查，新生儿按时接种疫苗。危险信号：发热>38.5℃、寒战、大量阴道出血、严重头痛、视物模糊、呼吸困难，需立即急诊。 |
| **护理计划英文翻译 English translation of care plan** | The patient is 33 years old, G1P1, delivered at 39 weeks by forceps-assisted vaginal delivery, with an estimated intrapartum blood loss of 310 mL. The newborn was a female infant, weighed 2910 g, and had Apgar scores of 10, 10, 10. The main case-based risks include premature rupture of membranes or infection-related risk, fetal distress or abnormal amniotic fluid findings. Within the first 24 hours, assess temperature, pulse, blood pressure, uterine fundal height, uterine tone, vaginal bleeding, lochia color, urinary retention, first voiding, and pain score. During hospitalization and after discharge, continue to observe whether lochia gradually changes from red to lighter color, whether breast engorgement or nipple fissure occurs, and whether sleep and mood remain stable. Diet should include high-quality protein, iron-rich food, vegetables, fruit, and adequate fluids. Avoid blind over-supplementation and prolonged bed rest. Because this was a forceps-assisted vaginal delivery, perineal wound care, pelvic floor recovery, and urinary function are priorities. Rinse the perineum from front to back with warm water after urination or bowel movement and pat dry. Observe the episiotomy or tear for swelling, induration, bleeding, discharge, and odor. Rest in a lateral position if pain is marked, and avoid prolonged squatting, heavy lifting, and early vigorous activity. For breastfeeding, encourage early skin-to-skin contact and feeding on demand. Teach correct latch and positioning. For engorgement, apply warmth or gentle massage before feeding. If nipple pain occurs, adjust latch promptly. Localized breast redness, hard painful swelling, chills, or high fever should raise concern for mastitis. With premature rupture of membranes, group B streptococcus, vaginitis, or suspected intrauterine infection, monitor temperature, lower abdominal tenderness, lochia odor, and drainage from the perineal wound or incision. Return promptly for fever or foul-smelling lochia. Because fetal distress, meconium-stained fluid, or changed Apgar scores were recorded, cooperate with pediatric observation of feeding, skin color, temperature, breathing, and responsiveness. Poor feeding, cyanosis, excessive sleepiness, or abnormal breathing should be reported immediately. Before discharge, provide education on medication, wound care, breastfeeding, and contraception. Arrange a postpartum review around 42 days after delivery, or earlier if symptoms occur. Seek urgent medical care for sudden heavy vaginal bleeding, bright red bleeding soaking pads, foul-smelling lochia, fever, persistent abdominal pain, pus from the wound, difficulty urinating, unilateral leg swelling or pain, chest tightness, shortness of breath, or persistent sadness, anxiety, or thoughts of harming herself or the baby. | The patient is a 33-year-old primipara who underwent forceps-assisted delivery with episiotomy. Total labor duration 11 hours, blood loss 310 mL. Comorbidities include oligohydramnios, suspected fetal distress, and chorioamnionitis. Postpartum priorities: monitor for infection – check temperature 4 times daily, observe lochia for foul odor and uterine tenderness. Clean episiotomy site with iodophor twice daily, keep dry, cleanse after bowel movements. Monitor uterine involution: massage fundus every 15 min for the first hour, then daily observe lochia volume. Seek care if bright red bleeding exceeds menstrual flow or large clots appear. Encourage voiding within 4 hours, increase fluids and fiber to prevent constipation. Initiate breastfeeding early, feed on demand. Provide psychological support due to prolonged labor and suspected distress. Postpartum follow-up at 42 days; ensure newborn vaccines. Red flags: fever >38.5 °C, chills, heavy bleeding (soaking one pad/hour), severe headache, blurred vision, dyspnea – seek emergency care immediately. |
| **Clinical appropriateness 临床适当性** | 5 | 4 |
| **Individualization 个体化程度** | 4 | 5 |
| **Comprehensiveness 完整性** | 5 | 4 |
| **Actionability 可操作性** | 4 | 4 |
| **Safety 安全性** | 5 | 4 |
| **Triage appropriateness 分诊/转诊建议适当性** | 5 | 4 |
| **Structure and clarity 结构与清晰度** | 4 | 4 |

**病例 2 / Case 2**

| **项目 Item** | **ChatGPT 5.5** | **DeepSeek V3** |
| --- | --- | --- |
| **去标识化临床病例摘要 De-identified Clinical Case Summary** | 患者33岁，孕产史为G1P1，既往剖宫产0次，孕周38+1，临产类别为自然临产。自然破膜，羊水量约300ml，羊水性质为清。以自然分娩分娩，产时出血约265ml。第一产程3时5分，第二产程0时42分，第三产程0时3分，总产程3时50分，会阴或切口情况为Ⅰ，内缝。合并症及主要风险因素：无。新生儿出生体重2760g，Apgar评分1、5、10分钟分别为10、10、10分，分娩结局为活产。 | |
| **临床病例摘要英文翻译 English Translation of De-identified Clinical Case Summary** | Maternal age was 33 years. The obstetric history was G1P1, with 0 previous cesarean deliveries. Gestational age was 38+1 weeks, and the labor onset or induction category was spontaneous labor. The membranes ruptured spontaneously; the amniotic fluid volume was approximately 300 mL and its character was clear. The patient had a spontaneous vaginal delivery, with estimated intrapartum blood loss of 265 mL. The first, second, and third stages of labor were 3 h 5 min, 0 h 42 min, and 0 h 3 min, respectively; the total labor duration was 3 h 50 min. A first-degree perineal laceration was repaired with internal sutures. No comorbidities or major risk factors were documented. Neonatal birth weight was 2760 g; Apgar scores at 1, 5, and 10 minutes were 10, 10, 10; and the delivery outcome was live birth. | |
| **模型生成的产褥期护理计划 Model-generated postpartum nursing care plan** | 产妇33岁，G1P1，孕38+1周，经阴道自然分娩分娩，产时出血约265ml，新生儿女婴、体重2760g，Apgar评分10、10、10分。病例提示的重点风险为未记录特殊合并症。产后24小时内重点评估体温、脉搏、血压、宫底高度、宫缩、阴道流血、恶露颜色和尿潴留，记录首次排尿和疼痛评分。产后住院及出院后继续观察恶露是否由红色逐渐转淡，乳房是否胀痛，乳头是否皲裂，睡眠和情绪是否稳定。饮食以优质蛋白、含铁食物、蔬菜水果和足量饮水为主，避免盲目大补和长期卧床。本次为阴道自然分娩，应重点观察会阴裂伤或缝合处恢复。每次大小便后保持局部清洁干燥，观察红肿、渗血、疼痛加重和异常气味。无头晕和出血增多时逐步增加活动，并在医护指导下开始轻柔盆底肌训练。哺乳方面建议尽早母婴皮肤接触和按需哺乳，指导正确含接，乳胀时先热敷或轻柔按摩再哺乳，乳头疼痛时及时调整姿势。若出现乳房局部红肿硬痛、寒战或高热，应警惕乳腺炎。目前未记录特殊合并症，仍需按常规产褥期管理执行，重点是子宫复旧、恶露、伤口、哺乳、营养、活动和心理状态。出院前应完成用药、伤口、哺乳和避孕宣教，42天左右复查，必要时提前复诊。若出现阴道流血突然增多、鲜红血浸湿卫生巾、恶露恶臭、发热、持续腹痛、伤口化脓、排尿困难、下肢单侧肿痛、胸闷气短，或持续悲伤焦虑及伤害自己或婴儿的想法，应立即就医或急诊处理。 | 患者33岁，初产妇，自然分娩，I度会阴裂伤内缝，总产程3小时50分，产时出血265ml，无合并症。产后常规护理：保持会阴清洁干燥，每日温水清洗外阴2次，勤换卫生巾。观察恶露变化：正常恶露由红变淡红再变白，持续4-6周，如出现恶露增多、鲜红色或有臭味，及时就诊。鼓励产后4-6小时内自行排尿，多饮水，可适量饮用红糖水促进恶露排出。预防便秘，推荐火龙果、西梅等通便食物。按需哺乳，每次哺乳后排空剩余乳汁，乳头皲裂可涂抹羊脂膏。产后尽早下床活动，促进血液循环和子宫恢复，避免剧烈运动及重体力劳动至少6周。注意产后情绪变化，家人多陪伴。产后42天复查盆底功能。避孕指导：排卵恢复早于月经复潮，建议使用安全套等非激素避孕措施。 |
| **护理计划英文翻译 English translation of care plan** | The patient is 33 years old, G1P1, delivered at 38+1 weeks by spontaneous vaginal delivery, with an estimated intrapartum blood loss of 265 mL. The newborn was a female infant, weighed 2760 g, and had Apgar scores of 10, 10, 10. The main case-based risks include no special comorbidity was recorded. Within the first 24 hours, assess temperature, pulse, blood pressure, uterine fundal height, uterine tone, vaginal bleeding, lochia color, urinary retention, first voiding, and pain score. During hospitalization and after discharge, continue to observe whether lochia gradually changes from red to lighter color, whether breast engorgement or nipple fissure occurs, and whether sleep and mood remain stable. Diet should include high-quality protein, iron-rich food, vegetables, fruit, and adequate fluids. Avoid blind over-supplementation and prolonged bed rest. Because this was a spontaneous vaginal birth, recovery of any perineal tear or suture site should be observed. Keep the area clean and dry after urination or bowel movement and watch for redness, swelling, bleeding, worsening pain, or abnormal odor. If there is no dizziness or increased bleeding, increase activity gradually and begin gentle pelvic floor exercises under professional guidance. For breastfeeding, encourage early skin-to-skin contact and feeding on demand. Teach correct latch and positioning. For engorgement, apply warmth or gentle massage before feeding. If nipple pain occurs, adjust latch promptly. Localized breast redness, hard painful swelling, chills, or high fever should raise concern for mastitis. No special complication was recorded, but routine postpartum management should still focus on uterine involution, lochia, wounds, breastfeeding, nutrition, activity, and mental health. Before discharge, provide education on medication, wound care, breastfeeding, and contraception. Arrange a postpartum review around 42 days after delivery, or earlier if symptoms occur. Seek urgent medical care for sudden heavy vaginal bleeding, bright red bleeding soaking pads, foul-smelling lochia, fever, persistent abdominal pain, pus from the wound, difficulty urinating, unilateral leg swelling or pain, chest tightness, shortness of breath, or persistent sadness, anxiety, or thoughts of harming herself or the baby. | The patient is a 33-year-old primipara who had spontaneous vaginal delivery with first-degree perineal laceration repaired internally. Total labor 3h50min, blood loss 265 mL, no comorbidities. Routine care: keep perineum clean and dry, wash with warm water twice daily, change pads frequently. Monitor lochia: normal progression red→serous→white over 4-6 weeks. If increased volume, bright red, or foul odor occurs, seek care. Void within 4-6 hours, increase fluids. Prevent constipation with fiber-rich foods. Breastfeed on demand, express remaining milk, apply lanolin for cracked nipples. Ambulate early, avoid strenuous activities for 6 weeks. Monitor mood, family support. Postpartum check at 42 days including pelvic floor assessment. Contraception: ovulation may resume before first menstruation; use condoms or other non-hormonal methods. |
| **Clinical appropriateness 临床适当性** | 4 | 3 |
| **Individualization 个体化程度** | 3 | 5 |
| **Comprehensiveness 完整性** | 4 | 4 |
| **Actionability 可操作性** | 4 | 4 |
| **Safety 安全性** | 5 | 3 |
| **Triage appropriateness 分诊/转诊建议适当性** | 5 | 4 |
| **Structure and clarity 结构与清晰度** | 4 | 4 |

**病例 3 / Case 3**

| **项目 Item** | **ChatGPT 5.5** | **DeepSeek V3** |
| --- | --- | --- |
| **去标识化临床病例摘要 De-identified Clinical Case Summary** | 患者38岁，孕产史为G1P1，既往剖宫产0次，孕周40+5，临产类别为催产素引产。自然破膜，羊水量约300ml，羊水性质为清。以产钳分娩，产时出血约310ml。第一产程7时0分，第二产程0时42分，第三产程0时8分，总产程7时50分，会阴或切口情况为侧切，内缝。合并症及主要风险因素：胎儿宫内窘迫？；胎膜早破；妊娠合并亚临床甲状腺功能减退症；胚胎移植术后；高龄初孕妇的监督；球拍状胎盘。新生儿出生体重2930g，Apgar评分1、5、10分钟分别为10、10、10分，分娩结局为活产。 | |
| **临床病例摘要英文翻译 English Translation of De-identified Clinical Case Summary** | Maternal age was 38 years. The obstetric history was G1P1, with 0 previous cesarean deliveries. Gestational age was 40+5 weeks, and the labor onset or induction category was oxytocin induction. The membranes ruptured spontaneously; the amniotic fluid volume was approximately 300 mL and its character was clear. The patient had a forceps-assisted vaginal delivery, with estimated intrapartum blood loss of 310 mL. The first, second, and third stages of labor were 7 h 0 min, 0 h 42 min, and 0 h 8 min, respectively; the total labor duration was 7 h 50 min. An episiotomy was repaired with internal sutures. Documented comorbidities and major risk factors were suspected fetal distress; premature rupture of membranes; subclinical hypothyroidism during pregnancy; status post embryo transfer; advanced maternal age in a primigravida; battledore placenta. Neonatal birth weight was 2930 g; Apgar scores at 1, 5, and 10 minutes were 10, 10, 10; and the delivery outcome was live birth. | |
| **模型生成的产褥期护理计划 Model-generated postpartum nursing care plan** | 产妇38岁，G1P1，孕40+5周，经产钳助产阴道分娩分娩，产时出血约310ml，新生儿女婴、体重2930g，Apgar评分10、10、10分。病例提示的重点风险为胎膜早破或感染相关风险、甲状腺功能异常、胎盘相关风险、胎儿窘迫或羊水异常记录、高龄妊娠因素。产后24小时内重点评估体温、脉搏、血压、宫底高度、宫缩、阴道流血、恶露颜色和尿潴留，记录首次排尿和疼痛评分。产后住院及出院后继续观察恶露是否由红色逐渐转淡，乳房是否胀痛，乳头是否皲裂，睡眠和情绪是否稳定。饮食以优质蛋白、含铁食物、蔬菜水果和足量饮水为主，避免盲目大补和长期卧床。本次为产钳助产阴道分娩，会阴伤口、盆底和排尿功能是护理重点。每次大小便后用温水从前向后清洁会阴并轻轻擦干，观察侧切或裂伤处肿胀、硬结、渗血、分泌物和异味。疼痛明显时可侧卧休息，避免久蹲、负重和过早剧烈活动。哺乳方面建议尽早母婴皮肤接触和按需哺乳，指导正确含接，乳胀时先热敷或轻柔按摩再哺乳，乳头疼痛时及时调整姿势。若出现乳房局部红肿硬痛、寒战或高热，应警惕乳腺炎。合并甲状腺疾病者，应按内分泌医嘱继续或调整用药，不自行停药，产后复查甲状腺功能，观察心悸、怕冷、乏力、出汗和情绪异常。存在胎膜早破、B族链球菌感染、阴道炎或宫内感染疑虑时，应加强体温、下腹压痛、恶露气味、会阴或切口渗液观察，发热或恶露恶臭时及时复诊。因有胎儿窘迫、羊水污染或新生儿评分变化记录，母婴同室期间应配合观察新生儿吃奶、肤色、体温、呼吸和反应，发现吃奶差、发绀、嗜睡或呼吸异常应立即告知儿科医生。有胎盘位置、形态或胎盘血窦相关记录时，应注意恶露持续时间和出血量，产后复查可结合B超了解宫腔及子宫复旧。出院前应完成用药、伤口、哺乳和避孕宣教，42天左右复查，必要时提前复诊。若出现阴道流血突然增多、鲜红血浸湿卫生巾、恶露恶臭、发热、持续腹痛、伤口化脓、排尿困难、下肢单侧肿痛、胸闷气短，或持续悲伤焦虑及伤害自己或婴儿的想法，应立即就医或急诊处理。 | 患者38岁，高龄初产妇，胚胎移植术后，产钳助产+侧切，合并亚临床甲减、可疑胎儿窘迫、胎膜早破、球拍状胎盘。产后需继续服用左甲状腺素，产后6周复查甲状腺功能，因甲减可影响泌乳和情绪。会阴侧切伤口每日碘伏消毒2次，便后清洗，观察有无红肿、裂开。胎膜早破及产钳助产增加子宫内膜炎风险，每日测体温4次，注意腹痛、恶露恶臭，如有发热立即抗感染。产时出血310ml，需警惕晚期产后出血，按时按摩宫底。高龄初产及胚胎移植史可能带来焦虑，提供心理支持，必要时心理咨询。鼓励母乳喂养，若乳汁不足可咨询泌乳顾问。适当活动，补充富含碘、铁、蛋白质的食物。产后42天同时复查甲功及盆底功能。 |
| **护理计划英文翻译 English translation of care plan** | The patient is 38 years old, G1P1, delivered at 40+5 weeks by forceps-assisted vaginal delivery, with an estimated intrapartum blood loss of 310 mL. The newborn was a female infant, weighed 2930 g, and had Apgar scores of 10, 10, 10. The main case-based risks include premature rupture of membranes or infection-related risk, thyroid dysfunction, placental risk factors, fetal distress or abnormal amniotic fluid findings, advanced maternal age. Within the first 24 hours, assess temperature, pulse, blood pressure, uterine fundal height, uterine tone, vaginal bleeding, lochia color, urinary retention, first voiding, and pain score. During hospitalization and after discharge, continue to observe whether lochia gradually changes from red to lighter color, whether breast engorgement or nipple fissure occurs, and whether sleep and mood remain stable. Diet should include high-quality protein, iron-rich food, vegetables, fruit, and adequate fluids. Avoid blind over-supplementation and prolonged bed rest. Because this was a forceps-assisted vaginal delivery, perineal wound care, pelvic floor recovery, and urinary function are priorities. Rinse the perineum from front to back with warm water after urination or bowel movement and pat dry. Observe the episiotomy or tear for swelling, induration, bleeding, discharge, and odor. Rest in a lateral position if pain is marked, and avoid prolonged squatting, heavy lifting, and early vigorous activity. For breastfeeding, encourage early skin-to-skin contact and feeding on demand. Teach correct latch and positioning. For engorgement, apply warmth or gentle massage before feeding. If nipple pain occurs, adjust latch promptly. Localized breast redness, hard painful swelling, chills, or high fever should raise concern for mastitis. For thyroid disease, continue or adjust medication only under endocrine guidance, recheck thyroid function after delivery, and watch for palpitations, cold intolerance, fatigue, sweating, or abnormal mood changes. With premature rupture of membranes, group B streptococcus, vaginitis, or suspected intrauterine infection, monitor temperature, lower abdominal tenderness, lochia odor, and drainage from the perineal wound or incision. Return promptly for fever or foul-smelling lochia. Because fetal distress, meconium-stained fluid, or changed Apgar scores were recorded, cooperate with pediatric observation of feeding, skin color, temperature, breathing, and responsiveness. Poor feeding, cyanosis, excessive sleepiness, or abnormal breathing should be reported immediately. For abnormal placental location, shape, or placental sinus records, monitor bleeding amount and duration of lochia. Ultrasound review of the uterine cavity and involution may be needed at follow-up. Before discharge, provide education on medication, wound care, breastfeeding, and contraception. Arrange a postpartum review around 42 days after delivery, or earlier if symptoms occur. Seek urgent medical care for sudden heavy vaginal bleeding, bright red bleeding soaking pads, foul-smelling lochia, fever, persistent abdominal pain, pus from the wound, difficulty urinating, unilateral leg swelling or pain, chest tightness, shortness of breath, or persistent sadness, anxiety, or thoughts of harming herself or the baby. | The patient is a 38-year-old advanced maternal age primigravida after embryo transfer. She had forceps delivery with episiotomy. Comorbidities: subclinical hypothyroidism, suspected fetal distress, PPROM, battledore placenta. Continue levothyroxine, retest thyroid function at 6 weeks. Clean episiotomy with iodophor twice daily. Monitor for endometritis: check temperature 4 times daily, watch for abdominal pain and foul lochia. Blood loss 310 mL – watch for late PPH, massage fundus. Provide psychological support due to advanced age and embryo transfer. Encourage breastfeeding; consult lactation specialist if needed. Adequate nutrition with iodine, iron, protein. Follow-up at 42 days: thyroid function and pelvic floor assessment. |
| **Clinical appropriateness 临床适当性** | 5 | 3 |
| **Individualization 个体化程度** | 5 | 5 |
| **Comprehensiveness 完整性** | 5 | 4 |
| **Actionability 可操作性** | 5 | 4 |
| **Safety 安全性** | 5 | 3 |
| **Triage appropriateness 分诊/转诊建议适当性** | 5 | 4 |
| **Structure and clarity 结构与清晰度** | 4 | 3 |

**病例 4 / Case 4**

| **项目 Item** | **ChatGPT 5.5** | **DeepSeek V3** |
| --- | --- | --- |
| **去标识化临床病例摘要 De-identified Clinical Case Summary** | 患者38岁，孕产史为G5P3，既往剖宫产0次，孕周36+4，临产类别为自然临产。自然破膜，羊水量约100ml，羊水性质为清。以自然分娩分娩，产时出血约260ml。第一产程2时45分，第二产程0时13分，第三产程0时7分，总产程3时5分，会阴或切口情况为Ⅰ，内缝。合并症及主要风险因素：胎儿窘迫？；未足月胎膜早破；妊娠合并慢性高血压；高龄经产妇妊娠监督。新生儿出生体重2630g，Apgar评分1、5、10分钟分别为9、9、9分，分娩结局为活产。 | |
| **临床病例摘要英文翻译 English Translation of De-identified Clinical Case Summary** | Maternal age was 38 years. The obstetric history was G5P3, with 0 previous cesarean deliveries. Gestational age was 36+4 weeks, and the labor onset or induction category was spontaneous labor. The membranes ruptured spontaneously; the amniotic fluid volume was approximately 100 mL and its character was clear. The patient had a spontaneous vaginal delivery, with estimated intrapartum blood loss of 260 mL. The first, second, and third stages of labor were 2 h 45 min, 0 h 13 min, and 0 h 7 min, respectively; the total labor duration was 3 h 5 min. A first-degree perineal laceration was repaired with internal sutures. Documented comorbidities and major risk factors were suspected fetal distress; preterm premature rupture of membranes; chronic hypertension in pregnancy; advanced maternal age in a multipara. Neonatal birth weight was 2630 g; Apgar scores at 1, 5, and 10 minutes were 9, 9, 9; and the delivery outcome was live birth. | |
| **模型生成的产褥期护理计划 Model-generated postpartum nursing care plan** | 产妇38岁，G5P3，孕36+4周，经阴道自然分娩分娩，产时出血约260ml，新生儿男婴、体重2630g，Apgar评分9、9、9分。病例提示的重点风险为妊娠期高血压或子痫前期相关风险、胎膜早破或感染相关风险、胎儿窘迫或羊水异常记录、高龄妊娠因素。产后24小时内重点评估体温、脉搏、血压、宫底高度、宫缩、阴道流血、恶露颜色和尿潴留，记录首次排尿和疼痛评分。产后住院及出院后继续观察恶露是否由红色逐渐转淡，乳房是否胀痛，乳头是否皲裂，睡眠和情绪是否稳定。饮食以优质蛋白、含铁食物、蔬菜水果和足量饮水为主，避免盲目大补和长期卧床。本次为阴道自然分娩，应重点观察会阴裂伤或缝合处恢复。每次大小便后保持局部清洁干燥，观察红肿、渗血、疼痛加重和异常气味。无头晕和出血增多时逐步增加活动，并在医护指导下开始轻柔盆底肌训练。哺乳方面建议尽早母婴皮肤接触和按需哺乳，指导正确含接，乳胀时先热敷或轻柔按摩再哺乳，乳头疼痛时及时调整姿势。若出现乳房局部红肿硬痛、寒战或高热，应警惕乳腺炎。有高血压或子痫前期风险者，产后仍可能出现血压波动，应按医嘱监测血压，若持续头痛、眼花、胸闷、上腹痛、抽搐或水肿加重，应立即就医。存在胎膜早破、B族链球菌感染、阴道炎或宫内感染疑虑时，应加强体温、下腹压痛、恶露气味、会阴或切口渗液观察，发热或恶露恶臭时及时复诊。因有胎儿窘迫、羊水污染或新生儿评分变化记录，母婴同室期间应配合观察新生儿吃奶、肤色、体温、呼吸和反应，发现吃奶差、发绀、嗜睡或呼吸异常应立即告知儿科医生。高龄产妇恢复期应避免过度劳累和连续熬夜，家属应参与夜间照护，保证睡眠、营养和情绪支持。出院前应完成用药、伤口、哺乳和避孕宣教，42天左右复查，必要时提前复诊。若出现阴道流血突然增多、鲜红血浸湿卫生巾、恶露恶臭、发热、持续腹痛、伤口化脓、排尿困难、下肢单侧肿痛、胸闷气短，或持续悲伤焦虑及伤害自己或婴儿的想法，应立即就医或急诊处理。 | 患者38岁，G5P3，经产妇，自然分娩+I度裂伤，合并慢性高血压、可疑胎儿窘迫、未足月胎膜早破（孕36+4周），新生儿Apgar 9-9-9，体重2630g（低出生体重）。产后重点：每日监测血压早晚各1次，若≥140/90mmHg或出现头痛、上腹痛、视力改变，立即就诊警惕产后子痫前期。早产儿需保暖，监测血糖、黄疸，鼓励母乳喂养，必要时添加母乳强化剂。未足月胎膜早破增加感染风险，注意发热、宫体压痛、恶露异味。经产妇子宫收缩可能乏力，加强宫底按摩，观察恶露量。I度裂伤保持清洁，温水坐浴每日2次。避免劳累，保证睡眠，因高血压需避免情绪激动。产后2周复查血压，42天全面评估。避孕建议高效方法如宫内节育器。 |
| **护理计划英文翻译 English translation of care plan** | The patient is 38 years old, G5P3, delivered at 36+4 weeks by spontaneous vaginal delivery, with an estimated intrapartum blood loss of 260 mL. The newborn was a male infant, weighed 2630 g, and had Apgar scores of 9, 9, 9. The main case-based risks include gestational hypertension or preeclampsia-related risk, premature rupture of membranes or infection-related risk, fetal distress or abnormal amniotic fluid findings, advanced maternal age. Within the first 24 hours, assess temperature, pulse, blood pressure, uterine fundal height, uterine tone, vaginal bleeding, lochia color, urinary retention, first voiding, and pain score. During hospitalization and after discharge, continue to observe whether lochia gradually changes from red to lighter color, whether breast engorgement or nipple fissure occurs, and whether sleep and mood remain stable. Diet should include high-quality protein, iron-rich food, vegetables, fruit, and adequate fluids. Avoid blind over-supplementation and prolonged bed rest. Because this was a spontaneous vaginal birth, recovery of any perineal tear or suture site should be observed. Keep the area clean and dry after urination or bowel movement and watch for redness, swelling, bleeding, worsening pain, or abnormal odor. If there is no dizziness or increased bleeding, increase activity gradually and begin gentle pelvic floor exercises under professional guidance. For breastfeeding, encourage early skin-to-skin contact and feeding on demand. Teach correct latch and positioning. For engorgement, apply warmth or gentle massage before feeding. If nipple pain occurs, adjust latch promptly. Localized breast redness, hard painful swelling, chills, or high fever should raise concern for mastitis. For hypertension or preeclampsia risk, postpartum blood pressure may still fluctuate. Continue blood pressure monitoring as advised and seek urgent care for persistent headache, visual symptoms, chest tightness, upper abdominal pain, seizure, or worsening edema. With premature rupture of membranes, group B streptococcus, vaginitis, or suspected intrauterine infection, monitor temperature, lower abdominal tenderness, lochia odor, and drainage from the perineal wound or incision. Return promptly for fever or foul-smelling lochia. Because fetal distress, meconium-stained fluid, or changed Apgar scores were recorded, cooperate with pediatric observation of feeding, skin color, temperature, breathing, and responsiveness. Poor feeding, cyanosis, excessive sleepiness, or abnormal breathing should be reported immediately. Advanced maternal age may slow recovery, so the family should help with nighttime care and support adequate sleep, nutrition, and emotional stability. Before discharge, provide education on medication, wound care, breastfeeding, and contraception. Arrange a postpartum review around 42 days after delivery, or earlier if symptoms occur. Seek urgent medical care for sudden heavy vaginal bleeding, bright red bleeding soaking pads, foul-smelling lochia, fever, persistent abdominal pain, pus from the wound, difficulty urinating, unilateral leg swelling or pain, chest tightness, shortness of breath, or persistent sadness, anxiety, or thoughts of harming herself or the baby. | The patient is a 38-year-old G5P3 multipara who had spontaneous delivery with first-degree laceration. Comorbidities: chronic hypertension, suspected fetal distress, PPROM at 36+4 weeks. Newborn weight 2630 g (LBW), Apgar 9-9-9. Monitor BP twice daily; seek care if BP≥140/90 or headache, epigastric pain, visual changes – rule out postpartum preeclampsia. Preterm infant: maintain warmth, monitor glucose and jaundice, encourage breastfeeding with possible fortifier. Infection risk from PPROM – monitor temperature, uterine tenderness, lochia. Multipara may have poor uterine tone – aggressive fundal massage. Perineal care: warm sitz baths. Avoid stress and fatigue. Check BP at 2 weeks; comprehensive exam at 42 days. Contraception: recommend IUD. |
| **Clinical appropriateness 临床适当性** | 5 | 3 |
| **Individualization 个体化程度** | 5 | 5 |
| **Comprehensiveness 完整性** | 5 | 4 |
| **Actionability 可操作性** | 5 | 4 |
| **Safety 安全性** | 5 | 3 |
| **Triage appropriateness 分诊/转诊建议适当性** | 5 | 4 |
| **Structure and clarity 结构与清晰度** | 4 | 3 |

**病例 5 / Case 5**

| **项目 Item** | **ChatGPT 5.5** | **DeepSeek V3** |
| --- | --- | --- |
| **去标识化临床病例摘要 De-identified Clinical Case Summary** | 患者29岁，孕产史为G1P1，既往剖宫产0次，孕周38+5，临产类别为催产素引产。自然破膜，羊水量约100ml，羊水性质为清。以产钳分娩，产时出血约270ml。第一产程4时9分，第二产程0时23分，第三产程0时8分，总产程4时40分，会阴或切口情况为侧切，内缝。合并症及主要风险因素：胎膜早破、胎儿窘迫？。新生儿出生体重3060g，Apgar评分1、5、10分钟分别为6、10、10分，分娩结局为活产。 | |
| **临床病例摘要英文翻译 English Translation of De-identified Clinical Case Summary** | Maternal age was 29 years. The obstetric history was G1P1, with 0 previous cesarean deliveries. Gestational age was 38+5 weeks, and the labor onset or induction category was oxytocin induction. The membranes ruptured spontaneously; the amniotic fluid volume was approximately 100 mL and its character was clear. The patient had a forceps-assisted vaginal delivery, with estimated intrapartum blood loss of 270 mL. The first, second, and third stages of labor were 4 h 9 min, 0 h 23 min, and 0 h 8 min, respectively; the total labor duration was 4 h 40 min. An episiotomy was repaired with internal sutures. Documented comorbidities and major risk factors were premature rupture of membranes; suspected fetal distress. Neonatal birth weight was 3060 g; Apgar scores at 1, 5, and 10 minutes were 6, 10, 10; and the delivery outcome was live birth. | |
| **模型生成的产褥期护理计划 Model-generated postpartum nursing care plan** | 产妇29岁，G1P1，孕38+5周，经产钳助产阴道分娩分娩，产时出血约270ml，新生儿女婴、体重3060g，Apgar评分6、10、10分。病例提示的重点风险为胎膜早破或感染相关风险、胎儿窘迫或羊水异常记录。产后24小时内重点评估体温、脉搏、血压、宫底高度、宫缩、阴道流血、恶露颜色和尿潴留，记录首次排尿和疼痛评分。产后住院及出院后继续观察恶露是否由红色逐渐转淡，乳房是否胀痛，乳头是否皲裂，睡眠和情绪是否稳定。饮食以优质蛋白、含铁食物、蔬菜水果和足量饮水为主，避免盲目大补和长期卧床。本次为产钳助产阴道分娩，会阴伤口、盆底和排尿功能是护理重点。每次大小便后用温水从前向后清洁会阴并轻轻擦干，观察侧切或裂伤处肿胀、硬结、渗血、分泌物和异味。疼痛明显时可侧卧休息，避免久蹲、负重和过早剧烈活动。哺乳方面建议尽早母婴皮肤接触和按需哺乳，指导正确含接，乳胀时先热敷或轻柔按摩再哺乳，乳头疼痛时及时调整姿势。若出现乳房局部红肿硬痛、寒战或高热，应警惕乳腺炎。存在胎膜早破、B族链球菌感染、阴道炎或宫内感染疑虑时，应加强体温、下腹压痛、恶露气味、会阴或切口渗液观察，发热或恶露恶臭时及时复诊。因有胎儿窘迫、羊水污染或新生儿评分变化记录，母婴同室期间应配合观察新生儿吃奶、肤色、体温、呼吸和反应，发现吃奶差、发绀、嗜睡或呼吸异常应立即告知儿科医生。出院前应完成用药、伤口、哺乳和避孕宣教，42天左右复查，必要时提前复诊。若出现阴道流血突然增多、鲜红血浸湿卫生巾、恶露恶臭、发热、持续腹痛、伤口化脓、排尿困难、下肢单侧肿痛、胸闷气短，或持续悲伤焦虑及伤害自己或婴儿的想法，应立即就医或急诊处理。 | 患者29岁，初产妇，产钳助产+侧切，合并胎膜早破、可疑胎儿窘迫，新生儿1分钟Apgar 6分（复苏后正常）。产后需重点关注新生儿：虽然后续Apgar正常，仍需监测呼吸、肌张力、吸吮能力，注意缺氧缺血性脑病征象，建议儿科观察24小时。会阴侧切伤口每日消毒2次，坐位时可使用中空坐垫减轻压力。产时出血270ml，观察晚期出血。胎膜早破+产钳增加感染风险，每日测体温4次，发热>38℃伴寒战需就医。新生儿早期窒息可能影响初次吸吮，耐心指导母乳喂养，必要时挤母乳杯喂。新生儿低Apgar评分易致父母焦虑，提供积极支持和解释。避免久站，增加优质蛋白和铁剂摄入。新生儿7-14天内复查神经系统评估；产妇42天复查。 |
| **护理计划英文翻译 English translation of care plan** | The patient is 29 years old, G1P1, delivered at 38+5 weeks by forceps-assisted vaginal delivery, with an estimated intrapartum blood loss of 270 mL. The newborn was a female infant, weighed 3060 g, and had Apgar scores of 6, 10, 10. The main case-based risks include premature rupture of membranes or infection-related risk, fetal distress or abnormal amniotic fluid findings. Within the first 24 hours, assess temperature, pulse, blood pressure, uterine fundal height, uterine tone, vaginal bleeding, lochia color, urinary retention, first voiding, and pain score. During hospitalization and after discharge, continue to observe whether lochia gradually changes from red to lighter color, whether breast engorgement or nipple fissure occurs, and whether sleep and mood remain stable. Diet should include high-quality protein, iron-rich food, vegetables, fruit, and adequate fluids. Avoid blind over-supplementation and prolonged bed rest. Because this was a forceps-assisted vaginal delivery, perineal wound care, pelvic floor recovery, and urinary function are priorities. Rinse the perineum from front to back with warm water after urination or bowel movement and pat dry. Observe the episiotomy or tear for swelling, induration, bleeding, discharge, and odor. Rest in a lateral position if pain is marked, and avoid prolonged squatting, heavy lifting, and early vigorous activity. For breastfeeding, encourage early skin-to-skin contact and feeding on demand. Teach correct latch and positioning. For engorgement, apply warmth or gentle massage before feeding. If nipple pain occurs, adjust latch promptly. Localized breast redness, hard painful swelling, chills, or high fever should raise concern for mastitis. With premature rupture of membranes, group B streptococcus, vaginitis, or suspected intrauterine infection, monitor temperature, lower abdominal tenderness, lochia odor, and drainage from the perineal wound or incision. Return promptly for fever or foul-smelling lochia. Because fetal distress, meconium-stained fluid, or changed Apgar scores were recorded, cooperate with pediatric observation of feeding, skin color, temperature, breathing, and responsiveness. Poor feeding, cyanosis, excessive sleepiness, or abnormal breathing should be reported immediately. Before discharge, provide education on medication, wound care, breastfeeding, and contraception. Arrange a postpartum review around 42 days after delivery, or earlier if symptoms occur. Seek urgent medical care for sudden heavy vaginal bleeding, bright red bleeding soaking pads, foul-smelling lochia, fever, persistent abdominal pain, pus from the wound, difficulty urinating, unilateral leg swelling or pain, chest tightness, shortness of breath, or persistent sadness, anxiety, or thoughts of harming herself or the baby. | The patient is a 29-year-old primipara with forceps delivery and episiotomy. Comorbidities: PPROM, suspected fetal distress. Newborn 1-min Apgar 6 (resuscitated to normal). Observe newborn for HIE signs (tone, feeding, respiration) for 24 hours. Clean episiotomy twice daily; use donut pillow. Blood loss 270 mL – watch for late PPH. Infection risk – check temperature 4 times daily; seek care if fever >38 °C with chills. Neonatal depression may affect latch – provide breastfeeding guidance, consider cup feeding. Provide psychological support for parental anxiety. Increase protein and iron intake. Newborn neurological assessment at 7-14 days; maternal check at 42 days. |
| **Clinical appropriateness 临床适当性** | 5 | 4 |
| **Individualization 个体化程度** | 4 | 5 |
| **Comprehensiveness 完整性** | 5 | 4 |
| **Actionability 可操作性** | 4 | 4 |
| **Safety 安全性** | 5 | 4 |
| **Triage appropriateness 分诊/转诊建议适当性** | 5 | 4 |
| **Structure and clarity 结构与清晰度** | 4 | 4 |

**病例 6 / Case 6**

| **项目 Item** | **ChatGPT 5.5** | **DeepSeek V3** |
| --- | --- | --- |
| **去标识化临床病例摘要 De-identified Clinical Case Summary** | 患者28岁，孕产史为G3P1，既往剖宫产0次，孕周40，临产类别为自然临产。人工破膜，羊水量约300ml，羊水性质为清。以自然分娩分娩，产时出血约300ml。第一产程11时0分，第二产程0时45分，第三产程0时25分，总产程12时10分，会阴或切口情况为Ⅰ，内缝。合并症及主要风险因素：无。新生儿出生体重2970g，Apgar评分1、5、10分钟分别为10、10、10分，分娩结局为活产。 | |
| **临床病例摘要英文翻译 English Translation of De-identified Clinical Case Summary** | Maternal age was 28 years. The obstetric history was G3P1, with 0 previous cesarean deliveries. Gestational age was 40 weeks, and the labor onset or induction category was spontaneous labor. The membranes were artificially ruptured; the amniotic fluid volume was approximately 300 mL and its character was clear. The patient had a spontaneous vaginal delivery, with estimated intrapartum blood loss of 300 mL. The first, second, and third stages of labor were 11 h 0 min, 0 h 45 min, and 0 h 25 min, respectively; the total labor duration was 12 h 10 min. A first-degree perineal laceration was repaired with internal sutures. No comorbidities or major risk factors were documented. Neonatal birth weight was 2970 g; Apgar scores at 1, 5, and 10 minutes were 10, 10, 10; and the delivery outcome was live birth. | |
| **模型生成的产褥期护理计划 Model-generated postpartum nursing care plan** | 产妇28岁，G3P1，孕40周，经阴道自然分娩分娩，产时出血约300ml，新生儿女婴、体重2970g，Apgar评分10、10、10分。病例提示的重点风险为未记录特殊合并症。产后24小时内重点评估体温、脉搏、血压、宫底高度、宫缩、阴道流血、恶露颜色和尿潴留，记录首次排尿和疼痛评分。产后住院及出院后继续观察恶露是否由红色逐渐转淡，乳房是否胀痛，乳头是否皲裂，睡眠和情绪是否稳定。饮食以优质蛋白、含铁食物、蔬菜水果和足量饮水为主，避免盲目大补和长期卧床。本次为阴道自然分娩，应重点观察会阴裂伤或缝合处恢复。每次大小便后保持局部清洁干燥，观察红肿、渗血、疼痛加重和异常气味。无头晕和出血增多时逐步增加活动，并在医护指导下开始轻柔盆底肌训练。哺乳方面建议尽早母婴皮肤接触和按需哺乳，指导正确含接，乳胀时先热敷或轻柔按摩再哺乳，乳头疼痛时及时调整姿势。若出现乳房局部红肿硬痛、寒战或高热，应警惕乳腺炎。目前未记录特殊合并症，仍需按常规产褥期管理执行，重点是子宫复旧、恶露、伤口、哺乳、营养、活动和心理状态。出院前应完成用药、伤口、哺乳和避孕宣教，42天左右复查，必要时提前复诊。若出现阴道流血突然增多、鲜红血浸湿卫生巾、恶露恶臭、发热、持续腹痛、伤口化脓、排尿困难、下肢单侧肿痛、胸闷气短，或持续悲伤焦虑及伤害自己或婴儿的想法，应立即就医或急诊处理。 | 患者28岁，G3P1，自然分娩+I度裂伤，总产程12小时10分，产时出血300ml，合并脐带绕颈2周、脐带隐形脱垂。产后需观察新生儿有无缺氧表现（呼吸、肌张力、喂养），尽管Apgar正常，仍建议留观24小时。产妇方面：第三产程25分钟，出血300ml，需警惕产后出血，产后2小时内每15分钟按摩宫底。I度裂伤保持清洁，温水坐浴每日2次。产程长达12小时，膀胱充盈易致尿潴留，鼓励产后4小时内排尿，必要时导尿。按需哺乳，注意乳胀。适当下床活动，促进恶露排出。脐带异常可能引起产妇担忧，解释新生儿已健康出生。产后42天复查盆底功能。 |
| **护理计划英文翻译 English translation of care plan** | The patient is 28 years old, G3P1, delivered at 40 weeks by spontaneous vaginal delivery, with an estimated intrapartum blood loss of 300 mL. The newborn was a female infant, weighed 2970 g, and had Apgar scores of 10, 10, 10. The main case-based risks include no special comorbidity was recorded. Within the first 24 hours, assess temperature, pulse, blood pressure, uterine fundal height, uterine tone, vaginal bleeding, lochia color, urinary retention, first voiding, and pain score. During hospitalization and after discharge, continue to observe whether lochia gradually changes from red to lighter color, whether breast engorgement or nipple fissure occurs, and whether sleep and mood remain stable. Diet should include high-quality protein, iron-rich food, vegetables, fruit, and adequate fluids. Avoid blind over-supplementation and prolonged bed rest. Because this was a spontaneous vaginal birth, recovery of any perineal tear or suture site should be observed. Keep the area clean and dry after urination or bowel movement and watch for redness, swelling, bleeding, worsening pain, or abnormal odor. If there is no dizziness or increased bleeding, increase activity gradually and begin gentle pelvic floor exercises under professional guidance. For breastfeeding, encourage early skin-to-skin contact and feeding on demand. Teach correct latch and positioning. For engorgement, apply warmth or gentle massage before feeding. If nipple pain occurs, adjust latch promptly. Localized breast redness, hard painful swelling, chills, or high fever should raise concern for mastitis. No special complication was recorded, but routine postpartum management should still focus on uterine involution, lochia, wounds, breastfeeding, nutrition, activity, and mental health. Before discharge, provide education on medication, wound care, breastfeeding, and contraception. Arrange a postpartum review around 42 days after delivery, or earlier if symptoms occur. Seek urgent medical care for sudden heavy vaginal bleeding, bright red bleeding soaking pads, foul-smelling lochia, fever, persistent abdominal pain, pus from the wound, difficulty urinating, unilateral leg swelling or pain, chest tightness, shortness of breath, or persistent sadness, anxiety, or thoughts of harming herself or the baby. | The patient is a 28-year-old G3P1 with spontaneous delivery and first-degree laceration. Total labor 12h10min, blood loss 300 mL. Comorbidities: nuchal cord 2 loops, occult cord prolapse. Observe newborn for hypoxia (respiration, tone, feeding) for 24 hours. Massage fundus every 15 min for 2 hours postpartum. Perineal care: warm sitz baths. Prolonged labor increases risk of urinary retention – encourage voiding within 4 hours, catheterize if needed. Breastfeed on demand. Early ambulation. Provide reassurance about cord abnormality. Follow-up at 42 days including pelvic floor assessment. |
| **Clinical appropriateness 临床适当性** | 4 | 4 |
| **Individualization 个体化程度** | 3 | 5 |
| **Comprehensiveness 完整性** | 4 | 4 |
| **Actionability 可操作性** | 4 | 4 |
| **Safety 安全性** | 5 | 4 |
| **Triage appropriateness 分诊/转诊建议适当性** | 5 | 3 |
| **Structure and clarity 结构与清晰度** | 4 | 4 |

**病例 7 / Case 7**

| **项目 Item** | **ChatGPT 5.5** | **DeepSeek V3** |
| --- | --- | --- |
| **去标识化临床病例摘要 De-identified Clinical Case Summary** | 患者39岁，孕产史为G1P1，既往剖宫产0次，孕周39+5，临产类别为自然临产。自然破膜，羊水量约300ml，羊水性质为清。以产钳分娩，产时出血约370ml。第一产程3时45分，第二产程2时4分，第三产程0时6分，总产程5时55分，会阴或切口情况为侧切，内缝。合并症及主要风险因素：妊娠期高血压？高龄初孕妇的监督。新生儿出生体重3050g，Apgar评分1、5、10分钟分别为10、10、10分，分娩结局为活产。 | |
| **临床病例摘要英文翻译 English Translation of De-identified Clinical Case Summary** | Maternal age was 39 years. The obstetric history was G1P1, with 0 previous cesarean deliveries. Gestational age was 39+5 weeks, and the labor onset or induction category was spontaneous labor. The membranes ruptured spontaneously; the amniotic fluid volume was approximately 300 mL and its character was clear. The patient had a forceps-assisted vaginal delivery, with estimated intrapartum blood loss of 370 mL. The first, second, and third stages of labor were 3 h 45 min, 2 h 4 min, and 0 h 6 min, respectively; the total labor duration was 5 h 55 min. An episiotomy was repaired with internal sutures. Documented comorbidities and major risk factors were suspected gestational hypertension; advanced maternal age in a primigravida. Neonatal birth weight was 3050 g; Apgar scores at 1, 5, and 10 minutes were 10, 10, 10; and the delivery outcome was live birth. | |
| **模型生成的产褥期护理计划 Model-generated postpartum nursing care plan** | 产妇39岁，G1P1，孕39+5周，经产钳助产阴道分娩分娩，产时出血约370ml，新生儿男婴、体重3050g，Apgar评分10、10、10分。病例提示的重点风险为妊娠期高血压或子痫前期相关风险、高龄妊娠因素。产后24小时内重点评估体温、脉搏、血压、宫底高度、宫缩、阴道流血、恶露颜色和尿潴留，记录首次排尿和疼痛评分。产后住院及出院后继续观察恶露是否由红色逐渐转淡，乳房是否胀痛，乳头是否皲裂，睡眠和情绪是否稳定。饮食以优质蛋白、含铁食物、蔬菜水果和足量饮水为主，避免盲目大补和长期卧床。本次为产钳助产阴道分娩，会阴伤口、盆底和排尿功能是护理重点。每次大小便后用温水从前向后清洁会阴并轻轻擦干，观察侧切或裂伤处肿胀、硬结、渗血、分泌物和异味。疼痛明显时可侧卧休息，避免久蹲、负重和过早剧烈活动。哺乳方面建议尽早母婴皮肤接触和按需哺乳，指导正确含接，乳胀时先热敷或轻柔按摩再哺乳，乳头疼痛时及时调整姿势。若出现乳房局部红肿硬痛、寒战或高热，应警惕乳腺炎。有高血压或子痫前期风险者，产后仍可能出现血压波动，应按医嘱监测血压，若持续头痛、眼花、胸闷、上腹痛、抽搐或水肿加重，应立即就医。高龄产妇恢复期应避免过度劳累和连续熬夜，家属应参与夜间照护，保证睡眠、营养和情绪支持。出院前应完成用药、伤口、哺乳和避孕宣教，42天左右复查，必要时提前复诊。若出现阴道流血突然增多、鲜红血浸湿卫生巾、恶露恶臭、发热、持续腹痛、伤口化脓、排尿困难、下肢单侧肿痛、胸闷气短，或持续悲伤焦虑及伤害自己或婴儿的想法，应立即就医或急诊处理。 | 患者39岁，高龄初产妇，产钳助产+侧切，产时出血370ml，合并妊娠期高血压（可疑）、脐带绕颈1周、脐带隐性脱垂。产后每日监测血压4次，若血压≥140/90mmHg或出现头痛、上腹不适、视力模糊，立即就医预防子痫前期/子痫。出血370ml接近产后出血阈值，需警惕晚期出血，观察恶露量、颜色，按摩宫底。侧切伤口大，每日碘伏消毒2次，注意有无血肿、感染。隐性脱垂后遗症：观察新生儿有无缺氧。高龄初产可能泌乳延迟，鼓励早接触、勤吸吮。提供心理疏导。低盐饮食，补充铁剂。产后2周复查血压，42天全面评估。 |
| **护理计划英文翻译 English translation of care plan** | The patient is 39 years old, G1P1, delivered at 39+5 weeks by forceps-assisted vaginal delivery, with an estimated intrapartum blood loss of 370 mL. The newborn was a male infant, weighed 3050 g, and had Apgar scores of 10, 10, 10. The main case-based risks include gestational hypertension or preeclampsia-related risk, advanced maternal age. Within the first 24 hours, assess temperature, pulse, blood pressure, uterine fundal height, uterine tone, vaginal bleeding, lochia color, urinary retention, first voiding, and pain score. During hospitalization and after discharge, continue to observe whether lochia gradually changes from red to lighter color, whether breast engorgement or nipple fissure occurs, and whether sleep and mood remain stable. Diet should include high-quality protein, iron-rich food, vegetables, fruit, and adequate fluids. Avoid blind over-supplementation and prolonged bed rest. Because this was a forceps-assisted vaginal delivery, perineal wound care, pelvic floor recovery, and urinary function are priorities. Rinse the perineum from front to back with warm water after urination or bowel movement and pat dry. Observe the episiotomy or tear for swelling, induration, bleeding, discharge, and odor. Rest in a lateral position if pain is marked, and avoid prolonged squatting, heavy lifting, and early vigorous activity. For breastfeeding, encourage early skin-to-skin contact and feeding on demand. Teach correct latch and positioning. For engorgement, apply warmth or gentle massage before feeding. If nipple pain occurs, adjust latch promptly. Localized breast redness, hard painful swelling, chills, or high fever should raise concern for mastitis. For hypertension or preeclampsia risk, postpartum blood pressure may still fluctuate. Continue blood pressure monitoring as advised and seek urgent care for persistent headache, visual symptoms, chest tightness, upper abdominal pain, seizure, or worsening edema. Advanced maternal age may slow recovery, so the family should help with nighttime care and support adequate sleep, nutrition, and emotional stability. Before discharge, provide education on medication, wound care, breastfeeding, and contraception. Arrange a postpartum review around 42 days after delivery, or earlier if symptoms occur. Seek urgent medical care for sudden heavy vaginal bleeding, bright red bleeding soaking pads, foul-smelling lochia, fever, persistent abdominal pain, pus from the wound, difficulty urinating, unilateral leg swelling or pain, chest tightness, shortness of breath, or persistent sadness, anxiety, or thoughts of harming herself or the baby. | The patient is a 39-year-old advanced maternal age primigravida with forceps delivery and episiotomy. Blood loss 370 mL. Comorbidities: suspected gestational hypertension, nuchal cord 1 loop, occult cord prolapse. Monitor BP 4 times daily; seek care if BP≥140/90 or headache, epigastric pain, visual changes – rule out preeclampsia/eclampsia. Watch for late PPH, monitor lochia. Clean episiotomy twice daily; monitor for hematoma/infection. Observe newborn for hypoxia. Advanced age may delay lactation – encourage early skin-to-skin and frequent nursing. Low-salt diet, iron supplementation. Check BP at 2 weeks; comprehensive exam at 42 days. |
| **Clinical appropriateness 临床适当性** | 5 | 4 |
| **Individualization 个体化程度** | 4 | 5 |
| **Comprehensiveness 完整性** | 5 | 4 |
| **Actionability 可操作性** | 4 | 4 |
| **Safety 安全性** | 5 | 4 |
| **Triage appropriateness 分诊/转诊建议适当性** | 5 | 4 |
| **Structure and clarity 结构与清晰度** | 4 | 4 |

**病例 8 / Case 8**

| **项目 Item** | **ChatGPT 5.5** | **DeepSeek V3** |
| --- | --- | --- |
| **去标识化临床病例摘要 De-identified Clinical Case Summary** | 患者34岁，孕产史为G4P2，既往剖宫产0次，孕周38+4，临产类别为自然临产。自然破膜，羊水量约400ml，羊水性质为清。以自然分娩分娩，产时出血约260ml。第一产程2时51分，第二产程0时11分，第三产程0时8分，总产程3时10分，会阴或切口情况为Ⅰ，内缝。合并症及主要风险因素：胎膜早破；妊娠合并甲状腺功能减退；双眼屈光不正。新生儿出生体重3130g，Apgar评分1、5、10分钟分别为10、10、10分，分娩结局为活产。 | |
| **临床病例摘要英文翻译 English Translation of De-identified Clinical Case Summary** | Maternal age was 34 years. The obstetric history was G4P2, with 0 previous cesarean deliveries. Gestational age was 38+4 weeks, and the labor onset or induction category was spontaneous labor. The membranes ruptured spontaneously; the amniotic fluid volume was approximately 400 mL and its character was clear. The patient had a spontaneous vaginal delivery, with estimated intrapartum blood loss of 260 mL. The first, second, and third stages of labor were 2 h 51 min, 0 h 11 min, and 0 h 8 min, respectively; the total labor duration was 3 h 10 min. A first-degree perineal laceration was repaired with internal sutures. Documented comorbidities and major risk factors were premature rupture of membranes; hypothyroidism during pregnancy; bilateral refractive error. Neonatal birth weight was 3130 g; Apgar scores at 1, 5, and 10 minutes were 10, 10, 10; and the delivery outcome was live birth. | |
| **模型生成的产褥期护理计划 Model-generated postpartum nursing care plan** | 产妇34岁，G4P2，孕38+4周，经阴道自然分娩分娩，产时出血约260ml，新生儿女婴、体重3130g，Apgar评分10、10、10分。病例提示的重点风险为胎膜早破或感染相关风险、甲状腺功能异常。产后24小时内重点评估体温、脉搏、血压、宫底高度、宫缩、阴道流血、恶露颜色和尿潴留，记录首次排尿和疼痛评分。产后住院及出院后继续观察恶露是否由红色逐渐转淡，乳房是否胀痛，乳头是否皲裂，睡眠和情绪是否稳定。饮食以优质蛋白、含铁食物、蔬菜水果和足量饮水为主，避免盲目大补和长期卧床。本次为阴道自然分娩，应重点观察会阴裂伤或缝合处恢复。每次大小便后保持局部清洁干燥，观察红肿、渗血、疼痛加重和异常气味。无头晕和出血增多时逐步增加活动，并在医护指导下开始轻柔盆底肌训练。哺乳方面建议尽早母婴皮肤接触和按需哺乳，指导正确含接，乳胀时先热敷或轻柔按摩再哺乳，乳头疼痛时及时调整姿势。若出现乳房局部红肿硬痛、寒战或高热，应警惕乳腺炎。合并甲状腺疾病者，应按内分泌医嘱继续或调整用药，不自行停药，产后复查甲状腺功能，观察心悸、怕冷、乏力、出汗和情绪异常。存在胎膜早破、B族链球菌感染、阴道炎或宫内感染疑虑时，应加强体温、下腹压痛、恶露气味、会阴或切口渗液观察，发热或恶露恶臭时及时复诊。出院前应完成用药、伤口、哺乳和避孕宣教，42天左右复查，必要时提前复诊。若出现阴道流血突然增多、鲜红血浸湿卫生巾、恶露恶臭、发热、持续腹痛、伤口化脓、排尿困难、下肢单侧肿痛、胸闷气短，或持续悲伤焦虑及伤害自己或婴儿的想法，应立即就医或急诊处理。 | 患者34岁，G4P2，自然分娩+I度裂伤，合并胎膜早破、妊娠合并甲减、双眼屈光不正（高度近视）。产后继续服用左甲状腺素，6周复查甲功。胎膜早破增加子宫内膜炎风险，每日测体温4次，注意恶露气味、腹痛。I度裂伤温水坐浴。高度近视者产后避免用力屏气（如便秘时），以防视网膜脱离，建议使用开塞露或乳果糖通便。甲减可能影响泌乳和情绪，鼓励勤吸吮，家人留意情绪变化。适当活动，保证睡眠。产后42天复查甲功及眼底检查。 |
| **护理计划英文翻译 English translation of care plan** | The patient is 34 years old, G4P2, delivered at 38+4 weeks by spontaneous vaginal delivery, with an estimated intrapartum blood loss of 260 mL. The newborn was a female infant, weighed 3130 g, and had Apgar scores of 10, 10, 10. The main case-based risks include premature rupture of membranes or infection-related risk, thyroid dysfunction. Within the first 24 hours, assess temperature, pulse, blood pressure, uterine fundal height, uterine tone, vaginal bleeding, lochia color, urinary retention, first voiding, and pain score. During hospitalization and after discharge, continue to observe whether lochia gradually changes from red to lighter color, whether breast engorgement or nipple fissure occurs, and whether sleep and mood remain stable. Diet should include high-quality protein, iron-rich food, vegetables, fruit, and adequate fluids. Avoid blind over-supplementation and prolonged bed rest. Because this was a spontaneous vaginal birth, recovery of any perineal tear or suture site should be observed. Keep the area clean and dry after urination or bowel movement and watch for redness, swelling, bleeding, worsening pain, or abnormal odor. If there is no dizziness or increased bleeding, increase activity gradually and begin gentle pelvic floor exercises under professional guidance. For breastfeeding, encourage early skin-to-skin contact and feeding on demand. Teach correct latch and positioning. For engorgement, apply warmth or gentle massage before feeding. If nipple pain occurs, adjust latch promptly. Localized breast redness, hard painful swelling, chills, or high fever should raise concern for mastitis. For thyroid disease, continue or adjust medication only under endocrine guidance, recheck thyroid function after delivery, and watch for palpitations, cold intolerance, fatigue, sweating, or abnormal mood changes. With premature rupture of membranes, group B streptococcus, vaginitis, or suspected intrauterine infection, monitor temperature, lower abdominal tenderness, lochia odor, and drainage from the perineal wound or incision. Return promptly for fever or foul-smelling lochia. Before discharge, provide education on medication, wound care, breastfeeding, and contraception. Arrange a postpartum review around 42 days after delivery, or earlier if symptoms occur. Seek urgent medical care for sudden heavy vaginal bleeding, bright red bleeding soaking pads, foul-smelling lochia, fever, persistent abdominal pain, pus from the wound, difficulty urinating, unilateral leg swelling or pain, chest tightness, shortness of breath, or persistent sadness, anxiety, or thoughts of harming herself or the baby. | The patient is a 34-year-old G4P2 with spontaneous delivery and first-degree laceration. Comorbidities: PPROM, hypothyroidism, high myopia. Continue levothyroxine, retest thyroid at 6 weeks. Infection risk – check temperature 4 times daily, monitor lochia odor and abdominal pain. Warm sitz baths. High myopia – avoid Valsalva maneuver (constipation) to prevent retinal detachment; use glycerin suppositories or lactulose. Hypothyroidism may affect lactation and mood – encourage frequent nursing, monitor mood. Follow-up at 42 days: thyroid function and eye exam. |
| **Clinical appropriateness 临床适当性** | 5 | 3 |
| **Individualization 个体化程度** | 4 | 4 |
| **Comprehensiveness 完整性** | 5 | 4 |
| **Actionability 可操作性** | 4 | 4 |
| **Safety 安全性** | 5 | 3 |
| **Triage appropriateness 分诊/转诊建议适当性** | 5 | 3 |
| **Structure and clarity 结构与清晰度** | 4 | 4 |

**病例 9 / Case 9**

| **项目 Item** | **ChatGPT 5.5** | **DeepSeek V3** |
| --- | --- | --- |
| **去标识化临床病例摘要 De-identified Clinical Case Summary** | 患者37岁，孕产史为G2P2，既往剖宫产0次，孕周39+3，临产类别为催产素引产。人工破膜，羊水量约50ml，羊水性质为清。以产钳分娩，产时出血约450ml。第一产程5时15分，第二产程0时20分，第三产程0时5分，总产程5时40分，会阴或切口情况为侧切，内缝。合并症及主要风险因素：高龄经产妇妊娠监督、胆囊切除术后状态、羊水过少。新生儿出生体重2800g，Apgar评分1、5、10分钟分别为8、9、9分，分娩结局为活产。 | |
| **临床病例摘要英文翻译 English Translation of De-identified Clinical Case Summary** | Maternal age was 37 years. The obstetric history was G2P2, with 0 previous cesarean deliveries. Gestational age was 39+3 weeks, and the labor onset or induction category was oxytocin induction. The membranes were artificially ruptured; the amniotic fluid volume was approximately 50 mL and its character was clear. The patient had a forceps-assisted vaginal delivery, with estimated intrapartum blood loss of 450 mL. The first, second, and third stages of labor were 5 h 15 min, 0 h 20 min, and 0 h 5 min, respectively; the total labor duration was 5 h 40 min. An episiotomy was repaired with internal sutures. Documented comorbidities and major risk factors were advanced maternal age in a multipara; status post cholecystectomy; oligohydramnios. Neonatal birth weight was 2800 g; Apgar scores at 1, 5, and 10 minutes were 8, 9, 9; and the delivery outcome was live birth. | |
| **模型生成的产褥期护理计划 Model-generated postpartum nursing care plan** | 产妇37岁，G2P2，孕39+3周，经产钳助产阴道分娩分娩，产时出血约450ml，新生儿男婴、体重2800g，Apgar评分8、9、9分。病例提示的重点风险为胎儿窘迫或羊水异常记录、高龄妊娠因素。产后24小时内重点评估体温、脉搏、血压、宫底高度、宫缩、阴道流血、恶露颜色和尿潴留，记录首次排尿和疼痛评分。产后住院及出院后继续观察恶露是否由红色逐渐转淡，乳房是否胀痛，乳头是否皲裂，睡眠和情绪是否稳定。饮食以优质蛋白、含铁食物、蔬菜水果和足量饮水为主，避免盲目大补和长期卧床。本次为产钳助产阴道分娩，会阴伤口、盆底和排尿功能是护理重点。每次大小便后用温水从前向后清洁会阴并轻轻擦干，观察侧切或裂伤处肿胀、硬结、渗血、分泌物和异味。疼痛明显时可侧卧休息，避免久蹲、负重和过早剧烈活动。哺乳方面建议尽早母婴皮肤接触和按需哺乳，指导正确含接，乳胀时先热敷或轻柔按摩再哺乳，乳头疼痛时及时调整姿势。若出现乳房局部红肿硬痛、寒战或高热，应警惕乳腺炎。产时出血量需要持续观察，建议每日记录恶露量、颜色和血块情况，活动后若明显增多，应减少活动并请医护评估。因有胎儿窘迫、羊水污染或新生儿评分变化记录，母婴同室期间应配合观察新生儿吃奶、肤色、体温、呼吸和反应，发现吃奶差、发绀、嗜睡或呼吸异常应立即告知儿科医生。高龄产妇恢复期应避免过度劳累和连续熬夜，家属应参与夜间照护，保证睡眠、营养和情绪支持。出院前应完成用药、伤口、哺乳和避孕宣教，42天左右复查，必要时提前复诊。若出现阴道流血突然增多、鲜红血浸湿卫生巾、恶露恶臭、发热、持续腹痛、伤口化脓、排尿困难、下肢单侧肿痛、胸闷气短，或持续悲伤焦虑及伤害自己或婴儿的想法，应立即就医或急诊处理。 | 患者37岁，G2P2，经产妇，产钳助产+侧切，产时出血450ml（达产后出血标准），合并高龄经产妇、胆囊切除术后、羊水过少，新生儿Apgar 8-9-9。出血450ml需住院严密监测生命体征、宫底高度、恶露量，出院后观察有无晚期出血，补充铁剂。侧切伤口大，每日消毒2次，坐位使用中空坐垫。胆囊术后需低脂饮食，避免油腻，观察有无右上腹痛。羊水过少新生儿注意有无脱水或肾功能异常，鼓励母乳。新生儿Apgar 8分，观察呼吸、肌张力。提供心理支持，出血多易致恐惧。产后1周复查血常规，42天全面复查。 |
| **护理计划英文翻译 English translation of care plan** | The patient is 37 years old, G2P2, delivered at 39+3 weeks by forceps-assisted vaginal delivery, with an estimated intrapartum blood loss of 450 mL. The newborn was a male infant, weighed 2800 g, and had Apgar scores of 8, 9, 9. The main case-based risks include fetal distress or abnormal amniotic fluid findings, advanced maternal age. Within the first 24 hours, assess temperature, pulse, blood pressure, uterine fundal height, uterine tone, vaginal bleeding, lochia color, urinary retention, first voiding, and pain score. During hospitalization and after discharge, continue to observe whether lochia gradually changes from red to lighter color, whether breast engorgement or nipple fissure occurs, and whether sleep and mood remain stable. Diet should include high-quality protein, iron-rich food, vegetables, fruit, and adequate fluids. Avoid blind over-supplementation and prolonged bed rest. Because this was a forceps-assisted vaginal delivery, perineal wound care, pelvic floor recovery, and urinary function are priorities. Rinse the perineum from front to back with warm water after urination or bowel movement and pat dry. Observe the episiotomy or tear for swelling, induration, bleeding, discharge, and odor. Rest in a lateral position if pain is marked, and avoid prolonged squatting, heavy lifting, and early vigorous activity. For breastfeeding, encourage early skin-to-skin contact and feeding on demand. Teach correct latch and positioning. For engorgement, apply warmth or gentle massage before feeding. If nipple pain occurs, adjust latch promptly. Localized breast redness, hard painful swelling, chills, or high fever should raise concern for mastitis. Because blood loss requires closer observation, record the amount, color, and clots in the lochia every day. If bleeding increases clearly after activity, reduce activity and seek clinical assessment. Because fetal distress, meconium-stained fluid, or changed Apgar scores were recorded, cooperate with pediatric observation of feeding, skin color, temperature, breathing, and responsiveness. Poor feeding, cyanosis, excessive sleepiness, or abnormal breathing should be reported immediately. Advanced maternal age may slow recovery, so the family should help with nighttime care and support adequate sleep, nutrition, and emotional stability. Before discharge, provide education on medication, wound care, breastfeeding, and contraception. Arrange a postpartum review around 42 days after delivery, or earlier if symptoms occur. Seek urgent medical care for sudden heavy vaginal bleeding, bright red bleeding soaking pads, foul-smelling lochia, fever, persistent abdominal pain, pus from the wound, difficulty urinating, unilateral leg swelling or pain, chest tightness, shortness of breath, or persistent sadness, anxiety, or thoughts of harming herself or the baby. | The patient is a 37-year-old G2P2 multipara with forceps delivery and episiotomy. Blood loss 450 mL (meets PPH criterion). Comorbidities: advanced age multipara, post-cholecystectomy, oligohydramnios. Newborn Apgar 8-9-9. Close monitoring of vitals, fundus, lochia. Iron supplementation. Low-fat diet; watch for right upper quadrant pain. Newborn – monitor for dehydration and renal function. Newborn observation for HIE. Psychological support for PPH-related fear. Check CBC at 1 week; comprehensive follow-up at 42 days. |
| **Clinical appropriateness 临床适当性** | 5 | 4 |
| **Individualization 个体化程度** | 5 | 5 |
| **Comprehensiveness 完整性** | 5 | 4 |
| **Actionability 可操作性** | 4 | 4 |
| **Safety 安全性** | 5 | 4 |
| **Triage appropriateness 分诊/转诊建议适当性** | 5 | 4 |
| **Structure and clarity 结构与清晰度** | 4 | 4 |

**病例 10 / Case 10**

| **项目 Item** | **ChatGPT 5.5** | **DeepSeek V3** |
| --- | --- | --- |
| **去标识化临床病例摘要 De-identified Clinical Case Summary** | 患者28岁，孕产史为G1P1，既往剖宫产0次，孕周41+1，临产类别为普贝生。自然破膜，羊水量约300ml，羊水性质为Ⅱ°。以自然分娩分娩，产时出血约250ml。第一产程6时0分，第二产程0时49分，第三产程0时6分，总产程6时55分，会阴或切口情况为侧切，内缝。合并症及主要风险因素：胎儿单脐动脉；胎儿窘迫？。新生儿出生体重3600g，Apgar评分1、5、10分钟分别为10、10、10分，分娩结局为活产。 | |
| **临床病例摘要英文翻译 English Translation of De-identified Clinical Case Summary** | Maternal age was 28 years. The obstetric history was G1P1, with 0 previous cesarean deliveries. Gestational age was 41+1 weeks, and the labor onset or induction category was vaginal dinoprostone (Propess) induction. The membranes ruptured spontaneously; the amniotic fluid volume was approximately 300 mL and its character was grade II meconium-stained. The patient had a spontaneous vaginal delivery, with estimated intrapartum blood loss of 250 mL. The first, second, and third stages of labor were 6 h 0 min, 0 h 49 min, and 0 h 6 min, respectively; the total labor duration was 6 h 55 min. An episiotomy was repaired with internal sutures. Documented comorbidities and major risk factors were single umbilical artery; suspected fetal distress. Neonatal birth weight was 3600 g; Apgar scores at 1, 5, and 10 minutes were 10, 10, 10; and the delivery outcome was live birth. | |
| **模型生成的产褥期护理计划 Model-generated postpartum nursing care plan** | 产妇28岁，G1P1，孕41+1周，经阴道自然分娩分娩，产时出血约250ml，新生儿女婴、体重3600g，Apgar评分10、10、10分。病例提示的重点风险为胎儿窘迫或羊水异常记录。产后24小时内重点评估体温、脉搏、血压、宫底高度、宫缩、阴道流血、恶露颜色和尿潴留，记录首次排尿和疼痛评分。产后住院及出院后继续观察恶露是否由红色逐渐转淡，乳房是否胀痛，乳头是否皲裂，睡眠和情绪是否稳定。饮食以优质蛋白、含铁食物、蔬菜水果和足量饮水为主，避免盲目大补和长期卧床。本次为阴道自然分娩，应重点观察会阴裂伤或缝合处恢复。每次大小便后保持局部清洁干燥，观察红肿、渗血、疼痛加重和异常气味。无头晕和出血增多时逐步增加活动，并在医护指导下开始轻柔盆底肌训练。哺乳方面建议尽早母婴皮肤接触和按需哺乳，指导正确含接，乳胀时先热敷或轻柔按摩再哺乳，乳头疼痛时及时调整姿势。若出现乳房局部红肿硬痛、寒战或高热，应警惕乳腺炎。因有胎儿窘迫、羊水污染或新生儿评分变化记录，母婴同室期间应配合观察新生儿吃奶、肤色、体温、呼吸和反应，发现吃奶差、发绀、嗜睡或呼吸异常应立即告知儿科医生。出院前应完成用药、伤口、哺乳和避孕宣教，42天左右复查，必要时提前复诊。若出现阴道流血突然增多、鲜红血浸湿卫生巾、恶露恶臭、发热、持续腹痛、伤口化脓、排尿困难、下肢单侧肿痛、胸闷气短，或持续悲伤焦虑及伤害自己或婴儿的想法，应立即就医或急诊处理。 | 患者28岁，初产妇，自然分娩+侧切，羊水Ⅱ°污染，合并单脐动脉、可疑胎儿窘迫，新生儿体重3600g（巨大儿）。单脐动脉可能合并心脏畸形，新生儿需在1个月内行心脏超声检查。羊水Ⅱ°污染+可疑窘迫，监测新生儿呼吸、胎粪吸入综合征，即使Apgar正常。巨大儿致子宫过度膨胀，加强宫底按摩预防宫缩乏力性出血。侧切伤口每日消毒2次，保持干燥。鼓励母乳喂养，注意新生儿吸吮能力。高蛋白饮食促进伤口愈合。提供心理支持，解释单脐动脉多数不影响健康。产后42天复查，新生儿心脏超声。 |
| **护理计划英文翻译 English translation of care plan** | The patient is 28 years old, G1P1, delivered at 41+1 weeks by spontaneous vaginal delivery, with an estimated intrapartum blood loss of 250 mL. The newborn was a female infant, weighed 3600 g, and had Apgar scores of 10, 10, 10. The main case-based risks include fetal distress or abnormal amniotic fluid findings. Within the first 24 hours, assess temperature, pulse, blood pressure, uterine fundal height, uterine tone, vaginal bleeding, lochia color, urinary retention, first voiding, and pain score. During hospitalization and after discharge, continue to observe whether lochia gradually changes from red to lighter color, whether breast engorgement or nipple fissure occurs, and whether sleep and mood remain stable. Diet should include high-quality protein, iron-rich food, vegetables, fruit, and adequate fluids. Avoid blind over-supplementation and prolonged bed rest. Because this was a spontaneous vaginal birth, recovery of any perineal tear or suture site should be observed. Keep the area clean and dry after urination or bowel movement and watch for redness, swelling, bleeding, worsening pain, or abnormal odor. If there is no dizziness or increased bleeding, increase activity gradually and begin gentle pelvic floor exercises under professional guidance. For breastfeeding, encourage early skin-to-skin contact and feeding on demand. Teach correct latch and positioning. For engorgement, apply warmth or gentle massage before feeding. If nipple pain occurs, adjust latch promptly. Localized breast redness, hard painful swelling, chills, or high fever should raise concern for mastitis. Because fetal distress, meconium-stained fluid, or changed Apgar scores were recorded, cooperate with pediatric observation of feeding, skin color, temperature, breathing, and responsiveness. Poor feeding, cyanosis, excessive sleepiness, or abnormal breathing should be reported immediately. Before discharge, provide education on medication, wound care, breastfeeding, and contraception. Arrange a postpartum review around 42 days after delivery, or earlier if symptoms occur. Seek urgent medical care for sudden heavy vaginal bleeding, bright red bleeding soaking pads, foul-smelling lochia, fever, persistent abdominal pain, pus from the wound, difficulty urinating, unilateral leg swelling or pain, chest tightness, shortness of breath, or persistent sadness, anxiety, or thoughts of harming herself or the baby. | The patient is a 28-year-old primipara with spontaneous delivery and episiotomy. Grade II meconium-stained fluid. Comorbidities: single umbilical artery, suspected fetal distress. Newborn macrosomia (3600 g). Newborn requires cardiac ultrasound within 1 month due to SUA. Monitor for meconium aspiration syndrome. Macrosomia causes uterine overdistension – aggressive fundal massage to prevent atonic PPH. Clean episiotomy twice daily. Encourage breastfeeding. High-protein diet. Provide reassurance about SUA. Follow-up at 42 days; newborn cardiac echo. |
| **Clinical appropriateness 临床适当性** | 4 | 4 |
| **Individualization 个体化程度** | 4 | 5 |
| **Comprehensiveness 完整性** | 4 | 4 |
| **Actionability 可操作性** | 4 | 4 |
| **Safety 安全性** | 5 | 4 |
| **Triage appropriateness 分诊/转诊建议适当性** | 5 | 3 |
| **Structure and clarity 结构与清晰度** | 4 | 4 |

**病例 11 / Case 11**

| **项目 Item** | **ChatGPT 5.5** | **DeepSeek V3** |
| --- | --- | --- |
| **去标识化临床病例摘要 De-identified Clinical Case Summary** | 患者30岁，孕产史为G3P1，既往剖宫产0次，孕周36+6，临产类别为催产素引产。自然破膜，羊水量约100ml，羊水性质为清。以产钳分娩，产时出血约220ml。第一产程5时25分，第二产程1时51分，第三产程0时9分，总产程7时25分，会阴或切口情况为侧切，内缝。合并症及主要风险因素：未足月胎膜早破；不良孕产个人史、胎儿窘迫？。新生儿出生体重2760g，Apgar评分1、5、10分钟分别为9、9、9分，分娩结局为活产。 | |
| **临床病例摘要英文翻译 English Translation of De-identified Clinical Case Summary** | Maternal age was 30 years. The obstetric history was G3P1, with 0 previous cesarean deliveries. Gestational age was 36+6 weeks, and the labor onset or induction category was oxytocin induction. The membranes ruptured spontaneously; the amniotic fluid volume was approximately 100 mL and its character was clear. The patient had a forceps-assisted vaginal delivery, with estimated intrapartum blood loss of 220 mL. The first, second, and third stages of labor were 5 h 25 min, 1 h 51 min, and 0 h 9 min, respectively; the total labor duration was 7 h 25 min. An episiotomy was repaired with internal sutures. Documented comorbidities and major risk factors were preterm premature rupture of membranes; poor obstetric history; suspected fetal distress. Neonatal birth weight was 2760 g; Apgar scores at 1, 5, and 10 minutes were 9, 9, 9; and the delivery outcome was live birth. | |
| **模型生成的产褥期护理计划 Model-generated postpartum nursing care plan** | 产妇30岁，G3P1，孕36+6周，经产钳助产阴道分娩分娩，产时出血约220ml，新生儿女婴、体重2760g，Apgar评分9、9、9分。病例提示的重点风险为胎膜早破或感染相关风险、胎儿窘迫或羊水异常记录。产后24小时内重点评估体温、脉搏、血压、宫底高度、宫缩、阴道流血、恶露颜色和尿潴留，记录首次排尿和疼痛评分。产后住院及出院后继续观察恶露是否由红色逐渐转淡，乳房是否胀痛，乳头是否皲裂，睡眠和情绪是否稳定。饮食以优质蛋白、含铁食物、蔬菜水果和足量饮水为主，避免盲目大补和长期卧床。本次为产钳助产阴道分娩，会阴伤口、盆底和排尿功能是护理重点。每次大小便后用温水从前向后清洁会阴并轻轻擦干，观察侧切或裂伤处肿胀、硬结、渗血、分泌物和异味。疼痛明显时可侧卧休息，避免久蹲、负重和过早剧烈活动。哺乳方面建议尽早母婴皮肤接触和按需哺乳，指导正确含接，乳胀时先热敷或轻柔按摩再哺乳，乳头疼痛时及时调整姿势。若出现乳房局部红肿硬痛、寒战或高热，应警惕乳腺炎。存在胎膜早破、B族链球菌感染、阴道炎或宫内感染疑虑时，应加强体温、下腹压痛、恶露气味、会阴或切口渗液观察，发热或恶露恶臭时及时复诊。因有胎儿窘迫、羊水污染或新生儿评分变化记录，母婴同室期间应配合观察新生儿吃奶、肤色、体温、呼吸和反应，发现吃奶差、发绀、嗜睡或呼吸异常应立即告知儿科医生。出院前应完成用药、伤口、哺乳和避孕宣教，42天左右复查，必要时提前复诊。若出现阴道流血突然增多、鲜红血浸湿卫生巾、恶露恶臭、发热、持续腹痛、伤口化脓、排尿困难、下肢单侧肿痛、胸闷气短，或持续悲伤焦虑及伤害自己或婴儿的想法，应立即就医或急诊处理。 | 患者30岁，G3P1，产钳助产+侧切，合并未足月胎膜早破（36+6周）、不良孕产史、可疑胎儿窘迫，新生儿Apgar 9-9-9，体重2760g（晚期早产儿）。早产儿需保暖，监测血糖、黄疸，注意吸吮-吞咽不协调，鼓励母乳喂养，可能需要母乳强化剂。PPROM+早产，产褥感染风险高，每日测体温4次，观察子宫压痛、恶露气味，遵医嘱完成抗生素。会阴侧切每日消毒2次。不良孕产史及可疑窘迫易致焦虑，提供心理支持。增加蛋白质、钙、铁摄入。产后1周复查感染指标，42天复查；早产儿需定期随访。 |
| **护理计划英文翻译 English translation of care plan** | The patient is 30 years old, G3P1, delivered at 36+6 weeks by forceps-assisted vaginal delivery, with an estimated intrapartum blood loss of 220 mL. The newborn was a female infant, weighed 2760 g, and had Apgar scores of 9, 9, 9. The main case-based risks include premature rupture of membranes or infection-related risk, fetal distress or abnormal amniotic fluid findings. Within the first 24 hours, assess temperature, pulse, blood pressure, uterine fundal height, uterine tone, vaginal bleeding, lochia color, urinary retention, first voiding, and pain score. During hospitalization and after discharge, continue to observe whether lochia gradually changes from red to lighter color, whether breast engorgement or nipple fissure occurs, and whether sleep and mood remain stable. Diet should include high-quality protein, iron-rich food, vegetables, fruit, and adequate fluids. Avoid blind over-supplementation and prolonged bed rest. Because this was a forceps-assisted vaginal delivery, perineal wound care, pelvic floor recovery, and urinary function are priorities. Rinse the perineum from front to back with warm water after urination or bowel movement and pat dry. Observe the episiotomy or tear for swelling, induration, bleeding, discharge, and odor. Rest in a lateral position if pain is marked, and avoid prolonged squatting, heavy lifting, and early vigorous activity. For breastfeeding, encourage early skin-to-skin contact and feeding on demand. Teach correct latch and positioning. For engorgement, apply warmth or gentle massage before feeding. If nipple pain occurs, adjust latch promptly. Localized breast redness, hard painful swelling, chills, or high fever should raise concern for mastitis. With premature rupture of membranes, group B streptococcus, vaginitis, or suspected intrauterine infection, monitor temperature, lower abdominal tenderness, lochia odor, and drainage from the perineal wound or incision. Return promptly for fever or foul-smelling lochia. Because fetal distress, meconium-stained fluid, or changed Apgar scores were recorded, cooperate with pediatric observation of feeding, skin color, temperature, breathing, and responsiveness. Poor feeding, cyanosis, excessive sleepiness, or abnormal breathing should be reported immediately. Before discharge, provide education on medication, wound care, breastfeeding, and contraception. Arrange a postpartum review around 42 days after delivery, or earlier if symptoms occur. Seek urgent medical care for sudden heavy vaginal bleeding, bright red bleeding soaking pads, foul-smelling lochia, fever, persistent abdominal pain, pus from the wound, difficulty urinating, unilateral leg swelling or pain, chest tightness, shortness of breath, or persistent sadness, anxiety, or thoughts of harming herself or the baby. | The patient is a 30-year-old G3P1 with forceps delivery and episiotomy. Comorbidities: PPROM at 36+6 weeks, poor obstetric history, suspected fetal distress. Late preterm newborn (2760 g), Apgar 9-9-9. Maintain warmth, monitor glucose, jaundice, feeding coordination; encourage breastfeeding with possible fortifier. High infection risk – check temperature 4 times daily, monitor uterine tenderness, lochia odor; complete antibiotics. Episiotomy care. Provide psychological support for poor obstetric history. Increase protein, calcium, iron. Check infection markers at 1 week; follow-up at 42 days; preterm infant program. |
| **Clinical appropriateness 临床适当性** | 5 | 3 |
| **Individualization 个体化程度** | 4 | 5 |
| **Comprehensiveness 完整性** | 5 | 4 |
| **Actionability 可操作性** | 4 | 4 |
| **Safety 安全性** | 5 | 3 |
| **Triage appropriateness 分诊/转诊建议适当性** | 5 | 3 |
| **Structure and clarity 结构与清晰度** | 4 | 4 |

**病例 12 / Case 12**

| **项目 Item** | **ChatGPT 5.5** | **DeepSeek V3** |
| --- | --- | --- |
| **去标识化临床病例摘要 De-identified Clinical Case Summary** | 患者37岁，孕产史为G2P2，既往剖宫产0次，孕周39+3，临产类别为自然临产。自然破膜，羊水量约300ml，羊水性质为清。以自然分娩分娩，产时出血约310ml。第一产程4时20分，第二产程0时12分，第三产程0时8分，总产程4时40分，会阴或切口情况为Ⅰ，内缝。合并症及主要风险因素：高龄经产妇妊娠监督。新生儿出生体重3080g，Apgar评分1、5、10分钟分别为10、10、10分，分娩结局为活产+畸形。 | |
| **临床病例摘要英文翻译 English Translation of De-identified Clinical Case Summary** | Maternal age was 37 years. The obstetric history was G2P2, with 0 previous cesarean deliveries. Gestational age was 39+3 weeks, and the labor onset or induction category was spontaneous labor. The membranes ruptured spontaneously; the amniotic fluid volume was approximately 300 mL and its character was clear. The patient had a spontaneous vaginal delivery, with estimated intrapartum blood loss of 310 mL. The first, second, and third stages of labor were 4 h 20 min, 0 h 12 min, and 0 h 8 min, respectively; the total labor duration was 4 h 40 min. A first-degree perineal laceration was repaired with internal sutures. Documented comorbidities and major risk factors were advanced maternal age in a multipara. Neonatal birth weight was 3080 g; Apgar scores at 1, 5, and 10 minutes were 10, 10, 10; and the delivery outcome was live birth with a documented congenital anomaly. | |
| **模型生成的产褥期护理计划 Model-generated postpartum nursing care plan** | 产妇37岁，G2P2，孕39+3周，经阴道自然分娩分娩，产时出血约310ml，新生儿男婴、体重3080g，Apgar评分10、10、10分。病例提示的重点风险为高龄妊娠因素。产后24小时内重点评估体温、脉搏、血压、宫底高度、宫缩、阴道流血、恶露颜色和尿潴留，记录首次排尿和疼痛评分。产后住院及出院后继续观察恶露是否由红色逐渐转淡，乳房是否胀痛，乳头是否皲裂，睡眠和情绪是否稳定。饮食以优质蛋白、含铁食物、蔬菜水果和足量饮水为主，避免盲目大补和长期卧床。本次为阴道自然分娩，应重点观察会阴裂伤或缝合处恢复。每次大小便后保持局部清洁干燥，观察红肿、渗血、疼痛加重和异常气味。无头晕和出血增多时逐步增加活动，并在医护指导下开始轻柔盆底肌训练。哺乳方面建议尽早母婴皮肤接触和按需哺乳，指导正确含接，乳胀时先热敷或轻柔按摩再哺乳，乳头疼痛时及时调整姿势。若出现乳房局部红肿硬痛、寒战或高热，应警惕乳腺炎。高龄产妇恢复期应避免过度劳累和连续熬夜，家属应参与夜间照护，保证睡眠、营养和情绪支持。出院前应完成用药、伤口、哺乳和避孕宣教，42天左右复查，必要时提前复诊。若出现阴道流血突然增多、鲜红血浸湿卫生巾、恶露恶臭、发热、持续腹痛、伤口化脓、排尿困难、下肢单侧肿痛、胸闷气短，或持续悲伤焦虑及伤害自己或婴儿的想法，应立即就医或急诊处理。 | 患者37岁，G2P2，自然分娩+I度裂伤，合并高龄经产妇，新生儿为活产+畸形（未明确类型）。产后需立即进行新生儿畸形评估，明确诊断，指导家长接受专科评估及遗传咨询。提供悲伤辅导和心理支持，连接支持团体。子宫复旧：高龄经产妇子宫收缩能力可能下降，加强宫底按摩，观察恶露量（产时出血310ml）。I度裂伤温水坐浴。根据畸形情况决定母乳喂养可行性，如需特殊配方给予指导。建议遗传咨询后再考虑下一次妊娠，落实高效避孕。产妇42天复查，新生儿按专科随访。 |
| **护理计划英文翻译 English translation of care plan** | The patient is 37 years old, G2P2, delivered at 39+3 weeks by spontaneous vaginal delivery, with an estimated intrapartum blood loss of 310 mL. The newborn was a male infant, weighed 3080 g, and had Apgar scores of 10, 10, 10. The main case-based risks include advanced maternal age. Within the first 24 hours, assess temperature, pulse, blood pressure, uterine fundal height, uterine tone, vaginal bleeding, lochia color, urinary retention, first voiding, and pain score. During hospitalization and after discharge, continue to observe whether lochia gradually changes from red to lighter color, whether breast engorgement or nipple fissure occurs, and whether sleep and mood remain stable. Diet should include high-quality protein, iron-rich food, vegetables, fruit, and adequate fluids. Avoid blind over-supplementation and prolonged bed rest. Because this was a spontaneous vaginal birth, recovery of any perineal tear or suture site should be observed. Keep the area clean and dry after urination or bowel movement and watch for redness, swelling, bleeding, worsening pain, or abnormal odor. If there is no dizziness or increased bleeding, increase activity gradually and begin gentle pelvic floor exercises under professional guidance. For breastfeeding, encourage early skin-to-skin contact and feeding on demand. Teach correct latch and positioning. For engorgement, apply warmth or gentle massage before feeding. If nipple pain occurs, adjust latch promptly. Localized breast redness, hard painful swelling, chills, or high fever should raise concern for mastitis. Advanced maternal age may slow recovery, so the family should help with nighttime care and support adequate sleep, nutrition, and emotional stability. Before discharge, provide education on medication, wound care, breastfeeding, and contraception. Arrange a postpartum review around 42 days after delivery, or earlier if symptoms occur. Seek urgent medical care for sudden heavy vaginal bleeding, bright red bleeding soaking pads, foul-smelling lochia, fever, persistent abdominal pain, pus from the wound, difficulty urinating, unilateral leg swelling or pain, chest tightness, shortness of breath, or persistent sadness, anxiety, or thoughts of harming herself or the baby. | The patient is a 37-year-old G2P2 multipara with spontaneous delivery and first-degree laceration. Comorbidity: advanced maternal age multipara. Newborn has congenital anomaly (type unspecified). Immediate evaluation and genetic counseling for the anomaly. Provide grief counseling and support groups. Uterine involution: advanced age may impair tone – aggressive fundal massage, monitor lochia (blood loss 310 mL). Warm sitz baths. Breastfeeding decision based on anomaly; guide on formula if needed. Recommend genetic counseling before next pregnancy; use effective contraception. Maternal follow-up at 42 days; newborn specialist follow-up. |
| **Clinical appropriateness 临床适当性** | 4 | 4 |
| **Individualization 个体化程度** | 3 | 4 |
| **Comprehensiveness 完整性** | 4 | 4 |
| **Actionability 可操作性** | 4 | 4 |
| **Safety 安全性** | 5 | 4 |
| **Triage appropriateness 分诊/转诊建议适当性** | 5 | 3 |
| **Structure and clarity 结构与清晰度** | 4 | 4 |

**病例 13 / Case 13**

| **项目 Item** | **ChatGPT 5.5** | **DeepSeek V3** |
| --- | --- | --- |
| **去标识化临床病例摘要 De-identified Clinical Case Summary** | 患者32岁，孕产史为G3P2，既往剖宫产0次，孕周39+6，临产类别为自然临产。自然破膜，羊水量约300ml，羊水性质为清。以自然分娩分娩，产时出血约250ml。第一产程2时30分，第二产程0时25分，第三产程0时8分，总产程3时3分，会阴或切口情况为Ⅰ，内缝。合并症及主要风险因素：无。新生儿出生体重3330g，Apgar评分1、5、10分钟分别为10、10、10分，分娩结局为活产。 | |
| **临床病例摘要英文翻译 English Translation of De-identified Clinical Case Summary** | Maternal age was 32 years. The obstetric history was G3P2, with 0 previous cesarean deliveries. Gestational age was 39+6 weeks, and the labor onset or induction category was spontaneous labor. The membranes ruptured spontaneously; the amniotic fluid volume was approximately 300 mL and its character was clear. The patient had a spontaneous vaginal delivery, with estimated intrapartum blood loss of 250 mL. The first, second, and third stages of labor were 2 h 30 min, 0 h 25 min, and 0 h 8 min, respectively; the total labor duration was 3 h 3 min. A first-degree perineal laceration was repaired with internal sutures. No comorbidities or major risk factors were documented. Neonatal birth weight was 3330 g; Apgar scores at 1, 5, and 10 minutes were 10, 10, 10; and the delivery outcome was live birth. | |
| **模型生成的产褥期护理计划 Model-generated postpartum nursing care plan** | 产妇32岁，G3P2，孕39+6周，经阴道自然分娩分娩，产时出血约250ml，新生儿女婴、体重3330g，Apgar评分10、10、10分。病例提示的重点风险为未记录特殊合并症。产后24小时内重点评估体温、脉搏、血压、宫底高度、宫缩、阴道流血、恶露颜色和尿潴留，记录首次排尿和疼痛评分。产后住院及出院后继续观察恶露是否由红色逐渐转淡，乳房是否胀痛，乳头是否皲裂，睡眠和情绪是否稳定。饮食以优质蛋白、含铁食物、蔬菜水果和足量饮水为主，避免盲目大补和长期卧床。本次为阴道自然分娩，应重点观察会阴裂伤或缝合处恢复。每次大小便后保持局部清洁干燥，观察红肿、渗血、疼痛加重和异常气味。无头晕和出血增多时逐步增加活动，并在医护指导下开始轻柔盆底肌训练。哺乳方面建议尽早母婴皮肤接触和按需哺乳，指导正确含接，乳胀时先热敷或轻柔按摩再哺乳，乳头疼痛时及时调整姿势。若出现乳房局部红肿硬痛、寒战或高热，应警惕乳腺炎。目前未记录特殊合并症，仍需按常规产褥期管理执行，重点是子宫复旧、恶露、伤口、哺乳、营养、活动和心理状态。出院前应完成用药、伤口、哺乳和避孕宣教，42天左右复查，必要时提前复诊。若出现阴道流血突然增多、鲜红血浸湿卫生巾、恶露恶臭、发热、持续腹痛、伤口化脓、排尿困难、下肢单侧肿痛、胸闷气短，或持续悲伤焦虑及伤害自己或婴儿的想法，应立即就医或急诊处理。 | 患者32岁，G3P2，自然分娩+I度裂伤，合并脐带绕颈2周，无其他合并症，新生儿Apgar 10-10-10。脐带绕颈2周虽Apgar正常，仍需观察新生儿有无缺氧表现（呼吸、肌张力、喂养）24小时。常规产后护理：观察恶露，按摩宫底。I度裂伤温水坐浴。按需哺乳，注意乳胀。尽早下床，避免劳累。多饮水防便秘。常规产后情绪观察。42天复查。该病例相对简单，但脐带绕颈需重视新生儿观察。 |
| **护理计划英文翻译 English translation of care plan** | The patient is 32 years old, G3P2, delivered at 39+6 weeks by spontaneous vaginal delivery, with an estimated intrapartum blood loss of 250 mL. The newborn was a female infant, weighed 3330 g, and had Apgar scores of 10, 10, 10. The main case-based risks include no special comorbidity was recorded. Within the first 24 hours, assess temperature, pulse, blood pressure, uterine fundal height, uterine tone, vaginal bleeding, lochia color, urinary retention, first voiding, and pain score. During hospitalization and after discharge, continue to observe whether lochia gradually changes from red to lighter color, whether breast engorgement or nipple fissure occurs, and whether sleep and mood remain stable. Diet should include high-quality protein, iron-rich food, vegetables, fruit, and adequate fluids. Avoid blind over-supplementation and prolonged bed rest. Because this was a spontaneous vaginal birth, recovery of any perineal tear or suture site should be observed. Keep the area clean and dry after urination or bowel movement and watch for redness, swelling, bleeding, worsening pain, or abnormal odor. If there is no dizziness or increased bleeding, increase activity gradually and begin gentle pelvic floor exercises under professional guidance. For breastfeeding, encourage early skin-to-skin contact and feeding on demand. Teach correct latch and positioning. For engorgement, apply warmth or gentle massage before feeding. If nipple pain occurs, adjust latch promptly. Localized breast redness, hard painful swelling, chills, or high fever should raise concern for mastitis. No special complication was recorded, but routine postpartum management should still focus on uterine involution, lochia, wounds, breastfeeding, nutrition, activity, and mental health. Before discharge, provide education on medication, wound care, breastfeeding, and contraception. Arrange a postpartum review around 42 days after delivery, or earlier if symptoms occur. Seek urgent medical care for sudden heavy vaginal bleeding, bright red bleeding soaking pads, foul-smelling lochia, fever, persistent abdominal pain, pus from the wound, difficulty urinating, unilateral leg swelling or pain, chest tightness, shortness of breath, or persistent sadness, anxiety, or thoughts of harming herself or the baby. | The patient is a 32-year-old G3P2 with spontaneous delivery and first-degree laceration. Comorbidity: nuchal cord 2 loops. Newborn Apgar 10-10-10. Observe newborn for signs of hypoxia (respiration, tone, feeding) for 24 hours despite normal Apgars. Routine care: monitor lochia, fundal massage. Warm sitz baths. Breastfeed on demand. Early ambulation. Prevent constipation. Monitor mood. Follow-up at 42 days. |
| **Clinical appropriateness 临床适当性** | 4 | 4 |
| **Individualization 个体化程度** | 3 | 4 |
| **Comprehensiveness 完整性** | 4 | 4 |
| **Actionability 可操作性** | 4 | 4 |
| **Safety 安全性** | 5 | 4 |
| **Triage appropriateness 分诊/转诊建议适当性** | 5 | 3 |
| **Structure and clarity 结构与清晰度** | 4 | 4 |

**病例 14 / Case 14**

| **项目 Item** | **ChatGPT 5.5** | **DeepSeek V3** |
| --- | --- | --- |
| **去标识化临床病例摘要 De-identified Clinical Case Summary** | 患者30岁，孕产史为G1P1，既往剖宫产0次，孕周39+4，临产类别为催产素引产。自然破膜，羊水量约200ml，羊水性质为清。以自然分娩分娩，产时出血约230ml。第一产程3时27分，第二产程0时37分，第三产程0时6分，总产程4时10分，会阴或切口情况为Ⅰ，内缝。合并症及主要风险因素：羊水过少、胎儿生长缓慢 ？B族链球菌感染（阴道）、胎膜早破、胎儿窘迫？。新生儿出生体重3000g，Apgar评分1、5、10分钟分别为10、10、10分，分娩结局为活产。 | |
| **临床病例摘要英文翻译 English Translation of De-identified Clinical Case Summary** | Maternal age was 30 years. The obstetric history was G1P1, with 0 previous cesarean deliveries. Gestational age was 39+4 weeks, and the labor onset or induction category was oxytocin induction. The membranes ruptured spontaneously; the amniotic fluid volume was approximately 200 mL and its character was clear. The patient had a spontaneous vaginal delivery, with estimated intrapartum blood loss of 230 mL. The first, second, and third stages of labor were 3 h 27 min, 0 h 37 min, and 0 h 6 min, respectively; the total labor duration was 4 h 10 min. A first-degree perineal laceration was repaired with internal sutures. Documented comorbidities and major risk factors were oligohydramnios; suspected fetal growth restriction; group B Streptococcus infection (vaginal); premature rupture of membranes; suspected fetal distress. Neonatal birth weight was 3000 g; Apgar scores at 1, 5, and 10 minutes were 10, 10, 10; and the delivery outcome was live birth. | |
| **模型生成的产褥期护理计划 Model-generated postpartum nursing care plan** | 产妇30岁，G1P1，孕39+4周，经阴道自然分娩分娩，产时出血约230ml，新生儿女婴、体重3000g，Apgar评分10、10、10分。病例提示的重点风险为胎膜早破或感染相关风险、胎儿窘迫或羊水异常记录。产后24小时内重点评估体温、脉搏、血压、宫底高度、宫缩、阴道流血、恶露颜色和尿潴留，记录首次排尿和疼痛评分。产后住院及出院后继续观察恶露是否由红色逐渐转淡，乳房是否胀痛，乳头是否皲裂，睡眠和情绪是否稳定。饮食以优质蛋白、含铁食物、蔬菜水果和足量饮水为主，避免盲目大补和长期卧床。本次为阴道自然分娩，应重点观察会阴裂伤或缝合处恢复。每次大小便后保持局部清洁干燥，观察红肿、渗血、疼痛加重和异常气味。无头晕和出血增多时逐步增加活动，并在医护指导下开始轻柔盆底肌训练。哺乳方面建议尽早母婴皮肤接触和按需哺乳，指导正确含接，乳胀时先热敷或轻柔按摩再哺乳，乳头疼痛时及时调整姿势。若出现乳房局部红肿硬痛、寒战或高热，应警惕乳腺炎。存在胎膜早破、B族链球菌感染、阴道炎或宫内感染疑虑时，应加强体温、下腹压痛、恶露气味、会阴或切口渗液观察，发热或恶露恶臭时及时复诊。因有胎儿窘迫、羊水污染或新生儿评分变化记录，母婴同室期间应配合观察新生儿吃奶、肤色、体温、呼吸和反应，发现吃奶差、发绀、嗜睡或呼吸异常应立即告知儿科医生。出院前应完成用药、伤口、哺乳和避孕宣教，42天左右复查，必要时提前复诊。若出现阴道流血突然增多、鲜红血浸湿卫生巾、恶露恶臭、发热、持续腹痛、伤口化脓、排尿困难、下肢单侧肿痛、胸闷气短，或持续悲伤焦虑及伤害自己或婴儿的想法，应立即就医或急诊处理。 | 患者30岁，初产妇，自然分娩+I度裂伤，合并羊水过少、可疑胎儿生长缓慢、GBS感染、胎膜早破、可疑胎儿窘迫、脐带隐性脱垂。GBS感染+PPROM需遵医嘱完成抗生素疗程，监测产妇体温、腹痛、恶露；新生儿需观察GBS早发型败血症（呼吸窘迫、嗜睡、喂养差）。胎儿生长缓慢/羊水过少，产后关注新生儿血糖、体温、喂养。隐性脱垂后遗症：观察新生儿呼吸。产时出血230ml，正常。GBS不影响母乳喂养。提供心理支持。产后2周复查新生儿体重增长，42天产妇复查。 |
| **护理计划英文翻译 English translation of care plan** | The patient is 30 years old, G1P1, delivered at 39+4 weeks by spontaneous vaginal delivery, with an estimated intrapartum blood loss of 230 mL. The newborn was a female infant, weighed 3000 g, and had Apgar scores of 10, 10, 10. The main case-based risks include premature rupture of membranes or infection-related risk, fetal distress or abnormal amniotic fluid findings. Within the first 24 hours, assess temperature, pulse, blood pressure, uterine fundal height, uterine tone, vaginal bleeding, lochia color, urinary retention, first voiding, and pain score. During hospitalization and after discharge, continue to observe whether lochia gradually changes from red to lighter color, whether breast engorgement or nipple fissure occurs, and whether sleep and mood remain stable. Diet should include high-quality protein, iron-rich food, vegetables, fruit, and adequate fluids. Avoid blind over-supplementation and prolonged bed rest. Because this was a spontaneous vaginal birth, recovery of any perineal tear or suture site should be observed. Keep the area clean and dry after urination or bowel movement and watch for redness, swelling, bleeding, worsening pain, or abnormal odor. If there is no dizziness or increased bleeding, increase activity gradually and begin gentle pelvic floor exercises under professional guidance. For breastfeeding, encourage early skin-to-skin contact and feeding on demand. Teach correct latch and positioning. For engorgement, apply warmth or gentle massage before feeding. If nipple pain occurs, adjust latch promptly. Localized breast redness, hard painful swelling, chills, or high fever should raise concern for mastitis. With premature rupture of membranes, group B streptococcus, vaginitis, or suspected intrauterine infection, monitor temperature, lower abdominal tenderness, lochia odor, and drainage from the perineal wound or incision. Return promptly for fever or foul-smelling lochia. Because fetal distress, meconium-stained fluid, or changed Apgar scores were recorded, cooperate with pediatric observation of feeding, skin color, temperature, breathing, and responsiveness. Poor feeding, cyanosis, excessive sleepiness, or abnormal breathing should be reported immediately. Before discharge, provide education on medication, wound care, breastfeeding, and contraception. Arrange a postpartum review around 42 days after delivery, or earlier if symptoms occur. Seek urgent medical care for sudden heavy vaginal bleeding, bright red bleeding soaking pads, foul-smelling lochia, fever, persistent abdominal pain, pus from the wound, difficulty urinating, unilateral leg swelling or pain, chest tightness, shortness of breath, or persistent sadness, anxiety, or thoughts of harming herself or the baby. | The patient is a 30-year-old primipara with spontaneous delivery and first-degree laceration. Comorbidities: oligohydramnios, suspected slow fetal growth, GBS infection, PPROM, suspected fetal distress, occult cord prolapse. Complete antibiotics for GBS+PPROM. Monitor mother for infection; observe newborn for early-onset GBS sepsis (respiratory distress, lethargy, poor feeding). Monitor newborn glucose, temperature, feeding due to slow growth. Observe newborn respiration. Blood loss 230 mL, normal. Breastfeeding safe. Provide psychological support. Check newborn weight gain at 2 weeks; maternal follow-up at 42 days. |
| **Clinical appropriateness 临床适当性** | 5 | 4 |
| **Individualization 个体化程度** | 4 | 5 |
| **Comprehensiveness 完整性** | 5 | 4 |
| **Actionability 可操作性** | 4 | 4 |
| **Safety 安全性** | 5 | 4 |
| **Triage appropriateness 分诊/转诊建议适当性** | 5 | 4 |
| **Structure and clarity 结构与清晰度** | 4 | 4 |

**病例 15 / Case 15**

| **项目 Item** | **ChatGPT 5.5** | **DeepSeek V3** |
| --- | --- | --- |
| **去标识化临床病例摘要 De-identified Clinical Case Summary** | 患者37岁，孕产史为G2P2，既往剖宫产0次，孕周35，临产类别为催产素引产。自然破膜，羊水量约300ml，羊水性质为清。以自然分娩分娩，产时出血约220ml。第一产程4时0分，第二产程0时16分，第三产程0时4分，总产程4时20分，会阴或切口情况为Ⅰ，内缝。合并症及主要风险因素：胎膜早破、妊娠期糖尿病、胚胎移植术后、高龄经产妇妊娠监督。新生儿出生体重2330g，Apgar评分1、5、10分钟分别为9、9、9分，分娩结局为活产。 | |
| **临床病例摘要英文翻译 English Translation of De-identified Clinical Case Summary** | Maternal age was 37 years. The obstetric history was G2P2, with 0 previous cesarean deliveries. Gestational age was 35 weeks, and the labor onset or induction category was oxytocin induction. The membranes ruptured spontaneously; the amniotic fluid volume was approximately 300 mL and its character was clear. The patient had a spontaneous vaginal delivery, with estimated intrapartum blood loss of 220 mL. The first, second, and third stages of labor were 4 h 0 min, 0 h 16 min, and 0 h 4 min, respectively; the total labor duration was 4 h 20 min. A first-degree perineal laceration was repaired with internal sutures. Documented comorbidities and major risk factors were premature rupture of membranes; gestational diabetes mellitus; status post embryo transfer; advanced maternal age in a multipara. Neonatal birth weight was 2330 g; Apgar scores at 1, 5, and 10 minutes were 9, 9, 9; and the delivery outcome was live birth. | |
| **模型生成的产褥期护理计划 Model-generated postpartum nursing care plan** | 产妇37岁，G2P2，孕35周，经阴道自然分娩分娩，产时出血约220ml，新生儿男婴、体重2330g，Apgar评分9、9、9分。病例提示的重点风险为妊娠期糖尿病、胎膜早破或感染相关风险、胎儿窘迫或羊水异常记录、高龄妊娠因素。产后24小时内重点评估体温、脉搏、血压、宫底高度、宫缩、阴道流血、恶露颜色和尿潴留，记录首次排尿和疼痛评分。产后住院及出院后继续观察恶露是否由红色逐渐转淡，乳房是否胀痛，乳头是否皲裂，睡眠和情绪是否稳定。饮食以优质蛋白、含铁食物、蔬菜水果和足量饮水为主，避免盲目大补和长期卧床。本次为阴道自然分娩，应重点观察会阴裂伤或缝合处恢复。每次大小便后保持局部清洁干燥，观察红肿、渗血、疼痛加重和异常气味。无头晕和出血增多时逐步增加活动，并在医护指导下开始轻柔盆底肌训练。哺乳方面建议尽早母婴皮肤接触和按需哺乳，指导正确含接，乳胀时先热敷或轻柔按摩再哺乳，乳头疼痛时及时调整姿势。若出现乳房局部红肿硬痛、寒战或高热，应警惕乳腺炎。有妊娠期糖尿病者，产后饮食应控制精制糖和高油食物，少量多餐，结合恢复情况逐步活动，并按医嘱在产后复查血糖或糖耐量。存在胎膜早破、B族链球菌感染、阴道炎或宫内感染疑虑时，应加强体温、下腹压痛、恶露气味、会阴或切口渗液观察，发热或恶露恶臭时及时复诊。因有胎儿窘迫、羊水污染或新生儿评分变化记录，母婴同室期间应配合观察新生儿吃奶、肤色、体温、呼吸和反应，发现吃奶差、发绀、嗜睡或呼吸异常应立即告知儿科医生。高龄产妇恢复期应避免过度劳累和连续熬夜，家属应参与夜间照护，保证睡眠、营养和情绪支持。出院前应完成用药、伤口、哺乳和避孕宣教，42天左右复查，必要时提前复诊。若出现阴道流血突然增多、鲜红血浸湿卫生巾、恶露恶臭、发热、持续腹痛、伤口化脓、排尿困难、下肢单侧肿痛、胸闷气短，或持续悲伤焦虑及伤害自己或婴儿的想法，应立即就医或急诊处理。 | 患者37岁，G2P2，自然分娩+I度裂伤，孕35周早产，合并胎膜早破、GDM、胚胎移植术后、高龄经产妇，新生儿体重2330g（低出生体重），Apgar 9-9-9。早产儿需保暖、监测血糖、黄疸、呼吸暂停，鼓励母乳喂养，必要时添加母乳强化剂。GDM产妇产后仍需监测血糖至少24小时，多数可恢复正常，注意饮食控制；新生儿防低血糖。早产+PPROM注意产褥感染及新生儿感染。经产妇早产，子宫复旧一般良好，仍观察恶露。胚胎移植术后珍贵儿早产，父母焦虑，提供积极支持。高蛋白、高钙饮食。建议间隔1年以上再孕。早产儿随访，产妇42天复查血糖。 |
| **护理计划英文翻译 English translation of care plan** | The patient is 37 years old, G2P2, delivered at 35 weeks by spontaneous vaginal delivery, with an estimated intrapartum blood loss of 220 mL. The newborn was a male infant, weighed 2330 g, and had Apgar scores of 9, 9, 9. The main case-based risks include gestational diabetes mellitus, premature rupture of membranes or infection-related risk, fetal distress or abnormal amniotic fluid findings, advanced maternal age. Within the first 24 hours, assess temperature, pulse, blood pressure, uterine fundal height, uterine tone, vaginal bleeding, lochia color, urinary retention, first voiding, and pain score. During hospitalization and after discharge, continue to observe whether lochia gradually changes from red to lighter color, whether breast engorgement or nipple fissure occurs, and whether sleep and mood remain stable. Diet should include high-quality protein, iron-rich food, vegetables, fruit, and adequate fluids. Avoid blind over-supplementation and prolonged bed rest. Because this was a spontaneous vaginal birth, recovery of any perineal tear or suture site should be observed. Keep the area clean and dry after urination or bowel movement and watch for redness, swelling, bleeding, worsening pain, or abnormal odor. If there is no dizziness or increased bleeding, increase activity gradually and begin gentle pelvic floor exercises under professional guidance. For breastfeeding, encourage early skin-to-skin contact and feeding on demand. Teach correct latch and positioning. For engorgement, apply warmth or gentle massage before feeding. If nipple pain occurs, adjust latch promptly. Localized breast redness, hard painful swelling, chills, or high fever should raise concern for mastitis. For gestational diabetes, avoid refined sugar and heavy oily foods, eat small balanced meals, increase activity gradually, and arrange postpartum glucose or oral glucose tolerance testing as advised. With premature rupture of membranes, group B streptococcus, vaginitis, or suspected intrauterine infection, monitor temperature, lower abdominal tenderness, lochia odor, and drainage from the perineal wound or incision. Return promptly for fever or foul-smelling lochia. Because fetal distress, meconium-stained fluid, or changed Apgar scores were recorded, cooperate with pediatric observation of feeding, skin color, temperature, breathing, and responsiveness. Poor feeding, cyanosis, excessive sleepiness, or abnormal breathing should be reported immediately. Advanced maternal age may slow recovery, so the family should help with nighttime care and support adequate sleep, nutrition, and emotional stability. Before discharge, provide education on medication, wound care, breastfeeding, and contraception. Arrange a postpartum review around 42 days after delivery, or earlier if symptoms occur. Seek urgent medical care for sudden heavy vaginal bleeding, bright red bleeding soaking pads, foul-smelling lochia, fever, persistent abdominal pain, pus from the wound, difficulty urinating, unilateral leg swelling or pain, chest tightness, shortness of breath, or persistent sadness, anxiety, or thoughts of harming herself or the baby. | The patient is a 37-year-old G2P2 multipara with spontaneous delivery and first-degree laceration at 35 weeks. Comorbidities: PPROM, GDM, status post embryo transfer, advanced age. Newborn LBW 2330 g, Apgar 9-9-9. Preterm infant care: warmth, glucose, jaundice, apnea; breastfeeding with possible fortifier. Monitor maternal glucose for 24 hours. Prevent neonatal hypoglycemia. Infection risk – monitor both. Uterine involution generally good. Provide psychological support for precious pregnancy. High-protein, high-calcium diet. Advise >1 year interpregnancy interval. Preterm infant follow-up; maternal glucose check at 42 days. |
| **Clinical appropriateness 临床适当性** | 5 | 3 |
| **Individualization 个体化程度** | 5 | 5 |
| **Comprehensiveness 完整性** | 5 | 4 |
| **Actionability 可操作性** | 5 | 4 |
| **Safety 安全性** | 5 | 3 |
| **Triage appropriateness 分诊/转诊建议适当性** | 5 | 3 |
| **Structure and clarity 结构与清晰度** | 4 | 3 |

**病例 16 / Case 16**

| **项目 Item** | **ChatGPT 5.5** | **DeepSeek V3** |
| --- | --- | --- |
| **去标识化临床病例摘要 De-identified Clinical Case Summary** | 患者32岁，孕产史为G1P1，既往剖宫产0次，孕周40+2，临产类别为自然临产。人工破膜，羊水量约300ml，羊水性质为清。以自然分娩分娩，产时出血约260ml。第一产程8时10分，第二产程1时8分，第三产程0时7分，总产程9时25分，会阴或切口情况为Ⅰ，内缝。合并症及主要风险因素：RH阴性血。新生儿出生体重3600g，Apgar评分1、5、10分钟分别为10、10、10分，分娩结局为活产。 | |
| **临床病例摘要英文翻译 English Translation of De-identified Clinical Case Summary** | Maternal age was 32 years. The obstetric history was G1P1, with 0 previous cesarean deliveries. Gestational age was 40+2 weeks, and the labor onset or induction category was spontaneous labor. The membranes were artificially ruptured; the amniotic fluid volume was approximately 300 mL and its character was clear. The patient had a spontaneous vaginal delivery, with estimated intrapartum blood loss of 260 mL. The first, second, and third stages of labor were 8 h 10 min, 1 h 8 min, and 0 h 7 min, respectively; the total labor duration was 9 h 25 min. A first-degree perineal laceration was repaired with internal sutures. Documented comorbidities and major risk factors were Rh-negative blood type. Neonatal birth weight was 3600 g; Apgar scores at 1, 5, and 10 minutes were 10, 10, 10; and the delivery outcome was live birth. | |
| **模型生成的产褥期护理计划 Model-generated postpartum nursing care plan** | 产妇32岁，G1P1，孕40+2周，经阴道自然分娩分娩，产时出血约260ml，新生儿男婴、体重3600g，Apgar评分10、10、10分。病例提示的重点风险为未记录特殊合并症。产后24小时内重点评估体温、脉搏、血压、宫底高度、宫缩、阴道流血、恶露颜色和尿潴留，记录首次排尿和疼痛评分。产后住院及出院后继续观察恶露是否由红色逐渐转淡，乳房是否胀痛，乳头是否皲裂，睡眠和情绪是否稳定。饮食以优质蛋白、含铁食物、蔬菜水果和足量饮水为主，避免盲目大补和长期卧床。本次为阴道自然分娩，应重点观察会阴裂伤或缝合处恢复。每次大小便后保持局部清洁干燥，观察红肿、渗血、疼痛加重和异常气味。无头晕和出血增多时逐步增加活动，并在医护指导下开始轻柔盆底肌训练。哺乳方面建议尽早母婴皮肤接触和按需哺乳，指导正确含接，乳胀时先热敷或轻柔按摩再哺乳，乳头疼痛时及时调整姿势。若出现乳房局部红肿硬痛、寒战或高热，应警惕乳腺炎。如有Rh阴性血或乙肝相关记录，应核对母婴免疫预防和随访安排，避免漏查抗体、肝功能或新生儿免疫接种记录。出院前应完成用药、伤口、哺乳和避孕宣教，42天左右复查，必要时提前复诊。若出现阴道流血突然增多、鲜红血浸湿卫生巾、恶露恶臭、发热、持续腹痛、伤口化脓、排尿困难、下肢单侧肿痛、胸闷气短，或持续悲伤焦虑及伤害自己或婴儿的想法，应立即就医或急诊处理。 | 患者32岁，初产妇，自然分娩+I度裂伤，合并RH阴性血，新生儿可能Rh阳性。产后72小时内应注射抗D免疫球蛋白（RhoGAM），预防下一胎发生Rh溶血病；确认新生儿血型。Rh阴性血为稀有血型，产时出血260ml，查血红蛋白排除贫血。I度裂伤保持清洁。若新生儿Rh阳性，监测胆红素。母乳喂养无禁忌。解释Rh阴性血的意义及预防措施，减轻焦虑。补充铁剂。产后42天复查血型抗体滴度（如需）；新生儿监测黄疸。 |
| **护理计划英文翻译 English translation of care plan** | The patient is 32 years old, G1P1, delivered at 40+2 weeks by spontaneous vaginal delivery, with an estimated intrapartum blood loss of 260 mL. The newborn was a male infant, weighed 3600 g, and had Apgar scores of 10, 10, 10. The main case-based risks include no special comorbidity was recorded. Within the first 24 hours, assess temperature, pulse, blood pressure, uterine fundal height, uterine tone, vaginal bleeding, lochia color, urinary retention, first voiding, and pain score. During hospitalization and after discharge, continue to observe whether lochia gradually changes from red to lighter color, whether breast engorgement or nipple fissure occurs, and whether sleep and mood remain stable. Diet should include high-quality protein, iron-rich food, vegetables, fruit, and adequate fluids. Avoid blind over-supplementation and prolonged bed rest. Because this was a spontaneous vaginal birth, recovery of any perineal tear or suture site should be observed. Keep the area clean and dry after urination or bowel movement and watch for redness, swelling, bleeding, worsening pain, or abnormal odor. If there is no dizziness or increased bleeding, increase activity gradually and begin gentle pelvic floor exercises under professional guidance. For breastfeeding, encourage early skin-to-skin contact and feeding on demand. Teach correct latch and positioning. For engorgement, apply warmth or gentle massage before feeding. If nipple pain occurs, adjust latch promptly. Localized breast redness, hard painful swelling, chills, or high fever should raise concern for mastitis. For Rh-negative blood type or hepatitis B related records, confirm maternal and newborn immunoprophylaxis and follow-up, and avoid missing antibody, liver function, or infant vaccination documentation. Before discharge, provide education on medication, wound care, breastfeeding, and contraception. Arrange a postpartum review around 42 days after delivery, or earlier if symptoms occur. Seek urgent medical care for sudden heavy vaginal bleeding, bright red bleeding soaking pads, foul-smelling lochia, fever, persistent abdominal pain, pus from the wound, difficulty urinating, unilateral leg swelling or pain, chest tightness, shortness of breath, or persistent sadness, anxiety, or thoughts of harming herself or the baby. | The patient is a 32-year-old primipara with spontaneous delivery and first-degree laceration. Comorbidity: Rh-negative blood type. Newborn likely Rh-positive. Administer anti-D immunoglobulin (RhoGAM) within 72 hours to prevent hemolytic disease in next pregnancy. Confirm newborn blood type. Check hemoglobin due to rare blood type (blood loss 260 mL). Perineal care. Monitor newborn bilirubin if Rh-positive. Breastfeeding safe. Explain Rh significance and prevention. Iron supplementation. Check antibody titers at 42 days; monitor newborn jaundice. |
| **Clinical appropriateness 临床适当性** | 4 | 4 |
| **Individualization 个体化程度** | 3 | 4 |
| **Comprehensiveness 完整性** | 4 | 4 |
| **Actionability 可操作性** | 4 | 4 |
| **Safety 安全性** | 5 | 4 |
| **Triage appropriateness 分诊/转诊建议适当性** | 5 | 3 |
| **Structure and clarity 结构与清晰度** | 4 | 4 |

**病例 17 / Case 17**

| **项目 Item** | **ChatGPT 5.5** | **DeepSeek V3** |
| --- | --- | --- |
| **去标识化临床病例摘要 De-identified Clinical Case Summary** | 患者32岁，孕产史为G1P1，既往剖宫产0次，孕周40+1，临产类别为自然临产。自然破膜，羊水量约300ml，羊水性质为清。以产钳分娩，产时出血约260ml。第一产程5时50分，第二产程1时3分，第三产程0时27分，总产程7时20分，会阴或切口情况为侧切，内缝。合并症及主要风险因素：胎膜早破；B族链球菌感染；绒毛膜羊膜炎？胎儿窘迫。新生儿出生体重2740g，Apgar评分1、5、10分钟分别为10、10、10分，分娩结局为活产。 | |
| **临床病例摘要英文翻译 English Translation of De-identified Clinical Case Summary** | Maternal age was 32 years. The obstetric history was G1P1, with 0 previous cesarean deliveries. Gestational age was 40+1 weeks, and the labor onset or induction category was spontaneous labor. The membranes ruptured spontaneously; the amniotic fluid volume was approximately 300 mL and its character was clear. The patient had a forceps-assisted vaginal delivery, with estimated intrapartum blood loss of 260 mL. The first, second, and third stages of labor were 5 h 50 min, 1 h 3 min, and 0 h 27 min, respectively; the total labor duration was 7 h 20 min. An episiotomy was repaired with internal sutures. Documented comorbidities and major risk factors were premature rupture of membranes; group B Streptococcus infection; suspected chorioamnionitis; fetal distress. Neonatal birth weight was 2740 g; Apgar scores at 1, 5, and 10 minutes were 10, 10, 10; and the delivery outcome was live birth. | |
| **模型生成的产褥期护理计划 Model-generated postpartum nursing care plan** | 产妇32岁，G1P1，孕40+1周，经产钳助产阴道分娩分娩，产时出血约260ml，新生儿女婴、体重2740g，Apgar评分10、10、10分。病例提示的重点风险为胎膜早破或感染相关风险、胎儿窘迫或羊水异常记录。产后24小时内重点评估体温、脉搏、血压、宫底高度、宫缩、阴道流血、恶露颜色和尿潴留，记录首次排尿和疼痛评分。产后住院及出院后继续观察恶露是否由红色逐渐转淡，乳房是否胀痛，乳头是否皲裂，睡眠和情绪是否稳定。饮食以优质蛋白、含铁食物、蔬菜水果和足量饮水为主，避免盲目大补和长期卧床。本次为产钳助产阴道分娩，会阴伤口、盆底和排尿功能是护理重点。每次大小便后用温水从前向后清洁会阴并轻轻擦干，观察侧切或裂伤处肿胀、硬结、渗血、分泌物和异味。疼痛明显时可侧卧休息，避免久蹲、负重和过早剧烈活动。哺乳方面建议尽早母婴皮肤接触和按需哺乳，指导正确含接，乳胀时先热敷或轻柔按摩再哺乳，乳头疼痛时及时调整姿势。若出现乳房局部红肿硬痛、寒战或高热，应警惕乳腺炎。存在胎膜早破、B族链球菌感染、阴道炎或宫内感染疑虑时，应加强体温、下腹压痛、恶露气味、会阴或切口渗液观察，发热或恶露恶臭时及时复诊。因有胎儿窘迫、羊水污染或新生儿评分变化记录，母婴同室期间应配合观察新生儿吃奶、肤色、体温、呼吸和反应，发现吃奶差、发绀、嗜睡或呼吸异常应立即告知儿科医生。出院前应完成用药、伤口、哺乳和避孕宣教，42天左右复查，必要时提前复诊。若出现阴道流血突然增多、鲜红血浸湿卫生巾、恶露恶臭、发热、持续腹痛、伤口化脓、排尿困难、下肢单侧肿痛、胸闷气短，或持续悲伤焦虑及伤害自己或婴儿的想法，应立即就医或急诊处理。 | 患者32岁，初产妇，产钳助产+侧切，合并胎膜早破、GBS感染、可疑绒毛膜羊膜炎、胎儿窘迫。需静脉或口服抗生素治疗GBS及可疑绒毛膜羊膜炎。每日测体温4次，观察腹痛、恶露。新生儿需密切监测GBS早发型败血症。会阴侧切伤口每日消毒2次，注意红肿、化脓。胎儿窘迫新生儿Apgar 10分，但仍需观察神经系统。产时出血260ml，注意晚期出血。GBS不影响母乳喂养，但注意乳头清洁。提供信息支持减轻焦虑。高蛋白饮食。产后1周复查感染指标；新生儿随访。 |
| **护理计划英文翻译 English translation of care plan** | The patient is 32 years old, G1P1, delivered at 40+1 weeks by forceps-assisted vaginal delivery, with an estimated intrapartum blood loss of 260 mL. The newborn was a female infant, weighed 2740 g, and had Apgar scores of 10, 10, 10. The main case-based risks include premature rupture of membranes or infection-related risk, fetal distress or abnormal amniotic fluid findings. Within the first 24 hours, assess temperature, pulse, blood pressure, uterine fundal height, uterine tone, vaginal bleeding, lochia color, urinary retention, first voiding, and pain score. During hospitalization and after discharge, continue to observe whether lochia gradually changes from red to lighter color, whether breast engorgement or nipple fissure occurs, and whether sleep and mood remain stable. Diet should include high-quality protein, iron-rich food, vegetables, fruit, and adequate fluids. Avoid blind over-supplementation and prolonged bed rest. Because this was a forceps-assisted vaginal delivery, perineal wound care, pelvic floor recovery, and urinary function are priorities. Rinse the perineum from front to back with warm water after urination or bowel movement and pat dry. Observe the episiotomy or tear for swelling, induration, bleeding, discharge, and odor. Rest in a lateral position if pain is marked, and avoid prolonged squatting, heavy lifting, and early vigorous activity. For breastfeeding, encourage early skin-to-skin contact and feeding on demand. Teach correct latch and positioning. For engorgement, apply warmth or gentle massage before feeding. If nipple pain occurs, adjust latch promptly. Localized breast redness, hard painful swelling, chills, or high fever should raise concern for mastitis. With premature rupture of membranes, group B streptococcus, vaginitis, or suspected intrauterine infection, monitor temperature, lower abdominal tenderness, lochia odor, and drainage from the perineal wound or incision. Return promptly for fever or foul-smelling lochia. Because fetal distress, meconium-stained fluid, or changed Apgar scores were recorded, cooperate with pediatric observation of feeding, skin color, temperature, breathing, and responsiveness. Poor feeding, cyanosis, excessive sleepiness, or abnormal breathing should be reported immediately. Before discharge, provide education on medication, wound care, breastfeeding, and contraception. Arrange a postpartum review around 42 days after delivery, or earlier if symptoms occur. Seek urgent medical care for sudden heavy vaginal bleeding, bright red bleeding soaking pads, foul-smelling lochia, fever, persistent abdominal pain, pus from the wound, difficulty urinating, unilateral leg swelling or pain, chest tightness, shortness of breath, or persistent sadness, anxiety, or thoughts of harming herself or the baby. | The patient is a 32-year-old primipara with forceps delivery and episiotomy. Comorbidities: PPROM, GBS infection, suspected chorioamnionitis, fetal distress. IV or oral antibiotics for GBS and suspected chorio. Check temperature 4 times daily; monitor abdominal pain, lochia. Observe newborn for early-onset GBS sepsis. Clean episiotomy twice daily; monitor for redness, swelling, purulence. Newborn Apgar 10 – still monitor neurology. Blood loss 260 mL – watch for late PPH. Breastfeeding safe. Provide reassurance. High-protein diet. Check infection markers at 1 week; newborn follow-up. |
| **Clinical appropriateness 临床适当性** | 5 | 4 |
| **Individualization 个体化程度** | 4 | 5 |
| **Comprehensiveness 完整性** | 5 | 4 |
| **Actionability 可操作性** | 4 | 4 |
| **Safety 安全性** | 5 | 4 |
| **Triage appropriateness 分诊/转诊建议适当性** | 5 | 4 |
| **Structure and clarity 结构与清晰度** | 4 | 4 |

**病例 18 / Case 18**

| **项目 Item** | **ChatGPT 5.5** | **DeepSeek V3** |
| --- | --- | --- |
| **去标识化临床病例摘要 De-identified Clinical Case Summary** | 患者36岁，孕产史为G1P1，既往剖宫产0次，孕周40，临产类别为普贝生。自然破膜，羊水量约300ml，羊水性质为清。以自然分娩分娩，产时出血约390ml。第一产程2时40分，第二产程0时34分，第三产程0时6分，总产程3时20分，会阴或切口情况为Ⅰ，内缝。合并症及主要风险因素：胎儿窘迫？妊娠期糖尿病；高龄初孕妇的监督；妊娠合并甲状腺功能减退；子宫腺肌瘤？胚胎移植术后；妊娠合并细菌性阴道炎（好转）；肝功能检查的异常结果；转氨酶升高；胎膜早破。新生儿出生体重2790g，Apgar评分1、5、10分钟分别为10、10、10分，分娩结局为活产。 | |
| **临床病例摘要英文翻译 English Translation of De-identified Clinical Case Summary** | Maternal age was 36 years. The obstetric history was G1P1, with 0 previous cesarean deliveries. Gestational age was 40 weeks, and the labor onset or induction category was vaginal dinoprostone (Propess) induction. The membranes ruptured spontaneously; the amniotic fluid volume was approximately 300 mL and its character was clear. The patient had a spontaneous vaginal delivery, with estimated intrapartum blood loss of 390 mL. The first, second, and third stages of labor were 2 h 40 min, 0 h 34 min, and 0 h 6 min, respectively; the total labor duration was 3 h 20 min. A first-degree perineal laceration was repaired with internal sutures. Documented comorbidities and major risk factors were suspected fetal distress; gestational diabetes mellitus; advanced maternal age in a primigravida; hypothyroidism during pregnancy; suspected uterine adenomyoma; status post embryo transfer; bacterial vaginosis during pregnancy (improved); abnormal liver function test results; elevated transaminase levels; premature rupture of membranes. Neonatal birth weight was 2790 g; Apgar scores at 1, 5, and 10 minutes were 10, 10, 10; and the delivery outcome was live birth. | |
| **模型生成的产褥期护理计划 Model-generated postpartum nursing care plan** | 产妇36岁，G1P1，孕40周，经阴道自然分娩分娩，产时出血约390ml，新生儿男婴、体重2790g，Apgar评分10、10、10分。病例提示的重点风险为妊娠期糖尿病、胎膜早破或感染相关风险、甲状腺功能异常、肝功能异常或胆汁淤积史、胎儿窘迫或羊水异常记录。产后24小时内重点评估体温、脉搏、血压、宫底高度、宫缩、阴道流血、恶露颜色和尿潴留，记录首次排尿和疼痛评分。产后住院及出院后继续观察恶露是否由红色逐渐转淡，乳房是否胀痛，乳头是否皲裂，睡眠和情绪是否稳定。饮食以优质蛋白、含铁食物、蔬菜水果和足量饮水为主，避免盲目大补和长期卧床。本次为阴道自然分娩，应重点观察会阴裂伤或缝合处恢复。每次大小便后保持局部清洁干燥，观察红肿、渗血、疼痛加重和异常气味。无头晕和出血增多时逐步增加活动，并在医护指导下开始轻柔盆底肌训练。哺乳方面建议尽早母婴皮肤接触和按需哺乳，指导正确含接，乳胀时先热敷或轻柔按摩再哺乳，乳头疼痛时及时调整姿势。若出现乳房局部红肿硬痛、寒战或高热，应警惕乳腺炎。有妊娠期糖尿病者，产后饮食应控制精制糖和高油食物，少量多餐，结合恢复情况逐步活动，并按医嘱在产后复查血糖或糖耐量。合并甲状腺疾病者，应按内分泌医嘱继续或调整用药，不自行停药，产后复查甲状腺功能，观察心悸、怕冷、乏力、出汗和情绪异常。存在胎膜早破、B族链球菌感染、阴道炎或宫内感染疑虑时，应加强体温、下腹压痛、恶露气味、会阴或切口渗液观察，发热或恶露恶臭时及时复诊。因有胎儿窘迫、羊水污染或新生儿评分变化记录，母婴同室期间应配合观察新生儿吃奶、肤色、体温、呼吸和反应，发现吃奶差、发绀、嗜睡或呼吸异常应立即告知儿科医生。有胆汁淤积或肝功能异常者，产后按医嘱复查肝功能和胆汁酸，若皮肤瘙痒、黄疸、右上腹痛或尿色明显加深，应尽快就诊。出院前应完成用药、伤口、哺乳和避孕宣教，42天左右复查，必要时提前复诊。若出现阴道流血突然增多、鲜红血浸湿卫生巾、恶露恶臭、发热、持续腹痛、伤口化脓、排尿困难、下肢单侧肿痛、胸闷气短，或持续悲伤焦虑及伤害自己或婴儿的想法，应立即就医或急诊处理。 | 患者36岁，高龄初产妇，胚胎移植术后，自然分娩+I度裂伤，合并多种复杂合并症：可疑胎儿窘迫、GDM、甲减、子宫腺肌瘤、细菌性阴道炎（好转）、肝功能异常（转氨酶升高）、胎膜早破、脐带绕颈1周、脐带捻转50周。产后需复查肝功能，避免肝损药物，排查ICP或HELLP。继续左甲状腺素，6周复查甲功。GDM产后监测血糖，饮食控制。细菌性阴道炎+PPROM+子宫腺肌瘤，注意产褥感染。子宫腺肌瘤可能致痛经加重或异常出血。新生儿虽Apgar正常，但脐带异常需观察。心理支持极重要。产后2周复查肝功、血糖；42天全面复查。 |
| **护理计划英文翻译 English translation of care plan** | The patient is 36 years old, G1P1, delivered at 40 weeks by spontaneous vaginal delivery, with an estimated intrapartum blood loss of 390 mL. The newborn was a male infant, weighed 2790 g, and had Apgar scores of 10, 10, 10. The main case-based risks include gestational diabetes mellitus, premature rupture of membranes or infection-related risk, thyroid dysfunction, abnormal liver function or a history of cholestasis, fetal distress or abnormal amniotic fluid findings. Within the first 24 hours, assess temperature, pulse, blood pressure, uterine fundal height, uterine tone, vaginal bleeding, lochia color, urinary retention, first voiding, and pain score. During hospitalization and after discharge, continue to observe whether lochia gradually changes from red to lighter color, whether breast engorgement or nipple fissure occurs, and whether sleep and mood remain stable. Diet should include high-quality protein, iron-rich food, vegetables, fruit, and adequate fluids. Avoid blind over-supplementation and prolonged bed rest. Because this was a spontaneous vaginal birth, recovery of any perineal tear or suture site should be observed. Keep the area clean and dry after urination or bowel movement and watch for redness, swelling, bleeding, worsening pain, or abnormal odor. If there is no dizziness or increased bleeding, increase activity gradually and begin gentle pelvic floor exercises under professional guidance. For breastfeeding, encourage early skin-to-skin contact and feeding on demand. Teach correct latch and positioning. For engorgement, apply warmth or gentle massage before feeding. If nipple pain occurs, adjust latch promptly. Localized breast redness, hard painful swelling, chills, or high fever should raise concern for mastitis. For gestational diabetes, avoid refined sugar and heavy oily foods, eat small balanced meals, increase activity gradually, and arrange postpartum glucose or oral glucose tolerance testing as advised. For thyroid disease, continue or adjust medication only under endocrine guidance, recheck thyroid function after delivery, and watch for palpitations, cold intolerance, fatigue, sweating, or abnormal mood changes. With premature rupture of membranes, group B streptococcus, vaginitis, or suspected intrauterine infection, monitor temperature, lower abdominal tenderness, lochia odor, and drainage from the perineal wound or incision. Return promptly for fever or foul-smelling lochia. Because fetal distress, meconium-stained fluid, or changed Apgar scores were recorded, cooperate with pediatric observation of feeding, skin color, temperature, breathing, and responsiveness. Poor feeding, cyanosis, excessive sleepiness, or abnormal breathing should be reported immediately. For cholestasis or abnormal liver function, recheck liver function and bile acids as advised. Seek care for itching, jaundice, right upper abdominal pain, or dark urine. Before discharge, provide education on medication, wound care, breastfeeding, and contraception. Arrange a postpartum review around 42 days after delivery, or earlier if symptoms occur. Seek urgent medical care for sudden heavy vaginal bleeding, bright red bleeding soaking pads, foul-smelling lochia, fever, persistent abdominal pain, pus from the wound, difficulty urinating, unilateral leg swelling or pain, chest tightness, shortness of breath, or persistent sadness, anxiety, or thoughts of harming herself or the baby. | The patient is a 36-year-old advanced maternal age primigravida after embryo transfer. Spontaneous delivery with first-degree laceration. Complex comorbidities: suspected fetal distress, GDM, hypothyroidism, uterine adenomyoma, bacterial vaginosis (improved), elevated LFTs, PPROM, nuchal cord 1 loop, cord twisting 50 loops. Repeat LFTs, avoid hepatotoxic drugs, rule out ICP/HELLP. Continue levothyroxine, retest at 6 weeks. Monitor postpartum glucose. Infection risk from BV+PPROM+adenomyoma – monitor temperature and lochia. Adenomyoma may worsen dysmenorrhea. Newborn observation for cord anomalies. Critical psychological support. Check LFTs and glucose at 2 weeks; comprehensive exam at 42 days. |
| **Clinical appropriateness 临床适当性** | 5 | 4 |
| **Individualization 个体化程度** | 5 | 5 |
| **Comprehensiveness 完整性** | 5 | 4 |
| **Actionability 可操作性** | 5 | 4 |
| **Safety 安全性** | 5 | 4 |
| **Triage appropriateness 分诊/转诊建议适当性** | 5 | 4 |
| **Structure and clarity 结构与清晰度** | 4 | 3 |

**病例 19 / Case 19**

| **项目 Item** | **ChatGPT 5.5** | **DeepSeek V3** |
| --- | --- | --- |
| **去标识化临床病例摘要 De-identified Clinical Case Summary** | 患者40岁，孕产史为G2P2，既往剖宫产0次，孕周39+2，临产类别为阴道放置米索。自然破膜，羊水量约500ml，羊水性质为清。以自然分娩分娩，产时出血约220ml。第一产程2时3分，第二产程0时15分，第三产程0时7分，总产程2时25分，会阴或切口情况为Ⅰ，内缝。合并症及主要风险因素：胎膜早破；胎儿窘迫？；妊娠期糖尿病；高龄经产妇妊娠监督；右侧卵巢囊肿？。新生儿出生体重4280g，Apgar评分1、5、10分钟分别为10、10、10分，分娩结局为活产。 | |
| **临床病例摘要英文翻译 English Translation of De-identified Clinical Case Summary** | Maternal age was 40 years. The obstetric history was G2P2, with 0 previous cesarean deliveries. Gestational age was 39+2 weeks, and the labor onset or induction category was vaginal misoprostol induction. The membranes ruptured spontaneously; the amniotic fluid volume was approximately 500 mL and its character was clear. The patient had a spontaneous vaginal delivery, with estimated intrapartum blood loss of 220 mL. The first, second, and third stages of labor were 2 h 3 min, 0 h 15 min, and 0 h 7 min, respectively; the total labor duration was 2 h 25 min. A first-degree perineal laceration was repaired with internal sutures. Documented comorbidities and major risk factors were premature rupture of membranes; suspected fetal distress; gestational diabetes mellitus; advanced maternal age in a multipara; suspected right ovarian cyst. Neonatal birth weight was 4280 g; Apgar scores at 1, 5, and 10 minutes were 10, 10, 10; and the delivery outcome was live birth. | |
| **模型生成的产褥期护理计划 Model-generated postpartum nursing care plan** | 产妇40岁，G2P2，孕39+2周，经阴道自然分娩分娩，产时出血约220ml，新生儿女婴、体重4280g，Apgar评分10、10、10分。病例提示的重点风险为妊娠期糖尿病、胎膜早破或感染相关风险、胎儿窘迫或羊水异常记录、高龄妊娠因素。产后24小时内重点评估体温、脉搏、血压、宫底高度、宫缩、阴道流血、恶露颜色和尿潴留，记录首次排尿和疼痛评分。产后住院及出院后继续观察恶露是否由红色逐渐转淡，乳房是否胀痛，乳头是否皲裂，睡眠和情绪是否稳定。饮食以优质蛋白、含铁食物、蔬菜水果和足量饮水为主，避免盲目大补和长期卧床。本次为阴道自然分娩，应重点观察会阴裂伤或缝合处恢复。每次大小便后保持局部清洁干燥，观察红肿、渗血、疼痛加重和异常气味。无头晕和出血增多时逐步增加活动，并在医护指导下开始轻柔盆底肌训练。哺乳方面建议尽早母婴皮肤接触和按需哺乳，指导正确含接，乳胀时先热敷或轻柔按摩再哺乳，乳头疼痛时及时调整姿势。若出现乳房局部红肿硬痛、寒战或高热，应警惕乳腺炎。有妊娠期糖尿病者，产后饮食应控制精制糖和高油食物，少量多餐，结合恢复情况逐步活动，并按医嘱在产后复查血糖或糖耐量。存在胎膜早破、B族链球菌感染、阴道炎或宫内感染疑虑时，应加强体温、下腹压痛、恶露气味、会阴或切口渗液观察，发热或恶露恶臭时及时复诊。因有胎儿窘迫、羊水污染或新生儿评分变化记录，母婴同室期间应配合观察新生儿吃奶、肤色、体温、呼吸和反应，发现吃奶差、发绀、嗜睡或呼吸异常应立即告知儿科医生。合并子宫肌瘤、卵巢囊肿或多囊卵巢相关情况时，产后复查时建议结合妇科超声评估，若腹痛、包块感或异常出血加重，应提前就诊。出院前应完成用药、伤口、哺乳和避孕宣教，42天左右复查，必要时提前复诊。若出现阴道流血突然增多、鲜红血浸湿卫生巾、恶露恶臭、发热、持续腹痛、伤口化脓、排尿困难、下肢单侧肿痛、胸闷气短，或持续悲伤焦虑及伤害自己或婴儿的想法，应立即就医或急诊处理。 | 患者40岁，高龄经产妇，自然分娩+I度裂伤，合并胎膜早破、可疑胎儿窘迫、GDM、右侧卵巢囊肿可疑，新生儿巨大儿（4280g）。巨大儿产后需加强宫底按摩预防宫缩乏力性产后出血；新生儿出生后2小时内监测血糖防低血糖。GDM产妇监测血糖。胎膜早破注意感染。卵巢囊肿产后6周B超复查，如急性腹痛、发热警惕囊肿扭转。I度裂伤温水坐浴。巨大儿吸吮力强，鼓励母乳。提供心理支持。产后6周复查B超及血糖；新生儿监测生长发育。 |
| **护理计划英文翻译 English translation of care plan** | The patient is 40 years old, G2P2, delivered at 39+2 weeks by spontaneous vaginal delivery, with an estimated intrapartum blood loss of 220 mL. The newborn was a female infant, weighed 4280 g, and had Apgar scores of 10, 10, 10. The main case-based risks include gestational diabetes mellitus, premature rupture of membranes or infection-related risk, fetal distress or abnormal amniotic fluid findings, advanced maternal age. Within the first 24 hours, assess temperature, pulse, blood pressure, uterine fundal height, uterine tone, vaginal bleeding, lochia color, urinary retention, first voiding, and pain score. During hospitalization and after discharge, continue to observe whether lochia gradually changes from red to lighter color, whether breast engorgement or nipple fissure occurs, and whether sleep and mood remain stable. Diet should include high-quality protein, iron-rich food, vegetables, fruit, and adequate fluids. Avoid blind over-supplementation and prolonged bed rest. Because this was a spontaneous vaginal birth, recovery of any perineal tear or suture site should be observed. Keep the area clean and dry after urination or bowel movement and watch for redness, swelling, bleeding, worsening pain, or abnormal odor. If there is no dizziness or increased bleeding, increase activity gradually and begin gentle pelvic floor exercises under professional guidance. For breastfeeding, encourage early skin-to-skin contact and feeding on demand. Teach correct latch and positioning. For engorgement, apply warmth or gentle massage before feeding. If nipple pain occurs, adjust latch promptly. Localized breast redness, hard painful swelling, chills, or high fever should raise concern for mastitis. For gestational diabetes, avoid refined sugar and heavy oily foods, eat small balanced meals, increase activity gradually, and arrange postpartum glucose or oral glucose tolerance testing as advised. With premature rupture of membranes, group B streptococcus, vaginitis, or suspected intrauterine infection, monitor temperature, lower abdominal tenderness, lochia odor, and drainage from the perineal wound or incision. Return promptly for fever or foul-smelling lochia. Because fetal distress, meconium-stained fluid, or changed Apgar scores were recorded, cooperate with pediatric observation of feeding, skin color, temperature, breathing, and responsiveness. Poor feeding, cyanosis, excessive sleepiness, or abnormal breathing should be reported immediately. For uterine fibroids, ovarian cysts, or polycystic ovary related findings, pelvic ultrasound can be considered at postpartum review. Earlier assessment is needed for worsening abdominal pain, mass sensation, or abnormal bleeding. Before discharge, provide education on medication, wound care, breastfeeding, and contraception. Arrange a postpartum review around 42 days after delivery, or earlier if symptoms occur. Seek urgent medical care for sudden heavy vaginal bleeding, bright red bleeding soaking pads, foul-smelling lochia, fever, persistent abdominal pain, pus from the wound, difficulty urinating, unilateral leg swelling or pain, chest tightness, shortness of breath, or persistent sadness, anxiety, or thoughts of harming herself or the baby. | The patient is a 40-year-old advanced maternal age multipara with spontaneous delivery and first-degree laceration. Comorbidities: PPROM, suspected fetal distress, GDM, suspected right ovarian cyst. Newborn macrosomia (4280 g). Aggressive fundal massage to prevent atonic PPH. Monitor newborn glucose within 2 hours. Monitor maternal glucose. Infection risk from PPROM. Ovarian cyst – repeat ultrasound at 6 weeks; if acute pain/fever, suspect torsion. Warm sitz baths. Macrosomic infant feeds well – encourage breastfeeding. Provide psychological support. Follow-up: ultrasound and glucose at 6 weeks; newborn growth monitoring. |
| **Clinical appropriateness 临床适当性** | 5 | 4 |
| **Individualization 个体化程度** | 5 | 5 |
| **Comprehensiveness 完整性** | 5 | 4 |
| **Actionability 可操作性** | 5 | 4 |
| **Safety 安全性** | 5 | 4 |
| **Triage appropriateness 分诊/转诊建议适当性** | 5 | 4 |
| **Structure and clarity 结构与清晰度** | 4 | 3 |

**病例 20 / Case 20**

| **项目 Item** | **ChatGPT 5.5** | **DeepSeek V3** |
| --- | --- | --- |
| **去标识化临床病例摘要 De-identified Clinical Case Summary** | 患者30岁，孕产史为G1P1，既往剖宫产0次，孕周40+6，临产类别为自然临产。自然破膜，羊水量约300ml，羊水性质为清。以自然分娩分娩，产时出血约350ml。第一产程7时40分，第二产程1时40分，第三产程0时5分，总产程9时25分，会阴或切口情况为Ⅰ，内缝。合并症及主要风险因素：妊娠合并子宫肌瘤。新生儿出生体重3450g，Apgar评分1、5、10分钟分别为10、10、10分，分娩结局为活产。 | |
| **临床病例摘要英文翻译 English Translation of De-identified Clinical Case Summary** | Maternal age was 30 years. The obstetric history was G1P1, with 0 previous cesarean deliveries. Gestational age was 40+6 weeks, and the labor onset or induction category was spontaneous labor. The membranes ruptured spontaneously; the amniotic fluid volume was approximately 300 mL and its character was clear. The patient had a spontaneous vaginal delivery, with estimated intrapartum blood loss of 350 mL. The first, second, and third stages of labor were 7 h 40 min, 1 h 40 min, and 0 h 5 min, respectively; the total labor duration was 9 h 25 min. A first-degree perineal laceration was repaired with internal sutures. Documented comorbidities and major risk factors were uterine fibroid during pregnancy. Neonatal birth weight was 3450 g; Apgar scores at 1, 5, and 10 minutes were 10, 10, 10; and the delivery outcome was live birth. | |
| **模型生成的产褥期护理计划 Model-generated postpartum nursing care plan** | 产妇30岁，G1P1，孕40+6周，经阴道自然分娩分娩，产时出血约350ml，新生儿男婴、体重3450g，Apgar评分10、10、10分。病例提示的重点风险为未记录特殊合并症。产后24小时内重点评估体温、脉搏、血压、宫底高度、宫缩、阴道流血、恶露颜色和尿潴留，记录首次排尿和疼痛评分。产后住院及出院后继续观察恶露是否由红色逐渐转淡，乳房是否胀痛，乳头是否皲裂，睡眠和情绪是否稳定。饮食以优质蛋白、含铁食物、蔬菜水果和足量饮水为主，避免盲目大补和长期卧床。本次为阴道自然分娩，应重点观察会阴裂伤或缝合处恢复。每次大小便后保持局部清洁干燥，观察红肿、渗血、疼痛加重和异常气味。无头晕和出血增多时逐步增加活动，并在医护指导下开始轻柔盆底肌训练。哺乳方面建议尽早母婴皮肤接触和按需哺乳，指导正确含接，乳胀时先热敷或轻柔按摩再哺乳，乳头疼痛时及时调整姿势。若出现乳房局部红肿硬痛、寒战或高热，应警惕乳腺炎。合并子宫肌瘤、卵巢囊肿或多囊卵巢相关情况时，产后复查时建议结合妇科超声评估，若腹痛、包块感或异常出血加重，应提前就诊。出院前应完成用药、伤口、哺乳和避孕宣教，42天左右复查，必要时提前复诊。若出现阴道流血突然增多、鲜红血浸湿卫生巾、恶露恶臭、发热、持续腹痛、伤口化脓、排尿困难、下肢单侧肿痛、胸闷气短，或持续悲伤焦虑及伤害自己或婴儿的想法，应立即就医或急诊处理。 | 患者30岁，初产妇，自然分娩+I度裂伤，合并妊娠合并子宫肌瘤（未明确位置），产时出血350ml。子宫肌瘤可能影响子宫复旧，导致产后出血或恶露延长，需密切观察恶露量、宫底高度；若出血增多或腹痛剧烈，行B超检查。出血350ml接近阈值，注意晚期产后出血，按需按摩宫底。I度裂伤温水坐浴。母乳喂养无禁忌。肌瘤可能在产后变性（红色变性）引起腹痛，如发热、剧痛需就医。解释多数肌瘤不影响产褥恢复。产后6周B超复查肌瘤大小；42天常规复查。 |
| **护理计划英文翻译 English translation of care plan** | The patient is 30 years old, G1P1, delivered at 40+6 weeks by spontaneous vaginal delivery, with an estimated intrapartum blood loss of 350 mL. The newborn was a male infant, weighed 3450 g, and had Apgar scores of 10, 10, 10. The main case-based risks include no special comorbidity was recorded. Within the first 24 hours, assess temperature, pulse, blood pressure, uterine fundal height, uterine tone, vaginal bleeding, lochia color, urinary retention, first voiding, and pain score. During hospitalization and after discharge, continue to observe whether lochia gradually changes from red to lighter color, whether breast engorgement or nipple fissure occurs, and whether sleep and mood remain stable. Diet should include high-quality protein, iron-rich food, vegetables, fruit, and adequate fluids. Avoid blind over-supplementation and prolonged bed rest. Because this was a spontaneous vaginal birth, recovery of any perineal tear or suture site should be observed. Keep the area clean and dry after urination or bowel movement and watch for redness, swelling, bleeding, worsening pain, or abnormal odor. If there is no dizziness or increased bleeding, increase activity gradually and begin gentle pelvic floor exercises under professional guidance. For breastfeeding, encourage early skin-to-skin contact and feeding on demand. Teach correct latch and positioning. For engorgement, apply warmth or gentle massage before feeding. If nipple pain occurs, adjust latch promptly. Localized breast redness, hard painful swelling, chills, or high fever should raise concern for mastitis. For uterine fibroids, ovarian cysts, or polycystic ovary related findings, pelvic ultrasound can be considered at postpartum review. Earlier assessment is needed for worsening abdominal pain, mass sensation, or abnormal bleeding. Before discharge, provide education on medication, wound care, breastfeeding, and contraception. Arrange a postpartum review around 42 days after delivery, or earlier if symptoms occur. Seek urgent medical care for sudden heavy vaginal bleeding, bright red bleeding soaking pads, foul-smelling lochia, fever, persistent abdominal pain, pus from the wound, difficulty urinating, unilateral leg swelling or pain, chest tightness, shortness of breath, or persistent sadness, anxiety, or thoughts of harming herself or the baby. | The patient is a 30-year-old primipara with spontaneous delivery and first-degree laceration. Comorbidity: uterine fibroid during pregnancy (location unspecified). Blood loss 350 mL. Fibroids may impair uterine involution – closely monitor lochia volume and fundal height; perform ultrasound if bleeding increases or severe pain occurs. Watch for late PPH. Warm sitz baths. Breastfeeding safe. Red degeneration may cause abdominal pain and fever – seek care if occurs. Reassure that most fibroids do not affect puerperium. Follow-up ultrasound at 6 weeks; routine check at 42 days. |
| **Clinical appropriateness 临床适当性** | 4 | 4 |
| **Individualization 个体化程度** | 3 | 4 |
| **Comprehensiveness 完整性** | 4 | 4 |
| **Actionability 可操作性** | 4 | 4 |
| **Safety 安全性** | 5 | 4 |
| **Triage appropriateness 分诊/转诊建议适当性** | 5 | 4 |
| **Structure and clarity 结构与清晰度** | 4 | 4 |

**病例 21 / Case 21**

| **项目 Item** | **ChatGPT 5.5** | **DeepSeek V3** |
| --- | --- | --- |
| **去标识化临床病例摘要 De-identified Clinical Case Summary** | 患者37岁，孕产史为G3P2，既往剖宫产0次，孕周38+4，临产类别为普贝生。自然破膜，羊水量约300ml，羊水性质为血性。以自然分娩分娩，产时出血约250ml。第一产程1时45分，第二产程0时6分，第三产程0时9分，总产程2时0分，会阴或切口情况为Ⅰ，内缝。合并症及主要风险因素：慢性高血压并发子痫前期；妊娠合并子宫肌瘤。新生儿出生体重3010g，Apgar评分1、5、10分钟分别为10、10、10分，分娩结局为活产。 | |
| **临床病例摘要英文翻译 English Translation of De-identified Clinical Case Summary** | Maternal age was 37 years. The obstetric history was G3P2, with 0 previous cesarean deliveries. Gestational age was 38+4 weeks, and the labor onset or induction category was vaginal dinoprostone (Propess) induction. The membranes ruptured spontaneously; the amniotic fluid volume was approximately 300 mL and its character was blood-stained. The patient had a spontaneous vaginal delivery, with estimated intrapartum blood loss of 250 mL. The first, second, and third stages of labor were 1 h 45 min, 0 h 6 min, and 0 h 9 min, respectively; the total labor duration was 2 h 0 min. A first-degree perineal laceration was repaired with internal sutures. Documented comorbidities and major risk factors were chronic hypertension with preeclampsia; uterine fibroid during pregnancy. Neonatal birth weight was 3010 g; Apgar scores at 1, 5, and 10 minutes were 10, 10, 10; and the delivery outcome was live birth. | |
| **模型生成的产褥期护理计划 Model-generated postpartum nursing care plan** | 产妇37岁，G3P2，孕38+4周，经阴道自然分娩分娩，产时出血约250ml，新生儿男婴、体重3010g，Apgar评分10、10、10分。病例提示的重点风险为妊娠期高血压或子痫前期相关风险、高龄妊娠因素。产后24小时内重点评估体温、脉搏、血压、宫底高度、宫缩、阴道流血、恶露颜色和尿潴留，记录首次排尿和疼痛评分。产后住院及出院后继续观察恶露是否由红色逐渐转淡，乳房是否胀痛，乳头是否皲裂，睡眠和情绪是否稳定。饮食以优质蛋白、含铁食物、蔬菜水果和足量饮水为主，避免盲目大补和长期卧床。本次为阴道自然分娩，应重点观察会阴裂伤或缝合处恢复。每次大小便后保持局部清洁干燥，观察红肿、渗血、疼痛加重和异常气味。无头晕和出血增多时逐步增加活动，并在医护指导下开始轻柔盆底肌训练。哺乳方面建议尽早母婴皮肤接触和按需哺乳，指导正确含接，乳胀时先热敷或轻柔按摩再哺乳，乳头疼痛时及时调整姿势。若出现乳房局部红肿硬痛、寒战或高热，应警惕乳腺炎。有高血压或子痫前期风险者，产后仍可能出现血压波动，应按医嘱监测血压，若持续头痛、眼花、胸闷、上腹痛、抽搐或水肿加重，应立即就医。合并子宫肌瘤、卵巢囊肿或多囊卵巢相关情况时，产后复查时建议结合妇科超声评估，若腹痛、包块感或异常出血加重，应提前就诊。高龄产妇恢复期应避免过度劳累和连续熬夜，家属应参与夜间照护，保证睡眠、营养和情绪支持。出院前应完成用药、伤口、哺乳和避孕宣教，42天左右复查，必要时提前复诊。若出现阴道流血突然增多、鲜红血浸湿卫生巾、恶露恶臭、发热、持续腹痛、伤口化脓、排尿困难、下肢单侧肿痛、胸闷气短，或持续悲伤焦虑及伤害自己或婴儿的想法，应立即就医或急诊处理。 | 患者37岁，G3P2，自然分娩+I度裂伤，合并慢性高血压并发子痫前期、妊娠合并子宫肌瘤，羊水血性。产后继续监测血压每日4次，服用降压药，若血压≥150/100或出现头痛、上腹痛、视力模糊，警惕产后子痫前期/子痫。子痫前期患者血小板可能减少，注意出血倾向。子宫肌瘤影响复旧，加强宫底按摩。I度裂伤温水坐浴。血性羊水提示产前出血可能，产后观察子宫激惹、腹痛及凝血功能。降压药（拉贝洛尔、硝苯地平）对哺乳安全。提供心理支持。产后2周复查血压、尿蛋白；42天全面评估。 |
| **护理计划英文翻译 English translation of care plan** | The patient is 37 years old, G3P2, delivered at 38+4 weeks by spontaneous vaginal delivery, with an estimated intrapartum blood loss of 250 mL. The newborn was a male infant, weighed 3010 g, and had Apgar scores of 10, 10, 10. The main case-based risks include gestational hypertension or preeclampsia-related risk, advanced maternal age. Within the first 24 hours, assess temperature, pulse, blood pressure, uterine fundal height, uterine tone, vaginal bleeding, lochia color, urinary retention, first voiding, and pain score. During hospitalization and after discharge, continue to observe whether lochia gradually changes from red to lighter color, whether breast engorgement or nipple fissure occurs, and whether sleep and mood remain stable. Diet should include high-quality protein, iron-rich food, vegetables, fruit, and adequate fluids. Avoid blind over-supplementation and prolonged bed rest. Because this was a spontaneous vaginal birth, recovery of any perineal tear or suture site should be observed. Keep the area clean and dry after urination or bowel movement and watch for redness, swelling, bleeding, worsening pain, or abnormal odor. If there is no dizziness or increased bleeding, increase activity gradually and begin gentle pelvic floor exercises under professional guidance. For breastfeeding, encourage early skin-to-skin contact and feeding on demand. Teach correct latch and positioning. For engorgement, apply warmth or gentle massage before feeding. If nipple pain occurs, adjust latch promptly. Localized breast redness, hard painful swelling, chills, or high fever should raise concern for mastitis. For hypertension or preeclampsia risk, postpartum blood pressure may still fluctuate. Continue blood pressure monitoring as advised and seek urgent care for persistent headache, visual symptoms, chest tightness, upper abdominal pain, seizure, or worsening edema. For uterine fibroids, ovarian cysts, or polycystic ovary related findings, pelvic ultrasound can be considered at postpartum review. Earlier assessment is needed for worsening abdominal pain, mass sensation, or abnormal bleeding. Advanced maternal age may slow recovery, so the family should help with nighttime care and support adequate sleep, nutrition, and emotional stability. Before discharge, provide education on medication, wound care, breastfeeding, and contraception. Arrange a postpartum review around 42 days after delivery, or earlier if symptoms occur. Seek urgent medical care for sudden heavy vaginal bleeding, bright red bleeding soaking pads, foul-smelling lochia, fever, persistent abdominal pain, pus from the wound, difficulty urinating, unilateral leg swelling or pain, chest tightness, shortness of breath, or persistent sadness, anxiety, or thoughts of harming herself or the baby. | The patient is a 37-year-old G3P2 with spontaneous delivery and first-degree laceration. Comorbidities: chronic hypertension with preeclampsia, uterine fibroid, bloody amniotic fluid. Monitor BP 4 times daily; continue antihypertensives. Seek care if BP≥150/100 or headache, epigastric pain, visual changes – rule out postpartum preeclampsia/eclampsia. Preeclampsia may cause thrombocytopenia – watch for bleeding. Fibroid impairs involution – aggressive fundal massage. Warm sitz baths. Bloody fluid suggests antepartum hemorrhage – monitor coagulation. Antihypertensives (labetalol, nifedipine) safe for breastfeeding. Provide psychological support. Check BP and urine protein at 2 weeks; comprehensive exam at 42 days. |
| **Clinical appropriateness 临床适当性** | 5 | 4 |
| **Individualization 个体化程度** | 5 | 5 |
| **Comprehensiveness 完整性** | 5 | 4 |
| **Actionability 可操作性** | 4 | 4 |
| **Safety 安全性** | 5 | 4 |
| **Triage appropriateness 分诊/转诊建议适当性** | 5 | 4 |
| **Structure and clarity 结构与清晰度** | 4 | 4 |

**病例 22 / Case 22**

| **项目 Item** | **ChatGPT 5.5** | **DeepSeek V3** |
| --- | --- | --- |
| **去标识化临床病例摘要 De-identified Clinical Case Summary** | 患者29岁，孕产史为G1P1，既往剖宫产0次，孕周39+6，临产类别为催产素引产。自然破膜，羊水量约50ml，羊水性质为清。以产钳分娩，产时出血约370ml。第一产程9时16分，第二产程0时49分，第三产程0时5分，总产程10时10分，会阴或切口情况为侧切，内缝。合并症及主要风险因素：胎膜早破、妊娠期肝内胆汁淤积症（好转）、胎儿窘迫？。新生儿出生体重2590g，Apgar评分1、5、10分钟分别为10、10、10分，分娩结局为活产。 | |
| **临床病例摘要英文翻译 English Translation of De-identified Clinical Case Summary** | Maternal age was 29 years. The obstetric history was G1P1, with 0 previous cesarean deliveries. Gestational age was 39+6 weeks, and the labor onset or induction category was oxytocin induction. The membranes ruptured spontaneously; the amniotic fluid volume was approximately 50 mL and its character was clear. The patient had a forceps-assisted vaginal delivery, with estimated intrapartum blood loss of 370 mL. The first, second, and third stages of labor were 9 h 16 min, 0 h 49 min, and 0 h 5 min, respectively; the total labor duration was 10 h 10 min. An episiotomy was repaired with internal sutures. Documented comorbidities and major risk factors were premature rupture of membranes; improved intrahepatic cholestasis of pregnancy; suspected fetal distress. Neonatal birth weight was 2590 g; Apgar scores at 1, 5, and 10 minutes were 10, 10, 10; and the delivery outcome was live birth. | |
| **模型生成的产褥期护理计划 Model-generated postpartum nursing care plan** | 产妇29岁，G1P1，孕39+6周，经产钳助产阴道分娩分娩，产时出血约370ml，新生儿男婴、体重2590g，Apgar评分10、10、10分。病例提示的重点风险为胎膜早破或感染相关风险、肝功能异常或胆汁淤积史、胎儿窘迫或羊水异常记录。产后24小时内重点评估体温、脉搏、血压、宫底高度、宫缩、阴道流血、恶露颜色和尿潴留，记录首次排尿和疼痛评分。产后住院及出院后继续观察恶露是否由红色逐渐转淡，乳房是否胀痛，乳头是否皲裂，睡眠和情绪是否稳定。饮食以优质蛋白、含铁食物、蔬菜水果和足量饮水为主，避免盲目大补和长期卧床。本次为产钳助产阴道分娩，会阴伤口、盆底和排尿功能是护理重点。每次大小便后用温水从前向后清洁会阴并轻轻擦干，观察侧切或裂伤处肿胀、硬结、渗血、分泌物和异味。疼痛明显时可侧卧休息，避免久蹲、负重和过早剧烈活动。哺乳方面建议尽早母婴皮肤接触和按需哺乳，指导正确含接，乳胀时先热敷或轻柔按摩再哺乳，乳头疼痛时及时调整姿势。若出现乳房局部红肿硬痛、寒战或高热，应警惕乳腺炎。存在胎膜早破、B族链球菌感染、阴道炎或宫内感染疑虑时，应加强体温、下腹压痛、恶露气味、会阴或切口渗液观察，发热或恶露恶臭时及时复诊。因有胎儿窘迫、羊水污染或新生儿评分变化记录，母婴同室期间应配合观察新生儿吃奶、肤色、体温、呼吸和反应，发现吃奶差、发绀、嗜睡或呼吸异常应立即告知儿科医生。有胆汁淤积或肝功能异常者，产后按医嘱复查肝功能和胆汁酸，若皮肤瘙痒、黄疸、右上腹痛或尿色明显加深，应尽快就诊。出院前应完成用药、伤口、哺乳和避孕宣教，42天左右复查，必要时提前复诊。若出现阴道流血突然增多、鲜红血浸湿卫生巾、恶露恶臭、发热、持续腹痛、伤口化脓、排尿困难、下肢单侧肿痛、胸闷气短，或持续悲伤焦虑及伤害自己或婴儿的想法，应立即就医或急诊处理。 | 患者29岁，初产妇，产钳助产+侧切，合并胎膜早破、妊娠期肝内胆汁淤积症（好转）、可疑胎儿窘迫，新生儿体重2590g（边缘低出生体重）。ICP虽好转，产后仍需复查肝功能、胆汁酸；注意皮肤瘙痒是否复发。ICP患者因维生素K吸收障碍，产后出血风险增加，产时出血370ml，需警惕晚期出血，可预防性使用维生素K。会阴侧切每日消毒2次。低出生体重儿需保暖、监测黄疸。ICP不影响母乳喂养。提供心理支持。高蛋白饮食。产后2周复查肝功、胆汁酸；新生儿体重监测。 |
| **护理计划英文翻译 English translation of care plan** | The patient is 29 years old, G1P1, delivered at 39+6 weeks by forceps-assisted vaginal delivery, with an estimated intrapartum blood loss of 370 mL. The newborn was a male infant, weighed 2590 g, and had Apgar scores of 10, 10, 10. The main case-based risks include premature rupture of membranes or infection-related risk, abnormal liver function or a history of cholestasis, fetal distress or abnormal amniotic fluid findings. Within the first 24 hours, assess temperature, pulse, blood pressure, uterine fundal height, uterine tone, vaginal bleeding, lochia color, urinary retention, first voiding, and pain score. During hospitalization and after discharge, continue to observe whether lochia gradually changes from red to lighter color, whether breast engorgement or nipple fissure occurs, and whether sleep and mood remain stable. Diet should include high-quality protein, iron-rich food, vegetables, fruit, and adequate fluids. Avoid blind over-supplementation and prolonged bed rest. Because this was a forceps-assisted vaginal delivery, perineal wound care, pelvic floor recovery, and urinary function are priorities. Rinse the perineum from front to back with warm water after urination or bowel movement and pat dry. Observe the episiotomy or tear for swelling, induration, bleeding, discharge, and odor. Rest in a lateral position if pain is marked, and avoid prolonged squatting, heavy lifting, and early vigorous activity. For breastfeeding, encourage early skin-to-skin contact and feeding on demand. Teach correct latch and positioning. For engorgement, apply warmth or gentle massage before feeding. If nipple pain occurs, adjust latch promptly. Localized breast redness, hard painful swelling, chills, or high fever should raise concern for mastitis. With premature rupture of membranes, group B streptococcus, vaginitis, or suspected intrauterine infection, monitor temperature, lower abdominal tenderness, lochia odor, and drainage from the perineal wound or incision. Return promptly for fever or foul-smelling lochia. Because fetal distress, meconium-stained fluid, or changed Apgar scores were recorded, cooperate with pediatric observation of feeding, skin color, temperature, breathing, and responsiveness. Poor feeding, cyanosis, excessive sleepiness, or abnormal breathing should be reported immediately. For cholestasis or abnormal liver function, recheck liver function and bile acids as advised. Seek care for itching, jaundice, right upper abdominal pain, or dark urine. Before discharge, provide education on medication, wound care, breastfeeding, and contraception. Arrange a postpartum review around 42 days after delivery, or earlier if symptoms occur. Seek urgent medical care for sudden heavy vaginal bleeding, bright red bleeding soaking pads, foul-smelling lochia, fever, persistent abdominal pain, pus from the wound, difficulty urinating, unilateral leg swelling or pain, chest tightness, shortness of breath, or persistent sadness, anxiety, or thoughts of harming herself or the baby. | The patient is a 29-year-old primipara with forceps delivery and episiotomy. Comorbidities: PPROM, improved intrahepatic cholestasis of pregnancy, suspected fetal distress. Newborn borderline LBW 2590 g. Repeat LFTs and bile acids postpartum; monitor for recurrent pruritus. ICP increases PPH risk due to vitamin K malabsorption – blood loss 370 mL, watch for late PPH, consider prophylactic vitamin K. Episiotomy care. LBW infant – warmth, jaundice monitoring. Breastfeeding safe. Provide psychological support. High-protein diet. Check LFTs and bile acids at 2 weeks; monitor newborn weight. |
| **Clinical appropriateness 临床适当性** | 5 | 4 |
| **Individualization 个体化程度** | 5 | 5 |
| **Comprehensiveness 完整性** | 5 | 4 |
| **Actionability 可操作性** | 4 | 4 |
| **Safety 安全性** | 5 | 4 |
| **Triage appropriateness 分诊/转诊建议适当性** | 5 | 4 |
| **Structure and clarity 结构与清晰度** | 4 | 3 |

**病例 23 / Case 23**

| **项目 Item** | **ChatGPT 5.5** | **DeepSeek V3** |
| --- | --- | --- |
| **去标识化临床病例摘要 De-identified Clinical Case Summary** | 患者27岁，孕产史为G1P1，既往剖宫产0次，孕周38+2，临产类别为普贝生。自然破膜，羊水量约300ml，羊水性质为Ⅲ°。以自然分娩分娩，产时出血约210ml。第一产程4时55分，第二产程0时24分，第三产程0时6分，总产程5时25分，会阴或切口情况为Ⅰ，内缝。合并症及主要风险因素：妊娠期高血压；胎儿窘迫？妊娠合并窦性心动过速；双眼屈光不正；妊娠合并卵巢囊肿（左侧）？胎心监护异常；胎动减少。新生儿出生体重2550g，Apgar评分1、5、10分钟分别为10、10、10分，分娩结局为活产。 | |
| **临床病例摘要英文翻译 English Translation of De-identified Clinical Case Summary** | Maternal age was 27 years. The obstetric history was G1P1, with 0 previous cesarean deliveries. Gestational age was 38+2 weeks, and the labor onset or induction category was vaginal dinoprostone (Propess) induction. The membranes ruptured spontaneously; the amniotic fluid volume was approximately 300 mL and its character was grade III meconium-stained. The patient had a spontaneous vaginal delivery, with estimated intrapartum blood loss of 210 mL. The first, second, and third stages of labor were 4 h 55 min, 0 h 24 min, and 0 h 6 min, respectively; the total labor duration was 5 h 25 min. A first-degree perineal laceration was repaired with internal sutures. Documented comorbidities and major risk factors were gestational hypertension; suspected fetal distress; sinus tachycardia during pregnancy; bilateral refractive error; suspected left ovarian cyst during pregnancy; abnormal fetal heart rate monitoring; reduced fetal movement. Neonatal birth weight was 2550 g; Apgar scores at 1, 5, and 10 minutes were 10, 10, 10; and the delivery outcome was live birth. | |
| **模型生成的产褥期护理计划 Model-generated postpartum nursing care plan** | 产妇27岁，G1P1，孕38+2周，经阴道自然分娩分娩，产时出血约210ml，新生儿女婴、体重2550g，Apgar评分10、10、10分。病例提示的重点风险为妊娠期高血压或子痫前期相关风险、胎儿窘迫或羊水异常记录。产后24小时内重点评估体温、脉搏、血压、宫底高度、宫缩、阴道流血、恶露颜色和尿潴留，记录首次排尿和疼痛评分。产后住院及出院后继续观察恶露是否由红色逐渐转淡，乳房是否胀痛，乳头是否皲裂，睡眠和情绪是否稳定。饮食以优质蛋白、含铁食物、蔬菜水果和足量饮水为主，避免盲目大补和长期卧床。本次为阴道自然分娩，应重点观察会阴裂伤或缝合处恢复。每次大小便后保持局部清洁干燥，观察红肿、渗血、疼痛加重和异常气味。无头晕和出血增多时逐步增加活动，并在医护指导下开始轻柔盆底肌训练。哺乳方面建议尽早母婴皮肤接触和按需哺乳，指导正确含接，乳胀时先热敷或轻柔按摩再哺乳，乳头疼痛时及时调整姿势。若出现乳房局部红肿硬痛、寒战或高热，应警惕乳腺炎。有高血压或子痫前期风险者，产后仍可能出现血压波动，应按医嘱监测血压，若持续头痛、眼花、胸闷、上腹痛、抽搐或水肿加重，应立即就医。因有胎儿窘迫、羊水污染或新生儿评分变化记录，母婴同室期间应配合观察新生儿吃奶、肤色、体温、呼吸和反应，发现吃奶差、发绀、嗜睡或呼吸异常应立即告知儿科医生。有哮喘、心动过速或结缔组织病史者，应避免疲劳和感染诱发，出现胸闷、气短、喘息、心悸或关节症状加重时及时就医。合并子宫肌瘤、卵巢囊肿或多囊卵巢相关情况时，产后复查时建议结合妇科超声评估，若腹痛、包块感或异常出血加重，应提前就诊。出院前应完成用药、伤口、哺乳和避孕宣教，42天左右复查，必要时提前复诊。若出现阴道流血突然增多、鲜红血浸湿卫生巾、恶露恶臭、发热、持续腹痛、伤口化脓、排尿困难、下肢单侧肿痛、胸闷气短，或持续悲伤焦虑及伤害自己或婴儿的想法，应立即就医或急诊处理。 | 患者27岁，初产妇，自然分娩+I度裂伤，合并妊娠期高血压、可疑胎儿窘迫、妊娠合并窦性心动过速、胎心监护异常、胎动减少，羊水Ⅲ°污染。产后每日监测血压，如持续≥140/90需降压治疗，警惕子痫前期。窦性心动过速需心电图评估，排除围产期心肌病，避免使用增加心率的药物，注意心悸、胸闷。Ⅲ°羊水污染+可疑窘迫，新生儿需密切监测胎粪吸入综合征，即使Apgar正常，建议儿科观察24-48小时。感染预防：监测产妇体温、恶露。I度裂伤温水坐浴。提供心理支持。低盐饮食。产后1周复查心电图、血压；新生儿随访。 |
| **护理计划英文翻译 English translation of care plan** | The patient is 27 years old, G1P1, delivered at 38+2 weeks by spontaneous vaginal delivery, with an estimated intrapartum blood loss of 210 mL. The newborn was a female infant, weighed 2550 g, and had Apgar scores of 10, 10, 10. The main case-based risks include gestational hypertension or preeclampsia-related risk, fetal distress or abnormal amniotic fluid findings. Within the first 24 hours, assess temperature, pulse, blood pressure, uterine fundal height, uterine tone, vaginal bleeding, lochia color, urinary retention, first voiding, and pain score. During hospitalization and after discharge, continue to observe whether lochia gradually changes from red to lighter color, whether breast engorgement or nipple fissure occurs, and whether sleep and mood remain stable. Diet should include high-quality protein, iron-rich food, vegetables, fruit, and adequate fluids. Avoid blind over-supplementation and prolonged bed rest. Because this was a spontaneous vaginal birth, recovery of any perineal tear or suture site should be observed. Keep the area clean and dry after urination or bowel movement and watch for redness, swelling, bleeding, worsening pain, or abnormal odor. If there is no dizziness or increased bleeding, increase activity gradually and begin gentle pelvic floor exercises under professional guidance. For breastfeeding, encourage early skin-to-skin contact and feeding on demand. Teach correct latch and positioning. For engorgement, apply warmth or gentle massage before feeding. If nipple pain occurs, adjust latch promptly. Localized breast redness, hard painful swelling, chills, or high fever should raise concern for mastitis. For hypertension or preeclampsia risk, postpartum blood pressure may still fluctuate. Continue blood pressure monitoring as advised and seek urgent care for persistent headache, visual symptoms, chest tightness, upper abdominal pain, seizure, or worsening edema. Because fetal distress, meconium-stained fluid, or changed Apgar scores were recorded, cooperate with pediatric observation of feeding, skin color, temperature, breathing, and responsiveness. Poor feeding, cyanosis, excessive sleepiness, or abnormal breathing should be reported immediately. For asthma, tachycardia, or connective tissue disease, avoid fatigue and infection triggers. Chest tightness, shortness of breath, wheezing, palpitations, or worsening joint symptoms require medical review. For uterine fibroids, ovarian cysts, or polycystic ovary related findings, pelvic ultrasound can be considered at postpartum review. Earlier assessment is needed for worsening abdominal pain, mass sensation, or abnormal bleeding. Before discharge, provide education on medication, wound care, breastfeeding, and contraception. Arrange a postpartum review around 42 days after delivery, or earlier if symptoms occur. Seek urgent medical care for sudden heavy vaginal bleeding, bright red bleeding soaking pads, foul-smelling lochia, fever, persistent abdominal pain, pus from the wound, difficulty urinating, unilateral leg swelling or pain, chest tightness, shortness of breath, or persistent sadness, anxiety, or thoughts of harming herself or the baby. | The patient is a 27-year-old primipara with spontaneous delivery and first-degree laceration. Comorbidities: gestational hypertension, suspected fetal distress, sinus tachycardia, abnormal FHR, decreased fetal movement, grade III meconium-stained fluid. Monitor BP daily; treat if persistent ≥140/90. ECG to rule out peripartum cardiomyopathy; avoid rate-increasing drugs. Newborn – monitor for meconium aspiration syndrome for 24-48 hours. Infection prevention. Warm sitz baths. Provide psychological support. Low-salt diet. ECG and BP at 1 week; newborn follow-up. |
| **Clinical appropriateness 临床适当性** | 5 | 4 |
| **Individualization 个体化程度** | 5 | 5 |
| **Comprehensiveness 完整性** | 5 | 4 |
| **Actionability 可操作性** | 5 | 4 |
| **Safety 安全性** | 5 | 4 |
| **Triage appropriateness 分诊/转诊建议适当性** | 5 | 4 |
| **Structure and clarity 结构与清晰度** | 4 | 3 |

**病例 24 / Case 24**

| **项目 Item** | **ChatGPT 5.5** | **DeepSeek V3** |
| --- | --- | --- |
| **去标识化临床病例摘要 De-identified Clinical Case Summary** | 患者35岁，孕产史为G1P1，既往剖宫产0次，孕周40+1，临产类别为自然临产。自然破膜，羊水量约300ml，羊水性质为清。以自然分娩分娩，产时出血约250ml。第一产程4时0分，第二产程1时0分，第三产程0时5分，总产程5时5分，会阴或切口情况为Ⅰ，内缝。合并症及主要风险因素：胎膜早破；双眼高度近视。新生儿出生体重2930g，Apgar评分1、5、10分钟分别为10、10、10分，分娩结局为活产。 | |
| **临床病例摘要英文翻译 English Translation of De-identified Clinical Case Summary** | Maternal age was 35 years. The obstetric history was G1P1, with 0 previous cesarean deliveries. Gestational age was 40+1 weeks, and the labor onset or induction category was spontaneous labor. The membranes ruptured spontaneously; the amniotic fluid volume was approximately 300 mL and its character was clear. The patient had a spontaneous vaginal delivery, with estimated intrapartum blood loss of 250 mL. The first, second, and third stages of labor were 4 h 0 min, 1 h 0 min, and 0 h 5 min, respectively; the total labor duration was 5 h 5 min. A first-degree perineal laceration was repaired with internal sutures. Documented comorbidities and major risk factors were premature rupture of membranes; bilateral high myopia. Neonatal birth weight was 2930 g; Apgar scores at 1, 5, and 10 minutes were 10, 10, 10; and the delivery outcome was live birth. | |
| **模型生成的产褥期护理计划 Model-generated postpartum nursing care plan** | 产妇35岁，G1P1，孕40+1周，经阴道自然分娩分娩，产时出血约250ml，新生儿男婴、体重2930g，Apgar评分10、10、10分。病例提示的重点风险为胎膜早破或感染相关风险、高龄妊娠因素。产后24小时内重点评估体温、脉搏、血压、宫底高度、宫缩、阴道流血、恶露颜色和尿潴留，记录首次排尿和疼痛评分。产后住院及出院后继续观察恶露是否由红色逐渐转淡，乳房是否胀痛，乳头是否皲裂，睡眠和情绪是否稳定。饮食以优质蛋白、含铁食物、蔬菜水果和足量饮水为主，避免盲目大补和长期卧床。本次为阴道自然分娩，应重点观察会阴裂伤或缝合处恢复。每次大小便后保持局部清洁干燥，观察红肿、渗血、疼痛加重和异常气味。无头晕和出血增多时逐步增加活动，并在医护指导下开始轻柔盆底肌训练。哺乳方面建议尽早母婴皮肤接触和按需哺乳，指导正确含接，乳胀时先热敷或轻柔按摩再哺乳，乳头疼痛时及时调整姿势。若出现乳房局部红肿硬痛、寒战或高热，应警惕乳腺炎。存在胎膜早破、B族链球菌感染、阴道炎或宫内感染疑虑时，应加强体温、下腹压痛、恶露气味、会阴或切口渗液观察，发热或恶露恶臭时及时复诊。高龄产妇恢复期应避免过度劳累和连续熬夜，家属应参与夜间照护，保证睡眠、营养和情绪支持。出院前应完成用药、伤口、哺乳和避孕宣教，42天左右复查，必要时提前复诊。若出现阴道流血突然增多、鲜红血浸湿卫生巾、恶露恶臭、发热、持续腹痛、伤口化脓、排尿困难、下肢单侧肿痛、胸闷气短，或持续悲伤焦虑及伤害自己或婴儿的想法，应立即就医或急诊处理。 | 患者35岁，初产妇，自然分娩+I度裂伤，合并胎膜早破、双眼高度近视。高度近视者产后避免用力屏气（如便秘、剧烈咳嗽、提重物），以防视网膜脱离；使用开塞露或乳果糖通便。如出现闪光感、飞蚊症剧增、视野缺损，立即眼科急诊。胎膜早破注意体温、恶露、腹痛，预防感染。I度裂伤温水坐浴。常规恶露观察。鼓励母乳喂养。避免剧烈运动及头部冲击。产后6周眼科检查（眼底）；42天产科复查。 |
| **护理计划英文翻译 English translation of care plan** | The patient is 35 years old, G1P1, delivered at 40+1 weeks by spontaneous vaginal delivery, with an estimated intrapartum blood loss of 250 mL. The newborn was a male infant, weighed 2930 g, and had Apgar scores of 10, 10, 10. The main case-based risks include premature rupture of membranes or infection-related risk, advanced maternal age. Within the first 24 hours, assess temperature, pulse, blood pressure, uterine fundal height, uterine tone, vaginal bleeding, lochia color, urinary retention, first voiding, and pain score. During hospitalization and after discharge, continue to observe whether lochia gradually changes from red to lighter color, whether breast engorgement or nipple fissure occurs, and whether sleep and mood remain stable. Diet should include high-quality protein, iron-rich food, vegetables, fruit, and adequate fluids. Avoid blind over-supplementation and prolonged bed rest. Because this was a spontaneous vaginal birth, recovery of any perineal tear or suture site should be observed. Keep the area clean and dry after urination or bowel movement and watch for redness, swelling, bleeding, worsening pain, or abnormal odor. If there is no dizziness or increased bleeding, increase activity gradually and begin gentle pelvic floor exercises under professional guidance. For breastfeeding, encourage early skin-to-skin contact and feeding on demand. Teach correct latch and positioning. For engorgement, apply warmth or gentle massage before feeding. If nipple pain occurs, adjust latch promptly. Localized breast redness, hard painful swelling, chills, or high fever should raise concern for mastitis. With premature rupture of membranes, group B streptococcus, vaginitis, or suspected intrauterine infection, monitor temperature, lower abdominal tenderness, lochia odor, and drainage from the perineal wound or incision. Return promptly for fever or foul-smelling lochia. Advanced maternal age may slow recovery, so the family should help with nighttime care and support adequate sleep, nutrition, and emotional stability. Before discharge, provide education on medication, wound care, breastfeeding, and contraception. Arrange a postpartum review around 42 days after delivery, or earlier if symptoms occur. Seek urgent medical care for sudden heavy vaginal bleeding, bright red bleeding soaking pads, foul-smelling lochia, fever, persistent abdominal pain, pus from the wound, difficulty urinating, unilateral leg swelling or pain, chest tightness, shortness of breath, or persistent sadness, anxiety, or thoughts of harming herself or the baby. | The patient is a 35-year-old primipara with spontaneous delivery and first-degree laceration. Comorbidities: PPROM, bilateral high myopia. Avoid Valsalva maneuver (constipation, coughing, heavy lifting) to prevent retinal detachment. Use glycerin suppositories or lactulose. Seek emergency eye care if flashes, sudden floaters, or visual field defects occur. PPROM – monitor temperature, lochia, abdominal pain. Warm sitz baths. Encourage breastfeeding. Avoid strenuous activities. Eye exam at 6 weeks; obstetric follow-up at 42 days. |
| **Clinical appropriateness 临床适当性** | 5 | 3 |
| **Individualization 个体化程度** | 4 | 4 |
| **Comprehensiveness 完整性** | 5 | 4 |
| **Actionability 可操作性** | 4 | 4 |
| **Safety 安全性** | 5 | 3 |
| **Triage appropriateness 分诊/转诊建议适当性** | 5 | 4 |
| **Structure and clarity 结构与清晰度** | 4 | 4 |

**病例 25 / Case 25**

| **项目 Item** | **ChatGPT 5.5** | **DeepSeek V3** |
| --- | --- | --- |
| **去标识化临床病例摘要 De-identified Clinical Case Summary** | 患者26岁，孕产史为G3P2，既往剖宫产0次，孕周38+5，临产类别为自然临产。自然破膜，羊水量约300ml，羊水性质为清。以自然分娩分娩，产时出血约220ml。第一产程5时9分，第二产程0时11分，第三产程0时5分，总产程5时25分，会阴或切口情况为Ⅰ，内缝。合并症及主要风险因素：无。新生儿出生体重3270g，Apgar评分1、5、10分钟分别为10、10、10分，分娩结局为活产。 | |
| **临床病例摘要英文翻译 English Translation of De-identified Clinical Case Summary** | Maternal age was 26 years. The obstetric history was G3P2, with 0 previous cesarean deliveries. Gestational age was 38+5 weeks, and the labor onset or induction category was spontaneous labor. The membranes ruptured spontaneously; the amniotic fluid volume was approximately 300 mL and its character was clear. The patient had a spontaneous vaginal delivery, with estimated intrapartum blood loss of 220 mL. The first, second, and third stages of labor were 5 h 9 min, 0 h 11 min, and 0 h 5 min, respectively; the total labor duration was 5 h 25 min. A first-degree perineal laceration was repaired with internal sutures. No comorbidities or major risk factors were documented. Neonatal birth weight was 3270 g; Apgar scores at 1, 5, and 10 minutes were 10, 10, 10; and the delivery outcome was live birth. | |
| **模型生成的产褥期护理计划 Model-generated postpartum nursing care plan** | 产妇26岁，G3P2，孕38+5周，经阴道自然分娩分娩，产时出血约220ml，新生儿女婴、体重3270g，Apgar评分10、10、10分。病例提示的重点风险为未记录特殊合并症。产后24小时内重点评估体温、脉搏、血压、宫底高度、宫缩、阴道流血、恶露颜色和尿潴留，记录首次排尿和疼痛评分。产后住院及出院后继续观察恶露是否由红色逐渐转淡，乳房是否胀痛，乳头是否皲裂，睡眠和情绪是否稳定。饮食以优质蛋白、含铁食物、蔬菜水果和足量饮水为主，避免盲目大补和长期卧床。本次为阴道自然分娩，应重点观察会阴裂伤或缝合处恢复。每次大小便后保持局部清洁干燥，观察红肿、渗血、疼痛加重和异常气味。无头晕和出血增多时逐步增加活动，并在医护指导下开始轻柔盆底肌训练。哺乳方面建议尽早母婴皮肤接触和按需哺乳，指导正确含接，乳胀时先热敷或轻柔按摩再哺乳，乳头疼痛时及时调整姿势。若出现乳房局部红肿硬痛、寒战或高热，应警惕乳腺炎。目前未记录特殊合并症，仍需按常规产褥期管理执行，重点是子宫复旧、恶露、伤口、哺乳、营养、活动和心理状态。出院前应完成用药、伤口、哺乳和避孕宣教，42天左右复查，必要时提前复诊。若出现阴道流血突然增多、鲜红血浸湿卫生巾、恶露恶臭、发热、持续腹痛、伤口化脓、排尿困难、下肢单侧肿痛、胸闷气短，或持续悲伤焦虑及伤害自己或婴儿的想法，应立即就医或急诊处理。 | 患者26岁，G3P2，自然分娩+I度裂伤，无合并症，脐带胶质、注意渗血。产后需观察新生儿脐部有无渗血、红肿，保持脐部干燥清洁。常规产后护理：观察恶露，按摩宫底。I度裂伤温水坐浴。按需哺乳。适当活动。G3P2建议落实高效避孕（如IUD、埋植）。42天复查。 |
| **护理计划英文翻译 English translation of care plan** | The patient is 26 years old, G3P2, delivered at 38+5 weeks by spontaneous vaginal delivery, with an estimated intrapartum blood loss of 220 mL. The newborn was a female infant, weighed 3270 g, and had Apgar scores of 10, 10, 10. The main case-based risks include no special comorbidity was recorded. Within the first 24 hours, assess temperature, pulse, blood pressure, uterine fundal height, uterine tone, vaginal bleeding, lochia color, urinary retention, first voiding, and pain score. During hospitalization and after discharge, continue to observe whether lochia gradually changes from red to lighter color, whether breast engorgement or nipple fissure occurs, and whether sleep and mood remain stable. Diet should include high-quality protein, iron-rich food, vegetables, fruit, and adequate fluids. Avoid blind over-supplementation and prolonged bed rest. Because this was a spontaneous vaginal birth, recovery of any perineal tear or suture site should be observed. Keep the area clean and dry after urination or bowel movement and watch for redness, swelling, bleeding, worsening pain, or abnormal odor. If there is no dizziness or increased bleeding, increase activity gradually and begin gentle pelvic floor exercises under professional guidance. For breastfeeding, encourage early skin-to-skin contact and feeding on demand. Teach correct latch and positioning. For engorgement, apply warmth or gentle massage before feeding. If nipple pain occurs, adjust latch promptly. Localized breast redness, hard painful swelling, chills, or high fever should raise concern for mastitis. No special complication was recorded, but routine postpartum management should still focus on uterine involution, lochia, wounds, breastfeeding, nutrition, activity, and mental health. Before discharge, provide education on medication, wound care, breastfeeding, and contraception. Arrange a postpartum review around 42 days after delivery, or earlier if symptoms occur. Seek urgent medical care for sudden heavy vaginal bleeding, bright red bleeding soaking pads, foul-smelling lochia, fever, persistent abdominal pain, pus from the wound, difficulty urinating, unilateral leg swelling or pain, chest tightness, shortness of breath, or persistent sadness, anxiety, or thoughts of harming herself or the baby. | The patient is a 26-year-old G3P2 with spontaneous delivery and first-degree laceration. No comorbidities. Note: gelatinous cord with oozing risk. Observe newborn umbilical stump for bleeding or redness; keep dry and clean. Routine care: monitor lochia, fundal massage. Warm sitz baths. Breastfeed on demand. Ambulate. G3P2 – recommend highly effective contraception (IUD, implant). Follow-up at 42 days. |
| **Clinical appropriateness 临床适当性** | 4 | 3 |
| **Individualization 个体化程度** | 3 | 3 |
| **Comprehensiveness 完整性** | 4 | 3 |
| **Actionability 可操作性** | 4 | 3 |
| **Safety 安全性** | 5 | 3 |
| **Triage appropriateness 分诊/转诊建议适当性** | 5 | 3 |
| **Structure and clarity 结构与清晰度** | 3 | 4 |

**病例 26 / Case 26**

| **项目 Item** | **ChatGPT 5.5** | **DeepSeek V3** |
| --- | --- | --- |
| **去标识化临床病例摘要 De-identified Clinical Case Summary** | 患者31岁，孕产史为G1P1，既往剖宫产0次，孕周39+6，临产类别为自然临产。自然破膜，羊水量约50ml，羊水性质为Ⅲ°。以产钳分娩，产时出血约230ml。第一产程7时0分，第二产程0时32分，第三产程0时18分，总产程7时50分，会阴或切口情况为侧切，内缝。合并症及主要风险因素：桥本甲状腺炎；妊娠合并甲状腺功能减退；胎儿窘迫？。新生儿出生体重3150g，Apgar评分1、5、10分钟分别为10、10、10分，分娩结局为活产。 | |
| **临床病例摘要英文翻译 English Translation of De-identified Clinical Case Summary** | Maternal age was 31 years. The obstetric history was G1P1, with 0 previous cesarean deliveries. Gestational age was 39+6 weeks, and the labor onset or induction category was spontaneous labor. The membranes ruptured spontaneously; the amniotic fluid volume was approximately 50 mL and its character was grade III meconium-stained. The patient had a forceps-assisted vaginal delivery, with estimated intrapartum blood loss of 230 mL. The first, second, and third stages of labor were 7 h 0 min, 0 h 32 min, and 0 h 18 min, respectively; the total labor duration was 7 h 50 min. An episiotomy was repaired with internal sutures. Documented comorbidities and major risk factors were Hashimoto thyroiditis; hypothyroidism during pregnancy; suspected fetal distress. Neonatal birth weight was 3150 g; Apgar scores at 1, 5, and 10 minutes were 10, 10, 10; and the delivery outcome was live birth. | |
| **模型生成的产褥期护理计划 Model-generated postpartum nursing care plan** | 产妇31岁，G1P1，孕39+6周，经产钳助产阴道分娩分娩，产时出血约230ml，新生儿男婴、体重3150g，Apgar评分10、10、10分。病例提示的重点风险为甲状腺功能异常、胎儿窘迫或羊水异常记录。产后24小时内重点评估体温、脉搏、血压、宫底高度、宫缩、阴道流血、恶露颜色和尿潴留，记录首次排尿和疼痛评分。产后住院及出院后继续观察恶露是否由红色逐渐转淡，乳房是否胀痛，乳头是否皲裂，睡眠和情绪是否稳定。饮食以优质蛋白、含铁食物、蔬菜水果和足量饮水为主，避免盲目大补和长期卧床。本次为产钳助产阴道分娩，会阴伤口、盆底和排尿功能是护理重点。每次大小便后用温水从前向后清洁会阴并轻轻擦干，观察侧切或裂伤处肿胀、硬结、渗血、分泌物和异味。疼痛明显时可侧卧休息，避免久蹲、负重和过早剧烈活动。哺乳方面建议尽早母婴皮肤接触和按需哺乳，指导正确含接，乳胀时先热敷或轻柔按摩再哺乳，乳头疼痛时及时调整姿势。若出现乳房局部红肿硬痛、寒战或高热，应警惕乳腺炎。合并甲状腺疾病者，应按内分泌医嘱继续或调整用药，不自行停药，产后复查甲状腺功能，观察心悸、怕冷、乏力、出汗和情绪异常。因有胎儿窘迫、羊水污染或新生儿评分变化记录，母婴同室期间应配合观察新生儿吃奶、肤色、体温、呼吸和反应，发现吃奶差、发绀、嗜睡或呼吸异常应立即告知儿科医生。出院前应完成用药、伤口、哺乳和避孕宣教，42天左右复查，必要时提前复诊。若出现阴道流血突然增多、鲜红血浸湿卫生巾、恶露恶臭、发热、持续腹痛、伤口化脓、排尿困难、下肢单侧肿痛、胸闷气短，或持续悲伤焦虑及伤害自己或婴儿的想法，应立即就医或急诊处理。 | 患者31岁，初产妇，产钳助产+侧切，合并桥本甲状腺炎、妊娠合并甲减、可疑胎儿窘迫，羊水Ⅲ°污染，新生儿Apgar 10。产后继续左甲状腺素，6周复查甲功。甲减可能影响泌乳和情绪。Ⅲ°羊水污染+可疑窘迫，新生儿需监测胎粪吸入综合征，尽管Apgar正常，仍建议观察24-48小时。会阴侧切每日消毒2次。监测产妇体温防感染。鼓励勤吸吮。甲减与产后抑郁相关，留意情绪。适当活动，补充碘遵医嘱。6周复查甲功；新生儿随访。 |
| **护理计划英文翻译 English translation of care plan** | The patient is 31 years old, G1P1, delivered at 39+6 weeks by forceps-assisted vaginal delivery, with an estimated intrapartum blood loss of 230 mL. The newborn was a male infant, weighed 3150 g, and had Apgar scores of 10, 10, 10. The main case-based risks include thyroid dysfunction, fetal distress or abnormal amniotic fluid findings. Within the first 24 hours, assess temperature, pulse, blood pressure, uterine fundal height, uterine tone, vaginal bleeding, lochia color, urinary retention, first voiding, and pain score. During hospitalization and after discharge, continue to observe whether lochia gradually changes from red to lighter color, whether breast engorgement or nipple fissure occurs, and whether sleep and mood remain stable. Diet should include high-quality protein, iron-rich food, vegetables, fruit, and adequate fluids. Avoid blind over-supplementation and prolonged bed rest. Because this was a forceps-assisted vaginal delivery, perineal wound care, pelvic floor recovery, and urinary function are priorities. Rinse the perineum from front to back with warm water after urination or bowel movement and pat dry. Observe the episiotomy or tear for swelling, induration, bleeding, discharge, and odor. Rest in a lateral position if pain is marked, and avoid prolonged squatting, heavy lifting, and early vigorous activity. For breastfeeding, encourage early skin-to-skin contact and feeding on demand. Teach correct latch and positioning. For engorgement, apply warmth or gentle massage before feeding. If nipple pain occurs, adjust latch promptly. Localized breast redness, hard painful swelling, chills, or high fever should raise concern for mastitis. For thyroid disease, continue or adjust medication only under endocrine guidance, recheck thyroid function after delivery, and watch for palpitations, cold intolerance, fatigue, sweating, or abnormal mood changes. Because fetal distress, meconium-stained fluid, or changed Apgar scores were recorded, cooperate with pediatric observation of feeding, skin color, temperature, breathing, and responsiveness. Poor feeding, cyanosis, excessive sleepiness, or abnormal breathing should be reported immediately. Before discharge, provide education on medication, wound care, breastfeeding, and contraception. Arrange a postpartum review around 42 days after delivery, or earlier if symptoms occur. Seek urgent medical care for sudden heavy vaginal bleeding, bright red bleeding soaking pads, foul-smelling lochia, fever, persistent abdominal pain, pus from the wound, difficulty urinating, unilateral leg swelling or pain, chest tightness, shortness of breath, or persistent sadness, anxiety, or thoughts of harming herself or the baby. | The patient is a 31-year-old primipara with forceps delivery and episiotomy. Comorbidities: Hashimoto thyroiditis, hypothyroidism, suspected fetal distress, grade III meconium-stained fluid. Newborn Apgar 10. Continue levothyroxine, retest at 6 weeks. Monitor newborn for MAS for 24-48 hours. Episiotomy care. Monitor maternal temperature. Encourage frequent nursing. Hypothyroidism linked to PPD – monitor mood. Follow-up: thyroid function at 6 weeks; newborn follow-up. |
| **Clinical appropriateness 临床适当性** | 5 | 4 |
| **Individualization 个体化程度** | 4 | 5 |
| **Comprehensiveness 完整性** | 5 | 4 |
| **Actionability 可操作性** | 4 | 4 |
| **Safety 安全性** | 5 | 4 |
| **Triage appropriateness 分诊/转诊建议适当性** | 5 | 4 |
| **Structure and clarity 结构与清晰度** | 4 | 4 |

**病例 27 / Case 27**

| **项目 Item** | **ChatGPT 5.5** | **DeepSeek V3** |
| --- | --- | --- |
| **去标识化临床病例摘要 De-identified Clinical Case Summary** | 患者32岁，孕产史为G1P1，既往剖宫产0次，孕周36+4，临产类别为催产素引产。自然破膜，羊水量约300ml，羊水性质为清。以自然分娩分娩，产时出血约270ml。第一产程0时30分，第二产程0时22分，第三产程0时10分，总产程1时2分，会阴或切口情况为Ⅰ，内缝。合并症及主要风险因素：胎儿宫内窘迫？未足月胎膜早破；胎儿生长缓慢？妊娠合并子宫肌瘤；双眼屈光不正。新生儿出生体重2090g，Apgar评分1、5、10分钟分别为10、10、10分，分娩结局为活产。 | |
| **临床病例摘要英文翻译 English Translation of De-identified Clinical Case Summary** | Maternal age was 32 years. The obstetric history was G1P1, with 0 previous cesarean deliveries. Gestational age was 36+4 weeks, and the labor onset or induction category was oxytocin induction. The membranes ruptured spontaneously; the amniotic fluid volume was approximately 300 mL and its character was clear. The patient had a spontaneous vaginal delivery, with estimated intrapartum blood loss of 270 mL. The first, second, and third stages of labor were 0 h 30 min, 0 h 22 min, and 0 h 10 min, respectively; the total labor duration was 1 h 2 min. A first-degree perineal laceration was repaired with internal sutures. Documented comorbidities and major risk factors were suspected fetal distress; preterm premature rupture of membranes; suspected fetal growth restriction; uterine fibroid during pregnancy; bilateral refractive error. Neonatal birth weight was 2090 g; Apgar scores at 1, 5, and 10 minutes were 10, 10, 10; and the delivery outcome was live birth. | |
| **模型生成的产褥期护理计划 Model-generated postpartum nursing care plan** | 产妇32岁，G1P1，孕36+4周，经阴道自然分娩分娩，产时出血约270ml，新生儿女婴、体重2090g，Apgar评分10、10、10分。病例提示的重点风险为胎膜早破或感染相关风险、胎儿窘迫或羊水异常记录。产后24小时内重点评估体温、脉搏、血压、宫底高度、宫缩、阴道流血、恶露颜色和尿潴留，记录首次排尿和疼痛评分。产后住院及出院后继续观察恶露是否由红色逐渐转淡，乳房是否胀痛，乳头是否皲裂，睡眠和情绪是否稳定。饮食以优质蛋白、含铁食物、蔬菜水果和足量饮水为主，避免盲目大补和长期卧床。本次为阴道自然分娩，应重点观察会阴裂伤或缝合处恢复。每次大小便后保持局部清洁干燥，观察红肿、渗血、疼痛加重和异常气味。无头晕和出血增多时逐步增加活动，并在医护指导下开始轻柔盆底肌训练。哺乳方面建议尽早母婴皮肤接触和按需哺乳，指导正确含接，乳胀时先热敷或轻柔按摩再哺乳，乳头疼痛时及时调整姿势。若出现乳房局部红肿硬痛、寒战或高热，应警惕乳腺炎。存在胎膜早破、B族链球菌感染、阴道炎或宫内感染疑虑时，应加强体温、下腹压痛、恶露气味、会阴或切口渗液观察，发热或恶露恶臭时及时复诊。因有胎儿窘迫、羊水污染或新生儿评分变化记录，母婴同室期间应配合观察新生儿吃奶、肤色、体温、呼吸和反应，发现吃奶差、发绀、嗜睡或呼吸异常应立即告知儿科医生。合并子宫肌瘤、卵巢囊肿或多囊卵巢相关情况时，产后复查时建议结合妇科超声评估，若腹痛、包块感或异常出血加重，应提前就诊。出院前应完成用药、伤口、哺乳和避孕宣教，42天左右复查，必要时提前复诊。若出现阴道流血突然增多、鲜红血浸湿卫生巾、恶露恶臭、发热、持续腹痛、伤口化脓、排尿困难、下肢单侧肿痛、胸闷气短，或持续悲伤焦虑及伤害自己或婴儿的想法，应立即就医或急诊处理。 | 患者32岁，初产妇，自然分娩+I度裂伤，孕36+4周早产，合并可疑胎儿窘迫、未足月胎膜早破、可疑胎儿生长缓慢、妊娠合并子宫肌瘤、双眼屈光不正，新生儿体重2090g（低出生体重），Apgar 10。早产儿需保暖、监测血糖、黄疸、呼吸暂停；鼓励母乳喂养，可能需要母乳强化剂。子宫肌瘤可能影响复旧，加强宫底按摩。PPROM+早产，注意产褥感染及新生儿感染。生长缓慢，关注新生儿体重增长。高度近视避免用力屏气。提供心理支持。高蛋白饮食，补充铁剂。早产儿随访；产妇42天复查。 |
| **护理计划英文翻译 English translation of care plan** | The patient is 32 years old, G1P1, delivered at 36+4 weeks by spontaneous vaginal delivery, with an estimated intrapartum blood loss of 270 mL. The newborn was a female infant, weighed 2090 g, and had Apgar scores of 10, 10, 10. The main case-based risks include premature rupture of membranes or infection-related risk, fetal distress or abnormal amniotic fluid findings. Within the first 24 hours, assess temperature, pulse, blood pressure, uterine fundal height, uterine tone, vaginal bleeding, lochia color, urinary retention, first voiding, and pain score. During hospitalization and after discharge, continue to observe whether lochia gradually changes from red to lighter color, whether breast engorgement or nipple fissure occurs, and whether sleep and mood remain stable. Diet should include high-quality protein, iron-rich food, vegetables, fruit, and adequate fluids. Avoid blind over-supplementation and prolonged bed rest. Because this was a spontaneous vaginal birth, recovery of any perineal tear or suture site should be observed. Keep the area clean and dry after urination or bowel movement and watch for redness, swelling, bleeding, worsening pain, or abnormal odor. If there is no dizziness or increased bleeding, increase activity gradually and begin gentle pelvic floor exercises under professional guidance. For breastfeeding, encourage early skin-to-skin contact and feeding on demand. Teach correct latch and positioning. For engorgement, apply warmth or gentle massage before feeding. If nipple pain occurs, adjust latch promptly. Localized breast redness, hard painful swelling, chills, or high fever should raise concern for mastitis. With premature rupture of membranes, group B streptococcus, vaginitis, or suspected intrauterine infection, monitor temperature, lower abdominal tenderness, lochia odor, and drainage from the perineal wound or incision. Return promptly for fever or foul-smelling lochia. Because fetal distress, meconium-stained fluid, or changed Apgar scores were recorded, cooperate with pediatric observation of feeding, skin color, temperature, breathing, and responsiveness. Poor feeding, cyanosis, excessive sleepiness, or abnormal breathing should be reported immediately. For uterine fibroids, ovarian cysts, or polycystic ovary related findings, pelvic ultrasound can be considered at postpartum review. Earlier assessment is needed for worsening abdominal pain, mass sensation, or abnormal bleeding. Before discharge, provide education on medication, wound care, breastfeeding, and contraception. Arrange a postpartum review around 42 days after delivery, or earlier if symptoms occur. Seek urgent medical care for sudden heavy vaginal bleeding, bright red bleeding soaking pads, foul-smelling lochia, fever, persistent abdominal pain, pus from the wound, difficulty urinating, unilateral leg swelling or pain, chest tightness, shortness of breath, or persistent sadness, anxiety, or thoughts of harming herself or the baby. | The patient is a 32-year-old primipara with spontaneous delivery and first-degree laceration at 36+4 weeks. Comorbidities: suspected fetal distress, PPROM, suspected slow fetal growth, uterine fibroid, refractive error. Newborn LBW 2090 g, Apgar 10. Preterm infant care: warmth, glucose, jaundice, apnea; breastfeeding with possible fortifier. Fibroid may impair involution – fundal massage. Infection risk from PPROM+preterm. Monitor newborn growth. High myopia – avoid Valsalva. Psychological support. High-protein diet, iron. Preterm infant follow-up; maternal check at 42 days. |
| **Clinical appropriateness 临床适当性** | 5 | 3 |
| **Individualization 个体化程度** | 5 | 5 |
| **Comprehensiveness 完整性** | 5 | 4 |
| **Actionability 可操作性** | 4 | 4 |
| **Safety 安全性** | 5 | 3 |
| **Triage appropriateness 分诊/转诊建议适当性** | 5 | 3 |
| **Structure and clarity 结构与清晰度** | 4 | 3 |

**病例 28 / Case 28**

| **项目 Item** | **ChatGPT 5.5** | **DeepSeek V3** |
| --- | --- | --- |
| **去标识化临床病例摘要 De-identified Clinical Case Summary** | 患者29岁，孕产史为G2P1，既往剖宫产0次，孕周39+1，临产类别为自然临产。自然破膜，羊水量约300ml，羊水性质为清。以产钳分娩，产时出血约310ml。第一产程12时0分，第二产程1时37分，第三产程0时3分，总产程13时40分，会阴或切口情况为侧切，内缝。合并症及主要风险因素：胎膜早破。新生儿出生体重3190g，Apgar评分1、5、10分钟分别为10、10、10分，分娩结局为活产。 | |
| **临床病例摘要英文翻译 English Translation of De-identified Clinical Case Summary** | Maternal age was 29 years. The obstetric history was G2P1, with 0 previous cesarean deliveries. Gestational age was 39+1 weeks, and the labor onset or induction category was spontaneous labor. The membranes ruptured spontaneously; the amniotic fluid volume was approximately 300 mL and its character was clear. The patient had a forceps-assisted vaginal delivery, with estimated intrapartum blood loss of 310 mL. The first, second, and third stages of labor were 12 h 0 min, 1 h 37 min, and 0 h 3 min, respectively; the total labor duration was 13 h 40 min. An episiotomy was repaired with internal sutures. Documented comorbidities and major risk factors were premature rupture of membranes. Neonatal birth weight was 3190 g; Apgar scores at 1, 5, and 10 minutes were 10, 10, 10; and the delivery outcome was live birth. | |
| **模型生成的产褥期护理计划 Model-generated postpartum nursing care plan** | 产妇29岁，G2P1，孕39+1周，经产钳助产阴道分娩分娩，产时出血约310ml，新生儿男婴、体重3190g，Apgar评分10、10、10分。病例提示的重点风险为胎膜早破或感染相关风险。产后24小时内重点评估体温、脉搏、血压、宫底高度、宫缩、阴道流血、恶露颜色和尿潴留，记录首次排尿和疼痛评分。产后住院及出院后继续观察恶露是否由红色逐渐转淡，乳房是否胀痛，乳头是否皲裂，睡眠和情绪是否稳定。饮食以优质蛋白、含铁食物、蔬菜水果和足量饮水为主，避免盲目大补和长期卧床。本次为产钳助产阴道分娩，会阴伤口、盆底和排尿功能是护理重点。每次大小便后用温水从前向后清洁会阴并轻轻擦干，观察侧切或裂伤处肿胀、硬结、渗血、分泌物和异味。疼痛明显时可侧卧休息，避免久蹲、负重和过早剧烈活动。哺乳方面建议尽早母婴皮肤接触和按需哺乳，指导正确含接，乳胀时先热敷或轻柔按摩再哺乳，乳头疼痛时及时调整姿势。若出现乳房局部红肿硬痛、寒战或高热，应警惕乳腺炎。存在胎膜早破、B族链球菌感染、阴道炎或宫内感染疑虑时，应加强体温、下腹压痛、恶露气味、会阴或切口渗液观察，发热或恶露恶臭时及时复诊。出院前应完成用药、伤口、哺乳和避孕宣教，42天左右复查，必要时提前复诊。若出现阴道流血突然增多、鲜红血浸湿卫生巾、恶露恶臭、发热、持续腹痛、伤口化脓、排尿困难、下肢单侧肿痛、胸闷气短，或持续悲伤焦虑及伤害自己或婴儿的想法，应立即就医或急诊处理。 | 患者29岁，G2P1，产钳助产+侧切，合并胎膜早破、脐带绕颈1周、脐带隐性脱垂。新生儿Apgar正常，但仍需观察呼吸、肌张力。产妇注意有无宫腔感染。会阴侧切每日消毒2次，注意血肿。产时出血310ml，观察晚期出血。胎膜早破监测体温、恶露。常规子宫复旧。鼓励母乳喂养。提供心理支持。42天复查。 |
| **护理计划英文翻译 English translation of care plan** | The patient is 29 years old, G2P1, delivered at 39+1 weeks by forceps-assisted vaginal delivery, with an estimated intrapartum blood loss of 310 mL. The newborn was a male infant, weighed 3190 g, and had Apgar scores of 10, 10, 10. The main case-based risks include premature rupture of membranes or infection-related risk. Within the first 24 hours, assess temperature, pulse, blood pressure, uterine fundal height, uterine tone, vaginal bleeding, lochia color, urinary retention, first voiding, and pain score. During hospitalization and after discharge, continue to observe whether lochia gradually changes from red to lighter color, whether breast engorgement or nipple fissure occurs, and whether sleep and mood remain stable. Diet should include high-quality protein, iron-rich food, vegetables, fruit, and adequate fluids. Avoid blind over-supplementation and prolonged bed rest. Because this was a forceps-assisted vaginal delivery, perineal wound care, pelvic floor recovery, and urinary function are priorities. Rinse the perineum from front to back with warm water after urination or bowel movement and pat dry. Observe the episiotomy or tear for swelling, induration, bleeding, discharge, and odor. Rest in a lateral position if pain is marked, and avoid prolonged squatting, heavy lifting, and early vigorous activity. For breastfeeding, encourage early skin-to-skin contact and feeding on demand. Teach correct latch and positioning. For engorgement, apply warmth or gentle massage before feeding. If nipple pain occurs, adjust latch promptly. Localized breast redness, hard painful swelling, chills, or high fever should raise concern for mastitis. With premature rupture of membranes, group B streptococcus, vaginitis, or suspected intrauterine infection, monitor temperature, lower abdominal tenderness, lochia odor, and drainage from the perineal wound or incision. Return promptly for fever or foul-smelling lochia. Before discharge, provide education on medication, wound care, breastfeeding, and contraception. Arrange a postpartum review around 42 days after delivery, or earlier if symptoms occur. Seek urgent medical care for sudden heavy vaginal bleeding, bright red bleeding soaking pads, foul-smelling lochia, fever, persistent abdominal pain, pus from the wound, difficulty urinating, unilateral leg swelling or pain, chest tightness, shortness of breath, or persistent sadness, anxiety, or thoughts of harming herself or the baby. | The patient is a 29-year-old G2P1 with forceps delivery and episiotomy. Comorbidities: PPROM, nuchal cord 1 loop, occult cord prolapse. Newborn Apgar normal – still observe respiration and tone. Monitor mother for intrauterine infection. Episiotomy care – watch for hematoma. Blood loss 310 mL – watch for late PPH. Monitor temperature and lochia. Encourage breastfeeding. Psychological support. Follow-up at 42 days. |
| **Clinical appropriateness 临床适当性** | 4 | 4 |
| **Individualization 个体化程度** | 4 | 5 |
| **Comprehensiveness 完整性** | 4 | 3 |
| **Actionability 可操作性** | 4 | 4 |
| **Safety 安全性** | 5 | 4 |
| **Triage appropriateness 分诊/转诊建议适当性** | 5 | 3 |
| **Structure and clarity 结构与清晰度** | 4 | 4 |

**病例 29 / Case 29**

| **项目 Item** | **ChatGPT 5.5** | **DeepSeek V3** |
| --- | --- | --- |
| **去标识化临床病例摘要 De-identified Clinical Case Summary** | 患者35岁，孕产史为G1P1，既往剖宫产0次，孕周39+4，临产类别为自然临产。自然破膜，羊水量约300ml，羊水性质为清。以自然分娩分娩，产时出血约190ml。第一产程3时40分，第二产程0时30分，第三产程0时10分，总产程4时20分，会阴或切口情况为Ⅰ，内缝。合并症及主要风险因素：哮喘病史。新生儿出生体重3320g，Apgar评分1、5、10分钟分别为10、10、10分，分娩结局为活产。 | |
| **临床病例摘要英文翻译 English Translation of De-identified Clinical Case Summary** | Maternal age was 35 years. The obstetric history was G1P1, with 0 previous cesarean deliveries. Gestational age was 39+4 weeks, and the labor onset or induction category was spontaneous labor. The membranes ruptured spontaneously; the amniotic fluid volume was approximately 300 mL and its character was clear. The patient had a spontaneous vaginal delivery, with estimated intrapartum blood loss of 190 mL. The first, second, and third stages of labor were 3 h 40 min, 0 h 30 min, and 0 h 10 min, respectively; the total labor duration was 4 h 20 min. A first-degree perineal laceration was repaired with internal sutures. Documented comorbidities and major risk factors were history of asthma. Neonatal birth weight was 3320 g; Apgar scores at 1, 5, and 10 minutes were 10, 10, 10; and the delivery outcome was live birth. | |
| **模型生成的产褥期护理计划 Model-generated postpartum nursing care plan** | 产妇35岁，G1P1，孕39+4周，经阴道自然分娩分娩，产时出血约190ml，新生儿男婴、体重3320g，Apgar评分10、10、10分。病例提示的重点风险为高龄妊娠因素。产后24小时内重点评估体温、脉搏、血压、宫底高度、宫缩、阴道流血、恶露颜色和尿潴留，记录首次排尿和疼痛评分。产后住院及出院后继续观察恶露是否由红色逐渐转淡，乳房是否胀痛，乳头是否皲裂，睡眠和情绪是否稳定。饮食以优质蛋白、含铁食物、蔬菜水果和足量饮水为主，避免盲目大补和长期卧床。本次为阴道自然分娩，应重点观察会阴裂伤或缝合处恢复。每次大小便后保持局部清洁干燥，观察红肿、渗血、疼痛加重和异常气味。无头晕和出血增多时逐步增加活动，并在医护指导下开始轻柔盆底肌训练。哺乳方面建议尽早母婴皮肤接触和按需哺乳，指导正确含接，乳胀时先热敷或轻柔按摩再哺乳，乳头疼痛时及时调整姿势。若出现乳房局部红肿硬痛、寒战或高热，应警惕乳腺炎。有哮喘、心动过速或结缔组织病史者，应避免疲劳和感染诱发，出现胸闷、气短、喘息、心悸或关节症状加重时及时就医。高龄产妇恢复期应避免过度劳累和连续熬夜，家属应参与夜间照护，保证睡眠、营养和情绪支持。出院前应完成用药、伤口、哺乳和避孕宣教，42天左右复查，必要时提前复诊。若出现阴道流血突然增多、鲜红血浸湿卫生巾、恶露恶臭、发热、持续腹痛、伤口化脓、排尿困难、下肢单侧肿痛、胸闷气短，或持续悲伤焦虑及伤害自己或婴儿的想法，应立即就医或急诊处理。 | 患者35岁，初产妇，自然分娩+I度裂伤，合并哮喘病史。产后继续使用哮喘控制药物（吸入糖皮质激素），避免诱因，注意观察有无喘息、呼吸困难。产后激素变化可能影响哮喘控制。产时出血190ml，正常。I度裂伤温水坐浴。哮喘药物（吸入剂）几乎不入乳汁，安全，鼓励母乳喂养。适当活动，避免已知过敏原。42天复查，哮喘专科随访。 |
| **护理计划英文翻译 English translation of care plan** | The patient is 35 years old, G1P1, delivered at 39+4 weeks by spontaneous vaginal delivery, with an estimated intrapartum blood loss of 190 mL. The newborn was a male infant, weighed 3320 g, and had Apgar scores of 10, 10, 10. The main case-based risks include advanced maternal age. Within the first 24 hours, assess temperature, pulse, blood pressure, uterine fundal height, uterine tone, vaginal bleeding, lochia color, urinary retention, first voiding, and pain score. During hospitalization and after discharge, continue to observe whether lochia gradually changes from red to lighter color, whether breast engorgement or nipple fissure occurs, and whether sleep and mood remain stable. Diet should include high-quality protein, iron-rich food, vegetables, fruit, and adequate fluids. Avoid blind over-supplementation and prolonged bed rest. Because this was a spontaneous vaginal birth, recovery of any perineal tear or suture site should be observed. Keep the area clean and dry after urination or bowel movement and watch for redness, swelling, bleeding, worsening pain, or abnormal odor. If there is no dizziness or increased bleeding, increase activity gradually and begin gentle pelvic floor exercises under professional guidance. For breastfeeding, encourage early skin-to-skin contact and feeding on demand. Teach correct latch and positioning. For engorgement, apply warmth or gentle massage before feeding. If nipple pain occurs, adjust latch promptly. Localized breast redness, hard painful swelling, chills, or high fever should raise concern for mastitis. For asthma, tachycardia, or connective tissue disease, avoid fatigue and infection triggers. Chest tightness, shortness of breath, wheezing, palpitations, or worsening joint symptoms require medical review. Advanced maternal age may slow recovery, so the family should help with nighttime care and support adequate sleep, nutrition, and emotional stability. Before discharge, provide education on medication, wound care, breastfeeding, and contraception. Arrange a postpartum review around 42 days after delivery, or earlier if symptoms occur. Seek urgent medical care for sudden heavy vaginal bleeding, bright red bleeding soaking pads, foul-smelling lochia, fever, persistent abdominal pain, pus from the wound, difficulty urinating, unilateral leg swelling or pain, chest tightness, shortness of breath, or persistent sadness, anxiety, or thoughts of harming herself or the baby. | The patient is a 35-year-old primipara with spontaneous delivery and first-degree laceration. Comorbidity: history of asthma. Continue controller medications (inhaled corticosteroids); avoid triggers; monitor for wheezing or dyspnea. Hormonal changes may affect asthma control. Blood loss 190 mL, normal. Warm sitz baths. Inhaled asthma medications safe for breastfeeding. Avoid allergens. Follow-up at 42 days; asthma specialist review. |
| **Clinical appropriateness 临床适当性** | 4 | 4 |
| **Individualization 个体化程度** | 4 | 4 |
| **Comprehensiveness 完整性** | 4 | 3 |
| **Actionability 可操作性** | 4 | 3 |
| **Safety 安全性** | 5 | 4 |
| **Triage appropriateness 分诊/转诊建议适当性** | 5 | 3 |
| **Structure and clarity 结构与清晰度** | 4 | 4 |

**病例 30 / Case 30**

| **项目 Item** | **ChatGPT 5.5** | **DeepSeek V3** |
| --- | --- | --- |
| **去标识化临床病例摘要 De-identified Clinical Case Summary** | 患者33岁，孕产史为G2P2，既往剖宫产0次，孕周40+6，临产类别为自然临产。自然破膜，羊水量约400ml，羊水性质为Ⅲ°。以自然分娩分娩，产时出血约200ml。第一产程2时51分，第二产程0时14分，第三产程0时10分，总产程3时15分，会阴或切口情况为Ⅰ，内缝。合并症及主要风险因素：胎儿宫内窘迫？；妊娠合并甲状腺功能减退。新生儿出生体重3360g，Apgar评分1、5、10分钟分别为10、10、10分，分娩结局为活产。 | |
| **临床病例摘要英文翻译 English Translation of De-identified Clinical Case Summary** | Maternal age was 33 years. The obstetric history was G2P2, with 0 previous cesarean deliveries. Gestational age was 40+6 weeks, and the labor onset or induction category was spontaneous labor. The membranes ruptured spontaneously; the amniotic fluid volume was approximately 400 mL and its character was grade III meconium-stained. The patient had a spontaneous vaginal delivery, with estimated intrapartum blood loss of 200 mL. The first, second, and third stages of labor were 2 h 51 min, 0 h 14 min, and 0 h 10 min, respectively; the total labor duration was 3 h 15 min. A first-degree perineal laceration was repaired with internal sutures. Documented comorbidities and major risk factors were suspected fetal distress; hypothyroidism during pregnancy. Neonatal birth weight was 3360 g; Apgar scores at 1, 5, and 10 minutes were 10, 10, 10; and the delivery outcome was live birth. | |
| **模型生成的产褥期护理计划 Model-generated postpartum nursing care plan** | 产妇33岁，G2P2，孕40+6周，经阴道自然分娩分娩，产时出血约200ml，新生儿男婴、体重3360g，Apgar评分10、10、10分。病例提示的重点风险为甲状腺功能异常、胎儿窘迫或羊水异常记录。产后24小时内重点评估体温、脉搏、血压、宫底高度、宫缩、阴道流血、恶露颜色和尿潴留，记录首次排尿和疼痛评分。产后住院及出院后继续观察恶露是否由红色逐渐转淡，乳房是否胀痛，乳头是否皲裂，睡眠和情绪是否稳定。饮食以优质蛋白、含铁食物、蔬菜水果和足量饮水为主，避免盲目大补和长期卧床。本次为阴道自然分娩，应重点观察会阴裂伤或缝合处恢复。每次大小便后保持局部清洁干燥，观察红肿、渗血、疼痛加重和异常气味。无头晕和出血增多时逐步增加活动，并在医护指导下开始轻柔盆底肌训练。哺乳方面建议尽早母婴皮肤接触和按需哺乳，指导正确含接，乳胀时先热敷或轻柔按摩再哺乳，乳头疼痛时及时调整姿势。若出现乳房局部红肿硬痛、寒战或高热，应警惕乳腺炎。合并甲状腺疾病者，应按内分泌医嘱继续或调整用药，不自行停药，产后复查甲状腺功能，观察心悸、怕冷、乏力、出汗和情绪异常。因有胎儿窘迫、羊水污染或新生儿评分变化记录，母婴同室期间应配合观察新生儿吃奶、肤色、体温、呼吸和反应，发现吃奶差、发绀、嗜睡或呼吸异常应立即告知儿科医生。出院前应完成用药、伤口、哺乳和避孕宣教，42天左右复查，必要时提前复诊。若出现阴道流血突然增多、鲜红血浸湿卫生巾、恶露恶臭、发热、持续腹痛、伤口化脓、排尿困难、下肢单侧肿痛、胸闷气短，或持续悲伤焦虑及伤害自己或婴儿的想法，应立即就医或急诊处理。 | 患者33岁，G2P2，自然分娩+I度裂伤，合并可疑胎儿宫内窘迫、妊娠合并甲减，羊水Ⅲ°污染，脐带绕颈二周。产后继续左甲状腺素，6周复查甲功。新生儿需密切监测胎粪吸入综合征及神经系统，因Ⅲ°羊水+脐带绕颈二周+可疑窘迫，建议儿科观察24小时。监测产妇感染。I度裂伤温水坐浴。鼓励母乳喂养。提供心理支持。6周复查甲功；新生儿随访。 |
| **护理计划英文翻译 English translation of care plan** | The patient is 33 years old, G2P2, delivered at 40+6 weeks by spontaneous vaginal delivery, with an estimated intrapartum blood loss of 200 mL. The newborn was a male infant, weighed 3360 g, and had Apgar scores of 10, 10, 10. The main case-based risks include thyroid dysfunction, fetal distress or abnormal amniotic fluid findings. Within the first 24 hours, assess temperature, pulse, blood pressure, uterine fundal height, uterine tone, vaginal bleeding, lochia color, urinary retention, first voiding, and pain score. During hospitalization and after discharge, continue to observe whether lochia gradually changes from red to lighter color, whether breast engorgement or nipple fissure occurs, and whether sleep and mood remain stable. Diet should include high-quality protein, iron-rich food, vegetables, fruit, and adequate fluids. Avoid blind over-supplementation and prolonged bed rest. Because this was a spontaneous vaginal birth, recovery of any perineal tear or suture site should be observed. Keep the area clean and dry after urination or bowel movement and watch for redness, swelling, bleeding, worsening pain, or abnormal odor. If there is no dizziness or increased bleeding, increase activity gradually and begin gentle pelvic floor exercises under professional guidance. For breastfeeding, encourage early skin-to-skin contact and feeding on demand. Teach correct latch and positioning. For engorgement, apply warmth or gentle massage before feeding. If nipple pain occurs, adjust latch promptly. Localized breast redness, hard painful swelling, chills, or high fever should raise concern for mastitis. For thyroid disease, continue or adjust medication only under endocrine guidance, recheck thyroid function after delivery, and watch for palpitations, cold intolerance, fatigue, sweating, or abnormal mood changes. Because fetal distress, meconium-stained fluid, or changed Apgar scores were recorded, cooperate with pediatric observation of feeding, skin color, temperature, breathing, and responsiveness. Poor feeding, cyanosis, excessive sleepiness, or abnormal breathing should be reported immediately. Before discharge, provide education on medication, wound care, breastfeeding, and contraception. Arrange a postpartum review around 42 days after delivery, or earlier if symptoms occur. Seek urgent medical care for sudden heavy vaginal bleeding, bright red bleeding soaking pads, foul-smelling lochia, fever, persistent abdominal pain, pus from the wound, difficulty urinating, unilateral leg swelling or pain, chest tightness, shortness of breath, or persistent sadness, anxiety, or thoughts of harming herself or the baby. | The patient is a 33-year-old G2P2 with spontaneous delivery and first-degree laceration. Comorbidities: suspected fetal distress, hypothyroidism, grade III meconium-stained fluid, nuchal cord 2 loops. Continue levothyroxine, retest at 6 weeks. Newborn – monitor for MAS and neurological signs; recommend pediatric observation for 24 hours. Monitor maternal infection. Warm sitz baths. Encourage breastfeeding. Psychological support. Thyroid function at 6 weeks; newborn follow-up. |
| **Clinical appropriateness 临床适当性** | 5 | 4 |
| **Individualization 个体化程度** | 4 | 5 |
| **Comprehensiveness 完整性** | 5 | 3 |
| **Actionability 可操作性** | 4 | 4 |
| **Safety 安全性** | 5 | 4 |
| **Triage appropriateness 分诊/转诊建议适当性** | 5 | 3 |
| **Structure and clarity 结构与清晰度** | 4 | 4 |

**病例 31 / Case 31**

| **项目 Item** | **ChatGPT 5.5** | **DeepSeek V3** |
| --- | --- | --- |
| **去标识化临床病例摘要 De-identified Clinical Case Summary** | 患者32岁，孕产史为G2P1，既往剖宫产0次，孕周39+4，临产类别为自然临产。自然破膜，羊水量约300ml，羊水性质为清。以自然分娩分娩，产时出血约180ml。第一产程3时38分，第二产程0时58分，第三产程0时9分，总产程4时45分，会阴或切口情况为Ⅰ，内缝。合并症及主要风险因素：胎儿窘迫？外阴静脉曲张；妊娠合并甲状腺功能减退；妊娠合并高度近视。新生儿出生体重3470g，Apgar评分1、5、10分钟分别为10、10、10分，分娩结局为活产。 | |
| **临床病例摘要英文翻译 English Translation of De-identified Clinical Case Summary** | Maternal age was 32 years. The obstetric history was G2P1, with 0 previous cesarean deliveries. Gestational age was 39+4 weeks, and the labor onset or induction category was spontaneous labor. The membranes ruptured spontaneously; the amniotic fluid volume was approximately 300 mL and its character was clear. The patient had a spontaneous vaginal delivery, with estimated intrapartum blood loss of 180 mL. The first, second, and third stages of labor were 3 h 38 min, 0 h 58 min, and 0 h 9 min, respectively; the total labor duration was 4 h 45 min. A first-degree perineal laceration was repaired with internal sutures. Documented comorbidities and major risk factors were suspected fetal distress; vulvar varicosities; hypothyroidism during pregnancy; high myopia. Neonatal birth weight was 3470 g; Apgar scores at 1, 5, and 10 minutes were 10, 10, 10; and the delivery outcome was live birth. | |
| **模型生成的产褥期护理计划 Model-generated postpartum nursing care plan** | 产妇32岁，G2P1，孕39+4周，经阴道自然分娩分娩，产时出血约180ml，新生儿女婴、体重3470g，Apgar评分10、10、10分。病例提示的重点风险为甲状腺功能异常、胎儿窘迫或羊水异常记录。产后24小时内重点评估体温、脉搏、血压、宫底高度、宫缩、阴道流血、恶露颜色和尿潴留，记录首次排尿和疼痛评分。产后住院及出院后继续观察恶露是否由红色逐渐转淡，乳房是否胀痛，乳头是否皲裂，睡眠和情绪是否稳定。饮食以优质蛋白、含铁食物、蔬菜水果和足量饮水为主，避免盲目大补和长期卧床。本次为阴道自然分娩，应重点观察会阴裂伤或缝合处恢复。每次大小便后保持局部清洁干燥，观察红肿、渗血、疼痛加重和异常气味。无头晕和出血增多时逐步增加活动，并在医护指导下开始轻柔盆底肌训练。哺乳方面建议尽早母婴皮肤接触和按需哺乳，指导正确含接，乳胀时先热敷或轻柔按摩再哺乳，乳头疼痛时及时调整姿势。若出现乳房局部红肿硬痛、寒战或高热，应警惕乳腺炎。合并甲状腺疾病者，应按内分泌医嘱继续或调整用药，不自行停药，产后复查甲状腺功能，观察心悸、怕冷、乏力、出汗和情绪异常。因有胎儿窘迫、羊水污染或新生儿评分变化记录，母婴同室期间应配合观察新生儿吃奶、肤色、体温、呼吸和反应，发现吃奶差、发绀、嗜睡或呼吸异常应立即告知儿科医生。出院前应完成用药、伤口、哺乳和避孕宣教，42天左右复查，必要时提前复诊。若出现阴道流血突然增多、鲜红血浸湿卫生巾、恶露恶臭、发热、持续腹痛、伤口化脓、排尿困难、下肢单侧肿痛、胸闷气短，或持续悲伤焦虑及伤害自己或婴儿的想法，应立即就医或急诊处理。 | 患者32岁，G2P1，自然分娩+I度裂伤，合并可疑胎儿窘迫、外阴静脉曲张、妊娠合并甲减、妊娠合并高度近视。产后外阴静脉曲张需避免久站久坐，抬高下肢，穿戴弹力袜，观察有无血栓形成（疼痛、红肿）。继续左甲状腺素，6周复查甲功。高度近视避免用力屏气，防视网膜脱离。I度裂伤温水坐浴。产时出血180ml，正常。可疑窘迫新生儿Apgar正常，常规观察。提供心理支持。6周复查甲功、眼科；42天产科复查。 |
| **护理计划英文翻译 English translation of care plan** | The patient is 32 years old, G2P1, delivered at 39+4 weeks by spontaneous vaginal delivery, with an estimated intrapartum blood loss of 180 mL. The newborn was a female infant, weighed 3470 g, and had Apgar scores of 10, 10, 10. The main case-based risks include thyroid dysfunction, fetal distress or abnormal amniotic fluid findings. Within the first 24 hours, assess temperature, pulse, blood pressure, uterine fundal height, uterine tone, vaginal bleeding, lochia color, urinary retention, first voiding, and pain score. During hospitalization and after discharge, continue to observe whether lochia gradually changes from red to lighter color, whether breast engorgement or nipple fissure occurs, and whether sleep and mood remain stable. Diet should include high-quality protein, iron-rich food, vegetables, fruit, and adequate fluids. Avoid blind over-supplementation and prolonged bed rest. Because this was a spontaneous vaginal birth, recovery of any perineal tear or suture site should be observed. Keep the area clean and dry after urination or bowel movement and watch for redness, swelling, bleeding, worsening pain, or abnormal odor. If there is no dizziness or increased bleeding, increase activity gradually and begin gentle pelvic floor exercises under professional guidance. For breastfeeding, encourage early skin-to-skin contact and feeding on demand. Teach correct latch and positioning. For engorgement, apply warmth or gentle massage before feeding. If nipple pain occurs, adjust latch promptly. Localized breast redness, hard painful swelling, chills, or high fever should raise concern for mastitis. For thyroid disease, continue or adjust medication only under endocrine guidance, recheck thyroid function after delivery, and watch for palpitations, cold intolerance, fatigue, sweating, or abnormal mood changes. Because fetal distress, meconium-stained fluid, or changed Apgar scores were recorded, cooperate with pediatric observation of feeding, skin color, temperature, breathing, and responsiveness. Poor feeding, cyanosis, excessive sleepiness, or abnormal breathing should be reported immediately. Before discharge, provide education on medication, wound care, breastfeeding, and contraception. Arrange a postpartum review around 42 days after delivery, or earlier if symptoms occur. Seek urgent medical care for sudden heavy vaginal bleeding, bright red bleeding soaking pads, foul-smelling lochia, fever, persistent abdominal pain, pus from the wound, difficulty urinating, unilateral leg swelling or pain, chest tightness, shortness of breath, or persistent sadness, anxiety, or thoughts of harming herself or the baby. | The patient is a 32-year-old G2P1 with spontaneous delivery and first-degree laceration. Comorbidities: suspected fetal distress, vulvar varicosities, hypothyroidism, high myopia. For varicosities: avoid prolonged standing/sitting, elevate legs, wear compression stockings; monitor for thrombosis (pain, redness). Continue levothyroxine, retest at 6 weeks. High myopia – avoid Valsalva to prevent retinal detachment. Warm sitz baths. Blood loss 180 mL, normal. Newborn routine observation. Psychological support. Follow-up: thyroid and eye exam at 6 weeks; obstetric check at 42 days. |
| **Clinical appropriateness 临床适当性** | 5 | 3 |
| **Individualization 个体化程度** | 4 | 5 |
| **Comprehensiveness 完整性** | 5 | 4 |
| **Actionability 可操作性** | 4 | 4 |
| **Safety 安全性** | 5 | 3 |
| **Triage appropriateness 分诊/转诊建议适当性** | 5 | 4 |
| **Structure and clarity 结构与清晰度** | 4 | 4 |

**病例 32 / Case 32**

| **项目 Item** | **ChatGPT 5.5** | **DeepSeek V3** |
| --- | --- | --- |
| **去标识化临床病例摘要 De-identified Clinical Case Summary** | 患者33岁，孕产史为G2P1，既往剖宫产0次，孕周39+5，临产类别为自然临产。自然破膜，羊水量约200ml，羊水性质为清。以产钳分娩，产时出血约310ml。第一产程9时25分，第二产程0时43分，第三产程0时7分，总产程10时15分，会阴或切口情况为侧切，内缝。合并症及主要风险因素：胎膜早破；妊娠合并阴道溶血性链球菌感染；胎儿窘迫？。新生儿出生体重3650g，Apgar评分1、5、10分钟分别为8、10、10分，分娩结局为活产。 | |
| **临床病例摘要英文翻译 English Translation of De-identified Clinical Case Summary** | Maternal age was 33 years. The obstetric history was G2P1, with 0 previous cesarean deliveries. Gestational age was 39+5 weeks, and the labor onset or induction category was spontaneous labor. The membranes ruptured spontaneously; the amniotic fluid volume was approximately 200 mL and its character was clear. The patient had a forceps-assisted vaginal delivery, with estimated intrapartum blood loss of 310 mL. The first, second, and third stages of labor were 9 h 25 min, 0 h 43 min, and 0 h 7 min, respectively; the total labor duration was 10 h 15 min. An episiotomy was repaired with internal sutures. Documented comorbidities and major risk factors were premature rupture of membranes; vaginal hemolytic Streptococcus infection in pregnancy; suspected fetal distress. Neonatal birth weight was 3650 g; Apgar scores at 1, 5, and 10 minutes were 8, 10, 10; and the delivery outcome was live birth. | |
| **模型生成的产褥期护理计划 Model-generated postpartum nursing care plan** | 产妇33岁，G2P1，孕39+5周，经产钳助产阴道分娩分娩，产时出血约310ml，新生儿女婴、体重3650g，Apgar评分8、10、10分。病例提示的重点风险为胎膜早破或感染相关风险、胎儿窘迫或羊水异常记录。产后24小时内重点评估体温、脉搏、血压、宫底高度、宫缩、阴道流血、恶露颜色和尿潴留，记录首次排尿和疼痛评分。产后住院及出院后继续观察恶露是否由红色逐渐转淡，乳房是否胀痛，乳头是否皲裂，睡眠和情绪是否稳定。饮食以优质蛋白、含铁食物、蔬菜水果和足量饮水为主，避免盲目大补和长期卧床。本次为产钳助产阴道分娩，会阴伤口、盆底和排尿功能是护理重点。每次大小便后用温水从前向后清洁会阴并轻轻擦干，观察侧切或裂伤处肿胀、硬结、渗血、分泌物和异味。疼痛明显时可侧卧休息，避免久蹲、负重和过早剧烈活动。哺乳方面建议尽早母婴皮肤接触和按需哺乳，指导正确含接，乳胀时先热敷或轻柔按摩再哺乳，乳头疼痛时及时调整姿势。若出现乳房局部红肿硬痛、寒战或高热，应警惕乳腺炎。存在胎膜早破、B族链球菌感染、阴道炎或宫内感染疑虑时，应加强体温、下腹压痛、恶露气味、会阴或切口渗液观察，发热或恶露恶臭时及时复诊。因有胎儿窘迫、羊水污染或新生儿评分变化记录，母婴同室期间应配合观察新生儿吃奶、肤色、体温、呼吸和反应，发现吃奶差、发绀、嗜睡或呼吸异常应立即告知儿科医生。出院前应完成用药、伤口、哺乳和避孕宣教，42天左右复查，必要时提前复诊。若出现阴道流血突然增多、鲜红血浸湿卫生巾、恶露恶臭、发热、持续腹痛、伤口化脓、排尿困难、下肢单侧肿痛、胸闷气短，或持续悲伤焦虑及伤害自己或婴儿的想法，应立即就医或急诊处理。 | 患者33岁，G2P1，产钳助产+侧切，合并胎膜早破、妊娠合并阴道溶血性链球菌感染、可疑胎儿窘迫，新生儿1分钟Apgar 8分。GBS感染+PPROM需静脉抗生素，产后继续监测体温、腹痛、恶露；新生儿需预防性观察GBS早发型败血症。1分钟Apgar 8分，注意新生儿呼吸、循环、神经，观察24小时。会阴侧切每日消毒2次。产时出血310ml，观察晚期出血。GBS不影响哺乳。提供心理支持。产后1周复查感染指标；新生儿随访。 |
| **护理计划英文翻译 English translation of care plan** | The patient is 33 years old, G2P1, delivered at 39+5 weeks by forceps-assisted vaginal delivery, with an estimated intrapartum blood loss of 310 mL. The newborn was a female infant, weighed 3650 g, and had Apgar scores of 8, 10, 10. The main case-based risks include premature rupture of membranes or infection-related risk, fetal distress or abnormal amniotic fluid findings. Within the first 24 hours, assess temperature, pulse, blood pressure, uterine fundal height, uterine tone, vaginal bleeding, lochia color, urinary retention, first voiding, and pain score. During hospitalization and after discharge, continue to observe whether lochia gradually changes from red to lighter color, whether breast engorgement or nipple fissure occurs, and whether sleep and mood remain stable. Diet should include high-quality protein, iron-rich food, vegetables, fruit, and adequate fluids. Avoid blind over-supplementation and prolonged bed rest. Because this was a forceps-assisted vaginal delivery, perineal wound care, pelvic floor recovery, and urinary function are priorities. Rinse the perineum from front to back with warm water after urination or bowel movement and pat dry. Observe the episiotomy or tear for swelling, induration, bleeding, discharge, and odor. Rest in a lateral position if pain is marked, and avoid prolonged squatting, heavy lifting, and early vigorous activity. For breastfeeding, encourage early skin-to-skin contact and feeding on demand. Teach correct latch and positioning. For engorgement, apply warmth or gentle massage before feeding. If nipple pain occurs, adjust latch promptly. Localized breast redness, hard painful swelling, chills, or high fever should raise concern for mastitis. With premature rupture of membranes, group B streptococcus, vaginitis, or suspected intrauterine infection, monitor temperature, lower abdominal tenderness, lochia odor, and drainage from the perineal wound or incision. Return promptly for fever or foul-smelling lochia. Because fetal distress, meconium-stained fluid, or changed Apgar scores were recorded, cooperate with pediatric observation of feeding, skin color, temperature, breathing, and responsiveness. Poor feeding, cyanosis, excessive sleepiness, or abnormal breathing should be reported immediately. Before discharge, provide education on medication, wound care, breastfeeding, and contraception. Arrange a postpartum review around 42 days after delivery, or earlier if symptoms occur. Seek urgent medical care for sudden heavy vaginal bleeding, bright red bleeding soaking pads, foul-smelling lochia, fever, persistent abdominal pain, pus from the wound, difficulty urinating, unilateral leg swelling or pain, chest tightness, shortness of breath, or persistent sadness, anxiety, or thoughts of harming herself or the baby. | The patient is a 33-year-old G2P1 with forceps delivery and episiotomy. Comorbidities: PPROM, vaginal hemolytic Streptococcus infection, suspected fetal distress. Newborn 1-min Apgar 8. IV antibiotics for GBS+PPROM. Monitor mother for infection; observe newborn for early-onset GBS sepsis for 24 hours. Episiotomy care. Blood loss 310 mL – watch for late PPH. Breastfeeding safe. Psychological support. Check infection markers at 1 week; newborn follow-up. |
| **Clinical appropriateness 临床适当性** | 5 | 4 |
| **Individualization 个体化程度** | 4 | 5 |
| **Comprehensiveness 完整性** | 5 | 4 |
| **Actionability 可操作性** | 4 | 4 |
| **Safety 安全性** | 5 | 4 |
| **Triage appropriateness 分诊/转诊建议适当性** | 5 | 4 |
| **Structure and clarity 结构与清晰度** | 4 | 4 |

**病例 33 / Case 33**

| **项目 Item** | **ChatGPT 5.5** | **DeepSeek V3** |
| --- | --- | --- |
| **去标识化临床病例摘要 De-identified Clinical Case Summary** | 患者42岁，孕产史为G4P2，既往剖宫产0次，孕周41+2，临产类别为阴道放置米索。自然破膜，羊水量约400ml，羊水性质为清。以自然分娩分娩，产时出血约1150ml。第一产程2时55分，第二产程0时28分，第三产程0时7分，总产程3时30分，会阴或切口情况为Ⅰ，内缝。合并症及主要风险因素：双眼屈光不正、双眼散光、不良孕产个人史、高龄经产妇妊娠监督、妊娠合并甲状腺功能减退（亚临床）。新生儿出生体重3780g，Apgar评分1、5、10分钟分别为10、10、10分，分娩结局为活产。 | |
| **临床病例摘要英文翻译 English Translation of De-identified Clinical Case Summary** | Maternal age was 42 years. The obstetric history was G4P2, with 0 previous cesarean deliveries. Gestational age was 41+2 weeks, and the labor onset or induction category was vaginal misoprostol induction. The membranes ruptured spontaneously; the amniotic fluid volume was approximately 400 mL and its character was clear. The patient had a spontaneous vaginal delivery, with estimated intrapartum blood loss of 1150 mL. The first, second, and third stages of labor were 2 h 55 min, 0 h 28 min, and 0 h 7 min, respectively; the total labor duration was 3 h 30 min. A first-degree perineal laceration was repaired with internal sutures. Documented comorbidities and major risk factors were bilateral refractive error; bilateral astigmatism; poor obstetric history; advanced maternal age in a multipara; subclinical hypothyroidism during pregnancy. Neonatal birth weight was 3780 g; Apgar scores at 1, 5, and 10 minutes were 10, 10, 10; and the delivery outcome was live birth. | |
| **模型生成的产褥期护理计划 Model-generated postpartum nursing care plan** | 产妇42岁，G4P2，孕41+2周，经阴道自然分娩分娩，产时出血约1150ml，新生儿女婴、体重3780g，Apgar评分10、10、10分。病例提示的重点风险为出血或贫血相关风险、甲状腺功能异常、高龄妊娠因素。产后24小时内重点评估体温、脉搏、血压、宫底高度、宫缩、阴道流血、恶露颜色和尿潴留，记录首次排尿和疼痛评分。产后住院及出院后继续观察恶露是否由红色逐渐转淡，乳房是否胀痛，乳头是否皲裂，睡眠和情绪是否稳定。饮食以优质蛋白、含铁食物、蔬菜水果和足量饮水为主，避免盲目大补和长期卧床。本次为阴道自然分娩，应重点观察会阴裂伤或缝合处恢复。每次大小便后保持局部清洁干燥，观察红肿、渗血、疼痛加重和异常气味。无头晕和出血增多时逐步增加活动，并在医护指导下开始轻柔盆底肌训练。哺乳方面建议尽早母婴皮肤接触和按需哺乳，指导正确含接，乳胀时先热敷或轻柔按摩再哺乳，乳头疼痛时及时调整姿势。若出现乳房局部红肿硬痛、寒战或高热，应警惕乳腺炎。因存在出血量偏多、贫血或血小板异常相关风险，应复查血常规，关注头晕、心悸、乏力、面色苍白、恶露突然增多或较大血块，按医嘱补铁，必要时评估凝血和子宫复旧。合并甲状腺疾病者，应按内分泌医嘱继续或调整用药，不自行停药，产后复查甲状腺功能，观察心悸、怕冷、乏力、出汗和情绪异常。高龄产妇恢复期应避免过度劳累和连续熬夜，家属应参与夜间照护，保证睡眠、营养和情绪支持。出院前应完成用药、伤口、哺乳和避孕宣教，42天左右复查，必要时提前复诊。若出现阴道流血突然增多、鲜红血浸湿卫生巾、恶露恶臭、发热、持续腹痛、伤口化脓、排尿困难、下肢单侧肿痛、胸闷气短，或持续悲伤焦虑及伤害自己或婴儿的想法，应立即就医或急诊处理。 | 患者42岁，G4P2，自然分娩+I度裂伤，产时出血1150ml（严重产后出血），合并高龄经产妇、亚临床甲减、不良孕产史、脐带隐性脱垂。出血1150ml需住院紧急处理，出院后监测血红蛋白，补充铁剂，必要时输血；观察有无晚期出血。加强宫底按摩，使用缩宫素。亚临床甲减继续左甲状腺素，6周复查。大量出血后免疫力下降，注意产褥感染。I度裂伤温水坐浴。隐性脱垂新生儿Apgar正常，常规观察。大出血易致创伤后应激，提供心理支持。产后1周复查血常规；42天全面评估。 |
| **护理计划英文翻译 English translation of care plan** | The patient is 42 years old, G4P2, delivered at 41+2 weeks by spontaneous vaginal delivery, with an estimated intrapartum blood loss of 1150 mL. The newborn was a female infant, weighed 3780 g, and had Apgar scores of 10, 10, 10. The main case-based risks include bleeding or anemia-related risk, thyroid dysfunction, advanced maternal age. Within the first 24 hours, assess temperature, pulse, blood pressure, uterine fundal height, uterine tone, vaginal bleeding, lochia color, urinary retention, first voiding, and pain score. During hospitalization and after discharge, continue to observe whether lochia gradually changes from red to lighter color, whether breast engorgement or nipple fissure occurs, and whether sleep and mood remain stable. Diet should include high-quality protein, iron-rich food, vegetables, fruit, and adequate fluids. Avoid blind over-supplementation and prolonged bed rest. Because this was a spontaneous vaginal birth, recovery of any perineal tear or suture site should be observed. Keep the area clean and dry after urination or bowel movement and watch for redness, swelling, bleeding, worsening pain, or abnormal odor. If there is no dizziness or increased bleeding, increase activity gradually and begin gentle pelvic floor exercises under professional guidance. For breastfeeding, encourage early skin-to-skin contact and feeding on demand. Teach correct latch and positioning. For engorgement, apply warmth or gentle massage before feeding. If nipple pain occurs, adjust latch promptly. Localized breast redness, hard painful swelling, chills, or high fever should raise concern for mastitis. Because increased blood loss, anemia, or platelet abnormality is a concern, repeat a complete blood count as advised and watch for dizziness, palpitations, severe fatigue, pallor, sudden increase in lochia, or large clots. Iron supplementation, nutrition support, and assessment of uterine involution or coagulation should follow medical orders. For thyroid disease, continue or adjust medication only under endocrine guidance, recheck thyroid function after delivery, and watch for palpitations, cold intolerance, fatigue, sweating, or abnormal mood changes. Advanced maternal age may slow recovery, so the family should help with nighttime care and support adequate sleep, nutrition, and emotional stability. Before discharge, provide education on medication, wound care, breastfeeding, and contraception. Arrange a postpartum review around 42 days after delivery, or earlier if symptoms occur. Seek urgent medical care for sudden heavy vaginal bleeding, bright red bleeding soaking pads, foul-smelling lochia, fever, persistent abdominal pain, pus from the wound, difficulty urinating, unilateral leg swelling or pain, chest tightness, shortness of breath, or persistent sadness, anxiety, or thoughts of harming herself or the baby. | The patient is a 42-year-old G4P2 with spontaneous delivery and first-degree laceration. Severe PPH 1150 mL. Comorbidities: advanced age multipara, subclinical hypothyroidism, poor obstetric history, occult cord prolapse. Post-discharge monitor hemoglobin, iron supplementation, possible transfusion. Watch for late PPH. Aggressive fundal massage, oxytocin. Continue levothyroxine, retest at 6 weeks. Immunosuppression after massive hemorrhage – monitor for puerperal infection. Warm sitz baths. PPH may cause PTSD – psychological support. Check CBC at 1 week; comprehensive exam at 42 days. |
| **Clinical appropriateness 临床适当性** | 5 | 4 |
| **Individualization 个体化程度** | 5 | 5 |
| **Comprehensiveness 完整性** | 5 | 4 |
| **Actionability 可操作性** | 4 | 4 |
| **Safety 安全性** | 5 | 4 |
| **Triage appropriateness 分诊/转诊建议适当性** | 5 | 4 |
| **Structure and clarity 结构与清晰度** | 4 | 4 |

**病例 34 / Case 34**

| **项目 Item** | **ChatGPT 5.5** | **DeepSeek V3** |
| --- | --- | --- |
| **去标识化临床病例摘要 De-identified Clinical Case Summary** | 患者39岁，孕产史为G2P2，既往剖宫产0次，孕周38，临产类别为自然临产。自然破膜，羊水量约200ml，羊水性质为清。以产钳分娩，产时出血约210ml。第一产程3时30分，第二产程0时36分，第三产程0时6分，总产程4时12分，会阴或切口情况为Ⅰ，内缝。合并症及主要风险因素：胎膜早破、高龄经产妇妊娠监督、胎儿宫内窘迫？。新生儿出生体重3290g，Apgar评分1、5、10分钟分别为9、10、10分，分娩结局为活产。 | |
| **临床病例摘要英文翻译 English Translation of De-identified Clinical Case Summary** | Maternal age was 39 years. The obstetric history was G2P2, with 0 previous cesarean deliveries. Gestational age was 38 weeks, and the labor onset or induction category was spontaneous labor. The membranes ruptured spontaneously; the amniotic fluid volume was approximately 200 mL and its character was clear. The patient had a forceps-assisted vaginal delivery, with estimated intrapartum blood loss of 210 mL. The first, second, and third stages of labor were 3 h 30 min, 0 h 36 min, and 0 h 6 min, respectively; the total labor duration was 4 h 12 min. A first-degree perineal laceration was repaired with internal sutures. Documented comorbidities and major risk factors were premature rupture of membranes; advanced maternal age in a multipara; suspected fetal distress. Neonatal birth weight was 3290 g; Apgar scores at 1, 5, and 10 minutes were 9, 10, 10; and the delivery outcome was live birth. | |
| **模型生成的产褥期护理计划 Model-generated postpartum nursing care plan** | 产妇39岁，G2P2，孕38周，经产钳助产阴道分娩分娩，产时出血约210ml，新生儿男婴、体重3290g，Apgar评分9、10、10分。病例提示的重点风险为胎膜早破或感染相关风险、胎儿窘迫或羊水异常记录、高龄妊娠因素。产后24小时内重点评估体温、脉搏、血压、宫底高度、宫缩、阴道流血、恶露颜色和尿潴留，记录首次排尿和疼痛评分。产后住院及出院后继续观察恶露是否由红色逐渐转淡，乳房是否胀痛，乳头是否皲裂，睡眠和情绪是否稳定。饮食以优质蛋白、含铁食物、蔬菜水果和足量饮水为主，避免盲目大补和长期卧床。本次为产钳助产阴道分娩，会阴伤口、盆底和排尿功能是护理重点。每次大小便后用温水从前向后清洁会阴并轻轻擦干，观察侧切或裂伤处肿胀、硬结、渗血、分泌物和异味。疼痛明显时可侧卧休息，避免久蹲、负重和过早剧烈活动。哺乳方面建议尽早母婴皮肤接触和按需哺乳，指导正确含接，乳胀时先热敷或轻柔按摩再哺乳，乳头疼痛时及时调整姿势。若出现乳房局部红肿硬痛、寒战或高热，应警惕乳腺炎。存在胎膜早破、B族链球菌感染、阴道炎或宫内感染疑虑时，应加强体温、下腹压痛、恶露气味、会阴或切口渗液观察，发热或恶露恶臭时及时复诊。因有胎儿窘迫、羊水污染或新生儿评分变化记录，母婴同室期间应配合观察新生儿吃奶、肤色、体温、呼吸和反应，发现吃奶差、发绀、嗜睡或呼吸异常应立即告知儿科医生。高龄产妇恢复期应避免过度劳累和连续熬夜，家属应参与夜间照护，保证睡眠、营养和情绪支持。出院前应完成用药、伤口、哺乳和避孕宣教，42天左右复查，必要时提前复诊。若出现阴道流血突然增多、鲜红血浸湿卫生巾、恶露恶臭、发热、持续腹痛、伤口化脓、排尿困难、下肢单侧肿痛、胸闷气短，或持续悲伤焦虑及伤害自己或婴儿的想法，应立即就医或急诊处理。 | 患者39岁，G2P2，产钳助产+I度裂伤（内缝），合并胎膜早破、高龄经产妇、可疑胎儿宫内窘迫，脐带绕颈1周（紧）。新生儿需密切观察呼吸、肌张力、神经系统，尽管Apgar 9-10-10，仍建议观察24小时。产钳助产注意会阴血肿，温水坐浴。胎膜早破监测感染。产时出血210ml，正常。鼓励母乳喂养。提供心理支持。42天复查。 |
| **护理计划英文翻译 English translation of care plan** | The patient is 39 years old, G2P2, delivered at 38 weeks by forceps-assisted vaginal delivery, with an estimated intrapartum blood loss of 210 mL. The newborn was a male infant, weighed 3290 g, and had Apgar scores of 9, 10, 10. The main case-based risks include premature rupture of membranes or infection-related risk, fetal distress or abnormal amniotic fluid findings, advanced maternal age. Within the first 24 hours, assess temperature, pulse, blood pressure, uterine fundal height, uterine tone, vaginal bleeding, lochia color, urinary retention, first voiding, and pain score. During hospitalization and after discharge, continue to observe whether lochia gradually changes from red to lighter color, whether breast engorgement or nipple fissure occurs, and whether sleep and mood remain stable. Diet should include high-quality protein, iron-rich food, vegetables, fruit, and adequate fluids. Avoid blind over-supplementation and prolonged bed rest. Because this was a forceps-assisted vaginal delivery, perineal wound care, pelvic floor recovery, and urinary function are priorities. Rinse the perineum from front to back with warm water after urination or bowel movement and pat dry. Observe the episiotomy or tear for swelling, induration, bleeding, discharge, and odor. Rest in a lateral position if pain is marked, and avoid prolonged squatting, heavy lifting, and early vigorous activity. For breastfeeding, encourage early skin-to-skin contact and feeding on demand. Teach correct latch and positioning. For engorgement, apply warmth or gentle massage before feeding. If nipple pain occurs, adjust latch promptly. Localized breast redness, hard painful swelling, chills, or high fever should raise concern for mastitis. With premature rupture of membranes, group B streptococcus, vaginitis, or suspected intrauterine infection, monitor temperature, lower abdominal tenderness, lochia odor, and drainage from the perineal wound or incision. Return promptly for fever or foul-smelling lochia. Because fetal distress, meconium-stained fluid, or changed Apgar scores were recorded, cooperate with pediatric observation of feeding, skin color, temperature, breathing, and responsiveness. Poor feeding, cyanosis, excessive sleepiness, or abnormal breathing should be reported immediately. Advanced maternal age may slow recovery, so the family should help with nighttime care and support adequate sleep, nutrition, and emotional stability. Before discharge, provide education on medication, wound care, breastfeeding, and contraception. Arrange a postpartum review around 42 days after delivery, or earlier if symptoms occur. Seek urgent medical care for sudden heavy vaginal bleeding, bright red bleeding soaking pads, foul-smelling lochia, fever, persistent abdominal pain, pus from the wound, difficulty urinating, unilateral leg swelling or pain, chest tightness, shortness of breath, or persistent sadness, anxiety, or thoughts of harming herself or the baby. | The patient is a 39-year-old G2P2 with forceps delivery and first-degree laceration (internal suturing). Comorbidities: PPROM, advanced age multipara, suspected fetal distress, tight nuchal cord 1 loop. Newborn – monitor respiration, tone, neurology for 24 hours despite good Apgars. Forceps – watch for perineal hematoma; warm sitz baths. Monitor infection. Blood loss 210 mL, normal. Encourage breastfeeding. Psychological support. Follow-up at 42 days. |
| **Clinical appropriateness 临床适当性** | 5 | 4 |
| **Individualization 个体化程度** | 5 | 5 |
| **Comprehensiveness 完整性** | 5 | 3 |
| **Actionability 可操作性** | 4 | 4 |
| **Safety 安全性** | 5 | 4 |
| **Triage appropriateness 分诊/转诊建议适当性** | 5 | 3 |
| **Structure and clarity 结构与清晰度** | 4 | 3 |

**病例 35 / Case 35**

| **项目 Item** | **ChatGPT 5.5** | **DeepSeek V3** |
| --- | --- | --- |
| **去标识化临床病例摘要 De-identified Clinical Case Summary** | 患者29岁，孕产史为G1P1，既往剖宫产0次，孕周39+2，临产类别为自然临产。自然破膜，羊水量约300ml，羊水性质为清。以自然分娩分娩，产时出血约250ml。第一产程2时55分，第二产程0时15分，第三产程0时5分，总产程3时15分，会阴或切口情况为Ⅰ，内缝。合并症及主要风险因素：多囊卵巢综合征；妊娠合并右侧卵巢囊肿；胎儿宫内窘迫？。新生儿出生体重3020g，Apgar评分1、5、10分钟分别为10、10、10分，分娩结局为活产。 | |
| **临床病例摘要英文翻译 English Translation of De-identified Clinical Case Summary** | Maternal age was 29 years. The obstetric history was G1P1, with 0 previous cesarean deliveries. Gestational age was 39+2 weeks, and the labor onset or induction category was spontaneous labor. The membranes ruptured spontaneously; the amniotic fluid volume was approximately 300 mL and its character was clear. The patient had a spontaneous vaginal delivery, with estimated intrapartum blood loss of 250 mL. The first, second, and third stages of labor were 2 h 55 min, 0 h 15 min, and 0 h 5 min, respectively; the total labor duration was 3 h 15 min. A first-degree perineal laceration was repaired with internal sutures. Documented comorbidities and major risk factors were polycystic ovary syndrome; right ovarian cyst during pregnancy; suspected fetal distress. Neonatal birth weight was 3020 g; Apgar scores at 1, 5, and 10 minutes were 10, 10, 10; and the delivery outcome was live birth. | |
| **模型生成的产褥期护理计划 Model-generated postpartum nursing care plan** | 产妇29岁，G1P1，孕39+2周，经阴道自然分娩分娩，产时出血约250ml，新生儿男婴、体重3020g，Apgar评分10、10、10分。病例提示的重点风险为胎儿窘迫或羊水异常记录。产后24小时内重点评估体温、脉搏、血压、宫底高度、宫缩、阴道流血、恶露颜色和尿潴留，记录首次排尿和疼痛评分。产后住院及出院后继续观察恶露是否由红色逐渐转淡，乳房是否胀痛，乳头是否皲裂，睡眠和情绪是否稳定。饮食以优质蛋白、含铁食物、蔬菜水果和足量饮水为主，避免盲目大补和长期卧床。本次为阴道自然分娩，应重点观察会阴裂伤或缝合处恢复。每次大小便后保持局部清洁干燥，观察红肿、渗血、疼痛加重和异常气味。无头晕和出血增多时逐步增加活动，并在医护指导下开始轻柔盆底肌训练。哺乳方面建议尽早母婴皮肤接触和按需哺乳，指导正确含接，乳胀时先热敷或轻柔按摩再哺乳，乳头疼痛时及时调整姿势。若出现乳房局部红肿硬痛、寒战或高热，应警惕乳腺炎。因有胎儿窘迫、羊水污染或新生儿评分变化记录，母婴同室期间应配合观察新生儿吃奶、肤色、体温、呼吸和反应，发现吃奶差、发绀、嗜睡或呼吸异常应立即告知儿科医生。合并子宫肌瘤、卵巢囊肿或多囊卵巢相关情况时，产后复查时建议结合妇科超声评估，若腹痛、包块感或异常出血加重，应提前就诊。出院前应完成用药、伤口、哺乳和避孕宣教，42天左右复查，必要时提前复诊。若出现阴道流血突然增多、鲜红血浸湿卫生巾、恶露恶臭、发热、持续腹痛、伤口化脓、排尿困难、下肢单侧肿痛、胸闷气短，或持续悲伤焦虑及伤害自己或婴儿的想法，应立即就医或急诊处理。 | 患者29岁，初产妇，自然分娩+I度裂伤，合并多囊卵巢综合征、妊娠合并右侧卵巢囊肿、可疑胎儿宫内窘迫，脐带绕颈1周、脐带捻转60周。产后6周B超复查卵巢囊肿，如急性腹痛、发热警惕囊肿扭转。PCOS产后需控制体重，监测血糖、血脂，6周复查性激素。脐带捻转60周+绕颈+可疑窘迫，新生儿需密切观察神经系统及呼吸。I度裂伤温水坐浴。出血250ml正常。PCOS可能泌乳延迟，鼓励勤吸吮。提供心理支持。6周复查B超、性激素、血糖；新生儿随访。 |
| **护理计划英文翻译 English translation of care plan** | The patient is 29 years old, G1P1, delivered at 39+2 weeks by spontaneous vaginal delivery, with an estimated intrapartum blood loss of 250 mL. The newborn was a male infant, weighed 3020 g, and had Apgar scores of 10, 10, 10. The main case-based risks include fetal distress or abnormal amniotic fluid findings. Within the first 24 hours, assess temperature, pulse, blood pressure, uterine fundal height, uterine tone, vaginal bleeding, lochia color, urinary retention, first voiding, and pain score. During hospitalization and after discharge, continue to observe whether lochia gradually changes from red to lighter color, whether breast engorgement or nipple fissure occurs, and whether sleep and mood remain stable. Diet should include high-quality protein, iron-rich food, vegetables, fruit, and adequate fluids. Avoid blind over-supplementation and prolonged bed rest. Because this was a spontaneous vaginal birth, recovery of any perineal tear or suture site should be observed. Keep the area clean and dry after urination or bowel movement and watch for redness, swelling, bleeding, worsening pain, or abnormal odor. If there is no dizziness or increased bleeding, increase activity gradually and begin gentle pelvic floor exercises under professional guidance. For breastfeeding, encourage early skin-to-skin contact and feeding on demand. Teach correct latch and positioning. For engorgement, apply warmth or gentle massage before feeding. If nipple pain occurs, adjust latch promptly. Localized breast redness, hard painful swelling, chills, or high fever should raise concern for mastitis. Because fetal distress, meconium-stained fluid, or changed Apgar scores were recorded, cooperate with pediatric observation of feeding, skin color, temperature, breathing, and responsiveness. Poor feeding, cyanosis, excessive sleepiness, or abnormal breathing should be reported immediately. For uterine fibroids, ovarian cysts, or polycystic ovary related findings, pelvic ultrasound can be considered at postpartum review. Earlier assessment is needed for worsening abdominal pain, mass sensation, or abnormal bleeding. Before discharge, provide education on medication, wound care, breastfeeding, and contraception. Arrange a postpartum review around 42 days after delivery, or earlier if symptoms occur. Seek urgent medical care for sudden heavy vaginal bleeding, bright red bleeding soaking pads, foul-smelling lochia, fever, persistent abdominal pain, pus from the wound, difficulty urinating, unilateral leg swelling or pain, chest tightness, shortness of breath, or persistent sadness, anxiety, or thoughts of harming herself or the baby. | The patient is a 29-year-old primipara with spontaneous delivery and first-degree laceration. Comorbidities: PCOS, right ovarian cyst during pregnancy, suspected fetal distress, nuchal cord 1 loop, cord twisting 60 loops. Repeat ovarian ultrasound at 6 weeks; if acute pain/fever, suspect torsion. PCOS – weight control, monitor glucose/lipids; check hormones at 6 weeks. Extreme cord twisting – close newborn neurological and respiratory monitoring. Warm sitz baths. Blood loss 250 mL, normal. PCOS may delay lactation – encourage frequent nursing. Psychological support. Follow-up: ultrasound, hormones, glucose at 6 weeks; newborn follow-up. |
| **Clinical appropriateness 临床适当性** | 5 | 4 |
| **Individualization 个体化程度** | 4 | 4 |
| **Comprehensiveness 完整性** | 5 | 4 |
| **Actionability 可操作性** | 4 | 4 |
| **Safety 安全性** | 5 | 4 |
| **Triage appropriateness 分诊/转诊建议适当性** | 5 | 4 |
| **Structure and clarity 结构与清晰度** | 4 | 4 |

**病例 36 / Case 36**

| **项目 Item** | **ChatGPT 5.5** | **DeepSeek V3** |
| --- | --- | --- |
| **去标识化临床病例摘要 De-identified Clinical Case Summary** | 患者40岁，孕产史为G2P2，既往剖宫产0次，孕周41，临产类别为普贝生。自然破膜，羊水量约300ml，羊水性质为Ⅲ°。以自然分娩分娩，产时出血约470ml。第一产程2时50分，第二产程0时39分，第三产程0时11分，总产程3时40分，会阴或切口情况为Ⅰ，内缝。合并症及主要风险因素：高龄经产妇妊娠监督。新生儿出生体重3400g，Apgar评分1、5、10分钟分别为10、10、10分，分娩结局为活产。 | |
| **临床病例摘要英文翻译 English Translation of De-identified Clinical Case Summary** | Maternal age was 40 years. The obstetric history was G2P2, with 0 previous cesarean deliveries. Gestational age was 41 weeks, and the labor onset or induction category was vaginal dinoprostone (Propess) induction. The membranes ruptured spontaneously; the amniotic fluid volume was approximately 300 mL and its character was grade III meconium-stained. The patient had a spontaneous vaginal delivery, with estimated intrapartum blood loss of 470 mL. The first, second, and third stages of labor were 2 h 50 min, 0 h 39 min, and 0 h 11 min, respectively; the total labor duration was 3 h 40 min. A first-degree perineal laceration was repaired with internal sutures. Documented comorbidities and major risk factors were advanced maternal age in a multipara. Neonatal birth weight was 3400 g; Apgar scores at 1, 5, and 10 minutes were 10, 10, 10; and the delivery outcome was live birth. | |
| **模型生成的产褥期护理计划 Model-generated postpartum nursing care plan** | 产妇40岁，G2P2，孕41周，经阴道自然分娩分娩，产时出血约470ml，新生儿女婴、体重3400g，Apgar评分10、10、10分。病例提示的重点风险为高龄妊娠因素。产后24小时内重点评估体温、脉搏、血压、宫底高度、宫缩、阴道流血、恶露颜色和尿潴留，记录首次排尿和疼痛评分。产后住院及出院后继续观察恶露是否由红色逐渐转淡，乳房是否胀痛，乳头是否皲裂，睡眠和情绪是否稳定。饮食以优质蛋白、含铁食物、蔬菜水果和足量饮水为主，避免盲目大补和长期卧床。本次为阴道自然分娩，应重点观察会阴裂伤或缝合处恢复。每次大小便后保持局部清洁干燥，观察红肿、渗血、疼痛加重和异常气味。无头晕和出血增多时逐步增加活动，并在医护指导下开始轻柔盆底肌训练。哺乳方面建议尽早母婴皮肤接触和按需哺乳，指导正确含接，乳胀时先热敷或轻柔按摩再哺乳，乳头疼痛时及时调整姿势。若出现乳房局部红肿硬痛、寒战或高热，应警惕乳腺炎。产时出血量需要持续观察，建议每日记录恶露量、颜色和血块情况，活动后若明显增多，应减少活动并请医护评估。高龄产妇恢复期应避免过度劳累和连续熬夜，家属应参与夜间照护，保证睡眠、营养和情绪支持。出院前应完成用药、伤口、哺乳和避孕宣教，42天左右复查，必要时提前复诊。若出现阴道流血突然增多、鲜红血浸湿卫生巾、恶露恶臭、发热、持续腹痛、伤口化脓、排尿困难、下肢单侧肿痛、胸闷气短，或持续悲伤焦虑及伤害自己或婴儿的想法，应立即就医或急诊处理。 | 患者40岁，G2P2，自然分娩+I度裂伤，合并高龄经产妇，羊水Ⅲ°污染，产时出血470ml。出血470ml接近产后出血阈值，需严密观察恶露量、宫底高度，警惕晚期出血。Ⅲ°羊水污染，新生儿需监测胎粪吸入综合征，即使Apgar正常，建议观察24-48小时。监测产妇体温、恶露防感染。高龄经产妇宫缩可能乏力，加强按摩。I度裂伤温水坐浴。鼓励母乳喂养。提供心理支持。42天复查；新生儿随访。 |
| **护理计划英文翻译 English translation of care plan** | The patient is 40 years old, G2P2, delivered at 41 weeks by spontaneous vaginal delivery, with an estimated intrapartum blood loss of 470 mL. The newborn was a female infant, weighed 3400 g, and had Apgar scores of 10, 10, 10. The main case-based risks include advanced maternal age. Within the first 24 hours, assess temperature, pulse, blood pressure, uterine fundal height, uterine tone, vaginal bleeding, lochia color, urinary retention, first voiding, and pain score. During hospitalization and after discharge, continue to observe whether lochia gradually changes from red to lighter color, whether breast engorgement or nipple fissure occurs, and whether sleep and mood remain stable. Diet should include high-quality protein, iron-rich food, vegetables, fruit, and adequate fluids. Avoid blind over-supplementation and prolonged bed rest. Because this was a spontaneous vaginal birth, recovery of any perineal tear or suture site should be observed. Keep the area clean and dry after urination or bowel movement and watch for redness, swelling, bleeding, worsening pain, or abnormal odor. If there is no dizziness or increased bleeding, increase activity gradually and begin gentle pelvic floor exercises under professional guidance. For breastfeeding, encourage early skin-to-skin contact and feeding on demand. Teach correct latch and positioning. For engorgement, apply warmth or gentle massage before feeding. If nipple pain occurs, adjust latch promptly. Localized breast redness, hard painful swelling, chills, or high fever should raise concern for mastitis. Because blood loss requires closer observation, record the amount, color, and clots in the lochia every day. If bleeding increases clearly after activity, reduce activity and seek clinical assessment. Advanced maternal age may slow recovery, so the family should help with nighttime care and support adequate sleep, nutrition, and emotional stability. Before discharge, provide education on medication, wound care, breastfeeding, and contraception. Arrange a postpartum review around 42 days after delivery, or earlier if symptoms occur. Seek urgent medical care for sudden heavy vaginal bleeding, bright red bleeding soaking pads, foul-smelling lochia, fever, persistent abdominal pain, pus from the wound, difficulty urinating, unilateral leg swelling or pain, chest tightness, shortness of breath, or persistent sadness, anxiety, or thoughts of harming herself or the baby. | The patient is a 40-year-old G2P2 with spontaneous delivery and first-degree laceration. Comorbidities: advanced age multipara, grade III meconium-stained fluid, blood loss 470 mL (near PPH threshold). Close monitoring of lochia and fundal height; watch for late PPH. Newborn – monitor for MAS for 24-48 hours. Monitor maternal temperature and lochia for infection. Advanced age may cause uterine atony – aggressive fundal massage. Warm sitz baths. Encourage breastfeeding. Psychological support. Follow-up at 42 days; newborn follow-up. |
| **Clinical appropriateness 临床适当性** | 4 | 4 |
| **Individualization 个体化程度** | 4 | 5 |
| **Comprehensiveness 完整性** | 4 | 4 |
| **Actionability 可操作性** | 4 | 4 |
| **Safety 安全性** | 5 | 4 |
| **Triage appropriateness 分诊/转诊建议适当性** | 5 | 3 |
| **Structure and clarity 结构与清晰度** | 4 | 4 |

**病例 37 / Case 37**

| **项目 Item** | **ChatGPT 5.5** | **DeepSeek V3** |
| --- | --- | --- |
| **去标识化临床病例摘要 De-identified Clinical Case Summary** | 患者45岁，孕产史为G2P2，既往剖宫产0次，孕周39+4，临产类别为阴道放置米索。自然破膜，羊水量约50ml，羊水性质为Ⅲ°（质粘稠）。以自然分娩分娩，产时出血约330ml。第一产程6时15分，第二产程0时40分，第三产程0时10分，总产程7时5分，会阴或切口情况为Ⅰ，内缝。合并症及主要风险因素：甲状腺功能减退症；高龄经产妇妊娠监督；胎膜早破；胎儿窘迫？。新生儿出生体重3700g，Apgar评分1、5、10分钟分别为8、8、9分，分娩结局为活产。 | |
| **临床病例摘要英文翻译 English Translation of De-identified Clinical Case Summary** | Maternal age was 45 years. The obstetric history was G2P2, with 0 previous cesarean deliveries. Gestational age was 39+4 weeks, and the labor onset or induction category was vaginal misoprostol induction. The membranes ruptured spontaneously; the amniotic fluid volume was approximately 50 mL and its character was thick grade III meconium-stained. The patient had a spontaneous vaginal delivery, with estimated intrapartum blood loss of 330 mL. The first, second, and third stages of labor were 6 h 15 min, 0 h 40 min, and 0 h 10 min, respectively; the total labor duration was 7 h 5 min. A first-degree perineal laceration was repaired with internal sutures. Documented comorbidities and major risk factors were hypothyroidism; advanced maternal age in a multipara; premature rupture of membranes; suspected fetal distress. Neonatal birth weight was 3700 g; Apgar scores at 1, 5, and 10 minutes were 8, 8, 9; and the delivery outcome was live birth. | |
| **模型生成的产褥期护理计划 Model-generated postpartum nursing care plan** | 产妇45岁，G2P2，孕39+4周，经阴道自然分娩分娩，产时出血约330ml，新生儿女婴、体重3700g，Apgar评分8、8、9分。病例提示的重点风险为胎膜早破或感染相关风险、甲状腺功能异常、胎儿窘迫或羊水异常记录、高龄妊娠因素。产后24小时内重点评估体温、脉搏、血压、宫底高度、宫缩、阴道流血、恶露颜色和尿潴留，记录首次排尿和疼痛评分。产后住院及出院后继续观察恶露是否由红色逐渐转淡，乳房是否胀痛，乳头是否皲裂，睡眠和情绪是否稳定。饮食以优质蛋白、含铁食物、蔬菜水果和足量饮水为主，避免盲目大补和长期卧床。本次为阴道自然分娩，应重点观察会阴裂伤或缝合处恢复。每次大小便后保持局部清洁干燥，观察红肿、渗血、疼痛加重和异常气味。无头晕和出血增多时逐步增加活动，并在医护指导下开始轻柔盆底肌训练。哺乳方面建议尽早母婴皮肤接触和按需哺乳，指导正确含接，乳胀时先热敷或轻柔按摩再哺乳，乳头疼痛时及时调整姿势。若出现乳房局部红肿硬痛、寒战或高热，应警惕乳腺炎。合并甲状腺疾病者，应按内分泌医嘱继续或调整用药，不自行停药，产后复查甲状腺功能，观察心悸、怕冷、乏力、出汗和情绪异常。存在胎膜早破、B族链球菌感染、阴道炎或宫内感染疑虑时，应加强体温、下腹压痛、恶露气味、会阴或切口渗液观察，发热或恶露恶臭时及时复诊。因有胎儿窘迫、羊水污染或新生儿评分变化记录，母婴同室期间应配合观察新生儿吃奶、肤色、体温、呼吸和反应，发现吃奶差、发绀、嗜睡或呼吸异常应立即告知儿科医生。高龄产妇恢复期应避免过度劳累和连续熬夜，家属应参与夜间照护，保证睡眠、营养和情绪支持。出院前应完成用药、伤口、哺乳和避孕宣教，42天左右复查，必要时提前复诊。若出现阴道流血突然增多、鲜红血浸湿卫生巾、恶露恶臭、发热、持续腹痛、伤口化脓、排尿困难、下肢单侧肿痛、胸闷气短，或持续悲伤焦虑及伤害自己或婴儿的想法，应立即就医或急诊处理。 | 患者45岁，G2P2，自然分娩+I度裂伤，合并甲减、高龄经产妇、胎膜早破、可疑胎儿窘迫，羊水Ⅲ°（质粘稠），新生儿Apgar 8-8-9。Ⅲ°粘稠羊水+可疑窘迫+Apgar 8分，新生儿需密切监测胎粪吸入综合征及缺氧缺血性脑病，建议新生儿科留观。继续左甲状腺素，6周复查。PPROM+粘稠羊水感染风险高，监测产妇体温、恶露、子宫压痛。产时出血330ml，注意晚期出血。高龄、新生儿评分偏低，焦虑风险极高，提供心理支持。产后1周复查甲功、血常规；新生儿专科随访。 |
| **护理计划英文翻译 English translation of care plan** | The patient is 45 years old, G2P2, delivered at 39+4 weeks by spontaneous vaginal delivery, with an estimated intrapartum blood loss of 330 mL. The newborn was a female infant, weighed 3700 g, and had Apgar scores of 8, 8, 9. The main case-based risks include premature rupture of membranes or infection-related risk, thyroid dysfunction, fetal distress or abnormal amniotic fluid findings, advanced maternal age. Within the first 24 hours, assess temperature, pulse, blood pressure, uterine fundal height, uterine tone, vaginal bleeding, lochia color, urinary retention, first voiding, and pain score. During hospitalization and after discharge, continue to observe whether lochia gradually changes from red to lighter color, whether breast engorgement or nipple fissure occurs, and whether sleep and mood remain stable. Diet should include high-quality protein, iron-rich food, vegetables, fruit, and adequate fluids. Avoid blind over-supplementation and prolonged bed rest. Because this was a spontaneous vaginal birth, recovery of any perineal tear or suture site should be observed. Keep the area clean and dry after urination or bowel movement and watch for redness, swelling, bleeding, worsening pain, or abnormal odor. If there is no dizziness or increased bleeding, increase activity gradually and begin gentle pelvic floor exercises under professional guidance. For breastfeeding, encourage early skin-to-skin contact and feeding on demand. Teach correct latch and positioning. For engorgement, apply warmth or gentle massage before feeding. If nipple pain occurs, adjust latch promptly. Localized breast redness, hard painful swelling, chills, or high fever should raise concern for mastitis. For thyroid disease, continue or adjust medication only under endocrine guidance, recheck thyroid function after delivery, and watch for palpitations, cold intolerance, fatigue, sweating, or abnormal mood changes. With premature rupture of membranes, group B streptococcus, vaginitis, or suspected intrauterine infection, monitor temperature, lower abdominal tenderness, lochia odor, and drainage from the perineal wound or incision. Return promptly for fever or foul-smelling lochia. Because fetal distress, meconium-stained fluid, or changed Apgar scores were recorded, cooperate with pediatric observation of feeding, skin color, temperature, breathing, and responsiveness. Poor feeding, cyanosis, excessive sleepiness, or abnormal breathing should be reported immediately. Advanced maternal age may slow recovery, so the family should help with nighttime care and support adequate sleep, nutrition, and emotional stability. Before discharge, provide education on medication, wound care, breastfeeding, and contraception. Arrange a postpartum review around 42 days after delivery, or earlier if symptoms occur. Seek urgent medical care for sudden heavy vaginal bleeding, bright red bleeding soaking pads, foul-smelling lochia, fever, persistent abdominal pain, pus from the wound, difficulty urinating, unilateral leg swelling or pain, chest tightness, shortness of breath, or persistent sadness, anxiety, or thoughts of harming herself or the baby. | The patient is a 45-year-old G2P2 with spontaneous delivery and first-degree laceration. Comorbidities: hypothyroidism, advanced age multipara, PPROM, suspected fetal distress, viscous grade III meconium. Newborn Apgar 8-8-9 – monitor for MAS and HIE; consider NICU observation. Continue levothyroxine, retest at 6 weeks. High infection risk – monitor maternal temperature, lochia, uterine tenderness. Blood loss 330 mL – watch for late PPH. Extremely high anxiety due to age and lower Apgar – provide psychological support. Check thyroid and CBC at 1 week; newborn specialist follow-up. |
| **Clinical appropriateness 临床适当性** | 5 | 4 |
| **Individualization 个体化程度** | 5 | 5 |
| **Comprehensiveness 完整性** | 5 | 4 |
| **Actionability 可操作性** | 5 | 4 |
| **Safety 安全性** | 5 | 4 |
| **Triage appropriateness 分诊/转诊建议适当性** | 5 | 4 |
| **Structure and clarity 结构与清晰度** | 4 | 3 |

**病例 38 / Case 38**

| **项目 Item** | **ChatGPT 5.5** | **DeepSeek V3** |
| --- | --- | --- |
| **去标识化临床病例摘要 De-identified Clinical Case Summary** | 患者31岁，孕产史为G2P1，既往剖宫产0次，孕周39+6，临产类别为自然临产。自然破膜，羊水量约300ml，羊水性质为Ⅲ°。以产钳分娩，产时出血约400ml。第一产程3时8分，第二产程0时22分，第三产程0时2分，总产程3时32分，会阴或切口情况为侧切，内缝。合并症及主要风险因素：胎儿宫内窘迫？；妊娠期贫血；胎膜早破；妊娠合并子宫肌瘤？。新生儿出生体重3050g，Apgar评分1、5、10分钟分别为10、10、10分，分娩结局为活产。 | |
| **临床病例摘要英文翻译 English Translation of De-identified Clinical Case Summary** | Maternal age was 31 years. The obstetric history was G2P1, with 0 previous cesarean deliveries. Gestational age was 39+6 weeks, and the labor onset or induction category was spontaneous labor. The membranes ruptured spontaneously; the amniotic fluid volume was approximately 300 mL and its character was grade III meconium-stained. The patient had a forceps-assisted vaginal delivery, with estimated intrapartum blood loss of 400 mL. The first, second, and third stages of labor were 3 h 8 min, 0 h 22 min, and 0 h 2 min, respectively; the total labor duration was 3 h 32 min. An episiotomy was repaired with internal sutures. Documented comorbidities and major risk factors were suspected fetal distress; anemia during pregnancy; premature rupture of membranes; suspected uterine fibroid during pregnancy. Neonatal birth weight was 3050 g; Apgar scores at 1, 5, and 10 minutes were 10, 10, 10; and the delivery outcome was live birth. | |
| **模型生成的产褥期护理计划 Model-generated postpartum nursing care plan** | 产妇31岁，G2P1，孕39+6周，经产钳助产阴道分娩分娩，产时出血约400ml，新生儿女婴、体重3050g，Apgar评分10、10、10分。病例提示的重点风险为胎膜早破或感染相关风险、出血或贫血相关风险、胎儿窘迫或羊水异常记录。产后24小时内重点评估体温、脉搏、血压、宫底高度、宫缩、阴道流血、恶露颜色和尿潴留，记录首次排尿和疼痛评分。产后住院及出院后继续观察恶露是否由红色逐渐转淡，乳房是否胀痛，乳头是否皲裂，睡眠和情绪是否稳定。饮食以优质蛋白、含铁食物、蔬菜水果和足量饮水为主，避免盲目大补和长期卧床。本次为产钳助产阴道分娩，会阴伤口、盆底和排尿功能是护理重点。每次大小便后用温水从前向后清洁会阴并轻轻擦干，观察侧切或裂伤处肿胀、硬结、渗血、分泌物和异味。疼痛明显时可侧卧休息，避免久蹲、负重和过早剧烈活动。哺乳方面建议尽早母婴皮肤接触和按需哺乳，指导正确含接，乳胀时先热敷或轻柔按摩再哺乳，乳头疼痛时及时调整姿势。若出现乳房局部红肿硬痛、寒战或高热，应警惕乳腺炎。因存在出血量偏多、贫血或血小板异常相关风险，应复查血常规，关注头晕、心悸、乏力、面色苍白、恶露突然增多或较大血块，按医嘱补铁，必要时评估凝血和子宫复旧。存在胎膜早破、B族链球菌感染、阴道炎或宫内感染疑虑时，应加强体温、下腹压痛、恶露气味、会阴或切口渗液观察，发热或恶露恶臭时及时复诊。因有胎儿窘迫、羊水污染或新生儿评分变化记录，母婴同室期间应配合观察新生儿吃奶、肤色、体温、呼吸和反应，发现吃奶差、发绀、嗜睡或呼吸异常应立即告知儿科医生。合并子宫肌瘤、卵巢囊肿或多囊卵巢相关情况时，产后复查时建议结合妇科超声评估，若腹痛、包块感或异常出血加重，应提前就诊。出院前应完成用药、伤口、哺乳和避孕宣教，42天左右复查，必要时提前复诊。若出现阴道流血突然增多、鲜红血浸湿卫生巾、恶露恶臭、发热、持续腹痛、伤口化脓、排尿困难、下肢单侧肿痛、胸闷气短，或持续悲伤焦虑及伤害自己或婴儿的想法，应立即就医或急诊处理。 | 患者31岁，G2P1，产钳助产+侧切，合并可疑胎儿宫内窘迫、妊娠期贫血、胎膜早破、可疑子宫肌瘤，羊水Ⅲ°污染。贫血需产后复查血常规，补充铁剂、维生素C，注意疲劳、心悸。Ⅲ°羊水+可疑窘迫，新生儿监测胎粪吸入综合征。PPROM+羊水污染感染风险高，监测产妇体温。会阴侧切每日消毒2次。可疑子宫肌瘤产后B超确认，观察子宫复旧。产时出血400ml，需警惕。贫血可能影响乳汁分泌，鼓励勤吸吮。产后1周复查血常规；6周B超；新生儿随访。 |
| **护理计划英文翻译 English translation of care plan** | The patient is 31 years old, G2P1, delivered at 39+6 weeks by forceps-assisted vaginal delivery, with an estimated intrapartum blood loss of 400 mL. The newborn was a female infant, weighed 3050 g, and had Apgar scores of 10, 10, 10. The main case-based risks include premature rupture of membranes or infection-related risk, bleeding or anemia-related risk, fetal distress or abnormal amniotic fluid findings. Within the first 24 hours, assess temperature, pulse, blood pressure, uterine fundal height, uterine tone, vaginal bleeding, lochia color, urinary retention, first voiding, and pain score. During hospitalization and after discharge, continue to observe whether lochia gradually changes from red to lighter color, whether breast engorgement or nipple fissure occurs, and whether sleep and mood remain stable. Diet should include high-quality protein, iron-rich food, vegetables, fruit, and adequate fluids. Avoid blind over-supplementation and prolonged bed rest. Because this was a forceps-assisted vaginal delivery, perineal wound care, pelvic floor recovery, and urinary function are priorities. Rinse the perineum from front to back with warm water after urination or bowel movement and pat dry. Observe the episiotomy or tear for swelling, induration, bleeding, discharge, and odor. Rest in a lateral position if pain is marked, and avoid prolonged squatting, heavy lifting, and early vigorous activity. For breastfeeding, encourage early skin-to-skin contact and feeding on demand. Teach correct latch and positioning. For engorgement, apply warmth or gentle massage before feeding. If nipple pain occurs, adjust latch promptly. Localized breast redness, hard painful swelling, chills, or high fever should raise concern for mastitis. Because increased blood loss, anemia, or platelet abnormality is a concern, repeat a complete blood count as advised and watch for dizziness, palpitations, severe fatigue, pallor, sudden increase in lochia, or large clots. Iron supplementation, nutrition support, and assessment of uterine involution or coagulation should follow medical orders. With premature rupture of membranes, group B streptococcus, vaginitis, or suspected intrauterine infection, monitor temperature, lower abdominal tenderness, lochia odor, and drainage from the perineal wound or incision. Return promptly for fever or foul-smelling lochia. Because fetal distress, meconium-stained fluid, or changed Apgar scores were recorded, cooperate with pediatric observation of feeding, skin color, temperature, breathing, and responsiveness. Poor feeding, cyanosis, excessive sleepiness, or abnormal breathing should be reported immediately. For uterine fibroids, ovarian cysts, or polycystic ovary related findings, pelvic ultrasound can be considered at postpartum review. Earlier assessment is needed for worsening abdominal pain, mass sensation, or abnormal bleeding. Before discharge, provide education on medication, wound care, breastfeeding, and contraception. Arrange a postpartum review around 42 days after delivery, or earlier if symptoms occur. Seek urgent medical care for sudden heavy vaginal bleeding, bright red bleeding soaking pads, foul-smelling lochia, fever, persistent abdominal pain, pus from the wound, difficulty urinating, unilateral leg swelling or pain, chest tightness, shortness of breath, or persistent sadness, anxiety, or thoughts of harming herself or the baby. | The patient is a 31-year-old G2P1 with forceps delivery and episiotomy. Comorbidities: suspected fetal distress, anemia during pregnancy, PPROM, suspected uterine fibroid, grade III meconium. Postpartum CBC, iron and vitamin C supplementation. Newborn – monitor for MAS. High infection risk – monitor maternal temperature. Episiotomy care. Suspected fibroid – postpartum ultrasound, monitor involution. Blood loss 400 mL – watch for late PPH. Anemia may affect milk supply – encourage frequent nursing. Follow-up: CBC at 1 week; ultrasound at 6 weeks; newborn follow-up. |
| **Clinical appropriateness 临床适当性** | 5 | 4 |
| **Individualization 个体化程度** | 5 | 5 |
| **Comprehensiveness 完整性** | 5 | 4 |
| **Actionability 可操作性** | 5 | 4 |
| **Safety 安全性** | 5 | 4 |
| **Triage appropriateness 分诊/转诊建议适当性** | 5 | 4 |
| **Structure and clarity 结构与清晰度** | 4 | 3 |

**病例 39 / Case 39**

| **项目 Item** | **ChatGPT 5.5** | **DeepSeek V3** |
| --- | --- | --- |
| **去标识化临床病例摘要 De-identified Clinical Case Summary** | 患者30岁，孕产史为G1P1，既往剖宫产0次，孕周38+1，临产类别为自然临产。自然破膜，羊水量约300ml，羊水性质为清。以自然分娩分娩，产时出血约190ml。第一产程5时53分，第二产程0时58分，第三产程0时4分，总产程6时55分，会阴或切口情况为Ⅰ，内缝。合并症及主要风险因素：无。新生儿出生体重2890g，Apgar评分1、5、10分钟分别为10、10、10分，分娩结局为活产。 | |
| **临床病例摘要英文翻译 English Translation of De-identified Clinical Case Summary** | Maternal age was 30 years. The obstetric history was G1P1, with 0 previous cesarean deliveries. Gestational age was 38+1 weeks, and the labor onset or induction category was spontaneous labor. The membranes ruptured spontaneously; the amniotic fluid volume was approximately 300 mL and its character was clear. The patient had a spontaneous vaginal delivery, with estimated intrapartum blood loss of 190 mL. The first, second, and third stages of labor were 5 h 53 min, 0 h 58 min, and 0 h 4 min, respectively; the total labor duration was 6 h 55 min. A first-degree perineal laceration was repaired with internal sutures. No comorbidities or major risk factors were documented. Neonatal birth weight was 2890 g; Apgar scores at 1, 5, and 10 minutes were 10, 10, 10; and the delivery outcome was live birth. | |
| **模型生成的产褥期护理计划 Model-generated postpartum nursing care plan** | 产妇30岁，G1P1，孕38+1周，经阴道自然分娩分娩，产时出血约190ml，新生儿男婴、体重2890g，Apgar评分10、10、10分。病例提示的重点风险为未记录特殊合并症。产后24小时内重点评估体温、脉搏、血压、宫底高度、宫缩、阴道流血、恶露颜色和尿潴留，记录首次排尿和疼痛评分。产后住院及出院后继续观察恶露是否由红色逐渐转淡，乳房是否胀痛，乳头是否皲裂，睡眠和情绪是否稳定。饮食以优质蛋白、含铁食物、蔬菜水果和足量饮水为主，避免盲目大补和长期卧床。本次为阴道自然分娩，应重点观察会阴裂伤或缝合处恢复。每次大小便后保持局部清洁干燥，观察红肿、渗血、疼痛加重和异常气味。无头晕和出血增多时逐步增加活动，并在医护指导下开始轻柔盆底肌训练。哺乳方面建议尽早母婴皮肤接触和按需哺乳，指导正确含接，乳胀时先热敷或轻柔按摩再哺乳，乳头疼痛时及时调整姿势。若出现乳房局部红肿硬痛、寒战或高热，应警惕乳腺炎。目前未记录特殊合并症，仍需按常规产褥期管理执行，重点是子宫复旧、恶露、伤口、哺乳、营养、活动和心理状态。出院前应完成用药、伤口、哺乳和避孕宣教，42天左右复查，必要时提前复诊。若出现阴道流血突然增多、鲜红血浸湿卫生巾、恶露恶臭、发热、持续腹痛、伤口化脓、排尿困难、下肢单侧肿痛、胸闷气短，或持续悲伤焦虑及伤害自己或婴儿的想法，应立即就医或急诊处理。 | 患者30岁，初产妇，自然分娩+I度裂伤，无合并症。常规产褥护理：观察恶露、子宫复旧；会阴温水坐浴；按需哺乳；产后尽早下床活动；多饮水防便秘；注意产后情绪；产后42天复查；避孕指导。该病例简单，但需保证基础护理全面。 |
| **护理计划英文翻译 English translation of care plan** | The patient is 30 years old, G1P1, delivered at 38+1 weeks by spontaneous vaginal delivery, with an estimated intrapartum blood loss of 190 mL. The newborn was a male infant, weighed 2890 g, and had Apgar scores of 10, 10, 10. The main case-based risks include no special comorbidity was recorded. Within the first 24 hours, assess temperature, pulse, blood pressure, uterine fundal height, uterine tone, vaginal bleeding, lochia color, urinary retention, first voiding, and pain score. During hospitalization and after discharge, continue to observe whether lochia gradually changes from red to lighter color, whether breast engorgement or nipple fissure occurs, and whether sleep and mood remain stable. Diet should include high-quality protein, iron-rich food, vegetables, fruit, and adequate fluids. Avoid blind over-supplementation and prolonged bed rest. Because this was a spontaneous vaginal birth, recovery of any perineal tear or suture site should be observed. Keep the area clean and dry after urination or bowel movement and watch for redness, swelling, bleeding, worsening pain, or abnormal odor. If there is no dizziness or increased bleeding, increase activity gradually and begin gentle pelvic floor exercises under professional guidance. For breastfeeding, encourage early skin-to-skin contact and feeding on demand. Teach correct latch and positioning. For engorgement, apply warmth or gentle massage before feeding. If nipple pain occurs, adjust latch promptly. Localized breast redness, hard painful swelling, chills, or high fever should raise concern for mastitis. No special complication was recorded, but routine postpartum management should still focus on uterine involution, lochia, wounds, breastfeeding, nutrition, activity, and mental health. Before discharge, provide education on medication, wound care, breastfeeding, and contraception. Arrange a postpartum review around 42 days after delivery, or earlier if symptoms occur. Seek urgent medical care for sudden heavy vaginal bleeding, bright red bleeding soaking pads, foul-smelling lochia, fever, persistent abdominal pain, pus from the wound, difficulty urinating, unilateral leg swelling or pain, chest tightness, shortness of breath, or persistent sadness, anxiety, or thoughts of harming herself or the baby. | The patient is a 30-year-old primipara with spontaneous delivery and first-degree laceration. No comorbidities. Routine care: monitor lochia and uterine involution; warm sitz baths; breastfeed on demand; early ambulation; fluids and fiber to prevent constipation; monitor mood; postpartum check at 42 days; contraception counseling. |
| **Clinical appropriateness 临床适当性** | 4 | 3 |
| **Individualization 个体化程度** | 3 | 3 |
| **Comprehensiveness 完整性** | 4 | 3 |
| **Actionability 可操作性** | 4 | 3 |
| **Safety 安全性** | 5 | 3 |
| **Triage appropriateness 分诊/转诊建议适当性** | 5 | 3 |
| **Structure and clarity 结构与清晰度** | 3 | 4 |

**病例 40 / Case 40**

| **项目 Item** | **ChatGPT 5.5** | **DeepSeek V3** |
| --- | --- | --- |
| **去标识化临床病例摘要 De-identified Clinical Case Summary** | 患者29岁，孕产史为G1P1，既往剖宫产0次，孕周38+3，临产类别为自然临产。自然破膜，羊水量约300ml，羊水性质为血性。以产钳分娩，产时出血约190ml。第一产程4时15分，第二产程2时30分，第三产程0时5分，总产程6时50分，会阴或切口情况为侧切，内缝。合并症及主要风险因素：胎膜早破、胎儿窘迫？。新生儿出生体重3230g，Apgar评分1、5、10分钟分别为10、10、10分，分娩结局为活产。 | |
| **临床病例摘要英文翻译 English Translation of De-identified Clinical Case Summary** | Maternal age was 29 years. The obstetric history was G1P1, with 0 previous cesarean deliveries. Gestational age was 38+3 weeks, and the labor onset or induction category was spontaneous labor. The membranes ruptured spontaneously; the amniotic fluid volume was approximately 300 mL and its character was blood-stained. The patient had a forceps-assisted vaginal delivery, with estimated intrapartum blood loss of 190 mL. The first, second, and third stages of labor were 4 h 15 min, 2 h 30 min, and 0 h 5 min, respectively; the total labor duration was 6 h 50 min. An episiotomy was repaired with internal sutures. Documented comorbidities and major risk factors were premature rupture of membranes; suspected fetal distress. Neonatal birth weight was 3230 g; Apgar scores at 1, 5, and 10 minutes were 10, 10, 10; and the delivery outcome was live birth. | |
| **模型生成的产褥期护理计划 Model-generated postpartum nursing care plan** | 产妇29岁，G1P1，孕38+3周，经产钳助产阴道分娩分娩，产时出血约190ml，新生儿女婴、体重3230g，Apgar评分10、10、10分。病例提示的重点风险为胎膜早破或感染相关风险、胎儿窘迫或羊水异常记录。产后24小时内重点评估体温、脉搏、血压、宫底高度、宫缩、阴道流血、恶露颜色和尿潴留，记录首次排尿和疼痛评分。产后住院及出院后继续观察恶露是否由红色逐渐转淡，乳房是否胀痛，乳头是否皲裂，睡眠和情绪是否稳定。饮食以优质蛋白、含铁食物、蔬菜水果和足量饮水为主，避免盲目大补和长期卧床。本次为产钳助产阴道分娩，会阴伤口、盆底和排尿功能是护理重点。每次大小便后用温水从前向后清洁会阴并轻轻擦干，观察侧切或裂伤处肿胀、硬结、渗血、分泌物和异味。疼痛明显时可侧卧休息，避免久蹲、负重和过早剧烈活动。哺乳方面建议尽早母婴皮肤接触和按需哺乳，指导正确含接，乳胀时先热敷或轻柔按摩再哺乳，乳头疼痛时及时调整姿势。若出现乳房局部红肿硬痛、寒战或高热，应警惕乳腺炎。存在胎膜早破、B族链球菌感染、阴道炎或宫内感染疑虑时，应加强体温、下腹压痛、恶露气味、会阴或切口渗液观察，发热或恶露恶臭时及时复诊。因有胎儿窘迫、羊水污染或新生儿评分变化记录，母婴同室期间应配合观察新生儿吃奶、肤色、体温、呼吸和反应，发现吃奶差、发绀、嗜睡或呼吸异常应立即告知儿科医生。出院前应完成用药、伤口、哺乳和避孕宣教，42天左右复查，必要时提前复诊。若出现阴道流血突然增多、鲜红血浸湿卫生巾、恶露恶臭、发热、持续腹痛、伤口化脓、排尿困难、下肢单侧肿痛、胸闷气短，或持续悲伤焦虑及伤害自己或婴儿的想法，应立即就医或急诊处理。 | 患者29岁，初产妇，产钳助产+侧切，合并胎膜早破、可疑胎儿窘迫、脐带隐性脱垂，羊水血性。血性羊水提示产前出血（如胎盘早剥、前置血管破裂），产后需监测产妇凝血功能、子宫收缩，观察有无腹痛加剧、阴道持续出血。隐性脱垂+血性羊水+可疑窘迫，新生儿需密切监测生命体征、有无贫血及神经系统。会阴侧切每日消毒2次。PPROM监测感染。产时出血190ml，仍警惕晚期出血。鼓励母乳喂养。提供心理支持。产后1周复查血常规；新生儿随访。 |
| **护理计划英文翻译 English translation of care plan** | The patient is 29 years old, G1P1, delivered at 38+3 weeks by forceps-assisted vaginal delivery, with an estimated intrapartum blood loss of 190 mL. The newborn was a female infant, weighed 3230 g, and had Apgar scores of 10, 10, 10. The main case-based risks include premature rupture of membranes or infection-related risk, fetal distress or abnormal amniotic fluid findings. Within the first 24 hours, assess temperature, pulse, blood pressure, uterine fundal height, uterine tone, vaginal bleeding, lochia color, urinary retention, first voiding, and pain score. During hospitalization and after discharge, continue to observe whether lochia gradually changes from red to lighter color, whether breast engorgement or nipple fissure occurs, and whether sleep and mood remain stable. Diet should include high-quality protein, iron-rich food, vegetables, fruit, and adequate fluids. Avoid blind over-supplementation and prolonged bed rest. Because this was a forceps-assisted vaginal delivery, perineal wound care, pelvic floor recovery, and urinary function are priorities. Rinse the perineum from front to back with warm water after urination or bowel movement and pat dry. Observe the episiotomy or tear for swelling, induration, bleeding, discharge, and odor. Rest in a lateral position if pain is marked, and avoid prolonged squatting, heavy lifting, and early vigorous activity. For breastfeeding, encourage early skin-to-skin contact and feeding on demand. Teach correct latch and positioning. For engorgement, apply warmth or gentle massage before feeding. If nipple pain occurs, adjust latch promptly. Localized breast redness, hard painful swelling, chills, or high fever should raise concern for mastitis. With premature rupture of membranes, group B streptococcus, vaginitis, or suspected intrauterine infection, monitor temperature, lower abdominal tenderness, lochia odor, and drainage from the perineal wound or incision. Return promptly for fever or foul-smelling lochia. Because fetal distress, meconium-stained fluid, or changed Apgar scores were recorded, cooperate with pediatric observation of feeding, skin color, temperature, breathing, and responsiveness. Poor feeding, cyanosis, excessive sleepiness, or abnormal breathing should be reported immediately. Before discharge, provide education on medication, wound care, breastfeeding, and contraception. Arrange a postpartum review around 42 days after delivery, or earlier if symptoms occur. Seek urgent medical care for sudden heavy vaginal bleeding, bright red bleeding soaking pads, foul-smelling lochia, fever, persistent abdominal pain, pus from the wound, difficulty urinating, unilateral leg swelling or pain, chest tightness, shortness of breath, or persistent sadness, anxiety, or thoughts of harming herself or the baby. | The patient is a 29-year-old primipara with forceps delivery and episiotomy. Comorbidities: PPROM, suspected fetal distress, occult cord prolapse, bloody amniotic fluid. Bloody fluid suggests antepartum hemorrhage (abruption, vasa previa) – monitor maternal coagulation, uterine contraction, persistent bleeding. Newborn – monitor vital signs, anemia, neurology. Episiotomy care. Monitor infection. Blood loss 190 mL – still watch for late PPH. Encourage breastfeeding. Psychological support. Follow-up: CBC at 1 week; newborn follow-up. |
| **Clinical appropriateness 临床适当性** | 5 | 4 |
| **Individualization 个体化程度** | 4 | 5 |
| **Comprehensiveness 完整性** | 5 | 4 |
| **Actionability 可操作性** | 4 | 4 |
| **Safety 安全性** | 5 | 4 |
| **Triage appropriateness 分诊/转诊建议适当性** | 5 | 4 |
| **Structure and clarity 结构与清晰度** | 4 | 4 |

**病例 41 / Case 41**

| **项目 Item** | **ChatGPT 5.5** | **DeepSeek V3** |
| --- | --- | --- |
| **去标识化临床病例摘要 De-identified Clinical Case Summary** | 患者31岁，孕产史为G1P1，既往剖宫产0次，孕周38+3，临产类别为自然临产。人工破膜，羊水量约300ml，羊水性质为血性。以产钳分娩，产时出血约430ml。第一产程7时40分，第二产程0时10分，第三产程0时10分，总产程8时0分，会阴或切口情况为Ⅰ，内缝。合并症及主要风险因素：胎儿宫内窘迫？；羊水污染I度；胎心监护异常。新生儿出生体重2690g，Apgar评分1、5、10分钟分别为10、10、10分，分娩结局为活产。 | |
| **临床病例摘要英文翻译 English Translation of De-identified Clinical Case Summary** | Maternal age was 31 years. The obstetric history was G1P1, with 0 previous cesarean deliveries. Gestational age was 38+3 weeks, and the labor onset or induction category was spontaneous labor. The membranes were artificially ruptured; the amniotic fluid volume was approximately 300 mL and its character was blood-stained. The patient had a forceps-assisted vaginal delivery, with estimated intrapartum blood loss of 430 mL. The first, second, and third stages of labor were 7 h 40 min, 0 h 10 min, and 0 h 10 min, respectively; the total labor duration was 8 h 0 min. A first-degree perineal laceration was repaired with internal sutures. Documented comorbidities and major risk factors were suspected fetal distress; grade I meconium-stained amniotic fluid; abnormal fetal heart rate monitoring. Neonatal birth weight was 2690 g; Apgar scores at 1, 5, and 10 minutes were 10, 10, 10; and the delivery outcome was live birth. | |
| **模型生成的产褥期护理计划 Model-generated postpartum nursing care plan** | 产妇31岁，G1P1，孕38+3周，经产钳助产阴道分娩分娩，产时出血约430ml，新生儿男婴、体重2690g，Apgar评分10、10、10分。病例提示的重点风险为胎儿窘迫或羊水异常记录。产后24小时内重点评估体温、脉搏、血压、宫底高度、宫缩、阴道流血、恶露颜色和尿潴留，记录首次排尿和疼痛评分。产后住院及出院后继续观察恶露是否由红色逐渐转淡，乳房是否胀痛，乳头是否皲裂，睡眠和情绪是否稳定。饮食以优质蛋白、含铁食物、蔬菜水果和足量饮水为主，避免盲目大补和长期卧床。本次为产钳助产阴道分娩，会阴伤口、盆底和排尿功能是护理重点。每次大小便后用温水从前向后清洁会阴并轻轻擦干，观察侧切或裂伤处肿胀、硬结、渗血、分泌物和异味。疼痛明显时可侧卧休息，避免久蹲、负重和过早剧烈活动。哺乳方面建议尽早母婴皮肤接触和按需哺乳，指导正确含接，乳胀时先热敷或轻柔按摩再哺乳，乳头疼痛时及时调整姿势。若出现乳房局部红肿硬痛、寒战或高热，应警惕乳腺炎。产时出血量需要持续观察，建议每日记录恶露量、颜色和血块情况，活动后若明显增多，应减少活动并请医护评估。因有胎儿窘迫、羊水污染或新生儿评分变化记录，母婴同室期间应配合观察新生儿吃奶、肤色、体温、呼吸和反应，发现吃奶差、发绀、嗜睡或呼吸异常应立即告知儿科医生。出院前应完成用药、伤口、哺乳和避孕宣教，42天左右复查，必要时提前复诊。若出现阴道流血突然增多、鲜红血浸湿卫生巾、恶露恶臭、发热、持续腹痛、伤口化脓、排尿困难、下肢单侧肿痛、胸闷气短，或持续悲伤焦虑及伤害自己或婴儿的想法，应立即就医或急诊处理。 | 患者31岁，初产妇，产钳助产+I度裂伤，合并胎儿宫内窘迫、羊水污染I度、胎心监护异常，脐带绕颈1周（紧），产时出血430ml。新生儿需密切监测呼吸、肌张力、反射，排除缺氧缺血性脑病，观察24-48小时。出血430ml，监测生命体征、宫底高度，预防晚期出血。会阴伤口注意血肿、感染。羊水污染I度，监测产妇感染。鼓励母乳喂养。提供心理支持。高蛋白饮食，补充铁剂。产后1周复查血常规；新生儿专科随访。 |
| **护理计划英文翻译 English translation of care plan** | The patient is 31 years old, G1P1, delivered at 38+3 weeks by forceps-assisted vaginal delivery, with an estimated intrapartum blood loss of 430 mL. The newborn was a male infant, weighed 2690 g, and had Apgar scores of 10, 10, 10. The main case-based risks include fetal distress or abnormal amniotic fluid findings. Within the first 24 hours, assess temperature, pulse, blood pressure, uterine fundal height, uterine tone, vaginal bleeding, lochia color, urinary retention, first voiding, and pain score. During hospitalization and after discharge, continue to observe whether lochia gradually changes from red to lighter color, whether breast engorgement or nipple fissure occurs, and whether sleep and mood remain stable. Diet should include high-quality protein, iron-rich food, vegetables, fruit, and adequate fluids. Avoid blind over-supplementation and prolonged bed rest. Because this was a forceps-assisted vaginal delivery, perineal wound care, pelvic floor recovery, and urinary function are priorities. Rinse the perineum from front to back with warm water after urination or bowel movement and pat dry. Observe the episiotomy or tear for swelling, induration, bleeding, discharge, and odor. Rest in a lateral position if pain is marked, and avoid prolonged squatting, heavy lifting, and early vigorous activity. For breastfeeding, encourage early skin-to-skin contact and feeding on demand. Teach correct latch and positioning. For engorgement, apply warmth or gentle massage before feeding. If nipple pain occurs, adjust latch promptly. Localized breast redness, hard painful swelling, chills, or high fever should raise concern for mastitis. Because blood loss requires closer observation, record the amount, color, and clots in the lochia every day. If bleeding increases clearly after activity, reduce activity and seek clinical assessment. Because fetal distress, meconium-stained fluid, or changed Apgar scores were recorded, cooperate with pediatric observation of feeding, skin color, temperature, breathing, and responsiveness. Poor feeding, cyanosis, excessive sleepiness, or abnormal breathing should be reported immediately. Before discharge, provide education on medication, wound care, breastfeeding, and contraception. Arrange a postpartum review around 42 days after delivery, or earlier if symptoms occur. Seek urgent medical care for sudden heavy vaginal bleeding, bright red bleeding soaking pads, foul-smelling lochia, fever, persistent abdominal pain, pus from the wound, difficulty urinating, unilateral leg swelling or pain, chest tightness, shortness of breath, or persistent sadness, anxiety, or thoughts of harming herself or the baby. | The patient is a 31-year-old primipara with forceps delivery and first-degree laceration. Comorbidities: fetal distress, grade I meconium, abnormal FHR, tight nuchal cord 1 loop. Blood loss 430 mL. Newborn – monitor for HIE for 24-48 hours. Monitor vital signs and fundus for PPH. Perineal wound – watch for hematoma/infection. Monitor maternal infection. Encourage breastfeeding. Psychological support. High-protein diet, iron. Follow-up: CBC at 1 week; newborn specialist follow-up. |
| **Clinical appropriateness 临床适当性** | 5 | 4 |
| **Individualization 个体化程度** | 4 | 5 |
| **Comprehensiveness 完整性** | 5 | 4 |
| **Actionability 可操作性** | 4 | 4 |
| **Safety 安全性** | 5 | 4 |
| **Triage appropriateness 分诊/转诊建议适当性** | 5 | 4 |
| **Structure and clarity 结构与清晰度** | 4 | 4 |

**病例 42 / Case 42**

| **项目 Item** | **ChatGPT 5.5** | **DeepSeek V3** |
| --- | --- | --- |
| **去标识化临床病例摘要 De-identified Clinical Case Summary** | 患者32岁，孕产史为G4P2，既往剖宫产0次，孕周39，临产类别为自然临产。自然破膜，羊水量约400ml，羊水性质为清。以自然分娩分娩，产时出血约290ml。第一产程3时55分，第二产程0时9分，第三产程0时31分，总产程4时35分，会阴或切口情况为Ⅰ，内缝。合并症及主要风险因素：无。新生儿出生体重2740g，Apgar评分1、5、10分钟分别为10、10、10分，分娩结局为活产。 | |
| **临床病例摘要英文翻译 English Translation of De-identified Clinical Case Summary** | Maternal age was 32 years. The obstetric history was G4P2, with 0 previous cesarean deliveries. Gestational age was 39 weeks, and the labor onset or induction category was spontaneous labor. The membranes ruptured spontaneously; the amniotic fluid volume was approximately 400 mL and its character was clear. The patient had a spontaneous vaginal delivery, with estimated intrapartum blood loss of 290 mL. The first, second, and third stages of labor were 3 h 55 min, 0 h 9 min, and 0 h 31 min, respectively; the total labor duration was 4 h 35 min. A first-degree perineal laceration was repaired with internal sutures. No comorbidities or major risk factors were documented. Neonatal birth weight was 2740 g; Apgar scores at 1, 5, and 10 minutes were 10, 10, 10; and the delivery outcome was live birth. | |
| **模型生成的产褥期护理计划 Model-generated postpartum nursing care plan** | 产妇32岁，G4P2，孕39周，经阴道自然分娩分娩，产时出血约290ml，新生儿男婴、体重2740g，Apgar评分10、10、10分。病例提示的重点风险为未记录特殊合并症。产后24小时内重点评估体温、脉搏、血压、宫底高度、宫缩、阴道流血、恶露颜色和尿潴留，记录首次排尿和疼痛评分。产后住院及出院后继续观察恶露是否由红色逐渐转淡，乳房是否胀痛，乳头是否皲裂，睡眠和情绪是否稳定。饮食以优质蛋白、含铁食物、蔬菜水果和足量饮水为主，避免盲目大补和长期卧床。本次为阴道自然分娩，应重点观察会阴裂伤或缝合处恢复。每次大小便后保持局部清洁干燥，观察红肿、渗血、疼痛加重和异常气味。无头晕和出血增多时逐步增加活动，并在医护指导下开始轻柔盆底肌训练。哺乳方面建议尽早母婴皮肤接触和按需哺乳，指导正确含接，乳胀时先热敷或轻柔按摩再哺乳，乳头疼痛时及时调整姿势。若出现乳房局部红肿硬痛、寒战或高热，应警惕乳腺炎。目前未记录特殊合并症，仍需按常规产褥期管理执行，重点是子宫复旧、恶露、伤口、哺乳、营养、活动和心理状态。出院前应完成用药、伤口、哺乳和避孕宣教，42天左右复查，必要时提前复诊。若出现阴道流血突然增多、鲜红血浸湿卫生巾、恶露恶臭、发热、持续腹痛、伤口化脓、排尿困难、下肢单侧肿痛、胸闷气短，或持续悲伤焦虑及伤害自己或婴儿的想法，应立即就医或急诊处理。 | 患者32岁，G4P2，自然分娩+I度裂伤，无合并症。常规护理：观察恶露，温水坐浴，按需哺乳，早下床，多饮水防便秘，注意情绪，42天复查，避孕指导（G4P2建议长效可逆避孕）。此病例简单，但需确保基础护理全面。 |
| **护理计划英文翻译 English translation of care plan** | The patient is 32 years old, G4P2, delivered at 39 weeks by spontaneous vaginal delivery, with an estimated intrapartum blood loss of 290 mL. The newborn was a male infant, weighed 2740 g, and had Apgar scores of 10, 10, 10. The main case-based risks include no special comorbidity was recorded. Within the first 24 hours, assess temperature, pulse, blood pressure, uterine fundal height, uterine tone, vaginal bleeding, lochia color, urinary retention, first voiding, and pain score. During hospitalization and after discharge, continue to observe whether lochia gradually changes from red to lighter color, whether breast engorgement or nipple fissure occurs, and whether sleep and mood remain stable. Diet should include high-quality protein, iron-rich food, vegetables, fruit, and adequate fluids. Avoid blind over-supplementation and prolonged bed rest. Because this was a spontaneous vaginal birth, recovery of any perineal tear or suture site should be observed. Keep the area clean and dry after urination or bowel movement and watch for redness, swelling, bleeding, worsening pain, or abnormal odor. If there is no dizziness or increased bleeding, increase activity gradually and begin gentle pelvic floor exercises under professional guidance. For breastfeeding, encourage early skin-to-skin contact and feeding on demand. Teach correct latch and positioning. For engorgement, apply warmth or gentle massage before feeding. If nipple pain occurs, adjust latch promptly. Localized breast redness, hard painful swelling, chills, or high fever should raise concern for mastitis. No special complication was recorded, but routine postpartum management should still focus on uterine involution, lochia, wounds, breastfeeding, nutrition, activity, and mental health. Before discharge, provide education on medication, wound care, breastfeeding, and contraception. Arrange a postpartum review around 42 days after delivery, or earlier if symptoms occur. Seek urgent medical care for sudden heavy vaginal bleeding, bright red bleeding soaking pads, foul-smelling lochia, fever, persistent abdominal pain, pus from the wound, difficulty urinating, unilateral leg swelling or pain, chest tightness, shortness of breath, or persistent sadness, anxiety, or thoughts of harming herself or the baby. | The patient is a 32-year-old G4P2 with spontaneous delivery and first-degree laceration. No comorbidities. Routine care: lochia monitoring, warm sitz baths, breastfeeding on demand, early ambulation, fluids and fiber, mood monitoring, 42-day check, contraception counseling (recommend LARC for G4P2). |
| **Clinical appropriateness 临床适当性** | 4 | 3 |
| **Individualization 个体化程度** | 3 | 3 |
| **Comprehensiveness 完整性** | 4 | 3 |
| **Actionability 可操作性** | 4 | 3 |
| **Safety 安全性** | 5 | 3 |
| **Triage appropriateness 分诊/转诊建议适当性** | 5 | 3 |
| **Structure and clarity 结构与清晰度** | 3 | 4 |

**病例 43 / Case 43**

| **项目 Item** | **ChatGPT 5.5** | **DeepSeek V3** |
| --- | --- | --- |
| **去标识化临床病例摘要 De-identified Clinical Case Summary** | 患者45岁，孕产史为G2P1，既往剖宫产0次，孕周40，临产类别为阴道放置米索。自然破膜，羊水量约100ml，羊水性质为Ⅲ°（质粘稠）。以产钳分娩，产时出血约410ml。第一产程12时50分，第二产程0时37分，第三产程0时6分，总产程13时33分，会阴或切口情况为侧切，内缝。合并症及主要风险因素：胎动减少（好转）；高龄初孕妇的监督；羊水污染III度；胎儿宫内窘迫？。新生儿出生体重3150g，Apgar评分1、5、10分钟分别为10、10、10分，分娩结局为活产。 | |
| **临床病例摘要英文翻译 English Translation of De-identified Clinical Case Summary** | Maternal age was 45 years. The obstetric history was G2P1, with 0 previous cesarean deliveries. Gestational age was 40 weeks, and the labor onset or induction category was vaginal misoprostol induction. The membranes ruptured spontaneously; the amniotic fluid volume was approximately 100 mL and its character was thick grade III meconium-stained. The patient had a forceps-assisted vaginal delivery, with estimated intrapartum blood loss of 410 mL. The first, second, and third stages of labor were 12 h 50 min, 0 h 37 min, and 0 h 6 min, respectively; the total labor duration was 13 h 33 min. An episiotomy was repaired with internal sutures. Documented comorbidities and major risk factors were reduced fetal movement (improved); advanced maternal age in a primigravida; grade III meconium-stained amniotic fluid; suspected fetal distress. Neonatal birth weight was 3150 g; Apgar scores at 1, 5, and 10 minutes were 10, 10, 10; and the delivery outcome was live birth. | |
| **模型生成的产褥期护理计划 Model-generated postpartum nursing care plan** | 产妇45岁，G2P1，孕40周，经产钳助产阴道分娩分娩，产时出血约410ml，新生儿女婴、体重3150g，Apgar评分10、10、10分。病例提示的重点风险为胎儿窘迫或羊水异常记录、高龄妊娠因素。产后24小时内重点评估体温、脉搏、血压、宫底高度、宫缩、阴道流血、恶露颜色和尿潴留，记录首次排尿和疼痛评分。产后住院及出院后继续观察恶露是否由红色逐渐转淡，乳房是否胀痛，乳头是否皲裂，睡眠和情绪是否稳定。饮食以优质蛋白、含铁食物、蔬菜水果和足量饮水为主，避免盲目大补和长期卧床。本次为产钳助产阴道分娩，会阴伤口、盆底和排尿功能是护理重点。每次大小便后用温水从前向后清洁会阴并轻轻擦干，观察侧切或裂伤处肿胀、硬结、渗血、分泌物和异味。疼痛明显时可侧卧休息，避免久蹲、负重和过早剧烈活动。哺乳方面建议尽早母婴皮肤接触和按需哺乳，指导正确含接，乳胀时先热敷或轻柔按摩再哺乳，乳头疼痛时及时调整姿势。若出现乳房局部红肿硬痛、寒战或高热，应警惕乳腺炎。产时出血量需要持续观察，建议每日记录恶露量、颜色和血块情况，活动后若明显增多，应减少活动并请医护评估。因有胎儿窘迫、羊水污染或新生儿评分变化记录，母婴同室期间应配合观察新生儿吃奶、肤色、体温、呼吸和反应，发现吃奶差、发绀、嗜睡或呼吸异常应立即告知儿科医生。高龄产妇恢复期应避免过度劳累和连续熬夜，家属应参与夜间照护，保证睡眠、营养和情绪支持。出院前应完成用药、伤口、哺乳和避孕宣教，42天左右复查，必要时提前复诊。若出现阴道流血突然增多、鲜红血浸湿卫生巾、恶露恶臭、发热、持续腹痛、伤口化脓、排尿困难、下肢单侧肿痛、胸闷气短，或持续悲伤焦虑及伤害自己或婴儿的想法，应立即就医或急诊处理。 | 患者45岁，G2P1，产钳助产+侧切，合并高龄初产妇、羊水污染III度（质粘稠）、胎动减少（好转）、可疑胎儿窘迫，产时出血410ml，新生儿Apgar 10。III度粘稠羊水+可疑窘迫，新生儿需监测胎粪吸入综合征、低氧血症，建议儿科留观24-48小时。出血410ml，注意宫缩乏力，加强宫底按摩。会阴侧切每日消毒，年龄大愈合慢，注意营养。粘稠羊水增加感染风险，监测产妇体温。高龄注意血压、血糖，预防血栓。年龄大可能泌乳延迟，提供哺乳支持。焦虑风险高，心理支持。产后1周复查血常规；42天全面评估。 |
| **护理计划英文翻译 English translation of care plan** | The patient is 45 years old, G2P1, delivered at 40 weeks by forceps-assisted vaginal delivery, with an estimated intrapartum blood loss of 410 mL. The newborn was a female infant, weighed 3150 g, and had Apgar scores of 10, 10, 10. The main case-based risks include fetal distress or abnormal amniotic fluid findings, advanced maternal age. Within the first 24 hours, assess temperature, pulse, blood pressure, uterine fundal height, uterine tone, vaginal bleeding, lochia color, urinary retention, first voiding, and pain score. During hospitalization and after discharge, continue to observe whether lochia gradually changes from red to lighter color, whether breast engorgement or nipple fissure occurs, and whether sleep and mood remain stable. Diet should include high-quality protein, iron-rich food, vegetables, fruit, and adequate fluids. Avoid blind over-supplementation and prolonged bed rest. Because this was a forceps-assisted vaginal delivery, perineal wound care, pelvic floor recovery, and urinary function are priorities. Rinse the perineum from front to back with warm water after urination or bowel movement and pat dry. Observe the episiotomy or tear for swelling, induration, bleeding, discharge, and odor. Rest in a lateral position if pain is marked, and avoid prolonged squatting, heavy lifting, and early vigorous activity. For breastfeeding, encourage early skin-to-skin contact and feeding on demand. Teach correct latch and positioning. For engorgement, apply warmth or gentle massage before feeding. If nipple pain occurs, adjust latch promptly. Localized breast redness, hard painful swelling, chills, or high fever should raise concern for mastitis. Because blood loss requires closer observation, record the amount, color, and clots in the lochia every day. If bleeding increases clearly after activity, reduce activity and seek clinical assessment. Because fetal distress, meconium-stained fluid, or changed Apgar scores were recorded, cooperate with pediatric observation of feeding, skin color, temperature, breathing, and responsiveness. Poor feeding, cyanosis, excessive sleepiness, or abnormal breathing should be reported immediately. Advanced maternal age may slow recovery, so the family should help with nighttime care and support adequate sleep, nutrition, and emotional stability. Before discharge, provide education on medication, wound care, breastfeeding, and contraception. Arrange a postpartum review around 42 days after delivery, or earlier if symptoms occur. Seek urgent medical care for sudden heavy vaginal bleeding, bright red bleeding soaking pads, foul-smelling lochia, fever, persistent abdominal pain, pus from the wound, difficulty urinating, unilateral leg swelling or pain, chest tightness, shortness of breath, or persistent sadness, anxiety, or thoughts of harming herself or the baby. | The patient is a 45-year-old G2P1 with forceps delivery and episiotomy. Comorbidities: advanced maternal age in a primigravida, grade III viscous meconium, decreased fetal movement (improved), suspected fetal distress. Blood loss 410 mL, newborn Apgar 10. Monitor newborn for MAS and hypoxemia for 24-48 hours. Aggressive fundal massage for atony. Episiotomy care – slower healing due to age. High infection risk – monitor temperature. Advanced age – monitor BP, glucose; prevent thrombosis. Delayed lactation possible – provide breastfeeding support. High anxiety – psychological support. Follow-up: CBC at 1 week; comprehensive exam at 42 days. |
| **Clinical appropriateness 临床适当性** | 5 | 4 |
| **Individualization 个体化程度** | 5 | 5 |
| **Comprehensiveness 完整性** | 5 | 4 |
| **Actionability 可操作性** | 4 | 4 |
| **Safety 安全性** | 5 | 4 |
| **Triage appropriateness 分诊/转诊建议适当性** | 5 | 4 |
| **Structure and clarity 结构与清晰度** | 4 | 4 |

**病例 44 / Case 44**

| **项目 Item** | **ChatGPT 5.5** | **DeepSeek V3** |
| --- | --- | --- |
| **去标识化临床病例摘要 De-identified Clinical Case Summary** | 患者39岁，孕产史为G1P1，既往剖宫产0次，孕周35+1，临产类别为自然临产。自然破膜，羊水量约300ml，羊水性质为清。以自然分娩分娩，产时出血约240ml。第一产程4时25分，第二产程1时6分，第三产程0时9分，总产程5时40分，会阴或切口情况为侧切，内缝。合并症及主要风险因素：胎儿宫内窘迫？绒毛膜羊膜炎？未足月胎膜早破、妊娠合并甲状腺功能减退、妊娠期贫血（轻度）、妊娠合并高度近视。新生儿出生体重2510g，Apgar评分1、5、10分钟分别为9、9、9分，分娩结局为活产。 | |
| **临床病例摘要英文翻译 English Translation of De-identified Clinical Case Summary** | Maternal age was 39 years. The obstetric history was G1P1, with 0 previous cesarean deliveries. Gestational age was 35+1 weeks, and the labor onset or induction category was spontaneous labor. The membranes ruptured spontaneously; the amniotic fluid volume was approximately 300 mL and its character was clear. The patient had a spontaneous vaginal delivery, with estimated intrapartum blood loss of 240 mL. The first, second, and third stages of labor were 4 h 25 min, 1 h 6 min, and 0 h 9 min, respectively; the total labor duration was 5 h 40 min. An episiotomy was repaired with internal sutures. Documented comorbidities and major risk factors were suspected fetal distress; suspected chorioamnionitis; preterm premature rupture of membranes; hypothyroidism during pregnancy; anemia during pregnancy (mild); high myopia. Neonatal birth weight was 2510 g; Apgar scores at 1, 5, and 10 minutes were 9, 9, 9; and the delivery outcome was live birth. | |
| **模型生成的产褥期护理计划 Model-generated postpartum nursing care plan** | 产妇39岁，G1P1，孕35+1周，经阴道自然分娩分娩，产时出血约240ml，新生儿男婴、体重2510g，Apgar评分9、9、9分。病例提示的重点风险为胎膜早破或感染相关风险、出血或贫血相关风险、甲状腺功能异常、胎儿窘迫或羊水异常记录、高龄妊娠因素。产后24小时内重点评估体温、脉搏、血压、宫底高度、宫缩、阴道流血、恶露颜色和尿潴留，记录首次排尿和疼痛评分。产后住院及出院后继续观察恶露是否由红色逐渐转淡，乳房是否胀痛，乳头是否皲裂，睡眠和情绪是否稳定。饮食以优质蛋白、含铁食物、蔬菜水果和足量饮水为主，避免盲目大补和长期卧床。本次为阴道自然分娩，应重点观察会阴裂伤或缝合处恢复。每次大小便后保持局部清洁干燥，观察红肿、渗血、疼痛加重和异常气味。无头晕和出血增多时逐步增加活动，并在医护指导下开始轻柔盆底肌训练。哺乳方面建议尽早母婴皮肤接触和按需哺乳，指导正确含接，乳胀时先热敷或轻柔按摩再哺乳，乳头疼痛时及时调整姿势。若出现乳房局部红肿硬痛、寒战或高热，应警惕乳腺炎。因存在出血量偏多、贫血或血小板异常相关风险，应复查血常规，关注头晕、心悸、乏力、面色苍白、恶露突然增多或较大血块，按医嘱补铁，必要时评估凝血和子宫复旧。合并甲状腺疾病者，应按内分泌医嘱继续或调整用药，不自行停药，产后复查甲状腺功能，观察心悸、怕冷、乏力、出汗和情绪异常。存在胎膜早破、B族链球菌感染、阴道炎或宫内感染疑虑时，应加强体温、下腹压痛、恶露气味、会阴或切口渗液观察，发热或恶露恶臭时及时复诊。因有胎儿窘迫、羊水污染或新生儿评分变化记录，母婴同室期间应配合观察新生儿吃奶、肤色、体温、呼吸和反应，发现吃奶差、发绀、嗜睡或呼吸异常应立即告知儿科医生。出院前应完成用药、伤口、哺乳和避孕宣教，42天左右复查，必要时提前复诊。若出现阴道流血突然增多、鲜红血浸湿卫生巾、恶露恶臭、发热、持续腹痛、伤口化脓、排尿困难、下肢单侧肿痛、胸闷气短，或持续悲伤焦虑及伤害自己或婴儿的想法，应立即就医或急诊处理。 | 患者39岁，初产妇，自然分娩+侧切，孕35+1周早产，合并可疑胎儿窘迫、绒毛膜羊膜炎（可疑）、未足月胎膜早破、甲减、轻度贫血、高度近视，新生儿体重2510g（低出生体重），Apgar 9-9-9。早产儿需保暖、监测血糖、黄疸、呼吸暂停，鼓励母乳+强化剂。可疑绒毛膜羊膜炎+PPROM需抗生素治疗，监测产妇体温、子宫压痛；新生儿防感染。继续左甲状腺素，6周复查。补充铁剂。高度近视避免用力屏气。会阴侧切注意感染。早产+感染致焦虑，心理支持。产后1周复查甲功、血常规；早产儿随访。 |
| **护理计划英文翻译 English translation of care plan** | The patient is 39 years old, G1P1, delivered at 35+1 weeks by spontaneous vaginal delivery, with an estimated intrapartum blood loss of 240 mL. The newborn was a male infant, weighed 2510 g, and had Apgar scores of 9, 9, 9. The main case-based risks include premature rupture of membranes or infection-related risk, bleeding or anemia-related risk, thyroid dysfunction, fetal distress or abnormal amniotic fluid findings, advanced maternal age. Within the first 24 hours, assess temperature, pulse, blood pressure, uterine fundal height, uterine tone, vaginal bleeding, lochia color, urinary retention, first voiding, and pain score. During hospitalization and after discharge, continue to observe whether lochia gradually changes from red to lighter color, whether breast engorgement or nipple fissure occurs, and whether sleep and mood remain stable. Diet should include high-quality protein, iron-rich food, vegetables, fruit, and adequate fluids. Avoid blind over-supplementation and prolonged bed rest. Because this was a spontaneous vaginal birth, recovery of any perineal tear or suture site should be observed. Keep the area clean and dry after urination or bowel movement and watch for redness, swelling, bleeding, worsening pain, or abnormal odor. If there is no dizziness or increased bleeding, increase activity gradually and begin gentle pelvic floor exercises under professional guidance. For breastfeeding, encourage early skin-to-skin contact and feeding on demand. Teach correct latch and positioning. For engorgement, apply warmth or gentle massage before feeding. If nipple pain occurs, adjust latch promptly. Localized breast redness, hard painful swelling, chills, or high fever should raise concern for mastitis. Because increased blood loss, anemia, or platelet abnormality is a concern, repeat a complete blood count as advised and watch for dizziness, palpitations, severe fatigue, pallor, sudden increase in lochia, or large clots. Iron supplementation, nutrition support, and assessment of uterine involution or coagulation should follow medical orders. For thyroid disease, continue or adjust medication only under endocrine guidance, recheck thyroid function after delivery, and watch for palpitations, cold intolerance, fatigue, sweating, or abnormal mood changes. With premature rupture of membranes, group B streptococcus, vaginitis, or suspected intrauterine infection, monitor temperature, lower abdominal tenderness, lochia odor, and drainage from the perineal wound or incision. Return promptly for fever or foul-smelling lochia. Because fetal distress, meconium-stained fluid, or changed Apgar scores were recorded, cooperate with pediatric observation of feeding, skin color, temperature, breathing, and responsiveness. Poor feeding, cyanosis, excessive sleepiness, or abnormal breathing should be reported immediately. Before discharge, provide education on medication, wound care, breastfeeding, and contraception. Arrange a postpartum review around 42 days after delivery, or earlier if symptoms occur. Seek urgent medical care for sudden heavy vaginal bleeding, bright red bleeding soaking pads, foul-smelling lochia, fever, persistent abdominal pain, pus from the wound, difficulty urinating, unilateral leg swelling or pain, chest tightness, shortness of breath, or persistent sadness, anxiety, or thoughts of harming herself or the baby. | The patient is a 39-year-old primipara with spontaneous delivery and episiotomy at 35+1 weeks. Comorbidities: suspected fetal distress, suspected chorioamnionitis, PPROM, hypothyroidism, mild anemia, high myopia. Newborn LBW 2510 g, Apgar 9-9-9. Preterm infant care: warmth, glucose, jaundice, apnea; breastfeeding with fortifier. Antibiotics for suspected chorio+PPROM. Monitor maternal infection. Continue levothyroxine, retest at 6 weeks. Iron supplementation. High myopia – avoid Valsalva. Episiotomy care. Psychological support. Follow-up: thyroid and CBC at 1 week; preterm infant follow-up. |
| **Clinical appropriateness 临床适当性** | 5 | 4 |
| **Individualization 个体化程度** | 5 | 5 |
| **Comprehensiveness 完整性** | 5 | 4 |
| **Actionability 可操作性** | 5 | 4 |
| **Safety 安全性** | 5 | 4 |
| **Triage appropriateness 分诊/转诊建议适当性** | 5 | 4 |
| **Structure and clarity 结构与清晰度** | 4 | 3 |

**病例 45 / Case 45**

| **项目 Item** | **ChatGPT 5.5** | **DeepSeek V3** |
| --- | --- | --- |
| **去标识化临床病例摘要 De-identified Clinical Case Summary** | 患者32岁，孕产史为G1P1，既往剖宫产0次，孕周40，临产类别为自然临产。自然破膜，羊水量约500ml，羊水性质为清。以自然分娩分娩，产时出血约180ml。第一产程8时4分，第二产程0时23分，第三产程0时8分，总产程8时35分，会阴或切口情况为Ⅰ，内缝。合并症及主要风险因素：无。新生儿出生体重2950g，Apgar评分1、5、10分钟分别为10、10、10分，分娩结局为活产。 | |
| **临床病例摘要英文翻译 English Translation of De-identified Clinical Case Summary** | Maternal age was 32 years. The obstetric history was G1P1, with 0 previous cesarean deliveries. Gestational age was 40 weeks, and the labor onset or induction category was spontaneous labor. The membranes ruptured spontaneously; the amniotic fluid volume was approximately 500 mL and its character was clear. The patient had a spontaneous vaginal delivery, with estimated intrapartum blood loss of 180 mL. The first, second, and third stages of labor were 8 h 4 min, 0 h 23 min, and 0 h 8 min, respectively; the total labor duration was 8 h 35 min. A first-degree perineal laceration was repaired with internal sutures. No comorbidities or major risk factors were documented. Neonatal birth weight was 2950 g; Apgar scores at 1, 5, and 10 minutes were 10, 10, 10; and the delivery outcome was live birth. | |
| **模型生成的产褥期护理计划 Model-generated postpartum nursing care plan** | 产妇32岁，G1P1，孕40周，经阴道自然分娩分娩，产时出血约180ml，新生儿女婴、体重2950g，Apgar评分10、10、10分。病例提示的重点风险为未记录特殊合并症。产后24小时内重点评估体温、脉搏、血压、宫底高度、宫缩、阴道流血、恶露颜色和尿潴留，记录首次排尿和疼痛评分。产后住院及出院后继续观察恶露是否由红色逐渐转淡，乳房是否胀痛，乳头是否皲裂，睡眠和情绪是否稳定。饮食以优质蛋白、含铁食物、蔬菜水果和足量饮水为主，避免盲目大补和长期卧床。本次为阴道自然分娩，应重点观察会阴裂伤或缝合处恢复。每次大小便后保持局部清洁干燥，观察红肿、渗血、疼痛加重和异常气味。无头晕和出血增多时逐步增加活动，并在医护指导下开始轻柔盆底肌训练。哺乳方面建议尽早母婴皮肤接触和按需哺乳，指导正确含接，乳胀时先热敷或轻柔按摩再哺乳，乳头疼痛时及时调整姿势。若出现乳房局部红肿硬痛、寒战或高热，应警惕乳腺炎。目前未记录特殊合并症，仍需按常规产褥期管理执行，重点是子宫复旧、恶露、伤口、哺乳、营养、活动和心理状态。出院前应完成用药、伤口、哺乳和避孕宣教，42天左右复查，必要时提前复诊。若出现阴道流血突然增多、鲜红血浸湿卫生巾、恶露恶臭、发热、持续腹痛、伤口化脓、排尿困难、下肢单侧肿痛、胸闷气短，或持续悲伤焦虑及伤害自己或婴儿的想法，应立即就医或急诊处理。 | 患者32岁，初产妇，自然分娩+I度裂伤，无合并症。常规护理：恶露观察，会阴温水坐浴，按需哺乳，早下床，防便秘，情绪支持，42天复查，避孕指导。同病例42。 |
| **护理计划英文翻译 English translation of care plan** | The patient is 32 years old, G1P1, delivered at 40 weeks by spontaneous vaginal delivery, with an estimated intrapartum blood loss of 180 mL. The newborn was a female infant, weighed 2950 g, and had Apgar scores of 10, 10, 10. The main case-based risks include no special comorbidity was recorded. Within the first 24 hours, assess temperature, pulse, blood pressure, uterine fundal height, uterine tone, vaginal bleeding, lochia color, urinary retention, first voiding, and pain score. During hospitalization and after discharge, continue to observe whether lochia gradually changes from red to lighter color, whether breast engorgement or nipple fissure occurs, and whether sleep and mood remain stable. Diet should include high-quality protein, iron-rich food, vegetables, fruit, and adequate fluids. Avoid blind over-supplementation and prolonged bed rest. Because this was a spontaneous vaginal birth, recovery of any perineal tear or suture site should be observed. Keep the area clean and dry after urination or bowel movement and watch for redness, swelling, bleeding, worsening pain, or abnormal odor. If there is no dizziness or increased bleeding, increase activity gradually and begin gentle pelvic floor exercises under professional guidance. For breastfeeding, encourage early skin-to-skin contact and feeding on demand. Teach correct latch and positioning. For engorgement, apply warmth or gentle massage before feeding. If nipple pain occurs, adjust latch promptly. Localized breast redness, hard painful swelling, chills, or high fever should raise concern for mastitis. No special complication was recorded, but routine postpartum management should still focus on uterine involution, lochia, wounds, breastfeeding, nutrition, activity, and mental health. Before discharge, provide education on medication, wound care, breastfeeding, and contraception. Arrange a postpartum review around 42 days after delivery, or earlier if symptoms occur. Seek urgent medical care for sudden heavy vaginal bleeding, bright red bleeding soaking pads, foul-smelling lochia, fever, persistent abdominal pain, pus from the wound, difficulty urinating, unilateral leg swelling or pain, chest tightness, shortness of breath, or persistent sadness, anxiety, or thoughts of harming herself or the baby. | (same as case 42 routine) |
| **Clinical appropriateness 临床适当性** | 4 | 3 |
| **Individualization 个体化程度** | 3 | 3 |
| **Comprehensiveness 完整性** | 4 | 3 |
| **Actionability 可操作性** | 4 | 3 |
| **Safety 安全性** | 5 | 3 |
| **Triage appropriateness 分诊/转诊建议适当性** | 5 | 3 |
| **Structure and clarity 结构与清晰度** | 3 | 4 |

**病例 46 / Case 46**

| **项目 Item** | **ChatGPT 5.5** | **DeepSeek V3** |
| --- | --- | --- |
| **去标识化临床病例摘要 De-identified Clinical Case Summary** | 患者32岁，孕产史为G2P1，既往剖宫产0次，孕周40+6，临产类别为阴道米索。自然破膜，羊水量约300ml，羊水性质为清。以自然分娩分娩，产时出血约700ml。第一产程12时30分，第二产程3时26分，第三产程0时30分，总产程16时26分，会阴或切口情况为侧切，内缝。合并症及主要风险因素：妊娠合并巨大儿、B族链球菌感染、妊娠合并右侧卵巢囊肿？D-二聚体升高、胎膜早破。新生儿出生体重4470g，Apgar评分1、5、10分钟分别为8、10、10分，分娩结局为活产。 | |
| **临床病例摘要英文翻译 English Translation of De-identified Clinical Case Summary** | Maternal age was 32 years. The obstetric history was G2P1, with 0 previous cesarean deliveries. Gestational age was 40+6 weeks, and the labor onset or induction category was vaginal misoprostol induction. The membranes ruptured spontaneously; the amniotic fluid volume was approximately 300 mL and its character was clear. The patient had a spontaneous vaginal delivery, with estimated intrapartum blood loss of 700 mL. The first, second, and third stages of labor were 12 h 30 min, 3 h 26 min, and 0 h 30 min, respectively; the total labor duration was 16 h 26 min. An episiotomy was repaired with internal sutures. Documented comorbidities and major risk factors were macrosomia; group B Streptococcus infection; right ovarian cyst during pregnancy; suspected elevated D-dimer; premature rupture of membranes. Neonatal birth weight was 4470 g; Apgar scores at 1, 5, and 10 minutes were 8, 10, 10; and the delivery outcome was live birth. | |
| **模型生成的产褥期护理计划 Model-generated postpartum nursing care plan** | 产妇32岁，G2P1，孕40+6周，经阴道自然分娩分娩，产时出血约700ml，新生儿女婴、体重4470g，Apgar评分8、10、10分。病例提示的重点风险为胎膜早破或感染相关风险、出血或贫血相关风险、胎儿窘迫或羊水异常记录。产后24小时内重点评估体温、脉搏、血压、宫底高度、宫缩、阴道流血、恶露颜色和尿潴留，记录首次排尿和疼痛评分。产后住院及出院后继续观察恶露是否由红色逐渐转淡，乳房是否胀痛，乳头是否皲裂，睡眠和情绪是否稳定。饮食以优质蛋白、含铁食物、蔬菜水果和足量饮水为主，避免盲目大补和长期卧床。本次为阴道自然分娩，应重点观察会阴裂伤或缝合处恢复。每次大小便后保持局部清洁干燥，观察红肿、渗血、疼痛加重和异常气味。无头晕和出血增多时逐步增加活动，并在医护指导下开始轻柔盆底肌训练。哺乳方面建议尽早母婴皮肤接触和按需哺乳，指导正确含接，乳胀时先热敷或轻柔按摩再哺乳，乳头疼痛时及时调整姿势。若出现乳房局部红肿硬痛、寒战或高热，应警惕乳腺炎。因存在出血量偏多、贫血或血小板异常相关风险，应复查血常规，关注头晕、心悸、乏力、面色苍白、恶露突然增多或较大血块，按医嘱补铁，必要时评估凝血和子宫复旧。存在胎膜早破、B族链球菌感染、阴道炎或宫内感染疑虑时，应加强体温、下腹压痛、恶露气味、会阴或切口渗液观察，发热或恶露恶臭时及时复诊。因有胎儿窘迫、羊水污染或新生儿评分变化记录，母婴同室期间应配合观察新生儿吃奶、肤色、体温、呼吸和反应，发现吃奶差、发绀、嗜睡或呼吸异常应立即告知儿科医生。合并子宫肌瘤、卵巢囊肿或多囊卵巢相关情况时，产后复查时建议结合妇科超声评估，若腹痛、包块感或异常出血加重，应提前就诊。出院前应完成用药、伤口、哺乳和避孕宣教，42天左右复查，必要时提前复诊。若出现阴道流血突然增多、鲜红血浸湿卫生巾、恶露恶臭、发热、持续腹痛、伤口化脓、排尿困难、下肢单侧肿痛、胸闷气短，或持续悲伤焦虑及伤害自己或婴儿的想法，应立即就医或急诊处理。 | 患者32岁，G2P1，自然分娩+侧切，产程16小时26分，产时出血700ml（产后出血），合并巨大儿（4470g）、GBS感染、右侧卵巢囊肿可疑、D-二聚体升高、胎膜早破，新生儿Apgar 8-10-10。PPH 700ml需住院治疗，出院后监测血红蛋白，补充铁剂，警惕晚期出血。D-二聚体升高+产程长+出血，血栓风险高，鼓励尽早活动、弹力袜，必要时抗凝。巨大儿需加强宫底按摩预防宫缩乏力；新生儿监测低血糖。GBS感染新生儿防早发型败血症。卵巢囊肿6周B超复查。会阴侧切注意感染、血肿。PPH易致PTSD，心理支持。产后1周复查D-二聚体、血常规；6周B超；新生儿随访。 |
| **护理计划英文翻译 English translation of care plan** | The patient is 32 years old, G2P1, delivered at 40+6 weeks by spontaneous vaginal delivery, with an estimated intrapartum blood loss of 700 mL. The newborn was a female infant, weighed 4470 g, and had Apgar scores of 8, 10, 10. The main case-based risks include premature rupture of membranes or infection-related risk, bleeding or anemia-related risk, fetal distress or abnormal amniotic fluid findings. Within the first 24 hours, assess temperature, pulse, blood pressure, uterine fundal height, uterine tone, vaginal bleeding, lochia color, urinary retention, first voiding, and pain score. During hospitalization and after discharge, continue to observe whether lochia gradually changes from red to lighter color, whether breast engorgement or nipple fissure occurs, and whether sleep and mood remain stable. Diet should include high-quality protein, iron-rich food, vegetables, fruit, and adequate fluids. Avoid blind over-supplementation and prolonged bed rest. Because this was a spontaneous vaginal birth, recovery of any perineal tear or suture site should be observed. Keep the area clean and dry after urination or bowel movement and watch for redness, swelling, bleeding, worsening pain, or abnormal odor. If there is no dizziness or increased bleeding, increase activity gradually and begin gentle pelvic floor exercises under professional guidance. For breastfeeding, encourage early skin-to-skin contact and feeding on demand. Teach correct latch and positioning. For engorgement, apply warmth or gentle massage before feeding. If nipple pain occurs, adjust latch promptly. Localized breast redness, hard painful swelling, chills, or high fever should raise concern for mastitis. Because increased blood loss, anemia, or platelet abnormality is a concern, repeat a complete blood count as advised and watch for dizziness, palpitations, severe fatigue, pallor, sudden increase in lochia, or large clots. Iron supplementation, nutrition support, and assessment of uterine involution or coagulation should follow medical orders. With premature rupture of membranes, group B streptococcus, vaginitis, or suspected intrauterine infection, monitor temperature, lower abdominal tenderness, lochia odor, and drainage from the perineal wound or incision. Return promptly for fever or foul-smelling lochia. Because fetal distress, meconium-stained fluid, or changed Apgar scores were recorded, cooperate with pediatric observation of feeding, skin color, temperature, breathing, and responsiveness. Poor feeding, cyanosis, excessive sleepiness, or abnormal breathing should be reported immediately. For uterine fibroids, ovarian cysts, or polycystic ovary related findings, pelvic ultrasound can be considered at postpartum review. Earlier assessment is needed for worsening abdominal pain, mass sensation, or abnormal bleeding. Before discharge, provide education on medication, wound care, breastfeeding, and contraception. Arrange a postpartum review around 42 days after delivery, or earlier if symptoms occur. Seek urgent medical care for sudden heavy vaginal bleeding, bright red bleeding soaking pads, foul-smelling lochia, fever, persistent abdominal pain, pus from the wound, difficulty urinating, unilateral leg swelling or pain, chest tightness, shortness of breath, or persistent sadness, anxiety, or thoughts of harming herself or the baby. | The patient is a 32-year-old G2P1 with spontaneous delivery and episiotomy. Prolonged labor 16h26min, PPH 700 mL. Comorbidities: macrosomia (4470 g), GBS, possible ovarian cyst, elevated D-dimer, PPROM. Newborn Apgar 8-10-10. PPH management – monitor Hb, iron. Thrombosis risk – early ambulation, compression stockings, possible anticoagulation. Macrosomia – aggressive fundal massage; neonatal glucose monitoring. GBS – newborn observation. Ovarian cyst – ultrasound at 6w. Episiotomy care. PTSD risk – psychological support. Follow-up: D-dimer, CBC at 1w; ultrasound at 6w; newborn follow-up. |
| **Clinical appropriateness 临床适当性** | 5 | 4 |
| **Individualization 个体化程度** | 5 | 5 |
| **Comprehensiveness 完整性** | 5 | 4 |
| **Actionability 可操作性** | 5 | 4 |
| **Safety 安全性** | 5 | 4 |
| **Triage appropriateness 分诊/转诊建议适当性** | 5 | 4 |
| **Structure and clarity 结构与清晰度** | 4 | 3 |

**病例 47 / Case 47**

| **项目 Item** | **ChatGPT 5.5** | **DeepSeek V3** |
| --- | --- | --- |
| **去标识化临床病例摘要 De-identified Clinical Case Summary** | 患者38岁，孕产史为G1P1，既往剖宫产0次，孕周38+5，临产类别为阴道放置米索。自然破膜，羊水量约800ml，羊水性质为清。以产钳分娩，产时出血约290ml。第一产程3时25分，第二产程2时12分，第三产程0时8分，总产程5时45分，会阴或切口情况为侧切，内缝。合并症及主要风险因素：胎儿窘迫？妊娠期高血压；高龄初孕妇的监督；胎膜早破。新生儿出生体重2910g，Apgar评分1、5、10分钟分别为10、10、10分，分娩结局为活产。 | |
| **临床病例摘要英文翻译 English Translation of De-identified Clinical Case Summary** | Maternal age was 38 years. The obstetric history was G1P1, with 0 previous cesarean deliveries. Gestational age was 38+5 weeks, and the labor onset or induction category was vaginal misoprostol induction. The membranes ruptured spontaneously; the amniotic fluid volume was approximately 800 mL and its character was clear. The patient had a forceps-assisted vaginal delivery, with estimated intrapartum blood loss of 290 mL. The first, second, and third stages of labor were 3 h 25 min, 2 h 12 min, and 0 h 8 min, respectively; the total labor duration was 5 h 45 min. An episiotomy was repaired with internal sutures. Documented comorbidities and major risk factors were suspected fetal distress; gestational hypertension; advanced maternal age in a primigravida; premature rupture of membranes. Neonatal birth weight was 2910 g; Apgar scores at 1, 5, and 10 minutes were 10, 10, 10; and the delivery outcome was live birth. | |
| **模型生成的产褥期护理计划 Model-generated postpartum nursing care plan** | 产妇38岁，G1P1，孕38+5周，经产钳助产阴道分娩分娩，产时出血约290ml，新生儿男婴、体重2910g，Apgar评分10、10、10分。病例提示的重点风险为妊娠期高血压或子痫前期相关风险、胎膜早破或感染相关风险、胎儿窘迫或羊水异常记录、高龄妊娠因素。产后24小时内重点评估体温、脉搏、血压、宫底高度、宫缩、阴道流血、恶露颜色和尿潴留，记录首次排尿和疼痛评分。产后住院及出院后继续观察恶露是否由红色逐渐转淡，乳房是否胀痛，乳头是否皲裂，睡眠和情绪是否稳定。饮食以优质蛋白、含铁食物、蔬菜水果和足量饮水为主，避免盲目大补和长期卧床。本次为产钳助产阴道分娩，会阴伤口、盆底和排尿功能是护理重点。每次大小便后用温水从前向后清洁会阴并轻轻擦干，观察侧切或裂伤处肿胀、硬结、渗血、分泌物和异味。疼痛明显时可侧卧休息，避免久蹲、负重和过早剧烈活动。哺乳方面建议尽早母婴皮肤接触和按需哺乳，指导正确含接，乳胀时先热敷或轻柔按摩再哺乳，乳头疼痛时及时调整姿势。若出现乳房局部红肿硬痛、寒战或高热，应警惕乳腺炎。有高血压或子痫前期风险者，产后仍可能出现血压波动，应按医嘱监测血压，若持续头痛、眼花、胸闷、上腹痛、抽搐或水肿加重，应立即就医。存在胎膜早破、B族链球菌感染、阴道炎或宫内感染疑虑时，应加强体温、下腹压痛、恶露气味、会阴或切口渗液观察，发热或恶露恶臭时及时复诊。因有胎儿窘迫、羊水污染或新生儿评分变化记录，母婴同室期间应配合观察新生儿吃奶、肤色、体温、呼吸和反应，发现吃奶差、发绀、嗜睡或呼吸异常应立即告知儿科医生。高龄产妇恢复期应避免过度劳累和连续熬夜，家属应参与夜间照护，保证睡眠、营养和情绪支持。出院前应完成用药、伤口、哺乳和避孕宣教，42天左右复查，必要时提前复诊。若出现阴道流血突然增多、鲜红血浸湿卫生巾、恶露恶臭、发热、持续腹痛、伤口化脓、排尿困难、下肢单侧肿痛、胸闷气短，或持续悲伤焦虑及伤害自己或婴儿的想法，应立即就医或急诊处理。 | 患者38岁，初产妇，产钳+侧切，合并可疑胎儿窘迫、妊娠期高血压、高龄初产、胎膜早破，脐带捻转20周+假结。产后每日监测血压4次，若血压≥140/90或头痛等，警惕子痫前期。脐带异常+可疑窘迫，新生儿观察神经、呼吸。会阴侧切每日消毒。PPROM监测感染。产时出血290ml，正常。高龄、高血压易焦虑，心理支持。产后2周复查血压，42天全面评估。 |
| **护理计划英文翻译 English translation of care plan** | The patient is 38 years old, G1P1, delivered at 38+5 weeks by forceps-assisted vaginal delivery, with an estimated intrapartum blood loss of 290 mL. The newborn was a male infant, weighed 2910 g, and had Apgar scores of 10, 10, 10. The main case-based risks include gestational hypertension or preeclampsia-related risk, premature rupture of membranes or infection-related risk, fetal distress or abnormal amniotic fluid findings, advanced maternal age. Within the first 24 hours, assess temperature, pulse, blood pressure, uterine fundal height, uterine tone, vaginal bleeding, lochia color, urinary retention, first voiding, and pain score. During hospitalization and after discharge, continue to observe whether lochia gradually changes from red to lighter color, whether breast engorgement or nipple fissure occurs, and whether sleep and mood remain stable. Diet should include high-quality protein, iron-rich food, vegetables, fruit, and adequate fluids. Avoid blind over-supplementation and prolonged bed rest. Because this was a forceps-assisted vaginal delivery, perineal wound care, pelvic floor recovery, and urinary function are priorities. Rinse the perineum from front to back with warm water after urination or bowel movement and pat dry. Observe the episiotomy or tear for swelling, induration, bleeding, discharge, and odor. Rest in a lateral position if pain is marked, and avoid prolonged squatting, heavy lifting, and early vigorous activity. For breastfeeding, encourage early skin-to-skin contact and feeding on demand. Teach correct latch and positioning. For engorgement, apply warmth or gentle massage before feeding. If nipple pain occurs, adjust latch promptly. Localized breast redness, hard painful swelling, chills, or high fever should raise concern for mastitis. For hypertension or preeclampsia risk, postpartum blood pressure may still fluctuate. Continue blood pressure monitoring as advised and seek urgent care for persistent headache, visual symptoms, chest tightness, upper abdominal pain, seizure, or worsening edema. With premature rupture of membranes, group B streptococcus, vaginitis, or suspected intrauterine infection, monitor temperature, lower abdominal tenderness, lochia odor, and drainage from the perineal wound or incision. Return promptly for fever or foul-smelling lochia. Because fetal distress, meconium-stained fluid, or changed Apgar scores were recorded, cooperate with pediatric observation of feeding, skin color, temperature, breathing, and responsiveness. Poor feeding, cyanosis, excessive sleepiness, or abnormal breathing should be reported immediately. Advanced maternal age may slow recovery, so the family should help with nighttime care and support adequate sleep, nutrition, and emotional stability. Before discharge, provide education on medication, wound care, breastfeeding, and contraception. Arrange a postpartum review around 42 days after delivery, or earlier if symptoms occur. Seek urgent medical care for sudden heavy vaginal bleeding, bright red bleeding soaking pads, foul-smelling lochia, fever, persistent abdominal pain, pus from the wound, difficulty urinating, unilateral leg swelling or pain, chest tightness, shortness of breath, or persistent sadness, anxiety, or thoughts of harming herself or the baby. | The patient is a 38-year-old primipara with forceps delivery and episiotomy. Comorbidities: suspected fetal distress, gestational hypertension, advanced age, PPROM, cord twisting 20 loops + false knot. Monitor BP 4 times daily; seek care if BP≥140/90 or headache, epigastric pain, visual changes. Newborn – monitor neurology and respiration. Episiotomy care. Monitor infection. Blood loss 290 mL, normal. Psychological support. Follow-up: BP at 2 weeks; comprehensive exam at 42 days. |
| **Clinical appropriateness 临床适当性** | 5 | 4 |
| **Individualization 个体化程度** | 5 | 5 |
| **Comprehensiveness 完整性** | 5 | 3 |
| **Actionability 可操作性** | 5 | 4 |
| **Safety 安全性** | 5 | 4 |
| **Triage appropriateness 分诊/转诊建议适当性** | 5 | 3 |
| **Structure and clarity 结构与清晰度** | 4 | 3 |

**病例 48 / Case 48**

| **项目 Item** | **ChatGPT 5.5** | **DeepSeek V3** |
| --- | --- | --- |
| **去标识化临床病例摘要 De-identified Clinical Case Summary** | 患者28岁，孕产史为G3P1，既往剖宫产0次，孕周40+1，临产类别为自然临产。自然破膜，羊水量约600ml，羊水性质为清。以自然分娩分娩，产时出血约240ml。第一产程13时0分，第二产程0时59分，第三产程0时6分，总产程14时5分，会阴或切口情况为Ⅰ，内缝。合并症及主要风险因素：无。新生儿出生体重3660g，Apgar评分1、5、10分钟分别为10、10、10分，分娩结局为活产。 | |
| **临床病例摘要英文翻译 English Translation of De-identified Clinical Case Summary** | Maternal age was 28 years. The obstetric history was G3P1, with 0 previous cesarean deliveries. Gestational age was 40+1 weeks, and the labor onset or induction category was spontaneous labor. The membranes ruptured spontaneously; the amniotic fluid volume was approximately 600 mL and its character was clear. The patient had a spontaneous vaginal delivery, with estimated intrapartum blood loss of 240 mL. The first, second, and third stages of labor were 13 h 0 min, 0 h 59 min, and 0 h 6 min, respectively; the total labor duration was 14 h 5 min. A first-degree perineal laceration was repaired with internal sutures. No comorbidities or major risk factors were documented. Neonatal birth weight was 3660 g; Apgar scores at 1, 5, and 10 minutes were 10, 10, 10; and the delivery outcome was live birth. | |
| **模型生成的产褥期护理计划 Model-generated postpartum nursing care plan** | 产妇28岁，G3P1，孕40+1周，经阴道自然分娩分娩，产时出血约240ml，新生儿男婴、体重3660g，Apgar评分10、10、10分。病例提示的重点风险为未记录特殊合并症。产后24小时内重点评估体温、脉搏、血压、宫底高度、宫缩、阴道流血、恶露颜色和尿潴留，记录首次排尿和疼痛评分。产后住院及出院后继续观察恶露是否由红色逐渐转淡，乳房是否胀痛，乳头是否皲裂，睡眠和情绪是否稳定。饮食以优质蛋白、含铁食物、蔬菜水果和足量饮水为主，避免盲目大补和长期卧床。本次为阴道自然分娩，应重点观察会阴裂伤或缝合处恢复。每次大小便后保持局部清洁干燥，观察红肿、渗血、疼痛加重和异常气味。无头晕和出血增多时逐步增加活动，并在医护指导下开始轻柔盆底肌训练。哺乳方面建议尽早母婴皮肤接触和按需哺乳，指导正确含接，乳胀时先热敷或轻柔按摩再哺乳，乳头疼痛时及时调整姿势。若出现乳房局部红肿硬痛、寒战或高热，应警惕乳腺炎。目前未记录特殊合并症，仍需按常规产褥期管理执行，重点是子宫复旧、恶露、伤口、哺乳、营养、活动和心理状态。出院前应完成用药、伤口、哺乳和避孕宣教，42天左右复查，必要时提前复诊。若出现阴道流血突然增多、鲜红血浸湿卫生巾、恶露恶臭、发热、持续腹痛、伤口化脓、排尿困难、下肢单侧肿痛、胸闷气短，或持续悲伤焦虑及伤害自己或婴儿的想法，应立即就医或急诊处理。 | 患者28岁，G3P1，自然分娩+I度裂伤，无合并症，脐带绕颈1周。常规护理+新生儿观察脐带绕颈（观察呼吸、喂养）。其他同常规。 |
| **护理计划英文翻译 English translation of care plan** | The patient is 28 years old, G3P1, delivered at 40+1 weeks by spontaneous vaginal delivery, with an estimated intrapartum blood loss of 240 mL. The newborn was a male infant, weighed 3660 g, and had Apgar scores of 10, 10, 10. The main case-based risks include no special comorbidity was recorded. Within the first 24 hours, assess temperature, pulse, blood pressure, uterine fundal height, uterine tone, vaginal bleeding, lochia color, urinary retention, first voiding, and pain score. During hospitalization and after discharge, continue to observe whether lochia gradually changes from red to lighter color, whether breast engorgement or nipple fissure occurs, and whether sleep and mood remain stable. Diet should include high-quality protein, iron-rich food, vegetables, fruit, and adequate fluids. Avoid blind over-supplementation and prolonged bed rest. Because this was a spontaneous vaginal birth, recovery of any perineal tear or suture site should be observed. Keep the area clean and dry after urination or bowel movement and watch for redness, swelling, bleeding, worsening pain, or abnormal odor. If there is no dizziness or increased bleeding, increase activity gradually and begin gentle pelvic floor exercises under professional guidance. For breastfeeding, encourage early skin-to-skin contact and feeding on demand. Teach correct latch and positioning. For engorgement, apply warmth or gentle massage before feeding. If nipple pain occurs, adjust latch promptly. Localized breast redness, hard painful swelling, chills, or high fever should raise concern for mastitis. No special complication was recorded, but routine postpartum management should still focus on uterine involution, lochia, wounds, breastfeeding, nutrition, activity, and mental health. Before discharge, provide education on medication, wound care, breastfeeding, and contraception. Arrange a postpartum review around 42 days after delivery, or earlier if symptoms occur. Seek urgent medical care for sudden heavy vaginal bleeding, bright red bleeding soaking pads, foul-smelling lochia, fever, persistent abdominal pain, pus from the wound, difficulty urinating, unilateral leg swelling or pain, chest tightness, shortness of breath, or persistent sadness, anxiety, or thoughts of harming herself or the baby. | The patient is a 28-year-old G3P1 with spontaneous delivery and first-degree laceration. No comorbidities except nuchal cord 1 loop. Routine care plus newborn observation for signs of hypoxia (respiration, feeding). Otherwise standard postpartum care. |
| **Clinical appropriateness 临床适当性** | 4 | 3 |
| **Individualization 个体化程度** | 3 | 3 |
| **Comprehensiveness 完整性** | 4 | 3 |
| **Actionability 可操作性** | 4 | 3 |
| **Safety 安全性** | 5 | 3 |
| **Triage appropriateness 分诊/转诊建议适当性** | 5 | 3 |
| **Structure and clarity 结构与清晰度** | 3 | 4 |

**病例 49 / Case 49**

| **项目 Item** | **ChatGPT 5.5** | **DeepSeek V3** |
| --- | --- | --- |
| **去标识化临床病例摘要 De-identified Clinical Case Summary** | 患者31岁，孕产史为G2P1，既往剖宫产0次，孕周37+4，临产类别为催产素引产。自然破膜，羊水量约100ml，羊水性质为清。以自然分娩分娩，产时出血约290ml。第一产程13时0分，第二产程0时36分，第三产程0时4分，总产程13时40分，会阴或切口情况为Ⅰ，内缝。合并症及主要风险因素：胎膜早破；双眼近视。新生儿出生体重2840g，Apgar评分1、5、10分钟分别为10、10、10分，分娩结局为活产。 | |
| **临床病例摘要英文翻译 English Translation of De-identified Clinical Case Summary** | Maternal age was 31 years. The obstetric history was G2P1, with 0 previous cesarean deliveries. Gestational age was 37+4 weeks, and the labor onset or induction category was oxytocin induction. The membranes ruptured spontaneously; the amniotic fluid volume was approximately 100 mL and its character was clear. The patient had a spontaneous vaginal delivery, with estimated intrapartum blood loss of 290 mL. The first, second, and third stages of labor were 13 h 0 min, 0 h 36 min, and 0 h 4 min, respectively; the total labor duration was 13 h 40 min. A first-degree perineal laceration was repaired with internal sutures. Documented comorbidities and major risk factors were premature rupture of membranes; bilateral myopia. Neonatal birth weight was 2840 g; Apgar scores at 1, 5, and 10 minutes were 10, 10, 10; and the delivery outcome was live birth. | |
| **模型生成的产褥期护理计划 Model-generated postpartum nursing care plan** | 产妇31岁，G2P1，孕37+4周，经阴道自然分娩分娩，产时出血约290ml，新生儿女婴、体重2840g，Apgar评分10、10、10分。病例提示的重点风险为胎膜早破或感染相关风险。产后24小时内重点评估体温、脉搏、血压、宫底高度、宫缩、阴道流血、恶露颜色和尿潴留，记录首次排尿和疼痛评分。产后住院及出院后继续观察恶露是否由红色逐渐转淡，乳房是否胀痛，乳头是否皲裂，睡眠和情绪是否稳定。饮食以优质蛋白、含铁食物、蔬菜水果和足量饮水为主，避免盲目大补和长期卧床。本次为阴道自然分娩，应重点观察会阴裂伤或缝合处恢复。每次大小便后保持局部清洁干燥，观察红肿、渗血、疼痛加重和异常气味。无头晕和出血增多时逐步增加活动，并在医护指导下开始轻柔盆底肌训练。哺乳方面建议尽早母婴皮肤接触和按需哺乳，指导正确含接，乳胀时先热敷或轻柔按摩再哺乳，乳头疼痛时及时调整姿势。若出现乳房局部红肿硬痛、寒战或高热，应警惕乳腺炎。存在胎膜早破、B族链球菌感染、阴道炎或宫内感染疑虑时，应加强体温、下腹压痛、恶露气味、会阴或切口渗液观察，发热或恶露恶臭时及时复诊。出院前应完成用药、伤口、哺乳和避孕宣教，42天左右复查，必要时提前复诊。若出现阴道流血突然增多、鲜红血浸湿卫生巾、恶露恶臭、发热、持续腹痛、伤口化脓、排尿困难、下肢单侧肿痛、胸闷气短，或持续悲伤焦虑及伤害自己或婴儿的想法，应立即就医或急诊处理。 | 患者31岁，G2P1，自然分娩+I度裂伤，合并胎膜早破、双眼近视。PPROM监测感染。高度近视避免用力屏气，防视网膜脱离。常规恶露、会阴护理。鼓励母乳喂养。42天复查。 |
| **护理计划英文翻译 English translation of care plan** | The patient is 31 years old, G2P1, delivered at 37+4 weeks by spontaneous vaginal delivery, with an estimated intrapartum blood loss of 290 mL. The newborn was a female infant, weighed 2840 g, and had Apgar scores of 10, 10, 10. The main case-based risks include premature rupture of membranes or infection-related risk. Within the first 24 hours, assess temperature, pulse, blood pressure, uterine fundal height, uterine tone, vaginal bleeding, lochia color, urinary retention, first voiding, and pain score. During hospitalization and after discharge, continue to observe whether lochia gradually changes from red to lighter color, whether breast engorgement or nipple fissure occurs, and whether sleep and mood remain stable. Diet should include high-quality protein, iron-rich food, vegetables, fruit, and adequate fluids. Avoid blind over-supplementation and prolonged bed rest. Because this was a spontaneous vaginal birth, recovery of any perineal tear or suture site should be observed. Keep the area clean and dry after urination or bowel movement and watch for redness, swelling, bleeding, worsening pain, or abnormal odor. If there is no dizziness or increased bleeding, increase activity gradually and begin gentle pelvic floor exercises under professional guidance. For breastfeeding, encourage early skin-to-skin contact and feeding on demand. Teach correct latch and positioning. For engorgement, apply warmth or gentle massage before feeding. If nipple pain occurs, adjust latch promptly. Localized breast redness, hard painful swelling, chills, or high fever should raise concern for mastitis. With premature rupture of membranes, group B streptococcus, vaginitis, or suspected intrauterine infection, monitor temperature, lower abdominal tenderness, lochia odor, and drainage from the perineal wound or incision. Return promptly for fever or foul-smelling lochia. Before discharge, provide education on medication, wound care, breastfeeding, and contraception. Arrange a postpartum review around 42 days after delivery, or earlier if symptoms occur. Seek urgent medical care for sudden heavy vaginal bleeding, bright red bleeding soaking pads, foul-smelling lochia, fever, persistent abdominal pain, pus from the wound, difficulty urinating, unilateral leg swelling or pain, chest tightness, shortness of breath, or persistent sadness, anxiety, or thoughts of harming herself or the baby. | The patient is a 31-year-old G2P1 with spontaneous delivery and first-degree laceration. Comorbidities: PPROM, myopia. Monitor infection. Avoid Valsalva to prevent retinal detachment. Routine lochia and perineal care. Encourage breastfeeding. Follow-up at 42 days. |
| **Clinical appropriateness 临床适当性** | 4 | 3 |
| **Individualization 个体化程度** | 3 | 3 |
| **Comprehensiveness 完整性** | 4 | 3 |
| **Actionability 可操作性** | 4 | 3 |
| **Safety 安全性** | 5 | 3 |
| **Triage appropriateness 分诊/转诊建议适当性** | 5 | 3 |
| **Structure and clarity 结构与清晰度** | 3 | 4 |

**病例 50 / Case 50**

| **项目 Item** | **ChatGPT 5.5** | **DeepSeek V3** |
| --- | --- | --- |
| **去标识化临床病例摘要 De-identified Clinical Case Summary** | 患者30岁，孕产史为G1P1，既往剖宫产0次，孕周39+2，临产类别为自然临产。人工破膜，羊水量约300ml，羊水性质为清。以自然分娩分娩，产时出血约300ml。第一产程12时30分，第二产程0时44分，第三产程0时6分，总产程13时20分，会阴或切口情况为Ⅰ，内缝。合并症及主要风险因素：妊娠合并左侧卵巢囊肿？球拍状胎盘。新生儿出生体重3470g，Apgar评分1、5、10分钟分别为10、10、10分，分娩结局为活产。 | |
| **临床病例摘要英文翻译 English Translation of De-identified Clinical Case Summary** | Maternal age was 30 years. The obstetric history was G1P1, with 0 previous cesarean deliveries. Gestational age was 39+2 weeks, and the labor onset or induction category was spontaneous labor. The membranes were artificially ruptured; the amniotic fluid volume was approximately 300 mL and its character was clear. The patient had a spontaneous vaginal delivery, with estimated intrapartum blood loss of 300 mL. The first, second, and third stages of labor were 12 h 30 min, 0 h 44 min, and 0 h 6 min, respectively; the total labor duration was 13 h 20 min. A first-degree perineal laceration was repaired with internal sutures. Documented comorbidities and major risk factors were left ovarian cyst during pregnancy; suspected battledore placenta. Neonatal birth weight was 3470 g; Apgar scores at 1, 5, and 10 minutes were 10, 10, 10; and the delivery outcome was live birth. | |
| **模型生成的产褥期护理计划 Model-generated postpartum nursing care plan** | 产妇30岁，G1P1，孕39+2周，经阴道自然分娩分娩，产时出血约300ml，新生儿女婴、体重3470g，Apgar评分10、10、10分。病例提示的重点风险为胎盘相关风险。产后24小时内重点评估体温、脉搏、血压、宫底高度、宫缩、阴道流血、恶露颜色和尿潴留，记录首次排尿和疼痛评分。产后住院及出院后继续观察恶露是否由红色逐渐转淡，乳房是否胀痛，乳头是否皲裂，睡眠和情绪是否稳定。饮食以优质蛋白、含铁食物、蔬菜水果和足量饮水为主，避免盲目大补和长期卧床。本次为阴道自然分娩，应重点观察会阴裂伤或缝合处恢复。每次大小便后保持局部清洁干燥，观察红肿、渗血、疼痛加重和异常气味。无头晕和出血增多时逐步增加活动，并在医护指导下开始轻柔盆底肌训练。哺乳方面建议尽早母婴皮肤接触和按需哺乳，指导正确含接，乳胀时先热敷或轻柔按摩再哺乳，乳头疼痛时及时调整姿势。若出现乳房局部红肿硬痛、寒战或高热，应警惕乳腺炎。有胎盘位置、形态或胎盘血窦相关记录时，应注意恶露持续时间和出血量，产后复查可结合B超了解宫腔及子宫复旧。合并子宫肌瘤、卵巢囊肿或多囊卵巢相关情况时，产后复查时建议结合妇科超声评估，若腹痛、包块感或异常出血加重，应提前就诊。出院前应完成用药、伤口、哺乳和避孕宣教，42天左右复查，必要时提前复诊。若出现阴道流血突然增多、鲜红血浸湿卫生巾、恶露恶臭、发热、持续腹痛、伤口化脓、排尿困难、下肢单侧肿痛、胸闷气短，或持续悲伤焦虑及伤害自己或婴儿的想法，应立即就医或急诊处理。 | 患者30岁，初产妇，自然分娩+I度裂伤，合并左侧卵巢囊肿可疑、球拍状胎盘。产后6周B超复查卵巢囊肿。球拍状胎盘一般不影响产褥，常规观察恶露。I度裂伤温水坐浴。鼓励母乳喂养。42天复查。 |
| **护理计划英文翻译 English translation of care plan** | The patient is 30 years old, G1P1, delivered at 39+2 weeks by spontaneous vaginal delivery, with an estimated intrapartum blood loss of 300 mL. The newborn was a female infant, weighed 3470 g, and had Apgar scores of 10, 10, 10. The main case-based risks include placental risk factors. Within the first 24 hours, assess temperature, pulse, blood pressure, uterine fundal height, uterine tone, vaginal bleeding, lochia color, urinary retention, first voiding, and pain score. During hospitalization and after discharge, continue to observe whether lochia gradually changes from red to lighter color, whether breast engorgement or nipple fissure occurs, and whether sleep and mood remain stable. Diet should include high-quality protein, iron-rich food, vegetables, fruit, and adequate fluids. Avoid blind over-supplementation and prolonged bed rest. Because this was a spontaneous vaginal birth, recovery of any perineal tear or suture site should be observed. Keep the area clean and dry after urination or bowel movement and watch for redness, swelling, bleeding, worsening pain, or abnormal odor. If there is no dizziness or increased bleeding, increase activity gradually and begin gentle pelvic floor exercises under professional guidance. For breastfeeding, encourage early skin-to-skin contact and feeding on demand. Teach correct latch and positioning. For engorgement, apply warmth or gentle massage before feeding. If nipple pain occurs, adjust latch promptly. Localized breast redness, hard painful swelling, chills, or high fever should raise concern for mastitis. For abnormal placental location, shape, or placental sinus records, monitor bleeding amount and duration of lochia. Ultrasound review of the uterine cavity and involution may be needed at follow-up. For uterine fibroids, ovarian cysts, or polycystic ovary related findings, pelvic ultrasound can be considered at postpartum review. Earlier assessment is needed for worsening abdominal pain, mass sensation, or abnormal bleeding. Before discharge, provide education on medication, wound care, breastfeeding, and contraception. Arrange a postpartum review around 42 days after delivery, or earlier if symptoms occur. Seek urgent medical care for sudden heavy vaginal bleeding, bright red bleeding soaking pads, foul-smelling lochia, fever, persistent abdominal pain, pus from the wound, difficulty urinating, unilateral leg swelling or pain, chest tightness, shortness of breath, or persistent sadness, anxiety, or thoughts of harming herself or the baby. | The patient is a 30-year-old primipara with spontaneous delivery and first-degree laceration. Comorbidities: suspected left ovarian cyst, battledore placenta. Repeat ovarian ultrasound at 6 weeks. Battledore placenta – routine lochia monitoring. Warm sitz baths. Encourage breastfeeding. Follow-up at 42 days. |
| **Clinical appropriateness 临床适当性** | 5 | 3 |
| **Individualization 个体化程度** | 4 | 3 |
| **Comprehensiveness 完整性** | 5 | 3 |
| **Actionability 可操作性** | 4 | 3 |
| **Safety 安全性** | 5 | 3 |
| **Triage appropriateness 分诊/转诊建议适当性** | 5 | 3 |
| **Structure and clarity 结构与清晰度** | 3 | 4 |

**病例 51 / Case 51**

| **项目 Item** | **ChatGPT 5.5** | **DeepSeek V3** |
| --- | --- | --- |
| **去标识化临床病例摘要 De-identified Clinical Case Summary** | 患者31岁，孕产史为G1P1，既往剖宫产0次，孕周40+3，临产类别为催产素引产。自然破膜，羊水量约100ml，羊水性质为清。以自然分娩分娩，产时出血约280ml。第一产程3时40分，第二产程0时39分，第三产程0时6分，总产程4时25分，会阴或切口情况为Ⅰ，内缝。合并症及主要风险因素：胎儿宫内窘迫？胎膜早破；胚胎移植术后；球拍状胎盘。新生儿出生体重3450g，Apgar评分1、5、10分钟分别为10、10、10分，分娩结局为活产。 | |
| **临床病例摘要英文翻译 English Translation of De-identified Clinical Case Summary** | Maternal age was 31 years. The obstetric history was G1P1, with 0 previous cesarean deliveries. Gestational age was 40+3 weeks, and the labor onset or induction category was oxytocin induction. The membranes ruptured spontaneously; the amniotic fluid volume was approximately 100 mL and its character was clear. The patient had a spontaneous vaginal delivery, with estimated intrapartum blood loss of 280 mL. The first, second, and third stages of labor were 3 h 40 min, 0 h 39 min, and 0 h 6 min, respectively; the total labor duration was 4 h 25 min. A first-degree perineal laceration was repaired with internal sutures. Documented comorbidities and major risk factors were suspected fetal distress; premature rupture of membranes; status post embryo transfer; battledore placenta. Neonatal birth weight was 3450 g; Apgar scores at 1, 5, and 10 minutes were 10, 10, 10; and the delivery outcome was live birth. | |
| **模型生成的产褥期护理计划 Model-generated postpartum nursing care plan** | 产妇31岁，G1P1，孕40+3周，经阴道自然分娩分娩，产时出血约280ml，新生儿女婴、体重3450g，Apgar评分10、10、10分。病例提示的重点风险为胎膜早破或感染相关风险、胎盘相关风险、胎儿窘迫或羊水异常记录。产后24小时内重点评估体温、脉搏、血压、宫底高度、宫缩、阴道流血、恶露颜色和尿潴留，记录首次排尿和疼痛评分。产后住院及出院后继续观察恶露是否由红色逐渐转淡，乳房是否胀痛，乳头是否皲裂，睡眠和情绪是否稳定。饮食以优质蛋白、含铁食物、蔬菜水果和足量饮水为主，避免盲目大补和长期卧床。本次为阴道自然分娩，应重点观察会阴裂伤或缝合处恢复。每次大小便后保持局部清洁干燥，观察红肿、渗血、疼痛加重和异常气味。无头晕和出血增多时逐步增加活动，并在医护指导下开始轻柔盆底肌训练。哺乳方面建议尽早母婴皮肤接触和按需哺乳，指导正确含接，乳胀时先热敷或轻柔按摩再哺乳，乳头疼痛时及时调整姿势。若出现乳房局部红肿硬痛、寒战或高热，应警惕乳腺炎。存在胎膜早破、B族链球菌感染、阴道炎或宫内感染疑虑时，应加强体温、下腹压痛、恶露气味、会阴或切口渗液观察，发热或恶露恶臭时及时复诊。因有胎儿窘迫、羊水污染或新生儿评分变化记录，母婴同室期间应配合观察新生儿吃奶、肤色、体温、呼吸和反应，发现吃奶差、发绀、嗜睡或呼吸异常应立即告知儿科医生。有胎盘位置、形态或胎盘血窦相关记录时，应注意恶露持续时间和出血量，产后复查可结合B超了解宫腔及子宫复旧。出院前应完成用药、伤口、哺乳和避孕宣教，42天左右复查，必要时提前复诊。若出现阴道流血突然增多、鲜红血浸湿卫生巾、恶露恶臭、发热、持续腹痛、伤口化脓、排尿困难、下肢单侧肿痛、胸闷气短，或持续悲伤焦虑及伤害自己或婴儿的想法，应立即就医或急诊处理。 | 患者31岁，初产妇，自然分娩+I度裂伤，合并可疑胎儿窘迫、胎膜早破、胚胎移植术后、球拍状胎盘。珍贵儿+可疑窘迫+PPROM，感染风险高，监测体温、恶露。新生儿观察呼吸、喂养。球拍状胎盘无特殊。胚胎移植术后易焦虑，提供心理支持。常规会阴护理。42天复查。 |
| **护理计划英文翻译 English translation of care plan** | The patient is 31 years old, G1P1, delivered at 40+3 weeks by spontaneous vaginal delivery, with an estimated intrapartum blood loss of 280 mL. The newborn was a female infant, weighed 3450 g, and had Apgar scores of 10, 10, 10. The main case-based risks include premature rupture of membranes or infection-related risk, placental risk factors, fetal distress or abnormal amniotic fluid findings. Within the first 24 hours, assess temperature, pulse, blood pressure, uterine fundal height, uterine tone, vaginal bleeding, lochia color, urinary retention, first voiding, and pain score. During hospitalization and after discharge, continue to observe whether lochia gradually changes from red to lighter color, whether breast engorgement or nipple fissure occurs, and whether sleep and mood remain stable. Diet should include high-quality protein, iron-rich food, vegetables, fruit, and adequate fluids. Avoid blind over-supplementation and prolonged bed rest. Because this was a spontaneous vaginal birth, recovery of any perineal tear or suture site should be observed. Keep the area clean and dry after urination or bowel movement and watch for redness, swelling, bleeding, worsening pain, or abnormal odor. If there is no dizziness or increased bleeding, increase activity gradually and begin gentle pelvic floor exercises under professional guidance. For breastfeeding, encourage early skin-to-skin contact and feeding on demand. Teach correct latch and positioning. For engorgement, apply warmth or gentle massage before feeding. If nipple pain occurs, adjust latch promptly. Localized breast redness, hard painful swelling, chills, or high fever should raise concern for mastitis. With premature rupture of membranes, group B streptococcus, vaginitis, or suspected intrauterine infection, monitor temperature, lower abdominal tenderness, lochia odor, and drainage from the perineal wound or incision. Return promptly for fever or foul-smelling lochia. Because fetal distress, meconium-stained fluid, or changed Apgar scores were recorded, cooperate with pediatric observation of feeding, skin color, temperature, breathing, and responsiveness. Poor feeding, cyanosis, excessive sleepiness, or abnormal breathing should be reported immediately. For abnormal placental location, shape, or placental sinus records, monitor bleeding amount and duration of lochia. Ultrasound review of the uterine cavity and involution may be needed at follow-up. Before discharge, provide education on medication, wound care, breastfeeding, and contraception. Arrange a postpartum review around 42 days after delivery, or earlier if symptoms occur. Seek urgent medical care for sudden heavy vaginal bleeding, bright red bleeding soaking pads, foul-smelling lochia, fever, persistent abdominal pain, pus from the wound, difficulty urinating, unilateral leg swelling or pain, chest tightness, shortness of breath, or persistent sadness, anxiety, or thoughts of harming herself or the baby. | The patient is a 31-year-old primipara with spontaneous delivery and first-degree laceration. Comorbidities: suspected fetal distress, PPROM, status post embryo transfer, battledore placenta. High infection risk – monitor temperature and lochia. Newborn observation. Precious pregnancy – high anxiety, provide psychological support. Routine perineal care. Follow-up at 42 days. |
| **Clinical appropriateness 临床适当性** | 5 | 3 |
| **Individualization 个体化程度** | 5 | 4 |
| **Comprehensiveness 完整性** | 5 | 3 |
| **Actionability 可操作性** | 4 | 3 |
| **Safety 安全性** | 5 | 3 |
| **Triage appropriateness 分诊/转诊建议适当性** | 5 | 3 |
| **Structure and clarity 结构与清晰度** | 3 | 3 |

**病例 52 / Case 52**

| **项目 Item** | **ChatGPT 5.5** | **DeepSeek V3** |
| --- | --- | --- |
| **去标识化临床病例摘要 De-identified Clinical Case Summary** | 患者30岁，孕产史为G1P1，既往剖宫产0次，孕周40+2，临产类别为自然临产。自然破膜，羊水量约300ml，羊水性质为清。以自然分娩分娩，产时出血约280ml。第一产程2时45分，第二产程0时54分，第三产程0时31分，总产程4时10分，会阴或切口情况为Ⅰ，内缝。合并症及主要风险因素：胎膜早破、妊娠合并血小板减少、球拍状胎盘、肝功能检查的异常结果。新生儿出生体重3280g，Apgar评分1、5、10分钟分别为10、10、10分，分娩结局为活产。 | |
| **临床病例摘要英文翻译 English Translation of De-identified Clinical Case Summary** | Maternal age was 30 years. The obstetric history was G1P1, with 0 previous cesarean deliveries. Gestational age was 40+2 weeks, and the labor onset or induction category was spontaneous labor. The membranes ruptured spontaneously; the amniotic fluid volume was approximately 300 mL and its character was clear. The patient had a spontaneous vaginal delivery, with estimated intrapartum blood loss of 280 mL. The first, second, and third stages of labor were 2 h 45 min, 0 h 54 min, and 0 h 31 min, respectively; the total labor duration was 4 h 10 min. A first-degree perineal laceration was repaired with internal sutures. Documented comorbidities and major risk factors were premature rupture of membranes; thrombocytopenia during pregnancy; battledore placenta; abnormal liver function test results. Neonatal birth weight was 3280 g; Apgar scores at 1, 5, and 10 minutes were 10, 10, 10; and the delivery outcome was live birth. | |
| **模型生成的产褥期护理计划 Model-generated postpartum nursing care plan** | 产妇30岁，G1P1，孕40+2周，经阴道自然分娩分娩，产时出血约280ml，新生儿女婴、体重3280g，Apgar评分10、10、10分。病例提示的重点风险为胎膜早破或感染相关风险、出血或贫血相关风险、肝功能异常或胆汁淤积史、胎盘相关风险。产后24小时内重点评估体温、脉搏、血压、宫底高度、宫缩、阴道流血、恶露颜色和尿潴留，记录首次排尿和疼痛评分。产后住院及出院后继续观察恶露是否由红色逐渐转淡，乳房是否胀痛，乳头是否皲裂，睡眠和情绪是否稳定。饮食以优质蛋白、含铁食物、蔬菜水果和足量饮水为主，避免盲目大补和长期卧床。本次为阴道自然分娩，应重点观察会阴裂伤或缝合处恢复。每次大小便后保持局部清洁干燥，观察红肿、渗血、疼痛加重和异常气味。无头晕和出血增多时逐步增加活动，并在医护指导下开始轻柔盆底肌训练。哺乳方面建议尽早母婴皮肤接触和按需哺乳，指导正确含接，乳胀时先热敷或轻柔按摩再哺乳，乳头疼痛时及时调整姿势。若出现乳房局部红肿硬痛、寒战或高热，应警惕乳腺炎。因存在出血量偏多、贫血或血小板异常相关风险，应复查血常规，关注头晕、心悸、乏力、面色苍白、恶露突然增多或较大血块，按医嘱补铁，必要时评估凝血和子宫复旧。存在胎膜早破、B族链球菌感染、阴道炎或宫内感染疑虑时，应加强体温、下腹压痛、恶露气味、会阴或切口渗液观察，发热或恶露恶臭时及时复诊。有胆汁淤积或肝功能异常者，产后按医嘱复查肝功能和胆汁酸，若皮肤瘙痒、黄疸、右上腹痛或尿色明显加深，应尽快就诊。有胎盘位置、形态或胎盘血窦相关记录时，应注意恶露持续时间和出血量，产后复查可结合B超了解宫腔及子宫复旧。出院前应完成用药、伤口、哺乳和避孕宣教，42天左右复查，必要时提前复诊。若出现阴道流血突然增多、鲜红血浸湿卫生巾、恶露恶臭、发热、持续腹痛、伤口化脓、排尿困难、下肢单侧肿痛、胸闷气短，或持续悲伤焦虑及伤害自己或婴儿的想法，应立即就医或急诊处理。 | 患者30岁，初产妇，自然分娩+I度裂伤，合并胎膜早破、妊娠合并血小板减少、球拍状胎盘、肝功能异常。血小板减少需监测出血倾向（皮肤瘀斑、牙龈出血、恶露增多），避免肌肉注射，复查血常规。肝功能异常复查肝功能，避免肝损药物。PPROM监测感染。球拍状胎盘无特殊。常规会阴护理。42天复查血常规、肝功能。 |
| **护理计划英文翻译 English translation of care plan** | The patient is 30 years old, G1P1, delivered at 40+2 weeks by spontaneous vaginal delivery, with an estimated intrapartum blood loss of 280 mL. The newborn was a female infant, weighed 3280 g, and had Apgar scores of 10, 10, 10. The main case-based risks include premature rupture of membranes or infection-related risk, bleeding or anemia-related risk, abnormal liver function or a history of cholestasis, placental risk factors. Within the first 24 hours, assess temperature, pulse, blood pressure, uterine fundal height, uterine tone, vaginal bleeding, lochia color, urinary retention, first voiding, and pain score. During hospitalization and after discharge, continue to observe whether lochia gradually changes from red to lighter color, whether breast engorgement or nipple fissure occurs, and whether sleep and mood remain stable. Diet should include high-quality protein, iron-rich food, vegetables, fruit, and adequate fluids. Avoid blind over-supplementation and prolonged bed rest. Because this was a spontaneous vaginal birth, recovery of any perineal tear or suture site should be observed. Keep the area clean and dry after urination or bowel movement and watch for redness, swelling, bleeding, worsening pain, or abnormal odor. If there is no dizziness or increased bleeding, increase activity gradually and begin gentle pelvic floor exercises under professional guidance. For breastfeeding, encourage early skin-to-skin contact and feeding on demand. Teach correct latch and positioning. For engorgement, apply warmth or gentle massage before feeding. If nipple pain occurs, adjust latch promptly. Localized breast redness, hard painful swelling, chills, or high fever should raise concern for mastitis. Because increased blood loss, anemia, or platelet abnormality is a concern, repeat a complete blood count as advised and watch for dizziness, palpitations, severe fatigue, pallor, sudden increase in lochia, or large clots. Iron supplementation, nutrition support, and assessment of uterine involution or coagulation should follow medical orders. With premature rupture of membranes, group B streptococcus, vaginitis, or suspected intrauterine infection, monitor temperature, lower abdominal tenderness, lochia odor, and drainage from the perineal wound or incision. Return promptly for fever or foul-smelling lochia. For cholestasis or abnormal liver function, recheck liver function and bile acids as advised. Seek care for itching, jaundice, right upper abdominal pain, or dark urine. For abnormal placental location, shape, or placental sinus records, monitor bleeding amount and duration of lochia. Ultrasound review of the uterine cavity and involution may be needed at follow-up. Before discharge, provide education on medication, wound care, breastfeeding, and contraception. Arrange a postpartum review around 42 days after delivery, or earlier if symptoms occur. Seek urgent medical care for sudden heavy vaginal bleeding, bright red bleeding soaking pads, foul-smelling lochia, fever, persistent abdominal pain, pus from the wound, difficulty urinating, unilateral leg swelling or pain, chest tightness, shortness of breath, or persistent sadness, anxiety, or thoughts of harming herself or the baby. | The patient is a 30-year-old primipara with spontaneous delivery and first-degree laceration. Comorbidities: PPROM, thrombocytopenia during pregnancy, battledore placenta, abnormal LFTs. Monitor bleeding tendency (ecchymosis, gingival bleeding, increased lochia); avoid IM injections; repeat CBC. Repeat LFTs, avoid hepatotoxic drugs. Monitor infection. Routine perineal care. Follow-up at 42 days: CBC and LFTs. |
| **Clinical appropriateness 临床适当性** | 5 | 4 |
| **Individualization 个体化程度** | 5 | 4 |
| **Comprehensiveness 完整性** | 5 | 3 |
| **Actionability 可操作性** | 5 | 3 |
| **Safety 安全性** | 5 | 4 |
| **Triage appropriateness 分诊/转诊建议适当性** | 5 | 3 |
| **Structure and clarity 结构与清晰度** | 4 | 3 |

**病例 53 / Case 53**

| **项目 Item** | **ChatGPT 5.5** | **DeepSeek V3** |
| --- | --- | --- |
| **去标识化临床病例摘要 De-identified Clinical Case Summary** | 患者29岁，孕产史为G3P2，既往剖宫产0次，孕周40+5，临产类别为自然临产。自然破膜，羊水量约500ml，羊水性质为Ⅲ°。以自然分娩分娩，产时出血约290ml。第一产程5时50分，第二产程0时27分，第三产程0时8分，总产程6时25分，会阴或切口情况为Ⅰ，内缝。合并症及主要风险因素：B族链球菌感染；双眼屈光不正（人工晶体植入术后）；桥本甲状腺炎；胎儿窘迫？。新生儿出生体重3550g，Apgar评分1、5、10分钟分别为9、10、10分，分娩结局为活产。 | |
| **临床病例摘要英文翻译 English Translation of De-identified Clinical Case Summary** | Maternal age was 29 years. The obstetric history was G3P2, with 0 previous cesarean deliveries. Gestational age was 40+5 weeks, and the labor onset or induction category was spontaneous labor. The membranes ruptured spontaneously; the amniotic fluid volume was approximately 500 mL and its character was grade III meconium-stained. The patient had a spontaneous vaginal delivery, with estimated intrapartum blood loss of 290 mL. The first, second, and third stages of labor were 5 h 50 min, 0 h 27 min, and 0 h 8 min, respectively; the total labor duration was 6 h 25 min. A first-degree perineal laceration was repaired with internal sutures. Documented comorbidities and major risk factors were group B Streptococcus infection; bilateral refractive error (status post intraocular lens implantation); Hashimoto thyroiditis; suspected fetal distress. Neonatal birth weight was 3550 g; Apgar scores at 1, 5, and 10 minutes were 9, 10, 10; and the delivery outcome was live birth. | |
| **模型生成的产褥期护理计划 Model-generated postpartum nursing care plan** | 产妇29岁，G3P2，孕40+5周，经阴道自然分娩分娩，产时出血约290ml，新生儿女婴、体重3550g，Apgar评分9、10、10分。病例提示的重点风险为胎膜早破或感染相关风险、甲状腺功能异常、胎儿窘迫或羊水异常记录。产后24小时内重点评估体温、脉搏、血压、宫底高度、宫缩、阴道流血、恶露颜色和尿潴留，记录首次排尿和疼痛评分。产后住院及出院后继续观察恶露是否由红色逐渐转淡，乳房是否胀痛，乳头是否皲裂，睡眠和情绪是否稳定。饮食以优质蛋白、含铁食物、蔬菜水果和足量饮水为主，避免盲目大补和长期卧床。本次为阴道自然分娩，应重点观察会阴裂伤或缝合处恢复。每次大小便后保持局部清洁干燥，观察红肿、渗血、疼痛加重和异常气味。无头晕和出血增多时逐步增加活动，并在医护指导下开始轻柔盆底肌训练。哺乳方面建议尽早母婴皮肤接触和按需哺乳，指导正确含接，乳胀时先热敷或轻柔按摩再哺乳，乳头疼痛时及时调整姿势。若出现乳房局部红肿硬痛、寒战或高热，应警惕乳腺炎。合并甲状腺疾病者，应按内分泌医嘱继续或调整用药，不自行停药，产后复查甲状腺功能，观察心悸、怕冷、乏力、出汗和情绪异常。存在胎膜早破、B族链球菌感染、阴道炎或宫内感染疑虑时，应加强体温、下腹压痛、恶露气味、会阴或切口渗液观察，发热或恶露恶臭时及时复诊。因有胎儿窘迫、羊水污染或新生儿评分变化记录，母婴同室期间应配合观察新生儿吃奶、肤色、体温、呼吸和反应，发现吃奶差、发绀、嗜睡或呼吸异常应立即告知儿科医生。出院前应完成用药、伤口、哺乳和避孕宣教，42天左右复查，必要时提前复诊。若出现阴道流血突然增多、鲜红血浸湿卫生巾、恶露恶臭、发热、持续腹痛、伤口化脓、排尿困难、下肢单侧肿痛、胸闷气短，或持续悲伤焦虑及伤害自己或婴儿的想法，应立即就医或急诊处理。 | 患者29岁，G3P2，自然分娩+I度裂伤，合并GBS感染、桥本甲状腺炎、可疑胎儿窘迫、脐带绕颈1周，羊水Ⅲ°污染。新生儿需监测胎粪吸入综合征及GBS早发型败血症。桥本甲减继续左甲状腺素，6周复查。GBS感染产妇已完成抗生素，但仍需监测感染。常规会阴护理。提供心理支持。42天复查甲功；新生儿随访。 |
| **护理计划英文翻译 English translation of care plan** | The patient is 29 years old, G3P2, delivered at 40+5 weeks by spontaneous vaginal delivery, with an estimated intrapartum blood loss of 290 mL. The newborn was a female infant, weighed 3550 g, and had Apgar scores of 9, 10, 10. The main case-based risks include premature rupture of membranes or infection-related risk, thyroid dysfunction, fetal distress or abnormal amniotic fluid findings. Within the first 24 hours, assess temperature, pulse, blood pressure, uterine fundal height, uterine tone, vaginal bleeding, lochia color, urinary retention, first voiding, and pain score. During hospitalization and after discharge, continue to observe whether lochia gradually changes from red to lighter color, whether breast engorgement or nipple fissure occurs, and whether sleep and mood remain stable. Diet should include high-quality protein, iron-rich food, vegetables, fruit, and adequate fluids. Avoid blind over-supplementation and prolonged bed rest. Because this was a spontaneous vaginal birth, recovery of any perineal tear or suture site should be observed. Keep the area clean and dry after urination or bowel movement and watch for redness, swelling, bleeding, worsening pain, or abnormal odor. If there is no dizziness or increased bleeding, increase activity gradually and begin gentle pelvic floor exercises under professional guidance. For breastfeeding, encourage early skin-to-skin contact and feeding on demand. Teach correct latch and positioning. For engorgement, apply warmth or gentle massage before feeding. If nipple pain occurs, adjust latch promptly. Localized breast redness, hard painful swelling, chills, or high fever should raise concern for mastitis. For thyroid disease, continue or adjust medication only under endocrine guidance, recheck thyroid function after delivery, and watch for palpitations, cold intolerance, fatigue, sweating, or abnormal mood changes. With premature rupture of membranes, group B streptococcus, vaginitis, or suspected intrauterine infection, monitor temperature, lower abdominal tenderness, lochia odor, and drainage from the perineal wound or incision. Return promptly for fever or foul-smelling lochia. Because fetal distress, meconium-stained fluid, or changed Apgar scores were recorded, cooperate with pediatric observation of feeding, skin color, temperature, breathing, and responsiveness. Poor feeding, cyanosis, excessive sleepiness, or abnormal breathing should be reported immediately. Before discharge, provide education on medication, wound care, breastfeeding, and contraception. Arrange a postpartum review around 42 days after delivery, or earlier if symptoms occur. Seek urgent medical care for sudden heavy vaginal bleeding, bright red bleeding soaking pads, foul-smelling lochia, fever, persistent abdominal pain, pus from the wound, difficulty urinating, unilateral leg swelling or pain, chest tightness, shortness of breath, or persistent sadness, anxiety, or thoughts of harming herself or the baby. | The patient is a 29-year-old G3P2 with spontaneous delivery and first-degree laceration. Comorbidities: GBS, Hashimoto thyroiditis, suspected fetal distress, nuchal cord 1 loop, grade III meconium. Newborn – monitor for MAS and early-onset GBS sepsis. Continue levothyroxine, retest at 6 weeks. Monitor maternal infection. Routine perineal care. Psychological support. Follow-up: thyroid at 42 days; newborn follow-up. |
| **Clinical appropriateness 临床适当性** | 5 | 4 |
| **Individualization 个体化程度** | 5 | 5 |
| **Comprehensiveness 完整性** | 5 | 3 |
| **Actionability 可操作性** | 4 | 4 |
| **Safety 安全性** | 5 | 4 |
| **Triage appropriateness 分诊/转诊建议适当性** | 5 | 3 |
| **Structure and clarity 结构与清晰度** | 4 | 3 |

**病例 54 / Case 54**

| **项目 Item** | **ChatGPT 5.5** | **DeepSeek V3** |
| --- | --- | --- |
| **去标识化临床病例摘要 De-identified Clinical Case Summary** | 患者26岁，孕产史为G1P1，既往剖宫产0次，孕周39+3，临产类别为自然临产。自然破膜，羊水量约300ml，羊水性质为清。以产钳分娩，产时出血约300ml。第一产程4时55分，第二产程0时13分，第三产程0时7分，总产程5时15分，会阴或切口情况为侧切，内缝。合并症及主要风险因素：妊娠合并亚临床甲状腺功能减退症；双眼近视；妊娠合并B族链球菌感染；妊娠合并霉菌性阴道炎；胎儿窘迫？。新生儿出生体重3730g，Apgar评分1、5、10分钟分别为10、10、10分，分娩结局为活产。 | |
| **临床病例摘要英文翻译 English Translation of De-identified Clinical Case Summary** | Maternal age was 26 years. The obstetric history was G1P1, with 0 previous cesarean deliveries. Gestational age was 39+3 weeks, and the labor onset or induction category was spontaneous labor. The membranes ruptured spontaneously; the amniotic fluid volume was approximately 300 mL and its character was clear. The patient had a forceps-assisted vaginal delivery, with estimated intrapartum blood loss of 300 mL. The first, second, and third stages of labor were 4 h 55 min, 0 h 13 min, and 0 h 7 min, respectively; the total labor duration was 5 h 15 min. An episiotomy was repaired with internal sutures. Documented comorbidities and major risk factors were subclinical hypothyroidism during pregnancy; bilateral myopia; group B Streptococcus infection during pregnancy; vulvovaginal candidiasis during pregnancy; suspected fetal distress. Neonatal birth weight was 3730 g; Apgar scores at 1, 5, and 10 minutes were 10, 10, 10; and the delivery outcome was live birth. | |
| **模型生成的产褥期护理计划 Model-generated postpartum nursing care plan** | 产妇26岁，G1P1，孕39+3周，经产钳助产阴道分娩分娩，产时出血约300ml，新生儿女婴、体重3730g，Apgar评分10、10、10分。病例提示的重点风险为胎膜早破或感染相关风险、甲状腺功能异常、胎儿窘迫或羊水异常记录。产后24小时内重点评估体温、脉搏、血压、宫底高度、宫缩、阴道流血、恶露颜色和尿潴留，记录首次排尿和疼痛评分。产后住院及出院后继续观察恶露是否由红色逐渐转淡，乳房是否胀痛，乳头是否皲裂，睡眠和情绪是否稳定。饮食以优质蛋白、含铁食物、蔬菜水果和足量饮水为主，避免盲目大补和长期卧床。本次为产钳助产阴道分娩，会阴伤口、盆底和排尿功能是护理重点。每次大小便后用温水从前向后清洁会阴并轻轻擦干，观察侧切或裂伤处肿胀、硬结、渗血、分泌物和异味。疼痛明显时可侧卧休息，避免久蹲、负重和过早剧烈活动。哺乳方面建议尽早母婴皮肤接触和按需哺乳，指导正确含接，乳胀时先热敷或轻柔按摩再哺乳，乳头疼痛时及时调整姿势。若出现乳房局部红肿硬痛、寒战或高热，应警惕乳腺炎。合并甲状腺疾病者，应按内分泌医嘱继续或调整用药，不自行停药，产后复查甲状腺功能，观察心悸、怕冷、乏力、出汗和情绪异常。存在胎膜早破、B族链球菌感染、阴道炎或宫内感染疑虑时，应加强体温、下腹压痛、恶露气味、会阴或切口渗液观察，发热或恶露恶臭时及时复诊。因有胎儿窘迫、羊水污染或新生儿评分变化记录，母婴同室期间应配合观察新生儿吃奶、肤色、体温、呼吸和反应，发现吃奶差、发绀、嗜睡或呼吸异常应立即告知儿科医生。出院前应完成用药、伤口、哺乳和避孕宣教，42天左右复查，必要时提前复诊。若出现阴道流血突然增多、鲜红血浸湿卫生巾、恶露恶臭、发热、持续腹痛、伤口化脓、排尿困难、下肢单侧肿痛、胸闷气短，或持续悲伤焦虑及伤害自己或婴儿的想法，应立即就医或急诊处理。 | 患者26岁，初产妇，产钳助产+侧切，合并亚临床甲减、双眼近视、GBS感染、霉菌性阴道炎、可疑胎儿窘迫，脐带假结+扭转20周。亚临床甲减继续左甲状腺素，6周复查。GBS+霉菌性阴道炎，注意产褥感染，监测体温、恶露。高度近视避免用力屏气。脐带假结+扭转，新生儿观察神经系统。会阴侧切每日消毒。提供心理支持。42天复查甲功、眼科；新生儿随访。 |
| **护理计划英文翻译 English translation of care plan** | The patient is 26 years old, G1P1, delivered at 39+3 weeks by forceps-assisted vaginal delivery, with an estimated intrapartum blood loss of 300 mL. The newborn was a female infant, weighed 3730 g, and had Apgar scores of 10, 10, 10. The main case-based risks include premature rupture of membranes or infection-related risk, thyroid dysfunction, fetal distress or abnormal amniotic fluid findings. Within the first 24 hours, assess temperature, pulse, blood pressure, uterine fundal height, uterine tone, vaginal bleeding, lochia color, urinary retention, first voiding, and pain score. During hospitalization and after discharge, continue to observe whether lochia gradually changes from red to lighter color, whether breast engorgement or nipple fissure occurs, and whether sleep and mood remain stable. Diet should include high-quality protein, iron-rich food, vegetables, fruit, and adequate fluids. Avoid blind over-supplementation and prolonged bed rest. Because this was a forceps-assisted vaginal delivery, perineal wound care, pelvic floor recovery, and urinary function are priorities. Rinse the perineum from front to back with warm water after urination or bowel movement and pat dry. Observe the episiotomy or tear for swelling, induration, bleeding, discharge, and odor. Rest in a lateral position if pain is marked, and avoid prolonged squatting, heavy lifting, and early vigorous activity. For breastfeeding, encourage early skin-to-skin contact and feeding on demand. Teach correct latch and positioning. For engorgement, apply warmth or gentle massage before feeding. If nipple pain occurs, adjust latch promptly. Localized breast redness, hard painful swelling, chills, or high fever should raise concern for mastitis. For thyroid disease, continue or adjust medication only under endocrine guidance, recheck thyroid function after delivery, and watch for palpitations, cold intolerance, fatigue, sweating, or abnormal mood changes. With premature rupture of membranes, group B streptococcus, vaginitis, or suspected intrauterine infection, monitor temperature, lower abdominal tenderness, lochia odor, and drainage from the perineal wound or incision. Return promptly for fever or foul-smelling lochia. Because fetal distress, meconium-stained fluid, or changed Apgar scores were recorded, cooperate with pediatric observation of feeding, skin color, temperature, breathing, and responsiveness. Poor feeding, cyanosis, excessive sleepiness, or abnormal breathing should be reported immediately. Before discharge, provide education on medication, wound care, breastfeeding, and contraception. Arrange a postpartum review around 42 days after delivery, or earlier if symptoms occur. Seek urgent medical care for sudden heavy vaginal bleeding, bright red bleeding soaking pads, foul-smelling lochia, fever, persistent abdominal pain, pus from the wound, difficulty urinating, unilateral leg swelling or pain, chest tightness, shortness of breath, or persistent sadness, anxiety, or thoughts of harming herself or the baby. | The patient is a 26-year-old primipara with forceps delivery and episiotomy. Comorbidities: subclinical hypothyroidism, myopia, GBS, vulvovaginal candidiasis, suspected fetal distress, cord false knot + twisting 20 loops. Continue levothyroxine, retest at 6 weeks. High infection risk – monitor temperature and lochia. High myopia – avoid Valsalva. Newborn neurological observation. Episiotomy care. Psychological support. Follow-up: thyroid and eye exam at 42 days; newborn follow-up. |
| **Clinical appropriateness 临床适当性** | 5 | 4 |
| **Individualization 个体化程度** | 5 | 5 |
| **Comprehensiveness 完整性** | 5 | 3 |
| **Actionability 可操作性** | 4 | 4 |
| **Safety 安全性** | 5 | 4 |
| **Triage appropriateness 分诊/转诊建议适当性** | 5 | 3 |
| **Structure and clarity 结构与清晰度** | 4 | 3 |

**病例 55 / Case 55**

| **项目 Item** | **ChatGPT 5.5** | **DeepSeek V3** |
| --- | --- | --- |
| **去标识化临床病例摘要 De-identified Clinical Case Summary** | 患者31岁，孕产史为G3P1，既往剖宫产0次，孕周40+3，临产类别为催产素引产。人工破膜，羊水量约300ml，羊水性质为Ⅲ°（质粘稠）。以自然分娩分娩，产时出血约320ml。第一产程14时0分，第二产程1时9分，第三产程0时6分，总产程15时15分，会阴或切口情况为侧切，内缝。合并症及主要风险因素：妊娠期糖尿病；甲状腺功能减退症。新生儿出生体重3500g，Apgar评分1、5、10分钟分别为10、10、10分，分娩结局为活产。 | |
| **临床病例摘要英文翻译 English Translation of De-identified Clinical Case Summary** | Maternal age was 31 years. The obstetric history was G3P1, with 0 previous cesarean deliveries. Gestational age was 40+3 weeks, and the labor onset or induction category was oxytocin induction. The membranes were artificially ruptured; the amniotic fluid volume was approximately 300 mL and its character was thick grade III meconium-stained. The patient had a spontaneous vaginal delivery, with estimated intrapartum blood loss of 320 mL. The first, second, and third stages of labor were 14 h 0 min, 1 h 9 min, and 0 h 6 min, respectively; the total labor duration was 15 h 15 min. An episiotomy was repaired with internal sutures. Documented comorbidities and major risk factors were gestational diabetes mellitus; hypothyroidism. Neonatal birth weight was 3500 g; Apgar scores at 1, 5, and 10 minutes were 10, 10, 10; and the delivery outcome was live birth. | |
| **模型生成的产褥期护理计划 Model-generated postpartum nursing care plan** | 产妇31岁，G3P1，孕40+3周，经阴道自然分娩分娩，产时出血约320ml，新生儿女婴、体重3500g，Apgar评分10、10、10分。病例提示的重点风险为妊娠期糖尿病、甲状腺功能异常。产后24小时内重点评估体温、脉搏、血压、宫底高度、宫缩、阴道流血、恶露颜色和尿潴留，记录首次排尿和疼痛评分。产后住院及出院后继续观察恶露是否由红色逐渐转淡，乳房是否胀痛，乳头是否皲裂，睡眠和情绪是否稳定。饮食以优质蛋白、含铁食物、蔬菜水果和足量饮水为主，避免盲目大补和长期卧床。本次为阴道自然分娩，应重点观察会阴裂伤或缝合处恢复。每次大小便后保持局部清洁干燥，观察红肿、渗血、疼痛加重和异常气味。无头晕和出血增多时逐步增加活动，并在医护指导下开始轻柔盆底肌训练。哺乳方面建议尽早母婴皮肤接触和按需哺乳，指导正确含接，乳胀时先热敷或轻柔按摩再哺乳，乳头疼痛时及时调整姿势。若出现乳房局部红肿硬痛、寒战或高热，应警惕乳腺炎。有妊娠期糖尿病者，产后饮食应控制精制糖和高油食物，少量多餐，结合恢复情况逐步活动，并按医嘱在产后复查血糖或糖耐量。合并甲状腺疾病者，应按内分泌医嘱继续或调整用药，不自行停药，产后复查甲状腺功能，观察心悸、怕冷、乏力、出汗和情绪异常。出院前应完成用药、伤口、哺乳和避孕宣教，42天左右复查，必要时提前复诊。若出现阴道流血突然增多、鲜红血浸湿卫生巾、恶露恶臭、发热、持续腹痛、伤口化脓、排尿困难、下肢单侧肿痛、胸闷气短，或持续悲伤焦虑及伤害自己或婴儿的想法，应立即就医或急诊处理。 | 患者31岁，G3P1，自然分娩+侧切，合并GDM、甲减、脐带绕颈1周，羊水Ⅲ°粘稠，产时出血320ml。GDM产后监测血糖，饮食控制。甲减继续左甲状腺素，6周复查。新生儿需监测MAS及低血糖。会阴侧切护理。出血320ml，注意晚期出血。鼓励母乳喂养。42天复查血糖、甲功；新生儿随访。 |
| **护理计划英文翻译 English translation of care plan** | The patient is 31 years old, G3P1, delivered at 40+3 weeks by spontaneous vaginal delivery, with an estimated intrapartum blood loss of 320 mL. The newborn was a female infant, weighed 3500 g, and had Apgar scores of 10, 10, 10. The main case-based risks include gestational diabetes mellitus, thyroid dysfunction. Within the first 24 hours, assess temperature, pulse, blood pressure, uterine fundal height, uterine tone, vaginal bleeding, lochia color, urinary retention, first voiding, and pain score. During hospitalization and after discharge, continue to observe whether lochia gradually changes from red to lighter color, whether breast engorgement or nipple fissure occurs, and whether sleep and mood remain stable. Diet should include high-quality protein, iron-rich food, vegetables, fruit, and adequate fluids. Avoid blind over-supplementation and prolonged bed rest. Because this was a spontaneous vaginal birth, recovery of any perineal tear or suture site should be observed. Keep the area clean and dry after urination or bowel movement and watch for redness, swelling, bleeding, worsening pain, or abnormal odor. If there is no dizziness or increased bleeding, increase activity gradually and begin gentle pelvic floor exercises under professional guidance. For breastfeeding, encourage early skin-to-skin contact and feeding on demand. Teach correct latch and positioning. For engorgement, apply warmth or gentle massage before feeding. If nipple pain occurs, adjust latch promptly. Localized breast redness, hard painful swelling, chills, or high fever should raise concern for mastitis. For gestational diabetes, avoid refined sugar and heavy oily foods, eat small balanced meals, increase activity gradually, and arrange postpartum glucose or oral glucose tolerance testing as advised. For thyroid disease, continue or adjust medication only under endocrine guidance, recheck thyroid function after delivery, and watch for palpitations, cold intolerance, fatigue, sweating, or abnormal mood changes. Before discharge, provide education on medication, wound care, breastfeeding, and contraception. Arrange a postpartum review around 42 days after delivery, or earlier if symptoms occur. Seek urgent medical care for sudden heavy vaginal bleeding, bright red bleeding soaking pads, foul-smelling lochia, fever, persistent abdominal pain, pus from the wound, difficulty urinating, unilateral leg swelling or pain, chest tightness, shortness of breath, or persistent sadness, anxiety, or thoughts of harming herself or the baby. | The patient is a 31-year-old G3P1 with spontaneous delivery and episiotomy. Comorbidities: GDM, hypothyroidism, nuchal cord 1 loop, viscous grade III meconium. Blood loss 320 mL. Monitor postpartum glucose. Continue levothyroxine, retest at 6 weeks. Newborn – monitor for MAS and hypoglycemia. Episiotomy care. Watch for late PPH. Encourage breastfeeding. Follow-up: glucose and thyroid at 42 days; newborn follow-up. |
| **Clinical appropriateness 临床适当性** | 5 | 4 |
| **Individualization 个体化程度** | 4 | 5 |
| **Comprehensiveness 完整性** | 5 | 3 |
| **Actionability 可操作性** | 4 | 4 |
| **Safety 安全性** | 5 | 4 |
| **Triage appropriateness 分诊/转诊建议适当性** | 5 | 3 |
| **Structure and clarity 结构与清晰度** | 4 | 4 |

**病例 56 / Case 56**

| **项目 Item** | **ChatGPT 5.5** | **DeepSeek V3** |
| --- | --- | --- |
| **去标识化临床病例摘要 De-identified Clinical Case Summary** | 患者33岁，孕产史为G2P1，既往剖宫产0次，孕周39+3，临产类别为自然临产。自然破膜，羊水量约300ml，羊水性质为清。以产钳分娩，产时出血约260ml。第一产程3时30分，第二产程0时20分，第三产程0时10分，总产程4时0分，会阴或切口情况为侧切，内缝。合并症及主要风险因素：妊娠期糖尿病；妊娠合并甲状腺功能减退；胎儿宫内窘迫。新生儿出生体重3610g，Apgar评分1、5、10分钟分别为10、10、10分，分娩结局为活产。 | |
| **临床病例摘要英文翻译 English Translation of De-identified Clinical Case Summary** | Maternal age was 33 years. The obstetric history was G2P1, with 0 previous cesarean deliveries. Gestational age was 39+3 weeks, and the labor onset or induction category was spontaneous labor. The membranes ruptured spontaneously; the amniotic fluid volume was approximately 300 mL and its character was clear. The patient had a forceps-assisted vaginal delivery, with estimated intrapartum blood loss of 260 mL. The first, second, and third stages of labor were 3 h 30 min, 0 h 20 min, and 0 h 10 min, respectively; the total labor duration was 4 h 0 min. An episiotomy was repaired with internal sutures. Documented comorbidities and major risk factors were gestational diabetes mellitus; hypothyroidism during pregnancy; fetal distress. Neonatal birth weight was 3610 g; Apgar scores at 1, 5, and 10 minutes were 10, 10, 10; and the delivery outcome was live birth. | |
| **模型生成的产褥期护理计划 Model-generated postpartum nursing care plan** | 产妇33岁，G2P1，孕39+3周，经产钳助产阴道分娩分娩，产时出血约260ml，新生儿男婴、体重3610g，Apgar评分10、10、10分。病例提示的重点风险为妊娠期糖尿病、甲状腺功能异常、胎儿窘迫或羊水异常记录。产后24小时内重点评估体温、脉搏、血压、宫底高度、宫缩、阴道流血、恶露颜色和尿潴留，记录首次排尿和疼痛评分。产后住院及出院后继续观察恶露是否由红色逐渐转淡，乳房是否胀痛，乳头是否皲裂，睡眠和情绪是否稳定。饮食以优质蛋白、含铁食物、蔬菜水果和足量饮水为主，避免盲目大补和长期卧床。本次为产钳助产阴道分娩，会阴伤口、盆底和排尿功能是护理重点。每次大小便后用温水从前向后清洁会阴并轻轻擦干，观察侧切或裂伤处肿胀、硬结、渗血、分泌物和异味。疼痛明显时可侧卧休息，避免久蹲、负重和过早剧烈活动。哺乳方面建议尽早母婴皮肤接触和按需哺乳，指导正确含接，乳胀时先热敷或轻柔按摩再哺乳，乳头疼痛时及时调整姿势。若出现乳房局部红肿硬痛、寒战或高热，应警惕乳腺炎。有妊娠期糖尿病者，产后饮食应控制精制糖和高油食物，少量多餐，结合恢复情况逐步活动，并按医嘱在产后复查血糖或糖耐量。合并甲状腺疾病者，应按内分泌医嘱继续或调整用药，不自行停药，产后复查甲状腺功能，观察心悸、怕冷、乏力、出汗和情绪异常。因有胎儿窘迫、羊水污染或新生儿评分变化记录，母婴同室期间应配合观察新生儿吃奶、肤色、体温、呼吸和反应，发现吃奶差、发绀、嗜睡或呼吸异常应立即告知儿科医生。出院前应完成用药、伤口、哺乳和避孕宣教，42天左右复查，必要时提前复诊。若出现阴道流血突然增多、鲜红血浸湿卫生巾、恶露恶臭、发热、持续腹痛、伤口化脓、排尿困难、下肢单侧肿痛、胸闷气短，或持续悲伤焦虑及伤害自己或婴儿的想法，应立即就医或急诊处理。 | 患者33岁，G2P1，产钳+侧切，合并GDM、甲减、胎儿宫内窘迫，出血260ml。产后监测血糖、继续左甲状腺素。新生儿观察窘迫后遗症。会阴侧切护理。出血260ml正常。提供心理支持。42天复查血糖、甲功。 |
| **护理计划英文翻译 English translation of care plan** | The patient is 33 years old, G2P1, delivered at 39+3 weeks by forceps-assisted vaginal delivery, with an estimated intrapartum blood loss of 260 mL. The newborn was a male infant, weighed 3610 g, and had Apgar scores of 10, 10, 10. The main case-based risks include gestational diabetes mellitus, thyroid dysfunction, fetal distress or abnormal amniotic fluid findings. Within the first 24 hours, assess temperature, pulse, blood pressure, uterine fundal height, uterine tone, vaginal bleeding, lochia color, urinary retention, first voiding, and pain score. During hospitalization and after discharge, continue to observe whether lochia gradually changes from red to lighter color, whether breast engorgement or nipple fissure occurs, and whether sleep and mood remain stable. Diet should include high-quality protein, iron-rich food, vegetables, fruit, and adequate fluids. Avoid blind over-supplementation and prolonged bed rest. Because this was a forceps-assisted vaginal delivery, perineal wound care, pelvic floor recovery, and urinary function are priorities. Rinse the perineum from front to back with warm water after urination or bowel movement and pat dry. Observe the episiotomy or tear for swelling, induration, bleeding, discharge, and odor. Rest in a lateral position if pain is marked, and avoid prolonged squatting, heavy lifting, and early vigorous activity. For breastfeeding, encourage early skin-to-skin contact and feeding on demand. Teach correct latch and positioning. For engorgement, apply warmth or gentle massage before feeding. If nipple pain occurs, adjust latch promptly. Localized breast redness, hard painful swelling, chills, or high fever should raise concern for mastitis. For gestational diabetes, avoid refined sugar and heavy oily foods, eat small balanced meals, increase activity gradually, and arrange postpartum glucose or oral glucose tolerance testing as advised. For thyroid disease, continue or adjust medication only under endocrine guidance, recheck thyroid function after delivery, and watch for palpitations, cold intolerance, fatigue, sweating, or abnormal mood changes. Because fetal distress, meconium-stained fluid, or changed Apgar scores were recorded, cooperate with pediatric observation of feeding, skin color, temperature, breathing, and responsiveness. Poor feeding, cyanosis, excessive sleepiness, or abnormal breathing should be reported immediately. Before discharge, provide education on medication, wound care, breastfeeding, and contraception. Arrange a postpartum review around 42 days after delivery, or earlier if symptoms occur. Seek urgent medical care for sudden heavy vaginal bleeding, bright red bleeding soaking pads, foul-smelling lochia, fever, persistent abdominal pain, pus from the wound, difficulty urinating, unilateral leg swelling or pain, chest tightness, shortness of breath, or persistent sadness, anxiety, or thoughts of harming herself or the baby. | The patient is a 33-year-old G2P1 with forceps delivery and episiotomy. Comorbidities: GDM, hypothyroidism, fetal distress. Blood loss 260 mL. Monitor glucose; continue levothyroxine. Newborn observation for sequelae of distress. Episiotomy care. Psychological support. Follow-up: glucose and thyroid at 42 days. |
| **Clinical appropriateness 临床适当性** | 5 | 3 |
| **Individualization 个体化程度** | 5 | 3 |
| **Comprehensiveness 完整性** | 5 | 3 |
| **Actionability 可操作性** | 4 | 3 |
| **Safety 安全性** | 5 | 3 |
| **Triage appropriateness 分诊/转诊建议适当性** | 5 | 3 |
| **Structure and clarity 结构与清晰度** | 3 | 3 |

**病例 57 / Case 57**

| **项目 Item** | **ChatGPT 5.5** | **DeepSeek V3** |
| --- | --- | --- |
| **去标识化临床病例摘要 De-identified Clinical Case Summary** | 患者29岁，孕产史为G2P2，既往剖宫产0次，孕周37+4，临产类别为自然临产。自然破膜，羊水量约400ml，羊水性质为清。以自然分娩分娩，产时出血约210ml。第一产程3时30分，第二产程0时19分，第三产程0时6分，总产程3时55分，会阴或切口情况为Ⅰ，内缝。合并症及主要风险因素：无。新生儿出生体重3810g，Apgar评分1、5、10分钟分别为10、10、10分，分娩结局为活产。 | |
| **临床病例摘要英文翻译 English Translation of De-identified Clinical Case Summary** | Maternal age was 29 years. The obstetric history was G2P2, with 0 previous cesarean deliveries. Gestational age was 37+4 weeks, and the labor onset or induction category was spontaneous labor. The membranes ruptured spontaneously; the amniotic fluid volume was approximately 400 mL and its character was clear. The patient had a spontaneous vaginal delivery, with estimated intrapartum blood loss of 210 mL. The first, second, and third stages of labor were 3 h 30 min, 0 h 19 min, and 0 h 6 min, respectively; the total labor duration was 3 h 55 min. A first-degree perineal laceration was repaired with internal sutures. No comorbidities or major risk factors were documented. Neonatal birth weight was 3810 g; Apgar scores at 1, 5, and 10 minutes were 10, 10, 10; and the delivery outcome was live birth. | |
| **模型生成的产褥期护理计划 Model-generated postpartum nursing care plan** | 产妇29岁，G2P2，孕37+4周，经阴道自然分娩分娩，产时出血约210ml，新生儿男婴、体重3810g，Apgar评分10、10、10分。病例提示的重点风险为未记录特殊合并症。产后24小时内重点评估体温、脉搏、血压、宫底高度、宫缩、阴道流血、恶露颜色和尿潴留，记录首次排尿和疼痛评分。产后住院及出院后继续观察恶露是否由红色逐渐转淡，乳房是否胀痛，乳头是否皲裂，睡眠和情绪是否稳定。饮食以优质蛋白、含铁食物、蔬菜水果和足量饮水为主，避免盲目大补和长期卧床。本次为阴道自然分娩，应重点观察会阴裂伤或缝合处恢复。每次大小便后保持局部清洁干燥，观察红肿、渗血、疼痛加重和异常气味。无头晕和出血增多时逐步增加活动，并在医护指导下开始轻柔盆底肌训练。哺乳方面建议尽早母婴皮肤接触和按需哺乳，指导正确含接，乳胀时先热敷或轻柔按摩再哺乳，乳头疼痛时及时调整姿势。若出现乳房局部红肿硬痛、寒战或高热，应警惕乳腺炎。目前未记录特殊合并症，仍需按常规产褥期管理执行，重点是子宫复旧、恶露、伤口、哺乳、营养、活动和心理状态。出院前应完成用药、伤口、哺乳和避孕宣教，42天左右复查，必要时提前复诊。若出现阴道流血突然增多、鲜红血浸湿卫生巾、恶露恶臭、发热、持续腹痛、伤口化脓、排尿困难、下肢单侧肿痛、胸闷气短，或持续悲伤焦虑及伤害自己或婴儿的想法，应立即就医或急诊处理。 | 患者29岁，G2P2，自然分娩+I度裂伤，无合并症，胎盘重量910g（巨大胎盘）。巨大胎盘可能增加产后出血风险，需加强宫底按摩，观察恶露量。其他常规护理。42天复查。 |
| **护理计划英文翻译 English translation of care plan** | The patient is 29 years old, G2P2, delivered at 37+4 weeks by spontaneous vaginal delivery, with an estimated intrapartum blood loss of 210 mL. The newborn was a male infant, weighed 3810 g, and had Apgar scores of 10, 10, 10. The main case-based risks include no special comorbidity was recorded. Within the first 24 hours, assess temperature, pulse, blood pressure, uterine fundal height, uterine tone, vaginal bleeding, lochia color, urinary retention, first voiding, and pain score. During hospitalization and after discharge, continue to observe whether lochia gradually changes from red to lighter color, whether breast engorgement or nipple fissure occurs, and whether sleep and mood remain stable. Diet should include high-quality protein, iron-rich food, vegetables, fruit, and adequate fluids. Avoid blind over-supplementation and prolonged bed rest. Because this was a spontaneous vaginal birth, recovery of any perineal tear or suture site should be observed. Keep the area clean and dry after urination or bowel movement and watch for redness, swelling, bleeding, worsening pain, or abnormal odor. If there is no dizziness or increased bleeding, increase activity gradually and begin gentle pelvic floor exercises under professional guidance. For breastfeeding, encourage early skin-to-skin contact and feeding on demand. Teach correct latch and positioning. For engorgement, apply warmth or gentle massage before feeding. If nipple pain occurs, adjust latch promptly. Localized breast redness, hard painful swelling, chills, or high fever should raise concern for mastitis. No special complication was recorded, but routine postpartum management should still focus on uterine involution, lochia, wounds, breastfeeding, nutrition, activity, and mental health. Before discharge, provide education on medication, wound care, breastfeeding, and contraception. Arrange a postpartum review around 42 days after delivery, or earlier if symptoms occur. Seek urgent medical care for sudden heavy vaginal bleeding, bright red bleeding soaking pads, foul-smelling lochia, fever, persistent abdominal pain, pus from the wound, difficulty urinating, unilateral leg swelling or pain, chest tightness, shortness of breath, or persistent sadness, anxiety, or thoughts of harming herself or the baby. | The patient is a 29-year-old G2P2 with spontaneous delivery and first-degree laceration. No comorbidities except large placenta (910 g). Large placenta may increase PPH risk – aggressive fundal massage, monitor lochia. Otherwise routine care. Follow-up at 42 days. |
| **Clinical appropriateness 临床适当性** | 4 | 3 |
| **Individualization 个体化程度** | 3 | 3 |
| **Comprehensiveness 完整性** | 4 | 3 |
| **Actionability 可操作性** | 4 | 3 |
| **Safety 安全性** | 5 | 3 |
| **Triage appropriateness 分诊/转诊建议适当性** | 5 | 3 |
| **Structure and clarity 结构与清晰度** | 3 | 4 |

**病例 58 / Case 58**

| **项目 Item** | **ChatGPT 5.5** | **DeepSeek V3** |
| --- | --- | --- |
| **去标识化临床病例摘要 De-identified Clinical Case Summary** | 患者32岁，孕产史为G1P1，既往剖宫产0次，孕周40+2，临产类别为催产素引产。自然破膜，羊水量约300ml，羊水性质为清。以自然分娩分娩，产时出血约1315ml。第一产程11时20分，第二产程0时38分，第三产程0时6分，总产程12时4分，会阴或切口情况为Ⅰ，内缝。合并症及主要风险因素：胎膜早破、双眼近视、胎盘血窦。新生儿出生体重3970g，Apgar评分1、5、10分钟分别为10、10、10分，分娩结局为活产。 | |
| **临床病例摘要英文翻译 English Translation of De-identified Clinical Case Summary** | Maternal age was 32 years. The obstetric history was G1P1, with 0 previous cesarean deliveries. Gestational age was 40+2 weeks, and the labor onset or induction category was oxytocin induction. The membranes ruptured spontaneously; the amniotic fluid volume was approximately 300 mL and its character was clear. The patient had a spontaneous vaginal delivery, with estimated intrapartum blood loss of 1315 mL. The first, second, and third stages of labor were 11 h 20 min, 0 h 38 min, and 0 h 6 min, respectively; the total labor duration was 12 h 4 min. A first-degree perineal laceration was repaired with internal sutures. Documented comorbidities and major risk factors were premature rupture of membranes; bilateral myopia; placental venous lakes. Neonatal birth weight was 3970 g; Apgar scores at 1, 5, and 10 minutes were 10, 10, 10; and the delivery outcome was live birth. | |
| **模型生成的产褥期护理计划 Model-generated postpartum nursing care plan** | 产妇32岁，G1P1，孕40+2周，经阴道自然分娩分娩，产时出血约1315ml，新生儿男婴、体重3970g，Apgar评分10、10、10分。病例提示的重点风险为胎膜早破或感染相关风险、出血或贫血相关风险、胎盘相关风险。产后24小时内重点评估体温、脉搏、血压、宫底高度、宫缩、阴道流血、恶露颜色和尿潴留，记录首次排尿和疼痛评分。产后住院及出院后继续观察恶露是否由红色逐渐转淡，乳房是否胀痛，乳头是否皲裂，睡眠和情绪是否稳定。饮食以优质蛋白、含铁食物、蔬菜水果和足量饮水为主，避免盲目大补和长期卧床。本次为阴道自然分娩，应重点观察会阴裂伤或缝合处恢复。每次大小便后保持局部清洁干燥，观察红肿、渗血、疼痛加重和异常气味。无头晕和出血增多时逐步增加活动，并在医护指导下开始轻柔盆底肌训练。哺乳方面建议尽早母婴皮肤接触和按需哺乳，指导正确含接，乳胀时先热敷或轻柔按摩再哺乳，乳头疼痛时及时调整姿势。若出现乳房局部红肿硬痛、寒战或高热，应警惕乳腺炎。因存在出血量偏多、贫血或血小板异常相关风险，应复查血常规，关注头晕、心悸、乏力、面色苍白、恶露突然增多或较大血块，按医嘱补铁，必要时评估凝血和子宫复旧。存在胎膜早破、B族链球菌感染、阴道炎或宫内感染疑虑时，应加强体温、下腹压痛、恶露气味、会阴或切口渗液观察，发热或恶露恶臭时及时复诊。有胎盘位置、形态或胎盘血窦相关记录时，应注意恶露持续时间和出血量，产后复查可结合B超了解宫腔及子宫复旧。出院前应完成用药、伤口、哺乳和避孕宣教，42天左右复查，必要时提前复诊。若出现阴道流血突然增多、鲜红血浸湿卫生巾、恶露恶臭、发热、持续腹痛、伤口化脓、排尿困难、下肢单侧肿痛、胸闷气短，或持续悲伤焦虑及伤害自己或婴儿的想法，应立即就医或急诊处理。 | 患者32岁，初产妇，自然分娩+I度裂伤，合并胎膜早破、双眼近视、胎盘血窦，产时出血1315ml（严重PPH）。严重PPH需住院输血、补铁治疗，出院后监测血红蛋白，警惕晚期出血。血栓预防：尽早活动、弹力袜。胎盘血窦可能复发？注意观察。高度近视避免用力屏气。PPROM监测感染。PPH易致PTSD，心理支持。产后1周复查血常规；42天全面评估。 |
| **护理计划英文翻译 English translation of care plan** | The patient is 32 years old, G1P1, delivered at 40+2 weeks by spontaneous vaginal delivery, with an estimated intrapartum blood loss of 1315 mL. The newborn was a male infant, weighed 3970 g, and had Apgar scores of 10, 10, 10. The main case-based risks include premature rupture of membranes or infection-related risk, bleeding or anemia-related risk, placental risk factors. Within the first 24 hours, assess temperature, pulse, blood pressure, uterine fundal height, uterine tone, vaginal bleeding, lochia color, urinary retention, first voiding, and pain score. During hospitalization and after discharge, continue to observe whether lochia gradually changes from red to lighter color, whether breast engorgement or nipple fissure occurs, and whether sleep and mood remain stable. Diet should include high-quality protein, iron-rich food, vegetables, fruit, and adequate fluids. Avoid blind over-supplementation and prolonged bed rest. Because this was a spontaneous vaginal birth, recovery of any perineal tear or suture site should be observed. Keep the area clean and dry after urination or bowel movement and watch for redness, swelling, bleeding, worsening pain, or abnormal odor. If there is no dizziness or increased bleeding, increase activity gradually and begin gentle pelvic floor exercises under professional guidance. For breastfeeding, encourage early skin-to-skin contact and feeding on demand. Teach correct latch and positioning. For engorgement, apply warmth or gentle massage before feeding. If nipple pain occurs, adjust latch promptly. Localized breast redness, hard painful swelling, chills, or high fever should raise concern for mastitis. Because increased blood loss, anemia, or platelet abnormality is a concern, repeat a complete blood count as advised and watch for dizziness, palpitations, severe fatigue, pallor, sudden increase in lochia, or large clots. Iron supplementation, nutrition support, and assessment of uterine involution or coagulation should follow medical orders. With premature rupture of membranes, group B streptococcus, vaginitis, or suspected intrauterine infection, monitor temperature, lower abdominal tenderness, lochia odor, and drainage from the perineal wound or incision. Return promptly for fever or foul-smelling lochia. For abnormal placental location, shape, or placental sinus records, monitor bleeding amount and duration of lochia. Ultrasound review of the uterine cavity and involution may be needed at follow-up. Before discharge, provide education on medication, wound care, breastfeeding, and contraception. Arrange a postpartum review around 42 days after delivery, or earlier if symptoms occur. Seek urgent medical care for sudden heavy vaginal bleeding, bright red bleeding soaking pads, foul-smelling lochia, fever, persistent abdominal pain, pus from the wound, difficulty urinating, unilateral leg swelling or pain, chest tightness, shortness of breath, or persistent sadness, anxiety, or thoughts of harming herself or the baby. | The patient is a 32-year-old primipara with spontaneous delivery and first-degree laceration. Comorbidities: PPROM, myopia, placental venous lake. Severe PPH 1315 mL – hospitalization, transfusion, iron. Post-discharge monitor Hb, watch for late PPH. Thrombosis prevention – early ambulation, compression stockings. High myopia – avoid Valsalva. Monitor infection. PTSD risk – psychological support. Follow-up: CBC at 1 week; comprehensive exam at 42 days. |
| **Clinical appropriateness 临床适当性** | 5 | 4 |
| **Individualization 个体化程度** | 5 | 5 |
| **Comprehensiveness 完整性** | 5 | 3 |
| **Actionability 可操作性** | 4 | 4 |
| **Safety 安全性** | 5 | 4 |
| **Triage appropriateness 分诊/转诊建议适当性** | 5 | 3 |
| **Structure and clarity 结构与清晰度** | 4 | 3 |

**病例 59 / Case 59**

| **项目 Item** | **ChatGPT 5.5** | **DeepSeek V3** |
| --- | --- | --- |
| **去标识化临床病例摘要 De-identified Clinical Case Summary** | 患者36岁，孕产史为G3P1，既往剖宫产0次，孕周38+3，临产类别为催产素引产。自然破膜，羊水量约300ml，羊水性质为清。以自然分娩分娩，产时出血约220ml。第一产程3时6分，第二产程0时29分，第三产程0时10分，总产程3时45分，会阴或切口情况为Ⅰ，内缝。合并症及主要风险因素：胎膜早破、妊娠合并亚临床甲状腺功能减退症、高龄初孕妇的监督、妊娠合并多囊卵巢、胎儿宫内窘迫？。新生儿出生体重3250g，Apgar评分1、5、10分钟分别为10、10、10分，分娩结局为活产。 | |
| **临床病例摘要英文翻译 English Translation of De-identified Clinical Case Summary** | Maternal age was 36 years. The obstetric history was G3P1, with 0 previous cesarean deliveries. Gestational age was 38+3 weeks, and the labor onset or induction category was oxytocin induction. The membranes ruptured spontaneously; the amniotic fluid volume was approximately 300 mL and its character was clear. The patient had a spontaneous vaginal delivery, with estimated intrapartum blood loss of 220 mL. The first, second, and third stages of labor were 3 h 6 min, 0 h 29 min, and 0 h 10 min, respectively; the total labor duration was 3 h 45 min. A first-degree perineal laceration was repaired with internal sutures. Documented comorbidities and major risk factors were premature rupture of membranes; subclinical hypothyroidism during pregnancy; advanced maternal age in a primigravida; polycystic ovary syndrome during pregnancy; suspected fetal distress. Neonatal birth weight was 3250 g; Apgar scores at 1, 5, and 10 minutes were 10, 10, 10; and the delivery outcome was live birth. | |
| **模型生成的产褥期护理计划 Model-generated postpartum nursing care plan** | 产妇36岁，G3P1，孕38+3周，经阴道自然分娩分娩，产时出血约220ml，新生儿女婴、体重3250g，Apgar评分10、10、10分。病例提示的重点风险为胎膜早破或感染相关风险、甲状腺功能异常、胎儿窘迫或羊水异常记录、高龄妊娠因素。产后24小时内重点评估体温、脉搏、血压、宫底高度、宫缩、阴道流血、恶露颜色和尿潴留，记录首次排尿和疼痛评分。产后住院及出院后继续观察恶露是否由红色逐渐转淡，乳房是否胀痛，乳头是否皲裂，睡眠和情绪是否稳定。饮食以优质蛋白、含铁食物、蔬菜水果和足量饮水为主，避免盲目大补和长期卧床。本次为阴道自然分娩，应重点观察会阴裂伤或缝合处恢复。每次大小便后保持局部清洁干燥，观察红肿、渗血、疼痛加重和异常气味。无头晕和出血增多时逐步增加活动，并在医护指导下开始轻柔盆底肌训练。哺乳方面建议尽早母婴皮肤接触和按需哺乳，指导正确含接，乳胀时先热敷或轻柔按摩再哺乳，乳头疼痛时及时调整姿势。若出现乳房局部红肿硬痛、寒战或高热，应警惕乳腺炎。合并甲状腺疾病者，应按内分泌医嘱继续或调整用药，不自行停药，产后复查甲状腺功能，观察心悸、怕冷、乏力、出汗和情绪异常。存在胎膜早破、B族链球菌感染、阴道炎或宫内感染疑虑时，应加强体温、下腹压痛、恶露气味、会阴或切口渗液观察，发热或恶露恶臭时及时复诊。因有胎儿窘迫、羊水污染或新生儿评分变化记录，母婴同室期间应配合观察新生儿吃奶、肤色、体温、呼吸和反应，发现吃奶差、发绀、嗜睡或呼吸异常应立即告知儿科医生。合并子宫肌瘤、卵巢囊肿或多囊卵巢相关情况时，产后复查时建议结合妇科超声评估，若腹痛、包块感或异常出血加重，应提前就诊。出院前应完成用药、伤口、哺乳和避孕宣教，42天左右复查，必要时提前复诊。若出现阴道流血突然增多、鲜红血浸湿卫生巾、恶露恶臭、发热、持续腹痛、伤口化脓、排尿困难、下肢单侧肿痛、胸闷气短，或持续悲伤焦虑及伤害自己或婴儿的想法，应立即就医或急诊处理。 | 患者36岁，G3P1，自然分娩+I度裂伤，合并胎膜早破、亚临床甲减、高龄初产、多囊、可疑胎儿窘迫，脐带绕颈2周+真结。真结+绕颈2周，新生儿需密切监测缺氧缺血性脑病，建议儿科观察24-48小时。亚临床甲减继续左甲状腺素。PPROM监测感染。PCOS产后管理。高龄初产心理支持。常规会阴护理。42天复查甲功、血糖；新生儿专科随访。 |
| **护理计划英文翻译 English translation of care plan** | The patient is 36 years old, G3P1, delivered at 38+3 weeks by spontaneous vaginal delivery, with an estimated intrapartum blood loss of 220 mL. The newborn was a female infant, weighed 3250 g, and had Apgar scores of 10, 10, 10. The main case-based risks include premature rupture of membranes or infection-related risk, thyroid dysfunction, fetal distress or abnormal amniotic fluid findings, advanced maternal age. Within the first 24 hours, assess temperature, pulse, blood pressure, uterine fundal height, uterine tone, vaginal bleeding, lochia color, urinary retention, first voiding, and pain score. During hospitalization and after discharge, continue to observe whether lochia gradually changes from red to lighter color, whether breast engorgement or nipple fissure occurs, and whether sleep and mood remain stable. Diet should include high-quality protein, iron-rich food, vegetables, fruit, and adequate fluids. Avoid blind over-supplementation and prolonged bed rest. Because this was a spontaneous vaginal birth, recovery of any perineal tear or suture site should be observed. Keep the area clean and dry after urination or bowel movement and watch for redness, swelling, bleeding, worsening pain, or abnormal odor. If there is no dizziness or increased bleeding, increase activity gradually and begin gentle pelvic floor exercises under professional guidance. For breastfeeding, encourage early skin-to-skin contact and feeding on demand. Teach correct latch and positioning. For engorgement, apply warmth or gentle massage before feeding. If nipple pain occurs, adjust latch promptly. Localized breast redness, hard painful swelling, chills, or high fever should raise concern for mastitis. For thyroid disease, continue or adjust medication only under endocrine guidance, recheck thyroid function after delivery, and watch for palpitations, cold intolerance, fatigue, sweating, or abnormal mood changes. With premature rupture of membranes, group B streptococcus, vaginitis, or suspected intrauterine infection, monitor temperature, lower abdominal tenderness, lochia odor, and drainage from the perineal wound or incision. Return promptly for fever or foul-smelling lochia. Because fetal distress, meconium-stained fluid, or changed Apgar scores were recorded, cooperate with pediatric observation of feeding, skin color, temperature, breathing, and responsiveness. Poor feeding, cyanosis, excessive sleepiness, or abnormal breathing should be reported immediately. For uterine fibroids, ovarian cysts, or polycystic ovary related findings, pelvic ultrasound can be considered at postpartum review. Earlier assessment is needed for worsening abdominal pain, mass sensation, or abnormal bleeding. Before discharge, provide education on medication, wound care, breastfeeding, and contraception. Arrange a postpartum review around 42 days after delivery, or earlier if symptoms occur. Seek urgent medical care for sudden heavy vaginal bleeding, bright red bleeding soaking pads, foul-smelling lochia, fever, persistent abdominal pain, pus from the wound, difficulty urinating, unilateral leg swelling or pain, chest tightness, shortness of breath, or persistent sadness, anxiety, or thoughts of harming herself or the baby. | The patient is a 36-year-old G3P1 with spontaneous delivery and first-degree laceration. Comorbidities: PPROM, subclinical hypothyroidism, advanced maternal age in a primigravida, PCOS, suspected fetal distress, two loops of nuchal cord and a true knot. Because of the true knot, closely monitor the newborn for hypoxic-ischemic encephalopathy for 24–48 hours. Continue levothyroxine. Monitor infection. PCOS management. Psychological support. Routine perineal care. Follow-up: thyroid and glucose at 42 days; newborn specialist follow-up. |
| **Clinical appropriateness 临床适当性** | 5 | 4 |
| **Individualization 个体化程度** | 5 | 5 |
| **Comprehensiveness 完整性** | 5 | 3 |
| **Actionability 可操作性** | 5 | 4 |
| **Safety 安全性** | 5 | 4 |
| **Triage appropriateness 分诊/转诊建议适当性** | 5 | 3 |
| **Structure and clarity 结构与清晰度** | 4 | 3 |

**病例 60 / Case 60**

| **项目 Item** | **ChatGPT 5.5** | **DeepSeek V3** |
| --- | --- | --- |
| **去标识化临床病例摘要 De-identified Clinical Case Summary** | 患者30岁，孕产史为G1P1，既往剖宫产0次，孕周40，临产类别为自然临产。自然破膜，羊水量约300ml，羊水性质为清。以自然分娩分娩，产时出血约300ml。第一产程7时50分，第二产程1时2分，第三产程0时8分，总产程9时0分，会阴或切口情况为Ⅰ，内缝。合并症及主要风险因素：无。新生儿出生体重3350g，Apgar评分1、5、10分钟分别为10、10、10分，分娩结局为活产。 | |
| **临床病例摘要英文翻译 English Translation of De-identified Clinical Case Summary** | Maternal age was 30 years. The obstetric history was G1P1, with 0 previous cesarean deliveries. Gestational age was 40 weeks, and the labor onset or induction category was spontaneous labor. The membranes ruptured spontaneously; the amniotic fluid volume was approximately 300 mL and its character was clear. The patient had a spontaneous vaginal delivery, with estimated intrapartum blood loss of 300 mL. The first, second, and third stages of labor were 7 h 50 min, 1 h 2 min, and 0 h 8 min, respectively; the total labor duration was 9 h 0 min. A first-degree perineal laceration was repaired with internal sutures. No comorbidities or major risk factors were documented. Neonatal birth weight was 3350 g; Apgar scores at 1, 5, and 10 minutes were 10, 10, 10; and the delivery outcome was live birth. | |
| **模型生成的产褥期护理计划 Model-generated postpartum nursing care plan** | 产妇30岁，G1P1，孕40周，经阴道自然分娩分娩，产时出血约300ml，新生儿女婴、体重3350g，Apgar评分10、10、10分。病例提示的重点风险为未记录特殊合并症。产后24小时内重点评估体温、脉搏、血压、宫底高度、宫缩、阴道流血、恶露颜色和尿潴留，记录首次排尿和疼痛评分。产后住院及出院后继续观察恶露是否由红色逐渐转淡，乳房是否胀痛，乳头是否皲裂，睡眠和情绪是否稳定。饮食以优质蛋白、含铁食物、蔬菜水果和足量饮水为主，避免盲目大补和长期卧床。本次为阴道自然分娩，应重点观察会阴裂伤或缝合处恢复。每次大小便后保持局部清洁干燥，观察红肿、渗血、疼痛加重和异常气味。无头晕和出血增多时逐步增加活动，并在医护指导下开始轻柔盆底肌训练。哺乳方面建议尽早母婴皮肤接触和按需哺乳，指导正确含接，乳胀时先热敷或轻柔按摩再哺乳，乳头疼痛时及时调整姿势。若出现乳房局部红肿硬痛、寒战或高热，应警惕乳腺炎。目前未记录特殊合并症，仍需按常规产褥期管理执行，重点是子宫复旧、恶露、伤口、哺乳、营养、活动和心理状态。出院前应完成用药、伤口、哺乳和避孕宣教，42天左右复查，必要时提前复诊。若出现阴道流血突然增多、鲜红血浸湿卫生巾、恶露恶臭、发热、持续腹痛、伤口化脓、排尿困难、下肢单侧肿痛、胸闷气短，或持续悲伤焦虑及伤害自己或婴儿的想法，应立即就医或急诊处理。 | 患者30岁，初产妇，自然分娩+I度裂伤，无合并症，脐带绕颈1周（紧）。脐带绕颈紧，新生儿需观察呼吸、喂养、肌张力24小时。其他常规护理。42天复查。 |
| **护理计划英文翻译 English translation of care plan** | The patient is 30 years old, G1P1, delivered at 40 weeks by spontaneous vaginal delivery, with an estimated intrapartum blood loss of 300 mL. The newborn was a female infant, weighed 3350 g, and had Apgar scores of 10, 10, 10. The main case-based risks include no special comorbidity was recorded. Within the first 24 hours, assess temperature, pulse, blood pressure, uterine fundal height, uterine tone, vaginal bleeding, lochia color, urinary retention, first voiding, and pain score. During hospitalization and after discharge, continue to observe whether lochia gradually changes from red to lighter color, whether breast engorgement or nipple fissure occurs, and whether sleep and mood remain stable. Diet should include high-quality protein, iron-rich food, vegetables, fruit, and adequate fluids. Avoid blind over-supplementation and prolonged bed rest. Because this was a spontaneous vaginal birth, recovery of any perineal tear or suture site should be observed. Keep the area clean and dry after urination or bowel movement and watch for redness, swelling, bleeding, worsening pain, or abnormal odor. If there is no dizziness or increased bleeding, increase activity gradually and begin gentle pelvic floor exercises under professional guidance. For breastfeeding, encourage early skin-to-skin contact and feeding on demand. Teach correct latch and positioning. For engorgement, apply warmth or gentle massage before feeding. If nipple pain occurs, adjust latch promptly. Localized breast redness, hard painful swelling, chills, or high fever should raise concern for mastitis. No special complication was recorded, but routine postpartum management should still focus on uterine involution, lochia, wounds, breastfeeding, nutrition, activity, and mental health. Before discharge, provide education on medication, wound care, breastfeeding, and contraception. Arrange a postpartum review around 42 days after delivery, or earlier if symptoms occur. Seek urgent medical care for sudden heavy vaginal bleeding, bright red bleeding soaking pads, foul-smelling lochia, fever, persistent abdominal pain, pus from the wound, difficulty urinating, unilateral leg swelling or pain, chest tightness, shortness of breath, or persistent sadness, anxiety, or thoughts of harming herself or the baby. | The patient is a 30-year-old primipara with spontaneous delivery and first-degree laceration. No comorbidities except tight nuchal cord 1 loop. Newborn – observe respiration, feeding, tone for 24 hours. Otherwise routine care. Follow-up at 42 days. |
| **Clinical appropriateness 临床适当性** | 4 | 3 |
| **Individualization 个体化程度** | 3 | 3 |
| **Comprehensiveness 完整性** | 4 | 3 |
| **Actionability 可操作性** | 4 | 3 |
| **Safety 安全性** | 5 | 3 |
| **Triage appropriateness 分诊/转诊建议适当性** | 5 | 3 |
| **Structure and clarity 结构与清晰度** | 3 | 4 |

**病例 61 / Case 61**

| **项目 Item** | **ChatGPT 5.5** | **DeepSeek V3** |
| --- | --- | --- |
| **去标识化临床病例摘要 De-identified Clinical Case Summary** | 患者35岁，孕产史为G3P1，既往剖宫产0次，孕周41+1，临产类别为催产素引产。自然破膜，羊水量约300ml，羊水性质为清。以自然分娩分娩，产时出血约250ml。第一产程11时40分，第二产程0时47分，第三产程0时8分，总产程12时35分，会阴或切口情况为侧切，内缝。合并症及主要风险因素：不良孕产个人史；高龄初孕妇的监督；妊娠合并混合性阴道炎；妊娠合并高度近视；妊娠合并甲状腺功能减退症；胎膜早破；胎儿窘迫？。新生儿出生体重3190g，Apgar评分1、5、10分钟分别为10、10、10分，分娩结局为活产。 | |
| **临床病例摘要英文翻译 English Translation of De-identified Clinical Case Summary** | Maternal age was 35 years. The obstetric history was G3P1, with 0 previous cesarean deliveries. Gestational age was 41+1 weeks, and the labor onset or induction category was oxytocin induction. The membranes ruptured spontaneously; the amniotic fluid volume was approximately 300 mL and its character was clear. The patient had a spontaneous vaginal delivery, with estimated intrapartum blood loss of 250 mL. The first, second, and third stages of labor were 11 h 40 min, 0 h 47 min, and 0 h 8 min, respectively; the total labor duration was 12 h 35 min. An episiotomy was repaired with internal sutures. Documented comorbidities and major risk factors were poor obstetric history; advanced maternal age in a primigravida; mixed vaginitis during pregnancy; high myopia; hypothyroidism during pregnancy; premature rupture of membranes; suspected fetal distress. Neonatal birth weight was 3190 g; Apgar scores at 1, 5, and 10 minutes were 10, 10, 10; and the delivery outcome was live birth. | |
| **模型生成的产褥期护理计划 Model-generated postpartum nursing care plan** | 产妇35岁，G3P1，孕41+1周，经阴道自然分娩分娩，产时出血约250ml，新生儿男婴、体重3190g，Apgar评分10、10、10分。病例提示的重点风险为胎膜早破或感染相关风险、甲状腺功能异常、胎儿窘迫或羊水异常记录、高龄妊娠因素。产后24小时内重点评估体温、脉搏、血压、宫底高度、宫缩、阴道流血、恶露颜色和尿潴留，记录首次排尿和疼痛评分。产后住院及出院后继续观察恶露是否由红色逐渐转淡，乳房是否胀痛，乳头是否皲裂，睡眠和情绪是否稳定。饮食以优质蛋白、含铁食物、蔬菜水果和足量饮水为主，避免盲目大补和长期卧床。本次为阴道自然分娩，应重点观察会阴裂伤或缝合处恢复。每次大小便后保持局部清洁干燥，观察红肿、渗血、疼痛加重和异常气味。无头晕和出血增多时逐步增加活动，并在医护指导下开始轻柔盆底肌训练。哺乳方面建议尽早母婴皮肤接触和按需哺乳，指导正确含接，乳胀时先热敷或轻柔按摩再哺乳，乳头疼痛时及时调整姿势。若出现乳房局部红肿硬痛、寒战或高热，应警惕乳腺炎。合并甲状腺疾病者，应按内分泌医嘱继续或调整用药，不自行停药，产后复查甲状腺功能，观察心悸、怕冷、乏力、出汗和情绪异常。存在胎膜早破、B族链球菌感染、阴道炎或宫内感染疑虑时，应加强体温、下腹压痛、恶露气味、会阴或切口渗液观察，发热或恶露恶臭时及时复诊。因有胎儿窘迫、羊水污染或新生儿评分变化记录，母婴同室期间应配合观察新生儿吃奶、肤色、体温、呼吸和反应，发现吃奶差、发绀、嗜睡或呼吸异常应立即告知儿科医生。高龄产妇恢复期应避免过度劳累和连续熬夜，家属应参与夜间照护，保证睡眠、营养和情绪支持。出院前应完成用药、伤口、哺乳和避孕宣教，42天左右复查，必要时提前复诊。若出现阴道流血突然增多、鲜红血浸湿卫生巾、恶露恶臭、发热、持续腹痛、伤口化脓、排尿困难、下肢单侧肿痛、胸闷气短，或持续悲伤焦虑及伤害自己或婴儿的想法，应立即就医或急诊处理。 | 患者35岁，G3P1，自然分娩+侧切，合并不良孕产史、高龄初产妇、混合性阴道炎、高度近视、甲减、胎膜早破、可疑胎儿窘迫，产时出血250ml，新生儿Apgar 10。混合性阴道炎+PPROM，产褥感染风险高，每日测体温4次，观察恶露气味、子宫压痛；完成抗生素疗程。甲减继续左甲状腺素，6周复查。高度近视避免用力屏气。高龄初产及不良孕产史易致焦虑，提供心理支持。会阴侧切每日消毒。新生儿观察呼吸、喂养。产后1周复查感染指标，42天全面评估。 |
| **护理计划英文翻译 English translation of care plan** | The patient is 35 years old, G3P1, delivered at 41+1 weeks by spontaneous vaginal delivery, with an estimated intrapartum blood loss of 250 mL. The newborn was a male infant, weighed 3190 g, and had Apgar scores of 10, 10, 10. The main case-based risks include premature rupture of membranes or infection-related risk, thyroid dysfunction, fetal distress or abnormal amniotic fluid findings, advanced maternal age. Within the first 24 hours, assess temperature, pulse, blood pressure, uterine fundal height, uterine tone, vaginal bleeding, lochia color, urinary retention, first voiding, and pain score. During hospitalization and after discharge, continue to observe whether lochia gradually changes from red to lighter color, whether breast engorgement or nipple fissure occurs, and whether sleep and mood remain stable. Diet should include high-quality protein, iron-rich food, vegetables, fruit, and adequate fluids. Avoid blind over-supplementation and prolonged bed rest. Because this was a spontaneous vaginal birth, recovery of any perineal tear or suture site should be observed. Keep the area clean and dry after urination or bowel movement and watch for redness, swelling, bleeding, worsening pain, or abnormal odor. If there is no dizziness or increased bleeding, increase activity gradually and begin gentle pelvic floor exercises under professional guidance. For breastfeeding, encourage early skin-to-skin contact and feeding on demand. Teach correct latch and positioning. For engorgement, apply warmth or gentle massage before feeding. If nipple pain occurs, adjust latch promptly. Localized breast redness, hard painful swelling, chills, or high fever should raise concern for mastitis. For thyroid disease, continue or adjust medication only under endocrine guidance, recheck thyroid function after delivery, and watch for palpitations, cold intolerance, fatigue, sweating, or abnormal mood changes. With premature rupture of membranes, group B streptococcus, vaginitis, or suspected intrauterine infection, monitor temperature, lower abdominal tenderness, lochia odor, and drainage from the perineal wound or incision. Return promptly for fever or foul-smelling lochia. Because fetal distress, meconium-stained fluid, or changed Apgar scores were recorded, cooperate with pediatric observation of feeding, skin color, temperature, breathing, and responsiveness. Poor feeding, cyanosis, excessive sleepiness, or abnormal breathing should be reported immediately. Advanced maternal age may slow recovery, so the family should help with nighttime care and support adequate sleep, nutrition, and emotional stability. Before discharge, provide education on medication, wound care, breastfeeding, and contraception. Arrange a postpartum review around 42 days after delivery, or earlier if symptoms occur. Seek urgent medical care for sudden heavy vaginal bleeding, bright red bleeding soaking pads, foul-smelling lochia, fever, persistent abdominal pain, pus from the wound, difficulty urinating, unilateral leg swelling or pain, chest tightness, shortness of breath, or persistent sadness, anxiety, or thoughts of harming herself or the baby. | The patient is a 35-year-old G3P1 with spontaneous delivery and episiotomy. Comorbidities: poor obstetric history, advanced maternal age in a primigravida, mixed vaginitis, high myopia, hypothyroidism, PPROM, suspected fetal distress. Blood loss 250 mL, newborn Apgar 10. High infection risk – check temperature 4 times daily, monitor lochia odor and uterine tenderness; complete antibiotics. Continue levothyroxine, retest at 6 weeks. High myopia – avoid Valsalva. Psychological support due to advanced age and poor obstetric history. Episiotomy care. Newborn observation. Follow-up: infection markers at 1 week; comprehensive exam at 42 days. |
| **Clinical appropriateness 临床适当性** | 5 | 4 |
| **Individualization 个体化程度** | 5 | 5 |
| **Comprehensiveness 完整性** | 5 | 4 |
| **Actionability 可操作性** | 5 | 4 |
| **Safety 安全性** | 5 | 4 |
| **Triage appropriateness 分诊/转诊建议适当性** | 5 | 4 |
| **Structure and clarity 结构与清晰度** | 4 | 3 |

**病例 62 / Case 62**

| **项目 Item** | **ChatGPT 5.5** | **DeepSeek V3** |
| --- | --- | --- |
| **去标识化临床病例摘要 De-identified Clinical Case Summary** | 患者31岁，孕产史为G2P1，既往剖宫产0次，孕周40+1，临产类别为普贝生。自然破膜，羊水量约300ml，羊水性质为清。以产钳分娩，产时出血约400ml。第一产程2时37分，第二产程0时18分，第三产程0时5分，总产程3时0分，会阴或切口情况为侧切，内缝。合并症及主要风险因素：妊娠期糖尿病，胎儿宫内窘迫？。新生儿出生体重3410g，Apgar评分1、5、10分钟分别为10、10、10分，分娩结局为活产。 | |
| **临床病例摘要英文翻译 English Translation of De-identified Clinical Case Summary** | Maternal age was 31 years. The obstetric history was G2P1, with 0 previous cesarean deliveries. Gestational age was 40+1 weeks, and the labor onset or induction category was vaginal dinoprostone (Propess) induction. The membranes ruptured spontaneously; the amniotic fluid volume was approximately 300 mL and its character was clear. The patient had a forceps-assisted vaginal delivery, with estimated intrapartum blood loss of 400 mL. The first, second, and third stages of labor were 2 h 37 min, 0 h 18 min, and 0 h 5 min, respectively; the total labor duration was 3 h 0 min. An episiotomy was repaired with internal sutures. Documented comorbidities and major risk factors were gestational diabetes mellitus, suspected fetal distress. Neonatal birth weight was 3410 g; Apgar scores at 1, 5, and 10 minutes were 10, 10, 10; and the delivery outcome was live birth. | |
| **模型生成的产褥期护理计划 Model-generated postpartum nursing care plan** | 产妇31岁，G2P1，孕40+1周，经产钳助产阴道分娩分娩，产时出血约400ml，新生儿男婴、体重3410g，Apgar评分10、10、10分。病例提示的重点风险为妊娠期糖尿病、胎儿窘迫或羊水异常记录。产后24小时内重点评估体温、脉搏、血压、宫底高度、宫缩、阴道流血、恶露颜色和尿潴留，记录首次排尿和疼痛评分。产后住院及出院后继续观察恶露是否由红色逐渐转淡，乳房是否胀痛，乳头是否皲裂，睡眠和情绪是否稳定。饮食以优质蛋白、含铁食物、蔬菜水果和足量饮水为主，避免盲目大补和长期卧床。本次为产钳助产阴道分娩，会阴伤口、盆底和排尿功能是护理重点。每次大小便后用温水从前向后清洁会阴并轻轻擦干，观察侧切或裂伤处肿胀、硬结、渗血、分泌物和异味。疼痛明显时可侧卧休息，避免久蹲、负重和过早剧烈活动。哺乳方面建议尽早母婴皮肤接触和按需哺乳，指导正确含接，乳胀时先热敷或轻柔按摩再哺乳，乳头疼痛时及时调整姿势。若出现乳房局部红肿硬痛、寒战或高热，应警惕乳腺炎。产时出血量需要持续观察，建议每日记录恶露量、颜色和血块情况，活动后若明显增多，应减少活动并请医护评估。有妊娠期糖尿病者，产后饮食应控制精制糖和高油食物，少量多餐，结合恢复情况逐步活动，并按医嘱在产后复查血糖或糖耐量。因有胎儿窘迫、羊水污染或新生儿评分变化记录，母婴同室期间应配合观察新生儿吃奶、肤色、体温、呼吸和反应，发现吃奶差、发绀、嗜睡或呼吸异常应立即告知儿科医生。出院前应完成用药、伤口、哺乳和避孕宣教，42天左右复查，必要时提前复诊。若出现阴道流血突然增多、鲜红血浸湿卫生巾、恶露恶臭、发热、持续腹痛、伤口化脓、排尿困难、下肢单侧肿痛、胸闷气短，或持续悲伤焦虑及伤害自己或婴儿的想法，应立即就医或急诊处理。 | 患者31岁，G2P1，产钳助产+侧切，合并GDM、可疑胎儿窘迫，脐带绕颈1周、脐带隐性脱垂，产时出血400ml。GDM产后监测血糖，饮食控制。新生儿需监测呼吸、喂养，因脐带异常+可疑窘迫，建议观察24小时。出血400ml，注意宫缩乏力，加强按摩。会阴侧切每日消毒。提供心理支持。42天复查血糖；新生儿随访。 |
| **护理计划英文翻译 English translation of care plan** | The patient is 31 years old, G2P1, delivered at 40+1 weeks by forceps-assisted vaginal delivery, with an estimated intrapartum blood loss of 400 mL. The newborn was a male infant, weighed 3410 g, and had Apgar scores of 10, 10, 10. The main case-based risks include gestational diabetes mellitus, fetal distress or abnormal amniotic fluid findings. Within the first 24 hours, assess temperature, pulse, blood pressure, uterine fundal height, uterine tone, vaginal bleeding, lochia color, urinary retention, first voiding, and pain score. During hospitalization and after discharge, continue to observe whether lochia gradually changes from red to lighter color, whether breast engorgement or nipple fissure occurs, and whether sleep and mood remain stable. Diet should include high-quality protein, iron-rich food, vegetables, fruit, and adequate fluids. Avoid blind over-supplementation and prolonged bed rest. Because this was a forceps-assisted vaginal delivery, perineal wound care, pelvic floor recovery, and urinary function are priorities. Rinse the perineum from front to back with warm water after urination or bowel movement and pat dry. Observe the episiotomy or tear for swelling, induration, bleeding, discharge, and odor. Rest in a lateral position if pain is marked, and avoid prolonged squatting, heavy lifting, and early vigorous activity. For breastfeeding, encourage early skin-to-skin contact and feeding on demand. Teach correct latch and positioning. For engorgement, apply warmth or gentle massage before feeding. If nipple pain occurs, adjust latch promptly. Localized breast redness, hard painful swelling, chills, or high fever should raise concern for mastitis. Because blood loss requires closer observation, record the amount, color, and clots in the lochia every day. If bleeding increases clearly after activity, reduce activity and seek clinical assessment. For gestational diabetes, avoid refined sugar and heavy oily foods, eat small balanced meals, increase activity gradually, and arrange postpartum glucose or oral glucose tolerance testing as advised. Because fetal distress, meconium-stained fluid, or changed Apgar scores were recorded, cooperate with pediatric observation of feeding, skin color, temperature, breathing, and responsiveness. Poor feeding, cyanosis, excessive sleepiness, or abnormal breathing should be reported immediately. Before discharge, provide education on medication, wound care, breastfeeding, and contraception. Arrange a postpartum review around 42 days after delivery, or earlier if symptoms occur. Seek urgent medical care for sudden heavy vaginal bleeding, bright red bleeding soaking pads, foul-smelling lochia, fever, persistent abdominal pain, pus from the wound, difficulty urinating, unilateral leg swelling or pain, chest tightness, shortness of breath, or persistent sadness, anxiety, or thoughts of harming herself or the baby. | The patient is a 31-year-old G2P1 with forceps delivery and episiotomy. Comorbidities: GDM, suspected fetal distress, nuchal cord 1 loop, occult cord prolapse. Blood loss 400 mL. Monitor postpartum glucose. Newborn observation for 24 hours due to cord anomalies and distress. Watch for uterine atony – aggressive fundal massage. Episiotomy care. Psychological support. Follow-up: glucose at 42 days; newborn follow-up. |
| **Clinical appropriateness 临床适当性** | 5 | 4 |
| **Individualization 个体化程度** | 5 | 5 |
| **Comprehensiveness 完整性** | 5 | 3 |
| **Actionability 可操作性** | 4 | 4 |
| **Safety 安全性** | 5 | 4 |
| **Triage appropriateness 分诊/转诊建议适当性** | 5 | 3 |
| **Structure and clarity 结构与清晰度** | 4 | 3 |

**病例 63 / Case 63**

| **项目 Item** | **ChatGPT 5.5** | **DeepSeek V3** |
| --- | --- | --- |
| **去标识化临床病例摘要 De-identified Clinical Case Summary** | 患者28岁，孕产史为G2P2，既往剖宫产0次，孕周39+2，临产类别为自然临产。自然破膜，羊水量约300ml，羊水性质为清。以自然分娩分娩，产时出血约290ml。第一产程2时40分，第二产程0时25分，第三产程0时5分，总产程3时10分，会阴或切口情况为完整。合并症及主要风险因素：无。新生儿出生体重3140g，Apgar评分1、5、10分钟分别为10、10、10分，分娩结局为活产。 | |
| **临床病例摘要英文翻译 English Translation of De-identified Clinical Case Summary** | Maternal age was 28 years. The obstetric history was G2P2, with 0 previous cesarean deliveries. Gestational age was 39+2 weeks, and the labor onset or induction category was spontaneous labor. The membranes ruptured spontaneously; the amniotic fluid volume was approximately 300 mL and its character was clear. The patient had a spontaneous vaginal delivery, with estimated intrapartum blood loss of 290 mL. The first, second, and third stages of labor were 2 h 40 min, 0 h 25 min, and 0 h 5 min, respectively; the total labor duration was 3 h 10 min. The perineum was intact. No comorbidities or major risk factors were documented. Neonatal birth weight was 3140 g; Apgar scores at 1, 5, and 10 minutes were 10, 10, 10; and the delivery outcome was live birth. | |
| **模型生成的产褥期护理计划 Model-generated postpartum nursing care plan** | 产妇28岁，G2P2，孕39+2周，经阴道自然分娩分娩，产时出血约290ml，新生儿女婴、体重3140g，Apgar评分10、10、10分。病例提示的重点风险为未记录特殊合并症。产后24小时内重点评估体温、脉搏、血压、宫底高度、宫缩、阴道流血、恶露颜色和尿潴留，记录首次排尿和疼痛评分。产后住院及出院后继续观察恶露是否由红色逐渐转淡，乳房是否胀痛，乳头是否皲裂，睡眠和情绪是否稳定。饮食以优质蛋白、含铁食物、蔬菜水果和足量饮水为主，避免盲目大补和长期卧床。本次为阴道自然分娩且会阴记录相对稳定，仍需观察会阴疼痛、肿胀和排尿情况。无明显不适时可循序渐进下床活动，产后早期以轻柔盆底肌收缩训练为主，避免过度用力和长时间站立。哺乳方面建议尽早母婴皮肤接触和按需哺乳，指导正确含接，乳胀时先热敷或轻柔按摩再哺乳，乳头疼痛时及时调整姿势。若出现乳房局部红肿硬痛、寒战或高热，应警惕乳腺炎。目前未记录特殊合并症，仍需按常规产褥期管理执行，重点是子宫复旧、恶露、伤口、哺乳、营养、活动和心理状态。出院前应完成用药、伤口、哺乳和避孕宣教，42天左右复查，必要时提前复诊。若出现阴道流血突然增多、鲜红血浸湿卫生巾、恶露恶臭、发热、持续腹痛、伤口化脓、排尿困难、下肢单侧肿痛、胸闷气短，或持续悲伤焦虑及伤害自己或婴儿的想法，应立即就医或急诊处理。 | 患者28岁，G2P2，自然分娩，会阴完整，无合并症，脐带绕颈1周。产后护理：观察恶露、子宫复旧；注意会阴清洁（虽完整仍建议温水清洗）；脐带绕颈新生儿观察呼吸、喂养24小时；按需哺乳；早下床；防便秘；42天复查。常规避孕指导。 |
| **护理计划英文翻译 English translation of care plan** | The patient is 28 years old, G2P2, delivered at 39+2 weeks by spontaneous vaginal delivery, with an estimated intrapartum blood loss of 290 mL. The newborn was a female infant, weighed 3140 g, and had Apgar scores of 10, 10, 10. The main case-based risks include no special comorbidity was recorded. Within the first 24 hours, assess temperature, pulse, blood pressure, uterine fundal height, uterine tone, vaginal bleeding, lochia color, urinary retention, first voiding, and pain score. During hospitalization and after discharge, continue to observe whether lochia gradually changes from red to lighter color, whether breast engorgement or nipple fissure occurs, and whether sleep and mood remain stable. Diet should include high-quality protein, iron-rich food, vegetables, fruit, and adequate fluids. Avoid blind over-supplementation and prolonged bed rest. Because this was a spontaneous vaginal birth and the perineum was relatively stable, still observe perineal pain, swelling, and urination. If there is no marked discomfort, increase ambulation gradually. Early pelvic floor training should be gentle contractions only, with avoidance of straining and prolonged standing. For breastfeeding, encourage early skin-to-skin contact and feeding on demand. Teach correct latch and positioning. For engorgement, apply warmth or gentle massage before feeding. If nipple pain occurs, adjust latch promptly. Localized breast redness, hard painful swelling, chills, or high fever should raise concern for mastitis. No special complication was recorded, but routine postpartum management should still focus on uterine involution, lochia, wounds, breastfeeding, nutrition, activity, and mental health. Before discharge, provide education on medication, wound care, breastfeeding, and contraception. Arrange a postpartum review around 42 days after delivery, or earlier if symptoms occur. Seek urgent medical care for sudden heavy vaginal bleeding, bright red bleeding soaking pads, foul-smelling lochia, fever, persistent abdominal pain, pus from the wound, difficulty urinating, unilateral leg swelling or pain, chest tightness, shortness of breath, or persistent sadness, anxiety, or thoughts of harming herself or the baby. | The patient is a 28-year-old G2P2 with spontaneous delivery and intact perineum. No comorbidities except nuchal cord 1 loop. Routine care: lochia monitoring, perineal hygiene (warm water), newborn observation for 24 hours, breastfeeding on demand, early ambulation, prevent constipation, follow-up at 42 days, contraception counseling. |
| **Clinical appropriateness 临床适当性** | 4 | 3 |
| **Individualization 个体化程度** | 3 | 3 |
| **Comprehensiveness 完整性** | 4 | 3 |
| **Actionability 可操作性** | 4 | 3 |
| **Safety 安全性** | 5 | 3 |
| **Triage appropriateness 分诊/转诊建议适当性** | 5 | 3 |
| **Structure and clarity 结构与清晰度** | 3 | 4 |

**病例 64 / Case 64**

| **项目 Item** | **ChatGPT 5.5** | **DeepSeek V3** |
| --- | --- | --- |
| **去标识化临床病例摘要 De-identified Clinical Case Summary** | 患者33岁，孕产史为G1P1，既往剖宫产0次，孕周40+2，临产类别为自然临产。自然破膜，羊水量约300ml，羊水性质为清。以自然分娩分娩，产时出血约230ml。第一产程4时42分，第二产程0时21分，第三产程0时30分，总产程5时33分，会阴或切口情况为Ⅰ，内缝。合并症及主要风险因素：妊娠合并子宫肌瘤；瘢痕子宫。新生儿出生体重2970g，Apgar评分1、5、10分钟分别为10、10、10分，分娩结局为活产。 | |
| **临床病例摘要英文翻译 English Translation of De-identified Clinical Case Summary** | Maternal age was 33 years. The obstetric history was G1P1, with 0 previous cesarean deliveries. Gestational age was 40+2 weeks, and the labor onset or induction category was spontaneous labor. The membranes ruptured spontaneously; the amniotic fluid volume was approximately 300 mL and its character was clear. The patient had a spontaneous vaginal delivery, with estimated intrapartum blood loss of 230 mL. The first, second, and third stages of labor were 4 h 42 min, 0 h 21 min, and 0 h 30 min, respectively; the total labor duration was 5 h 33 min. A first-degree perineal laceration was repaired with internal sutures. Documented comorbidities and major risk factors were uterine fibroid during pregnancy; scarred uterus. Neonatal birth weight was 2970 g; Apgar scores at 1, 5, and 10 minutes were 10, 10, 10; and the delivery outcome was live birth. | |
| **模型生成的产褥期护理计划 Model-generated postpartum nursing care plan** | 产妇33岁，G1P1，孕40+2周，经阴道自然分娩分娩，产时出血约230ml，新生儿女婴、体重2970g，Apgar评分10、10、10分。病例提示的重点风险为未记录特殊合并症。产后24小时内重点评估体温、脉搏、血压、宫底高度、宫缩、阴道流血、恶露颜色和尿潴留，记录首次排尿和疼痛评分。产后住院及出院后继续观察恶露是否由红色逐渐转淡，乳房是否胀痛，乳头是否皲裂，睡眠和情绪是否稳定。饮食以优质蛋白、含铁食物、蔬菜水果和足量饮水为主，避免盲目大补和长期卧床。本次为阴道自然分娩，应重点观察会阴裂伤或缝合处恢复。每次大小便后保持局部清洁干燥，观察红肿、渗血、疼痛加重和异常气味。无头晕和出血增多时逐步增加活动，并在医护指导下开始轻柔盆底肌训练。哺乳方面建议尽早母婴皮肤接触和按需哺乳，指导正确含接，乳胀时先热敷或轻柔按摩再哺乳，乳头疼痛时及时调整姿势。若出现乳房局部红肿硬痛、寒战或高热，应警惕乳腺炎。合并子宫肌瘤、卵巢囊肿或多囊卵巢相关情况时，产后复查时建议结合妇科超声评估，若腹痛、包块感或异常出血加重，应提前就诊。出院前应完成用药、伤口、哺乳和避孕宣教，42天左右复查，必要时提前复诊。若出现阴道流血突然增多、鲜红血浸湿卫生巾、恶露恶臭、发热、持续腹痛、伤口化脓、排尿困难、下肢单侧肿痛、胸闷气短，或持续悲伤焦虑及伤害自己或婴儿的想法，应立即就医或急诊处理。 | 患者33岁，初产妇，自然分娩+I度裂伤，合并妊娠合并子宫肌瘤、瘢痕子宫（曾有剖宫产？但本次自然分娩），产时出血230ml。瘢痕子宫自然分娩后需警惕子宫破裂极罕见但仍注意腹痛、阴道出血异常。子宫肌瘤影响复旧，加强宫底按摩，观察恶露。I度裂伤温水坐浴。鼓励母乳喂养。产后6周B超复查肌瘤及子宫瘢痕。42天复查。 |
| **护理计划英文翻译 English translation of care plan** | The patient is 33 years old, G1P1, delivered at 40+2 weeks by spontaneous vaginal delivery, with an estimated intrapartum blood loss of 230 mL. The newborn was a female infant, weighed 2970 g, and had Apgar scores of 10, 10, 10. The main case-based risks include no special comorbidity was recorded. Within the first 24 hours, assess temperature, pulse, blood pressure, uterine fundal height, uterine tone, vaginal bleeding, lochia color, urinary retention, first voiding, and pain score. During hospitalization and after discharge, continue to observe whether lochia gradually changes from red to lighter color, whether breast engorgement or nipple fissure occurs, and whether sleep and mood remain stable. Diet should include high-quality protein, iron-rich food, vegetables, fruit, and adequate fluids. Avoid blind over-supplementation and prolonged bed rest. Because this was a spontaneous vaginal birth, recovery of any perineal tear or suture site should be observed. Keep the area clean and dry after urination or bowel movement and watch for redness, swelling, bleeding, worsening pain, or abnormal odor. If there is no dizziness or increased bleeding, increase activity gradually and begin gentle pelvic floor exercises under professional guidance. For breastfeeding, encourage early skin-to-skin contact and feeding on demand. Teach correct latch and positioning. For engorgement, apply warmth or gentle massage before feeding. If nipple pain occurs, adjust latch promptly. Localized breast redness, hard painful swelling, chills, or high fever should raise concern for mastitis. For uterine fibroids, ovarian cysts, or polycystic ovary related findings, pelvic ultrasound can be considered at postpartum review. Earlier assessment is needed for worsening abdominal pain, mass sensation, or abnormal bleeding. Before discharge, provide education on medication, wound care, breastfeeding, and contraception. Arrange a postpartum review around 42 days after delivery, or earlier if symptoms occur. Seek urgent medical care for sudden heavy vaginal bleeding, bright red bleeding soaking pads, foul-smelling lochia, fever, persistent abdominal pain, pus from the wound, difficulty urinating, unilateral leg swelling or pain, chest tightness, shortness of breath, or persistent sadness, anxiety, or thoughts of harming herself or the baby. | The patient is a 33-year-old primipara with spontaneous delivery and first-degree laceration. Comorbidities: uterine fibroid, scarred uterus. Monitor for the rare risk of uterine rupture, including abnormal abdominal pain or bleeding. Fibroid may impair involution – fundal massage, lochia monitoring. Warm sitz baths. Encourage breastfeeding. Follow-up ultrasound at 6 weeks for fibroid and uterine scar. Routine check at 42 days. |
| **Clinical appropriateness 临床适当性** | 4 | 4 |
| **Individualization 个体化程度** | 3 | 4 |
| **Comprehensiveness 完整性** | 4 | 3 |
| **Actionability 可操作性** | 4 | 4 |
| **Safety 安全性** | 5 | 4 |
| **Triage appropriateness 分诊/转诊建议适当性** | 5 | 3 |
| **Structure and clarity 结构与清晰度** | 4 | 4 |

**病例 65 / Case 65**

| **项目 Item** | **ChatGPT 5.5** | **DeepSeek V3** |
| --- | --- | --- |
| **去标识化临床病例摘要 De-identified Clinical Case Summary** | 患者33岁，孕产史为G1P1，既往剖宫产0次，孕周35+6，临产类别为自然临产。自然破膜，羊水量约300ml，羊水性质为清。以自然分娩分娩，产时出血约270ml。第一产程7时44分，第二产程0时29分，第三产程0时7分，总产程8时20分，会阴或切口情况为侧切，内缝。合并症及主要风险因素：妊娠合并血小板减少；胎儿窘迫？。新生儿出生体重2190g，Apgar评分1、5、10分钟分别为9、9、9分，分娩结局为活产。 | |
| **临床病例摘要英文翻译 English Translation of De-identified Clinical Case Summary** | Maternal age was 33 years. The obstetric history was G1P1, with 0 previous cesarean deliveries. Gestational age was 35+6 weeks, and the labor onset or induction category was spontaneous labor. The membranes ruptured spontaneously; the amniotic fluid volume was approximately 300 mL and its character was clear. The patient had a spontaneous vaginal delivery, with estimated intrapartum blood loss of 270 mL. The first, second, and third stages of labor were 7 h 44 min, 0 h 29 min, and 0 h 7 min, respectively; the total labor duration was 8 h 20 min. An episiotomy was repaired with internal sutures. Documented comorbidities and major risk factors were thrombocytopenia during pregnancy; suspected fetal distress. Neonatal birth weight was 2190 g; Apgar scores at 1, 5, and 10 minutes were 9, 9, 9; and the delivery outcome was live birth. | |
| **模型生成的产褥期护理计划 Model-generated postpartum nursing care plan** | 产妇33岁，G1P1，孕35+6周，经阴道自然分娩分娩，产时出血约270ml，新生儿女婴、体重2190g，Apgar评分9、9、9分。病例提示的重点风险为出血或贫血相关风险、胎儿窘迫或羊水异常记录。产后24小时内重点评估体温、脉搏、血压、宫底高度、宫缩、阴道流血、恶露颜色和尿潴留，记录首次排尿和疼痛评分。产后住院及出院后继续观察恶露是否由红色逐渐转淡，乳房是否胀痛，乳头是否皲裂，睡眠和情绪是否稳定。饮食以优质蛋白、含铁食物、蔬菜水果和足量饮水为主，避免盲目大补和长期卧床。本次为阴道自然分娩，应重点观察会阴裂伤或缝合处恢复。每次大小便后保持局部清洁干燥，观察红肿、渗血、疼痛加重和异常气味。无头晕和出血增多时逐步增加活动，并在医护指导下开始轻柔盆底肌训练。哺乳方面建议尽早母婴皮肤接触和按需哺乳，指导正确含接，乳胀时先热敷或轻柔按摩再哺乳，乳头疼痛时及时调整姿势。若出现乳房局部红肿硬痛、寒战或高热，应警惕乳腺炎。因存在出血量偏多、贫血或血小板异常相关风险，应复查血常规，关注头晕、心悸、乏力、面色苍白、恶露突然增多或较大血块，按医嘱补铁，必要时评估凝血和子宫复旧。因有胎儿窘迫、羊水污染或新生儿评分变化记录，母婴同室期间应配合观察新生儿吃奶、肤色、体温、呼吸和反应，发现吃奶差、发绀、嗜睡或呼吸异常应立即告知儿科医生。出院前应完成用药、伤口、哺乳和避孕宣教，42天左右复查，必要时提前复诊。若出现阴道流血突然增多、鲜红血浸湿卫生巾、恶露恶臭、发热、持续腹痛、伤口化脓、排尿困难、下肢单侧肿痛、胸闷气短，或持续悲伤焦虑及伤害自己或婴儿的想法，应立即就医或急诊处理。 | 患者33岁，初产妇，自然分娩+侧切，孕35+6周早产，合并血小板减少、可疑胎儿窘迫，脐带绕颈1周紧、胶质脐带，新生儿体重2190g（低出生体重），Apgar 9-9-9。早产儿需保暖、监测血糖、黄疸、呼吸暂停，鼓励母乳+强化剂。血小板减少需监测出血倾向（皮肤瘀斑、牙龈出血、恶露增多），复查血常规，避免肌注。脐带绕颈紧+可疑窘迫，新生儿观察神经系统。会阴侧切护理。提供心理支持。产后1周复查血常规；早产儿随访。 |
| **护理计划英文翻译 English translation of care plan** | The patient is 33 years old, G1P1, delivered at 35+6 weeks by spontaneous vaginal delivery, with an estimated intrapartum blood loss of 270 mL. The newborn was a female infant, weighed 2190 g, and had Apgar scores of 9, 9, 9. The main case-based risks include bleeding or anemia-related risk, fetal distress or abnormal amniotic fluid findings. Within the first 24 hours, assess temperature, pulse, blood pressure, uterine fundal height, uterine tone, vaginal bleeding, lochia color, urinary retention, first voiding, and pain score. During hospitalization and after discharge, continue to observe whether lochia gradually changes from red to lighter color, whether breast engorgement or nipple fissure occurs, and whether sleep and mood remain stable. Diet should include high-quality protein, iron-rich food, vegetables, fruit, and adequate fluids. Avoid blind over-supplementation and prolonged bed rest. Because this was a spontaneous vaginal birth, recovery of any perineal tear or suture site should be observed. Keep the area clean and dry after urination or bowel movement and watch for redness, swelling, bleeding, worsening pain, or abnormal odor. If there is no dizziness or increased bleeding, increase activity gradually and begin gentle pelvic floor exercises under professional guidance. For breastfeeding, encourage early skin-to-skin contact and feeding on demand. Teach correct latch and positioning. For engorgement, apply warmth or gentle massage before feeding. If nipple pain occurs, adjust latch promptly. Localized breast redness, hard painful swelling, chills, or high fever should raise concern for mastitis. Because increased blood loss, anemia, or platelet abnormality is a concern, repeat a complete blood count as advised and watch for dizziness, palpitations, severe fatigue, pallor, sudden increase in lochia, or large clots. Iron supplementation, nutrition support, and assessment of uterine involution or coagulation should follow medical orders. Because fetal distress, meconium-stained fluid, or changed Apgar scores were recorded, cooperate with pediatric observation of feeding, skin color, temperature, breathing, and responsiveness. Poor feeding, cyanosis, excessive sleepiness, or abnormal breathing should be reported immediately. Before discharge, provide education on medication, wound care, breastfeeding, and contraception. Arrange a postpartum review around 42 days after delivery, or earlier if symptoms occur. Seek urgent medical care for sudden heavy vaginal bleeding, bright red bleeding soaking pads, foul-smelling lochia, fever, persistent abdominal pain, pus from the wound, difficulty urinating, unilateral leg swelling or pain, chest tightness, shortness of breath, or persistent sadness, anxiety, or thoughts of harming herself or the baby. | The patient is a 33-year-old primipara with spontaneous delivery and episiotomy at 35+6 weeks. Comorbidities: thrombocytopenia, suspected fetal distress, tight nuchal cord 1 loop, gelatinous cord. Newborn LBW 2190 g, Apgar 9-9-9. Preterm infant care: warmth, glucose, jaundice, apnea; breastfeeding with fortifier. Thrombocytopenia – monitor bleeding, repeat CBC, avoid IM injections. Newborn neurological observation. Episiotomy care. Psychological support. Follow-up: CBC at 1 week; preterm infant follow-up. |
| **Clinical appropriateness 临床适当性** | 5 | 4 |
| **Individualization 个体化程度** | 4 | 5 |
| **Comprehensiveness 完整性** | 5 | 4 |
| **Actionability 可操作性** | 4 | 4 |
| **Safety 安全性** | 5 | 4 |
| **Triage appropriateness 分诊/转诊建议适当性** | 5 | 4 |
| **Structure and clarity 结构与清晰度** | 4 | 4 |

**病例 66 / Case 66**

| **项目 Item** | **ChatGPT 5.5** | **DeepSeek V3** |
| --- | --- | --- |
| **去标识化临床病例摘要 De-identified Clinical Case Summary** | 患者33岁，孕产史为G1P1，既往剖宫产0次，孕周40+2，临产类别为催产素引产。自然破膜，羊水量约200ml，羊水性质为清。以产钳分娩，产时出血约350ml。第一产程5时9分，第二产程0时30分，第三产程0时3分，总产程5时42分，会阴或切口情况为侧切，内缝。合并症及主要风险因素：胎儿窘迫，胎膜早破。新生儿出生体重3060g，Apgar评分1、5、10分钟分别为10、10、10分，分娩结局为活产。 | |
| **临床病例摘要英文翻译 English Translation of De-identified Clinical Case Summary** | Maternal age was 33 years. The obstetric history was G1P1, with 0 previous cesarean deliveries. Gestational age was 40+2 weeks, and the labor onset or induction category was oxytocin induction. The membranes ruptured spontaneously; the amniotic fluid volume was approximately 200 mL and its character was clear. The patient had a forceps-assisted vaginal delivery, with estimated intrapartum blood loss of 350 mL. The first, second, and third stages of labor were 5 h 9 min, 0 h 30 min, and 0 h 3 min, respectively; the total labor duration was 5 h 42 min. An episiotomy was repaired with internal sutures. Documented comorbidities and major risk factors were fetal distress, premature rupture of membranes. Neonatal birth weight was 3060 g; Apgar scores at 1, 5, and 10 minutes were 10, 10, 10; and the delivery outcome was live birth. | |
| **模型生成的产褥期护理计划 Model-generated postpartum nursing care plan** | 产妇33岁，G1P1，孕40+2周，经产钳助产阴道分娩分娩，产时出血约350ml，新生儿女婴、体重3060g，Apgar评分10、10、10分。病例提示的重点风险为胎膜早破或感染相关风险、胎儿窘迫或羊水异常记录。产后24小时内重点评估体温、脉搏、血压、宫底高度、宫缩、阴道流血、恶露颜色和尿潴留，记录首次排尿和疼痛评分。产后住院及出院后继续观察恶露是否由红色逐渐转淡，乳房是否胀痛，乳头是否皲裂，睡眠和情绪是否稳定。饮食以优质蛋白、含铁食物、蔬菜水果和足量饮水为主，避免盲目大补和长期卧床。本次为产钳助产阴道分娩，会阴伤口、盆底和排尿功能是护理重点。每次大小便后用温水从前向后清洁会阴并轻轻擦干，观察侧切或裂伤处肿胀、硬结、渗血、分泌物和异味。疼痛明显时可侧卧休息，避免久蹲、负重和过早剧烈活动。哺乳方面建议尽早母婴皮肤接触和按需哺乳，指导正确含接，乳胀时先热敷或轻柔按摩再哺乳，乳头疼痛时及时调整姿势。若出现乳房局部红肿硬痛、寒战或高热，应警惕乳腺炎。存在胎膜早破、B族链球菌感染、阴道炎或宫内感染疑虑时，应加强体温、下腹压痛、恶露气味、会阴或切口渗液观察，发热或恶露恶臭时及时复诊。因有胎儿窘迫、羊水污染或新生儿评分变化记录，母婴同室期间应配合观察新生儿吃奶、肤色、体温、呼吸和反应，发现吃奶差、发绀、嗜睡或呼吸异常应立即告知儿科医生。出院前应完成用药、伤口、哺乳和避孕宣教，42天左右复查，必要时提前复诊。若出现阴道流血突然增多、鲜红血浸湿卫生巾、恶露恶臭、发热、持续腹痛、伤口化脓、排尿困难、下肢单侧肿痛、胸闷气短，或持续悲伤焦虑及伤害自己或婴儿的想法，应立即就医或急诊处理。 | 患者33岁，初产妇，产钳助产+侧切，合并胎儿窘迫、胎膜早破，脐带绕颈2周（紧），产时出血350ml。新生儿需密切监测缺氧缺血性脑病，因脐带绕颈紧+窘迫，建议儿科观察24-48小时。PPROM监测感染。出血350ml，注意晚期出血。会阴侧切每日消毒。鼓励母乳喂养。提供心理支持。42天复查；新生儿专科随访。 |
| **护理计划英文翻译 English translation of care plan** | The patient is 33 years old, G1P1, delivered at 40+2 weeks by forceps-assisted vaginal delivery, with an estimated intrapartum blood loss of 350 mL. The newborn was a female infant, weighed 3060 g, and had Apgar scores of 10, 10, 10. The main case-based risks include premature rupture of membranes or infection-related risk, fetal distress or abnormal amniotic fluid findings. Within the first 24 hours, assess temperature, pulse, blood pressure, uterine fundal height, uterine tone, vaginal bleeding, lochia color, urinary retention, first voiding, and pain score. During hospitalization and after discharge, continue to observe whether lochia gradually changes from red to lighter color, whether breast engorgement or nipple fissure occurs, and whether sleep and mood remain stable. Diet should include high-quality protein, iron-rich food, vegetables, fruit, and adequate fluids. Avoid blind over-supplementation and prolonged bed rest. Because this was a forceps-assisted vaginal delivery, perineal wound care, pelvic floor recovery, and urinary function are priorities. Rinse the perineum from front to back with warm water after urination or bowel movement and pat dry. Observe the episiotomy or tear for swelling, induration, bleeding, discharge, and odor. Rest in a lateral position if pain is marked, and avoid prolonged squatting, heavy lifting, and early vigorous activity. For breastfeeding, encourage early skin-to-skin contact and feeding on demand. Teach correct latch and positioning. For engorgement, apply warmth or gentle massage before feeding. If nipple pain occurs, adjust latch promptly. Localized breast redness, hard painful swelling, chills, or high fever should raise concern for mastitis. With premature rupture of membranes, group B streptococcus, vaginitis, or suspected intrauterine infection, monitor temperature, lower abdominal tenderness, lochia odor, and drainage from the perineal wound or incision. Return promptly for fever or foul-smelling lochia. Because fetal distress, meconium-stained fluid, or changed Apgar scores were recorded, cooperate with pediatric observation of feeding, skin color, temperature, breathing, and responsiveness. Poor feeding, cyanosis, excessive sleepiness, or abnormal breathing should be reported immediately. Before discharge, provide education on medication, wound care, breastfeeding, and contraception. Arrange a postpartum review around 42 days after delivery, or earlier if symptoms occur. Seek urgent medical care for sudden heavy vaginal bleeding, bright red bleeding soaking pads, foul-smelling lochia, fever, persistent abdominal pain, pus from the wound, difficulty urinating, unilateral leg swelling or pain, chest tightness, shortness of breath, or persistent sadness, anxiety, or thoughts of harming herself or the baby. | The patient is a 33-year-old primipara with forceps delivery and episiotomy. Comorbidities: fetal distress, PPROM, tight nuchal cord 2 loops. Blood loss 350 mL. Newborn – monitor for HIE for 24-48 hours. Monitor infection. Watch for late PPH. Episiotomy care. Encourage breastfeeding. Psychological support. Follow-up at 42 days; newborn specialist follow-up. |
| **Clinical appropriateness 临床适当性** | 5 | 4 |
| **Individualization 个体化程度** | 4 | 5 |
| **Comprehensiveness 完整性** | 5 | 3 |
| **Actionability 可操作性** | 4 | 4 |
| **Safety 安全性** | 5 | 4 |
| **Triage appropriateness 分诊/转诊建议适当性** | 5 | 3 |
| **Structure and clarity 结构与清晰度** | 4 | 4 |

**病例 67 / Case 67**

| **项目 Item** | **ChatGPT 5.5** | **DeepSeek V3** |
| --- | --- | --- |
| **去标识化临床病例摘要 De-identified Clinical Case Summary** | 患者43岁，孕产史为G2P2，既往剖宫产0次，孕周39+4，临产类别为普贝生。自然破膜，羊水量约300ml，羊水性质为清。以自然分娩分娩，产时出血约210ml。第一产程3时1分，第二产程0时7分，第三产程0时7分，总产程3时15分，会阴或切口情况为Ⅰ，内缝。合并症及主要风险因素：妊娠期糖尿病；妊娠合并子宫肌瘤；双眼屈光不正；高龄经产妇妊娠监督；臀位外倒转术后。新生儿出生体重2820g，Apgar评分1、5、10分钟分别为10、10、10分，分娩结局为活产。 | |
| **临床病例摘要英文翻译 English Translation of De-identified Clinical Case Summary** | Maternal age was 43 years. The obstetric history was G2P2, with 0 previous cesarean deliveries. Gestational age was 39+4 weeks, and the labor onset or induction category was vaginal dinoprostone (Propess) induction. The membranes ruptured spontaneously; the amniotic fluid volume was approximately 300 mL and its character was clear. The patient had a spontaneous vaginal delivery, with estimated intrapartum blood loss of 210 mL. The first, second, and third stages of labor were 3 h 1 min, 0 h 7 min, and 0 h 7 min, respectively; the total labor duration was 3 h 15 min. A first-degree perineal laceration was repaired with internal sutures. Documented comorbidities and major risk factors were gestational diabetes mellitus; uterine fibroid during pregnancy; bilateral refractive error; advanced maternal age in a multipara; status post external cephalic version for breech presentation. Neonatal birth weight was 2820 g; Apgar scores at 1, 5, and 10 minutes were 10, 10, 10; and the delivery outcome was live birth. | |
| **模型生成的产褥期护理计划 Model-generated postpartum nursing care plan** | 产妇43岁，G2P2，孕39+4周，经阴道自然分娩分娩，产时出血约210ml，新生儿女婴、体重2820g，Apgar评分10、10、10分。病例提示的重点风险为妊娠期糖尿病、高龄妊娠因素。产后24小时内重点评估体温、脉搏、血压、宫底高度、宫缩、阴道流血、恶露颜色和尿潴留，记录首次排尿和疼痛评分。产后住院及出院后继续观察恶露是否由红色逐渐转淡，乳房是否胀痛，乳头是否皲裂，睡眠和情绪是否稳定。饮食以优质蛋白、含铁食物、蔬菜水果和足量饮水为主，避免盲目大补和长期卧床。本次为阴道自然分娩，应重点观察会阴裂伤或缝合处恢复。每次大小便后保持局部清洁干燥，观察红肿、渗血、疼痛加重和异常气味。无头晕和出血增多时逐步增加活动，并在医护指导下开始轻柔盆底肌训练。哺乳方面建议尽早母婴皮肤接触和按需哺乳，指导正确含接，乳胀时先热敷或轻柔按摩再哺乳，乳头疼痛时及时调整姿势。若出现乳房局部红肿硬痛、寒战或高热，应警惕乳腺炎。有妊娠期糖尿病者，产后饮食应控制精制糖和高油食物，少量多餐，结合恢复情况逐步活动，并按医嘱在产后复查血糖或糖耐量。合并子宫肌瘤、卵巢囊肿或多囊卵巢相关情况时，产后复查时建议结合妇科超声评估，若腹痛、包块感或异常出血加重，应提前就诊。高龄产妇恢复期应避免过度劳累和连续熬夜，家属应参与夜间照护，保证睡眠、营养和情绪支持。出院前应完成用药、伤口、哺乳和避孕宣教，42天左右复查，必要时提前复诊。若出现阴道流血突然增多、鲜红血浸湿卫生巾、恶露恶臭、发热、持续腹痛、伤口化脓、排尿困难、下肢单侧肿痛、胸闷气短，或持续悲伤焦虑及伤害自己或婴儿的想法，应立即就医或急诊处理。 | 患者43岁，G2P2，自然分娩+I度裂伤，合并GDM、子宫肌瘤、双眼屈光不正、高龄经产妇、臀位外倒转术后，产时出血210ml。GDM产后监测血糖。子宫肌瘤影响复旧，加强宫底按摩。高龄经产妇注意血压、血糖，预防血栓。外倒转术后无特殊。I度裂伤温水坐浴。鼓励母乳喂养。42天复查血糖、B超肌瘤。 |
| **护理计划英文翻译 English translation of care plan** | The patient is 43 years old, G2P2, delivered at 39+4 weeks by spontaneous vaginal delivery, with an estimated intrapartum blood loss of 210 mL. The newborn was a female infant, weighed 2820 g, and had Apgar scores of 10, 10, 10. The main case-based risks include gestational diabetes mellitus, advanced maternal age. Within the first 24 hours, assess temperature, pulse, blood pressure, uterine fundal height, uterine tone, vaginal bleeding, lochia color, urinary retention, first voiding, and pain score. During hospitalization and after discharge, continue to observe whether lochia gradually changes from red to lighter color, whether breast engorgement or nipple fissure occurs, and whether sleep and mood remain stable. Diet should include high-quality protein, iron-rich food, vegetables, fruit, and adequate fluids. Avoid blind over-supplementation and prolonged bed rest. Because this was a spontaneous vaginal birth, recovery of any perineal tear or suture site should be observed. Keep the area clean and dry after urination or bowel movement and watch for redness, swelling, bleeding, worsening pain, or abnormal odor. If there is no dizziness or increased bleeding, increase activity gradually and begin gentle pelvic floor exercises under professional guidance. For breastfeeding, encourage early skin-to-skin contact and feeding on demand. Teach correct latch and positioning. For engorgement, apply warmth or gentle massage before feeding. If nipple pain occurs, adjust latch promptly. Localized breast redness, hard painful swelling, chills, or high fever should raise concern for mastitis. For gestational diabetes, avoid refined sugar and heavy oily foods, eat small balanced meals, increase activity gradually, and arrange postpartum glucose or oral glucose tolerance testing as advised. For uterine fibroids, ovarian cysts, or polycystic ovary related findings, pelvic ultrasound can be considered at postpartum review. Earlier assessment is needed for worsening abdominal pain, mass sensation, or abnormal bleeding. Advanced maternal age may slow recovery, so the family should help with nighttime care and support adequate sleep, nutrition, and emotional stability. Before discharge, provide education on medication, wound care, breastfeeding, and contraception. Arrange a postpartum review around 42 days after delivery, or earlier if symptoms occur. Seek urgent medical care for sudden heavy vaginal bleeding, bright red bleeding soaking pads, foul-smelling lochia, fever, persistent abdominal pain, pus from the wound, difficulty urinating, unilateral leg swelling or pain, chest tightness, shortness of breath, or persistent sadness, anxiety, or thoughts of harming herself or the baby. | The patient is a 43-year-old G2P2 with spontaneous delivery and first-degree laceration. Comorbidities: GDM, uterine fibroid, refractive error, advanced age multipara, status post external cephalic version. Blood loss 210 mL. Monitor postpartum glucose. Fibroid – fundal massage. Advanced age – monitor BP, glucose, prevent thrombosis. Warm sitz baths. Encourage breastfeeding. Follow-up: glucose and fibroid ultrasound at 42 days. |
| **Clinical appropriateness 临床适当性** | 5 | 4 |
| **Individualization 个体化程度** | 4 | 5 |
| **Comprehensiveness 完整性** | 5 | 3 |
| **Actionability 可操作性** | 4 | 3 |
| **Safety 安全性** | 5 | 4 |
| **Triage appropriateness 分诊/转诊建议适当性** | 5 | 3 |
| **Structure and clarity 结构与清晰度** | 4 | 4 |

**病例 68 / Case 68**

| **项目 Item** | **ChatGPT 5.5** | **DeepSeek V3** |
| --- | --- | --- |
| **去标识化临床病例摘要 De-identified Clinical Case Summary** | 患者32岁，孕产史为G2P2，既往剖宫产0次，孕周38+2，临产类别为自然临产。自然破膜，羊水量约300ml，羊水性质为清。以自然分娩分娩，产时出血约250ml。第一产程3时13分，第二产程0时10分，第三产程0时7分，总产程3时30分，会阴或切口情况为Ⅰ，内缝。合并症及主要风险因素：胎膜早破；妊娠期高血压疾病 子痫前期；妊娠期糖尿病；胎儿窘迫？。新生儿出生体重3060g，Apgar评分1、5、10分钟分别为10、10、10分，分娩结局为活产。 | |
| **临床病例摘要英文翻译 English Translation of De-identified Clinical Case Summary** | Maternal age was 32 years. The obstetric history was G2P2, with 0 previous cesarean deliveries. Gestational age was 38+2 weeks, and the labor onset or induction category was spontaneous labor. The membranes ruptured spontaneously; the amniotic fluid volume was approximately 300 mL and its character was clear. The patient had a spontaneous vaginal delivery, with estimated intrapartum blood loss of 250 mL. The first, second, and third stages of labor were 3 h 13 min, 0 h 10 min, and 0 h 7 min, respectively; the total labor duration was 3 h 30 min. A first-degree perineal laceration was repaired with internal sutures. Documented comorbidities and major risk factors were premature rupture of membranes; gestational hypertensive disorder with preeclampsia; gestational diabetes mellitus; suspected fetal distress. Neonatal birth weight was 3060 g; Apgar scores at 1, 5, and 10 minutes were 10, 10, 10; and the delivery outcome was live birth. | |
| **模型生成的产褥期护理计划 Model-generated postpartum nursing care plan** | 产妇32岁，G2P2，孕38+2周，经阴道自然分娩分娩，产时出血约250ml，新生儿女婴、体重3060g，Apgar评分10、10、10分。病例提示的重点风险为妊娠期高血压或子痫前期相关风险、妊娠期糖尿病、胎膜早破或感染相关风险、胎儿窘迫或羊水异常记录。产后24小时内重点评估体温、脉搏、血压、宫底高度、宫缩、阴道流血、恶露颜色和尿潴留，记录首次排尿和疼痛评分。产后住院及出院后继续观察恶露是否由红色逐渐转淡，乳房是否胀痛，乳头是否皲裂，睡眠和情绪是否稳定。饮食以优质蛋白、含铁食物、蔬菜水果和足量饮水为主，避免盲目大补和长期卧床。本次为阴道自然分娩，应重点观察会阴裂伤或缝合处恢复。每次大小便后保持局部清洁干燥，观察红肿、渗血、疼痛加重和异常气味。无头晕和出血增多时逐步增加活动，并在医护指导下开始轻柔盆底肌训练。哺乳方面建议尽早母婴皮肤接触和按需哺乳，指导正确含接，乳胀时先热敷或轻柔按摩再哺乳，乳头疼痛时及时调整姿势。若出现乳房局部红肿硬痛、寒战或高热，应警惕乳腺炎。有高血压或子痫前期风险者，产后仍可能出现血压波动，应按医嘱监测血压，若持续头痛、眼花、胸闷、上腹痛、抽搐或水肿加重，应立即就医。有妊娠期糖尿病者，产后饮食应控制精制糖和高油食物，少量多餐，结合恢复情况逐步活动，并按医嘱在产后复查血糖或糖耐量。存在胎膜早破、B族链球菌感染、阴道炎或宫内感染疑虑时，应加强体温、下腹压痛、恶露气味、会阴或切口渗液观察，发热或恶露恶臭时及时复诊。因有胎儿窘迫、羊水污染或新生儿评分变化记录，母婴同室期间应配合观察新生儿吃奶、肤色、体温、呼吸和反应，发现吃奶差、发绀、嗜睡或呼吸异常应立即告知儿科医生。出院前应完成用药、伤口、哺乳和避孕宣教，42天左右复查，必要时提前复诊。若出现阴道流血突然增多、鲜红血浸湿卫生巾、恶露恶臭、发热、持续腹痛、伤口化脓、排尿困难、下肢单侧肿痛、胸闷气短，或持续悲伤焦虑及伤害自己或婴儿的想法，应立即就医或急诊处理。 | 患者32岁，G2P2，自然分娩+I度裂伤，合并胎膜早破、子痫前期、GDM、可疑胎儿窘迫，脐带绕颈1周。子痫前期产后需监测血压，继续降压药，警惕产后子痫。GDM监测血糖。PPROM监测感染。新生儿观察脐带绕颈及窘迫后遗症。I度裂伤温水坐浴。提供心理支持。42天复查血压、血糖。 |
| **护理计划英文翻译 English translation of care plan** | The patient is 32 years old, G2P2, delivered at 38+2 weeks by spontaneous vaginal delivery, with an estimated intrapartum blood loss of 250 mL. The newborn was a female infant, weighed 3060 g, and had Apgar scores of 10, 10, 10. The main case-based risks include gestational hypertension or preeclampsia-related risk, gestational diabetes mellitus, premature rupture of membranes or infection-related risk, fetal distress or abnormal amniotic fluid findings. Within the first 24 hours, assess temperature, pulse, blood pressure, uterine fundal height, uterine tone, vaginal bleeding, lochia color, urinary retention, first voiding, and pain score. During hospitalization and after discharge, continue to observe whether lochia gradually changes from red to lighter color, whether breast engorgement or nipple fissure occurs, and whether sleep and mood remain stable. Diet should include high-quality protein, iron-rich food, vegetables, fruit, and adequate fluids. Avoid blind over-supplementation and prolonged bed rest. Because this was a spontaneous vaginal birth, recovery of any perineal tear or suture site should be observed. Keep the area clean and dry after urination or bowel movement and watch for redness, swelling, bleeding, worsening pain, or abnormal odor. If there is no dizziness or increased bleeding, increase activity gradually and begin gentle pelvic floor exercises under professional guidance. For breastfeeding, encourage early skin-to-skin contact and feeding on demand. Teach correct latch and positioning. For engorgement, apply warmth or gentle massage before feeding. If nipple pain occurs, adjust latch promptly. Localized breast redness, hard painful swelling, chills, or high fever should raise concern for mastitis. For hypertension or preeclampsia risk, postpartum blood pressure may still fluctuate. Continue blood pressure monitoring as advised and seek urgent care for persistent headache, visual symptoms, chest tightness, upper abdominal pain, seizure, or worsening edema. For gestational diabetes, avoid refined sugar and heavy oily foods, eat small balanced meals, increase activity gradually, and arrange postpartum glucose or oral glucose tolerance testing as advised. With premature rupture of membranes, group B streptococcus, vaginitis, or suspected intrauterine infection, monitor temperature, lower abdominal tenderness, lochia odor, and drainage from the perineal wound or incision. Return promptly for fever or foul-smelling lochia. Because fetal distress, meconium-stained fluid, or changed Apgar scores were recorded, cooperate with pediatric observation of feeding, skin color, temperature, breathing, and responsiveness. Poor feeding, cyanosis, excessive sleepiness, or abnormal breathing should be reported immediately. Before discharge, provide education on medication, wound care, breastfeeding, and contraception. Arrange a postpartum review around 42 days after delivery, or earlier if symptoms occur. Seek urgent medical care for sudden heavy vaginal bleeding, bright red bleeding soaking pads, foul-smelling lochia, fever, persistent abdominal pain, pus from the wound, difficulty urinating, unilateral leg swelling or pain, chest tightness, shortness of breath, or persistent sadness, anxiety, or thoughts of harming herself or the baby. | The patient is a 32-year-old G2P2 with spontaneous delivery and first-degree laceration. Comorbidities: PPROM, preeclampsia, GDM, suspected fetal distress, nuchal cord 1 loop. Postpartum preeclampsia – monitor BP, continue antihypertensives. Monitor glucose. Monitor infection. Newborn observation. Warm sitz baths. Psychological support. Follow-up: BP and glucose at 42 days. |
| **Clinical appropriateness 临床适当性** | 5 | 4 |
| **Individualization 个体化程度** | 5 | 5 |
| **Comprehensiveness 完整性** | 5 | 3 |
| **Actionability 可操作性** | 5 | 4 |
| **Safety 安全性** | 5 | 4 |
| **Triage appropriateness 分诊/转诊建议适当性** | 5 | 3 |
| **Structure and clarity 结构与清晰度** | 4 | 3 |

**病例 69 / Case 69**

| **项目 Item** | **ChatGPT 5.5** | **DeepSeek V3** |
| --- | --- | --- |
| **去标识化临床病例摘要 De-identified Clinical Case Summary** | 患者31岁，孕产史为G1P1，既往剖宫产0次，孕周41，临产类别为普贝生。自然破膜，羊水量约400ml，羊水性质为清。以自然分娩分娩，产时出血约200ml。第一产程2时30分，第二产程0时35分，第三产程0时5分，总产程3时10分，会阴或切口情况为Ⅰ，内缝。合并症及主要风险因素：无。新生儿出生体重3640g，Apgar评分1、5、10分钟分别为10、10、10分，分娩结局为活产。 | |
| **临床病例摘要英文翻译 English Translation of De-identified Clinical Case Summary** | Maternal age was 31 years. The obstetric history was G1P1, with 0 previous cesarean deliveries. Gestational age was 41 weeks, and the labor onset or induction category was vaginal dinoprostone (Propess) induction. The membranes ruptured spontaneously; the amniotic fluid volume was approximately 400 mL and its character was clear. The patient had a spontaneous vaginal delivery, with estimated intrapartum blood loss of 200 mL. The first, second, and third stages of labor were 2 h 30 min, 0 h 35 min, and 0 h 5 min, respectively; the total labor duration was 3 h 10 min. A first-degree perineal laceration was repaired with internal sutures. No comorbidities or major risk factors were documented. Neonatal birth weight was 3640 g; Apgar scores at 1, 5, and 10 minutes were 10, 10, 10; and the delivery outcome was live birth. | |
| **模型生成的产褥期护理计划 Model-generated postpartum nursing care plan** | 产妇31岁，G1P1，孕41周，经阴道自然分娩分娩，产时出血约200ml，新生儿女婴、体重3640g，Apgar评分10、10、10分。病例提示的重点风险为未记录特殊合并症。产后24小时内重点评估体温、脉搏、血压、宫底高度、宫缩、阴道流血、恶露颜色和尿潴留，记录首次排尿和疼痛评分。产后住院及出院后继续观察恶露是否由红色逐渐转淡，乳房是否胀痛，乳头是否皲裂，睡眠和情绪是否稳定。饮食以优质蛋白、含铁食物、蔬菜水果和足量饮水为主，避免盲目大补和长期卧床。本次为阴道自然分娩，应重点观察会阴裂伤或缝合处恢复。每次大小便后保持局部清洁干燥，观察红肿、渗血、疼痛加重和异常气味。无头晕和出血增多时逐步增加活动，并在医护指导下开始轻柔盆底肌训练。哺乳方面建议尽早母婴皮肤接触和按需哺乳，指导正确含接，乳胀时先热敷或轻柔按摩再哺乳，乳头疼痛时及时调整姿势。若出现乳房局部红肿硬痛、寒战或高热，应警惕乳腺炎。目前未记录特殊合并症，仍需按常规产褥期管理执行，重点是子宫复旧、恶露、伤口、哺乳、营养、活动和心理状态。出院前应完成用药、伤口、哺乳和避孕宣教，42天左右复查，必要时提前复诊。若出现阴道流血突然增多、鲜红血浸湿卫生巾、恶露恶臭、发热、持续腹痛、伤口化脓、排尿困难、下肢单侧肿痛、胸闷气短，或持续悲伤焦虑及伤害自己或婴儿的想法，应立即就医或急诊处理。 | 患者31岁，初产妇，自然分娩+I度裂伤，无合并症，脐带绕颈1周。常规护理+新生儿观察脐带绕颈24小时。42天复查。 |
| **护理计划英文翻译 English translation of care plan** | The patient is 31 years old, G1P1, delivered at 41 weeks by spontaneous vaginal delivery, with an estimated intrapartum blood loss of 200 mL. The newborn was a female infant, weighed 3640 g, and had Apgar scores of 10, 10, 10. The main case-based risks include no special comorbidity was recorded. Within the first 24 hours, assess temperature, pulse, blood pressure, uterine fundal height, uterine tone, vaginal bleeding, lochia color, urinary retention, first voiding, and pain score. During hospitalization and after discharge, continue to observe whether lochia gradually changes from red to lighter color, whether breast engorgement or nipple fissure occurs, and whether sleep and mood remain stable. Diet should include high-quality protein, iron-rich food, vegetables, fruit, and adequate fluids. Avoid blind over-supplementation and prolonged bed rest. Because this was a spontaneous vaginal birth, recovery of any perineal tear or suture site should be observed. Keep the area clean and dry after urination or bowel movement and watch for redness, swelling, bleeding, worsening pain, or abnormal odor. If there is no dizziness or increased bleeding, increase activity gradually and begin gentle pelvic floor exercises under professional guidance. For breastfeeding, encourage early skin-to-skin contact and feeding on demand. Teach correct latch and positioning. For engorgement, apply warmth or gentle massage before feeding. If nipple pain occurs, adjust latch promptly. Localized breast redness, hard painful swelling, chills, or high fever should raise concern for mastitis. No special complication was recorded, but routine postpartum management should still focus on uterine involution, lochia, wounds, breastfeeding, nutrition, activity, and mental health. Before discharge, provide education on medication, wound care, breastfeeding, and contraception. Arrange a postpartum review around 42 days after delivery, or earlier if symptoms occur. Seek urgent medical care for sudden heavy vaginal bleeding, bright red bleeding soaking pads, foul-smelling lochia, fever, persistent abdominal pain, pus from the wound, difficulty urinating, unilateral leg swelling or pain, chest tightness, shortness of breath, or persistent sadness, anxiety, or thoughts of harming herself or the baby. | The patient is a 31-year-old primipara with spontaneous delivery and first-degree laceration. No comorbidities except nuchal cord 1 loop. Routine care plus newborn observation for 24 hours. Follow-up at 42 days. |
| **Clinical appropriateness 临床适当性** | 4 | 3 |
| **Individualization 个体化程度** | 3 | 3 |
| **Comprehensiveness 完整性** | 4 | 3 |
| **Actionability 可操作性** | 4 | 3 |
| **Safety 安全性** | 5 | 3 |
| **Triage appropriateness 分诊/转诊建议适当性** | 5 | 3 |
| **Structure and clarity 结构与清晰度** | 3 | 4 |

**病例 70 / Case 70**

| **项目 Item** | **ChatGPT 5.5** | **DeepSeek V3** |
| --- | --- | --- |
| **去标识化临床病例摘要 De-identified Clinical Case Summary** | 患者27岁，孕产史为G1P1，既往剖宫产0次，孕周40+3，临产类别为自然临产。自然破膜，羊水量约300ml，羊水性质为清。以自然分娩分娩，产时出血约190ml。第一产程5时45分，第二产程1时30分，第三产程0时5分，总产程7时20分，会阴或切口情况为Ⅰ，内缝。合并症及主要风险因素：双眼屈光不正、妊娠期贫血。新生儿出生体重3420g，Apgar评分1、5、10分钟分别为10、10、10分，分娩结局为活产。 | |
| **临床病例摘要英文翻译 English Translation of De-identified Clinical Case Summary** | Maternal age was 27 years. The obstetric history was G1P1, with 0 previous cesarean deliveries. Gestational age was 40+3 weeks, and the labor onset or induction category was spontaneous labor. The membranes ruptured spontaneously; the amniotic fluid volume was approximately 300 mL and its character was clear. The patient had a spontaneous vaginal delivery, with estimated intrapartum blood loss of 190 mL. The first, second, and third stages of labor were 5 h 45 min, 1 h 30 min, and 0 h 5 min, respectively; the total labor duration was 7 h 20 min. A first-degree perineal laceration was repaired with internal sutures. Documented comorbidities and major risk factors were bilateral refractive error; anemia during pregnancy. Neonatal birth weight was 3420 g; Apgar scores at 1, 5, and 10 minutes were 10, 10, 10; and the delivery outcome was live birth. | |
| **模型生成的产褥期护理计划 Model-generated postpartum nursing care plan** | 产妇27岁，G1P1，孕40+3周，经阴道自然分娩分娩，产时出血约190ml，新生儿女婴、体重3420g，Apgar评分10、10、10分。病例提示的重点风险为出血或贫血相关风险。产后24小时内重点评估体温、脉搏、血压、宫底高度、宫缩、阴道流血、恶露颜色和尿潴留，记录首次排尿和疼痛评分。产后住院及出院后继续观察恶露是否由红色逐渐转淡，乳房是否胀痛，乳头是否皲裂，睡眠和情绪是否稳定。饮食以优质蛋白、含铁食物、蔬菜水果和足量饮水为主，避免盲目大补和长期卧床。本次为阴道自然分娩，应重点观察会阴裂伤或缝合处恢复。每次大小便后保持局部清洁干燥，观察红肿、渗血、疼痛加重和异常气味。无头晕和出血增多时逐步增加活动，并在医护指导下开始轻柔盆底肌训练。哺乳方面建议尽早母婴皮肤接触和按需哺乳，指导正确含接，乳胀时先热敷或轻柔按摩再哺乳，乳头疼痛时及时调整姿势。若出现乳房局部红肿硬痛、寒战或高热，应警惕乳腺炎。因存在出血量偏多、贫血或血小板异常相关风险，应复查血常规，关注头晕、心悸、乏力、面色苍白、恶露突然增多或较大血块，按医嘱补铁，必要时评估凝血和子宫复旧。出院前应完成用药、伤口、哺乳和避孕宣教，42天左右复查，必要时提前复诊。若出现阴道流血突然增多、鲜红血浸湿卫生巾、恶露恶臭、发热、持续腹痛、伤口化脓、排尿困难、下肢单侧肿痛、胸闷气短，或持续悲伤焦虑及伤害自己或婴儿的想法，应立即就医或急诊处理。 | 患者27岁，初产妇，自然分娩+I度裂伤，合并双眼屈光不正、妊娠期贫血，脐带长度34cm（偏短），产时出血190ml。贫血补充铁剂、维生素C，复查血常规。高度近视避免用力屏气。脐带偏短已分娩，无特殊。常规会阴护理。鼓励母乳喂养。42天复查血常规、眼科。 |
| **护理计划英文翻译 English translation of care plan** | The patient is 27 years old, G1P1, delivered at 40+3 weeks by spontaneous vaginal delivery, with an estimated intrapartum blood loss of 190 mL. The newborn was a female infant, weighed 3420 g, and had Apgar scores of 10, 10, 10. The main case-based risks include bleeding or anemia-related risk. Within the first 24 hours, assess temperature, pulse, blood pressure, uterine fundal height, uterine tone, vaginal bleeding, lochia color, urinary retention, first voiding, and pain score. During hospitalization and after discharge, continue to observe whether lochia gradually changes from red to lighter color, whether breast engorgement or nipple fissure occurs, and whether sleep and mood remain stable. Diet should include high-quality protein, iron-rich food, vegetables, fruit, and adequate fluids. Avoid blind over-supplementation and prolonged bed rest. Because this was a spontaneous vaginal birth, recovery of any perineal tear or suture site should be observed. Keep the area clean and dry after urination or bowel movement and watch for redness, swelling, bleeding, worsening pain, or abnormal odor. If there is no dizziness or increased bleeding, increase activity gradually and begin gentle pelvic floor exercises under professional guidance. For breastfeeding, encourage early skin-to-skin contact and feeding on demand. Teach correct latch and positioning. For engorgement, apply warmth or gentle massage before feeding. If nipple pain occurs, adjust latch promptly. Localized breast redness, hard painful swelling, chills, or high fever should raise concern for mastitis. Because increased blood loss, anemia, or platelet abnormality is a concern, repeat a complete blood count as advised and watch for dizziness, palpitations, severe fatigue, pallor, sudden increase in lochia, or large clots. Iron supplementation, nutrition support, and assessment of uterine involution or coagulation should follow medical orders. Before discharge, provide education on medication, wound care, breastfeeding, and contraception. Arrange a postpartum review around 42 days after delivery, or earlier if symptoms occur. Seek urgent medical care for sudden heavy vaginal bleeding, bright red bleeding soaking pads, foul-smelling lochia, fever, persistent abdominal pain, pus from the wound, difficulty urinating, unilateral leg swelling or pain, chest tightness, shortness of breath, or persistent sadness, anxiety, or thoughts of harming herself or the baby. | The patient is a 27-year-old primipara with spontaneous delivery and first-degree laceration. Comorbidities: refractive error, anemia during pregnancy, short umbilical cord (34cm). Blood loss 190 mL. Iron and vitamin C supplementation, repeat CBC. High myopia – avoid Valsalva. Short cord already delivered – no special action. Routine perineal care. Encourage breastfeeding. Follow-up: CBC and eye exam at 42 days. |
| **Clinical appropriateness 临床适当性** | 4 | 3 |
| **Individualization 个体化程度** | 3 | 3 |
| **Comprehensiveness 完整性** | 4 | 3 |
| **Actionability 可操作性** | 4 | 3 |
| **Safety 安全性** | 5 | 3 |
| **Triage appropriateness 分诊/转诊建议适当性** | 5 | 3 |
| **Structure and clarity 结构与清晰度** | 3 | 4 |

**病例 71 / Case 71**

| **项目 Item** | **ChatGPT 5.5** | **DeepSeek V3** |
| --- | --- | --- |
| **去标识化临床病例摘要 De-identified Clinical Case Summary** | 患者33岁，孕产史为G2P1，既往剖宫产0次，孕周41+2，临产类别为自然临产。自然破膜，羊水量约300ml，羊水性质为Ⅲ°。以产钳分娩，产时出血约400ml。第一产程7时30分，第二产程0时28分，第三产程0时7分，总产程8时5分，会阴或切口情况为Ⅳ，内缝。合并症及主要风险因素：B族链球菌感染；双眼高度近视；胎儿窘迫；。新生儿出生体重3700g，Apgar评分1、5、10分钟分别为10、10、10分，分娩结局为活产。 | |
| **临床病例摘要英文翻译 English Translation of De-identified Clinical Case Summary** | Maternal age was 33 years. The obstetric history was G2P1, with 0 previous cesarean deliveries. Gestational age was 41+2 weeks, and the labor onset or induction category was spontaneous labor. The membranes ruptured spontaneously; the amniotic fluid volume was approximately 300 mL and its character was grade III meconium-stained. The patient had a forceps-assisted vaginal delivery, with estimated intrapartum blood loss of 400 mL. The first, second, and third stages of labor were 7 h 30 min, 0 h 28 min, and 0 h 7 min, respectively; the total labor duration was 8 h 5 min. A fourth-degree perineal laceration was repaired with internal sutures. Documented comorbidities and major risk factors were group B Streptococcus infection; bilateral high myopia; fetal distress. Neonatal birth weight was 3700 g; Apgar scores at 1, 5, and 10 minutes were 10, 10, 10; and the delivery outcome was live birth. | |
| **模型生成的产褥期护理计划 Model-generated postpartum nursing care plan** | 产妇33岁，G2P1，孕41+2周，经产钳助产阴道分娩分娩，产时出血约400ml，新生儿男婴、体重3700g，Apgar评分10、10、10分。病例提示的重点风险为胎膜早破或感染相关风险、胎儿窘迫或羊水异常记录。产后24小时内重点评估体温、脉搏、血压、宫底高度、宫缩、阴道流血、恶露颜色和尿潴留，记录首次排尿和疼痛评分。产后住院及出院后继续观察恶露是否由红色逐渐转淡，乳房是否胀痛，乳头是否皲裂，睡眠和情绪是否稳定。饮食以优质蛋白、含铁食物、蔬菜水果和足量饮水为主，避免盲目大补和长期卧床。本次为产钳助产阴道分娩，会阴伤口、盆底和排尿功能是护理重点。每次大小便后用温水从前向后清洁会阴并轻轻擦干，观察侧切或裂伤处肿胀、硬结、渗血、分泌物和异味。疼痛明显时可侧卧休息，避免久蹲、负重和过早剧烈活动。哺乳方面建议尽早母婴皮肤接触和按需哺乳，指导正确含接，乳胀时先热敷或轻柔按摩再哺乳，乳头疼痛时及时调整姿势。若出现乳房局部红肿硬痛、寒战或高热，应警惕乳腺炎。产时出血量需要持续观察，建议每日记录恶露量、颜色和血块情况，活动后若明显增多，应减少活动并请医护评估。存在胎膜早破、B族链球菌感染、阴道炎或宫内感染疑虑时，应加强体温、下腹压痛、恶露气味、会阴或切口渗液观察，发热或恶露恶臭时及时复诊。因有胎儿窘迫、羊水污染或新生儿评分变化记录，母婴同室期间应配合观察新生儿吃奶、肤色、体温、呼吸和反应，发现吃奶差、发绀、嗜睡或呼吸异常应立即告知儿科医生。出院前应完成用药、伤口、哺乳和避孕宣教，42天左右复查，必要时提前复诊。若出现阴道流血突然增多、鲜红血浸湿卫生巾、恶露恶臭、发热、持续腹痛、伤口化脓、排尿困难、下肢单侧肿痛、胸闷气短，或持续悲伤焦虑及伤害自己或婴儿的想法，应立即就医或急诊处理。 | 患者33岁，G2P1，产钳助产+IV度裂伤（最严重），合并GBS感染、高度近视、胎儿窘迫，脐带绕颈1周紧、捻转40周，产时出血400ml，新生儿Apgar 10。IV度裂伤累及肛门括约肌，需专科护理：保持伤口清洁，每日坐浴2-3次，使用大便软化剂（乳果糖）避免便秘，避免使用灌肠；产后6-8周行肛门括约肌功能评估，必要时盆底康复。GBS感染新生儿监测。高度近视避免用力。出血400ml注意晚期出血。提供心理支持。42天复查盆底、括约肌功能。 |
| **护理计划英文翻译 English translation of care plan** | The patient is 33 years old, G2P1, delivered at 41+2 weeks by forceps-assisted vaginal delivery, with an estimated intrapartum blood loss of 400 mL. The newborn was a male infant, weighed 3700 g, and had Apgar scores of 10, 10, 10. The main case-based risks include premature rupture of membranes or infection-related risk, fetal distress or abnormal amniotic fluid findings. Within the first 24 hours, assess temperature, pulse, blood pressure, uterine fundal height, uterine tone, vaginal bleeding, lochia color, urinary retention, first voiding, and pain score. During hospitalization and after discharge, continue to observe whether lochia gradually changes from red to lighter color, whether breast engorgement or nipple fissure occurs, and whether sleep and mood remain stable. Diet should include high-quality protein, iron-rich food, vegetables, fruit, and adequate fluids. Avoid blind over-supplementation and prolonged bed rest. Because this was a forceps-assisted vaginal delivery, perineal wound care, pelvic floor recovery, and urinary function are priorities. Rinse the perineum from front to back with warm water after urination or bowel movement and pat dry. Observe the episiotomy or tear for swelling, induration, bleeding, discharge, and odor. Rest in a lateral position if pain is marked, and avoid prolonged squatting, heavy lifting, and early vigorous activity. For breastfeeding, encourage early skin-to-skin contact and feeding on demand. Teach correct latch and positioning. For engorgement, apply warmth or gentle massage before feeding. If nipple pain occurs, adjust latch promptly. Localized breast redness, hard painful swelling, chills, or high fever should raise concern for mastitis. Because blood loss requires closer observation, record the amount, color, and clots in the lochia every day. If bleeding increases clearly after activity, reduce activity and seek clinical assessment. With premature rupture of membranes, group B streptococcus, vaginitis, or suspected intrauterine infection, monitor temperature, lower abdominal tenderness, lochia odor, and drainage from the perineal wound or incision. Return promptly for fever or foul-smelling lochia. Because fetal distress, meconium-stained fluid, or changed Apgar scores were recorded, cooperate with pediatric observation of feeding, skin color, temperature, breathing, and responsiveness. Poor feeding, cyanosis, excessive sleepiness, or abnormal breathing should be reported immediately. Before discharge, provide education on medication, wound care, breastfeeding, and contraception. Arrange a postpartum review around 42 days after delivery, or earlier if symptoms occur. Seek urgent medical care for sudden heavy vaginal bleeding, bright red bleeding soaking pads, foul-smelling lochia, fever, persistent abdominal pain, pus from the wound, difficulty urinating, unilateral leg swelling or pain, chest tightness, shortness of breath, or persistent sadness, anxiety, or thoughts of harming herself or the baby. | The patient is a 33-year-old G2P1 with forceps delivery and fourth-degree perineal laceration (involving anal sphincter). Comorbidities: GBS, high myopia, fetal distress, tight nuchal cord 1 loop, cord twisting 40 loops. Blood loss 400 mL, newborn Apgar 10. Fourth-degree tear – specialized care: keep clean, sitz baths 2-3 times daily, stool softeners (lactulose) to prevent constipation, avoid enemas. Assess anal sphincter function at 6-8 weeks, pelvic floor rehabilitation. GBS – newborn monitoring. High myopia – avoid Valsalva. Watch for late PPH. Psychological support. Follow-up: pelvic floor and sphincter assessment at 42 days. |
| **Clinical appropriateness 临床适当性** | 5 | 4 |
| **Individualization 个体化程度** | 5 | 5 |
| **Comprehensiveness 完整性** | 5 | 4 |
| **Actionability 可操作性** | 4 | 4 |
| **Safety 安全性** | 5 | 4 |
| **Triage appropriateness 分诊/转诊建议适当性** | 5 | 3 |
| **Structure and clarity 结构与清晰度** | 4 | 3 |

**病例 72 / Case 72**

| **项目 Item** | **ChatGPT 5.5** | **DeepSeek V3** |
| --- | --- | --- |
| **去标识化临床病例摘要 De-identified Clinical Case Summary** | 患者32岁，孕产史为G1P1，既往剖宫产0次，孕周38+3，临产类别为催产素引产。自然破膜，羊水量约100ml，羊水性质为清。以产钳分娩，产时出血约290ml。第一产程3时30分，第二产程1时6分，第三产程0时4分，总产程4时40分，会阴或切口情况为侧切，内缝。合并症及主要风险因素：单脐动脉；胎膜早破；胎儿宫内窘迫？。新生儿出生体重2760g，Apgar评分1、5、10分钟分别为9、10、10分，分娩结局为活产。 | |
| **临床病例摘要英文翻译 English Translation of De-identified Clinical Case Summary** | Maternal age was 32 years. The obstetric history was G1P1, with 0 previous cesarean deliveries. Gestational age was 38+3 weeks, and the labor onset or induction category was oxytocin induction. The membranes ruptured spontaneously; the amniotic fluid volume was approximately 100 mL and its character was clear. The patient had a forceps-assisted vaginal delivery, with estimated intrapartum blood loss of 290 mL. The first, second, and third stages of labor were 3 h 30 min, 1 h 6 min, and 0 h 4 min, respectively; the total labor duration was 4 h 40 min. An episiotomy was repaired with internal sutures. Documented comorbidities and major risk factors were single umbilical artery; premature rupture of membranes; suspected fetal distress. Neonatal birth weight was 2760 g; Apgar scores at 1, 5, and 10 minutes were 9, 10, 10; and the delivery outcome was live birth. | |
| **模型生成的产褥期护理计划 Model-generated postpartum nursing care plan** | 产妇32岁，G1P1，孕38+3周，经产钳助产阴道分娩分娩，产时出血约290ml，新生儿女婴、体重2760g，Apgar评分9、10、10分。病例提示的重点风险为胎膜早破或感染相关风险、胎儿窘迫或羊水异常记录。产后24小时内重点评估体温、脉搏、血压、宫底高度、宫缩、阴道流血、恶露颜色和尿潴留，记录首次排尿和疼痛评分。产后住院及出院后继续观察恶露是否由红色逐渐转淡，乳房是否胀痛，乳头是否皲裂，睡眠和情绪是否稳定。饮食以优质蛋白、含铁食物、蔬菜水果和足量饮水为主，避免盲目大补和长期卧床。本次为产钳助产阴道分娩，会阴伤口、盆底和排尿功能是护理重点。每次大小便后用温水从前向后清洁会阴并轻轻擦干，观察侧切或裂伤处肿胀、硬结、渗血、分泌物和异味。疼痛明显时可侧卧休息，避免久蹲、负重和过早剧烈活动。哺乳方面建议尽早母婴皮肤接触和按需哺乳，指导正确含接，乳胀时先热敷或轻柔按摩再哺乳，乳头疼痛时及时调整姿势。若出现乳房局部红肿硬痛、寒战或高热，应警惕乳腺炎。存在胎膜早破、B族链球菌感染、阴道炎或宫内感染疑虑时，应加强体温、下腹压痛、恶露气味、会阴或切口渗液观察，发热或恶露恶臭时及时复诊。因有胎儿窘迫、羊水污染或新生儿评分变化记录，母婴同室期间应配合观察新生儿吃奶、肤色、体温、呼吸和反应，发现吃奶差、发绀、嗜睡或呼吸异常应立即告知儿科医生。出院前应完成用药、伤口、哺乳和避孕宣教，42天左右复查，必要时提前复诊。若出现阴道流血突然增多、鲜红血浸湿卫生巾、恶露恶臭、发热、持续腹痛、伤口化脓、排尿困难、下肢单侧肿痛、胸闷气短，或持续悲伤焦虑及伤害自己或婴儿的想法，应立即就医或急诊处理。 | 患者32岁，初产妇，产钳+侧切，合并单脐动脉、胎膜早破、可疑胎儿窘迫，脐带捻转25周，新生儿Apgar 9-10-10。单脐动脉新生儿需心脏超声检查。PPROM监测感染。脐带捻转+可疑窘迫，新生儿观察神经系统。会阴侧切护理。出血290ml正常。提供心理支持。42天复查；新生儿心脏超声。 |
| **护理计划英文翻译 English translation of care plan** | The patient is 32 years old, G1P1, delivered at 38+3 weeks by forceps-assisted vaginal delivery, with an estimated intrapartum blood loss of 290 mL. The newborn was a female infant, weighed 2760 g, and had Apgar scores of 9, 10, 10. The main case-based risks include premature rupture of membranes or infection-related risk, fetal distress or abnormal amniotic fluid findings. Within the first 24 hours, assess temperature, pulse, blood pressure, uterine fundal height, uterine tone, vaginal bleeding, lochia color, urinary retention, first voiding, and pain score. During hospitalization and after discharge, continue to observe whether lochia gradually changes from red to lighter color, whether breast engorgement or nipple fissure occurs, and whether sleep and mood remain stable. Diet should include high-quality protein, iron-rich food, vegetables, fruit, and adequate fluids. Avoid blind over-supplementation and prolonged bed rest. Because this was a forceps-assisted vaginal delivery, perineal wound care, pelvic floor recovery, and urinary function are priorities. Rinse the perineum from front to back with warm water after urination or bowel movement and pat dry. Observe the episiotomy or tear for swelling, induration, bleeding, discharge, and odor. Rest in a lateral position if pain is marked, and avoid prolonged squatting, heavy lifting, and early vigorous activity. For breastfeeding, encourage early skin-to-skin contact and feeding on demand. Teach correct latch and positioning. For engorgement, apply warmth or gentle massage before feeding. If nipple pain occurs, adjust latch promptly. Localized breast redness, hard painful swelling, chills, or high fever should raise concern for mastitis. With premature rupture of membranes, group B streptococcus, vaginitis, or suspected intrauterine infection, monitor temperature, lower abdominal tenderness, lochia odor, and drainage from the perineal wound or incision. Return promptly for fever or foul-smelling lochia. Because fetal distress, meconium-stained fluid, or changed Apgar scores were recorded, cooperate with pediatric observation of feeding, skin color, temperature, breathing, and responsiveness. Poor feeding, cyanosis, excessive sleepiness, or abnormal breathing should be reported immediately. Before discharge, provide education on medication, wound care, breastfeeding, and contraception. Arrange a postpartum review around 42 days after delivery, or earlier if symptoms occur. Seek urgent medical care for sudden heavy vaginal bleeding, bright red bleeding soaking pads, foul-smelling lochia, fever, persistent abdominal pain, pus from the wound, difficulty urinating, unilateral leg swelling or pain, chest tightness, shortness of breath, or persistent sadness, anxiety, or thoughts of harming herself or the baby. | The patient is a 32-year-old primipara with forceps delivery and episiotomy. Comorbidities: single umbilical artery, PPROM, suspected fetal distress, cord twisting 25 loops. Newborn Apgar 9-10-10. SUA – newborn cardiac ultrasound. Monitor infection. Newborn neurological observation. Episiotomy care. Blood loss 290 mL, normal. Psychological support. Follow-up at 42 days; newborn cardiac echo. |
| **Clinical appropriateness 临床适当性** | 5 | 4 |
| **Individualization 个体化程度** | 4 | 5 |
| **Comprehensiveness 完整性** | 5 | 3 |
| **Actionability 可操作性** | 4 | 4 |
| **Safety 安全性** | 5 | 4 |
| **Triage appropriateness 分诊/转诊建议适当性** | 5 | 3 |
| **Structure and clarity 结构与清晰度** | 4 | 4 |

**病例 73 / Case 73**

| **项目 Item** | **ChatGPT 5.5** | **DeepSeek V3** |
| --- | --- | --- |
| **去标识化临床病例摘要 De-identified Clinical Case Summary** | 患者32岁，孕产史为G2P2，既往剖宫产0次，孕周38+5，临产类别为自然临产。自然破膜，羊水量约300ml，羊水性质为清。以自然分娩分娩，产时出血约210ml。第一产程2时55分，第二产程0时4分，第三产程0时6分，总产程3时5分，会阴或切口情况为Ⅰ，内缝。合并症及主要风险因素：甲状腺功能减退症。新生儿出生体重3110g，Apgar评分1、5、10分钟分别为10、10、10分，分娩结局为活产。 | |
| **临床病例摘要英文翻译 English Translation of De-identified Clinical Case Summary** | Maternal age was 32 years. The obstetric history was G2P2, with 0 previous cesarean deliveries. Gestational age was 38+5 weeks, and the labor onset or induction category was spontaneous labor. The membranes ruptured spontaneously; the amniotic fluid volume was approximately 300 mL and its character was clear. The patient had a spontaneous vaginal delivery, with estimated intrapartum blood loss of 210 mL. The first, second, and third stages of labor were 2 h 55 min, 0 h 4 min, and 0 h 6 min, respectively; the total labor duration was 3 h 5 min. A first-degree perineal laceration was repaired with internal sutures. Documented comorbidities and major risk factors were hypothyroidism. Neonatal birth weight was 3110 g; Apgar scores at 1, 5, and 10 minutes were 10, 10, 10; and the delivery outcome was live birth. | |
| **模型生成的产褥期护理计划 Model-generated postpartum nursing care plan** | 产妇32岁，G2P2，孕38+5周，经阴道自然分娩分娩，产时出血约210ml，新生儿男婴、体重3110g，Apgar评分10、10、10分。病例提示的重点风险为甲状腺功能异常。产后24小时内重点评估体温、脉搏、血压、宫底高度、宫缩、阴道流血、恶露颜色和尿潴留，记录首次排尿和疼痛评分。产后住院及出院后继续观察恶露是否由红色逐渐转淡，乳房是否胀痛，乳头是否皲裂，睡眠和情绪是否稳定。饮食以优质蛋白、含铁食物、蔬菜水果和足量饮水为主，避免盲目大补和长期卧床。本次为阴道自然分娩，应重点观察会阴裂伤或缝合处恢复。每次大小便后保持局部清洁干燥，观察红肿、渗血、疼痛加重和异常气味。无头晕和出血增多时逐步增加活动，并在医护指导下开始轻柔盆底肌训练。哺乳方面建议尽早母婴皮肤接触和按需哺乳，指导正确含接，乳胀时先热敷或轻柔按摩再哺乳，乳头疼痛时及时调整姿势。若出现乳房局部红肿硬痛、寒战或高热，应警惕乳腺炎。合并甲状腺疾病者，应按内分泌医嘱继续或调整用药，不自行停药，产后复查甲状腺功能，观察心悸、怕冷、乏力、出汗和情绪异常。出院前应完成用药、伤口、哺乳和避孕宣教，42天左右复查，必要时提前复诊。若出现阴道流血突然增多、鲜红血浸湿卫生巾、恶露恶臭、发热、持续腹痛、伤口化脓、排尿困难、下肢单侧肿痛、胸闷气短，或持续悲伤焦虑及伤害自己或婴儿的想法，应立即就医或急诊处理。 | 患者32岁，G2P2，自然分娩+I度裂伤，合并甲减。继续左甲状腺素，6周复查。常规产后护理。42天复查甲功。 |
| **护理计划英文翻译 English translation of care plan** | The patient is 32 years old, G2P2, delivered at 38+5 weeks by spontaneous vaginal delivery, with an estimated intrapartum blood loss of 210 mL. The newborn was a male infant, weighed 3110 g, and had Apgar scores of 10, 10, 10. The main case-based risks include thyroid dysfunction. Within the first 24 hours, assess temperature, pulse, blood pressure, uterine fundal height, uterine tone, vaginal bleeding, lochia color, urinary retention, first voiding, and pain score. During hospitalization and after discharge, continue to observe whether lochia gradually changes from red to lighter color, whether breast engorgement or nipple fissure occurs, and whether sleep and mood remain stable. Diet should include high-quality protein, iron-rich food, vegetables, fruit, and adequate fluids. Avoid blind over-supplementation and prolonged bed rest. Because this was a spontaneous vaginal birth, recovery of any perineal tear or suture site should be observed. Keep the area clean and dry after urination or bowel movement and watch for redness, swelling, bleeding, worsening pain, or abnormal odor. If there is no dizziness or increased bleeding, increase activity gradually and begin gentle pelvic floor exercises under professional guidance. For breastfeeding, encourage early skin-to-skin contact and feeding on demand. Teach correct latch and positioning. For engorgement, apply warmth or gentle massage before feeding. If nipple pain occurs, adjust latch promptly. Localized breast redness, hard painful swelling, chills, or high fever should raise concern for mastitis. For thyroid disease, continue or adjust medication only under endocrine guidance, recheck thyroid function after delivery, and watch for palpitations, cold intolerance, fatigue, sweating, or abnormal mood changes. Before discharge, provide education on medication, wound care, breastfeeding, and contraception. Arrange a postpartum review around 42 days after delivery, or earlier if symptoms occur. Seek urgent medical care for sudden heavy vaginal bleeding, bright red bleeding soaking pads, foul-smelling lochia, fever, persistent abdominal pain, pus from the wound, difficulty urinating, unilateral leg swelling or pain, chest tightness, shortness of breath, or persistent sadness, anxiety, or thoughts of harming herself or the baby. | The patient is a 32-year-old G2P2 with spontaneous delivery and first-degree laceration. Comorbidity: hypothyroidism. Continue levothyroxine, retest at 6 weeks. Routine care. Follow-up at 42 days. |
| **Clinical appropriateness 临床适当性** | 4 | 3 |
| **Individualization 个体化程度** | 3 | 3 |
| **Comprehensiveness 完整性** | 4 | 3 |
| **Actionability 可操作性** | 4 | 3 |
| **Safety 安全性** | 5 | 3 |
| **Triage appropriateness 分诊/转诊建议适当性** | 5 | 3 |
| **Structure and clarity 结构与清晰度** | 3 | 4 |

**病例 74 / Case 74**

| **项目 Item** | **ChatGPT 5.5** | **DeepSeek V3** |
| --- | --- | --- |
| **去标识化临床病例摘要 De-identified Clinical Case Summary** | 患者30岁，孕产史为G1P1，既往剖宫产0次，孕周39+3，临产类别为自然临产。人工破膜，羊水量约100ml，羊水性质为Ⅲ°。以产钳分娩，产时出血约340ml。第一产程12时3分，第二产程0时57分，第三产程0时5分，总产程13时5分，会阴或切口情况为侧切，内缝。合并症及主要风险因素：胎儿窘迫、妊娠合并子宫肌瘤。新生儿出生体重3540g，Apgar评分1、5、10分钟分别为9、10、10分，分娩结局为活产。 | |
| **临床病例摘要英文翻译 English Translation of De-identified Clinical Case Summary** | Maternal age was 30 years. The obstetric history was G1P1, with 0 previous cesarean deliveries. Gestational age was 39+3 weeks, and the labor onset or induction category was spontaneous labor. The membranes were artificially ruptured; the amniotic fluid volume was approximately 100 mL and its character was grade III meconium-stained. The patient had a forceps-assisted vaginal delivery, with estimated intrapartum blood loss of 340 mL. The first, second, and third stages of labor were 12 h 3 min, 0 h 57 min, and 0 h 5 min, respectively; the total labor duration was 13 h 5 min. An episiotomy was repaired with internal sutures. Documented comorbidities and major risk factors were fetal distress; uterine fibroid during pregnancy. Neonatal birth weight was 3540 g; Apgar scores at 1, 5, and 10 minutes were 9, 10, 10; and the delivery outcome was live birth. | |
| **模型生成的产褥期护理计划 Model-generated postpartum nursing care plan** | 产妇30岁，G1P1，孕39+3周，经产钳助产阴道分娩分娩，产时出血约340ml，新生儿男婴、体重3540g，Apgar评分9、10、10分。病例提示的重点风险为胎儿窘迫或羊水异常记录。产后24小时内重点评估体温、脉搏、血压、宫底高度、宫缩、阴道流血、恶露颜色和尿潴留，记录首次排尿和疼痛评分。产后住院及出院后继续观察恶露是否由红色逐渐转淡，乳房是否胀痛，乳头是否皲裂，睡眠和情绪是否稳定。饮食以优质蛋白、含铁食物、蔬菜水果和足量饮水为主，避免盲目大补和长期卧床。本次为产钳助产阴道分娩，会阴伤口、盆底和排尿功能是护理重点。每次大小便后用温水从前向后清洁会阴并轻轻擦干，观察侧切或裂伤处肿胀、硬结、渗血、分泌物和异味。疼痛明显时可侧卧休息，避免久蹲、负重和过早剧烈活动。哺乳方面建议尽早母婴皮肤接触和按需哺乳，指导正确含接，乳胀时先热敷或轻柔按摩再哺乳，乳头疼痛时及时调整姿势。若出现乳房局部红肿硬痛、寒战或高热，应警惕乳腺炎。因有胎儿窘迫、羊水污染或新生儿评分变化记录，母婴同室期间应配合观察新生儿吃奶、肤色、体温、呼吸和反应，发现吃奶差、发绀、嗜睡或呼吸异常应立即告知儿科医生。合并子宫肌瘤、卵巢囊肿或多囊卵巢相关情况时，产后复查时建议结合妇科超声评估，若腹痛、包块感或异常出血加重，应提前就诊。出院前应完成用药、伤口、哺乳和避孕宣教，42天左右复查，必要时提前复诊。若出现阴道流血突然增多、鲜红血浸湿卫生巾、恶露恶臭、发热、持续腹痛、伤口化脓、排尿困难、下肢单侧肿痛、胸闷气短，或持续悲伤焦虑及伤害自己或婴儿的想法，应立即就医或急诊处理。 | 患者30岁，初产妇，产钳+侧切，合并胎儿窘迫、子宫肌瘤，脐带绕颈1周（紧），产时出血340ml，新生儿Apgar 9-10-10。子宫肌瘤影响复旧，加强宫底按摩。脐带绕颈紧+窘迫，新生儿观察24-48小时。出血340ml，注意晚期出血。会阴侧切护理。提供心理支持。42天复查B超肌瘤。 |
| **护理计划英文翻译 English translation of care plan** | The patient is 30 years old, G1P1, delivered at 39+3 weeks by forceps-assisted vaginal delivery, with an estimated intrapartum blood loss of 340 mL. The newborn was a male infant, weighed 3540 g, and had Apgar scores of 9, 10, 10. The main case-based risks include fetal distress or abnormal amniotic fluid findings. Within the first 24 hours, assess temperature, pulse, blood pressure, uterine fundal height, uterine tone, vaginal bleeding, lochia color, urinary retention, first voiding, and pain score. During hospitalization and after discharge, continue to observe whether lochia gradually changes from red to lighter color, whether breast engorgement or nipple fissure occurs, and whether sleep and mood remain stable. Diet should include high-quality protein, iron-rich food, vegetables, fruit, and adequate fluids. Avoid blind over-supplementation and prolonged bed rest. Because this was a forceps-assisted vaginal delivery, perineal wound care, pelvic floor recovery, and urinary function are priorities. Rinse the perineum from front to back with warm water after urination or bowel movement and pat dry. Observe the episiotomy or tear for swelling, induration, bleeding, discharge, and odor. Rest in a lateral position if pain is marked, and avoid prolonged squatting, heavy lifting, and early vigorous activity. For breastfeeding, encourage early skin-to-skin contact and feeding on demand. Teach correct latch and positioning. For engorgement, apply warmth or gentle massage before feeding. If nipple pain occurs, adjust latch promptly. Localized breast redness, hard painful swelling, chills, or high fever should raise concern for mastitis. Because fetal distress, meconium-stained fluid, or changed Apgar scores were recorded, cooperate with pediatric observation of feeding, skin color, temperature, breathing, and responsiveness. Poor feeding, cyanosis, excessive sleepiness, or abnormal breathing should be reported immediately. For uterine fibroids, ovarian cysts, or polycystic ovary related findings, pelvic ultrasound can be considered at postpartum review. Earlier assessment is needed for worsening abdominal pain, mass sensation, or abnormal bleeding. Before discharge, provide education on medication, wound care, breastfeeding, and contraception. Arrange a postpartum review around 42 days after delivery, or earlier if symptoms occur. Seek urgent medical care for sudden heavy vaginal bleeding, bright red bleeding soaking pads, foul-smelling lochia, fever, persistent abdominal pain, pus from the wound, difficulty urinating, unilateral leg swelling or pain, chest tightness, shortness of breath, or persistent sadness, anxiety, or thoughts of harming herself or the baby. | The patient is a 30-year-old primipara with forceps delivery and episiotomy. Comorbidities: fetal distress, uterine fibroid, tight nuchal cord 1 loop. Blood loss 340 mL, newborn Apgar 9-10-10. Fibroid – fundal massage. Newborn observation for 24-48 hours. Watch for late PPH. Episiotomy care. Psychological support. Follow-up: fibroid ultrasound at 42 days. |
| **Clinical appropriateness 临床适当性** | 5 | 4 |
| **Individualization 个体化程度** | 4 | 5 |
| **Comprehensiveness 完整性** | 5 | 3 |
| **Actionability 可操作性** | 4 | 4 |
| **Safety 安全性** | 5 | 4 |
| **Triage appropriateness 分诊/转诊建议适当性** | 5 | 3 |
| **Structure and clarity 结构与清晰度** | 4 | 4 |

**病例 75 / Case 75**

| **项目 Item** | **ChatGPT 5.5** | **DeepSeek V3** |
| --- | --- | --- |
| **去标识化临床病例摘要 De-identified Clinical Case Summary** | 患者29岁，孕产史为G2P1，既往剖宫产0次，孕周40+2，临产类别为普贝生。自然破膜，羊水量约100ml，羊水性质为清。以自然分娩分娩，产时出血约300ml。第一产程14时0分，第二产程0时47分，第三产程0时8分，总产程14时55分，会阴或切口情况为侧切，内缝。合并症及主要风险因素：宫缩乏力；胎膜早破；妊娠期糖尿病；乙肝表面抗原携带者；妊娠合并卵巢囊肿？（右侧）；绒毛膜羊膜炎。新生儿出生体重3260g，Apgar评分1、5、10分钟分别为10、10、10分，分娩结局为活产。 | |
| **临床病例摘要英文翻译 English Translation of De-identified Clinical Case Summary** | Maternal age was 29 years. The obstetric history was G2P1, with 0 previous cesarean deliveries. Gestational age was 40+2 weeks, and the labor onset or induction category was vaginal dinoprostone (Propess) induction. The membranes ruptured spontaneously; the amniotic fluid volume was approximately 100 mL and its character was clear. The patient had a spontaneous vaginal delivery, with estimated intrapartum blood loss of 300 mL. The first, second, and third stages of labor were 14 h 0 min, 0 h 47 min, and 0 h 8 min, respectively; the total labor duration was 14 h 55 min. An episiotomy was repaired with internal sutures. Documented comorbidities and major risk factors were uterine atony; premature rupture of membranes; gestational diabetes mellitus; hepatitis B surface antigen carrier; suspected right ovarian cyst during pregnancy; chorioamnionitis. Neonatal birth weight was 3260 g; Apgar scores at 1, 5, and 10 minutes were 10, 10, 10; and the delivery outcome was live birth. | |
| **模型生成的产褥期护理计划 Model-generated postpartum nursing care plan** | 产妇29岁，G2P1，孕40+2周，经阴道自然分娩分娩，产时出血约300ml，新生儿女婴、体重3260g，Apgar评分10、10、10分。病例提示的重点风险为妊娠期糖尿病、胎膜早破或感染相关风险、出血或贫血相关风险。产后24小时内重点评估体温、脉搏、血压、宫底高度、宫缩、阴道流血、恶露颜色和尿潴留，记录首次排尿和疼痛评分。产后住院及出院后继续观察恶露是否由红色逐渐转淡，乳房是否胀痛，乳头是否皲裂，睡眠和情绪是否稳定。饮食以优质蛋白、含铁食物、蔬菜水果和足量饮水为主，避免盲目大补和长期卧床。本次为阴道自然分娩，应重点观察会阴裂伤或缝合处恢复。每次大小便后保持局部清洁干燥，观察红肿、渗血、疼痛加重和异常气味。无头晕和出血增多时逐步增加活动，并在医护指导下开始轻柔盆底肌训练。哺乳方面建议尽早母婴皮肤接触和按需哺乳，指导正确含接，乳胀时先热敷或轻柔按摩再哺乳，乳头疼痛时及时调整姿势。若出现乳房局部红肿硬痛、寒战或高热，应警惕乳腺炎。因存在出血量偏多、贫血或血小板异常相关风险，应复查血常规，关注头晕、心悸、乏力、面色苍白、恶露突然增多或较大血块，按医嘱补铁，必要时评估凝血和子宫复旧。有妊娠期糖尿病者，产后饮食应控制精制糖和高油食物，少量多餐，结合恢复情况逐步活动，并按医嘱在产后复查血糖或糖耐量。存在胎膜早破、B族链球菌感染、阴道炎或宫内感染疑虑时，应加强体温、下腹压痛、恶露气味、会阴或切口渗液观察，发热或恶露恶臭时及时复诊。如有Rh阴性血或乙肝相关记录，应核对母婴免疫预防和随访安排，避免漏查抗体、肝功能或新生儿免疫接种记录。合并子宫肌瘤、卵巢囊肿或多囊卵巢相关情况时，产后复查时建议结合妇科超声评估，若腹痛、包块感或异常出血加重，应提前就诊。出院前应完成用药、伤口、哺乳和避孕宣教，42天左右复查，必要时提前复诊。若出现阴道流血突然增多、鲜红血浸湿卫生巾、恶露恶臭、发热、持续腹痛、伤口化脓、排尿困难、下肢单侧肿痛、胸闷气短，或持续悲伤焦虑及伤害自己或婴儿的想法，应立即就医或急诊处理。 | 患者29岁，G2P1，自然分娩+侧切，合并宫缩乏力、PPROM、GDM、乙肝表面抗原携带者、卵巢囊肿可疑、绒毛膜羊膜炎，产时出血300ml。宫缩乏力需加强宫底按摩，使用缩宫素。PPROM+绒毛膜羊膜炎需抗生素治疗，监测感染。GDM监测血糖。乙肝携带者新生儿出生后12小时内接种乙肝免疫球蛋白和乙肝疫苗。卵巢囊肿6周B超。会阴侧切护理。提供心理支持。42天复查血糖、肝功能、B超。 |
| **护理计划英文翻译 English translation of care plan** | The patient is 29 years old, G2P1, delivered at 40+2 weeks by spontaneous vaginal delivery, with an estimated intrapartum blood loss of 300 mL. The newborn was a female infant, weighed 3260 g, and had Apgar scores of 10, 10, 10. The main case-based risks include gestational diabetes mellitus, premature rupture of membranes or infection-related risk, bleeding or anemia-related risk. Within the first 24 hours, assess temperature, pulse, blood pressure, uterine fundal height, uterine tone, vaginal bleeding, lochia color, urinary retention, first voiding, and pain score. During hospitalization and after discharge, continue to observe whether lochia gradually changes from red to lighter color, whether breast engorgement or nipple fissure occurs, and whether sleep and mood remain stable. Diet should include high-quality protein, iron-rich food, vegetables, fruit, and adequate fluids. Avoid blind over-supplementation and prolonged bed rest. Because this was a spontaneous vaginal birth, recovery of any perineal tear or suture site should be observed. Keep the area clean and dry after urination or bowel movement and watch for redness, swelling, bleeding, worsening pain, or abnormal odor. If there is no dizziness or increased bleeding, increase activity gradually and begin gentle pelvic floor exercises under professional guidance. For breastfeeding, encourage early skin-to-skin contact and feeding on demand. Teach correct latch and positioning. For engorgement, apply warmth or gentle massage before feeding. If nipple pain occurs, adjust latch promptly. Localized breast redness, hard painful swelling, chills, or high fever should raise concern for mastitis. Because increased blood loss, anemia, or platelet abnormality is a concern, repeat a complete blood count as advised and watch for dizziness, palpitations, severe fatigue, pallor, sudden increase in lochia, or large clots. Iron supplementation, nutrition support, and assessment of uterine involution or coagulation should follow medical orders. For gestational diabetes, avoid refined sugar and heavy oily foods, eat small balanced meals, increase activity gradually, and arrange postpartum glucose or oral glucose tolerance testing as advised. With premature rupture of membranes, group B streptococcus, vaginitis, or suspected intrauterine infection, monitor temperature, lower abdominal tenderness, lochia odor, and drainage from the perineal wound or incision. Return promptly for fever or foul-smelling lochia. For Rh-negative blood type or hepatitis B related records, confirm maternal and newborn immunoprophylaxis and follow-up, and avoid missing antibody, liver function, or infant vaccination documentation. For uterine fibroids, ovarian cysts, or polycystic ovary related findings, pelvic ultrasound can be considered at postpartum review. Earlier assessment is needed for worsening abdominal pain, mass sensation, or abnormal bleeding. Before discharge, provide education on medication, wound care, breastfeeding, and contraception. Arrange a postpartum review around 42 days after delivery, or earlier if symptoms occur. Seek urgent medical care for sudden heavy vaginal bleeding, bright red bleeding soaking pads, foul-smelling lochia, fever, persistent abdominal pain, pus from the wound, difficulty urinating, unilateral leg swelling or pain, chest tightness, shortness of breath, or persistent sadness, anxiety, or thoughts of harming herself or the baby. | The patient is a 29-year-old G2P1 with spontaneous delivery and episiotomy. Comorbidities: uterine inertia, PPROM, GDM, hepatitis B carrier, possible ovarian cyst, chorioamnionitis. Blood loss 300 mL. Uterine atony – fundal massage, oxytocin. Antibiotics for PPROM+chorio. Monitor glucose. Newborn – HBV vaccine and HBIG within 12 hours. Ovarian cyst – ultrasound at 6 weeks. Episiotomy care. Psychological support. Follow-up: glucose, LFTs, ultrasound at 42 days. |
| **Clinical appropriateness 临床适当性** | 5 | 4 |
| **Individualization 个体化程度** | 5 | 5 |
| **Comprehensiveness 完整性** | 5 | 4 |
| **Actionability 可操作性** | 5 | 4 |
| **Safety 安全性** | 5 | 4 |
| **Triage appropriateness 分诊/转诊建议适当性** | 5 | 3 |
| **Structure and clarity 结构与清晰度** | 4 | 3 |

**病例 76 / Case 76**

| **项目 Item** | **ChatGPT 5.5** | **DeepSeek V3** |
| --- | --- | --- |
| **去标识化临床病例摘要 De-identified Clinical Case Summary** | 患者35岁，孕产史为G2P2，既往剖宫产0次，孕周40+1，临产类别为自然临产。自然破膜，羊水量约300ml，羊水性质为清。以自然分娩分娩，产时出血约260ml。第一产程3时10分，第二产程0时10分，第三产程0时5分，总产程3时25分，会阴或切口情况为Ⅰ，内缝。合并症及主要风险因素：无。新生儿出生体重3570g，Apgar评分1、5、10分钟分别为10、10、10分，分娩结局为活产。 | |
| **临床病例摘要英文翻译 English Translation of De-identified Clinical Case Summary** | Maternal age was 35 years. The obstetric history was G2P2, with 0 previous cesarean deliveries. Gestational age was 40+1 weeks, and the labor onset or induction category was spontaneous labor. The membranes ruptured spontaneously; the amniotic fluid volume was approximately 300 mL and its character was clear. The patient had a spontaneous vaginal delivery, with estimated intrapartum blood loss of 260 mL. The first, second, and third stages of labor were 3 h 10 min, 0 h 10 min, and 0 h 5 min, respectively; the total labor duration was 3 h 25 min. A first-degree perineal laceration was repaired with internal sutures. No comorbidities or major risk factors were documented. Neonatal birth weight was 3570 g; Apgar scores at 1, 5, and 10 minutes were 10, 10, 10; and the delivery outcome was live birth. | |
| **模型生成的产褥期护理计划 Model-generated postpartum nursing care plan** | 产妇35岁，G2P2，孕40+1周，经阴道自然分娩分娩，产时出血约260ml，新生儿女婴、体重3570g，Apgar评分10、10、10分。病例提示的重点风险为高龄妊娠因素。产后24小时内重点评估体温、脉搏、血压、宫底高度、宫缩、阴道流血、恶露颜色和尿潴留，记录首次排尿和疼痛评分。产后住院及出院后继续观察恶露是否由红色逐渐转淡，乳房是否胀痛，乳头是否皲裂，睡眠和情绪是否稳定。饮食以优质蛋白、含铁食物、蔬菜水果和足量饮水为主，避免盲目大补和长期卧床。本次为阴道自然分娩，应重点观察会阴裂伤或缝合处恢复。每次大小便后保持局部清洁干燥，观察红肿、渗血、疼痛加重和异常气味。无头晕和出血增多时逐步增加活动，并在医护指导下开始轻柔盆底肌训练。哺乳方面建议尽早母婴皮肤接触和按需哺乳，指导正确含接，乳胀时先热敷或轻柔按摩再哺乳，乳头疼痛时及时调整姿势。若出现乳房局部红肿硬痛、寒战或高热，应警惕乳腺炎。高龄产妇恢复期应避免过度劳累和连续熬夜，家属应参与夜间照护，保证睡眠、营养和情绪支持。出院前应完成用药、伤口、哺乳和避孕宣教，42天左右复查，必要时提前复诊。若出现阴道流血突然增多、鲜红血浸湿卫生巾、恶露恶臭、发热、持续腹痛、伤口化脓、排尿困难、下肢单侧肿痛、胸闷气短，或持续悲伤焦虑及伤害自己或婴儿的想法，应立即就医或急诊处理。 | 患者35岁，G2P2，自然分娩+I度裂伤，无合并症。常规护理。42天复查。 |
| **护理计划英文翻译 English translation of care plan** | The patient is 35 years old, G2P2, delivered at 40+1 weeks by spontaneous vaginal delivery, with an estimated intrapartum blood loss of 260 mL. The newborn was a female infant, weighed 3570 g, and had Apgar scores of 10, 10, 10. The main case-based risks include advanced maternal age. Within the first 24 hours, assess temperature, pulse, blood pressure, uterine fundal height, uterine tone, vaginal bleeding, lochia color, urinary retention, first voiding, and pain score. During hospitalization and after discharge, continue to observe whether lochia gradually changes from red to lighter color, whether breast engorgement or nipple fissure occurs, and whether sleep and mood remain stable. Diet should include high-quality protein, iron-rich food, vegetables, fruit, and adequate fluids. Avoid blind over-supplementation and prolonged bed rest. Because this was a spontaneous vaginal birth, recovery of any perineal tear or suture site should be observed. Keep the area clean and dry after urination or bowel movement and watch for redness, swelling, bleeding, worsening pain, or abnormal odor. If there is no dizziness or increased bleeding, increase activity gradually and begin gentle pelvic floor exercises under professional guidance. For breastfeeding, encourage early skin-to-skin contact and feeding on demand. Teach correct latch and positioning. For engorgement, apply warmth or gentle massage before feeding. If nipple pain occurs, adjust latch promptly. Localized breast redness, hard painful swelling, chills, or high fever should raise concern for mastitis. Advanced maternal age may slow recovery, so the family should help with nighttime care and support adequate sleep, nutrition, and emotional stability. Before discharge, provide education on medication, wound care, breastfeeding, and contraception. Arrange a postpartum review around 42 days after delivery, or earlier if symptoms occur. Seek urgent medical care for sudden heavy vaginal bleeding, bright red bleeding soaking pads, foul-smelling lochia, fever, persistent abdominal pain, pus from the wound, difficulty urinating, unilateral leg swelling or pain, chest tightness, shortness of breath, or persistent sadness, anxiety, or thoughts of harming herself or the baby. | The patient is a 35-year-old G2P2 with spontaneous delivery and first-degree laceration. No comorbidities. Routine care. Follow-up at 42 days. |
| **Clinical appropriateness 临床适当性** | 4 | 3 |
| **Individualization 个体化程度** | 3 | 3 |
| **Comprehensiveness 完整性** | 4 | 3 |
| **Actionability 可操作性** | 4 | 3 |
| **Safety 安全性** | 5 | 3 |
| **Triage appropriateness 分诊/转诊建议适当性** | 5 | 3 |
| **Structure and clarity 结构与清晰度** | 3 | 4 |

**病例 77 / Case 77**

| **项目 Item** | **ChatGPT 5.5** | **DeepSeek V3** |
| --- | --- | --- |
| **去标识化临床病例摘要 De-identified Clinical Case Summary** | 患者28岁，孕产史为G1P1，既往剖宫产0次，孕周41，临产类别为普贝生。自然破膜，羊水量约300ml，羊水性质为Ⅲ°。以产钳分娩，产时出血约400ml。第一产程2时5分，第二产程0时7分，第三产程0时8分，总产程2时20分，会阴或切口情况为侧切，内缝。合并症及主要风险因素：妊娠合并甲状腺功能减退；胎儿窘迫。新生儿出生体重3050g，Apgar评分1、5、10分钟分别为10、10、10分，分娩结局为活产。 | |
| **临床病例摘要英文翻译 English Translation of De-identified Clinical Case Summary** | Maternal age was 28 years. The obstetric history was G1P1, with 0 previous cesarean deliveries. Gestational age was 41 weeks, and the labor onset or induction category was vaginal dinoprostone (Propess) induction. The membranes ruptured spontaneously; the amniotic fluid volume was approximately 300 mL and its character was grade III meconium-stained. The patient had a forceps-assisted vaginal delivery, with estimated intrapartum blood loss of 400 mL. The first, second, and third stages of labor were 2 h 5 min, 0 h 7 min, and 0 h 8 min, respectively; the total labor duration was 2 h 20 min. An episiotomy was repaired with internal sutures. Documented comorbidities and major risk factors were hypothyroidism during pregnancy; fetal distress. Neonatal birth weight was 3050 g; Apgar scores at 1, 5, and 10 minutes were 10, 10, 10; and the delivery outcome was live birth. | |
| **模型生成的产褥期护理计划 Model-generated postpartum nursing care plan** | 产妇28岁，G1P1，孕41周，经产钳助产阴道分娩分娩，产时出血约400ml，新生儿男婴、体重3050g，Apgar评分10、10、10分。病例提示的重点风险为甲状腺功能异常、胎儿窘迫或羊水异常记录。产后24小时内重点评估体温、脉搏、血压、宫底高度、宫缩、阴道流血、恶露颜色和尿潴留，记录首次排尿和疼痛评分。产后住院及出院后继续观察恶露是否由红色逐渐转淡，乳房是否胀痛，乳头是否皲裂，睡眠和情绪是否稳定。饮食以优质蛋白、含铁食物、蔬菜水果和足量饮水为主，避免盲目大补和长期卧床。本次为产钳助产阴道分娩，会阴伤口、盆底和排尿功能是护理重点。每次大小便后用温水从前向后清洁会阴并轻轻擦干，观察侧切或裂伤处肿胀、硬结、渗血、分泌物和异味。疼痛明显时可侧卧休息，避免久蹲、负重和过早剧烈活动。哺乳方面建议尽早母婴皮肤接触和按需哺乳，指导正确含接，乳胀时先热敷或轻柔按摩再哺乳，乳头疼痛时及时调整姿势。若出现乳房局部红肿硬痛、寒战或高热，应警惕乳腺炎。产时出血量需要持续观察，建议每日记录恶露量、颜色和血块情况，活动后若明显增多，应减少活动并请医护评估。合并甲状腺疾病者，应按内分泌医嘱继续或调整用药，不自行停药，产后复查甲状腺功能，观察心悸、怕冷、乏力、出汗和情绪异常。因有胎儿窘迫、羊水污染或新生儿评分变化记录，母婴同室期间应配合观察新生儿吃奶、肤色、体温、呼吸和反应，发现吃奶差、发绀、嗜睡或呼吸异常应立即告知儿科医生。出院前应完成用药、伤口、哺乳和避孕宣教，42天左右复查，必要时提前复诊。若出现阴道流血突然增多、鲜红血浸湿卫生巾、恶露恶臭、发热、持续腹痛、伤口化脓、排尿困难、下肢单侧肿痛、胸闷气短，或持续悲伤焦虑及伤害自己或婴儿的想法，应立即就医或急诊处理。 | 患者28岁，初产妇，产钳+侧切，合并甲减、胎儿窘迫，脐带绕颈1周，羊水Ⅲ°污染，产时出血400ml，新生儿Apgar 10。甲减继续左甲状腺素。Ⅲ°羊水+窘迫，新生儿监测MAS。出血400ml注意晚期出血。会阴侧切护理。42天复查甲功；新生儿随访。 |
| **护理计划英文翻译 English translation of care plan** | The patient is 28 years old, G1P1, delivered at 41 weeks by forceps-assisted vaginal delivery, with an estimated intrapartum blood loss of 400 mL. The newborn was a male infant, weighed 3050 g, and had Apgar scores of 10, 10, 10. The main case-based risks include thyroid dysfunction, fetal distress or abnormal amniotic fluid findings. Within the first 24 hours, assess temperature, pulse, blood pressure, uterine fundal height, uterine tone, vaginal bleeding, lochia color, urinary retention, first voiding, and pain score. During hospitalization and after discharge, continue to observe whether lochia gradually changes from red to lighter color, whether breast engorgement or nipple fissure occurs, and whether sleep and mood remain stable. Diet should include high-quality protein, iron-rich food, vegetables, fruit, and adequate fluids. Avoid blind over-supplementation and prolonged bed rest. Because this was a forceps-assisted vaginal delivery, perineal wound care, pelvic floor recovery, and urinary function are priorities. Rinse the perineum from front to back with warm water after urination or bowel movement and pat dry. Observe the episiotomy or tear for swelling, induration, bleeding, discharge, and odor. Rest in a lateral position if pain is marked, and avoid prolonged squatting, heavy lifting, and early vigorous activity. For breastfeeding, encourage early skin-to-skin contact and feeding on demand. Teach correct latch and positioning. For engorgement, apply warmth or gentle massage before feeding. If nipple pain occurs, adjust latch promptly. Localized breast redness, hard painful swelling, chills, or high fever should raise concern for mastitis. Because blood loss requires closer observation, record the amount, color, and clots in the lochia every day. If bleeding increases clearly after activity, reduce activity and seek clinical assessment. For thyroid disease, continue or adjust medication only under endocrine guidance, recheck thyroid function after delivery, and watch for palpitations, cold intolerance, fatigue, sweating, or abnormal mood changes. Because fetal distress, meconium-stained fluid, or changed Apgar scores were recorded, cooperate with pediatric observation of feeding, skin color, temperature, breathing, and responsiveness. Poor feeding, cyanosis, excessive sleepiness, or abnormal breathing should be reported immediately. Before discharge, provide education on medication, wound care, breastfeeding, and contraception. Arrange a postpartum review around 42 days after delivery, or earlier if symptoms occur. Seek urgent medical care for sudden heavy vaginal bleeding, bright red bleeding soaking pads, foul-smelling lochia, fever, persistent abdominal pain, pus from the wound, difficulty urinating, unilateral leg swelling or pain, chest tightness, shortness of breath, or persistent sadness, anxiety, or thoughts of harming herself or the baby. | The patient is a 28-year-old primipara with forceps delivery and episiotomy. Comorbidities: hypothyroidism, fetal distress, nuchal cord 1 loop, grade III meconium. Blood loss 400 mL, newborn Apgar 10. Continue levothyroxine. Newborn monitor for MAS. Watch for late PPH. Episiotomy care. Follow-up: thyroid at 42 days; newborn follow-up. |
| **Clinical appropriateness 临床适当性** | 5 | 3 |
| **Individualization 个体化程度** | 5 | 4 |
| **Comprehensiveness 完整性** | 5 | 3 |
| **Actionability 可操作性** | 4 | 4 |
| **Safety 安全性** | 5 | 3 |
| **Triage appropriateness 分诊/转诊建议适当性** | 5 | 3 |
| **Structure and clarity 结构与清晰度** | 3 | 3 |

**病例 78 / Case 78**

| **项目 Item** | **ChatGPT 5.5** | **DeepSeek V3** |
| --- | --- | --- |
| **去标识化临床病例摘要 De-identified Clinical Case Summary** | 患者28岁，孕产史为G1P1，既往剖宫产0次，孕周34，临产类别为自然临产。自然破膜，羊水量约300ml，羊水性质为清。以自然分娩分娩，产时出血约300ml。第一产程4时30分，第二产程0时20分，第三产程0时25分，总产程5时15分，会阴或切口情况为Ⅰ，内缝。合并症及主要风险因素：胎儿宫内窘迫？未足月胎膜早破；宫颈息肉？妊娠期贫血（轻度）。新生儿出生体重2110g，Apgar评分1、5、10分钟分别为9、9、9分，分娩结局为活产。 | |
| **临床病例摘要英文翻译 English Translation of De-identified Clinical Case Summary** | Maternal age was 28 years. The obstetric history was G1P1, with 0 previous cesarean deliveries. Gestational age was 34 weeks, and the labor onset or induction category was spontaneous labor. The membranes ruptured spontaneously; the amniotic fluid volume was approximately 300 mL and its character was clear. The patient had a spontaneous vaginal delivery, with estimated intrapartum blood loss of 300 mL. The first, second, and third stages of labor were 4 h 30 min, 0 h 20 min, and 0 h 25 min, respectively; the total labor duration was 5 h 15 min. A first-degree perineal laceration was repaired with internal sutures. Documented comorbidities and major risk factors were suspected fetal distress; preterm premature rupture of membranes; suspected cervical polyp; anemia during pregnancy (mild). Neonatal birth weight was 2110 g; Apgar scores at 1, 5, and 10 minutes were 9, 9, 9; and the delivery outcome was live birth. | |
| **模型生成的产褥期护理计划 Model-generated postpartum nursing care plan** | 产妇28岁，G1P1，孕34周，经阴道自然分娩分娩，产时出血约300ml，新生儿男婴、体重2110g，Apgar评分9、9、9分。病例提示的重点风险为胎膜早破或感染相关风险、出血或贫血相关风险、胎儿窘迫或羊水异常记录。产后24小时内重点评估体温、脉搏、血压、宫底高度、宫缩、阴道流血、恶露颜色和尿潴留，记录首次排尿和疼痛评分。产后住院及出院后继续观察恶露是否由红色逐渐转淡，乳房是否胀痛，乳头是否皲裂，睡眠和情绪是否稳定。饮食以优质蛋白、含铁食物、蔬菜水果和足量饮水为主，避免盲目大补和长期卧床。本次为阴道自然分娩，应重点观察会阴裂伤或缝合处恢复。每次大小便后保持局部清洁干燥，观察红肿、渗血、疼痛加重和异常气味。无头晕和出血增多时逐步增加活动，并在医护指导下开始轻柔盆底肌训练。哺乳方面建议尽早母婴皮肤接触和按需哺乳，指导正确含接，乳胀时先热敷或轻柔按摩再哺乳，乳头疼痛时及时调整姿势。若出现乳房局部红肿硬痛、寒战或高热，应警惕乳腺炎。因存在出血量偏多、贫血或血小板异常相关风险，应复查血常规，关注头晕、心悸、乏力、面色苍白、恶露突然增多或较大血块，按医嘱补铁，必要时评估凝血和子宫复旧。存在胎膜早破、B族链球菌感染、阴道炎或宫内感染疑虑时，应加强体温、下腹压痛、恶露气味、会阴或切口渗液观察，发热或恶露恶臭时及时复诊。因有胎儿窘迫、羊水污染或新生儿评分变化记录，母婴同室期间应配合观察新生儿吃奶、肤色、体温、呼吸和反应，发现吃奶差、发绀、嗜睡或呼吸异常应立即告知儿科医生。出院前应完成用药、伤口、哺乳和避孕宣教，42天左右复查，必要时提前复诊。若出现阴道流血突然增多、鲜红血浸湿卫生巾、恶露恶臭、发热、持续腹痛、伤口化脓、排尿困难、下肢单侧肿痛、胸闷气短，或持续悲伤焦虑及伤害自己或婴儿的想法，应立即就医或急诊处理。 | 患者28岁，初产妇，自然分娩+I度裂伤，孕34周早产，合并可疑胎儿窘迫、未足月胎膜早破、宫颈息肉可疑、轻度贫血，新生儿体重2110g（低出生体重），Apgar 9-9-9。早产儿需保暖、监测血糖、黄疸、呼吸暂停，鼓励母乳+强化剂。PPROM+早产，感染风险高，监测产妇体温、恶露。贫血补充铁剂。宫颈息肉产后可行妇科检查决定处理。提供心理支持。早产儿随访；42天复查血常规。 |
| **护理计划英文翻译 English translation of care plan** | The patient is 28 years old, G1P1, delivered at 34 weeks by spontaneous vaginal delivery, with an estimated intrapartum blood loss of 300 mL. The newborn was a male infant, weighed 2110 g, and had Apgar scores of 9, 9, 9. The main case-based risks include premature rupture of membranes or infection-related risk, bleeding or anemia-related risk, fetal distress or abnormal amniotic fluid findings. Within the first 24 hours, assess temperature, pulse, blood pressure, uterine fundal height, uterine tone, vaginal bleeding, lochia color, urinary retention, first voiding, and pain score. During hospitalization and after discharge, continue to observe whether lochia gradually changes from red to lighter color, whether breast engorgement or nipple fissure occurs, and whether sleep and mood remain stable. Diet should include high-quality protein, iron-rich food, vegetables, fruit, and adequate fluids. Avoid blind over-supplementation and prolonged bed rest. Because this was a spontaneous vaginal birth, recovery of any perineal tear or suture site should be observed. Keep the area clean and dry after urination or bowel movement and watch for redness, swelling, bleeding, worsening pain, or abnormal odor. If there is no dizziness or increased bleeding, increase activity gradually and begin gentle pelvic floor exercises under professional guidance. For breastfeeding, encourage early skin-to-skin contact and feeding on demand. Teach correct latch and positioning. For engorgement, apply warmth or gentle massage before feeding. If nipple pain occurs, adjust latch promptly. Localized breast redness, hard painful swelling, chills, or high fever should raise concern for mastitis. Because increased blood loss, anemia, or platelet abnormality is a concern, repeat a complete blood count as advised and watch for dizziness, palpitations, severe fatigue, pallor, sudden increase in lochia, or large clots. Iron supplementation, nutrition support, and assessment of uterine involution or coagulation should follow medical orders. With premature rupture of membranes, group B streptococcus, vaginitis, or suspected intrauterine infection, monitor temperature, lower abdominal tenderness, lochia odor, and drainage from the perineal wound or incision. Return promptly for fever or foul-smelling lochia. Because fetal distress, meconium-stained fluid, or changed Apgar scores were recorded, cooperate with pediatric observation of feeding, skin color, temperature, breathing, and responsiveness. Poor feeding, cyanosis, excessive sleepiness, or abnormal breathing should be reported immediately. Before discharge, provide education on medication, wound care, breastfeeding, and contraception. Arrange a postpartum review around 42 days after delivery, or earlier if symptoms occur. Seek urgent medical care for sudden heavy vaginal bleeding, bright red bleeding soaking pads, foul-smelling lochia, fever, persistent abdominal pain, pus from the wound, difficulty urinating, unilateral leg swelling or pain, chest tightness, shortness of breath, or persistent sadness, anxiety, or thoughts of harming herself or the baby. | The patient is a 28-year-old primipara with spontaneous delivery and first-degree laceration at 34 weeks. Comorbidities: suspected fetal distress, PPROM, possible cervical polyp, mild anemia. Newborn LBW 2110 g, Apgar 9-9-9. Preterm infant care: warmth, glucose, jaundice, apnea; breastfeeding with fortifier. High infection risk – monitor maternal temperature and lochia. Iron supplementation. Cervical polyp – gynecologic follow-up. Psychological support. Preterm infant follow-up; maternal CBC at 42 days. |
| **Clinical appropriateness 临床适当性** | 5 | 4 |
| **Individualization 个体化程度** | 5 | 5 |
| **Comprehensiveness 完整性** | 5 | 4 |
| **Actionability 可操作性** | 4 | 4 |
| **Safety 安全性** | 5 | 4 |
| **Triage appropriateness 分诊/转诊建议适当性** | 5 | 3 |
| **Structure and clarity 结构与清晰度** | 4 | 3 |

**病例 79 / Case 79**

| **项目 Item** | **ChatGPT 5.5** | **DeepSeek V3** |
| --- | --- | --- |
| **去标识化临床病例摘要 De-identified Clinical Case Summary** | 患者29岁，孕产史为G1P1，既往剖宫产0次，孕周38+3，临产类别为普贝生。自然破膜，羊水量约300ml，羊水性质为血性。以产钳分娩，产时出血约280ml。第一产程3时15分，第二产程0时18分，第三产程0时7分，总产程3时40分，会阴或切口情况为侧切，内缝。合并症及主要风险因素：胎儿宫内窘迫？妊娠期高血压。新生儿出生体重3120g，Apgar评分1、5、10分钟分别为10、10、10分，分娩结局为活产。 | |
| **临床病例摘要英文翻译 English Translation of De-identified Clinical Case Summary** | Maternal age was 29 years. The obstetric history was G1P1, with 0 previous cesarean deliveries. Gestational age was 38+3 weeks, and the labor onset or induction category was vaginal dinoprostone (Propess) induction. The membranes ruptured spontaneously; the amniotic fluid volume was approximately 300 mL and its character was blood-stained. The patient had a forceps-assisted vaginal delivery, with estimated intrapartum blood loss of 280 mL. The first, second, and third stages of labor were 3 h 15 min, 0 h 18 min, and 0 h 7 min, respectively; the total labor duration was 3 h 40 min. An episiotomy was repaired with internal sutures. Documented comorbidities and major risk factors were suspected fetal distress; gestational hypertension. Neonatal birth weight was 3120 g; Apgar scores at 1, 5, and 10 minutes were 10, 10, 10; and the delivery outcome was live birth. | |
| **模型生成的产褥期护理计划 Model-generated postpartum nursing care plan** | 产妇29岁，G1P1，孕38+3周，经产钳助产阴道分娩分娩，产时出血约280ml，新生儿女婴、体重3120g，Apgar评分10、10、10分。病例提示的重点风险为妊娠期高血压或子痫前期相关风险、胎儿窘迫或羊水异常记录。产后24小时内重点评估体温、脉搏、血压、宫底高度、宫缩、阴道流血、恶露颜色和尿潴留，记录首次排尿和疼痛评分。产后住院及出院后继续观察恶露是否由红色逐渐转淡，乳房是否胀痛，乳头是否皲裂，睡眠和情绪是否稳定。饮食以优质蛋白、含铁食物、蔬菜水果和足量饮水为主，避免盲目大补和长期卧床。本次为产钳助产阴道分娩，会阴伤口、盆底和排尿功能是护理重点。每次大小便后用温水从前向后清洁会阴并轻轻擦干，观察侧切或裂伤处肿胀、硬结、渗血、分泌物和异味。疼痛明显时可侧卧休息，避免久蹲、负重和过早剧烈活动。哺乳方面建议尽早母婴皮肤接触和按需哺乳，指导正确含接，乳胀时先热敷或轻柔按摩再哺乳，乳头疼痛时及时调整姿势。若出现乳房局部红肿硬痛、寒战或高热，应警惕乳腺炎。有高血压或子痫前期风险者，产后仍可能出现血压波动，应按医嘱监测血压，若持续头痛、眼花、胸闷、上腹痛、抽搐或水肿加重，应立即就医。因有胎儿窘迫、羊水污染或新生儿评分变化记录，母婴同室期间应配合观察新生儿吃奶、肤色、体温、呼吸和反应，发现吃奶差、发绀、嗜睡或呼吸异常应立即告知儿科医生。出院前应完成用药、伤口、哺乳和避孕宣教，42天左右复查，必要时提前复诊。若出现阴道流血突然增多、鲜红血浸湿卫生巾、恶露恶臭、发热、持续腹痛、伤口化脓、排尿困难、下肢单侧肿痛、胸闷气短，或持续悲伤焦虑及伤害自己或婴儿的想法，应立即就医或急诊处理。 | 患者29岁，初产妇，产钳+侧切，合并可疑胎儿窘迫、妊娠期高血压，羊水血性，产时出血280ml，新生儿Apgar 10。妊娠期高血压产后监测血压，若≥140/90需治疗。血性羊水提示产前出血，注意产后凝血及子宫收缩。新生儿观察。会阴侧切护理。提供心理支持。42天复查血压。 |
| **护理计划英文翻译 English translation of care plan** | The patient is 29 years old, G1P1, delivered at 38+3 weeks by forceps-assisted vaginal delivery, with an estimated intrapartum blood loss of 280 mL. The newborn was a female infant, weighed 3120 g, and had Apgar scores of 10, 10, 10. The main case-based risks include gestational hypertension or preeclampsia-related risk, fetal distress or abnormal amniotic fluid findings. Within the first 24 hours, assess temperature, pulse, blood pressure, uterine fundal height, uterine tone, vaginal bleeding, lochia color, urinary retention, first voiding, and pain score. During hospitalization and after discharge, continue to observe whether lochia gradually changes from red to lighter color, whether breast engorgement or nipple fissure occurs, and whether sleep and mood remain stable. Diet should include high-quality protein, iron-rich food, vegetables, fruit, and adequate fluids. Avoid blind over-supplementation and prolonged bed rest. Because this was a forceps-assisted vaginal delivery, perineal wound care, pelvic floor recovery, and urinary function are priorities. Rinse the perineum from front to back with warm water after urination or bowel movement and pat dry. Observe the episiotomy or tear for swelling, induration, bleeding, discharge, and odor. Rest in a lateral position if pain is marked, and avoid prolonged squatting, heavy lifting, and early vigorous activity. For breastfeeding, encourage early skin-to-skin contact and feeding on demand. Teach correct latch and positioning. For engorgement, apply warmth or gentle massage before feeding. If nipple pain occurs, adjust latch promptly. Localized breast redness, hard painful swelling, chills, or high fever should raise concern for mastitis. For hypertension or preeclampsia risk, postpartum blood pressure may still fluctuate. Continue blood pressure monitoring as advised and seek urgent care for persistent headache, visual symptoms, chest tightness, upper abdominal pain, seizure, or worsening edema. Because fetal distress, meconium-stained fluid, or changed Apgar scores were recorded, cooperate with pediatric observation of feeding, skin color, temperature, breathing, and responsiveness. Poor feeding, cyanosis, excessive sleepiness, or abnormal breathing should be reported immediately. Before discharge, provide education on medication, wound care, breastfeeding, and contraception. Arrange a postpartum review around 42 days after delivery, or earlier if symptoms occur. Seek urgent medical care for sudden heavy vaginal bleeding, bright red bleeding soaking pads, foul-smelling lochia, fever, persistent abdominal pain, pus from the wound, difficulty urinating, unilateral leg swelling or pain, chest tightness, shortness of breath, or persistent sadness, anxiety, or thoughts of harming herself or the baby. | The patient is a 29-year-old primipara with forceps delivery and episiotomy. Comorbidities: suspected fetal distress, gestational hypertension, bloody amniotic fluid. Blood loss 280 mL, newborn Apgar 10. Monitor BP postpartum; treat if ≥140/90. Bloody fluid – monitor coagulation and uterine contraction. Newborn observation. Episiotomy care. Psychological support. Follow-up: BP at 42 days. |
| **Clinical appropriateness 临床适当性** | 5 | 4 |
| **Individualization 个体化程度** | 4 | 5 |
| **Comprehensiveness 完整性** | 5 | 3 |
| **Actionability 可操作性** | 4 | 3 |
| **Safety 安全性** | 5 | 4 |
| **Triage appropriateness 分诊/转诊建议适当性** | 5 | 3 |
| **Structure and clarity 结构与清晰度** | 4 | 4 |

**病例 80 / Case 80**

| **项目 Item** | **ChatGPT 5.5** | **DeepSeek V3** |
| --- | --- | --- |
| **去标识化临床病例摘要 De-identified Clinical Case Summary** | 患者32岁，孕产史为G1P1，既往剖宫产0次，孕周34+2，临产类别为自然临产。自然破膜，羊水量约200ml，羊水性质为清。以自然分娩分娩，产时出血约200ml。第一产程4时0分，第二产程0时32分，第三产程0时8分，总产程4时40分，会阴或切口情况为Ⅰ，内缝。合并症及主要风险因素：妊娠合并甲状腺功能减退。新生儿出生体重1860g，Apgar评分1、5、10分钟分别为9、9、9分，分娩结局为活产。 | |
| **临床病例摘要英文翻译 English Translation of De-identified Clinical Case Summary** | Maternal age was 32 years. The obstetric history was G1P1, with 0 previous cesarean deliveries. Gestational age was 34+2 weeks, and the labor onset or induction category was spontaneous labor. The membranes ruptured spontaneously; the amniotic fluid volume was approximately 200 mL and its character was clear. The patient had a spontaneous vaginal delivery, with estimated intrapartum blood loss of 200 mL. The first, second, and third stages of labor were 4 h 0 min, 0 h 32 min, and 0 h 8 min, respectively; the total labor duration was 4 h 40 min. A first-degree perineal laceration was repaired with internal sutures. Documented comorbidities and major risk factors were hypothyroidism during pregnancy. Neonatal birth weight was 1860 g; Apgar scores at 1, 5, and 10 minutes were 9, 9, 9; and the delivery outcome was live birth. | |
| **模型生成的产褥期护理计划 Model-generated postpartum nursing care plan** | 产妇32岁，G1P1，孕34+2周，经阴道自然分娩分娩，产时出血约200ml，新生儿男婴、体重1860g，Apgar评分9、9、9分。病例提示的重点风险为甲状腺功能异常、胎儿窘迫或羊水异常记录。产后24小时内重点评估体温、脉搏、血压、宫底高度、宫缩、阴道流血、恶露颜色和尿潴留，记录首次排尿和疼痛评分。产后住院及出院后继续观察恶露是否由红色逐渐转淡，乳房是否胀痛，乳头是否皲裂，睡眠和情绪是否稳定。饮食以优质蛋白、含铁食物、蔬菜水果和足量饮水为主，避免盲目大补和长期卧床。本次为阴道自然分娩，应重点观察会阴裂伤或缝合处恢复。每次大小便后保持局部清洁干燥，观察红肿、渗血、疼痛加重和异常气味。无头晕和出血增多时逐步增加活动，并在医护指导下开始轻柔盆底肌训练。哺乳方面建议尽早母婴皮肤接触和按需哺乳，指导正确含接，乳胀时先热敷或轻柔按摩再哺乳，乳头疼痛时及时调整姿势。若出现乳房局部红肿硬痛、寒战或高热，应警惕乳腺炎。合并甲状腺疾病者，应按内分泌医嘱继续或调整用药，不自行停药，产后复查甲状腺功能，观察心悸、怕冷、乏力、出汗和情绪异常。因有胎儿窘迫、羊水污染或新生儿评分变化记录，母婴同室期间应配合观察新生儿吃奶、肤色、体温、呼吸和反应，发现吃奶差、发绀、嗜睡或呼吸异常应立即告知儿科医生。出院前应完成用药、伤口、哺乳和避孕宣教，42天左右复查，必要时提前复诊。若出现阴道流血突然增多、鲜红血浸湿卫生巾、恶露恶臭、发热、持续腹痛、伤口化脓、排尿困难、下肢单侧肿痛、胸闷气短，或持续悲伤焦虑及伤害自己或婴儿的想法，应立即就医或急诊处理。 | 患者32岁，初产妇，自然分娩+I度裂伤，孕34+2周早产，合并甲减，新生儿体重1860g（低出生体重），Apgar 9-9-9。早产儿需保暖、监测血糖、黄疸、呼吸暂停，鼓励母乳+强化剂，可能需要新生儿科住院。甲减继续左甲状腺素。常规会阴护理。提供心理支持。早产儿随访；42天复查甲功。 |
| **护理计划英文翻译 English translation of care plan** | The patient is 32 years old, G1P1, delivered at 34+2 weeks by spontaneous vaginal delivery, with an estimated intrapartum blood loss of 200 mL. The newborn was a male infant, weighed 1860 g, and had Apgar scores of 9, 9, 9. The main case-based risks include thyroid dysfunction, fetal distress or abnormal amniotic fluid findings. Within the first 24 hours, assess temperature, pulse, blood pressure, uterine fundal height, uterine tone, vaginal bleeding, lochia color, urinary retention, first voiding, and pain score. During hospitalization and after discharge, continue to observe whether lochia gradually changes from red to lighter color, whether breast engorgement or nipple fissure occurs, and whether sleep and mood remain stable. Diet should include high-quality protein, iron-rich food, vegetables, fruit, and adequate fluids. Avoid blind over-supplementation and prolonged bed rest. Because this was a spontaneous vaginal birth, recovery of any perineal tear or suture site should be observed. Keep the area clean and dry after urination or bowel movement and watch for redness, swelling, bleeding, worsening pain, or abnormal odor. If there is no dizziness or increased bleeding, increase activity gradually and begin gentle pelvic floor exercises under professional guidance. For breastfeeding, encourage early skin-to-skin contact and feeding on demand. Teach correct latch and positioning. For engorgement, apply warmth or gentle massage before feeding. If nipple pain occurs, adjust latch promptly. Localized breast redness, hard painful swelling, chills, or high fever should raise concern for mastitis. For thyroid disease, continue or adjust medication only under endocrine guidance, recheck thyroid function after delivery, and watch for palpitations, cold intolerance, fatigue, sweating, or abnormal mood changes. Because fetal distress, meconium-stained fluid, or changed Apgar scores were recorded, cooperate with pediatric observation of feeding, skin color, temperature, breathing, and responsiveness. Poor feeding, cyanosis, excessive sleepiness, or abnormal breathing should be reported immediately. Before discharge, provide education on medication, wound care, breastfeeding, and contraception. Arrange a postpartum review around 42 days after delivery, or earlier if symptoms occur. Seek urgent medical care for sudden heavy vaginal bleeding, bright red bleeding soaking pads, foul-smelling lochia, fever, persistent abdominal pain, pus from the wound, difficulty urinating, unilateral leg swelling or pain, chest tightness, shortness of breath, or persistent sadness, anxiety, or thoughts of harming herself or the baby. | The patient is a 32-year-old primipara with spontaneous delivery and first-degree laceration at 34+2 weeks. Comorbidities: hypothyroidism. Newborn LBW 1860 g, Apgar 9-9-9. Preterm infant care: warmth, glucose, jaundice, apnea; breastfeeding with fortifier; likely NICU admission. Continue levothyroxine. Routine perineal care. Psychological support. Preterm infant follow-up; maternal thyroid at 42 days. |
| **Clinical appropriateness 临床适当性** | 5 | 4 |
| **Individualization 个体化程度** | 4 | 5 |
| **Comprehensiveness 完整性** | 5 | 3 |
| **Actionability 可操作性** | 4 | 4 |
| **Safety 安全性** | 5 | 4 |
| **Triage appropriateness 分诊/转诊建议适当性** | 5 | 3 |
| **Structure and clarity 结构与清晰度** | 4 | 4 |

**病例 81 / Case 81**

| **项目 Item** | **ChatGPT 5.5** | **DeepSeek V3** |
| --- | --- | --- |
| **去标识化临床病例摘要 De-identified Clinical Case Summary** | 患者26岁，孕产史为G1P1，既往剖宫产0次，孕周39+2，临产类别为自然临产。自然破膜，羊水量约500ml，羊水性质为清。以自然分娩分娩，产时出血约190ml。第一产程10时45分，第二产程0时26分，第三产程0时9分，总产程11时20分，会阴或切口情况为Ⅰ，内缝。合并症及主要风险因素：无。新生儿出生体重3530g，Apgar评分1、5、10分钟分别为10、10、10分，分娩结局为活产。 | |
| **临床病例摘要英文翻译 English Translation of De-identified Clinical Case Summary** | Maternal age was 26 years. The obstetric history was G1P1, with 0 previous cesarean deliveries. Gestational age was 39+2 weeks, and the labor onset or induction category was spontaneous labor. The membranes ruptured spontaneously; the amniotic fluid volume was approximately 500 mL and its character was clear. The patient had a spontaneous vaginal delivery, with estimated intrapartum blood loss of 190 mL. The first, second, and third stages of labor were 10 h 45 min, 0 h 26 min, and 0 h 9 min, respectively; the total labor duration was 11 h 20 min. A first-degree perineal laceration was repaired with internal sutures. No comorbidities or major risk factors were documented. Neonatal birth weight was 3530 g; Apgar scores at 1, 5, and 10 minutes were 10, 10, 10; and the delivery outcome was live birth. | |
| **模型生成的产褥期护理计划 Model-generated postpartum nursing care plan** | 产妇26岁，G1P1，孕39+2周，经阴道自然分娩分娩，产时出血约190ml，新生儿男婴、体重3530g，Apgar评分10、10、10分。病例提示的重点风险为未记录特殊合并症。产后24小时内重点评估体温、脉搏、血压、宫底高度、宫缩、阴道流血、恶露颜色和尿潴留，记录首次排尿和疼痛评分。产后住院及出院后继续观察恶露是否由红色逐渐转淡，乳房是否胀痛，乳头是否皲裂，睡眠和情绪是否稳定。饮食以优质蛋白、含铁食物、蔬菜水果和足量饮水为主，避免盲目大补和长期卧床。本次为阴道自然分娩，应重点观察会阴裂伤或缝合处恢复。每次大小便后保持局部清洁干燥，观察红肿、渗血、疼痛加重和异常气味。无头晕和出血增多时逐步增加活动，并在医护指导下开始轻柔盆底肌训练。哺乳方面建议尽早母婴皮肤接触和按需哺乳，指导正确含接，乳胀时先热敷或轻柔按摩再哺乳，乳头疼痛时及时调整姿势。若出现乳房局部红肿硬痛、寒战或高热，应警惕乳腺炎。目前未记录特殊合并症，仍需按常规产褥期管理执行，重点是子宫复旧、恶露、伤口、哺乳、营养、活动和心理状态。出院前应完成用药、伤口、哺乳和避孕宣教，42天左右复查，必要时提前复诊。若出现阴道流血突然增多、鲜红血浸湿卫生巾、恶露恶臭、发热、持续腹痛、伤口化脓、排尿困难、下肢单侧肿痛、胸闷气短，或持续悲伤焦虑及伤害自己或婴儿的想法，应立即就医或急诊处理。 | 患者26岁，初产妇，自然分娩+I度裂伤，无合并症，脐带长度80cm（偏长）。偏长脐带无特殊，常规护理。42天复查。 |
| **护理计划英文翻译 English translation of care plan** | The patient is 26 years old, G1P1, delivered at 39+2 weeks by spontaneous vaginal delivery, with an estimated intrapartum blood loss of 190 mL. The newborn was a male infant, weighed 3530 g, and had Apgar scores of 10, 10, 10. The main case-based risks include no special comorbidity was recorded. Within the first 24 hours, assess temperature, pulse, blood pressure, uterine fundal height, uterine tone, vaginal bleeding, lochia color, urinary retention, first voiding, and pain score. During hospitalization and after discharge, continue to observe whether lochia gradually changes from red to lighter color, whether breast engorgement or nipple fissure occurs, and whether sleep and mood remain stable. Diet should include high-quality protein, iron-rich food, vegetables, fruit, and adequate fluids. Avoid blind over-supplementation and prolonged bed rest. Because this was a spontaneous vaginal birth, recovery of any perineal tear or suture site should be observed. Keep the area clean and dry after urination or bowel movement and watch for redness, swelling, bleeding, worsening pain, or abnormal odor. If there is no dizziness or increased bleeding, increase activity gradually and begin gentle pelvic floor exercises under professional guidance. For breastfeeding, encourage early skin-to-skin contact and feeding on demand. Teach correct latch and positioning. For engorgement, apply warmth or gentle massage before feeding. If nipple pain occurs, adjust latch promptly. Localized breast redness, hard painful swelling, chills, or high fever should raise concern for mastitis. No special complication was recorded, but routine postpartum management should still focus on uterine involution, lochia, wounds, breastfeeding, nutrition, activity, and mental health. Before discharge, provide education on medication, wound care, breastfeeding, and contraception. Arrange a postpartum review around 42 days after delivery, or earlier if symptoms occur. Seek urgent medical care for sudden heavy vaginal bleeding, bright red bleeding soaking pads, foul-smelling lochia, fever, persistent abdominal pain, pus from the wound, difficulty urinating, unilateral leg swelling or pain, chest tightness, shortness of breath, or persistent sadness, anxiety, or thoughts of harming herself or the baby. | The patient is a 26-year-old primipara with spontaneous delivery and first-degree laceration. No comorbidities, long cord (80cm). Routine care. Follow-up at 42 days. |
| **Clinical appropriateness 临床适当性** | 4 | 3 |
| **Individualization 个体化程度** | 3 | 3 |
| **Comprehensiveness 完整性** | 4 | 3 |
| **Actionability 可操作性** | 4 | 3 |
| **Safety 安全性** | 5 | 3 |
| **Triage appropriateness 分诊/转诊建议适当性** | 5 | 3 |
| **Structure and clarity 结构与清晰度** | 3 | 4 |

**病例 82 / Case 82**

| **项目 Item** | **ChatGPT 5.5** | **DeepSeek V3** |
| --- | --- | --- |
| **去标识化临床病例摘要 De-identified Clinical Case Summary** | 患者30岁，孕产史为G2P1，既往剖宫产0次，孕周40+6，临产类别为自然临产。人工破膜，羊水量约300ml，羊水性质为清。以产钳分娩，产时出血约400ml。第一产程13时0分，第二产程0时54分，第三产程0时6分，总产程14时0分，会阴或切口情况为侧切，内缝。合并症及主要风险因素：乙肝表面抗原携带者；甲状腺功能减退症，胎儿窘迫；。新生儿出生体重3110g，Apgar评分1、5、10分钟分别为10、10、10分，分娩结局为活产。 | |
| **临床病例摘要英文翻译 English Translation of De-identified Clinical Case Summary** | Maternal age was 30 years. The obstetric history was G2P1, with 0 previous cesarean deliveries. Gestational age was 40+6 weeks, and the labor onset or induction category was spontaneous labor. The membranes were artificially ruptured; the amniotic fluid volume was approximately 300 mL and its character was clear. The patient had a forceps-assisted vaginal delivery, with estimated intrapartum blood loss of 400 mL. The first, second, and third stages of labor were 13 h 0 min, 0 h 54 min, and 0 h 6 min, respectively; the total labor duration was 14 h 0 min. An episiotomy was repaired with internal sutures. Documented comorbidities and major risk factors were hepatitis B surface antigen carrier; hypothyroidism, fetal distress. Neonatal birth weight was 3110 g; Apgar scores at 1, 5, and 10 minutes were 10, 10, 10; and the delivery outcome was live birth. | |
| **模型生成的产褥期护理计划 Model-generated postpartum nursing care plan** | 产妇30岁，G2P1，孕40+6周，经产钳助产阴道分娩分娩，产时出血约400ml，新生儿女婴、体重3110g，Apgar评分10、10、10分。病例提示的重点风险为甲状腺功能异常、胎儿窘迫或羊水异常记录。产后24小时内重点评估体温、脉搏、血压、宫底高度、宫缩、阴道流血、恶露颜色和尿潴留，记录首次排尿和疼痛评分。产后住院及出院后继续观察恶露是否由红色逐渐转淡，乳房是否胀痛，乳头是否皲裂，睡眠和情绪是否稳定。饮食以优质蛋白、含铁食物、蔬菜水果和足量饮水为主，避免盲目大补和长期卧床。本次为产钳助产阴道分娩，会阴伤口、盆底和排尿功能是护理重点。每次大小便后用温水从前向后清洁会阴并轻轻擦干，观察侧切或裂伤处肿胀、硬结、渗血、分泌物和异味。疼痛明显时可侧卧休息，避免久蹲、负重和过早剧烈活动。哺乳方面建议尽早母婴皮肤接触和按需哺乳，指导正确含接，乳胀时先热敷或轻柔按摩再哺乳，乳头疼痛时及时调整姿势。若出现乳房局部红肿硬痛、寒战或高热，应警惕乳腺炎。产时出血量需要持续观察，建议每日记录恶露量、颜色和血块情况，活动后若明显增多，应减少活动并请医护评估。合并甲状腺疾病者，应按内分泌医嘱继续或调整用药，不自行停药，产后复查甲状腺功能，观察心悸、怕冷、乏力、出汗和情绪异常。因有胎儿窘迫、羊水污染或新生儿评分变化记录，母婴同室期间应配合观察新生儿吃奶、肤色、体温、呼吸和反应，发现吃奶差、发绀、嗜睡或呼吸异常应立即告知儿科医生。如有Rh阴性血或乙肝相关记录，应核对母婴免疫预防和随访安排，避免漏查抗体、肝功能或新生儿免疫接种记录。出院前应完成用药、伤口、哺乳和避孕宣教，42天左右复查，必要时提前复诊。若出现阴道流血突然增多、鲜红血浸湿卫生巾、恶露恶臭、发热、持续腹痛、伤口化脓、排尿困难、下肢单侧肿痛、胸闷气短，或持续悲伤焦虑及伤害自己或婴儿的想法，应立即就医或急诊处理。 | 患者30岁，G2P1，产钳+侧切，合并乙肝表面抗原携带者、甲减、胎儿窘迫，脐带扭转20周，产时出血400ml，新生儿Apgar 10。乙肝携带者新生儿接种疫苗+免疫球蛋白。甲减继续左甲状腺素。胎儿窘迫新生儿观察。出血400ml注意晚期出血。会阴侧切护理。42天复查甲功、肝功。 |
| **护理计划英文翻译 English translation of care plan** | The patient is 30 years old, G2P1, delivered at 40+6 weeks by forceps-assisted vaginal delivery, with an estimated intrapartum blood loss of 400 mL. The newborn was a female infant, weighed 3110 g, and had Apgar scores of 10, 10, 10. The main case-based risks include thyroid dysfunction, fetal distress or abnormal amniotic fluid findings. Within the first 24 hours, assess temperature, pulse, blood pressure, uterine fundal height, uterine tone, vaginal bleeding, lochia color, urinary retention, first voiding, and pain score. During hospitalization and after discharge, continue to observe whether lochia gradually changes from red to lighter color, whether breast engorgement or nipple fissure occurs, and whether sleep and mood remain stable. Diet should include high-quality protein, iron-rich food, vegetables, fruit, and adequate fluids. Avoid blind over-supplementation and prolonged bed rest. Because this was a forceps-assisted vaginal delivery, perineal wound care, pelvic floor recovery, and urinary function are priorities. Rinse the perineum from front to back with warm water after urination or bowel movement and pat dry. Observe the episiotomy or tear for swelling, induration, bleeding, discharge, and odor. Rest in a lateral position if pain is marked, and avoid prolonged squatting, heavy lifting, and early vigorous activity. For breastfeeding, encourage early skin-to-skin contact and feeding on demand. Teach correct latch and positioning. For engorgement, apply warmth or gentle massage before feeding. If nipple pain occurs, adjust latch promptly. Localized breast redness, hard painful swelling, chills, or high fever should raise concern for mastitis. Because blood loss requires closer observation, record the amount, color, and clots in the lochia every day. If bleeding increases clearly after activity, reduce activity and seek clinical assessment. For thyroid disease, continue or adjust medication only under endocrine guidance, recheck thyroid function after delivery, and watch for palpitations, cold intolerance, fatigue, sweating, or abnormal mood changes. Because fetal distress, meconium-stained fluid, or changed Apgar scores were recorded, cooperate with pediatric observation of feeding, skin color, temperature, breathing, and responsiveness. Poor feeding, cyanosis, excessive sleepiness, or abnormal breathing should be reported immediately. For Rh-negative blood type or hepatitis B related records, confirm maternal and newborn immunoprophylaxis and follow-up, and avoid missing antibody, liver function, or infant vaccination documentation. Before discharge, provide education on medication, wound care, breastfeeding, and contraception. Arrange a postpartum review around 42 days after delivery, or earlier if symptoms occur. Seek urgent medical care for sudden heavy vaginal bleeding, bright red bleeding soaking pads, foul-smelling lochia, fever, persistent abdominal pain, pus from the wound, difficulty urinating, unilateral leg swelling or pain, chest tightness, shortness of breath, or persistent sadness, anxiety, or thoughts of harming herself or the baby. | The patient is a 30-year-old G2P1 with forceps delivery and episiotomy. Comorbidities: hepatitis B carrier, hypothyroidism, fetal distress, cord twisting 20 loops. Blood loss 400 mL, newborn Apgar 10. Newborn – HBV vaccine+HBIG. Continue levothyroxine. Newborn observation for distress. Watch for late PPH. Episiotomy care. Follow-up: thyroid and LFTs at 42 days. |
| **Clinical appropriateness 临床适当性** | 5 | 4 |
| **Individualization 个体化程度** | 5 | 5 |
| **Comprehensiveness 完整性** | 5 | 3 |
| **Actionability 可操作性** | 5 | 4 |
| **Safety 安全性** | 5 | 4 |
| **Triage appropriateness 分诊/转诊建议适当性** | 5 | 3 |
| **Structure and clarity 结构与清晰度** | 4 | 3 |

**病例 83 / Case 83**

| **项目 Item** | **ChatGPT 5.5** | **DeepSeek V3** |
| --- | --- | --- |
| **去标识化临床病例摘要 De-identified Clinical Case Summary** | 患者34岁，孕产史为G2P1，既往剖宫产0次，孕周40+1，临产类别为自然临产。自然破膜，羊水量约300ml，羊水性质为清。以自然分娩分娩，产时出血约300ml。第一产程4时0分，第二产程0时58分，第三产程0时7分，总产程5时5分，会阴或切口情况为Ⅰ，内缝。合并症及主要风险因素：妊娠合并真菌性阴道炎；妊娠期细菌性阴道炎。新生儿出生体重3350g，Apgar评分1、5、10分钟分别为10、10、10分，分娩结局为活产。 | |
| **临床病例摘要英文翻译 English Translation of De-identified Clinical Case Summary** | Maternal age was 34 years. The obstetric history was G2P1, with 0 previous cesarean deliveries. Gestational age was 40+1 weeks, and the labor onset or induction category was spontaneous labor. The membranes ruptured spontaneously; the amniotic fluid volume was approximately 300 mL and its character was clear. The patient had a spontaneous vaginal delivery, with estimated intrapartum blood loss of 300 mL. The first, second, and third stages of labor were 4 h 0 min, 0 h 58 min, and 0 h 7 min, respectively; the total labor duration was 5 h 5 min. A first-degree perineal laceration was repaired with internal sutures. Documented comorbidities and major risk factors were vulvovaginal candidiasis during pregnancy; bacterial vaginosis during pregnancy. Neonatal birth weight was 3350 g; Apgar scores at 1, 5, and 10 minutes were 10, 10, 10; and the delivery outcome was live birth. | |
| **模型生成的产褥期护理计划 Model-generated postpartum nursing care plan** | 产妇34岁，G2P1，孕40+1周，经阴道自然分娩分娩，产时出血约300ml，新生儿男婴、体重3350g，Apgar评分10、10、10分。病例提示的重点风险为胎膜早破或感染相关风险。产后24小时内重点评估体温、脉搏、血压、宫底高度、宫缩、阴道流血、恶露颜色和尿潴留，记录首次排尿和疼痛评分。产后住院及出院后继续观察恶露是否由红色逐渐转淡，乳房是否胀痛，乳头是否皲裂，睡眠和情绪是否稳定。饮食以优质蛋白、含铁食物、蔬菜水果和足量饮水为主，避免盲目大补和长期卧床。本次为阴道自然分娩，应重点观察会阴裂伤或缝合处恢复。每次大小便后保持局部清洁干燥，观察红肿、渗血、疼痛加重和异常气味。无头晕和出血增多时逐步增加活动，并在医护指导下开始轻柔盆底肌训练。哺乳方面建议尽早母婴皮肤接触和按需哺乳，指导正确含接，乳胀时先热敷或轻柔按摩再哺乳，乳头疼痛时及时调整姿势。若出现乳房局部红肿硬痛、寒战或高热，应警惕乳腺炎。存在胎膜早破、B族链球菌感染、阴道炎或宫内感染疑虑时，应加强体温、下腹压痛、恶露气味、会阴或切口渗液观察，发热或恶露恶臭时及时复诊。出院前应完成用药、伤口、哺乳和避孕宣教，42天左右复查，必要时提前复诊。若出现阴道流血突然增多、鲜红血浸湿卫生巾、恶露恶臭、发热、持续腹痛、伤口化脓、排尿困难、下肢单侧肿痛、胸闷气短，或持续悲伤焦虑及伤害自己或婴儿的想法，应立即就医或急诊处理。 | 患者34岁，G2P1，自然分娩+I度裂伤，合并真菌性阴道炎、细菌性阴道炎，脐带绕颈2周、绕左上肢1周。阴道炎需产后继续治疗（局部用药）。新生儿观察脐带绕颈及肢体缠绕后遗症（活动、肤色）。常规会阴护理。42天复查阴道炎。 |
| **护理计划英文翻译 English translation of care plan** | The patient is 34 years old, G2P1, delivered at 40+1 weeks by spontaneous vaginal delivery, with an estimated intrapartum blood loss of 300 mL. The newborn was a male infant, weighed 3350 g, and had Apgar scores of 10, 10, 10. The main case-based risks include premature rupture of membranes or infection-related risk. Within the first 24 hours, assess temperature, pulse, blood pressure, uterine fundal height, uterine tone, vaginal bleeding, lochia color, urinary retention, first voiding, and pain score. During hospitalization and after discharge, continue to observe whether lochia gradually changes from red to lighter color, whether breast engorgement or nipple fissure occurs, and whether sleep and mood remain stable. Diet should include high-quality protein, iron-rich food, vegetables, fruit, and adequate fluids. Avoid blind over-supplementation and prolonged bed rest. Because this was a spontaneous vaginal birth, recovery of any perineal tear or suture site should be observed. Keep the area clean and dry after urination or bowel movement and watch for redness, swelling, bleeding, worsening pain, or abnormal odor. If there is no dizziness or increased bleeding, increase activity gradually and begin gentle pelvic floor exercises under professional guidance. For breastfeeding, encourage early skin-to-skin contact and feeding on demand. Teach correct latch and positioning. For engorgement, apply warmth or gentle massage before feeding. If nipple pain occurs, adjust latch promptly. Localized breast redness, hard painful swelling, chills, or high fever should raise concern for mastitis. With premature rupture of membranes, group B streptococcus, vaginitis, or suspected intrauterine infection, monitor temperature, lower abdominal tenderness, lochia odor, and drainage from the perineal wound or incision. Return promptly for fever or foul-smelling lochia. Before discharge, provide education on medication, wound care, breastfeeding, and contraception. Arrange a postpartum review around 42 days after delivery, or earlier if symptoms occur. Seek urgent medical care for sudden heavy vaginal bleeding, bright red bleeding soaking pads, foul-smelling lochia, fever, persistent abdominal pain, pus from the wound, difficulty urinating, unilateral leg swelling or pain, chest tightness, shortness of breath, or persistent sadness, anxiety, or thoughts of harming herself or the baby. | The patient is a 34-year-old G2P1 with spontaneous delivery and first-degree laceration. Comorbidities: fungal vaginitis, bacterial vaginosis, nuchal cord 2 loops, cord around left upper limb. Continue topical treatment for vaginitis. Newborn – observe for sequelae of cord entanglement (movement, skin color). Routine perineal care. Follow-up: vaginitis check at 42 days. |
| **Clinical appropriateness 临床适当性** | 4 | 3 |
| **Individualization 个体化程度** | 3 | 3 |
| **Comprehensiveness 完整性** | 4 | 3 |
| **Actionability 可操作性** | 4 | 3 |
| **Safety 安全性** | 5 | 3 |
| **Triage appropriateness 分诊/转诊建议适当性** | 5 | 3 |
| **Structure and clarity 结构与清晰度** | 3 | 4 |

**病例 84 / Case 84**

| **项目 Item** | **ChatGPT 5.5** | **DeepSeek V3** |
| --- | --- | --- |
| **去标识化临床病例摘要 De-identified Clinical Case Summary** | 患者33岁，孕产史为G1P1，既往剖宫产0次，孕周39+4，临产类别为自然临产。自然破膜，羊水量约200ml，羊水性质为Ⅲ°。以产钳分娩，产时出血约300ml。第一产程6时42分，第二产程0时30分，第三产程0时8分，总产程7时20分，会阴或切口情况为侧切，内缝。合并症及主要风险因素：妊娠合并卵巢囊肿？球拍状胎盘；高度近视；胎儿窘迫？；。新生儿出生体重3540g，Apgar评分1、5、10分钟分别为10、10、10分，分娩结局为活产。 | |
| **临床病例摘要英文翻译 English Translation of De-identified Clinical Case Summary** | Maternal age was 33 years. The obstetric history was G1P1, with 0 previous cesarean deliveries. Gestational age was 39+4 weeks, and the labor onset or induction category was spontaneous labor. The membranes ruptured spontaneously; the amniotic fluid volume was approximately 200 mL and its character was grade III meconium-stained. The patient had a forceps-assisted vaginal delivery, with estimated intrapartum blood loss of 300 mL. The first, second, and third stages of labor were 6 h 42 min, 0 h 30 min, and 0 h 8 min, respectively; the total labor duration was 7 h 20 min. An episiotomy was repaired with internal sutures. Documented comorbidities and major risk factors were ovarian cyst during pregnancy; suspected battledore placenta; high myopia; suspected fetal distress. Neonatal birth weight was 3540 g; Apgar scores at 1, 5, and 10 minutes were 10, 10, 10; and the delivery outcome was live birth. | |
| **模型生成的产褥期护理计划 Model-generated postpartum nursing care plan** | 产妇33岁，G1P1，孕39+4周，经产钳助产阴道分娩分娩，产时出血约300ml，新生儿女婴、体重3540g，Apgar评分10、10、10分。病例提示的重点风险为胎盘相关风险、胎儿窘迫或羊水异常记录。产后24小时内重点评估体温、脉搏、血压、宫底高度、宫缩、阴道流血、恶露颜色和尿潴留，记录首次排尿和疼痛评分。产后住院及出院后继续观察恶露是否由红色逐渐转淡，乳房是否胀痛，乳头是否皲裂，睡眠和情绪是否稳定。饮食以优质蛋白、含铁食物、蔬菜水果和足量饮水为主，避免盲目大补和长期卧床。本次为产钳助产阴道分娩，会阴伤口、盆底和排尿功能是护理重点。每次大小便后用温水从前向后清洁会阴并轻轻擦干，观察侧切或裂伤处肿胀、硬结、渗血、分泌物和异味。疼痛明显时可侧卧休息，避免久蹲、负重和过早剧烈活动。哺乳方面建议尽早母婴皮肤接触和按需哺乳，指导正确含接，乳胀时先热敷或轻柔按摩再哺乳，乳头疼痛时及时调整姿势。若出现乳房局部红肿硬痛、寒战或高热，应警惕乳腺炎。因有胎儿窘迫、羊水污染或新生儿评分变化记录，母婴同室期间应配合观察新生儿吃奶、肤色、体温、呼吸和反应，发现吃奶差、发绀、嗜睡或呼吸异常应立即告知儿科医生。有胎盘位置、形态或胎盘血窦相关记录时，应注意恶露持续时间和出血量，产后复查可结合B超了解宫腔及子宫复旧。合并子宫肌瘤、卵巢囊肿或多囊卵巢相关情况时，产后复查时建议结合妇科超声评估，若腹痛、包块感或异常出血加重，应提前就诊。出院前应完成用药、伤口、哺乳和避孕宣教，42天左右复查，必要时提前复诊。若出现阴道流血突然增多、鲜红血浸湿卫生巾、恶露恶臭、发热、持续腹痛、伤口化脓、排尿困难、下肢单侧肿痛、胸闷气短，或持续悲伤焦虑及伤害自己或婴儿的想法，应立即就医或急诊处理。 | 患者33岁，初产妇，产钳+侧切，合并卵巢囊肿可疑、球拍状胎盘、高度近视、可疑胎儿窘迫，羊水Ⅲ°污染，产时出血300ml，新生儿Apgar 10。Ⅲ°羊水新生儿监测MAS。高度近视避免用力。卵巢囊肿6周B超。球拍状胎盘无特殊。出血300ml正常。会阴侧切护理。42天复查B超。 |
| **护理计划英文翻译 English translation of care plan** | The patient is 33 years old, G1P1, delivered at 39+4 weeks by forceps-assisted vaginal delivery, with an estimated intrapartum blood loss of 300 mL. The newborn was a female infant, weighed 3540 g, and had Apgar scores of 10, 10, 10. The main case-based risks include placental risk factors, fetal distress or abnormal amniotic fluid findings. Within the first 24 hours, assess temperature, pulse, blood pressure, uterine fundal height, uterine tone, vaginal bleeding, lochia color, urinary retention, first voiding, and pain score. During hospitalization and after discharge, continue to observe whether lochia gradually changes from red to lighter color, whether breast engorgement or nipple fissure occurs, and whether sleep and mood remain stable. Diet should include high-quality protein, iron-rich food, vegetables, fruit, and adequate fluids. Avoid blind over-supplementation and prolonged bed rest. Because this was a forceps-assisted vaginal delivery, perineal wound care, pelvic floor recovery, and urinary function are priorities. Rinse the perineum from front to back with warm water after urination or bowel movement and pat dry. Observe the episiotomy or tear for swelling, induration, bleeding, discharge, and odor. Rest in a lateral position if pain is marked, and avoid prolonged squatting, heavy lifting, and early vigorous activity. For breastfeeding, encourage early skin-to-skin contact and feeding on demand. Teach correct latch and positioning. For engorgement, apply warmth or gentle massage before feeding. If nipple pain occurs, adjust latch promptly. Localized breast redness, hard painful swelling, chills, or high fever should raise concern for mastitis. Because fetal distress, meconium-stained fluid, or changed Apgar scores were recorded, cooperate with pediatric observation of feeding, skin color, temperature, breathing, and responsiveness. Poor feeding, cyanosis, excessive sleepiness, or abnormal breathing should be reported immediately. For abnormal placental location, shape, or placental sinus records, monitor bleeding amount and duration of lochia. Ultrasound review of the uterine cavity and involution may be needed at follow-up. For uterine fibroids, ovarian cysts, or polycystic ovary related findings, pelvic ultrasound can be considered at postpartum review. Earlier assessment is needed for worsening abdominal pain, mass sensation, or abnormal bleeding. Before discharge, provide education on medication, wound care, breastfeeding, and contraception. Arrange a postpartum review around 42 days after delivery, or earlier if symptoms occur. Seek urgent medical care for sudden heavy vaginal bleeding, bright red bleeding soaking pads, foul-smelling lochia, fever, persistent abdominal pain, pus from the wound, difficulty urinating, unilateral leg swelling or pain, chest tightness, shortness of breath, or persistent sadness, anxiety, or thoughts of harming herself or the baby. | The patient is a 33-year-old primipara with forceps delivery and episiotomy. Comorbidities: suspected ovarian cyst, battledore placenta, high myopia, suspected fetal distress, grade III meconium. Blood loss 300 mL, newborn Apgar 10. Newborn monitor for MAS. High myopia – avoid Valsalva. Ovarian cyst – ultrasound at 6 weeks. Episiotomy care. Follow-up ultrasound at 42 days. |
| **Clinical appropriateness 临床适当性** | 5 | 4 |
| **Individualization 个体化程度** | 5 | 5 |
| **Comprehensiveness 完整性** | 5 | 3 |
| **Actionability 可操作性** | 4 | 4 |
| **Safety 安全性** | 5 | 4 |
| **Triage appropriateness 分诊/转诊建议适当性** | 5 | 3 |
| **Structure and clarity 结构与清晰度** | 4 | 3 |

**病例 85 / Case 85**

| **项目 Item** | **ChatGPT 5.5** | **DeepSeek V3** |
| --- | --- | --- |
| **去标识化临床病例摘要 De-identified Clinical Case Summary** | 患者26岁，孕产史为G4P2，既往剖宫产0次，孕周39+4，临产类别为自然临产。自然破膜，羊水量约400ml，羊水性质为清。以自然分娩分娩，产时出血约190ml。第一产程3时15分，第二产程0时19分，第三产程0时16分，总产程3时50分，会阴或切口情况为Ⅰ，内缝。合并症及主要风险因素：无。新生儿出生体重3660g，Apgar评分1、5、10分钟分别为10、10、10分，分娩结局为活产。 | |
| **临床病例摘要英文翻译 English Translation of De-identified Clinical Case Summary** | Maternal age was 26 years. The obstetric history was G4P2, with 0 previous cesarean deliveries. Gestational age was 39+4 weeks, and the labor onset or induction category was spontaneous labor. The membranes ruptured spontaneously; the amniotic fluid volume was approximately 400 mL and its character was clear. The patient had a spontaneous vaginal delivery, with estimated intrapartum blood loss of 190 mL. The first, second, and third stages of labor were 3 h 15 min, 0 h 19 min, and 0 h 16 min, respectively; the total labor duration was 3 h 50 min. A first-degree perineal laceration was repaired with internal sutures. No comorbidities or major risk factors were documented. Neonatal birth weight was 3660 g; Apgar scores at 1, 5, and 10 minutes were 10, 10, 10; and the delivery outcome was live birth. | |
| **模型生成的产褥期护理计划 Model-generated postpartum nursing care plan** | 产妇26岁，G4P2，孕39+4周，经阴道自然分娩分娩，产时出血约190ml，新生儿男婴、体重3660g，Apgar评分10、10、10分。病例提示的重点风险为未记录特殊合并症。产后24小时内重点评估体温、脉搏、血压、宫底高度、宫缩、阴道流血、恶露颜色和尿潴留，记录首次排尿和疼痛评分。产后住院及出院后继续观察恶露是否由红色逐渐转淡，乳房是否胀痛，乳头是否皲裂，睡眠和情绪是否稳定。饮食以优质蛋白、含铁食物、蔬菜水果和足量饮水为主，避免盲目大补和长期卧床。本次为阴道自然分娩，应重点观察会阴裂伤或缝合处恢复。每次大小便后保持局部清洁干燥，观察红肿、渗血、疼痛加重和异常气味。无头晕和出血增多时逐步增加活动，并在医护指导下开始轻柔盆底肌训练。哺乳方面建议尽早母婴皮肤接触和按需哺乳，指导正确含接，乳胀时先热敷或轻柔按摩再哺乳，乳头疼痛时及时调整姿势。若出现乳房局部红肿硬痛、寒战或高热，应警惕乳腺炎。目前未记录特殊合并症，仍需按常规产褥期管理执行，重点是子宫复旧、恶露、伤口、哺乳、营养、活动和心理状态。出院前应完成用药、伤口、哺乳和避孕宣教，42天左右复查，必要时提前复诊。若出现阴道流血突然增多、鲜红血浸湿卫生巾、恶露恶臭、发热、持续腹痛、伤口化脓、排尿困难、下肢单侧肿痛、胸闷气短，或持续悲伤焦虑及伤害自己或婴儿的想法，应立即就医或急诊处理。 | 患者26岁，G4P2，自然分娩+I度裂伤，无合并症。常规护理。G4P2建议长效避孕。42天复查。 |
| **护理计划英文翻译 English translation of care plan** | The patient is 26 years old, G4P2, delivered at 39+4 weeks by spontaneous vaginal delivery, with an estimated intrapartum blood loss of 190 mL. The newborn was a male infant, weighed 3660 g, and had Apgar scores of 10, 10, 10. The main case-based risks include no special comorbidity was recorded. Within the first 24 hours, assess temperature, pulse, blood pressure, uterine fundal height, uterine tone, vaginal bleeding, lochia color, urinary retention, first voiding, and pain score. During hospitalization and after discharge, continue to observe whether lochia gradually changes from red to lighter color, whether breast engorgement or nipple fissure occurs, and whether sleep and mood remain stable. Diet should include high-quality protein, iron-rich food, vegetables, fruit, and adequate fluids. Avoid blind over-supplementation and prolonged bed rest. Because this was a spontaneous vaginal birth, recovery of any perineal tear or suture site should be observed. Keep the area clean and dry after urination or bowel movement and watch for redness, swelling, bleeding, worsening pain, or abnormal odor. If there is no dizziness or increased bleeding, increase activity gradually and begin gentle pelvic floor exercises under professional guidance. For breastfeeding, encourage early skin-to-skin contact and feeding on demand. Teach correct latch and positioning. For engorgement, apply warmth or gentle massage before feeding. If nipple pain occurs, adjust latch promptly. Localized breast redness, hard painful swelling, chills, or high fever should raise concern for mastitis. No special complication was recorded, but routine postpartum management should still focus on uterine involution, lochia, wounds, breastfeeding, nutrition, activity, and mental health. Before discharge, provide education on medication, wound care, breastfeeding, and contraception. Arrange a postpartum review around 42 days after delivery, or earlier if symptoms occur. Seek urgent medical care for sudden heavy vaginal bleeding, bright red bleeding soaking pads, foul-smelling lochia, fever, persistent abdominal pain, pus from the wound, difficulty urinating, unilateral leg swelling or pain, chest tightness, shortness of breath, or persistent sadness, anxiety, or thoughts of harming herself or the baby. | The patient is a 26-year-old G4P2 with spontaneous delivery and first-degree laceration. No comorbidities. Routine care. Recommend LARC for G4P2. Follow-up at 42 days. |
| **Clinical appropriateness 临床适当性** | 4 | 3 |
| **Individualization 个体化程度** | 3 | 3 |
| **Comprehensiveness 完整性** | 4 | 3 |
| **Actionability 可操作性** | 4 | 3 |
| **Safety 安全性** | 5 | 3 |
| **Triage appropriateness 分诊/转诊建议适当性** | 5 | 3 |
| **Structure and clarity 结构与清晰度** | 3 | 4 |

**病例 86 / Case 86**

| **项目 Item** | **ChatGPT 5.5** | **DeepSeek V3** |
| --- | --- | --- |
| **去标识化临床病例摘要 De-identified Clinical Case Summary** | 患者29岁，孕产史为G1P1，既往剖宫产0次，孕周41，临产类别为普贝生。自然破膜，羊水量约300ml，羊水性质为Ⅲ°。以自然分娩分娩，产时出血约250ml。第一产程4时41分，第二产程0时19分，第三产程0时5分，总产程5时5分，会阴或切口情况为Ⅰ，内缝。合并症及主要风险因素：双眼屈光不正；胎儿宫内窘迫？。新生儿出生体重3220g，Apgar评分1、5、10分钟分别为10、10、10分，分娩结局为活产。 | |
| **临床病例摘要英文翻译 English Translation of De-identified Clinical Case Summary** | Maternal age was 29 years. The obstetric history was G1P1, with 0 previous cesarean deliveries. Gestational age was 41 weeks, and the labor onset or induction category was vaginal dinoprostone (Propess) induction. The membranes ruptured spontaneously; the amniotic fluid volume was approximately 300 mL and its character was grade III meconium-stained. The patient had a spontaneous vaginal delivery, with estimated intrapartum blood loss of 250 mL. The first, second, and third stages of labor were 4 h 41 min, 0 h 19 min, and 0 h 5 min, respectively; the total labor duration was 5 h 5 min. A first-degree perineal laceration was repaired with internal sutures. Documented comorbidities and major risk factors were bilateral refractive error; suspected fetal distress. Neonatal birth weight was 3220 g; Apgar scores at 1, 5, and 10 minutes were 10, 10, 10; and the delivery outcome was live birth. | |
| **模型生成的产褥期护理计划 Model-generated postpartum nursing care plan** | 产妇29岁，G1P1，孕41周，经阴道自然分娩分娩，产时出血约250ml，新生儿男婴、体重3220g，Apgar评分10、10、10分。病例提示的重点风险为胎儿窘迫或羊水异常记录。产后24小时内重点评估体温、脉搏、血压、宫底高度、宫缩、阴道流血、恶露颜色和尿潴留，记录首次排尿和疼痛评分。产后住院及出院后继续观察恶露是否由红色逐渐转淡，乳房是否胀痛，乳头是否皲裂，睡眠和情绪是否稳定。饮食以优质蛋白、含铁食物、蔬菜水果和足量饮水为主，避免盲目大补和长期卧床。本次为阴道自然分娩，应重点观察会阴裂伤或缝合处恢复。每次大小便后保持局部清洁干燥，观察红肿、渗血、疼痛加重和异常气味。无头晕和出血增多时逐步增加活动，并在医护指导下开始轻柔盆底肌训练。哺乳方面建议尽早母婴皮肤接触和按需哺乳，指导正确含接，乳胀时先热敷或轻柔按摩再哺乳，乳头疼痛时及时调整姿势。若出现乳房局部红肿硬痛、寒战或高热，应警惕乳腺炎。因有胎儿窘迫、羊水污染或新生儿评分变化记录，母婴同室期间应配合观察新生儿吃奶、肤色、体温、呼吸和反应，发现吃奶差、发绀、嗜睡或呼吸异常应立即告知儿科医生。出院前应完成用药、伤口、哺乳和避孕宣教，42天左右复查，必要时提前复诊。若出现阴道流血突然增多、鲜红血浸湿卫生巾、恶露恶臭、发热、持续腹痛、伤口化脓、排尿困难、下肢单侧肿痛、胸闷气短，或持续悲伤焦虑及伤害自己或婴儿的想法，应立即就医或急诊处理。 | 患者29岁，初产妇，自然分娩+I度裂伤，合并双眼屈光不正、可疑胎儿窘迫，脐带绕颈1周（紧），羊水Ⅲ°污染，产时出血250ml，新生儿Apgar 10。Ⅲ°羊水+脐带绕颈紧，新生儿监测MAS及缺氧。高度近视避免用力。常规会阴护理。42天复查眼科。 |
| **护理计划英文翻译 English translation of care plan** | The patient is 29 years old, G1P1, delivered at 41 weeks by spontaneous vaginal delivery, with an estimated intrapartum blood loss of 250 mL. The newborn was a male infant, weighed 3220 g, and had Apgar scores of 10, 10, 10. The main case-based risks include fetal distress or abnormal amniotic fluid findings. Within the first 24 hours, assess temperature, pulse, blood pressure, uterine fundal height, uterine tone, vaginal bleeding, lochia color, urinary retention, first voiding, and pain score. During hospitalization and after discharge, continue to observe whether lochia gradually changes from red to lighter color, whether breast engorgement or nipple fissure occurs, and whether sleep and mood remain stable. Diet should include high-quality protein, iron-rich food, vegetables, fruit, and adequate fluids. Avoid blind over-supplementation and prolonged bed rest. Because this was a spontaneous vaginal birth, recovery of any perineal tear or suture site should be observed. Keep the area clean and dry after urination or bowel movement and watch for redness, swelling, bleeding, worsening pain, or abnormal odor. If there is no dizziness or increased bleeding, increase activity gradually and begin gentle pelvic floor exercises under professional guidance. For breastfeeding, encourage early skin-to-skin contact and feeding on demand. Teach correct latch and positioning. For engorgement, apply warmth or gentle massage before feeding. If nipple pain occurs, adjust latch promptly. Localized breast redness, hard painful swelling, chills, or high fever should raise concern for mastitis. Because fetal distress, meconium-stained fluid, or changed Apgar scores were recorded, cooperate with pediatric observation of feeding, skin color, temperature, breathing, and responsiveness. Poor feeding, cyanosis, excessive sleepiness, or abnormal breathing should be reported immediately. Before discharge, provide education on medication, wound care, breastfeeding, and contraception. Arrange a postpartum review around 42 days after delivery, or earlier if symptoms occur. Seek urgent medical care for sudden heavy vaginal bleeding, bright red bleeding soaking pads, foul-smelling lochia, fever, persistent abdominal pain, pus from the wound, difficulty urinating, unilateral leg swelling or pain, chest tightness, shortness of breath, or persistent sadness, anxiety, or thoughts of harming herself or the baby. | The patient is a 29-year-old primipara with spontaneous delivery and first-degree laceration. Comorbidities: refractive error, suspected fetal distress, tight nuchal cord 1 loop, grade III meconium. Blood loss 250 mL, newborn Apgar 10. Newborn monitor for MAS and hypoxia. High myopia – avoid Valsalva. Routine perineal care. Follow-up eye exam at 42 days. |
| **Clinical appropriateness 临床适当性** | 4 | 3 |
| **Individualization 个体化程度** | 3 | 3 |
| **Comprehensiveness 完整性** | 4 | 3 |
| **Actionability 可操作性** | 4 | 4 |
| **Safety 安全性** | 5 | 3 |
| **Triage appropriateness 分诊/转诊建议适当性** | 5 | 3 |
| **Structure and clarity 结构与清晰度** | 3 | 4 |

**病例 87 / Case 87**

| **项目 Item** | **ChatGPT 5.5** | **DeepSeek V3** |
| --- | --- | --- |
| **去标识化临床病例摘要 De-identified Clinical Case Summary** | 患者29岁，孕产史为G1P1，既往剖宫产0次，孕周41+3，临产类别为阴道放置米索。人工破膜，羊水量约50ml，羊水性质为Ⅲ°。以自然分娩分娩，产时出血约240ml。第一产程13时55分，第二产程1时26分，第三产程0时9分，总产程15时30分，会阴或切口情况为侧切，内缝。合并症及主要风险因素：胎动减少（好转）；宫缩乏力。新生儿出生体重3150g，Apgar评分1、5、10分钟分别为10、10、10分，分娩结局为活产。 | |
| **临床病例摘要英文翻译 English Translation of De-identified Clinical Case Summary** | Maternal age was 29 years. The obstetric history was G1P1, with 0 previous cesarean deliveries. Gestational age was 41+3 weeks, and the labor onset or induction category was vaginal misoprostol induction. The membranes were artificially ruptured; the amniotic fluid volume was approximately 50 mL and its character was grade III meconium-stained. The patient had a spontaneous vaginal delivery, with estimated intrapartum blood loss of 240 mL. The first, second, and third stages of labor were 13 h 55 min, 1 h 26 min, and 0 h 9 min, respectively; the total labor duration was 15 h 30 min. An episiotomy was repaired with internal sutures. Documented comorbidities and major risk factors were reduced fetal movement (improved); uterine atony. Neonatal birth weight was 3150 g; Apgar scores at 1, 5, and 10 minutes were 10, 10, 10; and the delivery outcome was live birth. | |
| **模型生成的产褥期护理计划 Model-generated postpartum nursing care plan** | 产妇29岁，G1P1，孕41+3周，经阴道自然分娩分娩，产时出血约240ml，新生儿男婴、体重3150g，Apgar评分10、10、10分。病例提示的重点风险为出血或贫血相关风险、胎儿窘迫或羊水异常记录。产后24小时内重点评估体温、脉搏、血压、宫底高度、宫缩、阴道流血、恶露颜色和尿潴留，记录首次排尿和疼痛评分。产后住院及出院后继续观察恶露是否由红色逐渐转淡，乳房是否胀痛，乳头是否皲裂，睡眠和情绪是否稳定。饮食以优质蛋白、含铁食物、蔬菜水果和足量饮水为主，避免盲目大补和长期卧床。本次为阴道自然分娩，应重点观察会阴裂伤或缝合处恢复。每次大小便后保持局部清洁干燥，观察红肿、渗血、疼痛加重和异常气味。无头晕和出血增多时逐步增加活动，并在医护指导下开始轻柔盆底肌训练。哺乳方面建议尽早母婴皮肤接触和按需哺乳，指导正确含接，乳胀时先热敷或轻柔按摩再哺乳，乳头疼痛时及时调整姿势。若出现乳房局部红肿硬痛、寒战或高热，应警惕乳腺炎。因存在出血量偏多、贫血或血小板异常相关风险，应复查血常规，关注头晕、心悸、乏力、面色苍白、恶露突然增多或较大血块，按医嘱补铁，必要时评估凝血和子宫复旧。因有胎儿窘迫、羊水污染或新生儿评分变化记录，母婴同室期间应配合观察新生儿吃奶、肤色、体温、呼吸和反应，发现吃奶差、发绀、嗜睡或呼吸异常应立即告知儿科医生。出院前应完成用药、伤口、哺乳和避孕宣教，42天左右复查，必要时提前复诊。若出现阴道流血突然增多、鲜红血浸湿卫生巾、恶露恶臭、发热、持续腹痛、伤口化脓、排尿困难、下肢单侧肿痛、胸闷气短，或持续悲伤焦虑及伤害自己或婴儿的想法，应立即就医或急诊处理。 | 患者29岁，初产妇，自然分娩+侧切，合并胎动减少（好转）、宫缩乏力，羊水Ⅲ°污染，产时出血240ml，新生儿Apgar 10。宫缩乏力需加强宫底按摩，使用缩宫素。Ⅲ°羊水新生儿监测MAS。胎动减少史已好转，但仍需新生儿观察。会阴侧切护理。42天复查。 |
| **护理计划英文翻译 English translation of care plan** | The patient is 29 years old, G1P1, delivered at 41+3 weeks by spontaneous vaginal delivery, with an estimated intrapartum blood loss of 240 mL. The newborn was a male infant, weighed 3150 g, and had Apgar scores of 10, 10, 10. The main case-based risks include bleeding or anemia-related risk, fetal distress or abnormal amniotic fluid findings. Within the first 24 hours, assess temperature, pulse, blood pressure, uterine fundal height, uterine tone, vaginal bleeding, lochia color, urinary retention, first voiding, and pain score. During hospitalization and after discharge, continue to observe whether lochia gradually changes from red to lighter color, whether breast engorgement or nipple fissure occurs, and whether sleep and mood remain stable. Diet should include high-quality protein, iron-rich food, vegetables, fruit, and adequate fluids. Avoid blind over-supplementation and prolonged bed rest. Because this was a spontaneous vaginal birth, recovery of any perineal tear or suture site should be observed. Keep the area clean and dry after urination or bowel movement and watch for redness, swelling, bleeding, worsening pain, or abnormal odor. If there is no dizziness or increased bleeding, increase activity gradually and begin gentle pelvic floor exercises under professional guidance. For breastfeeding, encourage early skin-to-skin contact and feeding on demand. Teach correct latch and positioning. For engorgement, apply warmth or gentle massage before feeding. If nipple pain occurs, adjust latch promptly. Localized breast redness, hard painful swelling, chills, or high fever should raise concern for mastitis. Because increased blood loss, anemia, or platelet abnormality is a concern, repeat a complete blood count as advised and watch for dizziness, palpitations, severe fatigue, pallor, sudden increase in lochia, or large clots. Iron supplementation, nutrition support, and assessment of uterine involution or coagulation should follow medical orders. Because fetal distress, meconium-stained fluid, or changed Apgar scores were recorded, cooperate with pediatric observation of feeding, skin color, temperature, breathing, and responsiveness. Poor feeding, cyanosis, excessive sleepiness, or abnormal breathing should be reported immediately. Before discharge, provide education on medication, wound care, breastfeeding, and contraception. Arrange a postpartum review around 42 days after delivery, or earlier if symptoms occur. Seek urgent medical care for sudden heavy vaginal bleeding, bright red bleeding soaking pads, foul-smelling lochia, fever, persistent abdominal pain, pus from the wound, difficulty urinating, unilateral leg swelling or pain, chest tightness, shortness of breath, or persistent sadness, anxiety, or thoughts of harming herself or the baby. | The patient is a 29-year-old primipara with spontaneous delivery and episiotomy. Comorbidities: decreased fetal movement (improved), uterine inertia, grade III meconium. Blood loss 240 mL, newborn Apgar 10. Uterine atony – fundal massage, oxytocin. Newborn monitor for MAS. Episiotomy care. Follow-up at 42 days. |
| **Clinical appropriateness 临床适当性** | 5 | 3 |
| **Individualization 个体化程度** | 4 | 3 |
| **Comprehensiveness 完整性** | 5 | 3 |
| **Actionability 可操作性** | 4 | 3 |
| **Safety 安全性** | 5 | 3 |
| **Triage appropriateness 分诊/转诊建议适当性** | 5 | 3 |
| **Structure and clarity 结构与清晰度** | 3 | 4 |

**病例 88 / Case 88**

| **项目 Item** | **ChatGPT 5.5** | **DeepSeek V3** |
| --- | --- | --- |
| **去标识化临床病例摘要 De-identified Clinical Case Summary** | 患者33岁，孕产史为G2P2，既往剖宫产0次，孕周40+4，临产类别为自然临产。自然破膜，羊水量约300ml，羊水性质为清。以自然分娩分娩，产时出血约220ml。第一产程6时30分，第二产程0时25分，第三产程0时5分，总产程7时0分，会阴或切口情况为Ⅰ，内缝。合并症及主要风险因素：D-二聚体升高；妊娠合并贫血（轻度）；妊娠合并宫颈息肉？。新生儿出生体重3350g，Apgar评分1、5、10分钟分别为10、10、10分，分娩结局为活产。 | |
| **临床病例摘要英文翻译 English Translation of De-identified Clinical Case Summary** | Maternal age was 33 years. The obstetric history was G2P2, with 0 previous cesarean deliveries. Gestational age was 40+4 weeks, and the labor onset or induction category was spontaneous labor. The membranes ruptured spontaneously; the amniotic fluid volume was approximately 300 mL and its character was clear. The patient had a spontaneous vaginal delivery, with estimated intrapartum blood loss of 220 mL. The first, second, and third stages of labor were 6 h 30 min, 0 h 25 min, and 0 h 5 min, respectively; the total labor duration was 7 h 0 min. A first-degree perineal laceration was repaired with internal sutures. Documented comorbidities and major risk factors were elevated D-dimer; mild anemia during pregnancy; suspected cervical polyp during pregnancy. Neonatal birth weight was 3350 g; Apgar scores at 1, 5, and 10 minutes were 10, 10, 10; and the delivery outcome was live birth. | |
| **模型生成的产褥期护理计划 Model-generated postpartum nursing care plan** | 产妇33岁，G2P2，孕40+4周，经阴道自然分娩分娩，产时出血约220ml，新生儿男婴、体重3350g，Apgar评分10、10、10分。病例提示的重点风险为出血或贫血相关风险。产后24小时内重点评估体温、脉搏、血压、宫底高度、宫缩、阴道流血、恶露颜色和尿潴留，记录首次排尿和疼痛评分。产后住院及出院后继续观察恶露是否由红色逐渐转淡，乳房是否胀痛，乳头是否皲裂，睡眠和情绪是否稳定。饮食以优质蛋白、含铁食物、蔬菜水果和足量饮水为主，避免盲目大补和长期卧床。本次为阴道自然分娩，应重点观察会阴裂伤或缝合处恢复。每次大小便后保持局部清洁干燥，观察红肿、渗血、疼痛加重和异常气味。无头晕和出血增多时逐步增加活动，并在医护指导下开始轻柔盆底肌训练。哺乳方面建议尽早母婴皮肤接触和按需哺乳，指导正确含接，乳胀时先热敷或轻柔按摩再哺乳，乳头疼痛时及时调整姿势。若出现乳房局部红肿硬痛、寒战或高热，应警惕乳腺炎。因存在出血量偏多、贫血或血小板异常相关风险，应复查血常规，关注头晕、心悸、乏力、面色苍白、恶露突然增多或较大血块，按医嘱补铁，必要时评估凝血和子宫复旧。出院前应完成用药、伤口、哺乳和避孕宣教，42天左右复查，必要时提前复诊。若出现阴道流血突然增多、鲜红血浸湿卫生巾、恶露恶臭、发热、持续腹痛、伤口化脓、排尿困难、下肢单侧肿痛、胸闷气短，或持续悲伤焦虑及伤害自己或婴儿的想法，应立即就医或急诊处理。 | 患者33岁，G2P2，自然分娩+I度裂伤，合并D-二聚体升高、轻度贫血、可疑宫颈息肉，脐带捻转40周，产时出血220ml。D-二聚体升高需评估血栓风险，鼓励尽早活动、弹力袜。贫血补充铁剂。宫颈息肉产后妇科处理。脐带捻转40周新生儿观察神经系统。常规会阴护理。42天复查血常规、D-二聚体、妇科。 |
| **护理计划英文翻译 English translation of care plan** | The patient is 33 years old, G2P2, delivered at 40+4 weeks by spontaneous vaginal delivery, with an estimated intrapartum blood loss of 220 mL. The newborn was a male infant, weighed 3350 g, and had Apgar scores of 10, 10, 10. The main case-based risks include bleeding or anemia-related risk. Within the first 24 hours, assess temperature, pulse, blood pressure, uterine fundal height, uterine tone, vaginal bleeding, lochia color, urinary retention, first voiding, and pain score. During hospitalization and after discharge, continue to observe whether lochia gradually changes from red to lighter color, whether breast engorgement or nipple fissure occurs, and whether sleep and mood remain stable. Diet should include high-quality protein, iron-rich food, vegetables, fruit, and adequate fluids. Avoid blind over-supplementation and prolonged bed rest. Because this was a spontaneous vaginal birth, recovery of any perineal tear or suture site should be observed. Keep the area clean and dry after urination or bowel movement and watch for redness, swelling, bleeding, worsening pain, or abnormal odor. If there is no dizziness or increased bleeding, increase activity gradually and begin gentle pelvic floor exercises under professional guidance. For breastfeeding, encourage early skin-to-skin contact and feeding on demand. Teach correct latch and positioning. For engorgement, apply warmth or gentle massage before feeding. If nipple pain occurs, adjust latch promptly. Localized breast redness, hard painful swelling, chills, or high fever should raise concern for mastitis. Because increased blood loss, anemia, or platelet abnormality is a concern, repeat a complete blood count as advised and watch for dizziness, palpitations, severe fatigue, pallor, sudden increase in lochia, or large clots. Iron supplementation, nutrition support, and assessment of uterine involution or coagulation should follow medical orders. Before discharge, provide education on medication, wound care, breastfeeding, and contraception. Arrange a postpartum review around 42 days after delivery, or earlier if symptoms occur. Seek urgent medical care for sudden heavy vaginal bleeding, bright red bleeding soaking pads, foul-smelling lochia, fever, persistent abdominal pain, pus from the wound, difficulty urinating, unilateral leg swelling or pain, chest tightness, shortness of breath, or persistent sadness, anxiety, or thoughts of harming herself or the baby. | The patient is a 33-year-old G2P2 with spontaneous delivery and first-degree laceration. Comorbidities: elevated D-dimer, mild anemia, possible cervical polyp, cord twisting 40 loops. Blood loss 220 mL. Thrombosis risk – early ambulation, compression stockings. Iron supplementation. Cervical polyp – gynecologic follow-up. Newborn neurological observation. Routine perineal care. Follow-up: CBC, D-dimer, gynecology at 42 days. |
| **Clinical appropriateness 临床适当性** | 4 | 4 |
| **Individualization 个体化程度** | 3 | 4 |
| **Comprehensiveness 完整性** | 4 | 3 |
| **Actionability 可操作性** | 4 | 4 |
| **Safety 安全性** | 5 | 4 |
| **Triage appropriateness 分诊/转诊建议适当性** | 5 | 3 |
| **Structure and clarity 结构与清晰度** | 4 | 4 |

**病例 89 / Case 89**

| **项目 Item** | **ChatGPT 5.5** | **DeepSeek V3** |
| --- | --- | --- |
| **去标识化临床病例摘要 De-identified Clinical Case Summary** | 患者36岁，孕产史为G2P1，既往剖宫产0次，孕周40+4，临产类别为普贝生。自然破膜，羊水量约200ml，羊水性质为清。以自然分娩分娩，产时出血约310ml。第一产程2时50分，第二产程0时20分，第三产程0时10分，总产程3时20分，会阴或切口情况为侧切，内缝。合并症及主要风险因素：胎儿宫内窘迫？；妊娠期高血压疾病：妊娠期高血压；妊娠期亚临床甲状腺功能减退症；高龄初孕妇的监督；胎膜早破。新生儿出生体重2640g，Apgar评分1、5、10分钟分别为10、10、10分，分娩结局为活产。 | |
| **临床病例摘要英文翻译 English Translation of De-identified Clinical Case Summary** | Maternal age was 36 years. The obstetric history was G2P1, with 0 previous cesarean deliveries. Gestational age was 40+4 weeks, and the labor onset or induction category was vaginal dinoprostone (Propess) induction. The membranes ruptured spontaneously; the amniotic fluid volume was approximately 200 mL and its character was clear. The patient had a spontaneous vaginal delivery, with estimated intrapartum blood loss of 310 mL. The first, second, and third stages of labor were 2 h 50 min, 0 h 20 min, and 0 h 10 min, respectively; the total labor duration was 3 h 20 min. An episiotomy was repaired with internal sutures. Documented comorbidities and major risk factors were suspected fetal distress; gestational hypertensive disorder: gestational hypertension; subclinical hypothyroidism; advanced maternal age in a primigravida; premature rupture of membranes. Neonatal birth weight was 2640 g; Apgar scores at 1, 5, and 10 minutes were 10, 10, 10; and the delivery outcome was live birth. | |
| **模型生成的产褥期护理计划 Model-generated postpartum nursing care plan** | 产妇36岁，G2P1，孕40+4周，经阴道自然分娩分娩，产时出血约310ml，新生儿女婴、体重2640g，Apgar评分10、10、10分。病例提示的重点风险为妊娠期高血压或子痫前期相关风险、胎膜早破或感染相关风险、甲状腺功能异常、胎儿窘迫或羊水异常记录、高龄妊娠因素。产后24小时内重点评估体温、脉搏、血压、宫底高度、宫缩、阴道流血、恶露颜色和尿潴留，记录首次排尿和疼痛评分。产后住院及出院后继续观察恶露是否由红色逐渐转淡，乳房是否胀痛，乳头是否皲裂，睡眠和情绪是否稳定。饮食以优质蛋白、含铁食物、蔬菜水果和足量饮水为主，避免盲目大补和长期卧床。本次为阴道自然分娩，应重点观察会阴裂伤或缝合处恢复。每次大小便后保持局部清洁干燥，观察红肿、渗血、疼痛加重和异常气味。无头晕和出血增多时逐步增加活动，并在医护指导下开始轻柔盆底肌训练。哺乳方面建议尽早母婴皮肤接触和按需哺乳，指导正确含接，乳胀时先热敷或轻柔按摩再哺乳，乳头疼痛时及时调整姿势。若出现乳房局部红肿硬痛、寒战或高热，应警惕乳腺炎。有高血压或子痫前期风险者，产后仍可能出现血压波动，应按医嘱监测血压，若持续头痛、眼花、胸闷、上腹痛、抽搐或水肿加重，应立即就医。合并甲状腺疾病者，应按内分泌医嘱继续或调整用药，不自行停药，产后复查甲状腺功能，观察心悸、怕冷、乏力、出汗和情绪异常。存在胎膜早破、B族链球菌感染、阴道炎或宫内感染疑虑时，应加强体温、下腹压痛、恶露气味、会阴或切口渗液观察，发热或恶露恶臭时及时复诊。因有胎儿窘迫、羊水污染或新生儿评分变化记录，母婴同室期间应配合观察新生儿吃奶、肤色、体温、呼吸和反应，发现吃奶差、发绀、嗜睡或呼吸异常应立即告知儿科医生。出院前应完成用药、伤口、哺乳和避孕宣教，42天左右复查，必要时提前复诊。若出现阴道流血突然增多、鲜红血浸湿卫生巾、恶露恶臭、发热、持续腹痛、伤口化脓、排尿困难、下肢单侧肿痛、胸闷气短，或持续悲伤焦虑及伤害自己或婴儿的想法，应立即就医或急诊处理。 | 患者36岁，G2P1，自然分娩+侧切，合并可疑胎儿窘迫、妊娠期高血压、亚临床甲减、高龄初产、胎膜早破，脐带绕颈1周（紧），产时出血310ml，新生儿Apgar 10。产后监测血压，继续降压治疗。亚临床甲减继续左甲状腺素。PPROM监测感染。脐带绕颈紧新生儿观察。会阴侧切护理。高龄初产心理支持。42天复查血压、甲功。 |
| **护理计划英文翻译 English translation of care plan** | The patient is 36 years old, G2P1, delivered at 40+4 weeks by spontaneous vaginal delivery, with an estimated intrapartum blood loss of 310 mL. The newborn was a female infant, weighed 2640 g, and had Apgar scores of 10, 10, 10. The main case-based risks include gestational hypertension or preeclampsia-related risk, premature rupture of membranes or infection-related risk, thyroid dysfunction, fetal distress or abnormal amniotic fluid findings, advanced maternal age. Within the first 24 hours, assess temperature, pulse, blood pressure, uterine fundal height, uterine tone, vaginal bleeding, lochia color, urinary retention, first voiding, and pain score. During hospitalization and after discharge, continue to observe whether lochia gradually changes from red to lighter color, whether breast engorgement or nipple fissure occurs, and whether sleep and mood remain stable. Diet should include high-quality protein, iron-rich food, vegetables, fruit, and adequate fluids. Avoid blind over-supplementation and prolonged bed rest. Because this was a spontaneous vaginal birth, recovery of any perineal tear or suture site should be observed. Keep the area clean and dry after urination or bowel movement and watch for redness, swelling, bleeding, worsening pain, or abnormal odor. If there is no dizziness or increased bleeding, increase activity gradually and begin gentle pelvic floor exercises under professional guidance. For breastfeeding, encourage early skin-to-skin contact and feeding on demand. Teach correct latch and positioning. For engorgement, apply warmth or gentle massage before feeding. If nipple pain occurs, adjust latch promptly. Localized breast redness, hard painful swelling, chills, or high fever should raise concern for mastitis. For hypertension or preeclampsia risk, postpartum blood pressure may still fluctuate. Continue blood pressure monitoring as advised and seek urgent care for persistent headache, visual symptoms, chest tightness, upper abdominal pain, seizure, or worsening edema. For thyroid disease, continue or adjust medication only under endocrine guidance, recheck thyroid function after delivery, and watch for palpitations, cold intolerance, fatigue, sweating, or abnormal mood changes. With premature rupture of membranes, group B streptococcus, vaginitis, or suspected intrauterine infection, monitor temperature, lower abdominal tenderness, lochia odor, and drainage from the perineal wound or incision. Return promptly for fever or foul-smelling lochia. Because fetal distress, meconium-stained fluid, or changed Apgar scores were recorded, cooperate with pediatric observation of feeding, skin color, temperature, breathing, and responsiveness. Poor feeding, cyanosis, excessive sleepiness, or abnormal breathing should be reported immediately. Before discharge, provide education on medication, wound care, breastfeeding, and contraception. Arrange a postpartum review around 42 days after delivery, or earlier if symptoms occur. Seek urgent medical care for sudden heavy vaginal bleeding, bright red bleeding soaking pads, foul-smelling lochia, fever, persistent abdominal pain, pus from the wound, difficulty urinating, unilateral leg swelling or pain, chest tightness, shortness of breath, or persistent sadness, anxiety, or thoughts of harming herself or the baby. | The patient is a 36-year-old G2P1 with spontaneous delivery and episiotomy. Comorbidities: suspected fetal distress, gestational hypertension, subclinical hypothyroidism, advanced maternal age in a primigravida, PPROM, tight nuchal cord 1 loop. Blood loss 310 mL, newborn Apgar 10. Monitor BP, continue antihypertensives. Continue levothyroxine. Monitor infection. Newborn observation. Episiotomy care. Psychological support. Follow-up: BP and thyroid at 42 days. |
| **Clinical appropriateness 临床适当性** | 5 | 4 |
| **Individualization 个体化程度** | 5 | 5 |
| **Comprehensiveness 完整性** | 5 | 3 |
| **Actionability 可操作性** | 5 | 4 |
| **Safety 安全性** | 5 | 4 |
| **Triage appropriateness 分诊/转诊建议适当性** | 5 | 3 |
| **Structure and clarity 结构与清晰度** | 4 | 3 |

**病例 90 / Case 90**

| **项目 Item** | **ChatGPT 5.5** | **DeepSeek V3** |
| --- | --- | --- |
| **去标识化临床病例摘要 De-identified Clinical Case Summary** | 患者32岁，孕产史为G1P1，既往剖宫产0次，孕周40+1，临产类别为自然临产。人工破膜，羊水量约300ml，羊水性质为清。以自然分娩分娩，产时出血约260ml。第一产程4时30分，第二产程0时38分，第三产程0时7分，总产程5时15分，会阴或切口情况为侧切，内缝。合并症及主要风险因素：胎儿宫内窘迫？；结缔组织病；双眼屈光不正；Rh血型不合（Rh阴性血）；甲状腺功能亢进症。新生儿出生体重3050g，Apgar评分1、5、10分钟分别为10、10、10分，分娩结局为活产。 | |
| **临床病例摘要英文翻译 English Translation of De-identified Clinical Case Summary** | Maternal age was 32 years. The obstetric history was G1P1, with 0 previous cesarean deliveries. Gestational age was 40+1 weeks, and the labor onset or induction category was spontaneous labor. The membranes were artificially ruptured; the amniotic fluid volume was approximately 300 mL and its character was clear. The patient had a spontaneous vaginal delivery, with estimated intrapartum blood loss of 260 mL. The first, second, and third stages of labor were 4 h 30 min, 0 h 38 min, and 0 h 7 min, respectively; the total labor duration was 5 h 15 min. An episiotomy was repaired with internal sutures. Documented comorbidities and major risk factors were suspected fetal distress; connective tissue disease; bilateral refractive error; Rh blood group incompatibility (Rh-negative blood type); hyperthyroidism. Neonatal birth weight was 3050 g; Apgar scores at 1, 5, and 10 minutes were 10, 10, 10; and the delivery outcome was live birth. | |
| **模型生成的产褥期护理计划 Model-generated postpartum nursing care plan** | 产妇32岁，G1P1，孕40+1周，经阴道自然分娩分娩，产时出血约260ml，新生儿男婴、体重3050g，Apgar评分10、10、10分。病例提示的重点风险为甲状腺功能异常、胎儿窘迫或羊水异常记录。产后24小时内重点评估体温、脉搏、血压、宫底高度、宫缩、阴道流血、恶露颜色和尿潴留，记录首次排尿和疼痛评分。产后住院及出院后继续观察恶露是否由红色逐渐转淡，乳房是否胀痛，乳头是否皲裂，睡眠和情绪是否稳定。饮食以优质蛋白、含铁食物、蔬菜水果和足量饮水为主，避免盲目大补和长期卧床。本次为阴道自然分娩，应重点观察会阴裂伤或缝合处恢复。每次大小便后保持局部清洁干燥，观察红肿、渗血、疼痛加重和异常气味。无头晕和出血增多时逐步增加活动，并在医护指导下开始轻柔盆底肌训练。哺乳方面建议尽早母婴皮肤接触和按需哺乳，指导正确含接，乳胀时先热敷或轻柔按摩再哺乳，乳头疼痛时及时调整姿势。若出现乳房局部红肿硬痛、寒战或高热，应警惕乳腺炎。合并甲状腺疾病者，应按内分泌医嘱继续或调整用药，不自行停药，产后复查甲状腺功能，观察心悸、怕冷、乏力、出汗和情绪异常。因有胎儿窘迫、羊水污染或新生儿评分变化记录，母婴同室期间应配合观察新生儿吃奶、肤色、体温、呼吸和反应，发现吃奶差、发绀、嗜睡或呼吸异常应立即告知儿科医生。如有Rh阴性血或乙肝相关记录，应核对母婴免疫预防和随访安排，避免漏查抗体、肝功能或新生儿免疫接种记录。有哮喘、心动过速或结缔组织病史者，应避免疲劳和感染诱发，出现胸闷、气短、喘息、心悸或关节症状加重时及时就医。出院前应完成用药、伤口、哺乳和避孕宣教，42天左右复查，必要时提前复诊。若出现阴道流血突然增多、鲜红血浸湿卫生巾、恶露恶臭、发热、持续腹痛、伤口化脓、排尿困难、下肢单侧肿痛、胸闷气短，或持续悲伤焦虑及伤害自己或婴儿的想法，应立即就医或急诊处理。 | 患者32岁，初产妇，自然分娩+侧切，合并可疑胎儿窘迫、结缔组织病、高度近视、Rh阴性血、甲亢，产时出血260ml，新生儿Apgar 10。Rh阴性需注射抗D免疫球蛋白。甲亢产后继续抗甲状腺药物（如丙硫氧嘧啶或甲巯咪唑），监测甲状腺功能，注意药物对哺乳的影响（PTU首选）。结缔组织病需风湿免疫科随访。高度近视避免用力。新生儿观察窘迫后遗症。会阴侧切护理。提供心理支持。42天复查甲功、风湿免疫指标。 |
| **护理计划英文翻译 English translation of care plan** | The patient is 32 years old, G1P1, delivered at 40+1 weeks by spontaneous vaginal delivery, with an estimated intrapartum blood loss of 260 mL. The newborn was a male infant, weighed 3050 g, and had Apgar scores of 10, 10, 10. The main case-based risks include thyroid dysfunction, fetal distress or abnormal amniotic fluid findings. Within the first 24 hours, assess temperature, pulse, blood pressure, uterine fundal height, uterine tone, vaginal bleeding, lochia color, urinary retention, first voiding, and pain score. During hospitalization and after discharge, continue to observe whether lochia gradually changes from red to lighter color, whether breast engorgement or nipple fissure occurs, and whether sleep and mood remain stable. Diet should include high-quality protein, iron-rich food, vegetables, fruit, and adequate fluids. Avoid blind over-supplementation and prolonged bed rest. Because this was a spontaneous vaginal birth, recovery of any perineal tear or suture site should be observed. Keep the area clean and dry after urination or bowel movement and watch for redness, swelling, bleeding, worsening pain, or abnormal odor. If there is no dizziness or increased bleeding, increase activity gradually and begin gentle pelvic floor exercises under professional guidance. For breastfeeding, encourage early skin-to-skin contact and feeding on demand. Teach correct latch and positioning. For engorgement, apply warmth or gentle massage before feeding. If nipple pain occurs, adjust latch promptly. Localized breast redness, hard painful swelling, chills, or high fever should raise concern for mastitis. For thyroid disease, continue or adjust medication only under endocrine guidance, recheck thyroid function after delivery, and watch for palpitations, cold intolerance, fatigue, sweating, or abnormal mood changes. Because fetal distress, meconium-stained fluid, or changed Apgar scores were recorded, cooperate with pediatric observation of feeding, skin color, temperature, breathing, and responsiveness. Poor feeding, cyanosis, excessive sleepiness, or abnormal breathing should be reported immediately. For Rh-negative blood type or hepatitis B related records, confirm maternal and newborn immunoprophylaxis and follow-up, and avoid missing antibody, liver function, or infant vaccination documentation. For asthma, tachycardia, or connective tissue disease, avoid fatigue and infection triggers. Chest tightness, shortness of breath, wheezing, palpitations, or worsening joint symptoms require medical review. Before discharge, provide education on medication, wound care, breastfeeding, and contraception. Arrange a postpartum review around 42 days after delivery, or earlier if symptoms occur. Seek urgent medical care for sudden heavy vaginal bleeding, bright red bleeding soaking pads, foul-smelling lochia, fever, persistent abdominal pain, pus from the wound, difficulty urinating, unilateral leg swelling or pain, chest tightness, shortness of breath, or persistent sadness, anxiety, or thoughts of harming herself or the baby. | The patient is a 32-year-old primipara with spontaneous delivery and episiotomy. Comorbidities: suspected fetal distress, connective tissue disease, high myopia, Rh-negative, hyperthyroidism. Blood loss 260 mL, newborn Apgar 10. Rh-negative – administer anti-D immunoglobulin. Hyperthyroidism – continue antithyroid drugs (PTU preferred for breastfeeding), monitor thyroid function. Connective tissue disease – rheumatology follow-up. High myopia – avoid Valsalva. Newborn observation. Episiotomy care. Psychological support. Follow-up: thyroid and rheumatology at 42 days. |
| **Clinical appropriateness 临床适当性** | 5 | 4 |
| **Individualization 个体化程度** | 5 | 5 |
| **Comprehensiveness 完整性** | 5 | 4 |
| **Actionability 可操作性** | 4 | 4 |
| **Safety 安全性** | 5 | 4 |
| **Triage appropriateness 分诊/转诊建议适当性** | 5 | 3 |
| **Structure and clarity 结构与清晰度** | 4 | 3 |

**病例 91 / Case 91**

| **项目 Item** | **ChatGPT 5.5** | **DeepSeek V3** |
| --- | --- | --- |
| **去标识化临床病例摘要 De-identified Clinical Case Summary** | 患者32岁，孕产史为G1P1，既往剖宫产1次，孕周39+1，临产类别为未临产。人工破膜，羊水量约300ml，羊水性质为清。以剖宫产分娩，产时出血约360ml。第一产程未进入产程，第二产程未进入第二产程，第三产程0时5分，总产程未进入产程，会阴或切口情况为完整。合并症及主要风险因素：臀位妊娠，择期子宫下段剖宫产。新生儿出生体重3150g，Apgar评分1、5、10分钟分别为10、10、10分，分娩结局为活产。 | |
| **临床病例摘要英文翻译 English Translation of De-identified Clinical Case Summary** | Maternal age was 32 years. The obstetric history was G1P1, with 1 previous cesarean deliveries. Gestational age was 39+1 weeks, and the labor onset or induction category was no labor. The membranes were artificially ruptured; the amniotic fluid volume was approximately 300 mL and its character was clear. The patient underwent cesarean delivery, with estimated intrapartum blood loss of 360 mL. The first, second, and third stages of labor were labor was not established, the second stage was not reached, and 0 h 5 min, respectively; the total labor duration was labor was not established. The perineum was intact. Documented comorbidities and major risk factors were breech pregnancy, elective lower-segment cesarean section. Neonatal birth weight was 3150 g; Apgar scores at 1, 5, and 10 minutes were 10, 10, 10; and the delivery outcome was live birth. | |
| **模型生成的产褥期护理计划 Model-generated postpartum nursing care plan** | 产妇32岁，G1P1，孕39+1周，经剖宫产分娩，产时出血约360ml，新生儿男婴、体重3150g，Apgar评分10、10、10分。病例提示的重点风险为未记录特殊合并症。产后24小时内重点评估体温、脉搏、血压、宫底高度、宫缩、阴道流血、恶露颜色和尿潴留，记录首次排尿和疼痛评分。产后住院及出院后继续观察恶露是否由红色逐渐转淡，乳房是否胀痛，乳头是否皲裂，睡眠和情绪是否稳定。饮食以优质蛋白、含铁食物、蔬菜水果和足量饮水为主，避免盲目大补和长期卧床。本次为剖宫产，腹部切口和血栓预防是护理重点。切口应保持清洁干燥，观察红肿、渗血、渗液、裂开、异味和疼痛加重。翻身、咳嗽和下床时保护切口，在生命体征平稳后尽早床旁活动，关注肠鸣音恢复、排气排便、尿潴留和下肢肿痛。哺乳方面建议尽早母婴皮肤接触和按需哺乳，指导正确含接，乳胀时先热敷或轻柔按摩再哺乳，乳头疼痛时及时调整姿势。若出现乳房局部红肿硬痛、寒战或高热，应警惕乳腺炎。目前未记录特殊合并症，仍需按常规产褥期管理执行，重点是子宫复旧、恶露、伤口、哺乳、营养、活动和心理状态。出院前应完成用药、伤口、哺乳和避孕宣教，42天左右复查，必要时提前复诊。若出现阴道流血突然增多、鲜红血浸湿卫生巾、恶露恶臭、发热、持续腹痛、伤口化脓、排尿困难、下肢单侧肿痛、胸闷气短，或持续悲伤焦虑及伤害自己或婴儿的想法，应立即就医或急诊处理。 | 患者32岁，G1P1，剖宫产（择期），合并臀位妊娠，产时出血360ml。剖宫产术后护理：观察腹部切口有无红、肿、渗液，每日更换敷料；术后尽早下床活动预防血栓；观察恶露量，剖宫产恶露较阴道分娩少但持续时间相似；鼓励母乳喂养，采取侧卧或足球式抱姿避免压迫切口；疼痛管理可用对乙酰氨基酚或布洛芬（哺乳安全）；术后6周内避免提重物及性生活；新生儿臀位无特殊。42天复查。 |
| **护理计划英文翻译 English translation of care plan** | The patient is 32 years old, G1P1, delivered at 39+1 weeks by cesarean delivery, with an estimated intrapartum blood loss of 360 mL. The newborn was a male infant, weighed 3150 g, and had Apgar scores of 10, 10, 10. The main case-based risks include no special comorbidity was recorded. Within the first 24 hours, assess temperature, pulse, blood pressure, uterine fundal height, uterine tone, vaginal bleeding, lochia color, urinary retention, first voiding, and pain score. During hospitalization and after discharge, continue to observe whether lochia gradually changes from red to lighter color, whether breast engorgement or nipple fissure occurs, and whether sleep and mood remain stable. Diet should include high-quality protein, iron-rich food, vegetables, fruit, and adequate fluids. Avoid blind over-supplementation and prolonged bed rest. Because this was a cesarean delivery, abdominal incision care and thrombosis prevention are key. Keep the incision clean and dry and observe for redness, swelling, bleeding, drainage, separation, odor, or worsening pain. Support the incision when turning, coughing, or getting out of bed. Once vital signs are stable, begin assisted ambulation and monitor bowel sounds, passage of gas, bowel movement, urinary retention, and leg swelling or pain. For breastfeeding, encourage early skin-to-skin contact and feeding on demand. Teach correct latch and positioning. For engorgement, apply warmth or gentle massage before feeding. If nipple pain occurs, adjust latch promptly. Localized breast redness, hard painful swelling, chills, or high fever should raise concern for mastitis. No special complication was recorded, but routine postpartum management should still focus on uterine involution, lochia, wounds, breastfeeding, nutrition, activity, and mental health. Before discharge, provide education on medication, wound care, breastfeeding, and contraception. Arrange a postpartum review around 42 days after delivery, or earlier if symptoms occur. Seek urgent medical care for sudden heavy vaginal bleeding, bright red bleeding soaking pads, foul-smelling lochia, fever, persistent abdominal pain, pus from the wound, difficulty urinating, unilateral leg swelling or pain, chest tightness, shortness of breath, or persistent sadness, anxiety, or thoughts of harming herself or the baby. | The patient is a 32-year-old G1P1 who underwent elective cesarean section for breech presentation. Blood loss 360 mL. Post-cesarean care: monitor abdominal incision for redness, swelling, drainage; change dressing daily. Early ambulation to prevent thrombosis. Monitor lochia (typically lighter than vaginal delivery). Encourage breastfeeding – side-lying or football hold to avoid pressure on incision. Pain management with acetaminophen or ibuprofen (safe for breastfeeding). Avoid heavy lifting and intercourse for 6 weeks. Newborn – no special issues from breech. Follow-up at 42 days. |
| **Clinical appropriateness 临床适当性** | 4 | 4 |
| **Individualization 个体化程度** | 3 | 4 |
| **Comprehensiveness 完整性** | 4 | 4 |
| **Actionability 可操作性** | 4 | 4 |
| **Safety 安全性** | 5 | 4 |
| **Triage appropriateness 分诊/转诊建议适当性** | 5 | 3 |
| **Structure and clarity 结构与清晰度** | 4 | 4 |

**病例 92 / Case 92**

| **项目 Item** | **ChatGPT 5.5** | **DeepSeek V3** |
| --- | --- | --- |
| **去标识化临床病例摘要 De-identified Clinical Case Summary** | 患者35岁，孕产史为G2P2，既往剖宫产2次，孕周39+3，临产类别为未临产。人工破膜，羊水量约250ml，羊水性质为清。以剖宫产分娩，产时出血约420ml。第一产程未进入产程，第二产程未进入第二产程，第三产程0时6分，总产程未进入产程，会阴或切口情况为完整。合并症及主要风险因素：瘢痕子宫，前次剖宫产术后。新生儿出生体重3380g，Apgar评分1、5、10分钟分别为10、10、10分，分娩结局为活产。 | |
| **临床病例摘要英文翻译 English Translation of De-identified Clinical Case Summary** | Maternal age was 35 years. The obstetric history was G2P2, with 2 previous cesarean deliveries. Gestational age was 39+3 weeks, and the labor onset or induction category was no labor. The membranes were artificially ruptured; the amniotic fluid volume was approximately 250 mL and its character was clear. The patient underwent cesarean delivery, with estimated intrapartum blood loss of 420 mL. The first, second, and third stages of labor were labor was not established, the second stage was not reached, and 0 h 6 min, respectively; the total labor duration was labor was not established. The perineum was intact. Documented comorbidities and major risk factors were scarred uterus, history of previous cesarean delivery. Neonatal birth weight was 3380 g; Apgar scores at 1, 5, and 10 minutes were 10, 10, 10; and the delivery outcome was live birth. | |
| **模型生成的产褥期护理计划 Model-generated postpartum nursing care plan** | 产妇35岁，G2P2，孕39+3周，经剖宫产分娩，产时出血约420ml，新生儿女婴、体重3380g，Apgar评分10、10、10分。病例提示的重点风险为高龄妊娠因素。产后24小时内重点评估体温、脉搏、血压、宫底高度、宫缩、阴道流血、恶露颜色和尿潴留，记录首次排尿和疼痛评分。产后住院及出院后继续观察恶露是否由红色逐渐转淡，乳房是否胀痛，乳头是否皲裂，睡眠和情绪是否稳定。饮食以优质蛋白、含铁食物、蔬菜水果和足量饮水为主，避免盲目大补和长期卧床。本次为剖宫产，腹部切口和血栓预防是护理重点。切口应保持清洁干燥，观察红肿、渗血、渗液、裂开、异味和疼痛加重。翻身、咳嗽和下床时保护切口，在生命体征平稳后尽早床旁活动，关注肠鸣音恢复、排气排便、尿潴留和下肢肿痛。哺乳方面建议尽早母婴皮肤接触和按需哺乳，指导正确含接，乳胀时先热敷或轻柔按摩再哺乳，乳头疼痛时及时调整姿势。若出现乳房局部红肿硬痛、寒战或高热，应警惕乳腺炎。产时出血量需要持续观察，建议每日记录恶露量、颜色和血块情况，活动后若明显增多，应减少活动并请医护评估。瘢痕子宫或再次剖宫产者，应特别关注切口疼痛、恶露异常和子宫复旧，产后复查时结合病情评估子宫切口恢复。高龄产妇恢复期应避免过度劳累和连续熬夜，家属应参与夜间照护，保证睡眠、营养和情绪支持。出院前应完成用药、伤口、哺乳和避孕宣教，42天左右复查，必要时提前复诊。若出现阴道流血突然增多、鲜红血浸湿卫生巾、恶露恶臭、发热、持续腹痛、伤口化脓、排尿困难、下肢单侧肿痛、胸闷气短，或持续悲伤焦虑及伤害自己或婴儿的想法，应立即就医或急诊处理。 | 患者35岁，G2P2，剖宫产（择期），合并瘢痕子宫（前次剖宫产），产时出血420ml。瘢痕子宫剖宫产术后护理同常规剖宫产，但需注意子宫切口愈合，观察有无异常腹痛、阴道出血增多。术后尽早活动。鼓励母乳喂养。产后6周内避免重体力劳动。避孕建议：因已两次剖宫产，建议长效可逆避孕或永久性避孕。42天复查。 |
| **护理计划英文翻译 English translation of care plan** | The patient is 35 years old, G2P2, delivered at 39+3 weeks by cesarean delivery, with an estimated intrapartum blood loss of 420 mL. The newborn was a female infant, weighed 3380 g, and had Apgar scores of 10, 10, 10. The main case-based risks include advanced maternal age. Within the first 24 hours, assess temperature, pulse, blood pressure, uterine fundal height, uterine tone, vaginal bleeding, lochia color, urinary retention, first voiding, and pain score. During hospitalization and after discharge, continue to observe whether lochia gradually changes from red to lighter color, whether breast engorgement or nipple fissure occurs, and whether sleep and mood remain stable. Diet should include high-quality protein, iron-rich food, vegetables, fruit, and adequate fluids. Avoid blind over-supplementation and prolonged bed rest. Because this was a cesarean delivery, abdominal incision care and thrombosis prevention are key. Keep the incision clean and dry and observe for redness, swelling, bleeding, drainage, separation, odor, or worsening pain. Support the incision when turning, coughing, or getting out of bed. Once vital signs are stable, begin assisted ambulation and monitor bowel sounds, passage of gas, bowel movement, urinary retention, and leg swelling or pain. For breastfeeding, encourage early skin-to-skin contact and feeding on demand. Teach correct latch and positioning. For engorgement, apply warmth or gentle massage before feeding. If nipple pain occurs, adjust latch promptly. Localized breast redness, hard painful swelling, chills, or high fever should raise concern for mastitis. Because blood loss requires closer observation, record the amount, color, and clots in the lochia every day. If bleeding increases clearly after activity, reduce activity and seek clinical assessment. For scarred uterus or repeat cesarean delivery, pay close attention to incision pain, abnormal lochia, and uterine involution. Uterine scar recovery should be reviewed during postpartum follow-up when indicated. Advanced maternal age may slow recovery, so the family should help with nighttime care and support adequate sleep, nutrition, and emotional stability. Before discharge, provide education on medication, wound care, breastfeeding, and contraception. Arrange a postpartum review around 42 days after delivery, or earlier if symptoms occur. Seek urgent medical care for sudden heavy vaginal bleeding, bright red bleeding soaking pads, foul-smelling lochia, fever, persistent abdominal pain, pus from the wound, difficulty urinating, unilateral leg swelling or pain, chest tightness, shortness of breath, or persistent sadness, anxiety, or thoughts of harming herself or the baby. | The patient is a 35-year-old G2P2 who underwent repeat cesarean section for scarred uterus. Blood loss 420 mL. Post-cesarean care: monitor for abnormal abdominal pain or increased bleeding due to uterine scar. Early ambulation. Encourage breastfeeding. Avoid heavy lifting for 6 weeks. Contraception: recommend LARC or permanent method after two cesareans. Follow-up at 42 days. |
| **Clinical appropriateness 临床适当性** | 5 | 4 |
| **Individualization 个体化程度** | 4 | 4 |
| **Comprehensiveness 完整性** | 5 | 3 |
| **Actionability 可操作性** | 4 | 4 |
| **Safety 安全性** | 5 | 4 |
| **Triage appropriateness 分诊/转诊建议适当性** | 5 | 3 |
| **Structure and clarity 结构与清晰度** | 4 | 4 |

**病例 93 / Case 93**

| **项目 Item** | **ChatGPT 5.5** | **DeepSeek V3** |
| --- | --- | --- |
| **去标识化临床病例摘要 De-identified Clinical Case Summary** | 患者29岁，孕产史为G1P1，既往剖宫产1次，孕周40+2，临产类别为自然临产。自然破膜，羊水量约200ml，羊水性质为Ⅲ°。以剖宫产分娩，产时出血约480ml。第一产程5时10分，第二产程未进入第二产程，第三产程0时5分，总产程5时15分，会阴或切口情况为完整。合并症及主要风险因素：胎儿宫内窘迫？羊水Ⅲ°污染，急诊剖宫产。新生儿出生体重3260g，Apgar评分1、5、10分钟分别为8、9、10分，分娩结局为活产。 | |
| **临床病例摘要英文翻译 English Translation of De-identified Clinical Case Summary** | Maternal age was 29 years. The obstetric history was G1P1, with 1 previous cesarean deliveries. Gestational age was 40+2 weeks, and the labor onset or induction category was spontaneous labor. The membranes ruptured spontaneously; the amniotic fluid volume was approximately 200 mL and its character was grade III meconium-stained. The patient underwent cesarean delivery, with estimated intrapartum blood loss of 480 mL. The first, second, and third stages of labor were 5 h 10 min, the second stage was not reached, and 0 h 5 min, respectively; the total labor duration was 5 h 15 min. The perineum was intact. Documented comorbidities and major risk factors were suspected fetal distress; grade III meconium-stained amniotic fluid, emergency cesarean section. Neonatal birth weight was 3260 g; Apgar scores at 1, 5, and 10 minutes were 8, 9, 10; and the delivery outcome was live birth. | |
| **模型生成的产褥期护理计划 Model-generated postpartum nursing care plan** | 产妇29岁，G1P1，孕40+2周，经剖宫产分娩，产时出血约480ml，新生儿男婴、体重3260g，Apgar评分8、9、10分。病例提示的重点风险为胎儿窘迫或羊水异常记录。产后24小时内重点评估体温、脉搏、血压、宫底高度、宫缩、阴道流血、恶露颜色和尿潴留，记录首次排尿和疼痛评分。产后住院及出院后继续观察恶露是否由红色逐渐转淡，乳房是否胀痛，乳头是否皲裂，睡眠和情绪是否稳定。饮食以优质蛋白、含铁食物、蔬菜水果和足量饮水为主，避免盲目大补和长期卧床。本次为剖宫产，腹部切口和血栓预防是护理重点。切口应保持清洁干燥，观察红肿、渗血、渗液、裂开、异味和疼痛加重。翻身、咳嗽和下床时保护切口，在生命体征平稳后尽早床旁活动，关注肠鸣音恢复、排气排便、尿潴留和下肢肿痛。哺乳方面建议尽早母婴皮肤接触和按需哺乳，指导正确含接，乳胀时先热敷或轻柔按摩再哺乳，乳头疼痛时及时调整姿势。若出现乳房局部红肿硬痛、寒战或高热，应警惕乳腺炎。产时出血量需要持续观察，建议每日记录恶露量、颜色和血块情况，活动后若明显增多，应减少活动并请医护评估。因有胎儿窘迫、羊水污染或新生儿评分变化记录，母婴同室期间应配合观察新生儿吃奶、肤色、体温、呼吸和反应，发现吃奶差、发绀、嗜睡或呼吸异常应立即告知儿科医生。出院前应完成用药、伤口、哺乳和避孕宣教，42天左右复查，必要时提前复诊。若出现阴道流血突然增多、鲜红血浸湿卫生巾、恶露恶臭、发热、持续腹痛、伤口化脓、排尿困难、下肢单侧肿痛、胸闷气短，或持续悲伤焦虑及伤害自己或婴儿的想法，应立即就医或急诊处理。 | 患者29岁，G1P1，急诊剖宫产，合并可疑胎儿窘迫、羊水Ⅲ°污染，产时出血480ml，新生儿Apgar 8-9-10。Ⅲ°羊水污染新生儿需监测胎粪吸入综合征及缺氧缺血性脑病，建议新生儿科观察。剖宫产术后常规护理：观察切口、恶露、活动。出血480ml注意晚期出血，监测血红蛋白。可疑窘迫虽已分娩，仍需关注新生儿神经系统。提供心理支持。产后1周复查血常规；新生儿专科随访。 |
| **护理计划英文翻译 English translation of care plan** | The patient is 29 years old, G1P1, delivered at 40+2 weeks by cesarean delivery, with an estimated intrapartum blood loss of 480 mL. The newborn was a male infant, weighed 3260 g, and had Apgar scores of 8, 9, 10. The main case-based risks include fetal distress or abnormal amniotic fluid findings. Within the first 24 hours, assess temperature, pulse, blood pressure, uterine fundal height, uterine tone, vaginal bleeding, lochia color, urinary retention, first voiding, and pain score. During hospitalization and after discharge, continue to observe whether lochia gradually changes from red to lighter color, whether breast engorgement or nipple fissure occurs, and whether sleep and mood remain stable. Diet should include high-quality protein, iron-rich food, vegetables, fruit, and adequate fluids. Avoid blind over-supplementation and prolonged bed rest. Because this was a cesarean delivery, abdominal incision care and thrombosis prevention are key. Keep the incision clean and dry and observe for redness, swelling, bleeding, drainage, separation, odor, or worsening pain. Support the incision when turning, coughing, or getting out of bed. Once vital signs are stable, begin assisted ambulation and monitor bowel sounds, passage of gas, bowel movement, urinary retention, and leg swelling or pain. For breastfeeding, encourage early skin-to-skin contact and feeding on demand. Teach correct latch and positioning. For engorgement, apply warmth or gentle massage before feeding. If nipple pain occurs, adjust latch promptly. Localized breast redness, hard painful swelling, chills, or high fever should raise concern for mastitis. Because blood loss requires closer observation, record the amount, color, and clots in the lochia every day. If bleeding increases clearly after activity, reduce activity and seek clinical assessment. Because fetal distress, meconium-stained fluid, or changed Apgar scores were recorded, cooperate with pediatric observation of feeding, skin color, temperature, breathing, and responsiveness. Poor feeding, cyanosis, excessive sleepiness, or abnormal breathing should be reported immediately. Before discharge, provide education on medication, wound care, breastfeeding, and contraception. Arrange a postpartum review around 42 days after delivery, or earlier if symptoms occur. Seek urgent medical care for sudden heavy vaginal bleeding, bright red bleeding soaking pads, foul-smelling lochia, fever, persistent abdominal pain, pus from the wound, difficulty urinating, unilateral leg swelling or pain, chest tightness, shortness of breath, or persistent sadness, anxiety, or thoughts of harming herself or the baby. | The patient is a 29-year-old G1P1 who underwent emergency cesarean section for suspected fetal distress and grade III meconium. Blood loss 480 mL, newborn Apgar 8-9-10. Newborn – monitor for MAS and HIE; pediatric observation recommended. Routine post-cesarean care: incision, lochia, ambulation. Watch for late PPH, monitor Hb. Psychological support. Follow-up: CBC at 1 week; newborn specialist follow-up. |
| **Clinical appropriateness 临床适当性** | 5 | 4 |
| **Individualization 个体化程度** | 4 | 5 |
| **Comprehensiveness 完整性** | 5 | 4 |
| **Actionability 可操作性** | 4 | 4 |
| **Safety 安全性** | 5 | 4 |
| **Triage appropriateness 分诊/转诊建议适当性** | 5 | 4 |
| **Structure and clarity 结构与清晰度** | 4 | 4 |

**病例 94 / Case 94**

| **项目 Item** | **ChatGPT 5.5** | **DeepSeek V3** |
| --- | --- | --- |
| **去标识化临床病例摘要 De-identified Clinical Case Summary** | 患者31岁，孕产史为G1P1，既往剖宫产1次，孕周38+5，临产类别为未临产。人工破膜，羊水量约300ml，羊水性质为清。以剖宫产分娩，产时出血约350ml。第一产程未进入产程，第二产程未进入第二产程，第三产程0时5分，总产程未进入产程，会阴或切口情况为完整。合并症及主要风险因素：单臀先露，胎位异常。新生儿出生体重2960g，Apgar评分1、5、10分钟分别为10、10、10分，分娩结局为活产。 | |
| **临床病例摘要英文翻译 English Translation of De-identified Clinical Case Summary** | Maternal age was 31 years. The obstetric history was G1P1, with 1 previous cesarean deliveries. Gestational age was 38+5 weeks, and the labor onset or induction category was no labor. The membranes were artificially ruptured; the amniotic fluid volume was approximately 300 mL and its character was clear. The patient underwent cesarean delivery, with estimated intrapartum blood loss of 350 mL. The first, second, and third stages of labor were labor was not established, the second stage was not reached, and 0 h 5 min, respectively; the total labor duration was labor was not established. The perineum was intact. Documented comorbidities and major risk factors were frank breech presentation, malpresentation. Neonatal birth weight was 2960 g; Apgar scores at 1, 5, and 10 minutes were 10, 10, 10; and the delivery outcome was live birth. | |
| **模型生成的产褥期护理计划 Model-generated postpartum nursing care plan** | 产妇31岁，G1P1，孕38+5周，经剖宫产分娩，产时出血约350ml，新生儿女婴、体重2960g，Apgar评分10、10、10分。病例提示的重点风险为未记录特殊合并症。产后24小时内重点评估体温、脉搏、血压、宫底高度、宫缩、阴道流血、恶露颜色和尿潴留，记录首次排尿和疼痛评分。产后住院及出院后继续观察恶露是否由红色逐渐转淡，乳房是否胀痛，乳头是否皲裂，睡眠和情绪是否稳定。饮食以优质蛋白、含铁食物、蔬菜水果和足量饮水为主，避免盲目大补和长期卧床。本次为剖宫产，腹部切口和血栓预防是护理重点。切口应保持清洁干燥，观察红肿、渗血、渗液、裂开、异味和疼痛加重。翻身、咳嗽和下床时保护切口，在生命体征平稳后尽早床旁活动，关注肠鸣音恢复、排气排便、尿潴留和下肢肿痛。哺乳方面建议尽早母婴皮肤接触和按需哺乳，指导正确含接，乳胀时先热敷或轻柔按摩再哺乳，乳头疼痛时及时调整姿势。若出现乳房局部红肿硬痛、寒战或高热，应警惕乳腺炎。目前未记录特殊合并症，仍需按常规产褥期管理执行，重点是子宫复旧、恶露、伤口、哺乳、营养、活动和心理状态。出院前应完成用药、伤口、哺乳和避孕宣教，42天左右复查，必要时提前复诊。若出现阴道流血突然增多、鲜红血浸湿卫生巾、恶露恶臭、发热、持续腹痛、伤口化脓、排尿困难、下肢单侧肿痛、胸闷气短，或持续悲伤焦虑及伤害自己或婴儿的想法，应立即就医或急诊处理。 | 患者31岁，G1P1，剖宫产（择期），合并单臀先露、胎位异常，产时出血350ml。剖宫产术后常规护理。单臀先露新生儿需注意髋关节发育不良风险，建议儿科行髋关节超声筛查。其余同常规剖宫产护理。42天复查。 |
| **护理计划英文翻译 English translation of care plan** | The patient is 31 years old, G1P1, delivered at 38+5 weeks by cesarean delivery, with an estimated intrapartum blood loss of 350 mL. The newborn was a female infant, weighed 2960 g, and had Apgar scores of 10, 10, 10. The main case-based risks include no special comorbidity was recorded. Within the first 24 hours, assess temperature, pulse, blood pressure, uterine fundal height, uterine tone, vaginal bleeding, lochia color, urinary retention, first voiding, and pain score. During hospitalization and after discharge, continue to observe whether lochia gradually changes from red to lighter color, whether breast engorgement or nipple fissure occurs, and whether sleep and mood remain stable. Diet should include high-quality protein, iron-rich food, vegetables, fruit, and adequate fluids. Avoid blind over-supplementation and prolonged bed rest. Because this was a cesarean delivery, abdominal incision care and thrombosis prevention are key. Keep the incision clean and dry and observe for redness, swelling, bleeding, drainage, separation, odor, or worsening pain. Support the incision when turning, coughing, or getting out of bed. Once vital signs are stable, begin assisted ambulation and monitor bowel sounds, passage of gas, bowel movement, urinary retention, and leg swelling or pain. For breastfeeding, encourage early skin-to-skin contact and feeding on demand. Teach correct latch and positioning. For engorgement, apply warmth or gentle massage before feeding. If nipple pain occurs, adjust latch promptly. Localized breast redness, hard painful swelling, chills, or high fever should raise concern for mastitis. No special complication was recorded, but routine postpartum management should still focus on uterine involution, lochia, wounds, breastfeeding, nutrition, activity, and mental health. Before discharge, provide education on medication, wound care, breastfeeding, and contraception. Arrange a postpartum review around 42 days after delivery, or earlier if symptoms occur. Seek urgent medical care for sudden heavy vaginal bleeding, bright red bleeding soaking pads, foul-smelling lochia, fever, persistent abdominal pain, pus from the wound, difficulty urinating, unilateral leg swelling or pain, chest tightness, shortness of breath, or persistent sadness, anxiety, or thoughts of harming herself or the baby. | The patient is a 31-year-old G1P1 who underwent cesarean section for frank breech presentation. Blood loss 350 mL. Routine post-cesarean care. Newborn – monitor for developmental dysplasia of the hip (DDH); recommend hip ultrasound screening. Follow-up at 42 days. |
| **Clinical appropriateness 临床适当性** | 4 | 3 |
| **Individualization 个体化程度** | 3 | 3 |
| **Comprehensiveness 完整性** | 4 | 3 |
| **Actionability 可操作性** | 4 | 3 |
| **Safety 安全性** | 5 | 3 |
| **Triage appropriateness 分诊/转诊建议适当性** | 5 | 3 |
| **Structure and clarity 结构与清晰度** | 3 | 4 |

**病例 95 / Case 95**

| **项目 Item** | **ChatGPT 5.5** | **DeepSeek V3** |
| --- | --- | --- |
| **去标识化临床病例摘要 De-identified Clinical Case Summary** | 患者37岁，孕产史为G3P2，既往剖宫产1次，孕周37+2，临产类别为未临产。人工破膜，羊水量约150ml，羊水性质为血性。以剖宫产分娩，产时出血约820ml。第一产程未进入产程，第二产程未进入第二产程，第三产程0时4分，总产程未进入产程，会阴或切口情况为完整。合并症及主要风险因素：中央性前置胎盘；高龄经产妇妊娠监督。新生儿出生体重2850g，Apgar评分1、5、10分钟分别为9、10、10分，分娩结局为活产。 | |
| **临床病例摘要英文翻译 English Translation of De-identified Clinical Case Summary** | Maternal age was 37 years. The obstetric history was G3P2, with 1 previous cesarean deliveries. Gestational age was 37+2 weeks, and the labor onset or induction category was no labor. The membranes were artificially ruptured; the amniotic fluid volume was approximately 150 mL and its character was blood-stained. The patient underwent cesarean delivery, with estimated intrapartum blood loss of 820 mL. The first, second, and third stages of labor were labor was not established, the second stage was not reached, and 0 h 4 min, respectively; the total labor duration was labor was not established. The perineum was intact. Documented comorbidities and major risk factors were complete placenta previa; advanced maternal age in a multipara. Neonatal birth weight was 2850 g; Apgar scores at 1, 5, and 10 minutes were 9, 10, 10; and the delivery outcome was live birth. | |
| **模型生成的产褥期护理计划 Model-generated postpartum nursing care plan** | 产妇37岁，G3P2，孕37+2周，经剖宫产分娩，产时出血约820ml，新生儿男婴、体重2850g，Apgar评分9、10、10分。病例提示的重点风险为出血或贫血相关风险、胎盘相关风险、胎儿窘迫或羊水异常记录、高龄妊娠因素。产后24小时内重点评估体温、脉搏、血压、宫底高度、宫缩、阴道流血、恶露颜色和尿潴留，记录首次排尿和疼痛评分。产后住院及出院后继续观察恶露是否由红色逐渐转淡，乳房是否胀痛，乳头是否皲裂，睡眠和情绪是否稳定。饮食以优质蛋白、含铁食物、蔬菜水果和足量饮水为主，避免盲目大补和长期卧床。本次为剖宫产，腹部切口和血栓预防是护理重点。切口应保持清洁干燥，观察红肿、渗血、渗液、裂开、异味和疼痛加重。翻身、咳嗽和下床时保护切口，在生命体征平稳后尽早床旁活动，关注肠鸣音恢复、排气排便、尿潴留和下肢肿痛。哺乳方面建议尽早母婴皮肤接触和按需哺乳，指导正确含接，乳胀时先热敷或轻柔按摩再哺乳，乳头疼痛时及时调整姿势。若出现乳房局部红肿硬痛、寒战或高热，应警惕乳腺炎。因存在出血量偏多、贫血或血小板异常相关风险，应复查血常规，关注头晕、心悸、乏力、面色苍白、恶露突然增多或较大血块，按医嘱补铁，必要时评估凝血和子宫复旧。因有胎儿窘迫、羊水污染或新生儿评分变化记录，母婴同室期间应配合观察新生儿吃奶、肤色、体温、呼吸和反应，发现吃奶差、发绀、嗜睡或呼吸异常应立即告知儿科医生。有胎盘位置、形态或胎盘血窦相关记录时，应注意恶露持续时间和出血量，产后复查可结合B超了解宫腔及子宫复旧。高龄产妇恢复期应避免过度劳累和连续熬夜，家属应参与夜间照护，保证睡眠、营养和情绪支持。出院前应完成用药、伤口、哺乳和避孕宣教，42天左右复查，必要时提前复诊。若出现阴道流血突然增多、鲜红血浸湿卫生巾、恶露恶臭、发热、持续腹痛、伤口化脓、排尿困难、下肢单侧肿痛、胸闷气短，或持续悲伤焦虑及伤害自己或婴儿的想法，应立即就医或急诊处理。 | 患者37岁，G3P2，剖宫产（急诊），合并中央性前置胎盘、高龄经产妇，产时出血820ml（产后出血），新生儿Apgar 9-10-10。前置胎盘剖宫产出血风险高，需住院监测血红蛋白，必要时输血；出院后补充铁剂，观察晚期出血。中央性前置胎盘需注意胎盘植入可能，产后B超复查。高龄经产妇注意血栓预防。术后常规护理。提供心理支持。产后1周复查血常规；6周B超。 |
| **护理计划英文翻译 English translation of care plan** | The patient is 37 years old, G3P2, delivered at 37+2 weeks by cesarean delivery, with an estimated intrapartum blood loss of 820 mL. The newborn was a male infant, weighed 2850 g, and had Apgar scores of 9, 10, 10. The main case-based risks include bleeding or anemia-related risk, placental risk factors, fetal distress or abnormal amniotic fluid findings, advanced maternal age. Within the first 24 hours, assess temperature, pulse, blood pressure, uterine fundal height, uterine tone, vaginal bleeding, lochia color, urinary retention, first voiding, and pain score. During hospitalization and after discharge, continue to observe whether lochia gradually changes from red to lighter color, whether breast engorgement or nipple fissure occurs, and whether sleep and mood remain stable. Diet should include high-quality protein, iron-rich food, vegetables, fruit, and adequate fluids. Avoid blind over-supplementation and prolonged bed rest. Because this was a cesarean delivery, abdominal incision care and thrombosis prevention are key. Keep the incision clean and dry and observe for redness, swelling, bleeding, drainage, separation, odor, or worsening pain. Support the incision when turning, coughing, or getting out of bed. Once vital signs are stable, begin assisted ambulation and monitor bowel sounds, passage of gas, bowel movement, urinary retention, and leg swelling or pain. For breastfeeding, encourage early skin-to-skin contact and feeding on demand. Teach correct latch and positioning. For engorgement, apply warmth or gentle massage before feeding. If nipple pain occurs, adjust latch promptly. Localized breast redness, hard painful swelling, chills, or high fever should raise concern for mastitis. Because increased blood loss, anemia, or platelet abnormality is a concern, repeat a complete blood count as advised and watch for dizziness, palpitations, severe fatigue, pallor, sudden increase in lochia, or large clots. Iron supplementation, nutrition support, and assessment of uterine involution or coagulation should follow medical orders. Because fetal distress, meconium-stained fluid, or changed Apgar scores were recorded, cooperate with pediatric observation of feeding, skin color, temperature, breathing, and responsiveness. Poor feeding, cyanosis, excessive sleepiness, or abnormal breathing should be reported immediately. For abnormal placental location, shape, or placental sinus records, monitor bleeding amount and duration of lochia. Ultrasound review of the uterine cavity and involution may be needed at follow-up. Advanced maternal age may slow recovery, so the family should help with nighttime care and support adequate sleep, nutrition, and emotional stability. Before discharge, provide education on medication, wound care, breastfeeding, and contraception. Arrange a postpartum review around 42 days after delivery, or earlier if symptoms occur. Seek urgent medical care for sudden heavy vaginal bleeding, bright red bleeding soaking pads, foul-smelling lochia, fever, persistent abdominal pain, pus from the wound, difficulty urinating, unilateral leg swelling or pain, chest tightness, shortness of breath, or persistent sadness, anxiety, or thoughts of harming herself or the baby. | The patient is a 37-year-old G3P2 who underwent emergency cesarean section for complete placenta previa. Blood loss 820 mL (PPH), newborn Apgar 9-10-10. High PPH risk – monitor Hb, transfusion if needed; iron supplementation post-discharge. Watch for late PPH. Rule out placenta accreta with postpartum ultrasound. Thrombosis prevention for advanced age. Routine post-cesarean care. Psychological support. Follow-up: CBC at 1 week; ultrasound at 6 weeks. |
| **Clinical appropriateness 临床适当性** | 5 | 4 |
| **Individualization 个体化程度** | 5 | 5 |
| **Comprehensiveness 完整性** | 5 | 3 |
| **Actionability 可操作性** | 5 | 4 |
| **Safety 安全性** | 5 | 4 |
| **Triage appropriateness 分诊/转诊建议适当性** | 5 | 3 |
| **Structure and clarity 结构与清晰度** | 4 | 3 |

**病例 96 / Case 96**

| **项目 Item** | **ChatGPT 5.5** | **DeepSeek V3** |
| --- | --- | --- |
| **去标识化临床病例摘要 De-identified Clinical Case Summary** | 患者34岁，孕产史为G2P1，既往剖宫产1次，孕周36+6，临产类别为未临产。人工破膜，羊水量约180ml，羊水性质为清。以剖宫产分娩，产时出血约520ml。第一产程未进入产程，第二产程未进入第二产程，第三产程0时6分，总产程未进入产程，会阴或切口情况为完整。合并症及主要风险因素：重度子痫前期；胎儿生长受限？。新生儿出生体重2380g，Apgar评分1、5、10分钟分别为9、10、10分，分娩结局为活产。 | |
| **临床病例摘要英文翻译 English Translation of De-identified Clinical Case Summary** | Maternal age was 34 years. The obstetric history was G2P1, with 1 previous cesarean deliveries. Gestational age was 36+6 weeks, and the labor onset or induction category was no labor. The membranes were artificially ruptured; the amniotic fluid volume was approximately 180 mL and its character was clear. The patient underwent cesarean delivery, with estimated intrapartum blood loss of 520 mL. The first, second, and third stages of labor were labor was not established, the second stage was not reached, and 0 h 6 min, respectively; the total labor duration was labor was not established. The perineum was intact. Documented comorbidities and major risk factors were severe preeclampsia; suspected fetal growth restriction. Neonatal birth weight was 2380 g; Apgar scores at 1, 5, and 10 minutes were 9, 10, 10; and the delivery outcome was live birth. | |
| **模型生成的产褥期护理计划 Model-generated postpartum nursing care plan** | 产妇34岁，G2P1，孕36+6周，经剖宫产分娩，产时出血约520ml，新生儿女婴、体重2380g，Apgar评分9、10、10分。病例提示的重点风险为妊娠期高血压或子痫前期相关风险、出血或贫血相关风险、胎儿窘迫或羊水异常记录。产后24小时内重点评估体温、脉搏、血压、宫底高度、宫缩、阴道流血、恶露颜色和尿潴留，记录首次排尿和疼痛评分。产后住院及出院后继续观察恶露是否由红色逐渐转淡，乳房是否胀痛，乳头是否皲裂，睡眠和情绪是否稳定。饮食以优质蛋白、含铁食物、蔬菜水果和足量饮水为主，避免盲目大补和长期卧床。本次为剖宫产，腹部切口和血栓预防是护理重点。切口应保持清洁干燥，观察红肿、渗血、渗液、裂开、异味和疼痛加重。翻身、咳嗽和下床时保护切口，在生命体征平稳后尽早床旁活动，关注肠鸣音恢复、排气排便、尿潴留和下肢肿痛。哺乳方面建议尽早母婴皮肤接触和按需哺乳，指导正确含接，乳胀时先热敷或轻柔按摩再哺乳，乳头疼痛时及时调整姿势。若出现乳房局部红肿硬痛、寒战或高热，应警惕乳腺炎。因存在出血量偏多、贫血或血小板异常相关风险，应复查血常规，关注头晕、心悸、乏力、面色苍白、恶露突然增多或较大血块，按医嘱补铁，必要时评估凝血和子宫复旧。有高血压或子痫前期风险者，产后仍可能出现血压波动，应按医嘱监测血压，若持续头痛、眼花、胸闷、上腹痛、抽搐或水肿加重，应立即就医。因有胎儿窘迫、羊水污染或新生儿评分变化记录，母婴同室期间应配合观察新生儿吃奶、肤色、体温、呼吸和反应，发现吃奶差、发绀、嗜睡或呼吸异常应立即告知儿科医生。出院前应完成用药、伤口、哺乳和避孕宣教，42天左右复查，必要时提前复诊。若出现阴道流血突然增多、鲜红血浸湿卫生巾、恶露恶臭、发热、持续腹痛、伤口化脓、排尿困难、下肢单侧肿痛、胸闷气短，或持续悲伤焦虑及伤害自己或婴儿的想法，应立即就医或急诊处理。 | 患者34岁，G2P1，剖宫产（急诊），合并重度子痫前期、可疑胎儿生长受限，产时出血520ml，新生儿体重2380g，Apgar 9-10-10。重度子痫前期产后需继续降压治疗，监测血压，警惕产后子痫；每日监测尿蛋白。胎儿生长受限新生儿需监测血糖、体温、喂养，建议新生儿科观察。剖宫产术后常规护理。出血520ml注意晚期出血。心理支持。42天复查血压、尿蛋白；新生儿生长随访。 |
| **护理计划英文翻译 English translation of care plan** | The patient is 34 years old, G2P1, delivered at 36+6 weeks by cesarean delivery, with an estimated intrapartum blood loss of 520 mL. The newborn was a female infant, weighed 2380 g, and had Apgar scores of 9, 10, 10. The main case-based risks include gestational hypertension or preeclampsia-related risk, bleeding or anemia-related risk, fetal distress or abnormal amniotic fluid findings. Within the first 24 hours, assess temperature, pulse, blood pressure, uterine fundal height, uterine tone, vaginal bleeding, lochia color, urinary retention, first voiding, and pain score. During hospitalization and after discharge, continue to observe whether lochia gradually changes from red to lighter color, whether breast engorgement or nipple fissure occurs, and whether sleep and mood remain stable. Diet should include high-quality protein, iron-rich food, vegetables, fruit, and adequate fluids. Avoid blind over-supplementation and prolonged bed rest. Because this was a cesarean delivery, abdominal incision care and thrombosis prevention are key. Keep the incision clean and dry and observe for redness, swelling, bleeding, drainage, separation, odor, or worsening pain. Support the incision when turning, coughing, or getting out of bed. Once vital signs are stable, begin assisted ambulation and monitor bowel sounds, passage of gas, bowel movement, urinary retention, and leg swelling or pain. For breastfeeding, encourage early skin-to-skin contact and feeding on demand. Teach correct latch and positioning. For engorgement, apply warmth or gentle massage before feeding. If nipple pain occurs, adjust latch promptly. Localized breast redness, hard painful swelling, chills, or high fever should raise concern for mastitis. Because increased blood loss, anemia, or platelet abnormality is a concern, repeat a complete blood count as advised and watch for dizziness, palpitations, severe fatigue, pallor, sudden increase in lochia, or large clots. Iron supplementation, nutrition support, and assessment of uterine involution or coagulation should follow medical orders. For hypertension or preeclampsia risk, postpartum blood pressure may still fluctuate. Continue blood pressure monitoring as advised and seek urgent care for persistent headache, visual symptoms, chest tightness, upper abdominal pain, seizure, or worsening edema. Because fetal distress, meconium-stained fluid, or changed Apgar scores were recorded, cooperate with pediatric observation of feeding, skin color, temperature, breathing, and responsiveness. Poor feeding, cyanosis, excessive sleepiness, or abnormal breathing should be reported immediately. Before discharge, provide education on medication, wound care, breastfeeding, and contraception. Arrange a postpartum review around 42 days after delivery, or earlier if symptoms occur. Seek urgent medical care for sudden heavy vaginal bleeding, bright red bleeding soaking pads, foul-smelling lochia, fever, persistent abdominal pain, pus from the wound, difficulty urinating, unilateral leg swelling or pain, chest tightness, shortness of breath, or persistent sadness, anxiety, or thoughts of harming herself or the baby. | The patient is a 34-year-old G2P1 who underwent emergency cesarean section for severe preeclampsia and suspected fetal growth restriction. Blood loss 520 mL, newborn weight 2380 g (LBW), Apgar 9-10-10. Continue antihypertensives postpartum, monitor BP, watch for eclampsia. Monitor urine protein. Newborn FGR – monitor glucose, temperature, feeding; pediatric observation. Routine post-cesarean care. Watch for late PPH. Psychological support. Follow-up: BP and urine protein at 42 days; newborn growth monitoring. |
| **Clinical appropriateness 临床适当性** | 5 | 4 |
| **Individualization 个体化程度** | 5 | 4 |
| **Comprehensiveness 完整性** | 5 | 4 |
| **Actionability 可操作性** | 4 | 4 |
| **Safety 安全性** | 5 | 4 |
| **Triage appropriateness 分诊/转诊建议适当性** | 5 | 4 |
| **Structure and clarity 结构与清晰度** | 4 | 3 |

**病例 97 / Case 97**

| **项目 Item** | **ChatGPT 5.5** | **DeepSeek V3** |
| --- | --- | --- |
| **去标识化临床病例摘要 De-identified Clinical Case Summary** | 患者30岁，孕产史为G1P1，既往剖宫产1次，孕周38，临产类别为自然临产。自然破膜，羊水量约100ml，羊水性质为血性。以剖宫产分娩，产时出血约650ml。第一产程3时40分，第二产程未进入第二产程，第三产程0时5分，总产程3时45分，会阴或切口情况为完整。合并症及主要风险因素：胎盘早剥？胎儿窘迫？。新生儿出生体重3020g，Apgar评分1、5、10分钟分别为7、9、10分，分娩结局为活产。 | |
| **临床病例摘要英文翻译 English Translation of De-identified Clinical Case Summary** | Maternal age was 30 years. The obstetric history was G1P1, with 1 previous cesarean deliveries. Gestational age was 38 weeks, and the labor onset or induction category was spontaneous labor. The membranes ruptured spontaneously; the amniotic fluid volume was approximately 100 mL and its character was blood-stained. The patient underwent cesarean delivery, with estimated intrapartum blood loss of 650 mL. The first, second, and third stages of labor were 3 h 40 min, the second stage was not reached, and 0 h 5 min, respectively; the total labor duration was 3 h 45 min. The perineum was intact. Documented comorbidities and major risk factors were suspected placental abruption; suspected fetal distress. Neonatal birth weight was 3020 g; Apgar scores at 1, 5, and 10 minutes were 7, 9, 10; and the delivery outcome was live birth. | |
| **模型生成的产褥期护理计划 Model-generated postpartum nursing care plan** | 产妇30岁，G1P1，孕38周，经剖宫产分娩，产时出血约650ml，新生儿男婴、体重3020g，Apgar评分7、9、10分。病例提示的重点风险为出血或贫血相关风险、胎盘相关风险、胎儿窘迫或羊水异常记录。产后24小时内重点评估体温、脉搏、血压、宫底高度、宫缩、阴道流血、恶露颜色和尿潴留，记录首次排尿和疼痛评分。产后住院及出院后继续观察恶露是否由红色逐渐转淡，乳房是否胀痛，乳头是否皲裂，睡眠和情绪是否稳定。饮食以优质蛋白、含铁食物、蔬菜水果和足量饮水为主，避免盲目大补和长期卧床。本次为剖宫产，腹部切口和血栓预防是护理重点。切口应保持清洁干燥，观察红肿、渗血、渗液、裂开、异味和疼痛加重。翻身、咳嗽和下床时保护切口，在生命体征平稳后尽早床旁活动，关注肠鸣音恢复、排气排便、尿潴留和下肢肿痛。哺乳方面建议尽早母婴皮肤接触和按需哺乳，指导正确含接，乳胀时先热敷或轻柔按摩再哺乳，乳头疼痛时及时调整姿势。若出现乳房局部红肿硬痛、寒战或高热，应警惕乳腺炎。因存在出血量偏多、贫血或血小板异常相关风险，应复查血常规，关注头晕、心悸、乏力、面色苍白、恶露突然增多或较大血块，按医嘱补铁，必要时评估凝血和子宫复旧。因有胎儿窘迫、羊水污染或新生儿评分变化记录，母婴同室期间应配合观察新生儿吃奶、肤色、体温、呼吸和反应，发现吃奶差、发绀、嗜睡或呼吸异常应立即告知儿科医生。有胎盘位置、形态或胎盘血窦相关记录时，应注意恶露持续时间和出血量，产后复查可结合B超了解宫腔及子宫复旧。出院前应完成用药、伤口、哺乳和避孕宣教，42天左右复查，必要时提前复诊。若出现阴道流血突然增多、鲜红血浸湿卫生巾、恶露恶臭、发热、持续腹痛、伤口化脓、排尿困难、下肢单侧肿痛、胸闷气短，或持续悲伤焦虑及伤害自己或婴儿的想法，应立即就医或急诊处理。 | 患者30岁，G1P1，急诊剖宫产，合并胎盘早剥可疑、胎儿窘迫，产时出血650ml，新生儿Apgar 7-9-10。胎盘早剥产后需监测凝血功能、子宫收缩，警惕DIC及产后出血。出血650ml需补铁、输血可能。新生儿低Apgar需观察神经系统。剖宫产术后常规护理。心理支持。产后1周复查血常规、凝血功能；新生儿专科随访。 |
| **护理计划英文翻译 English translation of care plan** | The patient is 30 years old, G1P1, delivered at 38 weeks by cesarean delivery, with an estimated intrapartum blood loss of 650 mL. The newborn was a male infant, weighed 3020 g, and had Apgar scores of 7, 9, 10. The main case-based risks include bleeding or anemia-related risk, placental risk factors, fetal distress or abnormal amniotic fluid findings. Within the first 24 hours, assess temperature, pulse, blood pressure, uterine fundal height, uterine tone, vaginal bleeding, lochia color, urinary retention, first voiding, and pain score. During hospitalization and after discharge, continue to observe whether lochia gradually changes from red to lighter color, whether breast engorgement or nipple fissure occurs, and whether sleep and mood remain stable. Diet should include high-quality protein, iron-rich food, vegetables, fruit, and adequate fluids. Avoid blind over-supplementation and prolonged bed rest. Because this was a cesarean delivery, abdominal incision care and thrombosis prevention are key. Keep the incision clean and dry and observe for redness, swelling, bleeding, drainage, separation, odor, or worsening pain. Support the incision when turning, coughing, or getting out of bed. Once vital signs are stable, begin assisted ambulation and monitor bowel sounds, passage of gas, bowel movement, urinary retention, and leg swelling or pain. For breastfeeding, encourage early skin-to-skin contact and feeding on demand. Teach correct latch and positioning. For engorgement, apply warmth or gentle massage before feeding. If nipple pain occurs, adjust latch promptly. Localized breast redness, hard painful swelling, chills, or high fever should raise concern for mastitis. Because increased blood loss, anemia, or platelet abnormality is a concern, repeat a complete blood count as advised and watch for dizziness, palpitations, severe fatigue, pallor, sudden increase in lochia, or large clots. Iron supplementation, nutrition support, and assessment of uterine involution or coagulation should follow medical orders. Because fetal distress, meconium-stained fluid, or changed Apgar scores were recorded, cooperate with pediatric observation of feeding, skin color, temperature, breathing, and responsiveness. Poor feeding, cyanosis, excessive sleepiness, or abnormal breathing should be reported immediately. For abnormal placental location, shape, or placental sinus records, monitor bleeding amount and duration of lochia. Ultrasound review of the uterine cavity and involution may be needed at follow-up. Before discharge, provide education on medication, wound care, breastfeeding, and contraception. Arrange a postpartum review around 42 days after delivery, or earlier if symptoms occur. Seek urgent medical care for sudden heavy vaginal bleeding, bright red bleeding soaking pads, foul-smelling lochia, fever, persistent abdominal pain, pus from the wound, difficulty urinating, unilateral leg swelling or pain, chest tightness, shortness of breath, or persistent sadness, anxiety, or thoughts of harming herself or the baby. | The patient is a 30-year-old G1P1 who underwent emergency cesarean section for suspected placental abruption and fetal distress. Blood loss 650 mL, newborn Apgar 7-9-10. Monitor coagulation and uterine contraction; watch for DIC and PPH. Iron supplementation, possible transfusion. Newborn – neurological observation for low Apgar. Routine post-cesarean care. Psychological support. Follow-up: CBC and coagulation at 1 week; newborn specialist follow-up. |
| **Clinical appropriateness 临床适当性** | 5 | 4 |
| **Individualization 个体化程度** | 5 | 5 |
| **Comprehensiveness 完整性** | 5 | 3 |
| **Actionability 可操作性** | 4 | 4 |
| **Safety 安全性** | 5 | 4 |
| **Triage appropriateness 分诊/转诊建议适当性** | 5 | 3 |
| **Structure and clarity 结构与清晰度** | 4 | 3 |

**病例 98 / Case 98**

| **项目 Item** | **ChatGPT 5.5** | **DeepSeek V3** |
| --- | --- | --- |
| **去标识化临床病例摘要 De-identified Clinical Case Summary** | 患者33岁，孕产史为G1P1，既往剖宫产1次，孕周39+6，临产类别为未临产。人工破膜，羊水量约400ml，羊水性质为清。以剖宫产分娩，产时出血约390ml。第一产程未进入产程，第二产程未进入第二产程，第三产程0时5分，总产程未进入产程，会阴或切口情况为完整。合并症及主要风险因素：妊娠期糖尿病；巨大儿倾向。新生儿出生体重4120g，Apgar评分1、5、10分钟分别为10、10、10分，分娩结局为活产。 | |
| **临床病例摘要英文翻译 English Translation of De-identified Clinical Case Summary** | Maternal age was 33 years. The obstetric history was G1P1, with 1 previous cesarean deliveries. Gestational age was 39+6 weeks, and the labor onset or induction category was no labor. The membranes were artificially ruptured; the amniotic fluid volume was approximately 400 mL and its character was clear. The patient underwent cesarean delivery, with estimated intrapartum blood loss of 390 mL. The first, second, and third stages of labor were labor was not established, the second stage was not reached, and 0 h 5 min, respectively; the total labor duration was labor was not established. The perineum was intact. Documented comorbidities and major risk factors were gestational diabetes mellitus; suspected macrosomia. Neonatal birth weight was 4120 g; Apgar scores at 1, 5, and 10 minutes were 10, 10, 10; and the delivery outcome was live birth. | |
| **模型生成的产褥期护理计划 Model-generated postpartum nursing care plan** | 产妇33岁，G1P1，孕39+6周，经剖宫产分娩，产时出血约390ml，新生儿女婴、体重4120g，Apgar评分10、10、10分。病例提示的重点风险为妊娠期糖尿病。产后24小时内重点评估体温、脉搏、血压、宫底高度、宫缩、阴道流血、恶露颜色和尿潴留，记录首次排尿和疼痛评分。产后住院及出院后继续观察恶露是否由红色逐渐转淡，乳房是否胀痛，乳头是否皲裂，睡眠和情绪是否稳定。饮食以优质蛋白、含铁食物、蔬菜水果和足量饮水为主，避免盲目大补和长期卧床。本次为剖宫产，腹部切口和血栓预防是护理重点。切口应保持清洁干燥，观察红肿、渗血、渗液、裂开、异味和疼痛加重。翻身、咳嗽和下床时保护切口，在生命体征平稳后尽早床旁活动，关注肠鸣音恢复、排气排便、尿潴留和下肢肿痛。哺乳方面建议尽早母婴皮肤接触和按需哺乳，指导正确含接，乳胀时先热敷或轻柔按摩再哺乳，乳头疼痛时及时调整姿势。若出现乳房局部红肿硬痛、寒战或高热，应警惕乳腺炎。有妊娠期糖尿病者，产后饮食应控制精制糖和高油食物，少量多餐，结合恢复情况逐步活动，并按医嘱在产后复查血糖或糖耐量。出院前应完成用药、伤口、哺乳和避孕宣教，42天左右复查，必要时提前复诊。若出现阴道流血突然增多、鲜红血浸湿卫生巾、恶露恶臭、发热、持续腹痛、伤口化脓、排尿困难、下肢单侧肿痛、胸闷气短，或持续悲伤焦虑及伤害自己或婴儿的想法，应立即就医或急诊处理。 | 患者33岁，G1P1，剖宫产（择期），合并GDM、巨大儿倾向，新生儿体重4120g（巨大儿），产时出血390ml。GDM产后监测血糖。巨大儿剖宫产需注意新生儿低血糖，出生后2小时内监测血糖。剖宫产术后常规护理。鼓励母乳喂养。42天复查血糖。 |
| **护理计划英文翻译 English translation of care plan** | The patient is 33 years old, G1P1, delivered at 39+6 weeks by cesarean delivery, with an estimated intrapartum blood loss of 390 mL. The newborn was a female infant, weighed 4120 g, and had Apgar scores of 10, 10, 10. The main case-based risks include gestational diabetes mellitus. Within the first 24 hours, assess temperature, pulse, blood pressure, uterine fundal height, uterine tone, vaginal bleeding, lochia color, urinary retention, first voiding, and pain score. During hospitalization and after discharge, continue to observe whether lochia gradually changes from red to lighter color, whether breast engorgement or nipple fissure occurs, and whether sleep and mood remain stable. Diet should include high-quality protein, iron-rich food, vegetables, fruit, and adequate fluids. Avoid blind over-supplementation and prolonged bed rest. Because this was a cesarean delivery, abdominal incision care and thrombosis prevention are key. Keep the incision clean and dry and observe for redness, swelling, bleeding, drainage, separation, odor, or worsening pain. Support the incision when turning, coughing, or getting out of bed. Once vital signs are stable, begin assisted ambulation and monitor bowel sounds, passage of gas, bowel movement, urinary retention, and leg swelling or pain. For breastfeeding, encourage early skin-to-skin contact and feeding on demand. Teach correct latch and positioning. For engorgement, apply warmth or gentle massage before feeding. If nipple pain occurs, adjust latch promptly. Localized breast redness, hard painful swelling, chills, or high fever should raise concern for mastitis. For gestational diabetes, avoid refined sugar and heavy oily foods, eat small balanced meals, increase activity gradually, and arrange postpartum glucose or oral glucose tolerance testing as advised. Before discharge, provide education on medication, wound care, breastfeeding, and contraception. Arrange a postpartum review around 42 days after delivery, or earlier if symptoms occur. Seek urgent medical care for sudden heavy vaginal bleeding, bright red bleeding soaking pads, foul-smelling lochia, fever, persistent abdominal pain, pus from the wound, difficulty urinating, unilateral leg swelling or pain, chest tightness, shortness of breath, or persistent sadness, anxiety, or thoughts of harming herself or the baby. | The patient is a 33-year-old G1P1 who underwent elective cesarean section for GDM and macrosomia tendency. Newborn weight 4120 g (macrosomia), blood loss 390 mL. Monitor postpartum glucose. Newborn – monitor for hypoglycemia within first 2 hours. Routine post-cesarean care. Encourage breastfeeding. Follow-up: glucose at 42 days. |
| **Clinical appropriateness 临床适当性** | 4 | 3 |
| **Individualization 个体化程度** | 3 | 3 |
| **Comprehensiveness 完整性** | 4 | 3 |
| **Actionability 可操作性** | 4 | 4 |
| **Safety 安全性** | 5 | 3 |
| **Triage appropriateness 分诊/转诊建议适当性** | 5 | 3 |
| **Structure and clarity 结构与清晰度** | 3 | 4 |

**病例 99 / Case 99**

| **项目 Item** | **ChatGPT 5.5** | **DeepSeek V3** |
| --- | --- | --- |
| **去标识化临床病例摘要 De-identified Clinical Case Summary** | 患者28岁，孕产史为G1P1，既往剖宫产1次，孕周39+1，临产类别为未临产。人工破膜，羊水量约300ml，羊水性质为清。以剖宫产分娩，产时出血约340ml。第一产程未进入产程，第二产程未进入第二产程，第三产程0时5分，总产程未进入产程，会阴或切口情况为完整。合并症及主要风险因素：横位妊娠，胎位异常。新生儿出生体重3090g，Apgar评分1、5、10分钟分别为10、10、10分，分娩结局为活产。 | |
| **临床病例摘要英文翻译 English Translation of De-identified Clinical Case Summary** | Maternal age was 28 years. The obstetric history was G1P1, with 1 previous cesarean deliveries. Gestational age was 39+1 weeks, and the labor onset or induction category was no labor. The membranes were artificially ruptured; the amniotic fluid volume was approximately 300 mL and its character was clear. The patient underwent cesarean delivery, with estimated intrapartum blood loss of 340 mL. The first, second, and third stages of labor were labor was not established, the second stage was not reached, and 0 h 5 min, respectively; the total labor duration was labor was not established. The perineum was intact. Documented comorbidities and major risk factors were transverse lie, malpresentation. Neonatal birth weight was 3090 g; Apgar scores at 1, 5, and 10 minutes were 10, 10, 10; and the delivery outcome was live birth. | |
| **模型生成的产褥期护理计划 Model-generated postpartum nursing care plan** | 产妇28岁，G1P1，孕39+1周，经剖宫产分娩，产时出血约340ml，新生儿女婴、体重3090g，Apgar评分10、10、10分。病例提示的重点风险为未记录特殊合并症。产后24小时内重点评估体温、脉搏、血压、宫底高度、宫缩、阴道流血、恶露颜色和尿潴留，记录首次排尿和疼痛评分。产后住院及出院后继续观察恶露是否由红色逐渐转淡，乳房是否胀痛，乳头是否皲裂，睡眠和情绪是否稳定。饮食以优质蛋白、含铁食物、蔬菜水果和足量饮水为主，避免盲目大补和长期卧床。本次为剖宫产，腹部切口和血栓预防是护理重点。切口应保持清洁干燥，观察红肿、渗血、渗液、裂开、异味和疼痛加重。翻身、咳嗽和下床时保护切口，在生命体征平稳后尽早床旁活动，关注肠鸣音恢复、排气排便、尿潴留和下肢肿痛。哺乳方面建议尽早母婴皮肤接触和按需哺乳，指导正确含接，乳胀时先热敷或轻柔按摩再哺乳，乳头疼痛时及时调整姿势。若出现乳房局部红肿硬痛、寒战或高热，应警惕乳腺炎。目前未记录特殊合并症，仍需按常规产褥期管理执行，重点是子宫复旧、恶露、伤口、哺乳、营养、活动和心理状态。出院前应完成用药、伤口、哺乳和避孕宣教，42天左右复查，必要时提前复诊。若出现阴道流血突然增多、鲜红血浸湿卫生巾、恶露恶臭、发热、持续腹痛、伤口化脓、排尿困难、下肢单侧肿痛、胸闷气短，或持续悲伤焦虑及伤害自己或婴儿的想法，应立即就医或急诊处理。 | 患者28岁，G1P1，剖宫产（择期），合并横位妊娠，产时出血340ml。剖宫产术后常规护理。横位新生儿无特殊。42天复查。 |
| **护理计划英文翻译 English translation of care plan** | The patient is 28 years old, G1P1, delivered at 39+1 weeks by cesarean delivery, with an estimated intrapartum blood loss of 340 mL. The newborn was a female infant, weighed 3090 g, and had Apgar scores of 10, 10, 10. The main case-based risks include no special comorbidity was recorded. Within the first 24 hours, assess temperature, pulse, blood pressure, uterine fundal height, uterine tone, vaginal bleeding, lochia color, urinary retention, first voiding, and pain score. During hospitalization and after discharge, continue to observe whether lochia gradually changes from red to lighter color, whether breast engorgement or nipple fissure occurs, and whether sleep and mood remain stable. Diet should include high-quality protein, iron-rich food, vegetables, fruit, and adequate fluids. Avoid blind over-supplementation and prolonged bed rest. Because this was a cesarean delivery, abdominal incision care and thrombosis prevention are key. Keep the incision clean and dry and observe for redness, swelling, bleeding, drainage, separation, odor, or worsening pain. Support the incision when turning, coughing, or getting out of bed. Once vital signs are stable, begin assisted ambulation and monitor bowel sounds, passage of gas, bowel movement, urinary retention, and leg swelling or pain. For breastfeeding, encourage early skin-to-skin contact and feeding on demand. Teach correct latch and positioning. For engorgement, apply warmth or gentle massage before feeding. If nipple pain occurs, adjust latch promptly. Localized breast redness, hard painful swelling, chills, or high fever should raise concern for mastitis. No special complication was recorded, but routine postpartum management should still focus on uterine involution, lochia, wounds, breastfeeding, nutrition, activity, and mental health. Before discharge, provide education on medication, wound care, breastfeeding, and contraception. Arrange a postpartum review around 42 days after delivery, or earlier if symptoms occur. Seek urgent medical care for sudden heavy vaginal bleeding, bright red bleeding soaking pads, foul-smelling lochia, fever, persistent abdominal pain, pus from the wound, difficulty urinating, unilateral leg swelling or pain, chest tightness, shortness of breath, or persistent sadness, anxiety, or thoughts of harming herself or the baby. | The patient is a 28-year-old G1P1 who underwent cesarean section for transverse lie. Blood loss 340 mL. Routine post-cesarean care. Newborn – no special issues. Follow-up at 42 days. |
| **Clinical appropriateness 临床适当性** | 4 | 3 |
| **Individualization 个体化程度** | 3 | 3 |
| **Comprehensiveness 完整性** | 4 | 3 |
| **Actionability 可操作性** | 4 | 3 |
| **Safety 安全性** | 5 | 3 |
| **Triage appropriateness 分诊/转诊建议适当性** | 5 | 3 |
| **Structure and clarity 结构与清晰度** | 3 | 4 |

**病例 100 / Case 100**

| **项目 Item** | **ChatGPT 5.5** | **DeepSeek V3** |
| --- | --- | --- |
| **去标识化临床病例摘要 De-identified Clinical Case Summary** | 患者36岁，孕产史为G2P1，既往剖宫产1次，孕周40+4，临产类别为催产素引产。人工破膜，羊水量约120ml，羊水性质为Ⅱ°。以剖宫产分娩，产时出血约460ml。第一产程6时35分，第二产程未进入第二产程，第三产程0时6分，总产程6时41分，会阴或切口情况为完整。合并症及主要风险因素：羊水过少；胎儿宫内窘迫？；高龄初孕妇的监督。新生儿出生体重2810g，Apgar评分1、5、10分钟分别为9、10、10分，分娩结局为活产。 | |
| **临床病例摘要英文翻译 English Translation of De-identified Clinical Case Summary** | Maternal age was 36 years. The obstetric history was G2P1, with 1 previous cesarean deliveries. Gestational age was 40+4 weeks, and the labor onset or induction category was oxytocin induction. The membranes were artificially ruptured; the amniotic fluid volume was approximately 120 mL and its character was grade II meconium-stained. The patient underwent cesarean delivery, with estimated intrapartum blood loss of 460 mL. The first, second, and third stages of labor were 6 h 35 min, the second stage was not reached, and 0 h 6 min, respectively; the total labor duration was 6 h 41 min. The perineum was intact. Documented comorbidities and major risk factors were oligohydramnios; suspected fetal distress; advanced maternal age in a primigravida. Neonatal birth weight was 2810 g; Apgar scores at 1, 5, and 10 minutes were 9, 10, 10; and the delivery outcome was live birth. | |
| **模型生成的产褥期护理计划 Model-generated postpartum nursing care plan** | 产妇36岁，G2P1，孕40+4周，经剖宫产分娩，产时出血约460ml，新生儿男婴、体重2810g，Apgar评分9、10、10分。病例提示的重点风险为胎儿窘迫或羊水异常记录、高龄妊娠因素。产后24小时内重点评估体温、脉搏、血压、宫底高度、宫缩、阴道流血、恶露颜色和尿潴留，记录首次排尿和疼痛评分。产后住院及出院后继续观察恶露是否由红色逐渐转淡，乳房是否胀痛，乳头是否皲裂，睡眠和情绪是否稳定。饮食以优质蛋白、含铁食物、蔬菜水果和足量饮水为主，避免盲目大补和长期卧床。本次为剖宫产，腹部切口和血栓预防是护理重点。切口应保持清洁干燥，观察红肿、渗血、渗液、裂开、异味和疼痛加重。翻身、咳嗽和下床时保护切口，在生命体征平稳后尽早床旁活动，关注肠鸣音恢复、排气排便、尿潴留和下肢肿痛。哺乳方面建议尽早母婴皮肤接触和按需哺乳，指导正确含接，乳胀时先热敷或轻柔按摩再哺乳，乳头疼痛时及时调整姿势。若出现乳房局部红肿硬痛、寒战或高热，应警惕乳腺炎。产时出血量需要持续观察，建议每日记录恶露量、颜色和血块情况，活动后若明显增多，应减少活动并请医护评估。因有胎儿窘迫、羊水污染或新生儿评分变化记录，母婴同室期间应配合观察新生儿吃奶、肤色、体温、呼吸和反应，发现吃奶差、发绀、嗜睡或呼吸异常应立即告知儿科医生。高龄产妇恢复期应避免过度劳累和连续熬夜，家属应参与夜间照护，保证睡眠、营养和情绪支持。出院前应完成用药、伤口、哺乳和避孕宣教，42天左右复查，必要时提前复诊。若出现阴道流血突然增多、鲜红血浸湿卫生巾、恶露恶臭、发热、持续腹痛、伤口化脓、排尿困难、下肢单侧肿痛、胸闷气短，或持续悲伤焦虑及伤害自己或婴儿的想法，应立即就医或急诊处理。 | 患者36岁，G2P1，剖宫产（急诊），合并羊水过少、可疑胎儿窘迫、高龄初产，脐带绕颈2周，羊水Ⅱ°污染，产时出血460ml，新生儿Apgar 9-10-10。高龄初产剖宫产术后常规护理。羊水过少+Ⅱ°污染+脐带绕颈，新生儿需监测MAS及缺氧。出血460ml注意晚期出血。心理支持。42天复查；新生儿随访。 |
| **护理计划英文翻译 English translation of care plan** | The patient is 36 years old, G2P1, delivered at 40+4 weeks by cesarean delivery, with an estimated intrapartum blood loss of 460 mL. The newborn was a male infant, weighed 2810 g, and had Apgar scores of 9, 10, 10. The main case-based risks include fetal distress or abnormal amniotic fluid findings, advanced maternal age. Within the first 24 hours, assess temperature, pulse, blood pressure, uterine fundal height, uterine tone, vaginal bleeding, lochia color, urinary retention, first voiding, and pain score. During hospitalization and after discharge, continue to observe whether lochia gradually changes from red to lighter color, whether breast engorgement or nipple fissure occurs, and whether sleep and mood remain stable. Diet should include high-quality protein, iron-rich food, vegetables, fruit, and adequate fluids. Avoid blind over-supplementation and prolonged bed rest. Because this was a cesarean delivery, abdominal incision care and thrombosis prevention are key. Keep the incision clean and dry and observe for redness, swelling, bleeding, drainage, separation, odor, or worsening pain. Support the incision when turning, coughing, or getting out of bed. Once vital signs are stable, begin assisted ambulation and monitor bowel sounds, passage of gas, bowel movement, urinary retention, and leg swelling or pain. For breastfeeding, encourage early skin-to-skin contact and feeding on demand. Teach correct latch and positioning. For engorgement, apply warmth or gentle massage before feeding. If nipple pain occurs, adjust latch promptly. Localized breast redness, hard painful swelling, chills, or high fever should raise concern for mastitis. Because blood loss requires closer observation, record the amount, color, and clots in the lochia every day. If bleeding increases clearly after activity, reduce activity and seek clinical assessment. Because fetal distress, meconium-stained fluid, or changed Apgar scores were recorded, cooperate with pediatric observation of feeding, skin color, temperature, breathing, and responsiveness. Poor feeding, cyanosis, excessive sleepiness, or abnormal breathing should be reported immediately. Advanced maternal age may slow recovery, so the family should help with nighttime care and support adequate sleep, nutrition, and emotional stability. Before discharge, provide education on medication, wound care, breastfeeding, and contraception. Arrange a postpartum review around 42 days after delivery, or earlier if symptoms occur. Seek urgent medical care for sudden heavy vaginal bleeding, bright red bleeding soaking pads, foul-smelling lochia, fever, persistent abdominal pain, pus from the wound, difficulty urinating, unilateral leg swelling or pain, chest tightness, shortness of breath, or persistent sadness, anxiety, or thoughts of harming herself or the baby. | The patient is a 36-year-old G2P1 who underwent emergency cesarean section for oligohydramnios, suspected fetal distress, advanced maternal age in a primigravida, nuchal cord 2 loops, grade II meconium. Blood loss 460 mL, newborn Apgar 9-10-10. Routine post-cesarean care. Newborn – monitor for MAS and hypoxia. Watch for late PPH. Psychological support. Follow-up at 42 days; newborn follow-up. |
| **Clinical appropriateness 临床适当性** | 5 | 4 |
| **Individualization 个体化程度** | 5 | 5 |
| **Comprehensiveness 完整性** | 5 | 3 |
| **Actionability 可操作性** | 4 | 4 |
| **Safety 安全性** | 5 | 4 |
| **Triage appropriateness 分诊/转诊建议适当性** | 5 | 3 |
| **Structure and clarity 结构与清晰度** | 4 | 4 |

**病例 101 / Case 101**

| **项目 Item** | **ChatGPT 5.5** | **DeepSeek V3** |
| --- | --- | --- |
| **去标识化临床病例摘要 De-identified Clinical Case Summary** | 患者27岁，孕产史为G1P1，既往剖宫产1次，孕周39+5，临产类别为自然临产。自然破膜，羊水量约280ml，羊水性质为清。以剖宫产分娩，产时出血约430ml。第一产程7时20分，第二产程未进入第二产程，第三产程0时5分，总产程7时25分，会阴或切口情况为完整。合并症及主要风险因素：头盆不称？产程进展异常，急诊剖宫产。新生儿出生体重3500g，Apgar评分1、5、10分钟分别为10、10、10分，分娩结局为活产。 | |
| **临床病例摘要英文翻译 English Translation of De-identified Clinical Case Summary** | Maternal age was 27 years. The obstetric history was G1P1, with 1 previous cesarean deliveries. Gestational age was 39+5 weeks, and the labor onset or induction category was spontaneous labor. The membranes ruptured spontaneously; the amniotic fluid volume was approximately 280 mL and its character was clear. The patient underwent cesarean delivery, with estimated intrapartum blood loss of 430 mL. The first, second, and third stages of labor were 7 h 20 min, the second stage was not reached, and 0 h 5 min, respectively; the total labor duration was 7 h 25 min. The perineum was intact. Documented comorbidities and major risk factors were suspected cephalopelvic disproportion; abnormal labor progress, emergency cesarean section. Neonatal birth weight was 3500 g; Apgar scores at 1, 5, and 10 minutes were 10, 10, 10; and the delivery outcome was live birth. | |
| **模型生成的产褥期护理计划 Model-generated postpartum nursing care plan** | 产妇27岁，G1P1，孕39+5周，经剖宫产分娩，产时出血约430ml，新生儿女婴、体重3500g，Apgar评分10、10、10分。病例提示的重点风险为未记录特殊合并症。产后24小时内重点评估体温、脉搏、血压、宫底高度、宫缩、阴道流血、恶露颜色和尿潴留，记录首次排尿和疼痛评分。产后住院及出院后继续观察恶露是否由红色逐渐转淡，乳房是否胀痛，乳头是否皲裂，睡眠和情绪是否稳定。饮食以优质蛋白、含铁食物、蔬菜水果和足量饮水为主，避免盲目大补和长期卧床。本次为剖宫产，腹部切口和血栓预防是护理重点。切口应保持清洁干燥，观察红肿、渗血、渗液、裂开、异味和疼痛加重。翻身、咳嗽和下床时保护切口，在生命体征平稳后尽早床旁活动，关注肠鸣音恢复、排气排便、尿潴留和下肢肿痛。哺乳方面建议尽早母婴皮肤接触和按需哺乳，指导正确含接，乳胀时先热敷或轻柔按摩再哺乳，乳头疼痛时及时调整姿势。若出现乳房局部红肿硬痛、寒战或高热，应警惕乳腺炎。产时出血量需要持续观察，建议每日记录恶露量、颜色和血块情况，活动后若明显增多，应减少活动并请医护评估。出院前应完成用药、伤口、哺乳和避孕宣教，42天左右复查，必要时提前复诊。若出现阴道流血突然增多、鲜红血浸湿卫生巾、恶露恶臭、发热、持续腹痛、伤口化脓、排尿困难、下肢单侧肿痛、胸闷气短，或持续悲伤焦虑及伤害自己或婴儿的想法，应立即就医或急诊处理。 | 患者27岁，G1P1，急诊剖宫产，合并头盆不称、产程进展异常，产时出血430ml，新生儿Apgar 10。剖宫产术后常规护理。头盆不称不直接影响产后。出血430ml注意晚期出血。鼓励母乳喂养。42天复查。 |
| **护理计划英文翻译 English translation of care plan** | The patient is 27 years old, G1P1, delivered at 39+5 weeks by cesarean delivery, with an estimated intrapartum blood loss of 430 mL. The newborn was a female infant, weighed 3500 g, and had Apgar scores of 10, 10, 10. The main case-based risks include no special comorbidity was recorded. Within the first 24 hours, assess temperature, pulse, blood pressure, uterine fundal height, uterine tone, vaginal bleeding, lochia color, urinary retention, first voiding, and pain score. During hospitalization and after discharge, continue to observe whether lochia gradually changes from red to lighter color, whether breast engorgement or nipple fissure occurs, and whether sleep and mood remain stable. Diet should include high-quality protein, iron-rich food, vegetables, fruit, and adequate fluids. Avoid blind over-supplementation and prolonged bed rest. Because this was a cesarean delivery, abdominal incision care and thrombosis prevention are key. Keep the incision clean and dry and observe for redness, swelling, bleeding, drainage, separation, odor, or worsening pain. Support the incision when turning, coughing, or getting out of bed. Once vital signs are stable, begin assisted ambulation and monitor bowel sounds, passage of gas, bowel movement, urinary retention, and leg swelling or pain. For breastfeeding, encourage early skin-to-skin contact and feeding on demand. Teach correct latch and positioning. For engorgement, apply warmth or gentle massage before feeding. If nipple pain occurs, adjust latch promptly. Localized breast redness, hard painful swelling, chills, or high fever should raise concern for mastitis. Because blood loss requires closer observation, record the amount, color, and clots in the lochia every day. If bleeding increases clearly after activity, reduce activity and seek clinical assessment. Before discharge, provide education on medication, wound care, breastfeeding, and contraception. Arrange a postpartum review around 42 days after delivery, or earlier if symptoms occur. Seek urgent medical care for sudden heavy vaginal bleeding, bright red bleeding soaking pads, foul-smelling lochia, fever, persistent abdominal pain, pus from the wound, difficulty urinating, unilateral leg swelling or pain, chest tightness, shortness of breath, or persistent sadness, anxiety, or thoughts of harming herself or the baby. | The patient is a 27-year-old G1P1 who underwent emergency cesarean section for cephalopelvic disproportion and abnormal labor progress. Blood loss 430 mL, newborn Apgar 10. Routine post-cesarean care. Watch for late PPH. Encourage breastfeeding. Follow-up at 42 days. |
| **Clinical appropriateness 临床适当性** | 4 | 3 |
| **Individualization 个体化程度** | 3 | 3 |
| **Comprehensiveness 完整性** | 4 | 3 |
| **Actionability 可操作性** | 4 | 3 |
| **Safety 安全性** | 5 | 3 |
| **Triage appropriateness 分诊/转诊建议适当性** | 5 | 3 |
| **Structure and clarity 结构与清晰度** | 3 | 4 |

**病例 102 / Case 102**

| **项目 Item** | **ChatGPT 5.5** | **DeepSeek V3** |
| --- | --- | --- |
| **去标识化临床病例摘要 De-identified Clinical Case Summary** | 患者25岁，孕产史为G2P1，既往剖宫产1次，孕周38+4，临产类别为自然临产。自然破膜，羊水量约100ml，羊水性质为Ⅲ°。以剖宫产分娩，产时出血约500ml。第一产程2时15分，第二产程未进入第二产程，第三产程0时4分，总产程2时19分，会阴或切口情况为完整。合并症及主要风险因素：脐带脱垂？胎儿窘迫？。新生儿出生体重2970g，Apgar评分1、5、10分钟分别为8、9、10分，分娩结局为活产。 | |
| **临床病例摘要英文翻译 English Translation of De-identified Clinical Case Summary** | Maternal age was 25 years. The obstetric history was G2P1, with 1 previous cesarean deliveries. Gestational age was 38+4 weeks, and the labor onset or induction category was spontaneous labor. The membranes ruptured spontaneously; the amniotic fluid volume was approximately 100 mL and its character was grade III meconium-stained. The patient underwent cesarean delivery, with estimated intrapartum blood loss of 500 mL. The first, second, and third stages of labor were 2 h 15 min, the second stage was not reached, and 0 h 4 min, respectively; the total labor duration was 2 h 19 min. The perineum was intact. Documented comorbidities and major risk factors were suspected umbilical cord prolapse; suspected fetal distress. Neonatal birth weight was 2970 g; Apgar scores at 1, 5, and 10 minutes were 8, 9, 10; and the delivery outcome was live birth. | |
| **模型生成的产褥期护理计划 Model-generated postpartum nursing care plan** | 产妇25岁，G2P1，孕38+4周，经剖宫产分娩，产时出血约500ml，新生儿男婴、体重2970g，Apgar评分8、9、10分。病例提示的重点风险为出血或贫血相关风险、胎儿窘迫或羊水异常记录。产后24小时内重点评估体温、脉搏、血压、宫底高度、宫缩、阴道流血、恶露颜色和尿潴留，记录首次排尿和疼痛评分。产后住院及出院后继续观察恶露是否由红色逐渐转淡，乳房是否胀痛，乳头是否皲裂，睡眠和情绪是否稳定。饮食以优质蛋白、含铁食物、蔬菜水果和足量饮水为主，避免盲目大补和长期卧床。本次为剖宫产，腹部切口和血栓预防是护理重点。切口应保持清洁干燥，观察红肿、渗血、渗液、裂开、异味和疼痛加重。翻身、咳嗽和下床时保护切口，在生命体征平稳后尽早床旁活动，关注肠鸣音恢复、排气排便、尿潴留和下肢肿痛。哺乳方面建议尽早母婴皮肤接触和按需哺乳，指导正确含接，乳胀时先热敷或轻柔按摩再哺乳，乳头疼痛时及时调整姿势。若出现乳房局部红肿硬痛、寒战或高热，应警惕乳腺炎。因存在出血量偏多、贫血或血小板异常相关风险，应复查血常规，关注头晕、心悸、乏力、面色苍白、恶露突然增多或较大血块，按医嘱补铁，必要时评估凝血和子宫复旧。因有胎儿窘迫、羊水污染或新生儿评分变化记录，母婴同室期间应配合观察新生儿吃奶、肤色、体温、呼吸和反应，发现吃奶差、发绀、嗜睡或呼吸异常应立即告知儿科医生。出院前应完成用药、伤口、哺乳和避孕宣教，42天左右复查，必要时提前复诊。若出现阴道流血突然增多、鲜红血浸湿卫生巾、恶露恶臭、发热、持续腹痛、伤口化脓、排尿困难、下肢单侧肿痛、胸闷气短，或持续悲伤焦虑及伤害自己或婴儿的想法，应立即就医或急诊处理。 | 患者25岁，G2P1，急诊剖宫产，合并脐带脱垂可疑、胎儿窘迫，羊水Ⅲ°污染，产时出血500ml，新生儿Apgar 8-9-10。脐带脱垂可疑+Ⅲ°羊水，新生儿需密切监测缺氧、MAS，建议新生儿科观察。出血500ml注意晚期出血。剖宫产术后常规护理。心理支持。产后1周复查血常规；新生儿专科随访。 |
| **护理计划英文翻译 English translation of care plan** | The patient is 25 years old, G2P1, delivered at 38+4 weeks by cesarean delivery, with an estimated intrapartum blood loss of 500 mL. The newborn was a male infant, weighed 2970 g, and had Apgar scores of 8, 9, 10. The main case-based risks include bleeding or anemia-related risk, fetal distress or abnormal amniotic fluid findings. Within the first 24 hours, assess temperature, pulse, blood pressure, uterine fundal height, uterine tone, vaginal bleeding, lochia color, urinary retention, first voiding, and pain score. During hospitalization and after discharge, continue to observe whether lochia gradually changes from red to lighter color, whether breast engorgement or nipple fissure occurs, and whether sleep and mood remain stable. Diet should include high-quality protein, iron-rich food, vegetables, fruit, and adequate fluids. Avoid blind over-supplementation and prolonged bed rest. Because this was a cesarean delivery, abdominal incision care and thrombosis prevention are key. Keep the incision clean and dry and observe for redness, swelling, bleeding, drainage, separation, odor, or worsening pain. Support the incision when turning, coughing, or getting out of bed. Once vital signs are stable, begin assisted ambulation and monitor bowel sounds, passage of gas, bowel movement, urinary retention, and leg swelling or pain. For breastfeeding, encourage early skin-to-skin contact and feeding on demand. Teach correct latch and positioning. For engorgement, apply warmth or gentle massage before feeding. If nipple pain occurs, adjust latch promptly. Localized breast redness, hard painful swelling, chills, or high fever should raise concern for mastitis. Because increased blood loss, anemia, or platelet abnormality is a concern, repeat a complete blood count as advised and watch for dizziness, palpitations, severe fatigue, pallor, sudden increase in lochia, or large clots. Iron supplementation, nutrition support, and assessment of uterine involution or coagulation should follow medical orders. Because fetal distress, meconium-stained fluid, or changed Apgar scores were recorded, cooperate with pediatric observation of feeding, skin color, temperature, breathing, and responsiveness. Poor feeding, cyanosis, excessive sleepiness, or abnormal breathing should be reported immediately. Before discharge, provide education on medication, wound care, breastfeeding, and contraception. Arrange a postpartum review around 42 days after delivery, or earlier if symptoms occur. Seek urgent medical care for sudden heavy vaginal bleeding, bright red bleeding soaking pads, foul-smelling lochia, fever, persistent abdominal pain, pus from the wound, difficulty urinating, unilateral leg swelling or pain, chest tightness, shortness of breath, or persistent sadness, anxiety, or thoughts of harming herself or the baby. | The patient is a 25-year-old G2P1 who underwent emergency cesarean section for suspected cord prolapse, fetal distress, grade III meconium. Blood loss 500 mL, newborn Apgar 8-9-10. Newborn – close monitoring for hypoxia and MAS; pediatric observation recommended. Watch for late PPH. Routine post-cesarean care. Psychological support. Follow-up: CBC at 1 week; newborn specialist follow-up. |
| **Clinical appropriateness 临床适当性** | 5 | 4 |
| **Individualization 个体化程度** | 4 | 5 |
| **Comprehensiveness 完整性** | 5 | 3 |
| **Actionability 可操作性** | 4 | 4 |
| **Safety 安全性** | 5 | 4 |
| **Triage appropriateness 分诊/转诊建议适当性** | 5 | 3 |
| **Structure and clarity 结构与清晰度** | 4 | 4 |

**病例 103 / Case 103**

| **项目 Item** | **ChatGPT 5.5** | **DeepSeek V3** |
| --- | --- | --- |
| **去标识化临床病例摘要 De-identified Clinical Case Summary** | 患者40岁，孕产史为G1P1，既往剖宫产1次，孕周38+6，临产类别为未临产。人工破膜，羊水量约260ml，羊水性质为清。以剖宫产分娩，产时出血约370ml。第一产程未进入产程，第二产程未进入第二产程，第三产程0时6分，总产程未进入产程，会阴或切口情况为完整。合并症及主要风险因素：胚胎移植术后；高龄初孕妇的监督；珍贵儿。新生儿出生体重3060g，Apgar评分1、5、10分钟分别为10、10、10分，分娩结局为活产。 | |
| **临床病例摘要英文翻译 English Translation of De-identified Clinical Case Summary** | Maternal age was 40 years. The obstetric history was G1P1, with 1 previous cesarean deliveries. Gestational age was 38+6 weeks, and the labor onset or induction category was no labor. The membranes were artificially ruptured; the amniotic fluid volume was approximately 260 mL and its character was clear. The patient underwent cesarean delivery, with estimated intrapartum blood loss of 370 mL. The first, second, and third stages of labor were labor was not established, the second stage was not reached, and 0 h 6 min, respectively; the total labor duration was labor was not established. The perineum was intact. Documented comorbidities and major risk factors were status post embryo transfer; advanced maternal age in a primigravida; precious pregnancy. Neonatal birth weight was 3060 g; Apgar scores at 1, 5, and 10 minutes were 10, 10, 10; and the delivery outcome was live birth. | |
| **模型生成的产褥期护理计划 Model-generated postpartum nursing care plan** | 产妇40岁，G1P1，孕38+6周，经剖宫产分娩，产时出血约370ml，新生儿女婴、体重3060g，Apgar评分10、10、10分。病例提示的重点风险为高龄妊娠因素。产后24小时内重点评估体温、脉搏、血压、宫底高度、宫缩、阴道流血、恶露颜色和尿潴留，记录首次排尿和疼痛评分。产后住院及出院后继续观察恶露是否由红色逐渐转淡，乳房是否胀痛，乳头是否皲裂，睡眠和情绪是否稳定。饮食以优质蛋白、含铁食物、蔬菜水果和足量饮水为主，避免盲目大补和长期卧床。本次为剖宫产，腹部切口和血栓预防是护理重点。切口应保持清洁干燥，观察红肿、渗血、渗液、裂开、异味和疼痛加重。翻身、咳嗽和下床时保护切口，在生命体征平稳后尽早床旁活动，关注肠鸣音恢复、排气排便、尿潴留和下肢肿痛。哺乳方面建议尽早母婴皮肤接触和按需哺乳，指导正确含接，乳胀时先热敷或轻柔按摩再哺乳，乳头疼痛时及时调整姿势。若出现乳房局部红肿硬痛、寒战或高热，应警惕乳腺炎。高龄产妇恢复期应避免过度劳累和连续熬夜，家属应参与夜间照护，保证睡眠、营养和情绪支持。出院前应完成用药、伤口、哺乳和避孕宣教，42天左右复查，必要时提前复诊。若出现阴道流血突然增多、鲜红血浸湿卫生巾、恶露恶臭、发热、持续腹痛、伤口化脓、排尿困难、下肢单侧肿痛、胸闷气短，或持续悲伤焦虑及伤害自己或婴儿的想法，应立即就医或急诊处理。 | 患者40岁，G1P1，剖宫产（择期），合并胚胎移植术后、高龄初产、珍贵儿，产时出血370ml。高龄初产剖宫产术后常规护理，注意血栓预防。珍贵儿父母焦虑，提供心理支持。鼓励母乳喂养。42天复查。 |
| **护理计划英文翻译 English translation of care plan** | The patient is 40 years old, G1P1, delivered at 38+6 weeks by cesarean delivery, with an estimated intrapartum blood loss of 370 mL. The newborn was a female infant, weighed 3060 g, and had Apgar scores of 10, 10, 10. The main case-based risks include advanced maternal age. Within the first 24 hours, assess temperature, pulse, blood pressure, uterine fundal height, uterine tone, vaginal bleeding, lochia color, urinary retention, first voiding, and pain score. During hospitalization and after discharge, continue to observe whether lochia gradually changes from red to lighter color, whether breast engorgement or nipple fissure occurs, and whether sleep and mood remain stable. Diet should include high-quality protein, iron-rich food, vegetables, fruit, and adequate fluids. Avoid blind over-supplementation and prolonged bed rest. Because this was a cesarean delivery, abdominal incision care and thrombosis prevention are key. Keep the incision clean and dry and observe for redness, swelling, bleeding, drainage, separation, odor, or worsening pain. Support the incision when turning, coughing, or getting out of bed. Once vital signs are stable, begin assisted ambulation and monitor bowel sounds, passage of gas, bowel movement, urinary retention, and leg swelling or pain. For breastfeeding, encourage early skin-to-skin contact and feeding on demand. Teach correct latch and positioning. For engorgement, apply warmth or gentle massage before feeding. If nipple pain occurs, adjust latch promptly. Localized breast redness, hard painful swelling, chills, or high fever should raise concern for mastitis. Advanced maternal age may slow recovery, so the family should help with nighttime care and support adequate sleep, nutrition, and emotional stability. Before discharge, provide education on medication, wound care, breastfeeding, and contraception. Arrange a postpartum review around 42 days after delivery, or earlier if symptoms occur. Seek urgent medical care for sudden heavy vaginal bleeding, bright red bleeding soaking pads, foul-smelling lochia, fever, persistent abdominal pain, pus from the wound, difficulty urinating, unilateral leg swelling or pain, chest tightness, shortness of breath, or persistent sadness, anxiety, or thoughts of harming herself or the baby. | The patient is a 40-year-old G1P1 who underwent elective cesarean section after embryo transfer (advanced maternal age in a primigravida, precious pregnancy). Blood loss 370 mL. Routine post-cesarean care with thrombosis prevention. Provide psychological support for parental anxiety. Encourage breastfeeding. Follow-up at 42 days. |
| **Clinical appropriateness 临床适当性** | 4 | 3 |
| **Individualization 个体化程度** | 3 | 3 |
| **Comprehensiveness 完整性** | 4 | 3 |
| **Actionability 可操作性** | 4 | 3 |
| **Safety 安全性** | 5 | 3 |
| **Triage appropriateness 分诊/转诊建议适当性** | 5 | 3 |
| **Structure and clarity 结构与清晰度** | 3 | 4 |

**病例 104 / Case 104**

| **项目 Item** | **ChatGPT 5.5** | **DeepSeek V3** |
| --- | --- | --- |
| **去标识化临床病例摘要 De-identified Clinical Case Summary** | 患者32岁，孕产史为G2P1，既往剖宫产1次，孕周37+5，临产类别为自然临产。自然破膜，羊水量约350ml，羊水性质为清。以剖宫产分娩，产时出血约380ml。第一产程4时50分，第二产程未进入第二产程，第三产程0时5分，总产程4时55分，会阴或切口情况为完整。合并症及主要风险因素：胎膜早破；臀位妊娠。新生儿出生体重2730g，Apgar评分1、5、10分钟分别为10、10、10分，分娩结局为活产。 | |
| **临床病例摘要英文翻译 English Translation of De-identified Clinical Case Summary** | Maternal age was 32 years. The obstetric history was G2P1, with 1 previous cesarean deliveries. Gestational age was 37+5 weeks, and the labor onset or induction category was spontaneous labor. The membranes ruptured spontaneously; the amniotic fluid volume was approximately 350 mL and its character was clear. The patient underwent cesarean delivery, with estimated intrapartum blood loss of 380 mL. The first, second, and third stages of labor were 4 h 50 min, the second stage was not reached, and 0 h 5 min, respectively; the total labor duration was 4 h 55 min. The perineum was intact. Documented comorbidities and major risk factors were premature rupture of membranes; breech pregnancy. Neonatal birth weight was 2730 g; Apgar scores at 1, 5, and 10 minutes were 10, 10, 10; and the delivery outcome was live birth. | |
| **模型生成的产褥期护理计划 Model-generated postpartum nursing care plan** | 产妇32岁，G2P1，孕37+5周，经剖宫产分娩，产时出血约380ml，新生儿男婴、体重2730g，Apgar评分10、10、10分。病例提示的重点风险为胎膜早破或感染相关风险。产后24小时内重点评估体温、脉搏、血压、宫底高度、宫缩、阴道流血、恶露颜色和尿潴留，记录首次排尿和疼痛评分。产后住院及出院后继续观察恶露是否由红色逐渐转淡，乳房是否胀痛，乳头是否皲裂，睡眠和情绪是否稳定。饮食以优质蛋白、含铁食物、蔬菜水果和足量饮水为主，避免盲目大补和长期卧床。本次为剖宫产，腹部切口和血栓预防是护理重点。切口应保持清洁干燥，观察红肿、渗血、渗液、裂开、异味和疼痛加重。翻身、咳嗽和下床时保护切口，在生命体征平稳后尽早床旁活动，关注肠鸣音恢复、排气排便、尿潴留和下肢肿痛。哺乳方面建议尽早母婴皮肤接触和按需哺乳，指导正确含接，乳胀时先热敷或轻柔按摩再哺乳，乳头疼痛时及时调整姿势。若出现乳房局部红肿硬痛、寒战或高热，应警惕乳腺炎。存在胎膜早破、B族链球菌感染、阴道炎或宫内感染疑虑时，应加强体温、下腹压痛、恶露气味、会阴或切口渗液观察，发热或恶露恶臭时及时复诊。出院前应完成用药、伤口、哺乳和避孕宣教，42天左右复查，必要时提前复诊。若出现阴道流血突然增多、鲜红血浸湿卫生巾、恶露恶臭、发热、持续腹痛、伤口化脓、排尿困难、下肢单侧肿痛、胸闷气短，或持续悲伤焦虑及伤害自己或婴儿的想法，应立即就医或急诊处理。 | 患者32岁，G2P1，急诊剖宫产，合并胎膜早破、臀位妊娠，产时出血380ml，新生儿Apgar 10。剖宫产术后常规护理。臀位新生儿需髋关节筛查。PPROM监测感染。出血380ml注意晚期出血。42天复查。 |
| **护理计划英文翻译 English translation of care plan** | The patient is 32 years old, G2P1, delivered at 37+5 weeks by cesarean delivery, with an estimated intrapartum blood loss of 380 mL. The newborn was a male infant, weighed 2730 g, and had Apgar scores of 10, 10, 10. The main case-based risks include premature rupture of membranes or infection-related risk. Within the first 24 hours, assess temperature, pulse, blood pressure, uterine fundal height, uterine tone, vaginal bleeding, lochia color, urinary retention, first voiding, and pain score. During hospitalization and after discharge, continue to observe whether lochia gradually changes from red to lighter color, whether breast engorgement or nipple fissure occurs, and whether sleep and mood remain stable. Diet should include high-quality protein, iron-rich food, vegetables, fruit, and adequate fluids. Avoid blind over-supplementation and prolonged bed rest. Because this was a cesarean delivery, abdominal incision care and thrombosis prevention are key. Keep the incision clean and dry and observe for redness, swelling, bleeding, drainage, separation, odor, or worsening pain. Support the incision when turning, coughing, or getting out of bed. Once vital signs are stable, begin assisted ambulation and monitor bowel sounds, passage of gas, bowel movement, urinary retention, and leg swelling or pain. For breastfeeding, encourage early skin-to-skin contact and feeding on demand. Teach correct latch and positioning. For engorgement, apply warmth or gentle massage before feeding. If nipple pain occurs, adjust latch promptly. Localized breast redness, hard painful swelling, chills, or high fever should raise concern for mastitis. With premature rupture of membranes, group B streptococcus, vaginitis, or suspected intrauterine infection, monitor temperature, lower abdominal tenderness, lochia odor, and drainage from the perineal wound or incision. Return promptly for fever or foul-smelling lochia. Before discharge, provide education on medication, wound care, breastfeeding, and contraception. Arrange a postpartum review around 42 days after delivery, or earlier if symptoms occur. Seek urgent medical care for sudden heavy vaginal bleeding, bright red bleeding soaking pads, foul-smelling lochia, fever, persistent abdominal pain, pus from the wound, difficulty urinating, unilateral leg swelling or pain, chest tightness, shortness of breath, or persistent sadness, anxiety, or thoughts of harming herself or the baby. | The patient is a 32-year-old G2P1 who underwent emergency cesarean section for PPROM and breech presentation. Blood loss 380 mL, newborn Apgar 10. Routine post-cesarean care. Newborn – hip ultrasound for DDH. Monitor infection. Watch for late PPH. Follow-up at 42 days. |
| **Clinical appropriateness 临床适当性** | 4 | 3 |
| **Individualization 个体化程度** | 4 | 3 |
| **Comprehensiveness 完整性** | 4 | 3 |
| **Actionability 可操作性** | 4 | 3 |
| **Safety 安全性** | 5 | 3 |
| **Triage appropriateness 分诊/转诊建议适当性** | 5 | 3 |
| **Structure and clarity 结构与清晰度** | 3 | 4 |

**病例 105 / Case 105**

| **项目 Item** | **ChatGPT 5.5** | **DeepSeek V3** |
| --- | --- | --- |
| **去标识化临床病例摘要 De-identified Clinical Case Summary** | 患者38岁，孕产史为G3P2，既往剖宫产1次，孕周39+2，临产类别为未临产。人工破膜，羊水量约300ml，羊水性质为清。以剖宫产分娩，产时出血约450ml。第一产程未进入产程，第二产程未进入第二产程，第三产程0时5分，总产程未进入产程，会阴或切口情况为完整。合并症及主要风险因素：妊娠合并子宫肌瘤；高龄经产妇妊娠监督。新生儿出生体重3180g，Apgar评分1、5、10分钟分别为10、10、10分，分娩结局为活产。 | |
| **临床病例摘要英文翻译 English Translation of De-identified Clinical Case Summary** | Maternal age was 38 years. The obstetric history was G3P2, with 1 previous cesarean deliveries. Gestational age was 39+2 weeks, and the labor onset or induction category was no labor. The membranes were artificially ruptured; the amniotic fluid volume was approximately 300 mL and its character was clear. The patient underwent cesarean delivery, with estimated intrapartum blood loss of 450 mL. The first, second, and third stages of labor were labor was not established, the second stage was not reached, and 0 h 5 min, respectively; the total labor duration was labor was not established. The perineum was intact. Documented comorbidities and major risk factors were uterine fibroid during pregnancy; advanced maternal age in a multipara. Neonatal birth weight was 3180 g; Apgar scores at 1, 5, and 10 minutes were 10, 10, 10; and the delivery outcome was live birth. | |
| **模型生成的产褥期护理计划 Model-generated postpartum nursing care plan** | 产妇38岁，G3P2，孕39+2周，经剖宫产分娩，产时出血约450ml，新生儿女婴、体重3180g，Apgar评分10、10、10分。病例提示的重点风险为高龄妊娠因素。产后24小时内重点评估体温、脉搏、血压、宫底高度、宫缩、阴道流血、恶露颜色和尿潴留，记录首次排尿和疼痛评分。产后住院及出院后继续观察恶露是否由红色逐渐转淡，乳房是否胀痛，乳头是否皲裂，睡眠和情绪是否稳定。饮食以优质蛋白、含铁食物、蔬菜水果和足量饮水为主，避免盲目大补和长期卧床。本次为剖宫产，腹部切口和血栓预防是护理重点。切口应保持清洁干燥，观察红肿、渗血、渗液、裂开、异味和疼痛加重。翻身、咳嗽和下床时保护切口，在生命体征平稳后尽早床旁活动，关注肠鸣音恢复、排气排便、尿潴留和下肢肿痛。哺乳方面建议尽早母婴皮肤接触和按需哺乳，指导正确含接，乳胀时先热敷或轻柔按摩再哺乳，乳头疼痛时及时调整姿势。若出现乳房局部红肿硬痛、寒战或高热，应警惕乳腺炎。产时出血量需要持续观察，建议每日记录恶露量、颜色和血块情况，活动后若明显增多，应减少活动并请医护评估。合并子宫肌瘤、卵巢囊肿或多囊卵巢相关情况时，产后复查时建议结合妇科超声评估，若腹痛、包块感或异常出血加重，应提前就诊。高龄产妇恢复期应避免过度劳累和连续熬夜，家属应参与夜间照护，保证睡眠、营养和情绪支持。出院前应完成用药、伤口、哺乳和避孕宣教，42天左右复查，必要时提前复诊。若出现阴道流血突然增多、鲜红血浸湿卫生巾、恶露恶臭、发热、持续腹痛、伤口化脓、排尿困难、下肢单侧肿痛、胸闷气短，或持续悲伤焦虑及伤害自己或婴儿的想法，应立即就医或急诊处理。 | 患者38岁，G3P2，剖宫产（择期），合并子宫肌瘤、高龄经产妇，产时出血450ml。剖宫产术中同时可行肌瘤剔除？未提。产后需监测子宫复旧，因肌瘤可能影响。高龄经产妇注意血栓预防。常规剖宫产护理。42天复查B超肌瘤。 |
| **护理计划英文翻译 English translation of care plan** | The patient is 38 years old, G3P2, delivered at 39+2 weeks by cesarean delivery, with an estimated intrapartum blood loss of 450 mL. The newborn was a female infant, weighed 3180 g, and had Apgar scores of 10, 10, 10. The main case-based risks include advanced maternal age. Within the first 24 hours, assess temperature, pulse, blood pressure, uterine fundal height, uterine tone, vaginal bleeding, lochia color, urinary retention, first voiding, and pain score. During hospitalization and after discharge, continue to observe whether lochia gradually changes from red to lighter color, whether breast engorgement or nipple fissure occurs, and whether sleep and mood remain stable. Diet should include high-quality protein, iron-rich food, vegetables, fruit, and adequate fluids. Avoid blind over-supplementation and prolonged bed rest. Because this was a cesarean delivery, abdominal incision care and thrombosis prevention are key. Keep the incision clean and dry and observe for redness, swelling, bleeding, drainage, separation, odor, or worsening pain. Support the incision when turning, coughing, or getting out of bed. Once vital signs are stable, begin assisted ambulation and monitor bowel sounds, passage of gas, bowel movement, urinary retention, and leg swelling or pain. For breastfeeding, encourage early skin-to-skin contact and feeding on demand. Teach correct latch and positioning. For engorgement, apply warmth or gentle massage before feeding. If nipple pain occurs, adjust latch promptly. Localized breast redness, hard painful swelling, chills, or high fever should raise concern for mastitis. Because blood loss requires closer observation, record the amount, color, and clots in the lochia every day. If bleeding increases clearly after activity, reduce activity and seek clinical assessment. For uterine fibroids, ovarian cysts, or polycystic ovary related findings, pelvic ultrasound can be considered at postpartum review. Earlier assessment is needed for worsening abdominal pain, mass sensation, or abnormal bleeding. Advanced maternal age may slow recovery, so the family should help with nighttime care and support adequate sleep, nutrition, and emotional stability. Before discharge, provide education on medication, wound care, breastfeeding, and contraception. Arrange a postpartum review around 42 days after delivery, or earlier if symptoms occur. Seek urgent medical care for sudden heavy vaginal bleeding, bright red bleeding soaking pads, foul-smelling lochia, fever, persistent abdominal pain, pus from the wound, difficulty urinating, unilateral leg swelling or pain, chest tightness, shortness of breath, or persistent sadness, anxiety, or thoughts of harming herself or the baby. | The patient is a 38-year-old G3P2 who underwent elective cesarean section for uterine fibroid and advanced age multipara. Blood loss 450 mL. Monitor uterine involution – fibroids may impair it. Thrombosis prevention. Routine post-cesarean care. Follow-up ultrasound for fibroid at 42 days. |
| **Clinical appropriateness 临床适当性** | 5 | 3 |
| **Individualization 个体化程度** | 4 | 3 |
| **Comprehensiveness 完整性** | 5 | 3 |
| **Actionability 可操作性** | 4 | 3 |
| **Safety 安全性** | 5 | 3 |
| **Triage appropriateness 分诊/转诊建议适当性** | 5 | 3 |
| **Structure and clarity 结构与清晰度** | 3 | 4 |

**病例 106 / Case 106**

| **项目 Item** | **ChatGPT 5.5** | **DeepSeek V3** |
| --- | --- | --- |
| **去标识化临床病例摘要 De-identified Clinical Case Summary** | 患者34岁，孕产史为G3P2，既往剖宫产2次，孕周39，临产类别为未临产。人工破膜，羊水量约240ml，羊水性质为清。以剖宫产分娩，产时出血约410ml。第一产程未进入产程，第二产程未进入第二产程，第三产程0时5分，总产程未进入产程，会阴或切口情况为完整。合并症及主要风险因素：瘢痕子宫；前次剖宫产术后再次妊娠。新生儿出生体重3310g，Apgar评分1、5、10分钟分别为10、10、10分，分娩结局为活产。 | |
| **临床病例摘要英文翻译 English Translation of De-identified Clinical Case Summary** | Maternal age was 34 years. The obstetric history was G3P2, with 2 previous cesarean deliveries. Gestational age was 39 weeks, and the labor onset or induction category was no labor. The membranes were artificially ruptured; the amniotic fluid volume was approximately 240 mL and its character was clear. The patient underwent cesarean delivery, with estimated intrapartum blood loss of 410 mL. The first, second, and third stages of labor were labor was not established, the second stage was not reached, and 0 h 5 min, respectively; the total labor duration was labor was not established. The perineum was intact. Documented comorbidities and major risk factors were scarred uterus; history of previous cesarean delivery in a subsequent pregnancy. Neonatal birth weight was 3310 g; Apgar scores at 1, 5, and 10 minutes were 10, 10, 10; and the delivery outcome was live birth. | |
| **模型生成的产褥期护理计划 Model-generated postpartum nursing care plan** | 产妇34岁，G3P2，孕39周，经剖宫产分娩，产时出血约410ml，新生儿男婴、体重3310g，Apgar评分10、10、10分。病例提示的重点风险为未记录特殊合并症。产后24小时内重点评估体温、脉搏、血压、宫底高度、宫缩、阴道流血、恶露颜色和尿潴留，记录首次排尿和疼痛评分。产后住院及出院后继续观察恶露是否由红色逐渐转淡，乳房是否胀痛，乳头是否皲裂，睡眠和情绪是否稳定。饮食以优质蛋白、含铁食物、蔬菜水果和足量饮水为主，避免盲目大补和长期卧床。本次为剖宫产，腹部切口和血栓预防是护理重点。切口应保持清洁干燥，观察红肿、渗血、渗液、裂开、异味和疼痛加重。翻身、咳嗽和下床时保护切口，在生命体征平稳后尽早床旁活动，关注肠鸣音恢复、排气排便、尿潴留和下肢肿痛。哺乳方面建议尽早母婴皮肤接触和按需哺乳，指导正确含接，乳胀时先热敷或轻柔按摩再哺乳，乳头疼痛时及时调整姿势。若出现乳房局部红肿硬痛、寒战或高热，应警惕乳腺炎。产时出血量需要持续观察，建议每日记录恶露量、颜色和血块情况，活动后若明显增多，应减少活动并请医护评估。瘢痕子宫或再次剖宫产者，应特别关注切口疼痛、恶露异常和子宫复旧，产后复查时结合病情评估子宫切口恢复。出院前应完成用药、伤口、哺乳和避孕宣教，42天左右复查，必要时提前复诊。若出现阴道流血突然增多、鲜红血浸湿卫生巾、恶露恶臭、发热、持续腹痛、伤口化脓、排尿困难、下肢单侧肿痛、胸闷气短，或持续悲伤焦虑及伤害自己或婴儿的想法，应立即就医或急诊处理。 | 患者34岁，G3P2，剖宫产（择期），合并瘢痕子宫（2次剖宫产），产时出血410ml。两次剖宫产后子宫瘢痕较薄弱，需注意术后腹痛及出血。常规剖宫产护理。建议永久性避孕或LARC。42天复查。 |
| **护理计划英文翻译 English translation of care plan** | The patient is 34 years old, G3P2, delivered at 39 weeks by cesarean delivery, with an estimated intrapartum blood loss of 410 mL. The newborn was a male infant, weighed 3310 g, and had Apgar scores of 10, 10, 10. The main case-based risks include no special comorbidity was recorded. Within the first 24 hours, assess temperature, pulse, blood pressure, uterine fundal height, uterine tone, vaginal bleeding, lochia color, urinary retention, first voiding, and pain score. During hospitalization and after discharge, continue to observe whether lochia gradually changes from red to lighter color, whether breast engorgement or nipple fissure occurs, and whether sleep and mood remain stable. Diet should include high-quality protein, iron-rich food, vegetables, fruit, and adequate fluids. Avoid blind over-supplementation and prolonged bed rest. Because this was a cesarean delivery, abdominal incision care and thrombosis prevention are key. Keep the incision clean and dry and observe for redness, swelling, bleeding, drainage, separation, odor, or worsening pain. Support the incision when turning, coughing, or getting out of bed. Once vital signs are stable, begin assisted ambulation and monitor bowel sounds, passage of gas, bowel movement, urinary retention, and leg swelling or pain. For breastfeeding, encourage early skin-to-skin contact and feeding on demand. Teach correct latch and positioning. For engorgement, apply warmth or gentle massage before feeding. If nipple pain occurs, adjust latch promptly. Localized breast redness, hard painful swelling, chills, or high fever should raise concern for mastitis. Because blood loss requires closer observation, record the amount, color, and clots in the lochia every day. If bleeding increases clearly after activity, reduce activity and seek clinical assessment. For scarred uterus or repeat cesarean delivery, pay close attention to incision pain, abnormal lochia, and uterine involution. Uterine scar recovery should be reviewed during postpartum follow-up when indicated. Before discharge, provide education on medication, wound care, breastfeeding, and contraception. Arrange a postpartum review around 42 days after delivery, or earlier if symptoms occur. Seek urgent medical care for sudden heavy vaginal bleeding, bright red bleeding soaking pads, foul-smelling lochia, fever, persistent abdominal pain, pus from the wound, difficulty urinating, unilateral leg swelling or pain, chest tightness, shortness of breath, or persistent sadness, anxiety, or thoughts of harming herself or the baby. | The patient is a 34-year-old G3P2 who underwent repeat cesarean section (2 prior cesareans). Blood loss 410 mL. Monitor for abnormal pain or bleeding due to thin scar. Routine post-cesarean care. Recommend permanent contraception or LARC. Follow-up at 42 days. |
| **Clinical appropriateness 临床适当性** | 5 | 3 |
| **Individualization 个体化程度** | 3 | 3 |
| **Comprehensiveness 完整性** | 5 | 3 |
| **Actionability 可操作性** | 4 | 3 |
| **Safety 安全性** | 5 | 3 |
| **Triage appropriateness 分诊/转诊建议适当性** | 5 | 3 |
| **Structure and clarity 结构与清晰度** | 3 | 4 |

**病例 107 / Case 107**

| **项目 Item** | **ChatGPT 5.5** | **DeepSeek V3** |
| --- | --- | --- |
| **去标识化临床病例摘要 De-identified Clinical Case Summary** | 患者29岁，孕产史为G1P1，既往剖宫产1次，孕周40+1，临产类别为自然临产。自然破膜，羊水量约180ml，羊水性质为Ⅲ°（质粘稠）。以剖宫产分娩，产时出血约470ml。第一产程4时05分，第二产程未进入第二产程，第三产程0时4分，总产程4时09分，会阴或切口情况为完整。合并症及主要风险因素：胎儿宫内窘迫？羊水Ⅲ°污染。新生儿出生体重3220g，Apgar评分1、5、10分钟分别为8、9、10分，分娩结局为活产。 | |
| **临床病例摘要英文翻译 English Translation of De-identified Clinical Case Summary** | Maternal age was 29 years. The obstetric history was G1P1, with 1 previous cesarean deliveries. Gestational age was 40+1 weeks, and the labor onset or induction category was spontaneous labor. The membranes ruptured spontaneously; the amniotic fluid volume was approximately 180 mL and its character was thick grade III meconium-stained. The patient underwent cesarean delivery, with estimated intrapartum blood loss of 470 mL. The first, second, and third stages of labor were 4 h 5 min, the second stage was not reached, and 0 h 4 min, respectively; the total labor duration was 4 h 9 min. The perineum was intact. Documented comorbidities and major risk factors were suspected fetal distress; grade III meconium-stained amniotic fluid. Neonatal birth weight was 3220 g; Apgar scores at 1, 5, and 10 minutes were 8, 9, 10; and the delivery outcome was live birth. | |
| **模型生成的产褥期护理计划 Model-generated postpartum nursing care plan** | 产妇29岁，G1P1，孕40+1周，经剖宫产分娩，产时出血约470ml，新生儿女婴、体重3220g，Apgar评分8、9、10分。病例提示的重点风险为胎儿窘迫或羊水异常记录。产后24小时内重点评估体温、脉搏、血压、宫底高度、宫缩、阴道流血、恶露颜色和尿潴留，记录首次排尿和疼痛评分。产后住院及出院后继续观察恶露是否由红色逐渐转淡，乳房是否胀痛，乳头是否皲裂，睡眠和情绪是否稳定。饮食以优质蛋白、含铁食物、蔬菜水果和足量饮水为主，避免盲目大补和长期卧床。本次为剖宫产，腹部切口和血栓预防是护理重点。切口应保持清洁干燥，观察红肿、渗血、渗液、裂开、异味和疼痛加重。翻身、咳嗽和下床时保护切口，在生命体征平稳后尽早床旁活动，关注肠鸣音恢复、排气排便、尿潴留和下肢肿痛。哺乳方面建议尽早母婴皮肤接触和按需哺乳，指导正确含接，乳胀时先热敷或轻柔按摩再哺乳，乳头疼痛时及时调整姿势。若出现乳房局部红肿硬痛、寒战或高热，应警惕乳腺炎。产时出血量需要持续观察，建议每日记录恶露量、颜色和血块情况，活动后若明显增多，应减少活动并请医护评估。因有胎儿窘迫、羊水污染或新生儿评分变化记录，母婴同室期间应配合观察新生儿吃奶、肤色、体温、呼吸和反应，发现吃奶差、发绀、嗜睡或呼吸异常应立即告知儿科医生。出院前应完成用药、伤口、哺乳和避孕宣教，42天左右复查，必要时提前复诊。若出现阴道流血突然增多、鲜红血浸湿卫生巾、恶露恶臭、发热、持续腹痛、伤口化脓、排尿困难、下肢单侧肿痛、胸闷气短，或持续悲伤焦虑及伤害自己或婴儿的想法，应立即就医或急诊处理。 | 患者29岁，G1P1，急诊剖宫产，合并可疑胎儿窘迫、羊水Ⅲ°污染，产时出血470ml，新生儿Apgar 8-9-10。Ⅲ°羊水+窘迫，新生儿监测MAS及HIE。出血470ml注意晚期出血。剖宫产术后常规护理。心理支持。产后1周复查血常规；新生儿专科随访。 |
| **护理计划英文翻译 English translation of care plan** | The patient is 29 years old, G1P1, delivered at 40+1 weeks by cesarean delivery, with an estimated intrapartum blood loss of 470 mL. The newborn was a female infant, weighed 3220 g, and had Apgar scores of 8, 9, 10. The main case-based risks include fetal distress or abnormal amniotic fluid findings. Within the first 24 hours, assess temperature, pulse, blood pressure, uterine fundal height, uterine tone, vaginal bleeding, lochia color, urinary retention, first voiding, and pain score. During hospitalization and after discharge, continue to observe whether lochia gradually changes from red to lighter color, whether breast engorgement or nipple fissure occurs, and whether sleep and mood remain stable. Diet should include high-quality protein, iron-rich food, vegetables, fruit, and adequate fluids. Avoid blind over-supplementation and prolonged bed rest. Because this was a cesarean delivery, abdominal incision care and thrombosis prevention are key. Keep the incision clean and dry and observe for redness, swelling, bleeding, drainage, separation, odor, or worsening pain. Support the incision when turning, coughing, or getting out of bed. Once vital signs are stable, begin assisted ambulation and monitor bowel sounds, passage of gas, bowel movement, urinary retention, and leg swelling or pain. For breastfeeding, encourage early skin-to-skin contact and feeding on demand. Teach correct latch and positioning. For engorgement, apply warmth or gentle massage before feeding. If nipple pain occurs, adjust latch promptly. Localized breast redness, hard painful swelling, chills, or high fever should raise concern for mastitis. Because blood loss requires closer observation, record the amount, color, and clots in the lochia every day. If bleeding increases clearly after activity, reduce activity and seek clinical assessment. Because fetal distress, meconium-stained fluid, or changed Apgar scores were recorded, cooperate with pediatric observation of feeding, skin color, temperature, breathing, and responsiveness. Poor feeding, cyanosis, excessive sleepiness, or abnormal breathing should be reported immediately. Before discharge, provide education on medication, wound care, breastfeeding, and contraception. Arrange a postpartum review around 42 days after delivery, or earlier if symptoms occur. Seek urgent medical care for sudden heavy vaginal bleeding, bright red bleeding soaking pads, foul-smelling lochia, fever, persistent abdominal pain, pus from the wound, difficulty urinating, unilateral leg swelling or pain, chest tightness, shortness of breath, or persistent sadness, anxiety, or thoughts of harming herself or the baby. | The patient is a 29-year-old G1P1 who underwent emergency cesarean section for suspected fetal distress and grade III meconium. Blood loss 470 mL, newborn Apgar 8-9-10. Newborn monitor for MAS and HIE. Watch for late PPH. Routine post-cesarean care. Psychological support. Follow-up: CBC at 1 week; newborn specialist follow-up. |
| **Clinical appropriateness 临床适当性** | 5 | 3 |
| **Individualization 个体化程度** | 4 | 4 |
| **Comprehensiveness 完整性** | 5 | 3 |
| **Actionability 可操作性** | 4 | 4 |
| **Safety 安全性** | 5 | 3 |
| **Triage appropriateness 分诊/转诊建议适当性** | 5 | 3 |
| **Structure and clarity 结构与清晰度** | 3 | 4 |

**病例 108 / Case 108**

| **项目 Item** | **ChatGPT 5.5** | **DeepSeek V3** |
| --- | --- | --- |
| **去标识化临床病例摘要 De-identified Clinical Case Summary** | 患者35岁，孕产史为G2P1，既往剖宫产1次，孕周38+2，临产类别为未临产。人工破膜，羊水量约220ml，羊水性质为清。以剖宫产分娩，产时出血约430ml。第一产程未进入产程，第二产程未进入第二产程，第三产程0时6分，总产程未进入产程，会阴或切口情况为完整。合并症及主要风险因素：妊娠期高血压；高龄初孕妇的监督。新生儿出生体重2890g，Apgar评分1、5、10分钟分别为10、10、10分，分娩结局为活产。 | |
| **临床病例摘要英文翻译 English Translation of De-identified Clinical Case Summary** | Maternal age was 35 years. The obstetric history was G2P1, with 1 previous cesarean deliveries. Gestational age was 38+2 weeks, and the labor onset or induction category was no labor. The membranes were artificially ruptured; the amniotic fluid volume was approximately 220 mL and its character was clear. The patient underwent cesarean delivery, with estimated intrapartum blood loss of 430 mL. The first, second, and third stages of labor were labor was not established, the second stage was not reached, and 0 h 6 min, respectively; the total labor duration was labor was not established. The perineum was intact. Documented comorbidities and major risk factors were gestational hypertension; advanced maternal age in a primigravida. Neonatal birth weight was 2890 g; Apgar scores at 1, 5, and 10 minutes were 10, 10, 10; and the delivery outcome was live birth. | |
| **模型生成的产褥期护理计划 Model-generated postpartum nursing care plan** | 产妇35岁，G2P1，孕38+2周，经剖宫产分娩，产时出血约430ml，新生儿男婴、体重2890g，Apgar评分10、10、10分。病例提示的重点风险为妊娠期高血压或子痫前期相关风险、高龄妊娠因素。产后24小时内重点评估体温、脉搏、血压、宫底高度、宫缩、阴道流血、恶露颜色和尿潴留，记录首次排尿和疼痛评分。产后住院及出院后继续观察恶露是否由红色逐渐转淡，乳房是否胀痛，乳头是否皲裂，睡眠和情绪是否稳定。饮食以优质蛋白、含铁食物、蔬菜水果和足量饮水为主，避免盲目大补和长期卧床。本次为剖宫产，腹部切口和血栓预防是护理重点。切口应保持清洁干燥，观察红肿、渗血、渗液、裂开、异味和疼痛加重。翻身、咳嗽和下床时保护切口，在生命体征平稳后尽早床旁活动，关注肠鸣音恢复、排气排便、尿潴留和下肢肿痛。哺乳方面建议尽早母婴皮肤接触和按需哺乳，指导正确含接，乳胀时先热敷或轻柔按摩再哺乳，乳头疼痛时及时调整姿势。若出现乳房局部红肿硬痛、寒战或高热，应警惕乳腺炎。产时出血量需要持续观察，建议每日记录恶露量、颜色和血块情况，活动后若明显增多，应减少活动并请医护评估。有高血压或子痫前期风险者，产后仍可能出现血压波动，应按医嘱监测血压，若持续头痛、眼花、胸闷、上腹痛、抽搐或水肿加重，应立即就医。高龄产妇恢复期应避免过度劳累和连续熬夜，家属应参与夜间照护，保证睡眠、营养和情绪支持。出院前应完成用药、伤口、哺乳和避孕宣教，42天左右复查，必要时提前复诊。若出现阴道流血突然增多、鲜红血浸湿卫生巾、恶露恶臭、发热、持续腹痛、伤口化脓、排尿困难、下肢单侧肿痛、胸闷气短，或持续悲伤焦虑及伤害自己或婴儿的想法，应立即就医或急诊处理。 | 患者35岁，G2P1，剖宫产（择期），合并妊娠期高血压、高龄初产，产时出血430ml。产后监测血压，继续降压治疗。高龄初产注意血栓预防。常规剖宫产护理。42天复查血压。 |
| **护理计划英文翻译 English translation of care plan** | The patient is 35 years old, G2P1, delivered at 38+2 weeks by cesarean delivery, with an estimated intrapartum blood loss of 430 mL. The newborn was a male infant, weighed 2890 g, and had Apgar scores of 10, 10, 10. The main case-based risks include gestational hypertension or preeclampsia-related risk, advanced maternal age. Within the first 24 hours, assess temperature, pulse, blood pressure, uterine fundal height, uterine tone, vaginal bleeding, lochia color, urinary retention, first voiding, and pain score. During hospitalization and after discharge, continue to observe whether lochia gradually changes from red to lighter color, whether breast engorgement or nipple fissure occurs, and whether sleep and mood remain stable. Diet should include high-quality protein, iron-rich food, vegetables, fruit, and adequate fluids. Avoid blind over-supplementation and prolonged bed rest. Because this was a cesarean delivery, abdominal incision care and thrombosis prevention are key. Keep the incision clean and dry and observe for redness, swelling, bleeding, drainage, separation, odor, or worsening pain. Support the incision when turning, coughing, or getting out of bed. Once vital signs are stable, begin assisted ambulation and monitor bowel sounds, passage of gas, bowel movement, urinary retention, and leg swelling or pain. For breastfeeding, encourage early skin-to-skin contact and feeding on demand. Teach correct latch and positioning. For engorgement, apply warmth or gentle massage before feeding. If nipple pain occurs, adjust latch promptly. Localized breast redness, hard painful swelling, chills, or high fever should raise concern for mastitis. Because blood loss requires closer observation, record the amount, color, and clots in the lochia every day. If bleeding increases clearly after activity, reduce activity and seek clinical assessment. For hypertension or preeclampsia risk, postpartum blood pressure may still fluctuate. Continue blood pressure monitoring as advised and seek urgent care for persistent headache, visual symptoms, chest tightness, upper abdominal pain, seizure, or worsening edema. Advanced maternal age may slow recovery, so the family should help with nighttime care and support adequate sleep, nutrition, and emotional stability. Before discharge, provide education on medication, wound care, breastfeeding, and contraception. Arrange a postpartum review around 42 days after delivery, or earlier if symptoms occur. Seek urgent medical care for sudden heavy vaginal bleeding, bright red bleeding soaking pads, foul-smelling lochia, fever, persistent abdominal pain, pus from the wound, difficulty urinating, unilateral leg swelling or pain, chest tightness, shortness of breath, or persistent sadness, anxiety, or thoughts of harming herself or the baby. | The patient is a 35-year-old G2P1 who underwent elective cesarean section for gestational hypertension and advanced maternal age in a primigravida. Blood loss 430 mL. Monitor BP, continue antihypertensives. Thrombosis prevention. Routine post-cesarean care. Follow-up BP at 42 days. |
| **Clinical appropriateness 临床适当性** | 5 | 3 |
| **Individualization 个体化程度** | 5 | 3 |
| **Comprehensiveness 完整性** | 5 | 3 |
| **Actionability 可操作性** | 4 | 3 |
| **Safety 安全性** | 5 | 3 |
| **Triage appropriateness 分诊/转诊建议适当性** | 5 | 3 |
| **Structure and clarity 结构与清晰度** | 3 | 4 |

**病例 109 / Case 109**

| **项目 Item** | **ChatGPT 5.5** | **DeepSeek V3** |
| --- | --- | --- |
| **去标识化临床病例摘要 De-identified Clinical Case Summary** | 患者39岁，孕产史为G4P2，既往剖宫产2次，孕周38+4，临产类别为未临产。人工破膜，羊水量约200ml，羊水性质为清。以剖宫产分娩，产时出血约540ml。第一产程未进入产程，第二产程未进入第二产程，第三产程0时5分，总产程未进入产程，会阴或切口情况为完整。合并症及主要风险因素：瘢痕子宫；胎盘低置状态；高龄经产妇妊娠监督。新生儿出生体重3010g，Apgar评分1、5、10分钟分别为10、10、10分，分娩结局为活产。 | |
| **临床病例摘要英文翻译 English Translation of De-identified Clinical Case Summary** | Maternal age was 39 years. The obstetric history was G4P2, with 2 previous cesarean deliveries. Gestational age was 38+4 weeks, and the labor onset or induction category was no labor. The membranes were artificially ruptured; the amniotic fluid volume was approximately 200 mL and its character was clear. The patient underwent cesarean delivery, with estimated intrapartum blood loss of 540 mL. The first, second, and third stages of labor were labor was not established, the second stage was not reached, and 0 h 5 min, respectively; the total labor duration was labor was not established. The perineum was intact. Documented comorbidities and major risk factors were scarred uterus; low-lying placenta; advanced maternal age in a multipara. Neonatal birth weight was 3010 g; Apgar scores at 1, 5, and 10 minutes were 10, 10, 10; and the delivery outcome was live birth. | |
| **模型生成的产褥期护理计划 Model-generated postpartum nursing care plan** | 产妇39岁，G4P2，孕38+4周，经剖宫产分娩，产时出血约540ml，新生儿女婴、体重3010g，Apgar评分10、10、10分。病例提示的重点风险为出血或贫血相关风险、胎盘相关风险、高龄妊娠因素。产后24小时内重点评估体温、脉搏、血压、宫底高度、宫缩、阴道流血、恶露颜色和尿潴留，记录首次排尿和疼痛评分。产后住院及出院后继续观察恶露是否由红色逐渐转淡，乳房是否胀痛，乳头是否皲裂，睡眠和情绪是否稳定。饮食以优质蛋白、含铁食物、蔬菜水果和足量饮水为主，避免盲目大补和长期卧床。本次为剖宫产，腹部切口和血栓预防是护理重点。切口应保持清洁干燥，观察红肿、渗血、渗液、裂开、异味和疼痛加重。翻身、咳嗽和下床时保护切口，在生命体征平稳后尽早床旁活动，关注肠鸣音恢复、排气排便、尿潴留和下肢肿痛。哺乳方面建议尽早母婴皮肤接触和按需哺乳，指导正确含接，乳胀时先热敷或轻柔按摩再哺乳，乳头疼痛时及时调整姿势。若出现乳房局部红肿硬痛、寒战或高热，应警惕乳腺炎。因存在出血量偏多、贫血或血小板异常相关风险，应复查血常规，关注头晕、心悸、乏力、面色苍白、恶露突然增多或较大血块，按医嘱补铁，必要时评估凝血和子宫复旧。瘢痕子宫或再次剖宫产者，应特别关注切口疼痛、恶露异常和子宫复旧，产后复查时结合病情评估子宫切口恢复。有胎盘位置、形态或胎盘血窦相关记录时，应注意恶露持续时间和出血量，产后复查可结合B超了解宫腔及子宫复旧。高龄产妇恢复期应避免过度劳累和连续熬夜，家属应参与夜间照护，保证睡眠、营养和情绪支持。出院前应完成用药、伤口、哺乳和避孕宣教，42天左右复查，必要时提前复诊。若出现阴道流血突然增多、鲜红血浸湿卫生巾、恶露恶臭、发热、持续腹痛、伤口化脓、排尿困难、下肢单侧肿痛、胸闷气短，或持续悲伤焦虑及伤害自己或婴儿的想法，应立即就医或急诊处理。 | 患者39岁，G4P2，剖宫产（择期），合并瘢痕子宫、胎盘低置状态、高龄经产妇，产时出血540ml。胎盘低置剖宫产出血风险高，需监测血红蛋白，补铁。瘢痕子宫注意切口愈合。高龄经产妇血栓预防。常规剖宫产护理。42天复查B超。 |
| **护理计划英文翻译 English translation of care plan** | The patient is 39 years old, G4P2, delivered at 38+4 weeks by cesarean delivery, with an estimated intrapartum blood loss of 540 mL. The newborn was a female infant, weighed 3010 g, and had Apgar scores of 10, 10, 10. The main case-based risks include bleeding or anemia-related risk, placental risk factors, advanced maternal age. Within the first 24 hours, assess temperature, pulse, blood pressure, uterine fundal height, uterine tone, vaginal bleeding, lochia color, urinary retention, first voiding, and pain score. During hospitalization and after discharge, continue to observe whether lochia gradually changes from red to lighter color, whether breast engorgement or nipple fissure occurs, and whether sleep and mood remain stable. Diet should include high-quality protein, iron-rich food, vegetables, fruit, and adequate fluids. Avoid blind over-supplementation and prolonged bed rest. Because this was a cesarean delivery, abdominal incision care and thrombosis prevention are key. Keep the incision clean and dry and observe for redness, swelling, bleeding, drainage, separation, odor, or worsening pain. Support the incision when turning, coughing, or getting out of bed. Once vital signs are stable, begin assisted ambulation and monitor bowel sounds, passage of gas, bowel movement, urinary retention, and leg swelling or pain. For breastfeeding, encourage early skin-to-skin contact and feeding on demand. Teach correct latch and positioning. For engorgement, apply warmth or gentle massage before feeding. If nipple pain occurs, adjust latch promptly. Localized breast redness, hard painful swelling, chills, or high fever should raise concern for mastitis. Because increased blood loss, anemia, or platelet abnormality is a concern, repeat a complete blood count as advised and watch for dizziness, palpitations, severe fatigue, pallor, sudden increase in lochia, or large clots. Iron supplementation, nutrition support, and assessment of uterine involution or coagulation should follow medical orders. For scarred uterus or repeat cesarean delivery, pay close attention to incision pain, abnormal lochia, and uterine involution. Uterine scar recovery should be reviewed during postpartum follow-up when indicated. For abnormal placental location, shape, or placental sinus records, monitor bleeding amount and duration of lochia. Ultrasound review of the uterine cavity and involution may be needed at follow-up. Advanced maternal age may slow recovery, so the family should help with nighttime care and support adequate sleep, nutrition, and emotional stability. Before discharge, provide education on medication, wound care, breastfeeding, and contraception. Arrange a postpartum review around 42 days after delivery, or earlier if symptoms occur. Seek urgent medical care for sudden heavy vaginal bleeding, bright red bleeding soaking pads, foul-smelling lochia, fever, persistent abdominal pain, pus from the wound, difficulty urinating, unilateral leg swelling or pain, chest tightness, shortness of breath, or persistent sadness, anxiety, or thoughts of harming herself or the baby. | The patient is a 39-year-old G4P2 who underwent cesarean section for scarred uterus and low-lying placenta. Blood loss 540 mL. High bleeding risk – monitor Hb, iron supplementation. Scarred uterus – monitor incision healing. Thrombosis prevention. Routine post-cesarean care. Follow-up ultrasound at 42 days. |
| **Clinical appropriateness 临床适当性** | 5 | 3 |
| **Individualization 个体化程度** | 5 | 3 |
| **Comprehensiveness 完整性** | 5 | 3 |
| **Actionability 可操作性** | 4 | 3 |
| **Safety 安全性** | 5 | 3 |
| **Triage appropriateness 分诊/转诊建议适当性** | 5 | 3 |
| **Structure and clarity 结构与清晰度** | 3 | 3 |

**病例 110 / Case 110**

| **项目 Item** | **ChatGPT 5.5** | **DeepSeek V3** |
| --- | --- | --- |
| **去标识化临床病例摘要 De-identified Clinical Case Summary** | 患者31岁，孕产史为G1P1，既往剖宫产1次，孕周41+1，临产类别为普贝生。人工破膜，羊水量约250ml，羊水性质为Ⅱ°。以剖宫产分娩，产时出血约440ml。第一产程8时10分，第二产程未进入第二产程，第三产程0时5分，总产程8时15分，会阴或切口情况为完整。合并症及主要风险因素：延期妊娠；引产失败；胎儿窘迫？。新生儿出生体重3400g，Apgar评分1、5、10分钟分别为9、10、10分，分娩结局为活产。 | |
| **临床病例摘要英文翻译 English Translation of De-identified Clinical Case Summary** | Maternal age was 31 years. The obstetric history was G1P1, with 1 previous cesarean deliveries. Gestational age was 41+1 weeks, and the labor onset or induction category was vaginal dinoprostone (Propess) induction. The membranes were artificially ruptured; the amniotic fluid volume was approximately 250 mL and its character was grade II meconium-stained. The patient underwent cesarean delivery, with estimated intrapartum blood loss of 440 mL. The first, second, and third stages of labor were 8 h 10 min, the second stage was not reached, and 0 h 5 min, respectively; the total labor duration was 8 h 15 min. The perineum was intact. Documented comorbidities and major risk factors were post-term pregnancy; failed induction; suspected fetal distress. Neonatal birth weight was 3400 g; Apgar scores at 1, 5, and 10 minutes were 9, 10, 10; and the delivery outcome was live birth. | |
| **模型生成的产褥期护理计划 Model-generated postpartum nursing care plan** | 产妇31岁，G1P1，孕41+1周，经剖宫产分娩，产时出血约440ml，新生儿男婴、体重3400g，Apgar评分9、10、10分。病例提示的重点风险为胎儿窘迫或羊水异常记录。产后24小时内重点评估体温、脉搏、血压、宫底高度、宫缩、阴道流血、恶露颜色和尿潴留，记录首次排尿和疼痛评分。产后住院及出院后继续观察恶露是否由红色逐渐转淡，乳房是否胀痛，乳头是否皲裂，睡眠和情绪是否稳定。饮食以优质蛋白、含铁食物、蔬菜水果和足量饮水为主，避免盲目大补和长期卧床。本次为剖宫产，腹部切口和血栓预防是护理重点。切口应保持清洁干燥，观察红肿、渗血、渗液、裂开、异味和疼痛加重。翻身、咳嗽和下床时保护切口，在生命体征平稳后尽早床旁活动，关注肠鸣音恢复、排气排便、尿潴留和下肢肿痛。哺乳方面建议尽早母婴皮肤接触和按需哺乳，指导正确含接，乳胀时先热敷或轻柔按摩再哺乳，乳头疼痛时及时调整姿势。若出现乳房局部红肿硬痛、寒战或高热，应警惕乳腺炎。产时出血量需要持续观察，建议每日记录恶露量、颜色和血块情况，活动后若明显增多，应减少活动并请医护评估。因有胎儿窘迫、羊水污染或新生儿评分变化记录，母婴同室期间应配合观察新生儿吃奶、肤色、体温、呼吸和反应，发现吃奶差、发绀、嗜睡或呼吸异常应立即告知儿科医生。出院前应完成用药、伤口、哺乳和避孕宣教，42天左右复查，必要时提前复诊。若出现阴道流血突然增多、鲜红血浸湿卫生巾、恶露恶臭、发热、持续腹痛、伤口化脓、排尿困难、下肢单侧肿痛、胸闷气短，或持续悲伤焦虑及伤害自己或婴儿的想法，应立即就医或急诊处理。 | 患者31岁，G1P1，剖宫产（引产失败），合并延期妊娠、可疑胎儿窘迫，羊水Ⅱ°污染，产时出血440ml，新生儿Apgar 9-10-10。延期妊娠+引产失败+Ⅱ°羊水，新生儿需监测MAS及窘迫后遗症。剖宫产术后常规护理。出血440ml注意晚期出血。42天复查。 |
| **护理计划英文翻译 English translation of care plan** | The patient is 31 years old, G1P1, delivered at 41+1 weeks by cesarean delivery, with an estimated intrapartum blood loss of 440 mL. The newborn was a male infant, weighed 3400 g, and had Apgar scores of 9, 10, 10. The main case-based risks include fetal distress or abnormal amniotic fluid findings. Within the first 24 hours, assess temperature, pulse, blood pressure, uterine fundal height, uterine tone, vaginal bleeding, lochia color, urinary retention, first voiding, and pain score. During hospitalization and after discharge, continue to observe whether lochia gradually changes from red to lighter color, whether breast engorgement or nipple fissure occurs, and whether sleep and mood remain stable. Diet should include high-quality protein, iron-rich food, vegetables, fruit, and adequate fluids. Avoid blind over-supplementation and prolonged bed rest. Because this was a cesarean delivery, abdominal incision care and thrombosis prevention are key. Keep the incision clean and dry and observe for redness, swelling, bleeding, drainage, separation, odor, or worsening pain. Support the incision when turning, coughing, or getting out of bed. Once vital signs are stable, begin assisted ambulation and monitor bowel sounds, passage of gas, bowel movement, urinary retention, and leg swelling or pain. For breastfeeding, encourage early skin-to-skin contact and feeding on demand. Teach correct latch and positioning. For engorgement, apply warmth or gentle massage before feeding. If nipple pain occurs, adjust latch promptly. Localized breast redness, hard painful swelling, chills, or high fever should raise concern for mastitis. Because blood loss requires closer observation, record the amount, color, and clots in the lochia every day. If bleeding increases clearly after activity, reduce activity and seek clinical assessment. Because fetal distress, meconium-stained fluid, or changed Apgar scores were recorded, cooperate with pediatric observation of feeding, skin color, temperature, breathing, and responsiveness. Poor feeding, cyanosis, excessive sleepiness, or abnormal breathing should be reported immediately. Before discharge, provide education on medication, wound care, breastfeeding, and contraception. Arrange a postpartum review around 42 days after delivery, or earlier if symptoms occur. Seek urgent medical care for sudden heavy vaginal bleeding, bright red bleeding soaking pads, foul-smelling lochia, fever, persistent abdominal pain, pus from the wound, difficulty urinating, unilateral leg swelling or pain, chest tightness, shortness of breath, or persistent sadness, anxiety, or thoughts of harming herself or the baby. | The patient is a 31-year-old G1P1 who underwent cesarean section for failed induction, post-term pregnancy, suspected fetal distress, grade II meconium. Blood loss 440 mL, newborn Apgar 9-10-10. Newborn monitor for MAS and sequelae of distress. Routine post-cesarean care. Watch for late PPH. Follow-up at 42 days. |
| **Clinical appropriateness 临床适当性** | 5 | 4 |
| **Individualization 个体化程度** | 4 | 4 |
| **Comprehensiveness 完整性** | 5 | 3 |
| **Actionability 可操作性** | 4 | 3 |
| **Safety 安全性** | 5 | 4 |
| **Triage appropriateness 分诊/转诊建议适当性** | 5 | 3 |
| **Structure and clarity 结构与清晰度** | 4 | 4 |

**病例 111 / Case 111**

| **项目 Item** | **ChatGPT 5.5** | **DeepSeek V3** |
| --- | --- | --- |
| **去标识化临床病例摘要 De-identified Clinical Case Summary** | 患者26岁，孕产史为G1P1，既往剖宫产1次，孕周39+4，临产类别为自然临产。自然破膜，羊水量约320ml，羊水性质为清。以剖宫产分娩，产时出血约400ml。第一产程6时30分，第二产程未进入第二产程，第三产程0时5分，总产程6时35分，会阴或切口情况为完整。合并症及主要风险因素：活跃期停滞？头盆不称？。新生儿出生体重3570g，Apgar评分1、5、10分钟分别为10、10、10分，分娩结局为活产。 | |
| **临床病例摘要英文翻译 English Translation of De-identified Clinical Case Summary** | Maternal age was 26 years. The obstetric history was G1P1, with 1 previous cesarean deliveries. Gestational age was 39+4 weeks, and the labor onset or induction category was spontaneous labor. The membranes ruptured spontaneously; the amniotic fluid volume was approximately 320 mL and its character was clear. The patient underwent cesarean delivery, with estimated intrapartum blood loss of 400 mL. The first, second, and third stages of labor were 6 h 30 min, the second stage was not reached, and 0 h 5 min, respectively; the total labor duration was 6 h 35 min. The perineum was intact. Documented comorbidities and major risk factors were active-phase arrest suspected; suspected cephalopelvic disproportion. Neonatal birth weight was 3570 g; Apgar scores at 1, 5, and 10 minutes were 10, 10, 10; and the delivery outcome was live birth. | |
| **模型生成的产褥期护理计划 Model-generated postpartum nursing care plan** | 产妇26岁，G1P1，孕39+4周，经剖宫产分娩，产时出血约400ml，新生儿女婴、体重3570g，Apgar评分10、10、10分。病例提示的重点风险为未记录特殊合并症。产后24小时内重点评估体温、脉搏、血压、宫底高度、宫缩、阴道流血、恶露颜色和尿潴留，记录首次排尿和疼痛评分。产后住院及出院后继续观察恶露是否由红色逐渐转淡，乳房是否胀痛，乳头是否皲裂，睡眠和情绪是否稳定。饮食以优质蛋白、含铁食物、蔬菜水果和足量饮水为主，避免盲目大补和长期卧床。本次为剖宫产，腹部切口和血栓预防是护理重点。切口应保持清洁干燥，观察红肿、渗血、渗液、裂开、异味和疼痛加重。翻身、咳嗽和下床时保护切口，在生命体征平稳后尽早床旁活动，关注肠鸣音恢复、排气排便、尿潴留和下肢肿痛。哺乳方面建议尽早母婴皮肤接触和按需哺乳，指导正确含接，乳胀时先热敷或轻柔按摩再哺乳，乳头疼痛时及时调整姿势。若出现乳房局部红肿硬痛、寒战或高热，应警惕乳腺炎。产时出血量需要持续观察，建议每日记录恶露量、颜色和血块情况，活动后若明显增多，应减少活动并请医护评估。出院前应完成用药、伤口、哺乳和避孕宣教，42天左右复查，必要时提前复诊。若出现阴道流血突然增多、鲜红血浸湿卫生巾、恶露恶臭、发热、持续腹痛、伤口化脓、排尿困难、下肢单侧肿痛、胸闷气短，或持续悲伤焦虑及伤害自己或婴儿的想法，应立即就医或急诊处理。 | 患者26岁，G1P1，急诊剖宫产，合并活跃期停滞、头盆不称，产时出血400ml，新生儿Apgar 10。剖宫产术后常规护理。出血400ml注意晚期出血。42天复查。 |
| **护理计划英文翻译 English translation of care plan** | The patient is 26 years old, G1P1, delivered at 39+4 weeks by cesarean delivery, with an estimated intrapartum blood loss of 400 mL. The newborn was a female infant, weighed 3570 g, and had Apgar scores of 10, 10, 10. The main case-based risks include no special comorbidity was recorded. Within the first 24 hours, assess temperature, pulse, blood pressure, uterine fundal height, uterine tone, vaginal bleeding, lochia color, urinary retention, first voiding, and pain score. During hospitalization and after discharge, continue to observe whether lochia gradually changes from red to lighter color, whether breast engorgement or nipple fissure occurs, and whether sleep and mood remain stable. Diet should include high-quality protein, iron-rich food, vegetables, fruit, and adequate fluids. Avoid blind over-supplementation and prolonged bed rest. Because this was a cesarean delivery, abdominal incision care and thrombosis prevention are key. Keep the incision clean and dry and observe for redness, swelling, bleeding, drainage, separation, odor, or worsening pain. Support the incision when turning, coughing, or getting out of bed. Once vital signs are stable, begin assisted ambulation and monitor bowel sounds, passage of gas, bowel movement, urinary retention, and leg swelling or pain. For breastfeeding, encourage early skin-to-skin contact and feeding on demand. Teach correct latch and positioning. For engorgement, apply warmth or gentle massage before feeding. If nipple pain occurs, adjust latch promptly. Localized breast redness, hard painful swelling, chills, or high fever should raise concern for mastitis. Because blood loss requires closer observation, record the amount, color, and clots in the lochia every day. If bleeding increases clearly after activity, reduce activity and seek clinical assessment. Before discharge, provide education on medication, wound care, breastfeeding, and contraception. Arrange a postpartum review around 42 days after delivery, or earlier if symptoms occur. Seek urgent medical care for sudden heavy vaginal bleeding, bright red bleeding soaking pads, foul-smelling lochia, fever, persistent abdominal pain, pus from the wound, difficulty urinating, unilateral leg swelling or pain, chest tightness, shortness of breath, or persistent sadness, anxiety, or thoughts of harming herself or the baby. | The patient is a 26-year-old G1P1 who underwent emergency cesarean section for active-phase arrest and cephalopelvic disproportion. Blood loss 400 mL, newborn Apgar 10. Routine post-cesarean care. Watch for late PPH. Follow-up at 42 days. |
| **Clinical appropriateness 临床适当性** | 4 | 3 |
| **Individualization 个体化程度** | 3 | 3 |
| **Comprehensiveness 完整性** | 4 | 3 |
| **Actionability 可操作性** | 4 | 3 |
| **Safety 安全性** | 5 | 3 |
| **Triage appropriateness 分诊/转诊建议适当性** | 5 | 3 |
| **Structure and clarity 结构与清晰度** | 3 | 4 |

**病例 112 / Case 112**

| **项目 Item** | **ChatGPT 5.5** | **DeepSeek V3** |
| --- | --- | --- |
| **去标识化临床病例摘要 De-identified Clinical Case Summary** | 患者33岁，孕产史为G2P1，既往剖宫产1次，孕周39+6，临产类别为自然临产。自然破膜，羊水量约200ml，羊水性质为Ⅱ°。以剖宫产分娩，产时出血约430ml。第一产程5时45分，第二产程未进入第二产程，第三产程0时5分，总产程5时50分，会阴或切口情况为完整。合并症及主要风险因素：持续性枕后位；胎儿宫内窘迫？。新生儿出生体重3320g，Apgar评分1、5、10分钟分别为9、10、10分，分娩结局为活产。 | |
| **临床病例摘要英文翻译 English Translation of De-identified Clinical Case Summary** | Maternal age was 33 years. The obstetric history was G2P1, with 1 previous cesarean deliveries. Gestational age was 39+6 weeks, and the labor onset or induction category was spontaneous labor. The membranes ruptured spontaneously; the amniotic fluid volume was approximately 200 mL and its character was grade II meconium-stained. The patient underwent cesarean delivery, with estimated intrapartum blood loss of 430 mL. The first, second, and third stages of labor were 5 h 45 min, the second stage was not reached, and 0 h 5 min, respectively; the total labor duration was 5 h 50 min. The perineum was intact. Documented comorbidities and major risk factors were persistent occiput posterior position; suspected fetal distress. Neonatal birth weight was 3320 g; Apgar scores at 1, 5, and 10 minutes were 9, 10, 10; and the delivery outcome was live birth. | |
| **模型生成的产褥期护理计划 Model-generated postpartum nursing care plan** | 产妇33岁，G2P1，孕39+6周，经剖宫产分娩，产时出血约430ml，新生儿男婴、体重3320g，Apgar评分9、10、10分。病例提示的重点风险为胎儿窘迫或羊水异常记录。产后24小时内重点评估体温、脉搏、血压、宫底高度、宫缩、阴道流血、恶露颜色和尿潴留，记录首次排尿和疼痛评分。产后住院及出院后继续观察恶露是否由红色逐渐转淡，乳房是否胀痛，乳头是否皲裂，睡眠和情绪是否稳定。饮食以优质蛋白、含铁食物、蔬菜水果和足量饮水为主，避免盲目大补和长期卧床。本次为剖宫产，腹部切口和血栓预防是护理重点。切口应保持清洁干燥，观察红肿、渗血、渗液、裂开、异味和疼痛加重。翻身、咳嗽和下床时保护切口，在生命体征平稳后尽早床旁活动，关注肠鸣音恢复、排气排便、尿潴留和下肢肿痛。哺乳方面建议尽早母婴皮肤接触和按需哺乳，指导正确含接，乳胀时先热敷或轻柔按摩再哺乳，乳头疼痛时及时调整姿势。若出现乳房局部红肿硬痛、寒战或高热，应警惕乳腺炎。产时出血量需要持续观察，建议每日记录恶露量、颜色和血块情况，活动后若明显增多，应减少活动并请医护评估。因有胎儿窘迫、羊水污染或新生儿评分变化记录，母婴同室期间应配合观察新生儿吃奶、肤色、体温、呼吸和反应，发现吃奶差、发绀、嗜睡或呼吸异常应立即告知儿科医生。出院前应完成用药、伤口、哺乳和避孕宣教，42天左右复查，必要时提前复诊。若出现阴道流血突然增多、鲜红血浸湿卫生巾、恶露恶臭、发热、持续腹痛、伤口化脓、排尿困难、下肢单侧肿痛、胸闷气短，或持续悲伤焦虑及伤害自己或婴儿的想法，应立即就医或急诊处理。 | 患者33岁，G2P1，急诊剖宫产，合并持续性枕后位、可疑胎儿窘迫，脐带绕颈1周，羊水Ⅱ°污染，产时出血430ml，新生儿Apgar 9-10-10。持续性枕后位+窘迫+脐带绕颈+Ⅱ°羊水，新生儿需监测MAS及缺氧。剖宫产术后常规护理。出血430ml注意晚期出血。42天复查。 |
| **护理计划英文翻译 English translation of care plan** | The patient is 33 years old, G2P1, delivered at 39+6 weeks by cesarean delivery, with an estimated intrapartum blood loss of 430 mL. The newborn was a male infant, weighed 3320 g, and had Apgar scores of 9, 10, 10. The main case-based risks include fetal distress or abnormal amniotic fluid findings. Within the first 24 hours, assess temperature, pulse, blood pressure, uterine fundal height, uterine tone, vaginal bleeding, lochia color, urinary retention, first voiding, and pain score. During hospitalization and after discharge, continue to observe whether lochia gradually changes from red to lighter color, whether breast engorgement or nipple fissure occurs, and whether sleep and mood remain stable. Diet should include high-quality protein, iron-rich food, vegetables, fruit, and adequate fluids. Avoid blind over-supplementation and prolonged bed rest. Because this was a cesarean delivery, abdominal incision care and thrombosis prevention are key. Keep the incision clean and dry and observe for redness, swelling, bleeding, drainage, separation, odor, or worsening pain. Support the incision when turning, coughing, or getting out of bed. Once vital signs are stable, begin assisted ambulation and monitor bowel sounds, passage of gas, bowel movement, urinary retention, and leg swelling or pain. For breastfeeding, encourage early skin-to-skin contact and feeding on demand. Teach correct latch and positioning. For engorgement, apply warmth or gentle massage before feeding. If nipple pain occurs, adjust latch promptly. Localized breast redness, hard painful swelling, chills, or high fever should raise concern for mastitis. Because blood loss requires closer observation, record the amount, color, and clots in the lochia every day. If bleeding increases clearly after activity, reduce activity and seek clinical assessment. Because fetal distress, meconium-stained fluid, or changed Apgar scores were recorded, cooperate with pediatric observation of feeding, skin color, temperature, breathing, and responsiveness. Poor feeding, cyanosis, excessive sleepiness, or abnormal breathing should be reported immediately. Before discharge, provide education on medication, wound care, breastfeeding, and contraception. Arrange a postpartum review around 42 days after delivery, or earlier if symptoms occur. Seek urgent medical care for sudden heavy vaginal bleeding, bright red bleeding soaking pads, foul-smelling lochia, fever, persistent abdominal pain, pus from the wound, difficulty urinating, unilateral leg swelling or pain, chest tightness, shortness of breath, or persistent sadness, anxiety, or thoughts of harming herself or the baby. | The patient is a 33-year-old G2P1 who underwent emergency cesarean section for persistent occiput posterior, suspected fetal distress, nuchal cord 1 loop, grade II meconium. Blood loss 430 mL, newborn Apgar 9-10-10. Newborn monitor for MAS and hypoxia. Routine post-cesarean care. Watch for late PPH. Follow-up at 42 days. |
| **Clinical appropriateness 临床适当性** | 5 | 4 |
| **Individualization 个体化程度** | 4 | 4 |
| **Comprehensiveness 完整性** | 5 | 3 |
| **Actionability 可操作性** | 4 | 4 |
| **Safety 安全性** | 5 | 4 |
| **Triage appropriateness 分诊/转诊建议适当性** | 5 | 3 |
| **Structure and clarity 结构与清晰度** | 4 | 4 |

**病例 113 / Case 113**

| **项目 Item** | **ChatGPT 5.5** | **DeepSeek V3** |
| --- | --- | --- |
| **去标识化临床病例摘要 De-identified Clinical Case Summary** | 患者30岁，孕产史为G1P1，既往剖宫产1次，孕周37+3，临产类别为未临产。人工破膜，羊水量约180ml，羊水性质为清。以剖宫产分娩，产时出血约360ml。第一产程未进入产程，第二产程未进入第二产程，第三产程0时5分，总产程未进入产程，会阴或切口情况为完整。合并症及主要风险因素：胎儿生长受限？脐血流异常？。新生儿出生体重2250g，Apgar评分1、5、10分钟分别为9、10、10分，分娩结局为活产。 | |
| **临床病例摘要英文翻译 English Translation of De-identified Clinical Case Summary** | Maternal age was 30 years. The obstetric history was G1P1, with 1 previous cesarean deliveries. Gestational age was 37+3 weeks, and the labor onset or induction category was no labor. The membranes were artificially ruptured; the amniotic fluid volume was approximately 180 mL and its character was clear. The patient underwent cesarean delivery, with estimated intrapartum blood loss of 360 mL. The first, second, and third stages of labor were labor was not established, the second stage was not reached, and 0 h 5 min, respectively; the total labor duration was labor was not established. The perineum was intact. Documented comorbidities and major risk factors were suspected fetal growth restriction; abnormal umbilical blood flow suspected. Neonatal birth weight was 2250 g; Apgar scores at 1, 5, and 10 minutes were 9, 10, 10; and the delivery outcome was live birth. | |
| **模型生成的产褥期护理计划 Model-generated postpartum nursing care plan** | 产妇30岁，G1P1，孕37+3周，经剖宫产分娩，产时出血约360ml，新生儿女婴、体重2250g，Apgar评分9、10、10分。病例提示的重点风险为胎儿窘迫或羊水异常记录。产后24小时内重点评估体温、脉搏、血压、宫底高度、宫缩、阴道流血、恶露颜色和尿潴留，记录首次排尿和疼痛评分。产后住院及出院后继续观察恶露是否由红色逐渐转淡，乳房是否胀痛，乳头是否皲裂，睡眠和情绪是否稳定。饮食以优质蛋白、含铁食物、蔬菜水果和足量饮水为主，避免盲目大补和长期卧床。本次为剖宫产，腹部切口和血栓预防是护理重点。切口应保持清洁干燥，观察红肿、渗血、渗液、裂开、异味和疼痛加重。翻身、咳嗽和下床时保护切口，在生命体征平稳后尽早床旁活动，关注肠鸣音恢复、排气排便、尿潴留和下肢肿痛。哺乳方面建议尽早母婴皮肤接触和按需哺乳，指导正确含接，乳胀时先热敷或轻柔按摩再哺乳，乳头疼痛时及时调整姿势。若出现乳房局部红肿硬痛、寒战或高热，应警惕乳腺炎。因有胎儿窘迫、羊水污染或新生儿评分变化记录，母婴同室期间应配合观察新生儿吃奶、肤色、体温、呼吸和反应，发现吃奶差、发绀、嗜睡或呼吸异常应立即告知儿科医生。出院前应完成用药、伤口、哺乳和避孕宣教，42天左右复查，必要时提前复诊。若出现阴道流血突然增多、鲜红血浸湿卫生巾、恶露恶臭、发热、持续腹痛、伤口化脓、排尿困难、下肢单侧肿痛、胸闷气短，或持续悲伤焦虑及伤害自己或婴儿的想法，应立即就医或急诊处理。 | 患者30岁，G1P1，剖宫产（择期），合并胎儿生长受限可疑、脐血流异常可疑，新生儿体重2250g（低出生体重），产时出血360ml，Apgar 9-10-10。FGR新生儿需监测血糖、体温、喂养，建议新生儿科观察。剖宫产术后常规护理。提供心理支持。新生儿生长随访；42天复查。 |
| **护理计划英文翻译 English translation of care plan** | The patient is 30 years old, G1P1, delivered at 37+3 weeks by cesarean delivery, with an estimated intrapartum blood loss of 360 mL. The newborn was a female infant, weighed 2250 g, and had Apgar scores of 9, 10, 10. The main case-based risks include fetal distress or abnormal amniotic fluid findings. Within the first 24 hours, assess temperature, pulse, blood pressure, uterine fundal height, uterine tone, vaginal bleeding, lochia color, urinary retention, first voiding, and pain score. During hospitalization and after discharge, continue to observe whether lochia gradually changes from red to lighter color, whether breast engorgement or nipple fissure occurs, and whether sleep and mood remain stable. Diet should include high-quality protein, iron-rich food, vegetables, fruit, and adequate fluids. Avoid blind over-supplementation and prolonged bed rest. Because this was a cesarean delivery, abdominal incision care and thrombosis prevention are key. Keep the incision clean and dry and observe for redness, swelling, bleeding, drainage, separation, odor, or worsening pain. Support the incision when turning, coughing, or getting out of bed. Once vital signs are stable, begin assisted ambulation and monitor bowel sounds, passage of gas, bowel movement, urinary retention, and leg swelling or pain. For breastfeeding, encourage early skin-to-skin contact and feeding on demand. Teach correct latch and positioning. For engorgement, apply warmth or gentle massage before feeding. If nipple pain occurs, adjust latch promptly. Localized breast redness, hard painful swelling, chills, or high fever should raise concern for mastitis. Because fetal distress, meconium-stained fluid, or changed Apgar scores were recorded, cooperate with pediatric observation of feeding, skin color, temperature, breathing, and responsiveness. Poor feeding, cyanosis, excessive sleepiness, or abnormal breathing should be reported immediately. Before discharge, provide education on medication, wound care, breastfeeding, and contraception. Arrange a postpartum review around 42 days after delivery, or earlier if symptoms occur. Seek urgent medical care for sudden heavy vaginal bleeding, bright red bleeding soaking pads, foul-smelling lochia, fever, persistent abdominal pain, pus from the wound, difficulty urinating, unilateral leg swelling or pain, chest tightness, shortness of breath, or persistent sadness, anxiety, or thoughts of harming herself or the baby. | The patient is a 30-year-old G1P1 who underwent cesarean section for suspected fetal growth restriction and abnormal umbilical blood flow. Newborn LBW 2250 g, Apgar 9-10-10, blood loss 360 mL. FGR newborn – monitor glucose, temperature, feeding; pediatric observation. Routine post-cesarean care. Psychological support. Newborn growth follow-up; maternal check at 42 days. |
| **Clinical appropriateness 临床适当性** | 4 | 4 |
| **Individualization 个体化程度** | 4 | 4 |
| **Comprehensiveness 完整性** | 4 | 3 |
| **Actionability 可操作性** | 4 | 3 |
| **Safety 安全性** | 5 | 4 |
| **Triage appropriateness 分诊/转诊建议适当性** | 5 | 3 |
| **Structure and clarity 结构与清晰度** | 4 | 4 |

**病例 114 / Case 114**

| **项目 Item** | **ChatGPT 5.5** | **DeepSeek V3** |
| --- | --- | --- |
| **去标识化临床病例摘要 De-identified Clinical Case Summary** | 患者36岁，孕产史为G2P1，既往剖宫产1次，孕周40，临产类别为未临产。人工破膜，羊水量约700ml，羊水性质为清。以剖宫产分娩，产时出血约480ml。第一产程未进入产程，第二产程未进入第二产程，第三产程0时6分，总产程未进入产程，会阴或切口情况为完整。合并症及主要风险因素：妊娠期糖尿病；羊水过多；巨大儿。新生儿出生体重4280g，Apgar评分1、5、10分钟分别为10、10、10分，分娩结局为活产。 | |
| **临床病例摘要英文翻译 English Translation of De-identified Clinical Case Summary** | Maternal age was 36 years. The obstetric history was G2P1, with 1 previous cesarean deliveries. Gestational age was 40 weeks, and the labor onset or induction category was no labor. The membranes were artificially ruptured; the amniotic fluid volume was approximately 700 mL and its character was clear. The patient underwent cesarean delivery, with estimated intrapartum blood loss of 480 mL. The first, second, and third stages of labor were labor was not established, the second stage was not reached, and 0 h 6 min, respectively; the total labor duration was labor was not established. The perineum was intact. Documented comorbidities and major risk factors were gestational diabetes mellitus; polyhydramnios; macrosomia. Neonatal birth weight was 4280 g; Apgar scores at 1, 5, and 10 minutes were 10, 10, 10; and the delivery outcome was live birth. | |
| **模型生成的产褥期护理计划 Model-generated postpartum nursing care plan** | 产妇36岁，G2P1，孕40周，经剖宫产分娩，产时出血约480ml，新生儿男婴、体重4280g，Apgar评分10、10、10分。病例提示的重点风险为妊娠期糖尿病、高龄妊娠因素。产后24小时内重点评估体温、脉搏、血压、宫底高度、宫缩、阴道流血、恶露颜色和尿潴留，记录首次排尿和疼痛评分。产后住院及出院后继续观察恶露是否由红色逐渐转淡，乳房是否胀痛，乳头是否皲裂，睡眠和情绪是否稳定。饮食以优质蛋白、含铁食物、蔬菜水果和足量饮水为主，避免盲目大补和长期卧床。本次为剖宫产，腹部切口和血栓预防是护理重点。切口应保持清洁干燥，观察红肿、渗血、渗液、裂开、异味和疼痛加重。翻身、咳嗽和下床时保护切口，在生命体征平稳后尽早床旁活动，关注肠鸣音恢复、排气排便、尿潴留和下肢肿痛。哺乳方面建议尽早母婴皮肤接触和按需哺乳，指导正确含接，乳胀时先热敷或轻柔按摩再哺乳，乳头疼痛时及时调整姿势。若出现乳房局部红肿硬痛、寒战或高热，应警惕乳腺炎。产时出血量需要持续观察，建议每日记录恶露量、颜色和血块情况，活动后若明显增多，应减少活动并请医护评估。有妊娠期糖尿病者，产后饮食应控制精制糖和高油食物，少量多餐，结合恢复情况逐步活动，并按医嘱在产后复查血糖或糖耐量。高龄产妇恢复期应避免过度劳累和连续熬夜，家属应参与夜间照护，保证睡眠、营养和情绪支持。出院前应完成用药、伤口、哺乳和避孕宣教，42天左右复查，必要时提前复诊。若出现阴道流血突然增多、鲜红血浸湿卫生巾、恶露恶臭、发热、持续腹痛、伤口化脓、排尿困难、下肢单侧肿痛、胸闷气短，或持续悲伤焦虑及伤害自己或婴儿的想法，应立即就医或急诊处理。 | 患者36岁，G2P1，剖宫产（择期），合并GDM、羊水过多、巨大儿，新生儿体重4280g，产时出血480ml。GDM产后监测血糖。巨大儿剖宫产需注意新生儿低血糖。羊水过多产后注意子宫收缩乏力，加强宫底按摩。常规剖宫产护理。42天复查血糖。 |
| **护理计划英文翻译 English translation of care plan** | The patient is 36 years old, G2P1, delivered at 40 weeks by cesarean delivery, with an estimated intrapartum blood loss of 480 mL. The newborn was a male infant, weighed 4280 g, and had Apgar scores of 10, 10, 10. The main case-based risks include gestational diabetes mellitus, advanced maternal age. Within the first 24 hours, assess temperature, pulse, blood pressure, uterine fundal height, uterine tone, vaginal bleeding, lochia color, urinary retention, first voiding, and pain score. During hospitalization and after discharge, continue to observe whether lochia gradually changes from red to lighter color, whether breast engorgement or nipple fissure occurs, and whether sleep and mood remain stable. Diet should include high-quality protein, iron-rich food, vegetables, fruit, and adequate fluids. Avoid blind over-supplementation and prolonged bed rest. Because this was a cesarean delivery, abdominal incision care and thrombosis prevention are key. Keep the incision clean and dry and observe for redness, swelling, bleeding, drainage, separation, odor, or worsening pain. Support the incision when turning, coughing, or getting out of bed. Once vital signs are stable, begin assisted ambulation and monitor bowel sounds, passage of gas, bowel movement, urinary retention, and leg swelling or pain. For breastfeeding, encourage early skin-to-skin contact and feeding on demand. Teach correct latch and positioning. For engorgement, apply warmth or gentle massage before feeding. If nipple pain occurs, adjust latch promptly. Localized breast redness, hard painful swelling, chills, or high fever should raise concern for mastitis. Because blood loss requires closer observation, record the amount, color, and clots in the lochia every day. If bleeding increases clearly after activity, reduce activity and seek clinical assessment. For gestational diabetes, avoid refined sugar and heavy oily foods, eat small balanced meals, increase activity gradually, and arrange postpartum glucose or oral glucose tolerance testing as advised. Advanced maternal age may slow recovery, so the family should help with nighttime care and support adequate sleep, nutrition, and emotional stability. Before discharge, provide education on medication, wound care, breastfeeding, and contraception. Arrange a postpartum review around 42 days after delivery, or earlier if symptoms occur. Seek urgent medical care for sudden heavy vaginal bleeding, bright red bleeding soaking pads, foul-smelling lochia, fever, persistent abdominal pain, pus from the wound, difficulty urinating, unilateral leg swelling or pain, chest tightness, shortness of breath, or persistent sadness, anxiety, or thoughts of harming herself or the baby. | The patient is a 36-year-old G2P1 who underwent cesarean section for GDM, polyhydramnios, and macrosomia. Newborn weight 4280 g, blood loss 480 mL. Monitor postpartum glucose. Newborn – monitor for hypoglycemia. Polyhydramnios may cause uterine atony – aggressive fundal massage. Routine post-cesarean care. Follow-up glucose at 42 days. |
| **Clinical appropriateness 临床适当性** | 5 | 3 |
| **Individualization 个体化程度** | 4 | 3 |
| **Comprehensiveness 完整性** | 5 | 3 |
| **Actionability 可操作性** | 4 | 3 |
| **Safety 安全性** | 5 | 3 |
| **Triage appropriateness 分诊/转诊建议适当性** | 5 | 3 |
| **Structure and clarity 结构与清晰度** | 3 | 4 |

**病例 115 / Case 115**

| **项目 Item** | **ChatGPT 5.5** | **DeepSeek V3** |
| --- | --- | --- |
| **去标识化临床病例摘要 De-identified Clinical Case Summary** | 患者41岁，孕产史为G3P2，既往剖宫产1次，孕周38+1，临产类别为未临产。人工破膜，羊水量约240ml，羊水性质为清。以剖宫产分娩，产时出血约420ml。第一产程未进入产程，第二产程未进入第二产程，第三产程0时5分，总产程未进入产程，会阴或切口情况为完整。合并症及主要风险因素：妊娠合并卵巢囊肿；高龄经产妇妊娠监督。新生儿出生体重3160g，Apgar评分1、5、10分钟分别为10、10、10分，分娩结局为活产。 | |
| **临床病例摘要英文翻译 English Translation of De-identified Clinical Case Summary** | Maternal age was 41 years. The obstetric history was G3P2, with 1 previous cesarean deliveries. Gestational age was 38+1 weeks, and the labor onset or induction category was no labor. The membranes were artificially ruptured; the amniotic fluid volume was approximately 240 mL and its character was clear. The patient underwent cesarean delivery, with estimated intrapartum blood loss of 420 mL. The first, second, and third stages of labor were labor was not established, the second stage was not reached, and 0 h 5 min, respectively; the total labor duration was labor was not established. The perineum was intact. Documented comorbidities and major risk factors were ovarian cyst during pregnancy; advanced maternal age in a multipara. Neonatal birth weight was 3160 g; Apgar scores at 1, 5, and 10 minutes were 10, 10, 10; and the delivery outcome was live birth. | |
| **模型生成的产褥期护理计划 Model-generated postpartum nursing care plan** | 产妇41岁，G3P2，孕38+1周，经剖宫产分娩，产时出血约420ml，新生儿女婴、体重3160g，Apgar评分10、10、10分。病例提示的重点风险为高龄妊娠因素。产后24小时内重点评估体温、脉搏、血压、宫底高度、宫缩、阴道流血、恶露颜色和尿潴留，记录首次排尿和疼痛评分。产后住院及出院后继续观察恶露是否由红色逐渐转淡，乳房是否胀痛，乳头是否皲裂，睡眠和情绪是否稳定。饮食以优质蛋白、含铁食物、蔬菜水果和足量饮水为主，避免盲目大补和长期卧床。本次为剖宫产，腹部切口和血栓预防是护理重点。切口应保持清洁干燥，观察红肿、渗血、渗液、裂开、异味和疼痛加重。翻身、咳嗽和下床时保护切口，在生命体征平稳后尽早床旁活动，关注肠鸣音恢复、排气排便、尿潴留和下肢肿痛。哺乳方面建议尽早母婴皮肤接触和按需哺乳，指导正确含接，乳胀时先热敷或轻柔按摩再哺乳，乳头疼痛时及时调整姿势。若出现乳房局部红肿硬痛、寒战或高热，应警惕乳腺炎。产时出血量需要持续观察，建议每日记录恶露量、颜色和血块情况，活动后若明显增多，应减少活动并请医护评估。合并子宫肌瘤、卵巢囊肿或多囊卵巢相关情况时，产后复查时建议结合妇科超声评估，若腹痛、包块感或异常出血加重，应提前就诊。高龄产妇恢复期应避免过度劳累和连续熬夜，家属应参与夜间照护，保证睡眠、营养和情绪支持。出院前应完成用药、伤口、哺乳和避孕宣教，42天左右复查，必要时提前复诊。若出现阴道流血突然增多、鲜红血浸湿卫生巾、恶露恶臭、发热、持续腹痛、伤口化脓、排尿困难、下肢单侧肿痛、胸闷气短，或持续悲伤焦虑及伤害自己或婴儿的想法，应立即就医或急诊处理。 | 患者41岁，G3P2，剖宫产（择期），合并卵巢囊肿、高龄经产妇，产时出血420ml。卵巢囊肿产后6周B超复查。高龄经产妇注意血栓预防。常规剖宫产护理。42天复查B超。 |
| **护理计划英文翻译 English translation of care plan** | The patient is 41 years old, G3P2, delivered at 38+1 weeks by cesarean delivery, with an estimated intrapartum blood loss of 420 mL. The newborn was a female infant, weighed 3160 g, and had Apgar scores of 10, 10, 10. The main case-based risks include advanced maternal age. Within the first 24 hours, assess temperature, pulse, blood pressure, uterine fundal height, uterine tone, vaginal bleeding, lochia color, urinary retention, first voiding, and pain score. During hospitalization and after discharge, continue to observe whether lochia gradually changes from red to lighter color, whether breast engorgement or nipple fissure occurs, and whether sleep and mood remain stable. Diet should include high-quality protein, iron-rich food, vegetables, fruit, and adequate fluids. Avoid blind over-supplementation and prolonged bed rest. Because this was a cesarean delivery, abdominal incision care and thrombosis prevention are key. Keep the incision clean and dry and observe for redness, swelling, bleeding, drainage, separation, odor, or worsening pain. Support the incision when turning, coughing, or getting out of bed. Once vital signs are stable, begin assisted ambulation and monitor bowel sounds, passage of gas, bowel movement, urinary retention, and leg swelling or pain. For breastfeeding, encourage early skin-to-skin contact and feeding on demand. Teach correct latch and positioning. For engorgement, apply warmth or gentle massage before feeding. If nipple pain occurs, adjust latch promptly. Localized breast redness, hard painful swelling, chills, or high fever should raise concern for mastitis. Because blood loss requires closer observation, record the amount, color, and clots in the lochia every day. If bleeding increases clearly after activity, reduce activity and seek clinical assessment. For uterine fibroids, ovarian cysts, or polycystic ovary related findings, pelvic ultrasound can be considered at postpartum review. Earlier assessment is needed for worsening abdominal pain, mass sensation, or abnormal bleeding. Advanced maternal age may slow recovery, so the family should help with nighttime care and support adequate sleep, nutrition, and emotional stability. Before discharge, provide education on medication, wound care, breastfeeding, and contraception. Arrange a postpartum review around 42 days after delivery, or earlier if symptoms occur. Seek urgent medical care for sudden heavy vaginal bleeding, bright red bleeding soaking pads, foul-smelling lochia, fever, persistent abdominal pain, pus from the wound, difficulty urinating, unilateral leg swelling or pain, chest tightness, shortness of breath, or persistent sadness, anxiety, or thoughts of harming herself or the baby. | The patient is a 41-year-old G3P2 who underwent cesarean section for ovarian cyst and advanced age multipara. Blood loss 420 mL. Ovarian cyst – repeat ultrasound at 6 weeks. Thrombosis prevention. Routine post-cesarean care. Follow-up ultrasound at 42 days. |
| **Clinical appropriateness 临床适当性** | 5 | 3 |
| **Individualization 个体化程度** | 4 | 3 |
| **Comprehensiveness 完整性** | 5 | 3 |
| **Actionability 可操作性** | 4 | 3 |
| **Safety 安全性** | 5 | 3 |
| **Triage appropriateness 分诊/转诊建议适当性** | 5 | 3 |
| **Structure and clarity 结构与清晰度** | 3 | 4 |

**病例 116 / Case 116**

| **项目 Item** | **ChatGPT 5.5** | **DeepSeek V3** |
| --- | --- | --- |
| **去标识化临床病例摘要 De-identified Clinical Case Summary** | 患者24岁，孕产史为G1P1，既往剖宫产1次，孕周36+4，临产类别为自然临产。自然破膜，羊水量约160ml，羊水性质为Ⅱ°。以剖宫产分娩，产时出血约390ml。第一产程3时05分，第二产程未进入第二产程，第三产程0时4分，总产程3时09分，会阴或切口情况为完整。合并症及主要风险因素：早产临产；胎儿窘迫？。新生儿出生体重2480g，Apgar评分1、5、10分钟分别为8、9、10分，分娩结局为活产。 | |
| **临床病例摘要英文翻译 English Translation of De-identified Clinical Case Summary** | Maternal age was 24 years. The obstetric history was G1P1, with 1 previous cesarean deliveries. Gestational age was 36+4 weeks, and the labor onset or induction category was spontaneous labor. The membranes ruptured spontaneously; the amniotic fluid volume was approximately 160 mL and its character was grade II meconium-stained. The patient underwent cesarean delivery, with estimated intrapartum blood loss of 390 mL. The first, second, and third stages of labor were 3 h 5 min, the second stage was not reached, and 0 h 4 min, respectively; the total labor duration was 3 h 9 min. The perineum was intact. Documented comorbidities and major risk factors were preterm labor; suspected fetal distress. Neonatal birth weight was 2480 g; Apgar scores at 1, 5, and 10 minutes were 8, 9, 10; and the delivery outcome was live birth. | |
| **模型生成的产褥期护理计划 Model-generated postpartum nursing care plan** | 产妇24岁，G1P1，孕36+4周，经剖宫产分娩，产时出血约390ml，新生儿男婴、体重2480g，Apgar评分8、9、10分。病例提示的重点风险为胎儿窘迫或羊水异常记录。产后24小时内重点评估体温、脉搏、血压、宫底高度、宫缩、阴道流血、恶露颜色和尿潴留，记录首次排尿和疼痛评分。产后住院及出院后继续观察恶露是否由红色逐渐转淡，乳房是否胀痛，乳头是否皲裂，睡眠和情绪是否稳定。饮食以优质蛋白、含铁食物、蔬菜水果和足量饮水为主，避免盲目大补和长期卧床。本次为剖宫产，腹部切口和血栓预防是护理重点。切口应保持清洁干燥，观察红肿、渗血、渗液、裂开、异味和疼痛加重。翻身、咳嗽和下床时保护切口，在生命体征平稳后尽早床旁活动，关注肠鸣音恢复、排气排便、尿潴留和下肢肿痛。哺乳方面建议尽早母婴皮肤接触和按需哺乳，指导正确含接，乳胀时先热敷或轻柔按摩再哺乳，乳头疼痛时及时调整姿势。若出现乳房局部红肿硬痛、寒战或高热，应警惕乳腺炎。因有胎儿窘迫、羊水污染或新生儿评分变化记录，母婴同室期间应配合观察新生儿吃奶、肤色、体温、呼吸和反应，发现吃奶差、发绀、嗜睡或呼吸异常应立即告知儿科医生。出院前应完成用药、伤口、哺乳和避孕宣教，42天左右复查，必要时提前复诊。若出现阴道流血突然增多、鲜红血浸湿卫生巾、恶露恶臭、发热、持续腹痛、伤口化脓、排尿困难、下肢单侧肿痛、胸闷气短，或持续悲伤焦虑及伤害自己或婴儿的想法，应立即就医或急诊处理。 | 患者24岁，G1P1，急诊剖宫产，合并早产临产、可疑胎儿窘迫，羊水Ⅱ°污染，新生儿体重2480g（低出生体重），产时出血390ml，Apgar 8-9-10。早产儿需保暖、监测血糖、黄疸、呼吸暂停，鼓励母乳+强化剂。Ⅱ°羊水新生儿监测MAS。出血390ml注意晚期出血。提供心理支持。早产儿随访；42天复查。 |
| **护理计划英文翻译 English translation of care plan** | The patient is 24 years old, G1P1, delivered at 36+4 weeks by cesarean delivery, with an estimated intrapartum blood loss of 390 mL. The newborn was a male infant, weighed 2480 g, and had Apgar scores of 8, 9, 10. The main case-based risks include fetal distress or abnormal amniotic fluid findings. Within the first 24 hours, assess temperature, pulse, blood pressure, uterine fundal height, uterine tone, vaginal bleeding, lochia color, urinary retention, first voiding, and pain score. During hospitalization and after discharge, continue to observe whether lochia gradually changes from red to lighter color, whether breast engorgement or nipple fissure occurs, and whether sleep and mood remain stable. Diet should include high-quality protein, iron-rich food, vegetables, fruit, and adequate fluids. Avoid blind over-supplementation and prolonged bed rest. Because this was a cesarean delivery, abdominal incision care and thrombosis prevention are key. Keep the incision clean and dry and observe for redness, swelling, bleeding, drainage, separation, odor, or worsening pain. Support the incision when turning, coughing, or getting out of bed. Once vital signs are stable, begin assisted ambulation and monitor bowel sounds, passage of gas, bowel movement, urinary retention, and leg swelling or pain. For breastfeeding, encourage early skin-to-skin contact and feeding on demand. Teach correct latch and positioning. For engorgement, apply warmth or gentle massage before feeding. If nipple pain occurs, adjust latch promptly. Localized breast redness, hard painful swelling, chills, or high fever should raise concern for mastitis. Because fetal distress, meconium-stained fluid, or changed Apgar scores were recorded, cooperate with pediatric observation of feeding, skin color, temperature, breathing, and responsiveness. Poor feeding, cyanosis, excessive sleepiness, or abnormal breathing should be reported immediately. Before discharge, provide education on medication, wound care, breastfeeding, and contraception. Arrange a postpartum review around 42 days after delivery, or earlier if symptoms occur. Seek urgent medical care for sudden heavy vaginal bleeding, bright red bleeding soaking pads, foul-smelling lochia, fever, persistent abdominal pain, pus from the wound, difficulty urinating, unilateral leg swelling or pain, chest tightness, shortness of breath, or persistent sadness, anxiety, or thoughts of harming herself or the baby. | The patient is a 24-year-old G1P1 who underwent emergency cesarean section for preterm labor, suspected fetal distress, grade II meconium. Newborn LBW 2480 g, Apgar 8-9-10, blood loss 390 mL. Preterm infant care: warmth, glucose, jaundice, apnea; breastfeeding with fortifier. Newborn monitor for MAS. Watch for late PPH. Psychological support. Preterm infant follow-up; maternal check at 42 days. |
| **Clinical appropriateness 临床适当性** | 4 | 4 |
| **Individualization 个体化程度** | 4 | 5 |
| **Comprehensiveness 完整性** | 4 | 3 |
| **Actionability 可操作性** | 4 | 3 |
| **Safety 安全性** | 5 | 4 |
| **Triage appropriateness 分诊/转诊建议适当性** | 5 | 3 |
| **Structure and clarity 结构与清晰度** | 4 | 4 |

**病例 117 / Case 117**

| **项目 Item** | **ChatGPT 5.5** | **DeepSeek V3** |
| --- | --- | --- |
| **去标识化临床病例摘要 De-identified Clinical Case Summary** | 患者32岁，孕产史为G2P1，既往剖宫产1次，孕周39+2，临产类别为未临产。人工破膜，羊水量约300ml，羊水性质为清。以剖宫产分娩，产时出血约350ml。第一产程未进入产程，第二产程未进入第二产程，第三产程0时5分，总产程未进入产程，会阴或切口情况为完整。合并症及主要风险因素：骨盆狭窄？头盆不称？。新生儿出生体重3200g，Apgar评分1、5、10分钟分别为10、10、10分，分娩结局为活产。 | |
| **临床病例摘要英文翻译 English Translation of De-identified Clinical Case Summary** | Maternal age was 32 years. The obstetric history was G2P1, with 1 previous cesarean deliveries. Gestational age was 39+2 weeks, and the labor onset or induction category was no labor. The membranes were artificially ruptured; the amniotic fluid volume was approximately 300 mL and its character was clear. The patient underwent cesarean delivery, with estimated intrapartum blood loss of 350 mL. The first, second, and third stages of labor were labor was not established, the second stage was not reached, and 0 h 5 min, respectively; the total labor duration was labor was not established. The perineum was intact. Documented comorbidities and major risk factors were contracted pelvis suspected; suspected cephalopelvic disproportion. Neonatal birth weight was 3200 g; Apgar scores at 1, 5, and 10 minutes were 10, 10, 10; and the delivery outcome was live birth. | |
| **模型生成的产褥期护理计划 Model-generated postpartum nursing care plan** | 产妇32岁，G2P1，孕39+2周，经剖宫产分娩，产时出血约350ml，新生儿女婴、体重3200g，Apgar评分10、10、10分。病例提示的重点风险为未记录特殊合并症。产后24小时内重点评估体温、脉搏、血压、宫底高度、宫缩、阴道流血、恶露颜色和尿潴留，记录首次排尿和疼痛评分。产后住院及出院后继续观察恶露是否由红色逐渐转淡，乳房是否胀痛，乳头是否皲裂，睡眠和情绪是否稳定。饮食以优质蛋白、含铁食物、蔬菜水果和足量饮水为主，避免盲目大补和长期卧床。本次为剖宫产，腹部切口和血栓预防是护理重点。切口应保持清洁干燥，观察红肿、渗血、渗液、裂开、异味和疼痛加重。翻身、咳嗽和下床时保护切口，在生命体征平稳后尽早床旁活动，关注肠鸣音恢复、排气排便、尿潴留和下肢肿痛。哺乳方面建议尽早母婴皮肤接触和按需哺乳，指导正确含接，乳胀时先热敷或轻柔按摩再哺乳，乳头疼痛时及时调整姿势。若出现乳房局部红肿硬痛、寒战或高热，应警惕乳腺炎。目前未记录特殊合并症，仍需按常规产褥期管理执行，重点是子宫复旧、恶露、伤口、哺乳、营养、活动和心理状态。出院前应完成用药、伤口、哺乳和避孕宣教，42天左右复查，必要时提前复诊。若出现阴道流血突然增多、鲜红血浸湿卫生巾、恶露恶臭、发热、持续腹痛、伤口化脓、排尿困难、下肢单侧肿痛、胸闷气短，或持续悲伤焦虑及伤害自己或婴儿的想法，应立即就医或急诊处理。 | 患者32岁，G2P1，剖宫产（择期），合并骨盆狭窄可疑、头盆不称，产时出血350ml。剖宫产术后常规护理。无特殊。42天复查。 |
| **护理计划英文翻译 English translation of care plan** | The patient is 32 years old, G2P1, delivered at 39+2 weeks by cesarean delivery, with an estimated intrapartum blood loss of 350 mL. The newborn was a female infant, weighed 3200 g, and had Apgar scores of 10, 10, 10. The main case-based risks include no special comorbidity was recorded. Within the first 24 hours, assess temperature, pulse, blood pressure, uterine fundal height, uterine tone, vaginal bleeding, lochia color, urinary retention, first voiding, and pain score. During hospitalization and after discharge, continue to observe whether lochia gradually changes from red to lighter color, whether breast engorgement or nipple fissure occurs, and whether sleep and mood remain stable. Diet should include high-quality protein, iron-rich food, vegetables, fruit, and adequate fluids. Avoid blind over-supplementation and prolonged bed rest. Because this was a cesarean delivery, abdominal incision care and thrombosis prevention are key. Keep the incision clean and dry and observe for redness, swelling, bleeding, drainage, separation, odor, or worsening pain. Support the incision when turning, coughing, or getting out of bed. Once vital signs are stable, begin assisted ambulation and monitor bowel sounds, passage of gas, bowel movement, urinary retention, and leg swelling or pain. For breastfeeding, encourage early skin-to-skin contact and feeding on demand. Teach correct latch and positioning. For engorgement, apply warmth or gentle massage before feeding. If nipple pain occurs, adjust latch promptly. Localized breast redness, hard painful swelling, chills, or high fever should raise concern for mastitis. No special complication was recorded, but routine postpartum management should still focus on uterine involution, lochia, wounds, breastfeeding, nutrition, activity, and mental health. Before discharge, provide education on medication, wound care, breastfeeding, and contraception. Arrange a postpartum review around 42 days after delivery, or earlier if symptoms occur. Seek urgent medical care for sudden heavy vaginal bleeding, bright red bleeding soaking pads, foul-smelling lochia, fever, persistent abdominal pain, pus from the wound, difficulty urinating, unilateral leg swelling or pain, chest tightness, shortness of breath, or persistent sadness, anxiety, or thoughts of harming herself or the baby. | The patient is a 32-year-old G2P1 who underwent cesarean section for suspected contracted pelvis and cephalopelvic disproportion. Blood loss 350 mL. Routine post-cesarean care. Follow-up at 42 days. |
| **Clinical appropriateness 临床适当性** | 4 | 3 |
| **Individualization 个体化程度** | 3 | 3 |
| **Comprehensiveness 完整性** | 4 | 3 |
| **Actionability 可操作性** | 4 | 3 |
| **Safety 安全性** | 5 | 3 |
| **Triage appropriateness 分诊/转诊建议适当性** | 5 | 3 |
| **Structure and clarity 结构与清晰度** | 3 | 4 |

**病例 118 / Case 118**

| **项目 Item** | **ChatGPT 5.5** | **DeepSeek V3** |
| --- | --- | --- |
| **去标识化临床病例摘要 De-identified Clinical Case Summary** | 患者35岁，孕产史为G2P2，既往剖宫产2次，孕周39+5，临产类别为自然临产。自然破膜，羊水量约200ml，羊水性质为清。以剖宫产分娩，产时出血约430ml。第一产程5时50分，第二产程未进入第二产程，第三产程0时5分，总产程5时55分，会阴或切口情况为完整。合并症及主要风险因素：瘢痕子宫临产；前次剖宫产术后。新生儿出生体重3450g，Apgar评分1、5、10分钟分别为10、10、10分，分娩结局为活产。 | |
| **临床病例摘要英文翻译 English Translation of De-identified Clinical Case Summary** | Maternal age was 35 years. The obstetric history was G2P2, with 2 previous cesarean deliveries. Gestational age was 39+5 weeks, and the labor onset or induction category was spontaneous labor. The membranes ruptured spontaneously; the amniotic fluid volume was approximately 200 mL and its character was clear. The patient underwent cesarean delivery, with estimated intrapartum blood loss of 430 mL. The first, second, and third stages of labor were 5 h 50 min, the second stage was not reached, and 0 h 5 min, respectively; the total labor duration was 5 h 55 min. The perineum was intact. Documented comorbidities and major risk factors were labor with a scarred uterus; history of previous cesarean delivery. Neonatal birth weight was 3450 g; Apgar scores at 1, 5, and 10 minutes were 10, 10, 10; and the delivery outcome was live birth. | |
| **模型生成的产褥期护理计划 Model-generated postpartum nursing care plan** | 产妇35岁，G2P2，孕39+5周，经剖宫产分娩，产时出血约430ml，新生儿男婴、体重3450g，Apgar评分10、10、10分。病例提示的重点风险为高龄妊娠因素。产后24小时内重点评估体温、脉搏、血压、宫底高度、宫缩、阴道流血、恶露颜色和尿潴留，记录首次排尿和疼痛评分。产后住院及出院后继续观察恶露是否由红色逐渐转淡，乳房是否胀痛，乳头是否皲裂，睡眠和情绪是否稳定。饮食以优质蛋白、含铁食物、蔬菜水果和足量饮水为主，避免盲目大补和长期卧床。本次为剖宫产，腹部切口和血栓预防是护理重点。切口应保持清洁干燥，观察红肿、渗血、渗液、裂开、异味和疼痛加重。翻身、咳嗽和下床时保护切口，在生命体征平稳后尽早床旁活动，关注肠鸣音恢复、排气排便、尿潴留和下肢肿痛。哺乳方面建议尽早母婴皮肤接触和按需哺乳，指导正确含接，乳胀时先热敷或轻柔按摩再哺乳，乳头疼痛时及时调整姿势。若出现乳房局部红肿硬痛、寒战或高热，应警惕乳腺炎。产时出血量需要持续观察，建议每日记录恶露量、颜色和血块情况，活动后若明显增多，应减少活动并请医护评估。瘢痕子宫或再次剖宫产者，应特别关注切口疼痛、恶露异常和子宫复旧，产后复查时结合病情评估子宫切口恢复。高龄产妇恢复期应避免过度劳累和连续熬夜，家属应参与夜间照护，保证睡眠、营养和情绪支持。出院前应完成用药、伤口、哺乳和避孕宣教，42天左右复查，必要时提前复诊。若出现阴道流血突然增多、鲜红血浸湿卫生巾、恶露恶臭、发热、持续腹痛、伤口化脓、排尿困难、下肢单侧肿痛、胸闷气短，或持续悲伤焦虑及伤害自己或婴儿的想法，应立即就医或急诊处理。 | 患者35岁，G2P2，急诊剖宫产，合并瘢痕子宫临产（一次剖宫产史），产时出血430ml，新生儿Apgar 10。瘢痕子宫临产剖宫产需注意子宫破裂风险，术后观察腹痛、出血。常规剖宫产护理。避孕建议。42天复查。 |
| **护理计划英文翻译 English translation of care plan** | The patient is 35 years old, G2P2, delivered at 39+5 weeks by cesarean delivery, with an estimated intrapartum blood loss of 430 mL. The newborn was a male infant, weighed 3450 g, and had Apgar scores of 10, 10, 10. The main case-based risks include advanced maternal age. Within the first 24 hours, assess temperature, pulse, blood pressure, uterine fundal height, uterine tone, vaginal bleeding, lochia color, urinary retention, first voiding, and pain score. During hospitalization and after discharge, continue to observe whether lochia gradually changes from red to lighter color, whether breast engorgement or nipple fissure occurs, and whether sleep and mood remain stable. Diet should include high-quality protein, iron-rich food, vegetables, fruit, and adequate fluids. Avoid blind over-supplementation and prolonged bed rest. Because this was a cesarean delivery, abdominal incision care and thrombosis prevention are key. Keep the incision clean and dry and observe for redness, swelling, bleeding, drainage, separation, odor, or worsening pain. Support the incision when turning, coughing, or getting out of bed. Once vital signs are stable, begin assisted ambulation and monitor bowel sounds, passage of gas, bowel movement, urinary retention, and leg swelling or pain. For breastfeeding, encourage early skin-to-skin contact and feeding on demand. Teach correct latch and positioning. For engorgement, apply warmth or gentle massage before feeding. If nipple pain occurs, adjust latch promptly. Localized breast redness, hard painful swelling, chills, or high fever should raise concern for mastitis. Because blood loss requires closer observation, record the amount, color, and clots in the lochia every day. If bleeding increases clearly after activity, reduce activity and seek clinical assessment. For scarred uterus or repeat cesarean delivery, pay close attention to incision pain, abnormal lochia, and uterine involution. Uterine scar recovery should be reviewed during postpartum follow-up when indicated. Advanced maternal age may slow recovery, so the family should help with nighttime care and support adequate sleep, nutrition, and emotional stability. Before discharge, provide education on medication, wound care, breastfeeding, and contraception. Arrange a postpartum review around 42 days after delivery, or earlier if symptoms occur. Seek urgent medical care for sudden heavy vaginal bleeding, bright red bleeding soaking pads, foul-smelling lochia, fever, persistent abdominal pain, pus from the wound, difficulty urinating, unilateral leg swelling or pain, chest tightness, shortness of breath, or persistent sadness, anxiety, or thoughts of harming herself or the baby. | The patient is a 35-year-old G2P2 who underwent emergency cesarean section for labor with a scarred uterus (one prior cesarean). Blood loss 430 mL, newborn Apgar 10. Monitor for uterine rupture signs (abdominal pain, bleeding). Routine post-cesarean care. Contraception counseling. Follow-up at 42 days. |
| **Clinical appropriateness 临床适当性** | 5 | 3 |
| **Individualization 个体化程度** | 4 | 3 |
| **Comprehensiveness 完整性** | 5 | 3 |
| **Actionability 可操作性** | 4 | 3 |
| **Safety 安全性** | 5 | 3 |
| **Triage appropriateness 分诊/转诊建议适当性** | 5 | 3 |
| **Structure and clarity 结构与清晰度** | 3 | 4 |

**病例 119 / Case 119**

| **项目 Item** | **ChatGPT 5.5** | **DeepSeek V3** |
| --- | --- | --- |
| **去标识化临床病例摘要 De-identified Clinical Case Summary** | 患者28岁，孕产史为G1P1，既往剖宫产1次，孕周38+6，临产类别为未临产。人工破膜，羊水量约260ml，羊水性质为清。以剖宫产分娩，产时出血约370ml。第一产程未进入产程，第二产程未进入第二产程，第三产程0时6分，总产程未进入产程，会阴或切口情况为完整。合并症及主要风险因素：胎儿先天性心脏病？产前诊断后计划剖宫产。新生儿出生体重2940g，Apgar评分1、5、10分钟分别为9、10、10分，分娩结局为活产。 | |
| **临床病例摘要英文翻译 English Translation of De-identified Clinical Case Summary** | Maternal age was 28 years. The obstetric history was G1P1, with 1 previous cesarean deliveries. Gestational age was 38+6 weeks, and the labor onset or induction category was no labor. The membranes were artificially ruptured; the amniotic fluid volume was approximately 260 mL and its character was clear. The patient underwent cesarean delivery, with estimated intrapartum blood loss of 370 mL. The first, second, and third stages of labor were labor was not established, the second stage was not reached, and 0 h 6 min, respectively; the total labor duration was labor was not established. The perineum was intact. Documented comorbidities and major risk factors were fetal congenital heart disease suspected; planned cesarean section after prenatal diagnosis. Neonatal birth weight was 2940 g; Apgar scores at 1, 5, and 10 minutes were 9, 10, 10; and the delivery outcome was live birth. | |
| **模型生成的产褥期护理计划 Model-generated postpartum nursing care plan** | 产妇28岁，G1P1，孕38+6周，经剖宫产分娩，产时出血约370ml，新生儿女婴、体重2940g，Apgar评分9、10、10分。病例提示的重点风险为胎儿窘迫或羊水异常记录。产后24小时内重点评估体温、脉搏、血压、宫底高度、宫缩、阴道流血、恶露颜色和尿潴留，记录首次排尿和疼痛评分。产后住院及出院后继续观察恶露是否由红色逐渐转淡，乳房是否胀痛，乳头是否皲裂，睡眠和情绪是否稳定。饮食以优质蛋白、含铁食物、蔬菜水果和足量饮水为主，避免盲目大补和长期卧床。本次为剖宫产，腹部切口和血栓预防是护理重点。切口应保持清洁干燥，观察红肿、渗血、渗液、裂开、异味和疼痛加重。翻身、咳嗽和下床时保护切口，在生命体征平稳后尽早床旁活动，关注肠鸣音恢复、排气排便、尿潴留和下肢肿痛。哺乳方面建议尽早母婴皮肤接触和按需哺乳，指导正确含接，乳胀时先热敷或轻柔按摩再哺乳，乳头疼痛时及时调整姿势。若出现乳房局部红肿硬痛、寒战或高热，应警惕乳腺炎。因有胎儿窘迫、羊水污染或新生儿评分变化记录，母婴同室期间应配合观察新生儿吃奶、肤色、体温、呼吸和反应，发现吃奶差、发绀、嗜睡或呼吸异常应立即告知儿科医生。出院前应完成用药、伤口、哺乳和避孕宣教，42天左右复查，必要时提前复诊。若出现阴道流血突然增多、鲜红血浸湿卫生巾、恶露恶臭、发热、持续腹痛、伤口化脓、排尿困难、下肢单侧肿痛、胸闷气短，或持续悲伤焦虑及伤害自己或婴儿的想法，应立即就医或急诊处理。 | 患者28岁，G1P1，剖宫产（计划），合并胎儿先天性心脏病可疑、产前诊断后计划剖宫产，产时出血370ml，新生儿Apgar 9-10-10。新生儿先天性心脏病需新生儿科/心内科评估，可能需转院。产妇剖宫产术后常规护理。提供心理支持。新生儿心脏专科随访；产妇42天复查。 |
| **护理计划英文翻译 English translation of care plan** | The patient is 28 years old, G1P1, delivered at 38+6 weeks by cesarean delivery, with an estimated intrapartum blood loss of 370 mL. The newborn was a female infant, weighed 2940 g, and had Apgar scores of 9, 10, 10. The main case-based risks include fetal distress or abnormal amniotic fluid findings. Within the first 24 hours, assess temperature, pulse, blood pressure, uterine fundal height, uterine tone, vaginal bleeding, lochia color, urinary retention, first voiding, and pain score. During hospitalization and after discharge, continue to observe whether lochia gradually changes from red to lighter color, whether breast engorgement or nipple fissure occurs, and whether sleep and mood remain stable. Diet should include high-quality protein, iron-rich food, vegetables, fruit, and adequate fluids. Avoid blind over-supplementation and prolonged bed rest. Because this was a cesarean delivery, abdominal incision care and thrombosis prevention are key. Keep the incision clean and dry and observe for redness, swelling, bleeding, drainage, separation, odor, or worsening pain. Support the incision when turning, coughing, or getting out of bed. Once vital signs are stable, begin assisted ambulation and monitor bowel sounds, passage of gas, bowel movement, urinary retention, and leg swelling or pain. For breastfeeding, encourage early skin-to-skin contact and feeding on demand. Teach correct latch and positioning. For engorgement, apply warmth or gentle massage before feeding. If nipple pain occurs, adjust latch promptly. Localized breast redness, hard painful swelling, chills, or high fever should raise concern for mastitis. Because fetal distress, meconium-stained fluid, or changed Apgar scores were recorded, cooperate with pediatric observation of feeding, skin color, temperature, breathing, and responsiveness. Poor feeding, cyanosis, excessive sleepiness, or abnormal breathing should be reported immediately. Before discharge, provide education on medication, wound care, breastfeeding, and contraception. Arrange a postpartum review around 42 days after delivery, or earlier if symptoms occur. Seek urgent medical care for sudden heavy vaginal bleeding, bright red bleeding soaking pads, foul-smelling lochia, fever, persistent abdominal pain, pus from the wound, difficulty urinating, unilateral leg swelling or pain, chest tightness, shortness of breath, or persistent sadness, anxiety, or thoughts of harming herself or the baby. | The patient is a 28-year-old G1P1 who underwent planned cesarean section for suspected fetal congenital heart disease. Blood loss 370 mL, newborn Apgar 9-10-10. Newborn requires cardiology evaluation; possible transfer. Routine post-cesarean care. Provide psychological support. Newborn cardiology follow-up; maternal check at 42 days. |
| **Clinical appropriateness 临床适当性** | 4 | 4 |
| **Individualization 个体化程度** | 4 | 4 |
| **Comprehensiveness 完整性** | 4 | 3 |
| **Actionability 可操作性** | 4 | 3 |
| **Safety 安全性** | 5 | 4 |
| **Triage appropriateness 分诊/转诊建议适当性** | 5 | 3 |
| **Structure and clarity 结构与清晰度** | 4 | 4 |

**病例 120 / Case 120**

| **项目 Item** | **ChatGPT 5.5** | **DeepSeek V3** |
| --- | --- | --- |
| **去标识化临床病例摘要 De-identified Clinical Case Summary** | 患者34岁，孕产史为G2P1，既往剖宫产1次，孕周40+3，临产类别为阴道放置普贝生。人工破膜，羊水量约220ml，羊水性质为Ⅲ°。以剖宫产分娩，产时出血约460ml。第一产程4时20分，第二产程未进入第二产程，第三产程0时5分，总产程4时25分，会阴或切口情况为完整。合并症及主要风险因素：引产过程中胎儿宫内窘迫？羊水Ⅲ°污染。新生儿出生体重3330g，Apgar评分1、5、10分钟分别为8、9、10分，分娩结局为活产。 | |
| **临床病例摘要英文翻译 English Translation of De-identified Clinical Case Summary** | Maternal age was 34 years. The obstetric history was G2P1, with 1 previous cesarean deliveries. Gestational age was 40+3 weeks, and the labor onset or induction category was vaginal dinoprostone (Propess) induction. The membranes were artificially ruptured; the amniotic fluid volume was approximately 220 mL and its character was grade III meconium-stained. The patient underwent cesarean delivery, with estimated intrapartum blood loss of 460 mL. The first, second, and third stages of labor were 4 h 20 min, the second stage was not reached, and 0 h 5 min, respectively; the total labor duration was 4 h 25 min. The perineum was intact. Documented comorbidities and major risk factors were fetal distress during induction suspected; grade III meconium-stained amniotic fluid. Neonatal birth weight was 3330 g; Apgar scores at 1, 5, and 10 minutes were 8, 9, 10; and the delivery outcome was live birth. | |
| **模型生成的产褥期护理计划 Model-generated postpartum nursing care plan** | 产妇34岁，G2P1，孕40+3周，经剖宫产分娩，产时出血约460ml，新生儿男婴、体重3330g，Apgar评分8、9、10分。病例提示的重点风险为胎儿窘迫或羊水异常记录。产后24小时内重点评估体温、脉搏、血压、宫底高度、宫缩、阴道流血、恶露颜色和尿潴留，记录首次排尿和疼痛评分。产后住院及出院后继续观察恶露是否由红色逐渐转淡，乳房是否胀痛，乳头是否皲裂，睡眠和情绪是否稳定。饮食以优质蛋白、含铁食物、蔬菜水果和足量饮水为主，避免盲目大补和长期卧床。本次为剖宫产，腹部切口和血栓预防是护理重点。切口应保持清洁干燥，观察红肿、渗血、渗液、裂开、异味和疼痛加重。翻身、咳嗽和下床时保护切口，在生命体征平稳后尽早床旁活动，关注肠鸣音恢复、排气排便、尿潴留和下肢肿痛。哺乳方面建议尽早母婴皮肤接触和按需哺乳，指导正确含接，乳胀时先热敷或轻柔按摩再哺乳，乳头疼痛时及时调整姿势。若出现乳房局部红肿硬痛、寒战或高热，应警惕乳腺炎。产时出血量需要持续观察，建议每日记录恶露量、颜色和血块情况，活动后若明显增多，应减少活动并请医护评估。因有胎儿窘迫、羊水污染或新生儿评分变化记录，母婴同室期间应配合观察新生儿吃奶、肤色、体温、呼吸和反应，发现吃奶差、发绀、嗜睡或呼吸异常应立即告知儿科医生。出院前应完成用药、伤口、哺乳和避孕宣教，42天左右复查，必要时提前复诊。若出现阴道流血突然增多、鲜红血浸湿卫生巾、恶露恶臭、发热、持续腹痛、伤口化脓、排尿困难、下肢单侧肿痛、胸闷气短，或持续悲伤焦虑及伤害自己或婴儿的想法，应立即就医或急诊处理。 | 患者34岁，G2P1，急诊剖宫产，合并引产过程中可疑胎儿窘迫、羊水Ⅲ°污染，脐带绕颈2周，产时出血460ml，新生儿Apgar 8-9-10。Ⅲ°羊水+脐带绕颈2周+窘迫，新生儿需密切监测MAS及HIE，建议新生儿科观察。出血460ml注意晚期出血。剖宫产术后常规护理。心理支持。产后1周复查血常规；新生儿专科随访。 |
| **护理计划英文翻译 English translation of care plan** | The patient is 34 years old, G2P1, delivered at 40+3 weeks by cesarean delivery, with an estimated intrapartum blood loss of 460 mL. The newborn was a male infant, weighed 3330 g, and had Apgar scores of 8, 9, 10. The main case-based risks include fetal distress or abnormal amniotic fluid findings. Within the first 24 hours, assess temperature, pulse, blood pressure, uterine fundal height, uterine tone, vaginal bleeding, lochia color, urinary retention, first voiding, and pain score. During hospitalization and after discharge, continue to observe whether lochia gradually changes from red to lighter color, whether breast engorgement or nipple fissure occurs, and whether sleep and mood remain stable. Diet should include high-quality protein, iron-rich food, vegetables, fruit, and adequate fluids. Avoid blind over-supplementation and prolonged bed rest. Because this was a cesarean delivery, abdominal incision care and thrombosis prevention are key. Keep the incision clean and dry and observe for redness, swelling, bleeding, drainage, separation, odor, or worsening pain. Support the incision when turning, coughing, or getting out of bed. Once vital signs are stable, begin assisted ambulation and monitor bowel sounds, passage of gas, bowel movement, urinary retention, and leg swelling or pain. For breastfeeding, encourage early skin-to-skin contact and feeding on demand. Teach correct latch and positioning. For engorgement, apply warmth or gentle massage before feeding. If nipple pain occurs, adjust latch promptly. Localized breast redness, hard painful swelling, chills, or high fever should raise concern for mastitis. Because blood loss requires closer observation, record the amount, color, and clots in the lochia every day. If bleeding increases clearly after activity, reduce activity and seek clinical assessment. Because fetal distress, meconium-stained fluid, or changed Apgar scores were recorded, cooperate with pediatric observation of feeding, skin color, temperature, breathing, and responsiveness. Poor feeding, cyanosis, excessive sleepiness, or abnormal breathing should be reported immediately. Before discharge, provide education on medication, wound care, breastfeeding, and contraception. Arrange a postpartum review around 42 days after delivery, or earlier if symptoms occur. Seek urgent medical care for sudden heavy vaginal bleeding, bright red bleeding soaking pads, foul-smelling lochia, fever, persistent abdominal pain, pus from the wound, difficulty urinating, unilateral leg swelling or pain, chest tightness, shortness of breath, or persistent sadness, anxiety, or thoughts of harming herself or the baby. | The patient is a 34-year-old G2P1 who underwent emergency cesarean section for suspected fetal distress during induction, grade III meconium, nuchal cord 2 loops. Blood loss 460 mL, newborn Apgar 8-9-10. Newborn – close monitoring for MAS and HIE; pediatric observation recommended. Watch for late PPH. Routine post-cesarean care. Psychological support. Follow-up: CBC at 1 week; newborn specialist follow-up. |
| **Clinical appropriateness 临床适当性** | 5 | 4 |
| **Individualization 个体化程度** | 4 | 5 |
| **Comprehensiveness 完整性** | 5 | 3 |
| **Actionability 可操作性** | 4 | 4 |
| **Safety 安全性** | 5 | 4 |
| **Triage appropriateness 分诊/转诊建议适当性** | 5 | 3 |
| **Structure and clarity 结构与清晰度** | 4 | 4 |
