# Supplementary material for: Beyond accuracy: a three-stage evaluation of large language models for postpartum health consultation and nursing decision support
Source: Front Public Health. 2026 Oct 20;14:1961647. doi: 10.3389/fpubh.2026.1961647 (PMC13727638; doi:10.3389/fpubh.2026.1961647)
Supplement: Supplementary file 5 [file Table_2.docx]

**Supplementary Table S2. Chinese and English readability categories and reporting thresholds**

| **Readability measure** | **Score range or rule** | **Reported category** | **Interpretation** |
| --- | --- | --- | --- |
| **Panel A. Chinese readability outcomes** | | | |
| Chinese reading-difficulty score | <14.50 | Junior high school level | Lower Chinese reading difficulty |
| Chinese reading-difficulty score | ≥14.50 | Senior high school level | Higher Chinese reading difficulty |
| Recommended Chinese reading age | Score rounded to the nearest whole year | Approximate age in years | For example, 13.39 is reported as approximately 13 years, whereas 14.53 is reported as approximately 15 years |
| **Panel B. Flesch–Kincaid Grade Level** | | | |
| Flesch–Kincaid Grade Level | ≤7.00 | Grade 7 or below | Fairly easy to read |
| Flesch–Kincaid Grade Level | >7.00 to 9.00 | Grades 8–9 | Plain English |
| Flesch–Kincaid Grade Level | >9.00 to 12.00 | Grades 10–12 | Fairly difficult |
| Flesch–Kincaid Grade Level | >12.00 to 16.00 | College | Difficult |
| Flesch–Kincaid Grade Level | >16.00 | College graduate | Very difficult |

Note: Higher values indicate greater reading difficulty. The Chinese education-level threshold was applied to the Chinese reading-difficulty scores in the study dataset. Recommended Chinese reading age was obtained by rounding the score to the nearest whole year. For the English analysis, the original Flesch–Kincaid categories of Grade 5 or below, Grade 6, and Grade 7 were combined as “Grade 7 or below” to form the five prespecified ordinal categories used in the analysis. Flesch–Kincaid Grade Level estimates the US school grade required to understand a text. References correspond to references 20 and 21 in the main manuscript.
