# Supplementary material for: Beyond accuracy: a three-stage evaluation of large language models for postpartum health consultation and nursing decision support
Source: Front Public Health. 2026 Oct 20;14:1961647. doi: 10.3389/fpubh.2026.1961647 (PMC13727638; doi:10.3389/fpubh.2026.1961647)
Supplement: Supplementary file 6 [file Table_3.docx]

**Supplementary Table S3. Detailed between-model comparisons across the three study stages**

| **Outcome/domain** | **N** | **ChatGPT 5.5 Mean ± SD** | **DeepSeek V3 Mean ± SD** | **ChatGPT 5.5 Median (P25, P75)** | **DeepSeek V3 Median (P25, P75)** | **Test** | **Statistic** | **Raw P** | **Holm-adjusted P** |
| --- | --- | --- | --- | --- | --- | --- | --- | --- | --- |
| **Panel A. Stage 1: Guideline-based clinical questions and readability outcomes** | | | | | | | | | |
| Correctness | 90 | 4.00 ± 0.00 | 3.88 ± 0.33 | 4.00 (4.00, 4.00) | 4.00 (4.00, 4.00) | Wilcoxon signed-rank | W = 0.000 | 0.0009 | 0.0018 |
| Completeness | 90 | 3.99 ± 0.49 | 4.63 ± 0.61 | 4.00 (4.00, 4.00) | 5.00 (4.00, 5.00) | Wilcoxon signed-rank | W = 340.000 | <0.0001 | <0.0001 |
| Guideline consistency | 90 | 3.89 ± 0.44 | 3.88 ± 0.33 | 4.00 (4.00, 4.00) | 4.00 (4.00, 4.00) | Wilcoxon signed-rank | W = 196.000 | 0.8575 | 0.8575 |
| Safety | 90 | 4.24 ± 0.43 | 4.48 ± 0.57 | 4.00 (4.00, 4.00) | 5.00 (4.00, 5.00) | Wilcoxon signed-rank | W = 96.000 | 0.0004 | 0.0012 |
| Clinical management appropriateness | 90 | 3.91 ± 0.55 | 4.40 ± 0.75 | 4.00 (4.00, 4.00) | 5.00 (4.00, 5.00) | Wilcoxon signed-rank | W = 639.000 | <0.0001 | 0.0002 |
| Chinese reading-difficulty score | 90 | 13.68 ± 0.59 | 14.94 ± 0.63 | 13.54 (13.32, 13.86) | 14.86 (14.55, 15.34) | Wilcoxon signed-rank | W = 93.000 | <0.0001 | <0.0001 |
| Recommended Chinese reading age, years | 90 | 13.58 ± 0.76 | 14.97 ± 0.74 | 13.00 (13.00, 14.00) | 15.00 (15.00, 15.00) | Wilcoxon signed-rank | W = 112.500 | <0.0001 | <0.0001 |
| Chinese educational level | 90 | Junior high: 81 (90.0%); Senior high: 9 (10.0%) | Junior high: 21 (23.3%); Senior high: 69 (76.7%) | — | — | Wilcoxon signed-rank | W = 65.000 | <0.0001 | <0.0001 |
| Flesch–Kincaid Grade Level | 90 | 12.89 ± 2.29 | 13.85 ± 3.32 | 12.98 (11.40, 14.95) | 13.38 (11.87, 15.66) | Paired t test | t = 2.716 | 0.0079 | 0.0159 |
| English reading-difficulty category | 90 | 7th: 0 (0.0%); 8th–9th: 3 (3.3%); 10th–12th: 29 (32.2%); College: 53 (58.9%); Graduate: 5 (5.6%) | 7th: 1 (1.1%); 8th–9th: 5 (5.6%); 10th–12th: 19 (21.1%); College: 43 (47.8%); Graduate: 22 (24.4%) | — | — | Wilcoxon signed-rank | W = 359.000 | 0.0208 | 0.0208 |
| **Panel B. Stage 2: Responses to real postpartum consultation questions** | | | | | | | | | |
| Patient satisfaction | 120 | 4.30 ± 0.46 | 3.00 ± 0.00 | 4.00 (4.00, 5.00) | 3.00 (3.00, 3.00) | Wilcoxon signed-rank | Z = -9.973 | <0.0001 | <0.0001 |
| Patient understandability | 120 | 4.03 ± 0.16 | 4.00 ± 0.00 | 4.00 (4.00, 4.00) | 4.00 (4.00, 4.00) | Wilcoxon signed-rank | Z = -1.732 | 0.0833 | 0.0833 |
| Patient usefulness | 120 | 4.22 ± 0.41 | 3.00 ± 0.00 | 4.00 (4.00, 4.00) | 3.00 (3.00, 3.00) | Wilcoxon signed-rank | Z = -10.141 | <0.0001 | <0.0001 |
| Clinician-rated correctness | 120 | 4.75 ± 0.43 | 3.35 ± 0.48 | 5.00 (4.75, 5.00) | 3.00 (3.00, 4.00) | Wilcoxon signed-rank | Z = -9.361 | <0.0001 | <0.0001 |
| Clinician-rated completeness | 120 | 4.68 ± 0.47 | 3.00 ± 0.00 | 5.00 (4.00, 5.00) | 3.00 (3.00, 3.00) | Wilcoxon signed-rank | Z = -9.947 | <0.0001 | <0.0001 |
| Clinician-rated readability | 120 | 4.02 ± 0.13 | 4.60 ± 0.49 | 4.00 (4.00, 4.00) | 5.00 (4.00, 5.00) | Wilcoxon signed-rank | Z = 8.137 | <0.0001 | <0.0001 |
| Clinician-rated helpfulness | 120 | 4.71 ± 0.46 | 3.00 ± 0.00 | 5.00 (4.00, 5.00) | 3.00 (3.00, 3.00) | Wilcoxon signed-rank | Z = -9.987 | <0.0001 | <0.0001 |
| Clinician-rated safety | 120 | 4.90 ± 0.30 | 3.35 ± 0.48 | 5.00 (5.00, 5.00) | 3.00 (3.00, 4.00) | Wilcoxon signed-rank | Z = -9.661 | <0.0001 | <0.0001 |
| **Panel C. Stage 3: Model-generated postpartum maternal nursing care plans** | | | | | | | | | |
| Clinical appropriateness | 120 | 4.66 ± 0.48 | 3.61 ± 0.49 | 5.00 (4.00, 5.00) | 4.00 (3.00, 4.00) | Wilcoxon signed-rank | Z = -9.526 | <0.0001 | <0.0001 |
| Individualization | 120 | 4.07 ± 0.80 | 4.24 ± 0.88 | 4.00 (3.00, 5.00) | 5.00 (3.00, 5.00) | Wilcoxon signed-rank | Z = 2.559 | 0.0105 | 0.0210 |
| Comprehensiveness | 120 | 4.66 ± 0.48 | 3.39 ± 0.49 | 5.00 (4.00, 5.00) | 3.00 (3.00, 4.00) | Wilcoxon signed-rank | Z = -9.504 | <0.0001 | <0.0001 |
| Actionability | 120 | 4.16 ± 0.37 | 3.65 ± 0.48 | 4.00 (4.00, 4.00) | 4.00 (3.00, 4.00) | Wilcoxon signed-rank | Z = -7.685 | <0.0001 | <0.0001 |
| Safety | 120 | 5.00 ± 0.00 | 3.61 ± 0.49 | 5.00 (5.00, 5.00) | 4.00 (3.00, 4.00) | Wilcoxon signed-rank | Z = -9.860 | <0.0001 | <0.0001 |
| Triage appropriateness | 120 | 5.00 ± 0.00 | 3.27 ± 0.44 | 5.00 (5.00, 5.00) | 3.00 (3.00, 4.00) | Wilcoxon signed-rank | Z = -10.032 | <0.0001 | <0.0001 |
| Structure and clarity | 120 | 3.68 ± 0.47 | 3.70 ± 0.46 | 4.00 (3.00, 4.00) | 4.00 (3.00, 4.00) | Wilcoxon signed-rank | Z = -0.246 | 0.8055 | 0.8055 |

Note: Data are presented as mean ± SD and median (P25, P75) for continuous and Likert-scale outcomes, and as n (%) for categorical readability outcomes. N denotes the number of paired response units. Likert-scale outcomes and ordinal readability categories were compared using the Wilcoxon signed-rank test. Continuous readability outcomes were tested according to the distribution of paired differences; Flesch–Kincaid Grade Level was compared using a paired t test. Because multiple related outcome dimensions were compared between the two models, the Holm–Bonferroni procedure was applied within five conceptually defined outcome families: Stage 1 expert-rated clinical dimensions, Stage 1 readability outcomes, Stage 2 patient-rated outcomes, Stage 2 clinician-rated outcomes, and Stage 3 expert-rated nursing care-plan outcomes. Both unadjusted (Raw P) and Holm-adjusted P values are reported. Statistical significance after multiplicity correction was determined using the Holm-adjusted P values. All P values are two-sided. SD, standard deviation; P25, 25th percentile; P75, 75th percentile.
