# Supplementary material for: Beyond accuracy: a three-stage evaluation of large language models for postpartum health consultation and nursing decision support
Source: Front Public Health. 2026 Oct 20;14:1961647. doi: 10.3389/fpubh.2026.1961647 (PMC13727638; doi:10.3389/fpubh.2026.1961647)
Supplement: Supplementary file 7 [file Table_4.docx]

**Supplementary Table S4. Stage- and dimension-specific intraclass correlation coefficient results**

| **Domain** | **n** | **k** | **ICC(2,1)** | **Interpretation** | **ICC(2,3)** | **Interpretation** | **MSR** | **MSC** | **MSE** |
| --- | --- | --- | --- | --- | --- | --- | --- | --- | --- |
| **Panel A. Stage 1: Clinical expert ratings of Chinese responses to guideline-based clinical questions** | | | | | | | | | |
| Correctness | 180 | 3 | 0.204 | Poor | 0.435 | Poor | 0.361 | 13.072 | 0.173 |
| Completeness | 180 | 3 | 0.362 | Poor | 0.630 | Moderate | 0.721 | 2.924 | 0.257 |
| Guideline consistency | 180 | 3 | 0.325 | Poor | 0.591 | Moderate | 0.531 | 8.957 | 0.188 |
| Clinical management appropriateness | 180 | 3 | 0.470 | Poor | 0.726 | Moderate | 0.937 | 3.467 | 0.243 |
| Safety | 180 | 3 | 0.186 | Poor | 0.406 | Poor | 0.438 | 5.030 | 0.249 |
| **Panel B. Stage 2: Clinical expert ratings of responses to real postpartum consultation questions** | | | | | | | | | |
| Correctness | 240 | 3 | 0.741 | Moderate | 0.895 | Good | 1.645 | 0.485 | 0.171 |
| Completeness | 240 | 3 | 0.826 | Good | 0.934 | Excellent | 1.949 | 0.156 | 0.128 |
| Readability | 240 | 3 | 0.339 | Poor | 0.606 | Moderate | 0.564 | 1.851 | 0.218 |
| Helpfulness | 240 | 3 | 0.791 | Good | 0.919 | Excellent | 1.921 | 0.487 | 0.154 |
| Safety | 240 | 3 | 0.740 | Moderate | 0.895 | Good | 1.720 | 0.418 | 0.180 |
| **Panel C. Stage 3: Clinical expert ratings of model-generated postpartum maternal nursing care plans** | | | | | | | | | |
| Clinical appropriateness | 240 | 3 | 0.595 | Moderate | 0.815 | Good | 1.177 | 5.960 | 0.198 |
| Individualization | 240 | 3 | 0.699 | Moderate | 0.875 | Good | 1.563 | 2.606 | 0.187 |
| Comprehensiveness | 240 | 3 | 0.711 | Moderate | 0.880 | Good | 1.527 | 4.593 | 0.166 |
| Actionability | 240 | 3 | 0.477 | Poor | 0.732 | Moderate | 0.725 | 11.760 | 0.159 |
| Safety | 240 | 3 | 0.638 | Moderate | 0.841 | Good | 1.378 | 3.268 | 0.208 |
| Triage appropriateness | 240 | 3 | 0.749 | Moderate | 0.899 | Good | 1.870 | 1.918 | 0.182 |
| Structure and clarity | 240 | 3 | 0.446 | Poor | 0.708 | Moderate | 0.622 | 13.751 | 0.142 |

Note: ICCs were calculated using a two-way random-effects, absolute-agreement model. ICC(2,1) represents single-rater reliability, and ICC(2,3) represents the reliability of the mean rating of the three clinical experts. For each stage, response units generated by ChatGPT and DeepSeek were pooled before calculating ICCs for each domain. Stage 1 included 180 Chinese responses; Stage 2 included 240 composite evaluation units; and Stage 3 included 240 postpartum maternal nursing care plans. Reliability was interpreted as poor (<0.50), moderate (0.50 to <0.75), good (0.75 to 0.90), or excellent (>0.90). MSR, mean square for rows (targets); MSC, mean square for columns (raters); MSE, residual mean square; n, number of pooled response units or care plans across both models; k, number of raters.
