# Supplementary material for: Beyond accuracy: a three-stage evaluation of large language models for postpartum health consultation and nursing decision support
Source: Front Public Health. 2026 Oct 20;14:1961647. doi: 10.3389/fpubh.2026.1961647 (PMC13727638; doi:10.3389/fpubh.2026.1961647)
Supplement: Supplementary file 8 [file Table_5.docx]

**Supplementary Table S5. Model access, platform, testing-period, and generation settings**

| **Model** | **Provider** | **Access mode** | **Version/model name** | **Access period** | **Generation settings / decoding parameters** |
| --- | --- | --- | --- | --- | --- |
| ChatGPT 5.5 | OpenAI | Public web interface | ChatGPT 5.5 Thinking | 1 May to 30 June 2026 | The platform-displayed Thinking mode was enabled; web browsing/search was disabled; all other accessible web-interface settings remained at their defaults. Temperature, top-p, random seed, maximum output tokens, and other decoding parameters were not accessible or manually adjusted. |
| DeepSeek V3 | DeepSeek | Public web interface | DeepSeek V3 Deep Thinking | 1 May to 30 June 2026 | The platform-displayed Deep Thinking mode was enabled; web browsing/search was disabled; all other accessible web-interface settings remained at their defaults. Temperature, top-p, random seed, maximum output tokens, and other decoding parameters were not accessible or manually adjusted. |

Note: Both models were accessed through their official public web interfaces; no application programming interface was used. Web browsing/search was disabled for both models. Apart from the prespecified questions, prompts, or de-identified clinical case information, no additional background materials were provided. Each question or case was entered in a new independent chat. In Stage 1, the same prespecified task was administered separately in Chinese and English, and each question was submitted three independent times to each model. In Stages 2 and 3, prespecified Chinese prompts were used and only the first generated response was retained; regeneration, continuation, and manual follow-up were not used. All model outputs retained for analysis were saved verbatim without deletion, correction, or manual editing. Because the public web interfaces did not expose decoding controls, temperature, top-p, random seed, token limits, and related parameters could not be standardized manually.
