# Supplementary material for: Beyond accuracy: a three-stage evaluation of large language models for postpartum health consultation and nursing decision support
Source: Front Public Health. 2026 Oct 20;14:1961647. doi: 10.3389/fpubh.2026.1961647 (PMC13727638; doi:10.3389/fpubh.2026.1961647)
Supplement: Supplementary file 9 [file Table_6.docx]

**Supplementary Table S6. Stage 1 ChatGPT 5.5 English clinical knowledge responses and readability outcomes**

*30 postpartum maternal health clinical knowledge questions; each question was independently submitted to ChatGPT 5.5 three times.*

| Study stage | Stage 1: Clinical knowledge questions on postpartum maternal health |
| --- | --- |
| Model under evaluation | ChatGPT 5.5 |
| Exact model/version displayed | ChatGPT 5.5 Thinking |
| Access platform | Official ChatGPT web interface |
| Testing period | 1 May to 30 June 2026 |
| Web browsing/search | Off |
| Reasoning/deep-thinking mode | On; ChatGPT 5.5 Thinking |
| External files or reference materials uploaded | No |
| Submission procedure | Each question was entered into a new independent conversation and submitted to the model three times. |
| Unified prompt | Act as an evidence-informed obstetrics clinician and answer the following clinical knowledge question about postpartum maternal health. Base your answer on authoritative guidelines and clinical principles. Include key assessment points, possible causes, initial management principles, red flags, and referral indications. Avoid fabricating uncertain information. |
| Evaluator | Not applicable (automated readability assessment only) |
| Language | English |

# **Readability assessment**

Clinical expert scoring was not performed for the English-language responses. These responses were used exclusively for automated Flesch–Kincaid readability assessment.

## Question 1

| Item | Attempt 1 | Attempt 2 | Attempt 3 |
| --- | --- | --- | --- |
| Clinical knowledge question | What is the definition of the puerperium, and which domains should be prioritized in postpartum maternal health management? | | |
| Main guideline source(s) | WHO 2022; ACOG Optimizing Postpartum Care | | |
| Model response | The puerperium is the period after childbirth during which the mother’s reproductive organs and whole body gradually recover from pregnancy and delivery. In the traditional obstetric definition, it usually lasts about 6 weeks after birth, although many physical and psychological changes continue beyond this time. The postpartum period is also increasingly described as the fourth trimester, because maternal recovery and newborn adaptation require continuous care rather than only one checkup.  Postpartum maternal health management should prioritize several domains. The first is physical recovery, including uterine involution, lochia, perineal or cesarean wound healing, pain, bladder and bowel function, anemia, blood pressure, and signs of hemorrhage, infection, or thromboembolism. The second is lactation and breast health, including breastfeeding support, mastitis prevention, nipple trauma, and infant feeding problems. The third is mental health, especially screening for postpartum blues, postpartum depression, anxiety, trauma symptoms, and severe psychiatric symptoms. The fourth is chronic disease and pregnancy complication follow-up, such as hypertension, preeclampsia, gestational diabetes, thyroid disease, obesity, and cardiovascular risk. The fifth is family planning and sexual health, including contraception, return to sexual activity, pelvic floor recovery, and counseling on birth spacing. | The puerperium refers to the time from the completion of delivery of the placenta until the maternal body largely returns toward its pre-pregnancy state. Clinically, this is often considered the first 42 days postpartum, but modern postpartum care extends attention through the first 12 weeks and sometimes longer, depending on the woman’s condition and risks. ACOG emphasizes that postpartum care should be an ongoing, individualized process, not just a single 6-week visit.  The key management priorities are as follows.  First, clinicians should monitor maternal safety. This includes early recognition of postpartum hemorrhage, fever, uterine tenderness, wound infection, severe headache, visual symptoms, chest pain, dyspnea, leg swelling, seizures, and severe hypertension.  Second, care should address recovery of reproductive and pelvic function. This includes lochia changes, uterine involution, perineal pain, cesarean incision healing, urinary incontinence, constipation, hemorrhoids, pelvic floor symptoms, and dyspareunia.  Third, postpartum management must include emotional and social assessment. Sleep deprivation, fatigue, lack of support, intimate partner violence, birth trauma, depression, anxiety, and suicidal or infanticidal thoughts should be actively assessed.  Fourth, care should support infant feeding and maternal nutrition. Breastfeeding difficulties, breast engorgement, mastitis, maternal diet, hydration, anemia correction, and medication safety during lactation should be reviewed.  Fifth, the visit should include long-term health planning. Women with gestational diabetes, hypertensive disorders of pregnancy, preterm birth, obesity, or other complications need follow-up because these conditions may predict future metabolic and cardiovascular disease. | The puerperium is the postpartum recovery stage beginning immediately after birth. It includes the early period of physiological stabilization, the involution of the uterus, healing of birth-related trauma, establishment of lactation, and psychological adaptation to motherhood. Traditional teaching defines it as approximately 6 weeks, while many guidelines now recognize that recovery and health risks continue across the broader postpartum period. WHO recommends postnatal care contacts after birth, including contacts around 48 to 72 hours, 7 to 14 days, and 6 weeks for healthy women and newborns.  In postpartum maternal health management, priority should not be limited to checking whether the uterus has recovered. It should cover the whole person.  Clinicians should prioritize life-threatening complications first, including bleeding, infection, hypertensive emergencies, venous thromboembolism, cardiomyopathy, and severe psychiatric symptoms.  They should then assess functional recovery, including pain control, mobility, sleep, nutrition, bowel and bladder function, pelvic floor symptoms, wound healing, and ability to care for the newborn.  Another major domain is psychological adaptation, including mood, anxiety, bonding, family support, domestic safety, and adjustment to the maternal role.  Care should also include breastfeeding and newborn-related maternal concerns, because feeding problems often affect maternal sleep, pain, confidence, and emotional well-being.  Finally, postpartum care should prepare the woman for future health, including contraception, interpregnancy interval, chronic disease follow-up, vaccination, cervical screening when indicated, and transition back to primary or well-woman care. |
| **Flesch–Kincaid Grade Level** | 16.76 | 14.96 | 16.85 |
| **Corresponding English Reading-Difficulty Category** | College graduate: very difficult | College: difficult | College graduate: very difficult |

## Question 2

| Item | Attempt 1 | Attempt 2 | Attempt 3 |
| --- | --- | --- | --- |
| Clinical knowledge question | What are the main purposes of postnatal home visits and postpartum follow-up, and what should healthcare professionals ask about and examine in the early postpartum period? | | |
| Main guideline source(s) | WHO 2022; NICE NG194; Chinese Maternal Health Management Specification | | |
| Model response | The main purpose of postnatal home visits and postpartum follow-up is to make sure that the mother and newborn are recovering safely after birth, to detect complications early, and to support the family during the transition home. WHO recommends postnatal contacts after birth, including contact around 48 to 72 hours, 7 to 14 days, and 6 weeks, and also recommends home visits in the first week after birth for mother and newborn care.  In the early postpartum period, healthcare professionals should ask about vaginal bleeding, lochia color and odor, abdominal pain, fever, chills, headache, visual symptoms, chest pain, shortness of breath, leg swelling, wound pain, urination, bowel movements, sleep, appetite, fatigue, mood, anxiety, family support, and thoughts of self-harm or harm to the baby. They should also ask about breastfeeding or formula feeding, breast pain, nipple trauma, engorgement, and whether the baby is feeding and passing urine and stool.  The examination should include vital signs, especially temperature, pulse, and blood pressure. The uterus should be assessed for firmness and involution. Lochia should be evaluated for amount, color, and odor. The perineum or cesarean incision should be inspected for healing, redness, swelling, discharge, hematoma, or dehiscence. The breasts should be checked if there is pain, engorgement, nipple injury, or suspected mastitis. The legs should be assessed for calf tenderness, swelling, asymmetry, or signs of thrombosis. | Postnatal home visits and postpartum follow-up are not only routine checks. Their main aims are to identify danger signs, prevent avoidable maternal and neonatal morbidity, support breastfeeding and newborn care, assess the mother’s psychological state, and connect the family with further care when needed. ACOG emphasizes that postpartum care should be an ongoing, individualized process and should include physical, social, and psychological assessment, ending with a comprehensive visit no later than 12 weeks after birth.  Healthcare professionals should first ask whether the mother feels safe and whether she has any urgent symptoms. These include heavy bleeding, foul-smelling discharge, fever, severe abdominal pain, severe headache, visual changes, dizziness, fainting, chest pain, breathing difficulty, palpitations, unilateral leg pain or swelling, seizures, or thoughts of harming herself or the baby. These symptoms need immediate attention.  They should then ask about daily recovery. Important questions include whether pain is controlled, whether she can walk and care for the baby, whether she can pass urine normally, whether she has constipation or hemorrhoids, whether she is sleeping at all, whether she has enough help at home, and whether she feels persistently sad, anxious, overwhelmed, detached, or frightened.  The physical examination should focus on blood pressure, general appearance, pallor, hydration, uterine tone, abdominal tenderness, lochia, wound healing, breast condition, and lower-limb signs of venous thromboembolism. If there was hypertension, hemorrhage, infection, diabetes, cesarean delivery, severe perineal trauma, or preterm birth, the assessment should be more targeted and follow-up should be arranged sooner. | The purpose of postpartum follow-up is to shift care from delivery-centered care to recovery-centered care. After discharge, many serious problems appear at home rather than in hospital. Home visits help professionals observe the mother’s real living situation, check whether she understands warning signs, assess infant feeding, support maternal confidence, and decide whether referral or urgent evaluation is needed.  In the early postpartum period, the questioning should cover four areas.  First, ask about maternal danger signs. These include excessive vaginal bleeding, passing large clots, fever, foul lochia, worsening abdominal pain, severe headache, blurred vision, seizures, chest pain, shortness of breath, fainting, unilateral leg swelling, and thoughts of self-harm or harm to the infant. Urgent maternal warning signs after pregnancy include symptoms such as persistent or worsening headache, vision changes, fever, trouble breathing, chest pain, severe abdominal pain, heavy bleeding, and thoughts of harm.  Second, ask about normal recovery and function. This includes pain, mobility, eating, drinking, sleep, urination, bowel movement, perineal discomfort, cesarean wound symptoms, pelvic pressure, urinary leakage, and ability to perform daily care.  Third, ask about feeding and breast health. The professional should ask whether the baby can latch, how often the baby feeds, whether milk supply seems adequate, whether the mother has nipple pain, breast swelling, redness, fever, or localized breast tenderness. WHO recommends breastfeeding counseling and support at each postnatal contact.  Fourth, ask about mental health and family support. The professional should ask about mood, crying, anxiety, irritability, panic, sleep unrelated to baby care, bonding with the infant, partner or family support, domestic safety, and whether the mother has intrusive or frightening thoughts.  The examination should include vital signs, uterine height and tenderness, lochia, perineal or abdominal wound healing, breast assessment when indicated, leg swelling or tenderness, and overall signs of anemia, dehydration, infection, or exhaustion. The newborn should also be observed for feeding, jaundice, breathing, temperature, weight concerns, cord condition, and general activity, because maternal recovery and newborn wellbeing are closely connected. |
| **Flesch–Kincaid Grade Level** | 13.38 | 15.54 | 14.33 |
| **Corresponding English Reading-Difficulty Category** | College: difficult | College: difficult | College: difficult |

## Question 3

| Item | Attempt 1 | Attempt 2 | Attempt 3 |
| --- | --- | --- | --- |
| Clinical knowledge question | Which maternal symptoms or signs during the puerperium should be regarded as red flags requiring timely medical evaluation or referral? | | |
| Main guideline source(s) | NICE NG194; Chinese Maternal Health Management Specification | | |
| Model response | During the puerperium, the following symptoms should be treated as red flags and should prompt timely medical evaluation or referral.  First, abnormal bleeding is a major warning sign. This includes bleeding that suddenly increases, soaking pads quickly, passing large clots, feeling faint with bleeding, or bleeding accompanied by abdominal pain. It may suggest postpartum hemorrhage, retained products of conception, uterine atony, genital tract trauma, or infection. ACOG notes that heavy or gushing vaginal bleeding after birth needs urgent attention.  Second, fever or signs of infection should not be ignored. Fever of 38°C or higher, chills, foul-smelling lochia, increasing lower abdominal pain, uterine tenderness, breast redness with fever, or worsening wound pain may indicate endometritis, urinary tract infection, mastitis, wound infection, or sepsis. The CDC lists fever of 100.4°F, which is 38°C, or higher as an urgent maternal warning sign.  Third, neurological or hypertensive symptoms require urgent assessment. Severe headache, headache that does not improve, blurred vision, flashing lights, dizziness, fainting, seizure, confusion, or sudden swelling of the face and hands may indicate postpartum preeclampsia, eclampsia, stroke, or other serious disease. WHO postpartum danger signs include fits and severe headache with blurred vision.  Fourth, cardiopulmonary symptoms are red flags. Chest pain, shortness of breath, fast or difficult breathing, palpitations, fainting, coughing blood, or severe fatigue may suggest pulmonary embolism, cardiomyopathy, severe anemia, or hypertensive complications. WHO specifically lists fast or difficult breathing, calf pain with swelling, shortness of breath, and chest pain as postpartum danger signs.  Fifth, leg symptoms should be assessed promptly. One-sided calf pain, redness, warmth, swelling, or marked difference in leg size may suggest deep vein thrombosis.  Finally, mental health danger signs are equally urgent. Thoughts of self-harm, suicide, harming the baby, severe agitation, hallucinations, delusions, extreme confusion, or inability to sleep for a prolonged period with abnormal behavior may indicate severe postpartum depression or postpartum psychosis. These require immediate medical and psychiatric evaluation. | Red flags in the puerperium can be remembered by thinking of the main causes of severe postpartum morbidity and mortality.  The first group is bleeding and shock. Warning signs include heavy vaginal bleeding, bleeding that becomes heavier rather than lighter, passing large clots, dizziness, fainting, pale or clammy skin, rapid heartbeat, or severe weakness. These findings require urgent evaluation.  The second group is infection. Concerning symptoms include fever, chills, foul-smelling lochia, worsening pelvic or abdominal pain, uterine tenderness, painful urination, flank pain, breast redness with fever, pus from a wound, or a cesarean or perineal wound that becomes increasingly painful, red, swollen, or open.  The third group is hypertension and neurological complications. Severe headache, visual changes, upper abdominal pain, nausea or vomiting with high blood pressure, facial or hand swelling, seizure, confusion, weakness on one side of the body, slurred speech, or loss of consciousness are not normal postpartum symptoms. They may indicate preeclampsia, eclampsia, stroke, or intracranial disease. The CDC includes persistent or worsening headache, vision changes, dizziness or fainting, and extreme swelling of the hands or face among urgent maternal warning signs.  The fourth group is thromboembolism and heart disease. Chest pain, shortness of breath, rapid breathing, palpitations, coughing blood, sudden collapse, or one-sided painful leg swelling should prompt urgent referral. The postpartum period is a high-risk time for venous thromboembolism, and symptoms involving breathing, chest pain, or unilateral leg swelling should be taken seriously.  The fifth group is severe pain or functional deterioration. Severe abdominal pain that does not go away, worsening perineal pain, intense pelvic pressure, inability to pass urine, urinary retention with pain, severe constipation with abdominal distension, or inability to care for oneself or the baby should trigger assessment.  The sixth group is psychiatric emergency symptoms. Persistent thoughts of death, self-harm, harm to the infant, hallucinations, delusional beliefs, extreme paranoia, severe insomnia with agitation, or bizarre behavior require immediate referral. | In postpartum health education, I would tell the mother and family that they should seek medical care promptly if any of the following appear.  **Bleeding red flags**  Bleeding that is heavier than expected, suddenly increases, soaks through pads rapidly, contains large clots, or is associated with dizziness, fainting, abdominal pain, or rapid heartbeat.  **Infection red flags**  Fever of 38°C or above, chills, foul-smelling lochia, worsening lower abdominal pain, uterine tenderness, painful urination, flank pain, wound redness, wound discharge, wound opening, or breast redness and pain with fever.  **Blood pressure and brain red flags**  Severe or persistent headache, blurred vision, seeing spots, dizziness, fainting, seizure, confusion, slurred speech, weakness or numbness, severe swelling of the face or hands, or severe upper abdominal pain. These signs may suggest postpartum preeclampsia, eclampsia, stroke, or another acute neurological condition.  **Heart and lung red flags**  Chest pain, shortness of breath, difficulty breathing when lying flat, fast heartbeat, irregular heartbeat, coughing blood, sudden collapse, or extreme fatigue out of proportion to normal postpartum tiredness. CDC guidance advises immediate care for urgent warning signs during pregnancy or in the year after delivery.  **Leg red flags**  One leg becoming swollen, painful, warm, red, or clearly larger than the other. This may indicate deep vein thrombosis and should not be massaged or ignored.  **Mental health red flags**  Thoughts of suicide, self-harm, harming the baby, hearing voices, seeing things others do not see, believing things that are clearly unreal, severe agitation, severe confusion, or inability to sleep with abnormal behavior.  **General condition red flags**  The mother looks seriously unwell, cannot get out of bed because of weakness, cannot eat or drink, has persistent vomiting, has severe pain that does not improve, or family members feel that something is seriously wrong. In clinical practice, this last point is important because many severe postpartum problems are first noticed by relatives before the patient reaches care. |
| **Flesch–Kincaid Grade Level** | 12.30 | 13.21 | 14.55 |
| **Corresponding English Reading-Difficulty Category** | College: difficult | College: difficult | College: difficult |

## Question 4

| Item | Attempt 1 | Attempt 2 | Attempt 3 |
| --- | --- | --- | --- |
| Clinical knowledge question | How do the color, amount, and duration of normal lochia usually change, and which abnormal lochia findings require further assessment? | | |
| Main guideline source(s) | NICE NG194; Chinese Maternal Health Management Specification | | |
| Model response | **Normal lochia changes in a fairly predictable order.**  In the first 3 to 4 days, lochia is usually red or dark red. This is called lochia rubra. The amount is usually heaviest during this stage, similar to or slightly heavier than a menstrual period, and small clots may occur.  After that, it usually becomes pink, brown, or pale brown. This is called lochia serosa. The amount should gradually decrease, and the discharge becomes thinner and less bloody.  From about 10 to 14 days onward, it becomes yellowish white or creamy white. This is called lochia alba. The amount is usually light, and it may continue for several weeks. Many women have some discharge for up to 4 to 6 weeks after birth. Merck Manual describes the usual sequence as bloody lochia for 3 to 4 days, then pale brown discharge, then yellowish white discharge after the next 10 to 12 days.  Abnormal findings that need further assessment include very heavy bleeding, bleeding that suddenly increases after it had decreased, soaking pads quickly, passing large clots, persistent bright red bleeding beyond the early postpartum period, foul-smelling lochia, fever, chills, increasing lower abdominal pain, uterine tenderness, dizziness, fainting, or signs of shock. These may suggest postpartum hemorrhage, retained placental tissue, uterine infection, genital tract trauma, or coagulation problems. | Lochia is the normal vaginal discharge after delivery. It contains blood, decidual tissue, mucus, and wound exudate from the placental site. Its normal pattern is that the color becomes lighter, the amount becomes less, and the discharge gradually stops.  At first, lochia is bright red or dark red. This stage is mainly blood-stained discharge and is most obvious in the first few days. The flow should reduce day by day.  Then it changes to pinkish brown or brownish watery discharge. This usually occurs from around day 4 and reflects decreasing blood content. Cleveland Clinic describes lochia serosa as pinkish brown, thinner, more watery, and usually present around the fourth through twelfth day after childbirth.  Later, it becomes yellow-white or white discharge, with little or no blood. This final stage may last the longest, but the amount should be light.  Further assessment is needed when lochia does not follow this general trend. Red flags include lochia that becomes heavier instead of lighter, returns to heavy red bleeding after becoming pale, has a strong foul odor, is associated with fever or pelvic pain, contains repeated large clots, or continues as heavy bleeding for an unusually long time. New heavy bleeding 1 to 2 weeks postpartum can sometimes occur when tissue at the placental site separates, but heavy or prolonged bleeding should be evaluated because it may indicate infection or retained placenta. | A practical way to explain normal lochia to a postpartum woman is this: it should move from red, to brown or pink, to yellow-white, and the amount should gradually become less.  In the early days after birth, the discharge is red and may look like a heavy period. A few small clots can be normal. As the uterus contracts and the placental wound heals, the discharge becomes lighter in color and amount. By the second week, it should no longer be heavy bright red bleeding. After about two weeks, it is usually pale brown to yellowish white, and then slowly disappears. Merck’s postpartum changes table describes lochia as bloody in the first 3 to 4 days, pale brown from about 5 days to 2 weeks, and pale brown to yellowish white after 2 weeks.  The findings that should not be considered normal include the following.  Heavy bleeding that soaks a pad rapidly.  Large or frequent clots.  Bleeding that suddenly becomes bright red again and increases.  Foul-smelling discharge.  Fever, chills, uterine tenderness, or worsening abdominal pain.  Severe pelvic pain or increasing perineal pain.  Dizziness, fainting, palpitations, pallor, or marked weakness.  Lochia that remains heavy and bloody for too long.  These findings require further assessment of vital signs, uterine tone, uterine tenderness, wound condition, hemoglobin when indicated, and possible causes such as secondary postpartum hemorrhage, retained products of conception, endometritis, wound infection, or coagulation abnormality. |
| **Flesch–Kincaid Grade Level** | 9.54 | 9.34 | 9.02 |
| **Corresponding English Reading-Difficulty Category** | Grades 10–12: fairly difficult | Grades 10–12: fairly difficult | Grades 10–12: fairly difficult |

## Question 5

| Item | Attempt 1 | Attempt 2 | Attempt 3 |
| --- | --- | --- | --- |
| Clinical knowledge question | How should a sudden increase in vaginal bleeding after the first 24 hours postpartum and during the puerperium be clinically assessed? | | |
| Main guideline source(s) | NICE NG194 | | |
| Model response | A sudden increase in vaginal bleeding after the first 24 hours postpartum should be assessed as possible secondary postpartum hemorrhage until proven otherwise. Secondary postpartum hemorrhage usually refers to significant bleeding occurring after 24 hours and up to 6 weeks postpartum. Common causes include retained placental tissue, subinvolution of the placental site, infection, genital tract trauma, and coagulation problems.  The first step is to assess hemodynamic stability. Check blood pressure, pulse, respiratory rate, oxygen saturation, temperature, mental status, skin color, capillary refill, dizziness, syncope, and signs of shock. The amount of visible bleeding may underestimate actual blood loss, so maternal vital signs and symptoms are very important.  The second step is to ask targeted questions. Important points include when the bleeding increased, how many pads are soaked per hour, whether there are large clots, whether bleeding is continuous or intermittent, whether there is fever, chills, foul-smelling lochia, pelvic pain, uterine tenderness, dizziness, palpitations, or fainting. Also ask about mode of delivery, manual removal of placenta, retained placenta, uterine instrumentation, prolonged labor, infection during labor, cesarean birth, perineal trauma, anticoagulant use, and known bleeding disorders.  The examination should include abdominal palpation for uterine size, tone, and tenderness. A uterus that is larger than expected, soft, or tender may suggest subinvolution, retained products, or infection. A speculum examination may be needed to assess active bleeding from the cervix or vagina, retained tissue, laceration, hematoma, or wound breakdown. The perineum and cesarean incision should also be inspected.  Investigations depend on severity. In moderate or heavy bleeding, order a complete blood count, blood group and crossmatch, coagulation tests, fibrinogen when available, and inflammatory markers if infection is suspected. Pelvic ultrasound can help evaluate retained products of conception, intrauterine clots, or abnormal uterine findings, but treatment should not be delayed if the patient is unstable.  If the bleeding is heavy, recurrent, associated with shock, fever, severe pain, or abnormal vital signs, the patient should be referred urgently for hospital evaluation and management. | Clinically, the assessment should begin with one question: is the mother stable or unstable?  If she has heavy bleeding, pallor, cold sweating, tachycardia, hypotension, confusion, fainting, chest discomfort, shortness of breath, or severe weakness, this is an emergency. Immediate referral, intravenous access, fluid resuscitation, blood preparation, and obstetric review are needed. MSD Manual notes that postpartum hemorrhage diagnosis is clinical and treatment depends on the cause of hemorrhage.  If she is currently stable, the assessment should still be prompt. Sudden heavier bleeding after the first day is not simply normal lochia. The clinician should determine whether it is mild lochia fluctuation or true secondary hemorrhage.  Important history includes the amount of bleeding, pad count, clots, color, odor, pain, fever, breastfeeding status, recent increase in activity, and whether bleeding had already become lighter before becoming bright red again. Risk factors should be reviewed, including retained placenta, manual removal of placenta, placenta accreta spectrum, cesarean section, uterine infection, prolonged rupture of membranes, prolonged labor, multiple pregnancy, large baby, and previous postpartum hemorrhage.  Physical examination should focus on the following points.  The general condition and vital signs.  Uterine height, tone, and tenderness.  Lochia amount and odor.  Speculum examination for cervical or vaginal bleeding.  Assessment of perineal wounds, vaginal hematoma, and cesarean incision.  Signs of sepsis, anemia, or thromboembolism.  The likely causes can be considered using the same clinical logic as postpartum hemorrhage. Retained tissue and subinvolution are common causes of delayed bleeding. Infection is also important, especially if there is fever, uterine tenderness, foul-smelling lochia, or maternal malaise. Secondary PPH is commonly linked with retained placenta, subinvolution of placental sites, inherited coagulation problems, and infection. | A practical assessment can be organized into severity, source, and cause.  First, assess severity.  Ask how much bleeding there is. Soaking one pad quickly, continuous flowing blood, repeated large clots, dizziness, fainting, palpitations, or weakness suggests clinically significant blood loss. Check blood pressure, pulse, temperature, respiratory rate, oxygen saturation, and consciousness. If unstable, manage as an emergency.  Second, identify the source.  Do not assume all blood is coming from the uterine cavity. Examine the abdomen and pelvis. Assess uterine involution and tenderness. Inspect the perineum, vagina, cervix, and cesarean wound when appropriate. Look for laceration bleeding, hematoma, wound breakdown, retained tissue at the cervical os, or foul discharge.  Third, consider the cause.  The main causes after 24 hours include retained products of conception, infection or endometritis, subinvolution of the placental site, genital tract trauma, and coagulation abnormality. Secondary PPH is often defined as blood loss of more than 500 mL after 24 hours and up to 6 weeks postpartum, and many cases are related to uterine infection or retained products.  Further assessment may include complete blood count, coagulation profile, blood type and crossmatch, C-reactive protein or cultures if infection is suspected, and pelvic ultrasound. Ultrasound findings should be interpreted with the clinical picture, because postpartum clots and normal uterine contents may sometimes be difficult to distinguish from retained products.  Any sudden heavy bleeding, bleeding with large clots, bleeding with fever or foul-smelling lochia, bleeding with severe abdominal pain, or bleeding with dizziness or abnormal vital signs requires urgent medical evaluation. |
| **Flesch–Kincaid Grade Level** | 15.00 | 13.12 | 11.92 |
| **Corresponding English Reading-Difficulty Category** | College: difficult | College: difficult | Grades 10–12: fairly difficult |

## Question 6

| Item | Attempt 1 | Attempt 2 | Attempt 3 |
| --- | --- | --- | --- |
| Clinical knowledge question | What possible causes should be considered when a postpartum woman presents with heavy vaginal bleeding, blood clots, abdominal pain, fever, or foul-smelling lochia? | | |
| Main guideline source(s) | NICE NG194; Chinese Maternal Health Management Specification | | |
| Model response | When a postpartum woman presents with heavy vaginal bleeding, blood clots, abdominal pain, fever, or foul-smelling lochia, the clinician should first consider secondary postpartum hemorrhage with possible infection. These symptoms are not typical normal lochia and need timely assessment.  The main possible causes include the following.  Retained products of conception  Retained placental fragments or membranes can prevent normal uterine contraction and delay healing of the placental site. This may cause heavy bleeding, repeated clots, subinvolution of the uterus, abdominal pain, and sometimes infection.  Postpartum endometritis  This is infection of the uterine lining after delivery. Typical findings include fever, uterine tenderness, abdominal or pelvic pain, malaise, and sometimes abnormal or foul-smelling vaginal discharge. Merck Manual describes postpartum endometritis as a uterine infection with symptoms such as uterine tenderness, abdominal or pelvic pain, fever, malaise, and sometimes vaginal discharge.  Subinvolution of the placental site  The placental implantation site should gradually close and heal after delivery. If this process is delayed, bleeding may recur or become heavier after the first few days. This is an important cause of delayed postpartum bleeding.  Genital tract trauma or wound breakdown  Cervical, vaginal, perineal, or cesarean wound bleeding should be considered, especially if the uterus feels firm but bleeding continues. Hematoma, wound infection, or wound dehiscence may also cause pain and bleeding.  Coagulation abnormality  Inherited bleeding disorders, severe infection, massive bleeding, liver disease, thrombocytopenia, or anticoagulant use can contribute to persistent or heavy bleeding.  Sepsis or severe pelvic infection  Fever, chills, tachycardia, uterine tenderness, foul-smelling lochia, and maternal deterioration may indicate severe infection. StatPearls notes that findings suggestive of postpartum endomyometritis include fever, abdominal tenderness, and foul-smelling lochia. | This presentation should be approached as a possible delayed postpartum hemorrhage, especially if it occurs after the first 24 hours and during the puerperium.  The most important causes to consider are retained placental tissue, infection, and subinvolution of the placental site. A recent radiology review describes common causes of secondary postpartum hemorrhage as subinvolution of the placental site and retained or residual products of conception.  If the woman has heavy bleeding and clots, retained tissue is a key concern. The uterus may be larger than expected or poorly contracted. Ultrasound may show intrauterine material, although clinical judgment remains important.  If she has fever, lower abdominal pain, uterine tenderness, and foul-smelling lochia, postpartum endometritis is highly suspicious. Infection may occur after vaginal birth, but the risk is higher after cesarean delivery, prolonged labor, prolonged rupture of membranes, repeated vaginal examinations, or manual removal of the placenta.  If bleeding appears after it had already decreased, and the uterus is not involuting normally, subinvolution of the placental site should be considered. This may occur alone or with infection.  If bleeding is bright red and persistent while the uterus is firm, the clinician should look for cervical or vaginal laceration, perineal wound bleeding, hematoma, or cesarean incision problems.  Less common but important causes include uterine arteriovenous malformation, uterine artery pseudoaneurysm, placenta accreta spectrum remnants, coagulation disorders, gestational trophoblastic disease, and severe systemic infection. | In clinical practice, I would not explain these symptoms as normal postpartum recovery. I would think about five major diagnostic groups.  First, uterine cavity causes  Retained placental fragments, retained membranes, intrauterine clots, or abnormal placental adherence can cause heavy bleeding and repeated clots. These are common causes of secondary postpartum bleeding.  Second, uterine infection  Postpartum endometritis or endomyometritis should be suspected when bleeding is accompanied by fever, abdominal pain, uterine tenderness, foul-smelling lochia, chills, or maternal malaise. MSD Manual notes that uterine infections after delivery commonly cause lower abdominal or pelvic pain, fever, chills, rapid heart rate, uterine swelling and tenderness, and often foul-smelling vaginal discharge.  Third, poor healing of the placental site  Subinvolution means the placental bed does not close and heal as expected. It can cause delayed or recurrent bleeding, sometimes with a uterus that remains larger or softer than expected.  Fourth, trauma-related bleeding  Cervical tears, vaginal tears, perineal wound breakdown, episiotomy problems, vaginal hematoma, or cesarean scar complications can cause bleeding and pain. This is especially important if the uterus is firm but bleeding continues.  Fifth, systemic or rare causes  Coagulation problems, thrombocytopenia, anticoagulant medication, severe sepsis, uterine vascular lesions, and gestational trophoblastic disease should be considered when bleeding is severe, recurrent, unexplained, or not consistent with common causes.  Overall, heavy bleeding with clots plus fever, abdominal pain, or foul-smelling lochia requires prompt evaluation, because retained tissue and infection can worsen quickly and may lead to hemorrhage, anemia, sepsis, or the need for urgent uterine treatment. |
| **Flesch–Kincaid Grade Level** | 15.14 | 15.59 | 15.49 |
| **Corresponding English Reading-Difficulty Category** | College: difficult | College: difficult | College: difficult |

## Question 7

| Item | Attempt 1 | Attempt 2 | Attempt 3 |
| --- | --- | --- | --- |
| Clinical knowledge question | How should postpartum uterine involution be assessed, and what clinical problems may be suggested by poor uterine involution? | | |
| Main guideline source(s) | Chinese Maternal Health Management Specification; NICE NG194 | | |
| Model response | Postpartum uterine involution means that the uterus gradually returns from the enlarged pregnant state toward its non-pregnant size. It is assessed mainly by abdominal palpation of the uterine fundus, together with evaluation of lochia, pain, vital signs, and general condition.  During assessment, the mother is usually placed in a comfortable supine position. The bladder should be emptied first, because a full bladder can push the uterus upward or to one side and interfere with contraction. The clinician then palpates the fundus to assess its height, position, firmness, and tenderness.  Normally, shortly after birth the fundus is around the level of the umbilicus. After that, it should gradually descend day by day. The uterus should feel firm and centrally located. Lochia should become progressively lighter in amount and color. Merck Manual describes normal postpartum lochia as bloody for the first 3 to 4 days, then pale brown, and later yellowish white. This change supports normal uterine recovery.  Poor uterine involution may be suggested by a fundus that remains higher than expected, a uterus that feels enlarged or soft, uterine tenderness, persistent or increasing bleeding, large clots, foul-smelling lochia, fever, or lower abdominal pain.  Clinically, poor involution may suggest uterine atony, retained placental tissue, intrauterine clots, postpartum endometritis, subinvolution of the placental site, or delayed postpartum hemorrhage. Retained products of conception often present with heavy or irregular bleeding, enlarged or tender uterus, fever, pelvic pain, or infection. | Uterine involution should not be assessed by fundal height alone. It should be judged by combining fundal examination, lochia pattern, pain, fever, and bleeding trend.  The key points of examination are as follows.  First, check whether the uterus is firm or boggy. A firm uterus suggests good contraction. A soft or boggy uterus suggests poor contraction and increased risk of bleeding.  Second, check whether the fundus is at the expected level. The uterine fundus should gradually descend after delivery. If it remains high, becomes higher again, or is displaced to one side, the clinician should consider a full bladder, uterine atony, retained tissue, or abnormal intrauterine contents.  Third, check for uterine tenderness. Mild cramping can be normal, especially during breastfeeding, but significant tenderness with fever or foul lochia suggests infection.  Fourth, check lochia. Bleeding should gradually decrease. Return of bright red bleeding, sudden increase in bleeding, large clots, or foul odor is abnormal.  Poor uterine involution may indicate several problems. A soft uterus with heavy bleeding suggests uterine atony. A large uterus with persistent bleeding or clots suggests retained products of conception. Fever, uterine tenderness, abdominal pain, and foul-smelling lochia suggest postpartum endometritis. Delayed or recurrent bleeding after initial improvement may suggest subinvolution of the placental site or secondary postpartum hemorrhage. Retained products may present with continuous or irregular bleeding, lower abdominal or pelvic pain, and vaginal discharge. | A practical clinical assessment of postpartum uterine involution includes three questions.  Is the uterus shrinking as expected?  The fundus should be palpated regularly after birth. Its height should gradually fall over the postpartum days. If the uterus remains larger than expected, this is abnormal.  Is the uterus well contracted?  The uterus should feel firm. A soft, boggy uterus is concerning because it can bleed heavily. If the uterus is soft, the clinician should also check whether the bladder is distended, because bladder distension can prevent good uterine contraction.  Are the associated symptoms normal?  Lochia should reduce and change from red to brown or pink, and later to yellow-white. The mother should not have persistent fever, severe uterine tenderness, foul-smelling discharge, or increasing abdominal pain.  Poor uterine involution may suggest subinvolution, which means the uterus is not returning to its expected postpartum size. It may be associated with retained placental fragments, uterine infection, poor uterine contraction, or delayed healing of the placental site.  The main clinical risks are secondary postpartum hemorrhage, anemia, infection, and sepsis. If poor involution is accompanied by heavy bleeding, clots, fever, foul lochia, or abdominal pain, the woman needs timely evaluation. This may include pelvic examination, complete blood count, infection assessment, and ultrasound when retained products are suspected. Scottish NHS guidance notes that retained products commonly present with heavy or prolonged bleeding and pelvic pain, and infected retained products may include fever, offensive discharge, or uterine tenderness. |
| **Flesch–Kincaid Grade Level** | 12.72 | 11.03 | 11.11 |
| **Corresponding English Reading-Difficulty Category** | College: difficult | Grades 10–12: fairly difficult | Grades 10–12: fairly difficult |

## Question 8

| Item | Attempt 1 | Attempt 2 | Attempt 3 |
| --- | --- | --- | --- |
| Clinical knowledge question | What are the common clinical manifestations of puerperal infection, and how should fever, lower abdominal pain, and foul-smelling lochia be managed clinically? | | |
| Main guideline source(s) | NICE NG194; Chinese Maternal Health Management Specification | | |
| Model response | Puerperal infection refers to infection of the genital tract or related tissues after childbirth. It may involve the uterus, perineal wound, cesarean incision, urinary tract, breast, or pelvic tissues.  Common clinical manifestations include fever, chills, malaise, lower abdominal or pelvic pain, uterine tenderness, foul-smelling lochia, increased or abnormal vaginal discharge, delayed uterine involution, tachycardia, wound redness or discharge, breast pain and redness, and urinary symptoms. In postpartum uterine infection, symptoms commonly include lower abdominal or pelvic pain, fever, chills, general illness, rapid pulse, uterine tenderness, and often foul-smelling vaginal discharge.  When a postpartum woman has fever, lower abdominal pain, and foul-smelling lochia, postpartum endometritis should be strongly suspected. Clinically, the first step is to assess severity. Vital signs should be checked, including temperature, pulse, blood pressure, respiratory rate, and oxygen saturation. The clinician should also assess general appearance, mental status, hydration, abdominal tenderness, uterine size and tenderness, lochia amount and odor, and any perineal or cesarean wound findings.  Management should not be delayed if infection is suspected. The woman should be referred for medical evaluation, especially if fever is persistent or high, pain is worsening, lochia smells foul, or she appears unwell. Investigations may include complete blood count, inflammatory markers, urine testing, blood cultures if sepsis is suspected, wound culture if discharge is present, and pelvic ultrasound if retained placental tissue is suspected.  Treatment usually requires broad-spectrum antibiotics. For postpartum endometritis, MSD Manual states that diagnosis is mainly clinical and treatment is with broad-spectrum antibiotics, commonly intravenous therapy such as clindamycin plus gentamicin. | The common manifestations of puerperal infection can be grouped by site.  If the infection is in the uterus, the woman may have fever, lower abdominal pain, uterine tenderness, foul-smelling lochia, chills, fatigue, and delayed uterine involution.  If the infection is in a perineal wound or cesarean incision, there may be increasing wound pain, redness, swelling, warmth, pus, wound opening, or localized tenderness.  If it is a urinary tract infection, symptoms may include painful urination, urinary frequency, urgency, suprapubic pain, fever, or flank pain.  If it is mastitis, there may be breast pain, redness, localized swelling, fever, chills, and flu-like symptoms.  If infection progresses to sepsis, warning signs include high fever or low temperature, rapid pulse, rapid breathing, low blood pressure, confusion, severe weakness, cold extremities, or reduced urine output.  For fever with lower abdominal pain and foul-smelling lochia, the clinician should treat this as a possible postpartum uterine infection until proven otherwise. MSD Manual notes that postpartum uterine infections commonly cause lower abdominal pain, fever, and bad-smelling discharge, and that antibiotics are usually effective when started appropriately.  Clinical management should include prompt assessment of vital signs and uterine tenderness. The clinician should ask about onset of fever, maximum temperature, chills, lochia amount and smell, abdominal pain, wound symptoms, urinary symptoms, breast symptoms, delivery mode, prolonged rupture of membranes, prolonged labor, multiple vaginal examinations, manual removal of placenta, postpartum hemorrhage, and retained placental tissue.  If symptoms are mild and the woman is stable, she still needs timely clinical review. If she has high fever, severe pain, tachycardia, hypotension, altered consciousness, heavy bleeding, or signs of sepsis, she needs urgent referral and hospital management. | In the puerperium, infection should be suspected when normal postpartum recovery is replaced by systemic illness, worsening pain, or abnormal discharge.  Typical manifestations include fever, chills, feeling very unwell, loss of appetite, headache, rapid pulse, lower abdominal pain, uterine tenderness, foul-smelling lochia, abnormal bleeding, wound pain or discharge, breast redness and pain, painful urination, and delayed uterine involution. In endomyometritis, fever is often the first sign, and uterine tenderness and foul-smelling lochia are additional important findings.  When a woman presents with fever, lower abdominal pain, and foul-smelling lochia, the management should be active rather than observational.  First, assess whether she is stable. Check temperature, pulse, blood pressure, breathing, oxygen saturation, mental state, urine output, and signs of dehydration or shock.  Second, examine the likely sources of infection. Palpate the uterus for size, softness, and tenderness. Assess lochia for amount, color, and odor. Inspect the perineum, episiotomy site, laceration repair, and cesarean incision. Ask about breast symptoms and urinary symptoms.  Third, consider the main diagnoses. The most important are postpartum endometritis, retained products of conception with infection, wound infection, urinary tract infection, mastitis, pelvic abscess, and sepsis.  Fourth, arrange tests when needed. These may include blood count, C-reactive protein, blood cultures if febrile or septic, urine testing, wound swab if pus is present, and pelvic ultrasound if retained tissue or intrauterine collection is suspected.  Fifth, start or arrange appropriate treatment. Suspected postpartum endometritis usually needs broad-spectrum antibiotics. Severe illness, persistent fever, heavy bleeding, suspected retained tissue, or sepsis requires urgent hospital referral. A clinical guideline from Western Australia notes that severe postpartum endometritis is treated with intravenous antibiotics, continued until clinical signs such as fever, uterine tenderness, and purulent discharge have resolved for at least 24 to 48 hours. |
| **Flesch–Kincaid Grade Level** | 15.80 | 15.74 | 13.26 |
| **Corresponding English Reading-Difficulty Category** | College: difficult | College: difficult | College: difficult |

## Question 9

| Item | Attempt 1 | Attempt 2 | Attempt 3 |
| --- | --- | --- | --- |
| Clinical knowledge question | What should be included in the assessment of a perineal wound after vaginal delivery, and which findings suggest wound infection or dehiscence? | | |
| Main guideline source(s) | NICE NG194 | | |
| Model response | After vaginal delivery, assessment of a perineal wound should include both symptom inquiry and direct inspection.  The healthcare professional should ask about perineal pain, whether the pain is improving or worsening, difficulty sitting or walking, painful urination, bowel movement problems, constipation, wound bleeding, discharge, odor, fever, chills, and the mother’s ability to pass urine and control flatus or stool. If there was a third- or fourth-degree tear, she should also be asked about fecal urgency, fecal incontinence, flatus incontinence, and severe rectal pain.  On examination, the perineum should be inspected for wound approximation, redness, swelling, bruising, hematoma, pus, discharge, odor, bleeding, and whether sutures remain intact. The clinician should also check for tenderness, warmth, wound gap, tissue necrosis, and signs of abscess. If symptoms suggest deeper trauma or anal sphincter involvement, rectal examination may be needed.  Findings suggesting wound infection include increasing pain, redness, swelling, warmth, pus-like discharge, offensive odor, fever, wound tenderness, cellulitis around the wound, abscess formation, or the mother feeling generally unwell. RCOG describes infection signs after perineal tears as red or swollen skin around stitches, discharge or pus, increasing pain or tenderness, abnormal smell, and fever.  Findings suggesting wound dehiscence include separation of wound edges, gaping of the repair, loose or broken stitches, new bleeding from the wound, pus-like discharge, increasing pain, or visible opening of the wound. RCOG notes that infection or pressure from bleeding beneath the stitches can cause breakdown, leaving an open or gaping wound. | A perineal wound assessment after vaginal birth should begin with the mother’s main complaint. Pain is important. Mild soreness is common, but pain that becomes worse rather than better should raise concern. The clinician should ask whether the pain is localized, throbbing, associated with swelling, or severe enough to affect walking, sitting, urination, or sleep.  The next step is wound inspection. The examiner should look at the episiotomy or tear repair under good light. Important findings include the degree of swelling, bruising, wound edge alignment, suture integrity, skin color, discharge, bleeding, odor, and any open area. The surrounding vulva, vagina, anus, and perineal body should also be assessed. A tense, painful swelling may suggest hematoma.  Infection is suggested by increasing wound pain, red and swollen skin, local heat, tenderness, pus, foul-smelling discharge, fever, or systemic malaise. NHS guidance advises women to contact a midwife or GP if stitches become more painful, there is smelly discharge, or red swollen skin appears around the incision or tear, because these may indicate infection.  Dehiscence is suggested by an open wound, visible separation of the wound edges, loss of normal wound approximation, new wound bleeding, and sometimes discharge or pus. If the wound is infected, it is usually not re-sutured immediately, because closing an infected wound can trap infection inside. RCOG explains that if infection is present, the wound is generally not re-stitched until infection has been treated. | The assessment should cover five areas.  First, pain and function.  Ask about pain score, whether pain is improving, walking, sitting, urination, bowel movements, constipation, sexual concerns if relevant, and ability to care for the baby.  Second, systemic symptoms.  Ask about fever, chills, feeling unwell, fatigue out of proportion, dizziness, and poor appetite.  Third, wound appearance.  Inspect the wound for redness, edema, bruising, hematoma, wound edge separation, suture breakdown, discharge, pus, necrotic tissue, bleeding, and odor.  Fourth, deeper pelvic and anal symptoms.  Ask about rectal pain, fecal urgency, loss of control of stool or gas, and severe pressure. These symptoms are especially important after third- or fourth-degree tears. A clinical pearl on obstetric anal sphincter injury breakdown notes that women may present with pain, anal incontinence, fever, or malodorous discharge, and should be examined promptly, including rectal examination when needed.  Fifth, risk factors and wound care.  Review the type of tear, episiotomy, instrumental birth, prolonged second stage, large baby, postpartum hemorrhage, diabetes, obesity, anemia, hygiene difficulties, and whether antibiotics were used.  The findings most concerning for infection are increasing pain, redness, swelling, warmth, pus, foul smell, fever, wound tenderness, abscess, and general illness. The findings most concerning for dehiscence are gaping wound edges, visible opening of the repair, loose sutures, new bleeding, pus-like discharge, and sudden worsening pain. A European wound care document also lists increasing pain, edema, abscess formation, cellulitis, excessive or offensive discharge, feeling unwell, fever, and wound dehiscence as signs of perineal wound infection. |
| **Flesch–Kincaid Grade Level** | 15.11 | 10.72 | 12.10 |
| **Corresponding English Reading-Difficulty Category** | College: difficult | Grades 10–12: fairly difficult | College: difficult |

## Question 10

| Item | Attempt 1 | Attempt 2 | Attempt 3 |
| --- | --- | --- | --- |
| Clinical knowledge question | What conditions may be associated with persistent or worsening postpartum perineal pain, and how should it be assessed and managed? | | |
| Main guideline source(s) | NICE NG194 | | |
| Model response | Persistent or worsening postpartum perineal pain should not be dismissed as ordinary postpartum soreness. Mild pain after a tear or episiotomy is common, especially when sitting or walking, but the pain should gradually improve. RCOG notes that the skin part of a perineal wound usually heals within a few weeks, and tenderness should become much less over time.  Possible causes include perineal wound infection, wound dehiscence, hematoma, abscess, poorly healing episiotomy or laceration repair, obstetric anal sphincter injury, nerve pain, pelvic floor muscle spasm, and less commonly retained vaginal packing or foreign material. If pain is associated with fever, foul-smelling discharge, pus, or wound opening, infection or wound breakdown should be strongly suspected. RCOG explains that infection or bleeding under the stitches can cause perineal wound breakdown, leaving an open or gaping wound.  Assessment should include pain severity, onset, progression, location, effect on walking, sitting, urination, defecation, and sleep. The clinician should ask about fever, chills, wound discharge, bad odor, bleeding, constipation, urinary symptoms, rectal pain, fecal urgency, gas or stool incontinence, and whether the wound feels open.  Examination should include vital signs, inspection of the perineum and vagina under good light, wound edge approximation, redness, swelling, warmth, pus, odor, hematoma, abscess, necrotic tissue, and suture integrity. If there is severe rectal pain, anal incontinence, or a third- or fourth-degree tear, rectal examination is needed.  Management depends on the cause. Simple healing pain can be managed with perineal hygiene, cold packs in the early stage, sitz baths when appropriate, stool softeners, pelvic floor advice, and breastfeeding-compatible analgesia such as paracetamol or ibuprofen when suitable. NHS guidance notes that paracetamol can help perineal pain and is safe during breastfeeding, while ibuprofen is also generally considered safe for many breastfeeding women, though individual contraindications should be checked.  If infection is suspected, the woman needs clinical review, possible wound swab, antibiotics, and close follow-up. If there is abscess, hematoma, necrotic tissue, or wound breakdown, she may need drainage, debridement, specialist obstetric assessment, or delayed re-suturing after infection is controlled. | Persistent or worsening perineal pain after vaginal birth may be associated with several clinical problems.  The first is infected episiotomy or laceration repair. This is suggested by increasing pain, redness, swelling, local heat, pus, foul smell, fever, or maternal malaise. Exxcellence notes that early postpartum fever with worsening perineal pain suggests an infected episiotomy or laceration repair, and the wound may appear red, swollen, and may have purulent drainage.  The second is perineal wound dehiscence. The woman may report that the stitches have opened, the wound feels raw, or there is new discharge or bleeding. The wound may look gaping or separated.  The third is vulvovaginal or perineal hematoma. Pain is often severe, deep, throbbing, or pressure-like. It may be out of proportion to the visible wound. There may be swelling, bruising, urinary retention, or hemodynamic instability if bleeding is significant.  The fourth is obstetric anal sphincter injury or breakdown of a repaired third- or fourth-degree tear. This should be considered if there is severe perineal or rectal pain, fecal urgency, inability to control gas or stool, wound discharge, fever, or a feeling that the repair has failed. Severe lacerations involving the anal sphincter can lead to pelvic floor dysfunction and incontinence.  The fifth is non-infectious healing pain, including suture irritation, scar tenderness, pelvic floor spasm, constipation-related strain, hemorrhoids, or pudendal nerve irritation.  Assessment should be systematic. Ask when the pain started, whether it is improving or worsening, where it is located, whether it is sharp, burning, throbbing, or pressure-like, and what makes it worse. Ask about fever, discharge, odor, bleeding, urinary retention, dysuria, constipation, rectal pain, and incontinence.  Management should match the findings. Normal healing discomfort can be managed conservatively. Suspected infection requires antibiotics and wound care. Suspected abscess or expanding hematoma needs urgent obstetric assessment. Suspected anal sphincter repair breakdown needs prompt examination, often including rectal examination, and specialist management. | A useful clinical principle is that perineal pain should gradually improve after birth. Pain that persists without improvement, becomes worse, or is severe enough to prevent sitting, walking, urinating, or sleeping needs reassessment.  The main conditions to consider are:  Wound infection  This may cause increasing pain, redness, swelling, warmth, pus, offensive discharge, fever, or feeling unwell.  Wound dehiscence  This may cause a visible gap in the wound, loose stitches, raw tissue, bleeding, discharge, or sudden worsening pain. Infection is a common cause of perineal wound breakdown, and wound swabs and antibiotics may be needed.  Hematoma or abscess  This often causes severe localized pain or pressure. A hematoma may cause tense swelling and bruising. An abscess may cause fever, fluctuant swelling, and marked tenderness.  Missed or complicated anal sphincter injury  This should be suspected when pain is accompanied by rectal symptoms, fecal urgency, stool leakage, gas incontinence, or severe pain near the anus. Women with breakdown of obstetric anal sphincter repairs may present with pain, anal incontinence, fever, or malodorous discharge, and should be examined promptly, including rectal examination when needed.  Pelvic floor or nerve-related pain  Burning, shooting, or persistent deep pain may reflect nerve irritation, pelvic floor spasm, scar sensitivity, or musculoskeletal strain.  Clinical management should begin with vital signs and pain assessment, followed by direct inspection of the wound. The clinician should look for redness, edema, wound separation, discharge, pus, hematoma, abscess, necrotic tissue, and suture problems. If deeper injury is possible, vaginal and rectal examination may be required.  Treatment may include analgesia, hygiene advice, constipation prevention, pelvic floor guidance, antibiotics, wound swab, drainage of abscess, evacuation of significant hematoma, or referral to obstetrics, urogynecology, colorectal surgery, or pelvic floor physiotherapy depending on the suspected cause. Urgent referral is needed for severe pain, fever, foul discharge, wound gaping, expanding swelling, urinary retention, fecal incontinence, or signs of sepsis. |
| **Flesch–Kincaid Grade Level** | 15.15 | 11.38 | 13.93 |
| **Corresponding English Reading-Difficulty Category** | College: difficult | Grades 10–12: fairly difficult | College: difficult |

## Question 11

| Item | Attempt 1 | Attempt 2 | Attempt 3 |
| --- | --- | --- | --- |
| Clinical knowledge question | What are the basic principles of postpartum perineal wound care, and how should women be advised on perineal hygiene and pain management? | | |
| Main guideline source(s) | NICE NG194 | | |
| Model response | The basic principles of postpartum perineal wound care are to keep the wound clean, dry, protected from contamination, and comfortable, while watching for infection or wound separation.  Women should be advised to wash the perineal area gently with clean water every day and after passing urine or stool if needed. They should wipe or rinse from front to back, then pat dry rather than rub. Sanitary pads should be changed regularly, and tampons should not be used during lochia because they may increase infection risk. NHS guidance also advises keeping the area clean and changing maternity pads regularly after episiotomy or perineal tears.  Pain management should be stepwise. In the first 24 hours, cold packs can reduce swelling and pain. ACOG advises applying an ice or cold gel pack to the perineal area for 10 to 20 minutes at a time for postpartum perineal discomfort. After the early swelling improves, warm sitz baths or gentle warm water rinsing may help soreness and hygiene. Women can also sit on a soft cushion and avoid prolonged pressure on the wound.  Analgesics such as paracetamol or ibuprofen may be used if there are no contraindications and if they are appropriate during breastfeeding. Constipation should be prevented with adequate fluids, fiber, early mobilization, and stool softeners when needed, because straining can worsen pain and stress the repair.  Women should seek medical review if pain is worsening, the wound becomes red or swollen, there is pus or foul-smelling discharge, the stitches open, fever occurs, or there is difficulty controlling stool or gas. | Perineal wound care after vaginal birth should be based on gentle hygiene, pain relief, bowel protection, and early recognition of complications.  For hygiene, the woman should keep the area clean using water rather than harsh soaps or antiseptic solutions unless prescribed. After urination, pouring body-temperature water over the perineum can reduce stinging. RCOG notes that passing urine can sting after perineal tears, and pouring body-temperature water over the area during urination can help. After washing, the area should be dried gently. Pads should be changed often, and hands should be washed before and after pad changes.  For pain, it is normal to have soreness for about 2 to 3 weeks, especially when sitting or walking, but the pain should gradually improve. RCOG states that pain or soreness for 2 to 3 weeks after a tear is common, and the skin part of the wound usually heals within a few weeks. Cold compresses, suitable oral analgesics, comfortable positioning, and avoiding prolonged standing or sitting can help.  For bowel care, constipation should be avoided. Women should drink enough fluids, eat fiber-rich foods, and use stool softeners when recommended, especially after a third- or fourth-degree tear. They should not delay bowel movements because hard stool and straining increase pain.  For wound protection, the woman should avoid rubbing, tight clothing, and excessive pressure. Gentle walking is usually helpful, but overexertion should be avoided. Pelvic floor exercises can be started when comfortable, unless her clinician gives different instructions.  Medical review is needed for fever, increasing pain, redness, swelling, wound opening, pus, bad smell, heavy bleeding, severe rectal pain, or loss of control of wind or stool. | Women can be taught perineal care in simple practical terms.  First, keep it clean. Wash the perineum daily and after bowel movements. Use clean water, rinse from front to back, and avoid strong disinfectants unless a clinician prescribes them.  Second, keep it dry. After washing, pat the area dry. Change maternity pads frequently. NHS Lanarkshire advises keeping the perineal area clean and dry by washing daily, patting dry, and changing sanitary pads regularly. Some women may benefit from short periods of exposing the stitches to air while lying on a clean towel, as NHS guidance notes that fresh air can help healing.  Third, control pain early. Ice packs in the first day, warm water rinses, sitz baths, soft cushions, and regular simple pain relief can all help. Pain should become better over time. Pain that lasts longer than expected or becomes worse needs review.  Fourth, make urination and bowel movements easier. Warm water over the vulva while passing urine can reduce stinging. Drinking water, eating fiber, and using stool softeners when advised can reduce straining.  Fifth, observe the wound. The woman should be told to look for red, swollen, hot, increasingly painful skin, pus, bad smell, wound gaping, fever, or feeling generally unwell. These findings suggest infection or dehiscence and require clinical assessment.  In short, normal care is gentle cleaning, dryness, pad changing, pain relief, constipation prevention, and follow-up when symptoms do not improve. |
| **Flesch–Kincaid Grade Level** | 11.13 | 10.55 | 7.67 |
| **Corresponding English Reading-Difficulty Category** | Grades 10–12: fairly difficult | Grades 10–12: fairly difficult | Grades 8–9: plain English |

## Question 12

| Item | Attempt 1 | Attempt 2 | Attempt 3 |
| --- | --- | --- | --- |
| Clinical knowledge question | What should be observed in an abdominal incision after cesarean delivery, and which findings suggest wound infection, hematoma, or dehiscence? | | |
| Main guideline source(s) | NICE NG194; Chinese Maternal Health Management Specification | | |
| Model response | After cesarean delivery, the abdominal incision should be observed for healing, infection, bleeding, fluid collection, and wound separation.  The assessment should include the mother’s symptoms first. Ask about incision pain, whether the pain is improving or worsening, fever, chills, wound discharge, bad odor, bleeding, swelling, difficulty walking, and whether the wound feels open or pulling apart.  On inspection, observe the following points.  The skin color around the incision.  Local redness, warmth, swelling, or hardening.  The degree of tenderness.  Whether the wound edges are well approximated.  Whether there is bleeding, pus, serous fluid, or foul-smelling discharge.  Whether there is bruising, a lump, fluctuance, or tense swelling.  Whether the dressing is dry or soaked.  Whether the mother has fever or appears unwell.  Findings suggesting wound infection include increasing redness, warmth, swelling, worsening pain, pus, foul-smelling discharge, fever, or feeling generally unwell. NHS guidance lists redness, swelling, increasing pain, and wound discharge as signs of cesarean wound infection.  Findings suggesting hematoma include bruising, firm or tense swelling near the incision, increasing localized pain or pressure, wound bleeding, and sometimes a drop in hemoglobin or signs of blood loss. A hematoma is a collection of blood and may be visible as swelling or may extend deeper than what can be seen externally.  Findings suggesting dehiscence include separation of wound edges, gaping of the incision, visible deeper tissue, persistent drainage from the wound, broken sutures or staples, and sudden increase in pain. If the wound edge has separated, the incision should be assessed for the size of the defect and whether the deeper fascial layer remains intact. | The cesarean incision should be checked as part of every early postpartum assessment. The clinician should not only look at the skin surface, but also assess the woman’s pain, temperature, general condition, and risk factors such as obesity, diabetes, anemia, prolonged labor, prolonged rupture of membranes, emergency cesarean, or postpartum hemorrhage.  A normal healing incision may have mild tenderness, slight bruising, and a small amount of clear or blood-stained fluid early on. The pain should gradually lessen, and the skin edges should stay closed.  The concerning findings are different depending on the problem.  Wound infection is suggested by redness spreading around the incision, warmth, swelling, worsening pain despite pain relief, pus, blood-stained or foul-smelling drainage, fever, or systemic malaise. Cambridge University Hospitals advises women to monitor for redness, heat, increased pain, pus or blood leakage, offensive smell, fever, or feeling unwell.  Hematoma is suggested by increasing bruising, a firm painful lump, tense swelling, asymmetry around the wound, persistent bleeding, or pain out of proportion to the skin findings. If the hematoma is expanding, the woman may develop tachycardia, dizziness, pallor, hypotension, or anemia.  Dehiscence is suggested by wound gaping, skin edges no longer meeting, sudden drainage of fluid or blood, visible tissue in the wound, or a feeling that the wound has opened. This requires prompt assessment, especially to determine whether only the skin and subcutaneous tissue have opened or whether the fascial layer is involved.  Management depends on severity. Mild superficial redness may need review and oral antibiotics if infection is confirmed. Pus, fever, spreading cellulitis, abscess, expanding hematoma, or wound separation needs urgent obstetric or surgical assessment. | A practical cesarean wound assessment should answer four questions.  Is the wound closed?  The edges should be approximated. Any gaping, opening, broken skin closure, or visible deeper tissue suggests wound dehiscence and needs assessment.  Is there infection?  Look for increasing redness, swelling, warmth, tenderness, pus, foul smell, fever, or the mother feeling unwell. NHS recovery guidance advises contacting a midwife or GP if the cesarean wound becomes more red, painful, swollen, or has pus or foul-smelling fluid.  Is there a collection under the wound?  A hematoma or seroma may appear as a lump, swelling, bruising, fluid leakage, pressure pain, or asymmetry. Hematomas contain blood, while seromas contain serum. A cesarean wound review notes that small seromas or hematomas may be observed, but larger or infected collections may need opening, drainage, and further care.  Is the woman systemically well?  Check temperature, pulse, blood pressure, pain severity, dizziness, appetite, mobility, and signs of sepsis or anemia.  The incision should be referred for timely medical evaluation if there is fever, spreading redness, worsening pain, pus, foul odor, wound opening, heavy bleeding, expanding swelling, severe tenderness, or any sign that the mother is becoming systemically unwell. |
| **Flesch–Kincaid Grade Level** | 10.19 | 13.11 | 10.70 |
| **Corresponding English Reading-Difficulty Category** | Grades 10–12: fairly difficult | College: difficult | Grades 10–12: fairly difficult |

## Question 13

| Item | Attempt 1 | Attempt 2 | Attempt 3 |
| --- | --- | --- | --- |
| Clinical knowledge question | What types of pain are common during the puerperium, and what should be considered when providing analgesia to breastfeeding women? | | |
| Main guideline source(s) | NICE NG194 | | |
| Model response | Common pain during the puerperium includes uterine cramping, perineal pain, cesarean incision pain, breast pain, back or pelvic girdle pain, headache, and pain from hemorrhoids or constipation.  Uterine cramping is often called afterpain. It is more obvious during breastfeeding because oxytocin stimulates uterine contraction. Perineal pain is common after episiotomy, laceration, instrumental delivery, or swelling. Cesarean pain is related to the abdominal incision and deeper tissue healing. Breast pain may occur with engorgement, nipple trauma, blocked ducts, or mastitis. Back pain and pelvic pain may be related to pregnancy posture, labor position, neuraxial anesthesia soreness, pelvic floor strain, or musculoskeletal changes.  For breastfeeding women, analgesia should be effective but safe for the infant. ACOG recommends a stepwise multimodal approach, and states that acetaminophen and ibuprofen are first-line analgesics for postpartum pain in people who plan to provide breast milk.  Ibuprofen is often preferred when inflammation is present, such as perineal swelling or cesarean wound pain. LactMed describes ibuprofen as a preferred analgesic or anti-inflammatory drug in nursing mothers because milk levels are extremely low, it has a short half-life, and it is safely used in infants at much higher doses than those found in breast milk. Acetaminophen is also a good choice for pain and fever in breastfeeding women, with much lower milk exposure than infant therapeutic doses and rare reported adverse effects.  Opioids should be used cautiously. They may be needed for severe pain, especially after cesarean delivery or major perineal trauma, but the dose and duration should be minimized. The mother should be advised to watch the baby for excessive sleepiness, poor feeding, limpness, or breathing difficulty. Codeine and tramadol are generally avoided during breastfeeding because of safety concerns. | During the puerperium, pain may come from several sources.  The first is physiological uterine contraction pain. It is usually intermittent, cramp-like, and often stronger during breastfeeding.  The second is birth trauma pain. This includes perineal tears, episiotomy wounds, vulvar swelling, vaginal bruising, pelvic floor muscle pain, and hemorrhoids.  The third is surgical pain. After cesarean delivery, women may have incisional pain, abdominal wall pain, gas pain, and discomfort with coughing, walking, or changing position.  The fourth is breastfeeding-related pain. Engorgement, cracked nipples, blocked ducts, and mastitis may cause breast pain. Fever with breast redness or systemic symptoms should not be treated as simple engorgement.  The fifth is warning-sign pain. Severe headache, chest pain, severe abdominal pain, worsening wound pain, one-sided leg pain, or pain with fever needs medical evaluation rather than simple analgesia.  For breastfeeding women, pain treatment should consider maternal condition, infant age, prematurity, liver or kidney disease, drug allergy, bleeding risk, gastric ulcer history, asthma sensitive to NSAIDs, and whether the mother is taking other sedating medicines.  For most women, acetaminophen and ibuprofen are appropriate first choices. ACOG patient guidance says ibuprofen is usually the preferred first step because little passes into breast milk, and acetaminophen is also safe during breastfeeding. NHS guidance after cesarean delivery similarly advises that for most breastfeeding women, paracetamol or ibuprofen is preferred, but aspirin should be avoided.  If stronger analgesia is needed, it should be prescribed carefully, usually for a short time. The clinician should avoid unnecessary opioid exposure, advise against alcohol or other sedatives, and ensure the mother has enough pain control to walk, sleep, breastfeed, and care for the newborn. | Postpartum pain should be assessed by site, severity, timing, cause, and effect on function. Not all pain is normal.  Common types include cramping lower abdominal pain from uterine involution, perineal pain after vaginal birth, cesarean wound pain, breast and nipple pain, back pain, pelvic girdle pain, hemorrhoid pain, constipation-related pain, and headache. Mild to moderate pain that gradually improves is common. Pain that worsens, is severe, or is associated with fever, heavy bleeding, foul discharge, neurological symptoms, chest symptoms, or wound opening needs assessment.  Analgesia in breastfeeding women should follow several principles.  First, use non-drug measures when helpful. These include ice packs for early perineal swelling, warm sitz baths later, good positioning during breastfeeding, abdominal support after cesarean delivery, stool softeners for constipation, and breast support for engorgement.  Second, use regular simple analgesics before pain becomes severe. A hospital postnatal pain guide notes that taking analgesia after an operation or procedure such as cesarean birth or perineal stitches can help recovery, and taking it on a schedule may be better than waiting until pain is severe.  Third, choose medicines compatible with breastfeeding. Acetaminophen and ibuprofen are widely recommended first-line options. LactMed states acetaminophen is a good choice for analgesia and fever reduction in nursing mothers. LactMed also describes ibuprofen as a preferred choice during lactation because breast milk levels are very low.  Fourth, be careful with opioids. They should be reserved for pain not controlled by first-line medicines, used at the lowest effective dose for the shortest time, and paired with infant monitoring for sedation or feeding problems.  Finally, pain control should never replace diagnosis. Increasing perineal pain may mean infection, hematoma, or wound breakdown. Increasing abdominal pain with fever may mean endometritis. Severe headache may mean postpartum hypertension or preeclampsia. Chest pain or shortness of breath needs urgent evaluation. |
| **Flesch–Kincaid Grade Level** | 13.15 | 11.97 | 11.60 |
| **Corresponding English Reading-Difficulty Category** | College: difficult | Grades 10–12: fairly difficult | Grades 10–12: fairly difficult |

## Question 14

| Item | Attempt 1 | Attempt 2 | Attempt 3 |
| --- | --- | --- | --- |
| Clinical knowledge question | How can physiological breast engorgement be distinguished from pathological breast conditions after delivery, and how should healthcare professionals perform an initial assessment? | | |
| Main guideline source(s) | NICE NG194 | | |
| Model response | Physiological breast engorgement is usually a bilateral, diffuse fullness of the breasts that appears when milk secretion increases, most often around day 3 to day 5 postpartum. The breasts may feel tense, warm, heavy, and tender. The nipples and areola may become swollen, making latch difficult. However, the mother is usually systemically well, and symptoms should improve with effective milk removal and supportive care. ACOG describes breast engorgement as physiologic bilateral breast fullness that most often occurs between day 3 and day 5 postpartum.  Pathological breast conditions are more likely when the symptoms are localized, progressive, severe, or associated with systemic illness. For example, a localized painful lump may suggest ductal narrowing or a milk retention area. A red, hot, painful area with fever, chills, malaise, and myalgia suggests mastitis. A fluctuant, very tender mass or persistent fever despite treatment may suggest breast abscess. Nipple fissures, bleeding, burning pain, or poor latch may suggest nipple trauma or infection.  The initial assessment should include a focused history. Ask when breast fullness began, whether one or both breasts are affected, whether the pain is diffuse or localized, whether there is fever or flu-like illness, whether there is redness, whether feeding relieves symptoms, how often the baby feeds, whether milk transfer seems effective, whether the baby has enough wet diapers, whether there is nipple trauma, and whether the mother has skipped feeds or pumped excessively.  Physical examination should include temperature, pulse, general appearance, both breasts, nipples, areolae, and axillae. Look for diffuse swelling, localized erythema, warmth, tenderness, hard lumps, fluctuance, nipple cracks, bleeding, discharge, and lymph node tenderness. ACOG emphasizes that persistent breastfeeding pain requires a focused history and physical examination. | A simple distinction is this: physiological engorgement is usually generalized and temporary, while pathological breast problems are often focal, worsening, or accompanied by fever and maternal illness.  Physiological engorgement usually occurs during the early transition to mature milk. It often affects both breasts. The breasts feel full, tight, warm, and uncomfortable. The mother may have mild temperature elevation, but she should not appear acutely ill. With frequent effective breastfeeding, gentle milk expression for comfort, proper latch, and supportive measures, symptoms should gradually improve. ACOG advises that milk expression for engorgement should be minimal and mainly for symptom relief, because excessive emptying may worsen oversupply.  Pathological conditions should be considered in several situations. If there is a localized wedge-shaped red and tender area with fever and flu-like symptoms, mastitis is likely. If there is a persistent painful mass, especially with fever or no improvement, abscess should be considered. If there is a painful lump without systemic illness, it may be ductal narrowing or inflammatory milk stasis. If the main symptom is nipple pain, the clinician should check for shallow latch, nipple fissure, vasospasm, dermatitis, or infection.  Initial assessment should not begin with antibiotics automatically. It should begin with the mother and baby as a feeding pair. Observe a feed if possible. Assess latch, positioning, milk transfer, feeding frequency, nipple shape after feeding, infant output, and whether the mother is using pumps, nipple shields, or supplements. Then examine the breast carefully for diffuse versus focal findings.  The Academy of Breastfeeding Medicine describes lactation-related breast inflammation as a spectrum that can include engorgement, ductal narrowing, inflammatory mastitis, bacterial mastitis, phlegmon, abscess, and galactocele. This means clinicians should judge severity and progression rather than treating every painful breast as the same condition. | In early postpartum care, physiological engorgement can be identified by its pattern. It is usually bilateral, appears around the time milk “comes in,” and causes a sense of fullness, tightness, warmth, and tenderness. It may make the areola firm and the baby’s latch more difficult. It should improve with appropriate breastfeeding support.  Pathological breast conditions should be suspected when any of the following are present.  One breast is much more affected than the other.  There is a clearly localized painful lump.  There is spreading redness or marked heat.  The mother has fever, chills, body aches, or feels acutely unwell.  Pain is worsening instead of improving.  There is pus or abnormal nipple discharge.  There is a fluctuant mass.  Symptoms persist despite appropriate feeding support.  The ABM mastitis protocol notes that persistent systemic symptoms such as fever and tachycardia for more than 24 hours, or lack of response to conservative measures, should raise concern for bacterial mastitis or more severe disease.  The initial assessment should include four parts.  First, assess maternal condition. Check temperature, pulse, blood pressure if indicated, pain severity, hydration, and whether she looks systemically unwell.  Second, assess breast findings. Look and palpate for bilateral engorgement, focal induration, redness, warmth, tenderness, fluctuance, nipple fissures, bleeding, dermatitis, and axillary tenderness.  Third, assess feeding. Ask about feeding frequency, missed feeds, long intervals, latch pain, infant sleepiness, milk transfer, wet diapers, supplementation, and pumping habits.  Fourth, decide whether urgent review is needed. Urgent assessment is needed for high fever, severe pain, rapidly spreading redness, suspected abscess, immunocompromise, systemic illness, or a newborn who is not feeding well. Conservative support is reasonable for typical physiological engorgement, but persistent or worsening symptoms need reassessment. |
| **Flesch–Kincaid Grade Level** | 12.73 | 12.97 | 10.28 |
| **Corresponding English Reading-Difficulty Category** | College: difficult | College: difficult | Grades 10–12: fairly difficult |

## Question 15

| Item | Attempt 1 | Attempt 2 | Attempt 3 |
| --- | --- | --- | --- |
| Clinical knowledge question | What are the typical manifestations of acute mastitis, and how should breast redness, swelling, heat, pain, fever, or systemic symptoms be managed? | | |
| Main guideline source(s) | NICE NG194 | | |
| Model response | Acute mastitis usually presents as a painful inflammatory condition of the breast during lactation. It most often affects one breast. Typical manifestations include localized breast redness, swelling, warmth, tenderness, induration, and pain. The woman may also have fever, chills, fatigue, headache, muscle aches, and a flu-like feeling. Merck Manual describes mastitis symptoms as high fever with breast erythema, induration, tenderness, pain, swelling, and warmth to touch.  When a postpartum woman has breast redness, swelling, heat, pain, fever, or systemic symptoms, the clinician should first assess severity. Check temperature, pulse, blood pressure if indicated, hydration, general appearance, and whether she looks acutely unwell. Examine both breasts and nipples. Look for whether the finding is diffuse or localized, whether there is a painful lump, spreading redness, nipple fissure, purulent discharge, fluctuance, or axillary tenderness.  Management should include continued milk removal, but without aggressive massage or excessive pumping. The mother should be supported to continue breastfeeding or express milk if direct feeding is too painful. She should rest, drink fluids, optimize latch and positioning, and use analgesia such as ibuprofen or acetaminophen when suitable. ACOG recommends a focused history and physical examination for breastfeeding pain and problems, and emphasizes evaluation and management rather than early weaning.  If symptoms are mild and have been present for a short time, conservative care with feeding support, anti-inflammatory pain relief, cold compresses, rest, and close review may be reasonable. If fever, tachycardia, significant systemic symptoms, persistent symptoms, or progressive breast redness are present, bacterial mastitis should be considered and antibiotics may be needed. If there is a persistent mass, fluctuance, or failure to improve, breast abscess should be assessed, usually with ultrasound and drainage when confirmed. | Typical acute mastitis has both local breast symptoms and sometimes whole-body symptoms.  Local symptoms include a red, swollen, hot, painful area of the breast, breast hardness, tenderness, and sometimes a wedge-shaped area of inflammation. The nipple may be cracked or damaged, especially if latch has been poor. Systemic symptoms include fever, chills, body aches, fatigue, headache, and feeling as if the mother has influenza. NHS describes mastitis as a breast becoming swollen, hot, and painful, most commonly in breastfeeding women.  Management should begin with assessment of the mother and the feeding situation. Ask about onset, affected side, fever, chills, pain severity, feeding frequency, missed feeds, milk oversupply, pumping habits, nipple trauma, previous mastitis, and whether the baby is feeding effectively. Observe breastfeeding if possible, because poor latch, infrequent feeding, oversupply, or abrupt weaning can worsen milk stasis and inflammation.  For initial management, advise the woman not to stop breastfeeding abruptly. Continuing breastfeeding is usually safe for the baby and helps avoid worsening milk stasis. She may feed from the affected breast if tolerable, or express gently if feeding is too painful. She should avoid deep, forceful massage because it can worsen tissue inflammation. Supportive measures include rest, fluids, a well-fitting non-compressive bra, cold packs after feeding, and anti-inflammatory analgesia when not contraindicated.  If she has persistent fever, worsening redness, severe pain, systemic illness, immunocompromise, or no improvement with conservative care, she needs clinical review for antibiotics. The Academy of Breastfeeding Medicine describes mastitis as part of a spectrum that includes inflammatory mastitis, bacterial mastitis, phlegmon, abscess, and galactocele, so persistent or worsening symptoms require reassessment rather than repeated self-treatment. | Acute mastitis should be suspected when a breastfeeding woman develops sudden breast pain with redness, swelling, warmth, or hardness, especially if she also has fever, chills, muscle aches, or marked fatigue. It is often unilateral. The affected breast may be very tender, and feeding may become painful. Cleveland Clinic describes mastitis as a breast infection that can make the breast red, hard, and swollen, and may also cause fever or flu-like symptoms.  Clinically, breast redness, swelling, heat, pain, fever, or systemic symptoms should be managed in steps.  First, assess for emergency features. Urgent medical evaluation is needed if the mother appears septic, has very high fever, rapid heart rate, low blood pressure, confusion, severe weakness, rapidly spreading redness, severe pain, or inability to care for herself or the baby.  Second, examine for abscess. A fluctuant mass, persistent localized swelling, severe focal tenderness, or fever that does not improve with appropriate therapy should raise suspicion. Mastitis that is untreated or related to milk stasis can progress to abscess, which often needs drainage.  Third, support lactation. Correct latch, avoid long intervals between feeds, avoid abrupt weaning, and use gentle expression only as needed for comfort. The goal is effective milk flow without overstimulating supply.  Fourth, treat pain and inflammation. Ibuprofen or acetaminophen may be used if suitable. Cold packs can reduce swelling and pain. Warmth may be used briefly before feeding if it helps milk let-down, but prolonged heat is not necessary.  Fifth, use antibiotics when bacterial mastitis is likely. This is more likely when fever and systemic symptoms persist, the breast findings worsen, or symptoms fail to improve with conservative care. Choice of antibiotic should follow local guidance and take account of allergy history and local resistance patterns. Breastfeeding can usually continue during treatment, using antibiotics compatible with lactation. |
| **Flesch–Kincaid Grade Level** | 13.05 | 13.66 | 10.43 |
| **Corresponding English Reading-Difficulty Category** | College: difficult | College: difficult | Grades 10–12: fairly difficult |

## Question 16

| Item | Attempt 1 | Attempt 2 | Attempt 3 |
| --- | --- | --- | --- |
| Clinical knowledge question | What are the common causes of nipple pain or cracking during breastfeeding, and how should breastfeeding position and latch be assessed? | | |
| Main guideline source(s) | NICE NG194 | | |
| Model response | The most common cause of nipple pain or cracking during breastfeeding is shallow latch or poor positioning. When the baby attaches only to the nipple instead of taking enough breast tissue into the mouth, the nipple is compressed, rubbed, or pinched. This can cause pain, flattening, bruising, bleeding, blisters, and cracks. NICE states that suboptimal infant positioning and attachment is the most common cause of nipple damage.  Other common causes include nipple trauma from frequent friction, poor removal from the breast, incorrect breast pump flange size, suction that is too strong, engorgement that makes latch shallow, infant tongue-tie, high palate, oral motor difficulty, nipple blebs, dermatitis or eczema, candidal or bacterial infection, vasospasm, and biting in older infants. ACOG notes that persistent nipple pain and nipple injury are common breastfeeding challenges and require focused history and physical examination.  Assessment should include direct observation of a full breastfeed whenever possible. The clinician should check whether the mother is comfortable and well supported, whether the baby’s body is close to the mother, whether the baby’s head and body are aligned, and whether the baby is brought to the breast rather than the mother leaning forward to the baby.  A good latch is suggested by a wide-open mouth, chin touching the breast, more areola visible above the baby’s upper lip than below the lower lip, flanged lips, rounded cheeks, slow rhythmic sucking with swallowing, and little or no nipple pain after the first brief latch-on discomfort. After feeding, the nipple should look round rather than flattened, creased, white, or pinched.  Warning findings include pain throughout the feed, clicking sounds, cheeks dimpling, frequent slipping off the breast, poor milk transfer, nipple compression after feeds, bleeding cracks, baby not gaining weight, or too few wet diapers. These findings should prompt breastfeeding support, latch correction, and assessment for infant oral restrictions or maternal breast and nipple disease. | Nipple pain and cracking usually occur because the nipple is being repeatedly damaged during feeding. The most frequent mechanism is compression and friction from an ineffective latch. This often happens when the baby is too far from the breast, the head is turned, the baby latches onto the nipple tip, or the mother’s breast is very full and the areola is too tense for a deep latch.  Other causes should also be considered. These include flat or inverted nipples, engorgement, nipple edema, poor positioning after cesarean pain, infant tongue-tie, prematurity or weak sucking, inappropriate nipple shield use, breast pump trauma, eczema, contact allergy, thrush, bacterial infection, vasospasm, and milk blebs. ABM Clinical Protocol 26 emphasizes that persistent nipple and breast pain can have multiple causes and should be evaluated systematically rather than treated as a single diagnosis.  To assess position and latch, the healthcare professional should observe the mother and baby before, during, and after feeding.  Before feeding, assess the mother’s posture, breast fullness, nipple shape, and whether she can hold the baby close without tension. Also assess the baby’s alertness, mouth opening, tongue movement, and rooting behavior.  During latch, check whether the baby’s nose is opposite the nipple before attachment, whether the baby opens widely, whether the chin reaches the breast first, and whether the baby takes a large mouthful of breast tissue. The baby should not be hanging from the nipple.  During milk transfer, listen and look for swallowing, sustained rhythmic sucking, relaxed hands, and softening of the breast. Pain should reduce after the initial latch and should not continue sharply through the feed.  After feeding, inspect the nipple. A normal nipple should not be newly cracked, bleeding, blanched, wedge-shaped, or flattened. If it is damaged, the clinician should correct latch, assess the baby’s oral anatomy, and treat the nipple wound while supporting continued breastfeeding if possible. | Common causes of nipple pain or cracking can be divided into maternal factors, infant factors, and feeding technique factors.  Maternal factors include engorgement, nipple edema, sensitive or inverted nipples, eczema, dermatitis, infection, vasospasm, milk blebs, previous nipple trauma, and pain after cesarean delivery that makes positioning difficult.  Infant factors include shallow suck, tongue-tie, high palate, jaw tightness, prematurity, sleepiness, poor coordination, strong suction with poor attachment, and biting.  Technique factors include shallow latch, poor body alignment, pulling the baby away from the breast while still latched, long feeds with ineffective suckling, inappropriate nipple shield use, incorrect pump flange size, and excessive pump suction. AAP material notes that assessment of breastfeeding technique and latch is the most important part of preventing and managing nipple pain and skin breakdown.  Assessment should answer five questions.  Is the baby positioned well?  The baby’s head, neck, and body should be aligned. The baby should face the breast, be held close, and not need to twist the neck.  Is the latch deep enough?  A deep latch includes more than the nipple. The baby’s mouth should be wide, lips turned outward, chin close to the breast, and the nipple should be directed toward the roof of the mouth.  Is milk transfer effective?  There should be rhythmic sucking and swallowing. The baby should seem satisfied after feeding, and the breast may feel softer.  Is the mother in pain?  Brief tenderness at latch-on can occur early, but sharp, persistent, burning, or worsening pain is abnormal.  What does the nipple look like after feeding?  Cracks, bleeding, blisters, white color change, lipstick-shaped flattening, or a crease across the nipple suggest ongoing trauma or vasospasm.  Management starts with correcting positioning and latch, because ointment alone will not solve repeated mechanical damage. If pain persists despite good latch, further assessment should consider tongue-tie, infection, dermatitis, vasospasm, pump trauma, and referral to a lactation specialist. |
| **Flesch–Kincaid Grade Level** | 13.73 | 11.49 | 9.95 |
| **Corresponding English Reading-Difficulty Category** | College: difficult | Grades 10–12: fairly difficult | Grades 10–12: fairly difficult |

## Question 17

| Item | Attempt 1 | Attempt 2 | Attempt 3 |
| --- | --- | --- | --- |
| Clinical knowledge question | What should be asked and observed when assessing whether breastfeeding is effective? | | |
| Main guideline source(s) | NICE NG194; Chinese Maternal Health Management Specification | | |
| Model response | When assessing whether breastfeeding is effective, healthcare professionals should ask about both the mother’s experience and the baby’s intake.  For the mother, ask:  Whether feeding is painful.  Whether the nipples are cracked, bleeding, flattened, or misshapen after feeds.  Whether the breasts feel softer after feeding.  Whether milk has come in.  Whether she hears or sees swallowing.  How often the baby feeds in 24 hours.  Whether feeds are very short, very long, or constantly ineffective.  Whether she has engorgement, blocked ducts, mastitis symptoms, or concern about milk supply.  For the baby, ask:  Whether the baby wakes for feeds or is too sleepy.  Whether the baby latches and stays attached.  Whether sucking becomes slow and rhythmic.  Whether swallowing is heard.  Whether the baby seems satisfied after feeds.  How many wet and dirty diapers the baby has.  Whether stool color is transitioning normally.  Whether there is jaundice, excessive weight loss, or poor weight gain.  During observation, the clinician should watch a full feed if possible. Good signs include the baby held close with head and body aligned, wide mouth, deep latch, chin touching the breast, rounded cheeks, rhythmic sucking, audible swallowing, and relaxation after feeding. UNICEF describes good positioning as the baby’s head and body being in line, the baby being held close, and the whole body being supported.  Poor effectiveness is suggested by persistent nipple pain, shallow latch, clicking sounds, dimpling cheeks, frequent slipping off the breast, no swallowing, very sleepy feeding, poor urine or stool output, jaundice, excessive weight loss, or poor weight gain. ABM discharge guidance says breastfeeding assessment should include positioning, latch, milk transfer, jaundice, stool and urine output, and infant weight. | Effective breastfeeding should be assessed by looking at latch, milk transfer, infant output, infant growth, and maternal comfort.  The first thing to ask is how the feeding is going from the mother’s point of view. Ask whether she has pain, anxiety about milk supply, breast fullness, nipple injury, or difficulty getting the baby to attach. Pain that continues throughout a feed is not normal and often suggests poor latch or nipple trauma.  The second part is infant behavior. Ask how often the baby feeds, whether the baby actively sucks and swallows, whether the baby becomes calm after feeding, and whether the baby wakes for feeds. A baby who is always sleepy, feeds only briefly, or stays on the breast for a long time without swallowing may not be transferring milk well.  The third part is infant output and weight. Ask about wet diapers, stools, stool color, jaundice, and weight change. These are important because a baby can appear to latch well but still transfer too little milk. ABM notes that some infants, especially late preterm infants, may appear to latch, suck, and swallow but still fail to transfer adequate milk when checked more carefully.  The feeding observation should include the mother’s posture, the baby’s body alignment, mouth opening, depth of latch, cheek shape, suck-swallow rhythm, and nipple appearance after feeding. NHS guidance describes good attachment as a wide mouth, chin touching the breast, no breast or nipple pain during feeding apart from strong early sucks, and more areola visible above the top lip than below the bottom lip.  If the assessment suggests ineffective feeding, management should include correcting positioning and latch, checking the baby’s oral anatomy, supporting milk expression if needed, monitoring weight and hydration, and arranging lactation follow-up. | A practical assessment can be organized into four questions.  Is the baby attached well?  Observe whether the baby’s mouth opens wide, the chin is close to or touching the breast, the lips are flanged, the cheeks stay rounded, and the baby does not make repeated clicking or smacking sounds. A shallow latch, nipple-only sucking, cheek dimpling, or slipping off the breast suggests ineffective attachment.  Is milk actually being transferred?  Look for a pattern of sucking that changes from rapid sucks to slower deep sucks with pauses and swallowing. The mother may feel a pulling sensation but should not have sharp pain. The breast may feel softer after feeding. ABM states that formal assessment can include positioning and attachment, with careful attention to audible swallowing as a sign of milk transfer, as well as newborn weight, jaundice, and elimination patterns.  Is the baby clinically well?  Ask about alertness, feeding cues, satisfaction after feeding, wet diapers, stools, jaundice, and weight. Too few wet diapers, persistent dark urine, delayed stool transition, increasing jaundice, lethargy, excessive weight loss, or poor weight gain suggests inadequate intake and needs timely review.  Is the mother comfortable and able to continue?  Ask about nipple pain, cracking, bleeding, engorgement, anxiety, fatigue, and confidence. Maternal pain and poor latch can reduce milk transfer and may lead to early breastfeeding cessation if not corrected.  Healthcare professionals should observe at least one feed when there is concern. They should avoid judging effectiveness by feeding duration alone. A long feed without swallowing is not necessarily effective, and a short feed may be adequate if the baby transfers milk well and is growing appropriately. |
| **Flesch–Kincaid Grade Level** | 8.11 | 11.07 | 9.61 |
| **Corresponding English Reading-Difficulty Category** | Grades 8–9: plain English | Grades 10–12: fairly difficult | Grades 10–12: fairly difficult |

## Question 18

| Item | Attempt 1 | Attempt 2 | Attempt 3 |
| --- | --- | --- | --- |
| Clinical knowledge question | How should clinicians evaluate medication safety and breastfeeding-related risks when a breastfeeding woman requires medication during the puerperium? | | |
| Main guideline source(s) | NICE NG194 | | |
| Model response | When a breastfeeding woman needs medication during the puerperium, clinicians should assess both maternal benefit and infant risk. The aim is not to stop breastfeeding automatically, but to choose the safest effective treatment.  The clinician should first clarify the medication need. Important questions include the diagnosis, urgency of treatment, available non-drug options, whether treatment can be delayed, and whether a safer alternative exists.  Then the medication itself should be assessed. Key points include dose, route, duration, half-life, active metabolites, protein binding, oral bioavailability in the infant, and whether the drug is known to suppress lactation. Drugs with low milk transfer, short half-life, poor infant oral absorption, and good lactation safety data are generally preferred.  The infant’s condition is equally important. A full-term healthy 2-month-old infant has a different risk profile from a premature newborn in the first week of life. Higher caution is needed when the baby is premature, low birth weight, medically unstable, jaundiced, has liver or kidney disease, or is exclusively breastfed.  Clinicians should use reliable lactation references rather than relying only on package inserts. LactMed provides data on drug levels in breast milk and infant blood, possible infant adverse effects, and suggested alternatives when appropriate.  When treatment is started, the mother should be advised what to watch for in the baby, such as excessive sleepiness, poor feeding, vomiting, diarrhea, rash, irritability, poor weight gain, or breathing difficulty. Follow-up should be arranged if the medicine is high risk, long term, or newly started in a vulnerable infant. | Medication safety in breastfeeding should be evaluated through a structured clinical process.  First, identify whether the medicine is necessary. Untreated maternal illness can also harm breastfeeding and maternal recovery. For example, uncontrolled pain may impair mobility, sleep, mood, and infant care. ACOG notes that acetaminophen and ibuprofen are first-line analgesics for postpartum pain in people who intend to provide breast milk.  Second, choose the lowest-risk effective medicine. Prefer drugs with established breastfeeding safety, short half-life, low transfer into milk, and no serious reported infant toxicity. Avoid unnecessary combinations, sedating drugs when possible, and long-acting formulations if a safer short-acting option is available.  Third, assess the infant. Important questions include gestational age at birth, current age, weight gain, feeding pattern, jaundice, prematurity, neonatal illness, and liver or kidney problems. Younger and premature infants have less ability to metabolize and clear drugs.  Fourth, plan timing and monitoring. For some short-acting medicines, taking the dose just after breastfeeding or before the infant’s longest sleep interval may reduce peak exposure, although this is not always necessary. The mother should be told to monitor feeding, alertness, breathing, stool pattern, rash, and weight gain.  Fifth, avoid high-risk drugs when safer options exist. Opioids require caution because they can cause infant sedation and respiratory depression. Codeine and tramadol are generally avoided during breastfeeding because metabolism can be unpredictable and infant exposure may be unsafe. ACOG advises counseling breastfeeding people prescribed opioids about the risk of central nervous system depression in both the mother and infant. | A practical approach is to ask four questions.  What does the mother need?  The clinician should confirm the indication, severity of illness, expected treatment duration, and whether the medication is essential. Maternal treatment should not be withheld without good reason, because untreated infection, hypertension, pain, depression, or other illness may be more harmful than carefully selected medication.  How much drug may reach the baby?  This depends on the drug’s milk transfer and the infant’s ability to absorb it. Clinicians should consider milk-to-plasma ratio, relative infant dose when available, half-life, active metabolites, and oral bioavailability. A commonly used reference point is that a relative infant dose below about 10% is often considered low concern, but this is not absolute and does not replace drug-specific judgment.  How vulnerable is the infant?  Extra caution is needed for premature infants, newborns in the first days of life, infants with jaundice, dehydration, poor weight gain, apnea, liver disease, kidney disease, or other illness.  Can breastfeeding continue safely with monitoring?  In many cases, breastfeeding can continue. The CDC states that most medications are safe for breastfeeding mothers and infants, while also directing clinicians to LactMed for drug-specific recommendations.  For common postpartum pain, acetaminophen and ibuprofen are usually preferred. AAFP also identifies acetaminophen and ibuprofen as preferred analgesics during lactation and notes that maternal opioid use can cause infant sedation.  The final plan should document the medication name, dose, duration, lactation safety source used, counseling given, infant warning signs, and follow-up plan. Breastfeeding should only be interrupted when there is a clear evidence-based reason, and the mother should be helped to maintain milk supply if temporary interruption is unavoidable. |
| **Flesch–Kincaid Grade Level** | 12.05 | 12.22 | 12.48 |
| **Corresponding English Reading-Difficulty Category** | College: difficult | College: difficult | College: difficult |

## Question 19

| Item | Attempt 1 | Attempt 2 | Attempt 3 |
| --- | --- | --- | --- |
| Clinical knowledge question | What conditions may be suggested by postpartum urinary retention, dysuria, urinary frequency, or urinary incontinence, and how should initial management and referral decisions be made? | | |
| Main guideline source(s) | NICE NG194 | | |
| Model response | Postpartum urinary symptoms may reflect temporary physiological changes, but they can also indicate significant pelvic floor, neurological, infectious, or bladder dysfunction.  Urinary retention after delivery may suggest bladder overdistension, impaired bladder sensation after epidural anesthesia, pelvic floor trauma, severe perineal pain, vulvar edema, hematoma, or nerve injury. Overt postpartum urinary retention is commonly defined as inability to void within about 6 hours after vaginal birth or catheter removal following cesarean delivery.  Dysuria may suggest urinary tract infection, urethral irritation from catheterization, perineal trauma, concentrated urine, or wound irritation.  Urinary frequency and urgency may occur with urinary tract infection, bladder irritation, incomplete bladder emptying, postpartum diuresis, or overactive bladder symptoms.  Urinary incontinence may suggest pelvic floor muscle injury, urethral sphincter weakness, obstetric nerve injury, or severe perineal trauma, especially after instrumental birth or prolonged second stage. Stress incontinence is common postpartum, but persistent or severe symptoms require assessment.  Initial assessment should include timing of symptoms, ability to void, urine volume, pain, fever, hematuria, suprapubic pain, leakage pattern, neurological symptoms, constipation, perineal trauma, mode of delivery, epidural use, catheterization history, and fluid intake.  Examination should include abdominal palpation for bladder distension, assessment of perineal wounds and edema, neurological status when indicated, and vital signs. If retention is suspected, bladder scanning or catheterization may be needed to assess residual urine volume. RCOG notes that women should pass urine within 6 hours after delivery or catheter removal, and if not, bladder assessment is needed.  Referral is needed urgently if there is inability to void, severe suprapubic pain, large residual volume, fever, flank pain, hematuria, neurological deficits, suspected sepsis, worsening retention, or persistent incontinence affecting function and quality of life. | Different postpartum urinary symptoms point toward different possible problems.  Retention often suggests impaired bladder emptying. Risk factors include epidural anesthesia, prolonged labor, instrumental delivery, severe perineal trauma, vulvar swelling, cesarean birth, and postpartum pain. If untreated, bladder overdistension may damage bladder function.  Painful urination usually raises concern for urinary tract infection, but clinicians should also think about urine passing over perineal tears or episiotomy wounds, catheter trauma, dehydration, or local irritation.  Frequency and urgency may reflect cystitis, incomplete emptying, excessive postpartum diuresis, pelvic floor irritation, or bladder hypersensitivity.  Urinary leakage may indicate stress incontinence from pelvic floor weakness, urge incontinence from bladder instability, overflow incontinence from retention, or fistula in rare severe cases.  The initial management depends on the symptom pattern.  For retention, the priority is preventing bladder overdistension. The woman should be encouraged to void in a comfortable environment, mobilize if possible, use adequate analgesia, and try simple measures such as warm water over the perineum. If she still cannot void or has a large residual volume, catheterization is usually required. A postpartum bladder care review notes that untreated retention may lead to bladder dysfunction and recurrent urinary tract problems.  For dysuria or frequency, urine testing may be needed. Fever, flank pain, or systemic illness suggests upper urinary tract involvement and requires prompt treatment.  For urinary incontinence, clinicians should assess whether leakage occurs with coughing or exertion, or whether urgency is present. Pelvic floor exercises and postpartum rehabilitation may help mild early symptoms. Persistent symptoms, severe leakage, neurological findings, or suspected fistula should prompt specialist referral.  Referral decisions should consider severity, persistence, residual urine volume, infection signs, neurological symptoms, and impact on daily function. | Postpartum urinary symptoms should never be dismissed automatically as “normal after childbirth.” They may indicate temporary dysfunction, but they can also signal important complications.  Postpartum urinary retention may present as inability to void, incomplete emptying, weak stream, dribbling, suprapubic fullness, abdominal discomfort, or overflow leakage. It may be associated with epidural anesthesia, prolonged labor, operative vaginal delivery, severe perineal trauma, edema, pain, or pudendal nerve injury.  Dysuria commonly suggests urinary tract infection or irritation from catheterization or perineal trauma. If associated with fever, urgency, frequency, flank pain, or foul-smelling urine, infection becomes more likely.  Frequency and urgency may occur with cystitis, postpartum bladder irritation, incomplete emptying, or pelvic floor dysfunction.  Urinary incontinence may result from pelvic floor injury, connective tissue stretching, urethral sphincter weakness, nerve damage, or severe obstetric trauma. Cleveland Clinic notes that postpartum incontinence can follow pregnancy and childbirth because of strain and injury to pelvic floor structures.  The initial assessment should include:  When the symptoms began.  Delivery type and duration.  Instrumental delivery or epidural use.  Catheter history.  Fluid intake and urine output.  Pain, fever, hematuria, or flank pain.  Bowel symptoms and constipation.  Perineal pain or swelling.  Leakage pattern and triggers.  Neurological symptoms such as numbness or leg weakness.  Examination should include vital signs, abdominal examination for bladder distension, inspection of perineal wounds and edema, and neurological assessment when needed. Urinalysis and urine culture may be appropriate if infection is suspected. Residual bladder volume should be checked if retention or incomplete emptying is suspected.  Management may include timed voiding, pain control, hydration, constipation management, pelvic floor exercises, bladder scanning, intermittent or indwelling catheterization, antibiotics when indicated, and physiotherapy support.  Urgent referral is needed for severe retention, inability to void, fever with urinary symptoms, hematuria, severe pelvic pain, neurological deficits, suspected fistula, recurrent retention, or persistent significant incontinence beyond the expected early recovery period. |
| **Flesch–Kincaid Grade Level** | 19.99 | 15.51 | 15.30 |
| **Corresponding English Reading-Difficulty Category** | College graduate: very difficult | College: difficult | College: difficult |

## Question 20

| Item | Attempt 1 | Attempt 2 | Attempt 3 |
| --- | --- | --- | --- |
| Clinical knowledge question | What are the common contributing factors for postpartum constipation and hemorrhoids, and what should clinical nursing care and health education include? | | |
| Main guideline source(s) | Chinese Maternal Health Management Specification; NICE NG194 | | |
| Model response | Postpartum constipation is very common. It may be related to reduced mobility, dehydration, inadequate dietary fiber, fear of pain during defecation, perineal tears, episiotomy, cesarean pain, hemorrhoid pain, iron supplementation, and opioid analgesics. ACOG notes that bowel movements may be difficult for a few days after delivery, partly because women may fear pain from hemorrhoids or perineal tears.  Hemorrhoids are also common after childbirth. They may be caused or worsened by pregnancy-related venous pressure, constipation, straining, prolonged second stage of labor, pushing during delivery, and reduced bowel motility after birth. They usually present with anal pain, itching, swelling, discomfort when sitting, and sometimes bright red bleeding with bowel movements.  Clinical nursing care should include assessment of bowel pattern, time since last bowel movement, stool hardness, straining, anal pain, rectal bleeding, perineal wound pain, hemorrhoid swelling, fluid and fiber intake, mobility, medications, and fear of defecation. Nurses should also assess whether the woman has severe abdominal distension, vomiting, inability to pass gas, heavy rectal bleeding, severe anal pain, fever, or suspected thrombosed hemorrhoid.  Health education should emphasize fluids, fiber-rich food, early gentle ambulation, regular toileting, avoiding prolonged sitting on the toilet, and not straining. Stool softeners or mild laxatives may be used when needed, especially after severe perineal trauma or opioid use. For hemorrhoid discomfort, cold compresses, warm sitz baths, good anal hygiene, and suitable topical treatments can help. NHS advice for piles includes avoiding straining, using exercise to relieve constipation, applying cold cloths for pain, and cleaning gently after bowel movements. | The main contributing factors for postpartum constipation include hormonal and physical changes, pain, reduced activity, and medications. Progesterone-related bowel slowing may persist for a time after birth. Dehydration, especially during breastfeeding, can make stool harder. Women may also avoid defecation because they are afraid of tearing stitches, worsening hemorrhoids, or causing pain. Iron tablets and opioid pain medicines can further aggravate constipation. NHS Inform specifically notes that avoiding medicines that cause constipation, such as codeine-containing painkillers, may help hemorrhoid symptoms.  Hemorrhoids are often linked with constipation and straining. Pregnancy and childbirth increase pressure on the pelvic and rectal veins. Pushing during labor may enlarge pre-existing hemorrhoids or trigger new symptoms. Postpartum hemorrhoids can cause itching, swelling, pain, burning, and bright red blood on tissue or stool.  Clinical nursing care should include:  Assessment  Ask about bowel movement frequency, stool consistency, straining, rectal bleeding, anal pain, hemorrhoid symptoms, perineal wound pain, diet, fluid intake, activity level, and medications.  Comfort care  Encourage warm sitz baths, cold packs for swelling, side-lying rest, gentle cleaning, and avoiding rubbing the anus. Analgesics compatible with breastfeeding may be used when appropriate. ACOG recommends acetaminophen and ibuprofen as first-line postpartum analgesics for people providing breast milk.  Bowel support  Encourage water, fiber, fruits, vegetables, whole grains, and walking. Stool softeners or laxatives may be used according to local protocol and clinical condition.  Referral  Refer if there is severe or persistent rectal bleeding, black stool, severe anal pain, a hard bluish painful lump suggesting thrombosis, fever, worsening abdominal pain, vomiting, inability to pass stool or gas, wound breakdown, or symptoms that do not improve. | Postpartum constipation and hemorrhoids are usually caused by several factors acting together. The woman may be less mobile, drink less than needed, eat irregularly, use iron or opioid medication, and avoid defecation because of perineal pain. At the same time, pregnancy and pushing during birth increase pressure on rectal veins, making hemorrhoids more likely.  Nursing care should focus on prevention first. Women should be encouraged to drink enough water, eat fiber-containing foods, walk gently as soon as safe, and respond to the urge to defecate rather than holding stool. They should be reassured that normal defecation will not usually break properly repaired stitches. The Breastfeeding Network also advises avoiding constipation because it can worsen hemorrhoids.  For bowel movement education, women should be advised to sit comfortably on the toilet, support the feet if helpful, breathe slowly, avoid prolonged straining, and protect the perineum with a clean pad if they feel anxious about stitches. Stool softeners are often useful when stool is hard or when the woman has a painful tear, hemorrhoids, or opioid-related constipation.  For hemorrhoid care, education should include cold compresses for swelling, warm sitz baths for comfort, gentle cleaning with water or moist tissue, patting dry rather than rubbing, and avoiding long periods of sitting or standing. NHS guidance also recommends avoiding straining and cleaning the anal area gently after passing stool.  Medical review is needed if constipation is severe, persistent, associated with vomiting or abdominal distension, or if hemorrhoids cause heavy bleeding, severe pain, suspected thrombosis, infection, or failure to improve with conservative care. |
| **Flesch–Kincaid Grade Level** | 15.25 | 13.00 | 13.51 |
| **Corresponding English Reading-Difficulty Category** | College: difficult | College: difficult | College: difficult |

## Question 21

| Item | Attempt 1 | Attempt 2 | Attempt 3 |
| --- | --- | --- | --- |
| Clinical knowledge question | What symptoms may indicate postpartum pelvic floor dysfunction, and when should pelvic floor rehabilitation assessment or referral be recommended? | | |
| Main guideline source(s) | NICE NG194 | | |
| Model response | Postpartum pelvic floor dysfunction may involve the bladder, bowel, pelvic organs, sexual function, and pelvic pain. NICE defines pelvic floor dysfunction as problems in which the pelvic floor muscles around the bladder, anal canal, and vagina do not work properly, and includes urinary incontinence, bladder emptying problems, fecal incontinence, bowel emptying problems, pelvic organ prolapse, sexual dysfunction, and chronic pelvic pain.  Common symptoms include urinary leakage when coughing, sneezing, laughing, lifting, or walking. Some women have urgency, frequent urination, difficulty starting urine flow, incomplete bladder emptying, or recurrent urinary tract symptoms.  Bowel-related symptoms include fecal urgency, inability to control gas, leakage of stool, difficulty emptying the bowel, or needing to press around the vagina or perineum to pass stool. These symptoms are especially important after a third- or fourth-degree tear.  Symptoms of pelvic organ prolapse include pelvic heaviness, dragging sensation, vaginal bulge, pressure, feeling that something is coming down, difficulty using tampons later, or difficulty emptying the bladder or bowel. ACOG lists pelvic pressure or fullness, organs bulging from the vagina, urinary leakage, and difficulty with bowel movements as possible pelvic support symptoms.  Sexual and pain symptoms may include painful intercourse, reduced sensation, vaginal or perineal pain, pelvic pressure, scar pain, or persistent pelvic girdle pain.  Pelvic floor rehabilitation assessment or referral should be recommended when symptoms are moderate or severe, persist beyond the early recovery period, worsen over time, affect daily activity or emotional wellbeing, or occur after severe perineal trauma. Referral should also be made for fecal incontinence, gas incontinence, suspected prolapse, urinary retention, recurrent urinary leakage, pelvic pain that limits function, or uncertainty about how to perform pelvic floor exercises correctly. ACOG recommends assessing urinary and fecal continence postpartum, with referral to physical therapy or urogynecology when indicated. | Postpartum pelvic floor dysfunction may be suggested by several groups of symptoms.  The first group is urinary symptoms. These include stress urinary incontinence, urgency, frequent urination, nocturia, urine leakage before reaching the toilet, weak stream, incomplete emptying, or urinary retention.  The second group is bowel symptoms. These include constipation with outlet obstruction, fecal urgency, stool leakage, flatus incontinence, painful defecation, or needing manual pressure to empty the bowel.  The third group is prolapse symptoms. Women may report vaginal heaviness, pelvic dragging, a lump at the vaginal opening, pressure that worsens after standing, or a sensation that the uterus, bladder, or bowel is descending.  The fourth group is pain and sexual symptoms. These include persistent perineal pain, deep pelvic pain, painful intercourse, scar tenderness, pelvic floor spasm, and fear of returning to sexual activity.  Initial assessment should include childbirth history, mode of delivery, prolonged second stage, forceps or vacuum use, birth weight, episiotomy, third- or fourth-degree tear, wound healing, urinary symptoms, bowel symptoms, sexual symptoms, pain score, activity level, and psychological impact. Examination may include perineal inspection, pelvic floor muscle contraction assessment, prolapse assessment, wound or scar assessment, and neurological signs when indicated.  Referral is recommended if the woman has anal incontinence, fecal urgency, inability to control gas, significant urinary leakage, urinary retention, prolapse symptoms, persistent pelvic pain, dyspareunia, suspected wound or sphincter injury, or inability to correctly contract the pelvic floor. RCOG emphasizes that postnatal care should include information and support on pelvic floor health, including at discharge and the 6-week postnatal check, so women seek help early for symptoms. | A simple way to screen postpartum pelvic floor dysfunction is to ask whether the woman is leaking, unable to empty, feeling pressure, or having pain.  Symptoms that may indicate pelvic floor dysfunction include:  Urine leakage with coughing, sneezing, laughing, lifting, or exercise.  Sudden urgency to pass urine.  Difficulty emptying the bladder.  Repeated urinary tract symptoms.  Leakage of stool or gas.  Strong fecal urgency.  Difficulty emptying the bowel.  Vaginal bulge or heaviness.  Pelvic dragging or pressure.  Persistent perineal, vaginal, or pelvic pain.  Painful intercourse.  Reduced ability to return to walking, exercise, or daily care because of pelvic symptoms.  Assessment should include direct but sensitive questioning, because many women do not volunteer these symptoms unless asked. The clinician should ask about bladder control, bowel control, pelvic pressure, pain, sexual function when appropriate, quality of life, and whether the woman knows how to perform pelvic floor muscle exercises.  Pelvic floor muscle training should usually be encouraged after birth when appropriate. A Cochrane review notes that pelvic floor muscle training is commonly recommended during pregnancy and after birth for prevention and treatment of incontinence.  Referral for pelvic floor rehabilitation should be recommended when symptoms are persistent, bothersome, severe, or complex. It is also appropriate after obstetric anal sphincter injury, instrumental delivery with major symptoms, suspected prolapse, fecal or flatus incontinence, urinary retention, significant urinary leakage, pelvic pain, or if the woman is unsure whether she is exercising the correct muscles. Urgent specialist assessment is needed for new neurological deficits, severe pelvic pain, inability to pass urine, suspected fistula, or wound breakdown. |
| **Flesch–Kincaid Grade Level** | 17.57 | 15.10 | 11.92 |
| **Corresponding English Reading-Difficulty Category** | College graduate: very difficult | College: difficult | Grades 10–12: fairly difficult |

## Question 22

| Item | Attempt 1 | Attempt 2 | Attempt 3 |
| --- | --- | --- | --- |
| Clinical knowledge question | Which factors should guide decisions about when to resume physical activity and pelvic floor muscle training during the puerperium? | | |
| Main guideline source(s) | NICE NG194; WHO 2022 | | |
| Model response | Decisions about resuming physical activity and pelvic floor muscle training during the puerperium should be guided by the woman’s clinical recovery, not only by the number of days after birth.  Important factors include:  The mode of birth, whether vaginal or cesarean.  The degree of perineal trauma, especially third- or fourth-degree tears.  Cesarean incision healing.  Amount of vaginal bleeding and whether lochia is decreasing normally.  Pain level and whether pain is improving.  Fatigue, sleep, anemia, dizziness, or poor nutrition.  Bladder and bowel function.  Presence of urinary leakage, fecal leakage, pelvic heaviness, or prolapse symptoms.  Pregnancy or delivery complications, such as hemorrhage, infection, hypertensive disorders, or thromboembolism risk.  Previous activity level before and during pregnancy.  For uncomplicated births, gentle activity such as walking, stretching, and pelvic floor exercises can usually begin when the woman feels able. ACOG states that some women can resume physical activities within days after delivery, and pelvic floor exercises can be started in the immediate postpartum period.  The progression should be gradual. If activity causes increased bleeding, worsening pain, pelvic pressure, urinary leakage, wound discomfort, dizziness, or marked fatigue, the intensity should be reduced and the woman should be reassessed. High-impact exercise, running, heavy lifting, and intense abdominal exercises should usually wait until the woman has recovered adequately and has received postpartum review, especially if she has pelvic floor symptoms or surgical wounds. | The first principle is that postpartum exercise should begin with low-impact recovery activity, then gradually progress. It should not begin with intense training.  In the early puerperium, suitable activities may include short walks, gentle mobility, breathing exercises, posture correction, and gentle pelvic floor contractions. NHS guidance says that after a straightforward birth, women can start gentle exercise as soon as they feel up to it, including walking, gentle stretches, and pelvic floor exercises. It also advises getting advice at the 6-week postnatal check before starting high-impact exercise such as running or aerobics.  Pelvic floor muscle training should be started gently and correctly. The woman should be able to contract and relax the pelvic floor without pain, breath-holding, buttock clenching, or excessive abdominal straining. If she cannot identify the correct muscles, has pelvic heaviness, urinary or fecal leakage, or pain, she should be referred for pelvic floor rehabilitation assessment.  Key factors guiding the timing include wound healing, pain, bleeding, bladder emptying, bowel control, pelvic floor symptoms, abdominal muscle separation, breastfeeding fatigue, and medical complications. For cesarean birth, the incision must be healing well, and activities that strongly strain the abdomen should be delayed until recovery is adequate.  A safe progression means the woman can exercise without increased bleeding, wound pain, pelvic dragging, urine leakage, fecal leakage, dizziness, or severe exhaustion. If these occur, she should reduce activity and seek assessment. | Clinicians should individualize advice by asking three questions.  Is there any medical reason to delay activity?  Delay or modify exercise if there is heavy bleeding, fever, infection, uncontrolled hypertension, severe anemia, wound breakdown, thromboembolism symptoms, severe pain, urinary retention, or significant dizziness.  Is the pelvic floor ready for loading?  Pelvic floor muscle training can often start early, but only within comfort. NHS physiotherapy guidance advises starting pelvic floor exercises as soon as possible after delivery, but only if they do not cause pain. Women with urinary leakage, fecal leakage, pelvic heaviness, vaginal bulge, or inability to contract the pelvic floor correctly need pelvic floor physiotherapy assessment.  Is the activity level appropriate for the stage of recovery?  Early activity should be gentle and progressive. Walking and gentle exercises are usually safer than running, jumping, heavy weights, or high-intensity abdominal training. HSE guidance recommends starting with gentle physical activity after birth and gradually adding other activities between 6 and 12 weeks.  The woman should be educated to stop or reduce activity if she notices heavier bleeding, increasing abdominal or perineal pain, cesarean wound discomfort, pelvic pressure, urinary or fecal leakage, chest pain, shortness of breath, calf pain, or severe fatigue. These are signs that the body is not tolerating the activity or that a complication may be present. |
| **Flesch–Kincaid Grade Level** | 12.79 | 13.30 | 13.60 |
| **Corresponding English Reading-Difficulty Category** | College: difficult | College: difficult | College: difficult |

## Question 23

| Item | Attempt 1 | Attempt 2 | Attempt 3 |
| --- | --- | --- | --- |
| Clinical knowledge question | Why is the puerperium a high-risk period for venous thromboembolism, and which symptoms should raise concern for deep vein thrombosis or pulmonary embolism? | | |
| Main guideline source(s) | NICE NG194; RCOG Green-top Guideline No. 37a | | |
| Model response | The puerperium is a high-risk period for venous thromboembolism because pregnancy and the postpartum state create a strong tendency toward clot formation. The body becomes more hypercoagulable to reduce bleeding at delivery. At the same time, venous flow from the legs may be slower because of pregnancy-related venous pressure and reduced mobility. Vascular injury from delivery, cesarean section, hemorrhage, infection, dehydration, or prolonged bed rest can further increase the risk. ACOG states that women who are pregnant or postpartum have a fourfold to fivefold increased risk of thromboembolism compared with nonpregnant women.  Symptoms concerning for deep vein thrombosis include one-sided calf or thigh pain, swelling of one leg, redness, warmth, tenderness, and pain that is persistent or worse when standing or walking. ACOG lists DVT symptoms as warmth or tenderness, pain or sudden swelling, redness of the skin, and constant pain in one leg while standing or walking.  Symptoms concerning for pulmonary embolism include sudden shortness of breath, chest pain or tightness, rapid breathing, coughing blood, fainting, collapse, palpitations, or feeling suddenly very unwell. RCOG advises immediate help for sudden unexplained difficulty breathing, chest pain or tightness, coughing blood, feeling very unwell, or collapse.  Any postpartum woman with suspected pulmonary embolism needs urgent emergency evaluation. A painful swollen leg should also be assessed promptly, and the leg should not be massaged while DVT is being considered. | Postpartum VTE risk is high because the puerperium combines all three elements of Virchow’s triad. First, blood clotting activity is increased after pregnancy and delivery. Second, venous stasis may occur because pregnancy increases venous pressure in the legs and because postpartum women may be less mobile. Third, vessel injury may occur during childbirth, surgery, perineal trauma, or cesarean delivery. Merck Manual explains that pregnancy increases VTE risk because venous pressure and venous stasis increase, and pregnancy causes hypercoagulability.  The risk is highest soon after delivery. RCOG notes that the highest risk of DVT is just after having the baby, although the condition is still uncommon, occurring in about 1 to 2 in 1000 women during pregnancy or the first 6 weeks after birth.  Possible DVT symptoms include:  One leg swelling more than the other.  Calf or thigh pain.  Localized warmth.  Redness or skin color change.  Tenderness along the deep veins.  Pain that does not improve with rest.  Possible PE symptoms include:  Sudden breathlessness.  Chest pain.  Coughing blood.  Rapid heartbeat.  Dizziness, fainting, or collapse.  Severe unexplained anxiety or feeling of impending illness.  Clinical teaching should emphasize that PE symptoms are emergency symptoms. A normal postpartum explanation such as fatigue, anxiety, or musculoskeletal pain should not be accepted without assessment if shortness of breath or chest pain is present. | The puerperium is dangerous for VTE because the maternal body is still recovering from pregnancy and delivery. Clotting factors remain increased, the pelvic and leg veins may still be relatively slow-flowing, and many women have reduced activity because of pain, fatigue, cesarean wound healing, anemia, or postpartum complications. Additional risk factors include previous VTE, thrombophilia, obesity, age over 35, smoking, severe infection, postpartum hemorrhage, blood transfusion, prolonged labor, operative delivery, and cesarean section. RCOG includes long labor over 24 hours, cesarean birth, heavy blood loss, and blood transfusion among postpartum risk factors.  Concern for DVT should be raised by unilateral leg swelling, calf pain, thigh pain, redness, warmth, tenderness, or a leg that is clearly larger than the other. Bilateral mild ankle swelling can occur postpartum, but one-sided painful swelling is not something to reassure away.  Concern for PE should be raised by sudden shortness of breath, chest pain, chest tightness, rapid breathing, coughing blood, syncope, collapse, or marked unexplained weakness. Thromboembolic disorders, including DVT and PE, are a leading cause of maternal mortality, so postpartum cardiopulmonary symptoms require a low threshold for urgent evaluation.  Initial clinical response should include rapid assessment of vital signs, oxygen saturation, cardiopulmonary symptoms, leg findings, and VTE risk factors. Suspected PE requires emergency referral. Suspected DVT requires prompt diagnostic evaluation and anticoagulation decisions according to local obstetric or medical guidance. |
| **Flesch–Kincaid Grade Level** | 12.99 | 9.03 | 15.11 |
| **Corresponding English Reading-Difficulty Category** | College: difficult | Grades 10–12: fairly difficult | College: difficult |

## Question 24

| Item | Attempt 1 | Attempt 2 | Attempt 3 |
| --- | --- | --- | --- |
| Clinical knowledge question | For women with a history of gestational hypertension or pre-eclampsia, what should be monitored postpartum, and which symptoms require urgent management? | | |
| Main guideline source(s) | NICE NG194; ACOG Optimizing Postpartum Care | | |
| Model response | Women with a history of gestational hypertension or pre-eclampsia need close postpartum monitoring because blood pressure can remain high, worsen again, or first become dangerous after delivery. Postpartum pre-eclampsia can occur after birth, and new-onset hypertension from 48 hours to 6 weeks postpartum should be taken seriously.  Postpartum monitoring should include:  Blood pressure  Blood pressure should be checked regularly after birth, especially in the first week. NICE recommends that women with pre-eclampsia have blood pressure measured at least once between day 3 and day 5 after birth.  Symptoms of severe disease  Ask about headache, visual changes, upper abdominal pain, nausea, vomiting, shortness of breath, chest pain, reduced urine output, dizziness, fainting, and swelling of the face or hands.  Laboratory and organ function when indicated  If pre-eclampsia was present or symptoms develop, clinicians may need to monitor urine protein, platelet count, liver enzymes, kidney function, and sometimes fluid balance.  Medication safety and adherence  Check whether antihypertensive medication is being taken correctly. If breastfeeding, choose medicines compatible with lactation and monitor the baby if specific antihypertensives require caution.  Long-term cardiovascular risk  Women with hypertensive disorders of pregnancy have increased future risk of chronic hypertension and cardiovascular disease, so they need transition to primary care and long-term risk counseling.  Urgent management is required for severe headache that does not improve, blurred vision or flashing lights, seizure, severe upper abdominal or right upper quadrant pain, shortness of breath, chest pain, fainting, severe nausea or vomiting, marked swelling of the face or hands, very high blood pressure, confusion, or reduced urine output. CDC lists persistent or worsening headache, vision changes, extreme swelling of the hands or face, trouble breathing, chest pain, and dizziness or fainting as urgent maternal warning signs. | Postpartum care for women with gestational hypertension or pre-eclampsia should focus on blood pressure control, early recognition of complications, medication review, and long-term follow-up.  The most important monitoring item is blood pressure. Blood pressure should be measured before discharge and again after discharge according to local protocol. Women should also be taught home blood pressure monitoring if available. Severe hypertension, often defined clinically as systolic blood pressure 160 mmHg or above or diastolic blood pressure 110 mmHg or above, is an emergency and needs urgent treatment.  Clinicians should also monitor for symptoms suggesting progression to severe pre-eclampsia or eclampsia. These include severe headache, visual disturbance, epigastric or right upper abdominal pain, nausea, vomiting, shortness of breath, pulmonary edema symptoms, oliguria, seizure, or altered mental status. ACOG guidance lists severe headache, visual changes, epigastric pain, and shortness of breath among symptoms of pre-eclampsia with severe features.  If symptoms or abnormal blood pressure occur, assessment may include blood pressure confirmation, neurological assessment, urine protein testing, complete blood count, platelet count, serum creatinine, liver enzymes, and evaluation for pulmonary edema or other complications.  Urgent referral is needed if the woman has severe-range blood pressure, seizure, severe or persistent headache, visual symptoms, chest pain, shortness of breath, severe upper abdominal pain, confusion, syncope, or signs of stroke. These symptoms should not be explained as normal postpartum fatigue or anxiety without evaluation. | For a woman who had gestational hypertension or pre-eclampsia, postpartum follow-up should be active because delivery does not immediately remove risk. Blood pressure may peak several days after birth, and severe complications can occur after discharge.  Healthcare professionals should monitor:  Blood pressure trend.  Headache and visual symptoms.  Epigastric or right upper quadrant pain.  Nausea and vomiting.  Urine output.  Shortness of breath and chest symptoms.  Edema, especially sudden swelling of the face or hands.  Medication use and adverse effects.  Breastfeeding compatibility of antihypertensive drugs.  Recovery from any related complications, such as kidney, liver, platelet, or neurological problems.  Need for later cardiovascular and metabolic follow-up.  The woman and family should be taught clear warning signs. She should seek urgent care for severe headache, headache that gets worse, blurred vision, seeing spots, temporary vision loss, seizure, fainting, chest pain, fast heartbeat, shortness of breath, severe abdominal pain, severe nausea or vomiting, confusion, or sudden marked swelling. ACOG describes pre-eclampsia warning features such as low platelets, abnormal kidney or liver function, upper abdominal pain, pulmonary fluid, vision changes, and severe headache.  Management decisions depend on blood pressure level, symptoms, and organ involvement. Mild persistent hypertension may be managed with close follow-up and oral medication. Severe hypertension, neurological symptoms, chest or breathing symptoms, seizure, or abnormal organ function requires urgent hospital assessment and treatment. |
| **Flesch–Kincaid Grade Level** | 16.10 | 15.21 | 11.55 |
| **Corresponding English Reading-Difficulty Category** | College graduate: very difficult | College: difficult | Grades 10–12: fairly difficult |

## Question 25

| Item | Attempt 1 | Attempt 2 | Attempt 3 |
| --- | --- | --- | --- |
| Clinical knowledge question | For women with a history of gestational diabetes mellitus, what should postpartum glucose follow-up and long-term metabolic risk management include? | | |
| Main guideline source(s) | NICE NG194; ACOG Optimizing Postpartum Care | | |
| Model response | For women with a history of gestational diabetes mellitus, postpartum care should include both early glucose reassessment and long-term prevention of type 2 diabetes.  The first step is postpartum glucose testing. Most women with GDM no longer need glucose-lowering treatment after delivery, but they still need testing because some will have persistent diabetes or prediabetes. ADA recommends screening people with recent GDM at 4 to 12 weeks postpartum using a 75 g oral glucose tolerance test and non-pregnancy diagnostic criteria.  Follow-up should include:  Postpartum glucose test  A 75 g 2-hour oral glucose tolerance test is preferred in many guidelines because fasting glucose or HbA1c alone may miss impaired glucose tolerance. If the test is abnormal, the woman should be referred for diabetes or prediabetes management.  Lifestyle and weight management  Counsel about balanced diet, gradual postpartum physical activity, healthy weight, adequate sleep when possible, and reducing sugary drinks and highly processed foods. The aim is not rapid weight loss, but sustainable metabolic risk reduction.  Breastfeeding support  Breastfeeding should be encouraged when possible. It may support maternal metabolic health and infant health, and most routine postpartum diabetes follow-up can be arranged without stopping breastfeeding.  Long-term screening  Even if the 4 to 12 week test is normal, future screening is still needed. ADA recommends lifelong screening for type 2 diabetes or prediabetes every 1 to 3 years after GDM.  Future pregnancy planning  Before the next pregnancy, she should receive preconception counseling, early glucose testing, weight and lifestyle advice, and review of recurrence risk. | Postpartum follow-up after GDM should not end at delivery. GDM is a marker of future metabolic risk, especially type 2 diabetes.  Clinically, management should include the following.  First, stop or reassess pregnancy glucose treatment after birth. Insulin or oral medication used only for GDM is often stopped after delivery, but this should be individualized if glucose remains high or diabetes was suspected before pregnancy.  Second, arrange formal postpartum glucose testing. The usual recommended window is 4 to 12 weeks postpartum, and the preferred test is a 75 g 2-hour OGTT because it can detect both fasting and post-load glucose abnormalities. ACOG guidance also recommends postpartum screening in this time window for women with GDM.  Third, classify the result clearly. If diabetes is diagnosed, refer for diabetes care. If prediabetes or impaired glucose tolerance is found, provide structured lifestyle intervention and consider medical prevention according to local guidance. If the test is normal, explain that risk is reduced but not gone.  Fourth, provide long-term care. This includes periodic glucose testing, cardiovascular risk assessment, weight management, blood pressure monitoring, lipid assessment when appropriate, and support for physical activity and nutrition.  Fifth, prepare for future pregnancies. Women with prior GDM should be tested early in the next pregnancy. NICE recommends offering self-monitoring of blood glucose or a 75 g 2-hour OGTT as soon as possible for women with previous GDM who book in early pregnancy. | For a woman who had GDM, postpartum care should answer three questions.  Has glucose returned to normal?  She should have postpartum glucose testing, ideally with a 75 g OGTT at 4 to 12 weeks after delivery. This is important because some women have unrecognized type 2 diabetes or prediabetes after pregnancy. StatPearls notes that postpartum screening can use fasting plasma glucose or a 75 g 2-hour OGTT, but fasting glucose may miss some abnormal glucose metabolism.  What is her future risk?  Women with GDM have a substantially increased lifetime risk of type 2 diabetes. The woman should be told this clearly, but without blame. Long-term management should include regular diabetes screening, healthy eating support, gradual return to activity, weight management where appropriate, and attention to blood pressure and cardiovascular risk.  How can risk be reduced and future pregnancy made safer?  Education should include breastfeeding support, sleep and stress realities, nutrition, physical activity, contraception and pregnancy spacing, and preconception glucose testing before a future pregnancy. She should also be told to inform future maternity care providers about her GDM history.  Referral is recommended if postpartum glucose testing shows diabetes, prediabetes, persistent hyperglycemia, symptoms of hyperglycemia, difficulty with weight or nutrition management, or if she plans another pregnancy and needs preconception care. |
| **Flesch–Kincaid Grade Level** | 12.69 | 10.45 | 12.03 |
| **Corresponding English Reading-Difficulty Category** | College: difficult | Grades 10–12: fairly difficult | College: difficult |

## Question 26

| Item | Attempt 1 | Attempt 2 | Attempt 3 |
| --- | --- | --- | --- |
| Clinical knowledge question | How can postpartum blues, postpartum depression, and severe psychiatric symptoms be distinguished, and what should be specifically assessed during clinical evaluation? | | |
| Main guideline source(s) | NICE CG192; NICE NG194; Chinese Maternal Health Management Specification | | |
| Model response | Postpartum blues, postpartum depression, and severe psychiatric symptoms differ mainly by duration, severity, functional impairment, and risk level.  Postpartum blues are mild and common. They usually begin within the first few days after birth and may include tearfulness, mood swings, irritability, anxiety, emotional sensitivity, and sleep difficulty. The mother can usually still care for herself and the baby. Symptoms usually improve within several days and should not last beyond about 2 weeks. Mayo Clinic describes baby blues as commonly beginning within the first 2 to 3 days after delivery and lasting up to two weeks.  Postpartum depression is more persistent and impairing. It may include low mood, loss of interest, guilt, hopelessness, fatigue, sleep disturbance beyond infant care, appetite change, poor concentration, anxiety, poor bonding, feelings of being unable to cope, and thoughts of death or self-harm. Merck Manual defines postpartum depression as depressive symptoms during the first year after delivery that last more than 2 weeks and meet criteria for major depression.  Severe psychiatric symptoms include postpartum psychosis, severe depression with suicidal intent, severe bipolar symptoms, mania, delirium-like confusion, hallucinations, delusions, paranoia, disorganized behavior, or thoughts of harming the baby. These are emergencies. NICE includes severe mental illness such as psychosis, bipolar disorder, schizophrenia, and severe depression in postnatal mental health care.  Clinical evaluation should specifically assess mood, anxiety, sleep, appetite, energy, concentration, guilt, bonding with the infant, ability to care for self and baby, family support, domestic safety, substance use, previous depression or bipolar disorder, previous psychosis, current medication, and obstetric stressors.  The clinician must directly ask about self-harm, suicidal thoughts, thoughts of harming the baby, hallucinations, delusions, severe agitation, confusion, and loss of touch with reality. If any of these are present, the woman needs urgent psychiatric and obstetric assessment, and the baby’s safety must be protected. | A practical way to distinguish these conditions is to ask whether the symptoms are mild and short-lived, persistent and disabling, or dangerous and reality-distorting.  Postpartum blues are usually mild mood symptoms. The mother may cry easily, feel overwhelmed, or become irritable, but she remains oriented, does not have psychotic symptoms, and does not have persistent suicidal thoughts. Reassurance, sleep support, practical help, breastfeeding support, and follow-up are usually enough.  Postpartum depression is suspected when symptoms last more than 2 weeks, become more intense, or interfere with daily life. Important symptoms include persistent sadness, loss of pleasure, hopelessness, excessive guilt, anxiety, panic, poor sleep even when the baby sleeps, appetite change, poor bonding, social withdrawal, and feeling unable to care for the baby. Depression and anxiety affect about 15% to 20% of women in the first year after childbirth according to NICE-related guidance.  Severe psychiatric symptoms are suggested by hallucinations, delusions, paranoia, severe confusion, disorganized speech, severe insomnia with abnormal energy, reckless behavior, mania, suicidal intent, or thoughts of harming the infant. Postpartum psychosis is rare but dangerous, affecting about 1 to 2 per 1000 women after birth.  Clinical assessment should include screening tools when available, such as the Edinburgh Postnatal Depression Scale, but the interview is essential. Ask about symptom onset, duration, severity, prior mental illness, bipolar disorder, family history, trauma, birth experience, infant health problems, sleep deprivation, social support, intimate partner violence, and substance use.  The most important safety questions are direct ones. Ask whether she has wished she would not wake up, thought of killing herself, made a plan, heard voices, seen things, believed the baby is evil or unsafe, felt commanded to act, or had thoughts of harming the baby. Positive answers require urgent escalation. | Postpartum blues are a normal-range emotional reaction for many women after birth. Symptoms are brief, fluctuate during the day, and improve with rest, reassurance, and family support. The mother may cry easily but can usually feel pleasure and respond to comfort.  Postpartum depression is a clinical mood disorder. The symptoms are more persistent, more painful, and more impairing. The mother may feel empty, hopeless, guilty, numb, detached from the baby, or unable to cope. She may stop enjoying things, withdraw from others, have intrusive worries, or feel that the baby would be better without her. ACOG recommends screening for perinatal depression and anxiety at the initial prenatal visit, later in pregnancy, and postpartum visits.  Severe psychiatric symptoms are emergency symptoms. These include suicidal thoughts with intent or plan, thoughts of harming the baby, hallucinations, delusions, paranoia, severe agitation, mania, extreme insomnia, confusion, or behavior that is clearly unsafe. Postpartum psychosis needs immediate specialist care because judgment and reality testing may be impaired.  During clinical evaluation, healthcare professionals should assess:  Mood, anxiety, irritability, panic, intrusive thoughts, sleep, appetite, fatigue, concentration, guilt, hopelessness, and bonding.  Ability to feed, hold, comfort, and safely care for the baby.  Self-care, hydration, nutrition, hygiene, and ability to rest.  Support from partner, family, and community.  Domestic violence, coercion, or unsafe home environment.  Past history of depression, anxiety, bipolar disorder, psychosis, suicide attempt, trauma, or substance use.  Current medications and whether treatment was stopped during pregnancy or breastfeeding.  Infant factors such as prematurity, NICU admission, feeding problems, or excessive crying.  The key clinical rule is simple. Baby blues can be monitored with support. Postpartum depression needs active treatment and follow-up. Psychosis, suicidal intent, or harm-to-baby thoughts need urgent emergency management. |
| **Flesch–Kincaid Grade Level** | 14.90 | 13.60 | 11.89 |
| **Corresponding English Reading-Difficulty Category** | College: difficult | College: difficult | Grades 10–12: fairly difficult |

## Question 27

| Item | Attempt 1 | Attempt 2 | Attempt 3 |
| --- | --- | --- | --- |
| Clinical knowledge question | How should self-harm, suicidal ideation, or thoughts of harming the baby during the puerperium be risk-assessed and managed urgently? | | |
| Main guideline source(s) | NICE CG192 | | |
| Model response | Self-harm, suicidal ideation, or thoughts of harming the baby during the puerperium should be treated as urgent safety concerns, not as ordinary postpartum stress. ACOG recommends that when a woman answers positively to a self-harm or suicide question, clinicians should immediately assess the likelihood, acuity, and severity of suicide risk, then arrange management according to risk level.  The risk assessment should be direct and specific. Clinicians should ask:  Whether she has thoughts of death, self-harm, suicide, or harming the baby.  Whether the thoughts are passive or active.  Whether she has a plan, method, access to means, or a time frame.  Whether she has acted on these thoughts before.  Whether she feels able to stay safe.  Whether she has hallucinations, delusions, paranoia, confusion, or command voices.  Whether she has severe insomnia, agitation, impulsivity, mania, substance use, or domestic violence.  Whether there is a responsible adult who can stay with her and help protect the baby.  High-risk findings include a clear suicide plan, access to lethal means, intent to act, previous suicide attempt, command hallucinations, delusions involving the baby, severe agitation, confusion, intoxication, inability to care for the baby safely, or any active thought of harming the infant.  Urgent management should include not leaving the woman alone, ensuring the baby is safe, involving emergency psychiatric services, and arranging urgent hospital or crisis evaluation. If there is imminent risk, emergency services should be contacted. Postpartum psychosis requires immediate medical attention, and ACOG specifically recommends immediate medical attention for postpartum psychosis. | The clinical response should begin with safety. The clinician should calmly but clearly ask whether the mother is at risk of harming herself or the baby now. This includes asking about intent, plan, means, recent behavior, and ability to resist the thoughts.  The assessment should distinguish intrusive thoughts from high-risk thoughts. Some women with postpartum anxiety or obsessive-compulsive symptoms may have unwanted, frightening thoughts that distress them greatly, but they have no intent to act and actively avoid harm. These still need assessment and treatment. However, thoughts linked with intent, loss of control, psychosis, delusional beliefs, or command hallucinations require emergency management.  The clinician should assess:  Current suicidal thoughts.  Self-harm behavior.  Thoughts of harming the baby.  Intent and plan.  Access to medicines, weapons, high places, or other means.  Psychotic symptoms.  Manic symptoms.  Severe depression.  Substance use.  Sleep loss.  Domestic violence.  Social support.  Infant safety.  Previous psychiatric history, especially bipolar disorder, psychosis, or suicide attempt.  Postpartum psychosis is especially dangerous because reality testing may be impaired. Cleveland Clinic describes postpartum psychosis as a mental health emergency that can involve hallucinations, delusions, paranoia, and in severe cases attempts to harm oneself or the newborn.  Management should be risk-based. If there is active intent, plan, psychosis, severe agitation, confusion, or any immediate risk to the infant, the woman needs urgent emergency psychiatric assessment, often hospital admission. The baby should be placed under safe supervision by another responsible adult. The clinician should involve family or carers when appropriate and should not rely on routine outpatient follow-up alone. | Self-harm, suicidal thoughts, or thoughts of harming the baby in the puerperium should be assessed as a maternal and infant safety emergency until risk is clearly judged to be low.  The first step is immediate triage. Ask whether she has already harmed herself, taken medication, used alcohol or drugs, injured the baby, or feels she may act soon. If yes, emergency response is required.  The second step is structured risk assessment. Important questions include:  What exactly are the thoughts?  How often do they occur?  Are they unwanted and frightening, or do they feel reasonable to her?  Does she have a plan?  Does she have access to the method?  Has she rehearsed or prepared?  Has she made a suicide note or said goodbye?  Has she previously attempted suicide?  Does she hear voices or believe unusual things?  Does she believe the baby is evil, unsafe, better off dead, or not really hers?  Has she slept at all?  Is she severely agitated, confused, or manic?  Who is with her now?  Can the baby be safely cared for by another adult?  The third step is safety action. Remove or restrict access to means when possible. Do not leave her alone if risk is significant. Ensure another adult supervises the infant. Contact emergency mental health services, obstetric services, or emergency medical services according to severity. NICE guidance on antenatal and postnatal mental health emphasizes prompt recognition, assessment, treatment, and monitoring of mental health problems in the postnatal period, including severe mental illness.  The fourth step is treatment planning. Low-risk intrusive thoughts still need mental health referral, close follow-up, psychoeducation, sleep support, and family involvement. Moderate or high risk needs same-day specialist assessment. Psychosis, suicidal intent, infanticidal intent, severe mania, severe agitation, or inability to guarantee safety usually requires emergency psychiatric care and often inpatient treatment. |
| **Flesch–Kincaid Grade Level** | 12.70 | 10.14 | 8.60 |
| **Corresponding English Reading-Difficulty Category** | College: difficult | Grades 10–12: fairly difficult | Grades 8–9: plain English |

## Question 28

| Item | Attempt 1 | Attempt 2 | Attempt 3 |
| --- | --- | --- | --- |
| Clinical knowledge question | Which physiological, psychological, and wound-healing factors should be considered before resuming sexual activity after childbirth, and when is further assessment needed? | | |
| Main guideline source(s) | NICE NG194; Chinese Maternal Health Management Specification | | |
| Model response | Resuming sexual activity after childbirth should depend on physical recovery, emotional readiness, wound healing, and contraception, rather than a fixed date. There is no single rule that applies to every woman. NHS guidance states that there are no rules about when to start having sex again after birth, and women should not rush if they feel sore or tired.  Physiological factors include whether lochia has stopped or become minimal, whether vaginal bleeding has settled, whether pelvic or abdominal pain is controlled, whether the woman has enough energy, and whether breastfeeding-related vaginal dryness is present. Breastfeeding and postpartum hormonal changes can make the vagina drier and more sensitive, so lubricant may be helpful.  Wound-healing factors include recovery of perineal tears, episiotomy wounds, cesarean incision, vaginal bruising, and pelvic floor trauma. For women with third- or fourth-degree tears, RCOG advises that sex can be resumed when stitches have healed, bleeding has stopped, and the woman feels ready.  Psychological factors are also important. The clinician should ask about fear of pain, body image concerns, fatigue, low libido, birth trauma, anxiety, depression, relationship pressure, and whether the woman feels safe and willing. Sexual activity should be consensual and comfortable, not something resumed because of pressure from a partner.  Further assessment is needed if intercourse causes persistent or severe pain, bleeding, wound pain, foul discharge, fever, pelvic pressure, urinary or fecal leakage, suspected prolapse, severe vaginal dryness, loss of sexual desire with distress, fear related to traumatic birth, or symptoms of depression or anxiety. | Before resuming sexual activity after childbirth, clinicians should help the woman consider four questions.  Has the body recovered enough?  Bleeding should have stopped or become very light. Pain should be improving. The woman should not have fever, heavy lochia, foul-smelling discharge, severe pelvic pain, or signs of infection.  Has the wound healed?  Perineal tears, episiotomy wounds, and cesarean incisions should be healing well. There should be no wound opening, pus, increasing redness, swelling, or worsening tenderness. RCOG notes that after a third- or fourth-degree tear, sex can be resumed once stitches have healed and bleeding has stopped, when it feels right for the woman.  Is the woman emotionally ready?  Postpartum fatigue, sleep deprivation, breastfeeding demands, anxiety, depression, fear of pain, and changed body image may all affect sexual desire. These are common and should be discussed without judgment.  Is contraception in place?  Pregnancy can occur before the first postpartum period. RCOG reminds women that they can become pregnant very soon after birth and should consider contraception before intercourse. ACOG also emphasizes postpartum birth control to avoid unintended pregnancy and support pregnancy spacing.  Further clinical review is needed if there is pain that does not improve with lubricant and gentle attempts, bleeding after sex, wound discomfort, abnormal discharge, pelvic heaviness, urinary or fecal incontinence, or concern about anal sphincter injury. Women with severe perineal trauma, persistent dyspareunia, or pelvic floor symptoms may benefit from pelvic floor physiotherapy or specialist referral. | Sexual activity after childbirth should be resumed only when the woman feels physically comfortable, emotionally ready, and free to choose.  Physiological issues to consider include tiredness, sleep deprivation, breastfeeding, vaginal dryness, breast tenderness, hormonal changes, pelvic floor weakness, abdominal discomfort, and reduced libido. Mayo Clinic notes that postpartum hormonal changes can make the vagina dry and sore, especially during breastfeeding, which may make sex uncomfortable.  Wound-related issues include episiotomy healing, perineal tear repair, cesarean incision recovery, bruising, scar tenderness, and pelvic floor injury. A wound that is red, swollen, painful, open, draining pus, or associated with fever should be assessed before sexual activity is resumed.  Psychological and relationship issues include fear of pain, anxiety about the birth experience, depression, body image changes, pressure from a partner, lack of sleep, and reduced emotional closeness. These factors can be as important as physical healing.  Further assessment is needed when there is:  Persistent painful intercourse.  Bleeding during or after sex.  Foul-smelling discharge or fever.  Increasing perineal or abdominal pain.  Cesarean or perineal wound problems.  Vaginal bulge or pelvic heaviness.  Urinary leakage, fecal leakage, or inability to control gas.  Severe vaginal dryness not improving with lubricant.  Flashbacks, panic, depression, or fear related to childbirth.  Concern about coercion or lack of safety in the relationship.  The clinical message should be reassuring but clear: it is normal for sexual recovery to take time, but pain, bleeding, wound symptoms, infection signs, pelvic floor symptoms, or emotional distress should be assessed rather than ignored. |
| **Flesch–Kincaid Grade Level** | 14.14 | 10.44 | 12.38 |
| **Corresponding English Reading-Difficulty Category** | College: difficult | Grades 10–12: fairly difficult | College: difficult |

## Question 29

| Item | Attempt 1 | Attempt 2 | Attempt 3 |
| --- | --- | --- | --- |
| Clinical knowledge question | Why is contraceptive counselling needed in the early postpartum period, and what factors should be considered when selecting contraception for breastfeeding and non-breastfeeding women? | | |
| Main guideline source(s) | FSRH Contraception After Pregnancy; NICE NG194; Chinese Maternal Health Management Specification | | |
| Model response | Contraceptive counselling is needed early in the postpartum period because ovulation can return before the first menstrual period, and a woman can become pregnant again before she realizes fertility has returned. Early counselling helps avoid unintended pregnancy, supports healthy pregnancy spacing, and allows the woman to choose a method before sexual activity resumes. ACOG notes that some birth control methods can be started right after childbirth, while others require waiting a few weeks. It also emphasizes that postpartum birth control helps prevent pregnancy too soon after birth.  The choice of contraception should consider:  Whether she is breastfeeding.  Time since delivery.  Risk of venous thromboembolism.  Mode of delivery and postpartum complications.  Blood pressure, migraine, smoking, obesity, diabetes, liver disease, and thrombosis history.  Desire for short-term or long-term contraception.  Future fertility plan.  Need for protection against sexually transmitted infections.  Ease of use and adherence.  For breastfeeding women, non-estrogen methods are usually preferred early. These include condoms, copper IUD, levonorgestrel IUD, etonogestrel implant, progestin-only pill, and injectable progestin when appropriate. Estrogen-containing combined hormonal methods may reduce milk supply in some women and are restricted in the early postpartum period because of clot risk and lactation concerns. CDC’s 2024 U.S. Medical Eligibility Criteria gives method-specific postpartum recommendations and should be used for women with medical conditions or breastfeeding considerations.  For non-breastfeeding women, more options are available earlier, but clot risk remains important in the first postpartum weeks. Combined hormonal contraceptives should not be started immediately after birth in women with increased VTE risk. Long-acting reversible contraception, such as IUDs and implants, may be suitable if the woman wants highly effective contraception and has no contraindications. | Early postpartum contraception counselling is important because many women resume sexual activity before the routine postpartum visit, and fertility may return unpredictably. Waiting until the 6-week visit may miss the chance to prevent an unintended short-interval pregnancy. ACOG recommends that postpartum care be an ongoing process, with early contact in the first 3 weeks and comprehensive care by 12 weeks, which creates an opportunity to discuss contraception before risk occurs.  For breastfeeding women, the clinician should consider whether the method affects milk production, whether it is safe for the infant, and whether it fits the mother’s feeding plan. Progestin-only methods and IUDs are commonly used during breastfeeding. The lactational amenorrhea method can be effective only when strict criteria are met: exclusive or near-exclusive breastfeeding, amenorrhea, and less than 6 months postpartum. If any of these criteria are not met, another method is needed. WHO developed postpartum contraception guidance to help women choose safe options after childbirth whether or not they are breastfeeding.  For non-breastfeeding women, ovulation can return earlier, so contraception should be discussed before discharge or at the first postpartum contact. Options may include condoms, IUDs, implant, injectable contraception, progestin-only pills, combined pills, patch, or ring. However, combined hormonal contraception still requires attention to postpartum timing and thrombosis risk. The CDC 2024 U.S. MEC is the current evidence-based reference for matching contraceptive methods to postpartum timing and medical risk factors.  Counselling should also include dual protection. Most contraceptive methods do not prevent sexually transmitted infections. Condoms should be recommended when STI protection is needed. | The purpose of early postpartum contraceptive counselling is not simply to prescribe a method. It is to help the woman choose a method that is safe, acceptable, effective, and compatible with breastfeeding and recovery.  The counselling should cover three points.  First, pregnancy can occur again soon after childbirth. Menstruation is not a reliable sign of fertility returning, because ovulation may happen first.  Second, short pregnancy intervals may increase maternal and infant risks, so planning the next pregnancy matters.  Third, different methods have different postpartum timing rules. Some can be used immediately after delivery, while others should be delayed.  For breastfeeding women, the main factors are milk supply, infant safety, maternal clot risk, and convenience. Barrier methods and non-hormonal methods can be used. IUDs and implants are highly effective options. Progestin-only methods are generally preferred over estrogen-containing methods in early breastfeeding. Estrogen-containing pills, patches, or rings require caution because of both thrombotic risk in the early postpartum period and possible effects on milk production. A review comparing guidelines notes that recommendations differ somewhat, but combined hormonal contraceptives are restricted early postpartum, especially in breastfeeding women.  For non-breastfeeding women, the main factors are rapid return of ovulation, VTE risk, medical contraindications, and personal preference. Long-acting reversible contraception can be offered if desired. Combined hormonal methods may be considered after the early high-risk period if there are no contraindications.  Further counselling or specialist input is needed if the woman has previous VTE, thrombophilia, severe hypertension, migraine with aura, complex cardiac disease, liver disease, breast cancer, postpartum hemorrhage with severe anemia, severe infection, or uncertainty about IUD placement after complicated delivery. |
| **Flesch–Kincaid Grade Level** | 11.69 | 11.92 | 13.13 |
| **Corresponding English Reading-Difficulty Category** | Grades 10–12: fairly difficult | Grades 10–12: fairly difficult | College: difficult |

## Question 30

| Item | Attempt 1 | Attempt 2 | Attempt 3 |
| --- | --- | --- | --- |
| Clinical knowledge question | What core components should be included in postpartum maternal health education to ensure that women can recognize abnormal symptoms and seek timely help? | | |
| Main guideline source(s) | WHO 2022; NICE NG194; Chinese Maternal Health Management Specification | | |
| Model response | Postpartum maternal health education should help women and families understand two things: what is expected during recovery, and what symptoms mean they should seek help immediately. ACOG emphasizes that postpartum care should be an ongoing process rather than a single visit, with care tailored to the woman’s physical, social, and psychological needs.  Core education should include the following areas.  Normal postpartum recovery  Women should be taught about normal lochia changes, uterine cramping, breast changes, perineal or cesarean wound discomfort, fatigue, sweating, mood fluctuation, and gradual return of bowel and bladder function.  Danger signs  They should seek urgent care for heavy vaginal bleeding, large clots, bleeding that suddenly increases, fever of 38°C or higher, foul-smelling lochia, severe abdominal pain, severe headache, visual changes, seizure, chest pain, shortness of breath, fainting, one-sided leg swelling or pain, or feeling too weak to get out of bed. CDC lists several urgent maternal warning signs after pregnancy, including persistent or worsening headache, dizziness or fainting, vision changes, fever, extreme swelling of the hands or face, trouble breathing, chest pain, and thoughts of harming oneself or the baby.  Wound and infection education  Women should know how to observe a perineal wound or cesarean incision. Increasing redness, swelling, warmth, pain, pus, bad odor, wound opening, fever, or chills should prompt medical assessment.  Breastfeeding and breast problems  Education should include effective latch, signs of adequate milk transfer, engorgement care, nipple pain, mastitis symptoms, and when to seek help for breast redness, fever, flu-like symptoms, or a persistent painful lump.  Mental health  Women and families should be told that mild emotional changes can occur, but persistent sadness, anxiety, hopelessness, inability to sleep, poor bonding, hallucinations, confusion, suicidal thoughts, or thoughts of harming the baby require urgent help.  Follow-up and access to care  Before discharge, women should know when their postpartum follow-up is scheduled, whom to call during the day, whom to call at night, and when to go directly to emergency care. They should also be advised to tell any healthcare provider that they have recently given birth. | Postpartum health education should be practical, repeated, and easy for the woman and family to act on. WHO guidance states that postpartum danger signs include increased vaginal bleeding, fits, fast or difficult breathing, fever with severe weakness, severe headache with blurred vision, calf pain with redness or swelling, shortness of breath, and chest pain. Women should not wait if these symptoms occur.  The education should include:  Bleeding and lochia  Explain that lochia should gradually become lighter in color and amount. Heavy bleeding, soaking pads quickly, repeated large clots, foul smell, or bleeding that becomes bright red again after decreasing needs assessment.  Fever and pain  Teach that fever, chills, increasing lower abdominal pain, uterine tenderness, foul-smelling lochia, breast redness with fever, wound pain, or painful urination may indicate infection.  Blood pressure and neurological symptoms  Women with or without previous hypertension should know that severe headache, blurred vision, flashing lights, upper abdominal pain, seizure, fainting, chest pain, shortness of breath, or sudden swelling of the face and hands are warning signs.  Thrombosis symptoms  One-sided calf pain, leg swelling, redness, warmth, chest pain, coughing blood, sudden breathlessness, or collapse requires urgent evaluation.  Breast and infant feeding  Include feeding frequency, latch, swallowing, wet and dirty diapers, breast engorgement, nipple cracking, mastitis symptoms, and where to obtain lactation support.  Bladder, bowel, and pelvic floor  Teach women to report inability to pass urine, painful urination with fever, urinary leakage that is severe or persistent, fecal leakage, inability to control gas, severe constipation, rectal bleeding, pelvic heaviness, or vaginal bulge.  Medication, contraception, and activity  Education should include safe use of prescribed medicines during breastfeeding, contraception before resuming sex, gradual return to activity, and warning signs that activity is too much, such as heavier bleeding, worsening pain, or pelvic pressure. | A complete postpartum maternal health education plan should cover self-monitoring, symptom recognition, self-care, and help-seeking pathways.  First, women should know what to monitor every day. This includes vaginal bleeding, lochia odor, temperature if unwell, pain level, wound condition, breast symptoms, urination, bowel movements, mood, sleep, and ability to care for the baby.  Second, they should know which symptoms are urgent. CDC’s Hear Her campaign advises women to get medical care immediately for urgent warning signs during or after pregnancy, and to talk with a healthcare provider about anything unusual or worrying. Warning symptoms include heavy bleeding, fever, severe headache, vision changes, chest pain, trouble breathing, fainting, severe abdominal pain, severe nausea or vomiting, extreme swelling, leg pain or swelling, and thoughts of harming oneself or the baby.  Third, women should receive self-care guidance. This includes perineal hygiene, cesarean incision care, pain control, hydration, nutrition, rest, gradual activity, constipation prevention, breast care, pelvic floor exercises, and safe medication use while breastfeeding.  Fourth, education should include emotional and social safety. Ask women to seek help for persistent sadness, anxiety, panic, traumatic birth memories, inability to sleep, hallucinations, confusion, suicidal thoughts, harm-to-baby thoughts, domestic violence, or lack of support at home.  Fifth, the care team should provide a clear follow-up plan. ACOG recommends contact with obstetric care providers within the first 3 weeks postpartum, followed by ongoing care as needed and a comprehensive postpartum visit no later than 12 weeks after birth. The woman should leave care knowing whom to contact, where to go in an emergency, and which symptoms should not wait for a routine appointment. |
| **Flesch–Kincaid Grade Level** | 15.57 | 15.91 | 13.31 |
| **Corresponding English Reading-Difficulty Category** | College: difficult | College: difficult | College: difficult |

# Guideline sources / 指南来源

- WHO recommendations on maternal and newborn care for a positive postnatal experience, 2022.
- NICE guideline NG194: Postnatal care.
- ACOG Committee Opinion No. 736: Optimizing Postpartum Care.
- Chinese National Basic Public Health Service Specification: Maternal Health Management Service Specification.
- NICE guideline CG192: Antenatal and postnatal mental health.
- FSRH Clinical Guideline: Contraception After Pregnancy.
- RCOG Green-top Guideline No. 37a: Reducing the Risk of Thrombosis and Embolism during Pregnancy and the Puerperium.
