# Supplementary material for: Beyond accuracy: a three-stage evaluation of large language models for postpartum health consultation and nursing decision support
Source: Front Public Health. 2026 Oct 20;14:1961647. doi: 10.3389/fpubh.2026.1961647 (PMC13727638; doi:10.3389/fpubh.2026.1961647)
Supplement: Supplementary file 10 [file Table_7.docx]

**Supplementary Table S7. Stage 1 ChatGPT 5.5 Chinese responses, English translations, expert ratings, and readability outcomes**

*30 postpartum maternal health clinical knowledge questions; each question was independently submitted to ChatGPT 5.5 three times.*

| 研究阶段 | 第一阶段：产褥期母体健康临床知识问题 |
| --- | --- |
| 模型选择 | ChatGPT 5.5 |
| 页面显示的具体模型/版本 | ChatGPT 5.5 Thinking |
| 访问平台 | ChatGPT官方网页界面 |
| 测试时间 | 2026年5月1日至6月30日 |
| 是否开启联网搜索 | 否 |
| 是否开启推理/深度思考模式 | 是；ChatGPT 5.5 Thinking |
| 是否上传外部文件或参考资料 | 否 |
| 提问方式 | 每个问题均在独立新对话中向该模型提交3次。 |
| 统一提示词 | 请作为一名具有循证医学意识的妇产科临床医生，回答以下产褥期母体健康相关临床知识问题。请基于权威指南和临床原则作答，内容应包括关键评估要点、可能原因、初步处理原则、危险信号和转诊指征。请避免编造不确定信息。 |
| 评分者 | 3名临床专家（隐藏模型来源后独立评分） |
| 语言 | 中文 |

# 评分说明

评分采用5分Likert量表：1分表示很差或明显不符合要求，2分表示较差，3分表示一般或部分符合，4分表示较好，5分表示优秀或完全符合要求。

| 评分维度 | 评价含义 |
| --- | --- |
| Correctness（正确性） | 回答是否符合权威指南和临床原则，是否存在事实性错误。 |
| Completeness（完整性） | 是否覆盖该问题的核心评估要点、常见原因、处理原则及必要注意事项。 |
| Guideline consistency（指南一致性） | 回答是否与WHO、NICE、ACOG、RCOG、FSRH及中国孕产妇健康管理相关指南/规范保持一致。 |
| Safety（安全性） | 是否避免危险、误导性或延误就医的建议，是否强调不确定情况需专业评估。 |
| Clinical management appropriateness（临床处理适当性） | 回答的临床评估和处理思路是否清晰、合理，能否区分正常恢复与异常情况，并提出恰当管理原则。 |

评分汇总规则 / Score aggregation rule: 表中每条回答所列评分为3名临床专家独立评分的中位数。 / The score shown for each response is the median of the ratings assigned independently by the three clinical experts.

翻译说明 / Translation note: 英文翻译由网页翻译工具生成并经人工校正，仅用于方便国际读者阅读，不用于专家评分或可读性分析。 / English translations were generated using a web-based translation tool and manually corrected. They are provided solely for international readers and were not used for expert scoring or readability analysis.

## 问题 1

| 项目 | 第1次 | 第2次 | 第3次 |
| --- | --- | --- | --- |
| 产褥期临床知识问题 | 产褥期的定义是什么？产褥期母体健康管理应重点评估哪些内容？ | | |
| 主要指南来源 | WHO 2022；ACOG产后照护优化建议 | | |
| 模型回答 | 产褥期是指胎儿、胎盘娩出后，产妇全身各器官（除乳腺外）逐渐恢复至非孕状态的一段时期，一般规定为产后6周，即42天。此阶段是女性生理、心理和社会角色转变的重要时期，也是产后并发症高发阶段，因此加强产褥期健康管理十分重要。  产褥期母体健康管理主要应重点评估以下几个方面：  首先，应重点观察生命体征及一般情况。包括体温、脉搏、呼吸、血压等变化。尤其需要警惕产后感染、产后出血及妊娠期高血压疾病的延续。若出现持续发热、血压升高、头痛或胸闷等症状，应及时进一步检查。  其次，要评估子宫复旧情况。正常情况下，子宫会逐渐缩小，恶露量逐渐减少、颜色由红转白。医护人员需观察宫底高度、子宫硬度以及恶露的颜色、气味和持续时间。如恶露增多、有臭味或子宫复旧不良，可能提示感染或胎盘残留。  第三，应关注乳房及泌乳情况。产后乳汁分泌逐渐建立，应评估乳房是否胀痛、有无乳头皲裂、乳腺堵塞或乳腺炎，同时指导正确哺乳姿势，促进母乳喂养成功。  第四，要评估会阴伤口及剖宫产切口恢复情况。观察有无红肿、渗液、裂开及疼痛情况，及时发现感染征象，并指导保持局部清洁干燥。  第五，需要关注排尿和排便功能。部分产妇会出现尿潴留、尿失禁或便秘等问题，应指导适当活动、增加饮水和膳食纤维摄入，必要时给予干预。  此外，心理状态评估也是产褥期管理的重要内容。由于激素变化、角色转换及照护压力，部分产妇可能出现产后情绪低落，严重者可发展为产后抑郁。应重点关注睡眠、情绪变化及家庭支持情况，必要时进行心理干预。  最后，还应进行营养、运动及避孕指导。鼓励均衡饮食、适量活动，促进身体恢复，同时指导科学避孕，避免短期内再次妊娠对母体造成影响。  因此，产褥期健康管理不仅是对生理恢复的观察，更是对产妇身心健康的全面管理，对于降低产后并发症、提高母婴健康水平具有重要意义。 | 产褥期是女性在分娩结束后，机体由妊娠状态逐渐恢复至正常未孕状态的过渡时期。临床上通常将胎盘娩出后至产后42天定义为产褥期。在这一阶段，产妇经历生理、内分泌、心理以及家庭角色等多方面变化，因此需要系统化健康管理。  产褥期母体健康管理首先应重视产妇整体恢复状况的评估。包括精神状态、饮食情况、睡眠质量及活动能力等，了解产妇是否存在明显疲劳、虚弱或恢复不良。  其次，应重点评估产后出血及子宫恢复情况。正常恶露会随着时间推移逐渐减少，由鲜红色变为淡红色再转白色。若恶露持续鲜红、量多或伴有异味，需要警惕子宫复旧不良、感染或胎盘胎膜残留。同时需检查宫底下降情况，判断子宫收缩是否良好。  对于感染风险的监测也非常关键。产褥期女性抵抗力相对下降，容易发生生殖道感染、泌尿系统感染及乳腺炎等。因此应观察体温变化、会阴或腹部伤口情况，以及是否存在尿频、尿痛等症状。  乳房护理是健康管理的重要组成部分。应评估泌乳量、婴儿吸吮情况以及乳头完整性，及时处理乳汁淤积和乳房胀痛，指导母乳喂养技巧，提高母乳喂养成功率。  同时，还需关注盆底功能恢复。部分女性在分娩后可能出现盆底肌松弛，表现为尿失禁、盆腔器官脱垂等，因此应早期开展盆底功能筛查，并指导凯格尔运动等康复训练。  心理健康管理同样不可忽视。由于激素波动和育儿压力，部分产妇会出现焦虑、情绪低落甚至产后抑郁。医务人员需主动评估其情绪变化、人际关系及家庭支持情况，必要时给予专业心理帮助。  此外，还应进行慢性疾病随访。对于妊娠期糖尿病、高血压等患者，产后仍需监测血糖、血压变化，防止疾病持续或转化为慢性疾病。  总之，产褥期健康管理强调“身心一体化”评估，不仅要促进产妇生理恢复，更应保障其心理和社会适应能力。 | 产褥期是指分娩后产妇身体各系统逐步恢复的时期，自胎盘娩出开始，到产后6周结束。此期间子宫、生殖道、循环系统及内分泌系统等都会发生明显变化，是母体恢复的重要阶段，也是观察和预防产后并发症的关键时期。  在产褥期健康管理中，首先要重点评估产妇是否存在产后并发症。最常见且危险的是产后出血与感染。因此需要监测阴道流血情况、子宫收缩状态以及体温变化。如果出现大量出血、寒战、高热或腹痛，应及时处理。  其次，需关注子宫及生殖系统恢复情况。医护人员通常通过触诊宫底高度和观察恶露变化来判断子宫复旧是否正常。正常恶露无明显臭味，并会逐渐减少。若持续时间过长或出现脓性分泌物，则提示异常。  对于分娩相关损伤的恢复也应重点观察。阴道分娩产妇需要查看会阴侧切或裂伤愈合情况；剖宫产产妇则应观察腹部切口有无红肿、渗液及疼痛加重等表现。  乳房健康管理同样十分重要。应了解乳汁分泌是否顺畅、婴儿吸吮是否有效，并预防乳头皲裂和急性乳腺炎。母乳喂养不仅有利于新生儿健康，也能促进子宫收缩和母体恢复。  此外，产褥期还需重视代谢及排泄功能恢复。部分女性因分娩损伤、卧床或疼痛影响，可能出现排尿困难、便秘或痔疮加重，因此应指导合理饮食及适度活动。  随着现代医学模式转变，心理评估已成为产褥期管理的重要内容。产妇在照护婴儿、睡眠不足及家庭角色变化过程中，容易产生焦虑和抑郁情绪。若持续存在情绪低落、兴趣减退或消极想法，应尽早进行心理干预。  另外，还需开展健康教育，包括营养摄入、产后运动、盆底康复、性生活恢复及避孕措施等内容，提高产妇自我保健能力。  因此，产褥期健康管理是一项涵盖生理、心理及社会支持的综合性管理，其核心目标是促进产妇全面康复，保障母婴安全。 |
| 中文回答的英文翻译 English translation of Chinese response | The postpartum period refers to the time following the delivery of the fetus and placenta during which the mother’s organs (excluding the mammary glands) gradually return to their pre-pregnancy state. It is generally defined as the six weeks following childbirth, or 42 days. This stage marks a critical period of physiological, psychological, and social transition for women, and it is also a time when postpartum complications are most likely to occur; therefore, strengthening postpartum health management is of utmost importance.  Maternal health management during the postpartum period should primarily focus on assessing the following aspects:  First, close monitoring of vital signs and general condition is essential. This includes changes in body temperature, pulse, respiration, and blood pressure. Particular vigilance is required regarding postpartum infections, postpartum hemorrhage, and the persistence of pregnancy-induced hypertension. If symptoms such as persistent fever, elevated blood pressure, headache, or chest tightness occur, further examination should be conducted promptly.  Second, the progress of uterine involution must be assessed. Under normal circumstances, the uterus gradually shrinks, and lochia gradually decreases in volume and changes from red to white. Healthcare providers should monitor the fundal height, uterine firmness, as well as the color, odor, and duration of lochia. An increase in lochia, a foul odor, or poor uterine involution may indicate infection or retained placental tissue.  Third, attention should be paid to the breasts and lactation. As milk production gradually establishes itself postpartum, assess for breast tenderness, cracked nipples, blocked ducts, or mastitis. Additionally, provide guidance on proper breastfeeding techniques to promote successful breastfeeding.  Fourth, assess the healing of perineal wounds and cesarean incisions. Observe for redness, swelling, discharge, splitting, or pain to promptly identify signs of infection, and provide guidance on maintaining local cleanliness and dryness.  Fifth, monitor urinary and bowel function. Some postpartum women may experience urinary retention, urinary incontinence, or constipation. They should be advised to engage in appropriate physical activity, increase fluid intake, and consume more dietary fiber; interventions should be provided as necessary.  In addition, psychological assessment is a crucial component of postpartum care. Due to hormonal changes, role transitions, and the stress of caregiving, some women may experience postpartum low mood, which in severe cases can develop into postpartum depression. Special attention should be given to sleep patterns, emotional changes, and family support, with psychological intervention provided as needed.  Finally, guidance on nutrition, exercise, and contraception should be provided. Mothers should be encouraged to maintain a balanced diet and engage in moderate physical activity to promote physical recovery, while also receiving guidance on effective contraception to prevent the adverse effects of a subsequent pregnancy on the mother’s health in the short term.  Therefore, postpartum health management is not merely the observation of physical recovery but rather a comprehensive approach to the physical and mental well-being of the mother. It plays a crucial role in reducing postpartum complications and improving the health of both mother and infant. | The postpartum period is the transitional phase following childbirth during which a woman’s body gradually returns from a state of pregnancy to its normal, non-pregnant state. Clinically, the postpartum period is typically defined as the time from the delivery of the placenta until 42 days after childbirth. During this phase, new mothers undergo various changes—including physiological, endocrine, psychological, and shifts in family roles—and therefore require systematic health management.  Maternal health management during the postpartum period should first prioritize an assessment of the mother’s overall recovery. This includes evaluating her mental state, dietary intake, sleep quality, and mobility to determine whether she exhibits significant fatigue, weakness, or poor recovery.  Second, the focus should be on assessing postpartum hemorrhage and uterine involution. Normal lochia gradually decreases over time, changing from bright red to pale red and eventually to white. If lochia remains bright red, is heavy, or has an unusual odor, one must be vigilant for poor uterine involution, infection, or retained placental or membrane tissue. At the same time, the descent of the fundus should be checked to determine whether uterine contractions are adequate.  Monitoring for infection risks is also critical. Women in the postpartum period have relatively lower immunity and are prone to reproductive tract infections, urinary tract infections, and mastitis. Therefore, it is important to monitor changes in body temperature, the condition of perineal or abdominal wounds, and the presence of symptoms such as frequent urination or painful urination.  Breast care is an essential component of health management. It is important to assess milk production, the baby’s suckling ability, and the integrity of the nipples; promptly address milk stasis and breast engorgement; and provide guidance on breastfeeding techniques to improve the success rate of breastfeeding.  At the same time, attention must be paid to the recovery of pelvic floor function. Some women may experience pelvic floor muscle laxity after childbirth, manifesting as urinary incontinence or pelvic organ prolapse; therefore, early screening for pelvic floor function should be conducted, and rehabilitation exercises such as Kegel exercises should be recommended.  Mental health management is equally important. Due to hormonal fluctuations and the stress of parenting, some new mothers may experience anxiety, low mood, or even postpartum depression. Healthcare providers should proactively assess emotional changes, interpersonal relationships, and family support, and provide professional psychological assistance when necessary.  Furthermore, follow-up care for chronic conditions is essential. For patients with gestational diabetes, hypertension, and other conditions, blood glucose and blood pressure levels must continue to be monitored postpartum to prevent the persistence or progression of these conditions into chronic diseases.  In summary, postpartum health management emphasizes a “mind-body integrated” assessment approach, aiming not only to promote the mother’s physical recovery but also to ensure her psychological and social well-being. | The postpartum period refers to the time when a woman’s body gradually recovers after childbirth, beginning with the delivery of the placenta and ending six weeks postpartum. During this period, significant changes occur in the uterus, reproductive tract, circulatory system, and endocrine system. It is a crucial stage for maternal recovery and a critical time for monitoring and preventing postpartum complications.  In postpartum health management, the primary focus is on assessing whether the mother has any postpartum complications. The most common and dangerous complications are postpartum hemorrhage and infection. Therefore, it is necessary to monitor vaginal bleeding, uterine contractions, and changes in body temperature. If heavy bleeding, chills, high fever, or abdominal pain occur, prompt treatment is required.  Second, attention must be paid to the recovery of the uterus and reproductive system. Healthcare providers typically assess uterine involution by palpating the fundal height and observing changes in lochia. Normal lochia has no distinct odor and gradually decreases in volume. If it persists for an extended period or becomes purulent, this indicates an abnormality.  Recovery from delivery-related injuries should also be closely monitored. For women who delivered vaginally, the healing of perineal episiotomies or tears should be checked; for those who underwent cesarean sections, the abdominal incision should be monitored for signs such as redness, swelling, oozing, or increased pain.  Breast health management is equally important. It is essential to ensure that milk flow is unobstructed and that the infant is latching effectively, while also preventing nipple cracks and acute mastitis. Breastfeeding not only benefits the newborn’s health but also promotes uterine contractions and the mother’s recovery.  In addition, attention must be paid to the restoration of metabolic and excretory functions during the postpartum period. Some women may experience difficulty urinating, constipation, or worsening hemorrhoids due to birth-related injuries, bed rest, or pain; therefore, guidance on proper diet and moderate physical activity is essential.  With the shift in modern medical paradigms, psychological assessment has become a key component of postpartum care. New mothers are prone to anxiety and depression while caring for their infants, coping with sleep deprivation, and adjusting to changes in their family roles. If low mood, loss of interest, or negative thoughts persist, psychological intervention should be initiated as early as possible.  Furthermore, health education should be provided, covering topics such as nutritional intake, postpartum exercise, pelvic floor rehabilitation, resumption of sexual activity, and contraceptive methods, to enhance the mother’s self-care capabilities.  Consequently, postpartum health management is a comprehensive approach encompassing physiological, psychological, and social support, with the core objective of promoting the mother’s full recovery and ensuring the safety of both mother and infant. |
| Correctness（1-5） | 4 | 4 | 4 |
| Completeness（1-5） | 5 | 4 | 4 |
| Guideline consistency（1-5） | 4 | 3 | 3 |
| Safety（1-5） | 4 | 4 | 5 |
| Clinical management appropriateness（1-5） | 5 | 4 | 4 |
| Chinese Reading Difficulty Score | 13.80 | 13.39 | 14.53 |
| Corresponding Education Level (Chinese) | 初中学段 | 初中学段 | 高中学段 |
| Recommended Reading Age (Chinese) | 14岁左右 | 13岁左右 | 15岁左右 |

## 问题 2

| 项目 | 第1次 | 第2次 | 第3次 |
| --- | --- | --- | --- |
| 产褥期临床知识问题 | 产后访视和产后复查的主要目的是什么？医务人员在产后早期应重点询问和检查哪些方面？ | | |
| 主要指南来源 | WHO 2022；NICE NG194；中国孕产妇健康管理服务规范 | | |
| 模型回答 | 产后访视和产后复查是围产期保健的重要组成部分，其主要目的是及时了解产妇和新生儿的健康状况，促进母体恢复，早期发现并处理产后异常情况，提高母婴生活质量，降低产后并发症发生率。  产后访视通常在产妇出院后早期进行，多由社区医务人员、助产士或基层保健人员完成；而产后42天复查则是对整个产褥期恢复情况的系统评估。二者共同构成连续性母婴健康管理的重要环节。  在产后早期，医务人员首先应重点询问产妇的一般情况，包括精神状态、睡眠、饮食、活动能力及疲劳程度等。若产妇出现明显乏力、头晕、心悸等症状，应考虑贫血或恢复不良的可能。  其次，应重点了解阴道流血及恶露情况。包括恶露颜色、气味、持续时间和出血量。若恶露量突然增多、持续鲜红或伴有臭味，应警惕子宫复旧不良、感染或胎盘残留。  对子宫恢复情况的检查也十分重要。医务人员需触诊宫底高度及子宫硬度，判断子宫收缩是否良好。若子宫复旧缓慢、压痛明显，需进一步评估是否存在感染或宫腔残留。  乳房和母乳喂养情况是产后访视的重点内容之一。应询问乳汁分泌是否充足、哺乳是否顺利、有无乳房胀痛、乳头皲裂或发热等症状，同时观察新生儿吸吮情况，并给予母乳喂养指导。  此外，还应检查会阴伤口或剖宫产切口恢复情况。观察有无红肿、渗液、裂开及疼痛加重等感染征象，并指导保持局部卫生。  对于排尿和排便功能，也需要重点询问。部分产妇会出现尿潴留、尿失禁、便秘或痔疮等问题，需早期干预，避免影响恢复。  心理状态评估同样不可忽视。医务人员应主动询问产妇情绪变化、是否存在焦虑、易哭、失眠或兴趣减退等表现，警惕产后抑郁的发生，同时了解家庭支持情况。  最后，在产后复查阶段，还需进一步评估血压、体重、盆底功能、月经恢复及避孕需求。对于妊娠期糖尿病、高血压等高危产妇，应继续随访相关指标。  因此，产后访视和复查不仅是身体恢复的检查，更是对母婴健康、心理适应及家庭照护能力的全面评估。 | 产后访视和产后复查的核心目的在于保障母婴安全，促进产妇顺利度过产褥期，并通过连续性的健康管理，及时发现和处理异常问题。与此同时，还能够帮助产妇建立科学的育儿及自我保健意识，提高母乳喂养成功率和生活质量。  产后访视一般发生在出院后数天内，重点是发现早期危险信号；而产后42天复查则主要评估机体恢复程度及远期健康状况。  在产后早期，医务人员首先需要了解产妇是否存在异常症状，例如发热、腹痛、头痛、胸闷、呼吸困难等。这些症状可能提示感染、血栓形成或妊娠期高血压疾病持续存在。  其次，需要重点询问阴道流血情况。正常恶露会逐渐减少，颜色由红变淡。若产妇反映出血量增多、血块较多或恶露有异味，则需考虑晚期产后出血或感染。  对子宫恢复的检查也非常重要。医务人员通常通过腹部触诊判断宫底下降情况及子宫收缩状态。若子宫偏软、宫底下降不明显，提示子宫复旧不佳。  乳房情况也是访视重点。需询问是否存在乳汁不足、乳房疼痛、局部发热等症状，并观察哺乳姿势是否正确。早期有效指导有助于减少乳腺炎和母乳喂养失败。  对于伤口恢复，应根据分娩方式进行针对性检查。阴道分娩需观察会阴伤口愈合；剖宫产则应查看腹部切口是否存在感染、脂肪液化或裂开。  此外，还应评估产妇的生活功能恢复情况，包括是否能够正常排尿、排便及下床活动。有些女性产后会出现盆底功能障碍，表现为漏尿或盆腔坠胀，需要尽早识别。  心理和社会适应能力评估同样重要。医务人员应关注产妇是否存在持续情绪低落、焦虑、睡眠障碍及对婴儿照护缺乏信心等情况，并评估家庭支持系统。  在产后复查时，还需要进一步进行妇科检查及健康指导，包括盆底康复、性生活恢复、营养管理和避孕咨询等内容。  因此，产后访视与复查不仅是疾病筛查，更是保障女性产后全面康复的重要措施。 | 产后访视与产后复查是孕产妇保健体系中的关键内容，其主要目的在于监测产妇身体恢复情况、预防和发现产后并发症、指导科学育儿及母乳喂养，同时促进女性身心健康恢复。  产后早期属于母体变化最明显的阶段，很多严重并发症如产后出血、感染、乳腺炎及产后抑郁等，往往发生在这一时期。因此，医务人员的早期干预十分重要。  在询问病史时，首先要了解产妇整体恢复状态，包括是否有乏力、头晕、发热、腹痛或睡眠差等情况。特别是持续发热和明显腹痛，需要警惕产褥感染。  其次，应详细询问恶露情况。正常恶露会逐渐减少，颜色逐步变浅。如果产妇出现大量出血、血块较多或恶露有臭味，则可能存在子宫收缩不良或宫腔残留。  对子宫的检查是产后访视中的重要环节。通过触摸宫底高度及子宫硬度，可以判断子宫复旧是否正常。若宫底下降缓慢、子宫压痛明显，应进一步处理。  乳房检查同样非常关键。医务人员应观察乳房有无红肿、硬结及乳头损伤，并询问婴儿吸奶情况。及时指导正确哺乳方法，有助于减少乳汁淤积及乳腺炎。  对于会阴切口或剖宫产伤口，应检查是否存在红肿、渗液、疼痛加重等感染表现。同时指导产妇注意局部卫生和休息。  在产后早期，还应重点关注排尿排便情况。由于盆底损伤或疼痛影响，部分女性可能出现尿失禁、尿潴留及便秘，需要给予针对性指导。  另外，随着现代医学对心理健康的重视，产后情绪评估已成为重要内容。医务人员应主动询问产妇是否存在情绪低落、焦虑、易怒或对婴儿缺乏兴趣等表现，以便早期发现产后抑郁。  产后42天复查时，还会进一步评估月经恢复、盆底功能、血压、体重及避孕需求等，帮助女性恢复正常生活状态。  总而言之，产后访视和复查不仅关注疾病问题，更强调母婴整体健康和长期生活质量的改善。 |
| 中文回答的英文翻译 English translation of Chinese response | Postpartum visits and follow-up examinations are integral components of perinatal care. Their primary objectives are to promptly assess the health status of mothers and newborns, promote maternal recovery, detect and address postpartum complications early, improve the quality of life for both mothers and infants, and reduce the incidence of postpartum complications.  Postpartum home visits are typically conducted shortly after the mother is discharged from the hospital and are usually carried out by community health workers, midwives, or primary healthcare providers; the 42-day postpartum checkup, on the other hand, is a systematic assessment of recovery throughout the entire postpartum period. Together, these two components form a crucial part of continuous maternal and infant health management.  In the early postpartum period, healthcare providers should first focus on inquiring about the mother’s general condition, including her mental state, sleep, diet, mobility, and level of fatigue. If the mother exhibits symptoms such as marked weakness, dizziness, or palpitations, the possibility of anemia or poor recovery should be considered.  Second, attention should be given to vaginal bleeding and lochia, including its color, odor, duration, and volume. If lochia suddenly increases in volume, remains bright red, or is accompanied by a foul odor, one should be alert to the possibility of poor uterine involution, infection, or retained placental tissue.  Examination of uterine involution is also critical. Healthcare providers should palpate the fundal height and uterine firmness to assess the quality of uterine contractions. If uterine involution is slow or tenderness is prominent, further evaluation is needed to determine whether infection or retained products of conception are present.  Breast health and breastfeeding status are key components of postpartum visits. Inquire about milk supply, the ease of breastfeeding, and symptoms such as breast engorgement, cracked nipples, or fever. Observe the newborn’s suckling and provide breastfeeding guidance.  Additionally, examine the healing of perineal wounds or cesarean incisions. Look for signs of infection, such as redness, swelling, discharge, splitting, or increased pain, and provide guidance on maintaining local hygiene.  Urinary and bowel function should also be a key focus of the inquiry. Some postpartum women may experience urinary retention, urinary incontinence, constipation, or hemorrhoids, which require early intervention to prevent complications during recovery.  Psychological assessment is equally important. Healthcare providers should proactively inquire about emotional changes, including anxiety, tearfulness, insomnia, or loss of interest, to monitor for signs of postpartum depression, while also assessing the level of family support.  Finally, during the postpartum follow-up phase, further assessment is required of blood pressure, weight, pelvic floor function, return of menstruation, and contraceptive needs. For high-risk postpartum women, such as those with gestational diabetes or hypertension, relevant indicators should continue to be monitored.  Therefore, postpartum visits and follow-ups are not merely examinations of physical recovery but also comprehensive assessments of maternal and infant health, psychological adjustment, and the family’s ability to provide care. | The primary purpose of postpartum visits and follow-up examinations is to ensure the safety of both mother and baby, help new mothers smoothly navigate the postpartum period, and promptly identify and address any abnormalities through continuous health management. At the same time, these visits help new mothers develop a scientific approach to childcare and self-care, thereby improving breastfeeding success rates and quality of life.  Postpartum home visits typically occur within a few days of discharge and focus on identifying early warning signs, while the 42-day postpartum checkup primarily assesses the degree of physical recovery and long-term health status.  In the early postpartum period, healthcare providers must first determine whether the mother is experiencing any abnormal symptoms, such as fever, abdominal pain, headache, chest tightness, or shortness of breath. These symptoms may indicate infection, thrombosis, or the persistence of pregnancy-induced hypertension.  Second, it is essential to inquire specifically about vaginal bleeding. Normal lochia gradually decreases in volume and changes from red to a lighter color. If the mother reports increased bleeding, the presence of large blood clots, or foul-smelling lochia, late postpartum hemorrhage or infection should be considered.  Examination of uterine recovery is also critical. Healthcare providers typically use abdominal palpation to assess the descent of the fundus and the state of uterine contractions. If the uterus feels soft or the fundus has not descended significantly, this suggests poor uterine involution.  Breast health is another key focus of the visit. It is necessary to inquire about symptoms such as insufficient milk supply, breast pain, or localized fever, and to observe whether the breastfeeding posture is correct. Early, effective guidance helps reduce the incidence of mastitis and breastfeeding failure.  Regarding wound healing, targeted examinations should be conducted based on the mode of delivery. For vaginal deliveries, the healing of the perineal wound should be observed; for cesarean sections, the abdominal incision should be examined for signs of infection, fat liquefaction, or rupture.  Additionally, the mother’s recovery of daily functional abilities should be assessed, including her ability to urinate and defecate normally, as well as her mobility. Some women may develop pelvic floor dysfunction postpartum, manifesting as urinary incontinence or pelvic heaviness, which requires early identification.  Assessing psychological and social adjustment is equally important. Healthcare providers should monitor for persistent low mood, anxiety, sleep disturbances, and lack of confidence in infant care, and evaluate the family support system.  During postpartum follow-up visits, further gynecological examinations and health counseling are required, covering topics such as pelvic floor rehabilitation, resumption of sexual activity, nutritional management, and contraception counseling.  Therefore, postpartum visits and follow-up examinations are not merely disease screenings but also crucial measures to ensure women’s comprehensive postpartum recovery. | Postpartum visits and follow-up examinations are key components of the maternal and child health care system. Their primary objectives are to monitor the mother’s physical recovery, prevent and detect postpartum complications, provide guidance on proper childcare and breastfeeding, and promote the mother’s physical and mental well-being.  The early postpartum period is when the mother’s body undergoes the most significant changes, and many serious complications—such as postpartum hemorrhage, infections, mastitis, and postpartum depression—often occur during this time. Therefore, early intervention by healthcare professionals is crucial.  When taking a medical history, the first step is to assess the mother’s overall recovery, including whether she experiences fatigue, dizziness, fever, abdominal pain, or poor sleep. In particular, persistent fever and significant abdominal pain should raise suspicion of puerperal infection.  Second, a detailed inquiry into lochia should be conducted. Normal lochia gradually decreases in volume and lightens in color. If the mother experiences heavy bleeding, numerous blood clots, or foul-smelling lochia, it may indicate poor uterine contraction or retained products of conception.  Examination of the uterus is a critical component of postpartum visits. By palpating the fundal height and uterine firmness, healthcare providers can assess whether uterine involution is proceeding normally. If the fundus descends slowly or there is significant tenderness, further intervention is warranted.  Breast examination is equally critical. Healthcare providers should check for redness, swelling, lumps, or nipple damage, and inquire about the infant’s feeding patterns. Timely guidance on proper breastfeeding techniques helps reduce milk stasis and mastitis.  For perineal or cesarean incisions, check for signs of infection such as redness, swelling, discharge, or increased pain. Additionally, advise the mother on maintaining local hygiene and getting adequate rest.  In the early postpartum period, special attention should also be given to urinary and bowel function. Due to pelvic floor injuries or pain, some women may experience urinary incontinence, urinary retention, or constipation, requiring targeted guidance.  Furthermore, with the growing emphasis on mental health in modern medicine, postpartum emotional assessment has become a critical component. Healthcare providers should proactively inquire about symptoms such as low mood, anxiety, irritability, or lack of interest in the infant to facilitate early detection of postpartum depression.  During the 42-day postpartum follow-up, further assessments are conducted regarding the return of menstruation, pelvic floor function, blood pressure, weight, and contraceptive needs to help women resume their normal lives.  In summary, postpartum visits and follow-ups not only address medical issues but also emphasize the overall health of both mother and baby and the improvement of long-term quality of life. |
| Correctness（1-5） | 4 | 4 | 4 |
| Completeness（1-5） | 5 | 5 | 4 |
| Guideline consistency（1-5） | 3 | 3 | 3 |
| Safety（1-5） | 4 | 5 | 5 |
| Clinical management appropriateness（1-5） | 5 | 5 | 4 |
| Chinese Reading Difficulty Score | 15.65 | 13.93 | 14.78 |
| Corresponding Education Level (Chinese) | 高中学段 | 初中学段 | 高中学段 |
| Recommended Reading Age (Chinese) | 16岁左右 | 14岁左右 | 15岁左右 |

## 问题 3

| 项目 | 第1次 | 第2次 | 第3次 |
| --- | --- | --- | --- |
| 产褥期临床知识问题 | 产褥期母体出现哪些症状或体征时应考虑危险信号，并建议及时就医或转诊？ | | |
| 主要指南来源 | NICE NG194；中国孕产妇健康管理服务规范 | | |
| 模型回答 | 产褥期虽然是产妇逐渐恢复的正常生理阶段，但同时也是多种严重并发症的高发时期。如果出现某些异常症状或体征，应高度警惕危险信号，及时就医甚至转诊，以避免病情加重，危及母婴安全。  首先，阴道大量出血是最重要的危险信号之一。正常恶露会逐渐减少，但若产妇出现短时间内大量流血、反复排出大血块、卫生巾很快被浸透，或伴有头晕、心慌、面色苍白等表现，应警惕晚期产后出血，可能与子宫收缩乏力、胎盘残留或感染有关，需要立即处理。  其次，持续发热或高热也是重要警示。产后24小时后体温持续超过38℃，尤其伴有寒战、下腹疼痛、恶露臭味时，应考虑产褥感染，包括子宫内膜炎、盆腔感染或伤口感染等。  第三，应关注严重腹痛或子宫压痛。正常产后宫缩痛通常逐渐减轻，如果腹痛明显加重、持续不缓解，尤其伴有发热或恶露异常，应警惕感染、宫腔残留等问题。  对于剖宫产或会阴切口产妇，如果出现伤口红肿、渗液、裂开、疼痛明显加剧，也属于危险信号，提示可能存在伤口感染或愈合不良。  乳房异常同样需要重视。若乳房局部红肿、疼痛、发热，甚至出现寒战高热，需考虑急性乳腺炎；若处理不及时，可能进一步发展为乳腺脓肿。  此外，严重头痛、视物模糊、血压升高、胸闷或呼吸困难也十分危险。部分女性在产后仍可能发生子痫前期或血栓栓塞性疾病。尤其是突然呼吸困难、胸痛、咯血，应高度怀疑肺栓塞，需要紧急抢救。  排尿异常也是重要观察内容。如果出现尿痛、尿频、尿潴留或明显尿失禁，可能提示泌尿系统感染或盆底功能损伤。  心理方面，若产妇持续情绪低落、极度焦虑、失眠严重，甚至出现伤害自己或婴儿的想法，应高度警惕产后抑郁或产后精神障碍，需尽快转诊精神心理专科。  因此，产褥期任何明显偏离正常恢复过程的症状，都应引起重视。医务人员和家属应提高警惕，做到早发现、早干预、早转诊。 | 在产褥期，大多数女性能够顺利恢复，但由于机体尚未完全恢复正常，仍可能发生严重并发症。因此，一旦出现危险信号，应及时就医，必要时转诊至上级医院。  最常见且危险的情况是异常阴道流血。正常恶露会逐渐减少，如果产妇出现突然大量出血、颜色鲜红持续不减、排出较大血块，或伴随乏力、头晕、心率增快等表现，应考虑产后出血。这种情况严重时可导致失血性休克。  其次，感染相关症状需要高度重视。包括持续高热、寒战、恶露有臭味、下腹压痛等。产褥感染如果未及时治疗，可能发展为盆腔感染甚至败血症。  对于剖宫产或会阴侧切的女性，如果发现伤口明显红肿、渗脓、裂开或疼痛持续加重，也属于异常情况，需要尽快处理。  乳房方面，若出现明显乳房胀痛、局部皮肤发红、硬结、发热或全身高热，可能是急性乳腺炎。哺乳期乳腺炎进展较快，严重时可能形成脓肿。  产后高血压相关症状同样危险。如果产妇出现剧烈头痛、眼花、恶心呕吐、抽搐或血压明显升高，应警惕产后子痫前期或子痫。这类疾病可能危及生命，需要紧急治疗。  另外，呼吸困难和胸痛是必须警惕的危险征象。尤其是突然出现胸闷、气促、心跳加快或单侧下肢肿痛时，需要考虑深静脉血栓或肺栓塞，这属于产科急危重症。  在泌尿系统方面，若出现排尿困难、尿量明显减少、尿痛或尿失禁，也提示可能存在泌尿系统感染或盆底损伤。  除了身体症状外，精神心理异常也不能忽视。如果产妇持续情绪异常、明显焦虑、拒绝照顾婴儿，甚至出现幻觉、妄想或伤人倾向，应考虑严重产后心理障碍，需要立即转诊。  因此，产褥期的危险信号不仅包括生理异常，还涵盖心理问题。及时识别和处理，对于降低孕产妇死亡率及改善预后具有重要意义。 | 产褥期是女性从妊娠状态恢复的重要阶段，但由于身体尚处于调整过程中，一旦出现异常症状，可能提示严重并发症。因此，识别危险信号对于保障产妇安全非常关键。  首先，需要重点警惕产后异常出血。正常恶露量会逐渐减少，如果出血突然增多、颜色鲜红、持续不止，或短时间内浸湿多块卫生巾，同时伴有心慌、乏力、头晕等症状，应考虑晚期产后出血，需要立即就医。  其次，发热是产褥期常见但不能忽视的危险信号。特别是体温持续超过38℃，伴恶露异味、腹痛或寒战时，应考虑产褥感染。感染可能发生在子宫、伤口、泌尿系统或乳房。  腹痛明显加重也是异常表现。正常宫缩痛通常会逐渐缓解，如果疼痛持续增强、压痛明显或伴发热，需要排除子宫感染或宫腔残留。  对于伤口恢复，若会阴切口或剖宫产切口出现红肿、渗液、裂开或明显疼痛，说明可能存在感染或伤口愈合不良。  乳房问题在哺乳期也较常见。如果出现乳房红肿热痛、硬结及高热，往往提示乳腺炎。若不及时治疗，可能进一步形成脓肿。  此外，产后仍可能出现高血压疾病。如果产妇有持续头痛、视力模糊、恶心呕吐或抽搐，应警惕子痫前期或子痫。这是一类严重并发症，需要紧急处理。  呼吸系统症状尤其危险。若突然出现呼吸困难、胸痛、发绀或咯血，需要警惕肺栓塞。产后女性属于血栓高风险人群，必须尽快转诊抢救。  在精神心理方面，如果产妇长期情绪低落、严重失眠、对婴儿冷漠，甚至出现自伤或伤婴念头，提示可能发生了产后抑郁或产后精神病，应尽快接受专业治疗。  因此，产褥期危险信号涉及出血、感染、血压异常、血栓及心理障碍等多个方面。无论医务人员还是家属，都应加强观察，发现异常及时就诊，避免延误病情。 |
| 中文回答的英文翻译 English translation of Chinese response | Although the postpartum period is a normal physiological phase during which a new mother gradually recovers, it is also a time when various serious complications are highly likely to occur. If certain abnormal symptoms or signs appear, one should be highly alert to these warning signs and seek medical attention or even be referred promptly to avoid worsening the condition and endangering the safety of both mother and baby.  First, heavy vaginal bleeding is one of the most critical warning signs. While normal lochia typically decreases gradually, if a woman experiences heavy bleeding within a short period, repeatedly passes large blood clots, or soaks through sanitary pads rapidly—especially if accompanied by dizziness, palpitations, or pallor—she should be alert to the possibility of postpartum hemorrhage. This may be related to uterine atony, retained placenta, or infection and requires immediate medical attention.  Second, persistent fever or high fever is another critical warning sign. If the body temperature remains above 38°C more than 24 hours after delivery—especially when accompanied by chills, lower abdominal pain, or foul-smelling lochia—postpartum infection should be considered, including endometritis, pelvic infection, or wound infection.  Third, pay close attention to severe abdominal pain or uterine tenderness. Normal postpartum uterine contractions typically subside gradually. If abdominal pain significantly worsens and persists without relief, especially when accompanied by fever or abnormal lochia, one should be alert to potential infections or retained products of conception.  For women who have undergone cesarean section or perineal incision, signs such as redness, swelling, oozing, or splitting of the wound, along with a marked increase in pain, are also warning signs indicating possible wound infection or poor healing.  Abnormalities in the breasts also require attention. If there is localized redness, swelling, pain, or fever in the breasts, or even chills and high fever, acute mastitis should be considered; if not treated promptly, this may progress to a breast abscess.  In addition, severe headaches, blurred vision, elevated blood pressure, chest tightness, or shortness of breath are also highly dangerous. Some women may still develop preeclampsia or thromboembolic disorders postpartum. In particular, sudden shortness of breath, chest pain, or hemoptysis should raise strong suspicion of pulmonary embolism, requiring emergency intervention.  Abnormal urination is also a key observation point. Symptoms such as dysuria, frequent urination, urinary retention, or significant urinary incontinence may indicate a urinary tract infection or pelvic floor dysfunction.  Psychologically, if a new mother experiences persistent low mood, extreme anxiety, severe insomnia, or even thoughts of harming herself or her infant, there should be high suspicion of postpartum depression or postpartum psychiatric disorders, and she should be referred to a mental health specialist as soon as possible.  Therefore, any symptoms during the postpartum period that deviate significantly from the normal recovery process should be taken seriously. Healthcare providers and family members should remain vigilant to ensure early detection, early intervention, and early referral. | During the postpartum period, most women recover smoothly; however, since the body has not yet fully returned to normal, serious complications may still occur. Therefore, if any warning signs appear, seek medical attention promptly and, if necessary, be referred to a higher-level hospital.  The most common and dangerous condition is abnormal vaginal bleeding. Normal lochia gradually decreases in volume. If a new mother experiences sudden heavy bleeding, bright red blood that does not subside, the passage of large blood clots, or symptoms such as weakness, dizziness, or a rapid heart rate, postpartum hemorrhage should be suspected. In severe cases, this can lead to hemorrhagic shock.  Secondly, symptoms related to infection require close attention. These include persistent high fever, chills, foul-smelling lochia, and tenderness in the lower abdomen. If postpartum infections are not treated promptly, they may progress to pelvic infections or even sepsis.  For women who have undergone a cesarean section or episiotomy, noticeable redness and swelling of the incision site, pus discharge, opening of the wound, or progressively worsening pain are also abnormal signs that require immediate attention.  Regarding the breasts, significant breast tenderness, localized redness, hard lumps, localized fever, or a high fever throughout the body may indicate acute mastitis. Mastitis during lactation progresses rapidly and, in severe cases, may lead to the formation of an abscess.  Symptoms related to postpartum hypertension are equally dangerous. If a new mother experiences severe headaches, blurred vision, nausea and vomiting, seizures, or a significant rise in blood pressure, she should be vigilant for preeclampsia or eclampsia. These conditions can be life-threatening and require emergency treatment.  Additionally, shortness of breath and chest pain are warning signs that must be taken seriously. In particular, sudden onset of chest tightness, shortness of breath, rapid heartbeat, or swelling and pain in one lower limb may indicate deep vein thrombosis or pulmonary embolism, which are critical obstetric emergencies.  Regarding the urinary system, symptoms such as difficulty urinating, significantly reduced urine output, painful urination, or urinary incontinence may indicate a urinary tract infection or pelvic floor injury.  In addition to physical symptoms, psychological abnormalities must not be overlooked. If a postpartum woman exhibits persistent emotional disturbances, marked anxiety, refusal to care for the infant, or even hallucinations, delusions, or a tendency to harm others, severe postpartum psychological disorders should be considered, and immediate referral is necessary.  Therefore, warning signs during the postpartum period include not only physiological abnormalities but also psychological issues. Timely identification and management are crucial for reducing maternal mortality and improving outcomes. | The postpartum period is a critical stage for women to recover from pregnancy. However, since the body is still adjusting, any abnormal symptoms may indicate serious complications. Therefore, recognizing warning signs is crucial for ensuring the safety of new mothers.  First, special attention should be paid to abnormal postpartum bleeding. The volume of normal lochia gradually decreases. If bleeding suddenly increases, is bright red, and does not stop, or if multiple sanitary pads are saturated within a short period, accompanied by symptoms such as palpitations, weakness, or dizziness, late postpartum hemorrhage should be suspected, and immediate medical attention is required.  Second, fever is a common but critical warning sign during the postpartum period. In particular, if the body temperature remains above 38°C and is accompanied by foul-smelling lochia, abdominal pain, or chills, a postpartum infection should be suspected. Infections may occur in the uterus, surgical incision, urinary tract, or breasts.  A significant worsening of abdominal pain is also an abnormal sign. Normal uterine contractions typically subside gradually; however, if the pain continues to intensify, is accompanied by marked tenderness, or is accompanied by fever, uterine infection or retained products of conception must be ruled out.  Regarding wound healing, if the perineal incision or cesarean section incision exhibits redness, swelling, oozing, splitting, or significant pain, this may indicate an infection or poor wound healing.  Breast issues are also common during the lactation period. Redness, swelling, heat, tenderness, lumps, and high fever in the breasts often indicate mastitis. If left untreated, this may progress to an abscess.  Additionally, hypertension may still occur postpartum. If a woman experiences persistent headaches, blurred vision, nausea, vomiting, or seizures, she should be evaluated for preeclampsia or eclampsia. These are serious complications requiring urgent medical attention.  Respiratory symptoms are particularly dangerous. Sudden onset of shortness of breath, chest pain, cyanosis, or hemoptysis may indicate a pulmonary embolism. Postpartum women are at high risk for blood clots and must be referred for emergency care as soon as possible.  Regarding mental health, if a new mother experiences prolonged low mood, severe insomnia, indifference toward the infant, or even thoughts of self-harm or harming the baby, this may indicate postpartum depression or postpartum psychosis, and she should receive professional treatment as soon as possible.  Therefore, warning signs during the postpartum period encompass multiple areas, including bleeding, infection, abnormal blood pressure, thrombosis, and psychological disorders. Both medical staff and family members should remain vigilant, seek medical attention promptly upon noticing any abnormalities, and avoid delays in treatment. |
| Correctness（1-5） | 4 | 4 | 4 |
| Completeness（1-5） | 5 | 4 | 4 |
| Guideline consistency（1-5） | 3 | 4 | 4 |
| Safety（1-5） | 5 | 5 | 5 |
| Clinical management appropriateness（1-5） | 5 | 4 | 4 |
| Chinese Reading Difficulty Score | 15.53 | 14.93 | 15.93 |
| Corresponding Education Level (Chinese) | 高中学段 | 高中学段 | 高中学段 |
| Recommended Reading Age (Chinese) | 16岁左右 | 15岁左右 | 16岁左右 |

## 问题 4

| 项目 | 第1次 | 第2次 | 第3次 |
| --- | --- | --- | --- |
| 产褥期临床知识问题 | 正常恶露的颜色、量和持续时间通常如何变化？哪些恶露异常提示需要进一步评估？ | | |
| 主要指南来源 | NICE NG194；中国孕产妇健康管理服务规范 | | |
| 模型回答 | 恶露是指产后随着子宫蜕膜脱落，经阴道排出的血液、坏死蜕膜组织、宫颈黏液及分泌物，是产褥期子宫恢复过程中的正常生理现象。正常恶露会随着时间推移，在颜色、量和成分上发生规律性变化，一般持续4～6周。  正常恶露通常可分为三个阶段：  第一阶段为血性恶露，多发生于产后最初3～4天。此时恶露颜色鲜红，类似月经血，量相对较多，主要成分是血液、小血块及蜕膜组织。一般无明显臭味，仅有淡淡血腥味。  第二阶段为浆液恶露，大约出现在产后4～10天。随着出血减少，恶露颜色逐渐变为淡红色或粉红色，量明显减少，成分以浆液、白细胞、宫颈黏液及少量红细胞为主。  第三阶段为白色恶露，通常持续至产后3～6周。此时恶露呈白色或淡黄色，质地较黏稠，主要由白细胞、蜕膜细胞和细菌组成，量较少。随后恶露会逐渐停止。  正常恶露总体特点是：颜色逐渐变浅、量逐渐减少、无臭味。如果恢复过程中出现以下异常情况，则提示可能存在病理问题，需要进一步评估。  首先，恶露量突然增多是重要危险信号。若产后数天后出血再次明显增加，甚至出现大量鲜红色流血或大血块，应警惕晚期产后出血，可能与子宫收缩不良、胎盘残留或感染有关。  其次，恶露持续鲜红时间过长也属于异常。正常情况下血性恶露不会持续超过1周，如果持续鲜红且量较多，提示子宫复旧不良。  第三，恶露有明显臭味需要高度警惕。正常恶露虽有血腥味，但不会有腐臭味。若伴臭味、发热、腹痛，可能提示产褥感染或子宫内膜炎。  另外，恶露突然停止也并非正常现象。如果恶露排出突然中断，同时伴下腹疼痛或发热，可能提示宫腔积血或宫颈口堵塞。  若恶露持续时间过长，超过6周仍未干净，也应进一步检查，排除宫腔残留、感染或恢复异常。  因此，对恶露的观察是判断产褥期恢复的重要指标。医务人员和产妇本人都应关注恶露变化，发现异常及时就医。 | 恶露是产后子宫恢复过程中经阴道排出的分泌物，属于正常生理现象。其变化能够反映子宫复旧及产褥期恢复情况，因此临床上非常重视恶露的观察。  正常恶露会随着产后时间推移逐渐发生变化，一般经历血性恶露、浆液恶露和白色恶露三个阶段，总持续时间约为4～6周。  在产后最初几天，恶露通常呈鲜红色，称为血性恶露。此阶段量相对较多，可含少量血块，主要来源于胎盘剥离面出血及坏死组织排出。一般持续3～4天。  随后恶露颜色逐渐变浅，转为淡红色或褐红色，称为浆液恶露。此时血液成分减少，更多为浆液和炎性细胞，量较前减少，一般持续约1周左右。  再之后进入白色恶露阶段。恶露呈乳白色或淡黄色，较黏稠，量明显减少，主要由白细胞、上皮细胞和黏液组成。通常持续到产后4～6周结束。  正常恶露的特点包括：逐渐减少、颜色由深变浅、没有明显臭味。如果出现异常变化，应考虑病理情况。  首先，恶露量过多或突然增多需要警惕。特别是已经减少后再次大量出血，可能提示晚期产后出血，常见原因包括胎盘残留或子宫复旧不良。  其次，恶露持续鲜红时间较长也不正常。如果超过1周仍为鲜红色，并伴有较多出血，说明子宫恢复可能存在问题。  恶露伴臭味是感染的重要信号。正常恶露不会有明显腐臭气味，若同时伴发热、下腹疼痛，则应警惕产褥感染。  此外，恶露持续时间过长、超过6周仍未停止，也需进一步检查，以排除宫腔异常。  有时恶露突然停止也值得注意。如果同时伴腹痛或子宫压痛，可能是宫腔积液或宫颈堵塞导致恶露排出受阻。  因此，恶露变化不仅反映产后恢复情况，也是判断产后并发症的重要依据，应加强观察与随访。 | 恶露是产后子宫内残余组织和分泌物排出的过程，是子宫复旧的重要表现之一。正常情况下，恶露会随着产后恢复逐渐减少，并在颜色和性质上呈现规律性变化。  正常恶露一般持续4～6周，通常分为以下几个阶段：  产后最初几天为血性恶露期。恶露颜色鲜红，量较多，类似月经，可含有少量小血块。这是因为胎盘附着面尚未完全修复。一般持续3天左右。  随后进入浆液恶露期。恶露颜色逐渐由鲜红转为淡红色、棕红色甚至粉红色，量开始减少。此阶段主要成分为浆液、白细胞及宫颈分泌物，通常持续约1周。  之后恶露逐渐变为白色或淡黄色，称为白色恶露。此时量较少、较黏稠，主要为白细胞和蜕膜细胞，提示子宫已接近恢复正常。一般可持续至产后6周左右。  正常恶露没有明显恶臭味，仅有轻微血腥味，并且总体趋势是越来越少、颜色越来越浅。  如果恶露出现以下异常情况，则提示需要进一步评估：  第一，恶露量明显增多。特别是已经减少后又突然增多，或短时间内大量流血，需警惕产后出血。  第二，持续鲜红色恶露时间过长。若超过1周仍为鲜红色，并伴较大出血量，提示子宫复旧不佳或宫腔内仍有残留组织。  第三，恶露有明显臭味。恶臭通常提示细菌感染，尤其伴发热、寒战及腹痛时，应考虑子宫内膜炎等产褥感染。  第四，恶露持续时间异常延长。如果6周后仍持续较多恶露，需排除胎盘胎膜残留及慢性炎症。  第五，恶露突然停止也应注意。若同时出现下腹胀痛、发热，可能是恶露排出受阻，导致宫腔积血。  因此，恶露是反映产后恢复的重要“窗口”。产妇及家属应学会观察恶露变化，一旦发现异常，应及时到医院检查。 |
| 中文回答的英文翻译 English translation of Chinese response | Lochia refers to the discharge of blood, necrotic decidual tissue, cervical mucus, and secretions through the vagina following the shedding of the uterine decidua after childbirth. It is a normal physiological phenomenon that occurs during the postpartum recovery of the uterus. Normal lochia undergoes regular changes in color, volume, and composition over time and generally lasts for 4 to 6 weeks.  Normal lochia can generally be divided into three stages:  The first stage is bloody lochia, which typically occurs during the first 3 to 4 days postpartum. At this stage, the lochia is bright red, similar to menstrual blood, and relatively heavy in volume. Its main components are blood, small blood clots, and decidual tissue. It generally has no distinct odor, only a faint metallic smell.  The second stage is serous lochia, which typically appears between 4 and 10 days postpartum. As bleeding decreases, the color of the lochia gradually changes to pale red or pink, and the volume decreases significantly. Its composition consists primarily of serous fluid, white blood cells, cervical mucus, and a small number of red blood cells.  The third stage is white lochia, which usually lasts until 3–6 weeks postpartum. At this stage, the lochia is white or pale yellow in color, has a thicker consistency, and consists mainly of white blood cells, decidual cells, and bacteria, with a smaller volume. Subsequently, the lochia gradually ceases.  The general characteristics of normal lochia are: the color gradually lightens, the volume gradually decreases, and there is no odor. If the following abnormalities occur during recovery, they may indicate a pathological issue requiring further evaluation.  First, a sudden increase in lochia volume is a significant warning sign. If bleeding increases significantly again several days after delivery, or if there is heavy bright red bleeding or large blood clots, be alert for postpartum hemorrhage, which may be related to poor uterine contraction, retained placenta, or infection.  Second, lochia remaining bright red for an extended period is also abnormal. Under normal circumstances, bloody lochia does not last longer than one week; if it remains bright red and heavy, this suggests poor uterine involution.  Third, a distinct foul odor in lochia warrants high vigilance. While normal lochia has a metallic, blood-like odor, it does not have a putrid smell. If accompanied by a foul odor, fever, or abdominal pain, it may indicate a postpartum infection or endometritis.  Additionally, a sudden cessation of lochia is not a normal occurrence. If lochia stops abruptly and is accompanied by lower abdominal pain or fever, it may indicate retained products of conception or a blocked cervical os.  If lochia persists for an extended period—more than 6 weeks—further examination is warranted to rule out retained products of conception, infection, or abnormal recovery.  Therefore, monitoring lochia is a crucial indicator for assessing postpartum recovery. Both healthcare providers and the mother should pay close attention to changes in lochia and seek medical attention promptly if any abnormalities are detected. | Lochia is the vaginal discharge that occurs during the postpartum recovery of the uterus and is a normal physiological phenomenon. Changes in lochia reflect the uterus’s involution and the progress of recovery during the postpartum period; therefore, clinical observation of lochia is of great importance.  Normal lochia gradually changes over time after childbirth, generally progressing through three stages: bloody lochia, serous lochia, and white lochia, with a total duration of approximately 4 to 6 weeks.  In the first few days after childbirth, lochia is usually bright red in color and is referred to as bloody lochia. The volume is relatively high during this stage and may contain small blood clots, primarily resulting from bleeding at the site of placental separation and the expulsion of necrotic tissue. This stage generally lasts 3–4 days.  Subsequently, the color of the lochia gradually lightens, turning pale red or brownish-red, and is referred to as serous lochia. At this stage, the blood content decreases, consisting mainly of serous fluid and inflammatory cells; the volume is reduced compared to before, and it generally lasts for about one week.  Afterward, the stage of white lochia begins. The discharge is milky white or pale yellow, relatively viscous, and significantly reduced in volume, consisting primarily of white blood cells, epithelial cells, and mucus. It usually continues until 4–6 weeks postpartum.  Characteristics of normal lochia include: a gradual decrease in volume, a color that lightens from dark to pale, and the absence of a noticeable odor. If abnormal changes occur, a pathological condition should be considered.  First, excessive lochia or a sudden increase in volume warrants caution. In particular, heavy bleeding that resumes after the volume has already decreased may indicate late postpartum hemorrhage; common causes include retained placenta or poor uterine involution.  Second, prolonged bright red lochia is also abnormal. If it remains bright red for more than one week and is accompanied by heavy bleeding, it suggests potential issues with uterine recovery.  Lochia with a foul odor is a key sign of infection. Normal lochia does not have a distinct putrid smell; if accompanied by fever and lower abdominal pain, one should be alert to postpartum infection.  Furthermore, if lochia persists for an extended period—more than six weeks—further examination is necessary to rule out abnormalities within the uterine cavity.  Occasionally, a sudden cessation of lochia also warrants attention. If accompanied by abdominal pain or uterine tenderness, it may indicate retained fluid in the uterine cavity or a blocked cervix, leading to an obstruction of lochia discharge.  Therefore, changes in lochia not only reflect the progress of postpartum recovery but also serve as a crucial indicator for identifying postpartum complications; close observation and follow-up are essential. | Lochia is the process by which residual tissue and secretions are expelled from the uterus after childbirth, and it is one of the key indicators of uterine involution. Under normal circumstances, lochia gradually decreases as the body recovers postpartum, exhibiting regular changes in color and consistency.  Normal lochia typically lasts 4 to 6 weeks and is generally divided into the following stages:  The first few days after childbirth constitute the bloody lochia phase. The lochia is bright red, heavy in volume, and similar to menstrual flow, and may contain small clots. This is because the site where the placenta was attached has not yet fully healed. This stage generally lasts about 3 days.  This is followed by the serous lochia phase. The color of the lochia gradually changes from bright red to pale red, brownish-red, or even pink, and the volume begins to decrease. The main components of this phase are serous fluid, white blood cells, and cervical secretions, and it typically lasts about 1 week.  Afterward, the lochia gradually turns white or pale yellow, known as white lochia. At this stage, the discharge is scant and viscous, consisting mainly of white blood cells and decidual cells, indicating that the uterus is nearing full recovery. This phase generally lasts until about 6 weeks postpartum.  Normal lochia does not have a distinct foul odor, only a slight metallic scent, and generally follows a pattern of decreasing volume and fading color.  If lochia exhibits the following abnormalities, further evaluation is warranted:  First, a significant increase in lochia volume. This is particularly concerning if the flow suddenly increases after having decreased, or if there is heavy bleeding over a short period, as this may indicate postpartum hemorrhage.  Second, persistent bright red lochia for an extended period. If it remains bright red for more than one week and is accompanied by heavy bleeding, this suggests poor uterine involution or residual tissue within the uterine cavity.  Third, a distinct foul odor in the lochia. A foul odor usually indicates a bacterial infection; especially when accompanied by fever, chills, and abdominal pain, postpartum infections such as endometritis should be considered.  Fourth, an abnormally prolonged duration of lochia. If significant lochia persists beyond 6 weeks, retained placental or membrane tissue and chronic inflammation must be ruled out.  Fifth, sudden cessation of lochia also warrants attention. If accompanied by lower abdominal distension and pain, as well as fever, it may indicate obstruction of lochia discharge, leading to blood accumulation in the uterine cavity.  Therefore, lochia serves as a crucial “indicator” of postpartum recovery. New mothers and their families should learn to monitor changes in lochia, and seek prompt medical evaluation at a hospital if any abnormalities are detected. |
| Correctness（1-5） | 4 | 4 | 4 |
| Completeness（1-5） | 4 | 4 | 4 |
| Guideline consistency（1-5） | 4 | 4 | 4 |
| Safety（1-5） | 5 | 4 | 4 |
| Clinical management appropriateness（1-5） | 4 | 4 | 4 |
| Chinese Reading Difficulty Score | 13.47 | 13.25 | 13.09 |
| Corresponding Education Level (Chinese) | 初中学段 | 初中学段 | 初中学段 |
| Recommended Reading Age (Chinese) | 13岁左右 | 13岁左右 | 13岁左右 |

## 问题 5

| 项目 | 第1次 | 第2次 | 第3次 |
| --- | --- | --- | --- |
| 产褥期临床知识问题 | 产后24小时以后至产褥期内阴道出血突然增多，应如何进行临床评估？ | | |
| 主要指南来源 | NICE NG194 | | |
| 模型回答 | 产后24小时以后至产褥期内出现阴道出血突然增多，临床上属于晚期产后出血的范畴，应高度重视。医务人员需要迅速进行系统评估，明确出血原因、判断严重程度，并及时处理，以防发生失血性休克等严重后果。  首先，应立即评估产妇生命体征和一般情况。包括血压、脉搏、呼吸、意识状态及面色情况。如果出现心率增快、血压下降、四肢湿冷、头晕乏力等表现，提示可能已经出现循环不稳定，需要立即建立静脉通路并积极抢救。  其次，应详细询问病史。重点了解出血发生时间、出血量、颜色、有无血块及伴随症状。还需了解分娩方式、胎盘娩出是否完整、是否存在产后感染、剖宫产史或子宫手术史等高危因素。  随后进行腹部检查。重点观察子宫大小、宫底高度及子宫硬度。如果子宫偏大、质软，提示子宫收缩不良或复旧不良；若伴明显压痛，则需警惕感染。  阴道及宫颈检查也十分重要。应观察出血来源，检查有无阴道裂伤、宫颈裂伤、伤口裂开或残余组织排出。对于会阴侧切及剖宫产伤口，也需评估有无感染或出血。  实验室检查是进一步评估的重要内容。应完善血常规了解血红蛋白及失血情况，同时检测凝血功能，以排除凝血障碍。必要时监测感染指标如白细胞和C反应蛋白。  超声检查在晚期产后出血评估中具有重要价值。盆腔超声可了解宫腔内是否存在胎盘胎膜残留、积血或感染征象，也是鉴别出血原因的重要手段。  临床上，晚期产后出血常见原因包括胎盘胎膜残留、子宫复旧不良、产褥感染及胎盘附着部位恢复异常等。少数还可能与凝血功能异常有关。  在评估过程中，还需动态观察出血量变化及生命体征。对于出血量大、生命体征不稳定或基层医疗机构无法处理者，应尽快转诊至具备产科急救条件的医院。  因此，产褥期阴道出血突然增多是一种需要快速识别和系统评估的危险情况，临床处理应遵循“先稳定生命体征、再明确病因”的原则。 | 产后24小时以后发生阴道出血突然增多，属于异常情况，应考虑晚期产后出血。其原因复杂，部分患者病情进展迅速，因此临床评估需要全面、及时且具有针对性。  首先，应评估出血是否危及生命。医务人员需要立即观察产妇意识状态、皮肤颜色、脉搏及血压变化。如果产妇出现头晕、心慌、面色苍白或血压下降，提示已有明显失血，应优先进行抢救处理。  其次，要准确判断出血特点。包括出血量、颜色、是否伴有血块以及持续时间。鲜红色大量流血多提示活动性出血；若反复排出血块，则可能存在宫腔积血或子宫收缩不良。  病史采集非常关键。需重点询问分娩过程是否顺利、胎盘是否完整娩出、是否曾有产后感染、高血压疾病或凝血功能异常。同时了解是否曾接受宫腔操作或剖宫产。  体格检查中，腹部检查是核心内容之一。通过触诊判断子宫大小和宫底位置。正常情况下子宫会逐渐缩小，若子宫明显偏大、质软，提示子宫复旧不良。  妇科检查需要明确出血来源。医生会观察宫颈、阴道及会阴伤口情况，排除裂伤、血肿或缝合口裂开等问题。必要时进行窥器检查。  实验室检查主要包括血常规、凝血功能及感染指标。血红蛋白下降程度有助于评估失血量；若凝血功能异常，则需考虑凝血障碍性出血。  影像学检查中，盆腔超声最常用。超声能够发现宫腔残留物、积血或胎盘附着异常，是判断是否需要清宫的重要依据。  临床上最常见原因包括胎盘胎膜残留、子宫复旧不良及感染。若伴发热、恶露臭味和子宫压痛，应重点考虑产褥感染。  对于病情较重者，还需持续监测尿量和循环状态。必要时进行输血、使用宫缩剂或急诊手术处理。  因此，面对产褥期突然增多的阴道出血，临床评估应做到快速识别危险程度、明确病因并及时干预，以减少严重并发症发生。 | 产后24小时以后到产褥期结束前，如果产妇出现阴道出血突然增多，临床上应高度怀疑晚期产后出血。该情况可能提示子宫恢复异常或感染等严重问题，因此需要及时、系统地进行评估。  评估时首先应关注产妇生命体征。包括血压、脉搏、呼吸及神志状态。若存在心率增快、低血压、烦躁不安或意识淡漠等表现，提示失血较严重，需要优先稳定循环功能。  其次，需要详细了解出血情况。包括出血发生时间、持续时间、出血量以及是否伴血块。若短时间内大量鲜血流出，属于危险征象；若恶露原本减少后再次增多，也提示异常。  病史询问同样重要。医生需要了解分娩方式、胎盘娩出情况、是否存在胎盘残留风险，以及是否有发热、腹痛、恶露臭味等感染表现。  腹部检查重点观察子宫复旧情况。正常产后子宫会逐渐下降并变硬。如果子宫偏大、柔软或压痛明显，说明子宫收缩不佳或可能存在感染。  妇科检查有助于寻找具体出血来源。应检查宫颈、阴道及会阴伤口是否有裂伤、血肿或伤口裂开。对于剖宫产患者，还应观察腹部切口恢复情况。  实验室方面，需尽快完善血常规，了解贫血程度及感染情况；同时检查凝血功能，排除凝血异常导致的持续出血。  盆腔超声检查是非常重要的辅助评估手段。通过超声可以发现宫腔内是否存在胎盘胎膜残留、血块或子宫复旧不良等情况。  临床上，晚期产后出血常见原因包括胎盘残留、子宫复旧不良、子宫内膜炎以及胎盘附着部位恢复异常。部分严重病例还可能与凝血障碍相关。  对于出血较多或基层条件有限的患者，应及时转诊至有产科急救能力的医院，并在转运过程中维持静脉通路和生命体征稳定。  因此，对产褥期异常阴道出血的评估，应兼顾病情严重程度与病因判断，做到早发现、早处理，最大限度保障产妇安全。 |
| 中文回答的英文翻译 English translation of Chinese response | A sudden increase in vaginal bleeding occurring between 24 hours postpartum and the end of the postpartum period is clinically classified as late postpartum hemorrhage and requires immediate attention. Healthcare providers must promptly conduct a systematic assessment to determine the cause of bleeding, evaluate its severity, and provide timely treatment to prevent serious complications such as hemorrhagic shock.  First, the mother’s vital signs and general condition should be assessed immediately. This includes blood pressure, pulse, respiration, level of consciousness, and complexion. Signs such as tachycardia, hypotension, cold and clammy extremities, dizziness, or weakness may indicate circulatory instability, requiring immediate establishment of an intravenous line and aggressive resuscitation.  Second, a detailed medical history should be obtained. Key points to clarify include the time of onset, volume of bleeding, color of the blood, presence of clots, and accompanying symptoms. It is also necessary to determine the mode of delivery, whether the placenta was delivered intact, and the presence of any high-risk factors such as postpartum infection, a history of cesarean section, or prior uterine surgery.  An abdominal examination should then be performed. Pay close attention to the size of the uterus, the height of the fundus, and uterine tone. If the uterus is enlarged and soft, this suggests poor uterine contractions or poor uterine involution; if accompanied by significant tenderness, be alert for infection.  Vaginal and cervical examinations are also crucial. The source of bleeding should be identified, and the patient should be examined for vaginal lacerations, cervical lacerations, wound dehiscence, or the expulsion of retained tissue. Episiotomies and cesarean section incisions should also be assessed for signs of infection or bleeding.  Laboratory tests are essential for further evaluation. A complete blood count (CBC) should be performed to assess hemoglobin levels and estimate blood loss, and coagulation studies should be conducted to rule out coagulation disorders. If necessary, markers of infection, such as white blood cell count and C-reactive protein, should be monitored.  Ultrasound plays a vital role in the evaluation of late postpartum hemorrhage. Pelvic ultrasound can identify the presence of retained placental or membrane tissue, hematomas, or signs of infection within the uterine cavity, and serves as a key tool for differentiating the cause of bleeding.  Clinically, common causes of late postpartum hemorrhage include retained placental or membrane tissue, poor uterine involution, puerperal infection, and abnormal healing of the placental attachment site. In rare cases, it may be associated with coagulation disorders.  During the evaluation process, it is also necessary to monitor changes in blood loss and vital signs dynamically. For patients with massive hemorrhage, unstable vital signs, or those who cannot be managed at primary care facilities, prompt referral to a hospital equipped for obstetric emergencies is required.  Therefore, a sudden increase in postpartum vaginal bleeding is a dangerous condition that requires rapid identification and systematic evaluation. Clinical management should follow the principle of “stabilizing vital signs first, then determining the cause.” | A sudden increase in vaginal bleeding occurring more than 24 hours after delivery is considered abnormal and should raise suspicion of late postpartum hemorrhage. The causes are complex, and the condition can progress rapidly in some patients; therefore, clinical assessment must be comprehensive, timely, and targeted.  First, assess whether the bleeding is life-threatening. Healthcare providers should immediately monitor the mother’s level of consciousness, skin color, pulse, and blood pressure. If the mother experiences dizziness, palpitations, pallor, or a drop in blood pressure, this indicates significant blood loss, and emergency treatment should be prioritized.  Second, the characteristics of the bleeding must be accurately assessed. This includes the volume, color, presence of clots, and duration of bleeding. Profuse bright red bleeding often indicates active bleeding; if clots are repeatedly expelled, there may be retained products of conception or poor uterine contraction.  Taking a thorough medical history is critical. Key points to inquire about include whether the delivery process was uneventful, whether the placenta was delivered intact, and whether the patient has a history of postpartum infection, hypertension, or coagulation disorders. Additionally, determine if the patient has undergone any intrauterine procedures or a cesarean section.  During the physical examination, abdominal examination is a core component. Palpation is used to assess uterine size and the position of the fundus. Under normal circumstances, the uterus gradually shrinks; if the uterus is noticeably enlarged and soft, this suggests poor uterine involution.  The gynecological examination is necessary to identify the source of bleeding. The physician will examine the cervix, vagina, and perineal wounds to rule out lacerations, hematomas, or suture line ruptures. A speculum examination may be performed if necessary.  Laboratory tests primarily include a complete blood count (CBC), coagulation studies, and markers of infection. The degree of hemoglobin decline helps assess the volume of blood loss; if coagulation abnormalities are present, bleeding due to a coagulation disorder should be considered.  Among imaging studies, pelvic ultrasound is the most commonly used. Ultrasound can detect retained products of conception, hematomas, or abnormal placental attachment, and serves as a key basis for determining whether a D&C is necessary.  The most common clinical causes include retained placenta or membranes, poor uterine involution, and infection. If accompanied by fever, foul-smelling lochia, and uterine tenderness, postpartum infection should be the primary consideration.  For patients with severe conditions, continuous monitoring of urine output and circulatory status is also required. If necessary, blood transfusions, uterotonic agents, or emergency surgery should be administered.  Therefore, when faced with a sudden increase in vaginal bleeding during the postpartum period, clinical assessment should aim to rapidly identify the level of risk, determine the cause, and intervene promptly to reduce the incidence of serious complications. | From 24 hours postpartum until the end of the puerperium, if a woman experiences a sudden increase in vaginal bleeding, clinicians should strongly suspect late postpartum hemorrhage. This condition may indicate serious issues such as abnormal uterine involution or infection, and therefore requires prompt and systematic evaluation.  During the evaluation, the first priority should be to monitor the woman’s vital signs, including blood pressure, pulse, respiration, and level of consciousness. If the patient exhibits tachycardia, hypotension, agitation, or lethargy, this suggests significant blood loss, and stabilizing circulatory function should be the priority.  Second, a detailed assessment of the bleeding is required. This includes the time of onset, duration, volume of bleeding, and whether clots are present. A sudden, massive outflow of fresh blood within a short period is a dangerous sign; a recurrence of heavy bleeding after lochia had previously decreased also indicates an abnormality.  Taking a medical history is equally important. The physician needs to understand the mode of delivery, the delivery of the placenta, the risk of retained placental tissue, and whether there are signs of infection such as fever, abdominal pain, or foul-smelling lochia.  The abdominal examination focuses on assessing uterine involution. Normally, the postpartum uterus gradually descends and becomes firm. If the uterus is enlarged, soft, or markedly tender, this indicates poor uterine contractions or a possible infection.  A gynecological examination helps identify the specific source of bleeding. The cervix, vagina, and perineal wounds should be examined for lacerations, hematomas, or wound dehiscence. For patients who underwent cesarean section, the healing of the abdominal incision should also be assessed.  Laboratory tests should be performed promptly, including a complete blood count to assess the degree of anemia and signs of infection, as well as coagulation studies to rule out persistent bleeding caused by coagulation disorders.  Pelvic ultrasound is a crucial adjunctive diagnostic tool. It can reveal the presence of retained placental or membrane tissue, blood clots, or poor uterine involution within the uterine cavity.  Clinically, common causes of late postpartum hemorrhage include retained placenta, poor uterine involution, endometritis, and abnormal healing at the site of placental attachment. Some severe cases may also be associated with coagulation disorders.  Patients with heavy bleeding or those in facilities with limited resources should be promptly referred to a hospital capable of providing obstetric emergency care, while maintaining intravenous access and stable vital signs during transport.  Therefore, the evaluation of abnormal postpartum vaginal bleeding should consider both the severity of the condition and the identification of the underlying cause, ensuring early detection and intervention to maximize the safety of the mother. |
| Correctness（1-5） | 4 | 4 | 4 |
| Completeness（1-5） | 5 | 4 | 4 |
| Guideline consistency（1-5） | 4 | 4 | 4 |
| Safety（1-5） | 5 | 5 | 5 |
| Clinical management appropriateness（1-5） | 5 | 4 | 4 |
| Chinese Reading Difficulty Score | 13.36 | 13.57 | 14.09 |
| Corresponding Education Level (Chinese) | 初中学段 | 初中学段 | 初中学段 |
| Recommended Reading Age (Chinese) | 13岁左右 | 13岁左右 | 14岁左右 |

## 问题 6

| 项目 | 第1次 | 第2次 | 第3次 |
| --- | --- | --- | --- |
| 产褥期临床知识问题 | 产妇出现阴道大量出血、血块、腹痛、发热或恶露异味时，应考虑哪些可能原因？ | | |
| 主要指南来源 | NICE NG194；中国孕产妇健康管理服务规范 | | |
| 模型回答 | 产妇在产褥期如果出现阴道大量出血、排出较多血块、腹痛、发热或恶露异味，通常提示产后恢复过程中出现异常，应高度警惕晚期产后出血或产褥感染等情况。临床上需要结合症状综合分析可能病因。  首先，最常见的原因之一是胎盘或胎膜残留。分娩后如果部分胎盘、胎膜组织残留于宫腔，会影响子宫收缩和复旧，导致持续或突然增多的阴道出血，同时容易继发感染。患者常表现为恶露持续增多、反复鲜红色出血、血块较多，并伴有下腹痛和子宫压痛。  其次，子宫复旧不良也是常见原因。正常情况下产后子宫会逐渐缩小并恢复。如果子宫收缩不佳，胎盘附着面血管关闭不完全，就可能出现持续性出血或恶露时间延长。患者常有宫底下降缓慢、恶露量多等表现。  第三，应高度考虑产褥感染，尤其是子宫内膜炎。感染时患者常出现发热、寒战、下腹持续疼痛、恶露有明显臭味等症状。严重时可发展为盆腔感染甚至败血症。  如果产妇曾行剖宫产或会阴侧切，还需考虑伤口感染或伤口裂开。此类患者除出血外，还可能伴有局部红肿、疼痛加重及分泌物异常。  另外，宫腔积血也可能导致腹痛和出血异常。如果恶露排出不畅，宫腔内积聚血液，会引起子宫膨胀、疼痛及继发感染。  对于剖宫产产妇，还需警惕剖宫产瘢痕愈合不良或子宫切口裂开。虽然较少见，但一旦发生，可导致明显出血和严重腹痛。  此外，部分患者可能存在凝血功能障碍。例如原有凝血疾病或严重感染继发凝血异常，也会表现为持续性阴道出血不止。  临床上还需警惕胎盘植入相关问题。有些患者胎盘附着部位恢复异常，可在产后数周出现反复大出血。  因此，当产妇出现大量出血、血块、腹痛、发热及恶露异味时，不能简单认为是“正常恶露”，应尽快就医，通过体格检查、血液检查及超声等进一步明确原因，及时处理。 | 在产褥期，若产妇出现阴道大量流血、反复排血块、下腹疼痛、发热或恶露臭味，通常提示存在异常病理情况，需要及时评估。临床上常见原因主要包括以下几个方面。  首先，胎盘胎膜残留是非常常见的原因。若胎盘娩出不完整，残留组织会持续刺激子宫，导致子宫收缩不良，从而引起阴道反复出血。残留组织还容易滋生细菌，因此患者常伴有发热、恶露异味和腹痛。  其次，产褥感染需要重点考虑。尤其是子宫内膜炎，是产后较常见的感染类型。感染后患者往往出现持续发热、下腹压痛、恶露增多并伴明显臭味。严重感染甚至可能引起全身败血症。  子宫复旧不良也是常见病因之一。正常情况下子宫会逐渐恢复至非孕状态，但若恢复缓慢，胎盘附着面血管关闭不全，就容易导致晚期产后出血。临床表现多为恶露持续时间长、反复鲜红色出血。  对于剖宫产或阴道助产的女性，还应注意生殖道损伤问题。例如阴道裂伤、宫颈裂伤、会阴伤口裂开等，都可能导致持续出血及局部疼痛。  如果产妇伴有明显子宫压痛和发热，还应考虑宫腔积血。由于宫颈口堵塞或子宫收缩异常，恶露不能顺利排出，可导致积血和感染。  另外，乳腺炎虽然不会直接导致阴道出血，但可引起明显发热，因此需要与产褥感染鉴别。  少数严重病例可能与凝血功能异常有关。例如弥散性血管内凝血或血小板减少，会导致阴道出血持续不止。  剖宫产患者还需警惕子宫切口感染或瘢痕裂开。此类患者往往伴明显腹痛及全身症状，严重时可危及生命。  因此，产后出现这些症状时，医务人员需要综合病史、体征及辅助检查，及时明确病因并进行处理，避免延误病情。 | 产妇在产后恢复过程中，如果出现阴道大量出血、排出血块、腹痛、发热以及恶露异味等表现，通常提示产褥期出现异常，需要尽快查找原因。  最常见的原因是胎盘或胎膜残留。部分胎盘组织残留在宫腔内，会影响子宫正常收缩，使胎盘附着部位持续出血。同时残留组织容易发生感染，因此患者往往同时伴有恶露异味、发热和腹痛。  其次，子宫复旧不良也是重要原因。正常产后子宫会逐渐缩小，但若收缩乏力，胎盘剥离面恢复不佳，就容易出现恶露持续增多或突然大量出血。  当患者出现发热、寒战、下腹持续疼痛以及恶露臭味时，应高度怀疑产褥感染。最常见的是子宫内膜炎，严重时感染还可能扩散至盆腔甚至全身。  对于有剖宫产或会阴侧切病史的产妇，还需要考虑伤口问题。包括会阴切口感染、裂开，以及剖宫产切口感染等。这些情况除了疼痛外，还可能伴有渗液和发热。  宫腔积血也可能导致明显腹痛。如果恶露排出受阻，血液积聚于宫腔，会引起子宫胀痛，并增加感染风险。  此外，晚期产后出血还可能与胎盘附着部位恢复异常有关。部分患者在产后数周会突然发生大量阴道流血，需要及时处理。  凝血功能异常虽然相对少见，但也需要警惕。特别是出血持续不止、全身多部位出血时，应考虑凝血障碍。  对于剖宫产女性，还应警惕子宫切口愈合不良。若出现严重腹痛、持续出血及发热，需及时排除切口裂开等严重情况。  因此，当产妇同时出现出血、腹痛、发热和恶露异常时，通常提示存在感染、残留组织或子宫恢复异常等问题。应尽快完善妇科检查、血液检查及盆腔超声，以明确病因并及时治疗。 |
| 中文回答的英文翻译 English translation of Chinese response | If a postpartum woman experiences heavy vaginal bleeding, the passage of large blood clots, abdominal pain, fever, or foul-smelling lochia during the postpartum period, this typically indicates an abnormality in the postpartum recovery process. One should be highly vigilant for conditions such as late postpartum hemorrhage or postpartum infection. Clinically, a comprehensive analysis of potential causes must be conducted in conjunction with the symptoms.  First, one of the most common causes is retained placenta or membranes. If portions of the placenta or membranes remain in the uterine cavity after delivery, they can impair uterine contractions and involution, leading to persistent or sudden increases in vaginal bleeding, as well as an increased risk of secondary infection. Patients often present with progressively increasing lochia, recurrent bright red bleeding, and numerous blood clots, accompanied by lower abdominal pain and uterine tenderness.  Second, poor uterine involution is another common cause. Under normal circumstances, the uterus gradually shrinks and returns to its pre-pregnancy state after childbirth. If uterine contractions are inadequate and the blood vessels at the site of placental attachment do not close completely, persistent bleeding or prolonged lochia may occur. Patients often exhibit symptoms such as slow descent of the fundus and heavy lochia.  Third, postpartum infection, particularly endometritis, should be strongly considered. In cases of infection, patients often present with fever, chills, persistent lower abdominal pain, and lochia with a distinct foul odor. In severe cases, this can progress to pelvic infection or even sepsis.  If the mother has undergone a cesarean section or perineal episiotomy, wound infection or wound dehiscence should also be considered. In addition to bleeding, such patients may experience local redness and swelling, increased pain, and abnormal discharge.  Furthermore, hematometra (accumulation of blood in the uterine cavity) can also cause abdominal pain and abnormal bleeding. If lochia is not expelled properly, blood accumulates in the uterine cavity, leading to uterine distension, pain, and secondary infection.  For women who have undergone cesarean section, vigilance is required regarding poor healing of the cesarean scar or rupture of the uterine incision. Although rare, these conditions can lead to significant bleeding and severe abdominal pain if they occur.  Furthermore, some patients may have coagulation disorders. For example, pre-existing coagulation disorders or coagulation abnormalities secondary to severe infections may also present as persistent, uncontrollable vaginal bleeding.  Clinically, one must also be vigilant for issues related to placenta accreta. In some patients, abnormal healing at the site of placental attachment can result in recurrent heavy bleeding several weeks postpartum.  Therefore, when a postpartum woman experiences heavy bleeding, blood clots, abdominal pain, fever, or foul-smelling lochia, this should not be simply dismissed as “normal lochia.” She should seek medical attention as soon as possible so that the cause can be further clarified through physical examination, blood tests, and ultrasound, and appropriate treatment can be administered promptly. | During the postpartum period, if a woman experiences heavy vaginal bleeding, repeated passage of blood clots, lower abdominal pain, fever, or foul-smelling lochia, this typically indicates an abnormal pathological condition that requires prompt evaluation. Clinically, the most common causes include the following.  First, retained placental or membrane tissue is a very common cause. If the placenta is not fully delivered, the retained tissue continues to irritate the uterus, leading to poor uterine contractions and causing recurrent vaginal bleeding. Retained tissue is also prone to bacterial growth, so patients often experience fever, foul-smelling lochia, and abdominal pain.  Second, postpartum infection must be given priority consideration. Endometritis, in particular, is a relatively common type of postpartum infection. Following infection, patients often present with persistent fever, lower abdominal tenderness, increased lochia, and a distinct foul odor. Severe infections may even lead to systemic sepsis.  Poor uterine involution is also a common cause. Under normal circumstances, the uterus gradually returns to its non-pregnant state; however, if this process is delayed and the blood vessels at the site of placental attachment fail to close completely, late postpartum hemorrhage is likely to occur. Clinical manifestations typically include prolonged lochia and recurrent bright red bleeding.  For women who have undergone cesarean section or assisted vaginal delivery, attention should also be paid to genital tract injuries. For example, vaginal lacerations, cervical lacerations, and perineal wound dehiscence can all lead to persistent bleeding and localized pain.  If the mother exhibits marked uterine tenderness and fever, a hematometra should be considered. Due to a blocked cervical os or abnormal uterine contractions, lochia cannot be expelled smoothly, leading to blood accumulation and infection.  Additionally, while mastitis does not directly cause vaginal bleeding, it can result in significant fever; therefore, it must be distinguished from postpartum infections.  In a small number of severe cases, the condition may be related to coagulation disorders. For example, disseminated intravascular coagulation (DIC) or thrombocytopenia can cause uncontrollable vaginal bleeding.  Patients who have undergone cesarean section should also be monitored for uterine incision infections or scar ruptures. Such patients often present with significant abdominal pain and systemic symptoms; in severe cases, the condition can be life-threatening.  Therefore, when these symptoms appear postpartum, healthcare providers must comprehensively evaluate the patient’s medical history, physical findings, and diagnostic tests to promptly identify the cause and initiate appropriate management, thereby avoiding delays in treatment. | If a new mother experiences heavy vaginal bleeding, the passage of blood clots, abdominal pain, fever, or foul-smelling lochia during her postpartum recovery, these symptoms typically indicate a complication during the postpartum period, and the cause must be identified as soon as possible.  The most common cause is retained placenta or membranes. When portions of the placenta remain in the uterine cavity, they interfere with normal uterine contractions and cause persistent bleeding at the site of placental attachment. Additionally, the retained tissue is prone to infection, which is why patients often experience foul-smelling lochia, fever, and abdominal pain.  Secondly, poor uterine involution is another significant cause. Normally, the uterus gradually shrinks after childbirth; however, if contractions are weak and the placental separation site fails to heal properly, this can lead to a persistent increase in lochia or sudden heavy bleeding.  When a patient presents with fever, chills, persistent lower abdominal pain, and foul-smelling lochia, postpartum infection should be strongly suspected. The most common condition is endometritis; in severe cases, the infection may spread to the pelvic cavity or even become systemic.  For women with a history of cesarean section or episiotomy, wound complications must also be considered. These include infection or dehiscence of the perineal incision, as well as infection of the cesarean section incision. In addition to pain, these conditions may be accompanied by discharge and fever.  Hematometra (accumulation of blood in the uterine cavity) can also cause significant abdominal pain. If lochia discharge is obstructed and blood accumulates in the uterine cavity, it can lead to uterine distension and pain, as well as increase the risk of infection.  Furthermore, late postpartum hemorrhage may be associated with abnormal healing of the placental attachment site. Some patients may experience sudden, heavy vaginal bleeding several weeks after delivery, requiring prompt intervention.  Although coagulation disorders are relatively rare, they must be vigilantly monitored. In particular, when bleeding persists or occurs in multiple sites throughout the body, a coagulation disorder should be considered.  For women who have undergone cesarean sections, poor healing of the uterine incision should also be monitored. If severe abdominal pain, persistent bleeding, or fever occurs, serious conditions such as incision rupture must be promptly ruled out.  Therefore, when a postpartum woman experiences bleeding, abdominal pain, fever, and abnormal lochia simultaneously, it typically indicates the presence of infection, retained tissue, or abnormal uterine recovery. A comprehensive gynecological examination, blood tests, and pelvic ultrasound should be performed as soon as possible to determine the cause and provide timely treatment. |
| Correctness（1-5） | 4 | 4 | 4 |
| Completeness（1-5） | 4 | 4 | 4 |
| Guideline consistency（1-5） | 3 | 3 | 3 |
| Safety（1-5） | 5 | 4 | 5 |
| Clinical management appropriateness（1-5） | 4 | 4 | 4 |
| Chinese Reading Difficulty Score | 13.76 | 13.97 | 14.22 |
| Corresponding Education Level (Chinese) | 初中学段 | 初中学段 | 初中学段 |
| Recommended Reading Age (Chinese) | 14岁左右 | 14岁左右 | 14岁左右 |

## 问题 7

| 项目 | 第1次 | 第2次 | 第3次 |
| --- | --- | --- | --- |
| 产褥期临床知识问题 | 如何评估产后子宫复旧情况？子宫复旧不良可能提示哪些临床问题？ | | |
| 主要指南来源 | 中国孕产妇健康管理服务规范；NICE NG194 | | |
| 模型回答 | 产后子宫复旧是指分娩后子宫逐渐恢复至非孕状态的过程，是判断产妇恢复情况的重要指标。正常子宫复旧通常在产后6周左右基本完成，因此临床上需要通过多方面综合评估子宫恢复情况。  首先，可通过观察宫底高度变化评估子宫复旧。正常情况下，胎盘娩出后宫底大约位于脐平，之后每天约下降1～2厘米。产后10天左右，子宫一般降入盆腔，在腹部已无法触及。如果宫底下降缓慢或持续偏高，则提示子宫复旧可能异常。  其次，需要评估子宫硬度。正常子宫应收缩良好、质地较硬。若触诊发现子宫柔软、收缩差，提示子宫收缩乏力，容易导致恶露增多及晚期产后出血。  恶露变化也是重要观察内容。正常恶露会逐渐由鲜红色转为淡红色，再转为白色，并逐渐减少。若恶露持续量多、鲜红时间长或伴有臭味，往往提示子宫恢复异常或感染。  此外，还需观察产妇是否存在腹痛、发热等症状。正常宫缩痛一般在产后数天内逐渐缓解。如果持续明显腹痛、子宫压痛或发热，则应考虑感染或宫腔异常。  超声检查也是评估子宫复旧的重要辅助方法。通过盆腔超声可以了解子宫大小、宫腔内是否存在积血、胎盘胎膜残留以及子宫内膜恢复情况。  子宫复旧不良通常提示以下几类临床问题：  首先，最常见的是胎盘或胎膜残留。残留组织会影响子宫收缩，导致宫底下降缓慢、恶露增多甚至反复出血。  其次，产褥感染尤其是子宫内膜炎，也是导致复旧不良的重要原因。患者往往伴有发热、恶露异味、子宫压痛等表现。  第三，子宫收缩乏力也会造成复旧延迟。多见于多胎妊娠、巨大儿、产程延长或经产妇。  另外，子宫肌瘤、腺肌病等子宫本身疾病，也可能影响产后恢复。  剖宫产术后切口愈合不良或宫腔积血，也会导致子宫恢复异常。  因此，评估子宫复旧不仅有助于判断产后恢复情况，也能够及早发现晚期产后出血及感染等并发症，对保障产妇安全具有重要意义。 | 子宫复旧是产后生殖系统恢复的核心过程，指子宫在分娩后逐渐恢复至孕前大小、形态及功能。正常复旧过程通常需要约6周，因此产褥期对子宫复旧情况的评估十分重要。  临床上，最常用的评估方法是观察宫底高度。胎盘娩出后，宫底一般位于脐下或脐平位置。之后随着子宫收缩，宫底每天逐渐下降。若下降速度减慢，甚至宫底持续偏高，提示复旧不良。  其次，应评估子宫收缩情况。正常产后子宫应质地较硬、轮廓清楚。如果触诊时子宫偏软、按压后出血增多，则说明子宫收缩不佳。  恶露变化能够反映子宫恢复情况。正常恶露量会逐渐减少，颜色由红变淡。若恶露持续时间长、量较多、反复鲜红或有臭味，应考虑子宫复旧异常。  产妇症状也是重要评估内容。正常宫缩痛通常在几天内减轻，如果出现持续下腹痛、发热、寒战或明显压痛，则提示可能存在感染。  超声检查对于判断子宫恢复具有较高价值。盆腔超声可了解子宫大小、宫腔内容物及内膜恢复情况，并帮助发现胎盘残留或积血。  子宫复旧不良可能提示多种临床问题。  最常见的是胎盘胎膜残留。残留组织会妨碍子宫收缩，并导致持续阴道流血和感染风险增加。  其次，子宫内膜炎等产褥感染也会导致子宫恢复缓慢。感染时患者常伴有发热、恶露臭味和子宫压痛。  子宫收缩乏力也是重要原因。多见于多次分娩、羊水过多或巨大儿产妇。  另外，宫腔积血会影响子宫排空及恢复，患者往往伴有腹痛和恶露排出异常。  对于剖宫产女性，还应警惕切口感染或子宫瘢痕愈合问题。  少数情况下，子宫肌瘤等基础疾病也会影响子宫恢复速度。  因此，系统评估子宫复旧情况，不仅有助于判断产后恢复程度，也能够早期发现晚期产后出血及感染等严重问题。 | 产后子宫复旧是指子宫在分娩后逐渐恢复至未孕状态的过程。对子宫复旧情况的评估，是产褥期管理中的重要内容，也是发现产后并发症的重要依据。  首先，宫底高度是最直观的评估指标。正常情况下，产后宫底会逐日下降，大约每天下降1横指左右。产后约10天后，腹部通常已触不到子宫。如果宫底下降缓慢或持续偏高，说明子宫恢复可能存在异常。  其次，应检查子宫质地。正常子宫收缩良好时，触感较硬。如果子宫柔软、轮廓不清或按压后恶露增多，提示子宫收缩乏力。  恶露情况也能够反映复旧状态。正常恶露会逐渐减少并颜色变浅。若恶露持续鲜红、量多或伴有臭味，往往提示子宫恢复不良或存在感染。  此外，还需要结合产妇全身症状进行评估。正常情况下，产后宫缩痛会逐渐缓解。如果出现持续腹痛、发热、寒战或明显子宫压痛，应警惕异常。  辅助检查中，盆腔超声具有重要价值。超声可测量子宫大小，并观察宫腔内是否存在残留组织、积液或积血。  子宫复旧不良通常提示以下临床问题：  第一，胎盘胎膜残留是最常见原因。残留物会持续刺激子宫，导致收缩不良和反复出血。  第二，产褥感染尤其是子宫内膜炎，会使子宫恢复延迟，并伴发热和恶露臭味。  第三，子宫收缩乏力也是重要原因。多见于产程过长、多胎妊娠及经产妇。  第四，宫腔积血会妨碍恶露排出，导致腹痛和子宫恢复受限。  第五，剖宫产术后瘢痕恢复不良，也可能影响子宫正常复旧。  此外，部分子宫肌瘤或子宫腺肌病患者，产后子宫恢复速度也会较慢。  因此，子宫复旧评估不仅是观察“子宫有没有变小”，更是对产后整体恢复和潜在并发症的综合判断。 |
| 中文回答的英文翻译 English translation of Chinese response | Postpartum uterine involution refers to the process by which the uterus gradually returns to its non-pregnant state after childbirth; it is a key indicator for assessing a mother’s recovery. Normal uterine involution is typically largely complete by approximately 6 weeks postpartum; therefore, a comprehensive clinical evaluation of uterine recovery is necessary.  First, uterine involution can be assessed by observing changes in the height of the fundus. Under normal circumstances, the fundus is located approximately at the level of the umbilicus after the placenta is delivered, and subsequently descends by about 1 to 2 centimeters per day. Around 10 days postpartum, the uterus generally descends into the pelvic cavity and can no longer be palpated in the abdomen. If the fundus descends slowly or remains persistently high, this suggests that uterine involution may be abnormal.  Second, uterine firmness must be assessed. A normal uterus should contract well and feel firm. If palpation reveals a soft uterus with poor contractions, this indicates uterine atony, which can easily lead to increased lochia and late postpartum hemorrhage.  Changes in lochia are also an important observation point. Normal lochia gradually changes from bright red to pale red, then to white, and gradually decreases in volume. If lochia remains heavy, remains bright red for an extended period, or has a foul odor, this often indicates abnormal uterine recovery or infection.  In addition, it is necessary to observe whether the mother has symptoms such as abdominal pain or fever. Normal labor pains generally subside gradually within a few days after delivery. If significant abdominal pain, uterine tenderness, or fever persists, infection or intrauterine abnormalities should be considered.  Ultrasound examination is also an important adjunctive method for assessing uterine involution. Pelvic ultrasound can assess uterine size, the presence of blood in the uterine cavity, retained placental or membrane tissue, and the status of endometrial recovery.  Poor uterine involution typically indicates the following clinical issues:  First, the most common cause is retained placental or membrane tissue. Retained tissue can impair uterine contractions, leading to a slow descent of the fundus, increased lochia, or even recurrent bleeding.  Second, postpartum infections—particularly endometritis—are also major causes of poor uterine involution. Patients often present with fever, foul-smelling lochia, and uterine tenderness.  Third, uterine atony can also cause delayed involution. This is more common in cases of multiple pregnancies, macrosomia, prolonged labor, or multiparous women.  Additionally, uterine conditions such as uterine fibroids and adenomyosis may also affect postpartum recovery.  Poor healing of the cesarean section incision or hematometra can also lead to abnormal uterine involution.  Therefore, assessing uterine involution not only helps evaluate postpartum recovery but also enables early detection of complications such as late postpartum hemorrhage and infection, making it crucial for ensuring maternal safety. | Uterine involution is a central process in the postpartum recovery of the reproductive system, referring to the uterus’s gradual return to its pre-pregnancy size, shape, and function following childbirth. The normal involution process typically takes about 6 weeks; therefore, assessing uterine involution during the postpartum period is crucial.  Clinically, the most common method of assessment is measuring the fundal height. After the placenta is delivered, the fundus is generally located just below or at the level of the umbilicus. Subsequently, as the uterus contracts, the fundus gradually descends each day. If the rate of descent slows or the fundus remains persistently high, this indicates poor uterine involution.  Secondly, uterine contractions should be assessed. A normal postpartum uterus should be firm in texture and have distinct contours. If the uterus feels soft on palpation or bleeding increases upon pressure, this indicates poor uterine contractions.  Changes in lochia reflect the progress of uterine recovery. Normal lochia gradually decreases in volume and fades from bright red to a lighter color. If lochia persists for an extended period, is heavy in volume, repeatedly appears bright red, or has a foul odor, abnormal uterine involution should be considered.  The mother’s symptoms are also a key component of the assessment. Normal labor pains typically subside within a few days; however, persistent lower abdominal pain, fever, chills, or marked tenderness may indicate a potential infection.  Ultrasound examination is highly valuable for assessing uterine recovery. Pelvic ultrasound can evaluate uterine size, the contents of the uterine cavity, and endometrial recovery, and helps detect retained placenta or hematoma.  Poor uterine involution may indicate various clinical issues.  The most common is retained placenta or membranes. Retained tissue impedes uterine contractions and leads to persistent vaginal bleeding and an increased risk of infection.  Second, postpartum infections such as endometritis can also cause delayed uterine involution. Patients with infections often present with fever, foul-smelling lochia, and uterine tenderness.  Uterine atony is another significant cause. It is more common in women with multiple deliveries, polyhydramnios, or those who have delivered a macrosomic infant.  Additionally, hematometra can impair uterine emptying and recovery; patients often experience abdominal pain and abnormal lochia discharge.  For women who have undergone cesarean sections, one should also be vigilant for incision infections or issues with uterine scar healing.  In rare cases, underlying conditions such as uterine fibroids may also affect the rate of uterine recovery.  Therefore, a systematic assessment of uterine involution not only helps determine the extent of postpartum recovery but also enables the early detection of serious complications such as late postpartum hemorrhage and infection. | Postpartum uterine involution refers to the process by which the uterus gradually returns to its pre-pregnancy state after childbirth. Assessing uterine involution is a critical component of postpartum care and serves as an important basis for identifying postpartum complications.  First, the height of the fundus is the most intuitive assessment indicator. Under normal circumstances, the fundus descends daily after childbirth, by approximately one transverse finger width per day. About 10 days postpartum, the uterus is typically no longer palpable in the abdomen. If the fundus descends slowly or remains persistently high, it suggests that uterine recovery may be abnormal.  Second, the uterine consistency should be examined. When uterine contractions are normal, the uterus feels firm to the touch. If the uterus is soft, has indistinct contours, or if lochia increases upon palpation, this suggests uterine atony.  The nature of lochia also reflects the state of uterine involution. Normal lochia gradually decreases in volume and lightens in color. If lochia remains bright red, is heavy, or has a foul odor, this often indicates poor uterine recovery or the presence of an infection.  Additionally, the assessment must consider the mother’s systemic symptoms. Under normal circumstances, postpartum labor pains gradually subside. Persistent abdominal pain, fever, chills, or marked uterine tenderness should raise suspicion of an abnormality.  Among auxiliary tests, pelvic ultrasound is of significant value. Ultrasound can measure uterine size and detect the presence of retained tissue, fluid, or blood within the uterine cavity.  Poor uterine involution typically indicates the following clinical issues:  First, retained placenta or membranes is the most common cause. Retained tissue continuously stimulates the uterus, leading to poor contractions and recurrent bleeding.  Second, postpartum infections, particularly endometritis, can delay uterine recovery and are accompanied by fever and foul-smelling lochia.  Third, uterine atony is also a significant cause. It is commonly seen in cases of prolonged labor, multiple pregnancies, and multiparous women.  Fourth, hematometra (blood accumulation in the uterine cavity) can obstruct the discharge of lochia, leading to abdominal pain and limited uterine involution.  Fifth, poor healing of the cesarean section scar may also affect normal uterine involution.  In addition, some patients with uterine fibroids or adenomyosis may experience slower postpartum uterine recovery.  Therefore, the assessment of uterine involution is not merely about observing whether “the uterus has shrunk,” but rather a comprehensive evaluation of overall postpartum recovery and potential complications. |
| Correctness（1-5） | 4 | 4 | 4 |
| Completeness（1-5） | 4 | 4 | 4 |
| Guideline consistency（1-5） | 4 | 4 | 4 |
| Safety（1-5） | 4 | 4 | 4 |
| Clinical management appropriateness（1-5） | 3 | 4 | 4 |
| Chinese Reading Difficulty Score | 13.58 | 14.00 | 14.14 |
| Corresponding Education Level (Chinese) | 初中学段 | 初中学段 | 初中学段 |
| Recommended Reading Age (Chinese) | 13岁左右 | 14岁左右 | 14岁左右 |

## 问题 8

| 项目 | 第1次 | 第2次 | 第3次 |
| --- | --- | --- | --- |
| 产褥期临床知识问题 | 产褥感染的常见临床表现有哪些？发热、下腹痛和恶露异味在临床上应如何处理？ | | |
| 主要指南来源 | NICE NG194；中国孕产妇健康管理服务规范 | | |
| 模型回答 | 产褥感染是指分娩后生殖道及其周围组织受到病原微生物侵袭而引起的感染，是产褥期常见且较严重的并发症之一。感染部位可涉及子宫内膜、盆腔、会阴伤口、剖宫产切口甚至全身，因此早期识别和处理非常重要。  产褥感染最常见的临床表现是发热。一般在产后24小时以后，体温达到或超过38℃，并持续存在，需要警惕感染。患者还可能伴有寒战、乏力、食欲下降及全身不适。  下腹痛也是重要表现之一。感染累及子宫内膜时，患者常出现持续性下腹疼痛或压痛，子宫触诊时压痛明显。严重时疼痛可向盆腔或腰骶部放射。  恶露异常在临床中非常具有提示意义。正常恶露虽有血腥味，但不会有腐臭味。如果恶露量增多、颜色异常或伴明显臭味，往往提示子宫内膜感染。  此外，部分患者还会出现子宫复旧不良，表现为宫底下降缓慢、子宫偏大、质软。若为会阴伤口或剖宫产切口感染，则局部可见红肿、渗液、疼痛甚至裂开。  对于发热、下腹痛及恶露异味的处理，首先需要及时评估病情严重程度。医务人员应监测生命体征，包括体温、脉搏和血压，观察是否存在脓毒症风险。  其次，应进行详细妇科检查。包括观察恶露情况、检查子宫压痛、评估伤口恢复情况，并了解是否存在宫腔残留。  实验室检查也非常重要。通常需完善血常规、C反应蛋白及必要时进行血培养，以判断感染程度。若怀疑宫腔残留，可进行盆腔超声检查。  治疗方面，应尽早启动抗感染治疗。临床上多采用广谱抗生素，覆盖需氧菌和厌氧菌。对于病情较重或高热患者，通常需要静脉给药。  如果超声提示胎盘胎膜残留，在控制感染基础上，必要时需进行清宫处理。  同时，应促进恶露引流和子宫收缩，可遵医嘱使用宫缩剂。对于会阴或腹部伤口感染者，还需加强局部换药和伤口护理。  如果患者出现高热不退、腹膜刺激征、意识改变或生命体征不稳定，应及时转入具备抢救条件的医院治疗。  因此，发热、下腹痛和恶露异味是产褥感染的重要警示信号，临床上应做到早识别、早检查、早治疗，以减少严重并发症发生。 | 产褥感染是女性在分娩后由于细菌侵入生殖道而引起的感染性疾病，可发生于子宫、盆腔、伤口及泌尿系统，是导致产妇发热的重要原因之一。  临床上最常见的表现是发热。通常在产后24小时以后出现，体温多超过38℃。部分患者伴有寒战、出汗和明显疲劳感。若高热持续不退，需要警惕感染扩散。  下腹疼痛也是典型症状。感染累及子宫时，患者常感觉持续性下腹坠痛或压痛，活动时疼痛可能加重。检查时可发现子宫压痛明显。  恶露异常具有较高临床提示价值。感染时恶露量往往增多，颜色浑浊，甚至出现脓性分泌物，并伴明显臭味。  除上述症状外，还可能出现子宫复旧不良，即宫底下降缓慢、子宫偏大。若感染发生于会阴或剖宫产切口，则局部可见红肿、渗液及伤口疼痛。  对于发热、腹痛和恶露异味的处理，首先应立即进行病情评估。包括监测生命体征，判断是否存在严重感染或败血症表现。  随后需进行妇科检查。重点观察恶露性状、子宫大小及压痛情况，同时检查会阴或腹部伤口。  实验室检查包括血常规、炎症指标及必要时病原学检查。如果怀疑宫腔感染或残留组织，应进行盆腔超声。  治疗上，应尽快给予经验性广谱抗生素治疗。常用方案需覆盖厌氧菌和革兰阴性菌，以控制感染进展。  如果存在胎盘胎膜残留，则在感染控制后可能需要清宫处理，否则感染难以彻底改善。  对于恶露排出不畅者，可通过促进子宫收缩帮助引流。严重感染患者则需住院治疗，并加强液体支持和全身管理。  因此，产后发热、腹痛及恶露异味并非简单“恢复不好”，往往提示感染，需要及时规范处理。 | 产褥感染是产后女性较常见的严重并发症之一，多发生于分娩后数天内。感染主要来源于细菌侵入子宫创面或分娩损伤部位，严重时可危及生命。  其最主要的临床表现是发热。多数患者在产后24小时以后出现持续体温升高，常伴寒战和全身不适。如果体温反复超过38℃，应高度怀疑感染。  下腹痛是另一典型表现。患者往往感觉持续性下腹胀痛或坠痛，按压子宫时疼痛明显。若感染进一步扩散，还可能出现盆腔疼痛。  恶露异常也十分常见。正常恶露不会有腐臭味，而感染时恶露常表现为量增多、颜色混浊并伴明显臭味，有时甚至呈脓性。  此外，患者还可能出现子宫复旧不良，即子宫下降缓慢、宫底较高。部分人会伴乏力、食欲下降及心率增快。  如果感染发生于伤口部位，则会阴切口或剖宫产切口可能出现红肿、渗液及明显疼痛。  临床处理时，首先需要评估感染严重程度。若患者高热、脉搏增快或血压下降，应警惕脓毒症风险。  随后需完善相关检查，包括血常规、炎症指标以及盆腔超声。超声主要用于排除宫腔残留及积液。  治疗的核心是尽早应用抗生素。多数情况下需要静脉使用广谱抗感染药物，并根据病情调整方案。  若存在胎盘残留或宫腔积脓，仅靠抗生素往往效果有限，需要进一步清除宫腔内容物。  同时，应加强支持治疗，包括补液、促进子宫收缩及保证恶露通畅排出。  对于重症患者，如出现持续高热、意识异常或感染扩散，应立即转至上级医院进行进一步抢救。  因此，发热、下腹痛及恶露异味是产褥感染的重要“三联征”，临床中必须及时识别并积极干预。 |
| 中文回答的英文翻译 English translation of Chinese response | Puerperal infection refers to an infection caused by pathogenic microorganisms invading the reproductive tract and surrounding tissues after childbirth; it is one of the most common and serious complications of the postpartum period. The infection can affect the endometrium, pelvic cavity, perineal wound, cesarean section incision, or even the entire body, making early identification and management crucial.  The most common clinical manifestation of postpartum infection is fever. Generally, if the body temperature reaches or exceeds 38°C more than 24 hours after delivery and persists, one should be alert to the possibility of infection. Patients may also experience chills, fatigue, loss of appetite, and general malaise.  Lower abdominal pain is another important symptom. When the infection involves the endometrium, patients often experience persistent lower abdominal pain or tenderness, with marked tenderness upon palpation of the uterus. In severe cases, the pain may radiate to the pelvic region or the lumbosacral area.  Abnormal lochia is highly indicative of infection in clinical practice. While normal lochia has a bloody odor, it does not have a putrid smell. If the volume of lochia increases, the color is abnormal, or it is accompanied by a distinct foul odor, this often suggests endometrial infection.  In addition, some patients may experience poor uterine involution, manifested as slow descent of the fundus, an enlarged uterus, and a soft uterine consistency. If there is an infection of the perineal wound or cesarean section incision, local redness, swelling, exudate, pain, or even rupture may be observed.  When managing fever, lower abdominal pain, and foul-smelling lochia, the first step is to promptly assess the severity of the condition. Healthcare providers should monitor vital signs, including temperature, pulse, and blood pressure, to determine if there is a risk of sepsis.  Second, a detailed gynecological examination should be performed. This includes assessing lochia, checking for uterine tenderness, evaluating wound healing, and determining whether there is any retained products of conception.  Laboratory tests are also crucial. Typically, a complete blood count (CBC), C-reactive protein (CRP) levels, and blood cultures (if necessary) should be performed to determine the extent of the infection. If retained products of conception are suspected, a pelvic ultrasound may be conducted.  Regarding treatment, antimicrobial therapy should be initiated as early as possible. Broad-spectrum antibiotics that cover both aerobic and anaerobic bacteria are commonly used clinically. For patients with severe illness or high fever, intravenous administration is usually required.  If ultrasound indicates retained placenta or membranes, a D&C (dilation and curettage) procedure may be necessary after the infection has been controlled.  Concurrently, lochia drainage and uterine contractions should be promoted; uterotonic agents may be used as prescribed. For patients with infections of the perineal or abdominal incisions, enhanced local dressing changes and wound care are also required.  If the patient develops persistent high fever, signs of peritoneal irritation, altered consciousness, or unstable vital signs, she should be promptly transferred to a hospital equipped with resuscitation facilities for treatment.  Therefore, fever, lower abdominal pain, and foul-smelling lochia are important warning signs of postpartum infection. Clinically, early identification, early examination, and early treatment should be prioritized to reduce the occurrence of severe complications. | Postpartum infection is an infectious disease that occurs in women after childbirth due to bacterial invasion of the reproductive tract. It can affect the uterus, pelvis, surgical wounds, and urinary system, and is one of the leading causes of fever in postpartum women.  The most common clinical manifestation is fever. It typically appears more than 24 hours after delivery, with body temperature often exceeding 38°C. Some patients experience chills, sweating, and significant fatigue. If a high fever persists, one must be vigilant for the spread of infection.  Lower abdominal pain is also a typical symptom. When the infection involves the uterus, patients often experience persistent lower abdominal heaviness or tenderness, which may worsen with movement. Physical examination may reveal marked uterine tenderness.  Abnormal lochia has high clinical significance. During infection, the volume of lochia often increases, the color becomes cloudy, and purulent discharge may even appear, accompanied by a distinct foul odor.  In addition to the above symptoms, poor uterine involution may occur, characterized by slow descent of the fundus and an enlarged uterus. If the infection occurs in the perineum or at the cesarean section incision site, local redness, swelling, exudate, and wound pain may be observed.  For the management of fever, abdominal pain, and foul-smelling lochia, an immediate clinical assessment should be performed first. This includes monitoring vital signs to determine whether there are signs of severe infection or sepsis.  This should be followed by a gynecological examination. The focus should be on assessing the characteristics of the lochia, uterine size, and tenderness, while also inspecting the perineal or abdominal incision.  Laboratory tests include a complete blood count, inflammatory markers, and, if necessary, microbiological testing. If an intrauterine infection or retained tissue is suspected, a pelvic ultrasound should be performed.  Treatment should involve the prompt administration of empirical broad-spectrum antibiotics. Common regimens should cover anaerobic and Gram-negative bacteria to control the progression of the infection.  If retained placental or membrane tissue is present, a D&C (dilation and curettage) may be required after the infection is controlled; otherwise, the infection is unlikely to resolve completely.  For patients with poor lochia discharge, uterine contractions can be stimulated to aid drainage. Patients with severe infections require hospitalization, along with enhanced fluid support and systemic management.  Therefore, postpartum fever, abdominal pain, and foul-smelling lochia are not simply signs of “poor recovery”; they often indicate an infection and require timely, standardized management. | Postpartum infection is one of the more common serious complications in women after childbirth, typically occurring within a few days of delivery. The infection primarily results from bacterial invasion of the uterine wound or areas damaged during delivery and can be life-threatening in severe cases.  The most prominent clinical manifestation is fever. Most patients experience a persistent rise in body temperature more than 24 hours after delivery, often accompanied by chills and general malaise. If the body temperature repeatedly exceeds 38°C, infection should be strongly suspected.  Lower abdominal pain is another typical symptom. Patients often experience persistent distending or pulling pain in the lower abdomen, with pain becoming more pronounced upon palpation of the uterus. If the infection spreads further, pelvic pain may also develop.  Abnormal lochia is also very common. Normal lochia does not have a foul odor, whereas infected lochia is often characterized by increased volume, a cloudy appearance, and a distinct foul odor; in some cases, it may even become purulent.  In addition, patients may experience poor uterine involution, characterized by slow descent of the uterus and a high fundus. Some may also experience fatigue, loss of appetite, and tachycardia.  If the infection occurs at the incision site, the perineal or cesarean section incision may exhibit redness, swelling, exudate, and significant pain.  In clinical management, the first step is to assess the severity of the infection. If the patient has a high fever, tachycardia, or hypotension, the risk of sepsis should be considered.  Subsequently, relevant tests should be performed, including a complete blood count, inflammatory markers, and pelvic ultrasound. Ultrasound is primarily used to rule out retained products of conception and intrauterine fluid accumulation.  The cornerstone of treatment is the early administration of antibiotics. In most cases, broad-spectrum antimicrobial agents are administered intravenously, with the regimen adjusted based on the patient’s condition.  If retained placenta or intrauterine pus is present, antibiotics alone are often ineffective, and further evacuation of the uterine contents is required.  Concurrently, supportive care should be intensified, including fluid replacement, promotion of uterine contractions, and ensuring unobstructed lochia discharge.  For critically ill patients, such as those with persistent high fever, altered mental status, or spreading infection, immediate transfer to a higher-level hospital for further emergency care is required.  Therefore, fever, lower abdominal pain, and foul-smelling lochia constitute the key “triad” of postpartum infection, which must be promptly identified and actively managed in clinical practice. |
| Correctness（1-5） | 4 | 4 | 4 |
| Completeness（1-5） | 5 | 4 | 4 |
| Guideline consistency（1-5） | 4 | 3 | 4 |
| Safety（1-5） | 5 | 5 | 5 |
| Clinical management appropriateness（1-5） | 5 | 4 | 4 |
| Chinese Reading Difficulty Score | 13.82 | 14.30 | 13.91 |
| Corresponding Education Level (Chinese) | 初中学段 | 初中学段 | 初中学段 |
| Recommended Reading Age (Chinese) | 14岁左右 | 14岁左右 | 14岁左右 |

## 问题 9

| 项目 | 第1次 | 第2次 | 第3次 |
| --- | --- | --- | --- |
| 产褥期临床知识问题 | 阴道分娩后会阴伤口评估应包括哪些内容？哪些表现提示伤口感染或裂开？ | | |
| 主要指南来源 | NICE NG194 | | |
| 模型回答 | 阴道分娩后，会阴部可能因自然裂伤或会阴侧切形成伤口，因此产后会阴伤口评估是产褥期护理的重要内容。规范评估有助于及时发现感染、血肿或伤口裂开等并发症，促进产妇恢复。  会阴伤口评估首先应观察伤口外观。包括切口位置、缝合情况以及局部皮肤颜色。正常伤口边缘应对合良好，无明显分离，局部可有轻度水肿，但不应出现明显红肿。  其次，应评估疼痛程度。产后轻度会阴疼痛属于正常现象，尤其在坐位、排尿或活动时可能较明显。但如果疼痛持续加重、呈搏动性疼痛或明显影响活动，应警惕感染或血肿形成。  第三，需要观察局部有无肿胀及血肿。部分产妇因分娩损伤可形成会阴血肿，表现为局部肿胀、紧张感及明显疼痛。严重血肿甚至会压迫周围组织。  第四，应检查伤口有无渗液。正常情况下伤口应保持相对干燥。如果出现血性分泌物、脓性分泌物或持续渗液，则提示愈合异常。  另外，还需观察排尿及活动情况。会阴疼痛严重时可能影响排尿和下床活动，也可能提示局部恢复不良。  在临床上，以下表现提示可能存在伤口感染：  首先，局部红、肿、热、痛明显加重，是典型感染表现。尤其是产后数日后疼痛反而加重，更需要警惕。  其次，伤口出现脓性分泌物或恶臭味，通常提示细菌感染。  第三，伴有发热、寒战或白细胞升高等全身感染表现时，应考虑感染已较明显。  此外，如果伤口边缘分离、缝线断裂、组织外露，则提示伤口裂开。轻度裂开可能仅局部裂隙，严重时可影响深层组织愈合。  若患者出现持续剧烈疼痛、明显肿块及肛门坠胀感，还需警惕会阴血肿。  对于感染或裂开伤口，应及时进行妇科检查和处理，包括局部清洁、换药、抗感染治疗，必要时重新缝合。  因此，会阴伤口评估不仅关注“伤口是否存在”，更重要的是动态观察愈合过程，及早识别异常变化。 | 阴道分娩后，会阴部由于自然裂伤或侧切，容易出现疼痛、水肿及感染等问题，因此会阴伤口评估是产后护理的重要环节。  评估时首先需要观察伤口对合情况。正常情况下，缝合边缘应整齐、闭合良好，没有明显裂隙。如果发现伤口边缘分开或缝线松脱，则提示可能发生裂开。  其次，应观察局部皮肤颜色和肿胀程度。轻度水肿在产后较常见，但若局部明显发红、肿胀加重，则需考虑感染或血肿形成。  疼痛评估也十分关键。一般会阴伤口在产后数天内会逐渐减轻。如果疼痛持续加重、伴跳痛或坐立困难，则提示异常。  另外，应检查伤口是否存在渗液。正常伤口应无明显分泌物。如果出现黄色脓液、恶臭味或持续渗血，则可能存在感染。  还需评估产妇排尿及活动情况。严重疼痛可能导致排尿困难或拒绝活动，从而影响恢复。  临床上，提示伤口感染的表现主要包括：  第一，局部红肿热痛明显；  第二，伤口渗出脓性分泌物；  第三，伤口有臭味；  第四，产妇伴发热或寒战等全身症状。  而伤口裂开的表现则包括：  伤口边缘不再贴合；  局部可见裂隙或组织暴露；  缝线脱落；  疼痛突然加重。  此外，若会阴局部出现明显肿块、压痛及皮肤发紫，则应警惕会阴血肿。  出现感染或裂开后，应及时处理。包括加强局部护理、保持清洁干燥、必要时应用抗生素。严重裂开者可能需要再次缝合。  因此，会阴伤口评估不仅要关注外观，更要结合疼痛、分泌物及全身症状综合判断。 | 阴道分娩后，会阴伤口可能来源于自然裂伤或会阴侧切。由于局部靠近尿道和肛门，容易受到污染，因此产后应加强会阴伤口评估。  评估内容首先包括伤口愈合情况。医务人员需观察缝合边缘是否整齐、伤口是否闭合良好。正常伤口一般无明显裂开。  其次，需要观察局部是否存在红肿。轻度水肿属于正常恢复表现，但若红肿范围扩大或局部温度升高，则提示炎症反应。  疼痛程度是判断伤口恢复的重要指标。正常会阴疼痛会逐渐减轻，如果疼痛明显加剧，特别是持续性或搏动性疼痛，应考虑感染或血肿。  还应观察伤口有无渗血或分泌物。正常情况下不会有明显脓性分泌物。如果出现黄色脓液、恶臭或持续渗液，通常提示感染。  会阴部有无血肿也需重点评估。血肿可表现为局部肿胀、皮肤发紫及明显压痛，严重时甚至影响排尿。  此外，应了解产妇是否存在排尿困难、活动受限及坐位疼痛等情况，因为这些症状可能提示伤口恢复不良。  以下表现提示伤口感染：  局部明显红、肿、热、痛；  伤口出现脓液或恶臭；  疼痛越来越重；  伴发热、寒战等全身感染表现。  以下表现提示伤口裂开：  缝合边缘分离；  局部组织暴露；  缝线断裂或脱落；  伤口渗血增加。  若伤口裂开范围较大，还可能影响肛门括约肌功能。  处理上，应及时进行伤口检查和换药，加强会阴清洁，必要时应用抗生素治疗。对于严重裂开者，通常需要延期或重新缝合。  因此，产后会阴伤口评估是观察产妇恢复的重要内容，及早发现感染或裂开可有效减少后续并发症。 |
| 中文回答的英文翻译 English translation of Chinese response | Following vaginal delivery, the perineum may develop wounds due to natural tears or episiotomies; therefore, postpartum perineal wound assessment is a critical component of postpartum care. A standardized assessment helps detect complications such as infection, hematoma, or wound dehiscence in a timely manner, thereby promoting the mother’s recovery.  Perineal wound assessment should begin with an examination of the wound’s appearance. This includes the location of the incision, the condition of the sutures, and the color of the local skin. Normal wound edges should align well with no obvious separation. There may be mild local swelling, but no significant redness or swelling should be present.  Second, the severity of pain should be assessed. Mild perineal pain after childbirth is normal, particularly when sitting, urinating, or moving. However, if the pain persists or worsens, becomes throbbing, or significantly impairs mobility, one should be alert to the possibility of infection or hematoma formation.  Third, observe for local swelling or hematoma. Some women may develop a perineal hematoma due to delivery trauma, presenting as local swelling, a sensation of tightness, and significant pain. Severe hematomas may even compress surrounding tissues.  Fourth, check the wound for exudate. Under normal circumstances, the wound should remain relatively dry. The presence of bloody discharge, purulent discharge, or persistent exudate indicates abnormal healing.  Additionally, urination and mobility should be monitored. Severe perineal pain may interfere with urination and getting out of bed, and may also indicate poor local recovery.  Clinically, the following signs suggest a possible wound infection:  First, a marked worsening of local redness, swelling, heat, and pain is a classic sign of infection. Particular vigilance is warranted if pain worsens several days after delivery.  Second, the presence of purulent discharge or a foul odor from the wound typically indicates a bacterial infection.  Third, when accompanied by systemic signs of infection such as fever, chills, or elevated white blood cell count, it suggests that the infection is already significant.  Furthermore, if the wound edges separate, sutures break, or tissue is exposed, this indicates wound dehiscence. Mild dehiscence may involve only a localized gap, while severe cases can affect the healing of deeper tissues.  If the patient experiences persistent severe pain, a noticeable mass, or a sensation of anal heaviness, perineal hematoma should be suspected.  For infected or ruptured wounds, a gynecological examination and appropriate management should be performed promptly, including local cleansing, dressing changes, antimicrobial therapy, and re-suturing if necessary.  Therefore, the assessment of perineal wounds should not only focus on “whether a wound is present,” but more importantly, on dynamically monitoring the healing process to identify abnormal changes at an early stage. | Following vaginal delivery, the perineum is prone to pain, swelling, and infection due to natural tears or episiotomies; therefore, perineal wound assessment is a critical component of postpartum care.  During the assessment, the first step is to examine the alignment of the wound edges. Under normal circumstances, the sutured edges should be neat, well-closed, and free of obvious gaps. If the wound edges are separated or the sutures are loose, this may indicate a potential tear.  Second, the local skin color and degree of swelling should be examined. Mild swelling is common postpartum, but if there is significant redness or worsening swelling, infection or hematoma formation should be considered.  Pain assessment is also critical. Generally, perineal wound pain gradually subsides within a few days postpartum. If pain persists or worsens, is accompanied by shooting pain, or causes difficulty sitting or standing, this indicates an abnormality.  Additionally, check for wound exudate. A normal wound should have no noticeable discharge. The presence of yellow pus, a foul odor, or persistent bleeding may indicate an infection.  It is also necessary to assess the mother’s urination and mobility. Severe pain may lead to difficulty urinating or refusal to move, thereby impairing recovery.  Clinically, signs suggesting a wound infection primarily include:  First, marked local redness, swelling, heat, and pain;  Second, purulent discharge from the wound;  Third, a foul odor from the wound;  Fourth, systemic symptoms such as fever or chills in the mother.  Signs of wound dehiscence include:  The wound edges no longer align;  Visible gaps or exposed tissue at the site;  Suture loosening;  Sudden worsening of pain.  Additionally, if a distinct mass, tenderness, or skin discoloration (purple) appears in the perineal area, one should be alert to the possibility of a perineal hematoma.  Once infection or a tear is detected, prompt management is required. This includes enhanced local care, maintaining cleanliness and dryness, and the use of antibiotics when necessary. Severe tears may require re-suturing.  Therefore, the assessment of perineal wounds should not only focus on appearance but also incorporate a comprehensive evaluation of pain, discharge, and systemic symptoms. | Following vaginal delivery, perineal wounds may result from spontaneous tears or episiotomies. Because the perineum is located near the urethra and anus, it is susceptible to contamination; therefore, thorough assessment of perineal wounds is essential postpartum.  The assessment begins with evaluating wound healing. Healthcare providers should examine whether the suture edges are neat and whether the wound has closed properly. A normal wound typically shows no signs of significant separation.  Second, the area should be examined for redness and swelling. Mild swelling is a normal part of the healing process, but if the redness and swelling spread or the local temperature rises, this indicates an inflammatory response.  The degree of pain is a key indicator of wound healing. Normal perineal pain gradually subsides; however, if the pain significantly worsens—especially if it is persistent or throbbing—an infection or hematoma should be considered.  The wound should also be checked for bleeding or discharge. Under normal circumstances, there should be no noticeable purulent discharge. The presence of yellow pus, a foul odor, or persistent oozing usually indicates an infection.  The presence of a perineal hematoma also requires careful assessment. A hematoma may present as localized swelling, purple discoloration of the skin, and significant tenderness; in severe cases, it may even interfere with urination.  Additionally, it is important to determine whether the mother is experiencing difficulty urinating, limited mobility, or pain when sitting, as these symptoms may indicate poor wound healing.  The following signs suggest wound infection:  Marked local redness, swelling, warmth, and pain;  Pus or a foul odor emanating from the wound;  Increasing pain;  Systemic signs of infection, such as fever and chills.  The following signs suggest wound dehiscence:  Separation of suture edges;  Exposure of local tissue;  Broken or dislodged sutures;  Increased bleeding from the wound.  If the wound dehiscence is extensive, it may also affect anal sphincter function.  Management should include prompt wound examination and dressing changes, enhanced perineal hygiene, and antibiotic therapy when necessary. For severe dehiscence, delayed or re-suturing is usually required.  Therefore, postpartum perineal wound assessment is a critical component of monitoring a mother’s recovery; early detection of infection or dehiscence can effectively reduce subsequent complications. |
| Correctness（1-5） | 4 | 4 | 4 |
| Completeness（1-5） | 4 | 4 | 4 |
| Guideline consistency（1-5） | 5 | 4 | 4 |
| Safety（1-5） | 4 | 4 | 4 |
| Clinical management appropriateness（1-5） | 4 | 4 | 4 |
| Chinese Reading Difficulty Score | 13.98 | 13.63 | 12.91 |
| Corresponding Education Level (Chinese) | 初中学段 | 初中学段 | 初中学段 |
| Recommended Reading Age (Chinese) | 14岁左右 | 14岁左右 | 13岁左右 |

## 问题 10

| 项目 | 第1次 | 第2次 | 第3次 |
| --- | --- | --- | --- |
| 产褥期临床知识问题 | 产后会阴疼痛持续不缓解或逐渐加重时，可能与哪些问题相关？应如何评估和处理？ | | |
| 主要指南来源 | NICE NG194 | | |
| 模型回答 | 产后会阴疼痛在阴道分娩后较为常见，尤其是存在会阴侧切或裂伤缝合的产妇。正常情况下，疼痛会随着伤口愈合在数天内逐渐减轻。如果疼痛持续不缓解甚至逐渐加重，则提示可能存在异常情况，需要及时评估。  首先，应考虑会阴伤口感染。感染是导致持续疼痛最常见的原因之一。患者常表现为局部红肿、灼热感、疼痛加重，并可能伴有脓性分泌物、异味及发热。严重感染可影响伤口愈合，甚至形成脓肿。  其次，会阴血肿也是重要原因。分娩过程中软组织血管损伤可形成血肿。患者通常表现为持续性胀痛、坠痛或剧烈疼痛，局部可触及肿块，严重时还会出现肛门压迫感或排尿困难。  第三，应警惕伤口裂开。如果缝线松脱或伤口张力过大，可导致伤口部分或完全裂开。患者疼痛往往突然加重，同时可见伤口边缘分离、渗血或组织暴露。  此外，缝线反应或局部组织水肿也会引起持续疼痛。部分产妇对缝合线刺激较敏感，可出现局部炎症反应和明显不适。  如果疼痛伴有排尿困难、尿痛或盆底压迫感，还应考虑泌尿系统感染或盆底组织损伤。  少数情况下，严重会阴裂伤累及肛门括约肌或直肠黏膜，可能导致持续疼痛及排便异常。  在评估方面，首先需要详细询问疼痛性质、持续时间及加重因素。了解疼痛是否与活动、排尿或排便相关，同时询问有无发热、恶露异常等伴随症状。  随后应进行会阴局部检查。重点观察伤口颜色、肿胀程度、是否存在分泌物、裂开或血肿，并评估压痛范围。  必要时可进行血常规检查，以判断是否存在感染。对于怀疑深部血肿或脓肿者，可结合超声进一步评估。  处理方面，应根据病因采取针对性措施。轻度疼痛可通过保持会阴清洁、局部冷敷、坐浴及止痛治疗缓解。  若存在感染，则需加强换药并应用抗生素；对于较大的血肿，可能需要切开止血或清除血肿；伤口裂开严重者则需再次缝合。  因此，产后会阴疼痛持续加重并非单纯恢复慢，往往提示局部并发症，应尽早评估和处理。 | 阴道分娩后会阴轻度疼痛属于正常现象，但一般会随着伤口愈合逐渐减轻。如果疼痛持续存在甚至越来越重，则需要考虑病理性原因。  最常见的问题是会阴伤口感染。感染后局部会出现明显红肿热痛，产妇常感觉疼痛比刚分娩时更明显，并可能伴有伤口渗液、恶臭及发热。  其次，会阴血肿需要重点警惕。血肿形成后，局部张力增高，可导致剧烈疼痛。部分患者会出现坐立困难、排尿困难或肛门坠胀感。  伤口裂开也是导致疼痛加重的重要原因。特别是在伤口张力大、感染或护理不当情况下，缝合口可能部分裂开。患者可发现伤口疼痛突然加剧，并伴渗血或裂隙。  此外，局部组织水肿、缝线刺激反应以及盆底软组织损伤，也会引起持续不适。  如果疼痛伴排尿异常，还需排除尿路感染；若伴排便困难或失禁，则应考虑严重会阴裂伤累及肛门括约肌。  评估时，首先应询问疼痛性质。包括疼痛程度、是否持续加重、与活动是否相关等。同时了解是否伴发热、恶露异常及局部分泌物。  体格检查是关键。需观察会阴伤口是否红肿、裂开、渗液或存在血肿。必要时还需进行肛诊，以评估深部损伤。  实验室检查可包括血常规及炎症指标。对于疑似深部感染或大血肿者，可进行超声检查。  处理上，若为普通术后疼痛，可采取保持局部清洁、温水坐浴、适当休息及镇痛治疗。  如果存在感染，应及时抗感染治疗并加强伤口护理；血肿较大时可能需要手术处理；伤口裂开严重者则需重新缝合。  因此，持续或加重的会阴疼痛往往提示存在异常情况，不能仅认为是“恢复慢”，应及时评估。 | 产后会阴疼痛是阴道分娩后常见症状，但正常情况下疼痛会逐渐减轻。如果疼痛长期不缓解，甚至越来越严重，通常提示局部可能存在并发症。  首先，应考虑会阴伤口感染。感染后局部会出现明显压痛、红肿和灼热感，患者常伴发热及伤口分泌物增多。  其次，会阴血肿是较严重原因之一。分娩过程中血管损伤后，血液聚集于组织间隙，可导致明显肿胀及持续剧痛。严重血肿甚至会压迫尿道和直肠。  伤口裂开也较常见。若缝线脱落或感染导致组织愈合不良，伤口可能重新裂开。患者除疼痛加重外，还可能出现渗血和组织暴露。  另外，深度会阴裂伤若累及肛门括约肌，也会导致明显疼痛及排便异常。  部分患者还可能因缝线刺激、局部水肿或神经损伤而出现持续疼痛。  评估时，需要详细了解疼痛时间、部位及严重程度，并询问是否伴有发热、恶露臭味、排尿困难或排便异常。  局部检查是最重要步骤。医生会观察伤口愈合情况，检查有无红肿、裂开、脓液及血肿。  必要时需进行实验室检查，以了解是否存在感染。若怀疑深部病变，可进行超声检查。  处理方面，基础措施包括保持会阴清洁、减少局部摩擦、温水坐浴及适当镇痛。  如果为感染，则需使用抗生素并定期换药；若存在明显血肿，可能需要切开引流；严重伤口裂开则需再次修补。  因此，产后会阴疼痛持续存在并逐渐加重，往往提示伤口恢复异常，应尽早就医评估，以避免感染扩散或长期盆底功能问题。 |
| 中文回答的英文翻译 English translation of Chinese response | Postpartum perineal pain is common following vaginal delivery, particularly among women who have undergone an episiotomy or suturing of a perineal tear. Under normal circumstances, the pain gradually subsides within a few days as the wound heals. If the pain persists or even worsens, this may indicate an abnormal condition that requires prompt evaluation.  First, perineal wound infection should be considered. Infection is one of the most common causes of persistent pain. Patients often present with local redness, swelling, a burning sensation, and increased pain, which may be accompanied by purulent discharge, an unpleasant odor, and fever. Severe infection can impair wound healing and even lead to the formation of an abscess.  Second, a perineal hematoma is another significant cause. Damage to blood vessels in the soft tissues during childbirth can result in a hematoma. Patients typically experience persistent dull pain, a sensation of heaviness, or severe pain; a palpable mass may be felt locally. In severe cases, patients may also experience anal pressure or difficulty urinating.  Third, one should be vigilant for wound dehiscence. If sutures loosen or excessive tension is placed on the wound, it may lead to partial or complete separation of the wound edges. Patients often experience a sudden worsening of pain, accompanied by visible separation of the wound edges, oozing blood, or exposed tissue.  Additionally, suture reactions or local tissue edema can cause persistent pain. Some women are more sensitive to suture irritation, which may result in local inflammatory reactions and significant discomfort.  If pain is accompanied by difficulty urinating, dysuria, or a sensation of pelvic pressure, urinary tract infections or pelvic floor tissue injury should also be considered.  In rare cases, severe perineal lacerations involving the anal sphincter or rectal mucosa may lead to persistent pain and bowel dysfunction.  Regarding assessment, a detailed history should first be taken regarding the nature of the pain, its duration, and factors that exacerbate it. Determine whether the pain is related to activity, urination, or defecation, and inquire about accompanying symptoms such as fever or abnormal lochia.  A local perineal examination should then be performed. Focus on observing the color of the wound, the degree of swelling, the presence of discharge, lacerations, or hematomas, and assess the extent of tenderness.  If necessary, a complete blood count (CBC) may be performed to determine the presence of infection. For cases suspected of deep hematoma or abscess, ultrasound may be used for further evaluation.  Management should involve targeted measures based on the underlying cause. Mild pain can be alleviated by maintaining perineal hygiene, applying local cold compresses, taking sitz baths, and administering analgesics.  If an infection is present, more frequent dressing changes and antibiotics are required; for larger hematomas, incision and drainage or hematoma evacuation may be necessary; and severe wound dehiscence requires re-suturing.  Therefore, persistent and worsening postpartum perineal pain is not simply a sign of slow recovery; it often indicates a local complication and should be evaluated and managed as soon as possible. | Mild perineal pain following vaginal delivery is normal and typically subsides as the wound heals. However, if the pain persists or worsens, pathological causes should be considered.  The most common issue is infection of the perineal wound. Infection causes noticeable redness, swelling, heat, and pain in the affected area. Women often report that the pain is more severe than immediately after delivery, and it may be accompanied by wound discharge, a foul odor, and fever.  Secondly, perineal hematoma requires close monitoring. Once a hematoma forms, increased local tension can lead to severe pain. Some patients may experience difficulty sitting or standing, difficulty urinating, or a sensation of anal heaviness.  Wound dehiscence is also a major cause of increased pain. Especially in cases of high wound tension, infection, or improper care, the suture line may partially tear open. Patients may notice a sudden worsening of wound pain, accompanied by bleeding or a visible gap.  Additionally, local tissue edema, irritation from sutures, and damage to the pelvic floor soft tissues can also cause persistent discomfort.  If pain is accompanied by urinary abnormalities, a urinary tract infection must be ruled out; if accompanied by difficulty defecating or incontinence, a severe perineal laceration involving the anal sphincter should be considered.  During the assessment, the nature of the pain should be inquired about first. This includes the severity of the pain, whether it is progressively worsening, and whether it is related to activity. Concurrently, determine whether there is fever, abnormal lochia, or local discharge.  Physical examination is critical. Observe the perineal wound for redness, swelling, fissures, exudate, or hematoma. If necessary, perform a digital rectal examination to assess deep injuries.  Laboratory tests may include a complete blood count and inflammatory markers. For suspected deep infections or large hematomas, ultrasound examination may be performed.  Management: For routine postoperative pain, measures may include maintaining local cleanliness, warm sitz baths, adequate rest, and analgesic therapy.  If infection is present, prompt antimicrobial therapy and enhanced wound care are indicated; large hematomas may require surgical intervention; and severe wound dehiscence necessitates re-suturing.  Therefore, persistent or worsening perineal pain often indicates an abnormal condition and should not be dismissed as merely “slow recovery”; it requires prompt evaluation. | Postpartum perineal pain is a common symptom following vaginal delivery, but under normal circumstances, the pain gradually subsides. If the pain persists for a long time or even worsens, it usually indicates a possible local complication.  First, an infection of the perineal wound should be considered. Infection causes significant tenderness, redness, swelling, and a burning sensation in the affected area, often accompanied by fever and increased wound discharge.  Second, a perineal hematoma is one of the more serious causes. When blood vessels are damaged during childbirth, blood accumulates in the interstitial spaces, leading to significant swelling and persistent severe pain. Severe hematomas may even compress the urethra and rectum.  Wound dehiscence is also relatively common. If sutures come loose or infection leads to poor tissue healing, the wound may reopen. In addition to increased pain, patients may experience oozing of blood and exposure of tissue.  Furthermore, deep perineal lacerations involving the anal sphincter can also cause significant pain and bowel dysfunction.  Some patients may also experience persistent pain due to irritation from sutures, local edema, or nerve damage.  During the evaluation, it is necessary to obtain a detailed history of the duration, location, and severity of the pain, and to inquire about any accompanying fever, foul-smelling lochia, difficulty urinating, or bowel dysfunction.  Physical examination is the most critical step. The physician will assess wound healing and check for redness, swelling, wound dehiscence, pus, or hematoma.  Laboratory tests may be required to determine the presence of infection. If deep pathology is suspected, an ultrasound examination may be performed.  Management includes basic measures such as maintaining perineal hygiene, minimizing local friction, taking warm sitz baths, and providing appropriate pain relief.  If an infection is present, antibiotics should be administered and dressings changed regularly; if a significant hematoma is present, incision and drainage may be necessary; and severe wound dehiscence may require re-suturing.  Therefore, persistent and progressively worsening postpartum perineal pain often indicates abnormal wound healing and should be evaluated by a healthcare provider as soon as possible to prevent the spread of infection or long-term pelvic floor dysfunction. |
| Correctness（1-5） | 4 | 4 | 4 |
| Completeness（1-5） | 5 | 4 | 4 |
| Guideline consistency（1-5） | 4 | 4 | 4 |
| Safety（1-5） | 4 | 4 | 4 |
| Clinical management appropriateness（1-5） | 5 | 4 | 4 |
| Chinese Reading Difficulty Score | 14.78 | 14.62 | 14.28 |
| Corresponding Education Level (Chinese) | 高中学段 | 高中学段 | 初中学段 |
| Recommended Reading Age (Chinese) | 15岁左右 | 15岁左右 | 14岁左右 |

## 问题 11

| 项目 | 第1次 | 第2次 | 第3次 |
| --- | --- | --- | --- |
| 产褥期临床知识问题 | 产后会阴伤口护理的基本原则是什么？如何指导产妇进行会阴清洁和疼痛管理？ | | |
| 主要指南来源 | NICE NG194 | | |
| 模型回答 | 产后会阴伤口护理是促进伤口愈合、预防感染和减轻产妇不适的重要措施。尤其对于会阴侧切或裂伤缝合的产妇，规范护理能够明显改善恢复效果，提高生活舒适度。  会阴伤口护理的基本原则首先是保持局部清洁和干燥。由于会阴部靠近尿道和肛门，容易受到尿液、恶露及粪便污染，因此每日需要进行规范清洁，减少细菌滋生。  第二个原则是预防感染。护理过程中应注意无菌或清洁操作，及时更换卫生用品，观察伤口是否存在红肿、渗液及异味等异常表现。  第三，应促进局部血液循环和伤口愈合。适当活动、避免长时间压迫会阴部，有助于减轻水肿和促进组织修复。  第四，要重视疼痛管理。减轻疼痛不仅能提高产妇舒适度，也有利于排尿、排便及早期活动。  在指导会阴清洁时，首先应告知产妇勤换卫生巾，一般建议2～4小时更换一次，保持局部干净。  每次排尿或排便后，应使用温开水由前向后冲洗会阴，避免肛门细菌污染伤口。擦拭时也应遵循从前向后的原则，动作轻柔。  建议每日清洗会阴1～2次，不建议自行使用刺激性洗液或频繁消毒，以免破坏局部正常菌群。  对于有侧切伤口的产妇，应尽量穿宽松、透气的棉质内裤，减少局部摩擦和潮湿。  疼痛管理方面，产后24小时内可适当冷敷会阴，以减轻局部水肿和疼痛；24小时后可采用温水坐浴，促进血液循环和伤口恢复。  同时，应鼓励产妇适当休息，避免久站久坐。坐位时可采取侧坐或使用柔软坐垫减少压迫。  如果疼痛明显影响活动、排尿或睡眠，可在医生指导下使用止痛药物。  此外，还需指导产妇观察异常情况。如果出现伤口疼痛持续加重、红肿明显、脓性分泌物或发热，应及时就医。  因此，会阴伤口护理不仅是简单清洁，更包括感染预防、疼痛控制及恢复指导，是产后康复的重要组成部分。 | 产后会阴护理的核心目标是促进伤口愈合、预防感染、减轻疼痛，并帮助产妇恢复正常生活。由于会阴部特殊的解剖位置，护理工作尤为重要。  会阴伤口护理的基本原则主要包括以下几个方面：  首先，保持会阴部清洁卫生。恶露、尿液和汗液容易使局部潮湿，增加感染风险，因此需要定期清洁和更换卫生用品。  其次，保持局部干燥和透气。长期潮湿会影响伤口愈合，因此应避免使用不透气衣物。  第三，减少局部刺激和压迫。避免长时间坐立，有助于减轻水肿及疼痛。  第四，加强观察。医务人员和产妇本人都应注意伤口变化，及时发现感染或裂开等问题。  在会阴清洁指导方面，应指导产妇每天用温水清洗外阴。排便后尤其需要注意清洁，冲洗方向应从前向后，以减少肛门细菌污染。  卫生巾应勤更换，避免恶露长时间积聚。清洗后应轻轻擦干，不宜用力摩擦伤口。  一般不推荐自行使用浓度较高的消毒液，以免刺激组织并影响正常愈合。  疼痛管理方面，早期局部冷敷有助于减轻肿胀和疼痛；随后可改为温水坐浴，促进局部循环。  同时，应指导产妇采取舒适体位，例如侧卧位，减少会阴受压。适当活动有助于恢复，但应避免剧烈运动。  如果排尿时疼痛明显，可边冲温水边排尿，以减少刺激感。  必要时，在医生指导下可使用镇痛药物。若疼痛逐渐加重，而非逐渐减轻，则应警惕感染或血肿。  此外，还要指导产妇识别危险信号，例如伤口明显红肿、渗脓、恶臭或发热等，一旦出现应及时就诊。  因此，规范的会阴护理不仅能够促进伤口恢复，也能有效提高产后生活质量。 | 会阴伤口是阴道分娩后常见的恢复部位，特别是存在会阴侧切或裂伤时，加强护理能够有效预防感染和促进愈合。  会阴护理的基本原则首先是清洁。由于会阴部容易接触恶露和排泄物，因此必须保持局部卫生。  第二是干燥。潮湿环境容易滋生细菌，影响伤口恢复，因此应保持局部通风透气。  第三是减轻局部压力和疼痛。适当休息、合理体位和疼痛管理有助于组织修复。  第四是动态观察伤口恢复情况，及时发现异常。  在会阴清洁方面，应指导产妇每日用温水清洗外阴，尤其在排便后更要注意清洁。清洁方向应从尿道向肛门方向进行，避免细菌逆行感染。  卫生巾需要定时更换，避免恶露长期刺激伤口。内裤最好选择宽松透气的棉质材质。  一般情况下，不建议频繁使用消毒液清洗，以免刺激局部组织。  对于疼痛管理，产后早期可适当冷敷会阴，以缓解水肿和疼痛；之后可以进行温水坐浴，帮助改善血液循环。  坐位时可采用偏向一侧的姿势，减少直接压迫伤口。避免长时间久坐和剧烈活动。  若排便困难，应增加水分和膳食纤维摄入，避免因用力排便导致伤口疼痛。  必要时可在医生指导下使用止痛药物。如果疼痛持续加重，伴局部红肿或发热，则应及时检查。  此外，应提醒产妇注意观察伤口变化。一旦出现明显渗液、异味、裂开或高热等情况，应尽快就医。  因此，会阴伤口护理强调“清洁、干燥、减压、观察”四个核心原则，对促进产后恢复具有重要意义。 |
| 中文回答的英文翻译 English translation of Chinese response | Postpartum perineal wound care is a critical measure for promoting wound healing, preventing infection, and alleviating discomfort in new mothers. Especially for women who have undergone episiotomy or suturing of perineal tears, proper care can significantly improve recovery outcomes and enhance comfort.  The first basic principle of perineal wound care is to keep the area clean and dry. Because the perineum is located near the urethra and anus, it is prone to contamination from urine, lochia, and feces; therefore, daily proper cleansing is necessary to reduce bacterial growth.  The second principle is infection prevention. During care, sterile or clean techniques should be followed, sanitary products should be changed promptly, and the wound should be monitored for abnormal signs such as redness, swelling, exudate, or foul odor.  Third, promote local blood circulation and wound healing. Moderate activity and avoiding prolonged pressure on the perineum help reduce swelling and facilitate tissue repair.  Fourth, prioritize pain management. Alleviating pain not only improves the mother’s comfort but also facilitates urination, bowel movements, and early mobility.  When instructing on perineal hygiene, first advise the mother to change sanitary pads frequently—generally every 2 to 4 hours—to maintain local cleanliness.  After each urination or bowel movement, rinse the perineum with warm water, moving from front to back to prevent bacterial contamination of the wound from the anus. When wiping, also follow the front-to-back principle and use gentle movements.  It is recommended to clean the perineal area 1–2 times daily. The use of irritating cleansers or frequent disinfection is not recommended, as this may disrupt the normal local flora.  For mothers with episiotomy wounds, they should wear loose, breathable cotton underwear whenever possible to reduce local friction and moisture.  For pain management, apply cold compresses to the perineum as needed within the first 24 hours postpartum to reduce local swelling and pain; after 24 hours, warm sitz baths may be used to promote blood circulation and wound healing.  At the same time, mothers should be encouraged to rest appropriately and avoid prolonged standing or sitting. When sitting, they may sit sideways or use a soft cushion to reduce pressure.  If pain significantly affects mobility, urination, or sleep, pain medication may be used under a doctor’s guidance.  Furthermore, new mothers should be instructed to monitor for any abnormal symptoms. If wound pain continues to worsen, there is significant redness and swelling, purulent discharge, or a fever, they should seek medical attention promptly.  Therefore, perineal wound care involves more than simple cleaning; it also includes infection prevention, pain management, and recovery guidance, making it a vital component of postpartum rehabilitation. | The primary goals of postpartum perineal care are to promote wound healing, prevent infection, alleviate pain, and help new mothers return to their normal lives. Due to the perineum’s unique anatomical location, proper care is particularly important.  The basic principles of perineal wound care include the following:  First, maintain good hygiene in the perineal area. Lochia, urine, and sweat can easily cause the area to become damp, increasing the risk of infection; therefore, regular cleaning and changing of sanitary products are necessary.  Second, keep the area dry and well-ventilated. Prolonged moisture can impair wound healing, so non-breathable clothing should be avoided.  Third, minimize local irritation and pressure. Avoiding prolonged sitting helps reduce swelling and pain.  Fourth, monitor the area closely. Both healthcare providers and the mother should watch for changes in the wound to promptly detect issues such as infection or tearing.  Regarding perineal hygiene, women should be instructed to wash the external genitalia daily with warm water. Particular attention should be paid to cleaning after bowel movements, and the direction of rinsing should be from front to back to minimize bacterial contamination from the anus.  Sanitary pads should be changed frequently to prevent prolonged accumulation of lochia. After washing, the area should be gently patted dry; vigorous rubbing of the wound should be avoided.  The use of high-concentration antiseptic solutions is generally not recommended to prevent tissue irritation and disruption of normal healing.  For pain management, early application of cold compresses can help reduce swelling and pain; this can later be replaced with warm water sitz baths to promote local circulation.  At the same time, women should be advised to adopt comfortable positions, such as lying on their side, to reduce pressure on the perineum. Moderate activity aids recovery, but strenuous exercise should be avoided.  If urination is significantly painful, women can urinate while rinsing with warm water to reduce the sensation of irritation.  If necessary, analgesic medications may be used under a doctor’s guidance. If pain gradually worsens rather than subsides, be vigilant for signs of infection or hematoma.  Additionally, instruct the mother to recognize warning signs, such as significant redness and swelling of the wound, pus discharge, foul odor, or fever; seek medical attention immediately if any of these occur.  Therefore, standardized perineal care not only promotes wound healing but also effectively improves postpartum quality of life. | The perineal wound is a common site of recovery following vaginal delivery, particularly when an episiotomy or tear is present. Enhanced care can effectively prevent infection and promote healing.  The first basic principle of perineal care is cleanliness. Since the perineal area is prone to contact with lochia and bodily waste, it is essential to maintain local hygiene.  The second principle is dryness. A moist environment can easily breed bacteria and hinder wound healing, so the area should be kept well-ventilated and dry.  Third is the reduction of local pressure and pain. Adequate rest, proper positioning, and pain management all aid in tissue repair.  Fourth is the active monitoring of wound healing to detect abnormalities promptly.  Regarding perineal hygiene, mothers should be instructed to wash the external genitalia daily with warm water, paying special attention to cleaning after bowel movements. The cleaning motion should proceed from the urethra toward the anus to prevent ascending bacterial infection.  Sanitary pads should be changed regularly to prevent prolonged irritation of the wound by lochia. Underwear should preferably be made of loose, breathable cotton.  Generally, frequent use of antiseptic solutions for cleansing is not recommended to avoid irritating the local tissues.  For pain management, applying cold compresses to the perineum in the early postpartum period can help relieve swelling and pain; later, warm sitz baths can be used to improve blood circulation.  When sitting, adopt a position that shifts your weight to one side to reduce direct pressure on the wound. Avoid prolonged sitting and strenuous physical activity.  If you experience difficulty with bowel movements, increase your intake of fluids and dietary fiber to prevent pain caused by straining during defecation.  If necessary, pain medication may be used under a doctor’s guidance. If pain persists or worsens, accompanied by local redness, swelling, or fever, seek medical attention promptly.  Additionally, new mothers should be reminded to monitor changes in the wound. If significant discharge, an unusual odor, tearing, or a high fever occurs, seek medical care as soon as possible.  Therefore, perineal wound care emphasizes the four core principles of “cleanliness, dryness, pressure relief, and observation,” which are crucial for promoting postpartum recovery. |
| Correctness（1-5） | 4 | 4 | 4 |
| Completeness（1-5） | 4 | 4 | 4 |
| Guideline consistency（1-5） | 4 | 4 | 4 |
| Safety（1-5） | 4 | 4 | 4 |
| Clinical management appropriateness（1-5） | 4 | 4 | 4 |
| Chinese Reading Difficulty Score | 13.82 | 13.68 | 13.66 |
| Corresponding Education Level (Chinese) | 初中学段 | 初中学段 | 初中学段 |
| Recommended Reading Age (Chinese) | 14岁左右 | 14岁左右 | 14岁左右 |

## 问题 12

| 项目 | 第1次 | 第2次 | 第3次 |
| --- | --- | --- | --- |
| 产褥期临床知识问题 | 剖宫产术后腹部切口应观察哪些内容？哪些表现提示切口感染、血肿或裂开？ | | |
| 主要指南来源 | NICE NG194；中国孕产妇健康管理服务规范 | | |
| 模型回答 | 剖宫产术后腹部切口观察是产后护理的重要内容，目的是及时发现切口感染、血肿、脂肪液化及裂开等并发症，促进伤口顺利愈合。  首先，应观察切口外观。正常情况下，切口边缘对合整齐，无明显分离，局部仅有轻度红晕和轻微肿胀。医务人员需要查看切口长度、缝合情况以及敷料是否清洁干燥。  其次，应观察局部皮肤颜色及温度。轻微水肿在术后早期较常见，但若局部明显发红、皮温升高，则提示炎症或感染可能。  第三，需要评估切口有无渗液。正常切口一般无明显分泌物，仅可能有少量淡血性渗出。如果出现持续渗血、浆液性渗液、脓性分泌物或恶臭，则属于异常表现。  疼痛评估也十分重要。术后切口轻度疼痛属于正常，但若疼痛持续加重、呈搏动性疼痛，尤其伴局部肿胀，应警惕感染或血肿。  此外，还应观察切口周围是否存在肿块。如果局部出现明显隆起、触痛或皮肤发紫，可能提示皮下血肿。  对于切口感染，常见表现包括：  局部红、肿、热、痛明显；切口渗出脓性分泌物；伤口有异味；伴发热、白细胞升高等全身感染表现。感染严重时，切口周围皮肤可出现硬结甚至坏死。  切口血肿通常表现为：局部肿胀明显；疼痛加剧；皮肤青紫；触诊有波动感或张力增高。较大血肿还可能影响切口愈合并继发感染。  切口裂开则表现为：切口边缘分离；缝线松脱；渗液或渗血增多；严重时可见深层组织甚至腹腔内容物暴露。部分患者在咳嗽、起床或用力后会突然感觉切口“崩开”。  处理上，应根据具体情况采取措施。轻度红肿可加强观察及局部护理；感染者需换药并使用抗生素；血肿较大时可能需要切开清除；切口裂开严重者则需重新缝合。  因此，剖宫产切口观察不仅关注皮肤表面，更应动态评估疼痛、渗液及全身症状，做到早发现、早处理。 | 剖宫产术后腹部切口是产妇恢复的重要观察部位。由于手术切口涉及皮肤、脂肪及子宫等多层组织，如果护理不当，容易发生感染、血肿或切口裂开等并发症。  术后首先应观察切口愈合情况。正常切口边缘应贴合良好，没有明显裂隙。局部可能有轻微肿胀和压痛，但一般会逐渐减轻。  其次，要观察切口局部颜色。正常皮肤颜色变化不明显，如果出现明显发红、发热，则需警惕感染。  还应注意敷料及分泌物情况。正常切口敷料应保持干燥。如果敷料反复被血液或液体浸湿，则提示可能存在异常渗血或渗液。  疼痛评估也很关键。一般术后疼痛会逐渐缓解，若疼痛越来越严重，尤其伴局部肿块或发热，需要进一步检查。  此外，应观察有无局部隆起。切口周围出现肿块、皮肤发紫或波动感，提示可能形成血肿。  提示切口感染的表现包括：切口明显红肿热痛；出现黄色或脓性分泌物；伤口有臭味；伴发热、寒战等全身症状。感染严重时，还可能出现脂肪液化，表现为切口长期不愈合。  提示切口血肿的表现包括：局部明显肿胀；疼痛持续加重；皮肤颜色发紫；切口张力增高。  提示切口裂开的表现包括：伤口边缘分离；缝线断裂；渗血增加；严重时可见组织外翻。肥胖、糖尿病及感染患者更容易发生裂开。  临床处理时，应加强切口护理，保持局部清洁干燥。对于感染者，需要抗感染治疗及换药；血肿较大时可能需手术处理；裂开严重者则需再次缝合。  因此，剖宫产术后切口观察是预防严重并发症的重要措施，需要持续动态评估。 | 剖宫产术后腹部切口恢复情况，直接关系到产妇康复速度和感染风险，因此术后必须进行规范观察。  首先，应观察切口是否对合良好。正常情况下，切口边缘整齐，无明显裂隙或组织暴露。  其次，需要观察局部有无红肿。术后轻度肿胀属于正常反应，但如果红肿范围扩大、局部温度升高，则提示可能存在感染。  第三，应注意切口是否有渗液。正常切口一般较干燥。如果出现持续渗血、浆液样液体或脓液，则提示愈合异常。  疼痛程度也是重要指标。正常术后疼痛会逐渐减轻，若疼痛明显加重或呈持续跳痛，则需警惕感染或血肿。  另外，还应观察切口周围是否形成肿块。血肿形成时，局部会出现明显隆起、压痛和皮肤青紫。  切口感染的典型表现包括：局部红、肿、热、痛；脓性分泌物；恶臭味；发热及白细胞升高。部分患者还会出现切口愈合延迟。  切口血肿通常表现为：局部肿胀明显；切口周围淤青；疼痛持续加重；触摸时有紧张感。  而切口裂开则表现为：伤口边缘分离；缝线脱落；渗血或渗液增多；严重时深层组织外露。高危因素包括肥胖、糖尿病、营养不良及术后感染。  处理上，应加强切口清洁和换药，保持局部干燥。感染时需及时应用抗生素；较大血肿可能需切开引流；裂开严重者则需要再次手术修补。  因此，对剖宫产切口的观察不仅要关注表面愈合，更要结合疼痛、分泌物及全身症状综合判断。 |
| 中文回答的英文翻译 English translation of Chinese response | Monitoring the abdominal incision following a cesarean section is a critical component of postpartum care. The goal is to promptly detect complications such as incision infection, hematoma, fat necrosis, and incision dehiscence, thereby promoting proper wound healing.  First, the appearance of the incision should be examined. Under normal circumstances, the edges of the incision should align neatly with no obvious separation, and the area should exhibit only mild redness and slight swelling. Healthcare providers should check the length of the incision, the condition of the sutures, and whether the dressing is clean and dry.  Second, the color and temperature of the local skin should be assessed. Mild edema is common in the early postoperative period, but significant redness or elevated skin temperature may indicate inflammation or infection.  Third, the presence of wound exudate must be evaluated. A normal incision typically has no significant discharge, with only a small amount of pale bloody exudate possible. Persistent bleeding, serous exudate, purulent discharge, or a foul odor are considered abnormal findings.  Pain assessment is also crucial. Mild postoperative pain at the incision site is normal, but if the pain persists or worsens, becomes throbbing, or is accompanied by local swelling, one should be alert to the possibility of infection or a hematoma.  Additionally, check for any lumps around the incision. If there is a noticeable bulge, tenderness, or purple discoloration of the skin, this may indicate a subcutaneous hematoma.  Common signs of incision infection include:  marked local redness, swelling, warmth, and tenderness; purulent discharge from the incision; foul-smelling wound exudate; and systemic signs of infection such as fever and elevated white blood cell count. In severe cases, the skin around the incision may become indurated or even necrotic.  Incision hematoma typically presents as: marked local swelling; increased pain; skin cyanosis; and a fluctuating sensation or increased tension upon palpation. Larger hematomas may also impair wound healing and lead to secondary infection.  Incisional dehiscence is characterized by: separation of the wound edges; loosening of sutures; increased serous or bloody drainage; and, in severe cases, exposure of deep tissues or even abdominal contents. Some patients may suddenly feel the incision “tearing open” after coughing, getting out of bed, or straining.  Management should be tailored to the specific situation. Mild redness and swelling may be managed with close observation and local care; infected cases require dressing changes and antibiotics; large hematomas may require incision and drainage; and severe incision dehiscence requires re-suturing.  Therefore, monitoring of cesarean section incisions should not only focus on the skin surface but also involve dynamic assessment of pain, exudate, and systemic symptoms to ensure early detection and prompt intervention. | The abdominal incision following a cesarean section is a critical area to monitor during a mother’s recovery. Because the surgical incision involves multiple layers of tissue—including skin, fat, and the uterus—improper care can easily lead to complications such as infection, hematoma, or incision dehiscence.  First, monitor the healing of the incision. The edges of a normal incision should be well-aligned, with no obvious gaps. There may be slight swelling and tenderness at the site, but these symptoms generally subside gradually.  Second, observe the color of the incision site. Normal skin color should show no significant changes; however, if there is noticeable redness or warmth, be alert for signs of infection.  Also pay attention to the condition of the dressing and any discharge. A normal incision dressing should remain dry. If the dressing is repeatedly soaked with blood or fluid, this may indicate abnormal bleeding or fluid leakage.  Pain assessment is also critical. Postoperative pain usually subsides gradually. If the pain worsens, especially when accompanied by a local mass or fever, further examination is required.  Additionally, check for any local swelling. The presence of a mass, purple discoloration, or a fluctuating sensation around the incision suggests a possible hematoma.  Signs of incision infection include: marked redness, swelling, heat, and pain at the incision site; yellow or purulent discharge; a foul odor from the wound; and systemic symptoms such as fever and chills. In severe cases of infection, fat liquefaction may occur, manifesting as prolonged non-healing of the incision.  Signs of an incision hematoma include: marked local swelling; progressively worsening pain; purplish skin discoloration; and increased tension at the incision site.  Signs of incision dehiscence include: separation of wound edges; broken sutures; increased oozing of blood; and, in severe cases, visible everting of tissue. Obese patients, those with diabetes, and patients with infections are more prone to dehiscence.  Clinical management should focus on enhanced incision care to maintain local cleanliness and dryness. Patients with infection require antimicrobial therapy and dressing changes; large hematomas may require surgical intervention; and severe dehiscence necessitates re-suturing.  Therefore, monitoring the post-cesarean section incision is a critical measure for preventing serious complications and requires continuous, dynamic assessment. | The healing of the abdominal incision following a cesarean section directly affects the mother’s recovery rate and risk of infection; therefore, close monitoring is essential after surgery.  First, check whether the incision edges are properly aligned. Under normal circumstances, the edges should be neat, with no obvious gaps or exposed tissue.  Second, check for any redness or swelling in the area. Mild swelling after surgery is a normal reaction, but if the redness and swelling spread or the local temperature rises, this may indicate an infection.  Third, attention should be paid to whether there is any exudate from the incision. A normal incision is generally dry. Persistent bleeding, serous fluid, or pus indicates abnormal healing.  The severity of pain is also an important indicator. Normal postoperative pain gradually subsides; if the pain significantly worsens or presents as persistent throbbing pain, one should be alert to the possibility of infection or a hematoma.  Additionally, observe whether a mass has formed around the incision. When a hematoma develops, there will be noticeable swelling, tenderness, and skin discoloration (bruising) in the area.  Typical signs of an incision infection include: local redness, swelling, warmth, and pain; purulent discharge; a foul odor; fever; and elevated white blood cell count. Some patients may also experience delayed wound healing.  An incision hematoma typically presents as: marked local swelling; bruising around the incision; progressively worsening pain; and a sensation of tightness upon palpation.  An incision dehiscence, on the other hand, presents as: separation of the wound edges; loosening of sutures; increased bleeding or serous discharge; and, in severe cases, exposure of deep tissues. Risk factors include obesity, diabetes, malnutrition, and postoperative infection.  Management should focus on enhanced wound cleansing and dressing changes to maintain local dryness. Antibiotics should be administered promptly in cases of infection; larger hematomas may require incision and drainage; and severe dehiscence may necessitate reoperation for repair.  Therefore, monitoring of cesarean section incisions should not only focus on superficial healing but also involve a comprehensive assessment that considers pain, discharge, and systemic symptoms. |
| Correctness（1-5） | 4 | 4 | 4 |
| Completeness（1-5） | 4 | 4 | 4 |
| Guideline consistency（1-5） | 4 | 4 | 4 |
| Safety（1-5） | 4 | 4 | 4 |
| Clinical management appropriateness（1-5） | 4 | 4 | 4 |
| Chinese Reading Difficulty Score | 12.88 | 13.91 | 12.67 |
| Corresponding Education Level (Chinese) | 初中学段 | 初中学段 | 初中学段 |
| Recommended Reading Age (Chinese) | 13岁左右 | 14岁左右 | 13岁左右 |

## 问题 13

| 项目 | 第1次 | 第2次 | 第3次 |
| --- | --- | --- | --- |
| 产褥期临床知识问题 | 产褥期常见疼痛包括哪些类型？在哺乳期进行镇痛治疗时应注意什么？ | | |
| 主要指南来源 | NICE NG194 | | |
| 模型回答 | 产褥期由于分娩创伤、子宫恢复及哺乳等因素，产妇常会出现多种疼痛症状。适当识别疼痛类型并进行合理镇痛，不仅能够提高产妇舒适度，也有利于休息、活动及母乳喂养。  产褥期最常见的疼痛首先是宫缩痛。产后子宫为恢复至非孕状态，会持续收缩，因此产妇常感下腹阵发性疼痛，尤其在哺乳时更加明显。这种疼痛在经产妇中更常见，一般产后数日逐渐减轻。  第二，会阴疼痛也是常见问题。阴道分娩后如果存在会阴裂伤或侧切，局部组织损伤会导致疼痛，坐位、活动或排尿时可能更加明显。  第三，剖宫产术后切口疼痛较为常见。患者常表现为腹部切口牵拉痛或翻身、咳嗽时疼痛加重。  此外，乳房疼痛在哺乳期也比较多见。产后泌乳建立过程中，乳房胀痛、乳汁淤积甚至乳腺炎都可能引起明显不适。  腰背痛也是产褥期常见疼痛之一，与妊娠期间腰椎负荷增加、分娩劳累及照护婴儿姿势不良有关。  部分产妇还可能出现痔疮疼痛、盆底疼痛或下肢肌肉酸痛等。  在哺乳期进行镇痛治疗时，首先应重视药物安全性。因为部分药物可通过乳汁进入婴儿体内，因此应优先选择对哺乳影响较小、安全性较高的药物。  目前临床上常用且相对安全的镇痛药包括对乙酰氨基酚和布洛芬。这些药物进入乳汁量较少，一般不会对婴儿造成明显影响。  应尽量避免使用对新生儿有中枢抑制作用的药物，如部分阿片类镇痛药，以免引起婴儿嗜睡、呼吸抑制等风险。  其次，应遵循“小剂量、短疗程”原则。尽量使用最低有效剂量，并避免长期连续用药。  哺乳时间安排也需注意。一般建议产妇在服药后间隔一段时间再哺乳，以减少婴儿接触药物浓度高峰。  除了药物治疗，还应重视非药物镇痛措施。例如会阴冷敷、温水坐浴、乳房热敷、适当休息及正确哺乳姿势等。  如果疼痛持续加重或伴发热、红肿等异常表现，则应进一步评估是否存在感染、血肿或其他病理问题，而不能单纯依赖止痛。  因此，产褥期疼痛管理强调“有效、安全、兼顾哺乳”，既要缓解产妇不适，也要保障母婴安全。 | 产褥期是女性身体恢复的重要阶段，由于生理变化及分娩损伤，产妇常会经历不同类型的疼痛。  最常见的是子宫收缩痛，也称“后阵痛”。这是子宫收缩促进复旧的正常表现，哺乳时由于催产素释放增加，疼痛往往更明显。  其次，会阴伤口疼痛在阴道分娩后较为普遍。尤其是会阴侧切或较深裂伤患者，在活动、排尿和坐位时疼痛感较明显。  剖宫产患者则常出现腹部切口疼痛。疼痛通常在术后最明显，随后逐渐减轻，但咳嗽、翻身及下床活动时可能加重。  乳房疼痛也很常见。产后泌乳建立初期乳房胀满，若乳汁排空不畅，还可能形成乳汁淤积或乳腺炎。  此外，还包括腰背痛、盆底肌疼痛、痔疮疼痛以及肌肉酸痛等。  在哺乳期进行镇痛时，应特别注意药物对婴儿的影响。因为许多药物可经乳汁进入新生儿体内，因此必须谨慎选择。  一般推荐优先使用哺乳期安全性较高的药物，如对乙酰氨基酚和布洛芬。这类药物乳汁浓度较低，相对安全。  应避免随意使用强效镇痛药，特别是阿片类药物。因为部分药物可能导致婴儿嗜睡、吃奶差甚至呼吸抑制。  同时，应注意观察婴儿反应。如果母亲服药后婴儿出现异常嗜睡、喂养困难等，应及时停药并就医。  镇痛治疗还应结合非药物措施。例如：会阴疼痛可采取冷敷和坐浴；乳房疼痛可热敷并及时排乳；腰背痛可通过调整姿势和适度活动缓解。  此外，应寻找疼痛原因。如果疼痛越来越重、伴红肿发热或异常分泌物，则需排除感染等病理情况。  因此，哺乳期镇痛不仅要关注止痛效果，更要兼顾婴儿安全和母乳喂养的持续性。 | 产褥期常见疼痛主要与子宫恢复、分娩损伤及哺乳有关，是产后护理中的重要内容。  首先是宫缩痛。产后子宫不断收缩以恢复正常大小，因此会出现阵发性下腹痛，经产妇通常更明显。哺乳时疼痛加重属于常见现象。  第二是会阴疼痛。阴道分娩后的会阴裂伤或侧切，会导致局部组织疼痛，特别是在坐位、走动或排便时更加明显。  第三是剖宫产术后切口疼痛。术后早期患者活动时会感到腹部牵拉痛，部分人还会伴有切口麻木感。  第四是乳房疼痛。泌乳初期乳房胀满、乳汁淤积或乳头皲裂，都可能导致明显疼痛。  此外，还可能出现腰背痛、盆底疼痛、痔疮疼痛以及下肢肌肉酸痛等。  哺乳期镇痛治疗时，最重要的是药物安全性。因为药物可通过乳汁进入婴儿体内，因此需要优先选择对新生儿影响较小的药物。  目前常用且较安全的药物包括布洛芬和对乙酰氨基酚。一般在规范剂量下不会明显影响哺乳。  应尽量避免长期或大剂量使用镇静类及阿片类镇痛药，以免影响婴儿神经系统。  同时，应采用最低有效剂量和最短疗程原则。  在服药时间安排上，可建议产妇在哺乳后立即服药，以减少下一次哺乳时乳汁中的药物浓度。  非药物镇痛措施也非常重要。例如：会阴冷敷减轻肿胀；温水坐浴促进恢复；热敷乳房缓解胀痛；保持正确哺乳姿势减轻腰背负担。  如果疼痛持续加重或伴感染表现，如发热、红肿、恶臭分泌物等，则应及时就医，避免延误病情。  因此，产褥期疼痛管理应强调安全、有效和个体化，既帮助产妇恢复，也保障婴儿健康。 |
| 中文回答的英文翻译 English translation of Chinese response | During the postpartum period, new mothers often experience various types of pain due to factors such as the trauma of childbirth, uterine recovery, and breastfeeding. Properly identifying the type of pain and providing appropriate pain relief not only improves the mother’s comfort but also facilitates rest, mobility, and breastfeeding.  The most common type of pain during the postpartum period is uterine contractions. To return to its non-pregnant state, the uterus continues to contract after childbirth, causing women to experience intermittent lower abdominal pain, which is often more pronounced during breastfeeding. This pain is more common in multiparous women and generally subsides gradually over the first few days postpartum.  Second, perineal pain is also a common issue. Following vaginal delivery, if there is a perineal tear or episiotomy, local tissue damage can cause pain, which may be more pronounced when sitting, moving, or urinating.  Third, pain at the surgical incision site is relatively common after a cesarean section. Patients often experience a pulling sensation at the abdominal incision or increased pain when turning over or coughing.  In addition, breast pain is also relatively common during the lactation period. As lactation establishes postpartum, breast engorgement, milk stasis, and even mastitis can cause significant discomfort.  Lower back pain is another common complaint during the postpartum period, associated with increased lumbar load during pregnancy, the physical exertion of childbirth, and poor posture while caring for the infant.  Some new mothers may also experience pain from hemorrhoids, pelvic floor pain, or muscle soreness in the lower limbs.  When administering analgesic treatment during breastfeeding, drug safety must be the top priority. Since certain medications can pass into the infant’s system through breast milk, drugs with minimal impact on lactation and high safety profiles should be selected first.  Currently, commonly used and relatively safe analgesics in clinical practice include acetaminophen and ibuprofen. These drugs are excreted in breast milk in small amounts and generally do not have a significant impact on the infant.  Medications with central nervous system depressant effects on newborns, such as certain opioid analgesics, should be avoided whenever possible to prevent risks such as infant drowsiness and respiratory depression.  Secondly, the principle of “low dose, short duration” should be followed. Use the lowest effective dose whenever possible and avoid prolonged, continuous medication.  Careful attention should also be paid to the timing of breastfeeding. It is generally recommended that mothers wait a period of time after taking medication before breastfeeding to minimize the infant’s exposure to peak drug concentrations.  In addition to pharmacological treatment, non-pharmacological pain relief measures should be emphasized. These include cold compresses on the perineum, warm sitz baths, warm compresses on the breasts, adequate rest, and proper breastfeeding positions.  If pain persists or worsens, or is accompanied by abnormal symptoms such as fever, redness, or swelling, further evaluation is necessary to determine whether an infection, hematoma, or other pathological issues are present; reliance on analgesics alone is not sufficient.  Therefore, pain management during the postpartum period emphasizes “effectiveness, safety, and consideration for breastfeeding,” aiming to alleviate the mother’s discomfort while ensuring the safety of both mother and infant. | The postpartum period is a critical stage for a woman’s physical recovery. Due to physiological changes and injuries sustained during childbirth, new mothers often experience various types of pain.  The most common is uterine contraction pain, also known as “afterpains.” This is a normal manifestation of the uterus contracting to facilitate its return to its pre-pregnancy state; the pain is often more pronounced during breastfeeding due to increased oxytocin release.  Second, perineal wound pain is common following vaginal delivery. This is particularly true for women who have undergone an episiotomy or suffered a deep perineal tear, as pain is more pronounced during movement, urination, and sitting.  Women who have had a cesarean section often experience pain at the abdominal incision site. The pain is usually most intense immediately after surgery and gradually subsides, but it may worsen when coughing, turning over, or getting out of bed.  Breast pain is also common. During the initial phase of lactation, breasts may feel engorged; if milk is not fully expressed, this can lead to milk stasis or mastitis.  Other common discomforts include lower back pain, pelvic floor muscle pain, hemorrhoid pain, and muscle soreness.  When administering pain relief during breastfeeding, special attention must be paid to the potential effects of medications on the infant. Since many drugs can pass into breast milk and reach the newborn, careful selection is essential.  It is generally recommended to prioritize medications with a higher safety profile during breastfeeding, such as acetaminophen and ibuprofen. These medications have lower concentrations in breast milk and are relatively safe.  The indiscriminate use of potent analgesics, particularly opioids, should be avoided. This is because some medications may cause drowsiness, poor feeding, or even respiratory depression in the infant.  At the same time, the infant’s response should be closely monitored. If the infant exhibits abnormal drowsiness or feeding difficulties after the mother takes medication, the medication should be discontinued immediately and medical attention sought.  Pain management should also incorporate non-pharmacological measures. For example: perineal pain can be alleviated with cold compresses and sitz baths; breast pain can be relieved with warm compresses and timely milk expression; and lower back pain can be alleviated through posture adjustments and moderate activity.  Furthermore, the cause of the pain should be identified. If the pain worsens or is accompanied by redness, swelling, fever, or abnormal discharge, pathological conditions such as infection must be ruled out.  Therefore, pain management during breastfeeding should not only focus on pain relief but also prioritize the infant’s safety and the continuity of breastfeeding. | Common pain during the postpartum period is primarily related to uterine involution, birth-related injuries, and breastfeeding, and is an important aspect of postpartum care.  First is uterine contraction pain. After childbirth, the uterus continuously contracts to return to its normal size, resulting in intermittent lower abdominal pain, which is typically more pronounced in women who have given birth before. It is common for this pain to intensify during breastfeeding.  Second is perineal pain. Perineal tears or episiotomies following vaginal delivery can cause local tissue pain, which is particularly noticeable when sitting, walking, or having a bowel movement.  Third is pain from the cesarean section incision. In the early postoperative period, patients may experience a pulling sensation in the abdomen when moving, and some may also experience numbness around the incision.  Fourth is breast pain. Breast engorgement, milk stasis, or cracked nipples during the early stages of lactation can all cause significant pain.  In addition, lower back pain, pelvic floor pain, hemorrhoid pain, and muscle soreness in the lower limbs may also occur.  When administering pain relief during breastfeeding, drug safety is of the utmost importance. Since medications can pass into the infant’s body through breast milk, priority should be given to drugs that have minimal impact on the newborn.  Currently, commonly used and relatively safe medications include ibuprofen and acetaminophen. Generally, these do not significantly affect breastfeeding when taken at standard doses.  Long-term or high-dose use of sedatives and opioid analgesics should be avoided whenever possible to prevent adverse effects on the infant’s nervous system.  At the same time, the principles of the lowest effective dose and the shortest possible treatment duration should be followed.  Regarding medication timing, it is recommended that mothers take medication immediately after breastfeeding to reduce the concentration of the drug in breast milk by the next feeding.  Non-pharmacological pain relief measures are also crucial. For example: applying cold compresses to the perineum to reduce swelling; taking warm sitz baths to promote healing; applying warm compresses to the breasts to relieve engorgement; and maintaining proper breastfeeding posture to reduce strain on the lower back.  If pain persists or worsens, or is accompanied by signs of infection—such as fever, redness, swelling, or foul-smelling discharge—medical attention should be sought promptly to avoid delaying treatment.  Therefore, pain management during the postpartum period should emphasize safety, effectiveness, and individualization, supporting the mother’s recovery while ensuring the infant’s health. |
| Correctness（1-5） | 4 | 4 | 4 |
| Completeness（1-5） | 5 | 4 | 4 |
| Guideline consistency（1-5） | 4 | 4 | 4 |
| Safety（1-5） | 4 | 4 | 4 |
| Clinical management appropriateness（1-5） | 4 | 4 | 4 |
| Chinese Reading Difficulty Score | 13.68 | 13.46 | 13.42 |
| Corresponding Education Level (Chinese) | 初中学段 | 初中学段 | 初中学段 |
| Recommended Reading Age (Chinese) | 14岁左右 | 13岁左右 | 13岁左右 |

## 问题 14

| 项目 | 第1次 | 第2次 | 第3次 |
| --- | --- | --- | --- |
| 产褥期临床知识问题 | 产后乳房胀痛与病理性乳腺问题如何区分？医务人员应如何进行初步评估？ | | |
| 主要指南来源 | NICE NG194 | | |
| 模型回答 | 产后乳房胀痛是哺乳期非常常见的情况，大多数属于生理性变化，但部分也可能提示病理性乳腺问题。因此，正确区分生理性乳房胀痛与乳腺疾病，对于保障母乳喂养和预防严重并发症十分重要。  生理性乳房胀痛通常发生在产后2～5天泌乳开始建立阶段。由于乳汁分泌增加、乳房血流及淋巴循环增多，产妇会感觉双侧乳房胀满、发硬、轻度疼痛或发热感。其特点是：一般为双侧乳房同时出现；乳房整体均匀胀满；疼痛程度相对可耐受；哺乳或排乳后可明显缓解；通常无高热及明显全身症状。而病理性乳腺问题最常见的是乳汁淤积、乳腺炎及乳腺脓肿。  乳汁淤积时，患者常出现局部硬块、胀痛明显，哺乳后缓解不完全。局部皮肤一般无明显发红，但若持续不处理，容易进一步发展为感染。  急性乳腺炎则多表现为：乳房局部红、肿、热、痛；常为单侧发生；疼痛明显加重；伴发热、寒战及乏力；局部可触及压痛性硬块。  如果感染进一步加重形成乳腺脓肿，局部可能出现波动感，疼痛剧烈，高热持续不退。  医务人员进行初步评估时，首先应详细询问病史。包括发病时间、疼痛部位、是否与哺乳有关、乳汁排出是否通畅，以及是否伴发热等全身症状。  其次，需要观察乳房外观。评估是否存在局部红斑、肿胀、皮肤发亮或乳头皲裂。  随后进行乳房触诊。生理性胀奶通常为双侧均匀胀满；而病理性情况常出现局限性硬结、明显压痛或波动感。  同时，还需评估哺乳情况。包括婴儿吸吮姿势、哺乳频率及乳汁排空情况。很多乳腺问题与乳汁淤积和哺乳不当有关。  必要时，可完善血常规检查。若白细胞明显升高，提示感染可能。  对于疑似脓肿者，乳腺超声有助于明确诊断。  处理上，生理性胀奶主要通过增加哺乳次数、热敷及排空乳汁缓解；而乳腺炎则需要在继续排乳基础上及时抗感染治疗。  因此，区分生理性乳房胀痛和病理性乳腺疾病，关键在于是否存在局部炎症表现、明显单侧硬块及全身感染症状。 | 产后乳房胀痛在哺乳期十分常见，其中大部分属于正常泌乳过程中的生理现象，但也可能是乳腺炎等疾病的早期表现，因此临床上需要仔细鉴别。  生理性乳房胀痛通常发生于产后泌乳启动阶段。此时由于乳汁分泌增多、乳腺充血及组织水肿，乳房会出现胀满感和轻度疼痛。其特点包括：双侧乳房同时胀大；乳房整体均匀变硬；没有明显局部红肿；体温一般正常或仅轻度升高；哺乳后症状可减轻。  如果属于病理性乳腺问题，则症状往往更加局限和明显。  乳汁淤积时，患者会出现局部肿块和压痛，乳汁排出不畅，哺乳后缓解不明显。  急性乳腺炎则表现为局部红、肿、热、痛，通常为单侧乳房。患者还可能伴有发热、寒战、头痛和乏力等全身症状。  当形成乳腺脓肿时，局部可出现明显波动感，疼痛加重，高热持续存在。  医务人员在初步评估时，首先应询问疼痛发生时间及变化情况。了解是否存在乳汁淤积、哺乳间隔过长或乳头破损。  其次，应观察乳房外观。包括乳房大小、皮肤颜色、局部是否发红及乳头情况。  乳房触诊非常重要。生理性胀奶通常表现为广泛均匀胀满，而病理性问题常有局限性硬块、明显压痛或波动感。  还需评估哺乳方式。错误的哺乳姿势和乳汁排空不充分，是乳腺炎的重要诱因。  必要时，可进行实验室检查及乳腺超声，以明确是否存在感染或脓肿。  处理上，生理性胀奶以促进排乳为主，包括频繁哺乳、热敷和按摩；而病理性乳腺炎则需及时抗感染，并继续保持乳汁引流。  因此，是否存在局部炎症和全身症状，是区分正常胀奶与病理性乳腺问题的重要依据。 | 产后乳房胀痛既可能是正常泌乳反应，也可能提示乳腺疾病。临床中需要通过症状特点和体征进行区分。  生理性乳房胀痛一般发生在产后数天内，属于“生理性胀奶”。这是因为乳汁开始大量分泌，乳房充血、水肿所致。其主要特点包括：双侧乳房均匀胀满；乳房发硬但无明显局部红肿；疼痛程度较轻；哺乳或排乳后明显缓解；通常无高热。  而病理性乳腺问题则多表现为局部炎症或感染。  乳汁淤积时，乳房会出现局部硬结和明显压痛，乳汁排出不畅。  急性乳腺炎常表现为：单侧乳房红、肿、热、痛；局部疼痛持续加重；伴发热、寒战等全身感染表现；乳头皲裂常为诱因之一。  若病情进一步发展形成脓肿，则局部可能出现波动感和明显肿块。  医务人员初步评估时，首先应详细询问病史。包括哺乳频率、乳汁排空情况、是否存在乳头损伤以及疼痛是否持续加重。随后观察乳房外观。重点查看是否存在局部红斑、肿胀和皮肤紧张发亮。  乳房触诊时，需要判断硬块范围及性质。均匀胀满多为生理性；局限性硬块和明显压痛则提示病理改变。  同时，应评估产妇体温及全身情况。若伴高热和寒战，应高度怀疑感染。  必要时可进一步进行血常规和乳腺超声检查。超声对发现脓肿尤其有帮助。  处理上，生理性胀奶主要通过增加哺乳次数和促进排乳改善；病理性乳腺炎则需及时抗感染，必要时切开引流。  因此，乳房胀痛是否伴局部炎症和全身症状，是临床鉴别的关键。 |
| 中文回答的英文翻译 English translation of Chinese response | Postpartum breast tenderness is very common during breastfeeding; most cases are due to physiological changes, but some may indicate pathological breast issues. Therefore, correctly distinguishing between physiological breast tenderness and breast disease is crucial for ensuring successful breastfeeding and preventing serious complications.  Physiological breast engorgement typically occurs 2 to 5 days postpartum, during the initial stages of lactation. Due to increased milk production and enhanced blood and lymphatic circulation in the breasts, mothers may experience a sensation of fullness, firmness, mild pain, or warmth in both breasts. Its characteristics include: generally occurring in both breasts simultaneously; uniform fullness throughout the breasts; relatively tolerable pain; significant relief after breastfeeding or expressing milk; and there is usually no high fever or significant systemic symptoms. The most common pathological breast conditions are milk stasis, mastitis, and breast abscesses.  In cases of milk stasis, patients often experience localized lumps and significant tenderness, with incomplete relief after breastfeeding. The local skin is generally not noticeably reddened, but if left untreated, it can easily progress to infection.  Acute mastitis typically presents with local redness, swelling, warmth, and tenderness in the breast; it often occurs unilaterally; pain is markedly severe; and is accompanied by fever, chills, and fatigue; a tender, firm mass may be palpable locally.  If the infection worsens and forms a breast abscess, a fluctuating sensation may be present locally, accompanied by severe pain and persistent high fever.  When conducting an initial assessment, healthcare providers should first take a detailed medical history. This should include the onset time, location of pain, whether the condition is related to breastfeeding, whether milk flow is unobstructed, and whether there are systemic symptoms such as fever.  Second, the appearance of the breast should be observed. Assess for local erythema, swelling, shiny skin, or cracked nipples.  Next, perform a breast examination. Physiological engorgement typically presents as uniform fullness in both breasts, whereas pathological conditions often present with localized hard lumps, marked tenderness, or a fluctuating sensation.  At the same time, breastfeeding practices should be assessed. This includes the infant’s latch, feeding frequency, and the degree of milk removal. Many breast issues are related to milk stasis and improper breastfeeding techniques.  If necessary, a complete blood count (CBC) may be ordered. A significantly elevated white blood cell count suggests a possible infection.  For suspected abscesses, breast ultrasound is helpful in confirming the diagnosis.  Management-wise, physiological breast engorgement is primarily relieved by increasing the frequency of feedings, applying warm compresses, and emptying the breasts; mastitis, however, requires prompt antimicrobial treatment in addition to continued milk expression.  Therefore, the key to distinguishing between physiological breast tenderness and pathological breast disease lies in the presence of local inflammatory signs, a distinct unilateral hard mass, and systemic symptoms of infection. | Breast tenderness after childbirth is very common during the breastfeeding period. While most cases are a normal physiological part of the lactation process, they may also be early signs of conditions such as mastitis; therefore, careful clinical differentiation is necessary.  Physiological breast tenderness typically occurs during the initial stage of lactation after childbirth. At this time, increased milk production, breast engorgement, and tissue edema can cause a sensation of fullness and mild pain in the breasts. Characteristics include: simultaneous enlargement of both breasts; uniform hardening of the entire breast; no obvious localized redness or swelling; generally normal or only slightly elevated body temperature; and symptoms that may improve after breastfeeding.  If the issue is pathological, symptoms are often more localized and pronounced.  In cases of milk stasis, patients may experience localized lumps and tenderness, difficulty in milk ejection, and symptoms that do not significantly improve after breastfeeding.  Acute mastitis presents with localized redness, swelling, heat, and pain, typically affecting one breast. Patients may also experience systemic symptoms such as fever, chills, headache, and fatigue.  When a breast abscess forms, a distinct fluctuation may be felt locally, pain intensifies, and a high fever persists.  During the initial assessment, healthcare providers should first inquire about the onset and progression of pain. They should determine whether there is milk stasis, excessively long intervals between feedings, or cracked nipples.  Second, the appearance of the breast should be observed. This includes breast size, skin color, local redness, and the condition of the nipple.  Breast palpation is crucial. Physiological engorgement typically presents as widespread, uniform fullness, whereas pathological issues often involve localized lumps, marked tenderness, or a fluctuating sensation.  Breastfeeding techniques should also be assessed. Incorrect positioning and inadequate milk removal are major contributing factors to mastitis.  If necessary, laboratory tests and breast ultrasound may be performed to determine whether an infection or abscess is present.  In terms of management, physiological engorgement is primarily treated by promoting milk flow, including frequent breastfeeding, warm compresses, and massage; whereas pathological mastitis requires prompt treatment with antibiotics, along with continued milk drainage.  Therefore, the presence of local inflammation and systemic symptoms is a key factor in distinguishing normal engorgement from pathological breast issues. | Postpartum breast tenderness may be a normal response to lactation or may indicate a breast condition. Clinically, these conditions must be distinguished based on characteristic symptoms and physical signs.  Physiological breast engorgement generally occurs within a few days after childbirth and is classified as “physiological milk engorgement.” This is caused by the onset of abundant milk secretion, leading to breast congestion and edema. Its main characteristics include: uniform fullness in both breasts; firmness without significant localized redness or swelling; mild pain; significant relief after breastfeeding or expressing milk; and typically no high fever.  In contrast, pathological breast conditions often manifest as localized inflammation or infection.  When milk stasis occurs, the breast may develop localized hard lumps and significant tenderness, with poor milk flow.  Acute mastitis typically presents as: redness, swelling, warmth, and pain in one breast; progressively worsening localized pain; systemic signs of infection such as fever and chills; and cracked nipples are often a contributing factor.  If the condition progresses to form an abscess, a fluctuating sensation and a distinct mass may be palpable locally.  During the initial assessment, healthcare providers should first conduct a detailed medical history review. This includes inquiring about breastfeeding frequency, milk drainage, the presence of nipple injuries, and whether the pain is progressively worsening. Subsequently, the appearance of the breast should be examined, with particular attention to the presence of localized erythema, swelling, and taut, shiny skin.  During breast palpation, the extent and nature of any hardening should be assessed. Uniform fullness is typically physiological, whereas localized hardening and marked tenderness suggest pathological changes.  Concurrently, the mother’s temperature and general condition should be evaluated. If accompanied by high fever and chills, infection should be strongly suspected.  If necessary, further tests such as a complete blood count and breast ultrasound may be performed. Ultrasound is particularly helpful in detecting abscesses.  Management-wise, physiological engorgement is primarily improved by increasing the frequency of breastfeeding and promoting milk ejection; pathological mastitis requires prompt antimicrobial therapy and, if necessary, incision and drainage.  Therefore, whether breast tenderness is accompanied by local inflammation and systemic symptoms is key to clinical differentiation. |
| Correctness（1-5） | 4 | 4 | 4 |
| Completeness（1-5） | 5 | 4 | 4 |
| Guideline consistency（1-5） | 3 | 3 | 3 |
| Safety（1-5） | 4 | 4 | 4 |
| Clinical management appropriateness（1-5） | 5 | 4 | 4 |
| Chinese Reading Difficulty Score | 13.39 | 13.74 | 13.16 |
| Corresponding Education Level (Chinese) | 初中学段 | 初中学段 | 初中学段 |
| Recommended Reading Age (Chinese) | 13岁左右 | 14岁左右 | 13岁左右 |

## 问题 15

| 项目 | 第1次 | 第2次 | 第3次 |
| --- | --- | --- | --- |
| 产褥期临床知识问题 | 急性乳腺炎的典型表现有哪些？出现乳房红肿热痛、发热或全身不适时应如何处理？ | | |
| 主要指南来源 | NICE NG194 | | |
| 模型回答 | 急性乳腺炎是哺乳期较常见的乳腺感染性疾病，多发生于产后2～6周，尤其见于初产妇。其主要原因与乳汁淤积、乳头皲裂及细菌侵入有关。如果处理不及时，可能进一步发展为乳腺脓肿。  急性乳腺炎最典型的表现是乳房局部红、肿、热、痛。患者通常为单侧乳房发病，局部皮肤出现片状红斑，触摸时温度升高，并伴明显压痛。  其次，乳房内常可触及硬结或肿块。乳汁排出不畅时，局部胀痛会逐渐加重，哺乳时疼痛尤其明显。  全身症状也较突出。患者常出现发热，体温可超过38.5℃，并伴寒战、乏力、头痛、肌肉酸痛等类似“感冒”症状。  部分患者还会出现食欲下降和精神状态变差。如果病情进一步进展形成乳腺脓肿，局部疼痛会更加剧烈，并可能出现波动感。  当产妇出现乳房红肿热痛、发热或全身不适时，应尽早处理。  首先，应继续保持乳汁引流。很多产妇担心疼痛而停止哺乳，但实际上乳汁淤积会加重感染。通常建议继续母乳喂养或使用吸奶器排空乳汁。其次，应纠正哺乳姿势。医务人员需指导婴儿正确含接乳头，避免乳汁排空不充分。局部护理也很重要。哺乳前可适当热敷，促进乳汁流动；哺乳后可冷敷减轻水肿和疼痛。同时，应注意休息和补充水分，提高机体抵抗力。  如果症状较轻，可先通过充分排乳及局部护理观察。但若出现高热、明显红肿疼痛或症状持续加重，则需及时就医。  临床上通常需要应用抗生素治疗，常选择对哺乳期相对安全、针对金黄色葡萄球菌有效的药物。  必要时可使用对乙酰氨基酚或布洛芬缓解疼痛和发热。  若怀疑乳腺脓肿，应进行乳腺超声检查。一旦形成脓肿，可能需要穿刺抽吸或切开引流。  因此，急性乳腺炎的处理核心是“及时抗感染+持续排乳”，早期干预能够有效避免病情加重。 | 急性乳腺炎是哺乳期常见感染性疾病，多由乳汁淤积基础上细菌侵入乳腺组织引起。初产妇、哺乳姿势不正确及乳头破损者更容易发生。  其典型表现首先是乳房局部疼痛。患者常感觉单侧乳房某一区域明显胀痛，触碰时疼痛加重。随后局部会出现红肿热痛。皮肤发红、温度升高，并可触及质硬的肿块。发热是急性乳腺炎的重要表现之一。多数患者体温升高明显，可伴寒战和全身酸痛。此外，患者还可能出现乏力、头痛、食欲下降等全身不适症状。  如果治疗不及时，感染可进一步形成乳腺脓肿，此时局部会出现波动感，疼痛更加明显。当出现乳房红肿热痛及发热时，应尽快采取措施。  首先，必须保持乳汁通畅排出。停止哺乳反而会加重乳汁淤积，因此通常建议继续哺乳。哺乳前热敷和轻柔按摩，有助于促进乳汁排空；哺乳后可冷敷减轻肿胀。同时，应指导产妇纠正哺乳方式，避免婴儿含接不良导致乳头损伤。休息和营养支持也很重要。充足睡眠和补液有助于提高免疫力。  如果局部症状明显或伴高热，则需要尽早就医。医生通常会根据情况给予抗生素治疗。哺乳期常用抗生素一般对婴儿影响较小，多数情况下不需要停止母乳喂养。必要时可使用安全的退热止痛药缓解不适。若症状持续不缓解或局部出现明显波动感，应警惕脓肿形成，并进行乳腺超声检查。  因此，急性乳腺炎越早干预效果越好，关键是及时排乳和控制感染。 | 急性乳腺炎是产后哺乳期女性常见的乳腺感染，多与乳汁淤积和细菌感染有关。若处理不及时，可发展为乳腺脓肿。  典型症状首先表现为单侧乳房疼痛。疼痛通常局限于某一区域，并逐渐加重。随后乳房局部出现红、肿、热、痛，触诊时可发现压痛明显的硬结。患者常伴发热，部分可出现寒战，体温甚至达到39℃以上。此外，还会有全身不适表现，如乏力、头痛、肌肉酸痛及食欲减退。如果病情进一步发展，乳房局部可能形成脓肿，表现为明显肿块及波动感。  一旦出现乳房红肿热痛和发热，应及时处理。  首先，应尽量保证乳汁排空。可继续哺乳或使用吸奶器，避免乳汁淤积加重感染。其次，可在哺乳前进行热敷和按摩，帮助乳汁流出；哺乳后冷敷，有助于减轻疼痛和水肿。同时，需要纠正错误哺乳姿势，减少乳头损伤。对于发热及疼痛明显者，应及时就医。医生通常会给予抗感染治疗，并根据病情决定是否需要进一步检查。大多数哺乳期抗生素是可以继续哺乳的，因此一般不建议轻易停止母乳喂养。若局部疼痛持续加重、出现波动感或高热不退，则需警惕乳腺脓肿，必要时进行穿刺或切开引流。  因此，急性乳腺炎处理重点包括“排空乳汁、控制感染、缓解症状”三个方面。 |
| 中文回答的英文翻译 English translation of Chinese response | Acute mastitis is a common infectious breast condition during lactation, typically occurring 2 to 6 weeks after childbirth, particularly among first-time mothers. Its primary causes are related to milk stasis, cracked nipples, and bacterial invasion. If not treated promptly, it may progress to a breast abscess.  The most typical manifestations of acute mastitis are localized redness, swelling, warmth, and tenderness in the breast. The condition usually affects one breast, with patchy erythema appearing on the local skin, increased temperature upon touch, and marked tenderness.  Additionally, a hard lump or mass is often palpable within the breast. When milk flow is obstructed, localized distension and pain gradually worsen, and the pain is particularly pronounced during breastfeeding.  Systemic symptoms are also prominent. Patients often experience fever, with temperatures exceeding 38.5°C, accompanied by chills, fatigue, headache, muscle aches, and other “cold-like” symptoms.  Some patients may also experience a loss of appetite and a decline in mental well-being. If the condition progresses to form a breast abscess, local pain will become more severe, and a fluctuating sensation may be present.  When a new mother experiences redness, swelling, heat, or pain in the breast, along with fever or general malaise, prompt treatment is essential.  First, milk drainage should be maintained. Many new mothers stop breastfeeding due to pain, but in reality, milk stasis can worsen the infection. It is generally recommended to continue breastfeeding or use a breast pump to empty the breasts. Second, the breastfeeding position should be corrected. Healthcare providers should guide the infant to latch onto the nipple correctly to prevent incomplete milk removal. Local care is also crucial. Applying a warm compress before breastfeeding can help promote milk flow, while a cold compress after breastfeeding can reduce swelling and pain. Additionally, adequate rest and hydration are essential to boost the body’s immune response.  If symptoms are mild, the condition can initially be monitored through thorough milk expression and local care. However, if high fever, significant redness, swelling, or pain develops, or if symptoms continue to worsen, prompt medical attention is required.  Clinically, antibiotic treatment is usually necessary, with drugs selected that are relatively safe during breastfeeding and effective against Staphylococcus aureus.  If necessary, acetaminophen or ibuprofen may be used to relieve pain and fever.  If a breast abscess is suspected, a breast ultrasound should be performed. Once an abscess has formed, aspiration or incision and drainage may be required.  Therefore, the core of managing acute mastitis is “timely antimicrobial therapy + continuous milk expression”; early intervention can effectively prevent the condition from worsening. | Acute mastitis is a common infectious disease during lactation, often caused by bacterial invasion of breast tissue following milk stasis. First-time mothers, those with improper breastfeeding posture, and those with cracked nipples are more susceptible to this condition.  The typical presentation begins with localized breast pain. Patients often experience marked swelling and tenderness in a specific area of one breast, with pain worsening upon touch. Subsequently, the affected area becomes red, swollen, warm, and tender. The skin becomes red and warm, and a firm lump may be palpable. Fever is one of the key symptoms of acute mastitis. Most patients experience a significant rise in body temperature, which may be accompanied by chills and generalized body aches. Additionally, patients may experience systemic symptoms such as fatigue, headache, and loss of appetite.  If not treated promptly, the infection can progress to a breast abscess, at which point a fluctuating sensation may be felt locally, and the pain becomes more pronounced. When redness, swelling, heat, pain in the breast, and fever occur, measures should be taken as soon as possible.  First, it is essential to maintain unobstructed milk flow. Stopping breastfeeding can actually worsen milk stasis, so it is generally recommended to continue breastfeeding. Applying a warm compress and gently massaging the breast before feeding helps promote milk emptying; applying a cold compress after feeding can reduce swelling. At the same time, the mother should be instructed to correct her breastfeeding technique to avoid nipple damage caused by poor latch. Rest and nutritional support are also crucial. Adequate sleep and hydration help boost immunity.  If local symptoms are severe or accompanied by a high fever, medical attention should be sought immediately. Doctors will typically prescribe antibiotic treatment based on the situation. Antibiotics commonly used during breastfeeding generally have minimal impact on the infant, and in most cases, breastfeeding does not need to be discontinued. If necessary, safe antipyretic and analgesic medications may be used to relieve discomfort. If symptoms persist or a distinct fluctuation is felt in the affected area, be alert for the possibility of an abscess and undergo a breast ultrasound examination.  Therefore, the earlier acute mastitis is treated, the better the outcome. The key is timely milk drainage and infection control. | Acute mastitis is a common breast infection among women during the postpartum lactation period, often associated with milk stasis and bacterial infection. If not treated promptly, it can progress to a breast abscess.  Typical symptoms first manifest as pain in one breast. The pain is usually localized to a specific area and gradually intensifies. Subsequently, the affected area becomes red, swollen, warm, and tender, and a firm lump with marked tenderness may be felt on palpation. Patients often experience fever, and some may have chills, with body temperature rising to 39°C or higher. Additionally, they may exhibit general malaise, such as fatigue, headache, muscle aches, and loss of appetite. If the condition progresses further, a breast abscess may form, presenting as a distinct lump with a fluctuating sensation.  Once redness, swelling, heat, pain in the breast, and fever occur, prompt treatment is necessary.  First, ensure that the breast is emptied as much as possible. Continue breastfeeding or use a breast pump to prevent milk stasis from worsening the infection. Second, apply a warm compress and massage the breast before feeding to help milk flow; apply a cold compress after feeding to help reduce pain and swelling. At the same time, correct any incorrect breastfeeding positions to minimize nipple damage. For those with significant fever and pain, seek medical attention promptly. Doctors typically prescribe antimicrobial therapy and will determine whether further testing is necessary based on the condition. Most antibiotics used during breastfeeding are safe for continued breastfeeding, so it is generally not recommended to stop breastfeeding prematurely. If local pain continues to worsen, a fluctuating sensation develops, or a high fever persists, be alert for a breast abscess; puncture or incision and drainage may be necessary.  Therefore, the key aspects of managing acute mastitis include “emptying the breast, controlling the infection, and relieving symptoms.” |
| Correctness（1-5） | 4 | 4 | 4 |
| Completeness（1-5） | 4 | 4 | 3 |
| Guideline consistency（1-5） | 4 | 4 | 4 |
| Safety（1-5） | 4 | 4 | 4 |
| Clinical management appropriateness（1-5） | 4 | 4 | 3 |
| Chinese Reading Difficulty Score | 13.28 | 13.32 | 13.44 |
| Corresponding Education Level (Chinese) | 初中学段 | 初中学段 | 初中学段 |
| Recommended Reading Age (Chinese) | 13岁左右 | 13岁左右 | 13岁左右 |

## 问题 16

| 项目 | 第1次 | 第2次 | 第3次 |
| --- | --- | --- | --- |
| 产褥期临床知识问题 | 哺乳期乳头疼痛或皲裂常见原因有哪些？应从哪些方面评估哺乳姿势和含接情况？ | | |
| 主要指南来源 | NICE NG194 | | |
| 模型回答 | 哺乳期乳头疼痛和乳头皲裂是母乳喂养中非常常见的问题，也是导致部分产妇提前停止母乳喂养的重要原因。多数情况下与哺乳方式不正确有关，但也可能与感染或局部刺激等因素相关。  最常见的原因首先是婴儿含接不正确。如果婴儿只含住乳头而没有充分含住乳晕，吸吮时乳头受到持续摩擦和挤压，就容易导致疼痛和破损。其次，哺乳姿势不当也很常见。例如婴儿身体未与母亲贴近、头颈扭转或位置偏低，都会影响有效吸吮，增加乳头损伤风险。第三，频繁错误拔离乳头也会导致皲裂。有些产妇在婴儿未停止吸吮时强行拉开乳头，容易造成机械性损伤。  乳头局部潮湿刺激也是诱因之一。乳汁长期残留、频繁使用刺激性清洁剂或乳垫不透气，都可能导致皮肤屏障受损。此外，乳头扁平、内陷以及婴儿舌系带过短，也会影响正确含接。部分患者还可能存在真菌感染，如乳头念珠菌感染。此时疼痛常呈烧灼样，哺乳后仍持续明显疼痛。  医务人员在评估时，首先应观察哺乳姿势。 需要评估母亲是否处于舒适放松状态，是否有背部和手臂支撑。婴儿应做到“耳、肩、臀成一直线”，身体面向母亲，而不是仅头部转向乳房。其次，要重点观察婴儿含接情况。正确含接时，婴儿口张大，下唇外翻，下巴贴近乳房，含入口中的不仅是乳头，还应包括大部分乳晕。如果看到婴儿仅咬住乳头、口角发出“啧啧”声、吸吮浅快或母亲明显疼痛，则提示含接不良。还需观察哺乳后乳头形态。正常情况下乳头应保持圆形；如果哺乳后乳头被压扁、发白或出现裂口，则说明受压异常。同时，还应评估哺乳频率、单次哺乳时间以及乳房排空情况。  处理上，应首先纠正哺乳姿势和含接方法，这是最关键的措施。必要时可短期使用乳头保护剂、保持局部干燥并继续母乳喂养。  因此，乳头疼痛和皲裂的核心问题往往不是“乳头本身”，而是哺乳技术问题，需要系统评估和指导。 | 哺乳期乳头疼痛和皲裂在初产妇中尤为常见，多数发生在产后最初几周。如果处理不及时，不仅影响母乳喂养，还可能诱发乳腺炎。  最常见原因是婴儿含乳方式错误。婴儿如果只吸吮乳头而没有充分含住乳晕，会使乳头局部长期摩擦，最终导致疼痛和裂口。其次，哺乳姿势不正确也会增加损伤风险。例如婴儿位置偏低、身体没有贴近母亲，都会导致吸吮角度异常。第三，乳头护理不当也会引起问题。频繁清洗、使用酒精或肥皂擦拭，会破坏乳头表面保护层，使皮肤更容易裂开。此外，乳汁淤积、乳房胀满时，婴儿含接困难，也容易导致乳头损伤。  某些特殊情况，如婴儿舌系带过短、吸吮力异常，或母亲乳头内陷，也会造成反复疼痛。如果乳头疼痛呈烧灼感，并在哺乳后持续存在，还需考虑真菌感染。  评估时，首先需要观察母婴体位。母亲应处于舒适放松状态，婴儿身体应贴近母亲，头、肩、臀保持一条直线。其次，应观察婴儿口部动作。正确含接时，婴儿嘴巴张得较大，下唇外翻，下巴紧贴乳房。同时，应看婴儿是否含住大部分乳晕，而不仅仅是乳头。  如果哺乳过程中母亲明显疼痛、婴儿频繁松口或发出吸空声音，说明含接不良。哺乳结束后还应检查乳头状态。正常乳头不应出现明显压痕、发白或裂口。此外，还要了解哺乳次数、是否单侧长期喂养以及乳房是否充分排空。处理重点是纠正含接和姿势，同时减少乳头刺激。必要时可局部使用乳头修复剂，并保持局部清洁干燥。  因此，乳头疼痛和皲裂往往是哺乳技术问题的表现，规范指导是最有效的干预措施。 | 乳头疼痛和皲裂是哺乳期最常见的不适之一，通常与机械性损伤有关，但也可能与感染及乳房问题相关。  最主要原因是含接不良。婴儿如果只含住乳头而没有充分含住乳晕，会导致乳头受到持续摩擦和挤压。其次，错误哺乳姿势也是常见原因。例如婴儿头部偏斜、身体未贴近母亲，都会影响吸吮效率。第三，乳房过度胀满时，乳头变平，婴儿不易正确含接，也容易造成损伤。乳头局部过度清洁、频繁使用消毒液或乳垫不透气，也会破坏皮肤屏障。此外，乳头真菌感染、婴儿鹅口疮以及舌系带过短等问题，也会引起持续乳头疼痛。  在评估哺乳姿势时，应观察母亲是否姿势舒适，有无前倾或肩颈紧张。婴儿应“腹对腹”贴近母亲，耳、肩、臀保持一条直线。在含接评估中，重点观察婴儿是否张大嘴巴，下唇是否外翻，下巴是否贴近乳房。正确含接时，婴儿应含住大部分乳晕，而不是仅吸乳头。吸吮过程中应能听到吞咽声，而不是频繁“咂嘴”声音。哺乳后若乳头形状正常、无压痕和发白，通常说明含接较好。如果乳头明显变形、疼痛剧烈或出现裂口，则提示含接异常。处理方面，应优先纠正哺乳姿势和含接方式，同时减少乳头刺激并促进局部修复。  因此，对乳头疼痛和皲裂的评估，不仅要看乳头损伤本身，更要关注整个母婴哺乳过程。 |
| 中文回答的英文翻译 English translation of Chinese response | Nipple pain and nipple cracks are very common issues during breastfeeding and are major reasons why some new mothers stop breastfeeding prematurely. In most cases, these problems are related to improper latching, but they may also be caused by factors such as infection or local irritation.  The most common cause is incorrect latch. If the baby latches onto only the nipple without fully covering the areola, the nipple is subjected to continuous friction and compression during suckling, which can easily lead to pain and damage. Secondly, improper breastfeeding posture is also common. For example, if the baby is not positioned close to the mother, or if the baby’s head and neck are twisted or positioned too low, this can impair effective suckling and increase the risk of nipple injury. Third, frequent and improper removal of the nipple can also cause cracks. Some mothers forcibly pull the nipple away before the baby has finished suckling, which can easily cause mechanical injury.  Local moisture and irritation of the nipple are also contributing factors. Prolonged milk residue, frequent use of harsh cleansers, or non-breathable nursing pads can all compromise the skin barrier. Additionally, flat or inverted nipples, as well as a short lingual frenulum in the infant, can interfere with proper latch-on. Some patients may also have fungal infections, such as nipple candidiasis. In such cases, the pain is often burning in nature and persists significantly even after breastfeeding.  When conducting an assessment, healthcare providers should first observe the breastfeeding position. It is important to evaluate whether the mother is in a comfortable and relaxed state and whether she has adequate back and arm support. The infant should be positioned so that the “ear, shoulder, and hip are in a straight line,” with the body facing the mother, rather than simply turning the head toward the breast. Second, close attention should be paid to the infant’s latch. With a proper latch, the infant’s mouth is wide open, the lower lip is everted, and the chin is close to the breast; the infant should take not only the nipple but also most of the areola into the mouth. If the infant is seen biting only the nipple, making a “smacking” sound at the corners of the mouth, sucking shallowly and rapidly, or if the mother experiences significant pain, this indicates a poor latch. The shape of the nipple after feeding should also be observed. Under normal circumstances, the nipple should remain round; if the nipple is flattened, turns white, or develops cracks after feeding, this indicates abnormal pressure. At the same time, the frequency of feedings, the duration of each feeding, and the degree of breast emptying should be assessed.  Management should first focus on correcting the feeding position and latch technique, which is the most critical measure. If necessary, nipple protectors may be used for a short period, while maintaining local dryness and continuing breastfeeding.  Therefore, the core issue behind nipple pain and cracking is often not the “nipple itself,” but rather a problem with breastfeeding technique, requiring systematic assessment and guidance. | Nipple pain and cracking are particularly common among first-time mothers, with most cases occurring in the first few weeks after childbirth. If not addressed promptly, this can not only affect breastfeeding but also lead to mastitis.  The most common cause is improper latching. If a baby sucks only on the nipple without fully taking the areola into the mouth, it causes prolonged friction on the nipple, eventually leading to pain and cracks. Second, incorrect breastfeeding positions can also increase the risk of injury. For example, positioning the baby too low or not close enough to the mother can result in an abnormal sucking angle. Third, improper nipple care can also cause problems. Frequent washing or wiping with alcohol or soap can damage the protective layer on the nipple surface, making the skin more prone to cracking. Additionally, when milk becomes engorged or the breasts are full, the baby may have difficulty latching on, which can also lead to nipple damage.  Certain specific conditions, such as a short frenulum, abnormal sucking strength, or inverted nipples, can also cause recurring pain. If the nipple pain feels like a burning sensation and persists after breastfeeding, a fungal infection should also be considered.  During an assessment, the first step is to observe the positioning of the mother and baby. The mother should be in a comfortable, relaxed position, with the baby close to her body, and the baby’s head, shoulders, and hips aligned in a straight line. Next, observe the baby’s mouth movements. With a proper latch, the baby’s mouth is wide open, the lower lip is everted, and the chin is pressed firmly against the breast. At the same time, check whether the baby has latched onto most of the areola, not just the nipple.  If the mother experiences significant pain during breastfeeding, or if the infant frequently releases the nipple or makes a hollow sucking sound, this indicates poor latch. After breastfeeding, the condition of the nipple should also be checked. A normal nipple should not show obvious indentations, blanching, or cracks. Additionally, it is important to assess the frequency of feedings, whether there is prolonged feeding on one side, and whether the breast is being fully emptied. The focus of management is on correcting the latch and posture while minimizing nipple irritation. If necessary, apply a nipple repair cream topically and keep the area clean and dry.  Therefore, nipple pain and cracking are often manifestations of breastfeeding technique issues, and standardized guidance is the most effective intervention. | Sore and cracked nipples are among the most common discomforts during breastfeeding. They are usually related to mechanical trauma but may also be associated with infections or breast-related issues.  The primary cause is poor latch. If a baby latches onto only the nipple without fully covering the areola, it can lead to constant friction and pressure on the nipple. Secondly, incorrect breastfeeding positions are also a common cause. For example, if the baby’s head is tilted or their body is not close to the mother, it can affect the efficiency of suckling. Third, when the breasts are overly engorged, the nipples flatten, making it difficult for the baby to latch correctly and increasing the risk of injury. Excessive cleaning of the nipple area, frequent use of antiseptics, or non-breathable nursing pads can also damage the skin barrier. Additionally, fungal infections of the nipple, thrush in the baby, and a short frenulum can cause persistent nipple pain.  When assessing breastfeeding posture, observe whether the mother is comfortable and whether she is leaning forward or experiencing tension in her shoulders and neck. The baby should be positioned “tummy-to-tummy” close to the mother, with the ear, shoulder, and hip aligned in a straight line. When evaluating latch-on, focus on whether the baby has opened their mouth wide, whether the lower lip is everted, and whether the chin is close to the breast. With a proper latch, the baby should take in most of the areola, rather than just the nipple. During suckling, swallowing sounds should be audible, rather than frequent “smacking” noises. If the nipple retains its normal shape after feeding, with no indentations or blanching, this typically indicates a good latch. If the nipple is noticeably deformed, there is severe pain, or cracks are present, this suggests an abnormal latch. Management should prioritize correcting the breastfeeding position and latch technique, while simultaneously reducing nipple irritation and promoting local healing.  Therefore, when assessing nipple pain and cracking, it is essential to consider not only the nipple injury itself but also the entire mother-infant breastfeeding process. |
| Correctness（1-5） | 4 | 4 | 4 |
| Completeness（1-5） | 4 | 4 | 4 |
| Guideline consistency（1-5） | 4 | 4 | 4 |
| Safety（1-5） | 4 | 4 | 4 |
| Clinical management appropriateness（1-5） | 4 | 4 | 3 |
| Chinese Reading Difficulty Score | 13.53 | 13.51 | 13.60 |
| Corresponding Education Level (Chinese) | 初中学段 | 初中学段 | 初中学段 |
| Recommended Reading Age (Chinese) | 13岁左右 | 13岁左右 | 14岁左右 |

## 问题 17

| 项目 | 第1次 | 第2次 | 第3次 |
| --- | --- | --- | --- |
| 产褥期临床知识问题 | 评估母乳喂养是否有效时，应询问和观察哪些内容？ | | |
| 主要指南来源 | NICE NG194；中国孕产妇健康管理服务规范 | | |
| 模型回答 | 评估母乳喂养是否有效，是产后母婴护理中的重要内容。有效的母乳喂养不仅关系到新生儿营养摄入，也影响乳汁分泌建立及母亲乳房健康。因此，医务人员需要从母亲、婴儿以及哺乳过程多个方面进行综合评估。  首先，应询问哺乳频率和持续时间。正常新生儿一般每日哺乳8～12次，按需喂养。如果喂奶次数过少、间隔过长，容易导致乳汁分泌不足或乳汁淤积。其次，需要了解母亲是否有“下奶”感觉。例如哺乳时乳房变软、乳汁流出、另一侧乳房溢乳等，都提示泌乳建立较好。还应询问母亲在哺乳时是否疼痛。轻微不适可以接受，但明显乳头疼痛或皲裂往往提示含接不正确。观察哺乳姿势和含接情况是重点内容。有效喂养时，婴儿应身体贴近母亲，头、肩、臀呈一直线；婴儿口张大，下唇外翻，下巴贴近乳房，并含住大部分乳晕，而不仅是乳头。  其次，应观察婴儿吸吮方式。有效吸吮通常表现为节律性深吸吮，并能听到吞咽声。如果仅快速浅吸或频繁松口，则提示吸吮效率差。还需观察婴儿喂奶后的状态。有效喂养后，婴儿通常表现满足、安静、能够自行松口并进入睡眠。婴儿排尿排便情况也是重要指标。一般出生后数天内，每日尿布逐渐增至6次以上，大便颜色由胎便转为黄色糊状，提示摄入量较充足。体重变化同样关键。新生儿出生后短期内可有生理性体重下降，但通常在10～14天恢复出生体重。如果持续下降，则需警惕喂养不足。  此外，还应观察乳房情况。有效哺乳后乳房通常较哺乳前柔软；若长期胀痛、硬结明显，则提示乳汁排空不充分。  因此，评估母乳喂养是否有效，不仅要看“有没有喂”，更要关注婴儿是否真正获得足够乳汁，以及母亲是否建立了良好的哺乳过程。 | 判断母乳喂养是否有效，需要同时评估母亲、婴儿及哺乳过程中的多个方面。只有母婴双方配合良好，才能说明喂养是有效的。  首先，应询问哺乳次数。新生儿通常需要按需喂养，每天大约8～12次。如果喂养次数明显偏少，可能影响乳汁分泌和婴儿摄入。其次，应了解母亲乳汁分泌情况。例如是否有乳房胀满感、喷乳反射以及哺乳后乳房变软等。还应询问母亲是否存在乳头疼痛、皲裂或乳房胀痛。如果哺乳过程持续疼痛，多提示姿势或含接存在问题。观察哺乳姿势是重要环节。母亲应处于舒适放松状态，婴儿身体贴近母亲，头部不扭转。含接情况尤其关键。有效含接时，婴儿口张大、下唇外翻、下巴贴近乳房，并含住较多乳晕。  吸吮动作方面，正常有效吸吮应表现为缓慢、有力且有节律，能够听到吞咽声。如果婴儿吸几口就松开、频繁哭闹或吃奶时间过长却不满足，则提示可能喂养不足。此外，需要观察婴儿喂奶后的表现。有效喂养后婴儿通常较安静、满足并能自然入睡。  排尿和排便情况也很重要。尿量充足、大便逐渐转黄，说明乳汁摄入较好。  体重监测是客观指标。若婴儿体重增长正常，通常说明母乳喂养有效。  同时，还需观察母亲乳房状态。哺乳后乳房应较前柔软，没有明显胀痛和硬结。  因此，评估母乳喂养是否有效，需要结合“母亲感受、婴儿表现和客观指标”综合判断。 | 母乳喂养效果评估是产后护理中的重要内容，其核心是判断婴儿是否获得足够乳汁，以及母亲哺乳过程是否顺利。  首先，应询问母亲哺乳情况，包括每日喂奶次数、每次持续时间以及夜间是否哺乳。新生儿一般需要频繁按需喂养。其次，要了解母亲乳汁分泌感觉。例如哺乳前乳房胀满、哺乳时有喷乳感、哺乳后乳房变软等，都是泌乳良好的表现。还应询问是否存在乳头疼痛或乳房问题。如果哺乳时持续明显疼痛，通常提示含接不正确。  在观察方面，首先要评估哺乳姿势。婴儿应紧贴母亲身体，头部与身体保持一条直线。其次，需要重点观察含接。有效含接时，婴儿嘴巴张大，下唇外翻，含住大部分乳晕，而不仅仅是乳头。吸吮动作应有节律，并能看到或听到吞咽动作。如果只是浅吸或频繁脱离乳房，则说明吸吮效率较低。婴儿吃奶后的状态也是重要指标。有效喂养后，婴儿通常会表现满足、安静，能够自然停止吸吮。另外，需要观察婴儿排尿和排便。出生后数日内，如果每天尿量达到6次以上，大便呈黄色，说明摄入基本充足。体重增长情况是最客观的评估指标之一。如果体重恢复和增长正常，通常说明母乳摄入足够。  同时，还要观察母亲乳房是否得到充分排空。长期乳房胀硬可能提示乳汁排出不足。  因此，母乳喂养有效性的评估，既要看“喂养过程”，也要看“婴儿结果”，需要动态综合判断。 |
| 中文回答的英文翻译 English translation of Chinese response | Assessing the effectiveness of breastfeeding is a critical component of postpartum care for both mother and infant. Successful breastfeeding not only ensures the newborn’s nutritional intake but also influences the establishment of milk supply and the mother’s breast health. Therefore, healthcare providers must conduct a comprehensive assessment that considers the mother, the infant, and the breastfeeding process.  First, inquire about the frequency and duration of feedings. A healthy newborn typically feeds 8 to 12 times a day, on demand. If feedings are too infrequent or the intervals between them are too long, this can easily lead to insufficient milk production or milk stasis. Second, it is necessary to determine whether the mother experiences the sensation of “milk let-down.” For example, softening of the breasts during feeding, milk flow, or milk leakage from the opposite breast all indicate that lactation has been established well. The mother should also be asked whether she experiences pain during breastfeeding. Mild discomfort is acceptable, but significant nipple pain or cracking often indicates an incorrect latch. Observing the feeding position and latch is a key focus. During an effective feed, the infant should be close to the mother, with the head, shoulders, and hips aligned in a straight line; the infant’s mouth should be wide open, the lower lip everted, the chin close to the breast, and the infant should be latching onto most of the areola, not just the nipple.  Second, observe the infant’s sucking pattern. Effective sucking typically involves rhythmic, deep sucks, accompanied by audible swallowing sounds. Rapid, shallow sucks or frequent release of the nipple suggest inefficient feeding. Additionally, observe the infant’s state after feeding. Following an effective feeding, the infant usually appears satisfied, calm, and is able to release the nipple on their own and fall asleep. The infant’s urination and bowel movements are also important indicators. Generally, within a few days after birth, the number of wet diapers gradually increases to more than six per day, and the color of the stool changes from meconium to a yellow, paste-like consistency, indicating adequate intake. Weight changes are equally critical. Newborns may experience physiological weight loss shortly after birth, but they typically return to their birth weight within 10 to 14 days. If weight continues to decline, it is important to be vigilant for signs of inadequate feeding.  Additionally, the condition of the breasts should be observed. After an effective feeding, the breasts are usually softer than before; if there is prolonged tenderness or noticeable hard lumps, this suggests that the breasts are not being fully emptied.  Therefore, when assessing whether breastfeeding is effective, it is not enough to simply look at “whether feeding has occurred”; one must also focus on whether the infant is truly receiving sufficient milk and whether the mother has established a good breastfeeding routine. | To determine whether breastfeeding is successful, it is necessary to evaluate multiple aspects of the mother, the infant, and the breastfeeding process. Only when both mother and baby are well-coordinated can breastfeeding be considered successful.  First, inquire about the frequency of feedings. Newborns typically need to be fed on demand, approximately 8 to 12 times a day. If the number of feedings is significantly lower, it may affect milk production and the infant’s intake. Second, the mother’s milk supply should be assessed. For example, check for breast fullness, the let-down reflex, and softening of the breasts after feeding. Also inquire whether the mother experiences nipple pain, cracking, or breast tenderness. Persistent pain during breastfeeding often indicates issues with positioning or latch. Observing the feeding position is a critical step. The mother should be in a comfortable and relaxed state, with the baby’s body close to her and the baby’s head not turned to the side. The latch is particularly critical. During an effective latch, the baby’s mouth is wide open, the lower lip is everted, the chin is close to the breast, and a significant portion of the areola is taken into the mouth.  Regarding sucking, normal and effective sucking should be slow, strong, and rhythmic, and swallowing sounds should be audible. If the infant lets go after a few sucks, cries frequently, or feeds for an extended period without appearing satisfied, this may indicate insufficient feeding. Additionally, observe the infant’s behavior after feeding. After an effective feeding, the infant is typically calm, content, and able to fall asleep naturally.  Urination and bowel movements are also important indicators. Adequate urine output and stools that gradually turn yellow indicate good milk intake.  Weight monitoring serves as an objective indicator. If the infant is gaining weight normally, this generally indicates that breastfeeding is effective.  At the same time, the condition of the mother’s breasts should be observed. After breastfeeding, the breasts should feel softer than before, with no significant tenderness or lumps.  Therefore, assessing the effectiveness of breastfeeding requires a comprehensive evaluation that combines “the mother’s feelings, the infant’s behavior, and objective indicators.” | Assessing the effectiveness of breastfeeding is a crucial component of postpartum care. Its core objective is to determine whether the infant is receiving sufficient milk and whether the mother is breastfeeding successfully.  First, the mother should be asked about her breastfeeding routine, including the number of feedings per day, the duration of each feeding, and whether she breastfeeds at night. Newborns generally require frequent, on-demand feedings. Second, assess the mother’s perception of her milk supply. For example, breast fullness before feeding, a let-down sensation during feeding, and softening of the breasts after feeding are all signs of good lactation. Also inquire about any nipple pain or breast issues. Persistent, significant pain during feeding usually indicates an incorrect latch.  In terms of observation, first evaluate the feeding position. The infant should be held close to the mother’s body, with the head and body aligned in a straight line. Next, focus on observing the latch. During an effective latch, the infant’s mouth is wide open, the lower lip is everted, and the baby takes in most of the areola, not just the nipple. Sucking should be rhythmic, and swallowing should be visible or audible. If the infant only sucks shallowly or frequently releases the breast, this indicates poor sucking efficiency. The infant’s state after feeding is also a key indicator. Following an effective feeding, the infant typically appears satisfied and calm, and is able to stop sucking naturally. Additionally, the infant’s urination and bowel movements should be observed. Within the first few days after birth, if the infant urinates more than six times a day and the stool is yellow, this indicates that intake is generally adequate. Weight gain is one of the most objective assessment indicators. If weight recovery and gain are normal, this usually indicates sufficient breast milk intake.  At the same time, it is important to observe whether the mother’s breasts are being fully emptied. Persistent breast engorgement may indicate insufficient milk ejection.  Therefore, assessing the effectiveness of breastfeeding requires evaluating both the “feeding process” and the “infant’s outcome,” necessitating a dynamic and comprehensive evaluation. |
| Correctness（1-5） | 4 | 4 | 4 |
| Completeness（1-5） | 4 | 4 | 4 |
| Guideline consistency（1-5） | 4 | 4 | 4 |
| Safety（1-5） | 4 | 4 | 4 |
| Clinical management appropriateness（1-5） | 4 | 3 | 3 |
| Chinese Reading Difficulty Score | 13.31 | 13.11 | 13.31 |
| Corresponding Education Level (Chinese) | 初中学段 | 初中学段 | 初中学段 |
| Recommended Reading Age (Chinese) | 13岁左右 | 13岁左右 | 13岁左右 |

## 问题 18

| 项目 | 第1次 | 第2次 | 第3次 |
| --- | --- | --- | --- |
| 产褥期临床知识问题 | 产褥期哺乳妇女需要用药时，临床上应如何评估药物安全性和哺乳风险？ | | |
| 主要指南来源 | NICE NG194 | | |
| 模型回答 | 产褥期哺乳妇女在使用药物时，需要同时考虑母亲治疗需求和婴儿安全，因此临床上必须进行系统的药物安全性评估。总体原则是在保证母体疾病得到有效治疗的前提下，尽量减少药物通过乳汁对婴儿产生的不良影响。  首先，应评估是否确实需要用药。对于轻症或可通过非药物方式改善的问题，应优先选择非药物干预，例如物理治疗、饮食调整或局部护理等，以减少婴儿暴露风险。  其次，需要评估药物本身特点。重点包括：药物是否容易进入乳汁；乳汁中的药物浓度高低；药物半衰期长短；药物口服吸收率；以及对婴儿可能产生的毒性。一般来说，分子量较小、脂溶性高、蛋白结合率低的药物更容易进入乳汁。  第三，应评估婴儿情况。新生儿尤其是早产儿、低体重儿及肝肾功能尚未成熟的婴儿，对药物更敏感，因此风险更高。  临床上通常优先选择哺乳期相对安全、研究资料较充分的药物。例如：退热镇痛常选对乙酰氨基酚或布洛芬；多数青霉素类和头孢类抗生素也相对安全。  而对于可能影响婴儿神经系统、呼吸系统或生长发育的药物，则需谨慎使用。例如：部分阿片类镇痛药可能导致婴儿嗜睡和呼吸抑制；某些化疗药物、放射性药物及部分精神类药物，通常不建议哺乳期使用。  第四，应评估用药时间和剂量。原则上应使用最低有效剂量和最短疗程。  在时间安排上，通常建议母亲在哺乳后立即服药，这样下一次哺乳时乳汁中的药物浓度相对较低。  此外，还应密切观察婴儿反应。包括是否出现异常嗜睡、喂养困难、腹泻、皮疹或体重增长异常等。  必要时，可参考专业数据库或指南，如哺乳期药物安全分级资料，以辅助决策。  如果药物风险较高但又必须使用，则需评估是否暂停哺乳，并指导产妇规律排乳以维持泌乳。  因此，哺乳期用药管理强调“个体化风险—获益评估”，既不能因担心哺乳而拒绝必要治疗，也不能忽视药物对婴儿的潜在影响。 | 哺乳期妇女用药时，药物不仅作用于母体，还可能通过乳汁进入婴儿体内，因此临床上需要综合评估药物安全性和哺乳风险。  首先，应明确母亲是否必须使用该药。对于轻度不适，如果可以通过休息、饮食调整或局部治疗改善，则应尽量避免系统性用药。其次，要评估药物进入乳汁的可能性。药物是否进入乳汁，与其分子量、脂溶性及蛋白结合率有关。一般脂溶性高、半衰期长的药物，更容易在乳汁中聚集。第三，需要评估药物对婴儿的潜在影响。重点关注是否可能影响婴儿神经系统、呼吸系统、肝肾功能及生长发育。  新生儿尤其是早产儿代谢能力较弱，因此对药物更敏感。  临床上通常优先选择哺乳期安全性较高的药物。例如：青霉素类、头孢类抗生素；布洛芬、对乙酰氨基酚等止痛退热药。  而对于某些高风险药物，则需谨慎。例如：化疗药物；放射性药物；部分抗癫痫药及镇静催眠药。  此外，还应关注药物剂量和疗程。尽量采用最小有效剂量，并缩短用药时间。  在给药时间安排上，一般建议在哺乳后立即服药，避免药物浓度高峰期喂奶。  临床中，还应动态观察婴儿状态。如果婴儿出现嗜睡、吃奶差、呕吐、腹泻或异常烦躁，应及时评估是否与药物有关。  对于必须使用高风险药物的母亲，可能需要暂时停止母乳喂养，并指导规律排乳维持泌乳功能。  因此，哺乳期用药管理需要兼顾母亲治疗需求和婴儿安全，核心是“风险最小化”。 | 产褥期哺乳妇女用药时，临床评估的重点是药物是否会通过乳汁影响婴儿，同时还要兼顾母亲疾病治疗的必要性。  首先，应评估母体疾病严重程度。如果疾病较轻，可优先考虑非药物治疗；若必须用药，则不能因为哺乳而延误治疗。  其次，需要分析药物特性。包括：药物进入乳汁的比例；药物半衰期；是否容易被婴儿吸收；以及是否存在已知婴儿毒性。一般而言，半衰期短、蛋白结合率高的药物，对哺乳影响相对较小。  第三，应评估婴儿情况。足月健康婴儿对少量药物耐受较好，但早产儿、新生儿或患病婴儿风险更高。  临床上提倡优先使用已有较多哺乳期安全数据的药物。例如多数常用抗生素和解热镇痛药，在规范使用下通常可以继续哺乳。  对于可能导致婴儿中枢抑制、肝肾损伤或骨髓抑制的药物，则应严格评估风险。  此外，还要注意用药方式。局部用药通常比口服或静脉用药风险更低。用药时应采用最低有效剂量和最短疗程，并尽量避开哺乳高峰期。  临床中，还应指导母亲观察婴儿表现。例如是否出现嗜睡、吃奶减少、黄疸加重或腹泻等。如果使用的是高风险药物，则需考虑暂停母乳喂养，并做好替代喂养指导。  因此，哺乳期药物使用不是简单“能不能用”的问题，而是一个综合的风险—收益评估过程。 |
| 中文回答的英文翻译 English translation of Chinese response | When prescribing medications for breastfeeding women during the postpartum period, both the mother’s treatment needs and the infant’s safety must be considered; therefore, a systematic assessment of drug safety is essential in clinical practice. The overarching principle is to ensure effective treatment of the mother’s condition while minimizing any adverse effects on the infant caused by the drug passing into breast milk.  First, the necessity of medication should be assessed. For mild conditions or issues that can be improved through non-pharmacological means, non-pharmacological interventions—such as physical therapy, dietary adjustments, or local care—should be prioritized to reduce the infant’s exposure risk.  Second, the characteristics of the drug itself must be evaluated. Key considerations include: whether the drug readily enters breast milk; the concentration of the drug in breast milk; the drug’s half-life; oral bioavailability; and potential toxicity to the infant. Generally, drugs with a small molecular weight, high lipid solubility, and low protein binding are more likely to enter breast milk.  Third, the infant’s condition should be assessed. Newborns—especially preterm infants, low-birth-weight infants, and those with immature liver and kidney function—are more sensitive to medications and therefore face higher risks.  Clinically, medications that are relatively safe during breastfeeding and have sufficient research data are typically prioritized. For example, acetaminophen or ibuprofen are commonly chosen for fever reduction and pain relief; most penicillin and cephalosporin antibiotics are also relatively safe.  However, drugs that may affect the infant’s nervous system, respiratory system, or growth and development should be used with caution. For example, some opioid analgesics may cause drowsiness and respiratory depression in infants; certain chemotherapy drugs, radiopharmaceuticals, and some psychiatric medications are generally not recommended during breastfeeding.  Fourth, the timing and dosage of medication should be evaluated. In principle, the lowest effective dose and the shortest possible course of treatment should be used.  Regarding timing, it is generally recommended that mothers take medication immediately after breastfeeding, so that the drug concentration in breast milk is relatively low by the next feeding.  In addition, the infant’s response should be closely monitored. This includes checking for abnormal drowsiness, feeding difficulties, diarrhea, rashes, or abnormal weight gain.  If necessary, professional databases or guidelines, such as drug safety classification resources for breastfeeding, can be consulted to aid decision-making.  If a medication carries a high risk but is essential, it is necessary to assess whether breastfeeding should be temporarily suspended and to instruct the mother to express milk regularly to maintain lactation.  Therefore, medication management during breastfeeding emphasizes an “individualized risk-benefit assessment”; necessary treatment should not be refused out of concern for breastfeeding, nor should the potential effects of the medication on the infant be overlooked. | When prescribing medications to breastfeeding women, it is important to note that drugs not only affect the mother but may also pass into the infant’s body through breast milk. Therefore, a comprehensive clinical assessment of drug safety and the risks associated with breastfeeding is necessary.  First, it should be determined whether the mother must take the medication. For mild discomfort that can be alleviated through rest, dietary adjustments, or topical treatment, systemic medication should be avoided whenever possible. Second, the likelihood of the drug entering breast milk must be assessed. Whether a drug enters breast milk depends on its molecular weight, lipophilicity, and protein binding rate. Generally, drugs with high lipophilicity and long half-lives are more likely to accumulate in breast milk. Third, the potential effects of the drug on the infant must be evaluated. Particular attention should be paid to whether the drug may affect the infant’s nervous system, respiratory system, liver and kidney function, and growth and development.  Newborns, especially preterm infants, have weaker metabolic capacity and are therefore more sensitive to drugs.  Clinically, drugs with a higher safety profile during breastfeeding are typically prioritized. Examples include penicillin and cephalosporin antibiotics, as well as analgesics and antipyretics such as ibuprofen and acetaminophen.  However, caution is required with certain high-risk drugs, such as chemotherapy agents, radiopharmaceuticals, and some antiepileptic and sedative-hypnotic medications.  Additionally, attention should be paid to drug dosage and duration of treatment. The lowest effective dose should be used whenever possible, and the duration of treatment should be minimized.  Regarding the timing of administration, it is generally recommended to take medication immediately after breastfeeding to avoid feeding during the peak drug concentration period.  Clinically, the infant’s condition should also be monitored closely. If the infant exhibits drowsiness, poor feeding, vomiting, diarrhea, or unusual irritability, a prompt assessment should be made to determine whether these symptoms are related to the medication.  For mothers who must use high-risk medications, it may be necessary to temporarily discontinue breastfeeding, while providing guidance on regular milk expression to maintain lactation.  Therefore, medication management during breastfeeding must balance the mother’s treatment needs with the infant’s safety, with the core principle being “risk minimization.” | When prescribing medications for breastfeeding women during the postpartum period, the clinical assessment focuses on whether the drug will affect the infant through breast milk, while also considering the necessity of treating the mother’s condition.  First, the severity of the mother’s condition should be assessed. If the condition is mild, non-pharmacological treatments should be prioritized; if medication is necessary, treatment should not be delayed due to breastfeeding.  Second, the characteristics of the drug must be analyzed. These include: the proportion of the drug that enters breast milk; the drug’s half-life; its ease of absorption by the infant; and whether there is known infant toxicity. Generally, drugs with a short half-life and high protein binding have a relatively minor impact on breastfeeding.  Third, the infant’s condition should be assessed. Full-term, healthy infants tolerate small amounts of medication well, but preterm infants, newborns, or sick infants are at higher risk.  Clinically, it is recommended to prioritize drugs with extensive safety data regarding use during breastfeeding. For example, most commonly used antibiotics and antipyretic analgesics can generally be continued while breastfeeding when used appropriately.  For drugs that may cause central nervous system depression, liver or kidney damage, or bone marrow suppression in infants, the risks should be strictly evaluated.  In addition, attention should be paid to the route of administration. Topical administration generally carries lower risks than oral or intravenous administration. The lowest effective dose and the shortest possible course of treatment should be used, and breastfeeding should be avoided during peak milk production times whenever possible.  Clinically, mothers should also be instructed to monitor their infants for signs such as drowsiness, decreased feeding, worsening jaundice, or diarrhea. If high-risk medications are used, consideration should be given to temporarily suspending breastfeeding, and guidance on alternative feeding methods should be provided.  Therefore, the use of medications during lactation is not simply a matter of “whether or not to use them,” but rather a comprehensive risk-benefit assessment process. |
| Correctness（1-5） | 4 | 4 | 4 |
| Completeness（1-5） | 4 | 4 | 3 |
| Guideline consistency（1-5） | 4 | 4 | 5 |
| Safety（1-5） | 4 | 4 | 4 |
| Clinical management appropriateness（1-5） | 5 | 4 | 3 |
| Chinese Reading Difficulty Score | 13.30 | 13.25 | 13.07 |
| Corresponding Education Level (Chinese) | 初中学段 | 初中学段 | 初中学段 |
| Recommended Reading Age (Chinese) | 13岁左右 | 13岁左右 | 13岁左右 |

## 问题 19

| 项目 | 第1次 | 第2次 | 第3次 |
| --- | --- | --- | --- |
| 产褥期临床知识问题 | 产后尿潴留、尿痛、尿频或尿失禁分别可能提示哪些问题？应如何进行初步处理和转诊判断？ | | |
| 主要指南来源 | NICE NG194 | | |
| 模型回答 | 产后泌尿系统问题较为常见，包括尿潴留、尿痛、尿频及尿失禁等。这些症状可能与分娩损伤、感染或盆底功能障碍有关。及时识别和处理，有助于预防长期并发症。  首先，产后尿潴留是指产后不能自行排尿或排尿后膀胱仍有大量残余尿。常见原因包括：会阴水肿或疼痛导致排尿反射受抑；硬膜外麻醉影响膀胱功能；膀胱过度充盈；盆底及神经损伤。  患者常表现为排尿困难、下腹胀满、尿量少但有尿意。严重时可出现膀胱明显膨隆。  初步处理包括鼓励早下床活动、听流水声、温水冲洗会阴等促进排尿措施。若仍无法排尿，应及时导尿。若反复尿潴留或残余尿较多，则需进一步转诊评估。  其次，尿痛和尿频多提示泌尿系统感染。由于分娩后免疫力下降、导尿操作及会阴损伤，产妇容易发生尿路感染。患者常表现为排尿疼痛、尿急、尿频，严重时可伴发热和腰痛。初步处理应鼓励多饮水、保持会阴清洁，并完善尿常规检查。若存在明显感染症状，则需给予抗感染治疗。若伴高热、腰痛或寒战，应警惕肾盂肾炎并及时转诊。  第三，产后尿失禁主要与盆底肌和尿道支持结构损伤有关。阴道分娩、巨大儿及产程延长均为高危因素。最常见的是压力性尿失禁，即咳嗽、打喷嚏或活动时漏尿。初步处理以盆底康复训练为主，例如凯格尔运动。同时应指导避免长期憋尿和便秘。如果尿失禁持续时间较长、症状明显影响生活，或伴盆腔器官脱垂，则需转诊盆底康复或泌尿妇科专科。  此外，如果产妇出现持续不能排尿、血尿、严重腰痛或反复感染，也应及时进一步检查。  因此，产后泌尿系统症状虽然常见，但不同表现对应不同病理问题，需要根据症状严重程度及时处理和判断是否转诊。 | 产后女性由于盆底组织松弛、分娩损伤及导尿等因素，容易出现各种排尿异常。不同症状往往提示不同临床问题。  产后尿潴留通常提示膀胱功能暂时受损。常见原因包括会阴疼痛、盆底水肿、神经牵拉损伤以及麻醉影响。患者可能表现为排尿困难、尿意明显却排不出尿，或排尿后仍有下腹胀感。  处理上应先采取非侵入性措施，例如鼓励排尿、热敷下腹、温水刺激等。如果超过一定时间仍不能自行排尿，则需导尿。若导尿后仍反复发生尿潴留，则需进一步转诊评估神经或盆底功能。  尿痛和尿频则常提示尿路感染。特别是留置导尿后，更容易发生细菌感染。患者多伴尿急、排尿烧灼感，严重时可出现发热和腰痛。初步处理包括增加饮水量、保持会阴清洁，并根据尿检结果给予抗生素治疗。如果患者出现高热、寒战、腰部叩击痛，则需考虑上尿路感染并及时转诊。  产后尿失禁主要提示盆底支持结构受损。最常见的是压力性尿失禁，即腹压增加时漏尿。多数轻度患者可通过盆底肌训练逐渐恢复。但如果漏尿严重、持续时间长，或伴阴道膨出感，则可能存在盆底功能障碍，需要专科康复治疗。此外，若出现持续血尿、排尿剧烈疼痛或尿液明显异常，也需要进一步检查。  因此，产后排尿异常虽然部分属于暂时性功能变化，但也可能提示感染或盆底损伤，应重视早期识别。 | 产后泌尿系统症状较常见，主要与分娩损伤、盆底松弛及感染有关。不同症状提示的问题不同。尿潴留主要提示膀胱排空障碍。常见于会阴水肿、产程过长、硬膜外麻醉后或盆底神经受损。  患者通常表现为排尿困难、尿不尽感及下腹胀痛。  初步处理包括鼓励尽早排尿、热敷膀胱区、流水刺激及减轻疼痛。如果仍无法排尿，则需导尿。若尿潴留持续存在或反复发生，应转诊进一步评估。尿频和尿痛最常提示尿路感染。特别是产后导尿、会阴损伤及卫生不良时风险更高。患者会出现尿急、烧灼感及小便次数增多。  处理上应增加饮水、完善尿常规检查，并根据情况给予抗感染治疗。若伴发热、寒战或腰痛，应警惕肾盂肾炎，需要及时转诊。尿失禁则多与盆底损伤有关。阴道分娩后盆底肌松弛，可导致咳嗽或运动时漏尿。轻度患者通常通过凯格尔运动等盆底康复训练改善。若症状严重、长期不恢复或伴盆腔器官脱垂，则需转诊盆底专科。  此外，还应注意是否存在持续血尿、明显残余尿感或神经症状，因为这些可能提示更严重问题。  因此，产后排尿问题既可能是短暂功能改变，也可能提示感染或盆底损伤，需结合症状严重程度及时处理与转诊。 |
| 中文回答的英文翻译 English translation of Chinese response | Postpartum urinary problems are relatively common and include urinary retention, dysuria, frequent urination, and urinary incontinence. These symptoms may be related to birth injuries, infections, or pelvic floor dysfunction. Timely identification and management can help prevent long-term complications.  First, postpartum urinary retention refers to the inability to urinate spontaneously after childbirth or the presence of a large volume of residual urine in the bladder after urination. Common causes include: inhibition of the micturition reflex due to perineal edema or pain; effects of epidural anesthesia on bladder function; bladder overdistension; and pelvic floor or nerve injury.  Patients often present with difficulty urinating, lower abdominal distension, and a strong urge to urinate despite producing only a small amount of urine. In severe cases, the bladder may become noticeably distended.  Initial management includes measures to promote urination, such as encouraging early ambulation, listening to the sound of running water, and washing the perineum with warm water. If urination remains impossible, catheterization should be performed promptly. If urinary retention recurs or residual urine volume is high, the patient should be referred for further evaluation.  Second, dysuria and frequent urination often indicate a urinary tract infection. Due to decreased immunity after childbirth, catheterization procedures, and perineal trauma, postpartum women are prone to urinary tract infections. Patients often present with painful urination, urinary urgency, and frequent urination; severe cases may be accompanied by fever and lower back pain. Initial management should include encouraging increased fluid intake, maintaining perineal hygiene, and performing a urinalysis. If significant signs of infection are present, antimicrobial therapy should be initiated. If accompanied by high fever, lower back pain, or chills, pyelonephritis should be suspected, and the patient should be promptly referred.  Third, postpartum urinary incontinence is primarily associated with damage to the pelvic floor muscles and urethral support structures. Vaginal delivery, macrosomia, and prolonged labor are all risk factors. The most common type is stress urinary incontinence, characterized by urine leakage during coughing, sneezing, or physical activity. Initial management focuses on pelvic floor rehabilitation exercises, such as Kegel exercises. Patients should also be advised to avoid prolonged urine retention and constipation. If urinary incontinence persists for an extended period, significantly impacts daily life, or is accompanied by pelvic organ prolapse, referral to a pelvic floor rehabilitation specialist or a urogynecologist is necessary.  Additionally, if a postpartum woman experiences persistent inability to urinate, hematuria, severe lower back pain, or recurrent infections, further evaluation should be conducted promptly.  Therefore, while postpartum urinary symptoms are common, different presentations correspond to different pathological issues. Timely management and determination of whether referral is necessary should be based on the severity of symptoms. | Postpartum women are prone to various urinary abnormalities due to factors such as pelvic floor tissue laxity, birth-related injuries, and catheterization. Different symptoms often indicate different clinical issues.  Postpartum urinary retention typically indicates temporary bladder dysfunction. Common causes include perineal pain, pelvic floor edema, nerve strain injuries, and the effects of anesthesia. Patients may experience difficulty urinating, a strong urge to urinate without being able to do so, or a sensation of lower abdominal distension after urination.  Management should initially involve non-invasive measures, such as encouraging urination, applying heat to the lower abdomen, or using warm water stimulation. If the patient is unable to urinate spontaneously after a certain period, catheterization is necessary. If urinary retention recurs after catheterization, the patient should be referred for further evaluation of neurological or pelvic floor function.  Dysuria and frequent urination often indicate a urinary tract infection. Bacterial infections are particularly common following indwelling catheterization. Patients typically experience urinary urgency and a burning sensation during urination; in severe cases, fever and lower back pain may occur. Initial management includes increasing fluid intake, maintaining perineal hygiene, and administering antibiotic therapy based on urine test results. If the patient develops a high fever, chills, or tenderness on percussion of the lower back, an upper urinary tract infection should be considered, and the patient should be promptly referred for evaluation.  Postpartum urinary incontinence primarily indicates damage to the pelvic floor support structures. The most common type is stress urinary incontinence, characterized by urine leakage during increased abdominal pressure. Most patients with mild symptoms can gradually recover through pelvic floor muscle exercises. However, if leakage is severe, persistent, or accompanied by a sensation of vaginal prolapse, pelvic floor dysfunction may be present, requiring specialized rehabilitation treatment. Additionally, further evaluation is necessary if persistent hematuria, severe pain during urination, or significant abnormalities in urine appearance occur.  Therefore, while some postpartum urinary abnormalities are temporary functional changes, they may also indicate infection or pelvic floor injury, and early identification should be prioritized. | Postpartum urinary symptoms are relatively common and are primarily associated with birth trauma, pelvic floor laxity, and infection. Different symptoms indicate different underlying issues. Urinary retention primarily indicates an inability to empty the bladder. It is commonly seen in cases of perineal edema, prolonged labor, following epidural anesthesia, or pelvic floor nerve damage.  Patients typically present with difficulty urinating, a sensation of incomplete emptying, and lower abdominal distension and pain.  Initial management includes encouraging early urination, applying heat to the bladder area, using running water to stimulate urination, and alleviating pain. If urination remains impossible, catheterization is necessary. If urinary retention persists or recurs, the patient should be referred for further evaluation. Frequent urination and dysuria most commonly indicate a urinary tract infection. The risk is particularly higher following postpartum catheterization, perineal trauma, or poor hygiene. Patients may experience urinary urgency, a burning sensation, and increased urinary frequency.  Management should involve increasing fluid intake, performing a urinalysis, and administering antimicrobial therapy as indicated. If accompanied by fever, chills, or lower back pain, pyelonephritis should be suspected, and prompt referral is necessary. Urinary incontinence is often associated with pelvic floor injury. Pelvic floor muscle laxity following vaginal delivery can lead to urine leakage during coughing or physical activity. Mild cases typically improve with pelvic floor rehabilitation exercises such as Kegel exercises. If symptoms are severe, persist for a long time, or are accompanied by pelvic organ prolapse, referral to a pelvic floor specialist is necessary.  Additionally, attention should be paid to the presence of persistent hematuria, a marked sensation of residual urine, or neurological symptoms, as these may indicate more serious underlying issues.  Therefore, postpartum urinary issues may represent either transient functional changes or may indicate an infection or pelvic floor injury; timely management and referral should be based on the severity of symptoms. |
| Correctness（1-5） | 4 | 4 | 4 |
| Completeness（1-5） | 4 | 4 | 3 |
| Guideline consistency（1-5） | 4 | 4 | 4 |
| Safety（1-5） | 5 | 4 | 4 |
| Clinical management appropriateness（1-5） | 4 | 4 | 3 |
| Chinese Reading Difficulty Score | 13.51 | 13.67 | 13.57 |
| Corresponding Education Level (Chinese) | 初中学段 | 初中学段 | 初中学段 |
| Recommended Reading Age (Chinese) | 13岁左右 | 14岁左右 | 13岁左右 |

## 问题 20

| 项目 | 第1次 | 第2次 | 第3次 |
| --- | --- | --- | --- |
| 产褥期临床知识问题 | 产后便秘和痔疮的常见诱因有哪些？临床护理和健康教育应包括哪些内容？ | | |
| 主要指南来源 | 中国孕产妇健康管理服务规范；NICE NG194 | | |
| 模型回答 | 产后便秘和痔疮是产褥期非常常见的问题，尤其在阴道分娩后更容易发生。其发生与妊娠期生理变化、分娩损伤以及产后活动减少等多种因素有关。  产后便秘的常见诱因首先是肠蠕动减慢。妊娠及分娩后胃肠功能恢复较慢，加上产妇疲劳、卧床时间长，容易导致排便困难。其次，会阴疼痛也是重要原因。会阴侧切或裂伤产妇因害怕疼痛，不敢主动排便，从而导致粪便滞留。第三，饮水不足和膳食纤维摄入不足也十分常见。部分产妇存在传统“坐月子”观念，饮食过于精细，水果蔬菜摄入少。此外，剖宫产术后疼痛、麻醉影响及活动减少，也会进一步加重便秘。  痔疮则主要与妊娠和分娩过程中盆腔静脉压力增高有关。妊娠期子宫压迫静脉回流，容易形成痔静脉曲张。分娩时长时间用力屏气，会进一步加重痔疮脱出和水肿。便秘导致排便用力，也会形成恶性循环，使痔疮症状加重。在临床护理方面，首先应鼓励产妇尽早下床活动。适度活动能够促进肠蠕动，减少便秘发生。  其次，应指导合理饮食。建议增加膳食纤维摄入，例如蔬菜、水果、粗粮等，同时保证充足饮水。对于排便困难者，应建立规律排便习惯，避免长期忍便。会阴疼痛明显时，可指导采取舒适体位，必要时适当镇痛，以减轻排便恐惧。  对于痔疮护理，可采用温水坐浴促进局部血液循环，缓解疼痛和水肿。保持肛周清洁也非常重要，尤其在排便后及时清洗，避免局部刺激。必要时可在医生指导下使用软便剂或局部外用药物。健康教育中，还应强调避免长时间久坐和过度用力排便。如果出现严重便血、痔核嵌顿、持续剧烈疼痛或长期便秘不缓解，则需进一步就医或转诊。  因此，产后便秘和痔疮的护理重点在于“促进排便、减轻压力、改善生活方式”，以帮助产妇顺利恢复。 | 产后便秘和痔疮在产褥期较为普遍，其形成与妊娠、生理变化及分娩因素密切相关。  产后便秘最常见的诱因包括：第一，产后活动减少。很多产妇长时间卧床，肠道蠕动减慢，容易导致大便干结。第二，会阴切口或剖宫产疼痛。部分产妇因害怕疼痛而不敢排便。第三，饮食结构不合理。一些传统观念导致产妇水果、蔬菜摄入不足，膳食纤维缺乏。第四，饮水不足及失血后体液减少，也会导致粪便干硬。  痔疮则主要与盆底及静脉压力增加有关。妊娠期间盆腔静脉回流受阻，加上分娩时用力屏气，容易使原有痔疮加重或新发痔疮。  长期便秘和排便用力也是痔疮的重要诱因。  护理方面，首先应鼓励产妇早期活动。适度下床有助于肠道恢复。其次，要加强饮食指导。鼓励摄入富含纤维的食物，如蔬菜、水果及全谷类，并增加饮水量。对于便秘患者，应指导建立固定排便时间，避免过度用力。会阴疼痛明显者，可采取疼痛缓解措施，减轻排便恐惧。痔疮护理中，温水坐浴是常用方法，可改善局部血液循环并减轻疼痛。保持肛周清洁干燥也十分重要。必要时，可在医生指导下使用缓泻剂、软便剂或痔疮外用药。  健康教育还应包括：避免久坐；避免长期憋便；保持规律作息；加强盆底功能恢复。如果产妇出现大量便血、痔疮嵌顿、水肿严重或持续排便困难，则应及时转诊。因此，产后便秘和痔疮虽常见，但通过规范护理和生活方式调整，多数可以明显改善。 | 产后便秘和痔疮是很多产妇在产褥期会遇到的问题，其发生与分娩创伤、活动减少以及饮食改变等因素有关。  产后便秘的常见原因包括：肠蠕动恢复较慢；产后卧床时间长；会阴伤口疼痛导致不敢排便；饮水和膳食纤维摄入不足；剖宫产术后麻醉及止痛药影响。  痔疮则多与妊娠期盆腔压力增加及分娩时用力有关。怀孕期间静脉回流受阻容易形成痔静脉扩张，而分娩时长时间屏气会进一步加重。便秘导致反复用力排便，也会使痔疮恶化。  护理中，首先应鼓励产妇适当活动，促进胃肠功能恢复。其次，应指导合理饮食，包括增加水果、蔬菜、粗粮和水分摄入。对于排便困难者，可建议晨起温水刺激肠蠕动，并建立规律排便习惯。如果存在会阴疼痛，应积极缓解疼痛，减少对排便的恐惧。痔疮患者可采用温水坐浴，减轻局部肿胀和疼痛。同时要保持肛周卫生，避免局部感染和刺激。必要时，可遵医嘱使用软便药或局部药膏。  健康教育方面，应提醒产妇避免久坐久站、避免憋便以及避免过度用力排便。若出现严重便血、痔核脱出无法回纳、剧烈疼痛或长期顽固性便秘，则需要及时就医。  因此，产后便秘和痔疮的管理重点在于改善生活方式、促进肠道功能恢复及减轻局部压力。 |
| 中文回答的英文翻译 English translation of Chinese response | Postpartum constipation and hemorrhoids are very common issues during the postpartum period, and they are particularly likely to occur after vaginal delivery. Their occurrence is related to a variety of factors, including physiological changes during pregnancy, injuries sustained during childbirth, and reduced physical activity after delivery.  The primary cause of postpartum constipation is a slowing of intestinal motility. Gastrointestinal function recovers slowly after pregnancy and childbirth, and when combined with maternal fatigue and prolonged bed rest, this can easily lead to difficulty defecating. Second, perineal pain is another significant factor. Women who have undergone an episiotomy or suffered a perineal tear may avoid active bowel movements due to fear of pain, leading to fecal retention. Third, inadequate fluid intake and insufficient dietary fiber are also very common. Some women adhere to traditional “confinement” practices, resulting in overly refined diets with low intake of fruits and vegetables. Additionally, post-cesarean pain, the effects of anesthesia, and reduced physical activity can further exacerbate constipation.  Hemorrhoids are primarily associated with increased pelvic venous pressure during pregnancy and childbirth. During pregnancy, the uterus compresses venous return, making it easier for hemorrhoidal veins to become varicose. Prolonged straining during childbirth further exacerbates hemorrhoidal prolapse and edema. Constipation leads to straining during bowel movements, creating a vicious cycle that worsens hemorrhoidal symptoms. In terms of clinical care, the first step is to encourage new mothers to get out of bed and move around as soon as possible. Moderate activity promotes intestinal peristalsis and reduces the incidence of constipation.  Second, proper dietary guidance should be provided. It is recommended to increase dietary fiber intake—such as vegetables, fruits, and whole grains—while ensuring adequate fluid intake. For those experiencing difficulty with bowel movements, a regular bowel routine should be established to avoid prolonged suppression of the urge to defecate. When perineal pain is severe, patients can be advised to adopt a comfortable position and, if necessary, receive appropriate pain relief to alleviate fear of defecation.  For hemorrhoid care, warm water sitz baths can be used to promote local blood circulation and relieve pain and swelling. Maintaining perianal hygiene is also crucial, particularly by washing promptly after bowel movements to avoid local irritation. If necessary, stool softeners or topical medications may be used under a doctor’s guidance. Health education should also emphasize avoiding prolonged sitting and excessive straining during bowel movements. If severe rectal bleeding, prolapsed hemorrhoids, persistent severe pain, or chronic constipation that does not resolve occurs, further medical evaluation or referral is necessary.  Therefore, the focus of postpartum constipation and hemorrhoid care lies in “promoting bowel movements, reducing strain, and improving lifestyle” to help new mothers recover smoothly. | Postpartum constipation and hemorrhoids are common during the postpartum period, and their development is closely related to pregnancy, physiological changes, and factors associated with childbirth.  The most common causes of postpartum constipation include: First, reduced physical activity after childbirth. Many new mothers spend long periods in bed, which slows intestinal motility and can easily lead to hard, dry stools. Second, pain from perineal incisions or cesarean sections. Some new mothers are afraid to have a bowel movement due to fear of pain. Third, an unbalanced diet. Certain traditional beliefs lead to insufficient intake of fruits and vegetables, resulting in a lack of dietary fiber. Fourth, inadequate fluid intake and reduced body fluid volume following blood loss can also cause hard, dry stools.  Hemorrhoids are primarily associated with increased pressure in the pelvic floor and veins. During pregnancy, impaired pelvic venous return, combined with straining during childbirth, can easily exacerbate existing hemorrhoids or lead to new ones.  Chronic constipation and straining during bowel movements are also significant contributing factors to hemorrhoids.  In terms of care, first, new mothers should be encouraged to get up and move around early on. Moderate mobility helps restore bowel function. Second, dietary guidance should be emphasized. Encourage the consumption of fiber-rich foods, such as vegetables, fruits, and whole grains, and increase fluid intake. For patients with constipation, they should be advised to establish a regular bowel movement schedule and avoid excessive straining. For those with significant perineal pain, pain relief measures can be implemented to alleviate fear of defecation. In hemorrhoid care, warm water sitz baths are a common method that can improve local blood circulation and reduce pain. Keeping the perianal area clean and dry is also very important. If necessary, laxatives, stool softeners, or topical hemorrhoid medications may be used under a doctor’s guidance.  Health education should also include: avoiding prolonged sitting; avoiding prolonged holding of stools; maintaining a regular daily routine; and strengthening pelvic floor function. If a postpartum woman experiences significant rectal bleeding, prolapsed hemorrhoids, severe edema, or persistent difficulty with bowel movements, she should be referred promptly. Therefore, although postpartum constipation and hemorrhoids are common, most cases can be significantly improved through standardized care and lifestyle adjustments. | Postpartum constipation and hemorrhoids are common issues many new mothers face during the postpartum period, and their occurrence is associated with factors such as the trauma of childbirth, reduced physical activity, and dietary changes.  Common causes of postpartum constipation include: slow recovery of intestinal motility; prolonged bed rest after childbirth; fear of defecation due to pain from perineal wounds; insufficient intake of water and dietary fiber; and the effects of anesthesia and pain medication following a cesarean section.  Hemorrhoids, on the other hand, are often associated with increased pelvic pressure during pregnancy and straining during childbirth. Impaired venous return during pregnancy can lead to the dilation of hemorrhoidal veins, and prolonged straining during childbirth can further exacerbate this condition. Constipation, which leads to repeated straining during bowel movements, can also worsen hemorrhoids.  In terms of care, first, encourage the mother to engage in appropriate physical activity to promote the recovery of gastrointestinal function. Second, provide guidance on a balanced diet, including increasing intake of fruits, vegetables, whole grains, and fluids. For those experiencing difficulty with bowel movements, it is recommended to use warm water upon waking to stimulate intestinal peristalsis and establish a regular bowel routine. If perineal pain is present, active pain relief should be provided to reduce fear of defecation. Patients with hemorrhoids may use warm water sitz baths to alleviate local swelling and pain. At the same time, perianal hygiene should be maintained to prevent local infection and irritation. If necessary, laxatives or topical ointments may be used as directed by a physician.  In terms of health education, new mothers should be advised to avoid prolonged sitting or standing, holding in bowel movements, and straining during defecation. If severe rectal bleeding, prolapsed hemorrhoids that cannot be reduced, severe pain, or long-term, intractable constipation occurs, prompt medical attention is required.  Therefore, the key to managing postpartum constipation and hemorrhoids lies in improving lifestyle habits, promoting the recovery of bowel function, and reducing local pressure. |
| Correctness（1-5） | 4 | 4 | 4 |
| Completeness（1-5） | 4 | 4 | 3 |
| Guideline consistency（1-5） | 4 | 4 | 4 |
| Safety（1-5） | 4 | 4 | 4 |
| Clinical management appropriateness（1-5） | 4 | 4 | 3 |
| Chinese Reading Difficulty Score | 13.34 | 13.21 | 13.35 |
| Corresponding Education Level (Chinese) | 初中学段 | 初中学段 | 初中学段 |
| Recommended Reading Age (Chinese) | 13岁左右 | 13岁左右 | 13岁左右 |

## 问题 21

| 项目 | 第1次 | 第2次 | 第3次 |
| --- | --- | --- | --- |
| 产褥期临床知识问题 | 产后盆底功能障碍可表现为哪些症状？何时应建议盆底康复评估或转诊？ | | |
| 主要指南来源 | NICE NG194 | | |
| 模型回答 | 产后盆底功能障碍是女性分娩后较常见的问题，主要由于妊娠期间盆底长期受压以及分娩过程中肌肉、筋膜和神经损伤所致。如果未及时发现和干预，可能影响女性长期生活质量。  盆底功能障碍最常见的表现首先是尿失禁。尤其是压力性尿失禁，产妇在咳嗽、打喷嚏、提重物或运动时出现漏尿，这是盆底肌支持功能减弱的典型表现。其次，部分女性会出现尿频、尿急或排尿困难，提示盆底肌协调功能异常。第三，盆腔器官脱垂也是常见表现。患者可能感觉阴道内有“下坠感”“异物感”，严重时可见阴道口膨出。此外，还可能出现排便异常。例如便秘、排便费力、排便不尽感，部分患者甚至需要手法辅助排便。盆底功能障碍还可能影响性生活。患者可出现阴道松弛、性生活疼痛或性生活满意度下降。部分女性还会出现慢性盆底疼痛、腰骶部酸痛及会阴坠胀感。  高危因素包括：阴道分娩；巨大儿；产程延长；器械助产；多次分娩；严重会阴裂伤等。  对于何时进行盆底康复评估，一般建议产后42天复查时进行初步筛查。  如果产妇存在以下情况，更应尽早评估：持续漏尿；明显阴道坠胀感；排尿排便异常；严重会阴损伤；剖宫产合并孕期盆底症状；或长期盆底疼痛。  临床上通常可通过问诊、盆底肌力检查及专业盆底功能评估进行判断。轻度患者可通过凯格尔运动、生活方式调整及盆底肌训练改善。但如果症状明显影响生活，或保守训练效果差，则应转诊盆底康复中心、妇科或泌尿妇科专科进一步治疗。对于出现明显器官脱垂、严重尿失禁或神经损伤表现者，更需要早期专科干预。  因此，产后盆底功能障碍不仅仅是“漏尿”问题，而是涉及泌尿、生殖及生活质量的综合性问题，早期筛查和康复十分重要。 | 产后盆底功能障碍是由于妊娠和分娩导致盆底肌肉、筋膜及神经受损后出现的一系列功能异常，近年来越来越受到重视。  最常见症状是尿失禁。尤其是在咳嗽、跑跳或大笑时出现漏尿，属于典型压力性尿失禁。其次，部分女性会出现尿急、尿频或尿不尽感，提示盆底支持和控尿功能受损。盆腔器官脱垂也是重要表现之一。患者常感觉阴道有坠胀感、异物感，严重时甚至可见阴道膨出。在排便方面，可出现便秘、排便困难或排便不尽。部分女性还会出现性生活相关问题，如阴道松弛、性交疼痛或性生活满意度下降。此外，慢性盆底疼痛、腰骶酸痛及久站后下坠感，也可能与盆底功能障碍有关。  高危人群包括：经阴道分娩者；巨大儿分娩者；第二产程延长者；多胎妊娠及多次分娩女性；严重会阴裂伤患者。  临床上，一般建议所有产妇在产后42天复查时进行盆底功能初步评估。如果产妇已经出现漏尿、阴道膨出感或长期盆底不适，则应提前评估。  初步评估包括症状问诊、盆底肌力检测以及必要时盆底超声或电生理检查。轻度功能障碍可通过盆底肌训练、凯格尔运动和生活方式调整改善。若症状持续存在超过数月，或明显影响日常生活，应转诊盆底康复专科。对于明显盆腔器官脱垂或严重尿失禁患者，可能需要进一步专科治疗甚至手术干预。  因此，产后盆底问题应强调“早发现、早康复”，避免长期发展影响女性健康。 | 产后盆底功能障碍是产褥期较常见的问题，主要由于妊娠期盆底长期受压以及分娩损伤导致。  其最典型的症状是压力性尿失禁。很多女性在咳嗽、打喷嚏、抱孩子或运动时会出现漏尿。其次，还可能出现尿频、尿急和排尿困难等排尿异常。盆腔器官脱垂也是常见表现。患者会感觉阴道下坠、会阴胀痛，严重时可摸到阴道内突出物。部分患者会出现排便困难、便秘或排便不尽感。性生活方面，可能表现为阴道松弛、性交不适甚至疼痛。另外，一些女性会长期感觉腰骶部酸痛、盆底坠胀或会阴部不适。  导致盆底功能障碍的高危因素包括：阴道分娩；巨大儿；难产；器械助产；多次妊娠分娩；严重会阴裂伤等。  一般建议产后42天进行常规盆底功能筛查。如果产妇在产后早期已经出现明显漏尿、阴道坠胀或排便异常，则应尽早进行盆底康复评估。评估内容通常包括盆底肌力检查、盆底电生理检测及盆底超声。对于轻度问题，可指导进行凯格尔运动和盆底康复训练。若症状持续不缓解、严重影响生活或存在明显器官脱垂，则应转诊盆底康复中心或妇科专科。  因此，产后盆底功能障碍是一类需要长期关注的问题，及时评估和康复能够明显改善女性远期生活质量。 |
| 中文回答的英文翻译 English translation of Chinese response | Postpartum pelvic floor dysfunction is a common issue among women following childbirth, primarily caused by prolonged pressure on the pelvic floor during pregnancy and damage to muscles, fascia, and nerves during delivery. If not detected and addressed promptly, it can have a long-term impact on a woman’s quality of life.  The most common manifestation of pelvic floor dysfunction is urinary incontinence. Stress urinary incontinence, in particular, occurs when women experience urine leakage while coughing, sneezing, lifting heavy objects, or exercising—a classic sign of weakened pelvic floor muscle support. Additionally, some women may experience frequent urination, urinary urgency, or difficulty urinating, indicating impaired coordination of the pelvic floor muscles. Third, pelvic organ prolapse is also a common manifestation. Patients may feel a “sinking sensation” or “foreign body sensation” in the vagina; in severe cases, protrusion at the vaginal opening may be visible. Additionally, bowel dysfunction may occur, such as constipation, straining during bowel movements, or a sensation of incomplete evacuation; some patients may even require manual assistance to defecate. Pelvic floor dysfunction may also affect sexual function, leading to vaginal laxity, pain during intercourse, or decreased sexual satisfaction. Some women may also experience chronic pelvic floor pain, lumbosacral aching, and a sensation of heaviness in the perineum.  Risk factors include: vaginal delivery; macrosomia; prolonged labor; instrumental delivery; multiple pregnancies; and severe perineal tears.  Regarding the timing of pelvic floor rehabilitation assessment, it is generally recommended to conduct an initial screening during the 42-day postpartum follow-up visit.  Early assessment is particularly recommended if the mother experiences any of the following: persistent urinary incontinence; significant vaginal heaviness; abnormal urination or bowel movements; severe perineal injury; cesarean delivery combined with pelvic floor symptoms during pregnancy; or chronic pelvic floor pain.  Clinically, diagnosis is typically made through medical history, pelvic floor muscle strength testing, and specialized pelvic floor function assessment. Mild cases can be improved through Kegel exercises, lifestyle adjustments, and pelvic floor muscle training. However, if symptoms significantly impact daily life or conservative training proves ineffective, the patient should be referred to a pelvic floor rehabilitation center, gynecology department, or urogynecology specialist for further treatment. Early specialist intervention is particularly necessary for patients presenting with significant organ prolapse, severe urinary incontinence, or signs of nerve damage.  Therefore, postpartum pelvic floor dysfunction is not merely a matter of “urinary leakage,” but a comprehensive issue involving the urinary and reproductive systems as well as quality of life; early screening and rehabilitation are crucial. | Postpartum pelvic floor dysfunction refers to a series of functional abnormalities resulting from damage to the pelvic floor muscles, fascia, and nerves caused by pregnancy and childbirth, and has received increasing attention in recent years.  The most common symptom is urinary incontinence. Urine leakage that occurs particularly when coughing, running, jumping, or laughing is classified as typical stress urinary incontinence. Secondly, some women experience urinary urgency, frequency, or a sensation of incomplete emptying, indicating impaired pelvic floor support and urinary control. Pelvic organ prolapse is also a significant manifestation. Patients often feel a sensation of heaviness or a foreign body in the vagina; in severe cases, vaginal protrusion may even be visible. Regarding bowel movements, constipation, difficulty defecating, or a sensation of incomplete evacuation may occur. Some women may also experience sexual dysfunction, such as vaginal laxity, dyspareunia, or decreased sexual satisfaction. Additionally, chronic pelvic pain, lumbosacral aching, and a sensation of heaviness after prolonged standing may be related to pelvic floor dysfunction.  High-risk groups include: women who have delivered vaginally; those who have delivered macrosomic infants; those with prolonged second stage of labor; women with multiple pregnancies or multiple deliveries; and patients with severe perineal tears.  Clinically, it is generally recommended that all postpartum women undergo an initial pelvic floor function assessment during their 42-day postpartum follow-up visit. If a woman is already experiencing urinary incontinence, a sensation of vaginal bulging, or persistent pelvic floor discomfort, the assessment should be conducted earlier.  The initial assessment includes a symptom history, pelvic floor muscle strength testing, and, if necessary, pelvic floor ultrasound or electrophysiological testing. Mild dysfunction can be improved through pelvic floor muscle training, Kegel exercises, and lifestyle adjustments. If symptoms persist for more than a few months or significantly impact daily life, the patient should be referred to a pelvic floor rehabilitation specialist. For patients with significant pelvic organ prolapse or severe urinary incontinence, further specialized treatment or even surgical intervention may be required.  Therefore, the management of postpartum pelvic floor issues should emphasize “early detection and early rehabilitation” to prevent long-term complications that could affect women’s health. | Postpartum pelvic floor dysfunction is a common issue during the postpartum period, primarily caused by prolonged pressure on the pelvic floor during pregnancy and injuries sustained during childbirth.  The most typical symptom is stress urinary incontinence. Many women experience urine leakage when coughing, sneezing, holding a child, or exercising. Additionally, urinary abnormalities such as frequent urination, urinary urgency, and difficulty urinating may occur. Pelvic organ prolapse is also a common manifestation. Patients may feel a sensation of vaginal heaviness or swelling and pain in the perineum; in severe cases, a protrusion may be palpable within the vagina. Some patients may experience difficulty defecating, constipation, or a sensation of incomplete bowel movement. Regarding sexual activity, symptoms may include vaginal laxity, discomfort during intercourse, or even pain. Additionally, some women may experience chronic lower back and sacral pain, pelvic heaviness, or perineal discomfort.  High-risk factors for pelvic floor dysfunction include: vaginal delivery; macrosomia; difficult labor; instrumental delivery; multiple pregnancies and deliveries; and severe perineal tears.  It is generally recommended to undergo routine pelvic floor function screening 42 days postpartum. If a new mother experiences significant urinary incontinence, vaginal heaviness, or bowel dysfunction in the early postpartum period, a pelvic floor rehabilitation assessment should be conducted as soon as possible. The assessment typically includes pelvic floor muscle strength testing, pelvic floor electrophysiology, and pelvic floor ultrasound. For mild issues, guidance on Kegel exercises and pelvic floor rehabilitation training may be provided. If symptoms persist without improvement, significantly impact daily life, or indicate obvious organ prolapse, the patient should be referred to a pelvic floor rehabilitation center or a gynecological specialist.  Therefore, postpartum pelvic floor dysfunction is a condition requiring long-term attention; timely assessment and rehabilitation can significantly improve women’s long-term quality of life. |
| Correctness（1-5） | 4 | 4 | 4 |
| Completeness（1-5） | 4 | 4 | 3 |
| Guideline consistency（1-5） | 5 | 5 | 4 |
| Safety（1-5） | 4 | 4 | 4 |
| Clinical management appropriateness（1-5） | 4 | 4 | 3 |
| Chinese Reading Difficulty Score | 13.44 | 13.71 | 13.53 |
| Corresponding Education Level (Chinese) | 初中学段 | 初中学段 | 初中学段 |
| Recommended Reading Age (Chinese) | 13岁左右 | 14岁左右 | 13岁左右 |

## 问题 22

| 项目 | 第1次 | 第2次 | 第3次 |
| --- | --- | --- | --- |
| 产褥期临床知识问题 | 产褥期恢复运动和盆底肌训练时，应根据哪些因素判断开始时间和运动强度？ | | |
| 主要指南来源 | NICE NG194；WHO 2022 | | |
| 模型回答 | 产褥期恢复运动和盆底肌训练有助于促进子宫复旧、改善血液循环、预防血栓形成以及恢复盆底功能，但开始时间和运动强度需要根据产妇个体情况综合判断，避免过早或过度运动影响恢复。  首先，应根据分娩方式判断。阴道分娩且无明显并发症者，一般产后当天即可进行轻度活动，如翻身、下床走动及简单肢体活动。盆底肌轻度收缩训练通常可在产后24小时后逐渐开始。而剖宫产产妇由于存在腹部手术创伤，恢复时间相对较长。通常需待生命体征稳定、麻醉恢复后尽早下床活动，但腹部核心训练及强度较大的运动应适当延后。其次，应评估会阴及切口恢复情况。如果存在严重会阴裂伤、明显疼痛、会阴血肿或剖宫产切口愈合不良，则需延缓运动强度增加。第三，需要考虑产妇整体身体状况。包括是否存在贫血、产后出血、感染、头晕乏力或妊娠期高血压疾病等。如果身体恢复较差，应循序渐进。  盆底肌训练开始时间也与盆底损伤程度有关。对于一般阴道分娩产妇，可较早开始凯格尔运动；但若存在严重盆底损伤、器械助产或神经损伤，则需专业评估后再进行。运动强度应遵循“循序渐进”原则。早期以轻度活动和低强度盆底肌收缩为主，避免剧烈跑跳、负重或增加腹压的运动。  临床上，可根据以下表现判断运动耐受性：活动后无明显疲劳；恶露未明显增多；无头晕、腹痛或伤口疼痛加重；无漏尿或明显盆底坠胀。  如果运动后出现恶露突然增多、下腹疼痛、伤口不适或明显乏力，则提示运动量过大，应及时调整。此外，还需结合产妇既往运动习惯。长期有运动基础者恢复可能更快，但仍不建议过早进行高强度训练。通常产后6周复查后，可根据恢复情况逐步增加运动量，并在必要时进行专业盆底康复指导。  因此，产褥期运动恢复强调个体化、安全性和渐进性，核心目标是促进恢复而非追求快速“恢复身材”。 | 产褥期适当运动和盆底肌训练，对促进身体恢复和预防盆底功能障碍非常重要。但开始时间和运动强度需要综合评估，不能一概而论。  首先，应根据产妇分娩方式决定。自然分娩恢复相对较快，若无异常情况，一般可在产后早期开始轻度活动。剖宫产由于涉及腹部手术，运动恢复需更加谨慎。术后早期主要以翻身、坐起和缓慢步行为主，避免增加腹压。其次，应考虑是否存在产后并发症。如果产妇有产后出血、感染、重度贫血、高血压或严重疲劳，则需推迟运动计划。会阴及切口恢复情况也十分关键。若存在严重会阴裂伤、明显疼痛或伤口裂开风险，则不宜过早进行强度较大的训练。  盆底肌训练方面，轻度凯格尔运动通常可以较早开始，但应避免过度用力。  运动强度判断应重点观察产妇反应。合适运动应表现为：活动后精神状态良好；没有明显腹痛；恶露量无明显增加；切口无疼痛加重。如果运动后出现头晕、心慌、恶露增多或盆底下坠感，则提示运动量过大。此外，还应考虑产妇既往体能状态。有运动习惯者可适当恢复较快，但仍需循序渐进。  产后早期运动重点应放在：促进循环；恢复肌力；改善盆底功能；预防血栓和便秘。通常建议避免过早跑步、跳跃及高强度腹肌训练，以免影响盆底恢复。产后42天复查后，可根据恢复情况进一步增加运动量，必要时接受专业盆底康复指导。  因此，产褥期运动恢复应强调“个体化评估、循序渐进、安全优先”。 | 产褥期恢复运动和盆底肌训练，对促进身体康复和预防远期盆底问题具有重要意义。但运动开始时间和强度必须根据产妇具体恢复情况决定。  首先，需要看分娩方式。阴道分娩恢复较快，一般产后即可逐渐增加轻度活动；剖宫产则因手术创伤，需要更谨慎安排运动。其次，应评估伤口恢复情况。如果会阴伤口疼痛明显、存在血肿或剖宫产切口未愈合，则应暂缓增加运动强度。第三，要考虑产妇全身情况。若存在明显贫血、感染、持续头晕或产后出血，则说明身体尚未恢复，不适合较强运动。盆底肌训练开始时间也需个体化。  轻度凯格尔运动通常可较早进行，但如果有严重会阴裂伤、器械助产史或明显盆底损伤，则建议先进行专业评估。运动强度判断时，应重点观察以下情况：运动后恶露是否增多；是否出现腹痛或切口疼痛；是否感到明显疲劳；是否出现漏尿或盆底下坠感。  如果出现上述不适，说明运动量过大。  产后早期运动主要以低强度为主，例如散步、轻柔拉伸和呼吸训练。盆底肌训练应从短时间、低强度开始，逐渐增加次数和持续时间。同时，应避免过早进行跑步、跳绳、卷腹等增加腹压的运动，以免影响盆底恢复。通常在产后42天复查并确认恢复良好后，才适合逐渐恢复中高强度运动。  因此，产褥期运动恢复的关键是“因人而异、循序渐进”，既促进恢复，又避免二次损伤。 |
| 中文回答的英文翻译 English translation of Chinese response | Postpartum recovery exercises and pelvic floor muscle training help promote uterine involution, improve blood circulation, prevent blood clots, and restore pelvic floor function. However, the timing and intensity of these activities should be determined based on the individual circumstances of the new mother to avoid premature or excessive exercise that could hinder recovery.  First, the decision should be based on the mode of delivery. For women who had a vaginal delivery without significant complications, light activities such as turning over, getting out of bed to walk around, and simple limb movements can generally begin on the day of delivery. Mild pelvic floor muscle contraction exercises can usually be gradually introduced 24 hours after delivery. However, women who have undergone cesarean sections require a relatively longer recovery period due to the abdominal surgical trauma. They should get out of bed and move around as soon as possible once vital signs are stable and anesthesia has worn off; however, core abdominal exercises and high-intensity activities should be postponed appropriately. Second, the recovery of the perineum and incision should be assessed. If there are severe perineal tears, significant pain, perineal hematoma, or poor healing of the cesarean section incision, increasing the intensity of exercise should be delayed. Third, the mother’s overall physical condition must be considered. This includes the presence of anemia, postpartum hemorrhage, infection, dizziness, fatigue, or gestational hypertension. If physical recovery is poor, a gradual approach should be taken.  The timing of pelvic floor muscle training also depends on the extent of pelvic floor injury. For women who had a normal vaginal delivery, Kegel exercises can be started relatively early; however, if there is severe pelvic floor injury, instrumental delivery, or nerve damage, professional evaluation is required before beginning. Exercise intensity should follow the principle of “gradual progression.” In the early stages, focus on light activities and low-intensity pelvic floor muscle contractions, avoiding vigorous running, jumping, weight-bearing exercises, or activities that increase abdominal pressure.  Clinically, exercise tolerance can be assessed based on the following indicators: no significant fatigue after activity; no marked increase in lochia; no dizziness, abdominal pain, or worsening wound pain; and no urinary incontinence or noticeable pelvic heaviness.  If sudden increases in lochia, lower abdominal pain, wound discomfort, or significant fatigue occur after exercise, this indicates excessive exertion, and adjustments should be made promptly. Additionally, the mother’s previous exercise habits should be taken into account. Women with a long-term history of exercise may recover more quickly, but high-intensity training is still not recommended too early. Typically, after a follow-up examination 6 weeks postpartum, exercise intensity can be gradually increased based on recovery progress, and professional pelvic floor rehabilitation guidance should be sought when necessary.  Therefore, exercise during the postpartum period emphasizes individualization, safety, and progression, with the core goal being to promote recovery rather than pursuing rapid “body restoration.” | Appropriate exercise and pelvic floor muscle training during the postpartum period are crucial for promoting physical recovery and preventing pelvic floor dysfunction. However, the timing and intensity of exercise require a comprehensive assessment and cannot be generalized.  First, the decision should be based on the mother’s mode of delivery. Recovery from vaginal delivery is relatively faster; barring any complications, mild physical activity can generally begin in the early postpartum period. Because cesarean section involves abdominal surgery, a more cautious approach to resuming exercise is necessary. In the early postoperative period, activities should primarily consist of turning over, sitting up, and slow walking, while avoiding actions that increase abdominal pressure. Second, the presence of postpartum complications must be considered. If the mother experiences postpartum hemorrhage, infection, severe anemia, hypertension, or severe fatigue, the exercise plan should be postponed. The healing status of the perineum and incision is also critical. If there are severe perineal tears, significant pain, or a risk of wound dehiscence, high-intensity training should not be initiated too early.  Regarding pelvic floor muscle training, mild Kegel exercises can usually be started relatively early, but excessive straining should be avoided.  When assessing exercise intensity, close attention should be paid to the mother’s response. Appropriate exercise is characterized by: a good mental state after activity; no significant abdominal pain; no marked increase in lochia; and no worsening of incision pain. If dizziness, palpitations, increased lochia, or a sensation of pelvic floor heaviness occurs after exercise, this indicates that the exercise intensity is too high. Additionally, the mother’s prior physical fitness level should be considered. Those accustomed to exercise may recover somewhat more quickly, but a gradual approach is still necessary.  Early postpartum exercise should focus on: promoting circulation; restoring muscle strength; improving pelvic floor function; and preventing blood clots and constipation. It is generally recommended to avoid running, jumping, and high-intensity abdominal exercises too early, so as not to interfere with pelvic floor recovery. After the 42-day postpartum follow-up, exercise intensity may be increased based on recovery progress, and professional pelvic floor rehabilitation guidance should be sought if necessary.  Therefore, exercise during the postpartum recovery period should emphasize “individualized assessment, gradual progression, and safety first.” | Resuming exercise and pelvic floor muscle training during the postpartum period is crucial for promoting physical recovery and preventing long-term pelvic floor issues. However, the timing and intensity of exercise must be determined based on the mother’s specific recovery progress.  First, the mode of delivery must be considered. Recovery from vaginal delivery is generally faster, and mild physical activity can typically be gradually increased immediately after childbirth; in contrast, due to surgical trauma, exercise must be planned with greater caution following a cesarean section. Second, the healing status of the wound must be assessed. If there is significant pain at the perineal wound site, a hematoma is present, or the cesarean incision has not healed, increasing exercise intensity should be postponed. Third, the mother’s overall condition must be considered. If there is significant anemia, infection, persistent dizziness, or postpartum hemorrhage, this indicates that the body has not yet recovered and is not ready for strenuous exercise. The timing for starting pelvic floor muscle training also requires individualization.  Mild Kegel exercises can usually be started early, but if there is a severe perineal tear, a history of instrumental delivery, or significant pelvic floor injury, a professional evaluation is recommended first. When determining exercise intensity, pay close attention to the following: whether lochia increases after exercise; whether abdominal or incision pain occurs; whether significant fatigue is felt; and whether urinary incontinence or a sensation of pelvic floor heaviness occurs.  If any of the above discomforts occur, it indicates that the exercise intensity is too high.  Early postpartum exercise should primarily consist of low-intensity activities, such as walking, gentle stretching, and breathing exercises. Pelvic floor muscle training should begin with short sessions and low intensity, gradually increasing the frequency and duration. At the same time, activities that increase abdominal pressure—such as running, jumping rope, and sit-ups—should be avoided too early to prevent hindering pelvic floor recovery. Generally, it is appropriate to gradually resume moderate-to-high-intensity exercise only after a follow-up examination at 42 days postpartum confirms good recovery.  Therefore, the key to postpartum exercise recovery is to “tailor the approach to the individual and proceed gradually,” promoting recovery while avoiding secondary injury. |
| Correctness（1-5） | 4 | 4 | 4 |
| Completeness（1-5） | 5 | 4 | 4 |
| Guideline consistency（1-5） | 4 | 4 | 4 |
| Safety（1-5） | 4 | 4 | 4 |
| Clinical management appropriateness（1-5） | 4 | 4 | 4 |
| Chinese Reading Difficulty Score | 14.11 | 13.50 | 13.66 |
| Corresponding Education Level (Chinese) | 初中学段 | 初中学段 | 初中学段 |
| Recommended Reading Age (Chinese) | 14岁左右 | 13岁左右 | 14岁左右 |

## 问题 23

| 项目 | 第1次 | 第2次 | 第3次 |
| --- | --- | --- | --- |
| 产褥期临床知识问题 | 产褥期为什么是静脉血栓栓塞的高风险时期？哪些症状提示需要警惕深静脉血栓或肺栓塞？ | | |
| 主要指南来源 | NICE NG194；RCOG Green-top Guideline No. 37a | | |
| 模型回答 | 产褥期是静脉血栓栓塞（VTE）的高风险阶段，其中包括深静脉血栓形成（DVT）和肺栓塞（PE）。这是因为妊娠和分娩过程中，女性机体会发生一系列促进凝血的生理变化，以减少分娩出血，但同时也增加了血栓形成风险。  首先，妊娠和产后机体处于“高凝状态”。凝血因子增加，而抗凝和纤溶功能相对下降，这是最重要原因之一。其次，增大的子宫在孕期长期压迫盆腔和下肢静脉，导致静脉回流减慢。虽然分娩后压迫解除，但静脉功能恢复需要时间，因此产后仍易发生血液淤滞。第三，分娩过程中血管内皮可能受到损伤。尤其是剖宫产、难产、产后出血及感染等情况，会进一步增加血栓形成风险。此外，产后卧床、活动减少、肥胖、高龄、多胎妊娠及既往血栓病史，也都是重要危险因素。  深静脉血栓最常发生于下肢静脉。需要警惕的症状包括：单侧下肢肿胀；小腿疼痛或压痛；局部皮肤发热；皮肤发红或发紫；活动时疼痛加重。部分患者还会感觉患侧下肢沉重或紧绷。  如果血栓脱落进入肺循环，则可能发生肺栓塞，这是产科危重症之一。  肺栓塞的典型症状包括：突然呼吸困难；胸痛；胸闷；心率明显增快；咳嗽或咯血；严重时出现发绀、晕厥甚至休克。有些患者症状并不典型，仅表现为突然气促或无法解释的低氧血症，因此需要高度警惕。  一旦怀疑深静脉血栓或肺栓塞，应立即就医。  初步处理包括：避免按摩患肢；减少剧烈活动；保持平卧；及时完善下肢静脉超声或CT肺动脉造影等检查。对于高危产妇，临床上还需加强预防措施，例如早期下床活动、穿弹力袜以及必要时使用抗凝药物。  因此，产褥期静脉血栓栓塞虽然相对少见，但病情进展快、危险性高，早期识别症状至关重要。 | 产褥期女性发生静脉血栓栓塞的风险明显高于普通人群，尤其是在产后最初几周。这主要与妊娠和分娩带来的生理变化有关。  首先，妊娠后机体为了减少分娩时出血，会自然进入高凝状态。凝血因子水平升高，使血液更容易形成血栓。其次，孕期子宫增大压迫下腔静脉和盆腔静脉，导致血流缓慢。产后虽然压迫解除，但静脉回流恢复并非立即完成。第三，分娩本身会造成血管内皮损伤。尤其是剖宫产、产钳助产及产后感染等情况，更容易诱发血栓。此外，长期卧床、肥胖、高龄、多胎妊娠及既往血栓史，也会进一步增加风险。  深静脉血栓主要发生于下肢，常见症状包括：一侧小腿或大腿肿胀；疼痛或压痛；局部皮肤温度升高；皮肤颜色改变；行走时疼痛加重。如果左右腿粗细明显不对称，更要高度警惕。  肺栓塞则更加危险。典型症状包括：突发呼吸困难；胸痛；呼吸急促；心慌；咳嗽甚至咯血。严重患者可迅速出现低血压、意识障碍甚至猝死。  由于部分肺栓塞症状并不典型，因此对于产后突然出现胸闷气促的女性，必须高度重视。一旦怀疑血栓形成，应立即转诊或急诊处理。  检查方面，常需要进行下肢静脉超声、血气分析及CT肺动脉造影等。  对于高危产妇，临床中强调早期预防，包括尽早活动、避免久卧、补充水分及必要时抗凝治疗。  因此，产褥期是血栓栓塞高风险阶段，及时识别危险信号能够明显降低孕产妇死亡风险。 | 产褥期属于静脉血栓栓塞的高风险时期，尤其在产后6周内风险明显增加。其发生机制主要与“高凝状态、血流淤滞和血管损伤”三方面有关。  首先，妊娠和产后女性体内凝血功能增强，这是机体防止分娩出血的自然保护机制，但也会增加血栓形成风险。其次，孕期增大的子宫长期压迫下肢和盆腔静脉，使静脉回流减慢，形成血液淤滞。第三，分娩过程中血管内皮受到损伤，尤其是剖宫产和难产患者，更容易形成血栓。  此外，长期卧床、肥胖、吸烟、高龄、多胎妊娠及既往血栓病史，也会增加风险。  深静脉血栓常见于下肢，需警惕以下症状：单侧腿肿胀；小腿疼痛；局部发热；皮肤发红或颜色改变；活动时疼痛加重。  如果突然出现单侧下肢明显增粗，应高度怀疑。  肺栓塞则是最严重并发症。典型表现包括：突发胸闷气促；呼吸困难；胸痛；心跳加快；咯血；严重时可晕厥或休克。部分患者仅表现为不明原因呼吸急促，因此临床不能忽视。一旦怀疑血栓或肺栓塞，应避免随意活动患肢，并尽快进行专科评估。  对于高危人群，应强调预防措施，包括早期活动、避免脱水及必要时应用抗凝药物。  因此，产褥期出现单侧下肢肿痛或突发呼吸困难时，应高度警惕静脉血栓栓塞，及时就医。 |
| 中文回答的英文翻译 English translation of Chinese response | The postpartum period is a high-risk phase for venous thromboembolism (VTE), which includes deep vein thrombosis (DVT) and pulmonary embolism (PE). This is because during pregnancy and childbirth, a woman’s body undergoes a series of physiological changes that promote blood clotting to reduce postpartum bleeding, but these changes also increase the risk of thrombosis.  First, the body is in a “hypercoagulable state” during pregnancy and the postpartum period. An increase in clotting factors, coupled with a relative decline in anticoagulant and fibrinolytic functions, is one of the most significant contributing factors. Second, the enlarged uterus exerts prolonged pressure on the pelvic and lower limb veins during pregnancy, leading to slowed venous return. Although this pressure is relieved after delivery, it takes time for venous function to recover, making blood stasis more likely to occur postpartum. Third, the vascular endothelium may be damaged during childbirth. In particular, conditions such as cesarean section, difficult labor, postpartum hemorrhage, and infection further increase the risk of thrombosis. Additionally, postpartum bed rest, reduced physical activity, obesity, advanced maternal age, multiple pregnancies, and a history of thrombosis are all significant risk factors.  Deep vein thrombosis most commonly occurs in the veins of the lower extremities. Symptoms to watch for include: swelling in one lower limb; pain or tenderness in the calf; warmth in the local skin; redness or purple discoloration of the skin; and pain that worsens with movement. Some patients may also feel heaviness or tightness in the affected lower limb.  If a blood clot breaks loose and enters the pulmonary circulation, it can cause a pulmonary embolism, which is one of the most critical conditions in obstetrics.  Typical symptoms of a pulmonary embolism include: sudden shortness of breath; chest pain; chest tightness; a marked increase in heart rate; coughing or hemoptysis; and, in severe cases, cyanosis, syncope, or even shock. Some patients may present with atypical symptoms, such as sudden shortness of breath or unexplained hypoxemia, so high vigilance is required.  If deep vein thrombosis or pulmonary embolism is suspected, seek medical attention immediately.  Initial management includes: avoiding massage of the affected limb; reducing strenuous activity; remaining in a supine position; and promptly undergoing diagnostic tests such as lower extremity venous ultrasound or CT pulmonary angiography. For high-risk postpartum women, additional preventive measures are clinically necessary, such as early ambulation, wearing compression stockings, and the use of anticoagulants when indicated.  Therefore, although venous thromboembolism during the postpartum period is relatively rare, it progresses rapidly and carries a high risk; early recognition of symptoms is crucial. | Women in the postpartum period are at a significantly higher risk of venous thromboembolism than the general population, particularly during the first few weeks after childbirth. This is primarily due to the physiological changes associated with pregnancy and childbirth.  First, after conception, the body naturally enters a hypercoagulable state to reduce bleeding during childbirth. Elevated levels of clotting factors make it easier for blood clots to form. Second, the enlargement of the uterus during pregnancy compresses the inferior vena cava and pelvic veins, leading to slowed blood flow. Although this compression is relieved after childbirth, venous return does not return to normal immediately. Third, the delivery process itself causes damage to the vascular endothelium. In particular, conditions such as cesarean section, forceps delivery, and postpartum infections are more likely to trigger thrombosis. Additionally, prolonged bed rest, obesity, advanced maternal age, multiple pregnancies, and a history of thrombosis further increase the risk.  Deep vein thrombosis primarily occurs in the lower limbs. Common symptoms include: swelling in one calf or thigh; pain or tenderness; increased local skin temperature; changes in skin color; and worsening pain when walking. If there is a noticeable asymmetry in the thickness of the left and right legs, heightened vigilance is warranted.  Pulmonary embolism is even more dangerous. Typical symptoms include: sudden shortness of breath; chest pain; rapid breathing; palpitations; coughing, and even coughing up blood. In severe cases, patients may rapidly develop hypotension, impaired consciousness, or even sudden death.  Since some symptoms of pulmonary embolism are not typical, women who suddenly experience chest tightness or shortness of breath after childbirth must be taken very seriously. If thrombosis is suspected, immediate referral or emergency care is required.  Diagnostic tests often include lower extremity venous ultrasound, arterial blood gas analysis, and CT pulmonary angiography.  For high-risk postpartum women, clinical practice emphasizes early prevention, including early mobilization, avoiding prolonged bed rest, staying hydrated, and anticoagulant therapy when necessary.  Therefore, the postpartum period is a high-risk phase for thromboembolism, and timely recognition of warning signs can significantly reduce the risk of maternal mortality. | The postpartum period is a high-risk time for venous thromboembolism, with the risk increasing significantly within the first 6 weeks after childbirth. The underlying mechanisms are primarily related to three factors: a hypercoagulable state, stasis of blood flow, and vascular injury.  First, coagulation function is enhanced in women during pregnancy and the postpartum period. While this serves as a natural protective mechanism to prevent hemorrhage during childbirth, it also increases the risk of thrombosis. Second, the enlarged uterus during pregnancy exerts prolonged pressure on the veins of the lower limbs and pelvis, slowing venous return and leading to stasis. Third, vascular endothelial damage during childbirth—particularly in cases of cesarean section or difficult labor—makes patients more susceptible to thrombosis.  Additionally, prolonged bed rest, obesity, smoking, advanced age, multiple pregnancies, and a history of thrombosis also increase the risk.  Deep vein thrombosis commonly occurs in the lower limbs. Be alert for the following symptoms: swelling in one leg; pain in the calf; localized warmth; redness or discoloration of the skin; and pain that worsens with movement.  If there is a sudden, noticeable thickening of one lower limb, it should be highly suspected.  Pulmonary embolism is the most serious complication. Typical symptoms include: sudden chest tightness and shortness of breath; difficulty breathing; chest pain; rapid heartbeat; hemoptysis; and, in severe cases, syncope or shock. Some patients present only with unexplained shortness of breath, so this clinical presentation should not be overlooked. Once thrombosis or pulmonary embolism is suspected, avoid moving the affected limb unnecessarily and seek specialist evaluation as soon as possible.  For high-risk individuals, preventive measures should be emphasized, including early mobilization, avoiding dehydration, and the use of anticoagulants when necessary.  Therefore, when unilateral lower limb swelling and pain or sudden dyspnea occur during the postpartum period, one should be highly vigilant for venous thromboembolism and seek medical attention promptly. |
| Correctness（1-5） | 4 | 4 | 4 |
| Completeness（1-5） | 4 | 4 | 3 |
| Guideline consistency（1-5） | 3 | 4 | 4 |
| Safety（1-5） | 5 | 4 | 4 |
| Clinical management appropriateness（1-5） | 4 | 4 | 3 |
| Chinese Reading Difficulty Score | 13.41 | 13.37 | 13.36 |
| Corresponding Education Level (Chinese) | 初中学段 | 初中学段 | 初中学段 |
| Recommended Reading Age (Chinese) | 13岁左右 | 13岁左右 | 13岁左右 |

## 问题 24

| 项目 | 第1次 | 第2次 | 第3次 |
| --- | --- | --- | --- |
| 产褥期临床知识问题 | 有妊娠期高血压或子痫前期病史的产妇，产后应重点监测哪些内容？哪些症状提示需要紧急处理？ | | |
| 主要指南来源 | NICE NG194；ACOG产后照护优化建议 | | |
| 模型回答 | 有妊娠期高血压或子痫前期病史的产妇，在产后仍属于高风险人群。虽然分娩后胎盘娩出，疾病通常会逐渐缓解，但部分患者在产后数天甚至数周内仍可能发生高血压危象、子痫、心力衰竭或脑血管并发症，因此必须加强监测。  首先，应重点监测血压变化。产后血压并不会立即恢复正常，部分患者甚至会在产后3～6天出现血压再次升高。因此需要定期测量血压，尤其关注收缩压≥140mmHg或舒张压≥90mmHg的情况。其次，应监测神经系统症状。包括头痛、头晕、视物模糊、眼前闪光感等。这些症状可能提示脑血管痉挛或子痫前期加重。第三，需要观察尿量及水肿情况。少尿、水肿加重或体重短时间明显增加，可能提示肾功能受损或液体潴留。此外，还需关注心肺功能。患者若出现胸闷、呼吸困难、端坐呼吸或咳粉红色泡沫痰，应警惕急性左心衰或肺水肿。  实验室监测同样重要，包括：尿蛋白；肝肾功能；血小板计数；凝血功能等。  因为部分患者产后仍可能存在肝功能异常、HELLP综合征或凝血异常。  对于曾有重度子痫前期者，还需继续观察是否存在抽搐风险。必要时产后仍需短期使用硫酸镁预防子痫。  以下症状提示需要紧急处理：持续或剧烈头痛；视力模糊或突然视力下降；抽搐；意识改变；胸闷气促；严重高血压；少尿或无尿；持续上腹痛或右上腹疼痛。  尤其是抽搐和呼吸困难，属于产科急危重症信号，应立即急诊处理。  护理方面，应保证充分休息，避免情绪波动，同时指导低盐饮食及规律复查。  因此，妊娠期高血压和子痫前期患者在产后并非“分娩后就安全”，仍需要持续监测和规范随访。 | 妊娠期高血压和子痫前期虽然多在分娩后逐渐改善，但产后仍可能发生严重并发症，因此必须继续重点监测。  最重要的监测内容是血压。部分产妇在产后数日血压仍持续升高，甚至出现高血压危象。因此需规律监测，并记录血压变化趋势。其次，应重点关注神经系统症状。持续头痛、头晕、烦躁、视物模糊或眼前黑影，都提示脑血管受累风险增加。  尿量和水肿情况也很关键。若尿量明显减少、下肢或全身水肿加重，可能提示肾功能异常。  此外，还需观察是否存在肺水肿表现。例如胸闷、呼吸困难、咳嗽及夜间不能平卧等。  实验室方面，需定期复查：尿蛋白；肝功能；肾功能；血小板及凝血指标。因为部分患者在产后仍可能出现HELLP综合征或器官功能损害。  对于重度子痫前期患者，产后24小时内仍属于子痫高发期，因此需严密观察抽搐征象。  需要紧急处理的危险症状包括：血压明显升高；剧烈头痛；抽搐；视力障碍；意识模糊；严重胸闷气促；持续少尿。如果出现这些情况，应立即就医甚至进入重症监护。  临床处理上，可能需要继续降压治疗、硫酸镁预防抽搐以及液体管理。  因此，对有妊娠期高血压病史的产妇，产后监测重点不仅是“血压”，更包括神经、肾脏和心肺等多系统功能。 | 有妊娠期高血压或子痫前期病史的女性，在产后仍属于高危产妇。因为即使胎盘娩出后，疾病也不会立刻完全恢复，部分严重并发症甚至可能首次出现在产后。  首先，应重点监测血压。产后血压可能波动，特别是在产后3～7天容易再次升高。其次，应观察是否存在神经系统异常。例如持续头痛、眼花、视力下降、烦躁不安等，这些都可能是子痫前期恶化信号。第三，需要关注尿量变化。少尿或无尿提示肾脏灌注不足或肾功能受损。同时，还需观察水肿及体重变化。如果短时间体重明显增加，可能提示液体潴留。  心肺症状也必须重视。若出现胸闷、呼吸困难、咳嗽或无法平卧，应警惕肺水肿。  实验室监测包括：尿蛋白；肝肾功能；血小板；凝血功能。因为部分患者产后仍可发生HELLP综合征。  需要紧急处理的症状包括：血压持续重度升高；剧烈头痛；抽搐；视物模糊；胸闷气促；意识异常；严重少尿；持续右上腹痛。其中抽搐和呼吸困难最危险，需要立即急救。  对于重度患者，产后仍可能需要继续使用降压药及硫酸镁。  因此，妊娠期高血压相关疾病的管理并不会随着分娩结束，而需要延续到整个产褥期。 |
| 中文回答的英文翻译 English translation of Chinese response | Women with a history of gestational hypertension or preeclampsia remain at high risk after childbirth. Although the condition typically improves gradually following delivery and the expulsion of the placenta, some patients may still experience hypertensive crises, eclampsia, heart failure, or cerebrovascular complications within days or even weeks after delivery; therefore, close monitoring is essential.  First, close monitoring of blood pressure changes is essential. Blood pressure does not immediately return to normal after delivery; in some patients, it may even rise again 3 to 6 days postpartum. Therefore, blood pressure should be measured regularly, with particular attention to systolic pressure ≥140 mmHg or diastolic pressure ≥90 mmHg. Second, neurological symptoms should be monitored. These include headache, dizziness, blurred vision, and flashes of light before the eyes. These symptoms may indicate cerebral vasospasm or worsening preeclampsia. Third, urine output and edema should be monitored. Oliguria, worsening edema, or a significant increase in body weight over a short period may indicate impaired renal function or fluid retention. Additionally, cardiac and pulmonary function should be closely monitored. If a patient experiences chest tightness, dyspnea, orthopnea, or coughs up pink frothy sputum, acute left heart failure or pulmonary edema should be suspected.  Laboratory monitoring is equally important, including: urine protein; liver and kidney function; platelet count; and coagulation function.  This is because some patients may still have abnormal liver function, HELLP syndrome, or coagulation abnormalities postpartum.  For patients with a history of severe preeclampsia, continued monitoring for the risk of seizures is necessary. If necessary, short-term postpartum use of magnesium sulfate is required to prevent eclampsia.  The following symptoms indicate the need for urgent intervention: persistent or severe headache; blurred vision or sudden vision loss; seizures; altered consciousness; chest tightness or shortness of breath; severe hypertension; oliguria or anuria; persistent upper abdominal pain or right upper quadrant pain.  In particular, seizures and dyspnea are signs of obstetric emergencies and require immediate emergency care.  In terms of nursing care, adequate rest should be ensured, emotional fluctuations should be avoided, and patients should be advised to follow a low-sodium diet and attend regular follow-up appointments.  Therefore, patients with gestational hypertension and preeclampsia are not “safe once delivery is complete”; they still require continuous monitoring and standardized follow-up care. | Although gestational hypertension and preeclampsia often improve gradually after delivery, serious complications may still occur postpartum; therefore, close monitoring must continue.  The most critical aspect of monitoring is blood pressure. In some women, blood pressure remains elevated for several days after delivery, and some may even experience a hypertensive crisis. Therefore, regular monitoring is necessary to track trends in blood pressure. Secondly, close attention should be paid to neurological symptoms. Persistent headaches, dizziness, irritability, blurred vision, or dark spots in the visual field all indicate an increased risk of cerebrovascular involvement.  Urine output and edema are also critical. A significant decrease in urine output or worsening edema in the lower extremities or throughout the body may indicate renal dysfunction.  In addition, it is necessary to observe for signs of pulmonary edema, such as chest tightness, shortness of breath, coughing, and an inability to lie flat at night.  Laboratory tests should be monitored regularly, including urine protein, liver function, kidney function, platelet count, and coagulation parameters. This is because some patients may still develop HELLP syndrome or organ dysfunction postpartum.  For patients with severe preeclampsia, the first 24 hours postpartum remain a high-risk period for eclampsia; therefore, close monitoring for signs of seizures is required.  Symptoms requiring urgent intervention include: a marked rise in blood pressure; severe headache; seizures; visual disturbances; confusion; severe chest tightness and shortness of breath; and persistent oliguria. If any of these conditions arise, immediate medical attention is required, and the patient may need to be admitted to the intensive care unit.  Clinically, continued antihypertensive therapy, magnesium sulfate for seizure prevention, and fluid management may be required.  Therefore, for women with a history of gestational hypertension, postpartum monitoring should focus not only on “blood pressure” but also on the function of multiple systems, including the nervous, renal, and cardiopulmonary systems. | Women with a history of gestational hypertension or preeclampsia remain at high risk even after childbirth. This is because the condition does not resolve completely immediately after the placenta is delivered, and some severe complications may even first appear postpartum.  First, close monitoring of blood pressure is essential. Blood pressure may fluctuate postpartum, and it is particularly prone to rising again between 3 and 7 days after delivery. Second, observe for any neurological abnormalities. For example, persistent headaches, blurred vision, decreased visual acuity, or restlessness may all be signs of worsening preeclampsia. Third, monitor changes in urine output. Oliguria or anuria may indicate inadequate renal perfusion or impaired renal function. Additionally, observe for edema and changes in body weight. A significant weight gain over a short period may indicate fluid retention.  Cardiopulmonary symptoms must also be taken seriously. If chest tightness, shortness of breath, coughing, or an inability to lie flat occur, be alert for pulmonary edema.  Laboratory monitoring includes: urine protein; liver and kidney function; platelet count; and coagulation function. This is because some patients may still develop HELLP syndrome postpartum.  Symptoms requiring urgent intervention include: persistently severe hypertension; severe headache; seizures; blurred vision; chest tightness and shortness of breath; altered consciousness; severe oliguria; persistent right upper quadrant pain. Among these, seizures and dyspnea are the most dangerous and require immediate emergency care.  For severe cases, continued use of antihypertensive medications and magnesium sulfate may still be necessary postpartum.  Therefore, the management of pregnancy-related hypertension does not end with delivery but must continue throughout the entire postpartum period. |
| Correctness（1-5） | 4 | 4 | 4 |
| Completeness（1-5） | 4 | 3 | 3 |
| Guideline consistency（1-5） | 4 | 4 | 4 |
| Safety（1-5） | 5 | 4 | 4 |
| Clinical management appropriateness（1-5） | 4 | 3 | 3 |
| Chinese Reading Difficulty Score | 13.73 | 13.48 | 13.26 |
| Corresponding Education Level (Chinese) | 初中学段 | 初中学段 | 初中学段 |
| Recommended Reading Age (Chinese) | 14岁左右 | 13岁左右 | 13岁左右 |

## 问题 25

| 项目 | 第1次 | 第2次 | 第3次 |
| --- | --- | --- | --- |
| 产褥期临床知识问题 | 有妊娠期糖尿病病史的产妇，产后血糖随访和远期代谢风险管理应包括哪些内容？ | | |
| 主要指南来源 | NICE NG194；ACOG产后照护优化建议 | | |
| 模型回答 | 有妊娠期糖尿病（GDM）病史的产妇，即使在分娩后血糖多数能够恢复正常，仍属于未来发生2型糖尿病和代谢综合征的高风险人群。因此，产后血糖随访和长期代谢管理非常重要。  首先，产后应进行血糖复查。一般建议在产后6～12周进行75g口服葡萄糖耐量试验（OGTT），以评估糖代谢是否恢复正常。  检查结果可能表现为：血糖恢复正常；糖耐量受损；空腹血糖受损；或已发展为糖尿病。其中OGTT比单纯空腹血糖更敏感，因此是推荐的标准筛查方法。  其次，需要进行长期随访。即使产后血糖恢复正常，未来发生2型糖尿病的风险仍明显高于普通女性。  通常建议：至少每1～3年进行一次血糖筛查；如果存在肥胖、家族史或再次妊娠计划，则需更密切监测。第三，应加强体重管理。妊娠期糖尿病患者产后如果持续超重或腹型肥胖，会进一步增加糖尿病风险。因此应指导合理饮食和规律运动，避免高糖、高脂饮食。母乳喂养也有积极作用。研究表明，母乳喂养有助于改善母体糖代谢，并降低未来糖尿病发生风险，因此应鼓励坚持母乳喂养。  此外，还应关注其他代谢风险因素。例如：高血压；血脂异常；脂肪肝；代谢综合征等。  必要时需联合内分泌科进一步评估。  对于再次妊娠计划者，应在孕前进行血糖评估和体重管理，因为再次妊娠时复发GDM的风险较高。  健康教育中，还需强调：保持规律生活；增加有氧运动；控制体重；减少含糖饮料和精制碳水摄入。如果出现明显口渴、多尿、体重下降等高血糖症状，应及时就医。  因此，妊娠期糖尿病的管理不仅限于妊娠期间，更需要贯穿产后及长期代谢健康管理。 | 妊娠期糖尿病虽然多数在分娩后血糖会下降，但这类女性未来发生2型糖尿病和心血管代谢疾病的风险明显增加，因此产后管理十分重要。  首先，产后血糖随访是核心内容。通常建议在产后6～12周进行75g OGTT检查，以明确糖代谢状态是否恢复正常。因为部分患者虽然空腹血糖正常，但糖耐量仍异常，因此不能仅依靠空腹血糖判断。如果检查结果异常，则需进一步由内分泌专科随访管理。其次，即使血糖恢复正常，也需要长期监测。建议至少每1～3年复查一次血糖。对于存在肥胖、糖尿病家族史或再次妊娠计划的女性，监测频率应更高。  体重管理同样非常关键。产后应鼓励逐步恢复健康体重，避免长期超重。  饮食方面，应继续保持均衡饮食结构，减少高糖、高热量食物摄入。  规律运动也有助于改善胰岛素敏感性。一般推荐中等强度有氧运动，例如快走、游泳等。  此外，母乳喂养不仅有利于婴儿，也有助于母体糖代谢恢复，因此应积极鼓励。  远期管理中，还需关注其他代谢问题。例如：高血压；高脂血症；肥胖；代谢综合征。因为这些问题常与糖代谢异常同时存在。  对于计划再次妊娠者，应提前进行血糖和体重评估，以减少再次发生妊娠期糖尿病的风险。  因此，妊娠期糖尿病的产后管理重点不仅是“查一次血糖”，更是长期代谢风险干预。 | 有妊娠期糖尿病病史的女性，在产后虽然大多数血糖可恢复正常，但未来发生2型糖尿病的风险明显升高，因此需要长期随访和代谢管理。  首先，产后应进行规范血糖复查。一般建议在产后6～12周完成75g口服葡萄糖耐量试验。  通过检查可以判断：血糖是否恢复正常；是否存在糖耐量减低；是否已经发展为糖尿病。之后还应进行长期血糖监测。即使首次复查正常，也建议定期随访。对于高危女性，如肥胖、再次妊娠或有糖尿病家族史者，应更频繁复查。其次，远期风险管理重点是生活方式干预。  包括：合理控制饮食；减少精制糖摄入；增加膳食纤维；坚持规律运动；控制体重。  体重管理尤其重要，因为肥胖会显著增加未来糖尿病风险。  母乳喂养同样值得鼓励。研究发现，长期母乳喂养有助于改善母体代谢状态。  此外，还应监测其他代谢异常，例如高血压、血脂异常和脂肪肝。  对于计划再次怀孕的女性，应在孕前完成血糖评估，并尽量将体重控制在合理范围。  健康教育中，应提醒产妇注意糖尿病相关症状，例如口渴、多尿和体重异常下降。  因此，妊娠期糖尿病的管理重点不仅是产后短期血糖恢复，更包括未来代谢疾病的长期预防。 |
| 中文回答的英文翻译 English translation of Chinese response | Women with a history of gestational diabetes (GDM) remain at high risk for developing type 2 diabetes and metabolic syndrome in the future, even though their blood glucose levels typically return to normal after delivery. Therefore, postpartum blood glucose follow-up and long-term metabolic management are crucial.  First, blood glucose levels should be rechecked postpartum. It is generally recommended to perform a 75-gram oral glucose tolerance test (OGTT) 6 to 12 weeks after delivery to assess whether glucose metabolism has returned to normal.  Test results may indicate: normal blood glucose levels; impaired glucose tolerance; impaired fasting glucose; or the development of diabetes. Among these, the OGTT is more sensitive than fasting blood glucose alone and is therefore the recommended standard screening method.  Second, long-term follow-up is necessary. Even if blood glucose levels return to normal postpartum, the risk of developing type 2 diabetes in the future remains significantly higher than in the general female population.  It is generally recommended to undergo blood glucose screening at least once every 1 to 3 years; closer monitoring is required if obesity, a family history of diabetes, or plans for another pregnancy are present. Third, weight management should be prioritized. If women with gestational diabetes remain overweight or have abdominal obesity after childbirth, their risk of developing diabetes increases further. Therefore, they should be advised to follow a balanced diet and engage in regular exercise, avoiding high-sugar and high-fat foods. Breastfeeding also has a positive effect. Studies indicate that breastfeeding helps improve maternal glucose metabolism and reduces the future risk of diabetes; therefore, continued breastfeeding should be encouraged.  Additionally, attention should be paid to other metabolic risk factors, such as hypertension, dyslipidemia, fatty liver disease, and metabolic syndrome.  If necessary, further evaluation by an endocrinologist should be sought.  For those planning a subsequent pregnancy, blood glucose assessment and weight management should be conducted before conception, as the risk of GDM recurrence is higher in subsequent pregnancies.  Health education should also emphasize maintaining a regular lifestyle, increasing aerobic exercise, controlling weight, and reducing intake of sugary beverages and refined carbohydrates. If symptoms of hyperglycemia, such as excessive thirst, frequent urination, or weight loss, occur, medical attention should be sought promptly.  Therefore, the management of gestational diabetes is not limited to the pregnancy period but must extend through the postpartum period and into long-term metabolic health management. | Although blood glucose levels in most women with gestational diabetes decrease after delivery, these women have a significantly increased risk of developing type 2 diabetes and cardiovascular and metabolic diseases in the future; therefore, postpartum management is crucial.  First, postpartum blood glucose follow-up is essential. It is generally recommended to undergo a 75g oral glucose tolerance test (OGTT) 6 to 12 weeks postpartum to determine whether glucose metabolism has returned to normal. This is because some patients may have normal fasting blood glucose levels but still exhibit impaired glucose tolerance; therefore, fasting blood glucose alone is not sufficient for diagnosis. If test results are abnormal, further follow-up and management by an endocrinologist are required. Second, even if blood glucose levels return to normal, long-term monitoring is necessary. It is recommended to have blood glucose levels rechecked at least once every 1 to 3 years. For women who are obese, have a family history of diabetes, or plan to become pregnant again, monitoring should be more frequent.  Weight management is equally critical. Postpartum women should be encouraged to gradually return to a healthy weight and avoid prolonged overweight conditions.  Regarding diet, a balanced dietary structure should be maintained, and intake of high-sugar and high-calorie foods should be reduced.  Regular exercise also helps improve insulin sensitivity. Moderate-intensity aerobic exercise, such as brisk walking or swimming, is generally recommended.  In addition, breastfeeding benefits not only the infant but also aids in the mother’s recovery of glucose metabolism; therefore, it should be actively encouraged.  In long-term management, attention should also be paid to other metabolic issues, such as hypertension, hyperlipidemia, obesity, and metabolic syndrome, as these conditions often coexist with glucose metabolism abnormalities.  For women planning another pregnancy, blood glucose and weight assessments should be conducted in advance to reduce the risk of recurrent gestational diabetes.  Therefore, the focus of postpartum management for gestational diabetes is not merely a “single blood glucose test,” but rather long-term intervention for metabolic risks. | Although blood glucose levels return to normal in most women with a history of gestational diabetes after childbirth, their risk of developing type 2 diabetes in the future is significantly elevated; therefore, long-term follow-up and metabolic management are necessary.  First, standardized blood glucose follow-up testing should be conducted postpartum. It is generally recommended to complete a 75-gram oral glucose tolerance test 6 to 12 weeks after delivery.  This test can determine whether blood glucose levels have returned to normal, whether impaired glucose tolerance is present, or whether diabetes has already developed. Long-term blood glucose monitoring should follow. Even if the initial follow-up is normal, regular follow-ups are recommended. For high-risk women—such as those who are obese, planning another pregnancy, or have a family history of diabetes—more frequent follow-ups are advised. Second, long-term risk management focuses on lifestyle interventions.  These include: maintaining a balanced diet; reducing refined sugar intake; increasing dietary fiber; engaging in regular physical activity; and managing weight.  Weight management is particularly important, as obesity significantly increases the risk of future diabetes.  Breastfeeding is also strongly encouraged. Studies have shown that long-term breastfeeding helps improve maternal metabolic health.  In addition, other metabolic abnormalities, such as hypertension, dyslipidemia, and fatty liver disease, should be monitored.  For women planning another pregnancy, blood glucose assessment should be completed before conception, and weight should be maintained within a healthy range as much as possible.  Health education should remind postpartum women to be aware of diabetes-related symptoms, such as excessive thirst, frequent urination, and unexplained weight loss.  Therefore, the focus of gestational diabetes management is not only on short-term postpartum blood glucose recovery but also on the long-term prevention of future metabolic diseases. |
| Correctness（1-5） | 4 | 4 | 4 |
| Completeness（1-5） | 4 | 4 | 3 |
| Guideline consistency（1-5） | 4 | 4 | 4 |
| Safety（1-5） | 4 | 4 | 4 |
| Clinical management appropriateness（1-5） | 4 | 4 | 3 |
| Chinese Reading Difficulty Score | 13.64 | 13.89 | 13.62 |
| Corresponding Education Level (Chinese) | 初中学段 | 初中学段 | 初中学段 |
| Recommended Reading Age (Chinese) | 14岁左右 | 14岁左右 | 14岁左右 |

## 问题 26

| 项目 | 第1次 | 第2次 | 第3次 |
| --- | --- | --- | --- |
| 产褥期临床知识问题 | 如何区分产后情绪低落、产后抑郁和严重精神症状？临床评估时应重点询问哪些内容？ | | |
| 主要指南来源 | NICE CG192；NICE NG194；中国孕产妇健康管理服务规范 | | |
| 模型回答 | 产后女性由于激素变化、睡眠不足、角色转换及育儿压力，容易出现情绪波动。但不同程度的心理问题在严重性和处理方式上差异很大，因此临床上需要区分产后情绪低落、产后抑郁以及严重精神症状。  产后情绪低落，也称“产后忧郁”，是最常见、相对轻微的情绪反应。通常发生于产后3～5天，表现为容易哭泣、情绪敏感、焦虑、疲惫及睡眠差。其特点是：症状较轻；持续时间短；一般不超过2周；产妇仍能照顾婴儿和完成日常生活；在家人支持下多可自行缓解。  而产后抑郁则属于真正的精神心理疾病。通常发生于产后数周至数月，持续时间较长。患者常表现为：持续情绪低落；兴趣减退；明显疲乏；睡眠和食欲改变；自我评价低；对婴儿缺乏兴趣或照顾意愿。部分患者还会出现明显焦虑、内疚感甚至无价值感。严重时可出现自伤或轻生想法。  严重精神症状则包括产后精神病等精神障碍，虽然较少见，但危险性极高。患者可能出现：幻觉；妄想；行为紊乱；明显意识异常；情绪极端波动；伤害自己或婴儿的想法。  部分患者会出现“有人要害孩子”等妄想内容，需要紧急精神科处理。  临床评估时，首先应重点询问情绪持续时间和严重程度。短暂波动更倾向情绪低落，而持续超过2周需警惕抑郁。其次，应了解睡眠情况。即使婴儿睡觉时仍无法入睡，往往提示心理问题较重。第三，要询问兴趣和动力变化。例如是否对以往喜欢的事情失去兴趣，是否不愿照顾婴儿。  还需重点评估是否存在：明显焦虑；绝望感；自责感；无价值感。  尤其重要的是询问是否存在自伤、自杀或伤害婴儿的想法，这是危险评估核心内容。同时，还应了解家庭支持情况、婚姻关系及既往精神病史。必要时可使用爱丁堡产后抑郁量表等工具进行筛查。  如果患者出现幻觉、妄想、行为异常或危险倾向，则需立即精神科会诊甚至住院治疗。  因此，产后心理问题评估不仅关注“心情不好”，更重要的是识别持续性抑郁和危险精神症状。 | 产后女性情绪变化较常见，但临床上需要区分正常情绪波动与精神疾病，以便及时干预。  产后情绪低落通常属于轻度短暂反应。患者可能会哭泣、敏感、容易烦躁，但总体仍能正常生活和照顾婴儿。症状多在产后1～2周内自行缓解。  产后抑郁则更严重，持续时间更长。患者会出现明显情绪低落、兴趣减退、疲劳感增加，并常伴睡眠障碍和食欲改变。她们可能觉得自己“不称职”，甚至对婴儿缺乏情感连接。而严重精神症状，如产后精神病，则属于精神科急症。患者可能出现幻听、妄想、意识混乱及行为异常。  这类患者可能伤害自己或婴儿，因此危险性极高。  在评估时，首先应询问情绪变化持续多久。短暂情绪波动较常见，而持续超过2周则需警惕抑郁。其次，应了解患者是否仍能完成日常照护。如果已经明显无法照顾自己或婴儿，提示问题较严重。睡眠情况也是重点。严重失眠尤其是“即使有机会也睡不着”，常提示抑郁或焦虑。  还应询问：是否经常感到绝望；是否持续自责；是否失去生活兴趣；是否出现明显焦虑和恐惧。  危险性评估尤其重要，需要直接询问：有没有伤害自己的想法；有没有伤害孩子的念头。  同时，还应了解既往精神疾病史、家庭支持及生活压力。若患者出现幻觉、妄想、言语混乱或行为异常，应立即转精神专科处理。  因此，产后心理评估的关键是区分“短暂情绪波动”和“持续功能受损的精神问题”。 | 产后女性常会经历不同程度的情绪变化，但并不是所有情绪低落都属于疾病。临床上通常需要区分产后情绪低落、产后抑郁以及严重精神障碍。  产后情绪低落最常见，多与激素变化和疲劳有关。患者可能表现为爱哭、情绪敏感和焦虑，但症状通常较轻，并在两周内逐渐缓解。  产后抑郁则属于持续性心理疾病。患者会长期感到悲伤、无助、兴趣下降，甚至不愿与家人交流。很多患者会出现明显疲惫感、自我否定和睡眠障碍。严重时可能出现轻生想法。  而严重精神症状，例如产后精神病，则更加危险。患者可能出现：幻觉；妄想；极端行为；意识混乱；严重情绪失控。部分患者甚至会出现伤害婴儿的危险行为。  临床评估时，首先应询问情绪持续时间和严重程度。其次，要评估功能状态。例如是否还能正常照顾孩子、饮食和睡眠是否明显受影响。  重点问题包括：是否长期情绪低落；是否对任何事情都失去兴趣；是否持续感到绝望或无价值。  还应重点询问是否存在：自伤念头；自杀计划；伤害婴儿的想法。此外，还需了解家庭支持情况及既往精神病史。  如果出现幻觉、妄想、行为异常或明显现实判断力下降，应立即精神科急诊处理。  因此，产后心理问题的核心在于早期识别危险信号，及时给予专业帮助。 |
| 中文回答的英文翻译 English translation of Chinese response | Due to hormonal changes, sleep deprivation, role transitions, and the pressures of parenting, women are prone to emotional fluctuations after childbirth. However, psychological issues vary greatly in severity and require different approaches; therefore, it is clinically necessary to distinguish between postpartum blues, postpartum depression, and severe psychiatric symptoms.  Postpartum blues, also known as “postpartum melancholy,” is the most common and relatively mild emotional reaction. It typically occurs 3 to 5 days after childbirth and manifests as tearfulness, emotional sensitivity, anxiety, fatigue, and poor sleep. Its characteristics include: mild symptoms; short duration (generally no more than 2 weeks); the ability to care for the infant and manage daily life; and resolution often occurring spontaneously with family support.  Postpartum depression, however, is a true psychiatric disorder. It typically occurs several weeks to several months after childbirth and has a longer duration. Patients often exhibit: persistent low mood; loss of interest; marked fatigue; changes in sleep and appetite; low self-esteem; and a lack of interest in or willingness to care for the infant. Some patients may also experience significant anxiety, feelings of guilt, or even a sense of worthlessness. In severe cases, self-harm or suicidal thoughts may occur.  Severe psychiatric symptoms include mental disorders such as postpartum psychosis, which, although rare, are extremely dangerous. Patients may experience: hallucinations; delusions; behavioral disturbances; significant impairment of consciousness; extreme mood swings; and thoughts of harming themselves or the infant.  Some patients may develop delusions such as “someone is trying to harm the child,” requiring urgent psychiatric intervention.  During clinical assessment, the first priority is to inquire about the duration and severity of the mood symptoms. Brief fluctuations are more indicative of low mood, while symptoms persisting for more than two weeks warrant suspicion of depression. Second, sleep patterns should be assessed. Inability to fall asleep even when the infant is sleeping often indicates a more severe psychological issue. Third, changes in interests and motivation should be explored. For example, has the patient lost interest in previously enjoyed activities, or is she reluctant to care for the infant?  It is also crucial to assess the presence of: marked anxiety; feelings of hopelessness; self-blame; and feelings of worthlessness.  Of particular importance is inquiring about thoughts of self-harm, suicide, or harming the infant, as this is central to the risk assessment. At the same time, assess family support, marital relationships, and past history of mental illness. If necessary, use screening tools such as the Edinburgh Postnatal Depression Scale.  If the patient exhibits hallucinations, delusions, abnormal behavior, or dangerous tendencies, an immediate psychiatric consultation or even hospitalization is required. Therefore, the assessment of postpartum mental health issues should not only focus on “feeling down,” but more importantly, on identifying persistent depression and dangerous psychiatric symptoms. | Mood changes are common among women after childbirth, but it is clinically important to distinguish between normal emotional fluctuations and mental illness in order to provide timely intervention.  Postpartum blues are typically a mild, temporary reaction. Women may experience crying, heightened sensitivity, and irritability, but they are generally still able to function normally and care for their infants. Symptoms usually resolve on their own within 1 to 2 weeks after childbirth.  Postpartum depression, however, is more severe and lasts longer. Patients experience marked low mood, loss of interest, and increased fatigue, often accompanied by sleep disturbances and changes in appetite. They may feel “inadequate” or even lack an emotional connection with their infant. Severe psychiatric symptoms, such as postpartum psychosis, constitute a psychiatric emergency. Patients may experience auditory hallucinations, delusions, confusion, and abnormal behavior.  Such patients may harm themselves or their infants, making them extremely high-risk.  During the assessment, the first step is to ask how long the mood changes have persisted. Transient mood fluctuations are common, but if symptoms last longer than two weeks, depression should be suspected. Second, determine whether the patient is still able to perform daily caregiving tasks. If she is clearly unable to care for herself or the infant, this indicates a more serious problem. Sleep patterns are also a key focus. Severe insomnia—especially the inability to sleep “even when given the opportunity”—often indicates depression or anxiety.  Additionally, ask whether the patient frequently feels hopeless; experiences persistent self-blame; has lost interest in life; or exhibits marked anxiety and fear.  Risk assessment is particularly important; direct questions should be asked regarding whether the patient has thoughts of harming themselves or their child.  Additionally, it is essential to assess the patient’s history of mental illness, family support, and life stressors. If the patient exhibits hallucinations, delusions, disorganized speech, or abnormal behavior, they should be immediately referred to a psychiatric specialist for evaluation.  Therefore, the key to postpartum psychological assessment is distinguishing between “transient emotional fluctuations” and “persistent mental health issues causing functional impairment.” | Women often experience varying degrees of emotional changes after childbirth, but not all cases of low mood constitute a medical condition. Clinically, it is important to distinguish between postpartum low mood, postpartum depression, and severe mental disorders.  Postpartum low mood is the most common condition and is often related to hormonal changes and fatigue. Patients may exhibit symptoms such as tearfulness, emotional sensitivity, and anxiety, but these symptoms are typically mild and gradually subside within two weeks.  Postpartum depression, on the other hand, is a persistent mental health condition. Patients experience prolonged feelings of sadness, helplessness, and loss of interest, and may even be reluctant to communicate with family members. Many patients exhibit significant fatigue, self-doubt, and sleep disturbances. In severe cases, suicidal thoughts may arise.  Severe psychiatric symptoms, such as postpartum psychosis, are even more dangerous. Patients may experience hallucinations, delusions, extreme behavior, confusion, and severe emotional instability. Some patients may even engage in dangerous behaviors that endanger the infant.  During clinical assessment, the first step is to inquire about the duration and severity of the symptoms. Second, functional status should be evaluated. For example, whether the patient is still able to care for the child normally, and whether eating and sleeping patterns are significantly affected.  Key questions include: whether there has been prolonged low mood; whether interest in everything has been lost; and whether there is a persistent feeling of hopelessness or worthlessness.  Special attention should also be given to inquiring about: thoughts of self-harm; suicide plans; and thoughts of harming the infant. Additionally, it is necessary to understand the level of family support and any history of mental illness.  If hallucinations, delusions, abnormal behavior, or a marked decline in reality testing are present, immediate psychiatric emergency care is required.  Therefore, the key to addressing postpartum mental health issues lies in early identification of warning signs and the timely provision of professional help. |
| Correctness（1-5） | 4 | 4 | 4 |
| Completeness（1-5） | 4 | 4 | 3 |
| Guideline consistency（1-5） | 4 | 4 | 4 |
| Safety（1-5） | 5 | 4 | 4 |
| Clinical management appropriateness（1-5） | 5 | 4 | 3 |
| Chinese Reading Difficulty Score | 13.09 | 13.11 | 12.85 |
| Corresponding Education Level (Chinese) | 初中学段 | 初中学段 | 初中学段 |
| Recommended Reading Age (Chinese) | 13岁左右 | 13岁左右 | 13岁左右 |

## 问题 27

| 项目 | 第1次 | 第2次 | 第3次 |
| --- | --- | --- | --- |
| 产褥期临床知识问题 | 产褥期产妇出现自伤、自杀想法或伤害婴儿想法时，应如何进行风险判断和紧急处理？ | | |
| 主要指南来源 | NICE CG192 | | |
| 模型回答 | 产褥期如果产妇出现自伤、自杀或伤害婴儿的想法，属于严重精神心理危险信号，需要立即进行风险评估和紧急干预。这类情况常见于严重产后抑郁、产后精神病或其他精神障碍，若处理不及时，可能危及母婴生命安全。  首先，临床上需要快速进行危险程度判断。医务人员应直接、明确地询问：是否曾想过伤害自己；是否想过结束生命；是否有具体计划；是否曾实施相关行为；是否有伤害婴儿的念头。询问时应保持平静、非指责性态度，因为直接询问不会“诱导”自杀，反而有助于识别高危患者。  其次，需要评估危险性等级。如果仅为短暂消极念头、无具体计划、无实施行为且仍有求助意愿，属于相对较低风险，但仍需严密随访。若患者已经存在：明确自杀计划；准备工具或药物；拒绝照顾婴儿；明显绝望感；幻觉或妄想；伤害婴儿想法；则属于高危状态，需要立即处理。尤其是出现“有人要害孩子”“孩子不应该活”等妄想内容时，要高度怀疑产后精神病。  紧急处理首先应确保安全。绝不能让患者单独照顾婴儿，也不应让其独处。  应立即通知家属并安排专人陪护，移除危险物品，例如刀具、药物、绳索等。  其次，需要立即进行精神专科会诊或转诊。对于存在明确危险行为或精神症状者，应尽快住院治疗，必要时精神科强制保护性住院。同时，应评估患者意识状态、睡眠情况及是否存在药物滥用。  药物治疗通常需要由精神科医生评估后进行，包括抗抑郁药、抗精神病药及必要时镇静治疗。  在哺乳方面，也需根据用药和病情评估是否继续母乳喂养。此外，家庭支持非常重要。家属需要被告知：患者目前属于医疗危机状态；不能简单认为是“想太多”；需要持续陪伴和专业治疗。  因此，产褥期出现自伤或伤婴想法时，处理核心是“立即识别风险、确保母婴安全、紧急精神科干预”。 | 产褥期出现自伤、自杀或伤害婴儿想法，是精神心理急症，需要高度重视。因为部分患者病情进展迅速，尤其是产后精神病，可能在短时间内发生严重后果。  临床评估时，首先要明确患者是否存在真实危险。医务人员应直接询问：有没有想过不想活；有没有伤害自己的计划；有没有伤害孩子的念头；是否听到别人听不到的声音；是否觉得有人要害自己或孩子。  如果患者仅有短暂情绪低落，但能主动求助、仍有现实判断能力，则风险相对较低。  但若出现以下情况，则属于高危：明确自杀计划；准备实施行为；严重失眠；幻觉、妄想；拒绝进食或拒绝照顾孩子；认为孩子“不值得活”。这类情况提示严重精神障碍可能。  紧急处理时，第一原则是保障安全。应立即让家属陪护，避免患者单独与婴儿相处。同时移除危险工具和药物。  第二，应尽快精神科会诊。若患者存在明显精神病性症状、自杀风险或攻击风险，通常需要急诊转诊甚至住院。  第三，应保持稳定、支持性的沟通方式。避免指责、争吵或简单安慰，例如“你不要胡思乱想”。此外，还需关注患者身体状况，例如是否长期失眠、极度疲惫或存在严重焦虑。对于高危患者，单纯家庭照顾往往不足，必须依靠专业精神科治疗。  因此，产褥期出现伤害自己或婴儿的想法时，关键是把它视为真正的医疗危机，而不是“情绪不好”。 | 产褥期如果出现自伤、自杀或伤害婴儿的想法，提示可能存在严重产后抑郁或产后精神病，属于需要立即处理的危险情况。  风险判断时，首先要评估患者想法的严重程度。  需要重点询问：是否只是偶尔闪过念头；是否有持续反复想法；是否已有具体实施计划；是否准备了相关工具；是否出现幻觉或妄想。  例如，患者如果说“孩子活着没有意义”或“有人让我伤害孩子”，危险性极高。  其次，要评估现实功能。如果患者已经无法正常照顾自己或婴儿，长期失眠、拒绝进食或行为异常，则提示病情严重。对于紧急处理，首先必须确保母婴安全。不能让患者单独带孩子，也不能让其独处。需要立即通知家属，并安排持续陪护。同时，应移除所有可能用于自伤的危险物品。  第二，需要立即精神科评估。如果存在明确自杀风险、幻觉、妄想或攻击行为，应尽快送往有精神科能力的医院。  第三，医务人员应保持冷静和支持性沟通。避免否定患者感受，也不要简单说“你要坚强”。严重患者可能需要药物治疗甚至住院管理。部分患者在急性期可能暂时不适合独立哺乳或照顾婴儿。  此外，还应加强家属教育，让家属认识到这是严重疾病，而不是性格问题。  因此，产褥期出现伤害自己或婴儿想法时，处理重点是“快速识别危险、保障安全、立即专科干预”。 |
| 中文回答的英文翻译 English translation of Chinese response | If a new mother experiences thoughts of self-harm, suicide, or harming her infant during the postpartum period, these are serious mental health warning signs that require immediate risk assessment and emergency intervention. Such situations are commonly associated with severe postpartum depression, postpartum psychosis, or other mental disorders; if not addressed promptly, they may endanger the lives of both mother and infant.  First, clinicians must rapidly assess the level of risk. Healthcare providers should ask direct and explicit questions: Have you ever thought about harming yourself? Have you ever thought about ending your life? Do you have a specific plan? Have you ever acted on such thoughts? Do you have thoughts of harming your baby? When asking these questions, maintain a calm, non-judgmental attitude, as direct questioning does not “induce” suicidal behavior but rather helps identify high-risk patients.  Second, the level of risk must be assessed. If the patient has only experienced fleeting negative thoughts, has no specific plan, has not acted on these thoughts, and remains willing to seek help, the risk is relatively low, though close follow-up is still required. However, if the patient exhibits any of the following: a clear suicide plan; preparation of tools or medications; refusal to care for the infant; marked feelings of despair; hallucinations or delusions; or thoughts of harming the infant, the patient is considered high-risk and requires immediate intervention. In particular, delusional content such as “Someone is trying to harm the child” or “ “the child shouldn’t live,” postpartum psychosis should be strongly suspected.  Emergency management must prioritize safety. Under no circumstances should the patient be left alone with the infant or left unattended.  Family members should be notified immediately, and a designated caregiver should be arranged. Remove hazardous items, such as knives, medications, and ropes.  Second, an immediate psychiatric consultation or referral is required. For patients exhibiting clear dangerous behaviors or psychiatric symptoms, hospitalization should be arranged as soon as possible, with involuntary protective admission to a psychiatric ward if necessary. At the same time, the patient’s level of consciousness, sleep patterns, and potential substance abuse should be assessed.  Pharmacological treatment typically requires evaluation by a psychiatrist and may include antidepressants, antipsychotics, and sedatives if necessary.  Regarding breastfeeding, a decision on whether to continue should be made based on the specific medications and the patient’s clinical condition. Furthermore, family support is crucial. Family members should be informed that the patient is currently in a medical crisis; this should not be dismissed as simply “overthinking”; and that continuous support and professional treatment are required.  Therefore, when thoughts of self-harm or harming the infant arise during the postpartum period, the core principles of management are “immediate risk identification, ensuring the safety of mother and infant, and urgent psychiatric intervention.” | Thoughts of self-harm, suicide, or harming the infant during the postpartum period constitute a psychiatric emergency that requires serious attention. Because the condition can progress rapidly in some patients—particularly in cases of postpartum psychosis—serious consequences may arise within a short period of time.  During clinical assessment, the first step is to determine whether the patient poses a genuine risk. Healthcare providers should ask directly: Have you thought about not wanting to live? “Do you have a plan to harm yourself?”; “Do you have thoughts of harming your child?”; “Do you hear voices that others cannot hear?”; and “Do you feel that someone is trying to harm you or your child?”  If the patient experiences only brief episodes of low mood but is able to seek help proactively and retains a sense of reality, the risk is relatively low.  However, the following situations indicate high risk: a clear suicide plan; preparation to carry out the act; severe insomnia; hallucinations or delusions; refusal to eat or care for the child; or the belief that the child is “not worth living.” These signs may indicate a serious mental disorder.  When providing emergency care, the first priority is ensuring safety. Family members should be asked to stay with the patient immediately to prevent the patient from being alone with the infant. At the same time, remove dangerous tools and medications.  Second, a psychiatric consultation should be arranged as soon as possible. If the patient exhibits obvious psychotic symptoms, a risk of suicide, or a risk of aggression, emergency referral or even hospitalization is usually required.  Third, maintain a calm and supportive communication style. Avoid blame, arguments, or simplistic reassurances such as “Don’t let your mind wander.” Additionally, pay attention to the patient’s physical condition, such as chronic insomnia, extreme fatigue, or severe anxiety. For high-risk patients, family care alone is often insufficient; professional psychiatric treatment is essential.  Therefore, when thoughts of harming oneself or the infant arise during the postpartum period, it is crucial to treat this as a genuine medical crisis rather than merely “feeling down.” | If thoughts of self-harm, suicide, or harming the infant arise during the postpartum period, this may indicate severe postpartum depression or postpartum psychosis, which constitutes a dangerous situation requiring immediate intervention.  When assessing risk, the first step is to evaluate the severity of the patient’s thoughts.  Key questions to ask include: Are these merely fleeting thoughts? Are the thoughts persistent and recurrent? Has the patient already formulated a specific plan of action? Have they prepared any tools for this purpose? Are they experiencing hallucinations or delusions?  For example, if the patient says, “There is no point in the child living” or “Someone is telling me to harm the child,” the risk is extremely high.  Second, assess the patient’s functional status. If the patient is unable to care for themselves or the infant, suffers from prolonged insomnia, refuses to eat, or exhibits abnormal behavior, this indicates a severe condition. For emergency management, the safety of both mother and infant must be ensured first. The patient must not be left alone with the child or left unattended. Family members must be notified immediately, and continuous supervision must be arranged. At the same time, all potentially dangerous items that could be used for self-harm should be removed.  Second, an immediate psychiatric evaluation is required. If there is a clear risk of suicide, hallucinations, delusions, or aggressive behavior, the patient should be transferred as soon as possible to a hospital with psychiatric capabilities.  Third, healthcare providers should remain calm and communicate in a supportive manner. Avoid dismissing the patient’s feelings, and do not simply tell them to “be strong.” Severe cases may require medication or even hospitalization. Some patients may be temporarily unable to breastfeed or care for their infants independently during the acute phase.  Furthermore, family education should be strengthened to help family members recognize that this is a serious illness, not a personality issue.  Therefore, when thoughts of harming oneself or the infant arise during the postpartum period, the key to management is “rapid risk identification, ensuring safety, and immediate specialized intervention.” |
| Correctness（1-5） | 4 | 4 | 4 |
| Completeness（1-5） | 4 | 4 | 4 |
| Guideline consistency（1-5） | 4 | 4 | 4 |
| Safety（1-5） | 5 | 4 | 4 |
| Clinical management appropriateness（1-5） | 4 | 4 | 4 |
| Chinese Reading Difficulty Score | 13.06 | 13.01 | 13.16 |
| Corresponding Education Level (Chinese) | 初中学段 | 初中学段 | 初中学段 |
| Recommended Reading Age (Chinese) | 13岁左右 | 13岁左右 | 13岁左右 |

## 问题 28

| 项目 | 第1次 | 第2次 | 第3次 |
| --- | --- | --- | --- |
| 产褥期临床知识问题 | 产后恢复性生活前应考虑哪些生理、心理和伤口恢复因素？何时需要进一步评估？ | | |
| 主要指南来源 | NICE NG194；中国孕产妇健康管理服务规范 | | |
| 模型回答 | 产后恢复性生活是产褥期健康管理中的重要内容，但恢复时间并不完全固定，需要综合考虑生理恢复、心理状态及伤口愈合情况，避免因过早性生活导致疼痛、感染或心理压力。  首先，需要评估生理恢复情况。一般建议至少待产褥期结束后，即产后约6周，再考虑恢复性生活。因为此时子宫复旧基本完成，恶露通常已经干净，宫颈口也逐渐恢复关闭，可降低感染风险。其次，应重点关注恶露情况。如果恶露仍未干净、仍有明显阴道流血或分泌物异常，则不建议恢复性生活，以免增加感染机会。第三，要评估会阴或腹部伤口恢复情况。阴道分娩后存在会阴侧切或裂伤的女性，应确认伤口已愈合、无明显疼痛和压痛。剖宫产女性则需评估腹部切口恢复情况，避免因疼痛影响性生活。  此外，还需考虑盆底功能恢复。部分女性产后存在阴道松弛、盆底坠胀或尿失禁等问题，如果症状明显，应先进行盆底康复评估。  激素变化也是重要因素。哺乳期雌激素水平较低，容易出现阴道干涩和性交疼痛，因此必要时可指导使用润滑剂。心理因素同样非常关键。产后女性可能因疲劳、照护婴儿压力、体型变化或对疼痛的担忧，而出现性欲下降。部分患者还可能因为分娩创伤产生恐惧心理，因此伴侣沟通和心理支持非常重要。  如果产妇存在以下情况，则需要进一步评估后再恢复性生活：持续会阴疼痛；性交疼痛明显；恶露长期不净；阴道异常出血；明显盆底坠胀；尿失禁；严重阴道干涩；或持续情绪低落、对性生活明显恐惧。对于严重会阴裂伤、盆底损伤或产后抑郁患者，更应谨慎评估。  同时，还需进行避孕指导。因为即使月经未恢复，产后也可能排卵，因此恢复性生活前应明确避孕方案。  因此，产后性生活恢复不仅仅是“时间到了”，而是需要综合评估身体、心理及关系适应情况。 | 产后恢复性生活需要综合评估女性身体恢复情况和心理状态，不能单纯以“满月”作为标准。  首先，应考虑生殖系统恢复情况。一般建议在产后6周左右、产褥期结束后恢复性生活，因为此时子宫和宫颈基本恢复正常。其次，恶露是否完全停止非常重要。如果仍有阴道流血或异常分泌物，说明生殖道尚未完全恢复，不适合性生活。第三，应重点评估伤口恢复情况。阴道分娩后若有会阴侧切或裂伤，需要确认伤口愈合良好，没有明显疼痛。剖宫产患者则需关注腹部切口是否恢复，避免牵拉疼痛。  盆底功能恢复也是重要因素。部分女性产后会出现阴道松弛、漏尿或盆底下坠感，如果症状明显，性生活可能加重不适。  此外，哺乳期由于雌激素水平较低，阴道分泌减少，容易出现阴道干涩和性交疼痛。  心理方面同样需要关注。很多女性在产后会因为疲劳、睡眠不足及育儿压力而暂时缺乏性欲。部分女性还会担心疼痛、再次怀孕或身体形象改变。如果存在明显焦虑、恐惧或夫妻关系紧张，也会影响性生活恢复。  以下情况提示需要进一步评估：性生活疼痛明显；伤口持续疼痛；阴道异常出血；盆底功能障碍；长期恶露不净；严重阴道干涩；持续情绪问题。  对于严重会阴裂伤、盆腔器官脱垂或产后抑郁患者，更应在医生指导下恢复性生活。  同时，应提前做好避孕指导，避免短期再次妊娠。  因此，产后性生活恢复应强调“身体恢复+心理准备+安全避孕”三方面综合评估。 | 产后恢复性生活前，需要综合考虑产妇身体恢复、心理状态以及分娩损伤恢复情况。  首先，从生理角度来看，一般建议至少在产后6周以后恢复性生活。因为此时子宫复旧基本完成，恶露停止，感染风险相对较低。其次，要确认恶露已经完全干净。如果仍有阴道流血或异味分泌物，则提示恢复尚未完成。第三，应评估伤口愈合情况。有会阴侧切或裂伤的女性，需要确保伤口无疼痛、无裂开及无感染。剖宫产患者则应确认腹部切口恢复良好。  盆底功能恢复同样重要。如果存在明显漏尿、阴道坠胀或盆底疼痛，应先进行盆底康复。  哺乳期女性由于激素变化，容易出现阴道干涩，因此性生活时可能疼痛，需要适当使用润滑措施。  心理方面，很多产妇在产后会经历疲劳、焦虑和角色转换压力。部分女性对性生活存在恐惧，尤其经历难产或严重会阴损伤者更明显。因此，伴侣理解和沟通非常重要。  以下情况需要进一步评估：持续性交疼痛；会阴长期不适；阴道异常出血；恶露不净；严重盆底症状；情绪低落或明显焦虑。  对于有严重盆底损伤、产后抑郁或性生活恐惧的患者，应及时转诊相关专科。  此外，恢复性生活前必须进行避孕咨询，因为产后即使未恢复月经，也可能已经恢复排卵。  因此，产后性生活恢复应建立在“恢复良好、心理接受、安全避孕”的基础上，而不是单纯依据时间决定。 |
| 中文回答的英文翻译 English translation of Chinese response | Resuming sexual activity after childbirth is an important aspect of postpartum health management, but the timing for resumption is not set in stone. It requires a comprehensive consideration of physical recovery, psychological state, and wound healing to avoid pain, infection, or psychological stress caused by resuming sexual activity too soon.  First, it is necessary to assess physical recovery. It is generally recommended to wait until at least the end of the postpartum period—approximately 6 weeks after childbirth—before considering resuming sexual activity. By this time, uterine involution is largely complete, lochia has typically ceased, and the cervical os has gradually closed, reducing the risk of infection. Second, close attention should be paid to the status of lochia. If lochia has not yet ceased, there is still significant vaginal bleeding, or there are abnormal secretions, resuming sexual activity is not recommended to avoid increasing the risk of infection. Third, the healing of perineal or abdominal incisions must be assessed. Women who underwent a vaginal delivery with an episiotomy or perineal tear should confirm that the wound has healed and that there is no significant pain or tenderness. Women who had a cesarean section should assess the healing of the abdominal incision to avoid pain interfering with sexual activity.  Additionally, the recovery of pelvic floor function must be considered. Some women experience vaginal laxity, pelvic heaviness, or urinary incontinence after childbirth. If symptoms are pronounced, a pelvic floor rehabilitation assessment should be conducted first.  Hormonal changes are also a significant factor. During breastfeeding, lower estrogen levels can lead to vaginal dryness and pain during intercourse; therefore, the use of lubricants may be recommended when necessary. Psychological factors are equally critical. Postpartum women may experience a decreased sex drive due to fatigue, the stress of caring for an infant, body image changes, or concerns about pain. Some patients may also develop fear due to the trauma of childbirth, making communication with a partner and psychological support essential.  If a postpartum woman experiences any of the following conditions, further evaluation is required before resuming sexual activity: persistent perineal pain; significant pain during intercourse; prolonged lochia; abnormal vaginal bleeding; marked pelvic heaviness; urinary incontinence; severe vaginal dryness; or persistent low mood and significant fear of sexual activity. Particular caution is warranted in the evaluation of patients with severe perineal tears, pelvic floor injuries, or postpartum depression.  Additionally, contraception guidance is essential. Since ovulation can occur even before menstruation resumes, a clear contraceptive plan should be established before resuming sexual activity.  Therefore, resuming sexual activity after childbirth is not merely a matter of “the time being right,” but requires a comprehensive assessment of physical, psychological, and relational readiness. | Resuming sexual activity after childbirth requires a comprehensive assessment of a woman’s physical recovery and psychological state; it should not be based solely on the “one-month postpartum” milestone.  First, the recovery of the reproductive system should be considered. It is generally recommended to resume sexual activity around six weeks postpartum, after the postpartum period has ended, as the uterus and cervix have largely returned to normal by this time. Second, it is crucial to confirm that lochia has completely ceased. If there is still vaginal bleeding or abnormal discharge, it indicates that the reproductive tract has not fully recovered, and sexual activity is not appropriate. Third, the healing of any wounds should be carefully assessed. For women who had a vaginal delivery with an episiotomy or perineal tear, it is necessary to confirm that the wound has healed well and that there is no significant pain. For those who had a cesarean section, attention should be paid to the healing of the abdominal incision to avoid pain caused by stretching.  The recovery of pelvic floor function is also a key factor. Some women may experience vaginal laxity, urinary incontinence, or a sensation of pelvic organ prolapse after childbirth. If these symptoms are pronounced, sexual activity may exacerbate the discomfort.  Additionally, during breastfeeding, lower estrogen levels lead to reduced vaginal secretions, making vaginal dryness and pain during intercourse more likely.  Psychological factors also require attention. Many women experience a temporary lack of sexual desire after childbirth due to fatigue, sleep deprivation, and the stress of childcare. Some women may also worry about pain, the possibility of another pregnancy, or changes in their body image. Significant anxiety, fear, or marital tension can also hinder the resumption of sexual activity.  The following conditions indicate the need for further evaluation: significant pain during sexual intercourse; persistent wound pain; abnormal vaginal bleeding; pelvic floor dysfunction; prolonged lochia; severe vaginal dryness; or persistent emotional issues.  For women with severe perineal tears, pelvic organ prolapse, or postpartum depression, resuming sexual activity should be done under medical supervision.  At the same time, contraceptive guidance should be provided in advance to prevent another pregnancy in the short term.  Therefore, the resumption of sexual activity after childbirth should emphasize a comprehensive assessment of three aspects: “physical recovery + psychological readiness + safe contraception.” | Before resuming sexual activity after childbirth, it is necessary to comprehensively consider the mother’s physical recovery, psychological state, and the healing of any birth-related injuries.  First, from a physiological perspective, it is generally recommended to wait at least six weeks after childbirth before resuming sexual activity. By this time, uterine involution is largely complete, lochia has ceased, and the risk of infection is relatively low. Second, it is important to confirm that lochia has completely stopped. If there is still vaginal bleeding or foul-smelling discharge, this indicates that recovery is not yet complete. Third, the healing of any wounds should be assessed. Women who have undergone an episiotomy or suffered a perineal tear must ensure that the wound is pain-free, has not reopened, and is free of infection. Those who have had a cesarean section should confirm that the abdominal incision has healed well.  The recovery of pelvic floor function is equally important. If there is significant urinary incontinence, vaginal heaviness, or pelvic floor pain, pelvic floor rehabilitation should be undertaken first.  Due to hormonal changes, breastfeeding women are prone to vaginal dryness, which may cause pain during sexual intercourse; therefore, appropriate lubrication is necessary.  Psychologically, many new mothers experience fatigue, anxiety, and stress related to role transition after childbirth. Some women may feel fear regarding sexual activity, particularly those who have experienced difficult labor or severe perineal trauma. Therefore, understanding and communication between partners are crucial.  Further evaluation is warranted in the following situations: persistent pain during intercourse; prolonged perineal discomfort; abnormal vaginal bleeding; prolonged lochia; severe pelvic floor symptoms; or low mood or marked anxiety.  Patients with severe pelvic floor injuries, postpartum depression, or fear of sexual intercourse should be promptly referred to the appropriate specialists.  In addition, contraception counseling is essential before resuming sexual activity, as ovulation may have resumed even if menstruation has not yet returned.  Therefore, the resumption of sexual activity after childbirth should be based on “good physical recovery, psychological readiness, and safe contraception,” rather than being determined solely by the passage of time. |
| Correctness（1-5） | 4 | 4 | 4 |
| Completeness（1-5） | 4 | 4 | 4 |
| Guideline consistency（1-5） | 4 | 4 | 4 |
| Safety（1-5） | 4 | 4 | 4 |
| Clinical management appropriateness（1-5） | 4 | 3 | 4 |
| Chinese Reading Difficulty Score | 13.87 | 13.48 | 13.55 |
| Corresponding Education Level (Chinese) | 初中学段 | 初中学段 | 初中学段 |
| Recommended Reading Age (Chinese) | 14岁左右 | 13岁左右 | 13岁左右 |

## 问题 29

| 项目 | 第1次 | 第2次 | 第3次 |
| --- | --- | --- | --- |
| 产褥期临床知识问题 | 为什么产后早期需要进行避孕咨询？哺乳期和非哺乳期产妇选择避孕方法时应考虑哪些因素？ | | |
| 主要指南来源 | FSRH产后避孕指南；NICE NG194；中国孕产妇健康管理服务规范 | | |
| 模型回答 | 产后早期进行避孕咨询非常重要，因为很多女性误认为“没有来月经就不会怀孕”或“哺乳期间绝对安全”，从而忽视避孕。但实际上，部分女性在月经恢复前就已经恢复排卵，因此如果没有采取避孕措施，可能发生意外妊娠。  产后短期再次妊娠会增加多种风险，包括：子宫恢复不完全；贫血和营养消耗加重；早产和低出生体重儿风险增加；剖宫产切口愈合不良甚至子宫破裂风险增加。  因此，世界卫生组织通常建议两次妊娠间隔至少18个月以上。  另外，产后女性在恢复性生活前往往很少主动咨询避孕，因此医务人员应在产褥期主动开展健康教育。  在选择避孕方式时，首先需要考虑是否处于哺乳期。对于哺乳期产妇，应优先选择不影响乳汁分泌及婴儿安全的方法。  常见适合哺乳期的方法包括：避孕套；宫内节育器；单纯孕激素类避孕方法；以及哺乳闭经法。  其中哺乳闭经法只有在：产后6个月内；纯母乳喂养；月经尚未来潮；这三个条件同时满足时，避孕效果才相对可靠。而含雌激素的复方口服避孕药，在产后早期通常不推荐，因为可能影响乳汁分泌，并增加血栓风险。  对于非哺乳期产妇，避孕选择范围更广。可根据年龄、基础疾病、生育计划及个人偏好选择：复方口服避孕药；宫内节育器；皮下埋植；避孕套等。  此外，还需要考虑以下因素：是否剖宫产；是否存在高血压、糖尿病或血栓风险；性生活恢复情况；以及长期还是短期避孕需求。例如，有血栓高风险者应避免含雌激素避孕方法。对于希望长期避孕且短期无再生育计划者，宫内节育器是较理想选择。  因此，产后避孕咨询不仅是“告诉产妇怎么避孕”，更是结合母乳喂养、健康状况和生育计划进行个体化指导。 | 产后早期进行避孕咨询非常必要，因为很多女性在月经恢复前就可能已经恢复排卵。如果没有有效避孕，容易发生非计划妊娠。产后短时间再次怀孕，对母婴健康都不利。因为此时女性身体尚未完全恢复，尤其剖宫产女性，子宫瘢痕恢复需要时间。  此外，频繁妊娠还会增加：贫血；营养不良；胎盘异常；早产等风险。  因此，产后避孕属于产褥期健康管理的重要内容。在选择避孕方式时，首先要区分哺乳期和非哺乳期。哺乳期女性避孕时，需要特别考虑对乳汁分泌和婴儿安全的影响。  一般优先推荐：避孕套；宫内节育器；单纯孕激素避孕方法。哺乳闭经法也可作为短期避孕方式，但必须满足严格条件，否则失败率较高。而含雌激素避孕药在产后早期尤其哺乳期通常不首选，因为可能减少乳汁分泌。  非哺乳期女性则可选择更多方法，例如：复方短效口服避孕药；宫内节育器；皮下埋植；安全套等。  避孕方法选择时，还应综合考虑：年龄；是否有基础疾病；未来生育计划；分娩方式；以及个人接受程度。  例如：高血压或血栓风险患者不宜使用含雌激素方法；近期有再次妊娠计划者适合可逆性避孕方法。此外，还应强调“双重保护”理念。避孕套不仅避孕，还能预防性传播疾病。  因此，产后避孕咨询核心是帮助女性在“安全恢复”和“未来生育计划”之间取得平衡。 | 产后早期进行避孕咨询的原因，主要是很多女性在月经恢复前就可能已经恢复排卵，因此即使没有来月经，也存在怀孕可能。  如果产后短期再次妊娠，容易增加母体负担，并提高早产、贫血及妊娠并发症风险。对于剖宫产女性，间隔过短还可能增加子宫瘢痕妊娠和子宫破裂风险。因此，避孕咨询应作为产后健康管理的重要内容之一。  哺乳期选择避孕方法时，首先要考虑是否影响乳汁分泌。一般不推荐产后早期使用含雌激素避孕药，因为可能抑制泌乳并增加血栓风险。  适合哺乳期的方法包括：避孕套；宫内节育器；孕激素类避孕方式；以及哺乳闭经法。  但哺乳闭经法并非绝对安全，仅在纯母乳喂养且月经未恢复的情况下效果较好。非哺乳期女性则避孕选择相对更多。  例如：复方口服避孕药；避孕贴；皮下埋植；宫内节育器等。  此外，选择避孕方法时还应综合评估：年龄；是否有高血压、糖尿病等基础疾病；是否存在血栓高风险；未来是否还计划生育。  长期无生育计划者可考虑长效避孕方式；近期有再孕计划者则更适合短效可逆方法。  同时，还应关注性生活恢复情况及伴侣配合程度。  因此，产后避孕不是简单“选一种方法”，而是需要结合哺乳状态、健康状况和生育需求进行个体化指导。 |
| 中文回答的英文翻译 English translation of Chinese response | Contraceptive counseling in the early postpartum period is crucial, as many women mistakenly believe that “they cannot get pregnant if they haven’t resumed menstruation” or that “breastfeeding provides absolute protection,” leading them to neglect contraception. In reality, however, some women resume ovulation before their menstrual periods return; therefore, an unintended pregnancy may occur if contraceptive measures are not taken.  A subsequent pregnancy shortly after childbirth increases the risk of various complications, including: incomplete uterine recovery; worsening anemia and nutritional depletion; increased risk of preterm birth and low birth weight; and increased risk of poor healing of the cesarean section incision or even uterine rupture.  Therefore, the World Health Organization generally recommends an interval of at least 18 months between pregnancies.  Additionally, postpartum women rarely seek contraception proactively before resuming sexual activity; therefore, healthcare providers should proactively provide health education during the postpartum period.  When selecting a contraceptive method, the first consideration should be whether the woman is breastfeeding. For breastfeeding mothers, priority should be given to methods that do not affect milk production or the infant’s safety.  Common methods suitable for breastfeeding include: condoms; intrauterine devices (IUDs); progestin-only contraceptives; and the lactational amenorrhea method.  Among these, the lactational amenorrhea method is only relatively reliable for contraception when all three of the following conditions are met: within 6 months postpartum; exclusive breastfeeding; and menstruation has not yet resumed. Combined oral contraceptives containing estrogen are generally not recommended in the early postpartum period, as they may affect milk production and increase the risk of blood clots.  For non-breastfeeding mothers, the range of contraceptive options is broader. Based on age, underlying medical conditions, family planning goals, and personal preferences, they may choose from: combined oral contraceptives; intrauterine devices (IUDs); subdermal implants; or condoms.  Additionally, the following factors should be considered: whether a cesarean section was performed; the presence of hypertension, diabetes, or a risk of thrombosis; the status of sexual activity; and whether long-term or short-term contraception is needed. For example, women at high risk of thrombosis should avoid estrogen-containing contraceptive methods. For those seeking long-term contraception with no immediate plans for further childbearing, an intrauterine device (IUD) is an ideal choice.  Therefore, postpartum contraceptive counseling is not merely about “telling women how to prevent pregnancy,” but rather providing individualized guidance that takes into account breastfeeding, health status, and family planning goals. | Contraceptive counseling in the early postpartum period is essential, as many women may resume ovulation before their menstrual periods return. Without effective contraception, unplanned pregnancies are likely to occur. Becoming pregnant again shortly after childbirth is detrimental to the health of both mother and baby. This is because a woman’s body has not yet fully recovered, particularly in the case of women who have had a cesarean section, as the uterine scar requires time to heal.  Furthermore, frequent pregnancies increase the risk of anemia, malnutrition, placental abnormalities, and preterm birth.  Therefore, postpartum contraception is a crucial component of postpartum health management. When selecting a contraceptive method, it is essential to distinguish between breastfeeding and non-breastfeeding periods. For breastfeeding women, special consideration must be given to the impact on milk production and infant safety.  Generally, the following are recommended as first-line options: condoms; intrauterine devices (IUDs); and progestin-only methods. The lactational amenorrhea method can also serve as a short-term contraceptive option, but it must meet strict criteria; otherwise, the failure rate is high. Estrogen-containing contraceptives are typically not the first choice in the early postpartum period, especially during breastfeeding, as they may reduce milk production.  Women who are not breastfeeding have more options available, such as: combined short-acting oral contraceptives; intrauterine devices (IUDs); subdermal implants; and condoms.  When selecting a contraceptive method, the following factors should also be comprehensively considered: age; presence of underlying medical conditions; future fertility plans; mode of delivery; and personal acceptance.  For example, women with hypertension or a risk of thrombosis should avoid estrogen-containing methods, while those planning another pregnancy in the near future are better suited for reversible contraceptive methods. Additionally, the concept of “dual protection” should be emphasized. Condoms not only prevent pregnancy but also protect against sexually transmitted infections.  Therefore, the core of postpartum contraceptive counseling is to help women strike a balance between “safe recovery” and “future fertility plans.” | The primary reason for providing contraceptive counseling in the early postpartum period is that many women may resume ovulation before their menstrual periods return; therefore, even if menstruation has not yet resumed, there is still a possibility of pregnancy.  A subsequent pregnancy shortly after childbirth can place a greater burden on the mother and increase the risk of preterm birth, anemia, and pregnancy complications. For women who have had a cesarean section, a short interval between pregnancies may also increase the risk of placenta accreta and uterine rupture. Therefore, contraceptive counseling should be an essential component of postpartum health management.  When selecting a contraceptive method during breastfeeding, the first consideration should be whether it affects milk production. Estrogen-containing contraceptives are generally not recommended in the early postpartum period, as they may suppress lactation and increase the risk of blood clots.  Methods suitable for breastfeeding include: condoms; intrauterine devices (IUDs); progestin-only contraceptives; and the lactational amenorrhea method.  However, the lactational amenorrhea method is not absolutely reliable; it is most effective only in cases of exclusive breastfeeding where menstruation has not yet resumed. Women who are not breastfeeding have a relatively wider range of contraceptive options.  For example: combined oral contraceptives; contraceptive patches; subdermal implants; and intrauterine devices (IUDs).  In addition, when selecting a contraceptive method, a comprehensive assessment should be made of: age; the presence of underlying conditions such as hypertension or diabetes; the risk of thrombosis; future family planning intentions.  Those with no long-term plans for pregnancy may consider long-acting contraceptive methods; those planning to conceive again in the near future are better suited for short-acting, reversible methods.  At the same time, attention should be paid to the resumption of sexual activity and the partner’s level of cooperation.  Therefore, postpartum contraception is not simply a matter of “choosing a method,” but requires individualized guidance that takes into account breastfeeding status, health conditions, and family planning needs. |
| Correctness（1-5） | 4 | 4 | 4 |
| Completeness（1-5） | 4 | 4 | 3 |
| Guideline consistency（1-5） | 4 | 4 | 4 |
| Safety（1-5） | 4 | 4 | 4 |
| Clinical management appropriateness（1-5） | 3 | 3 | 3 |
| Chinese Reading Difficulty Score | 13.52 | 13.32 | 13.59 |
| Corresponding Education Level (Chinese) | 初中学段 | 初中学段 | 初中学段 |
| Recommended Reading Age (Chinese) | 13岁左右 | 13岁左右 | 13岁左右 |

## 问题 30

| 项目 | 第1次 | 第2次 | 第3次 |
| --- | --- | --- | --- |
| 产褥期临床知识问题 | 制定产褥期母体健康教育方案时，应包括哪些核心内容，以保证产妇能够识别异常情况并获得及时帮助？ | | |
| 主要指南来源 | WHO 2022；NICE NG194；中国孕产妇健康管理服务规范 | | |
| 模型回答 | 制定产褥期母体健康教育方案时，核心目标是帮助产妇及家属了解正常恢复过程、识别危险信号，并在出现异常时及时就医，从而降低产后并发症风险，保障母婴安全。  首先，应包括产褥期基本生理恢复知识。向产妇解释正常恶露变化、子宫复旧、乳汁分泌及月经恢复等内容，帮助其区分正常现象与异常表现。其次，要重点进行危险信号教育。这是健康教育最重要部分之一。  应明确告知以下症状需及时就医：阴道大量出血或血块增多；持续高热；恶露恶臭；剧烈腹痛；胸闷气促；单侧下肢肿痛；持续头痛或视物模糊；抽搐；明显情绪异常；以及自伤或伤婴想法等。  第三，应加强伤口护理指导。包括：会阴伤口和剖宫产切口清洁方法；观察感染表现；疼痛管理；以及何时需要复诊。  第四，需要进行母乳喂养指导。内容包括：正确哺乳姿势；有效含接；乳房护理；识别乳腺炎及乳汁淤积等问题。同时，应指导产妇识别婴儿摄入是否充足。  第五，要开展盆底及康复教育。包括：早期活动的重要性；盆底肌训练；预防尿失禁和盆底功能障碍；以及运动恢复原则。  第六，应进行心理健康教育。帮助产妇了解产后情绪变化，识别产后抑郁和严重精神症状，并强调家属支持的重要性。  第七，应加强慢病随访教育。对于妊娠期高血压、糖尿病等患者，应说明血压、血糖复查及长期随访的重要性。  此外，还必须包括避孕和性生活恢复指导。帮助产妇了解恢复性生活时间、避孕必要性及适合的避孕方式。  健康教育还应强调：规律复查；按时产后42天检查；必要时及时转诊。在实施方式上，应尽量采用通俗易懂语言，并结合书面资料、视频或家属共同教育，提高依从性。  因此，完整的产褥期健康教育不仅是“告诉产妇注意什么”，更是帮助其建立主动识别风险和寻求帮助的能力。 | 产褥期健康教育是产后管理的重要组成部分，其目标是帮助产妇安全恢复，并提高异常情况的识别能力。  首先，应介绍产褥期正常生理变化。例如恶露颜色变化、子宫恢复、乳房泌乳及情绪波动等，让产妇了解哪些属于正常恢复过程。  其次，危险症状识别必须作为重点内容。应明确告知产妇以下情况需要立即就医：阴道出血突然增多；发热；恶露异味；伤口红肿渗液；胸闷呼吸困难；单侧腿肿痛；严重头痛；视力异常；抽搐；以及明显心理异常。  第三，需要进行伤口护理教育。包括会阴及剖宫产切口的清洁、观察感染迹象以及疼痛缓解方法。母乳喂养指导也十分重要。应教会产妇正确哺乳姿势、乳头护理及识别乳汁淤积和乳腺炎。同时，还应指导如何判断婴儿是否摄入足够乳汁。在身体恢复方面，应强调早期活动的重要性，并指导适度运动和盆底肌训练。心理健康教育不可忽视。应帮助产妇和家属认识产后情绪低落与产后抑郁的区别，并鼓励主动求助。  对于有妊娠期高血压或糖尿病病史的女性，还应强调血压、血糖复查及长期代谢管理。  此外，应进行避孕教育。很多女性不知道产后月经恢复前也可能排卵，因此需提前选择合适避孕方式。教育中还应说明何时需要复查，例如产后42天复诊的重要性。在形式上，应尽量简单直观，并鼓励家属共同参与，因为家庭支持对产后恢复非常关键。  因此，产褥期健康教育的核心是帮助产妇“知道什么是正常、什么是危险、何时需要求助”。 | 产褥期母体健康教育方案，应围绕“促进恢复、预防并发症、识别危险信号和及时就医”四个目标展开。  首先，应向产妇讲解正常产后恢复过程。例如：恶露逐渐减少；子宫逐渐恢复；乳房泌乳建立；以及情绪波动等。这样有助于减少不必要焦虑。  其次，必须重点教育危险信号识别。包括：大量阴道流血；持续腹痛；高热；恶露恶臭；乳房红肿热痛；切口感染；胸闷气促；单侧腿肿痛；严重头痛和视物模糊；以及自伤、自杀或伤害婴儿想法。这些都属于需要尽快就医的情况。  第三，应进行伤口护理和个人卫生指导。包括会阴及剖宫产切口清洁、保持干燥以及观察感染表现。母乳喂养教育同样重要。需要指导正确含接姿势、乳房护理及识别乳汁淤积和乳腺炎。同时，应教会产妇判断婴儿是否吃饱。  康复指导方面，应强调：早期活动；合理饮食；盆底肌训练；逐渐恢复运动。  心理健康教育也非常关键。应帮助产妇和家属了解产后情绪变化，并识别产后抑郁和严重精神症状。此外，对于妊娠期高血压或糖尿病患者，应强调长期随访和代谢管理。避孕指导也应纳入教育内容，包括性生活恢复时间及适合的避孕方式。  最后，还应明确告知复诊时间及紧急就医渠道，确保产妇在出现问题时能够及时获得帮助。  因此，一个完整的产褥期健康教育方案，不仅是知识宣教，更是提高产妇自我管理和风险识别能力的重要措施。 |
| 中文回答的英文翻译 English translation of Chinese response | When developing a maternal health education program for the postpartum period, the core objective is to help new mothers and their families understand the normal recovery process, recognize warning signs, and seek medical attention promptly if abnormalities arise, thereby reducing the risk of postpartum complications and ensuring the safety of both mother and baby.  First, the program should cover basic knowledge of physiological recovery during the postpartum period. Explain to new mothers the normal changes in lochia, uterine involution, milk production, and the return of menstruation, helping them distinguish between normal and abnormal symptoms. Second, education on warning signs should be emphasized. This is one of the most critical components of health education.  It should be clearly communicated that immediate medical attention is required for the following symptoms: heavy vaginal bleeding or an increase in blood clots; persistent high fever; foul-smelling lochia; severe abdominal pain; chest tightness and shortness of breath; swelling and pain in one lower limb; persistent headache or blurred vision; seizures; significant emotional disturbances; and thoughts of harming oneself or the infant.  Third, guidance on wound care should be reinforced. This includes: methods for cleaning perineal wounds and cesarean incisions; monitoring for signs of infection; pain management; and determining when a follow-up visit is necessary.  Fourth, breastfeeding guidance is necessary. This should cover: proper breastfeeding positions; effective latch-on; breast care; and recognition of issues such as mastitis and milk stasis. Additionally, mothers should be taught to assess whether their infants are feeding adequately.  Fifth, pelvic floor and postpartum rehabilitation education should be provided. This includes: the importance of early mobilization; pelvic floor muscle exercises; prevention of urinary incontinence and pelvic floor dysfunction; and principles of exercise recovery.  Sixth, mental health education should be provided. This helps new mothers understand postpartum emotional changes, recognize postpartum depression and severe psychiatric symptoms, and emphasizes the importance of family support.  Seventh, education on follow-up for chronic conditions should be strengthened. For patients with conditions such as gestational hypertension or diabetes, the importance of follow-up blood pressure and blood glucose monitoring, as well as long-term follow-up, should be explained.  In addition, guidance on contraception and resuming sexual activity must be included. This helps new mothers understand the appropriate timing for resuming sexual activity, the necessity of contraception, and suitable contraceptive methods.  Health education should also emphasize: regular follow-up visits; attending the 42-day postpartum checkup on time; and timely referral when necessary. In terms of delivery methods, plain language should be used as much as possible, combined with written materials, videos, or family-involved education to improve compliance.  Therefore, comprehensive postpartum health education is not merely about “telling mothers what to watch out for,” but rather about helping them develop the ability to proactively identify risks and seek help. | Postpartum health education is a vital component of postpartum care, aimed at helping new mothers recover safely and improving their ability to recognize abnormal conditions.  First, normal physiological changes during the postpartum period should be explained. For example, changes in the color of lochia, uterine involution, lactation, and emotional fluctuations should be discussed to help new mothers understand what constitutes a normal recovery process.  Second, recognizing warning signs must be a key focus. New mothers should be clearly informed that they need to seek immediate medical attention if they experience any of the following: a sudden increase in vaginal bleeding; fever; foul-smelling lochia; redness, swelling, or discharge from the wound; chest tightness or difficulty breathing; swelling and pain in one leg; severe headache; vision changes; seizures; or significant psychological distress.  Third, education on wound care is essential. This includes cleaning the perineal and cesarean incisions, monitoring for signs of infection, and methods for pain relief. Breastfeeding guidance is also crucial. New mothers should be taught proper breastfeeding positions, nipple care, and how to recognize milk stasis and mastitis. Additionally, they should be instructed on how to determine if the infant is receiving sufficient milk. Regarding physical recovery, the importance of early mobilization should be emphasized, and guidance should be provided on moderate exercise and pelvic floor muscle exercises. Mental health education must not be overlooked. Mothers and their families should be helped to understand the difference between postpartum blues and postpartum depression, and encouraged to seek help proactively.  For women with a history of gestational hypertension or diabetes, the importance of follow-up blood pressure and blood glucose monitoring, as well as long-term metabolic management, should be emphasized.  In addition, contraception education should be provided. Many women are unaware that ovulation can occur before menstruation resumes postpartum; therefore, they need to select an appropriate contraceptive method in advance. Education should also explain when follow-up visits are necessary, such as the importance of the 42-day postpartum checkup. The format should be as simple and intuitive as possible, and family members should be encouraged to participate, as family support is crucial for postpartum recovery.  Therefore, the core of postpartum health education is to help new mothers “know what is normal, what is dangerous, and when to seek help.” | A postpartum maternal health education program should focus on four key objectives: “promoting recovery, preventing complications, recognizing warning signs, and seeking timely medical care.”  First, new mothers should be educated about the normal postpartum recovery process. This includes, for example, the gradual decrease in lochia; the gradual return of the uterus to its normal size; the establishment of milk production; and emotional fluctuations. This helps reduce unnecessary anxiety.  Second, education must emphasize the recognition of warning signs. These include: heavy vaginal bleeding; persistent abdominal pain; high fever; foul-smelling lochia; redness, swelling, heat, or pain in the breasts; incision infection; chest tightness or shortness of breath; swelling and pain in one leg; severe headache and blurred vision; and thoughts of self-harm, suicide, or harming the infant. These are all situations requiring immediate medical attention.  Third, guidance on wound care and personal hygiene should be provided. This includes cleaning and keeping the perineal area and cesarean section incision dry, as well as monitoring for signs of infection. Breastfeeding education is equally important. Guidance should cover proper latching techniques, breast care, and how to recognize milk stasis and mastitis. Additionally, mothers should be taught how to determine if their baby is getting enough milk.  Regarding rehabilitation guidance, the following should be emphasized: early mobilization; a balanced diet; pelvic floor muscle exercises; and a gradual return to physical activity.  Mental health education is also crucial. Mothers and their families should be helped to understand postpartum emotional changes and to recognize postpartum depression and severe psychiatric symptoms. Furthermore, for patients with gestational hypertension or diabetes, the importance of long-term follow-up and metabolic management should be emphasized. Contraceptive guidance should also be included in the educational program, covering the appropriate timing for resuming sexual activity and suitable contraceptive methods.  Finally, follow-up appointment dates and emergency medical contact information should be clearly communicated to ensure that mothers can seek timely assistance if problems arise.  Therefore, a comprehensive postpartum health education program is not merely about disseminating knowledge; it is a vital measure for enhancing mothers’ self-management and risk identification capabilities. |
| Correctness（1-5） | 4 | 4 | 4 |
| Completeness（1-5） | 4 | 4 | 4 |
| Guideline consistency（1-5） | 4 | 4 | 4 |
| Safety（1-5） | 5 | 5 | 4 |
| Clinical management appropriateness（1-5） | 4 | 4 | 4 |
| Chinese Reading Difficulty Score | 13.54 | 13.63 | 14.92 |
| Corresponding Education Level (Chinese) | 初中学段 | 初中学段 | 高中学段 |
| Recommended Reading Age (Chinese) | 13岁左右 | 14岁左右 | 15岁左右 |

# Guideline sources / 指南来源

- WHO recommendations on maternal and newborn care for a positive postnatal experience, 2022.
- NICE guideline NG194: Postnatal care.
- ACOG Committee Opinion No. 736: Optimizing Postpartum Care.
- 国家基本公共卫生服务规范（第三版）——孕产妇健康管理服务规范。
- NICE guideline CG192: Antenatal and postnatal mental health.
- FSRH Clinical Guideline: Contraception After Pregnancy.
- RCOG Green-top Guideline No. 37a: Reducing the Risk of Thrombosis and Embolism during Pregnancy and the Puerperium.
