# Supplementary material for: Beyond accuracy: a three-stage evaluation of large language models for postpartum health consultation and nursing decision support
Source: Front Public Health. 2026 Oct 20;14:1961647. doi: 10.3389/fpubh.2026.1961647 (PMC13727638; doi:10.3389/fpubh.2026.1961647)
Supplement: Supplementary file 11 [file Table_8.docx]

**Supplementary Table S8. Stage 1 DeepSeek V3 English clinical knowledge responses and readability outcomes**

*30 postpartum maternal health clinical knowledge questions; each question was independently submitted to DeepSeek V3 three times.*

| Study stage | Stage 1: Clinical knowledge questions on postpartum maternal health |
| --- | --- |
| Model under evaluation | DeepSeek V3 |
| Exact model/version displayed | DeepSeek V3 Deep Thinking |
| Access platform | Official DeepSeek web interface |
| Testing period | 1 May to 30 June 2026 |
| Web browsing/search | Off |
| Reasoning/deep-thinking mode | On; DeepSeek V3 Deep Thinking |
| External files or reference materials uploaded | No |
| Submission procedure | Each question was entered into a new independent conversation and submitted to the model three times. |
| Unified prompt | Act as an evidence-informed obstetrics clinician and answer the following clinical knowledge question about postpartum maternal health. Base your answer on authoritative guidelines and clinical principles. Include key assessment points, possible causes, initial management principles, red flags, and referral indications. Avoid fabricating uncertain information. |
| Evaluator | Not applicable (automated readability assessment only) |
| Language | English |

# **Readability assessment**

Clinical expert scoring was not performed for the English-language responses. These responses were used exclusively for automated Flesch–Kincaid readability assessment.

## Question 1

| Item | Attempt 1 | Attempt 2 | Attempt 3 |
| --- | --- | --- | --- |
| Clinical knowledge question | What is the definition of the puerperium, and which domains should be prioritized in postpartum maternal health management? | | |
| Main guideline source(s) | WHO 2022; ACOG Optimizing Postpartum Care | | |
| Model response | The puerperium is defined as the period from delivery of the placenta until approximately 6 weeks (42 days) postpartum, during which the mother’s body—especially the reproductive system—returns to a non-pregnant state. Priority domains in postpartum management include: (1) uterine involution and hemorrhage monitoring, (2) perineal or cesarean wound healing, (3) lactation support and breast health, (4) blood pressure screening for preeclampsia persistence, (5) mental health assessment for postpartum depression, and (6) family planning counseling. Early detection of infection (endometritis, mastitis, UTI) is also critical. | The puerperium traditionally covers the first 6–8 weeks after childbirth, but physiologically it can extend to full tissue remodeling over several months. Prioritized postpartum domains should include: (1) thromboembolism prevention (especially after cesarean or with risk factors), (2) pelvic floor rehabilitation (assess diastasis recti, levator ani injury, urinary incontinence), (3) nutritional restoration (iron, folate, vitamin D, and hydration for lactation), (4) maternal–infant bonding and breastfeeding success, (5) management of postpartum thyroiditis and other endocrine shifts, and (6) chronic disease re-evaluation (e.g., gestational diabetes, hypertensive disorders). Emotional support and screening for PTSD after traumatic birth are also essential. | The puerperium is the post-delivery interval from expulsion of the placenta until return of the reproductive organs to near-pre-pregnancy state, usually 6 weeks, but some guidelines recognize up to 12 weeks for comprehensive care. Priority domains: (1) life-threatening condition vigilance (postpartum hemorrhage, sepsis, stroke, cardiomyopathy), (2) pain management—perineal, back, or uterine cramping during lactation, (3) contraception initiation (LARC as appropriate), (4) mental health—anxiety, depression, psychosis screening at multiple visits, (5) sexual health and resumption of intercourse guidance, (6) support for social determinants (safe housing, domestic violence screening, paid leave, partner support). Integration of telehealth and home visits improves adherence and outcomes. |
| **Flesch–Kincaid Grade Level** | 19.45 | 17.76 | 21.59 |
| **Corresponding English Reading-Difficulty Category** | College graduate: very difficult | College graduate: very difficult | College graduate: very difficult |

## Question 2

| Item | Attempt 1 | Attempt 2 | Attempt 3 |
| --- | --- | --- | --- |
| Clinical knowledge question | What are the main purposes of postnatal home visits and postpartum follow-up, and what should healthcare professionals ask about and examine in the early postpartum period? | | |
| Main guideline source(s) | WHO 2022; NICE NG194; Chinese Maternal Health Management Specification | | |
| Model response | The main purposes of postnatal home visits and follow-up are to: reduce maternal and neonatal mortality, detect complications early (hemorrhage, infection, thromboembolism, severe hypertension), support breastfeeding, assess mental health, and ensure infant growth. In the early postpartum period (first 2 weeks), healthcare professionals should ask about: lochia color/odor/amount, perineal or abdominal wound pain, fever/chills, headache with visual changes, calf pain/swelling, urinary/fecal incontinence, breastfeeding difficulties, and mood (sadness, anxiety, intrusive thoughts). Examination includes: vital signs (BP, pulse, temperature), uterine fundal height and tenderness, perineal/cesarean incision inspection, breast engorgement/cracks/mastitis signs, and lower limb edema/Homan’s sign. | Postnatal home visits aim to bridge the gap between hospital discharge and clinic follow-up, especially for vulnerable populations, by enabling direct observation of mother–infant interaction, home safety, and feeding technique. Purposes include: reinforcing discharge instructions, administering vitamin K to the newborn if missed, and screening for postpartum depression in a familiar environment. Early assessment should ask about: unusual fatigue, palpitations (possible anemia or cardiomyopathy), dysuria (UTI or urethral trauma), hemorrhoid pain, sleep deprivation coping, partner support, and any thoughts of harming self or baby. Examination covers: mucous membrane color (pallor), abdominal muscle diastasis (fingerbreadth test), perineal healing (redness, discharge, gaping), breast symmetry and redness, bowel sounds (constipation/ileus), and newborn weight/pulse oximetry if indicated. | Primary purposes of postpartum follow-up (including home visits) are to identify “silent” postpartum complications (late-onset preeclampsia, occult infection, venous thromboembolism), offer contraception counseling, and provide psychosocial support—especially for those without transportation or childcare. Healthcare professionals in the early period (days 3–10) must ask about: return to prepregnancy medications (antihypertensives, antidiabetics), persistent lochia rubra (subinvolution or retained products), dyspareunia concerns, lactation mastitis risk factors, and newborn jaundice/feeding patterns. Examination includes: fundal height (should decrease ~1 cm/day), cervical os patency if there’s bleeding, perineal edema, C-section scar for seepage or hematoma, thyroid palpation (postpartum thyroiditis can appear week 1–4), and reflexes/clonus for preeclampsia. Document also maternal weight and hemoglobin if anemia suspected. |
| **Flesch–Kincaid Grade Level** | 21.76 | 20.63 | 21.48 |
| **Corresponding English Reading-Difficulty Category** | College graduate: very difficult | College graduate: very difficult | College graduate: very difficult |

## Question 3

| Item | Attempt 1 | Attempt 2 | Attempt 3 |
| --- | --- | --- | --- |
| Clinical knowledge question | Which maternal symptoms or signs during the puerperium should be regarded as red flags requiring timely medical evaluation or referral? | | |
| Main guideline source(s) | NICE NG194; Chinese Maternal Health Management Specification | | |
| Model response | Red flags during the puerperium requiring urgent evaluation include:  Hemorrhage: Sudden increase in lochia (soaking >1 pad/hour), large clots (>egg-size), or return to bright red bleeding after having turned brown.  Severe headache that does not respond to acetaminophen, especially with visual disturbances (flashing lights, blurring) – may indicate preeclampsia or reversible cerebral vasoconstriction syndrome.  Chest pain or dyspnea – possible pulmonary embolism, peripartum cardiomyopathy, or amniotic fluid embolism sequelae.  Calf pain/swelling (unilateral) – deep vein thrombosis.  Fever >38.0°C (100.4°F) with chills or foul lochia – endometritis or sepsis.  Seizure or altered mental status – eclampsia, stroke, or meningitis. | Additional red flags often overlooked but critical:  Wound complications: Perineal or cesarean incision with spreading erythema, purulent drainage, dehiscence, or severe pain disproportionate to exam – necrotizing fasciitis or surgical site infection.  Inability to urinate beyond 6 hours after delivery or after catheter removal – postpartum urinary retention that can lead to bladder overdistension and long-term dysfunction.  Severe perineal or pelvic pressure with constipation or sensation of a “bulge” – possible pelvic hematoma, rectus sheath hematoma, or uterine prolapse/eviscration (rare but surgical emergency).  Suicidal or infanticidal thoughts – postpartum psychosis or severe depression requiring immediate psychiatric referral.  Sudden severe abdominal pain unrelieved by analgesics – consider retained products of conception with infection, bowel obstruction, or appendicitis. | From a systems-based perspective, urgent referral is warranted for:  Hemodynamic instability – tachycardia (>110 bpm), hypotension (systolic <90 mmHg), pallor, syncope – rule out occult hemorrhage or septic shock.  New-onset or worsening hypertension (≥160/110 mmHg) with or without symptoms – hypertensive emergency requiring antihypertensive therapy and possible readmission.  Respiratory symptoms – hypoxia (O2 saturation <95%), dry cough or hemoptysis (pulmonary edema from cardiomyopathy or fluid overload).  Severe breast signs – entire breast swollen, red, hot, with high fever (mastitis) ± fluctuance (abscess); if not responding to antibiotics within 48 hours, need ultrasound and drainage.  Acute leg pain, pallor, or pulselessness – rare but consider arterial thrombosis (especially with thrombophilia).  Persistent vomiting or inability to tolerate oral intake – preeclampsia, diabetic ketoacidosis (gestational or preexisting diabetes), or bowel obstruction. |
| **Flesch–Kincaid Grade Level** | 12.18 | 20.48 | 18.02 |
| **Corresponding English Reading-Difficulty Category** | College: difficult | College graduate: very difficult | College graduate: very difficult |

## Question 4

| Item | Attempt 1 | Attempt 2 | Attempt 3 |
| --- | --- | --- | --- |
| Clinical knowledge question | How do the color, amount, and duration of normal lochia usually change, and which abnormal lochia findings require further assessment? | | |
| Main guideline source(s) | NICE NG194; Chinese Maternal Health Management Specification | | |
| Model response | Normal lochia follows a predictable pattern:  Lochia rubra (days 1–3): bright to dark red, moderate to heavy flow (similar to a heavy menstrual period), small clots ≤1 cm are acceptable, fleshy odor.  Lochia serosa (days 4–10): pinkish-brown to yellowish, serosanguineous, lighter flow (requires pad change 2–4x/day).  Lochia alba (days 11– up to 6 weeks): yellowish-white, creamy, scant spotting or discharge.  Abnormal findings requiring further assessment:  Prolonged rubra (>3 days without transition to serosa) or recurrent rubra after having changed to alba – possible retained products of conception (RPOC) or subinvolution.  Large clots (>2.5 cm / egg-sized) or soaking >1 pad/hour for >2 hours – delayed postpartum hemorrhage.  Foul, fishy, or purulent odor – suggests endometritis or infected RPOC.  Sudden cessation of flow with pelvic pain – consider cervical stenosis from hematometra. | Color progression:  Rubra (hemoglobin breakdown) → serosa (hemosiderin + leukocytes + debris) → alba (mucus, decidual cells, bacteria).  Amount: Total cumulative lochia averages 200–300 mL; heavy days 1–2, then decreasing.  Concerning findings:  Watery, serosanguineous discharge persisting >2 weeks with associated fever – consider endometritis even without foul odor.  Persistent lochia serosa >14 days with lower abdominal pain – retained placental fragments (RPOC) should be investigated by ultrasound.  Complete absence of lochia within 24h postpartum with suprapubic pain and enlarged uterus – cervical obstruction or hematometra (after cesarean or cervical surgery).  Bright red bleeding after 5–7 days often triggered by overactivity – subinvolution of the placental site; if heavy, rule out pseudoaneurysm or arteriovenous malformation (rare). | Physiologic timeline varies:  Primiparas often have shorter lochia duration (≈3 weeks), multiparas up to 6 weeks.  Breastfeeding accelerates involution and may reduce lochia duration but can also cause intermittent gushes (post-nursing contractions).  Red flags requiring further assessment (including ultrasound, labs, or exam):  Lochia rubra persisting >10 days – sensitivity for RPOC moderate; combine with history of incomplete delivery or placenta accreta spectrum.  Any lochia with fever >38°C, uterine tenderness, tachycardia – treat as endometritis; need pelvic exam + endometrial culture.  Sudden increase in volume (≥2 pads/hour) after day 3 – late postpartum hemorrhage workup (RPOC, uterine atony, or coagulation disorder).  Sandpaper-like or tissue-like fragments in lochia – retained membranes.  Unilateral pelvic pain with purulent lochia – consider septic pelvic thrombophlebitis or adnexal infection (not just endometritis). |
| **Flesch–Kincaid Grade Level** | 11.79 | 17.41 | 16.11 |
| **Corresponding English Reading-Difficulty Category** | Grades 10–12: fairly difficult | College graduate: very difficult | College graduate: very difficult |

## Question 5

| Item | Attempt 1 | Attempt 2 | Attempt 3 |
| --- | --- | --- | --- |
| Clinical knowledge question | How should a sudden increase in vaginal bleeding after the first 24 hours postpartum and during the puerperium be clinically assessed? | | |
| Main guideline source(s) | NICE NG194 | | |
| Model response | Sudden increased bleeding after the first 24 hours (late postpartum hemorrhage, 24h–6 weeks) requires stepwise assessment:  1.Hemodynamic status: Check pulse, BP, shock index (>0.9), pallor, altered consciousness. Unstable patients require immediate resuscitation and transfer.  2.History: Timing (day postpartum), provoking factors (exercise, sexual intercourse, breastfeeding), associated pain (crampy vs constant vs severe), fever, retained placenta history.  3.Physical exam:  Uterine size and tone – boggy uterus suggests subinvolution or retained products; tender uterus suggests endometritis.  Speculum exam (without blind curettage) – identify source: cervical laceration, vaginal hematoma, retained tissue protruding through os.  Bimanual – cervical patency, uterine tenderness, adnexal masses.  4.Bedside ultrasound: Endometrial thickness (>10–15 mm with vascularity suggests retained products of conception - RPOC); subinvolution of placental site (linear vascular channel).  5.Labs: CBC, coagulation profile, β-hCG (if trophoblastic disease suspected). | Clinical assessment prioritizes distinguishing structural causes from functional causes:  Step 1 – Exclude life-threatening bleeding: If saturation of pads >1 per hour for >2 hours, hypotension, or tachycardia → IV access, crystalloid, oxytocin (if boggy uterus), tranexamic acid, and consider uterine artery embolization or surgery.  Step 2 – Focal physical clues:  Boggy uterus + clots → uterine atony (less common after 24h but can occur with retained membranes).  Firm, tender, larger-than-expected uterus → subinvolution or RPOC.  Foul discharge + fever + bleeding → endometritis with RPOC.  Severe localized pain (lateral to uterus) → broad ligament hematoma or pseudoaneurysm.  Step 3 – Imaging: Transvaginal ultrasound with color Doppler – look for:  Retained products (heterogeneous mass with vascularity, especially with “vascular pedicle” sign).  Subinvolution of placental site (large, tortuous vessels without mass).  Uterine artery pseudoaneurysm (to-and-fro flow on Doppler).  Step 4 – Lab: D-dimer less useful (normally elevated postpartum); perform coagulation studies if bleeding prolonged. | Focused clinical algorithm for puerperal bleeding increase:  Initial assessment (5 minutes) – PQRST: Precipitating events (delivery type, manual placental removal), Quantity (pad count, clots size), Relief (passing clots relieves cramping?), Symptoms (dizziness, syncope), Timing (day 2–14 common for RPOC; day 10–21 for pseudoaneurysm).  Key clinical features to differentiate –  RPOC: intermittent heavy bleeding + cramping + open cervical os + heterogenous echogenic mass on ultrasound.  Subinvolution: persistent heavy bleeding, uterus remains 12–14 weeks size at 2 weeks, Doppler shows prominent vessels at placental site.  Pseudoaneurysm: sudden massive bleeding (can be episodic, “sentinel bleed”), often after cesarean or myomectomy, requires angiography.  Endometritis: bleeding + fever + uterine tenderness + leukocytosis.  Examination pearls: Never perform sharp curettage in late postpartum (high perforation risk). Office endometrial biopsy or gentle suction curettage under ultrasound guidance if imaging confirms RPOC.  Referral triggers: Hemoglobin drop >2 g/dL, need for blood transfusion, failed medical management (methylergonovine or misoprostol for atony), or persistent bleeding after 2 weeks → gynecologic consultation for possible hysteroscopy or embolization. |
| **Flesch–Kincaid Grade Level** | 13.50 | 15.31 | 17.22 |
| **Corresponding English Reading-Difficulty Category** | College: difficult | College: difficult | College graduate: very difficult |

## Question 6

| Item | Attempt 1 | Attempt 2 | Attempt 3 |
| --- | --- | --- | --- |
| Clinical knowledge question | What possible causes should be considered when a postpartum woman presents with heavy vaginal bleeding, blood clots, abdominal pain, fever, or foul-smelling lochia? | | |
| Main guideline source(s) | NICE NG194; Chinese Maternal Health Management Specification | | |
| Model response | When a postpartum woman presents with heavy bleeding, clots, abdominal pain, fever, or foul lochia, consider the following causes systematically:  Endometritis – most common cause of fever + foul lochia + uterine tenderness. Pain is usually suprapubic, constant; bleeding can be moderate.  Retained products of conception (RPOC) – presents with heavy or intermittent bleeding, clots, crampy abdominal pain, open cervical os; fever may develop if superinfection occurs.  Subinvolution of the placental site – heavy bleeding (often without fever) beyond day 10–14, uterus remains enlarged, Doppler shows prominent vessels.  Septic pelvic thrombophlebitis – high fever, tachycardia, abdominal pain, but lochia may not be foul; bleeding not predominant.  Uterine artery pseudoaneurysm – episodic gushes of bright red blood, often painless or with mild pain, history of cesarean or myomectomy.  Pelvic abscess or necrotizing fasciitis – severe pain, high fever, septic appearance; foul discharge with possible tissue necrosis.  Degenerating or infected leiomyoma – known fibroids; acute pain, fever, bleeding. | Differential diagnosis based on symptom clusters (combination of bleeding, pain, fever, discharge):  Bleeding + clots + pain (without fever) → RPOC, subinvolution, pseudoaneurysm, or late uterine atony (rare after 24h).  Bleeding + foul lochia + fever + uterine tenderness → endometritis or infected RPOC.  Bleeding + severe unilateral or broad ligament pain → broad ligament hematoma, pseudoaneurysm rupture, or ovarian vein thrombosis.  Fever + pain + minimal bleeding → septic pelvic thrombophlebitis, acute pyelonephritis (flank pain), appendicitis (rare but possible).  Fever + foul lochia without heavy bleeding → endometritis or infected hematoma.  Massive sudden bleeding without pain → pseudoaneurysm or arteriovenous malformation (AVM).  Fever + heavy bleeding + shock → septic shock (Group A Streptococcus, Clostridium sordellii) or toxic shock syndrome – medical emergency.  Less common but critical: retained membranes, placental polyp, gestational trophoblastic disease (hCG elevated), and uterine rupture (history of scar). | Organized approach for clinical evaluation – use mnemonic “POST-PARTUM”:  P – Placenta: retained fragments or succenturiate lobe.  O – Obstetric trauma: cervical/vaginal lacerations, uterine incision dehiscence (after cesarean).  S – Subinvolution of placental site.  T – Thrombosis (septic pelvic thrombophlebitis, DVT).  P – Pseudoaneurysm / AVM.  A – Abscess (pelvic, adnexal, or wound).  R – Retained blood products (hematometra, lochiometra).  T – Tumor (GTD, degenerated fibroid).  U – Uterine atony (rare but can recur with infection).  M – Myometritis / Endometritis.  Key diagnostic steps:  Ultrasound with Doppler to distinguish RPOC (vascular mass) vs pseudoaneurysm (cystic structure with turbulent flow) vs subinvolution.  Labs: WBC, CRP, blood cultures, endometrial culture (avoid blind swab – use double-lumen catheter to prevent contamination).  CT angiogram if pseudoaneurysm suspected and ultrasound inconclusive.  Surgical exploration if unstable or failed conservative management. |
| **Flesch–Kincaid Grade Level** | 15.54 | 16.43 | 13.55 |
| **Corresponding English Reading-Difficulty Category** | College: difficult | College graduate: very difficult | College: difficult |

## Question 7

| Item | Attempt 1 | Attempt 2 | Attempt 3 |
| --- | --- | --- | --- |
| Clinical knowledge question | How should postpartum uterine involution be assessed, and what clinical problems may be suggested by poor uterine involution? | | |
| Main guideline source(s) | Chinese Maternal Health Management Specification; NICE NG194 | | |
| Model response | Postpartum uterine involution is best assessed by daily palpation of the uterine fundus. On the first day after delivery, the fundus is typically at the level of the umbilicus; it then descends approximately one fingerbreadth (about 1 cm) per day, becoming non-palpable behind the symphysis pubis by day 10-14. Poor involution—where the fundus remains higher than expected or fails to descend—suggests subinvolution. Common clinical problems associated with poor involution include retained products of conception (RPOC), endometritis, or a full bladder displacing the uterus. Uterine fibroids or an overdistended uterus (e.g., from polyhydramnios or multiple gestation) can also mechanically impair involution. | Assessment combines fundal palpation with evaluation of lochia consistency and odor. After uterine massage, a well-involuted uterus feels firm and globular; poor involution yields a boggy, soft fundus. Clinically, poor involution with persistent heavy lochia or clots suggests subinvolution, often linked to retained placental fragments or endometritis. If associated with fever, abdominal pain, and malodorous discharge, suspect postpartum endometritis. Delayed involution beyond two weeks may indicate RPOC requiring sonographic evaluation. A less common but serious cause is uterine artery pseudoaneurysm, which can present with late or intermittent heavy bleeding despite partial involution. | In addition to serial fundal height measurements, use ultrasound to assess endometrial cavity thickness and exclude RPOC or a subinvolution of the placental site. Poor involution (fundus >2 cm above umbilicus at day 5 or >4 cm at day 7) should prompt investigation for retained tissue, but also for underlying conditions like adenomyosis or large submucosal fibroids. Rarely, poor involution with scant lochia and cramping pain suggests a hematometra. Clinically, risk factors include prolonged labor, manual placental removal, uterine atony, and grand multiparity. Persistent subinvolution without infection or retained products may reflect abnormal trophoblast persistence or an exaggerated inflammatory response. |
| **Flesch–Kincaid Grade Level** | 13.75 | 15.70 | 16.75 |
| **Corresponding English Reading-Difficulty Category** | College: difficult | College: difficult | College graduate: very difficult |

## Question 8

| Item | Attempt 1 | Attempt 2 | Attempt 3 |
| --- | --- | --- | --- |
| Clinical knowledge question | What are the common clinical manifestations of puerperal infection, and how should fever, lower abdominal pain, and foul-smelling lochia be managed clinically? | | |
| Main guideline source(s) | NICE NG194; Chinese Maternal Health Management Specification | | |
| Model response | Common manifestations include fever (≥38°C after the first 24 hours postpartum), lower abdominal or pelvic pain, foul-smelling lochia, uterine tenderness, and sometimes systemic signs like chills or tachycardia. The classic triad is fever, pain, and malodorous discharge.  Management:  Fever → Obtain blood cultures and CBC with differential. Start broad-spectrum antibiotics (e.g., clindamycin + gentamicin, or ampicillin-sulbactam) after collecting cultures.  Lower abdominal pain → Palpate for uterine tenderness. Exclude surgical site or adnexal causes. Provide analgesia and uterine massage.  Foul-smelling lochia → Perform speculum exam to collect endocervical and lochia cultures; avoid blind uterine curettage. Consider retained products of conception (RPOC) if pain and fever persist despite antibiotics → then ultrasound and possible gentle evacuation. | Manifestations:  Fever may be low-grade or high, often with rigors if bacteremia.  Lower abdominal pain is typically suprapubic or bilateral lower quadrant, worsened by fundal palpation.  Foul lochia suggests anaerobic or polymicrobial infection (e.g., Group B Strep, E. coli, Bacteroides).  Clinical management:  Fever + foul lochia → Start IV antibiotics without waiting for cultures if patient appears toxic. Common regimen: gentamicin + clindamycin + ampicillin for suspected endometritis.  Lower abdominal pain → Rule out septic pelvic thrombophlebitis (persistent fever despite adequate antibiotics) or appendicitis.  If no improvement in 48-72h, perform pelvic ultrasound to check for RPOC or abscess. Drain any pelvic collection (e.g., via transvaginal or transabdominal route). Avoid NSAIDs if suspecting uterine atony contribution to infection. | Common manifestations include sustained fever >38.5°C, severe lower abdominal pain with guarding, and profuse foul-smelling lochia that may be brownish or purulent. Other signs: tachycardia, ileus, or peritonitis if infection spreads.  Management:  Fever → Monitor q4h; if >39°C or hypotension, consider septic shock protocol. Use acetaminophen for symptomatic fever but not as substitute for antibiotics.  Lower abdominal pain → Perform gentle bimanual exam to assess cervical motion tenderness and adnexal masses. Persistent pain after 48h of antibiotics → CT or MRI to rule out ovarian vein thrombosis or myometritis.  Foul-smelling lochia → Send specimen for Gram stain and anaerobic culture. Avoid instrumentation of the uterine cavity in early infection to prevent bacteremia, unless RPOC with hemorrhage is suspected. If infection progresses despite antibiotics, consider surgical debridement (rare, for necrotizing myometritis). |
| **Flesch–Kincaid Grade Level** | 12.70 | 12.67 | 13.75 |
| **Corresponding English Reading-Difficulty Category** | College: difficult | College: difficult | College: difficult |

## Question 9

| Item | Attempt 1 | Attempt 2 | Attempt 3 |
| --- | --- | --- | --- |
| Clinical knowledge question | What should be included in the assessment of a perineal wound after vaginal delivery, and which findings suggest wound infection or dehiscence? | | |
| Main guideline source(s) | NICE NG194 | | |
| Model response | Assessment should include: visual inspection of the perineum for erythema, edema, ecchymosis, discharge, and approximation of wound edges; palpation for warmth, tenderness, and induration; assessment of pain out of proportion to expected postpartum discomfort; and evaluation of the patient’s ability to void and defecate without worsening pain.  Findings suggesting infection or dehiscence:  Infection – Purulent drainage, foul odor, increasing erythema extending beyond the suture line, fever, and systemic malaise.  Dehiscence – Separation of wound edges (partial or complete) visualized during gentle separation of the labia, sometimes exposing subcutaneous tissue or fascia.  If either is present, obtain a wound culture and consider early opening of the skin sutures to allow drainage. | Assessment should include documenting the time of repair and current postpartum day (infection risk peaks between day 3–7). Evaluate for:  Wound edges: check for loss of normal epithelial approximation.  Palpate the entire perineal body and introitus to identify any fluctuance or deep tenderness, suggesting abscess.  Speculum exam if needed to visualize the posterior vaginal wall extension of the tear.  Look for signs of necrotizing fasciitis (rare but critical: crepitus, rapid progression, severe pain, systemic toxicity).  Findings suggesting infection:  Infection – Spreading cellulitis (>2 cm from wound edge), purulent discharge, persistent fever despite NSAIDs.  Dehiscence – Complete separation of the fourchette or perineal body, with or without visible granulation tissue at the base. Partial dehiscence with a small gap (<1 cm) may still heal secondarily.  High-risk signs – Wound breakdown with exposed fascia → requires operating room evaluation. | Assessment should combine patient-reported symptoms (increasing perineal pain unrelieved by analgesics, sensation of “giving way,” bleeding, or feeling a bulge) with systematic exam:  Gently separate the labia majora.  Use a cotton swab to probe the suture line for tenderness and any sinus tract.  Evaluate for urine or feces leaking through the wound (suggesting rectovaginal or urethrovaginal fistula).  Note wound breakdown and surrounding skin maceration.  Findings suggesting infection or dehiscence:  Infection – Foul-smelling serosanguinous or purulent drainage, wound edges that are hot and shiny, and systemic signs (tachycardia, leukocytosis).  Dehiscence – Total loss of suture integrity, exposing deep tissue; partial dehiscence with gaping but intact deep sutures.  If dehiscence with infection is present: remove all visible sutures, start broad-spectrum antibiotics (e.g., clindamycin + gentamicin or amoxicillin-clavulanate), and advise warm sitz baths plus wound packing. Delayed secondary closure may be considered after infection resolves and healthy granulation tissue appears. |
| **Flesch–Kincaid Grade Level** | 18.96 | 12.55 | 13.58 |
| **Corresponding English Reading-Difficulty Category** | College graduate: very difficult | College: difficult | College: difficult |

## Question 10

| Item | Attempt 1 | Attempt 2 | Attempt 3 |
| --- | --- | --- | --- |
| Clinical knowledge question | What conditions may be associated with persistent or worsening postpartum perineal pain, and how should it be assessed and managed? | | |
| Main guideline source(s) | NICE NG194 | | |
| Model response | Conditions that may cause persistent or worsening perineal pain include:  Infected episiotomy or laceration repair (cellulitis or abscess)  Hematoma (vulvar, vaginal, or ischiorectal fossa)  Retained foreign body (e.g., broken suture, vaginal packing)  Suture granuloma or keloid formation  Perineal wound dehiscence  Assessment:  Visual and bimanual exam (speculum if tolerated) to identify fluctuance, bulging, or purulence.  Pelvic ultrasound or MRI if deep hematoma or abscess is suspected.  CBC, CRP, and wound culture if infection is suspected.  Management:  Hematoma → small (<5 cm) may resolve with ice and compression; large or enlarging requires surgical evacuation.  Infected or dehisced wound → open sutures, drain pus, start broad-spectrum antibiotics (e.g., amoxicillin-clavulanate or clindamycin + gentamicin).  Suture granuloma → removal of retained suture material and topical estrogen cream to promote healing. | Persistent perineal pain beyond 2–3 weeks postpartum may be due to:  Pudendal nerve entrapment (from prolonged labor, forceps delivery, or excessive stretching)  Pelvic floor myofascial pain (hypertonic levator ani or obturator internus muscles)  Coccydynia (posterior perineal and coccygeal pain from trauma during delivery)  Perineal scarring with neuroma formation  Assessment:  Detailed neurological exam: perineal sensation, anal sphincter tone, and voluntary pelvic floor contraction.  Pelvic floor physical therapy evaluation for trigger points.  Diagnostic pudendal nerve block if entrapment suspected.  Management:  Pudendal neuralgia → neuropathic agents (gabapentin, amitriptyline) and physical therapy.  Myofascial pain → manual pelvic floor therapy, vaginal diazepam suppositories, and warm sitz baths.  Coccydynia → coccygeal cushion, NSAIDs, and avoid prolonged sitting.  Symptomatic neuroma → surgical excision (rare) after failed conservative measures (6-12 months). | Less common but critical causes of worsening perineal pain include:  Necrotizing fasciitis (Fournier’s gangrene; rapid progression, systemic toxicity, crepitus)  Perirectal or ischiorectal abscess (often from an unrecognized fourth-degree laceration)  Rectovaginal or urethrovaginal fistula (persistent malodorous discharge and deep pain)  Perineal scar endometriosis (cyclical pain, rare)  Postpartum pelvic girdle pain radiating to perineum  Assessment:  Immediate CT or MRI for necrotizing fasciitis or deep abscess.  Examination under anesthesia for suspected deep infection or fistula.  Multidisciplinary evaluation: gynecology, colorectal surgery, urology, pain medicine, and pelvic floor physiotherapy.  Management:  Necrotizing fasciitis → urgent surgical debridement, broad-spectrum IV antibiotics (carbapenems + vancomycin + clindamycin), intensive care.  Deep abscess → image-guided drainage or surgical incision.  Fistula → delayed repair (≥3-6 months postpartum) after inflammation resolves, often involving specialty surgery.  Endometriosis of episiotomy scar → hormonal suppression or surgical excision.  Persistent idiopathic perineal pain → referral to a chronic pain clinic and pelvic floor rehabilitation. |
| **Flesch–Kincaid Grade Level** | 13.73 | 17.58 | 20.38 |
| **Corresponding English Reading-Difficulty Category** | College: difficult | College graduate: very difficult | College graduate: very difficult |

## Question 11

| Item | Attempt 1 | Attempt 2 | Attempt 3 |
| --- | --- | --- | --- |
| Clinical knowledge question | What are the basic principles of postpartum perineal wound care, and how should women be advised on perineal hygiene and pain management? | | |
| Main guideline source(s) | NICE NG194 | | |
| Model response | Basic principles of postpartum perineal wound care include keeping the area clean and dry, promoting healing, and preventing infection.  Hygiene advice:  Clean the perineum from front to back after each toileting.  Use a peri-bottle filled with warm water to irrigate the area (instead of wiping).  Pat dry gently with a clean, soft cloth or allow to air dry.  Change perineal pads every 3–4 hours and avoid prolonged use of pads with plastic linings.  Take warm sitz baths 2–3 times daily for 10–15 minutes, starting 24 hours after delivery.  Pain management:  Apply ice packs (wrapped in a cloth) to the perineum for the first 24–48 hours (20 minutes on, 20 minutes off).  Topical anesthetic sprays or witch hazel pads (e.g., Tucks pads) can provide local relief.  Encourage acetaminophen or ibuprofen as first-line oral analgesics, avoiding aspirin if breastfeeding is not a concern (aspirin is generally avoided postpartum). | Basic principles: minimize bacterial contamination, reduce friction and moisture, and monitor for signs of infection.  Hygiene advice:  After bowel movements, consider using a handheld showerhead or peri-bottle with dilute betadine or saline (if no allergy).  Avoid tampons, douching, sexual intercourse, and swimming until the wound is fully healed (typically ≥ 4–6 weeks).  Wear loose cotton underwear to allow airflow.  Change pads frequently and avoid using scented wipes or bubble baths.  Pain management:  Moderate pain → scheduled ibuprofen (600–800 mg every 8 hours) plus acetaminophen (500–1000 mg every 6 hours) in alternating intervals.  Severe pain → short-term low-dose opioids (e.g., tramadol or codeine) may be considered if NSAIDs and acetaminophen are insufficient, but be aware of maternal sedation and neonatal effects if breastfeeding (avoid codeine in poor metabolizers).  For burning pain → topical lidocaine 2–5% gel or spray can be used sparingly.  Counsel patients to report worsening pain, fever, or foul discharge. | Basic principles: promote granulation tissue formation, prevent secondary infection, and support patient comfort to enable mobility and breastfeeding.  Hygiene advice:  Routine tap water without antiseptics is as effective as saline or antiseptic solutions for cleaning perineal wounds.  Empty the bladder regularly (every 2–4 hours) to avoid urine pooling on the wound.  For women with extensive lacerations (third/fourth degree), stool softeners (e.g., docusate) and high-fiber diet should be recommended to prevent constipation and reduce strain.  Sitz baths with plain warm water (no additives) reduce pain and improve blood flow without increasing infection risk.  Pain management:  Non-pharmacologic: cold therapy (first 24h) then warm sitz baths (after 24h), positioning to reduce pressure (side-lying or sitting on a soft donut cushion).  Pharmacologic: ibuprofen is preferred for its anti-inflammatory effect on edematous perineal tissues. Avoid excessive use of topical benzocaine (rare methemoglobinemia risk).  If pain persists beyond 2 weeks despite adequate care → reassess for hematoma, infection, or retained suture granuloma. |
| **Flesch–Kincaid Grade Level** | 8.96 | 11.53 | 12.95 |
| **Corresponding English Reading-Difficulty Category** | Grades 8–9: plain English | Grades 10–12: fairly difficult | College: difficult |

## Question 12

| Item | Attempt 1 | Attempt 2 | Attempt 3 |
| --- | --- | --- | --- |
| Clinical knowledge question | What should be observed in an abdominal incision after cesarean delivery, and which findings suggest wound infection, hematoma, or dehiscence? | | |
| Main guideline source(s) | NICE NG194; Chinese Maternal Health Management Specification | | |
| Model response | Observation should include:  Edges: well-approximated vs. separated.  Skin color: normal or erythematous (note extent).  Drainage: serous, serosanguinous, purulent, or none.  Swelling: localized or spreading.  Temperature of the skin (warmth).  Palpation for tenderness and induration.  Findings suggesting:  Infection – Purulent drainage, foul odor, spreading erythema (>2 cm from suture line), warmth, fever, and increasing pain.  Hematoma – Bulging, firm, bluish or tense swelling under or near the incision; may be fluctuant; often without fever initially.  Dehiscence – Partial or complete separation of fascial edges; may expose subcutaneous tissue or, in severe cases, bowel; often preceded by serosanguinous drainage (the “surgical scarlet” sign).  Immediate action: measure erythema size, obtain wound culture, and consider opening 1–2 sutures to assess deep involvement. | Observation should be done daily while hospitalized and taught to the patient for home monitoring:  Inspect the incision with gentle traction on the skin edges.  Use a sterile cotton swab to probe any gap or sinus tract.  Assess for crepitus or subcutaneous air (necrotizing infection).  Compare with contralateral side for asymmetry of swelling.  Findings suggesting:  Infection (usually day 3–7) – Purulent discharge, induration, and systemic signs (tachycardia, fever >38.5°C). Cellulitis without fluctuance may be early infection.  Hematoma (usually <48 hours) – Rapidly expanding subcutaneous mass, significant pain out of proportion, drop in hemoglobin, and sometimes hypotension if large.  Dehiscence (usually day 5–8) – Sudden gush of pink or serous fluid (“sentinel leak”), followed by visible separation of skin and fascial edges; may be partial (fascia intact) or complete (fascial defect with bowel or omentum visible).  Management note: any fascial dehiscence requires immediate surgical evaluation; superficial skin separation may be managed conservatively. | Observation should include monitoring for systemic symptoms as well as local signs:  Fever curve, white blood cell count, and pain trajectory.  Palpation of deeper tissues beyond the visible incision.  Ultrasound if subfascial fluid collection is suspected.  Findings suggesting:  Infection – Necrotizing fasciitis: dusky skin, bullae, crepitus, severe pain that may be disproportionate or loss of sensation over the wound, rapid progression, septic shock.  Hematoma – Underlying myofascial or rectus sheath hematoma: firm, non-compressible mass with overlying skin ecchymosis; may present with marked tachycardia and hypotension even without visible bulging.  Dehiscence – Complete evisceration: bowel loops protruding through the wound; preceded by profuse serosanguinous drainage. Partial dehiscence: probing reveals fascial gap >1 cm without protrusion.  Additional clues:  Infection + hematoma → suspect infected hematoma requiring drainage.  Dehiscence with fever and purulence → infected fascial breakdown requiring debridement.  Blackened or necrotic skin edges → ischemia from tight sutures or hypotension. |
| **Flesch–Kincaid Grade Level** | 11.86 | 13.55 | 14.38 |
| **Corresponding English Reading-Difficulty Category** | Grades 10–12: fairly difficult | College: difficult | College: difficult |

## Question 13

| Item | Attempt 1 | Attempt 2 | Attempt 3 |
| --- | --- | --- | --- |
| Clinical knowledge question | What types of pain are common during the puerperium, and what should be considered when providing analgesia to breastfeeding women? | | |
| Main guideline source(s) | NICE NG194 | | |
| Model response | Common puerperal pain types include:  Perineal pain (from episiotomy, lacerations, or hematoma)  Uterine cramping (afterpains, especially during breastfeeding)  Cesarean incision pain  Breast pain (engorgement, cracked nipples, mastitis)  Back pain (from labor, positioning, or epidural)  When providing analgesia to breastfeeding women:  Acetaminophen (paracetamol) – considered first-line, safe at usual doses (500–1000 mg q6h).  Ibuprofen – preferred NSAID; minimal transfer into breast milk, compatible with breastfeeding.  Avoid aspirin (risk of Reye’s syndrome in infant, theoretical bleeding risk).  Opioids – use cautiously; codeine and tramadol are generally avoided due to variable metabolism and neonatal sedation risk.  Topical anesthetics (lidocaine, benzocaine) – safe for perineal or nipple pain if used sparingly.  Always consider infant age (neonates more vulnerable), maternal liver/kidney function, and potential for drowsiness affecting infant care. | Common pain types:  Afterpains (more severe in multiparas and during oxytocin release with breastfeeding)  Cesarean pain – visceral (from uterine incision) and somatic (from abdominal wall)  Perineal pain – may radiate to buttocks or thighs  Hemorrhoidal pain (common after vaginal delivery)  Analgesia considerations in breastfeeding:  Acetaminophen and NSAIDs (ibuprofen, naproxen) – low infant exposure; ibuprofen has shorter half-life and is preferred for inflammatory pain.  Opioids – if needed (e.g., after cesarean or severe perineal trauma):  Morphine and hydromorphone are acceptable short-term, but monitor infant for sedation and poor feeding.  Codeine contraindicated due to ultra-rapid metabolism leading to neonatal respiratory depression.  Tramadol also avoided (insufficient safety data, risk of serotonin syndrome and seizures in mother).  Ketorolac – can be used for short-term (≤48h) post-cesarean; not for prolonged use.  Regional anesthesia techniques (TAP block for cesarean, pudendal block for perineal pain) reduce need for systemic opioids.  Non-pharmacologic: cool packs, positioning, warm sitz baths, support belts.  Counsel women: if baby becomes unusually sleepy, weak suck, or less responsive, stop opioid and seek help. | Less common but important puerperal pain types:  Neuropathic perineal pain (pudendal neuralgia after forceps or prolonged second stage)  Nipple pain with deep burning (possible Raynaud’s phenomenon or Candida infection)  Pelvic girdle pain (sacroiliac or pubic symphysis dysfunction)  Post-dural puncture headache (not “pain” but requires distinct management)  Analgesia considerations for breastfeeding women:  Neuropathic pain – gabapentin may be used if necessary; infant exposure is low but monitor for sedation. Amitriptyline has higher infant transfer potential; prefer nortriptyline if a TCA is indicated.  Breast pain – treat underlying cause (e.g., lanolin for cracks, fluconazole for thrush). Topical ibuprofen or low-dose oral ibuprofen is safe.  Avoid prolonged opioid use beyond 3–5 days due to neonatal withdrawal risk and maternal constipation.  Multimodal analgesia (acetaminophen + NSAIDs + local blocks) reduces need for opioids.  Deprescribing – taper analgesics as soon as possible; afterpains usually resolve by day 5–7; cesarean pain improves by day 10–14.  Special caution:  Women on SSRIs or other serotonergic drugs should avoid tramadol, meperidine (pethidine), and possibly codeine due to serotonin syndrome risk.  Consider neonatal withdrawal scoring if mother used chronic opioids during pregnancy. |
| **Flesch–Kincaid Grade Level** | 17.78 | 14.72 | 14.79 |
| **Corresponding English Reading-Difficulty Category** | College graduate: very difficult | College: difficult | College: difficult |

## Question 14

| Item | Attempt 1 | Attempt 2 | Attempt 3 |
| --- | --- | --- | --- |
| Clinical knowledge question | How can physiological breast engorgement be distinguished from pathological breast conditions after delivery, and how should healthcare professionals perform an initial assessment? | | |
| Main guideline source(s) | NICE NG194 | | |
| Model response | Physiological breast engorgement typically occurs 48–72 hours postpartum, is bilateral, and presents as generalized, symmetric breast fullness, tenderness, and warmth without erythema. The breasts feel firm but not tense or shiny; milk expression provides relief, and the woman remains afebrile or has low-grade temperature (<38.0°C).  Pathological conditions (e.g., mastitis, abscess) usually appear after day 5–7, often unilateral. Features include a localized, wedge-shaped area of erythema, exquisite tenderness, induration, and often fever >38.5°C with chills and malaise.  Initial assessment by healthcare professionals:  Take temperature and pulse.  Inspect both breasts for symmetry, redness, and skin changes.  Palpate systematically to identify discrete masses, fluctuance, or retroareolar tenderness.  Check ipsilateral axillary lymph nodes.  Ask about systemic symptoms (myalgia, rigors).  Evaluate milk expression: easy flow suggests engorgement; difficulty expressing with purulent or blood-streaked discharge suggests pathology.  If mastitis suspected, send milk culture and CBC; ultrasound if abscess is possible. | Physiological engorgement: milk flows easily once latch is established; breast softens after feeding/pumping. The skin is smooth (not peau d’orange), and there is no nipple discharge except normal milk.  Pathological mastitis or abscess: milk may appear clumpy, blood-tinged, or purulent. Pain continues or worsens after emptying. The breast may feel hot, and the skin may show cellulitic streaks or peau d’orange changes. Abscess presents as a palpable, fluctuant, often tender mass.  Initial assessment steps:  Obtain history: prior mastitis, nipple trauma, ineffective feeding, prolonged engorgement.  Assess infant latch and feeding efficiency.  Perform breast exam with patient supine, arm overhead.  Use bedside ultrasound if available to differentiate edema (engorgement) from fluid collection (abscess).  Check WBC and C-reactive protein (CRP): both elevated in infection.  Inflammatory breast cancer is extremely rare in puerperium but should be considered if erythema persists despite antibiotics.  Key distinguishing maneuver: Therapeutic trial of complete breast emptying – improvement within 4–6 hours supports engorgement; persistent or worsening fever and pain suggests infection. | Physiological engorgement: ultrasound shows diffusely thickened glandular tissue without discrete fluid collections, with prominent ducts filled with milk. No increased vascularity on Doppler.  Pathological conditions:  Mastitis – hypoechoic, ill-defined area with increased vascularity, sometimes with small periductal fluid pockets.  Abscess – well-defined anechoic or complex fluid collection with thick walls and internal debris; surrounding hyperemia.  Rare malignancies (e.g., pregnancy-associated breast cancer) may mimic mastitis – persistent mass after infection treatment.  Initial assessment by healthcare professionals (clinical + imaging + labs):  Perform systematic inspection with patient sitting and supine; note dimpling, nipple inversion, or peau d’orange.  Axillary and supraclavicular lymph node exam.  Order complete blood count (leukocytosis with left shift suggests infection).  C-reactive protein >5 mg/dL favors inflammation/infection.  Breast ultrasound for any mass or persistent focal pain after 48 hours of antibiotics.  If abscess suspected: diagnostic aspiration with 18-gauge needle (purulent content confirms abscess).  Red flags requiring immediate surgical consultation: progressive erythema despite antibiotics, hypotension, rapidly expanding mass, or necrotizing skin changes (very rare). |
| **Flesch–Kincaid Grade Level** | 11.86 | 11.75 | 14.08 |
| **Corresponding English Reading-Difficulty Category** | Grades 10–12: fairly difficult | Grades 10–12: fairly difficult | College: difficult |

## Question 15

| Item | Attempt 1 | Attempt 2 | Attempt 3 |
| --- | --- | --- | --- |
| Clinical knowledge question | What are the typical manifestations of acute mastitis, and how should breast redness, swelling, heat, pain, fever, or systemic symptoms be managed? | | |
| Main guideline source(s) | NICE NG194 | | |
| Model response | Typical manifestations of acute mastitis include:  Local signs – Unilateral breast redness, swelling, warmth, and tenderness, often wedge-shaped.  Systemic signs – Fever (often >38.5°C), chills, myalgia, malaise, and sometimes nausea or tachycardia.  The affected area may feel indurated, and the woman may experience pain during breastfeeding or with palpation.  Management of each component:  Redness/swelling/heat – Encourage continued breastfeeding or pumping from the affected side to drain milk; apply warm compresses before feeding and cold packs after feeding for comfort.  Pain – Ibuprofen (600–800 mg q8h) reduces inflammation; acetaminophen as adjunct.  Fever/systemic symptoms – Acetaminophen for fever; rest and hydration.  Infection – Start dicloxacillin or cephalexin (mastitis is typically Staphylococcus aureus). For penicillin-allergic (non-anaphylactic), use clindamycin or erythromycin. Antibiotics for 10–14 days.  Review symptoms in 48 hours; failure to improve → ultrasound to rule out abscess. | Typical manifestations: Acute mastitis usually presents unilaterally after the first 1–2 weeks postpartum (though can occur earlier). The breast shows a tender, erythematous, warm, swollen area. Fever ≥38.5°C, rigors, and flu-like symptoms are common. Nipple may have fissure or cracking.  Management by symptom:  Redness, swelling, heat, pain –  Continue milk removal (breastfeeding or expression) from affected breast; if too painful, start with unaffected side then switch.  Anti-inflammatory agents (ibuprofen) are first-line for pain and swelling.  Fever –  Acetaminophen 500–1000 mg q6h as needed.  Do not suppress fever with NSAIDs alone if antibiotics are indicated.  Systemic symptoms –  Bed rest, oral hydration, nutrition.  If severely ill (dehydrated, high fever >39°C), consider hospital admission for IV antibiotics (e.g., cefazolin or vancomycin if MRSA risk).  If no improvement within 48–72h of antibiotics →  Perform breast ultrasound.  If abscess is found: needle aspiration (repeat as needed) or surgical drainage; antibiotics alone are insufficient.  Counsel – Stop using expressed milk from the infected side if pus is visible; otherwise, breastfeeding is safe (antibiotics are compatible). | Typical manifestations: Acute postpartum mastitis presents with rapid onset (hours) of a painful, erythematous, edematous breast segment, often associated with fever, chills, and elevated CRP (>5–10 mg/dL) and WBC count (>12,000). Distinguish from engorgement: engorgement is bilateral, afebrile, and resolves with feeding.  Management plan:  Redness/swelling/heat – Therapeutic ultrasound (low-intensity pulsed) may be used in some centers to enhance antibiotic penetration, but mainstay is complete milk removal every 2–3 hours (hand expression or pump) from affected breast.  Pain – Multimodal: ibuprofen + acetaminophen + cool gel packs after feeds. Topical lidocaine (sparingly, washed off before next feed) if severe nipple pain.  Fever – Antipyretics as above; measure temperature every 4–6 hours. Persistent fever >48 hours despite antibiotics → investigate for abscess or resistant organism.  Systemic symptoms (tachycardia, hypotension, rigors) – Admit for IV antibiotics (e.g., ampicillin-sulbactam or piperacillin-tazobactam) + IV fluids. Monitor for sepsis.  Antibiotics – Start empiric covering MRSA if risk factors (hospital-acquired infection, recurrent mastitis, known colonization, severe illness). Use clindamycin or trimethoprim-sulfamethoxazole with MRSA coverage + a beta-lactam (if non-allergic).  Complications – If untreated, mastitis → abscess → sepsis → lactational breast gangrene (extremely rare).  Follow-up – Reassess in 48 hours; if worse, perform diagnostic aspiration and consider ductoscopy (rare) for recurrent infections in same quadrant. |
| **Flesch–Kincaid Grade Level** | 12.82 | 10.52 | 14.40 |
| **Corresponding English Reading-Difficulty Category** | College: difficult | Grades 10–12: fairly difficult | College: difficult |

## Question 16

| Item | Attempt 1 | Attempt 2 | Attempt 3 |
| --- | --- | --- | --- |
| Clinical knowledge question | What are the common causes of nipple pain or cracking during breastfeeding, and how should breastfeeding position and latch be assessed? | | |
| Main guideline source(s) | NICE NG194 | | |
| Model response | Common causes of nipple pain or cracking include:  Poor latch (shallow or asymmetric latch causing nipple compression against the hard palate)  Incorrect positioning (baby not “belly to belly,” straining neck to reach breast)  Nipple trauma from inappropriate use of breast pump or pulling baby off without breaking suction  Tongue-tie (ankyloglossia) restricting proper tongue extension  Engorgement making areola too firm for deep latch  Assessment of position and latch:  Observe the feeding session: baby’s body should be aligned, facing breast, nose opposite nipple.  Check for wide mouth opening (gape) before latch.  Signs of good latch: chin touching breast, more areola visible above baby’s upper lip than below, lips flanged outward, no dimpling of cheeks.  Pain during latch → suspect shallow latch.  Assess nipple after feeding: compressed (lipstick-shaped) or cracked nipple indicates poor latch.  Quick intervention: Correct positioning to deep latch usually resolves pain within days. | Common causes:  Nipple blanching (vasospasm) – often secondary to trauma, Raynaud’s phenomenon, or cold exposure  Candida infection – burning pain that persists between feeds, with pink, shiny, or flaky nipples  Dermatitis or eczema – may be due to soaps, lanolin allergy, or topical creams  Short frenulum (tongue-tie) and lip-tie – limit flange of upper lip and proper tongue cupping  Flat or inverted nipples – make latch difficult  Assessment of position and latch (systematic):  Ask mother to sit comfortably with back support, baby at breast level (use pillows).  Latch assessment:  Tease lower lip with nipple, wait for wide mouth.  Aim nipple toward roof of mouth; baby’s tongue should extend over lower gum.  Listen for rhythmic suck-swallow pattern (clicking suggests poor seal).  Palpate mother’s breast for engorgement; firm areola → express milk before latch.  Examine baby’s oral cavity: lift upper lip (look for lip tie), lift tongue (check frenulum attachment).  Observe for pain on initial latch that improves after 10-15 seconds (normal) vs. pain throughout feed (pathologic latch or thrush).  Tools: Use a nipple assessment score (e.g., LATCH score) to quantify latch quality. | Common causes include above, plus:  Nipple bleb/blister – from friction or blocked duct orifice  Milk fistula (rare) – from previous breast surgery or biopsy  Subacute mastitis – deep nipple pain radiating into breast  Infant oral anomalies (e.g., high arched palate, micrognathia)  Hyperlactation or forceful let-down – baby clamps down to slow flow, causing trauma  Assessment of position and latch (advanced):  Videotape a feeding session for frame-by-frame analysis of latch dynamics.  “Sandwich” technique assessment: mother compresses areola to match baby’s mouth shape.  Evaluate pain timing:  Latch onset pain → shallow latch  Mid-feed pain → tongue-tie or palatal issues  Post-feed persistent burning → Candida or vasospasm  Use infant oral motor exam: check tongue lateralization and lift; a freely mobile tongue should touch the alveolar ridge.  Palpate maternal breast for asymmetric latch preference – suggests underlying breast pathology (e.g., prior abscess scar) or infant torticollis.  Refer for lactation consultant assessment and consider diagnostic ultrasound of nipple-areola complex if persistent unilateral pain without visible cause (exclude Paget’s disease of nipple – extremely rare in puerperium but excludes if non-healing erosion). |
| **Flesch–Kincaid Grade Level** | 13.86 | 10.43 | 14.17 |
| **Corresponding English Reading-Difficulty Category** | College: difficult | Grades 10–12: fairly difficult | College: difficult |

## Question 17

| Item | Attempt 1 | Attempt 2 | Attempt 3 |
| --- | --- | --- | --- |
| Clinical knowledge question | What should be asked and observed when assessing whether breastfeeding is effective? | | |
| Main guideline source(s) | NICE NG194; Chinese Maternal Health Management Specification | | |
| Model response | When assessing breastfeeding effectiveness, the clinician should ask about and observe the following:  Ask the mother:  Does the baby seem content and sleepy after most feeds, or is the baby frequently fussy and still hungry?  How many wet diapers and stools per day (day 3+: ≥6 wet, ≥3-4 yellow seedy stools)?  Is there audible swallowing during feeds (not just sucking)?  Does breastfeeding hurt (pain suggests ineffective latch)?  Does the mother feel breast fullness before feeding and softening after?  Observe:  A full feed (minimum 10-15 minutes of effective swallowing per breast).  Baby’s posture: relaxed, not tense or arching away.  Latch: wide mouth, lips flanged, chin touching breast, more areola above top lip.  Auditory: rhythmic suck-swallow pattern (1-2 sucks per swallow early, 1:1 later).  Weight gain: 20-30 g/day after day 4 (birth weight regained by day 10-14).  Red flags (ineffective): poor weight gain, persistent jaundice, cracked nipples, baby falling asleep within 5 minutes of start. | Effective breastfeeding assessment combines maternal report with objective markers:  Ask:  How often does the baby feed (effective = 8–12 times/24 hours, not constant non-nutritive sucking)?  Does the baby have at least 6 heavy wet diapers/day (pale, not dark urine)?  Are stools yellow and seedy by day 5 (transition from meconium)?  Is the baby back to birth weight by 2 weeks?  Does the mother feel a “let-down” sensation (tingling, milk spraying)?  Observe/measure:  Pre- and post-feed infant weight (test weighing) – a transfer of 50–70 mL per feed suggests effective feeding.  Breast softening after feeding.  Baby’s hands: open and relaxed (not clenched fists, which signal hunger).  Jaw movement: full jaw excursion, not just cheek twitching.  Swallow count: >5 swallows per burst of sucking is adequate.  Use validated tools: The Infant Breastfeeding Assessment Tool (IBFAT) or LATCH score, which assess latch, audible swallowing, nipple condition, and maternal comfort. | To assess breastfeeding effectiveness beyond the immediate newborn period, consider these elements:  Ask (days 3–10 postpartum):  Is the baby sleeping too deeply and missing feeds (risk of hypoglycemia)?  Does the baby lose suction easily, make clicking sounds, or detach repeatedly?  Is there maternal nipple compression after feeds (flattened, ridged, or lipstick-shaped)?  How is milk supply perceived? Effective feeding maintains supply, ineffective feeding may lead to engorgement or low supply.  Observe:  Infant state – Effective feeding: baby is alert at start, relaxed at end, falls off breast spontaneously. Ineffective feeding: frantic rooting, difficulty latching, crying or falling asleep instantly.  Breast changes – Visible milk at baby’s mouth or milk dripping from opposite breast during feed suggests effective transfer.  Post-feed behavior – Satisfied, sleeping 1–3 hours between feeds.  Excretory efficiency – By day 5, consistent weight gain and stooling pattern; fewer than 2 stools/day or continued meconium after day 4 suggests underfeeding.  Special observations for high-risk situations (e.g., preterm, small for gestational age, jaundice, Down syndrome, cleft palate):  Pulse oximetry before and after feed to rule out silent aspiration.  Assess for fatigue during feeding (e.g., desaturation or apnea).  In such cases, test weighing with a sensitive infant scale (accuracy ±2 g) is strongly recommended. |
| **Flesch–Kincaid Grade Level** | 8.29 | 8.20 | 10.36 |
| **Corresponding English Reading-Difficulty Category** | Grades 8–9: plain English | Grades 8–9: plain English | Grades 10–12: fairly difficult |

## Question 18

| Item | Attempt 1 | Attempt 2 | Attempt 3 |
| --- | --- | --- | --- |
| Clinical knowledge question | How should clinicians evaluate medication safety and breastfeeding-related risks when a breastfeeding woman requires medication during the puerperium? | | |
| Main guideline source(s) | NICE NG194 | | |
| Model response | Clinicians should evaluate medication safety by considering:  Drug characteristics – Molecular weight (<200 Da pass easily, >500 Da poorly transferred), protein binding (highly bound → less milk transfer), half-life, oral bioavailability in infant, volume of distribution.  Timing of medication relative to breastfeeding – advise breastfeeding just before the next dose if drug peak is 1–3 hours post-ingestion.  Infant factors – Prematurity, age (neonates <2 weeks are most vulnerable due to immature hepatic/renal clearance), and any existing illness (e.g., conjugated hyperbilirubinemia, G6PD deficiency).  Stepwise risk assessment:  Check if drug is absolutely contraindicated (e.g., antineoplastics, radiopharmaceuticals, ergotamine, lithium, amiodarone).  Use reliable references (LactMed, Hale’s Medications & Mothers’ Milk).  Choose drugs with lowest infant dose (RID <10% of mother’s weight-adjusted dose is generally safe).  Monitor infant for sedation, poor feeding, irritability, or rash.  Document the shared decision-making and planned monitoring. | Clinicians should evaluate medication safety using a risk-to-benefit framework:  Three key questions:  Is the medication necessary for maternal health?  Is there a safer alternative with equal efficacy?  Can the drug regimen be adjusted to reduce infant exposure?  Practical strategies:  Choose topical/intranasal/inhaled over systemic when possible (e.g., steroid inhaler for asthma > prednisone).  Use drugs with short half-life and no active metabolites.  Time feeds to avoid peak milk concentration:  Take drug immediately after feeding.  Delay next feeding for one half-life if safe for infant.  Relative Infant Dose (RID) – if <10% of weight-adjusted maternal dose, generally acceptable; RID >25% reconsider necessity.  Special considerations:  Pain management: ibuprofen and acetaminophen preferred. Codeine and tramadol contraindicated.  Antibiotics: penicillins, cephalosporins, macrolides (except azithromycin) are safe; tetracyclines (except doxycycline <3 weeks) avoid long-term; metronidazole causes bitter taste and theoretical mutagenicity – pump and discard for 12–24h.  Psychiatric meds: sertraline, paroxetine preferred; avoid lithium and valproate if possible.  Counsel mother on signs of adverse effects in infant (poor suck, excessive sleepiness, diarrhea, rash) and when to seek care. | Evaluation of medication safety in breastfeeding requires a pharmacokinetically-informed risk assessment, especially for vulnerable infants or long-term treatments.  Step-by-step clinical approach:  Gather data – maternal age, renal/hepatic function, infant gestational age and postnatal age, infant weight, feeding frequency.  Calculate  Relative Infant Dose (RID) = [(Milk concentration × Daily milk intake) / (Maternal dose per kg)] × 100%.  If RID <10% – usually safe.  If RID >10% – consider alternative or therapeutic drug monitoring in infant.  Consider active metabolites (e.g., prodrugs like codeine convert to morphine; avoid).  Special drug classes requiring caution:  Anticoagulants – warfarin safe (protein-bound, low milk transfer); heparin/enoxaparin not absorbed orally → safe; DOACs (apixaban, rivaroxaban) avoid due to lack of data.  Antiepileptics – lamotrigine transfers significantly (RID up to 20%) → monitor infant for sedation and rash. Valproate safe but monitor infant liver function if prolonged use.  Immunosuppressants – prednisolone safe (<20 mg/day; >20 mg: consider 4h discard); cyclosporine and tacrolimus – measure infant trough levels.  Radiological contrast – iodinated contrast minimal excretion; withhold breastfeeding for 12–24h after radioactive iodine.  When in doubt:  Consult clinical pharmacist and LactMed.  Consider pumped and discarded milk for 1–2 days if drug is essential but with high RID.  Perform infant serum drug level if chronic high-risk drug required. |
| **Flesch–Kincaid Grade Level** | 12.96 | 12.77 | 12.55 |
| **Corresponding English Reading-Difficulty Category** | College: difficult | College: difficult | College: difficult |

## Question 19

| Item | Attempt 1 | Attempt 2 | Attempt 3 |
| --- | --- | --- | --- |
| Clinical knowledge question | What conditions may be suggested by postpartum urinary retention, dysuria, urinary frequency, or urinary incontinence, and how should initial management and referral decisions be made? | | |
| Main guideline source(s) | NICE NG194 | | |
| Model response | Postpartum symptoms suggest specific conditions:  Urinary retention – may indicate detrusor areflexia (from prolonged labor, epidural anesthesia, or vaginal trauma), pelvic hematoma compressing the urethra, or urethral edema.  Dysuria – often from birth trauma (lacerations, sutures near urethra), urinary tract infection (UTI), or chemical irritation (antiseptics, catheters).  Urinary frequency – common transiently due to diuresis, but if persistent, consider UTI or bladder irritability from retained clots.  Urinary incontinence – stress incontinence suggests pelvic floor weakness or sphincter injury; urge incontinence suggests detrusor overactivity.  Initial management:  Retention: bladder scan post-void; if residual >150 mL, perform straight catheterization or indwelling catheter × 24–48h.  Dysuria/frequency: urinalysis and culture; treat UTI with nitrofurantoin or cephalexin (safe in breastfeeding).  Incontinence: pelvic floor exercises (Kegels) and bladder training.  Referral decision:  Urology referral if retention persists >3 days post-catheter removal, recurrent UTIs, or incontinence not improving after 6 weeks of physiotherapy.  Urgent referral for suspected pelvic hematoma with retention and pain – needs imaging (ultrasound) and possible drainage. | Postpartum urinary symptoms may indicate:  Overt urinary retention (post-void residual >150 mL or inability to void 6 hours after delivery) – associated with pudendal nerve block, epidural, vacuum/forceps delivery, or perineal hematoma.  Dysuria without infection – consider urethral trauma or retained vaginal packing.  Frequency and urgency – overactive bladder post-delivery, but if with fever, suspect pyelonephritis.  Incontinence – immediate postpartum incontinence often resolves, but persistent >3 months suggests occult sphincter defect or severe pelvic floor injury.  Initial management (by condition):  Retention – intermittent catheterization q4-6h until residual <100 mL; if failed, continuous catheter for 48h.  Dysuria/frequency – treat empiric UTI; if negative culture and persistent, consider interstitial cystitis (rare postpartum) – refer after 2 weeks.  Incontinence – supervised pelvic floor muscle training (PFMT) with biofeedback if available.  Referral decisions:  Urodynamics indicated for incontinence that does not respond to PFMT by 6 months postpartum.  Urology/gynecology referral for recurrent retention, hematuria, or suspicion of urethral diverticulum or fistula.  Pelvic floor physiotherapist immediately for incontinence with prolapse symptoms.  Red flags requiring immediate referral:  Complete retention with suprapubic pain despite catheterization → bladder rupture (rare)  High-grade fever + retention → pyonephrosis or pelvic abscess. | Postpartum urinary symptoms can reveal significant pathology:  Condition associations:  Urinary retention – after severe perineal tear (3rd/4th degree) → occult urethral injury; also bilateral pudendal neuropathy.  Dysuria – persistent burning despite negative cultures → vulvar vestibulitis, lichen sclerosus, or urethral caruncle.  Frequency – gestational diabetes with polyuria; also iatrogenic from diuretics (for preeclampsia).  Incontinence – continuous leakage suggests vesicovaginal fistula (prolonged obstructed labor or unrecognized bladder injury during cesarean).  Initial management:  Retention – trial without catheter after 48h; if fails, perform cystoscopy to exclude clot retention or mucosal flap.  Dysuria/frequency – if no UTI, perform pelvic exam to assess for suture irritation or vaginal foreign body.  Incontinence – for suspected fistula: tampon test (dye test), then cystoscopy and CT urogram.  Referral decisions:  Urogynecology – for fistula (usually repair delayed 3–6 months), complex incontinence, or prolapse causing retention.  Neurology – if urinary symptoms accompanied by lower extremity numbness or altered anal tone (suspected cauda equina or conus medullaris injury from epidural hematoma).  Urgent surgical referral – complete retention with inability to pass a catheter (urethral disruption) – rare after vaginal delivery, possible after forceps rotation.  Pelvic floor rehabilitation – for all women with incontinence persisting beyond 6 weeks, but especially for those with forceps delivery or 3rd/4th degree tears. |
| **Flesch–Kincaid Grade Level** | 18.54 | 17.90 | 18.84 |
| **Corresponding English Reading-Difficulty Category** | College graduate: very difficult | College graduate: very difficult | College graduate: very difficult |

## Question 20

| Item | Attempt 1 | Attempt 2 | Attempt 3 |
| --- | --- | --- | --- |
| Clinical knowledge question | What are the common contributing factors for postpartum constipation and hemorrhoids, and what should clinical nursing care and health education include? | | |
| Main guideline source(s) | Chinese Maternal Health Management Specification; NICE NG194 | | |
| Model response | Common contributing factors:  Constipation – decreased abdominal muscle tone after delivery, perineal pain (leading to stool avoidance), dehydration, iron supplementation, progesterone effect causing slowed intestinal transit, and reduced physical activity.  Hemorrhoids – increased intra-abdominal pressure during pregnancy and labor, prolonged second stage of labor, constipation straining, and engorgement of pelvic vessels.  Clinical nursing care:  Early ambulation post-delivery.  Adequate hydration (2–3 L water daily).  Stool softeners (docusate sodium) for first 5–7 days, especially after perineal laceration or cesarean.  Warm sitz baths for hemorrhoids (2–3 times daily).  Topical hemorrhoid creams (e.g., witch hazel, lidocaine) for pain.  Health education:  Instruct to avoid straining; encourage toilet sitting with feet elevated on a small stool.  High-fiber diet (fruits, vegetables, whole grains).  Do not delay bowel movement when urge is felt.  Iron supplements: ask about constipation; consider switching to a different formulation (e.g., ferrous bisglycinate) or adding stool softener. | Contributing factors:  Constipation – fear of pain from episiotomy or hemorrhoids; opioid analgesics (post-cesarean) slow gut motility; postpartum hypothyroidism (rare but relevant).  Hemorrhoids – pre-existing varicose veins; prolonged pushing; postpartum fluid shifts worsening venous congestion.  Clinical nursing care:  Assess bowel movements daily – day 2 postpartum without bowel movement requires intervention.  Offer stool softeners before constipation develops (especially if on opioids).  For hemorrhoids: cold compresses first 24h, then warm sitz baths; manual reduction of prolapsed hemorrhoids if needed.  Osmotic laxatives (lactulose, polyethylene glycol) if stool softeners fail. Avoid stimulant laxatives (senna, bisacodyl) unless severe constipation.  Health education:  Pelvic floor relaxation techniques – deep breathing during defecation, avoid holding breath and bearing down.  Perineal support: apply gentle pressure with a clean pad over perineum or episiotomy site while having bowel movement.  Post-cesarean: use abdominal binder for comfort and gentle walking.  Teach signs of fecal impaction (overflow diarrhea, inability to pass stool, abdominal pain) – requires manual disimpaction in severe cases. | Contributing factors beyond the obvious:  Constipation – anal sphincter spasm due to pain (especially after 3rd/4th degree tear); subclinical hypothyroidism; postpartum depression (loss of appetite, inactivity); rectocele from pelvic floor injury.  Hemorrhoids – prolapsed internal hemorrhoids may mimic rectal prolapse; thrombosed external hemorrhoid presents as a painful perianal lump.  Clinical nursing care (advanced):  Digital rectal exam before prescribing laxatives if patient reports severe pain or inability to pass stool despite softeners – rule out fecal impaction or perianal abscess.  For thrombosed hemorrhoid within 72h: consider incision and clot evacuation (by clinician).  Prescribe docusate + senna for opioid-induced constipation.  For recalcitrant constipation: add polyethylene glycol 17 g/day; if no response in 48h, check serum calcium, thyroid function, and consider abdominal X-ray.  Health education – specific scenarios:  After 3rd/4th degree laceration: avoid any straining; prescribe psyllium + stool softeners routinely; advise against enemas or suppositories without physician approval.  Hemorrhoids with heavy bleeding → not typical; evaluate for other causes (anal fissure, inflammatory bowel disease).  Long-term: prevent recurrence by maintaining high fluid/fiber intake for at least 3 months postpartum; encourage pelvic floor physiotherapy for any associated prolapse symptoms.  When to refer:  Constipation persisting >2 weeks despite optimal management → gastroenterology.  Large, painful, recurrent thrombosed hemorrhoids → colorectal surgery.  Inability to defecate without manual splinting (vaginal or perineal) → pelvic floor assessment for rectocele. |
| **Flesch–Kincaid Grade Level** | 12.28 | 15.00 | 16.86 |
| **Corresponding English Reading-Difficulty Category** | College: difficult | College: difficult | College graduate: very difficult |

## Question 21

| Item | Attempt 1 | Attempt 2 | Attempt 3 |
| --- | --- | --- | --- |
| Clinical knowledge question | What symptoms may indicate postpartum pelvic floor dysfunction, and when should pelvic floor rehabilitation assessment or referral be recommended? | | |
| Main guideline source(s) | NICE NG194 | | |
| Model response | Symptoms indicating possible postpartum pelvic floor dysfunction include:  Urinary – stress incontinence (leakage with cough, laugh, exercise), urgency, frequency, or hesitancy.  Anal – fecal incontinence (solid or liquid), urgency, or inability to control flatus.  Pelvic organ prolapse symptoms – sensation of a vaginal bulge or “something coming down,” pelvic heaviness, or needing to splint to defecate.  Sexual – dyspareunia, reduced sensation, or obstructive symptoms during intercourse.  Perineal pain – persistent or deep pelvic pain.  When to recommend pelvic floor rehabilitation assessment:  Early (4–6 weeks postpartum) – for any woman with persistent incontinence, prolapse symptoms, or significant perineal trauma (3rd/4th degree tear, forceps delivery).  Urgent referral – complete fecal incontinence, large symptomatic prolapse beyond hymen, or postpartum urinary retention.  Routine consideration – all women after vaginal delivery, especially multiparous or after instrumental birth, can benefit from pelvic floor assessment at 6–8 weeks postpartum.  Referral: to pelvic floor physiotherapist or urogynecology. | Less obvious symptoms of pelvic floor dysfunction:  Lower back or sacral pain with prolonged standing.  Voiding difficulties – slow stream, intermittent stream, sensation of incomplete emptying.  Pelvic floor overactivity – chronic pelvic pain, vaginismus, or pain with tampon use.  Inability to perform a pelvic floor contraction (on palpation or biofeedback).  Obstructed defecation symptoms – excessive straining, need for digital evacuation, feeling of incomplete evacuation.  When to recommend pelvic floor rehabilitation assessment:  By 6–8 weeks postpartum for all women with:  Third or fourth degree perineal tear.  Prolonged second stage of labor (>3 hours).  Newborn birth weight >4000 g.  Operative vaginal delivery (forceps or vacuum).  Pre-existing pelvic floor symptoms during pregnancy.  At 3 months postpartum if mild symptoms persist (occasional stress incontinence, mild prolapse sensation).  Immediate referral if symptoms affect daily activities or quality of life (e.g., cannot exercise due to leakage, avoids social situations due to urgency).  Referral pathway: primary care → pelvic floor physiotherapist ± urogynecology for persistent prolapse or incontinence after 6 months of conservative therapy. | Red flag symptoms suggesting significant pelvic floor injury:  Early postpartum (first week) – inability to void, complete fecal incontinence, or visible vaginal tear extending into anal sphincter.  Persistent perineal wound breakdown with fecal soiling → possible unrecognized fourth degree tear or rectovaginal fistula.  New onset bowel urgency after previous normal control → suggests external anal sphincter defect.  Severe pelvic pain unresponsive to analgesics → consider levator ani avulsion or hematoma.  When to recommend pelvic floor rehabilitation assessment:  Within 4–6 weeks for women with obstetric anal sphincter injury (OASIS) – all require specialized postpartum follow-up and endoanal ultrasound at 3–6 months if symptoms persist.  At 6–8 weeks for women with prolapse stage 2 or higher on POP-Q exam.  At 3 months for stress incontinence that has not resolved spontaneously.  Referral to specialist (urogynecology, colorectal surgery) if:  No improvement after 3–6 months of supervised pelvic floor muscle training.  Symptomatic prolapse past the hymen.  Fecal incontinence requiring pad use.  Urinary retention with post-void residual >150 mL.  Imaging referral – pelvic floor ultrasound or MRI for suspected levator ani avulsion, occult prolapse, or sphincter defect. |
| **Flesch–Kincaid Grade Level** | 16.63 | 12.30 | 13.93 |
| **Corresponding English Reading-Difficulty Category** | College graduate: very difficult | College: difficult | College: difficult |

## Question 22

| Item | Attempt 1 | Attempt 2 | Attempt 3 |
| --- | --- | --- | --- |
| Clinical knowledge question | Which factors should guide decisions about when to resume physical activity and pelvic floor muscle training during the puerperium? | | |
| Main guideline source(s) | NICE NG194; WHO 2022 | | |
| Model response | Decisions should be guided by:  Type of delivery –  Uncomplicated vaginal delivery: light activity (walking) as tolerated immediately; pelvic floor muscle training (PFMT) can begin day 1–3 if comfortable.  Cesarean section: avoid heavy lifting (>5–10 kg) and core-intensive exercises for 6–8 weeks; walking encouraged from day 1; PFMT can begin in first week if no pain.  Perineal wound healing –  Episiotomy or 2nd degree tear: start gentle PFMT after pain subsides (usually 1 week).  3rd/4th degree tear: wait for formal pelvic floor assessment (usually 6–8 weeks) before structured PFMT.  Bleeding – avoid strenuous activity if heavy lochia increases with activity.  Pain – no exercise causing perineal or incisional pain.  General timeline:  Week 1–2: walking, gentle PFMT (low intensity).  Week 3–6: increase PFMT repetitions, add core activation.  Week 6–8: resume moderate aerobic exercise (swimming, stationary cycling) if lochia has stopped and no pain.  Week 8–12: high-impact activity (running, jumping) if pelvic floor symptoms are absent. | Key guiding factors:  Pelvic floor symptoms –  Do NOT resume high-impact activity if stress incontinence, pelvic heaviness, or prolapse sensation is present.  PFMT should be supervised if unable to contract correctly (visible belly bulge instead of pelvic floor lift).  Diastasis recti abdominis –  Assess at 6 weeks by palpation. If gap >2 cm with fascial laxity, avoid crunches, planks, and twisting exercises; refer to physiotherapist.  Lochia pattern –  Resuming activity should NOT increase bleeding or turn lochia red again. If so, reduce intensity.  Fatigue and sleep deprivation –  Exercise should not worsen exhaustion; prioritize rest and gentle movement initially.  Practical guidance:  Start PFMT only when able to lie supine without pain and contract without breath-holding.  Use the “talk test” for aerobic activity – should be able to speak in short sentences.  Avoid intra-abdominal pressure (straining, heavy lifting) until at least 6 weeks postpartum to reduce prolapse risk.  Referral indications – if incontinence or prolapse symptoms appear with early activity, stop and refer to pelvic floor physiotherapist before progressing. | Factors guiding return to activity:  Prepartum fitness level – Women who exercised throughout pregnancy can often resume earlier (but not sooner than 2 weeks for high-impact). Sedentary women should start with walking and PFMT only.  Peripartum complications –  Severe hemorrhage, manual placental removal, or uterine atony → delay strenuous activity for 8–10 weeks.  Symphyseal pubis diastasis or pelvic fracture (rare) → physiotherapy-led rehabilitation.  Postpartum preeclampsia → avoid exercise until blood pressure controlled (usually 6–8 weeks).  Objective PFMT readiness –  Ability to contract pelvic floor muscles without Valsalva (verified by palpation or perineometer).  No sign of levator ani muscle avulsion (suspected if inability to lift or asymmetric contraction).  Biomarkers of healing –  Complete cessation of lochia (usually 4–6 weeks) suggests endometrial healing; earlier high-impact activity may increase bleeding risk.  Structured decision-making:  Weeks 0–2: only rest, walking, and PFMT in supine or side-lying position.  Weeks 3–6: add standing PFMT and very light resistance (Theraband) if no incontinence.  Week 6–8: perform a pelvic floor symptom questionnaire (e.g., PFDI-20) and a prolapse assessment before clearing high-impact sports.  Consider postpartum return-to-running testing (e.g., single-leg squat, jump test without leakage) at ≥12 weeks for elite athletes.  When to delay return to exercise:  Persistent pelvic girdle pain.  Urinary incontinence with cough or jogging.  PFMT contraction strength <3/10 on Oxford scale.  Any feeling of pelvic floor “heaviness” during or after activity. |
| **Flesch–Kincaid Grade Level** | 11.65 | 12.07 | 11.09 |
| **Corresponding English Reading-Difficulty Category** | Grades 10–12: fairly difficult | College: difficult | Grades 10–12: fairly difficult |

## Question 23

| Item | Attempt 1 | Attempt 2 | Attempt 3 |
| --- | --- | --- | --- |
| Clinical knowledge question | Why is the puerperium a high-risk period for venous thromboembolism, and which symptoms should raise concern for deep vein thrombosis or pulmonary embolism? | | |
| Main guideline source(s) | NICE NG194; RCOG Green-top Guideline No. 37a | | |
| Model response | The puerperium is a high-risk period for venous thromboembolism (VTE) due to:  Hypercoagulable state – pregnancy-induced elevation of clotting factors (I, VII, VIII, X, fibrinogen) and decreased natural anticoagulants (protein S). This persists for 6–8 weeks postpartum.  Venous stasis – from uterine involution compressing iliac veins, reduced mobility, and increased lower extremity venous capacitance.  Vascular damage – from delivery (especially operative vaginal or cesarean section).  Symptoms raising concern for DVT:  Unilateral leg swelling, pain, warmth, erythema (especially calf or thigh).  Tenderness along deep veins (palpable cord).  Homan’s sign (dorsiflexion pain) – low sensitivity/specificity, but persistent pain warrants investigation.  Symptoms of PE:  Sudden dyspnea, tachypnea, pleuritic chest pain.  Hemoptysis, tachycardia, syncope, or unexplained hypoxia.  Action: Any suspicion requires immediate objective testing (compression ultrasound for DVT; CTPA or V/Q scan for PE, with appropriate radiation precautions for breastfeeding). | Elevated VTE risk in puerperium stems from:  Pregnancy-related changes persist postpartum; risk highest first week after delivery, remains elevated up to 12 weeks.  Additional postpartum risk factors – cesarean delivery (especially emergency or repeat), postpartum hemorrhage, infection, prolonged bed rest, obesity, age >35, and preeclampsia.  Prior VTE or thrombophilia – further increases risk.  Symptoms raising concern for DVT (including atypical):  Asymmetric leg swelling (>2 cm difference in calf circumference).  Entire leg swelling (iliofemoral DVT – phlegmasia cerulea dolens: painful blue leg).  Isolated thigh pain without calf swelling (femoral vein thrombosis).  Symptoms of PE (including subtle signs):  Unexplained anxiety or sense of impending doom.  Low-grade fever without infection source.  Syncope (massive PE) or sustained hypotension.  Red flags: Postpartum tachypnea (respiratory rate >20) + any leg symptom → immediate workup.  Special consideration: After cesarean section, DVT may present with isolated lower abdominal or flank pain (iliac vein thrombosis). | Why puerperium is high-risk:  Relative risk of VTE is 5–10x higher than in non-pregnant state, highest in first 3 weeks postpartum.  Inflammatory response after delivery (especially placental separation) triggers coagulation cascade.  Immobilization – post-cesarean pain, regional anesthesia, or prolonged labor reduces venous return.  Symptoms that should raise high concern (including warning signs often missed):  DVT:  Acute-onset unilateral lower extremity pain with Homan’s sign (though unreliable) → still warrants imaging.  Superficial thrombophlebitis extending into deep system via perforator veins.  Asymmetrical leg warmth on palpation.  PE:  Isolated pleuritic pain without dyspnea (small peripheral PE).  Postpartum syncope or seizure-like activity – consider massive PE.  Unexplained persistent cough or hemoptysis.  High-risk groups requiring heightened surveillance:  Women with thrombophilia (Factor V Leiden, Prothrombin mutation, Protein C/S deficiency).  History of superficial or previous VTE.  Obesity (BMI >30) especially with additional risk factors.  Emergency cesarean or postpartum hemorrhage >1L with transfusion.  Clinical decision rule: Use Wells score for DVT/PE in postpartum women (modified for pregnancy).  Any Wells score ≥2 in puerperium → perform compression ultrasound (DVT) or CTPA (PE).  Do not rely on D-dimer (normally elevated postpartum). |
| **Flesch–Kincaid Grade Level** | 14.59 | 12.91 | 12.82 |
| **Corresponding English Reading-Difficulty Category** | College: difficult | College: difficult | College: difficult |

## Question 24

| Item | Attempt 1 | Attempt 2 | Attempt 3 |
| --- | --- | --- | --- |
| Clinical knowledge question | For women with a history of gestational hypertension or pre-eclampsia, what should be monitored postpartum, and which symptoms require urgent management? | | |
| Main guideline source(s) | NICE NG194; ACOG Optimizing Postpartum Care | | |
| Model response | Postpartum monitoring for women with prior gestational hypertension or pre-eclampsia should include:  Blood pressure (BP) – twice daily for first 7 days, then at least weekly until normal (target <140/90 mmHg).  Urine dipstick – for proteinuria weekly if previously elevated.  Weight and edema – daily self-monitoring for sudden weight gain (>1 kg/week).  Symptoms of pre-eclampsia progression – headache, visual disturbances, epigastric pain, nausea/vomiting.  Medication review – continue antihypertensives as prescribed (labetalol, nifedipine, methyldopa are breastfeeding-safe).  Symptoms requiring urgent management (immediate medical evaluation):  Severe headache unrelieved by acetaminophen.  Blurred vision, flashing lights, or scotoma.  Right upper quadrant or epigastric pain (suggesting HELLP syndrome).  Shortness of breath (pulmonary edema).  New-onset confusion or altered mental status.  BP ≥160/110 mmHg on repeated measurement.  Action: If any of these occur, patient should go to emergency department for BP control, labs (CBC, LFTs, creatinine, uric acid), and possible magnesium sulfate if severe features. | Postpartum monitoring beyond BP:  Home BP log – measure 2 hours postpartum, then daily for 2 weeks, then at 6-week visit.  Labs at 48–72 hours postpartum – platelets, AST, ALT, creatinine (especially if pre-eclampsia with severe features at delivery).  Infant monitoring – if mother had severe pre-eclampsia, assess for neonatal thrombocytopenia (rare but can occur).  Long-term renal function – check BP and urinalysis at 6–12 weeks, then annually (increased risk of chronic hypertension and CKD).  Symptoms requiring urgent management (including atypical presentations):  Persistent nausea/vomiting – often mistaken for post-cesarean ileus, but can signal HELLP recurrence.  Sudden visual loss or diplopia – cortical blindness or retinal detachment (rare).  Seizure – eclampsia can occur up to 6 weeks postpartum.  Oliguria (<400 mL/24h) – acute kidney injury.  Severe anxiety or agitation – sometimes prodromal eclampsia symptom.  Red flags that are often missed:  Epigastric pain radiating to back (HELLP vs. pancreatitis vs. cholecystitis).  Dyspnea with minimal exertion (pulmonary edema from fluid shifts).  Persistent severe headache despite antihypertensives → needs neuroimaging to rule out RCVS (reversible cerebral vasoconstriction syndrome) or posterior reversible encephalopathy syndrome (PRES).  Management threshold: Any BP ≥150/100 mmHg with symptoms → urgent evaluation; asymptomatic BP ≥160/110 mmHg → oral or IV antihypertensive within 60 minutes. | Postpartum monitoring should be stratified by severity:  For women with gestational hypertension or mild pre-eclampsia (no severe features):  Self-monitor BP at least twice daily × 14 days.  Check proteinuria once at 6 weeks (if still positive, refer nephrology).  Routine follow-up at 2 weeks and 6 weeks postpartum.  For women with pre-eclampsia with severe features or HELLP:  Monitor BP 4×/day × 7 days, then twice daily × 7 days.  Repeat labs (CBC, LFTs, creatinine) at 48h and 1 week postpartum.  Consider daily telephone check for first 7–10 days.  Prolong antihypertensives until BP <140/90 for at least 48h off medication (may need 6–8 weeks).  Symptoms requiring urgent management (including delayed postpartum pre-eclampsia):  New-onset or worsening symptoms beyond 48 hours postpartum – especially days 3–14 (peak incidence of delayed postpartum pre-eclampsia).  Rapidly escalating BP – increase of 30 mmHg systolic or 15 mmHg diastolic over 4 hours.  Thrombocytopenia symptoms – petechiae, bleeding gums, large bruising.  Upper abdominal pain with elevated LFTs (>2× normal) → treat as HELLP; urgent delivery not needed (already postpartum), but needs magnesium and BP control.  Who needs inpatient management:  BP ≥160/110 despite oral medication.  Any severe symptom (headache, visual changes, epigastric pain).  Lab evidence of HELLP (platelets <100,000, AST/ALT >70).  Eclamptic seizure.  Discharge planning: Prescribe a BP monitor, provide a symptom diary, and ensure access to 24/7 phone triage. Women with delayed pre-eclampsia may present 2–4 weeks postpartum with no prior warning, so educate partners/family as well.  Long-term counseling: Lifetime risk of cardiovascular disease is increased – recommend annual BP check, weight management, and lifestyle modification. |
| **Flesch–Kincaid Grade Level** | 12.68 | 13.26 | 11.07 |
| **Corresponding English Reading-Difficulty Category** | College: difficult | College: difficult | Grades 10–12: fairly difficult |

## Question 25

| Item | Attempt 1 | Attempt 2 | Attempt 3 |
| --- | --- | --- | --- |
| Clinical knowledge question | For women with a history of gestational diabetes mellitus, what should postpartum glucose follow-up and long-term metabolic risk management include? | | |
| Main guideline source(s) | NICE NG194; ACOG Optimizing Postpartum Care | | |
| Model response | Postpartum glucose follow-up for women with gestational diabetes mellitus (GDM) should include:  Early testing – Fasting plasma glucose (FPG) 6–12 weeks postpartum.  Alternative – 75g 2-hour oral glucose tolerance test (OGTT) using non-pregnant diagnostic criteria (fasting ≥7.0 mmol/L [126 mg/dL] or 2h ≥11.1 mmol/L [200 mg/dL] for diabetes; impaired fasting glucose or impaired glucose tolerance for prediabetes).  If normal – repeat testing every 1–3 years (sooner if symptoms or weight gain).  Long-term metabolic risk management:  Lifestyle – achieve pre-pregnancy weight (or BMI <23 in Asian populations; <25 in Caucasians).  Diet – low glycemic index, high fiber, limit sugary beverages.  Exercise – 150 minutes moderate-intensity weekly (e.g., brisk walking).  Annual screening – HbA1c or fasting glucose once stable.  Contraception – plan future pregnancies with glucose optimization before conception.  Refer to primary care if FPG 5.6–6.9 mmol/L (100–125 mg/dL) for diabetes prevention program; treat if frank diabetes. | Postpartum follow-up should be stratified:  Low risk (GDM controlled by diet, normal pre-pregnancy BMI, no family history) → FPG at 6–12 weeks, then every 3 years.  High risk (GDM requiring insulin, overweight/obese, non-white ethnicity, history of macrosomia or recurrent GDM) → OGTT at 6–12 weeks, then annually.  Long-term management includes:  Breastfeeding – promotes weight loss and improves glucose metabolism; women with GDM should be strongly encouraged to breastfeed exclusively for at least 6 months.  Metformin – consider for women with persistent postpartum impaired glucose tolerance or high progression risk, especially if overweight and unable to lose weight.  Annual HbA1c – target <5.7% normal; 5.7–6.4% prediabetes; ≥6.5% diabetes.  Lipid and blood pressure checks – annually (GDM increases future CVD risk).  Timeline:  6–12 weeks: OGTT or FPG.  1 year postpartum: repeat OGTT if initial was prediabetes; otherwise FPG.  Every 1–3 years thereafter.  Interventions: Diabetes prevention programs (e.g., lifestyle change, metformin if BMI >35) reduce progression by 50%. Refer women with prediabetes to structured lifestyle intervention. | Postpartum glucose follow-up often missed – systematic strategies required:  Routine order – place OGTT lab slip at discharge or at 6-week visit.  Remote monitoring – use HbA1c obtained with routine postpartum labs (CBC, lipids) to improve adherence.  Consider early testing if persistent glycosuria or polyuria.  Long-term metabolic risk management should include:  Interconception care – between pregnancies, achieve HbA1c <6.5% (preferably <5.7%) before next conception.  Annual assessment of –  FPG or HbA1c  Blood pressure  Lipid panel (LDL, HDL, triglycerides)  BMI trend  Pharmacologic prevention – metformin 850 mg twice daily reduces diabetes risk in prediabetic women with prior GDM and BMI >35 or age <45.  Bariatric surgery – may be considered for women with BMI >40 and multiple risk factors after childbearing is complete.  Special populations:  Women with prior GDM and postpartum diabetes → treat as type 2 diabetes (metformin first-line if breastfeeding).  Women with β-cell autoantibodies (some GDM cases) → monitor more frequently for progression to autoimmune diabetes/LADA.  Documentation and handoff:  Clearly document results and plan in discharge summary.  Notify primary care provider even if postpartum OGTT is normal (high risk for diabetes in 5–10 years).  Emerging tools: Continuous glucose monitor (CGM) can be used briefly postpartum for women with persistent hypoglycemia or weight loss resistance.  Patient education: “GDM is a window to future health” – emphasize 50% risk of developing type 2 diabetes within 5–10 years; up to 70% if overweight. Prevention is possible with 5–7% weight loss and 150 min exercise/week. |
| **Flesch–Kincaid Grade Level** | 8.83 | 9.07 | 11.69 |
| **Corresponding English Reading-Difficulty Category** | Grades 8–9: plain English | Grades 10–12: fairly difficult | Grades 10–12: fairly difficult |

## Question 26

| Item | Attempt 1 | Attempt 2 | Attempt 3 |
| --- | --- | --- | --- |
| Clinical knowledge question | How can postpartum blues, postpartum depression, and severe psychiatric symptoms be distinguished, and what should be specifically assessed during clinical evaluation? | | |
| Main guideline source(s) | NICE CG192; NICE NG194; Chinese Maternal Health Management Specification | | |
| Model response | Postpartum blues, postpartum depression (PPD), and severe psychiatric symptoms (postpartum psychosis) are distinguished primarily by onset, duration, and symptom severity.  Postpartum blues begins within the first few days after delivery (typically days 2–5), lasts less than two weeks, and is characterized by mood lability, tearfulness, anxiety, and mild insomnia. The mother’s overall function remains normal, and there are no suicidal or psychotic thoughts.  Postpartum depression usually begins within the first four weeks (but can occur up to 12 months postpartum), lasts more than two weeks, and includes depressed mood, anhedonia (loss of interest), excessive guilt, fatigue, changes in sleep and appetite, and possible suicidal ideation. There are no hallucinations or delusions.  Severe psychiatric symptoms (postpartum psychosis) has a rapid onset within the first 1–4 weeks, with severe and rapidly progressing symptoms including hallucinations (often auditory and commanding), delusions (frequently involving the infant), disorganized behavior, mania, and a high risk of infanticide or suicide.  Clinical evaluation should specifically include:  Use of the Edinburgh Postnatal Depression Scale (EPDS) – a score of 10 or higher suggests possible PPD; 13 or higher indicates probable PPD.  Direct questioning about mood, energy, guilt, sleep pattern (especially "cannot sleep even when the baby sleeps"), and suicidal or homicidal thoughts.  A focused mental status examination, including thought content (delusions about the baby) and perception (hallucinations).  Risk assessment: any plan or intent to harm self or the infant requires immediate intervention.  Immediate action: Suspected psychosis or active suicidality requires emergency psychiatric referral. PPD without psychosis can be managed with psychotherapy and/or antidepressants, with close follow-up. Blues requires support and monitoring only. | The distinction between these conditions hinges on specific red flags and the presence of psychotic features.  Postpartum blues is common (50–80% of women), peaks on day 5, and resolves spontaneously by day 10–14. There is no impairment in daily function, and the mother retains full insight.  Postpartum depression presents with persistent low mood, tearfulness, loss of enjoyment, and often physical symptoms such as changes in appetite or sleep. Mothers may have thoughts of harming themselves but are distressed by these thoughts and do not act on them without severe illness. Importantly, obsessive thoughts about infant harm (e.g., fear of dropping the baby) are common in PPD and are ego-dystonic (the mother finds them upsetting and tries to resist them).  Postpartum psychosis is rare (1–2 per 1000 deliveries) but a psychiatric emergency. Hallucinations are typically auditory (e.g., voices commanding the mother to harm the baby). Delusions are often bizarre and infant-related (e.g., the baby is the devil, has died, or is being replaced). The mother may appear confused, disorganized, or manic, and she may have no insight into her illness.  Clinical evaluation should specifically include:  A detailed sleep assessment: in psychosis, the mother may feel "no need for sleep" despite severe sleep deprivation.  Direct questioning: "Have you ever felt that your baby is evil or possessed?" "Do you hear voices that others do not hear?" "Do you ever feel that your baby is not really yours?"  Distinguishing ego-dystonic obsessions (PPD) from ego-syntonic delusions (psychosis) – the former causes distress and resistance, the latter is believed as true.  Use of the Mood Disorder Questionnaire (MDQ) to screen for mania or hypomania, which may point to bipolar disorder.  Urgent psychiatric consultation is mandatory if the patient is paranoid, disorganized, or responding to internal stimuli. Hospitalization is required for any woman with psychosis or imminent risk of harm. | A critical but often overlooked aspect is that many women with postpartum psychosis have previously undiagnosed bipolar I disorder. Therefore, the assessment must screen for a history of hypomanic or manic episodes before pregnancy.  Differentiating clues:  Postpartum blues – Self-limited, no functional impairment. Reassure the mother and schedule routine follow-up.  Postpartum depression – Persistent sadness and loss of interest, but reality testing remains intact. Anxiety is the dominant feature in up to 50% of cases.  Postpartum psychosis – Key warning signs: acute confusion, rapid mood swings, bizarre or disorganized behavior, disorientation, and delusions about the infant. The risk of infanticide is approximately 4%. Severe insomnia that does not respond to fatigue is often a prodromal symptom.  Clinical evaluation must specifically include the following five components:  Past psychiatric and family history – Ask about prior episodes of PPD, bipolar disorder, mania, psychosis, or a family history of postpartum psychosis in a first-degree relative.  Sleep pattern assessment – Severe, persistent insomnia -infant interaction observation – During the visit, observe how the mother holds, looks at, and speaks to the infant. Avoidance, hostility, indifference, or a complete lack of engagement are concerning signs.  Direct safety questions – Ask gently but explicitly: "Have you had any thoughts of hurting yourself or your baby?" "Do you ever hear voices telling you to do things?" "Do you believe something strange has happened to your baby?"  Safety planning and referral thresholds:  Blues → Watchful waiting, social support, follow-up EPDS in 2 weeks.  PPD (no psychosis) → First-line: sertraline (breastfeeding-safe) + psychotherapy. Follow up in 1–2 weeks. Remove lethal means from the home.  PPD with active suicidal ideation or obsessive infant harm thoughts → Same-day psychiatric evaluation.  Postpartum psychosis or clear homicidal/suicidal plan → Immediate emergency department transfer, psychiatric admission, antipsychotics (e.g., olanzapine), and if bipolar, mood stabilizers. Also, notify child protective services as per local legal requirements. The infant should not be left alone with the mother until she is stabilized. |
| **Flesch–Kincaid Grade Level** | 15.40 | 9.10 | 11.97 |
| **Corresponding English Reading-Difficulty Category** | College: difficult | Grades 10–12: fairly difficult | Grades 10–12: fairly difficult |

## Question 27

| Item | Attempt 1 | Attempt 2 | Attempt 3 |
| --- | --- | --- | --- |
| Clinical knowledge question | How should self-harm, suicidal ideation, or thoughts of harming the baby during the puerperium be risk-assessed and managed urgently? | | |
| Main guideline source(s) | NICE CG192 | | |
| Model response | When a puerperal woman reports self-harm, suicidal ideation, or thoughts of harming the baby, the first step is immediate risk stratification using a clear protocol.  Risk assessment must determine:  Plan and intent – Does she have a specific method, time, or place? Has she taken any steps (e.g., collecting pills, writing a note, buying a weapon)?  Protective factors – Does she have someone who can stay with her and the baby at all times? Does she want help?  Impulsivity and substance use – Alcohol, benzodiazepines, or stimulants increase disinhibition.  Access to means – Are there firearms, medications, ropes, or sharp objects in the home?  Acute management (within minutes to hours):  Immediate safety – Do not leave the woman alone. Remove any visible dangerous objects from the room.  Suicidal ideation with plan + intent → Emergency medical services (call emergency number) and transfer to emergency department. Do not allow her to drive herself.  Thoughts of harming the baby → The same urgency applies. Additionally, the baby must not remain in her sole care until psychiatric evaluation is complete. A partner, family member, or social worker must take temporary custody.  No plan but passive thoughts (“sometimes I wish I wouldn’t wake up”) → Same-day urgent psychiatric assessment, preferably in a hospital setting. The baby should be accompanied by another responsible adult.  Documentation: Record verbatim quotes, risk level, actions taken, and handover to psychiatry. | Not all thoughts of harming the baby carry the same risk. A critical clinical distinction is between obsessive, ego-dystonic thoughts (common in postpartum depression) and delusional, ego-syntonic thoughts (seen in postpartum psychosis).  Ego-dystonic thoughts (postpartum depression) – The mother is horrified by the thoughts, tries to suppress them, has no plan, and has good insight. She is distressed and seeks help. Risk of acting on these thoughts is very low, but it is not zero, especially if severe depression coexists.  Ego-syntonic thoughts (postpartum psychosis) – The mother believes the thoughts are justified (e.g., “the baby is evil and must be killed”). She may have a plan and shows no distress. She may deny anything is wrong. This is a psychiatric emergency.  Risk assessment specific to puerperium:  Ask directly: “Do you feel you are being told to harm your baby?” (hallucinations)  Ask: “Do you believe your baby is not really yours, or that something is terribly wrong with the baby?” (delusions)  Assess command hallucinations: “Do you hear voices telling you to hurt yourself or the baby? Can you resist them?”  Urgent management and legal obligations:  Any homicidal ideation with plan → Immediate psychiatric admission. In most jurisdictions, this requires emergency detention if the patient refuses.  Child protection notification – In many countries, thoughts of harming the baby, especially with plan or psychosis, mandate reporting to child protective services (or equivalent). This is not a breach of confidentiality; it is a duty to protect a vulnerable infant.  Medications – In the emergency setting, antipsychotics (e.g., olanzapine 5–10 mg orally or intramuscularly) and benzodiazepines (e.g., lorazepam) can be given while awaiting psychiatric admission. Do not delay transfer to initiate oral medications.  Discharge is not an option for a woman with active homicidal or suicidal intent until she has been fully evaluated by psychiatry and a safe plan is in place for the infant. | Postpartum self-harm and infanticidal thoughts require a broader differential and consideration of unique puerperal risk factors.  Differential diagnoses to consider urgently:  Postpartum psychosis – Highest risk. May present with command hallucinations, disorganization, and bizarre delusions.  Postpartum depression with severe suicidal ideation – Risk increases with prior suicide attempts, lack of social support, and unplanned pregnancy.  Postpartum obsessive-compulsive disorder – Recurrent, intrusive, ego-dystonic thoughts of harming the baby without intent. Risk is low, but severe OCD can co-occur with depression and require inpatient care.  Bipolar disorder, mixed or manic episode – Agitation, impulsivity, and poor judgment can lead to sudden violent acts.  Postpartum post-traumatic stress disorder – Rarely associated with homicidal thoughts, but flashbacks and hyperarousal can lead to dissociation and unintentional harm.  Risk assessment protocol (in addition to standard suicide risk assessment):  Assess infant age – Risk of infanticide is highest in the first two weeks postpartum.  Assess recent stressors – Infant colic, breastfeeding difficulties, lack of sleep, lack of partner support, financial stress, or a recent traumatic birth.  Assess past history – Previous self-harm, previous infanticidal thoughts , history of child protective services involvement.  Assess current command hallucinations – Ask: “Do the voices tell you to hurt the baby? Have you ever felt you were losing control?”  Urgent management – environmental control:  Admission to a mother-baby psychiatric unit – This is the ideal setting, allowing supervised contact while treating the mother. If unavailable, admit to a general psychiatric unit and arrange for the baby to stay with a safe caregiver.  Involuntary hospitalization – If the woman refuses but has active homicidal or suicidal thoughts, use local mental health laws to detain her for emergency evaluation.  Weapons and means – Police or social services should remove firearms and dangerous objects from the home if the woman has already returned home.  Follow-up within 24 hours – If the patient is deemed low-risk after full psychiatric assessment (rare in this context), arrange a follow-up visit the next day with a perinatal psychiatrist and ensure the baby is safe. |
| **Flesch–Kincaid Grade Level** | 9.78 | 9.58 | 13.90 |
| **Corresponding English Reading-Difficulty Category** | Grades 10–12: fairly difficult | Grades 10–12: fairly difficult | College: difficult |

## Question 28

| Item | Attempt 1 | Attempt 2 | Attempt 3 |
| --- | --- | --- | --- |
| Clinical knowledge question | Which physiological, psychological, and wound-healing factors should be considered before resuming sexual activity after childbirth, and when is further assessment needed? | | |
| Main guideline source(s) | NICE NG194; Chinese Maternal Health Management Specification | | |
| Model response | Before resuming sexual activity after childbirth, three categories of factors must be considered: physiological, psychological, and wound-healing.  Physiological factors:  Lochia cessation – Resumption of intercourse should generally be delayed until lochia has stopped (typically 4–6 weeks). Bleeding indicates an open endometrial surface, which increases infection risk.  Hormonal changes – Lactation causes hypoestrogenism, leading to vaginal dryness and thinning of the vaginal epithelium. This can cause dyspareunia (pain with intercourse).  Perineal and pelvic floor recovery – Even without a visible tear, the pelvic floor muscles and connective tissue need time to regain tone.  Psychological factors:  Fatigue and sleep deprivation – Lack of restorative sleep significantly reduces libido and interest in intimacy.  Mood – Postpartum depression or anxiety directly reduces sexual desire and increases pain perception.  Body image – Concerns about weight, stretch marks, or changes in breast appearance may cause avoidance.  Fear of pain – Anticipatory fear, especially after a traumatic delivery or perineal laceration, can lead to vaginismus.  Wound-healing factors:  Episiotomy or perineal laceration – For first- and second-degree tears, healing of epithelial surfaces occurs by 4–6 weeks, but deeper tissue remodeling continues for months.  Third- or fourth-degree tears – Formal assessment at 6–8 weeks by a clinician before considering intercourse. These wounds require longer healing and may benefit from pelvic floor physiotherapy.  Cesarean incision – The skin incision heals within 2–3 weeks, but fascial and uterine incisions require 6–8 weeks. Heavy lifting and deep penetration should be avoided until cleared.  When is further assessment needed?  Pain with intercourse persisting beyond 6–8 weeks postpartum despite lubrication and gentle trial.  Inability to insert a finger or tampon due to pain (suspected vaginismus or severe scarring).  Persistent perineal wound breakdown or granulation tissue.  Heavy bleeding after intercourse.  Ongoing incontinence or prolapse symptoms.  In such cases, refer to a pelvic floor physiotherapist or a gynecologist for speculum and bimanual examination. | A more detailed approach considers the specific impact of breastfeeding and the distinction between "cleared for intercourse" vs. "ready for pleasurable intercourse."  Physiological factors specific to breastfeeding:  Vaginal dryness – Due to low estrogen. This is the most common cause of postpartum dyspareunia in lactating women. Water-based lubricants are strongly recommended.  Dyspareunia – Up to 60% of women report pain at first postpartum intercourse, but persistent pain beyond 3 months is abnormal.  Pelvic floor muscle overactivity – Hypertonicity (not weakness) can cause deep pain with penetration. This is often missed if only strength is assessed.  Psychological factors beyond mood:  Relationship dynamics – New parenting stress, unequal distribution of childcare, and lack of non-sexual physical intimacy can reduce desire.  Previous sexual trauma – May be re-triggered by pain or loss of control during delivery.  Breastfeeding-related sexual changes – Some women experience aversion or negative emotions with breast stimulation during intercourse (not universal, but clinically important if present).  Wound-healing specific factors:  Scar tissue (keloid or fibrotic) – Can cause focal, sharp pain at the episiotomy or tear site. Gentle massage with vitamin E or a topical estrogen cream (after 6 weeks) may help.  Granulation tissue – Overgrowth of healing tissue at the perineal wound site, often appearing as a small, red, bleeding nodule. This requires silver nitrate cauterization or surgical excision.  When is further assessment needed?  Pain that prevents any penetration at 12 weeks postpartum.  Persistent localized tenderness at the episiotomy scar.  Any leakage of stool or flatus during intercourse (possible unrecognized sphincter defect).  Feelings of a "bulge" during intercourse (possible prolapse).  Inability to achieve arousal or orgasm that causes significant distress.  Referral pathway: Pelvic floor physiotherapist for biofeedback and manual therapy, and a perinatal mental health professional for psychological barriers. | For women with complicated deliveries (forceps, vacuum, third/fourth degree tears, or postpartum hemorrhage), additional factors must be considered before advising resumption of sexual activity.  Physiological factors – complex cases:  Levator ani muscle injury – Occurs in up to 40% of vaginal deliveries, especially forceps. This can cause deep pelvic pain and obstructive defecation, which indirectly affects sexual function.  Pudendal nerve neuropathy – Causes perineal burning, numbness, or hypersensation. Sexual activity may be extremely painful or impossible.  Post-cesarean adhesions – Rarely, intra-abdominal adhesions cause deep dyspareunia (pain with deep thrusting) that is position-dependent.  Psychological factors – high-risk groups:  Postpartum post-traumatic stress disorder – After a traumatic delivery (e.g., hemorrhage, emergency cesarean, neonatal intensive care unit admission). Flashbacks triggered by pelvic exams or intercourse require trauma-informed care and referral to a psychologist.  Fear of another pregnancy – May cause avoidance of intercourse despite physical readiness.  Wound-healing factors – surgical considerations:  Dehiscence or infection – Healed but weakened tissue may tear again with early intercourse.  Episiotomy scar endometriosis – Very rare, but presents as cyclical, worsening pain with intercourse, often months after delivery.  Vesicovaginal or rectovaginal fistula – Extremely rare but should be suspected if intercourse is painful and accompanied by passage of urine or gas via the vagina. This requires surgical repair.  When is further assessment needed – objective criteria:  No attempt by 6 months postpartum (in a woman who desires sexual activity) – requires evaluation.  Repeated superficial or deep pain with intercourse despite adequate lubrication and relaxation.  Clinical findings on examination:  Speculum exam: visible scar tenderness, granulation tissue, prolapse (stage ≥2), or fistula.  Bimanual exam: levator ani tenderness, pelvic organ mobility, or adnexal masses.  Persistent perineal wound not healed by 8 weeks.  Objective assessment tools:  Female Sexual Function Index (FSFI) – Validated questionnaire to quantify sexual dysfunction.  Pelvic floor ultrasound – To assess for levator ani avulsion or occult prolapse.  Perineometry or vaginal manometry – To evaluate pelvic floor muscle strength and resting tone.  Recommendation: All women with third/fourth degree tears should have a dedicated postpartum visit at 6–8 weeks that includes a discussion of sexual function and a pelvic exam. Routine advice: wait until lochia has stopped, use lubricants, start with external stimulation and gradual penetration, and stop if pain persists beyond mild discomfort. Further assessment is needed if pain or fear continues beyond 12 weeks postpartum. |
| **Flesch–Kincaid Grade Level** | 13.11 | 12.46 | 14.05 |
| **Corresponding English Reading-Difficulty Category** | College: difficult | College: difficult | College: difficult |

## Question 29

| Item | Attempt 1 | Attempt 2 | Attempt 3 |
| --- | --- | --- | --- |
| Clinical knowledge question | Why is contraceptive counselling needed in the early postpartum period, and what factors should be considered when selecting contraception for breastfeeding and non-breastfeeding women? | | |
| Main guideline source(s) | FSRH Contraception After Pregnancy; NICE NG194; Chinese Maternal Health Management Specification | | |
| Model response | Contraceptive counselling is needed in the early postpartum period for two main reasons. First, ovulation can resume as early as 21 days after delivery in non-breastfeeding women, and by 6 weeks in some breastfeeding women, before the first menstrual period returns. This means a woman can become pregnant again without knowing she is fertile. Second, short interpregnancy intervals (less than 12–18 months) are associated with increased risks of preterm birth, low birth weight, and maternal complications. Therefore, early counselling helps women plan the timing of their next pregnancy.  For breastfeeding women, factors to consider when selecting contraception:  Lactational Amenorrhea Method (LAM) – Effective for the first 6 months only if three conditions are met: exclusive or nearly exclusive breastfeeding (no formula, no long gaps between feeds), no return of menses, and the infant is less than 6 months old. LAM has a failure rate of <2% when used correctly.  Progestin-only methods – Preferred for breastfeeding women because they do not reduce milk supply. Options include:  Progestin-only pills (mini-pill) – can be started immediately postpartum.  Etonogestrel implant (Nexplanon) – can be inserted immediately postpartum; safe and effective.  Depot medroxyprogesterone acetate (DMPA injection) – safe at any time postpartum, but may delay return to fertility.  Intrauterine devices (IUDs) – Both copper IUD (non-hormonal) and levonorgestrel IUD can be placed immediately postpartum or at the 6-week visit. They do not affect breastfeeding.  Combined hormonal contraceptives (CHCs – pills, patch, ring) – Avoid in the first 6 weeks postpartum in breastfeeding women because estrogen may reduce milk supply. After 6 weeks, CHCs can be used if no other risk factors exist.  For non-breastfeeding women:  CHCs can be started immediately postpartum (day 1–3) or after 21 days (to avoid theoretical thrombotic risk; current guidelines allow earlier in some cases).  All other methods (progestin-only, IUDs, implant, barrier methods, sterilization) are safe and can be offered immediately.  Additional factors for both groups: Medical comorbidities (e.g., hypertension, thrombophilia, liver disease), patient preference, access to follow-up, and cost. | Contraceptive counselling in the early postpartum period is critical because the risk of venous thromboembolism (VTE) remains elevated for up to 12 weeks after delivery. Selecting the wrong method – particularly combined hormonal contraceptives (CHCs) – can further increase this risk. Additionally, many women leave the hospital without a postpartum visit or with long delays, so counselling and method provision should occur before discharge.  Factors to consider for breastfeeding women (with WHO Medical Eligibility Criteria, MEC):  Lactational Amenorrhea Method – MEC category 1 (safe and effective) when criteria met.  Progestin-only methods (pill, implant, injection, IUD) – MEC category 1 (no restriction). They do not affect milk production, quality, or infant growth.  Copper IUD – MEC category 1; can be inserted within 48 hours of delivery or after 4 weeks (insertion between 48 hours and 4 weeks is associated with higher expulsion rates).  CHCs (estrogen-containing) – MEC category 3 (usually not recommended) from delivery to 6 weeks in breastfeeding women due to concerns about milk supply. After 6 weeks, MEC category 2 (benefits outweigh risks) if the woman is otherwise healthy.  Emergency contraception – Levonorgestrel and ulipristal acetate are safe in breastfeeding, but the copper IUD is the most effective.  Factors for non-breastfeeding women:  CHCs – MEC category 2 from day 1 to 21 postpartum (theoretical VTE risk but lower than pregnancy risk). MEC category 1 after 21 days.  Progestin-only methods – MEC category 1 at any time postpartum.  IUDs – MEC category 1. Immediate postpartum insertion is safe, though expulsion rates are slightly higher than interval insertion.  Female sterilization – Can be performed immediately after vaginal or cesarean delivery (postpartum sterilization) or at a later date. Counselling must emphasize permanence and allow time for informed consent without labor pain.  Special considerations for both groups:  Thrombophilia – CHCs are contraindicated (MEC category 4) in women with known thrombophilia (e.g., Factor V Leiden, protein C/S deficiency).  Postpartum pre-eclampsia or hypertension – Avoid CHCs until blood pressure is normal.  Depression – Progestin-only methods and IUDs are safe; DMPA injection has a small possible association with mood symptoms, but evidence is mixed.  HIV or high HIV risk – Progestin-only implants and IUDs are safe; DMPA has no restriction.  Practical note: Provide counselling during antenatal care and again in the early postpartum period. Document the chosen method and provide the method before discharge when possible (e.g., implant, IUD, mini-pill prescription). | Contraceptive counselling is needed early postpartum because the return to fertility is unpredictable, and many women do not attend a 6-week follow-up visit. Moreover, the early postpartum period is a "teachable moment" when women are highly motivated to avoid another pregnancy too soon. Unintended pregnancy rates are high in the first year postpartum, especially among adolescents and women with limited access to care.  For breastfeeding women – patient-centered factors beyond medical safety:  Desire for very long-acting contraception – The etonogestrel implant (3 years) and levonorgestrel IUD (5–8 years) are ideal. They can be inserted immediately postpartum (after placental delivery) or during the first 48 hours, with convenience and high satisfaction.  Concerns about hormonal effects – The copper IUD is hormone-free and does not affect milk supply. It can be left in place for up to 10 years.  Uncertainty about breastfeeding duration – Progestin-only pills require daily adherence and have a narrow window for missed doses; they are less effective if breastfeeding is not exclusive. Implant or IUD is a better choice for women who are inconsistent with daily pills.  Cultural or religious preferences – Some women may prefer fertility awareness-based methods, but these are difficult to use in the postpartum period due to irregular cycles. LAM is an acceptable option for the first 6 months if criteria are strictly followed.  For non-breastfeeding women:  All methods are available. Many women in this group prefer to resume CHCs (pills, patch, ring) because they regulate cycles and provide effective contraception. These can be started immediately postpartum or at 3–4 weeks.  If the woman has completed childbearing, discuss tubal ligation (postpartum or interval) or vasectomy for her partner.  For women who cannot or do not want to take daily pills, long-acting reversible contraceptives (LARC) – implant or IUD – have the highest continuation rates and lowest failure rates.  When is further assessment or referral needed before prescribing contraception?  Medical contraindications – Deep vein thrombosis history, current breast cancer, severe liver disease, or migraine with aura (CHCs contraindicated).  Postpartum complications – Endometritis or sepsis: delay IUD insertion until infection resolves (usually 2–4 weeks).  Undiagnosed abnormal uterine bleeding – Evaluate before inserting an IUD.  Difficulty with decision-making – Refer to a family planning specialist or use a decision aid.  Key counselling messages for both groups:  "You can ovulate before your first period returns. Do not rely on breastfeeding alone unless you meet all three LAM criteria."  "The safest time to get pregnant again is at least 12–18 months after giving birth, especially if you had a cesarean section."  "Long-acting reversible contraceptives are safe during breastfeeding, have very high effectiveness, and do not require daily action."  "If you change your mind, the implant or IUD can be removed at any time, and fertility returns quickly."  Final recommendation: Offer counselling during the third trimester, then again on the postpartum ward. Provide the chosen method (if immediate placement is appropriate) or a prescription before discharge. Document the discussion and follow-up plan in the medical record. |
| **Flesch–Kincaid Grade Level** | 11.92 | 12.18 | 12.27 |
| **Corresponding English Reading-Difficulty Category** | Grades 10–12: fairly difficult | College: difficult | College: difficult |

## Question 30

| Item | Attempt 1 | Attempt 2 | Attempt 3 |
| --- | --- | --- | --- |
| Clinical knowledge question | What core components should be included in postpartum maternal health education to ensure that women can recognize abnormal symptoms and seek timely help? | | |
| Main guideline source(s) | WHO 2022; NICE NG194; Chinese Maternal Health Management Specification | | |
| Model response | Postpartum maternal health education should include core components that empower women to recognize dangerous symptoms and seek help immediately. A practical approach is to teach a short list of "red flag" warnings using simple mnemonics.  Core components to include:  1. Hemorrhage warning signs (first 24 hours to 6 weeks)  Soaking more than one pad per hour for two consecutive hours.  Passing clots larger than an egg (or larger than a golf ball).  Bright red bleeding that returns after having tapered to brownish discharge.  Feeling faint, dizzy, or having a racing heart.  2. Infection warning signs  Fever over 38.0°C (100.4°F) after the first 24 hours postpartum.  Foul-smelling vaginal discharge.  Worsening abdominal or perineal pain not relieved by routine analgesics.  Redness, swelling, or oozing from a cesarean or perineal wound.  3. Thromboembolism warning signs (deep vein thrombosis and pulmonary embolism)  Unilateral leg swelling, pain, warmth, or redness (one leg only).  Sudden shortness of breath, chest pain, or coughing up blood.  4. Pre-eclampsia warning signs (up to 6 weeks postpartum)  Severe headache that does not improve with acetaminophen.  Visual changes (blurring, flashing lights, spots).  Severe pain under the ribs or in the upper abdomen.  Nausea or vomiting after the first few days.  5. Mental health emergencies  Thoughts of harming yourself or your baby.  Hearing voices or believing things that others do not believe.  Inability to sleep even when the baby sleeps (feeling "wired").  Mnemonic for patients: Use “R.E.D. – Bleeding, Pain, Fever” plus “H.E.A.D. – Headache, Eyes, Abdomen, Difficulty breathing” plus “M.O.M. – Mood changes, Overwhelming thoughts, Missing sleep.”  When to seek help: Teach women to call their obstetric provider immediately, go to an emergency department, or call emergency services if any red flag symptom appears. Provide written discharge instructions with clinic and emergency contact numbers. | Effective postpartum health education must be tailored to the mode of delivery (vaginal vs. cesarean) and use the teach-back method to confirm understanding. Education should start antenatally and be reinforced before hospital discharge.  Core components by category:  A. General postpartum recovery (all women)  Normal lochia progression: red (days 1–3) → pink/brown (days 4–10) → white/yellow (days 11–20). Any return to bright red with increased flow is abnormal.  Pain expectations: afterpains (worse with breastfeeding) peak at days 2–3; perineal pain improves daily; cesarean pain improves after day 3.  Bowel and bladder function: void within 6 hours of delivery; bowel movement by day 3–4; report dysuria, retention, or incontinence.  B. Vaginal delivery-specific advice  Perineal care: front-to-back cleaning, peri-bottle use, sitz baths, pad changes every 3–4 hours.  Signs of perineal wound infection or dehiscence: increasing pain, purulent drainage, wound gaping, fever.  Hemorrhoid management: ice packs, topical witch hazel, stool softeners.  C. Cesarean delivery-specific advice  Incision care: keep dry, inspect daily for redness, drainage, or separation. No submersion in water (baths, pools) until 6 weeks.  Activity restrictions: no lifting more than 5–10 kg (baby weight is fine), no driving for 2–3 weeks (or as long as taking narcotics), no strenuous exercise for 6–8 weeks.  Signs of wound complication: fever, increasing pain, drainage, or wound edges pulling apart.  D. Danger signs requiring immediate medical attention (all women)  Red (bleeding) – Hemorrhage criteria above.  Yellow (infection) – Fever, foul lochia, painful urination.  Blue (VTE) – Leg swelling/redness, chest pain, dyspnea.  Green (hypertension) – Severe headache, visual changes, epigastric pain.  Black (mental health) – Self-harm or infanticidal thoughts.  E. Self-care and support  Pelvic floor exercises (Kegels) from day 1–3 (if comfortable).  Nutrition and hydration: increase fluid and fiber; continue prenatal vitamins if breastfeeding.  When to call vs. when to go to emergency department: Provide a written one-page summary.  Teach-back questions: "Please show me how you will check your cesarean incision each day." "What would you do if you suddenly felt short of breath?" "What symptoms would make you call the emergency number?"  Timeline: Give verbal instructions at 24 hours postpartum, reinforce at discharge, and provide a written checklist with contact numbers for the office, after-hours triage, and emergency services. | To ensure that all women can recognize abnormal symptoms and seek timely help, postpartum health education must be adapted to the woman's health literacy level, language, and cultural context. A one-size-fits-all handout is insufficient.  Core components – structured as a simple action plan:  Component 1: Normal vs. Abnormal – The "Stop and Call" list  Create a short list of seven absolute reasons to call or go to the hospital (the "Seven Sins" approach):  Fever (temperature >38°C / 100.4°F) – not just chills.  Fainting or dizziness – especially when standing.  Flooding – soaking a pad in less than one hour.  Foul discharge – bad smell from vagina or wound.  Failing leg – one leg swollen, red, or painful.  Fierce headache – not relieved by over-the-counter painkillers.  Frightening thoughts – thoughts of harming self or baby.  Component 2: Practical "how to seek help" instructions  Phone numbers – Write down the clinic number, after-hours triage, and emergency number. Put it on the refrigerator or inside the phone case.  Transport plan – Discuss who will drive her if she needs urgent care. If no car, know the nearest public transport or emergency medical service.  What to say – "Tell the person on the phone: 'I gave birth [X days/weeks ago] and now I have [specific symptom].'"  Component 3: Cultural and low-literacy adaptations  Use pictograms or illustrated cards showing danger signs (e.g., pad soaked with blood, swollen leg, woman with hand on head indicating severe headache).  For women who do not read: provide audio recordings in their language or a video shown on a mobile phone before discharge.  Involve family members (partner, mother, mother-in-law) in the education session, as they often make decisions about seeking care.  Component 4: Early warning system for home  Provide a simple "green-yellow-red" chart:  Green (safe): mild discomfort, normal lochia, able to care for baby.  Yellow (call clinic within 24 hours): low-grade fever, mild headache, constipation.  Red (emergency, go now): any of the Seven Sins above.  Component 5: Follow-up and reinforcement  Schedule a phone call or text message at 3–5 days postpartum to review danger signs.  Provide a checklist for the 6-week visit, including mental health, contraception, and pelvic floor symptoms.  For low-resource settings (home births, early discharge):  Train community health workers to visit at days 1, 3, and 7 postpartum.  Use a one-page visual aid (laminated) with emergency contact numbers and transport options.  Teach a simple "three questions" to ask herself each day:  Am I bleeding more than my usual period?  Do I have a fever or bad pain?  Do I feel very sad or scared about hurting myself or my baby?  Documentation: Ensure that education was provided, understood, and that the woman (or her family) can repeat back at least three danger signs and the correct action. Sign and date the education record. |
| **Flesch–Kincaid Grade Level** | 6.95 | 9.39 | 8.43 |
| **Corresponding English Reading-Difficulty Category** | Grade 7 or below: fairly easy to read | Grades 10–12: fairly difficult | Grades 8–9: plain English |

# Guideline sources / 指南来源

- WHO recommendations on maternal and newborn care for a positive postnatal experience, 2022.
- NICE guideline NG194: Postnatal care.
- ACOG Committee Opinion No. 736: Optimizing Postpartum Care.
- Chinese National Basic Public Health Service Specification: Maternal Health Management Service Specification.
- NICE guideline CG192: Antenatal and postnatal mental health.
- FSRH Clinical Guideline: Contraception After Pregnancy.
- RCOG Green-top Guideline No. 37a: Reducing the Risk of Thrombosis and Embolism during Pregnancy and the Puerperium.
